# Getting closer to each other? Convergence and divergence patterns of life expectancy in 277 border regions of Western Europe 1995–2019

**DOI:** 10.1007/s10654-025-01279-w

**Published:** 2025-07-19

**Authors:** Sophie Stroisch, Pavel Grigoriev, Michael Mühlichen, Rok Hrzic, Tobias Vogt

**Affiliations:** 1 Population Research Centre, Faculty of Spatial Science, University of Groningen, Groningen, The Netherlands; 2 Institute for Social Sciences, Carl von Ossietzky University, Oldenburg, Germany; 3 Federal Institute for Population Research, Wiesbaden, Germany; 4 Department of International Health, Care and Public Health Research Institute (CAPHRI), Maastricht University, Maastricht, The Netherlands; 5 Prasana School of Public Health, Manipal Academy of Higher Education, Manipal, India

**Keywords:** European Union, Cross-border, Mortality convergence, Life expectancy, Spatial differences

## Introduction

The establishment of the European Union (EU) through the 1992 Maastricht Treaty and the opening of internal EU borders through the 1995 Schengen Agreement have made cross-border regions an important target of EU policy, which aims to harmonise living standards across national boundaries. Research indicates that these efforts have led to economic convergence in border areas, with increased economic activity resulting from open borders and enhanced cross-border cooperation [1]. While the link between economic development and population health has been extensively studied at the national level [2–4], it remains uncertain whether improved local cross-border cooperation has also led to convergence in population health.

Given the increasing political, economic, legal, and cultural interdependencies among member states, referred to as European integration [5], cross-border regions offer a unique opportunity to study differences in mortality, presenting one-third of the European population [6]. While non-border regions can be highly diverse, ranging from urban hubs to rural peripheries, border regions tend to share structural features that distinguish them from the rest of the country. These regions have a history of marginalisation due to their peripheral position within national economies [7]. On the other hand, and especially with the opening of the internal EU border, these regions have a greater exposure to cross-border trade, labour mobility, and economic spillovers. These regions often share similar cultural and historical backgrounds, and their geographical proximity facilitates cross-border work, service provision, and access to healthcare. At the same time, they are governed by country-specific social policies and healthcare frameworks [8, 9], allowing for a systematic comparison of how these differing policies can lead to varying health outcomes, even among populations with many shared characteristics.

The harmonisation of health and healthcare quality is one of the central aims of the EU, along with the equalisation of living standards and economic prosperity, as set forth by Article 174 of the Treaty on the Functioning of the European Union [10]. Following this, the law Directive 2011/24/EU was established, granting patients the right to access cross-border healthcare within the EU. Its main objective is to allow patients to receive medical treatment in other EU countries and be reimbursed by their home country’s healthcare system. Additionally, it aims to promote mutual learning and coordination in healthcare provision to facilitate smooth access to medical treatment across borders [11].

Despite these efforts, and even though life expectancy has increased in all 28 EU member states [12, 13], regional and socio-economic inequalities in health outcomes persist both between and within countries (e.g., [14, 15]). They are often attributed to factors such as varying economic success, health behaviours, and access to healthcare infrastructure. Moreover, the EU’s efforts to equalise living standards should also lead to convergence in life expectancy. This idea has been framed in Vallin and Meslé’s convergence-divergence theory [16], which suggests that countries’ mortality rates converge or diverge based on their adoption of medical technologies, public health policies, and health behaviours. For instance, the European East-West divide in mortality has been attributed to the slow diffusion of innovations, especially in cardiovascular health. Scholars have applied the concept of mortality convergence to explore geographical and socio-economic differences at the sub-national level, finding that regions and member states with high initial mortality also experienced the greatest improvements over time [17].

Although these studies have provided an overview of regional mortality convergence in the EU, three limitations can be identified. First, there has been a focus on the persistent gap in life expectancy between old and new member states, particularly after the EU enlargement in 2004 [12, 18, 19]. However, studies on the persistent differences between old member states have received less attention. As of 2023, life expectancy disparities among these nations remain, spanning nearly 3 years, from 81.2 years in Germany to 84 years in Spain for both sexes combined [20]. Second, their regional scope does not go beyond the state level or larger political units such as provinces. A few studies have looked at smaller administrative units, namely NUTS-3 regions, e.g. in the Netherlands [21] and Germany [22]. However, these analyses are restricted to a single country, leading to the third limitation. Most of the studies focus on regional differences within or between countries. A Europe-wide comparison at such a small regional level is very limited in the current literature. A recent study by Sauerberg and colleagues [23] is a notable exception. It was the first to examine mortality trajectories at the NUTS-3 level for 16 European countries. The study found that although mortality differences did decrease over time, with high-mortality regions catching up with low-mortality regions, large regional inequalities within and between countries persist. However, to our knowledge, none of the existing studies have considered the role of national borders in shaping regional mortality convergence or divergence. A recent scoping review highlights this gap, indicating that there is only scattered evidence on health outcomes in cross-border regions with no systematic focus on mortality trends across border regions [24].

The present study investigates mortality convergence in the EU’s internal border regions, a perspective that has not been explored before. We examine life expectancy trends in 277 EU border regions from 1995, the year the Schengen Treaty came into force, until 2019, the last year before the onset of the COVID-19 pandemic. We chose 1995 as our starting point, as it marks the implementation of the Schengen Agreement, which brought about a notable shift in cross-border mobility for the founding members of the European Union. This development fostered greater economic, social, and healthcare integration among these countries. During this time, new cohesion policy instruments were also launched, such as the Cohesion Fund [25] and the Interreg programme. While these initiatives aimed primarily at enhancing economic and territorial integration, their potential long-term effects on health convergence have yet to be thoroughly examined, as much of the previous research has concentrated on economic outcomes (e.g., [26, 27]). By extending our study into the late 1990s, we not only capture the initial impacts of these institutional changes but also observe the longer-term trends in life expectancy. In addition, we quantify the relative improvements in life expectancy in border regions by comparing them with several reference groups: (i) neighbouring border regions, (ii) non-border regions within the same country, and (iii) all selected countries combined.

## Data and methods

### Data

We used population and death counts, collected from national statistical offices for 878 regional units, of which 277 are border regions belonging to 12 countries. For the third reference group, we additionally retrieved data from Eurostat [20]. To classify border regions, we follow the *methodological manual on territorial typologies* by Eurostat [28], which is applied at level 3 of the Nomenclature of Territorial Units for Statistics (NUTS-3) and identifies regions that are located at the border of one or more adjacent member states. As we were dealing with data from various countries, we had to harmonise the data to ensure comparable estimates over time and place (see supplementary material S1 for more details).

The countries included are Germany, the Netherlands, Belgium, France, Austria, Denmark, Sweden, Finland, Spain, Switzerland, Italy, and Portugal. We selected Western European countries because they have been member states of the EU or the European Free Trade Association, as is the case for Switzerland, throughout the observation period. We focused on the older member states, which form a homogeneous group of countries sharing common aspects of political and economic integration, similar mortality rates, and reliable data availability. Regional data from the United Kingdom, a founding member of the EU, and Norway, a member of the Schengen area, were not available to us for the whole study period.

### Life expectancy at birth

To investigate the mortality trends between 1995 and 2019 at the district level, we estimated life expectancy at birth for all 277 border regions. Estimating life expectancy at a small geographical scale, such as NUTS-3 regions, may be affected by missing data or small numbers of deaths, leading to random fluctuations in the trend line. To tackle this, we utilised a two-dimensional p-spline model to smooth mortality rates [29]. This model applies smoothing parameters over the entire mortality surface, i.e. over age groups (0, 1–4, 5–9, 10–14,…, 90–94, 95+) and years (1995 to 2019) simultaneously. This approach helps to minimise fluctuations, making it easier to discern long-term mortality trends without being influenced by short-term anomalies and zero counts. We applied this model for each spatial unit and sex, constructed period life tables, and estimated life expectancy. Additionally, we computed 95% confidence intervals using bootstrapping life tables with 1000 iterations, based on binomial assumptions [30]. For additional model diagnostics, supplementary material S4 shows the life expectancy trendlines with actual and smoothed mortality rates, including their confidence intervals for all regions. Additionally, supplementary material S5 provides the Pearson residuals from the penalised spline model. We visualised these residuals using heatmaps to illustrate their distribution across the entire mortality surface, covering all age groups and years, as well as for every geographical unit and sex.

### Reference groups to assess life expectancy convergence

To quantify relative improvements in life expectancy over time and to identify patterns of convergence or divergence in border regions, we examined their life expectancy trajectories relative to three reference groups. First, we analysed a cross-country comparison to determine whether border region of one country performed better than border region in neighbouring countries. Second, we considered a within-country comparison, assessing whether a border region improved relative to other regions within its own country. Finally, we benchmarked these regions against a Western European standard to provide an overarching reference point. By structuring the comparison in this way, we aim to capture different dimensions of regional performance and assess whether border regions are converging or diverging in terms of life expectancy. One way to assess such convergence is through delta convergence, which measures progress towards a benchmark by evaluating whether a region’s indicator is moving closer to or further from it [31, 32].

#### Reference 1: neighbouring border regions

To compare convergence between neighbouring border regions, we compare only those regions that are directly adjacent across national borders. For each pair of neighbouring countries, we calculate life expectancy separately for the border regions on either side of the shared border, using abridged life tables based on aggregated death and population data. For example, in the case of Germany and Denmark, we compare German regions bordering Denmark with Danish regions bordering Germany. This targeted approach allows us to assess whether life expectancy has converged specifically in regions that might be affected by the removal of hard borders and increased cross-border integration at most. To determine the delta difference, we subtracted the average life expectancy of the border regions of one country from the neighbouring regions of the other country, with the reference group being the country’s border region that performed better than the other at the beginning of our observation period in 1995. We categorised all cross-border regions according to their life expectancy trendlines. We speak of delta-convergence, if the gap between the trendlines of the two bordering regions has narrowed over time, and of divergence if this gap has widened. We added a third category to highlight regions where the change in the gap was less than 0.5 years and greater than − 0.5 years, indicating stagnation or minor changes.

#### Reference 2: non-border regions of the same country

Furthermore, we compared the average life expectancy of all border regions with that of non-border regions within the same country. This approach gives valuable insights into whether changes in life expectancy in border regions reflect broader national trends or are unique to those specific regions. Relying solely on cross-country comparisons may present an incomplete picture. If a border region is improving but remains overshadowed by even greater progress in neighbouring regions across the border, its relative success might be underestimated. Similarly, suppose a previously peripheral border region is converging with other regions within its country. In that case, this still represents meaningful progress, even if the improvements are not as pronounced as those observed across national boundaries. Additionally, it helps us to identify whether the border regions of different countries are structurally distinct from the rest of the nation.

Based on the performance of border regions relative to non-border regions, we categorised the countries into four groups (see Hrzic et al. for further details) [22]. First, *decreasing advantages* refer to cases where life expectancy in border regions was higher than in the reference group in 1995, but the gap between the two groups narrowed by 2019. Second, *decreasing disadvantages* occur when life expectancy in border regions was initially lower than in the reference group in 1995, and this difference also became smaller by 2019. Third, *increasing advantages* describe situations where life expectancy in border regions was higher than in the reference group in 1995, and this gap continued to widen by 2019. Finally, *increasing disadvantages* occurred when life expectancy in border regions was lower than in the reference group in 1995, with the difference growing over time until 2019. We deliberately did not use the same categorisation for the earlier reference group of neighbouring border regions to avoid determining any specific border region as a control group. The trends in the differences between the two neighbouring border regions are interdependent. If one region experiences an increasing advantage, the other automatically experiences an increasing disadvantage and vice versa. Moreover, we added another category to indicate regions with stagnation or minor change.

#### Reference 3: mean of all selected countries

For comparison with the Western European average, we estimated the annual average life expectancy of all 12 countries combined as a benchmark to compare all border regions across all countries. This reference group enables a thorough comparison of all Western European border regions while taking into account the previously discussed EU objective of harmonising living standards, including life expectancy. It also helps identify well- and underperforming regions and assess whether border regions are converging towards the broader European average, potentially reflecting benefits from EU integration, such as economic development, improved infrastructure, or better access to services. To determine delta convergence, we subtracted the average life expectancy of the benchmark from each border region individually. We used the same categorisations for the reference groups for non-border regions.

### Sensitivity analysis

To determine whether border regions are converging or diverging relative to their reference groups, we employ a two-endpoint approach. This method, which captures changes between the initial and final time points of the study period, provides a clear and interpretable assessment of long-term trends. It is consistent with well-established convergence methodologies [22, 33] and aligns with EU reports assessing convergence (e.g., [34, 35]). Additionally, previous studies [34] highlight that the selection of time points can influence findings, as different time horizons may lead to varying conclusions about convergence. To account for this, we include an alternative starting year of 2000 as a sensitivity check to evaluate whether our findings hold when using a shorter time horizon (see supplementary material S1). Additionally, in supplementary material S6, we included boxplots to visually compare the distributions of life expectancy outcomes in border regions across different reference groups.

All computations and visualisations were conducted in R. For smoothing mortality rates, we employed the ‘Mort2Dsmooth’ function from the *MortalitySmooth* R package [29].

## Results

**Fig. 1:**
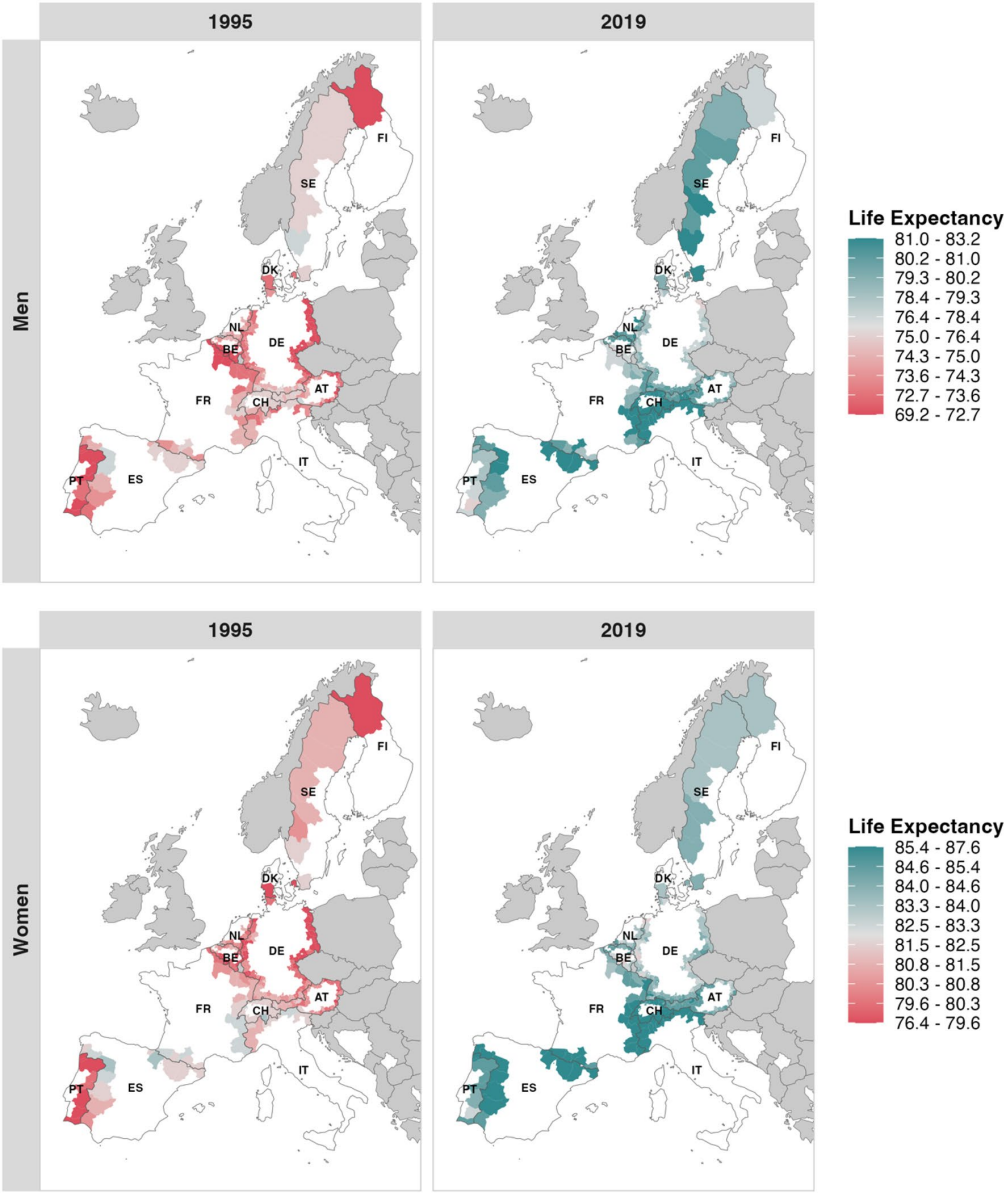
Life expectancy at birth for NUTS-3 regions by sex (rows) and years (columns). Coloured by division

As shown in Fig. 1, life expectancy has increased in all Western European border regions since the establishment of the Schengen area. Still, larger regional differences exist in the number of additional life years gained. In 2019, the highest life expectancy was 83.2 years (CI: 82.7; 83.7) in *Neuchâtel*, Switzerland for men and 87.6 years (CI: 87.1; 88.1) in *Salamanca*, Spain for women. The lowest life expectancy for men was 75.9 years (CI: 75.1; 76.5) in *Vorpommern-Greifswald*, Germany, and 80.5 years (CI: 78.5; 82.3) in *Pirmasens*, Germany, for women. The Portuguese region of *Cávado* shows the greatest increase in life expectancy of all the border regions studied. Men gained 8.9 years and women 7.4 years. The smallest gain in life expectancy was 3.8 years for men in *Krefeld*, Germany, and 1.3 years for women in *Pirmasens*, Germany. In general, men gained more years of life than women over the observation period (men: +5.8 years; women: +3.7 years). Nevertheless, women still have a higher average life expectancy in 2019 (women: 84.3 years; men: 79.7 years). A complete overview of life expectancy in all 277 border districts in 2019 can be found in the supplementary material (Supplementary material S2 for male life expectancy and Supplementary material S3 for female life expectancy).

**Fig. 2:**
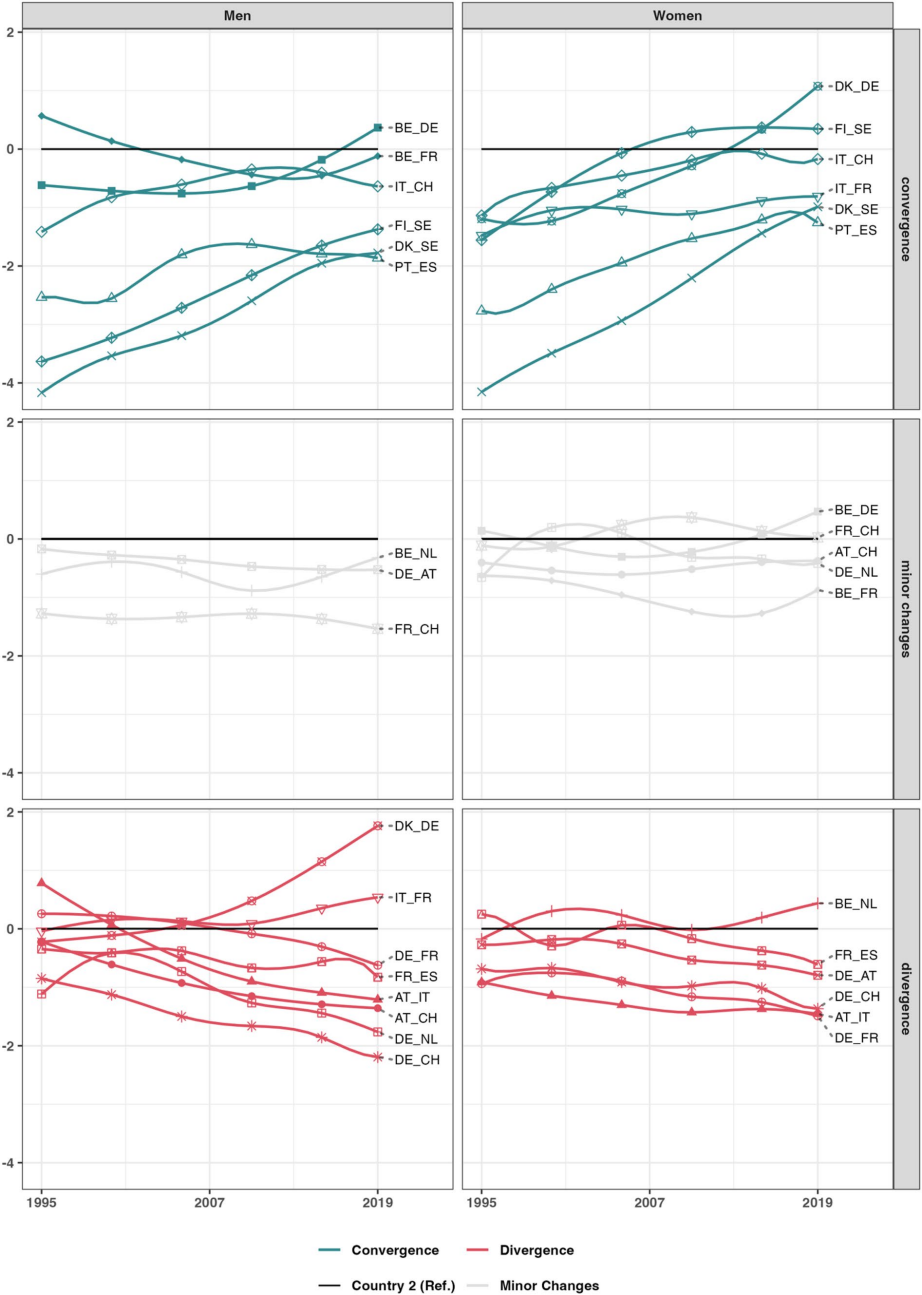
The difference in life expectancy trajectories between 1995 and 2019 of border regions in years compared to the first reference groups, the neighbouring countries’ border regions. The label represents the countries’ border pairs. The regions with consistently higher life expectancy over the years were chosen as the baseline reference. The first mentioned country corresponds to the coloured line and the second country serves as the baseline. The lines are coloured according to the convergence category

Figure 2 illustrates the trends in life expectancy in cross-border regions, indicating that convergence occurred in 6 cross-border regions. For men, the gap between the Danish and Swedish (DK-SE) border regions narrowed by 2.4 years, while the Finnish and Swedish (FI-SE) regions experienced a reduction of 2.2 years. Similarly, among women, the Portuguese and Spanish (PT-ES) border regions saw the gap close by 1.5 years, and the Danish and Swedish (DK-SE) regions witnessed a reduction of 3.2 years. The boxplots, shown in Supplementary Material S6, provide additional insights. For example, the top-performing Danish region adjacent to Sweden achieved the same life expectancy as its Swedish neighbouring region by the end of the observation period. Furthermore, while the gap between cross-border regions has been narrowing throughout the entire period, the development of life expectancy was not consistent among the border regions themselves. Spanish border regions exhibited a stable life expectancy trend with minimal variation in regional distribution. In contrast, the Portuguese border regions experienced significant internal shifts. From 1999 onward, regional disparities initially decreased but then widened again, with one particular region consistently standing out due to its markedly lower life expectancy. This observation indicates that certain Portuguese regions progressed at a slower pace, and the overall convergence observed may obscure the underlying diversity in regional developments. Despite improvements in Portuguese border regions, they still lag behind Spanish border regions. Contrarily, men in the German-Swiss (DE-CH), German-Dutch (DE-NL), German-Danish (DE-DK), Austrian-Swiss (AT-CH), and Austrian-Italian (AT-IT) border regions exhibited a diverging trend, where the gap increased by a year or more. These trends consistently disadvantaged the German and Austrian border regions, respectively, as the increase in life expectancy of the neighbouring border regions was higher than those of German and Austrian border regions. Additionally, in many German border regions, the increase in life expectancy slowed down since the 2010s. Although women showed similar diverging trends, they were less pronounced compared to men. The DK-DE border region presents an exceptional case, where the female life expectancy of both areas converged until 2013, after which it began to diverge again.

**Fig. 3:**
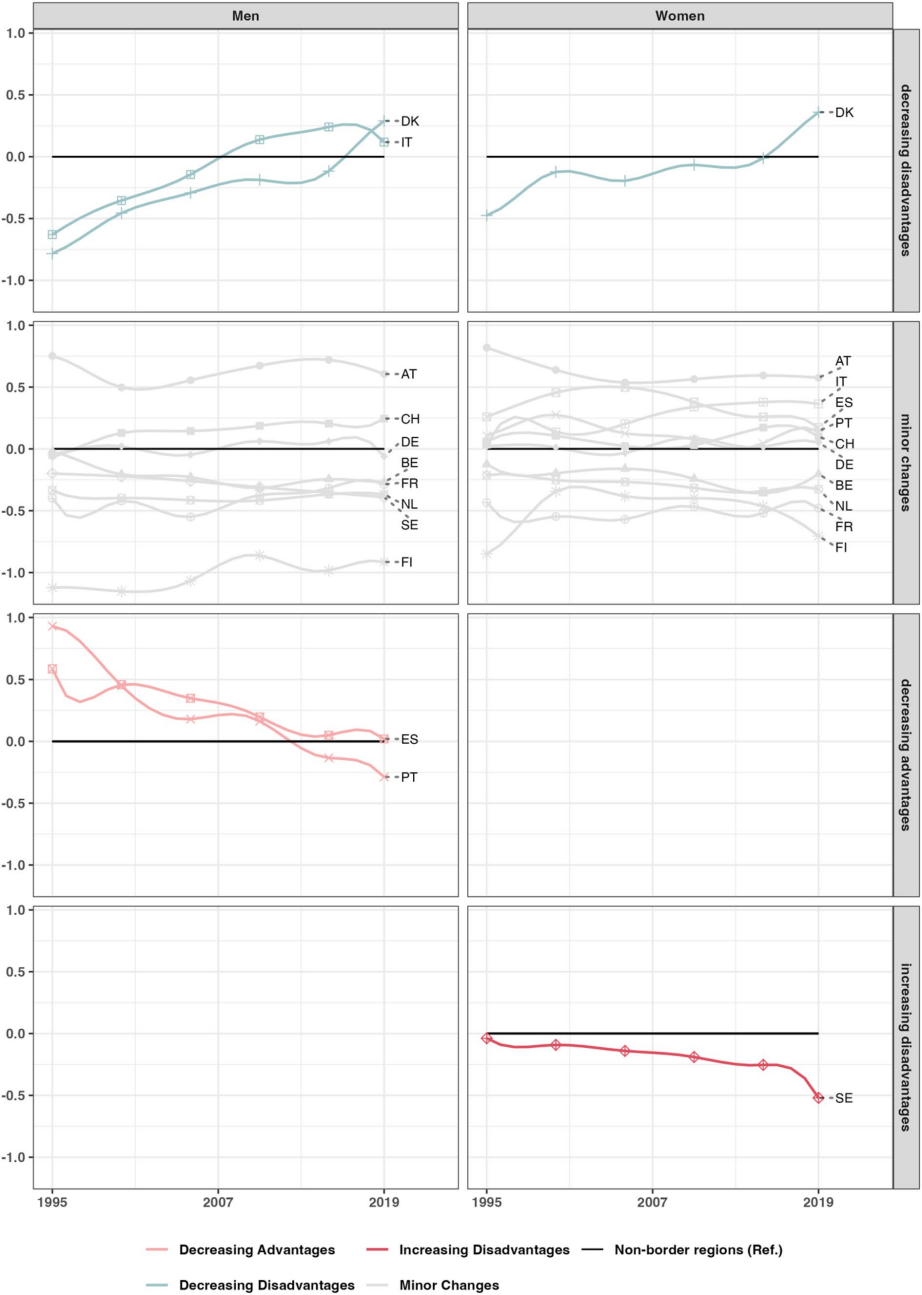
The difference in life expectancy trajectories between 1995 and 2019 of border regions (coloured line) in years compared to the second reference groups, the non-border regions of the respective country (baseline). The lines are coloured according to the convergence category

For most countries, there is no evidence of either convergence or divergence in life expectancy between border and non-border regions among Western European countries. The differences between the two groups do not change more than 0.5 years during the observation period, as shown in Fig. 3. The Finnish border region has consistently recorded a lower life expectancy, typically around one year less than the non-border regions of the country. Danish men (1995: -0.8 years, 2019: 0.3 years) and women (1995: -0.5 years, 2019: 0.4 years) and Italian men (1995: -0.6 years, 2019: 0.2 years) exhibited decreasing disadvantages. In all three cases, the border regions initially lagged behind their non-border counterparts, but by the end of the observation period, they had surpassed them in life expectancy. Furthermore, the boxplots reveal that regional variability in life expectancy among the Danish and Italian border regions decreased throughout the period, while their respective non-border regions saw either rising (Denmark) or stagnant (Italy) variability. Therefore, border regions in these countries not only caught up in average life expectancy but also became more internally equal, indicating successful regional convergence within border regions. Conversely, Portuguese (1995: +0.9 years, 2019: -0.3 years) and Spanish (1995: +0.6 years, 2019: 0 years) men in border regions experienced decreasing advantages. Swedish women (1995: 0 years, 2019: 0.5 years) showed a trend of increasing disadvantages. Overall, differences between border regions and their reference groups are more pronounced across neighbouring countries (see Fig. 2) than within the same country. In the earliest period, the maximum difference between neighbouring border regions exceeded four years, while the gap between border and non-border regions was just over one year.

**Fig. 4:**
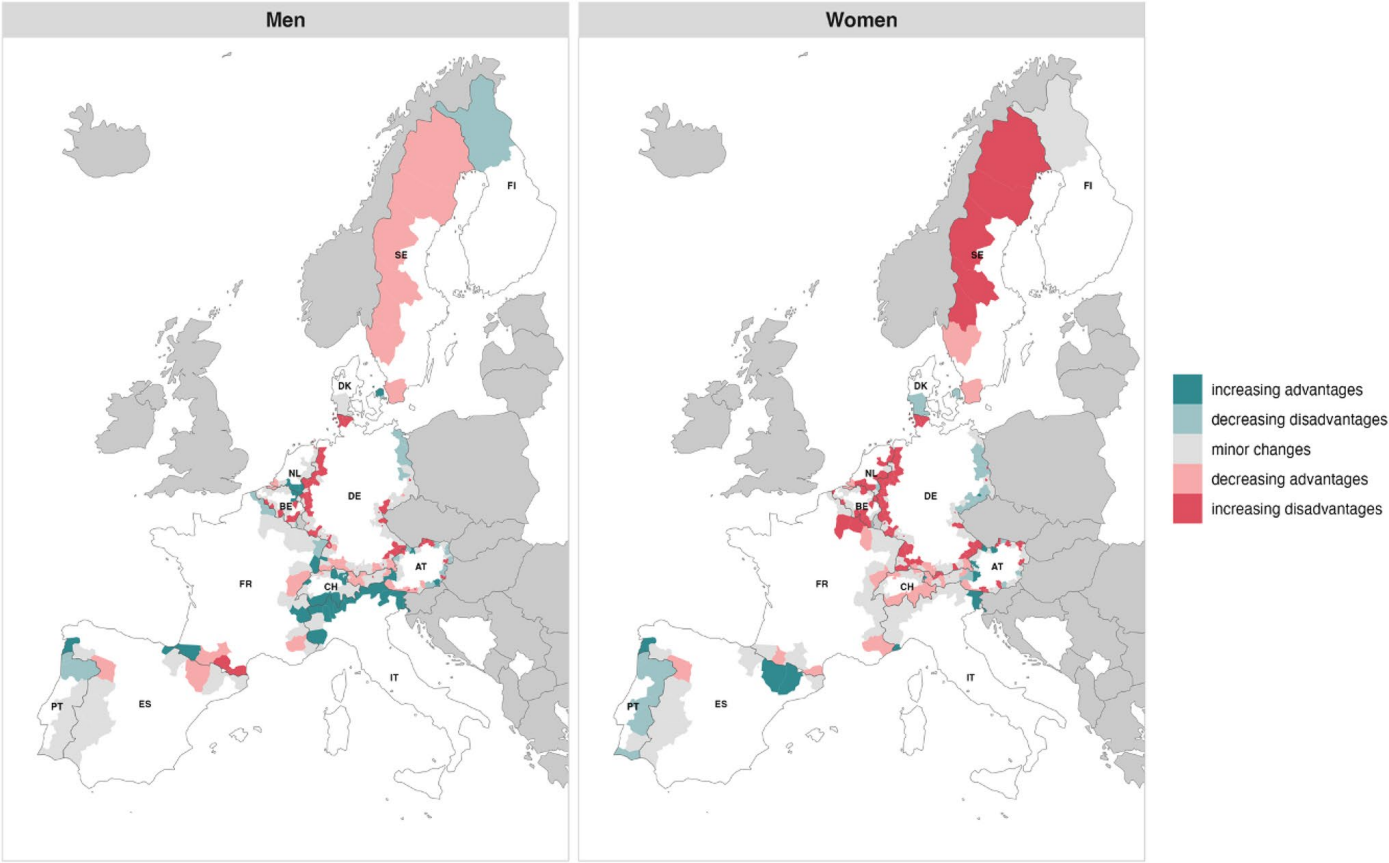
Convergence and divergence patterns of life expectancy trajectories from 1995 to 2019 between individual border regions (NUTS-3) and the third reference group, the average of all 12 selected countries

Figure 4 shows the convergence or divergence towards the mean of all 12 Western European countries included in this study. It reveals a discernible trend of increasing advantages among men in the Alpine region, particularly in the Italian and Swiss border regions. In particular, the Italian regions of *Valle d’Aosta* and *Belluno* exceeded the EU average by 1.5 years, whereas in 1995 they were below the average. Moreover, the northern Portuguese border regions and the Finnish border region exhibit a decrease in disadvantages. Conversely, Sweden shows a decrease in advantages in all border regions. Additionally, several German border regions, especially those bordering Denmark, the Netherlands and Belgium, show increasing disadvantages. For instance, the gap between the Western European mean and the German regions of *Krefeld* and *Flensburg* where life expectancy was already lower than the Western European average in 1995, widened by almost 2 years. For women, the Alpine regions do not show the same clear pattern as for men. However, a few regions along the Austrian border, two Spanish regions, one bordering Portugal and one bordering France, show an increase in advantages. Conversely, almost all Portuguese border regions, Danish border regions, and German regions bordering Poland and Czechia experienced decreasing disadvantages. Meanwhile, Swedish border regions witnessed increasing disadvantages, as did many regions in German, Dutch, Belgian, and French border regions.

## Discussion

This study offers a comprehensive analysis of life expectancy patterns across Western European border regions, using novel granular mortality data to identify potentially successful regions. We examined life expectancy trends in 277 border regions in 12 Western European countries from 1995 to 2019, measuring improvements in these regions by comparing them with neighbouring border regions, non-border regions of the same country, and all selected countries combined. This analysis yielded two major results. First, despite overall increases in life expectancy in all border regions, the pace of improvement varied notably across countries and regions, revealing clusters of successful and less successful border regions regarding mortality convergence. Second, we found that disparities between border regions of neighbouring countries were more pronounced than those between border and non-border regions within the same country, suggesting that, despite European integration efforts, national and regional contexts significantly shape mortality patterns across Europe.

The observed increases in life expectancy in all border regions are consistent with previous studies on mortality improvements at national [12, 36], provincial [13, 19], and district [23] levels. Despite these overall gains, the absolute differences in life expectancy between individual border regions remain large, suggesting that there is no uniform European pattern. Although greater gains in life years in certain regions could be explained by European integration, the persistent large differences between them are most likely determined by country- and region-specific factors. Moreover, the differences in life expectancy were more pronounced when comparing border regions with those of neighbouring countries, as opposed to the non-border regions of the same country. This further underscores the importance of the national and regional context in shaping mortality differences, despite ongoing harmonisation efforts at the EU level. EU cohesion policies have been an important tool in addressing regional disparities. Between 2014 and 2020, approximately €347 billion, accounting for nearly one-third of the total EU budget, was allocated to this policy [37]. A substantial portion of these funds is designated for the European Regional Development Fund, which aims to mitigate developmental disparities across European regions, prioritising those that are disadvantaged [38]. Despite these efforts, there seems to be limited success in achieving the intended outcomes, as reflected in persistent mortality disparities. While life expectancy has increased overall, the timing and magnitude of these improvements differ across regions. Some border regions show strong improvements in life expectancy, while others lag compared to neighbouring border regions, non-border regions or the Western European average.

At the national level, Vallin and Meslé have identified vanguards– countries leading in the international ranking– and laggards– countries following the vanguards at slower or similar improvement rates [16]. Sauerberg and colleagues confirmed the complex landscape of mortality development by highlighting the dichotomy of leading and lagging regions at the district level [23]. While these previous studies have focused primarily on the East-West mortality gap, our research reveals that persistent mortality differences also exist within and across Western European border regions. As a result, we identified distinct clusters of leading and lagging border regions concerning mortality convergence.

For example, the Alpine regions, especially along the Swiss and Italian borders, show increasing advantages in life expectancy, thus exceeding the Western European average, for men. Additionally, northern Italian border regions are not only catching up but are even outperforming the predominantly southern, non-border regions. However, this trend may not be solely attributed to their border status but rather to broader regional patterns in Italy. Previous research has found gains in various provinces and districts of northern Italy [13, 23]. Nevertheless, male mortality in the north has not always been low and leading. This reciprocal north-south shift in mortality has been attributed to decreases in lung cancer and ischemic heart diseases in the north [39]. Moreover, the economically prosperous northern regions have been more effective in reducing their initially high mortality levels among adult men. Examining the Swiss side of the Alpine border region reveals the highest life expectancy figures, which is consistent with Switzerland’s status as one of the wealthiest nations in Europe, as measured by GDP per capita [40]. This economic prosperity is correlated with life expectancy outcomes [2, 4]. In addition, the specific characteristics of the Alpine environment, particularly its mountainous terrain, have been associated with lower mortality rates, as residing at high altitudes may confer health benefits [41].

Furthermore, we detected a convergence in life expectancy between the Nordic border regions, with the Finnish and Danish border regions catching up with their neighbouring Swedish border regions. Sweden’s global ranking has declined as mortality at higher ages decreases more slowly at older ages [42]. Improvement for Finland has been much faster than in Sweden [43], resulting in mortality convergence at national and regional levels. This is in line with our findings, which suggest convergence in the Finnish-Swedish border region.

The Finnish border region has made progress in catching up with its neighbouring Swedish regions and the rest of Finland, yet it still lags behind. This region, located in Finland’s far north, suffers from mortality disadvantages typically associated with peripheral areas. Previous research highlights higher mortality rates in northern Finland compared to western Finland [43]. The underlying reasons for these patterns remain somewhat elusive, though a higher prevalence of risky health behaviours in the north-east and possible genetic differences have been proposed as contributing factors [44].

Within the economic centre of Western Europe, we observed another cluster of lagging regions along the western German border. These areas reported the lowest life expectancy in 2019 and showed minimal gains in life expectancy for both sexes over time. This trend reflects previous studies that identified Germany as “one of the worst performers among high-income countries” regarding life expectancy at the national level [45]. Factors contributing to this include higher mortality rates from cardiovascular diseases and potential shortcomings in primary care and disease prevention. We also observed overall negative trends for western German border regions compared with border regions in neighbouring countries and the Western European average. For the latter, these disadvantaging trends extend to the Dutch, Belgian, and parts of the French border regions among females. This finding is particularly surprising given that, as mentioned previously, these border regions are located within a highly industrialised area of Europe, also known as the *European dorsal* [46]. This densely populated corridor, stretching from north-western England through the Benelux countries and western Germany to northern Italy, is known for its economic prosperity and advanced infrastructure. These factors could arguably have a positive impact on life expectancy. However, environmental and socio-economic factors may counteract these benefits. For example, industrial areas often face higher pollution levels, which leads to lower life expectancy [47]. Additionally, these regions have experienced significant deindustrialisation since the 1970s, leading to high unemployment [48] and selective out-migration, contributing to higher mortality rates as healthier, working-age individuals may move elsewhere in search of better opportunities [49]. Yet, these disadvantaging trends in life expectancy apply only to women.

Our sensitivity check reveals that while some regional classifications change when using a shorter time frame, the overall patterns largely remain consistent. For example, the border regions between Italy and France, as well as Italy and Switzerland, shift from showing convergence to displaying only minor changes. In contrast, for women in the German-Dutch and German-Belgian regions, the classification changes from minor changes to divergence, indicating short-term fluctuations. Additionally, the Danish border regions have shown increasing advantages since 2000, whereas they were previously categorised as experiencing decreasing disadvantages over a longer period. Despite these specific shifts, our sensitivity check confirms that the results remain stable in most cases when the starting point is adjusted to 2000. Most importantly, when comparing the border regions to the Western European average, the broader spatial patterns and cluster structures remain unchanged, with only a few isolated instances of category shifts.

While our findings are robust across various starting years, a two-time-point approach for determining convergence should be used with caution. This approach groups regions based on differences in life expectancy at the start and end points, potentially overlooking dissimilar intermediate trends. Although this approach helps summarise long-term patterns and offers easier interpretability, it may obscure short-term temporal dynamics. Moreover, our study utilised highly granular geographical data at the district level, providing a unique opportunity to explore an understudied group of regions: cross-border regions. This approach allowed for the identification of localised clusters of high and low mortality, providing valuable insights for targeted health interventions. However, using small-area death counts posed some challenges, particularly in sparsely populated regions. Sparse data can lead to high variability and potential instability in model estimates. To address these fluctuations, we employed penalised spline models [29], which effectively smooth irregularities in spatial or temporal data while preserving the underlying data structures [50] Despite their advantages, smoothing techniques can lead to over- or under-smoothing of critical trends. In areas with very few deaths, mortality patterns may be more influenced by random variation, potentially concealing important local differences [51]. However, as our observation period spans 24 years, individual outliers are unlikely to alter the overall pattern. Furthermore, our research investigated long-term mortality trends within a consistent demographic and epidemiological framework, intentionally excluding the COVID-19 period from our analysis. The pandemic has introduced exceptional short-term fluctuations in mortality rates, which may be especially relevant for border regions. These dynamics are inherently different from the trends observed before the pandemic. Consequently, incorporating these disparate periods could obscure the structural patterns we intend to scrutinise. Finally, our analysis is confined to the border regions of Western Europe, which have benefited from an extended period of open borders and are characterised by comparatively low mortality rates. The inclusion of Eastern European border regions could potentially uncover additional patterns worthy of investigation in future research; however, such inclusion falls outside the scope of our study.

Despite these limitations, our study adds to the literature in several ways. It provides a novel perspective on mortality convergence through the lens of cross-border life expectancy trends. Furthermore, we identify distinct clusters of leading and lagging regions across national borders, such as the high-performing Alpine regions of Italy and Switzerland, and the underperforming areas in the old industrial belt.

Our study adopts an intuitive approach to promote accessibility, yet its findings also carry significant policy implications. Our findings align with ongoing EU efforts to harmonise living standards, including health outcomes, and underline the challenges that remain in achieving this goal. While EU initiatives such as the directive on patient’s rights in cross-border healthcare aim to enhance healthcare coordination and accessibility across borders [11], their full potential has yet to be realised [52]. Furthermore, our findings suggest that differences between countries play a greater role in life expectancy disparities than a region’s location within a country, whether inland or along the border. These insights should be considered when developing new European Union directives and regional initiatives to enhance health integration.

To address these challenges, the EU could consider revising the allocation criteria for regional and cohesion funds, which currently rely heavily on GDP measures [37] and is motivated by economic convergences. Incorporating health indicators, such as mortality measures, might better target regions facing significant health disparities. Furthermore, the EU could explore developing tailored regional policies that extend national borders to address specific public health challenges in border areas. Cross-border regions, given their unique positioning, serve as experimental laboratories for policy learning and collaboration, enabling lagging regions to learn from the more successful. In this regard, initiatives like Interreg, which foster cross-border cooperation in healthcare, infrastructure, and economic development, are vital for promoting regional integration. Our study serves as an initial step in evaluating the impact of such initiatives by identifying ongoing disparities and pinpointing areas where closer collaboration could lead to meaningful health improvements.

Furthermore, the persistent disparities in Western European border regions highlight the important influence of structural and contextual factors, such as economic conditions, healthcare accessibility, and demographic composition, on these trends. It is essential to address these underlying differences to understand why some regions continue to fall behind, even as broader improvements are made. Thus, to improve the effectiveness of EU funding tools, future research should focus on structural and contextual factors and seek to disentangle the role of EU, national and regional policies. Cross-border regions offer a great setting to do this. To our knowledge, this is the first study that offers new insights into how regional trajectories of border regions may converge or diverge in the context of continued European integration. Finally, it provides a departure point for future studies aimed at unravelling the complex factors shaping health outcomes in European border regions.

## Electronic supplementary material

Below is the link to the electronic supplementary material.

Supplementary Material 1

Supplementary Material 2

Supplementary Material 3

Supplementary Material 4

Supplementary Material 5

Supplementary Material 6
