# Supplementary material for: Getting closer to each other? Convergence and divergence patterns of life expectancy in 277 border regions of Western Europe 1995–2019
Source: Eur J Epidemiol. 2025 Jul 19;40(9):1031–43. doi: 10.1007/s10654-025-01279-w (PMC12537618; doi:10.1007/s10654-025-01279-w)

Supplementary Material

# Data harmonisation

We used data from several countries from different sources. Therefore, we had to harmonise the data to obtain results that allow comparison between different countries and along the observation period. For almost all countries we estimated life expectancy on the NUTS-3 level except for Austria where we have used the domestic administrative unit, *Bezirke*, which is comparable to the NUTS-3 level. For Portugal and Italy, we aggregated regions together since there had been several regional reallocations over the years. Death counts of all-cause mortality were mostly available for the ages 0-95, except for Germany (0-90) and Portugal (0-85). In many cases, data were available in 1-year age intervals, however, we aggregated the data for all countries into 5-year age intervals leaving the first interval as a 1-year age interval to consider infant mortality. As an exception, Germany aggregates the ages between 1-15 into one age interval due to data privacy regulations. Population counts of the respective regions were mostly matching the same pattern as the mortality data. However, we used a univariate penalized composite link model (PCLM) to ungroup the first age interval of the Spanish population data to account for the infant interval (Rizzi et al., 2015). Austria provided annual population counts since 2002. Before 2002, only census data for 1991, 2001 and 2011 are available. We, therefore, estimated the annual population for the missing years (1995-2002) with a linear interpolation model.

Table 1. Summary of data adjustments

| Country | Adjustments |
| --- | --- |
| Austria | Linear interpolation model to compute missing population counts between 1995 and 2002;  Harmonisation of administrative units, due to regional reallocations over time |
| Belgium |  |
| Denmark |  |
| Finland |  |
| France |  |
| Germany |  |
| Italy | Harmonisation of administrative units, due to regional reallocations over time |
| Portugal | Harmonisation of administrative units, due to regional reallocations over time |
| Spain | Univariate penalized composite link model (PCLM) to ungroup the population counts for the first age interval |
| Sweden |  |
| Switzerland |  |
| The Netherlands |  |

# Categorisation of convergence


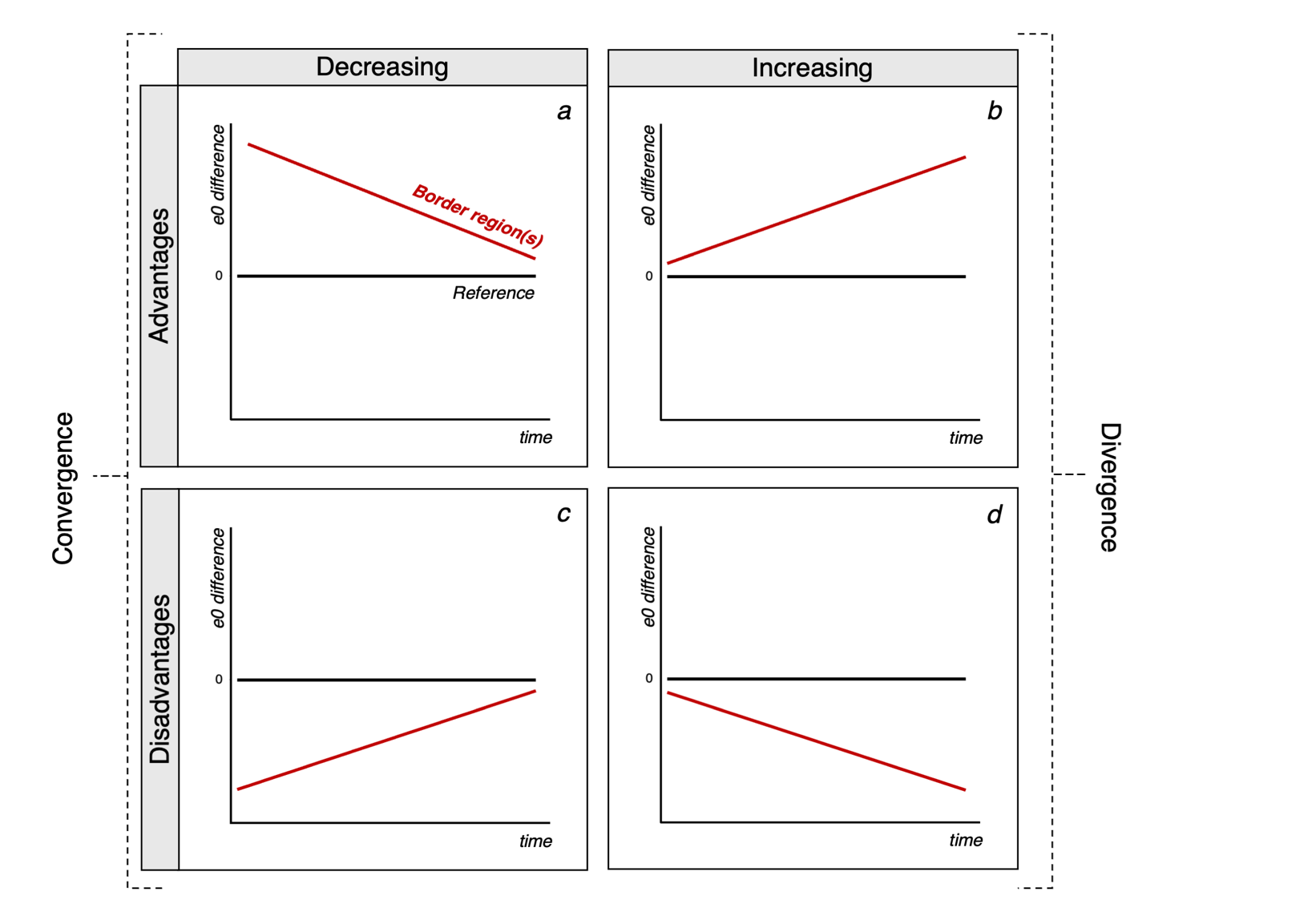


Illustration of different trends of delta convergence or divergence. Based on Hrzic et al., 2023

We classified the categories according to relative performance as suggested by Hrzic et al. (2023), which delineates four distinct categories. The first category encompasses the decreasing advantages of border regions, wherein the e0 of the border regions was higher than that of the reference group in 1995 (Figure 1a). The second category pertains to the decreasing disadvantages of border regions, wherein the e0 in the border region was lower than that of the reference group in 1995; however, the gap between the two regions lessened over time (Figure 1c). Nevertheless, the gap in e0 between the two regions decreased. The third category is associated with the increasing advantages of border regions, where the gap between the two regions also widened, but the e0 of border regions began at a higher level than the reference group (Figure 1b). The fourth category involves the increasing disadvantages of border regions in comparison to the reference group, where the e0 of border regions initiated at a lower level than the reference group, and the gap between the two widened over time (Figure 1d). For using neighbouring border regions as a reference, we combined the categories and solely differentiated between convergence and divergence. If the distance of $\delta$1995 to zero was bigger than the distance of $\delta$2019 to zero, then we speak of convergence, since the gap of e0 became smaller (Figures 1a & 1c). Divergence was identified when the distance of $\delta$1995 to zero was smaller than the distance of $\delta$2019 to zero (Figures 1b & 1d).

# Sensitivity Analysis

**Adding 2000 as an alternative starting point**

The selection of starting and ending points can influence findings, as different time horizons may lead to varying conclusions about convergence. Acknowledging this, we have included an alternative starting point, the year 2000, as a sensitivity check to assess the stability of our results.

**Reference group 1: neighbouring border regions**

Convergence patterns among border regions of neighbouring countries from 2000 to 2019, and for the periods 1995 to 2019, separately

|  | **Period** | |
| --- | --- | --- |
|  | **2000-2019** | **1995-2019** |
| **AT_CH** | | |
| Men | divergence | divergence |
| Women | minor changes | minor changes |
| **AT_IT** | | |
| Men | divergence | divergence |
| Women | minor changes | divergence |
| **BE_DE** | | |
| Men | convergence | convergence |
| Women | divergence | minor changes |
| **BE_FR** | | |
| Men | minor changes | convergence |
| Women | minor changes | minor changes |
| **BE_NL** | | |
| Men | minor changes | minor changes |
| Women | minor changes | divergence |
| **DE_AT** | | |
| Men | minor changes | minor changes |
| Women | divergence | divergence |
| **DE_CH** | | |
| Men | divergence | divergence |
| Women | divergence | divergence |
| **DE_FR** | | |
| Men | divergence | divergence |
| Women | divergence | divergence |
| **DE_NL** | | |
| Men | divergence | divergence |
| Women | divergence | minor changes |
| **DK_DE** | | |
| Men | divergence | divergence |
| Women | convergence | convergence |
| **DK_SE** | | |
| Men | convergence | convergence |
| Women | convergence | convergence |
| **FI_SE** | | |
| Men | convergence | convergence |
| Women | convergence | convergence |
| **FR_CH** | | |
| Men | minor changes | minor changes |
| Women | minor changes | minor changes |
| **FR_ES** | | |
| Men | minor changes | divergence |
| Women | minor changes | divergence |
| **IT_CH** | | |
| Men | minor changes | convergence |
| Women | convergence | convergence |
| **IT_FR** | | |
| Men | minor changes | divergence |
| Women | minor changes | convergence |
| **PT_ES** | | |
| Men | convergence | convergence |
| Women | convergence | convergence |

**Reference group 2: Non-border regions of the same country**

Convergence patterns among border regions and non-border regions from 2000 to 2019, and for the periods 1995 to 2019, separately

|  | **Period** | |
| --- | --- | --- |
|  | **2000-2019** | **1995-2019** |
| **Austria** | | |
| Men | minor changes | minor changes |
| Women | minor changes | minor changes |
| **Belgium** | | |
| Men | minor changes | minor changes |
| Women | minor changes | minor changes |
| **Denmark** | | |
| Men | decreasing disadvantages | decreasing disadvantages |
| Women | increasing advantages | decreasing disadvantages |
| **Finland** | | |
| Men | minor changes | minor changes |
| Women | minor changes | minor changes |
| **France** | | |
| Men | minor changes | minor changes |
| Women | minor changes | minor changes |
| **Germany** | | |
| Men | minor changes | minor changes |
| Women | minor changes | minor changes |
| Italy | | |
| **Men** | decreasing disadvantages | decreasing disadvantages |
| Women | minor changes | minor changes |
| **Portugal** | | |
| Men | decreasing advantages | decreasing advantages |
| Women | minor changes | minor changes |
| **Spain** | | |
| Men | minor changes | decreasing advantages |
| Women | minor changes | minor changes |
| **Sweden** | | |
| Men | minor changes | minor changes |
| Women | minor changes | increasing disadvantages |
| **Switzerland** | | |
| Men | minor changes | minor changes |
| Women | minor changes | minor changes |
| **The Netherlands** | | |
| Men | minor changes | minor changes |
| Women | minor changes | minor changes |

**Reference group 3: Mean of all selected countries**

Convergence patterns of border regions based on the 12 countries’ average for the periods 2000 to 2019


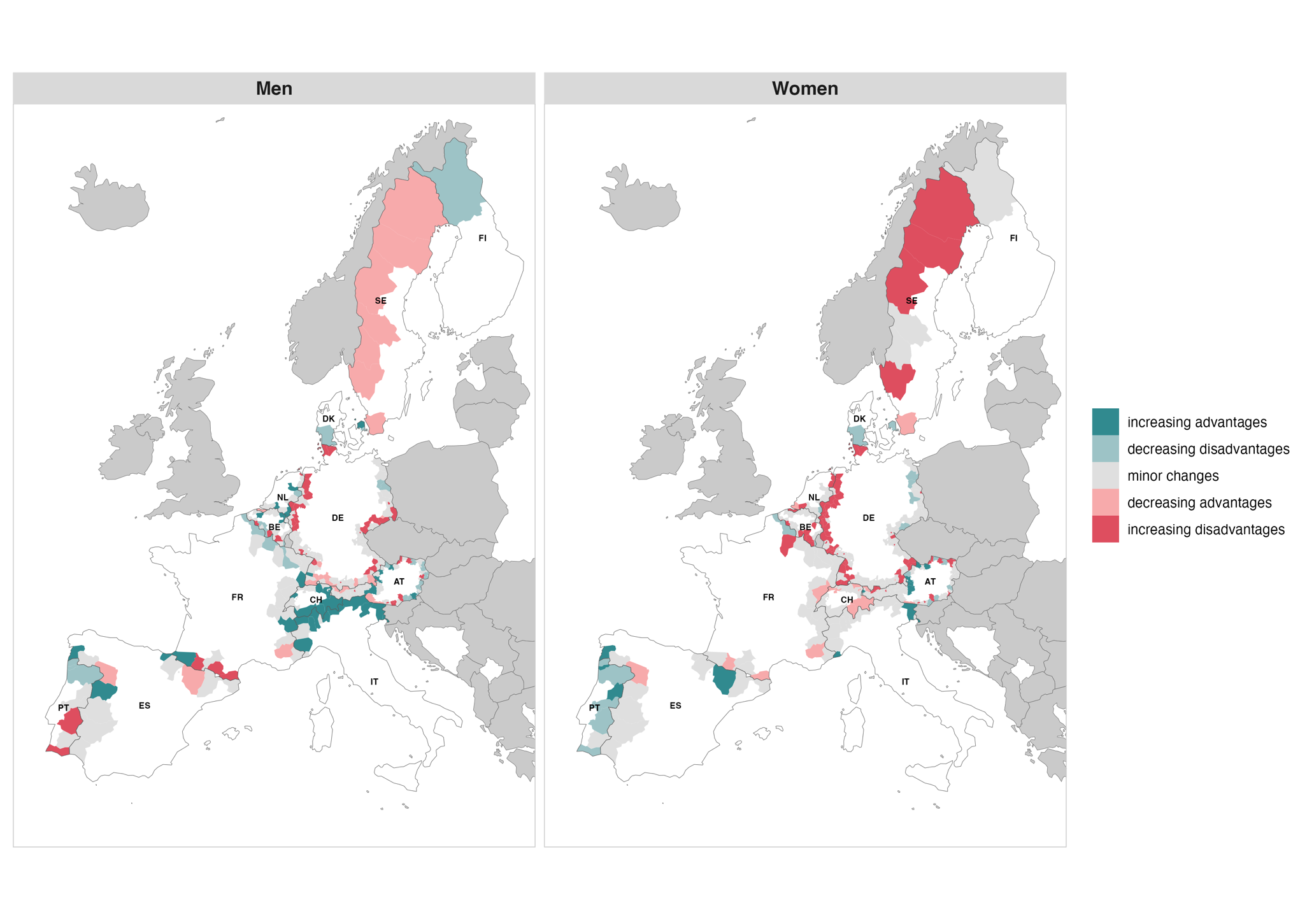

Supplement: Supplementary file 1 — Supplementary Material 1 [file 10654_2025_1279_MOESM1_ESM.docx]
