# Supplementary material for: Getting closer to each other? Convergence and divergence patterns of life expectancy in 277 border regions of Western Europe 1995–2019
Source: Eur J Epidemiol. 2025 Jul 19;40(9):1031–43. doi: 10.1007/s10654-025-01279-w (PMC12537618; doi:10.1007/s10654-025-01279-w)
Supplement: Supplementary file 2 — Supplementary Material 2 [file 10654_2025_1279_MOESM2_ESM.docx]

Table 1: Life expectancy at birth of Western European Border Regions

Men, 2019

|  |  | Men | | | | | | | |
| --- | --- | --- | --- | --- | --- | --- | --- | --- | --- |
|  |  | Life expectancy | | | Reference 3 | Reference 2 | | Reference 1 | |
|  | Border region | Life expectancy | 95% - Confidence Interval (lower) | 95% - Confidence Interval (upper) | Difference to the EU-12 average | Average all countries' border regions | Difference to countries' non-border regions | Average cross-border region | Difference to neighbouring cross-border region |
| Sweden | | | | | | | | | |
| Norrbottens län | FI_SE | 79.7 | 78.9 | 80.4 | -0.3 | 80.9 | -0.4 | 78.3 | -1.4 |
| Skåne län | DK_SE | 81.2 | 80.9 | 81.4 | 1.2 | 80.9 | -0.4 | 79.4 | -1.8 |
| Västra Götalands län | NA | 81.1 | 80.8 | 81.3 | 1.1 | 80.9 | -0.4 | NA | NA |
| Värmlands län | NA | 80.4 | 79.8 | 81.1 | 0.5 | 80.9 | -0.4 | NA | NA |
| Dalarnas län | NA | 81.1 | 80.5 | 81.6 | 1.1 | 80.9 | -0.4 | NA | NA |
| Jämtlands län | NA | 80.5 | 79.7 | 81.4 | 0.5 | 80.9 | -0.4 | NA | NA |
| Västerbottens län | NA | 80.9 | 80.2 | 81.5 | 0.9 | 80.9 | -0.4 | NA | NA |
| Finland | | | | | | | | | |
| Lappi | FI_SE | 78.3 | 77.4 | 79.2 | -1.7 | 78.3 | -0.9 | 79.7 | 1.4 |
| Denmark | | | | | | | | | |
| Byen København | DK_SE | 78.2 | 77.8 | 78.7 | -1.7 | 79.5 | 0.3 | 81.2 | 1.8 |
| Københavns omegn | DK_SE | 79.7 | 79.2 | 80.2 | -0.3 | 79.5 | 0.3 | 81.2 | 1.8 |
| Nordsjælland | DK_SE | 80.7 | 80.2 | 81.2 | 0.7 | 79.5 | 0.3 | 81.2 | 1.8 |
| Sydjylland | DK_DE | 79.8 | 79.4 | 80.2 | -0.2 | 79.5 | 0.3 | 78.0 | -1.8 |
| Germany | | | | | | | | | |
| Flensburg, Stadt | DK_DE | 76.9 | 75.8 | 78.1 | -3.1 | 78.7 | -0.1 | 79.8 | 1.8 |
| Nordfriesland | DK_DE | 78.2 | 77.4 | 79.1 | -1.7 | 78.7 | -0.1 | 79.8 | 1.8 |
| Schleswig-Flensburg | DK_DE | 78.6 | 77.8 | 79.3 | -1.4 | 78.7 | -0.1 | 79.8 | 1.8 |
| Emsland | DE_NL | 78.6 | 77.9 | 79.2 | -1.4 | 78.7 | -0.1 | 80.2 | 1.8 |
| Grafschaft Bentheim | DE_NL | 79.9 | 78.9 | 80.7 | -0.1 | 78.7 | -0.1 | 80.2 | 1.8 |
| Leer | DE_NL | 77.9 | 77.1 | 78.7 | -2.1 | 78.7 | -0.1 | 80.2 | 1.8 |
| Krefeld, Stadt | DE_NL | 77.3 | 76.6 | 78.1 | -2.6 | 78.7 | -0.1 | 80.2 | 1.8 |
| Mönchengladbach, Stadt | DE_NL | 77.3 | 76.6 | 78.0 | -2.7 | 78.7 | -0.1 | 80.2 | 1.8 |
| Kleve | DE_NL | 78.2 | 77.6 | 78.8 | -1.8 | 78.7 | -0.1 | 80.2 | 1.8 |
| Rhein-Kreis Neuss | DE_NL | 79.2 | 78.7 | 79.7 | -0.7 | 78.7 | -0.1 | 80.2 | 1.8 |
| Viersen | DE_NL | 79.6 | 79.0 | 80.2 | -0.4 | 78.7 | -0.1 | 80.2 | 1.8 |
| Wesel | DE_NL | 78.8 | 78.3 | 79.3 | -1.2 | 78.7 | -0.1 | 80.2 | 1.8 |
| Städteregion Aachen | DE_NL | 79.0 | 78.5 | 79.4 | -1.0 | 78.7 | -0.1 | 80.2 | 1.8 |
| Düren | DE_NL | 78.1 | 77.5 | 78.7 | -1.8 | 78.7 | -0.1 | 80.2 | 1.8 |
| Rhein-Erft-Kreis | DE_NL | 79.1 | 78.6 | 79.6 | -0.8 | 78.7 | -0.1 | 80.2 | 1.8 |
| Heinsberg | DE_NL | 78.9 | 78.2 | 79.5 | -1.0 | 78.7 | -0.1 | 80.2 | 1.8 |
| Borken | DE_NL | 79.2 | 78.7 | 79.8 | -0.7 | 78.7 | -0.1 | 80.2 | 1.8 |
| Coesfeld | DE_NL | 79.9 | 79.2 | 80.6 | -0.1 | 78.7 | -0.1 | 80.2 | 1.8 |
| Recklinghausen | DE_NL | 77.7 | 77.3 | 78.2 | -2.2 | 78.7 | -0.1 | 80.2 | 1.8 |
| Steinfurt | DE_NL | 79.0 | 78.6 | 79.6 | -0.9 | 78.7 | -0.1 | 80.2 | 1.8 |
| Städteregion Aachen | BE_DE | 79.0 | 78.5 | 79.4 | -1.0 | 78.7 | -0.1 | 79.1 | 0.4 |
| Düren | BE_DE | 78.1 | 77.5 | 78.7 | -1.8 | 78.7 | -0.1 | 79.1 | 0.4 |
| Rhein-Erft-Kreis | BE_DE | 79.1 | 78.6 | 79.6 | -0.8 | 78.7 | -0.1 | 79.1 | 0.4 |
| Euskirchen | BE_DE | 78.4 | 77.7 | 79.2 | -1.5 | 78.7 | -0.1 | 79.1 | 0.4 |
| Eifelkreis Bitburg-Prüm | BE_DE | 79.2 | 78.1 | 80.2 | -0.8 | 78.7 | -0.1 | 79.1 | 0.4 |
| Vulkaneifel | BE_DE | 79.5 | 78.1 | 80.6 | -0.5 | 78.7 | -0.1 | 79.1 | 0.4 |
| Regionalverband Saarbrücken | DE_FR | 77.5 | 76.9 | 78.1 | -2.4 | 78.7 | -0.1 | 79.7 | 0.6 |
| Merzig-Wadern | DE_FR | 78.0 | 77.0 | 79.1 | -2.0 | 78.7 | -0.1 | 79.7 | 0.6 |
| Neunkirchen | DE_FR | 77.2 | 76.3 | 78.2 | -2.7 | 78.7 | -0.1 | 79.7 | 0.6 |
| Saarlouis | DE_FR | 78.2 | 77.5 | 79.0 | -1.7 | 78.7 | -0.1 | 79.7 | 0.6 |
| Saarpfalz-Kreis | DE_FR | 79.0 | 78.3 | 80.0 | -0.9 | 78.7 | -0.1 | 79.7 | 0.6 |
| St. Wendel | DE_FR | 79.3 | 78.3 | 80.4 | -0.6 | 78.7 | -0.1 | 79.7 | 0.6 |
| Landau in der Pfalz, kreisfreie Stadt | DE_FR | 79.2 | 77.6 | 80.8 | -0.8 | 78.7 | -0.1 | 79.7 | 0.6 |
| Pirmasens, kreisfreie Stadt | DE_FR | 76.3 | 74.6 | 77.9 | -3.7 | 78.7 | -0.1 | 79.7 | 0.6 |
| Zweibrücken, kreisfreie Stadt | DE_FR | 78.5 | 76.7 | 80.3 | -1.5 | 78.7 | -0.1 | 79.7 | 0.6 |
| Germersheim | DE_FR | 79.3 | 78.4 | 80.2 | -0.7 | 78.7 | -0.1 | 79.7 | 0.6 |
| Kaiserslautern | DE_FR | 79.3 | 78.4 | 80.3 | -0.7 | 78.7 | -0.1 | 79.7 | 0.6 |
| Kusel | DE_FR | 78.7 | 77.3 | 80.0 | -1.3 | 78.7 | -0.1 | 79.7 | 0.6 |
| Südliche Weinstraße | DE_FR | 79.7 | 78.7 | 80.8 | -0.2 | 78.7 | -0.1 | 79.7 | 0.6 |
| Südwestpfalz | DE_FR | 79.7 | 78.7 | 80.8 | -0.2 | 78.7 | -0.1 | 79.7 | 0.6 |
| Baden-Baden, Stadtkreis | DE_FR | 79.5 | 78.0 | 81.0 | -0.4 | 78.7 | -0.1 | 79.7 | 0.6 |
| Karlsruhe, Stadtkreis | DE_FR | 79.6 | 78.9 | 80.2 | -0.4 | 78.7 | -0.1 | 79.7 | 0.6 |
| Karlsruhe | DE_FR | 80.0 | 79.5 | 80.5 | 0.0 | 78.7 | -0.1 | 79.7 | 0.6 |
| Rastatt | DE_FR | 79.7 | 78.9 | 80.3 | -0.3 | 78.7 | -0.1 | 79.7 | 0.6 |
| Calw | DE_FR | 80.0 | 79.2 | 80.9 | 0.1 | 78.7 | -0.1 | 79.7 | 0.6 |
| Enzkreis | DE_FR | 80.9 | 80.1 | 81.6 | 0.9 | 78.7 | -0.1 | 79.7 | 0.6 |
| Freudenstadt | DE_FR | 79.9 | 79.0 | 80.8 | 0.0 | 78.7 | -0.1 | 79.7 | 0.6 |
| Freiburg im Breisgau, Stadtkreis | DE_FR | 80.4 | 79.6 | 81.2 | 0.4 | 78.7 | -0.1 | 79.7 | 0.6 |
| Breisgau-Hochschwarzwald | DE_FR | 81.4 | 80.7 | 82.0 | 1.4 | 78.7 | -0.1 | 79.7 | 0.6 |
| Emmendingen | DE_FR | 80.8 | 80.0 | 81.5 | 0.8 | 78.7 | -0.1 | 79.7 | 0.6 |
| Ortenaukreis | DE_FR | 79.9 | 79.4 | 80.3 | -0.1 | 78.7 | -0.1 | 79.7 | 0.6 |
| Lörrach | DE_FR | 79.8 | 79.1 | 80.6 | -0.1 | 78.7 | -0.1 | 79.7 | 0.6 |
| Bodenseekreis | DE_AT | 80.9 | 80.2 | 81.7 | 1.0 | 78.7 | -0.1 | 80.3 | 0.5 |
| Ravensburg | DE_AT | 80.6 | 79.9 | 81.2 | 0.6 | 78.7 | -0.1 | 80.3 | 0.5 |
| Rosenheim | DE_AT | 79.5 | 78.2 | 81.0 | -0.5 | 78.7 | -0.1 | 80.3 | 0.5 |
| Rosenheim | DE_AT | 80.3 | 79.7 | 80.9 | 0.4 | 78.7 | -0.1 | 80.3 | 0.5 |
| Altötting | DE_AT | 79.2 | 78.2 | 80.3 | -0.7 | 78.7 | -0.1 | 80.3 | 0.5 |
| Berchtesgadener Land | DE_AT | 80.1 | 79.0 | 81.1 | 0.2 | 78.7 | -0.1 | 80.3 | 0.5 |
| Bad Tölz-Wolfratshausen | DE_AT | 80.5 | 79.6 | 81.4 | 0.5 | 78.7 | -0.1 | 80.3 | 0.5 |
| Garmisch-Partenkirchen | DE_AT | 80.3 | 79.3 | 81.4 | 0.4 | 78.7 | -0.1 | 80.3 | 0.5 |
| Miesbach | DE_AT | 80.5 | 79.5 | 81.6 | 0.6 | 78.7 | -0.1 | 80.3 | 0.5 |
| Mühldorf a.Inn | DE_AT | 78.2 | 77.3 | 79.3 | -1.8 | 78.7 | -0.1 | 80.3 | 0.5 |
| Rosenheim | DE_AT | 79.5 | 78.2 | 81.0 | -0.5 | 78.7 | -0.1 | 80.3 | 0.5 |
| Rosenheim | DE_AT | 80.3 | 79.7 | 80.9 | 0.4 | 78.7 | -0.1 | 80.3 | 0.5 |
| Traunstein | DE_AT | 80.1 | 79.3 | 80.9 | 0.1 | 78.7 | -0.1 | 80.3 | 0.5 |
| Weilheim-Schongau | DE_AT | 80.6 | 79.7 | 81.5 | 0.7 | 78.7 | -0.1 | 80.3 | 0.5 |
| Passau | DE_AT | 78.7 | 77.1 | 80.2 | -1.2 | 78.7 | -0.1 | 80.3 | 0.5 |
| Passau | DE_AT | 79.1 | 78.3 | 79.9 | -0.9 | 78.7 | -0.1 | 80.3 | 0.5 |
| Deggendorf | DE_AT | 78.5 | 77.4 | 79.4 | -1.5 | 78.7 | -0.1 | 80.3 | 0.5 |
| Freyung-Grafenau | DE_AT | 78.4 | 77.2 | 79.5 | -1.6 | 78.7 | -0.1 | 80.3 | 0.5 |
| Passau | DE_AT | 78.7 | 77.1 | 80.2 | -1.2 | 78.7 | -0.1 | 80.3 | 0.5 |
| Passau | DE_AT | 79.1 | 78.3 | 79.9 | -0.9 | 78.7 | -0.1 | 80.3 | 0.5 |
| Rottal-Inn | DE_AT | 79.0 | 77.9 | 79.9 | -1.0 | 78.7 | -0.1 | 80.3 | 0.5 |
| Kempten (Allgäu) | DE_AT | 79.3 | 78.0 | 80.7 | -0.7 | 78.7 | -0.1 | 80.3 | 0.5 |
| Lindau (Bodensee) | DE_AT | 80.0 | 78.7 | 81.2 | 0.0 | 78.7 | -0.1 | 80.3 | 0.5 |
| Ostallgäu | DE_AT | 80.8 | 79.8 | 81.7 | 0.8 | 78.7 | -0.1 | 80.3 | 0.5 |
| Oberallgäu | DE_AT | 81.0 | 80.1 | 81.8 | 1.0 | 78.7 | -0.1 | 80.3 | 0.5 |
| Schwarzwald-Baar-Kreis | DE_CH | 80.0 | 79.2 | 80.7 | 0.0 | 78.7 | -0.1 | 82.1 | 2.2 |
| Tuttlingen | DE_CH | 79.9 | 78.9 | 80.7 | -0.1 | 78.7 | -0.1 | 82.1 | 2.2 |
| Konstanz | DE_CH | 80.4 | 79.8 | 81.0 | 0.4 | 78.7 | -0.1 | 82.1 | 2.2 |
| Lörrach | DE_CH | 79.8 | 79.1 | 80.6 | -0.1 | 78.7 | -0.1 | 82.1 | 2.2 |
| Waldshut | DE_CH | 79.8 | 78.9 | 80.6 | -0.2 | 78.7 | -0.1 | 82.1 | 2.2 |
| Bodenseekreis | DE_CH | 80.9 | 80.2 | 81.7 | 1.0 | 78.7 | -0.1 | 82.1 | 2.2 |
| Ravensburg | DE_CH | 80.6 | 79.9 | 81.2 | 0.6 | 78.7 | -0.1 | 82.1 | 2.2 |
| Sigmaringen | DE_CH | 79.8 | 78.8 | 80.7 | -0.2 | 78.7 | -0.1 | 82.1 | 2.2 |
| Lindau (Bodensee) | DE_CH | 80.0 | 78.7 | 81.2 | 0.0 | 78.7 | -0.1 | 82.1 | 2.2 |
| Cottbus, Stadt | NA | 77.2 | 76.0 | 78.4 | -2.8 | 78.7 | -0.1 | NA | NA |
| Frankfurt (Oder), Stadt | NA | 77.5 | 76.0 | 79.1 | -2.4 | 78.7 | -0.1 | NA | NA |
| Barnim | NA | 78.4 | 77.6 | 79.1 | -1.6 | 78.7 | -0.1 | NA | NA |
| Dahme-Spreewald | NA | 78.9 | 78.2 | 79.6 | -1.1 | 78.7 | -0.1 | NA | NA |
| Märkisch-Oderland | NA | 78.9 | 78.1 | 79.6 | -1.1 | 78.7 | -0.1 | NA | NA |
| Oder-Spree | NA | 77.8 | 77.0 | 78.6 | -2.2 | 78.7 | -0.1 | NA | NA |
| Spree-Neiße | NA | 77.4 | 76.4 | 78.4 | -2.5 | 78.7 | -0.1 | NA | NA |
| Uckermark | NA | 76.7 | 75.5 | 77.6 | -3.3 | 78.7 | -0.1 | NA | NA |
| Vorpommern-Greifswald | NA | 75.9 | 75.1 | 76.5 | -4.1 | 78.7 | -0.1 | NA | NA |
| Erzgebirgskreis | NA | 77.9 | 77.4 | 78.5 | -2.0 | 78.7 | -0.1 | NA | NA |
| Mittelsachsen | NA | 78.1 | 77.6 | 78.7 | -1.9 | 78.7 | -0.1 | NA | NA |
| Vogtlandkreis | NA | 77.1 | 76.4 | 77.8 | -2.9 | 78.7 | -0.1 | NA | NA |
| Zwickau | NA | 77.3 | 76.7 | 77.9 | -2.7 | 78.7 | -0.1 | NA | NA |
| Dresden, Stadt | NA | 79.7 | 79.2 | 80.2 | -0.3 | 78.7 | -0.1 | NA | NA |
| Bautzen | NA | 78.2 | 77.5 | 78.8 | -1.8 | 78.7 | -0.1 | NA | NA |
| Görlitz | NA | 77.5 | 76.8 | 78.1 | -2.5 | 78.7 | -0.1 | NA | NA |
| Sächsische Schweiz-Osterzgebirge | NA | 78.7 | 78.0 | 79.3 | -1.3 | 78.7 | -0.1 | NA | NA |
| Saale-Orla-Kreis | NA | 77.9 | 76.6 | 79.0 | -2.1 | 78.7 | -0.1 | NA | NA |
| Trier, kreisfreie Stadt | NA | 78.8 | 77.7 | 79.8 | -1.2 | 78.7 | -0.1 | NA | NA |
| Bernkastel-Wittlich | NA | 79.6 | 78.6 | 80.7 | -0.3 | 78.7 | -0.1 | NA | NA |
| Trier-Saarburg | NA | 80.0 | 79.1 | 80.8 | 0.0 | 78.7 | -0.1 | NA | NA |
| Regen | NA | 77.9 | 76.7 | 79.0 | -2.1 | 78.7 | -0.1 | NA | NA |
| Weiden i.d.OPf. | NA | 77.9 | 76.1 | 79.6 | -2.1 | 78.7 | -0.1 | NA | NA |
| Cham | NA | 78.6 | 77.6 | 79.5 | -1.3 | 78.7 | -0.1 | NA | NA |
| Neustadt a.d.Waldnaab | NA | 78.9 | 77.8 | 79.8 | -1.1 | 78.7 | -0.1 | NA | NA |
| Schwandorf | NA | 78.6 | 77.7 | 79.5 | -1.4 | 78.7 | -0.1 | NA | NA |
| Tirschenreuth | NA | 77.9 | 76.7 | 79.1 | -2.1 | 78.7 | -0.1 | NA | NA |
| Hof | NA | 76.7 | 74.9 | 78.3 | -3.2 | 78.7 | -0.1 | NA | NA |
| Bayreuth | NA | 79.0 | 78.0 | 80.0 | -1.0 | 78.7 | -0.1 | NA | NA |
| Hof | NA | 78.4 | 77.4 | 79.4 | -1.5 | 78.7 | -0.1 | NA | NA |
| Wunsiedel i.Fichtelgebirge | NA | 77.7 | 76.4 | 78.9 | -2.3 | 78.7 | -0.1 | NA | NA |
| The Netherlands | | | | | | | | | |
| Oost-Groningen | DE_NL | 79.4 | 78.4 | 80.3 | -0.6 | 80.3 | -0.4 | 78.4 | -1.8 |
| Delfzijl en omgeving | DE_NL | 79.4 | 77.5 | 81.0 | -0.6 | 80.3 | -0.4 | 78.4 | -1.8 |
| Zuidoost-Drenthe | DE_NL | 80.1 | 79.2 | 80.9 | 0.1 | 80.3 | -0.4 | 78.4 | -1.8 |
| Noord-Overijssel | DE_NL | 81.1 | 80.5 | 81.7 | 1.2 | 80.3 | -0.4 | 78.4 | -1.8 |
| Twente | DE_NL | 80.1 | 79.7 | 80.5 | 0.1 | 80.3 | -0.4 | 78.4 | -1.8 |
| Achterhoek | DE_NL | 80.7 | 80.2 | 81.2 | 0.7 | 80.3 | -0.4 | 78.4 | -1.8 |
| Arnhem/Nijmegen | DE_NL | 80.5 | 80.1 | 80.9 | 0.5 | 80.3 | -0.4 | 78.4 | -1.8 |
| Noord-Limburg | DE_NL | 81.3 | 80.7 | 81.9 | 1.4 | 80.3 | -0.4 | 78.4 | -1.8 |
| Midden-Limburg | DE_NL | 80.8 | 80.1 | 81.5 | 0.8 | 80.3 | -0.4 | 78.4 | -1.8 |
| Zuid-Limburg | DE_NL | 79.7 | 79.2 | 80.1 | -0.3 | 80.3 | -0.4 | 78.4 | -1.8 |
| Zeeuwsch-Vlaanderen | BE_NL | 80.6 | 79.6 | 81.6 | 0.7 | 80.3 | -0.4 | 80.0 | -0.3 |
| Overig Zeeland | BE_NL | 81.2 | 80.6 | 81.9 | 1.3 | 80.3 | -0.4 | 80.0 | -0.3 |
| West-Noord-Brabant | BE_NL | 80.5 | 80.0 | 80.9 | 0.5 | 80.3 | -0.4 | 80.0 | -0.3 |
| Midden-Noord-Brabant | BE_NL | 80.6 | 80.1 | 81.0 | 0.6 | 80.3 | -0.4 | 80.0 | -0.3 |
| Zuidoost-Noord-Brabant | BE_NL | 80.8 | 80.5 | 81.2 | 0.9 | 80.3 | -0.4 | 80.0 | -0.3 |
| Midden-Limburg | BE_NL | 80.8 | 80.1 | 81.5 | 0.8 | 80.3 | -0.4 | 80.0 | -0.3 |
| Zuid-Limburg | BE_NL | 79.7 | 79.2 | 80.1 | -0.3 | 80.3 | -0.4 | 80.0 | -0.3 |
| Belgium | | | | | | | | | |
| Arr. Antwerpen | BE_NL | 80.4 | 80.0 | 80.7 | 0.4 | 79.4 | -0.3 | 80.3 | 0.3 |
| Arr. Turnhout | BE_NL | 81.0 | 80.5 | 81.5 | 1.0 | 79.4 | -0.3 | 80.3 | 0.3 |
| Arr. Tongeren | BE_NL | 80.7 | 79.9 | 81.4 | 0.7 | 79.4 | -0.3 | 80.3 | 0.3 |
| Arr. Maaseik | BE_NL | 81.4 | 80.8 | 82.1 | 1.5 | 79.4 | -0.3 | 80.3 | 0.3 |
| Arr. Eeklo | BE_NL | 80.3 | 79.1 | 81.5 | 0.3 | 79.4 | -0.3 | 80.3 | 0.3 |
| Arr. Gent | BE_NL | 80.3 | 79.8 | 80.7 | 0.3 | 79.4 | -0.3 | 80.3 | 0.3 |
| Arr. Sint-Niklaas | BE_NL | 80.4 | 79.7 | 81.0 | 0.4 | 79.4 | -0.3 | 80.3 | 0.3 |
| Arr. Brugge | BE_NL | 80.7 | 80.0 | 81.3 | 0.7 | 79.4 | -0.3 | 80.3 | 0.3 |
| Arr. Liège | BE_NL | 77.3 | 76.9 | 77.8 | -2.6 | 79.4 | -0.3 | 80.3 | 0.3 |
| Bezirk Verviers ? Deutschsprachige Gemeinschaft | BE_NL | 79.1 | 78.5 | 79.8 | -0.9 | 79.4 | -0.3 | 80.3 | 0.3 |
| Bezirk Verviers ? Deutschsprachige Gemeinschaft | BE_DE | 79.1 | 78.5 | 79.8 | -0.9 | 79.4 | -0.3 | 78.7 | -0.4 |
| Arr. Ieper | BE_FR | 80.3 | 79.2 | 81.5 | 0.3 | 79.4 | -0.3 | 78.1 | 0.1 |
| Arr. Kortrijk | BE_FR | 79.9 | 79.2 | 80.5 | -0.1 | 79.4 | -0.3 | 78.1 | 0.1 |
| Arr. Veurne | BE_FR | 80.0 | 78.5 | 81.4 | 0.0 | 79.4 | -0.3 | 78.1 | 0.1 |
| Arr. Mons | BE_FR | 75.9 | 75.2 | 76.6 | -4.1 | 79.4 | -0.3 | 78.1 | 0.1 |
| Arr. Tournai-Mouscron | BE_FR | 76.4 | 75.6 | 77.2 | -3.6 | 79.4 | -0.3 | 78.1 | 0.1 |
| Arr. Ath | BE_FR | 78.1 | 77.4 | 78.7 | -1.9 | 79.4 | -0.3 | 78.1 | 0.1 |
| Arr. Neufchâteau | BE_FR | 77.1 | 75.5 | 78.6 | -2.9 | 79.4 | -0.3 | 78.1 | 0.1 |
| Arr. Virton | BE_FR | 77.9 | 76.3 | 79.7 | -2.1 | 79.4 | -0.3 | 78.1 | 0.1 |
| Arr. Dinant | BE_FR | 76.6 | 75.5 | 77.7 | -3.4 | 79.4 | -0.3 | 78.1 | 0.1 |
| Arr. Philippeville | BE_FR | 76.0 | 74.6 | 77.4 | -3.9 | 79.4 | -0.3 | 78.1 | 0.1 |
| Arr. Arlon | NA | 78.4 | 76.9 | 79.8 | -1.5 | 79.4 | -0.3 | NA | NA |
| Arr. Bastogne | NA | 77.4 | 75.8 | 79.0 | -2.5 | 79.4 | -0.3 | NA | NA |
| France | | | | | | | | | |
| Moselle | DE_FR | 79.0 | 78.7 | 79.4 | -0.9 | 79.6 | -0.3 | 79.1 | -0.6 |
| Bas-Rhin | DE_FR | 80.2 | 79.9 | 80.6 | 0.2 | 79.6 | -0.3 | 79.1 | -0.6 |
| Haut-Rhin | DE_FR | 80.3 | 79.9 | 80.7 | 0.4 | 79.6 | -0.3 | 79.1 | -0.6 |
| Aisne | BE_FR | 77.5 | 77.0 | 78.1 | -2.4 | 79.6 | -0.3 | 78.0 | -0.1 |
| Meurthe-et-Moselle | BE_FR | 79.6 | 79.2 | 80.1 | -0.3 | 79.6 | -0.3 | 78.0 | -0.1 |
| Meuse | BE_FR | 78.7 | 77.7 | 79.5 | -1.3 | 79.6 | -0.3 | 78.0 | -0.1 |
| Nord | BE_FR | 77.8 | 77.5 | 78.0 | -2.2 | 79.6 | -0.3 | 78.0 | -0.1 |
| Ardennes | BE_FR | 78.3 | 77.6 | 79.1 | -1.7 | 79.6 | -0.3 | 78.0 | -0.1 |
| Ain | FR_CH | 81.3 | 80.8 | 81.7 | 1.3 | 79.6 | -0.3 | 82.0 | 1.5 |
| Doubs | FR_CH | 80.1 | 79.6 | 80.6 | 0.1 | 79.6 | -0.3 | 82.0 | 1.5 |
| Jura | FR_CH | 79.9 | 79.1 | 80.7 | 0.0 | 79.6 | -0.3 | 82.0 | 1.5 |
| Haut-Rhin | FR_CH | 80.3 | 79.9 | 80.7 | 0.4 | 79.6 | -0.3 | 82.0 | 1.5 |
| Haute-Saône | FR_CH | 79.2 | 78.4 | 80.0 | -0.7 | 79.6 | -0.3 | 82.0 | 1.5 |
| Haute-Savoie | FR_CH | 81.5 | 81.1 | 81.9 | 1.5 | 79.6 | -0.3 | 82.0 | 1.5 |
| Territoire de Belfort | FR_CH | 79.7 | 78.6 | 80.6 | -0.3 | 79.6 | -0.3 | 82.0 | 1.5 |
| Alpes-de-Haute-Provence | IT_FR | 79.9 | 79.0 | 80.8 | 0.0 | 79.6 | -0.3 | 81.3 | 0.5 |
| Hautes-Alpes | IT_FR | 81.4 | 80.4 | 82.3 | 1.4 | 79.6 | -0.3 | 81.3 | 0.5 |
| Alpes-Maritimes | IT_FR | 80.5 | 80.1 | 80.8 | 0.5 | 79.6 | -0.3 | 81.3 | 0.5 |
| Savoie | IT_FR | 81.1 | 80.6 | 81.7 | 1.2 | 79.6 | -0.3 | 81.3 | 0.5 |
| Haute-Savoie | IT_FR | 81.5 | 81.1 | 81.9 | 1.5 | 79.6 | -0.3 | 81.3 | 0.5 |
| Haute-Garonne | FR_ES | 81.5 | 81.2 | 81.8 | 1.5 | 79.6 | -0.3 | 81.4 | 0.8 |
| Pyrénées-Atlantiques | FR_ES | 80.8 | 80.4 | 81.2 | 0.8 | 79.6 | -0.3 | 81.4 | 0.8 |
| Hautes-Pyrénées | FR_ES | 79.7 | 78.9 | 80.5 | -0.3 | 79.6 | -0.3 | 81.4 | 0.8 |
| Pyrénées-Orientales | FR_ES | 79.2 | 78.7 | 79.8 | -0.8 | 79.6 | -0.3 | 81.4 | 0.8 |
| Ariège | FR_ES | 79.3 | 78.2 | 80.2 | -0.7 | 79.6 | -0.3 | 81.4 | 0.8 |
| Switzerland | | | | | | | | | |
| Vaud | FR_CH | 82.2 | 81.8 | 82.6 | 2.3 | 82.1 | 0.2 | 80.5 | -1.5 |
| Valais | FR_CH | 81.6 | 80.9 | 82.1 | 1.6 | 82.1 | 0.2 | 80.5 | -1.5 |
| Genève | FR_CH | 81.0 | 80.1 | 81.9 | 1.0 | 82.1 | 0.2 | 80.5 | -1.5 |
| Solothurn | FR_CH | 81.8 | 81.2 | 82.4 | 1.8 | 82.1 | 0.2 | 80.5 | -1.5 |
| Neuchâtel | FR_CH | 83.2 | 82.7 | 83.7 | 3.2 | 82.1 | 0.2 | 80.5 | -1.5 |
| Jura | FR_CH | 81.0 | 79.7 | 82.3 | 1.1 | 82.1 | 0.2 | 80.5 | -1.5 |
| Basel-Stadt | FR_CH | 81.2 | 80.4 | 82.0 | 1.2 | 82.1 | 0.2 | 80.5 | -1.5 |
| Basel-Landschaft | FR_CH | 82.9 | 82.2 | 83.5 | 2.9 | 82.1 | 0.2 | 80.5 | -1.5 |
| Appenzell Ausserrhoden | AT_CH | 81.7 | 80.1 | 83.2 | 1.7 | 82.1 | 0.2 | 80.6 | -1.4 |
| Appenzell Innerrhoden | AT_CH | 81.7 | 78.6 | 84.3 | 1.7 | 82.1 | 0.2 | 80.6 | -1.4 |
| St. Gallen | AT_CH | 82.0 | 81.6 | 82.5 | 2.1 | 82.1 | 0.2 | 80.6 | -1.4 |
| Graubünden | AT_CH | 82.1 | 81.3 | 82.8 | 2.1 | 82.1 | 0.2 | 80.6 | -1.4 |
| Basel-Stadt | DE_CH | 81.2 | 80.4 | 82.0 | 1.2 | 82.1 | 0.2 | 79.9 | -2.2 |
| Basel-Landschaft | DE_CH | 82.9 | 82.2 | 83.5 | 2.9 | 82.1 | 0.2 | 79.9 | -2.2 |
| Aargau | DE_CH | 82.4 | 81.9 | 82.8 | 2.4 | 82.1 | 0.2 | 79.9 | -2.2 |
| Zürich | DE_CH | 82.4 | 82.1 | 82.7 | 2.4 | 82.1 | 0.2 | 79.9 | -2.2 |
| Schaffhausen | DE_CH | 81.9 | 80.8 | 83.0 | 2.0 | 82.1 | 0.2 | 79.9 | -2.2 |
| Appenzell Ausserrhoden | DE_CH | 81.7 | 80.1 | 83.2 | 1.7 | 82.1 | 0.2 | 79.9 | -2.2 |
| Appenzell Innerrhoden | DE_CH | 81.7 | 78.6 | 84.3 | 1.7 | 82.1 | 0.2 | 79.9 | -2.2 |
| St. Gallen | DE_CH | 82.0 | 81.6 | 82.5 | 2.1 | 82.1 | 0.2 | 79.9 | -2.2 |
| Thurgau | DE_CH | 82.2 | 81.5 | 82.9 | 2.2 | 82.1 | 0.2 | 79.9 | -2.2 |
| Valais | IT_CH | 81.6 | 80.9 | 82.1 | 1.6 | 82.1 | 0.2 | 81.5 | -0.6 |
| Graubünden | IT_CH | 82.1 | 81.3 | 82.8 | 2.1 | 82.1 | 0.2 | 81.5 | -0.6 |
| Ticino | IT_CH | 82.9 | 82.3 | 83.4 | 2.9 | 82.1 | 0.2 | 81.5 | -0.6 |
| Austria | | | | | | | | | |
| Landeck | AT_CH | 81.0 | 79.3 | 82.5 | 1.0 | 79.8 | 0.6 | 82.0 | 1.4 |
| Bludenz | AT_CH | 80.6 | 79.2 | 82.0 | 0.6 | 79.8 | 0.6 | 82.0 | 1.4 |
| Bregenz | AT_CH | 80.4 | 79.6 | 81.5 | 0.5 | 79.8 | 0.6 | 82.0 | 1.4 |
| Dornbirn | AT_CH | 80.2 | 78.9 | 81.5 | 0.2 | 79.8 | 0.6 | 82.0 | 1.4 |
| Feldkirch | AT_CH | 81.0 | 79.9 | 82.1 | 1.0 | 79.8 | 0.6 | 82.0 | 1.4 |
| Braunau am Inn | DE_AT | 79.9 | 78.8 | 80.9 | -0.1 | 79.8 | 0.6 | 79.8 | -0.5 |
| Ried im Innkreis | DE_AT | 80.6 | 79.2 | 82.0 | 0.6 | 79.8 | 0.6 | 79.8 | -0.5 |
| Rohrbach | DE_AT | 80.3 | 78.8 | 81.8 | 0.3 | 79.8 | 0.6 | 79.8 | -0.5 |
| Schärding | DE_AT | 78.9 | 77.4 | 80.5 | -1.0 | 79.8 | 0.6 | 79.8 | -0.5 |
| Salzburg(Stadt) | DE_AT | 79.3 | 78.2 | 80.2 | -0.7 | 79.8 | 0.6 | 79.8 | -0.5 |
| Hallein | DE_AT | 80.7 | 79.3 | 82.3 | 0.8 | 79.8 | 0.6 | 79.8 | -0.5 |
| Salzburg-Umgebung | DE_AT | 81.4 | 80.5 | 82.3 | 1.4 | 79.8 | 0.6 | 79.8 | -0.5 |
| Sankt Johann im Pongau | DE_AT | 80.6 | 79.3 | 81.8 | 0.6 | 79.8 | 0.6 | 79.8 | -0.5 |
| Zell am See | DE_AT | 80.8 | 79.5 | 82.0 | 0.8 | 79.8 | 0.6 | 79.8 | -0.5 |
| Innsbruck-Stadt | DE_AT | 79.3 | 78.3 | 80.4 | -0.6 | 79.8 | 0.6 | 79.8 | -0.5 |
| Innsbruck-Land | DE_AT | 81.3 | 80.5 | 82.1 | 1.4 | 79.8 | 0.6 | 79.8 | -0.5 |
| Kitzbühel | DE_AT | 81.4 | 79.8 | 82.7 | 1.4 | 79.8 | 0.6 | 79.8 | -0.5 |
| Kufstein | DE_AT | 81.0 | 79.9 | 82.1 | 1.0 | 79.8 | 0.6 | 79.8 | -0.5 |
| Reutte | DE_AT | 80.0 | 78.1 | 81.7 | 0.0 | 79.8 | 0.6 | 79.8 | -0.5 |
| Schwaz | DE_AT | 81.2 | 80.0 | 82.4 | 1.3 | 79.8 | 0.6 | 79.8 | -0.5 |
| Bregenz | DE_AT | 80.4 | 79.6 | 81.5 | 0.5 | 79.8 | 0.6 | 79.8 | -0.5 |
| Hermagor | AT_IT | 80.4 | 77.6 | 83.0 | 0.4 | 79.8 | 0.6 | 81.9 | 1.2 |
| Zell am See | AT_IT | 80.8 | 79.5 | 82.0 | 0.8 | 79.8 | 0.6 | 81.9 | 1.2 |
| Innsbruck-Stadt | AT_IT | 79.3 | 78.3 | 80.4 | -0.6 | 79.8 | 0.6 | 81.9 | 1.2 |
| Imst | AT_IT | 80.6 | 79.3 | 82.1 | 0.7 | 79.8 | 0.6 | 81.9 | 1.2 |
| Innsbruck-Land | AT_IT | 81.3 | 80.5 | 82.1 | 1.4 | 79.8 | 0.6 | 81.9 | 1.2 |
| Landeck | AT_IT | 81.0 | 79.3 | 82.5 | 1.0 | 79.8 | 0.6 | 81.9 | 1.2 |
| Lienz | AT_IT | 80.4 | 78.7 | 82.1 | 0.5 | 79.8 | 0.6 | 81.9 | 1.2 |
| Schwaz | AT_IT | 81.2 | 80.0 | 82.4 | 1.3 | 79.8 | 0.6 | 81.9 | 1.2 |
| Eisenstadt-Umgebung und Rust | NA | 79.4 | 78.1 | 80.8 | -0.5 | 79.8 | 0.6 | NA | NA |
| Güssing | NA | 78.9 | 76.6 | 81.0 | -1.1 | 79.8 | 0.6 | NA | NA |
| Jennersdorf | NA | 79.7 | 77.1 | 82.0 | -0.2 | 79.8 | 0.6 | NA | NA |
| Mattersburg | NA | 79.6 | 78.1 | 81.2 | -0.4 | 79.8 | 0.6 | NA | NA |
| Neusiedl am See | NA | 79.8 | 78.4 | 81.1 | -0.1 | 79.8 | 0.6 | NA | NA |
| Oberpullendorf | NA | 79.1 | 77.3 | 80.8 | -0.8 | 79.8 | 0.6 | NA | NA |
| Oberwart | NA | 78.7 | 77.3 | 80.2 | -1.2 | 79.8 | 0.6 | NA | NA |
| Klagenfurt Stadt | NA | 78.3 | 77.0 | 79.5 | -1.7 | 79.8 | 0.6 | NA | NA |
| Villach Stadt | NA | 79.2 | 77.7 | 80.6 | -0.8 | 79.8 | 0.6 | NA | NA |
| Klagenfurt Land | NA | 79.7 | 78.2 | 81.1 | -0.3 | 79.8 | 0.6 | NA | NA |
| Villach Land | NA | 79.7 | 78.2 | 81.0 | -0.3 | 79.8 | 0.6 | NA | NA |
| Völkermarkt | NA | 79.1 | 77.4 | 80.7 | -0.9 | 79.8 | 0.6 | NA | NA |
| Wolfsberg | NA | 79.4 | 77.9 | 81.0 | -0.6 | 79.8 | 0.6 | NA | NA |
| Bruck an der Leitha | NA | 78.9 | 77.8 | 79.9 | -1.1 | 79.8 | 0.6 | NA | NA |
| Gänserndorf | NA | 79.3 | 78.3 | 80.4 | -0.6 | 79.8 | 0.6 | NA | NA |
| Gmünd | NA | 78.6 | 76.9 | 80.3 | -1.4 | 79.8 | 0.6 | NA | NA |
| Hollabrunn | NA | 79.3 | 77.7 | 80.7 | -0.6 | 79.8 | 0.6 | NA | NA |
| Horn | NA | 78.5 | 76.4 | 80.4 | -1.5 | 79.8 | 0.6 | NA | NA |
| Mistelbach | NA | 78.9 | 77.7 | 80.1 | -1.1 | 79.8 | 0.6 | NA | NA |
| Waidhofen an der Thaya | NA | 78.4 | 76.0 | 80.7 | -1.6 | 79.8 | 0.6 | NA | NA |
| Freistadt | NA | 79.9 | 78.6 | 81.2 | -0.1 | 79.8 | 0.6 | NA | NA |
| Urfahr-Umgebung | NA | 81.7 | 80.6 | 82.8 | 1.7 | 79.8 | 0.6 | NA | NA |
| Deutschlandsberg | NA | 79.5 | 78.1 | 80.8 | -0.5 | 79.8 | 0.6 | NA | NA |
| Leibnitz | NA | 79.3 | 78.1 | 80.4 | -0.7 | 79.8 | 0.6 | NA | NA |
| Südoststeiermark | NA | 80.4 | 79.3 | 81.4 | 0.4 | 79.8 | 0.6 | NA | NA |
| Italy | | | | | | | | | |
| Bolzano-Bozen | AT_IT | 82.6 | 82.1 | 83.0 | 2.6 | 81.4 | 0.1 | 80.7 | -1.2 |
| Belluno | AT_IT | 81.6 | 81.0 | 82.2 | 1.6 | 81.4 | 0.1 | 80.7 | -1.2 |
| Udine | AT_IT | 81.8 | 81.3 | 82.2 | 1.8 | 81.4 | 0.1 | 80.7 | -1.2 |
| Varese | IT_CH | 81.9 | 81.5 | 82.2 | 1.9 | 81.4 | 0.1 | 82.1 | 0.6 |
| Como | IT_CH | 82.1 | 81.8 | 82.4 | 2.1 | 81.4 | 0.1 | 82.1 | 0.6 |
| Sondrio | IT_CH | 81.0 | 80.3 | 81.7 | 1.0 | 81.4 | 0.1 | 82.1 | 0.6 |
| Vercelli | IT_CH | 81.2 | 80.7 | 81.7 | 1.3 | 81.4 | 0.1 | 82.1 | 0.6 |
| Novara | IT_CH | 81.4 | 80.9 | 81.8 | 1.4 | 81.4 | 0.1 | 82.1 | 0.6 |
| Valle d'Aosta/Vallée d'Aoste | IT_CH | 81.5 | 80.6 | 82.4 | 1.6 | 81.4 | 0.1 | 82.1 | 0.6 |
| Torino | IT_FR | 81.4 | 81.2 | 81.7 | 1.5 | 81.4 | 0.1 | 80.8 | -0.5 |
| Cuneo | IT_FR | 81.2 | 80.8 | 81.6 | 1.2 | 81.4 | 0.1 | 80.8 | -0.5 |
| Valle d'Aosta/Vallée d'Aoste | IT_FR | 81.5 | 80.6 | 82.4 | 1.6 | 81.4 | 0.1 | 80.8 | -0.5 |
| Imperia | IT_FR | 81.3 | 80.7 | 82.0 | 1.4 | 81.4 | 0.1 | 80.8 | -0.5 |
| Gorizia | NA | 81.6 | 80.8 | 82.3 | 1.6 | 81.4 | 0.1 | NA | NA |
| Trieste | NA | 81.3 | 80.6 | 81.9 | 1.3 | 81.4 | 0.1 | NA | NA |
| Spain | | | | | | | | | |
| Gipuzkoa | FR_ES | 81.4 | 81.0 | 81.7 | 1.4 | 81.0 | 0.0 | 80.6 | -0.8 |
| Navarra | FR_ES | 81.9 | 81.5 | 82.3 | 1.9 | 81.0 | 0.0 | 80.6 | -0.8 |
| Huesca | FR_ES | 81.5 | 80.9 | 82.2 | 1.5 | 81.0 | 0.0 | 80.6 | -0.8 |
| Girona | FR_ES | 81.1 | 80.7 | 81.5 | 1.1 | 81.0 | 0.0 | 80.6 | -0.8 |
| Lleida | FR_ES | 81.1 | 80.6 | 81.6 | 1.1 | 81.0 | 0.0 | 80.6 | -0.8 |
| Ourense | PT_ES | 81.0 | 80.4 | 81.6 | 1.0 | 81.0 | 0.0 | 78.7 | -1.9 |
| Pontevedra | PT_ES | 80.8 | 80.5 | 81.2 | 0.8 | 81.0 | 0.0 | 78.7 | -1.9 |
| Salamanca | PT_ES | 82.7 | 82.1 | 83.2 | 2.7 | 81.0 | 0.0 | 78.7 | -1.9 |
| Zamora | PT_ES | 81.4 | 80.6 | 82.1 | 1.4 | 81.0 | 0.0 | 78.7 | -1.9 |
| Badajoz | PT_ES | 79.8 | 79.4 | 80.2 | -0.2 | 81.0 | 0.0 | 78.7 | -1.9 |
| Cáceres | PT_ES | 80.8 | 80.2 | 81.3 | 0.8 | 81.0 | 0.0 | 78.7 | -1.9 |
| Huelva | PT_ES | 79.6 | 79.1 | 80.0 | -0.4 | 81.0 | 0.0 | 78.7 | -1.9 |
| Portugal | | | | | | | | | |
| Alto Minho | PT_ES | 79.2 | 78.4 | 79.9 | -0.8 | 78.7 | -0.3 | 80.6 | 1.9 |
| Algarve | PT_ES | 76.9 | 76.4 | 77.5 | -3.0 | 78.7 | -0.3 | 80.6 | 1.9 |
| Baixo Alentejo | PT_ES | 76.3 | 75.3 | 77.2 | -3.7 | 78.7 | -0.3 | 80.6 | 1.9 |
| Ave, Área Metropolitana do Porto, Alto Tâmega, Tâmega e Sousa, Douro, Terras de Trás-os-Montes | PT_ES | 79.1 | 78.9 | 79.3 | -0.8 | 78.7 | -0.3 | 80.6 | 1.9 |
| Beira Baixa, Médio Tejo | PT_ES | 78.6 | 78.0 | 79.2 | -1.3 | 78.7 | -0.3 | 80.6 | 1.9 |
| Beiras e Serra da Estrela | PT_ES | 78.7 | 78.0 | 79.5 | -1.2 | 78.7 | -0.3 | 80.6 | 1.9 |
| Alto Alentejo, Alentejo Central | PT_ES | 78.3 | 77.6 | 78.9 | -1.7 | 78.7 | -0.3 | 80.6 | 1.9 |
| Cávado | PT_ES | 80.8 | 80.3 | 81.4 | 0.9 | 78.7 | -0.3 | 80.6 | 1.9 |
