# Supplementary material for: Getting closer to each other? Convergence and divergence patterns of life expectancy in 277 border regions of Western Europe 1995–2019
Source: Eur J Epidemiol. 2025 Jul 19;40(9):1031–43. doi: 10.1007/s10654-025-01279-w (PMC12537618; doi:10.1007/s10654-025-01279-w)
Supplement: Supplementary file 4 — Supplementary Material 4 [file 10654_2025_1279_MOESM4_ESM.pdf]

Austria − Eisenstadt−Umgebung und Rust

Trendline of Life Expectancy by Sex, with smoothed and actual mortality rates

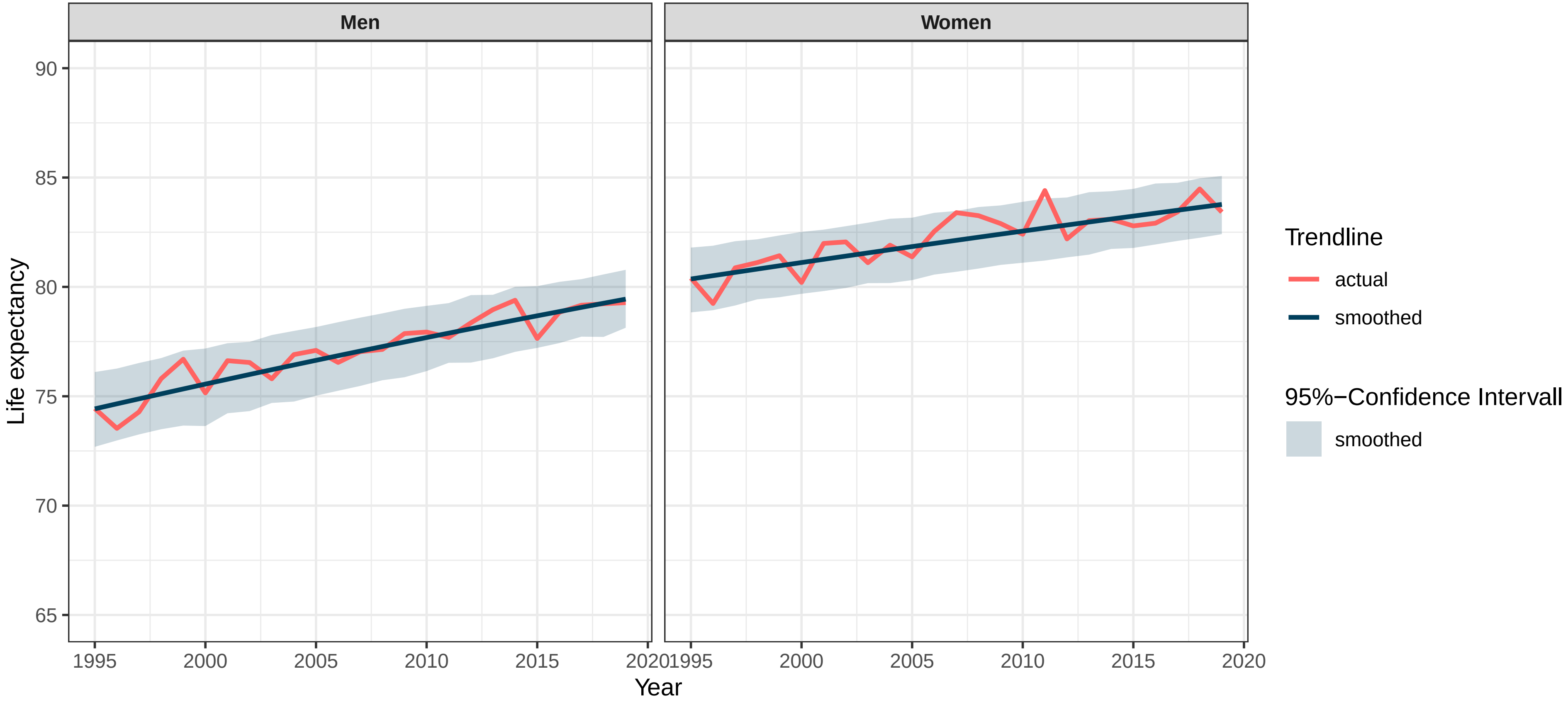

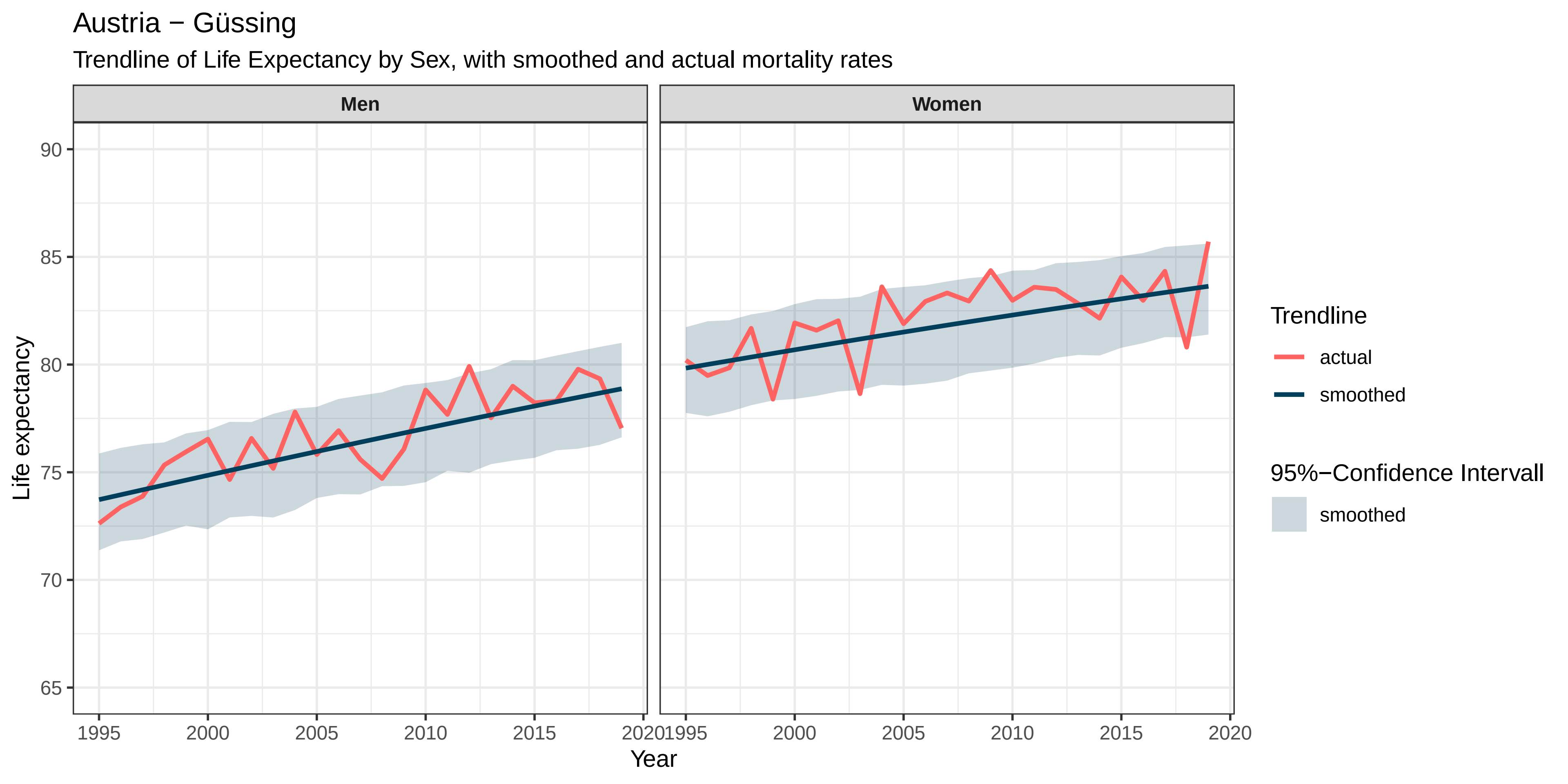

Austria − Güssing
Trendline of Life Expectancy by Sex, with smoothed and actual mortality rates
Men
Women
90
85
80
75
70
65
Life expectancy
1995
2000
2005
2010
2015
2020
Year
Trendline
actual
smoothed
95%−Confidence Intervall
smoothed

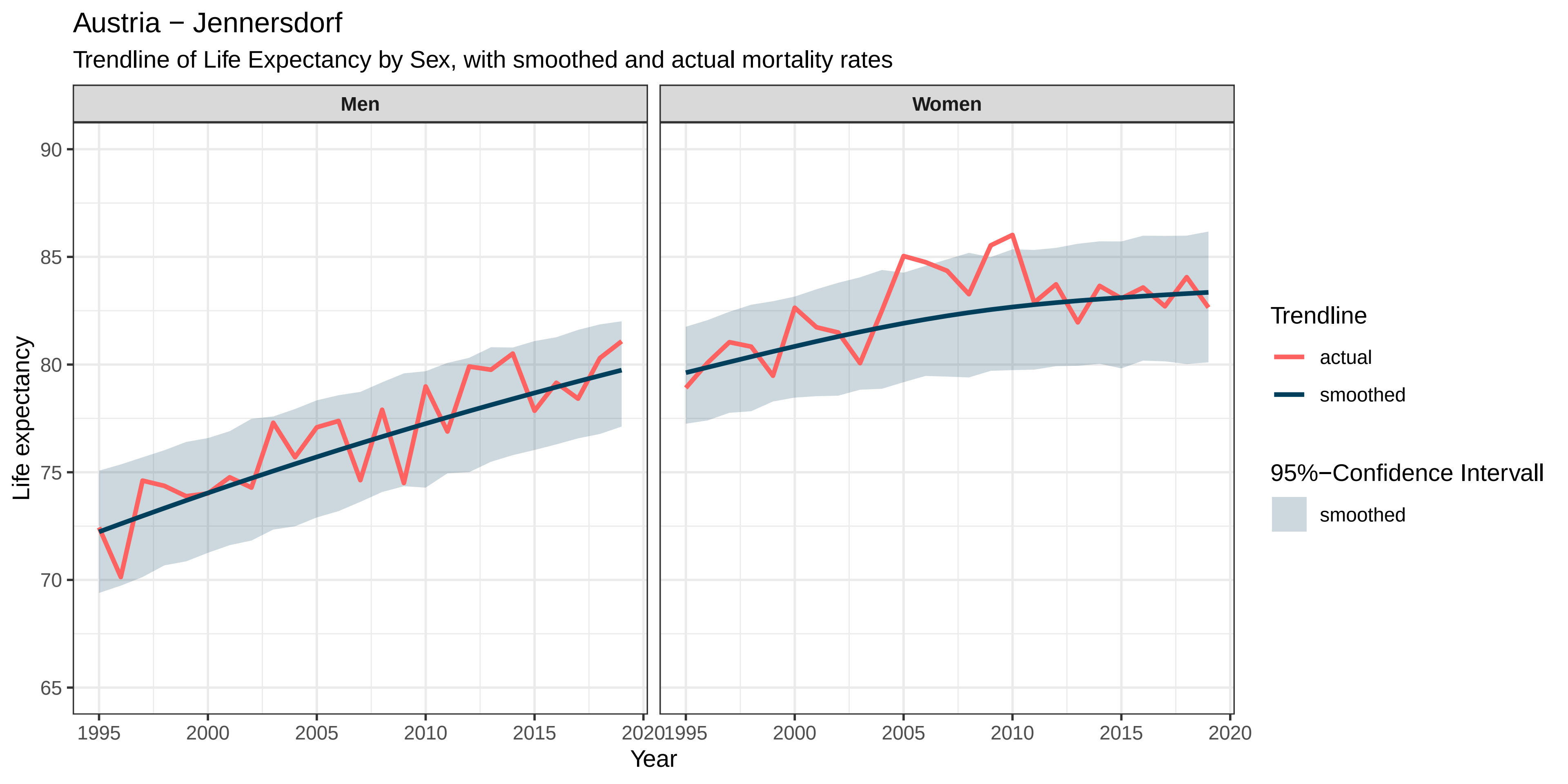
Austria − Jennersdorf
Trendline of Life Expectancy by Sex, with smoothed and actual mortality rates
Men
Women
90
85
80
75
70
65
Life expectancy
1995
2000
2005
2010
2015
2020
Year
Trendline
actual
smoothed
95%−Confidence Intervall
smoothed

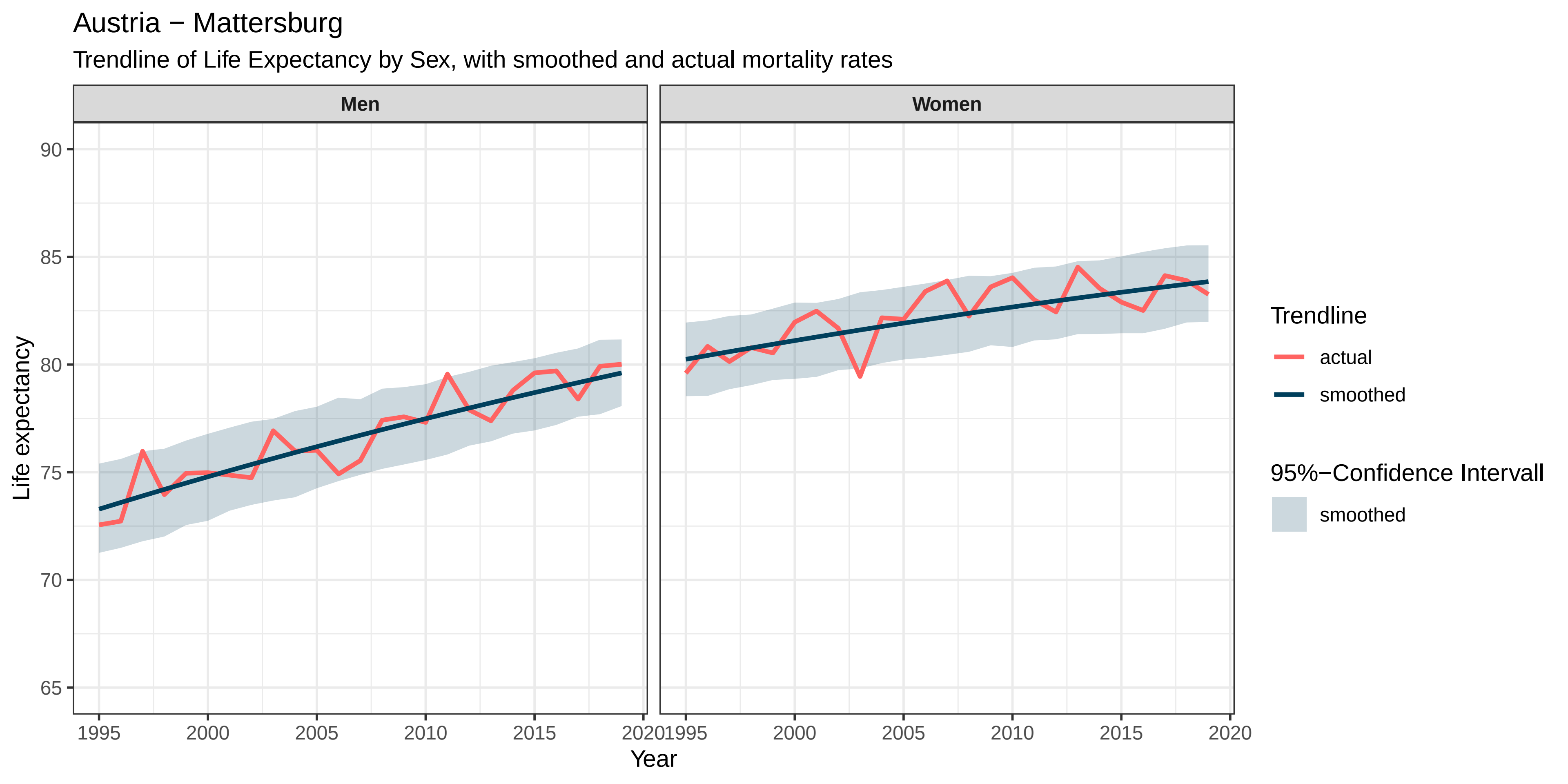

Austria − Mattersburg
Trendline of Life Expectancy by Sex, with smoothed and actual mortality rates
Men
Women
Life expectancy
Year
90
85
80
75
70
65
1995
2000
2005
2010
2015
2020
Trendline
actual
smoothed
95%−Confidence Intervall
smoothed

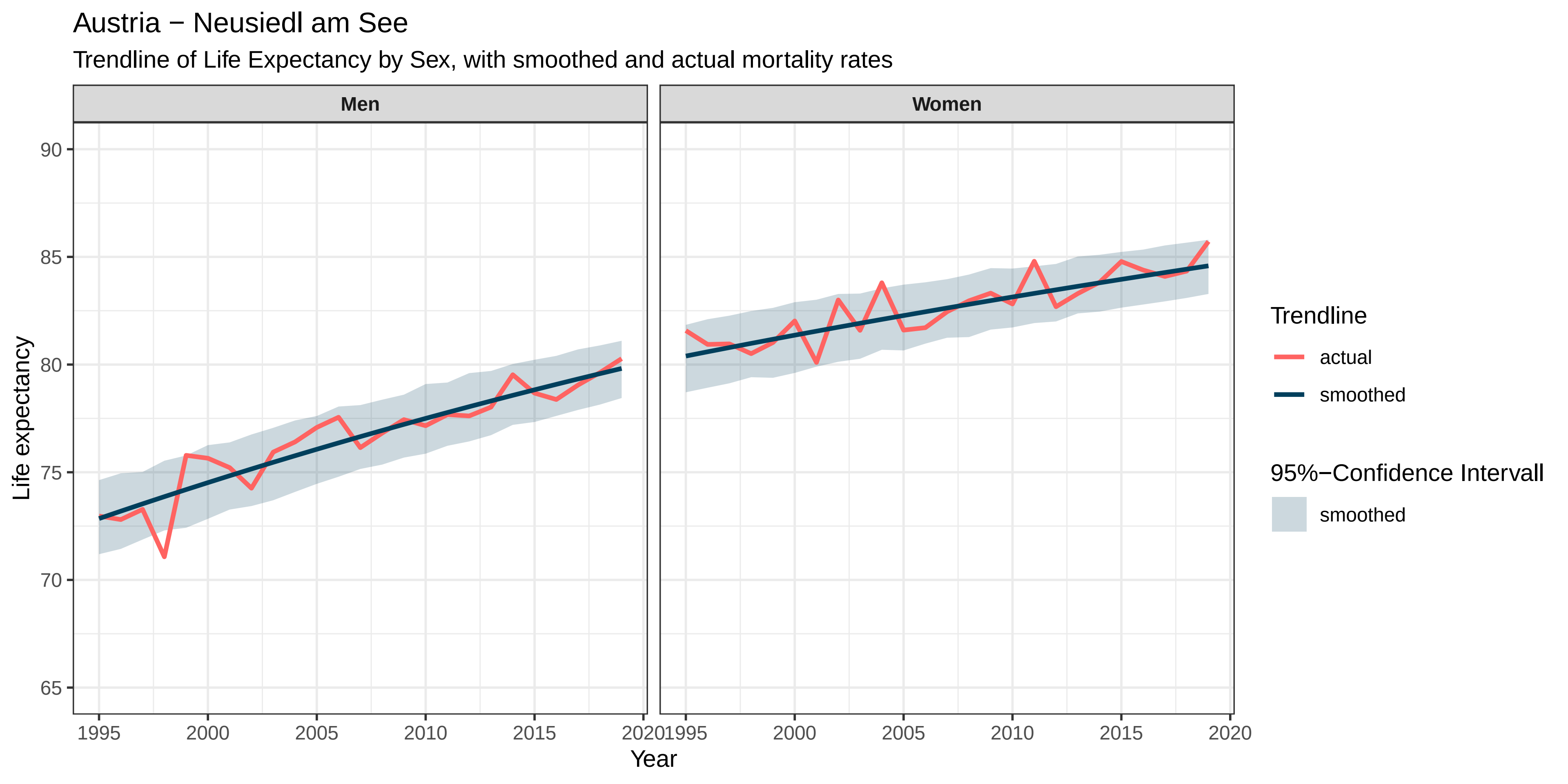
Austria − Neusiedl am See
Trendline of Life Expectancy by Sex, with smoothed and actual mortality rates
Men
Women
Life expectancy
90
85
80
75
70
65
1995
2000
2005
2010
2015
2020
Year
Trendline
actual
smoothed
95%−Confidence Intervall
smoothed

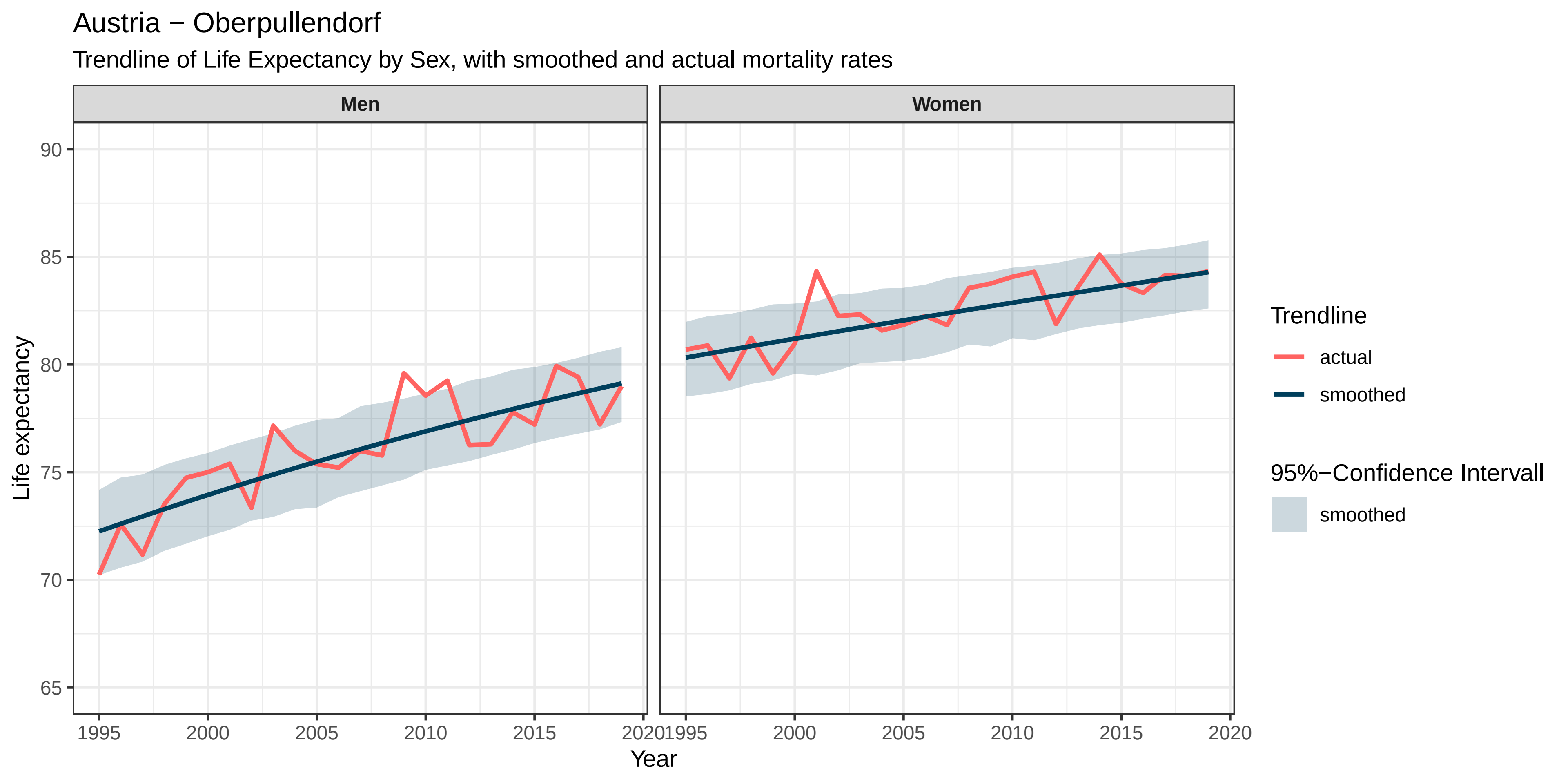
Austria − Oberpullendorf
Trendline of Life Expectancy by Sex, with smoothed and actual mortality rates
Men
Women
90
85
80
75
70
65
1995
2000
2005
2010
2015
2020
Year
Life expectancy
Trendline
actual
smoothed
95%−Confidence Intervall
smoothed

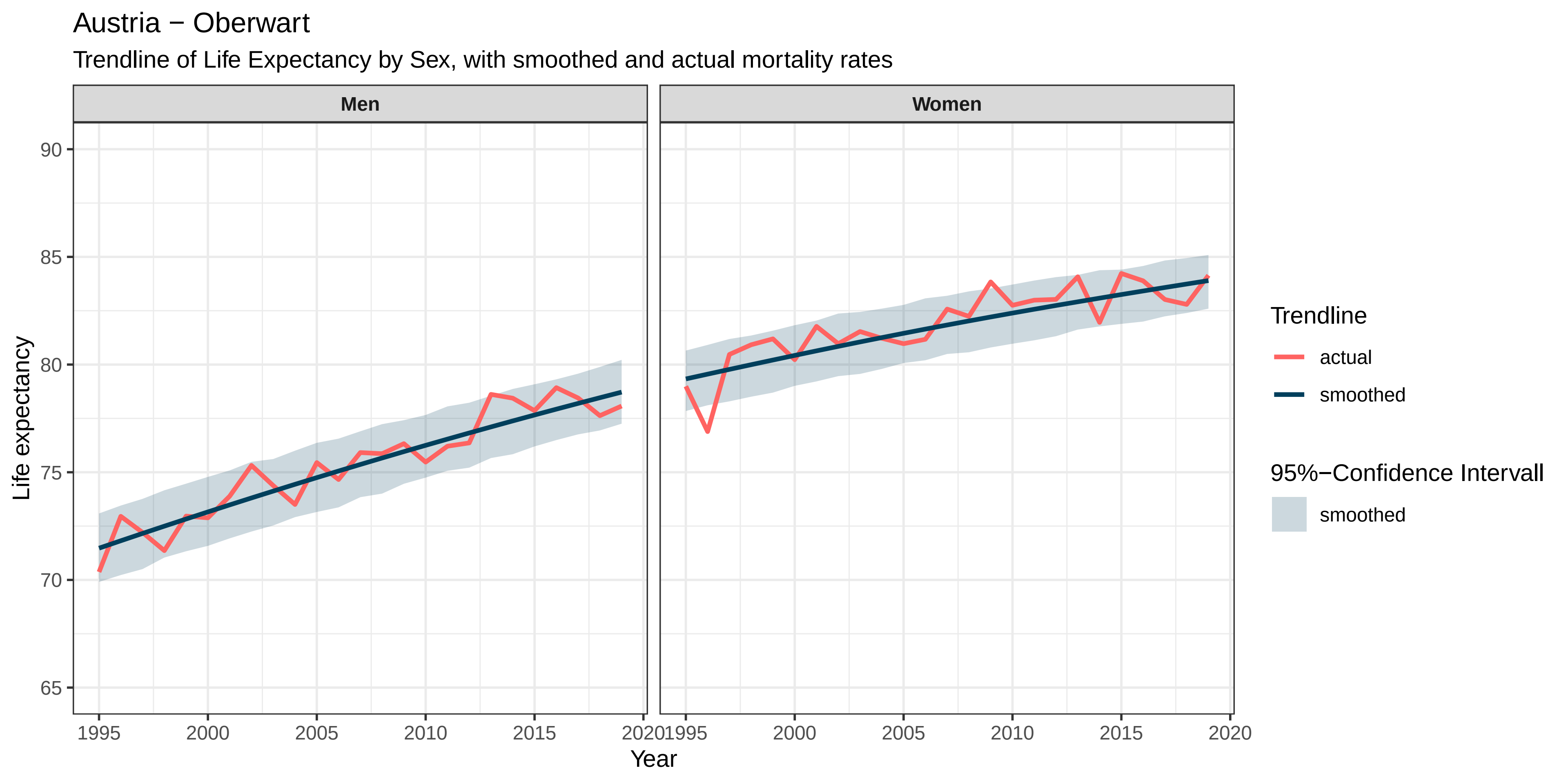

Austria − Oberwart
Trendline of Life Expectancy by Sex, with smoothed and actual mortality rates
Men
Women
Life expectancy
90
85
80
75
70
65
1995
2000
2005
2010
2015
2020
Year
Trendline
actual
smoothed
95%−Confidence Intervall
smoothed

Austria − Klagenfurt Stadt

Trendline of Life Expectancy by Sex, with smoothed and actual mortality rates

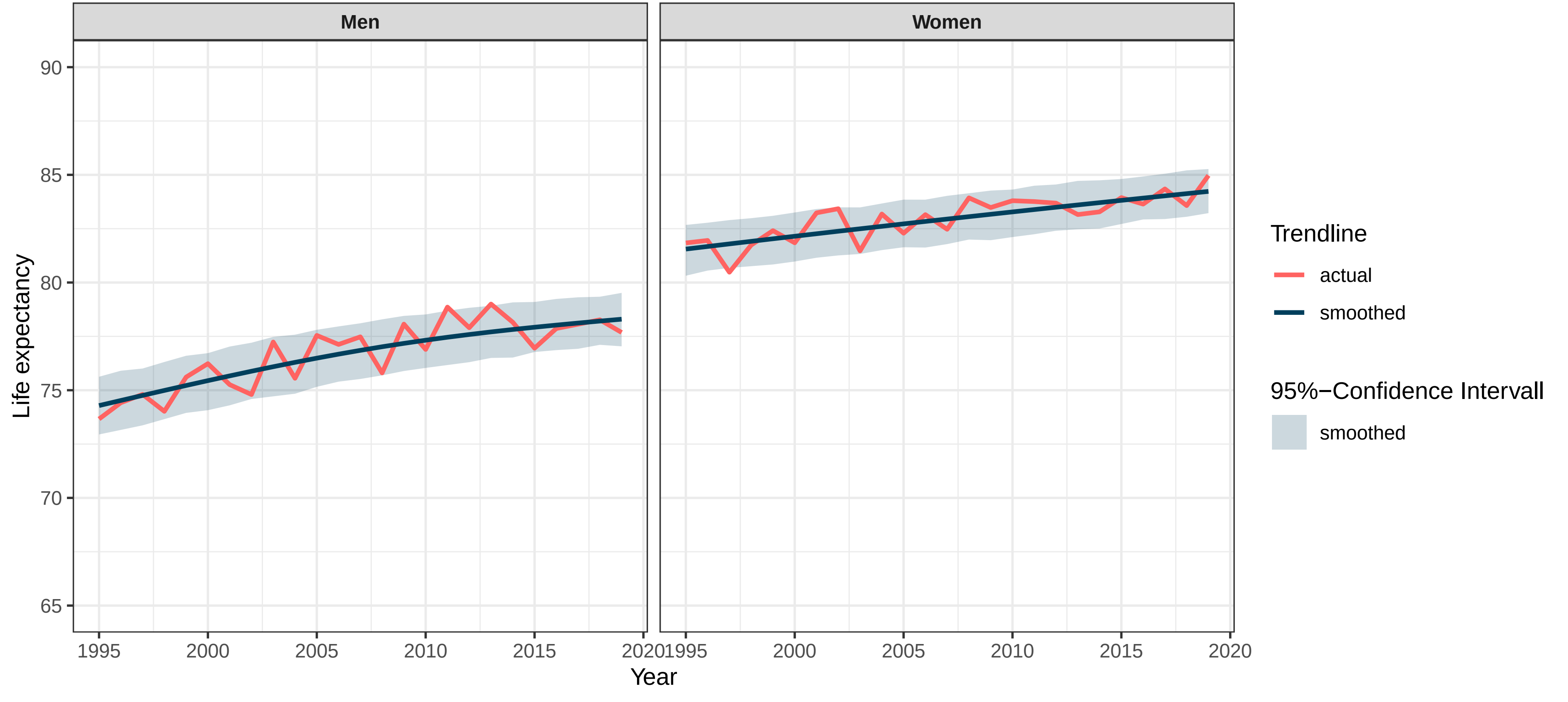

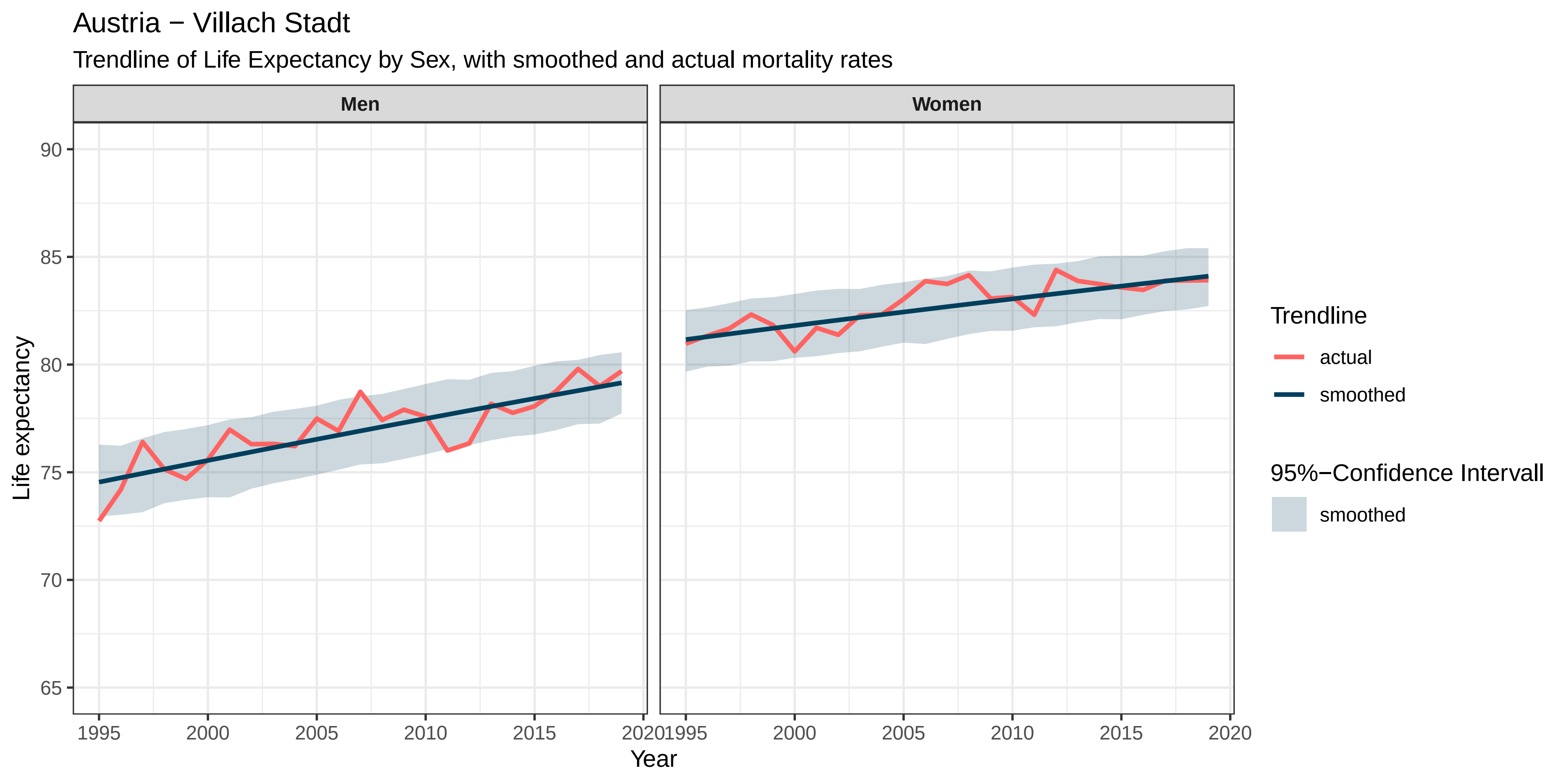

Austria − Villach Stadt
Trendline of Life Expectancy by Sex, with smoothed and actual mortality rates
Men
Women
Life expectancy
90
85
80
75
70
65
1995
2000
2005
2010
2015
2020
Year
Trendline
actual
smoothed
95%−Confidence Intervall
smoothed

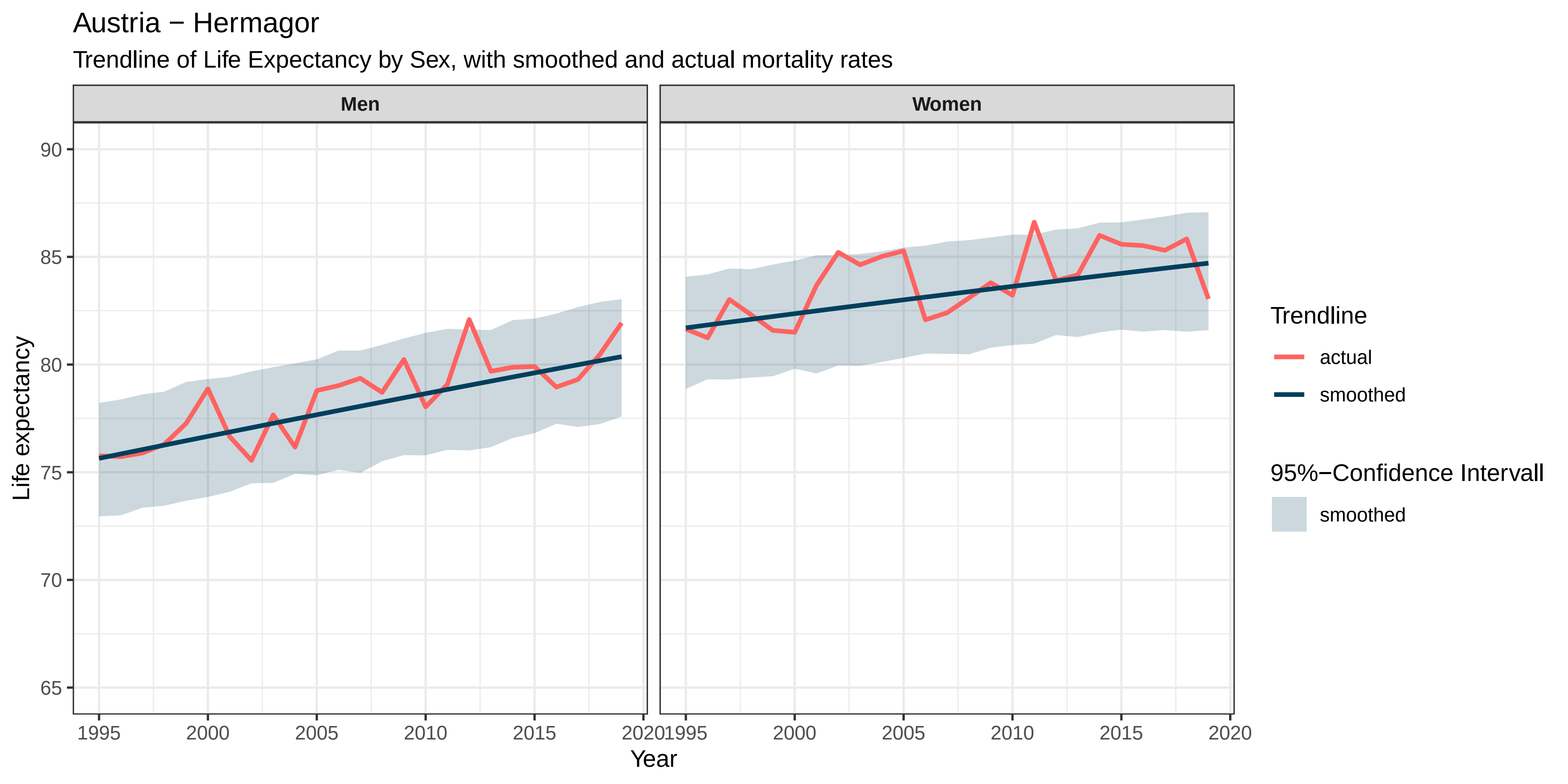
Austria − Hermagor
Trendline of Life Expectancy by Sex, with smoothed and actual mortality rates
Men
Women
Life expectancy
Year
90
85
80
75
70
65
1995
2000
2005
2010
2015
2020
Trendline
actual
smoothed
95%−Confidence Intervall
smoothed

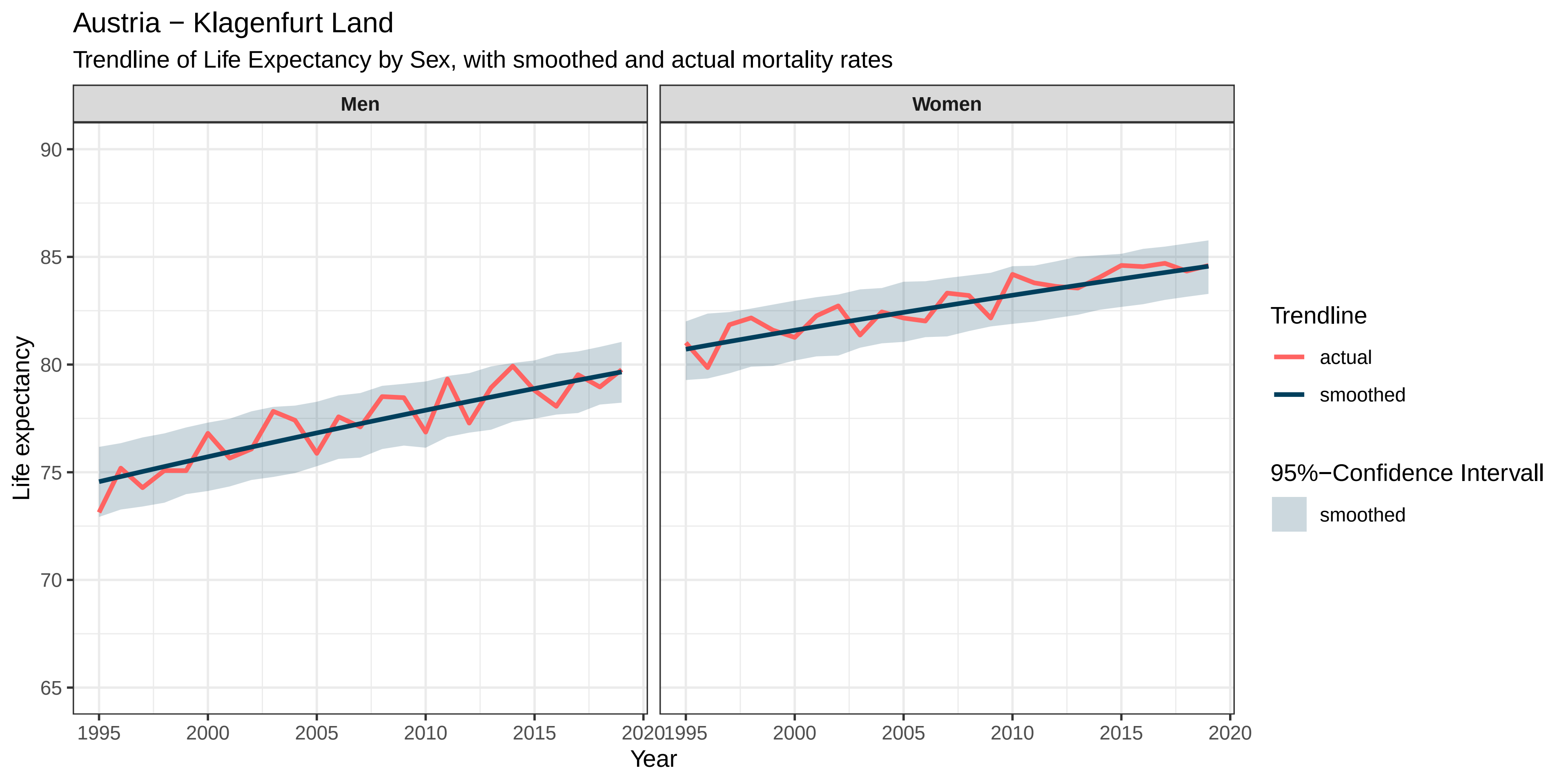

Austria − Klagenfurt Land
Trendline of Life Expectancy by Sex, with smoothed and actual mortality rates
Men
Women
Life expectancy
Year
90
85
80
75
70
65
1995
2000
2005
2010
2015
2020
Trendline
actual
smoothed
95%−Confidence Intervall
smoothed

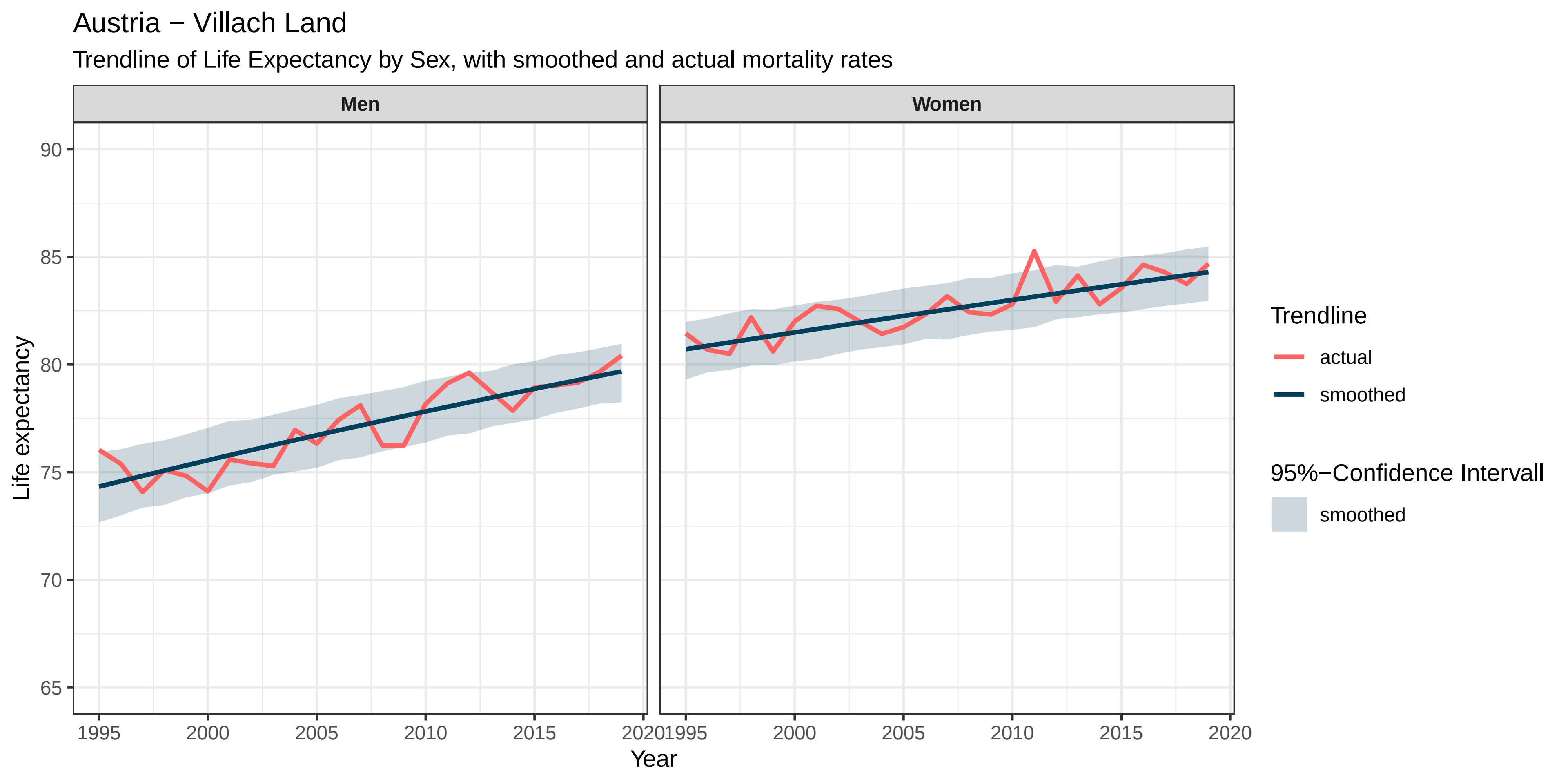
Austria − Villach Land
Trendline of Life Expectancy by Sex, with smoothed and actual mortality rates
Men
Women
Life expectancy
Year
Trendline
actual
smoothed
95%−Confidence Intervall
smoothed

Austria − Völkermarkt

Trendline of Life Expectancy by Sex, with smoothed and actual mortality rates

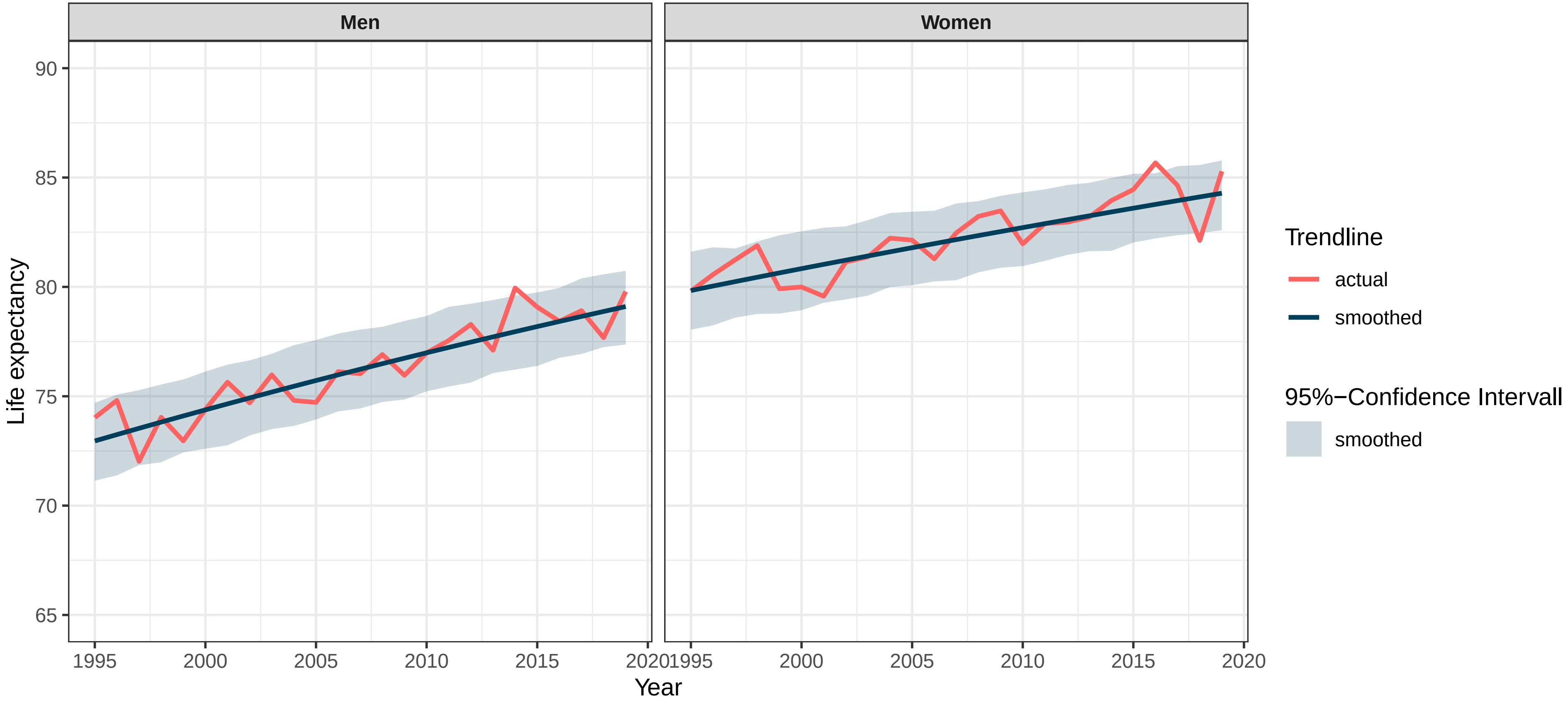

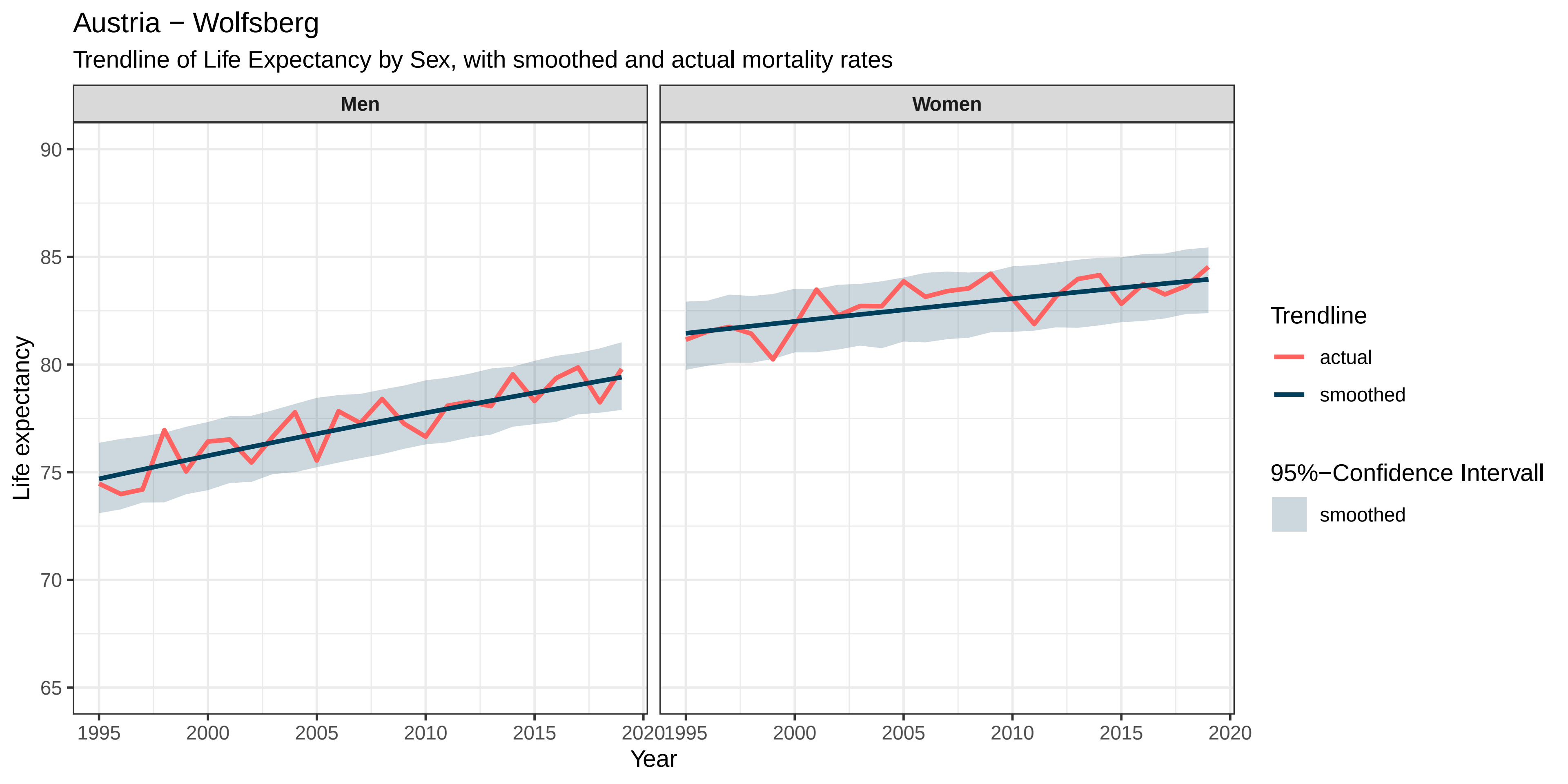

Austria − Wolfsberg
Trendline of Life Expectancy by Sex, with smoothed and actual mortality rates
Men
Women
90
85
80
75
70
65
1995
2000
2005
2010
2015
2020
Life expectancy
Year
Trendline
actual
smoothed
95%−Confidence Intervall
smoothed

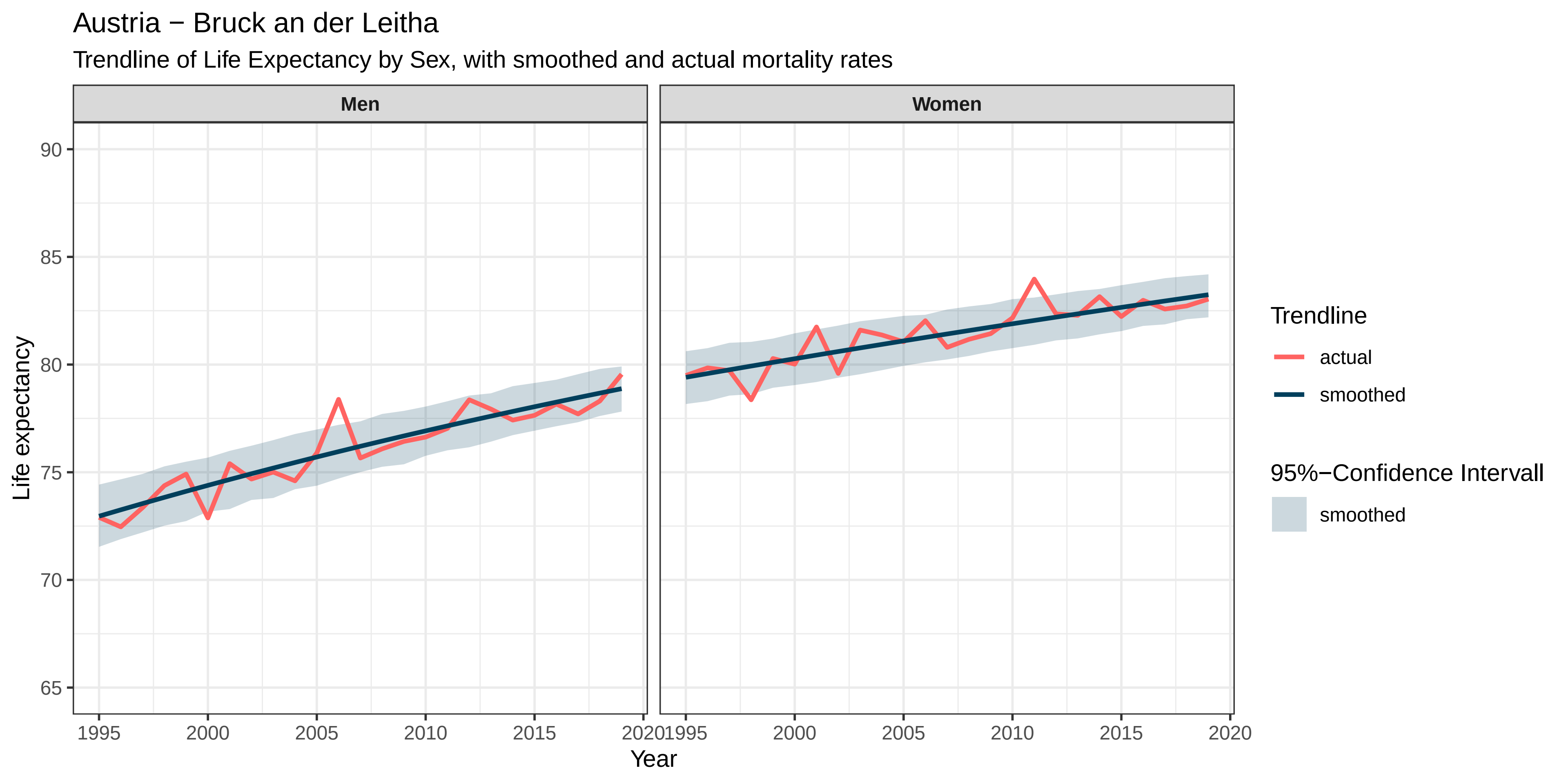
Austria − Bruck an der Leitha
Trendline of Life Expectancy by Sex, with smoothed and actual mortality rates
Men
Women
Life expectancy
Year
Trendline
actual
smoothed
95%−Confidence Intervall
smoothed

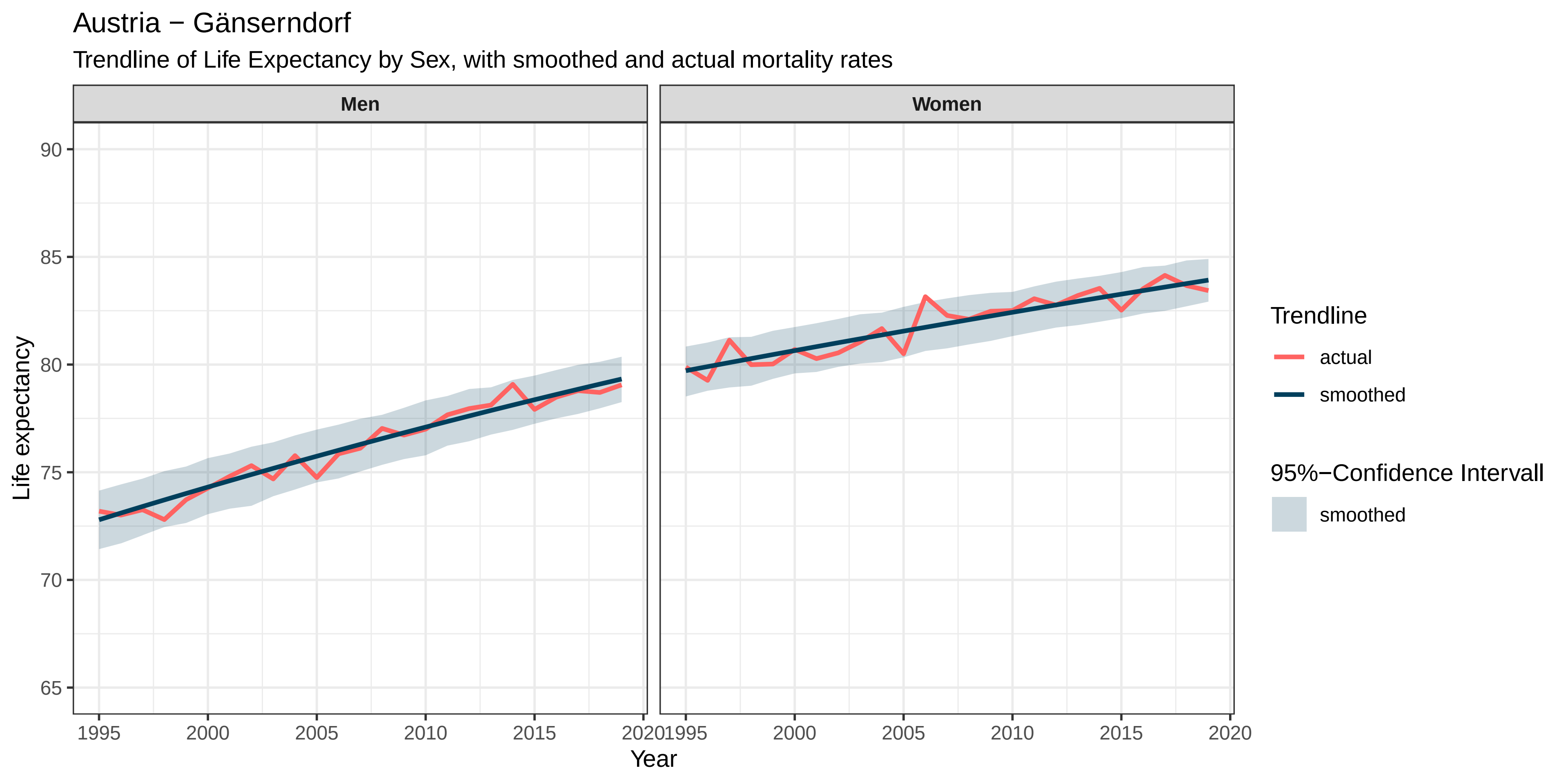
Austria − Gänserndorf
Trendline of Life Expectancy by Sex, with smoothed and actual mortality rates
Men
Women
90
85
80
75
70
65
Life expectancy
1995
2000
2005
2010
2015
2020
Year
Trendline
actual
smoothed
95%−Confidence Intervall
smoothed

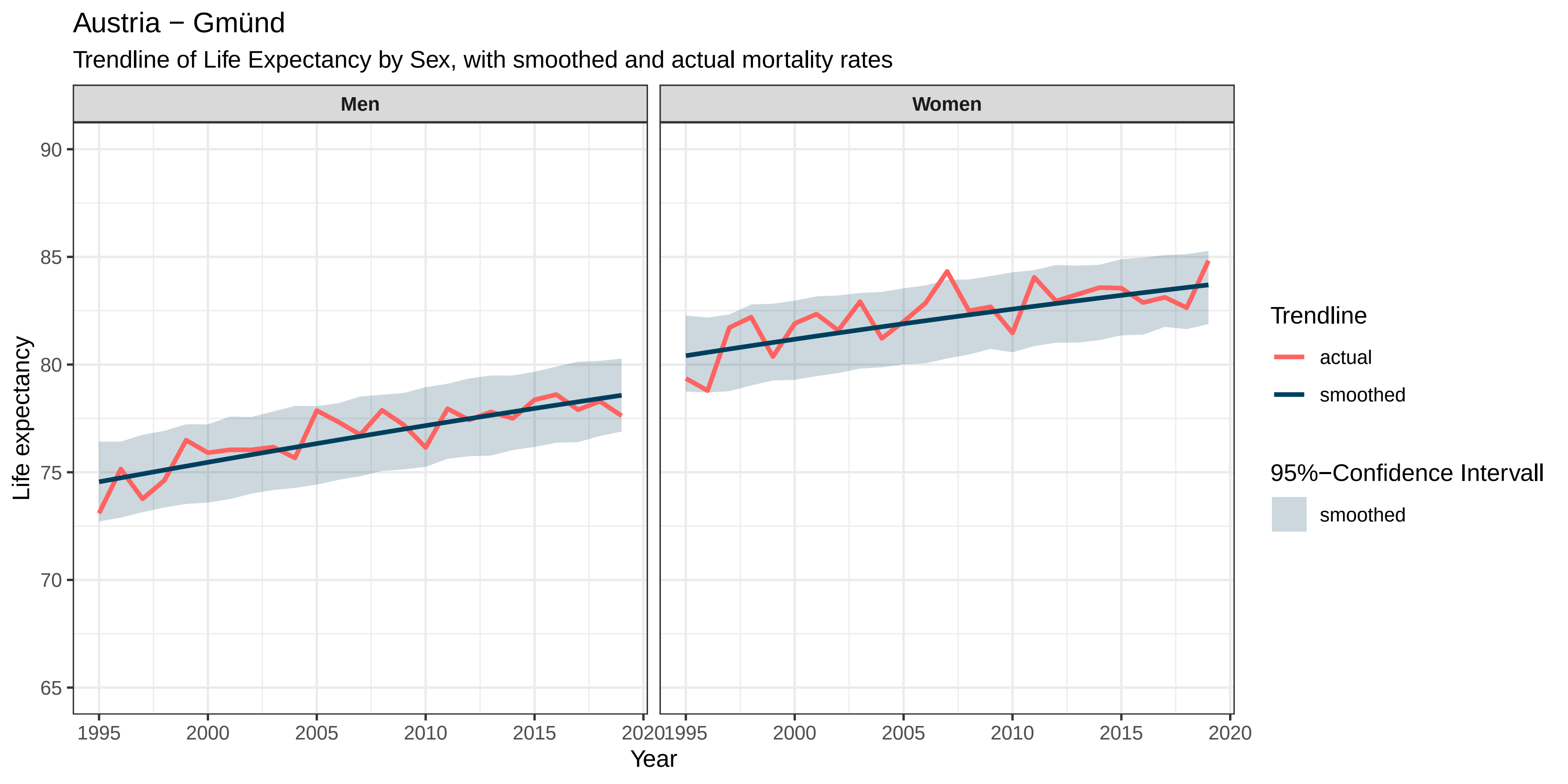

Austria − Gmünd
Trendline of Life Expectancy by Sex, with smoothed and actual mortality rates
Men
Women
90
85
80
75
70
65
Life expectancy
1995
2000
2005
2010
2015
2020
Year
Trendline
actual
smoothed
95%−Confidence Intervall
smoothed

Austria − Hollabrunn

Trendline of Life Expectancy by Sex, with smoothed and actual mortality rates

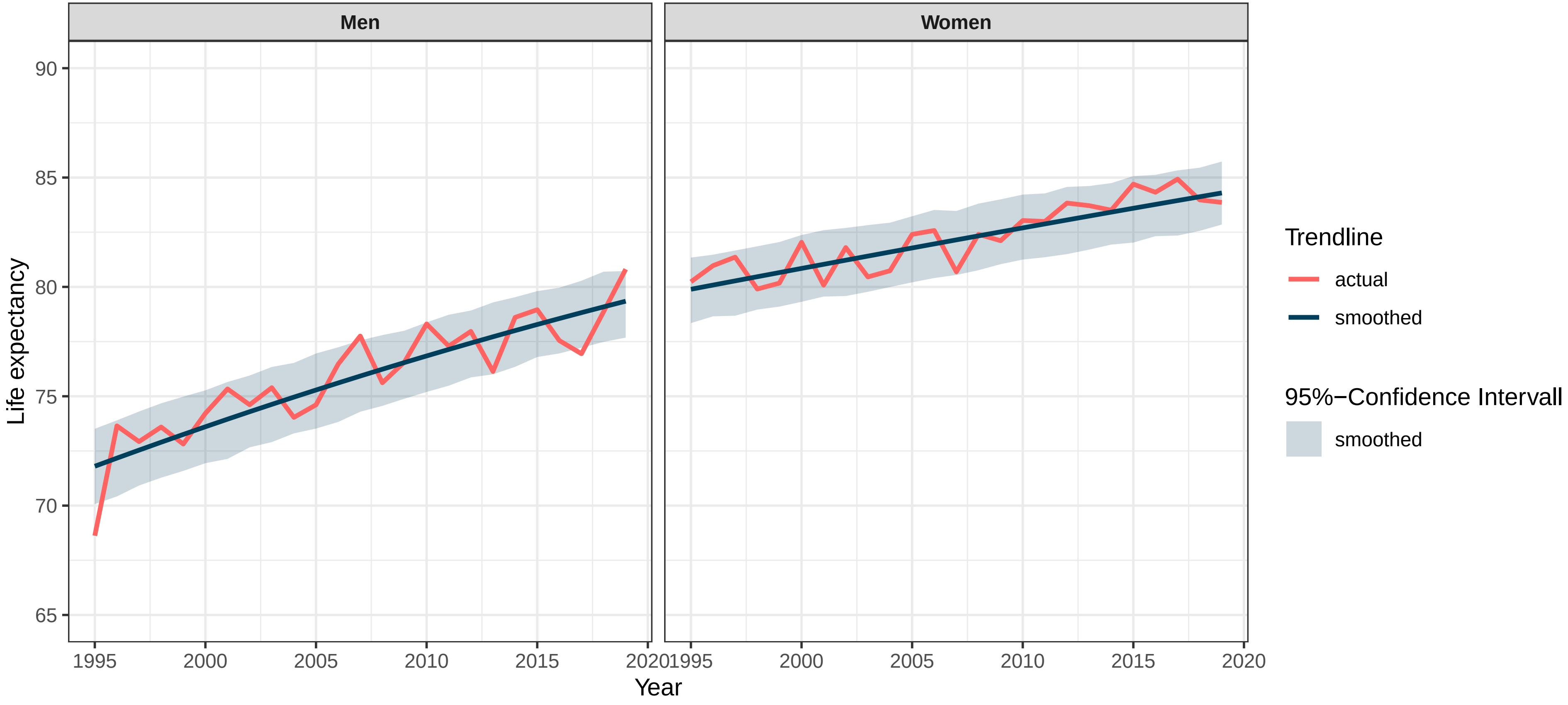

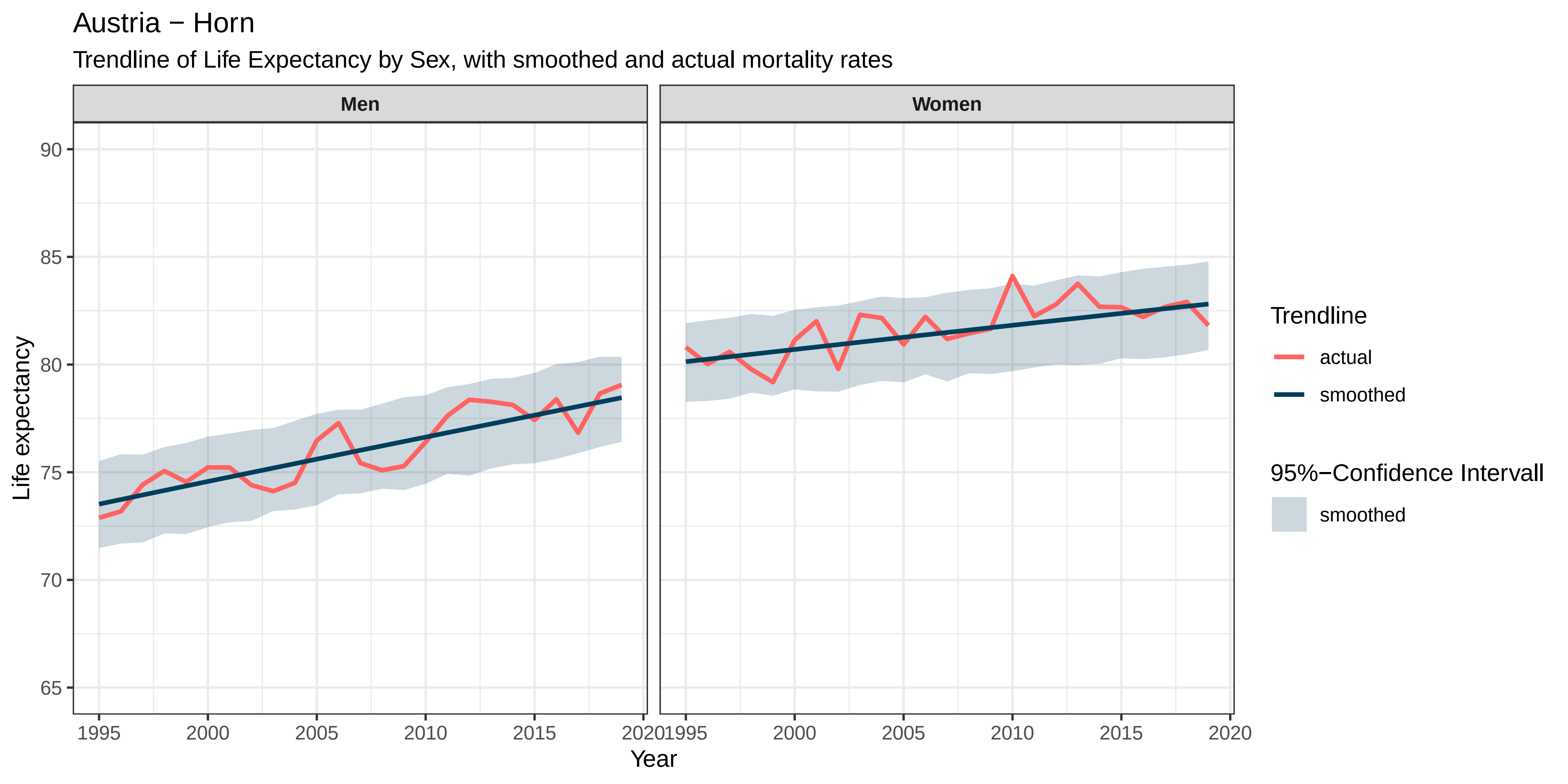

Austria − Horn
Trendline of Life Expectancy by Sex, with smoothed and actual mortality rates
Men
Women
Life expectancy
90
85
80
75
70
65
1995
2000
2005
2010
2015
2020
Year
Trendline
actual
smoothed
95%−Confidence Intervall
smoothed

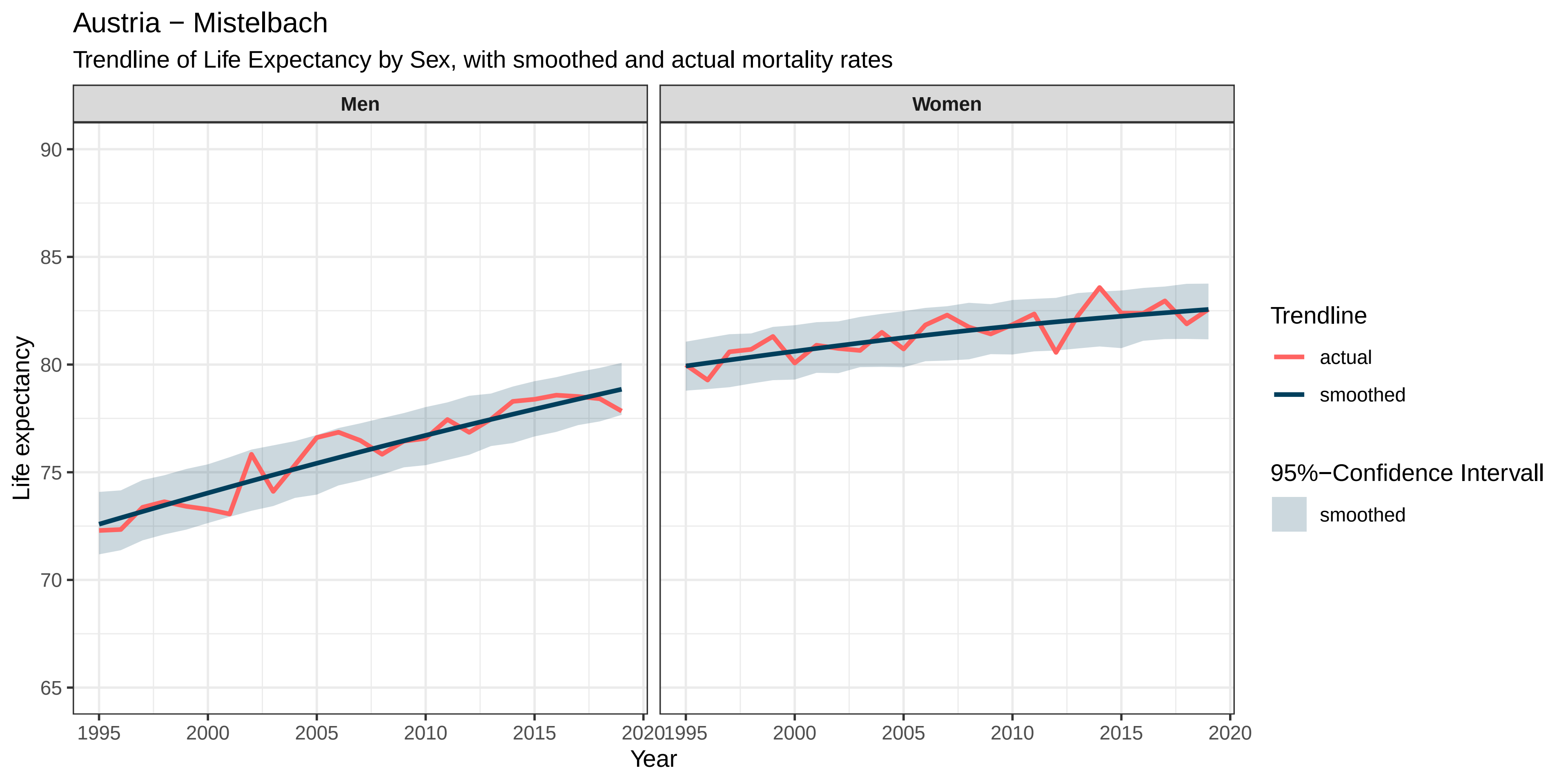
Austria − Mistelbach
Trendline of Life Expectancy by Sex, with smoothed and actual mortality rates
Men
Women
Life expectancy
90
85
80
75
70
65
1995
2000
2005
2010
2015
2020
Year
Trendline
actual
smoothed
95%−Confidence Intervall
smoothed

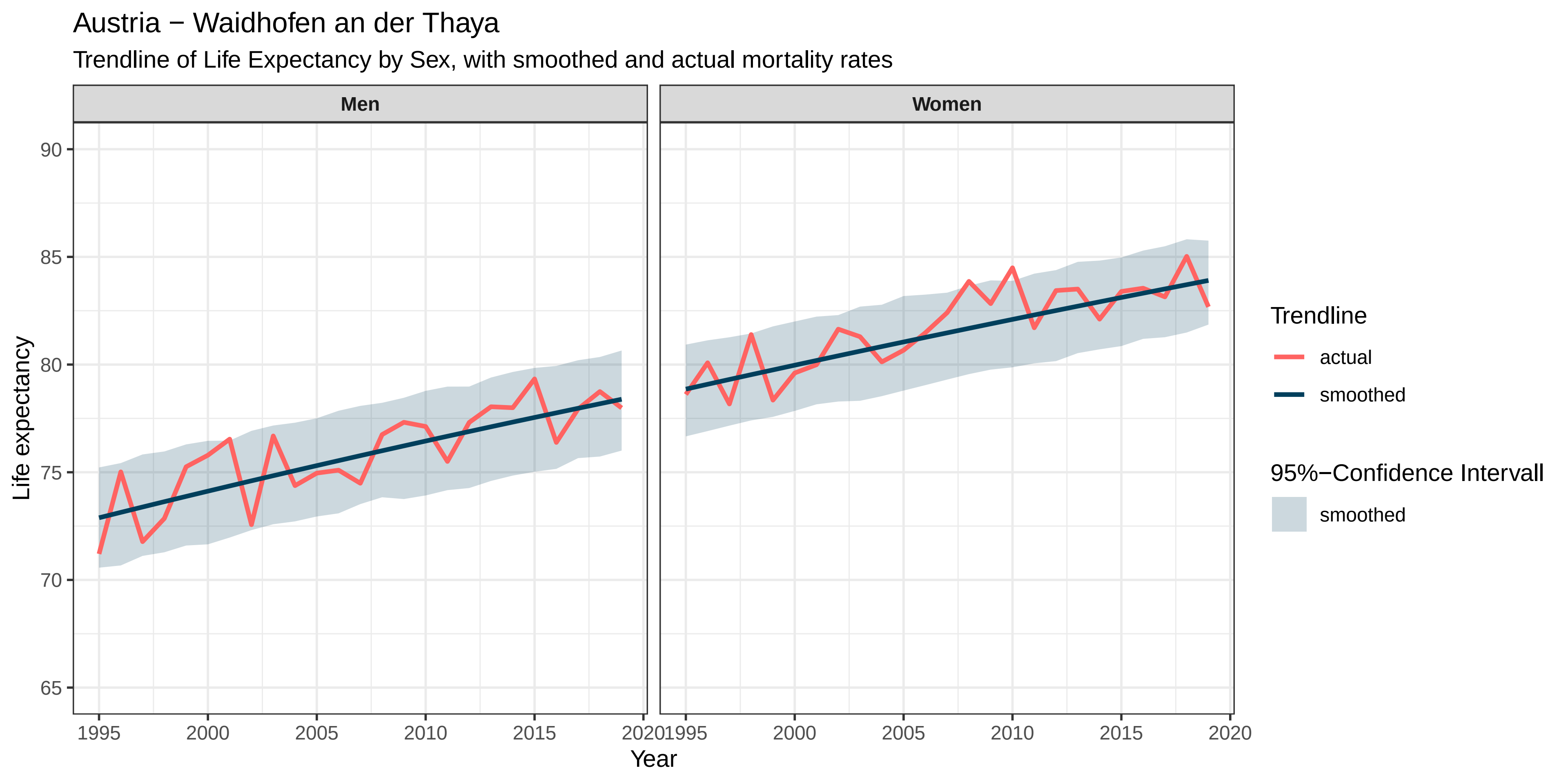

Austria − Waidhofen an der Thaya
Trendline of Life Expectancy by Sex, with smoothed and actual mortality rates
Men
Women
Life expectancy
90
85
80
75
70
65
1995
2000
2005
2010
2015
2020
Year
Trendline
actual
smoothed
95%−Confidence Intervall
smoothed

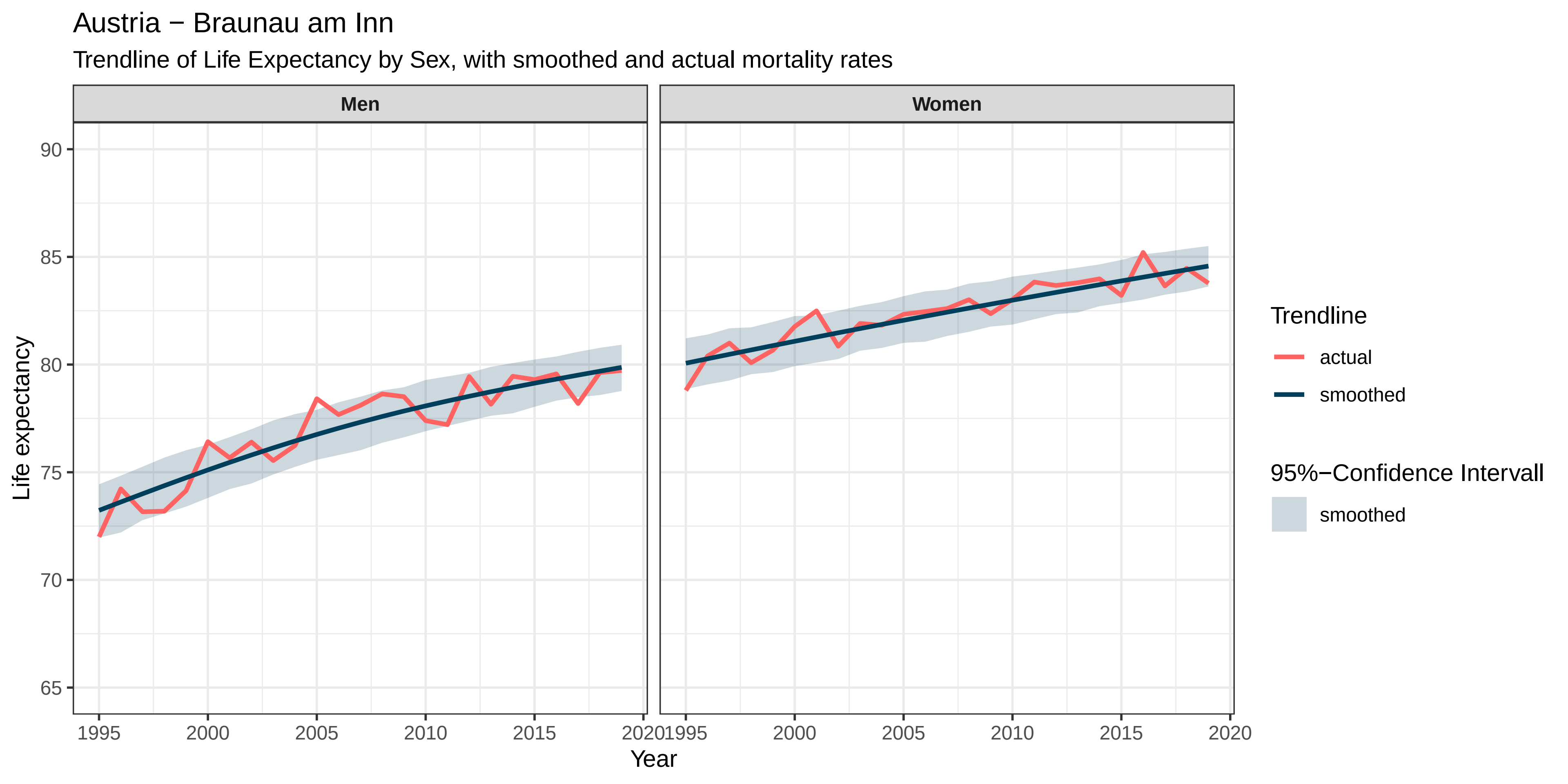

Austria − Braunau am Inn
Trendline of Life Expectancy by Sex, with smoothed and actual mortality rates
Men
Women
Life expectancy
90
85
80
75
70
65
1995
2000
2005
2010
2015
2020
Year
Trendline
actual
smoothed
95%−Confidence Intervall
smoothed

Austria − Freistadt

Trendline of Life Expectancy by Sex, with smoothed and actual mortality rates

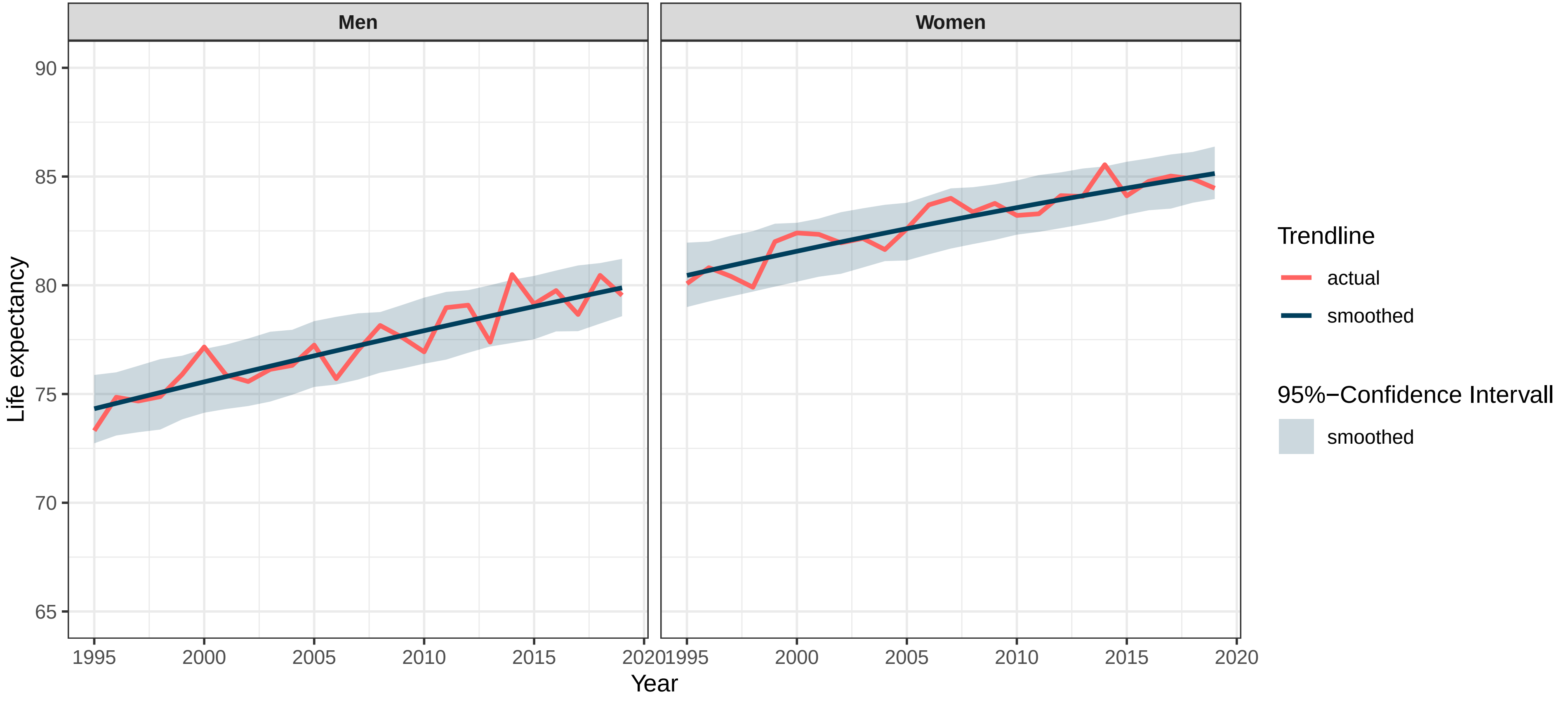

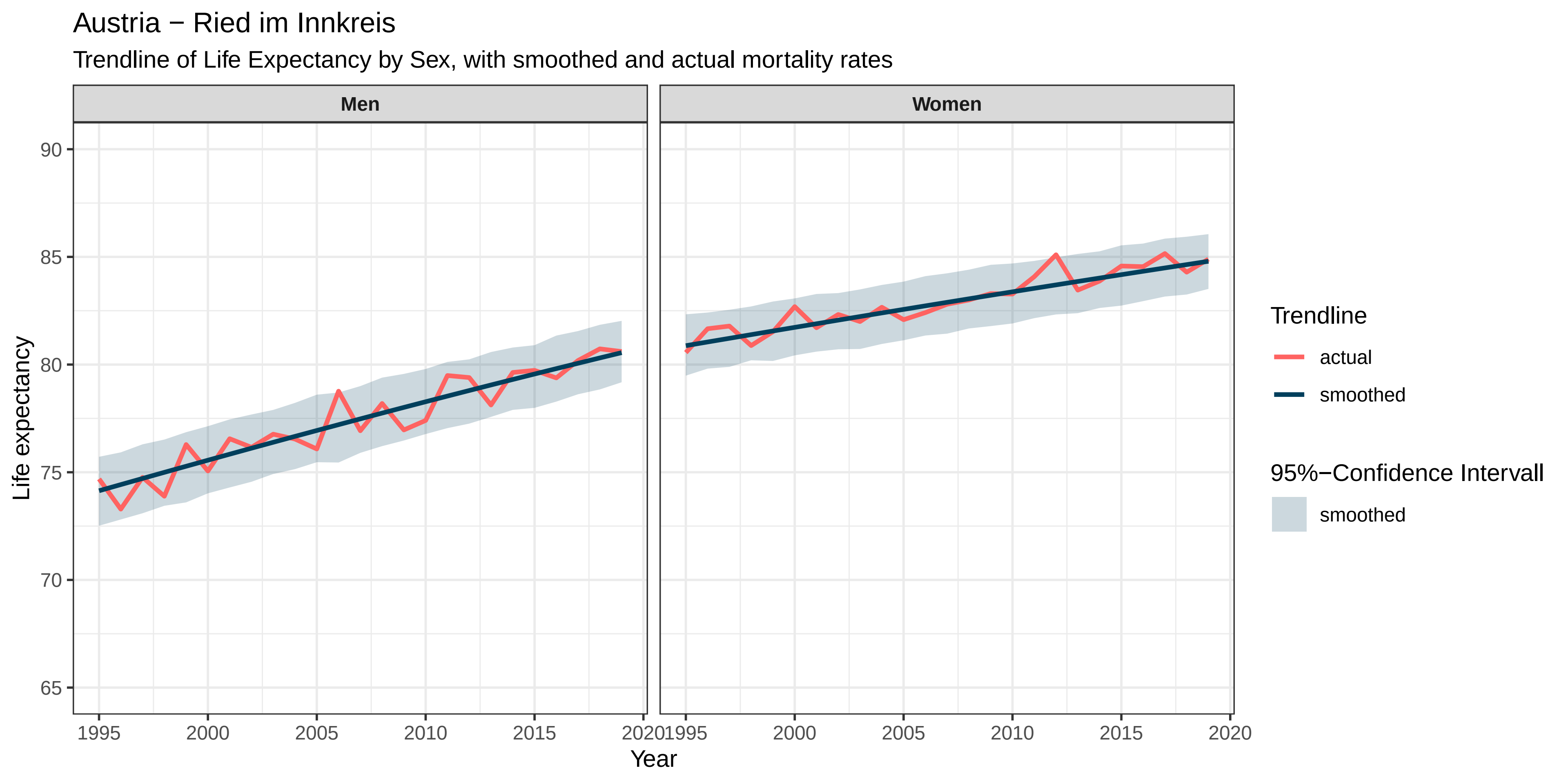
Austria − Ried im Innkreis
Trendline of Life Expectancy by Sex, with smoothed and actual mortality rates
Men
Women
Life expectancy
Year
90
85
80
75
70
65
1995
2000
2005
2010
2015
2020
Trendline
actual
smoothed
95%−Confidence Intervall
smoothed

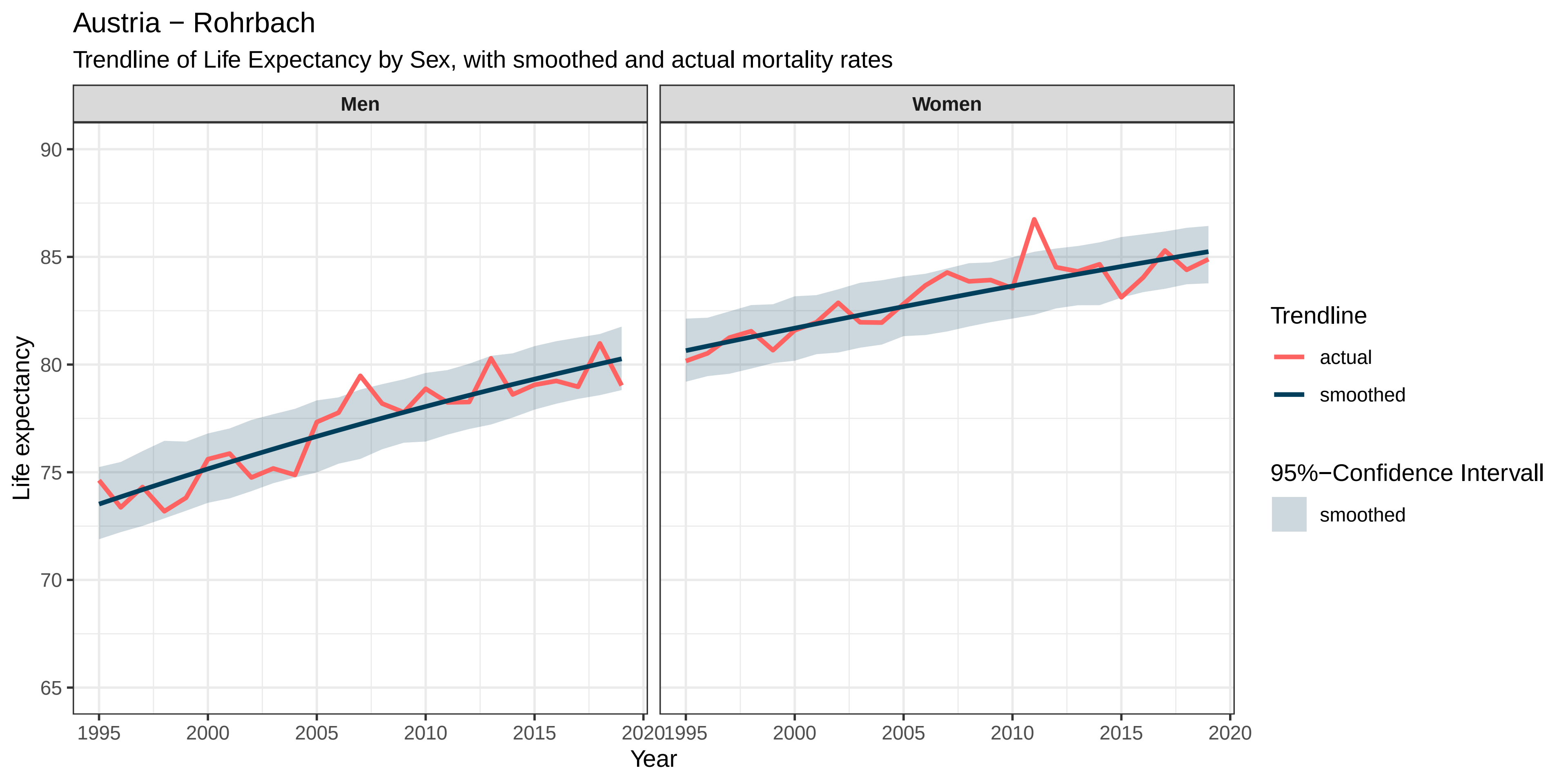

Austria − Rohrbach
Trendline of Life Expectancy by Sex, with smoothed and actual mortality rates
Men
Women
Life expectancy
90
85
80
75
70
65
1995
2000
2005
2010
2015
2020
Year
Trendline
actual
smoothed
95%−Confidence Intervall
smoothed

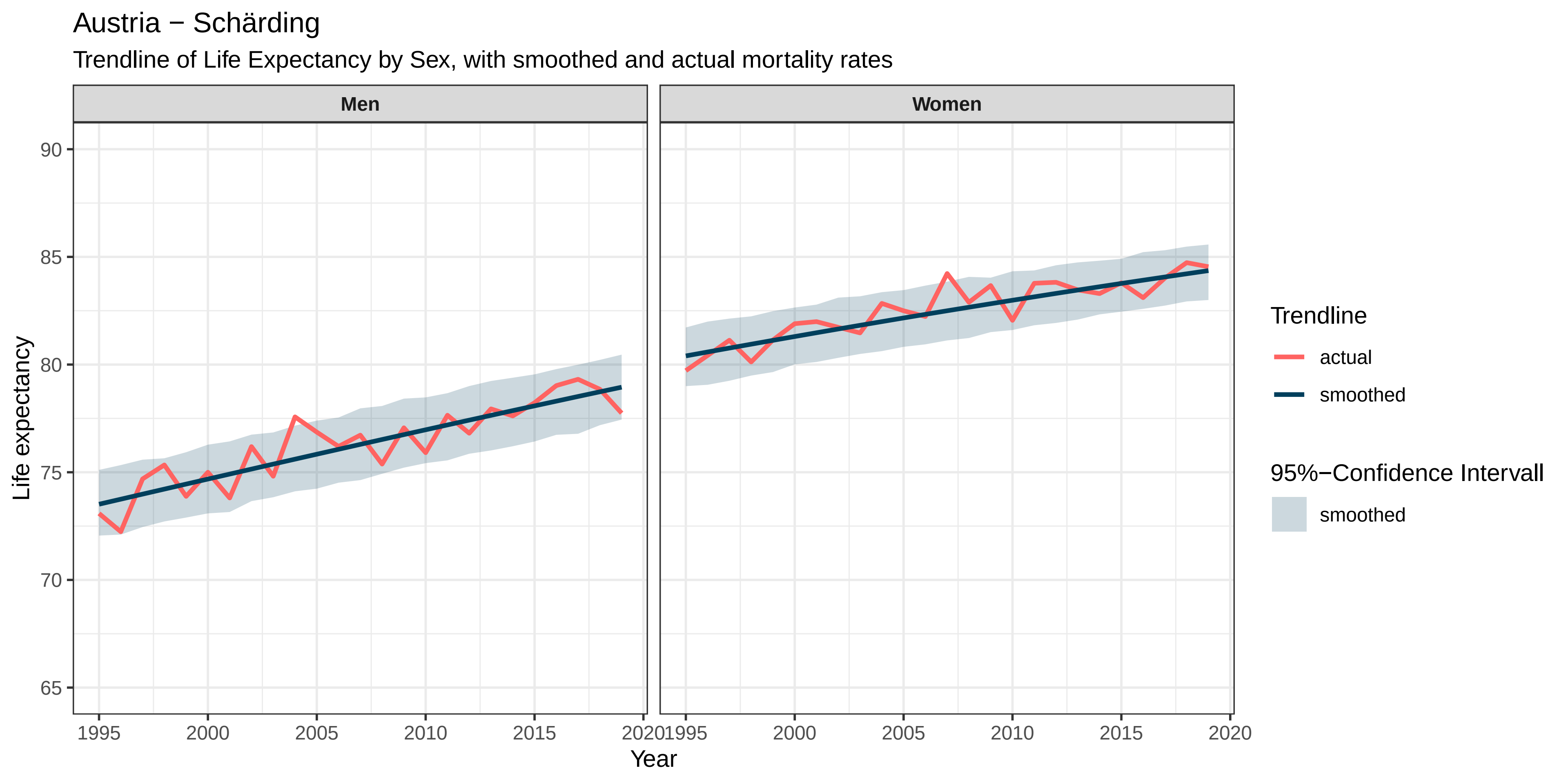

Austria − Schärding
Trendline of Life Expectancy by Sex, with smoothed and actual mortality rates
Men
Women
Life expectancy
90
85
80
75
70
65
1995
2000
2005
2010
2015
2020
Year
Trendline
actual
smoothed
95%−Confidence Intervall
smoothed

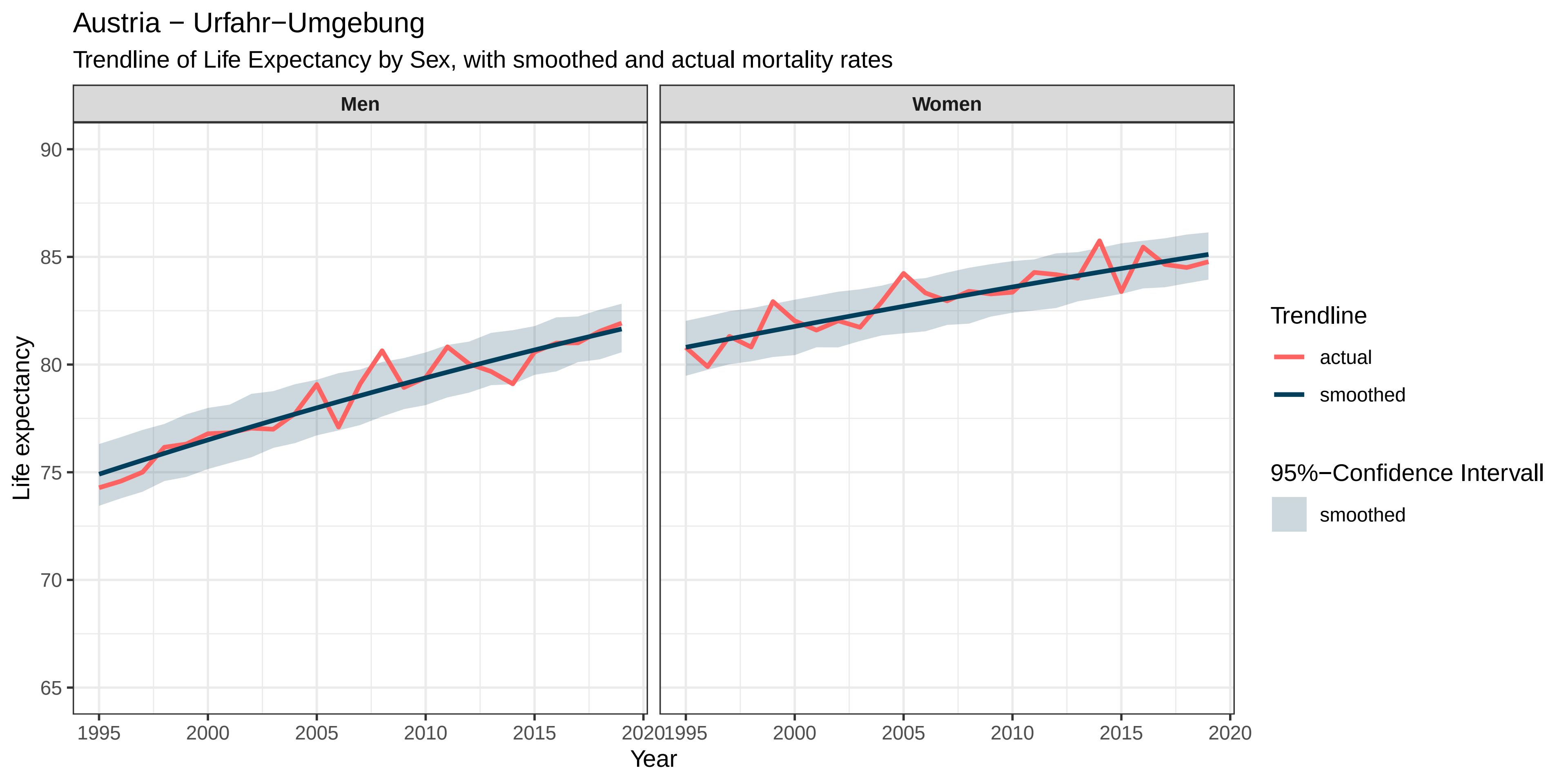
Austria − Urfahr−Umgebung
Trendline of Life Expectancy by Sex, with smoothed and actual mortality rates
Men
Women
Life expectancy
90
85
80
75
70
65
1995
2000
2005
2010
2015
2020
Year
Trendline
actual
smoothed
95%−Confidence Intervall
smoothed

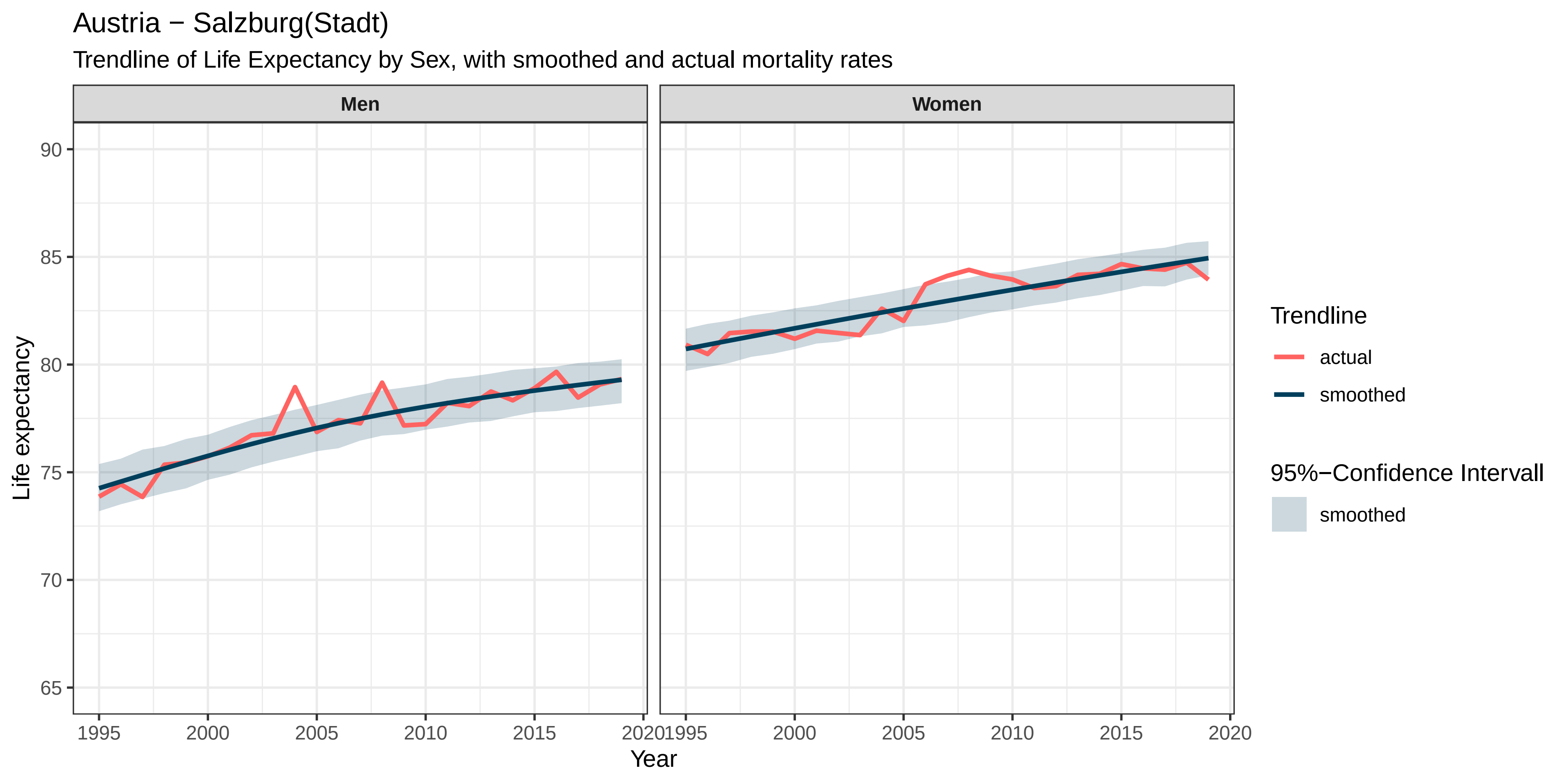
Austria − Salzburg(Stadt)
Trendline of Life Expectancy by Sex, with smoothed and actual mortality rates
Men
Women
Life expectancy
90
85
80
75
70
65
1995
2000
2005
2010
2015
2020
Year
Trendline
actual
smoothed
95%−Confidence Intervall
smoothed

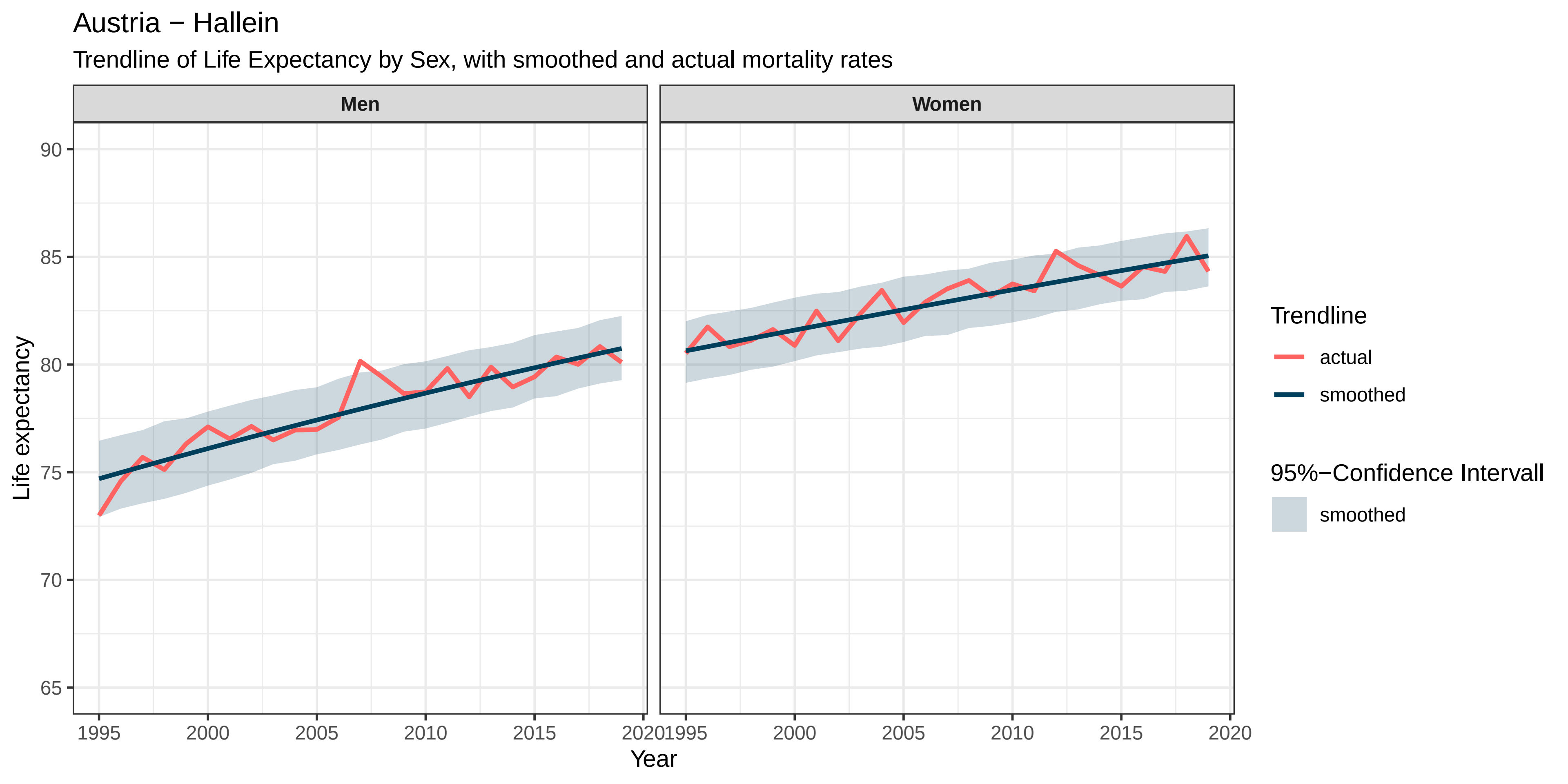

Austria − Hallein
Trendline of Life Expectancy by Sex, with smoothed and actual mortality rates
Men
Women
Life expectancy
65
70
75
80
85
90
1995
2000
2005
2010
2015
2020
Year
Trendline
actual
smoothed
95%−Confidence Intervall
smoothed

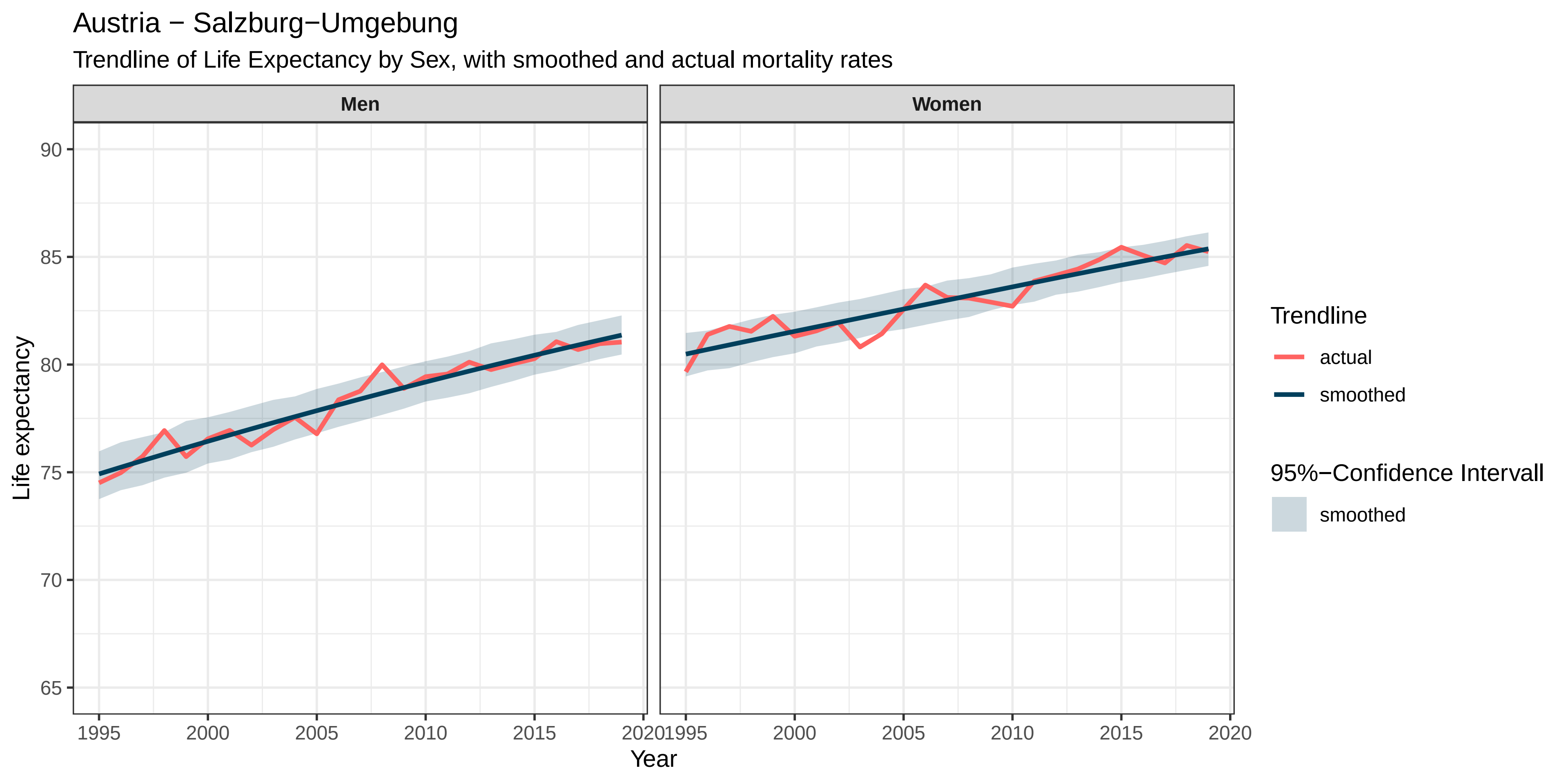

Austria − Salzburg−Umgebung
Trendline of Life Expectancy by Sex, with smoothed and actual mortality rates
Men
Women
Life expectancy
90
85
80
75
70
65
1995
2000
2005
2010
2015
2020
Year
Trendline
actual
smoothed
95%−Confidence Intervall
smoothed

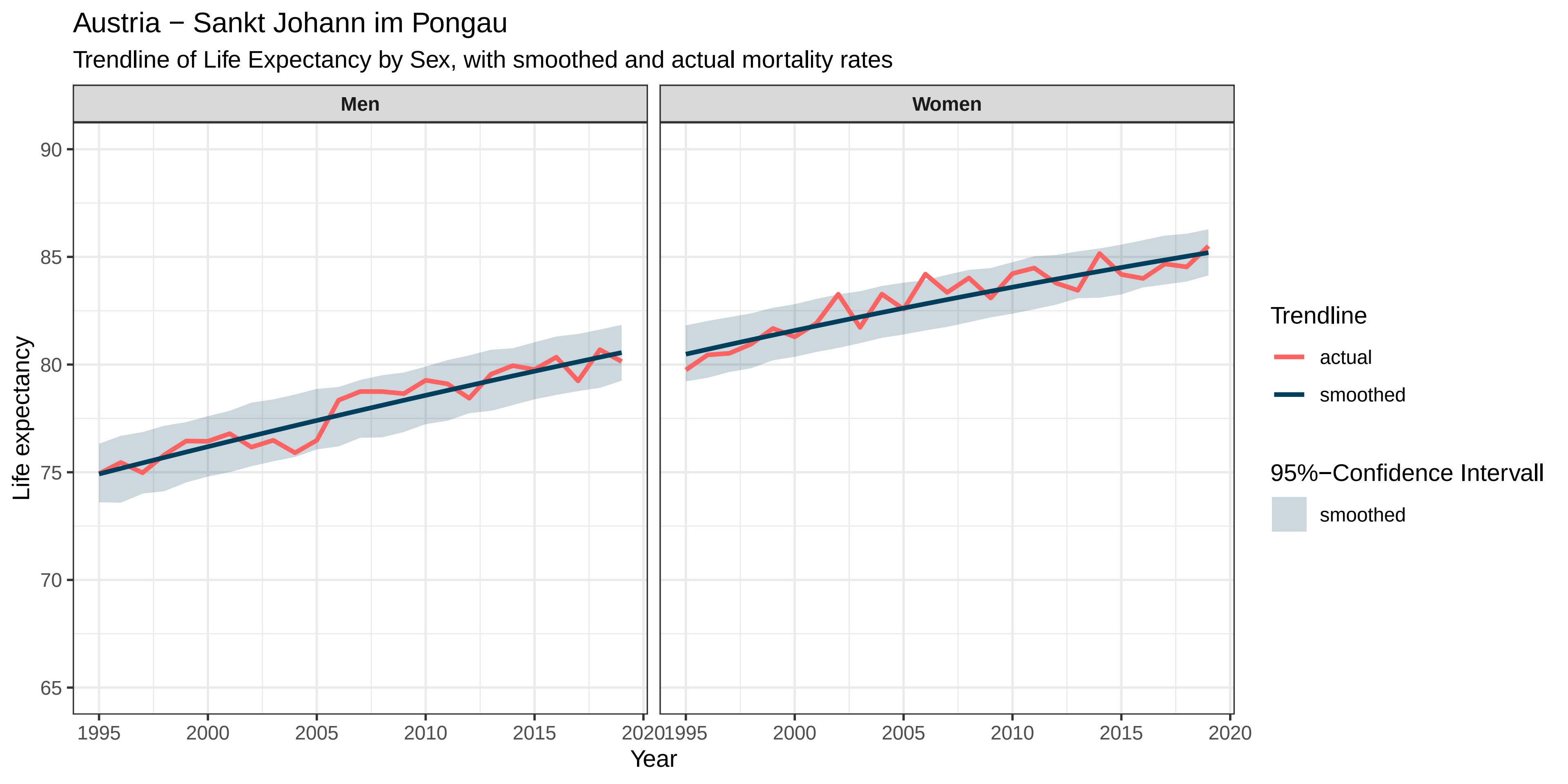

Austria − Sankt Johann im Pongau
Trendline of Life Expectancy by Sex, with smoothed and actual mortality rates
Men
Women
Life expectancy
90
85
80
75
70
65
1995
2000
2005
2010
2015
2020
Year
Trendline
actual
smoothed
95%−Confidence Intervall
smoothed

Austria − Zell am See

Trendline of Life Expectancy by Sex, with smoothed and actual mortality rates

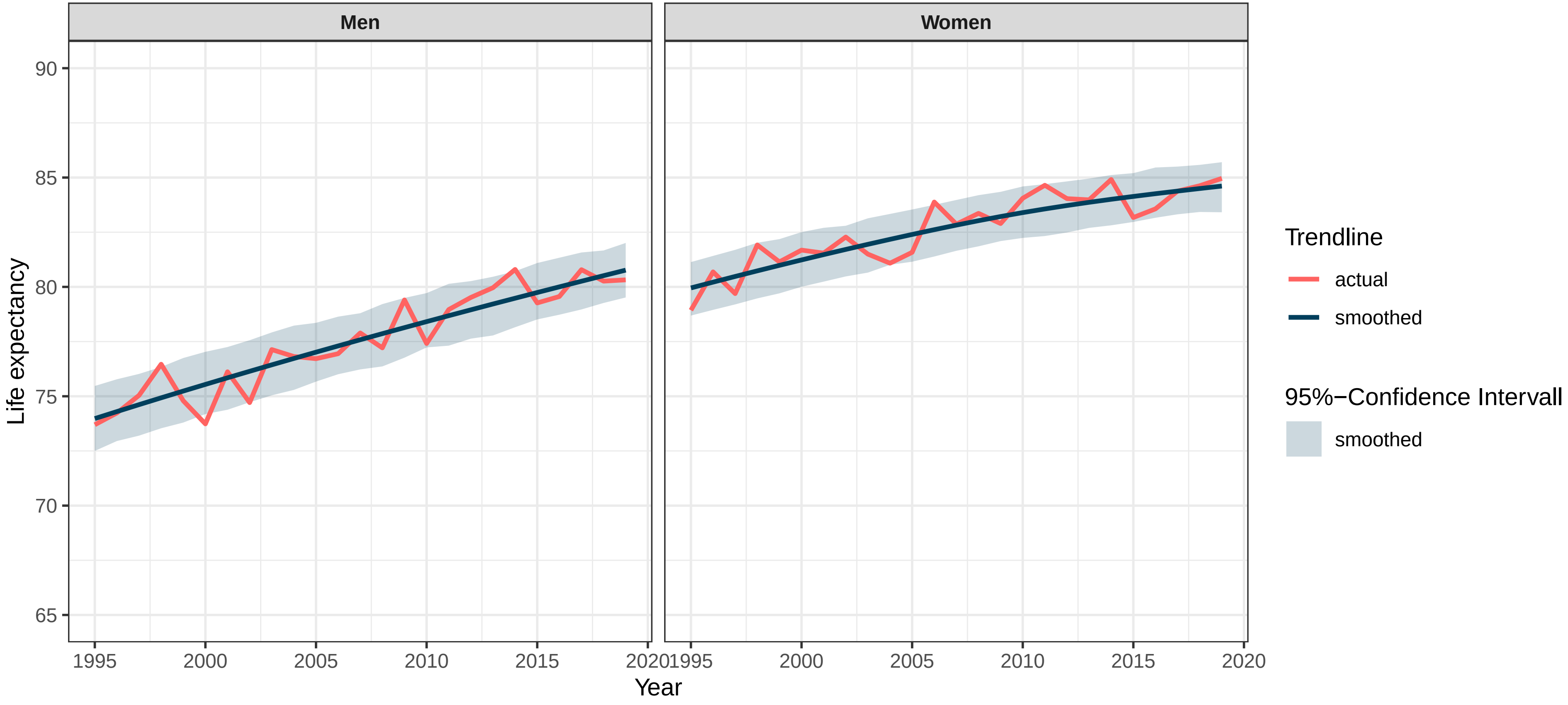

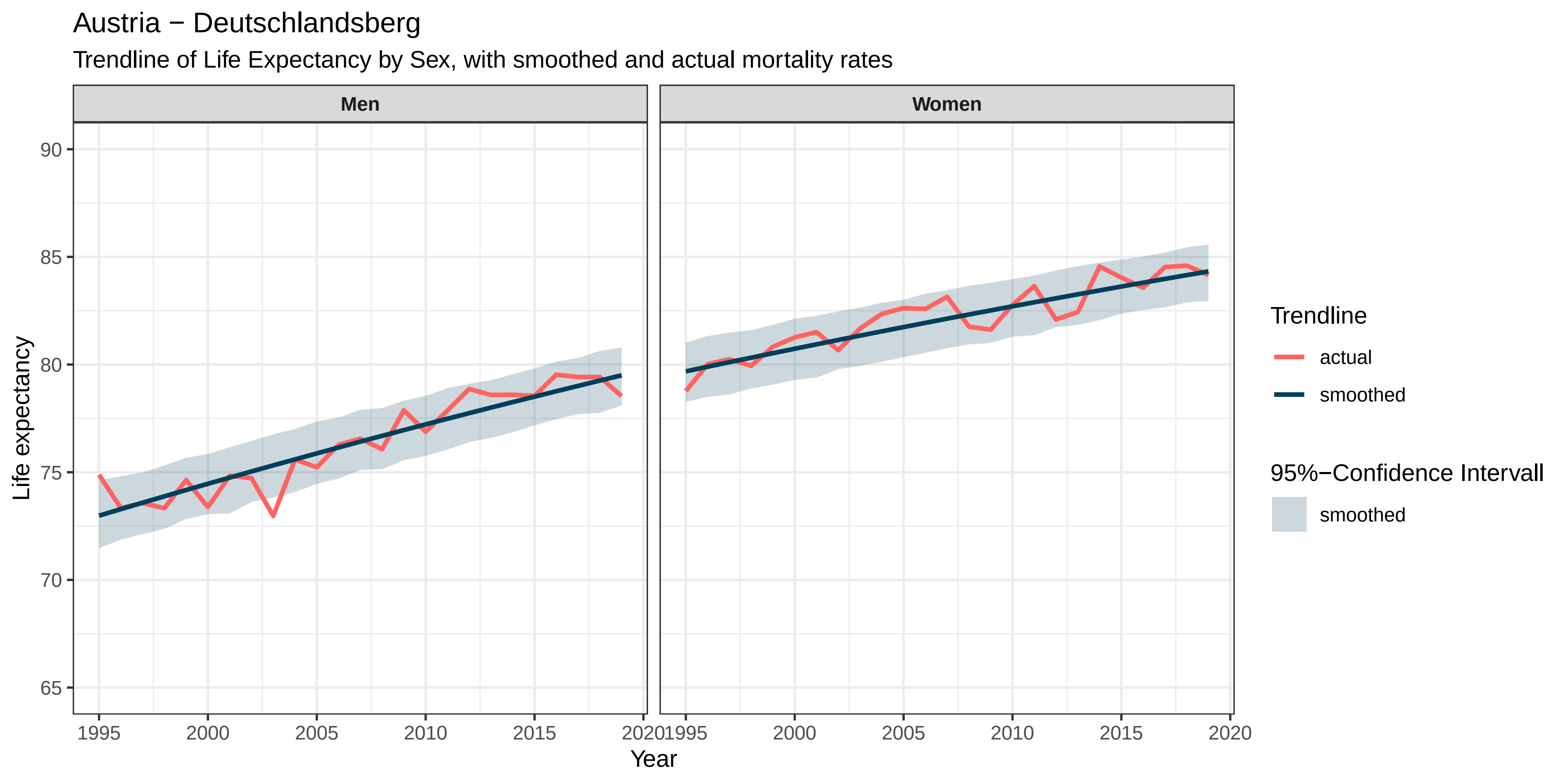

Austria − Deutschlandsberg
Trendline of Life Expectancy by Sex, with smoothed and actual mortality rates
Men
Women
Life expectancy
90
85
80
75
70
65
1995
2000
2005
2010
2015
2020
Year
Trendline
actual
smoothed
95%−Confidence Intervall
smoothed

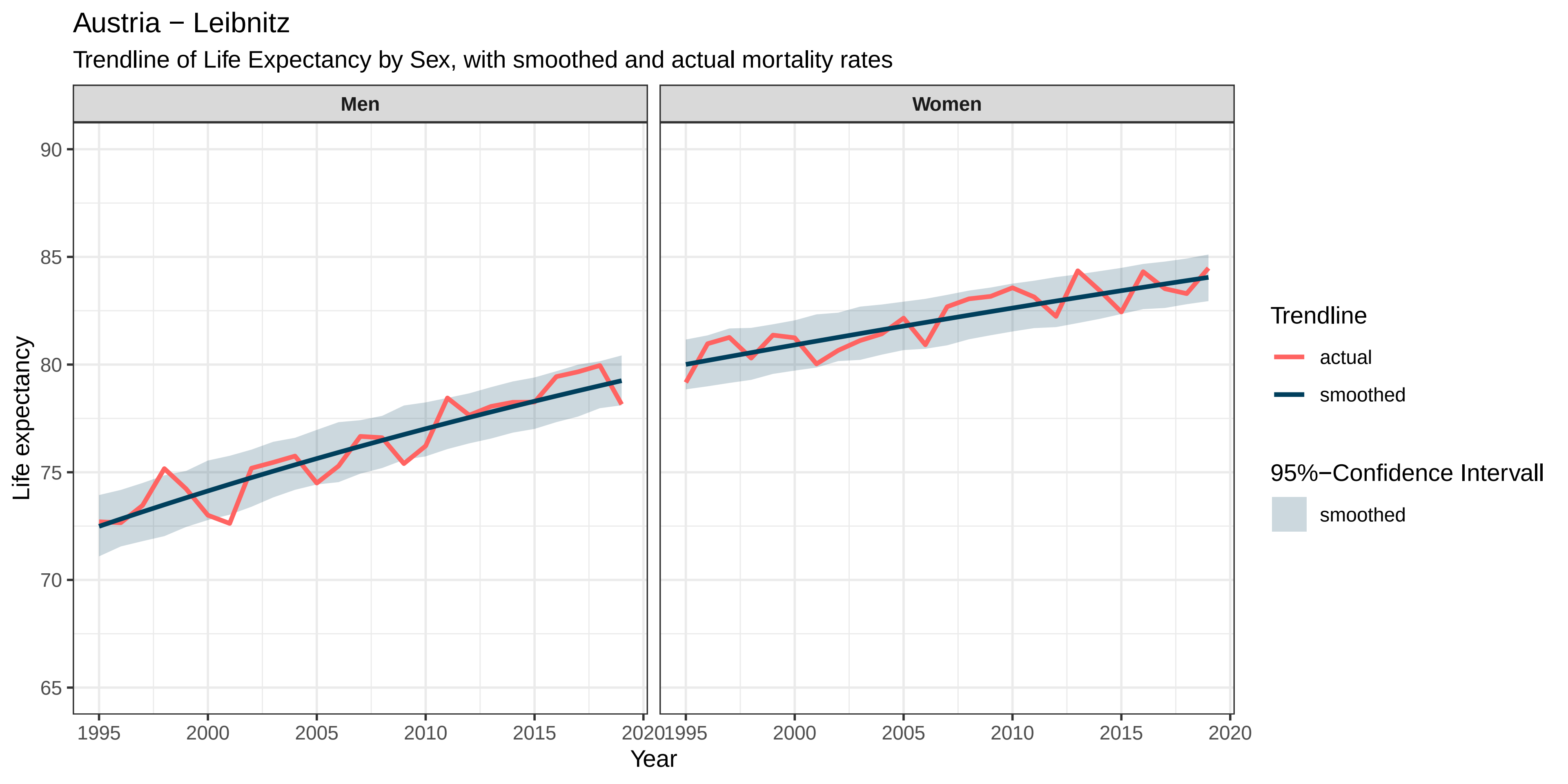
Austria − Leibnitz
Trendline of Life Expectancy by Sex, with smoothed and actual mortality rates
Men
Women
Life expectancy
90
85
80
75
70
65
1995
2000
2005
2010
2015
2020
Year
Trendline
actual
smoothed
95%−Confidence Intervall
smoothed

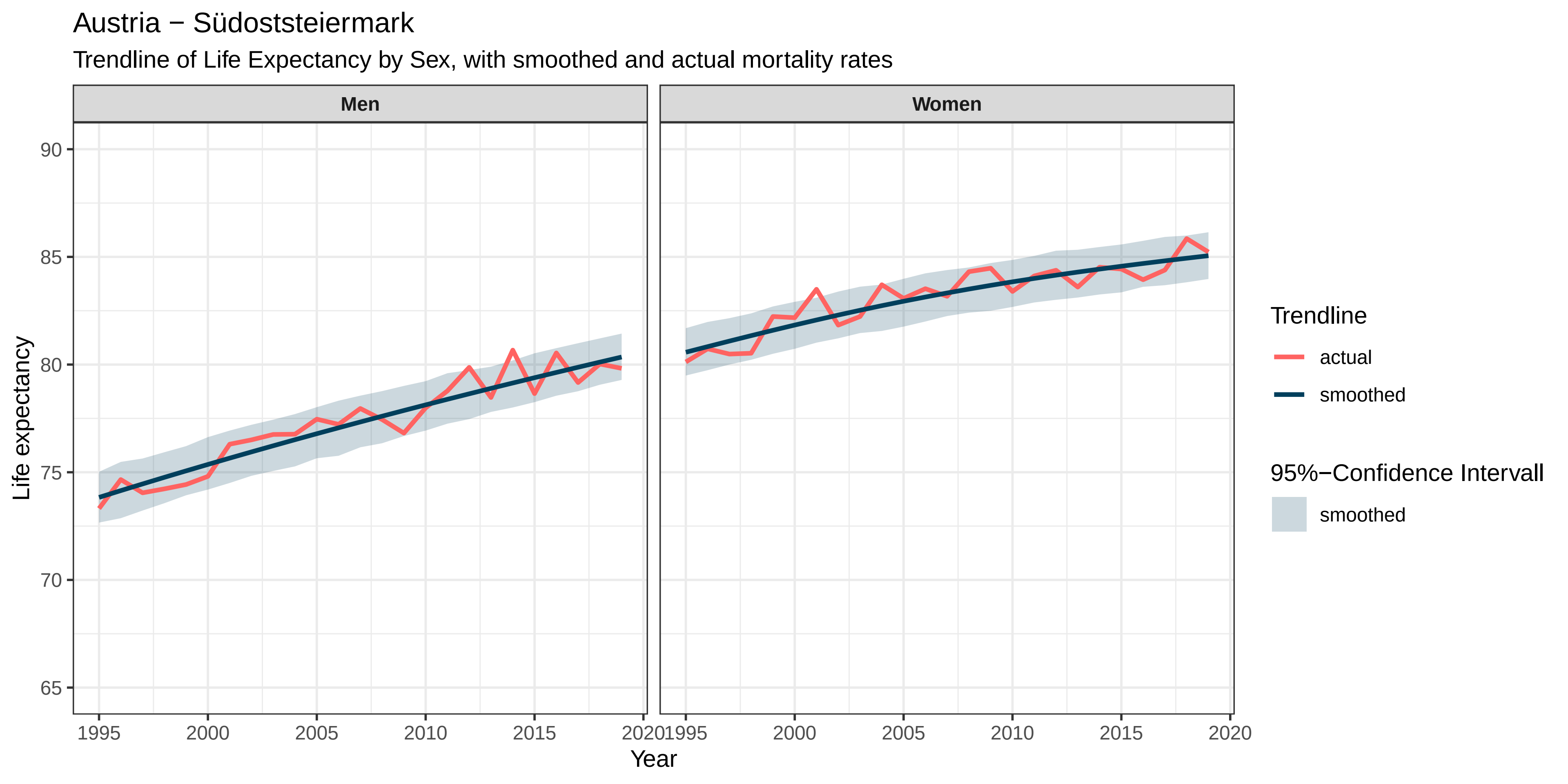

Austria − Südoststeiermark
Trendline of Life Expectancy by Sex, with smoothed and actual mortality rates
Men
Women
Life expectancy
90
85
80
75
70
65
1995
2000
2005
2010
2015
2020
Year
Trendline
actual
smoothed
95%−Confidence Intervall
smoothed

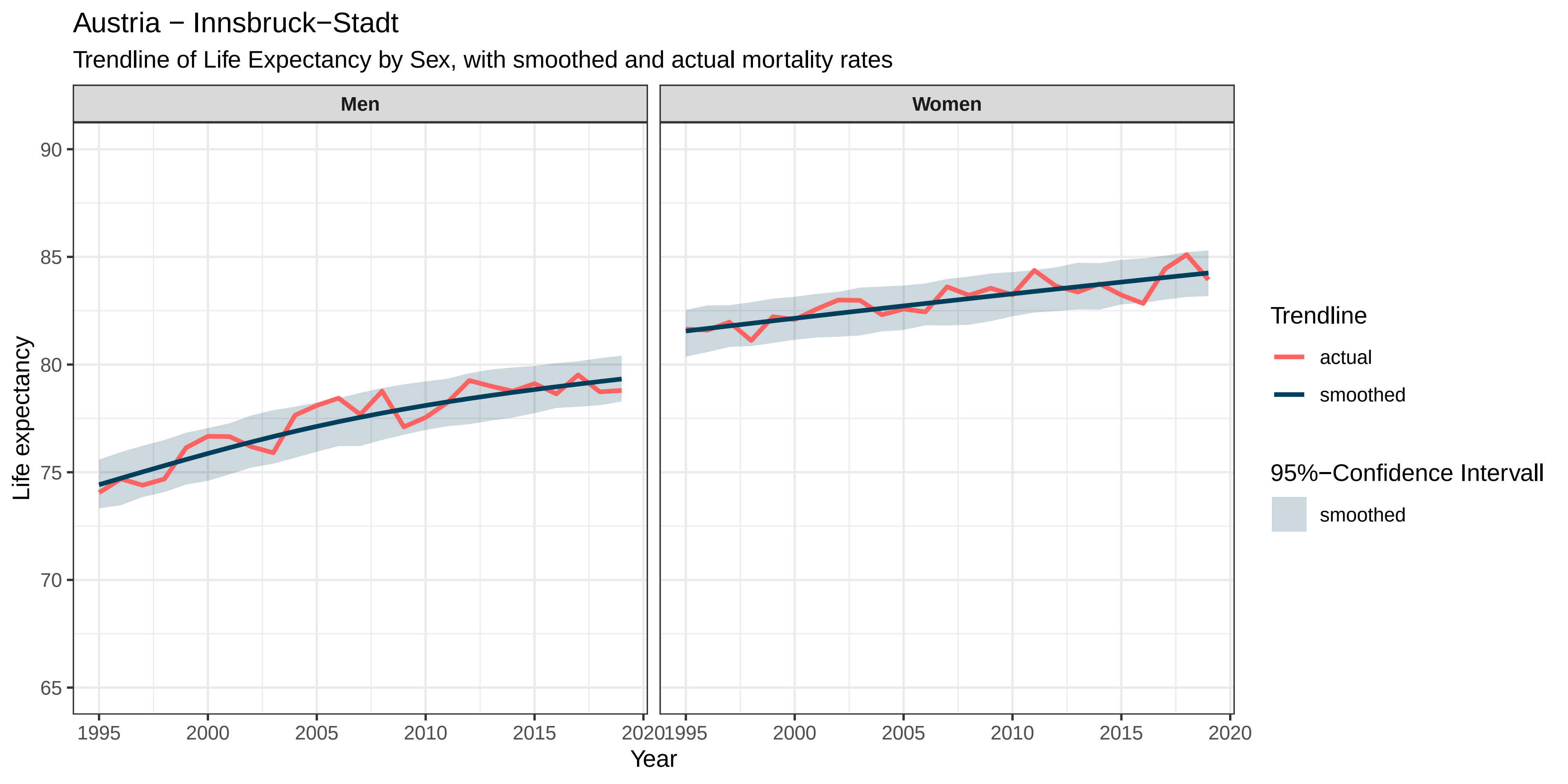

Austria − Innsbruck−Stadt
Trendline of Life Expectancy by Sex, with smoothed and actual mortality rates
Men
Women
90
85
80
75
70
65
Life expectancy
1995
2000
2005
2010
2015
2020
Year
Trendline
actual
smoothed
95%−Confidence Intervall
smoothed

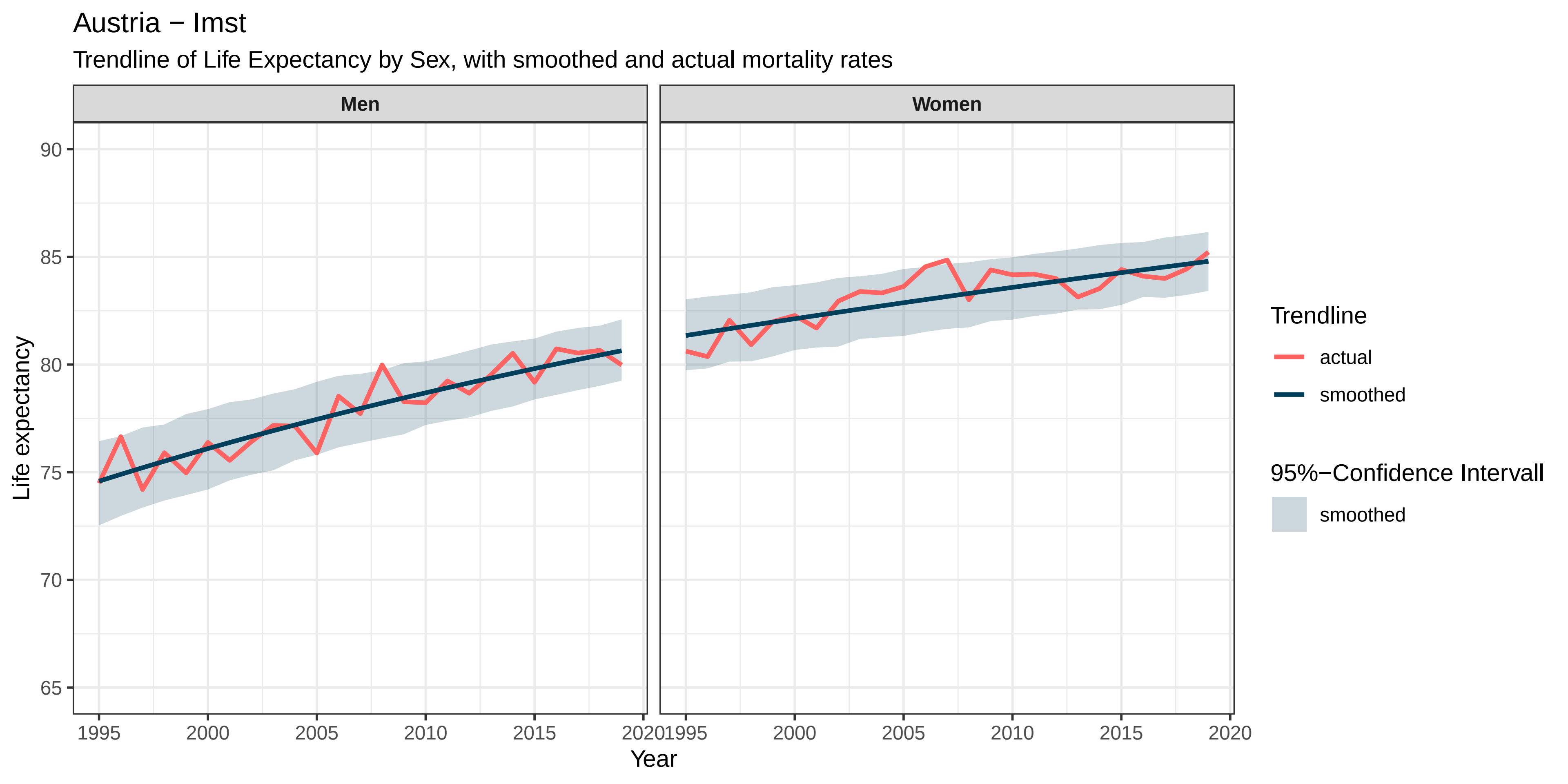

Austria − Imst
Trendline of Life Expectancy by Sex, with smoothed and actual mortality rates
Men
Women
90
85
80
75
70
65
Life expectancy
1995
2000
2005
2010
2015
2020
Year
Trendline
actual
smoothed
95%−Confidence Intervall
smoothed

Austria − Innsbruck−Land

Trendline of Life Expectancy by Sex, with smoothed and actual mortality rates

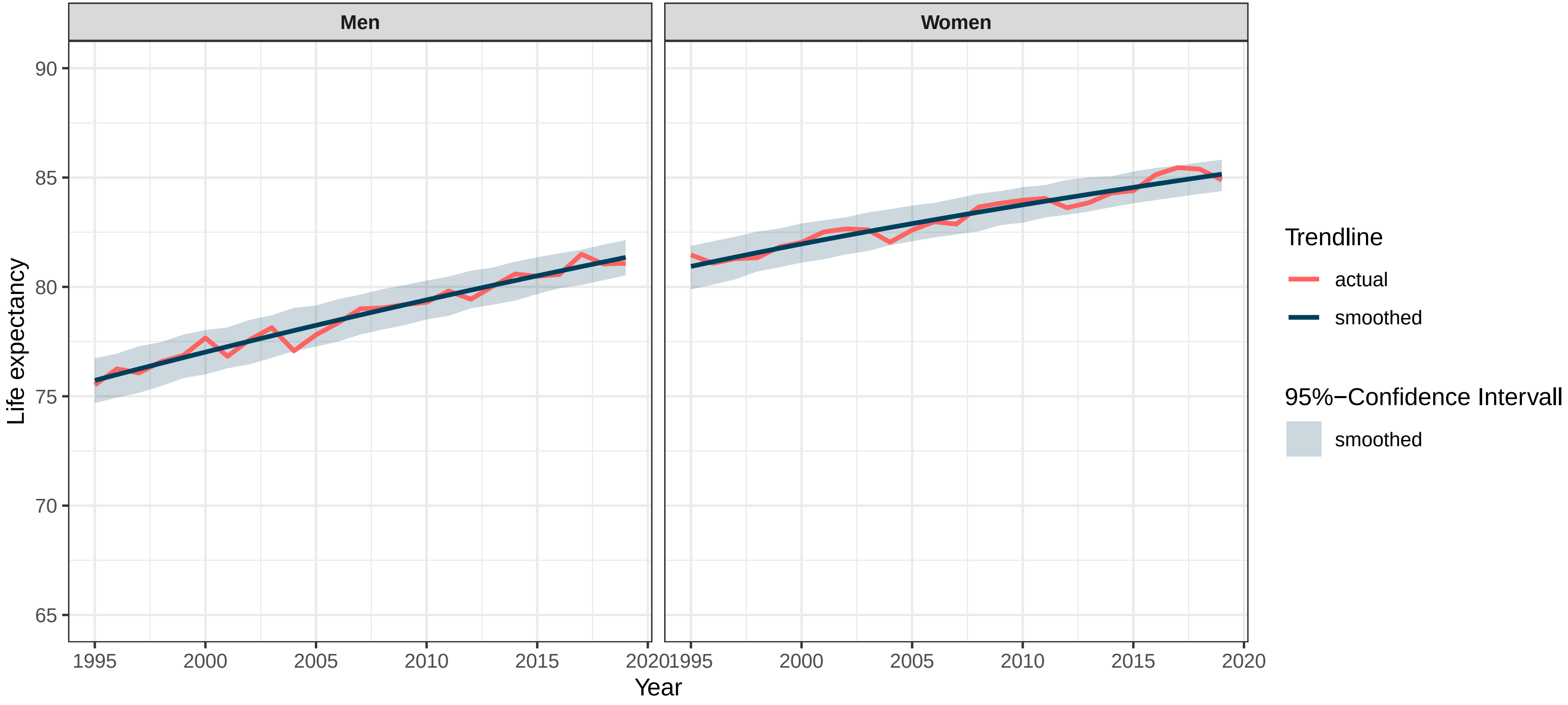

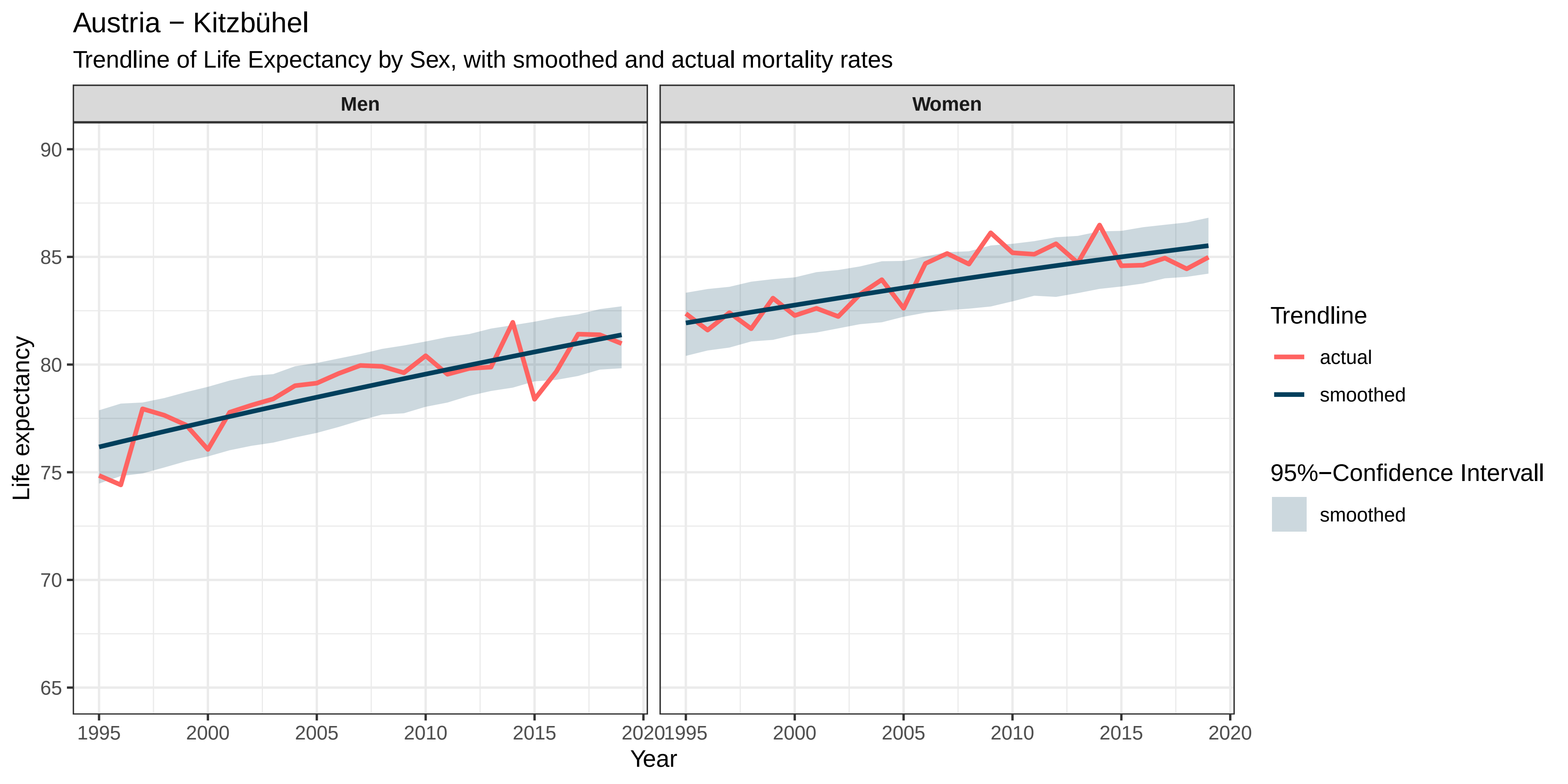

Austria − Kitzbühel
Trendline of Life Expectancy by Sex, with smoothed and actual mortality rates
Men
Women
Life expectancy
90
85
80
75
70
65
1995
2000
2005
2010
2015
2020
Year
Trendline
actual
smoothed
95%−Confidence Intervall
smoothed

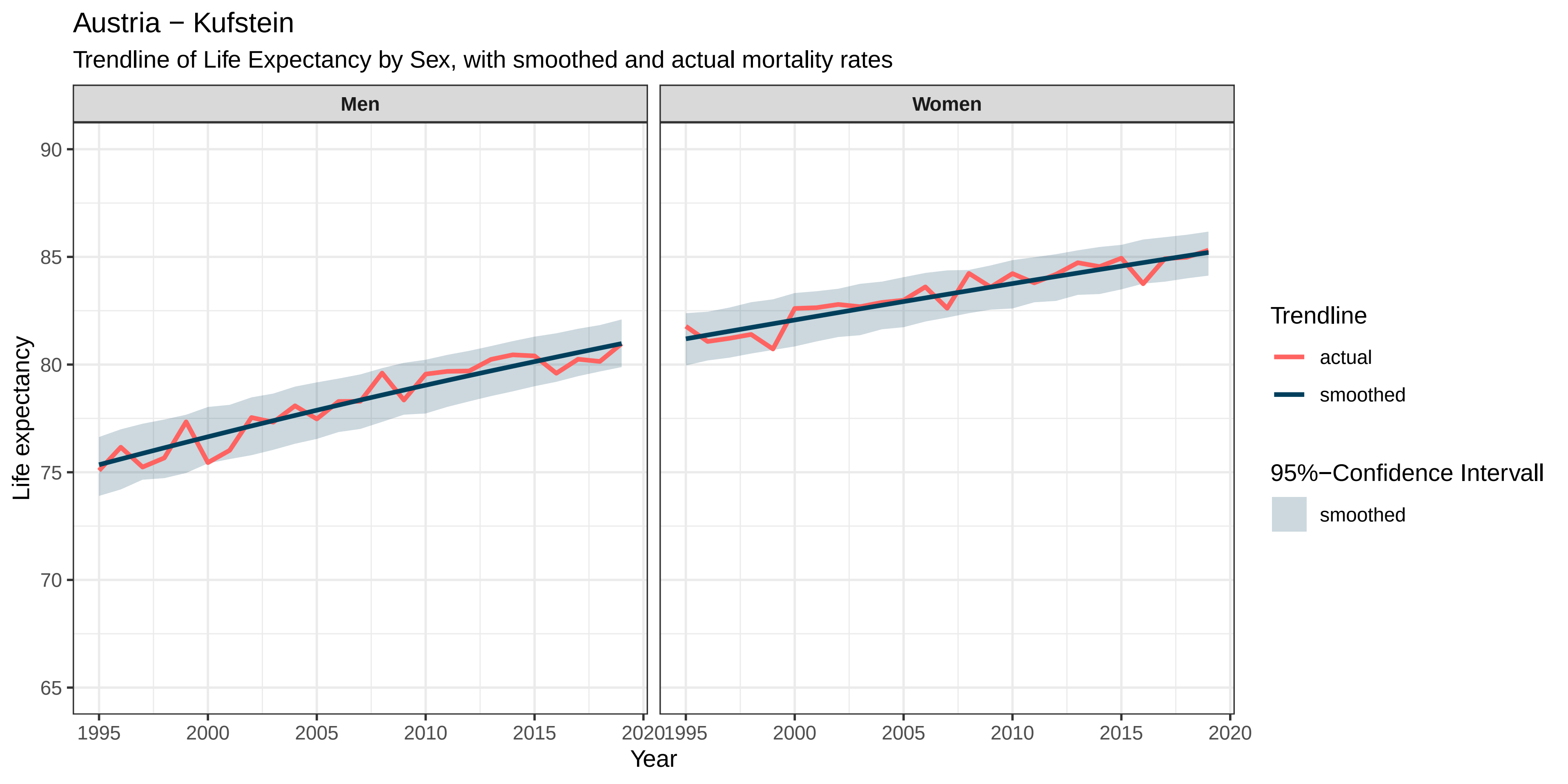
Austria − Kufstein
Trendline of Life Expectancy by Sex, with smoothed and actual mortality rates
Men
Women
Life expectancy
Year
Trendline
actual
smoothed
95%−Confidence Intervall
smoothed

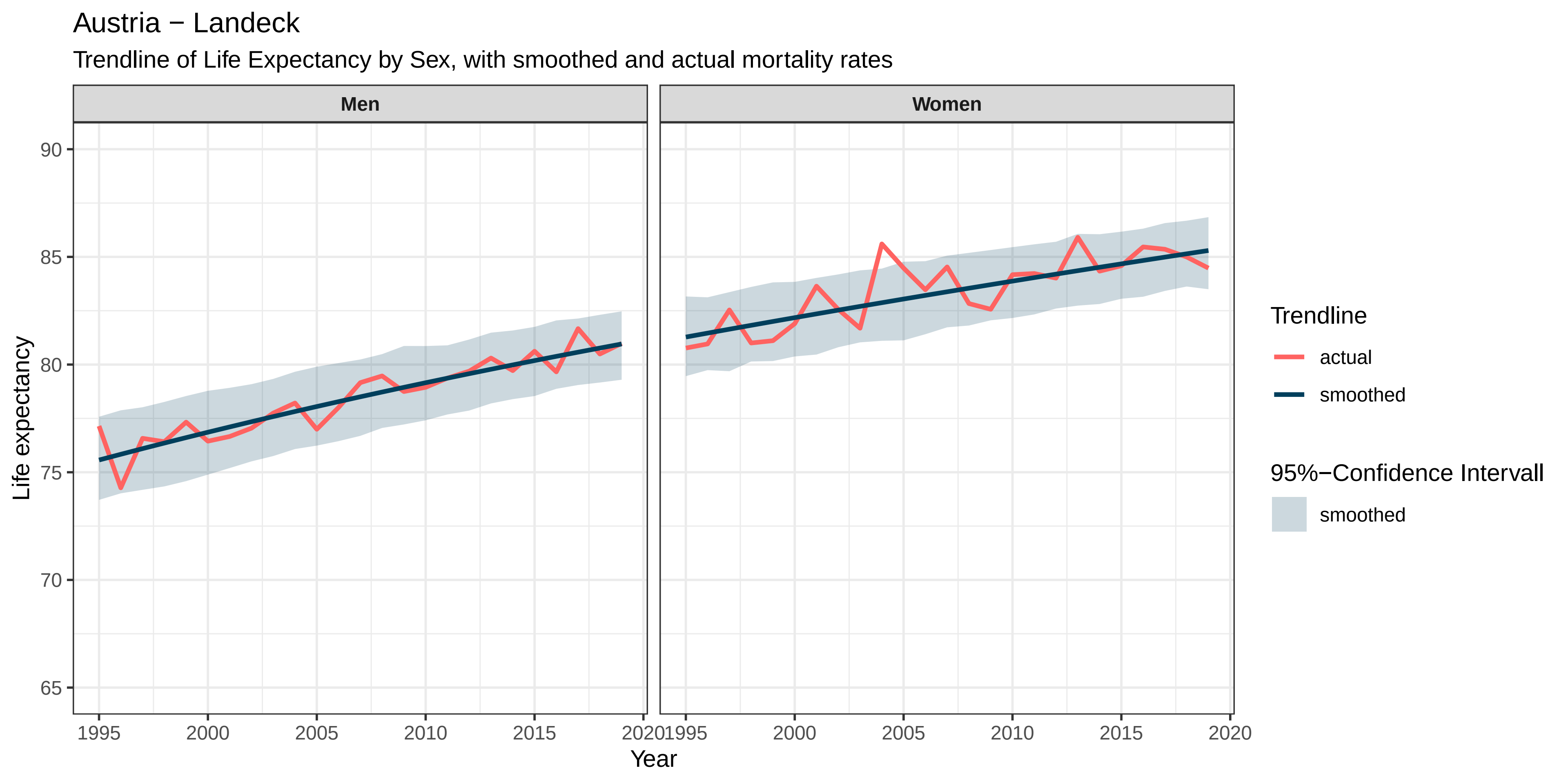
Austria − Landeck
Trendline of Life Expectancy by Sex, with smoothed and actual mortality rates
Men
Women
Life expectancy
90
85
80
75
70
65
1995
2000
2005
2010
2015
2020
Year
Trendline
actual
smoothed
95%−Confidence Intervall
smoothed

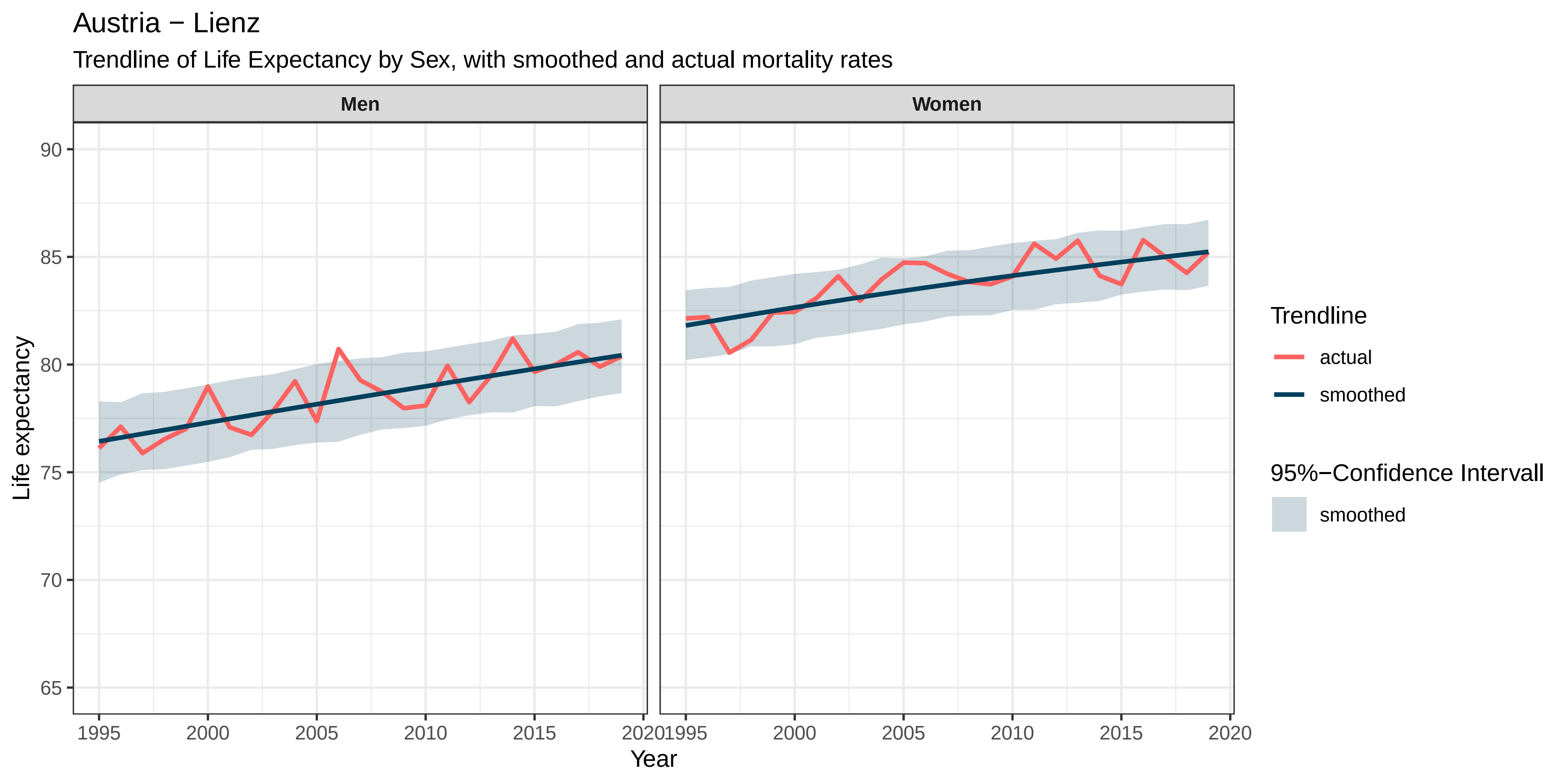
Austria − Lienz
Trendline of Life Expectancy by Sex, with smoothed and actual mortality rates
Men
Women
Life expectancy
Year
90
85
80
75
70
65
1995
2000
2005
2010
2015
2020
Trendline
actual
smoothed
95%−Confidence Intervall
smoothed

Austria − Reutte

Trendline of Life Expectancy by Sex, with smoothed and actual mortality rates

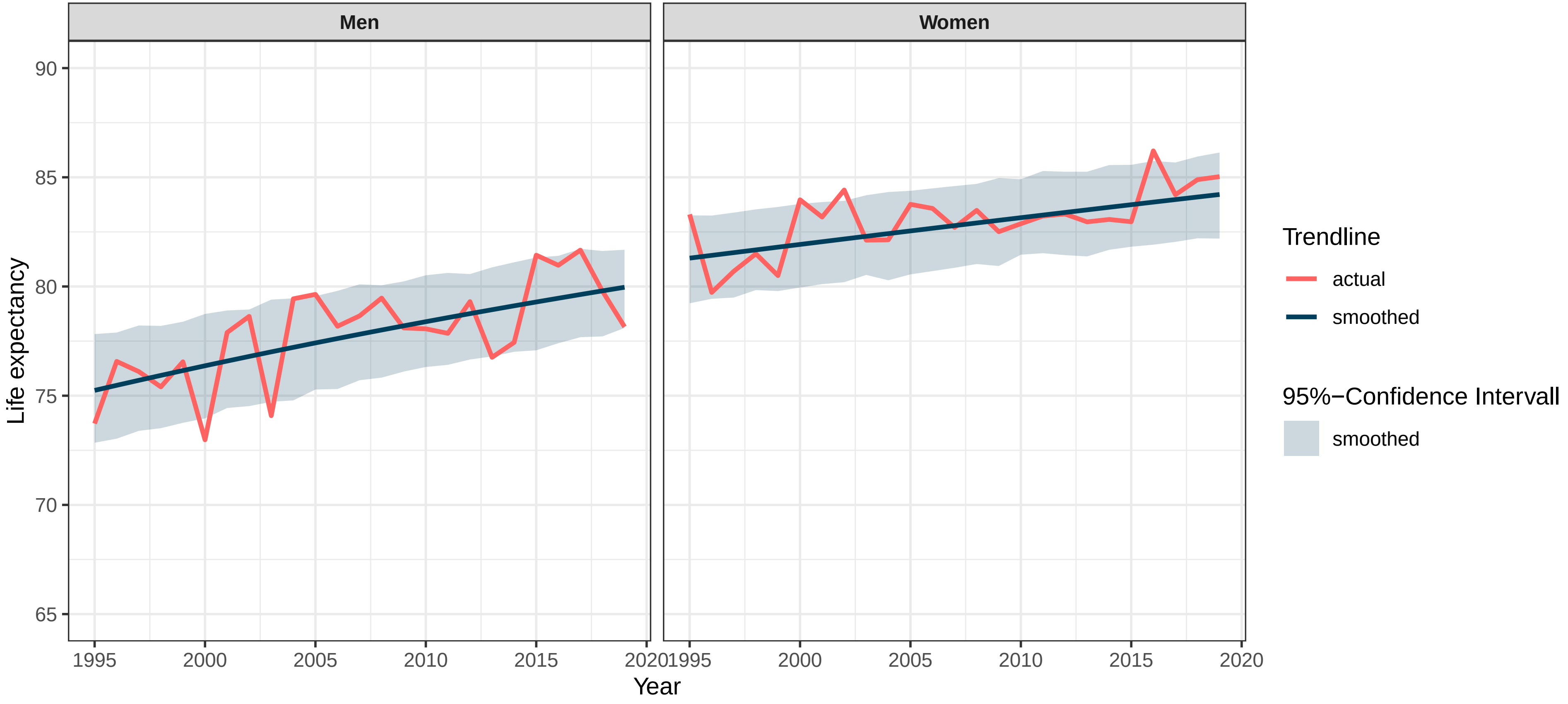

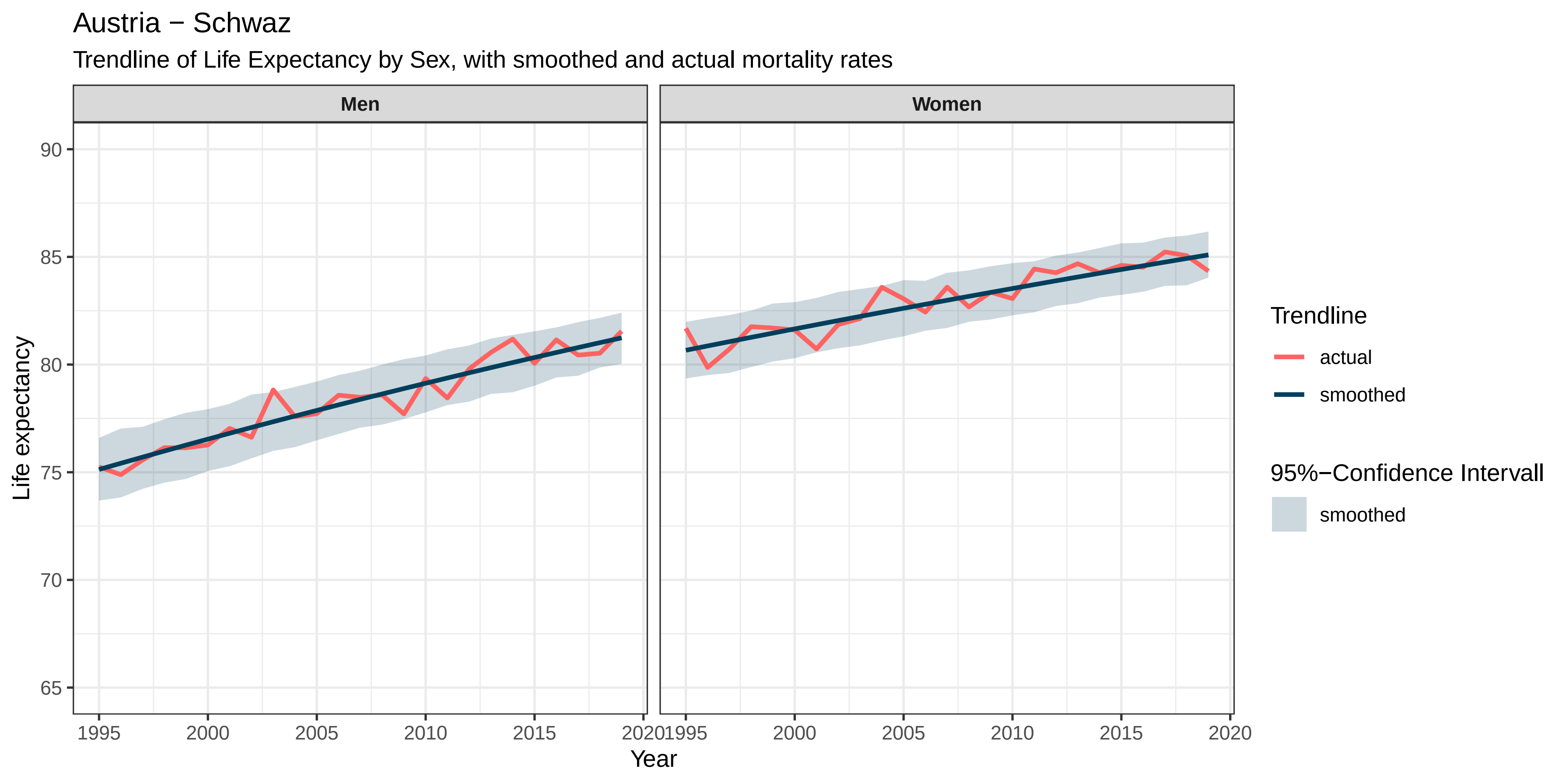

Austria − Schwaz
Trendline of Life Expectancy by Sex, with smoothed and actual mortality rates
Men
Women
Life expectancy
90
85
80
75
70
65
1995
2000
2005
2010
2015
2020
Year
Trendline
actual
smoothed
95%−Confidence Intervall
smoothed

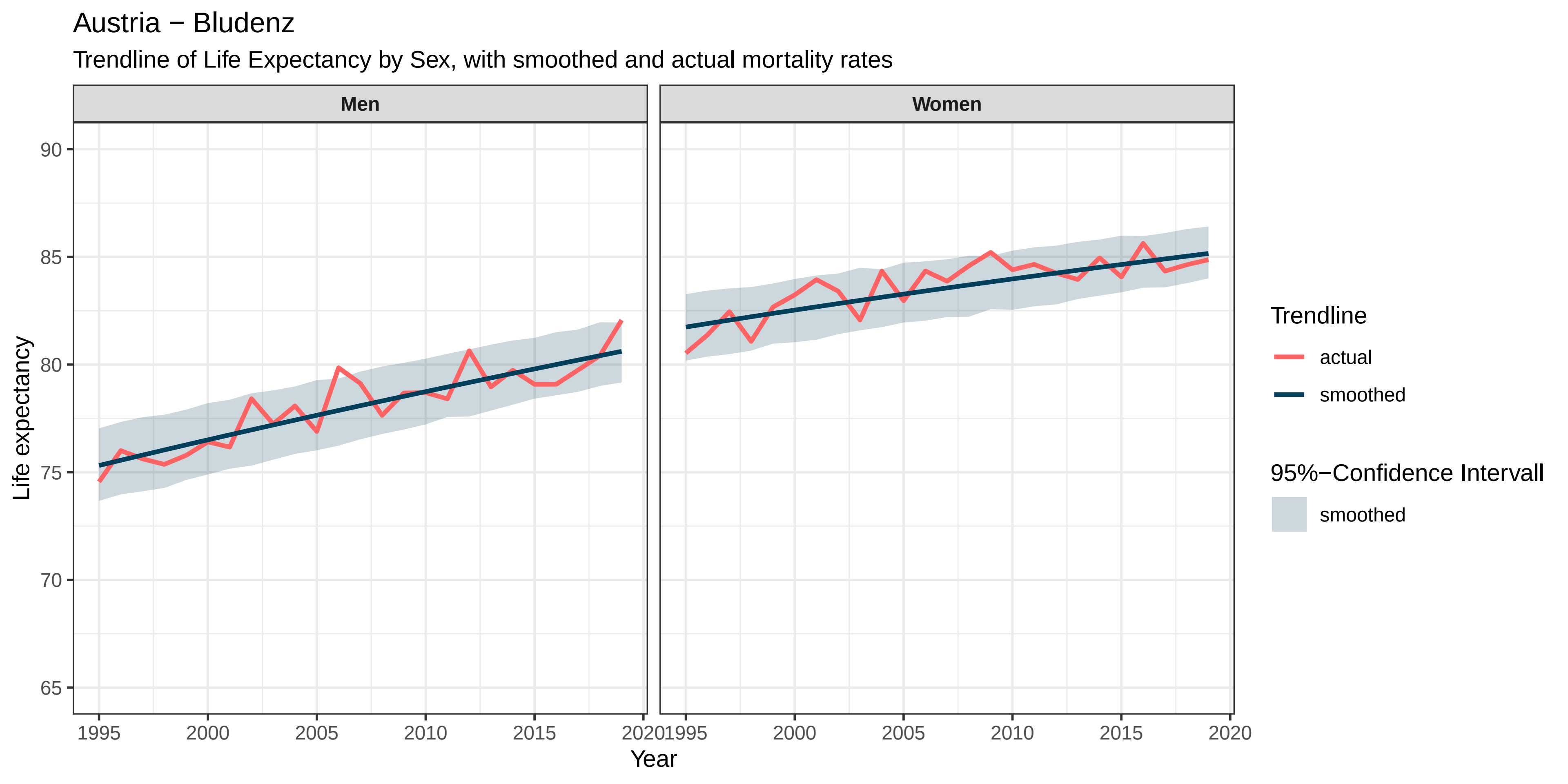

Austria − Bludenz
Trendline of Life Expectancy by Sex, with smoothed and actual mortality rates
Men
Women
Life expectancy
90
85
80
75
70
65
1995
2000
2005
2010
2015
2020
Year
Trendline
actual
smoothed
95%−Confidence Intervall
smoothed

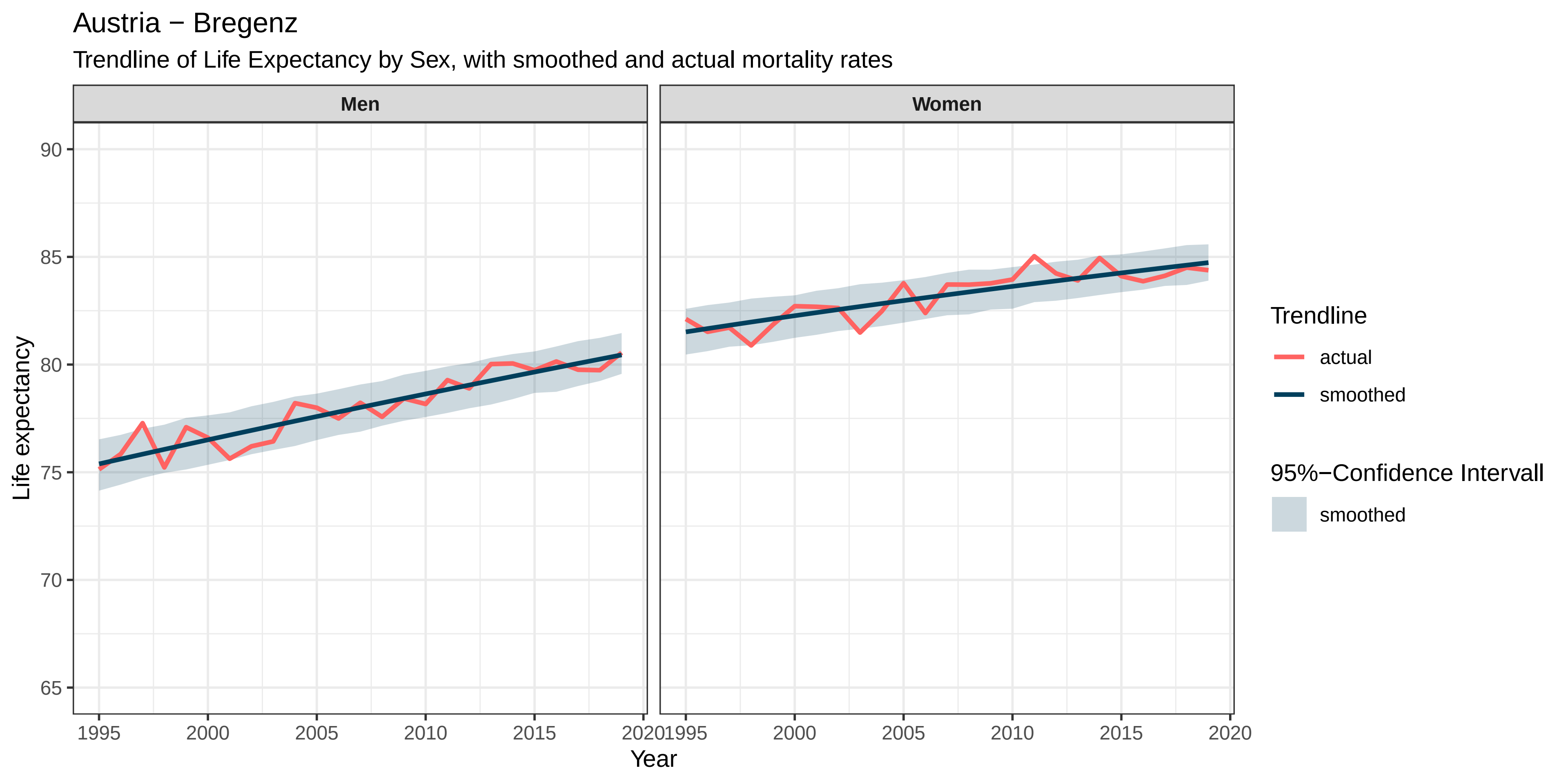
Austria − Bregenz
Trendline of Life Expectancy by Sex, with smoothed and actual mortality rates
Men
Women
Life expectancy
90
85
80
75
70
65
1995
2000
2005
2010
2015
2020
Year
Trendline
actual
smoothed
95%−Confidence Intervall
smoothed

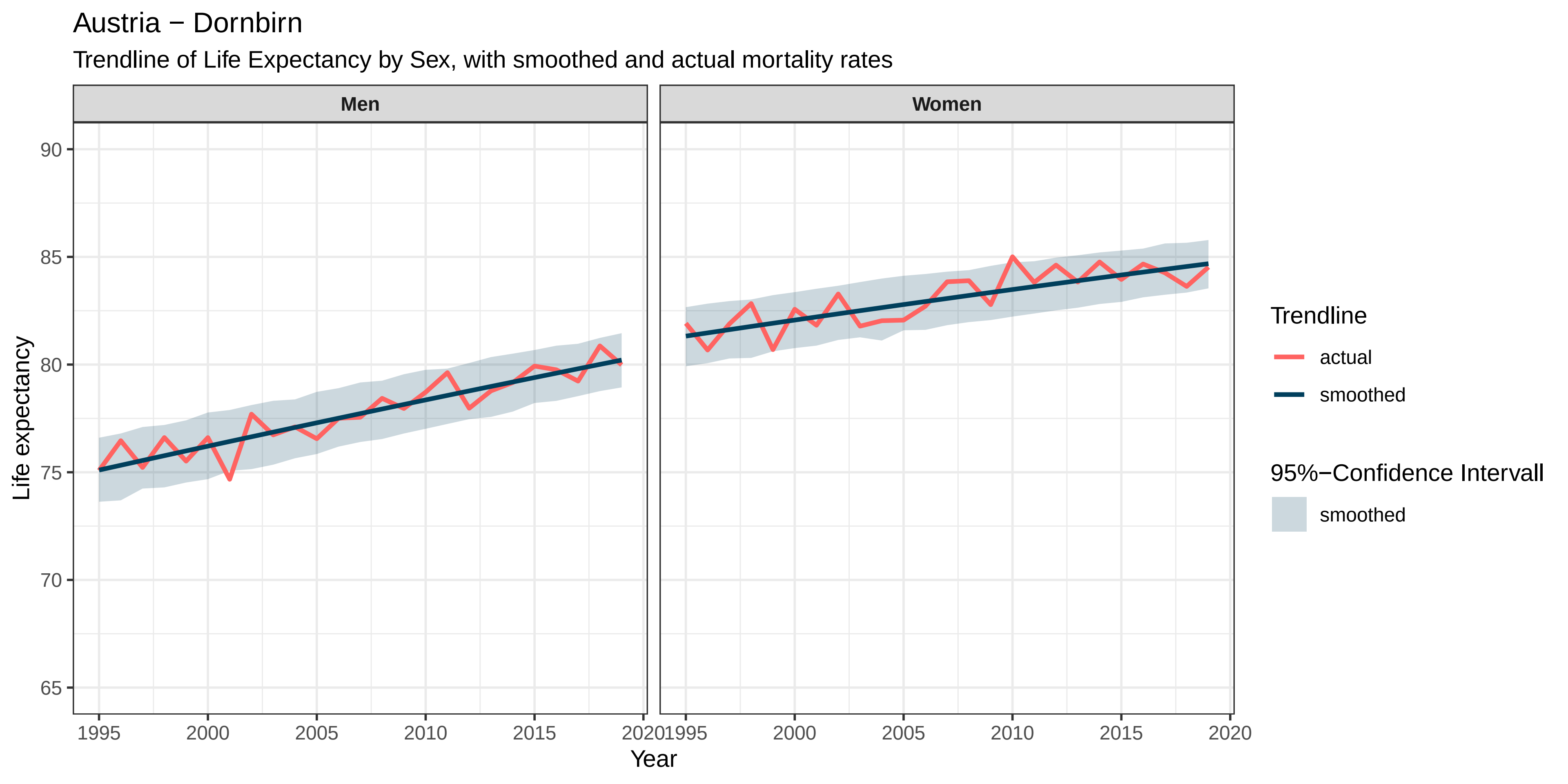
Austria − Dornbirn
Trendline of Life Expectancy by Sex, with smoothed and actual mortality rates
Men
Women
Life expectancy
90
85
80
75
70
65
1995
2000
2005
2010
2015
2020
Year
Trendline
actual
smoothed
95%−Confidence Intervall
smoothed

Austria − Feldkirch

Trendline of Life Expectancy by Sex, with smoothed and actual mortality rates

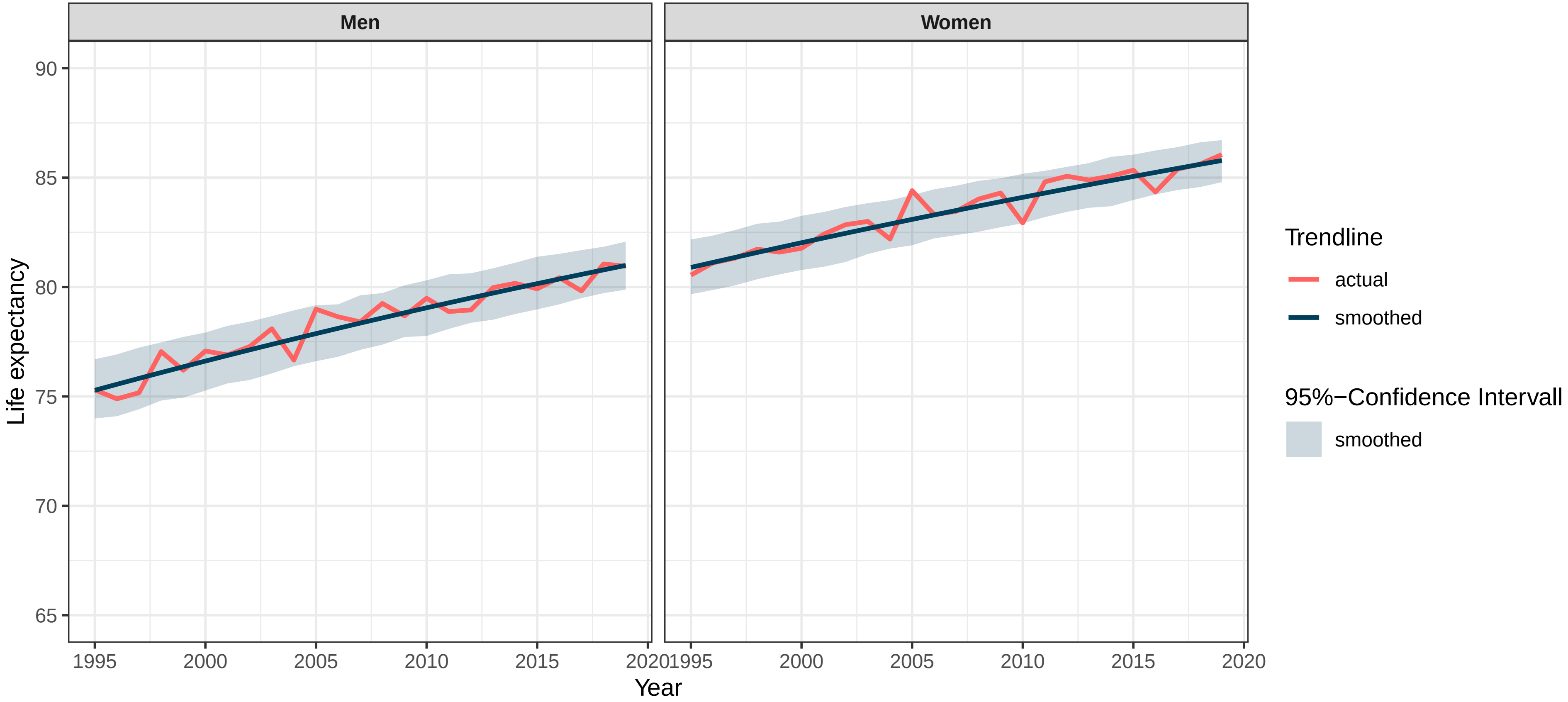

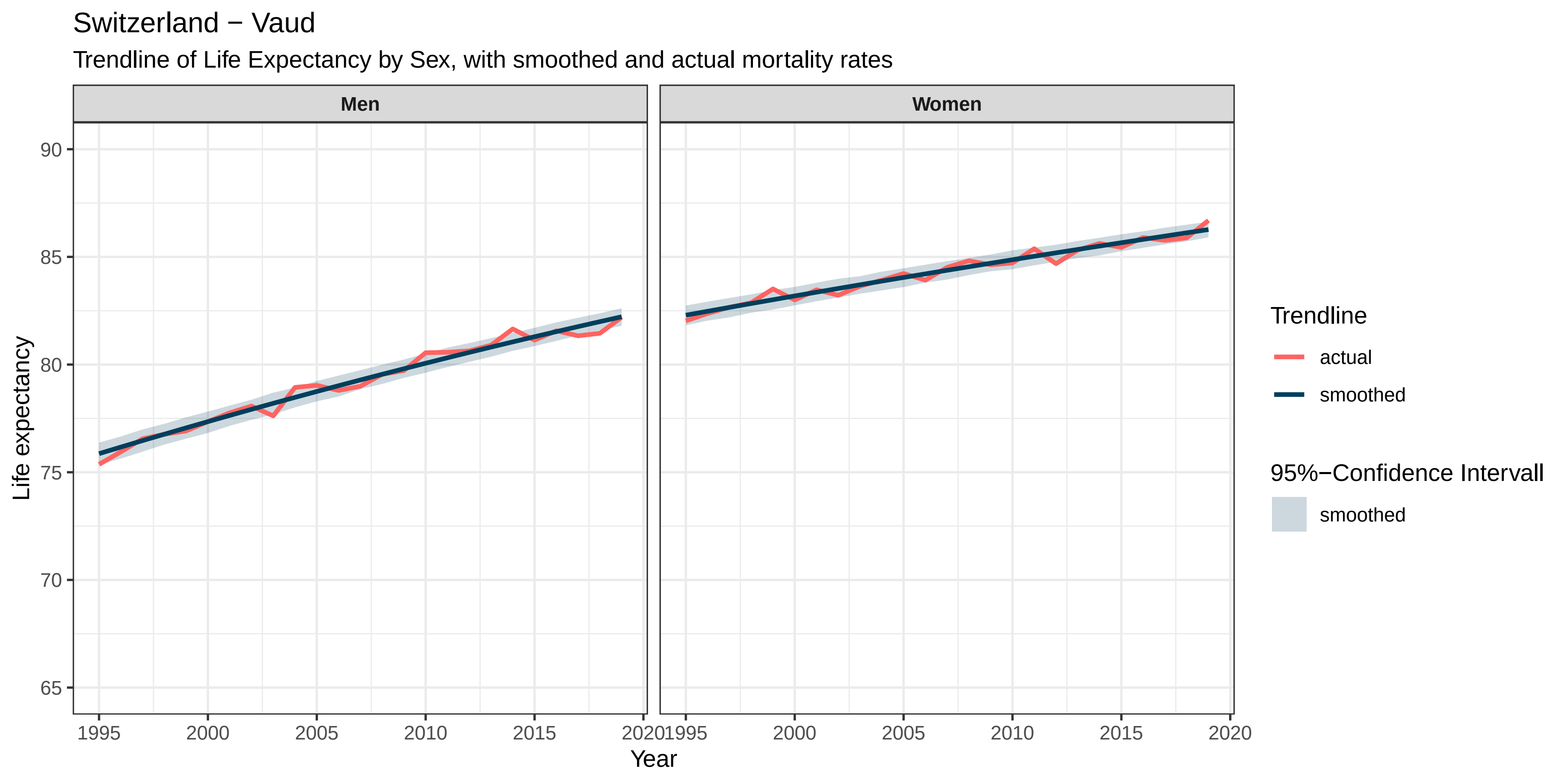

Switzerland − Vaud
Trendline of Life Expectancy by Sex, with smoothed and actual mortality rates
Men
Women
90
85
80
75
70
65
Life expectancy
1995
2000
2005
2010
2015
2020
Year
Trendline
actual
smoothed
95%−Confidence Intervall
smoothed

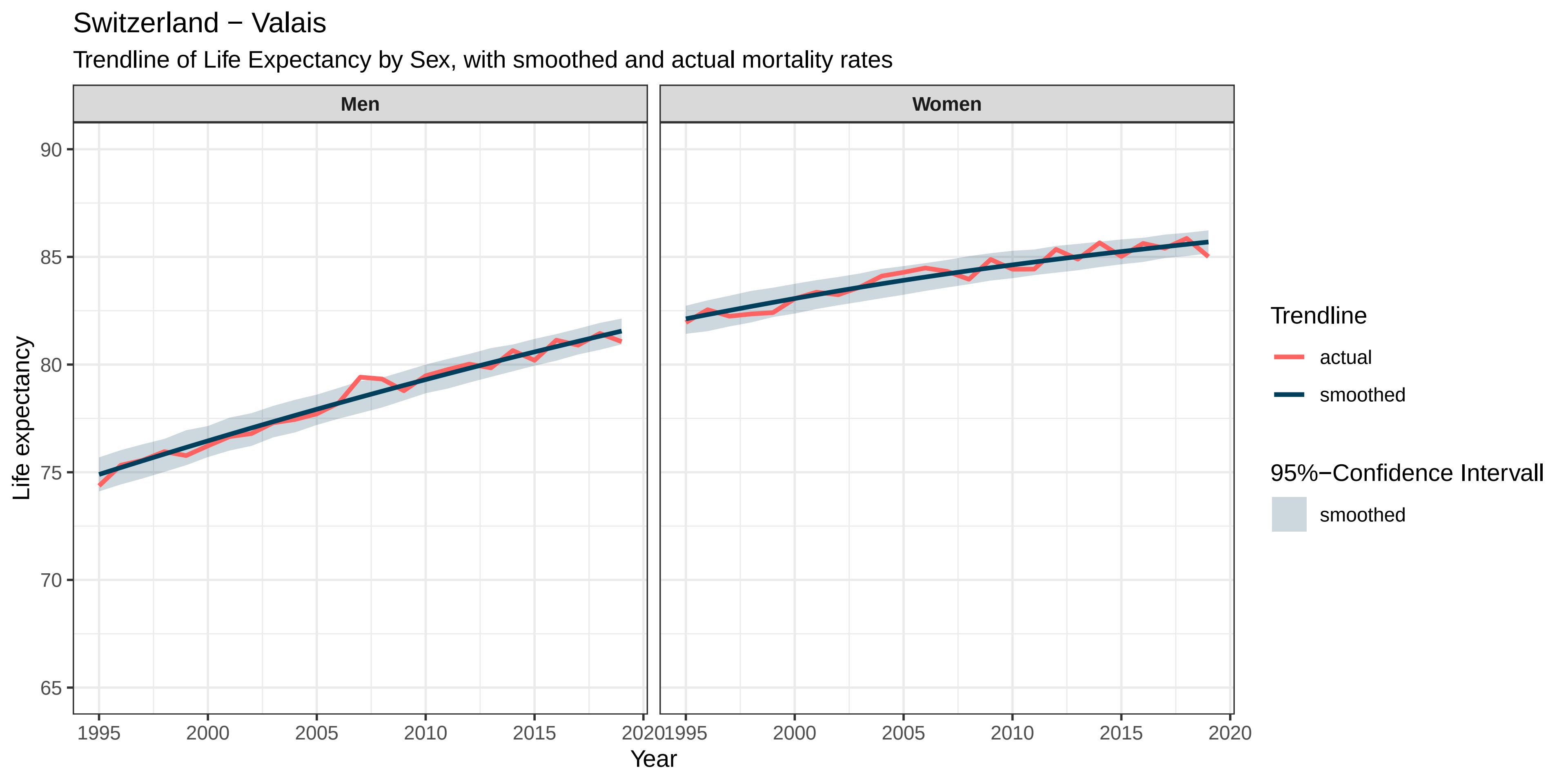

Switzerland − Valais
Trendline of Life Expectancy by Sex, with smoothed and actual mortality rates
Men
Women
90
85
80
75
70
65
Life expectancy
1995
2000
2005
2010
2015
2020
Year
Trendline
actual
smoothed
95%−Confidence Intervall
smoothed

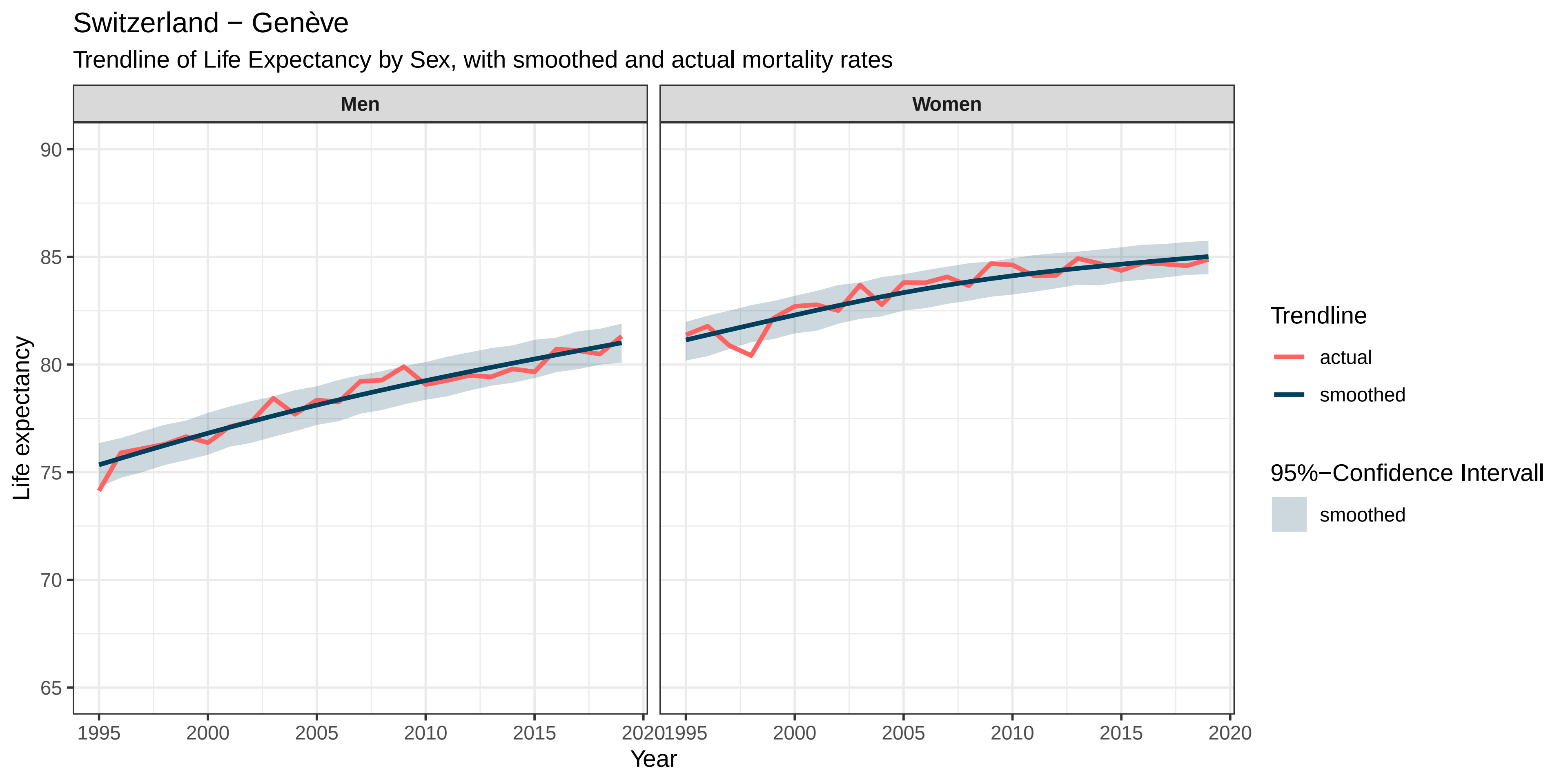

Switzerland − Genève
Trendline of Life Expectancy by Sex, with smoothed and actual mortality rates
Men
Women
Life expectancy
Year
90
85
80
75
70
65
1995
2000
2005
2010
2015
2020
Trendline
actual
smoothed
95%−Confidence Intervall
smoothed

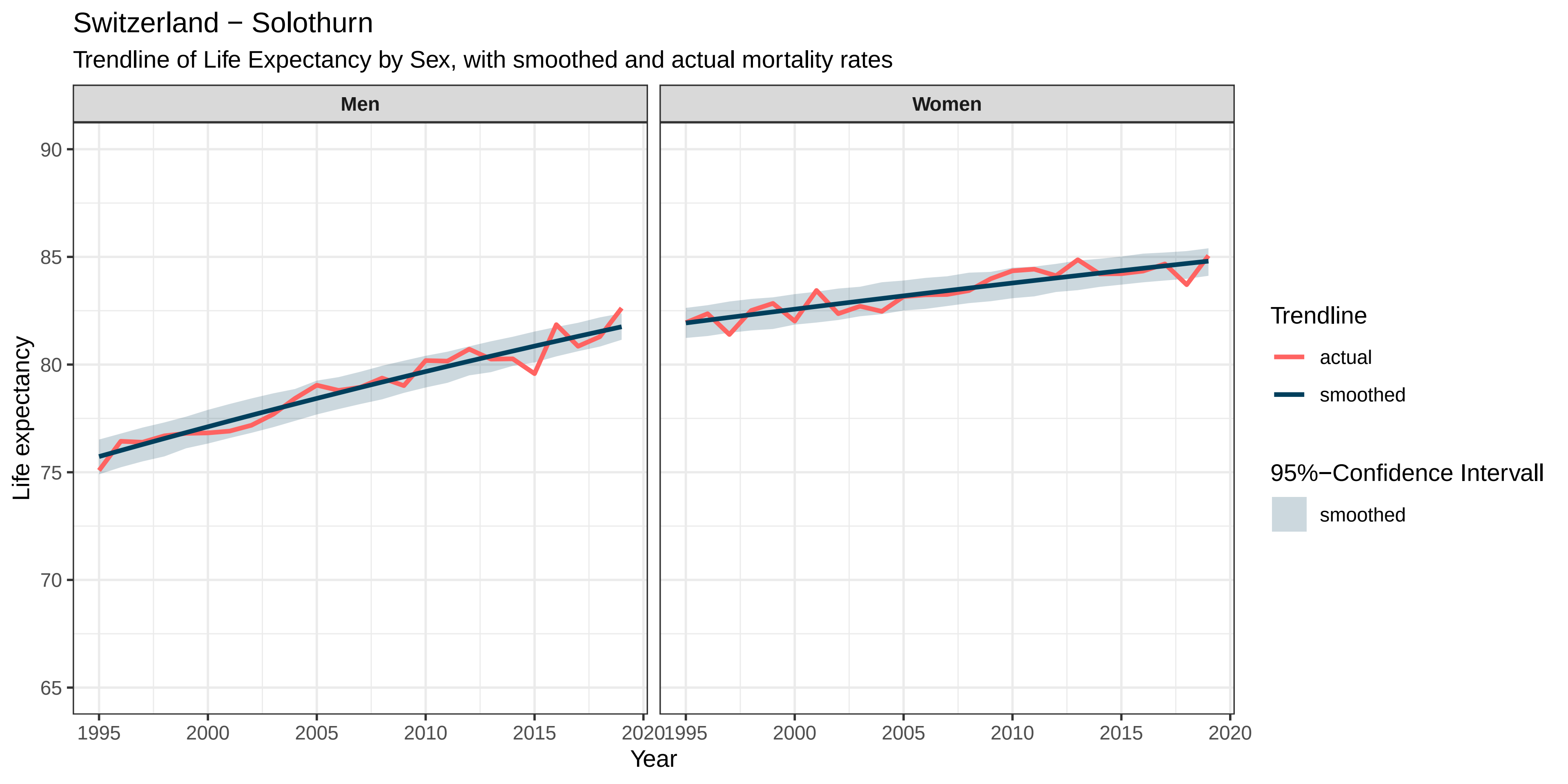
Switzerland − Solothurn
Trendline of Life Expectancy by Sex, with smoothed and actual mortality rates
Men
Women
Life expectancy
Year
90
85
80
75
70
65
1995
2000
2005
2010
2015
2020
Trendline
actual
smoothed
95%−Confidence Intervall
smoothed

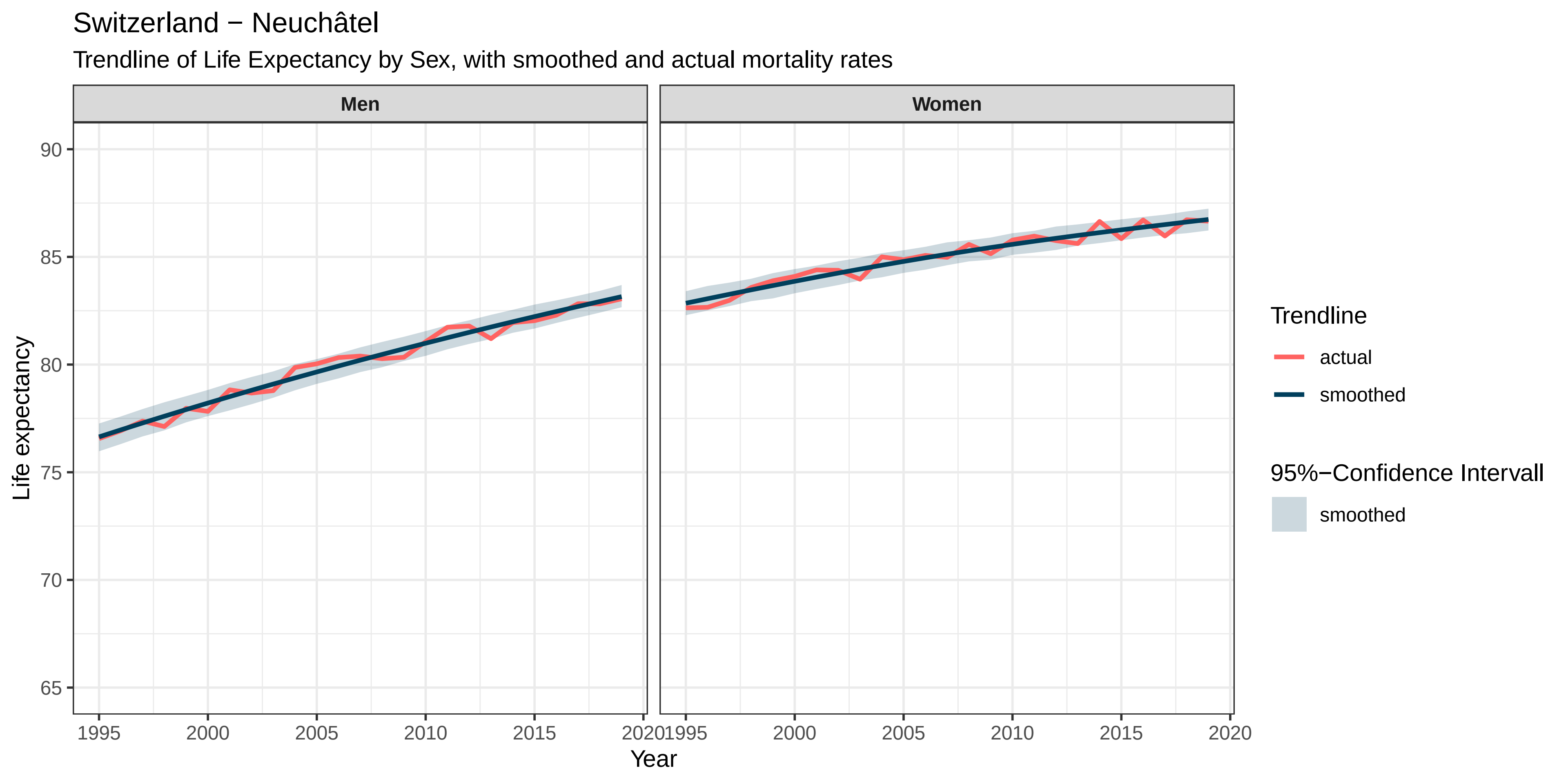

Switzerland − Neuchâtel
Trendline of Life Expectancy by Sex, with smoothed and actual mortality rates
Men
Women
Life expectancy
90
85
80
75
70
65
1995
2000
2005
2010
2015
2020
Year
Trendline
actual
smoothed
95%−Confidence Intervall
smoothed

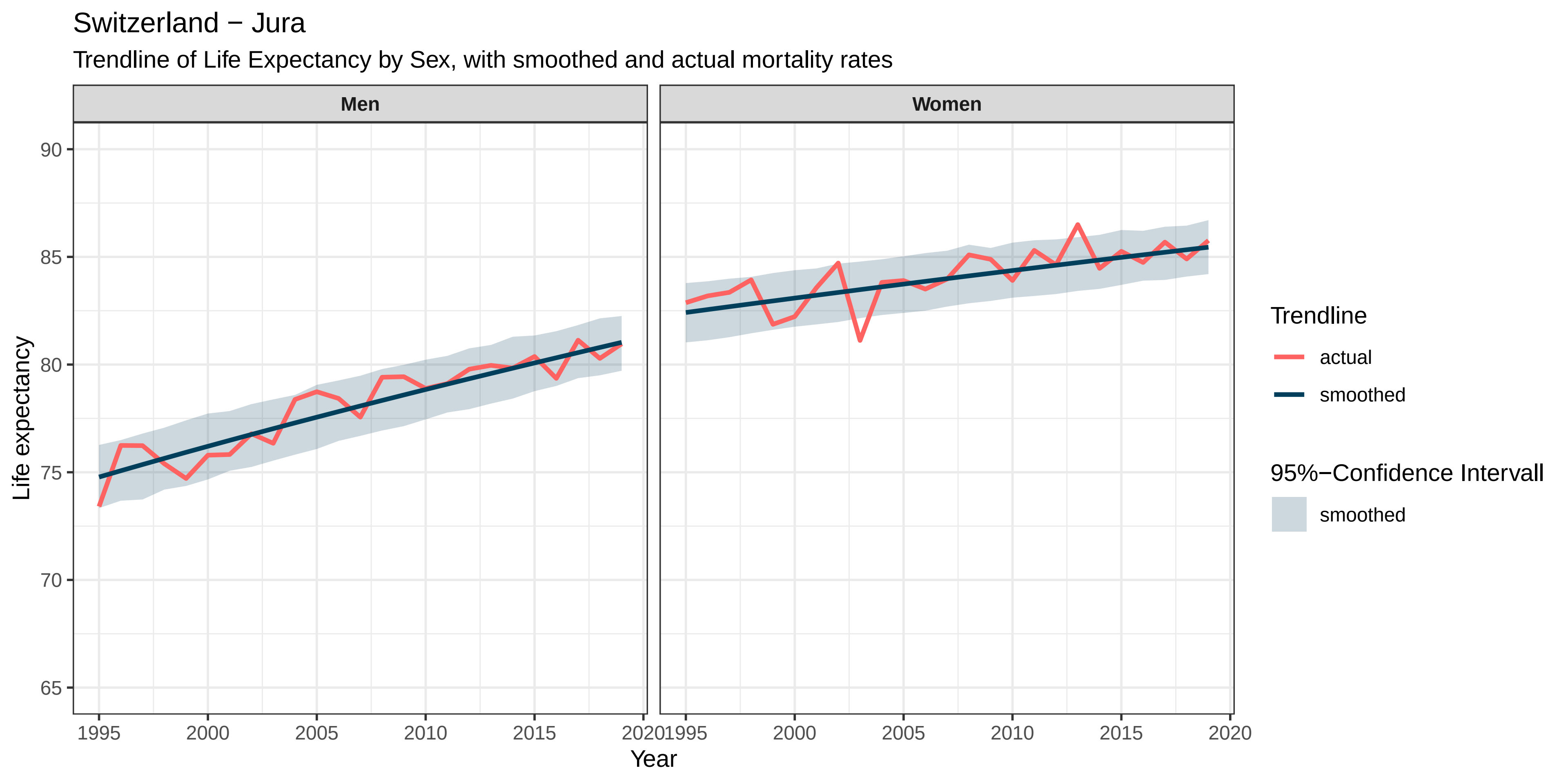

Switzerland − Jura
Trendline of Life Expectancy by Sex, with smoothed and actual mortality rates
Men
Women
90
85
80
75
70
65
Life expectancy
1995
2000
2005
2010
2015
2020
Year
Trendline
actual
smoothed
95%−Confidence Intervall
smoothed

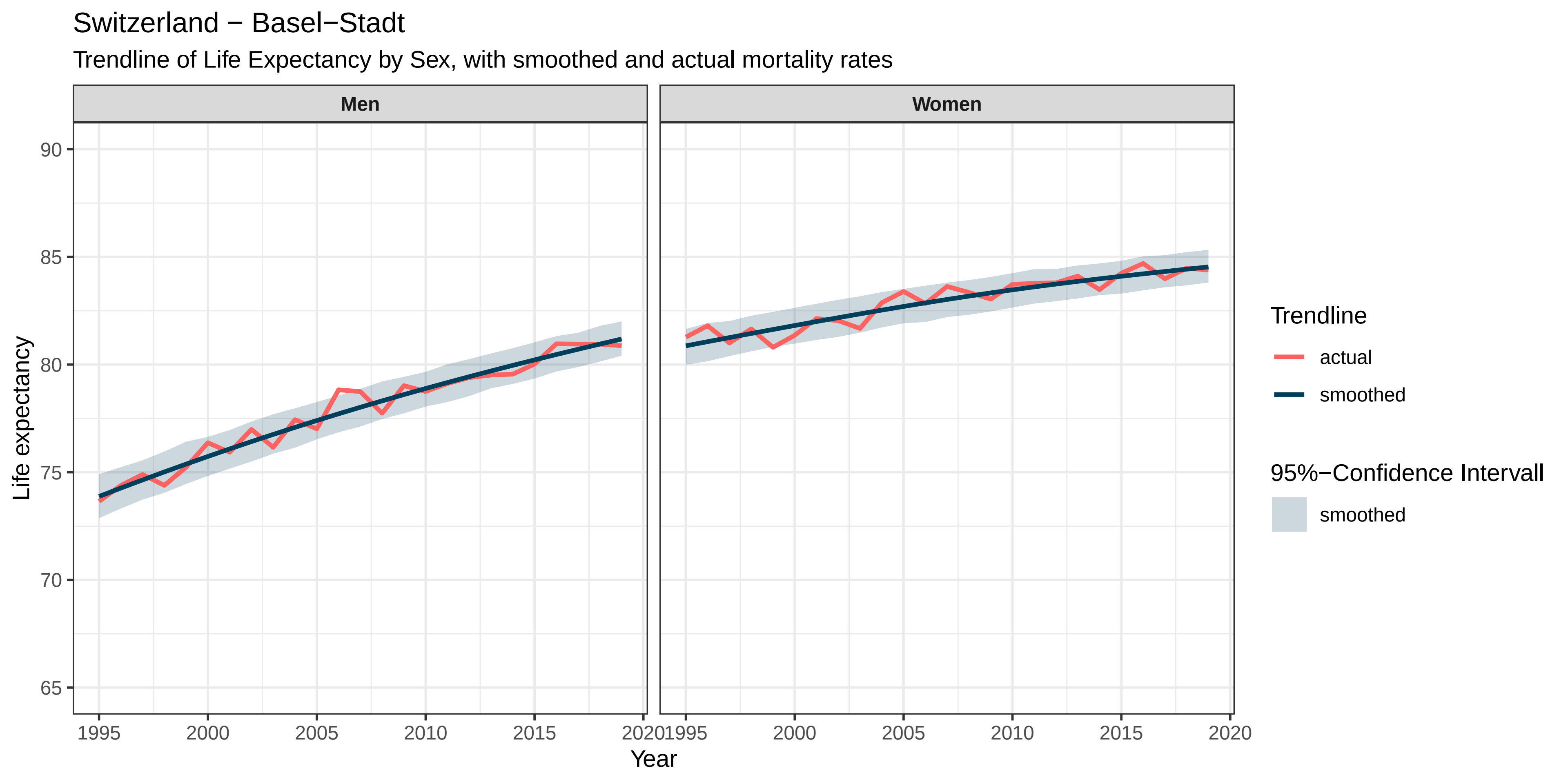

Switzerland − Basel−Stadt
Trendline of Life Expectancy by Sex, with smoothed and actual mortality rates
Men
Women
Life expectancy
90
85
80
75
70
65
1995
2000
2005
2010
2015
2020
Year
Trendline
actual
smoothed
95%−Confidence Intervall
smoothed

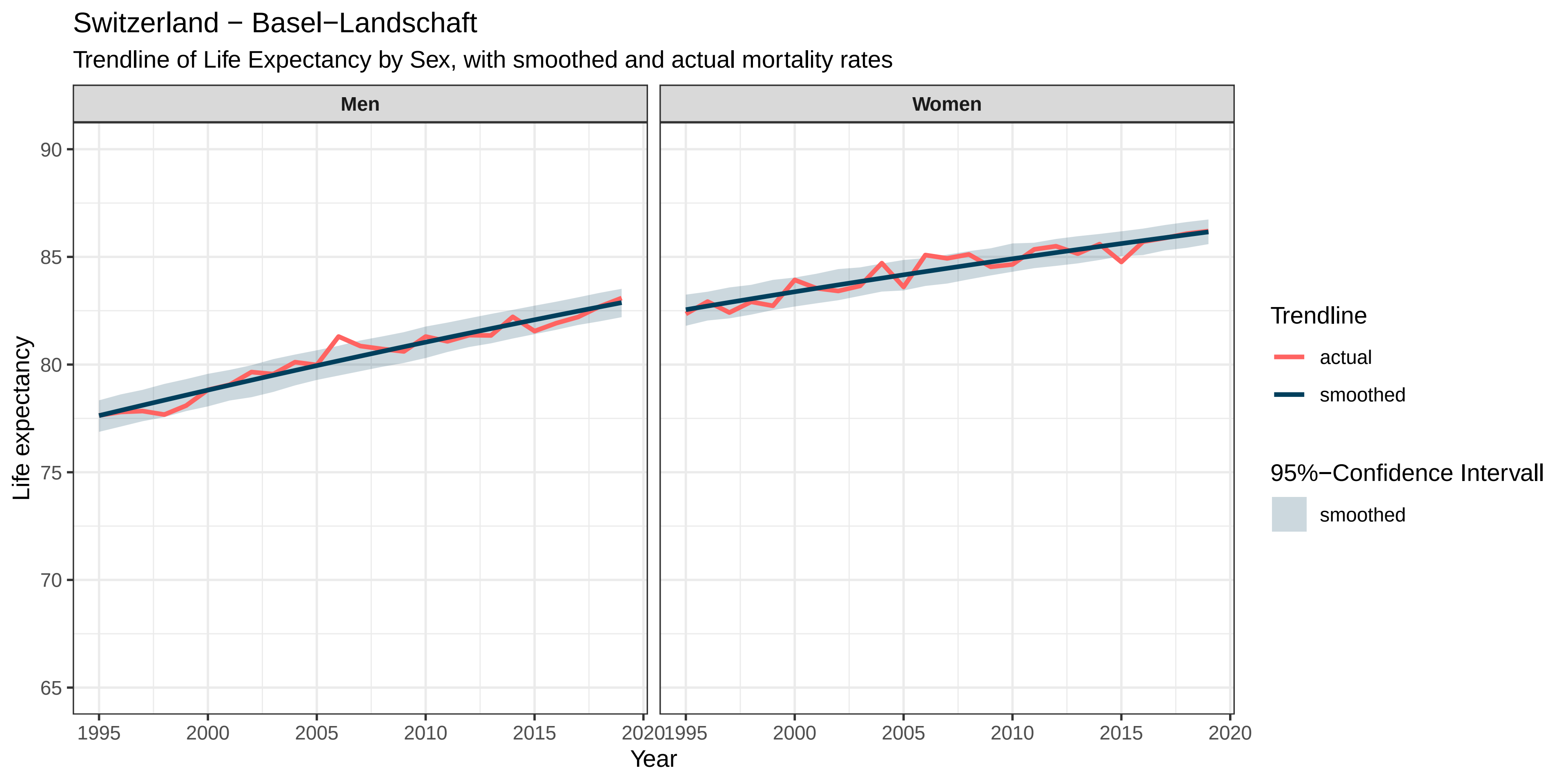

Switzerland − Basel−Landschaft
Trendline of Life Expectancy by Sex, with smoothed and actual mortality rates
Men
Women
Life expectancy
90
85
80
75
70
65
1995
2000
2005
2010
2015
2020
Year
Trendline
actual
smoothed
95%−Confidence Intervall
smoothed

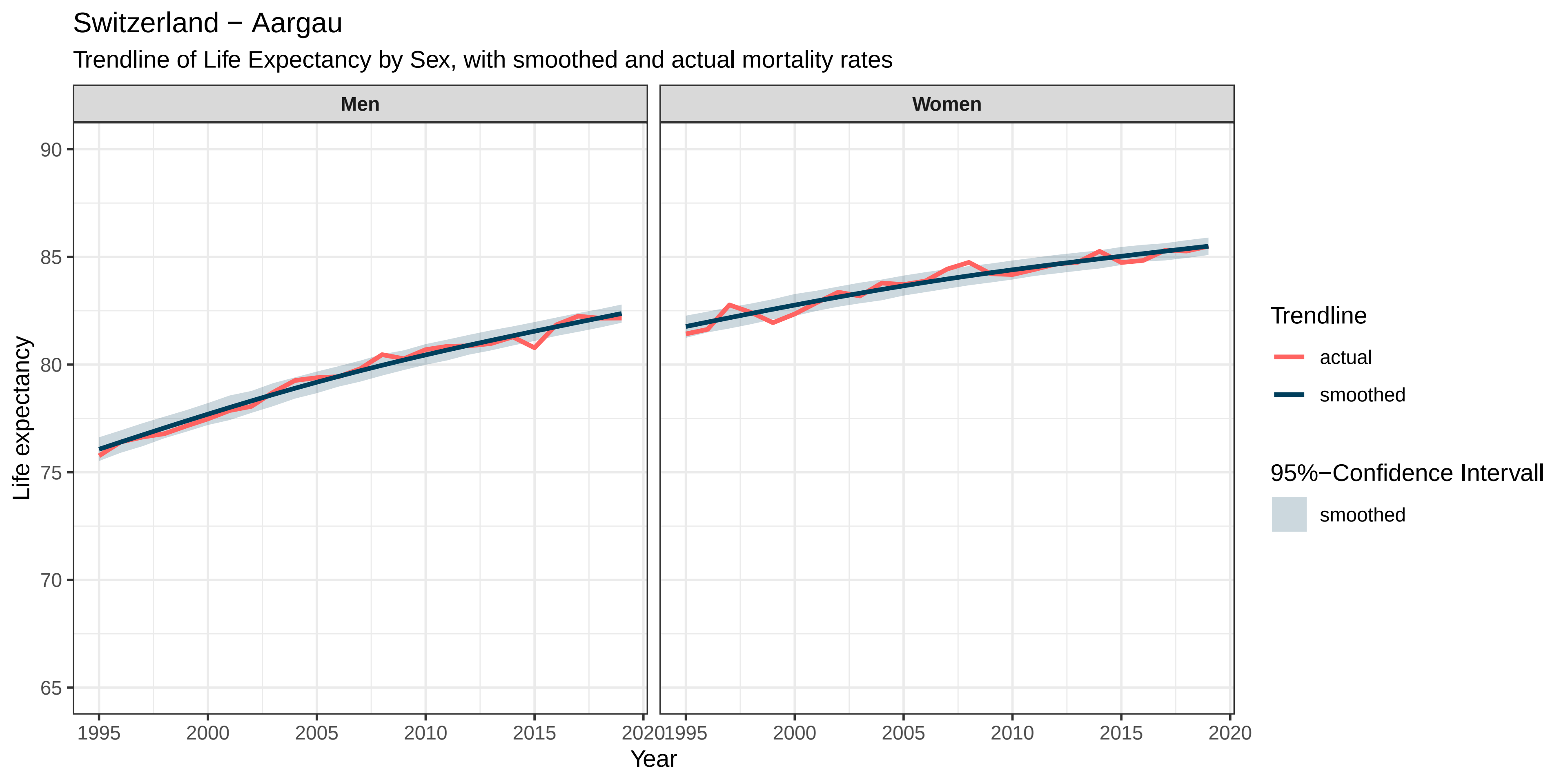
Switzerland − Aargau
Trendline of Life Expectancy by Sex, with smoothed and actual mortality rates
Men
Women
Life expectancy
90
85
80
75
70
65
1995
2000
2005
2010
2015
2020
Year
Trendline
actual
smoothed
95%−Confidence Intervall
smoothed

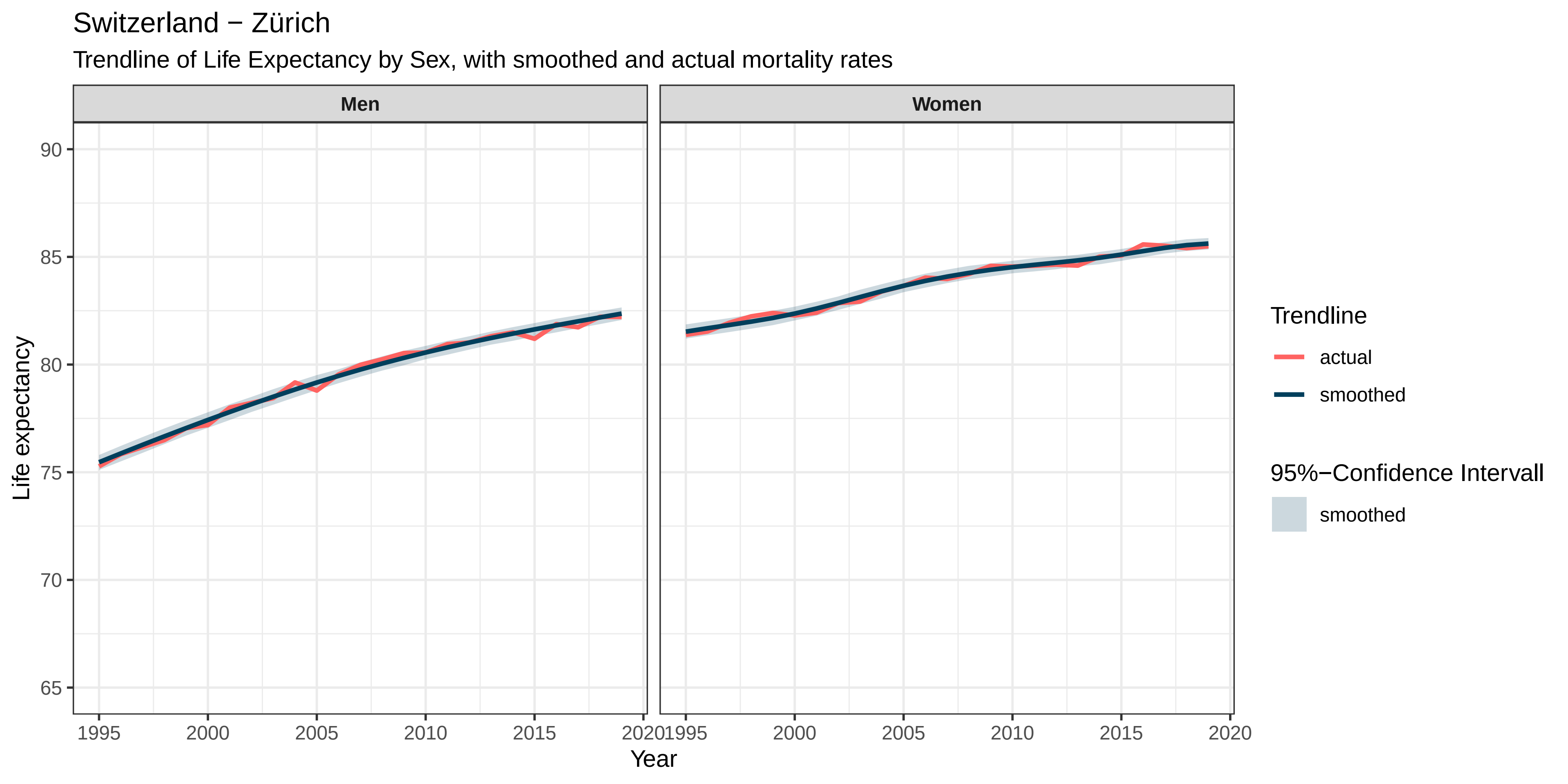

Switzerland − Zürich
Trendline of Life Expectancy by Sex, with smoothed and actual mortality rates
Men
Women
Life expectancy
90
85
80
75
70
65
1995
2000
2005
2010
2015
2020
Year
Trendline
actual
smoothed
95%−Confidence Intervall
smoothed

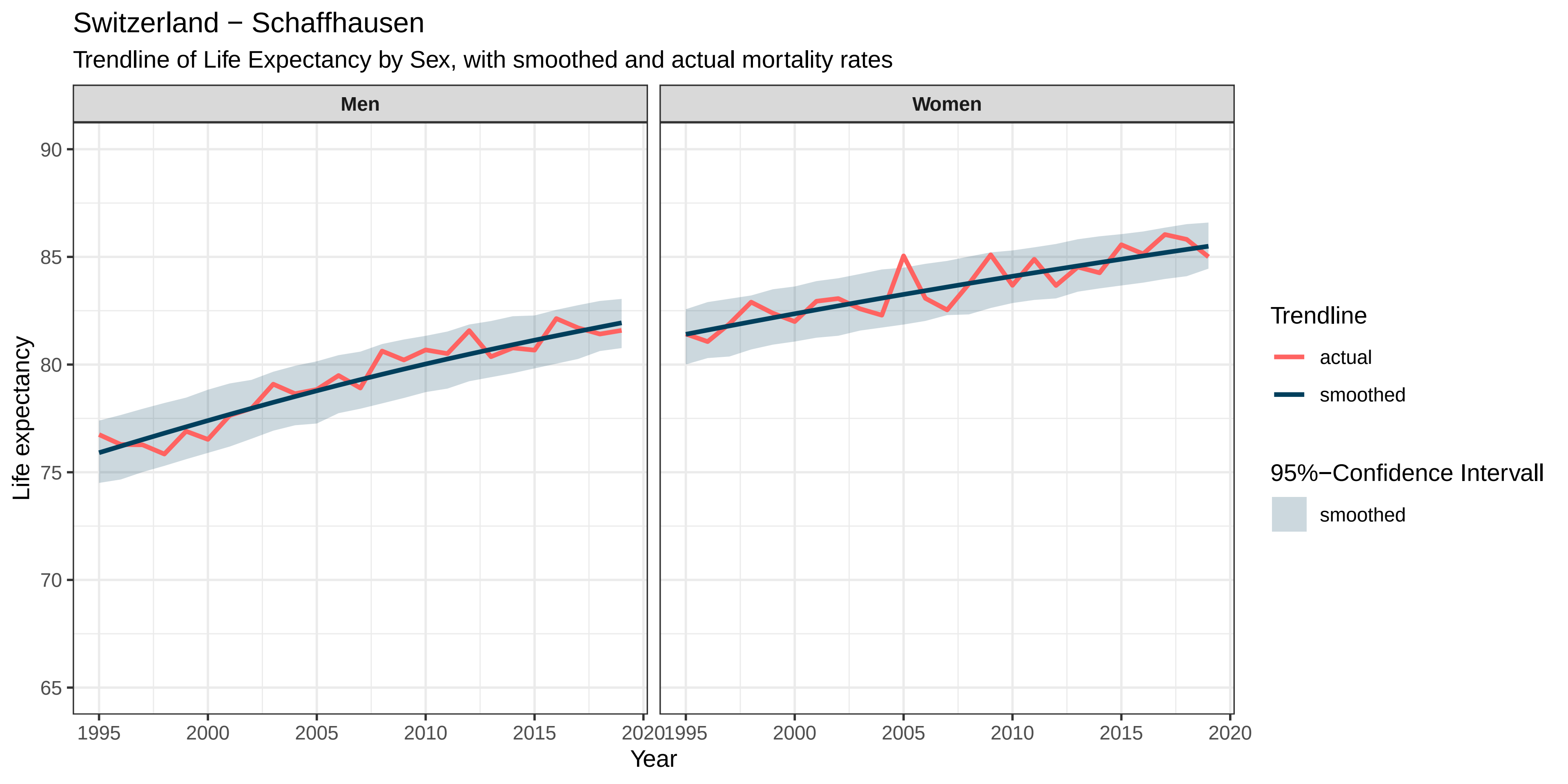

Switzerland − Schaffhausen
Trendline of Life Expectancy by Sex, with smoothed and actual mortality rates
Men
Women
Life expectancy
90
85
80
75
70
65
1995
2000
2005
2010
2015
2020
Year
Trendline
actual
smoothed
95%−Confidence Intervall
smoothed

Switzerland − Appenzell Ausserrhoden

Trendline of Life Expectancy by Sex, with smoothed and actual mortality rates

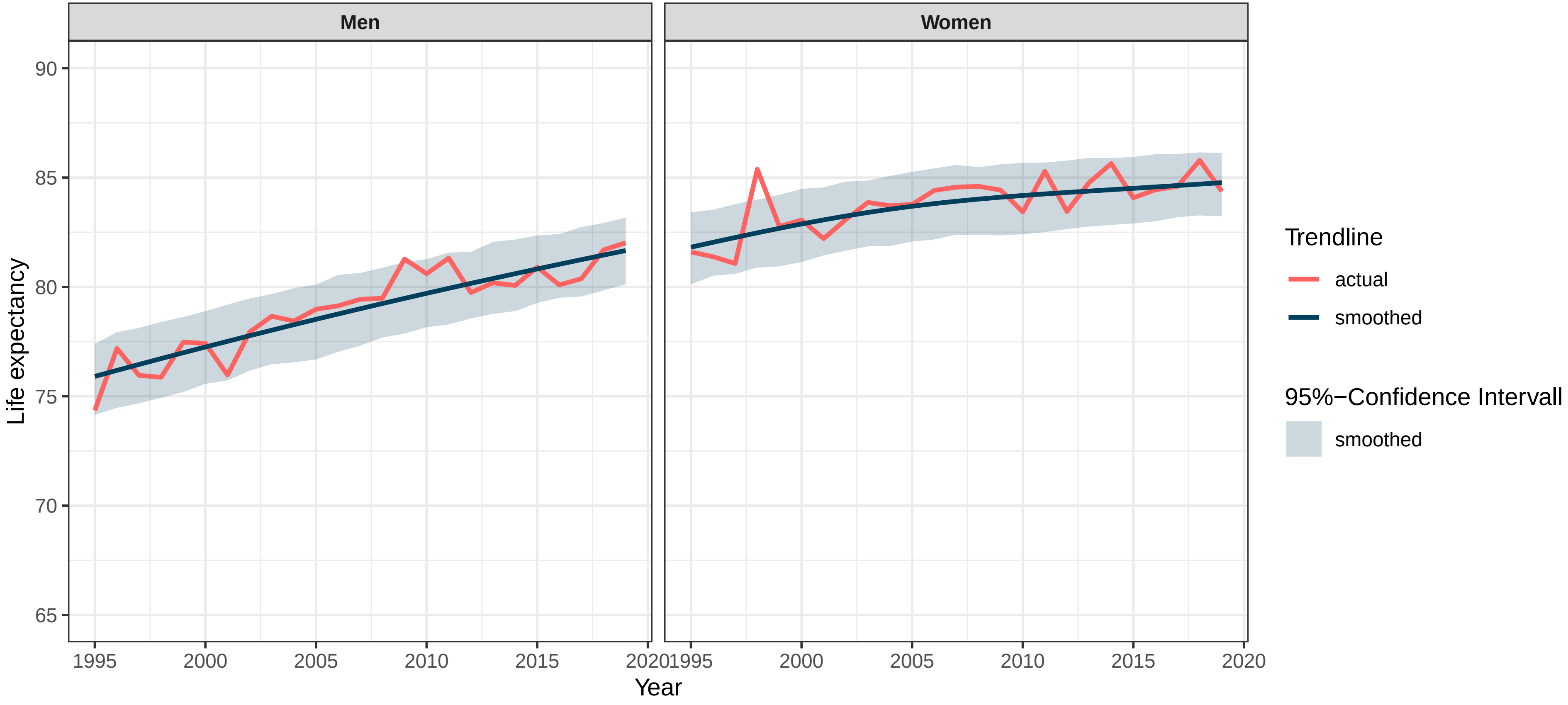

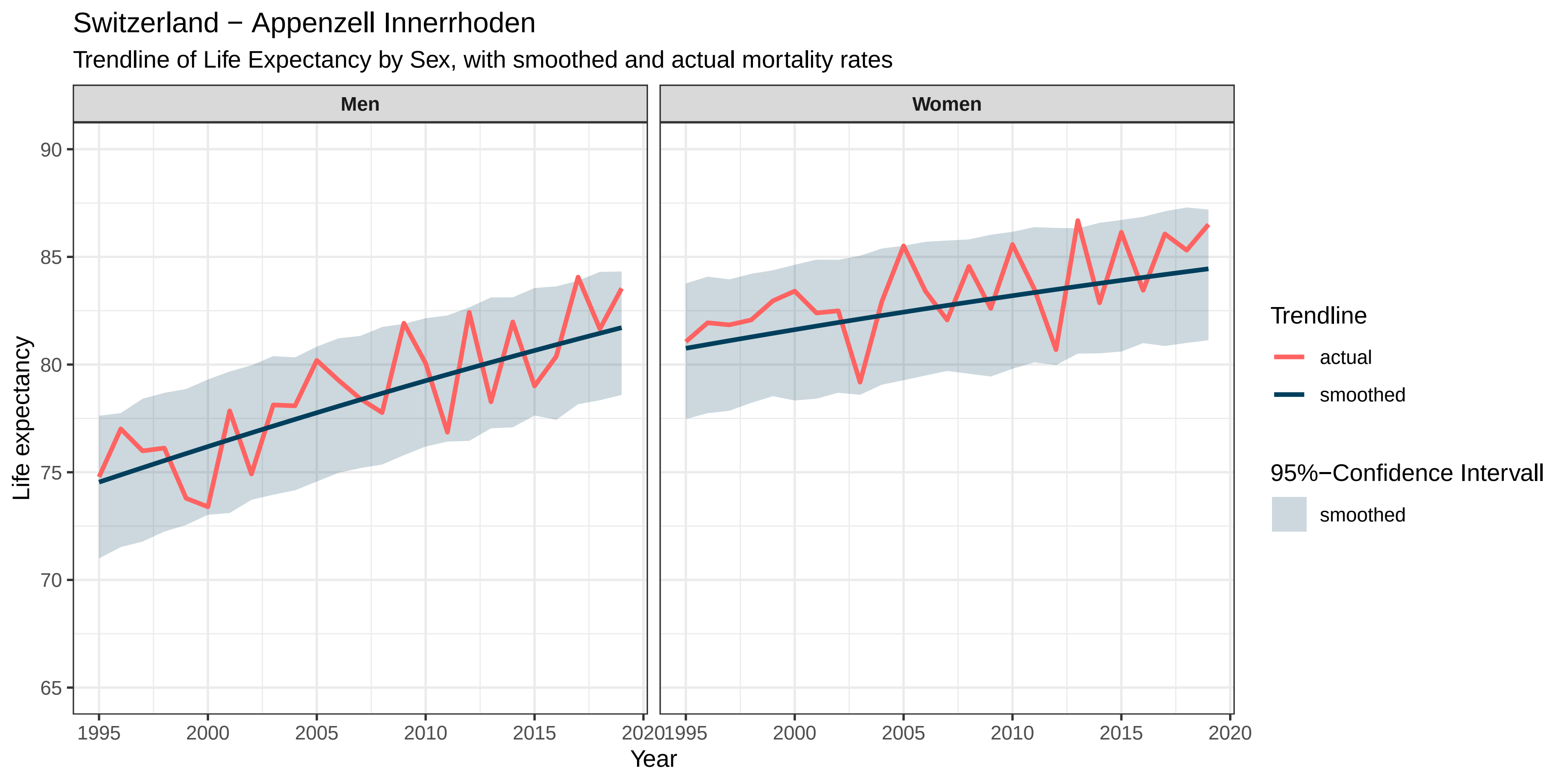

Switzerland − Appenzell Innerrhoden
Trendline of Life Expectancy by Sex, with smoothed and actual mortality rates
Men
Women
Life expectancy
90
85
80
75
70
65
1995
2000
2005
2010
2015
2020
Year
Trendline
actual
smoothed
95%−Confidence Intervall
smoothed

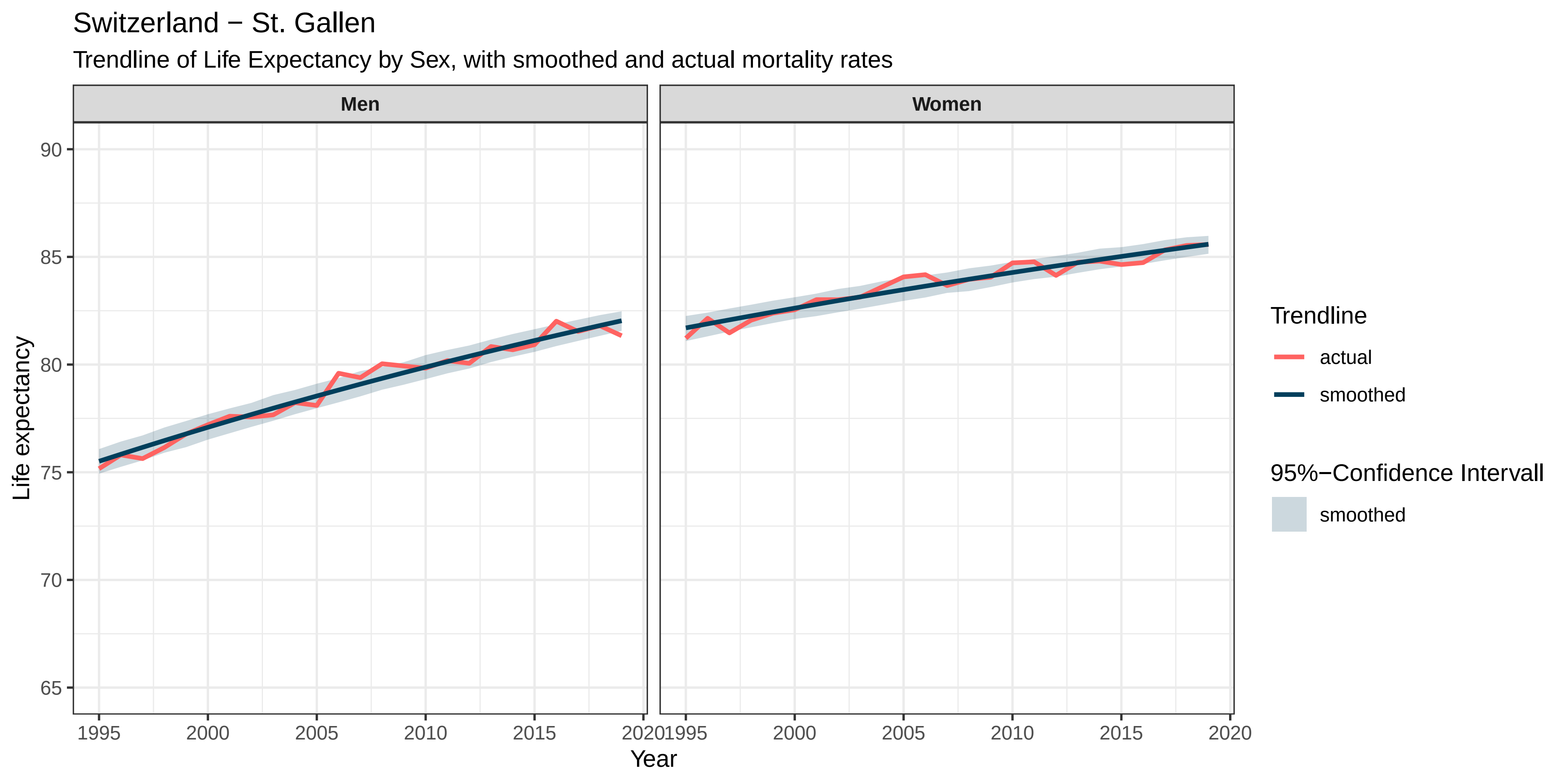

Switzerland − St. Gallen
Trendline of Life Expectancy by Sex, with smoothed and actual mortality rates
Men
Women
90
85
80
75
70
65
Life expectancy
1995
2000
2005
2010
2015
2020
Year
Trendline
actual
smoothed
95%−Confidence Intervall
smoothed

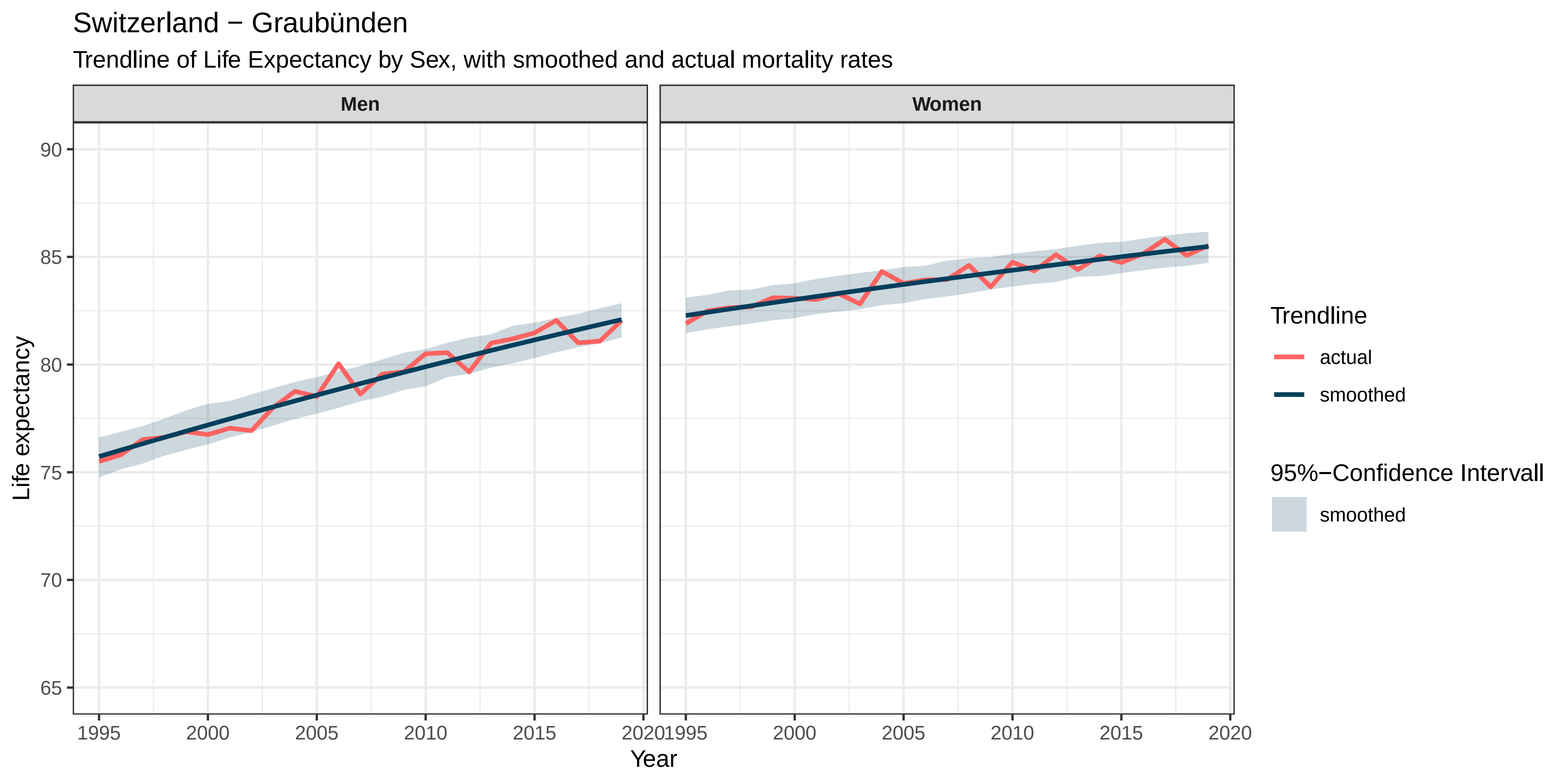
Switzerland − Graubünden
Trendline of Life Expectancy by Sex, with smoothed and actual mortality rates
Men
Women
90
85
80
75
70
65
Life expectancy
1995
2000
2005
2010
2015
2020
Year
Trendline
actual
smoothed
95%−Confidence Intervall
smoothed

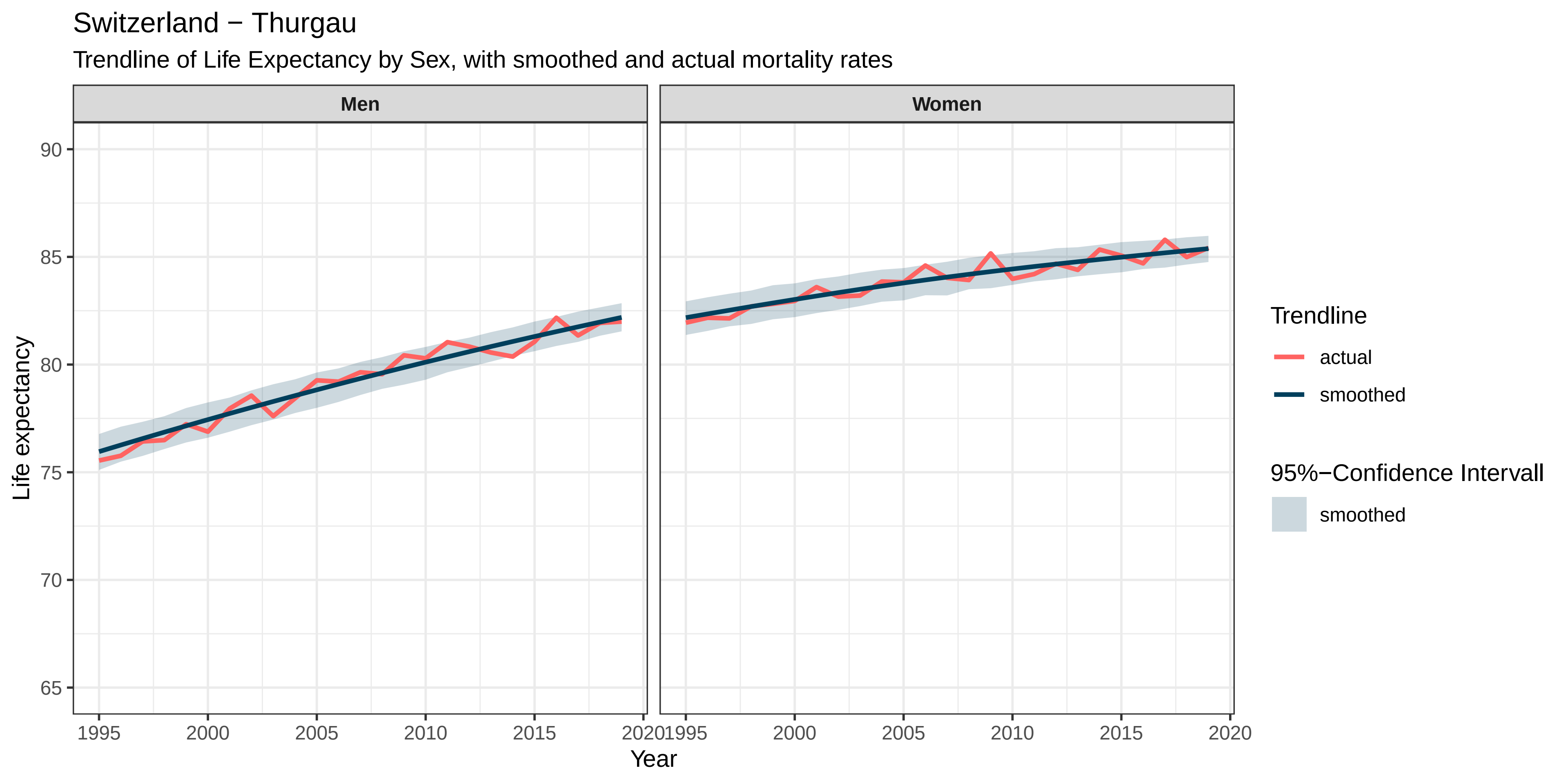

Switzerland − Thurgau
Trendline of Life Expectancy by Sex, with smoothed and actual mortality rates
Men
Women
Life expectancy
90
85
80
75
70
65
1995
2000
2005
2010
2015
2020
Year
Trendline
actual
smoothed
95%−Confidence Intervall
smoothed

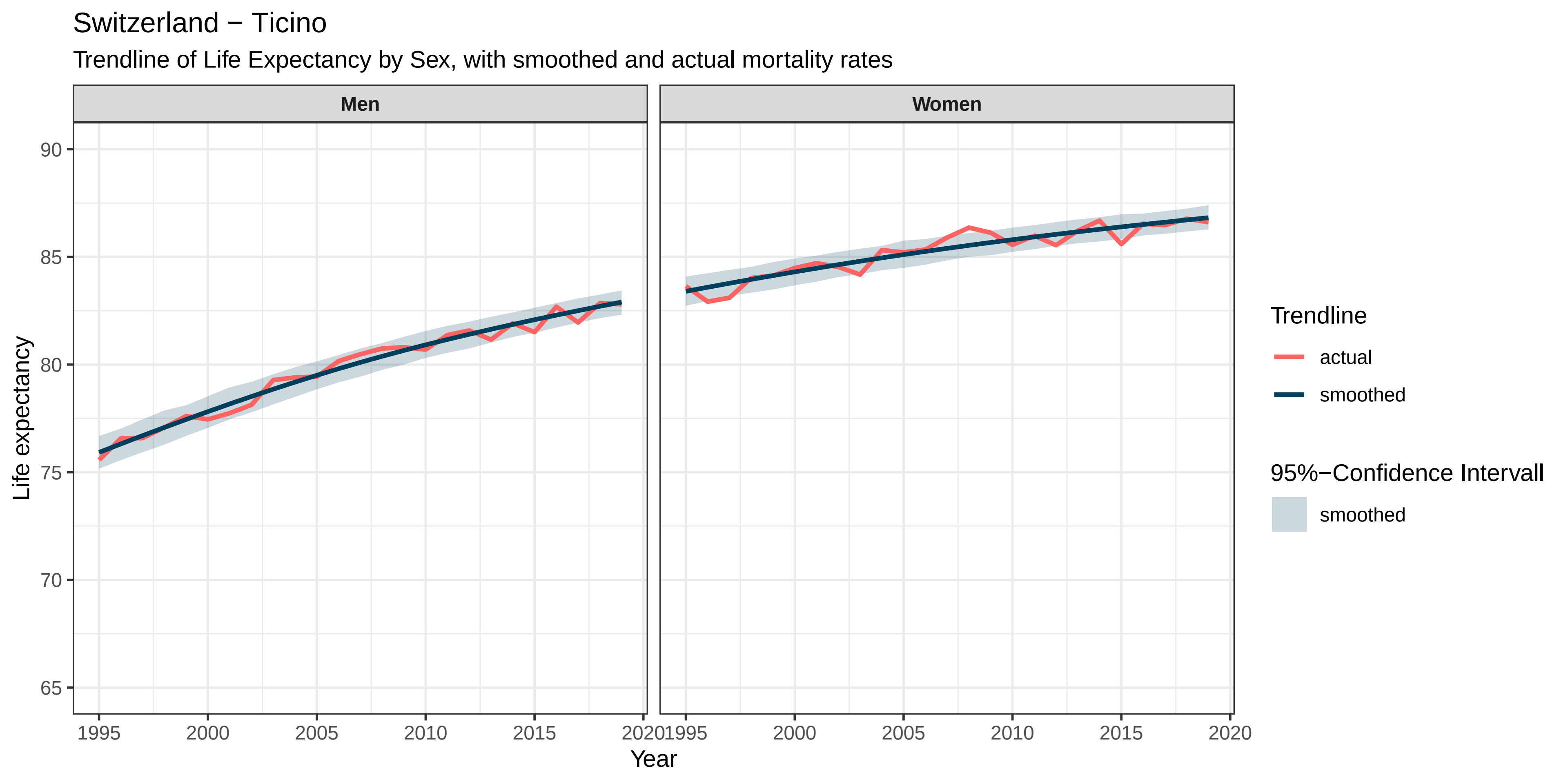

Switzerland − Ticino
Trendline of Life Expectancy by Sex, with smoothed and actual mortality rates
Men
Women
Life expectancy
90
85
80
75
70
65
1995
2000
2005
2010
2015
2020
Year
Trendline
actual
smoothed
95%−Confidence Intervall
smoothed

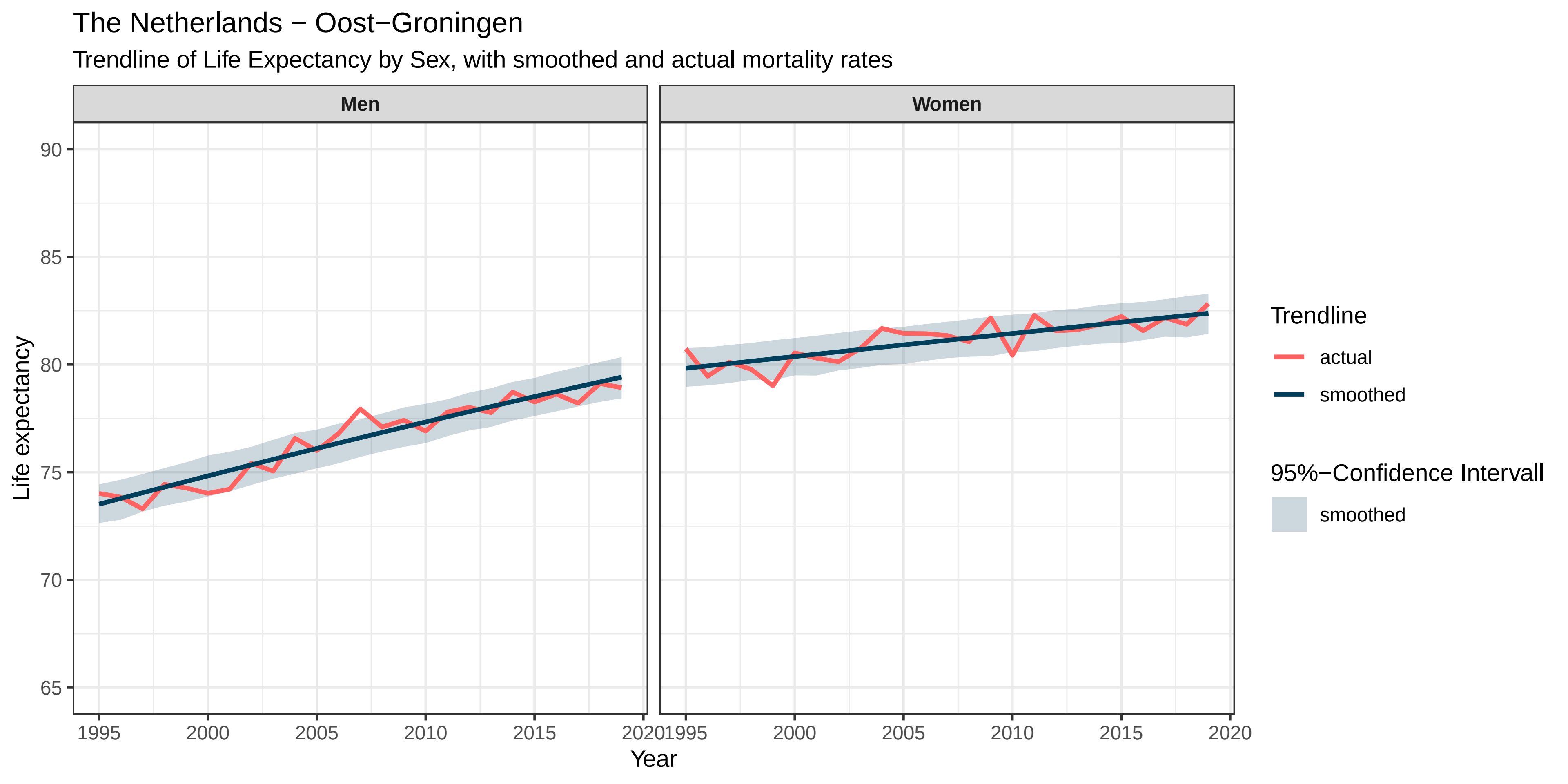

The Netherlands − Oost−Groningen
Trendline of Life Expectancy by Sex, with smoothed and actual mortality rates
Men
Women
90
85
80
75
70
65
Life expectancy
1995
2000
2005
2010
2015
2020
Year
Trendline
actual
smoothed
95%−Confidence Intervall
smoothed

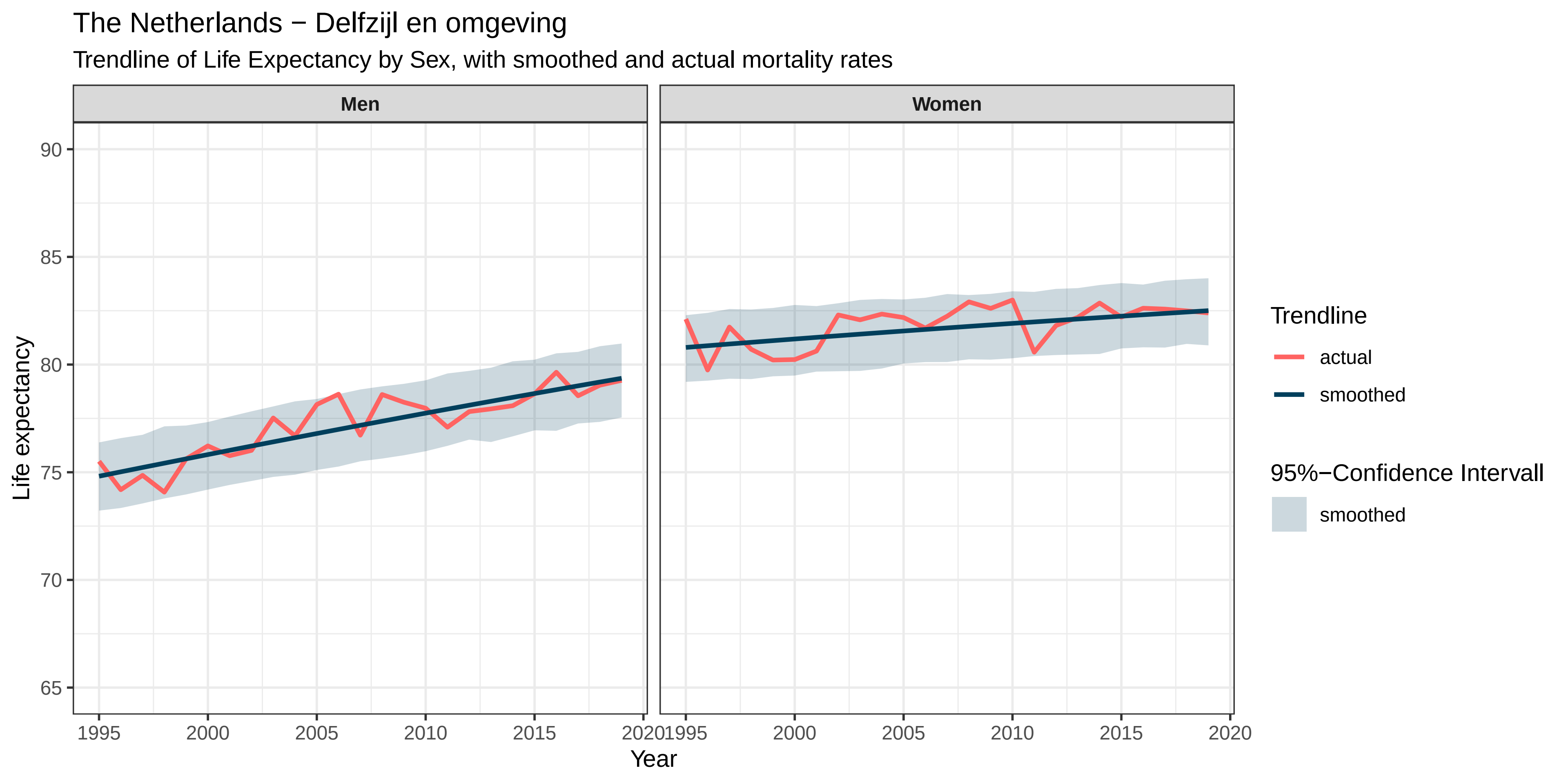
The Netherlands − Delfzijl en omgeving
Trendline of Life Expectancy by Sex, with smoothed and actual mortality rates
Men
Women
Life expectancy
90
85
80
75
70
65
1995
2000
2005
2010
2015
2020
Year
Trendline
actual
smoothed
95%−Confidence Intervall
smoothed

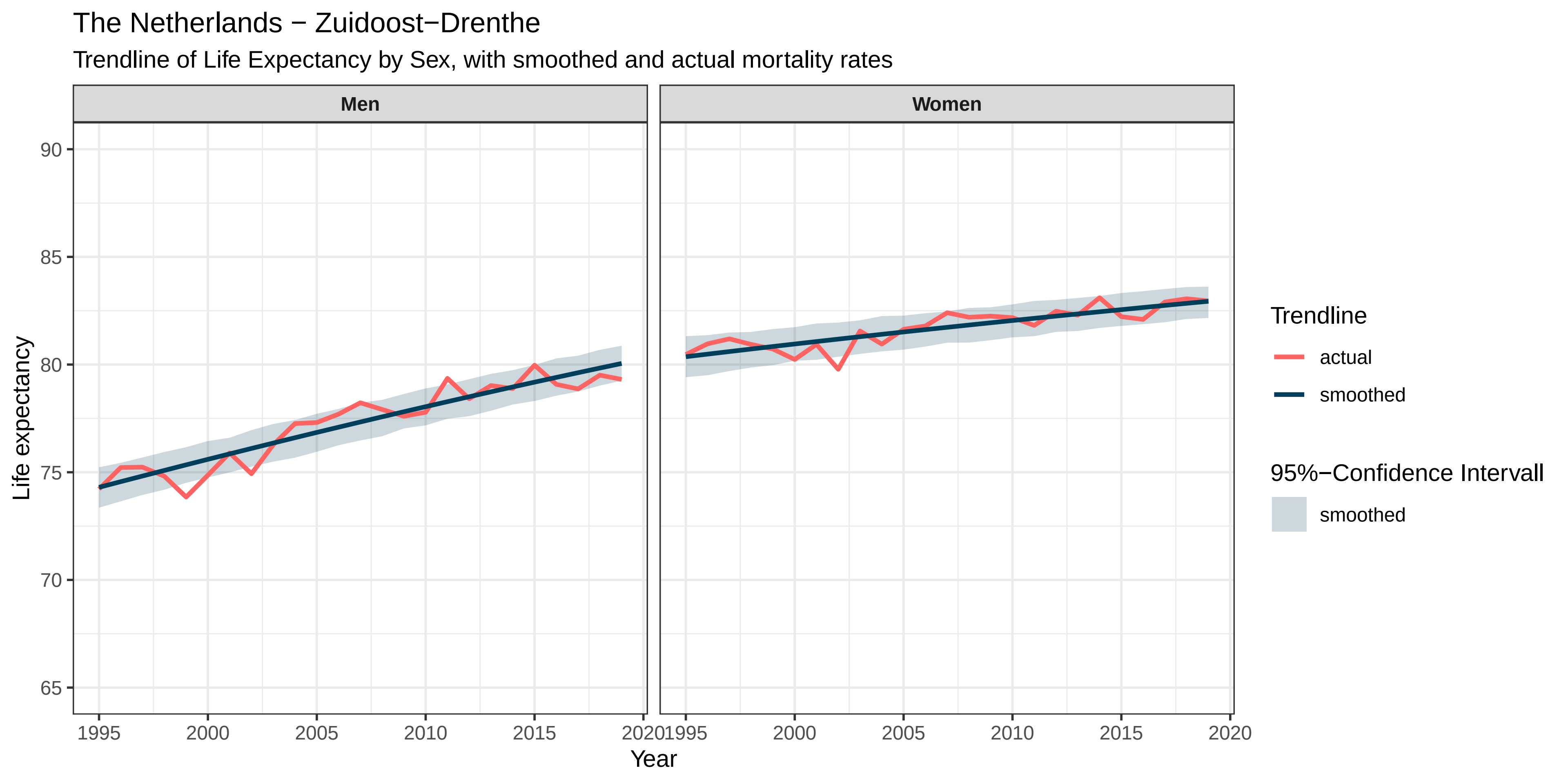

The Netherlands − Zuidoost−Drenthe
Trendline of Life Expectancy by Sex, with smoothed and actual mortality rates
Men
Women
Life expectancy
90
85
80
75
70
65
1995
2000
2005
2010
2015
2020
Year
Trendline
actual
smoothed
95%−Confidence Intervall
smoothed

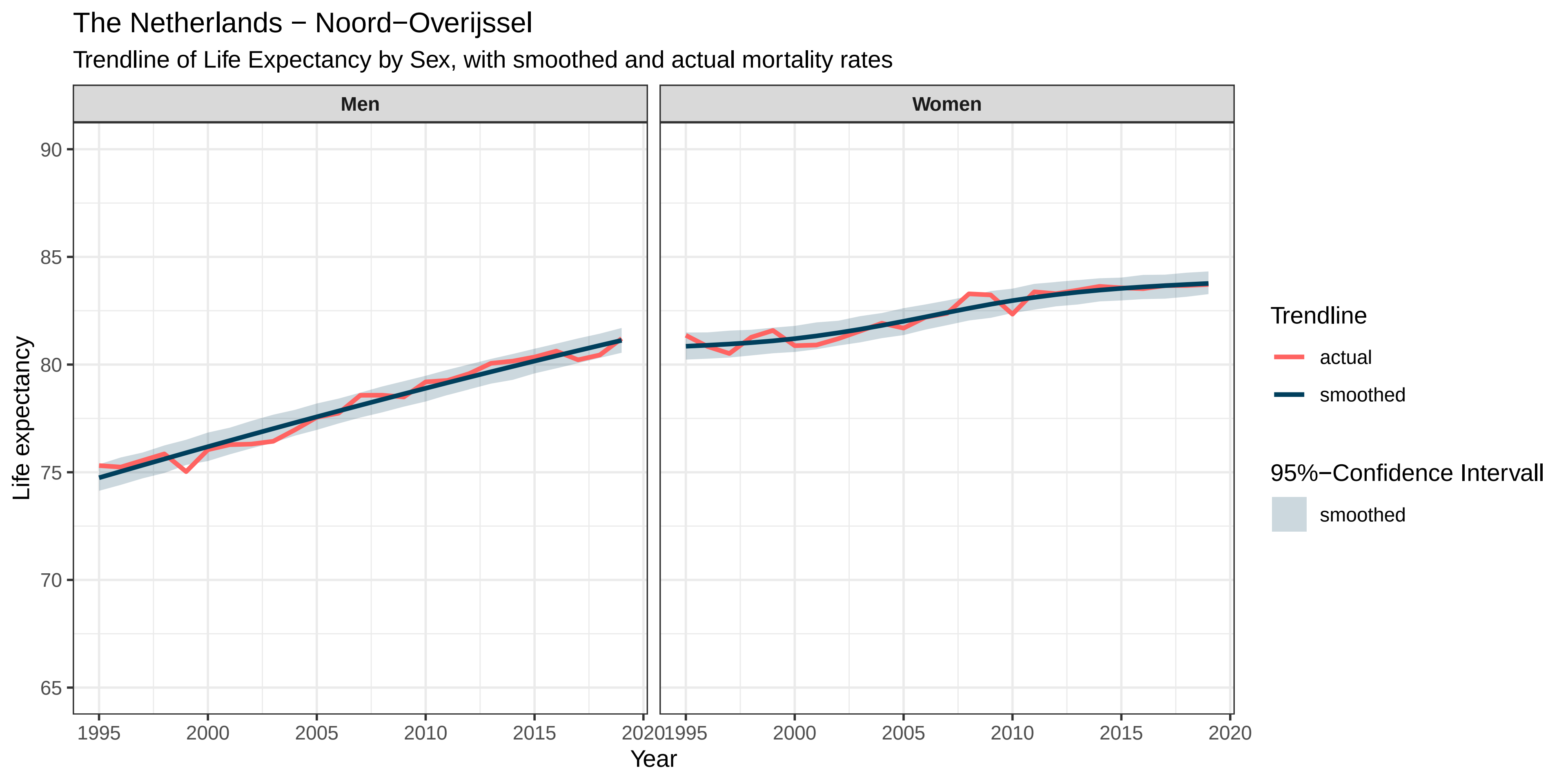
The Netherlands − Noord−Overijssel
Trendline of Life Expectancy by Sex, with smoothed and actual mortality rates
Men
Women
Life expectancy
90
85
80
75
70
65
1995
2000
2005
2010
2015
2020
Year
Trendline
actual
smoothed
95%−Confidence Intervall
smoothed

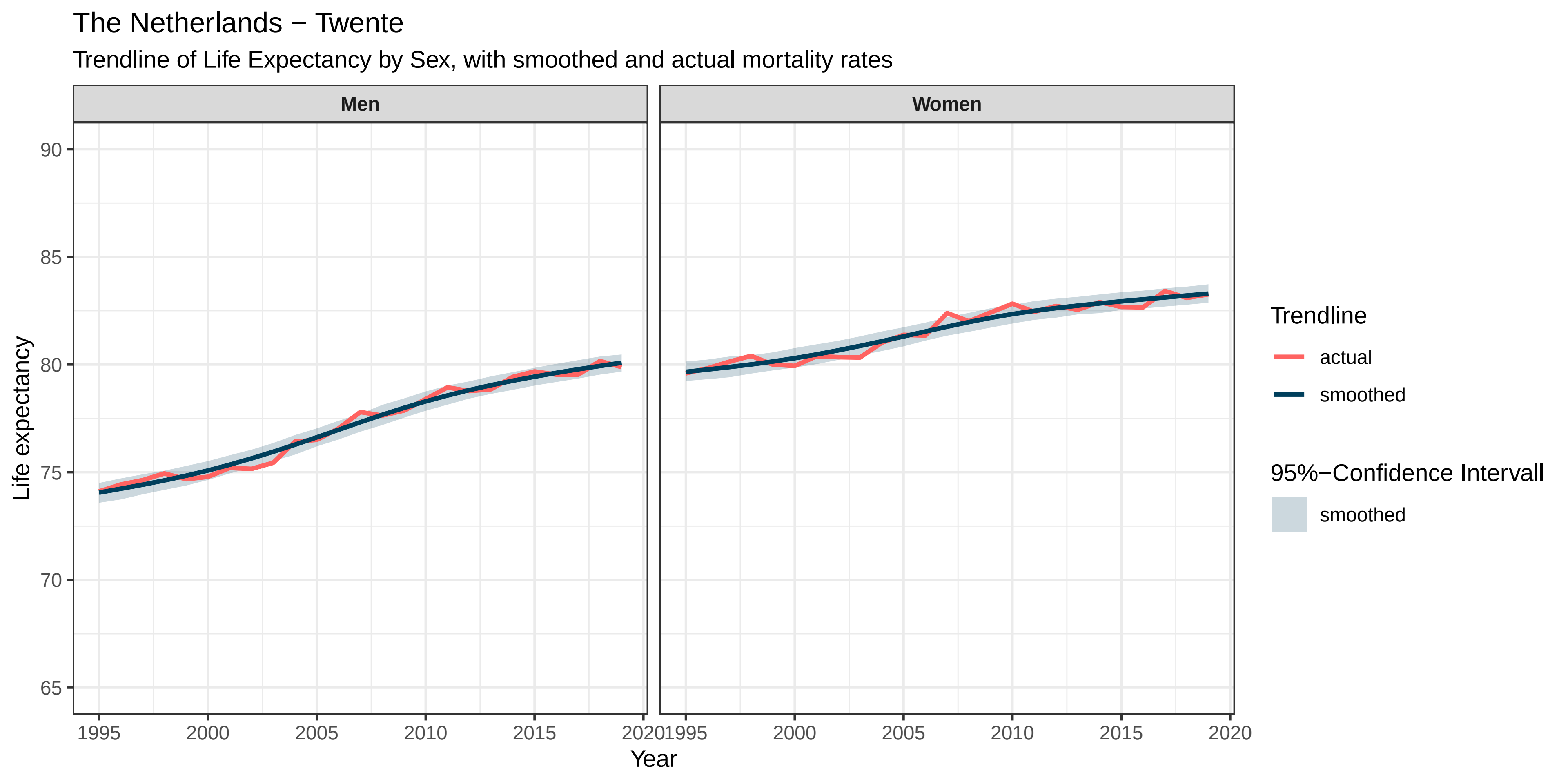
The Netherlands − Twente
Trendline of Life Expectancy by Sex, with smoothed and actual mortality rates
Men
Women
Life expectancy
90
85
80
75
70
65
1995
2000
2005
2010
2015
2020
Year
Trendline
actual
smoothed
95%−Confidence Intervall
smoothed

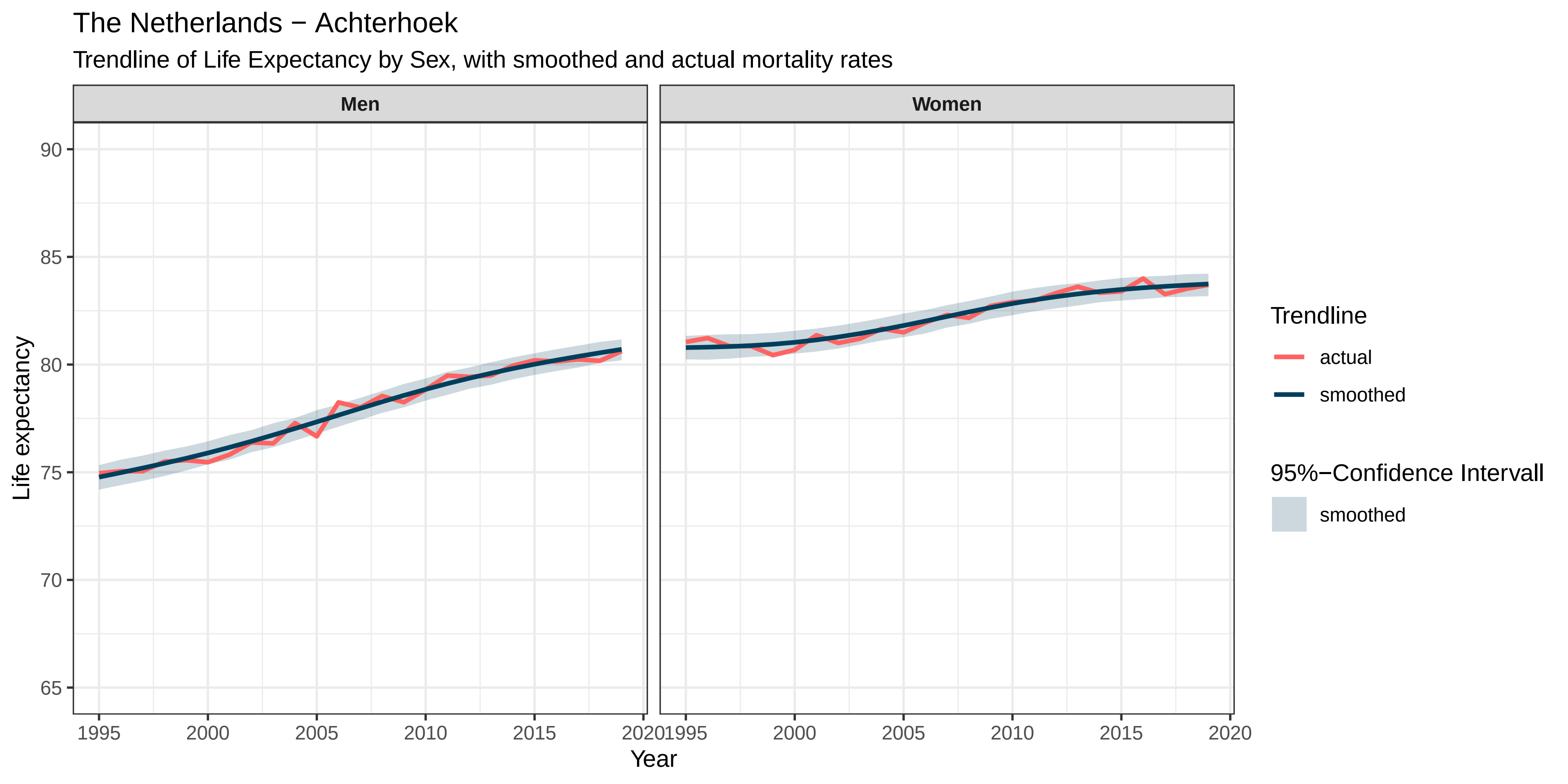

The Netherlands − Achterhoek
Trendline of Life Expectancy by Sex, with smoothed and actual mortality rates
Men
Women
90
85
80
75
70
65
Life expectancy
1995
2000
2005
2010
2015
2020
Year
Trendline
actual
smoothed
95%−Confidence Intervall
smoothed

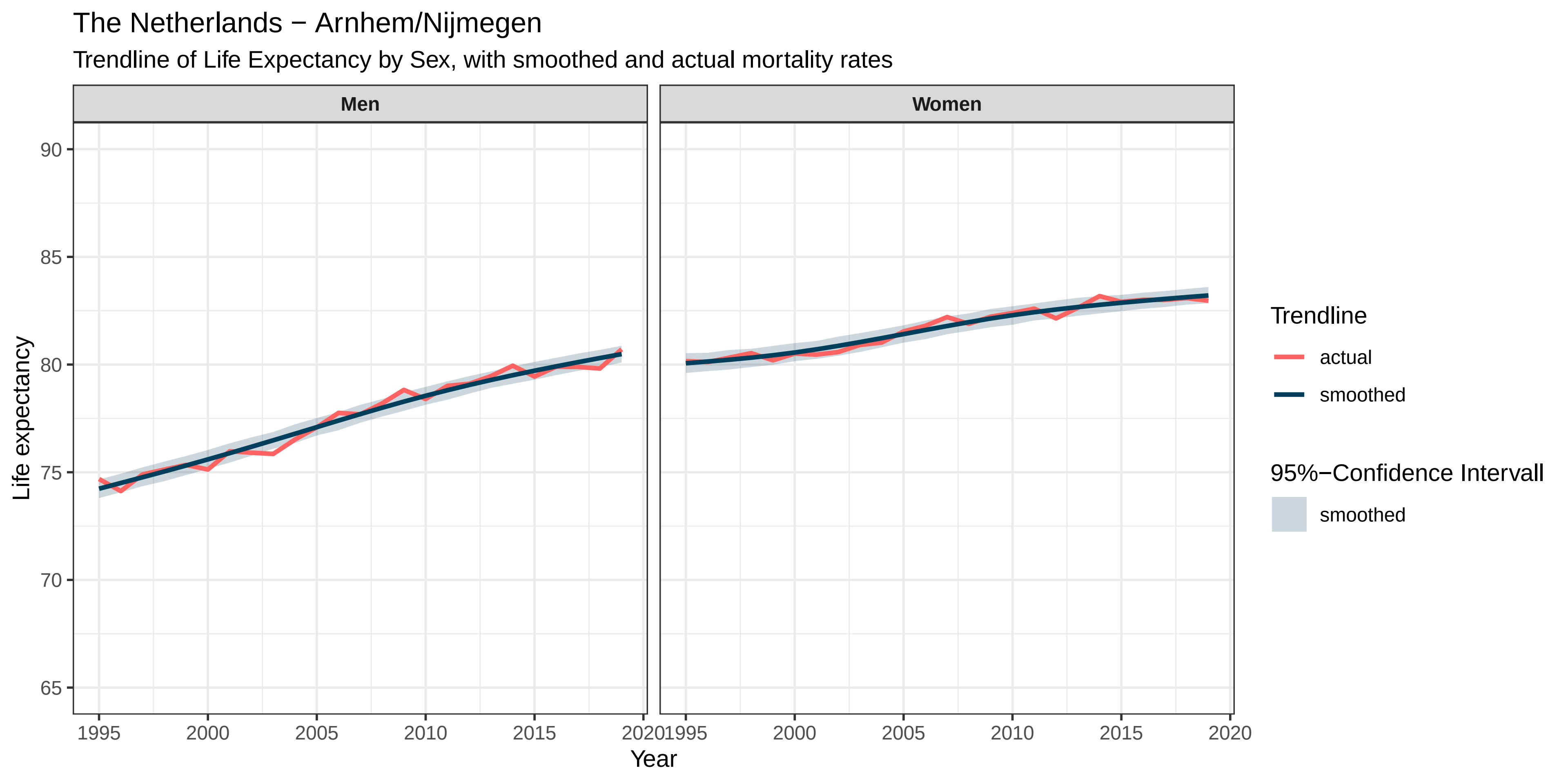

The Netherlands − Arnhem/Nijmegen
Trendline of Life Expectancy by Sex, with smoothed and actual mortality rates
Men
Women
Life expectancy
Year
90
85
80
75
70
65
1995
2000
2005
2010
2015
2020
Trendline
actual
smoothed
95%−Confidence Intervall
smoothed

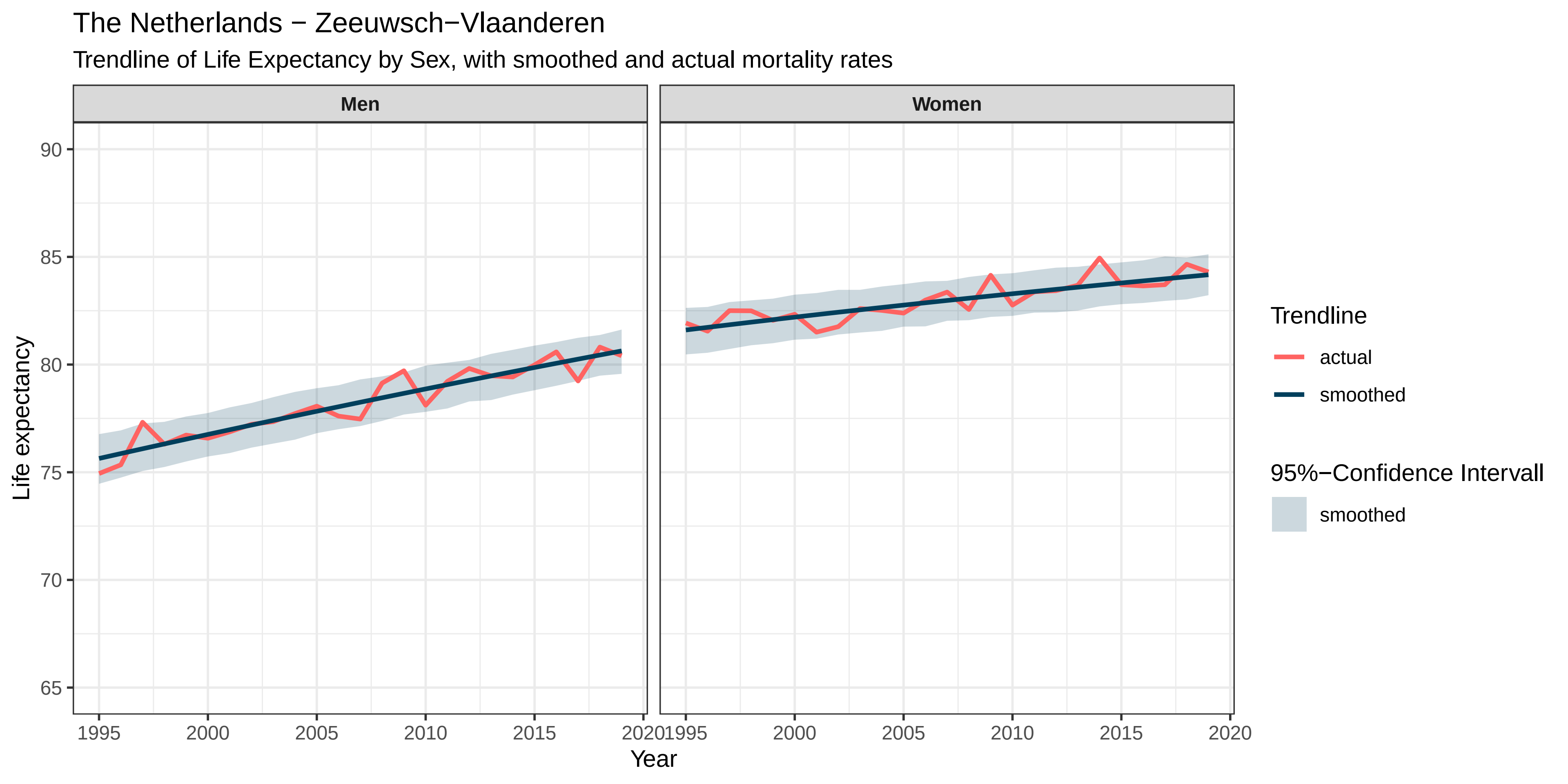
The Netherlands − Zeeuwsch−Vlaanderen
Trendline of Life Expectancy by Sex, with smoothed and actual mortality rates
Men
Women
90
85
80
75
70
65
Life expectancy
1995
2000
2005
2010
2015
2020
Year
Trendline
actual
smoothed
95%−Confidence Intervall
smoothed

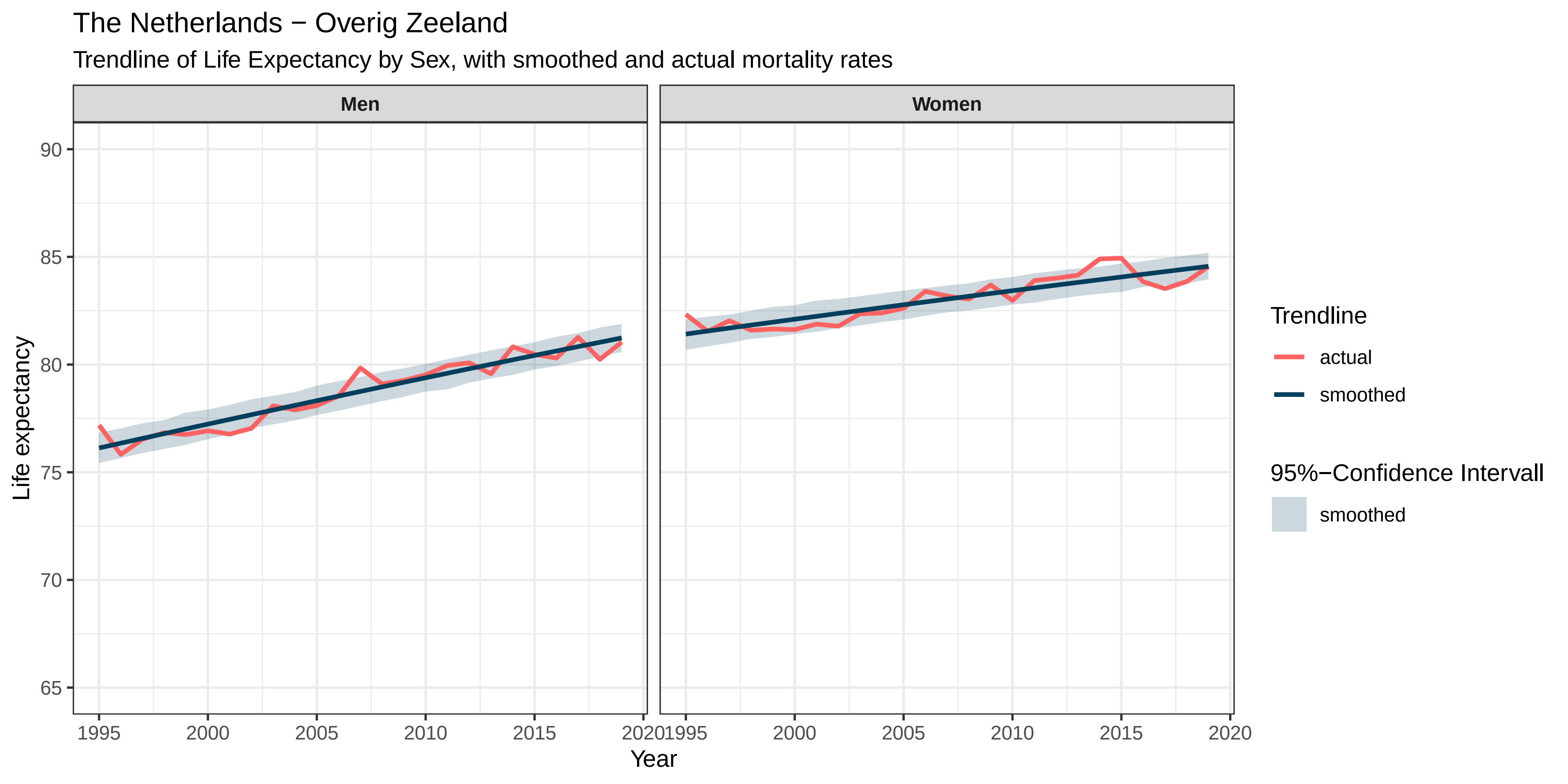
The Netherlands − Overig Zeeland
Trendline of Life Expectancy by Sex, with smoothed and actual mortality rates
Men
Women
Life expectancy
90
85
80
75
70
65
1995
2000
2005
2010
2015
2020
Year
Trendline
actual
smoothed
95%−Confidence Intervall
smoothed

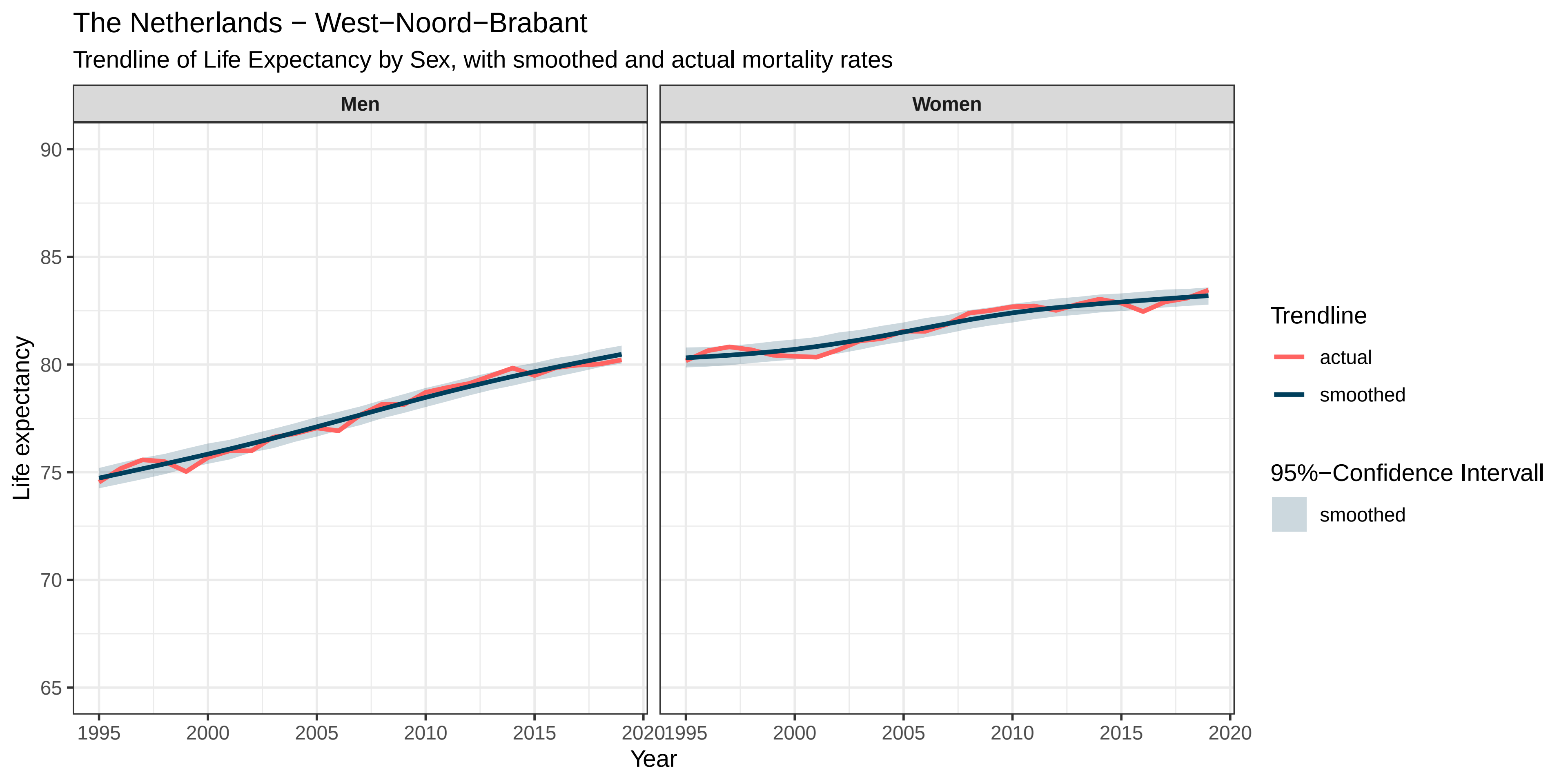

The Netherlands − West−Noord−Brabant
Trendline of Life Expectancy by Sex, with smoothed and actual mortality rates
Men
Women
Life expectancy
90
85
80
75
70
65
1995
2000
2005
2010
2015
2020
Year
Trendline
actual
smoothed
95%−Confidence Intervall
smoothed

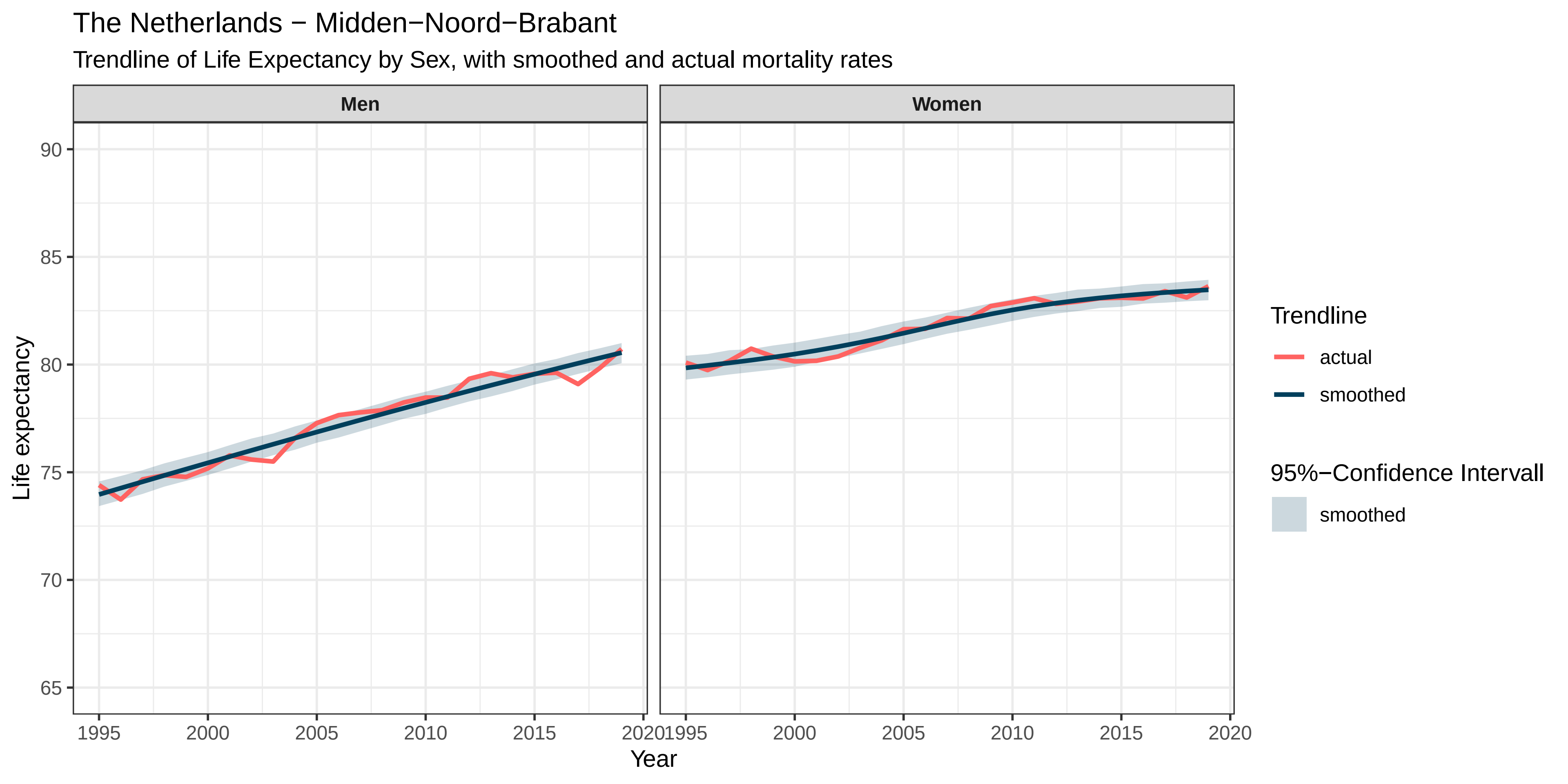

The Netherlands − Midden−Noord−Brabant
Trendline of Life Expectancy by Sex, with smoothed and actual mortality rates
Men
Women
90
85
80
75
70
65
Life expectancy
1995
2000
2005
2010
2015
2020
Year
Trendline
actual
smoothed
95%−Confidence Intervall
smoothed

The Netherlands − Zuidoost−Noord−Brabant

Trendline of Life Expectancy by Sex, with smoothed and actual mortality rates

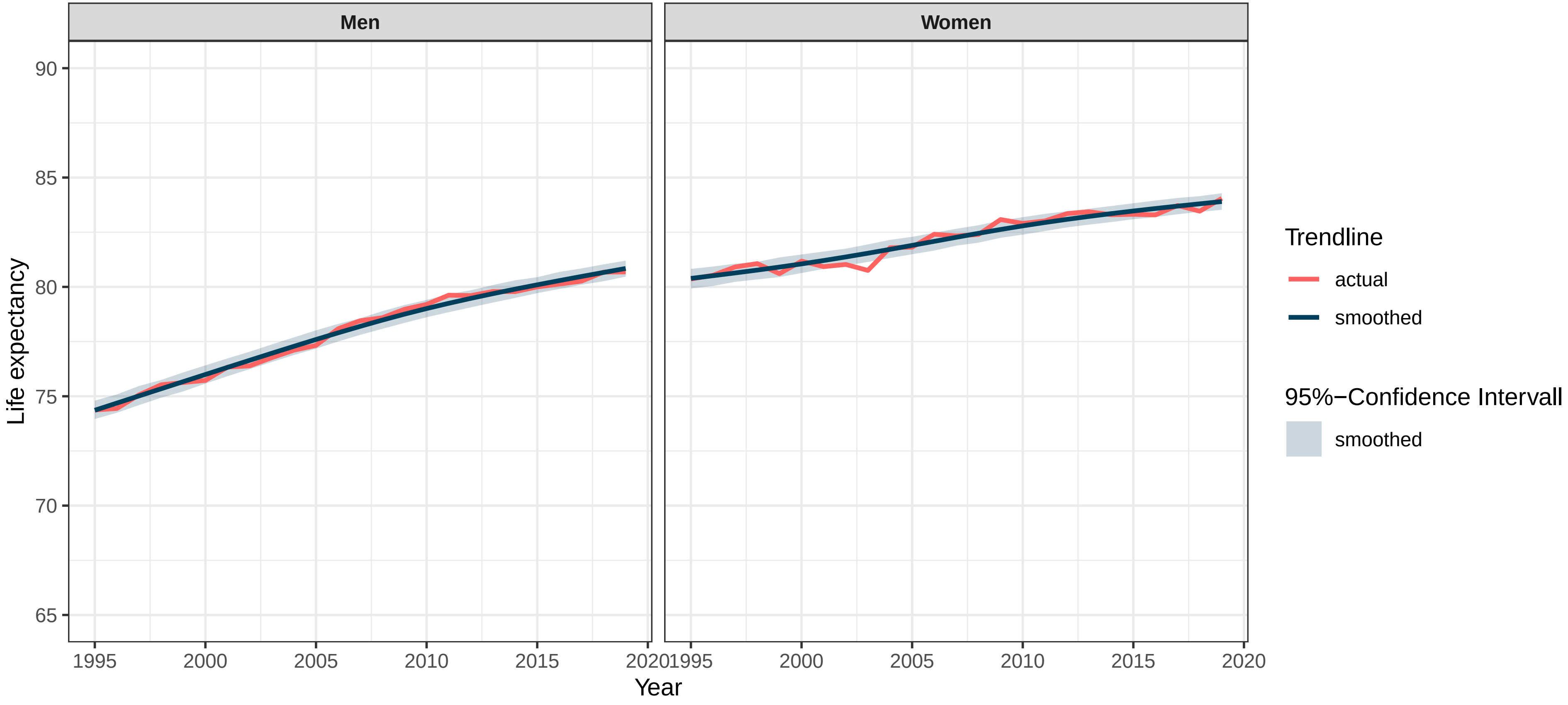

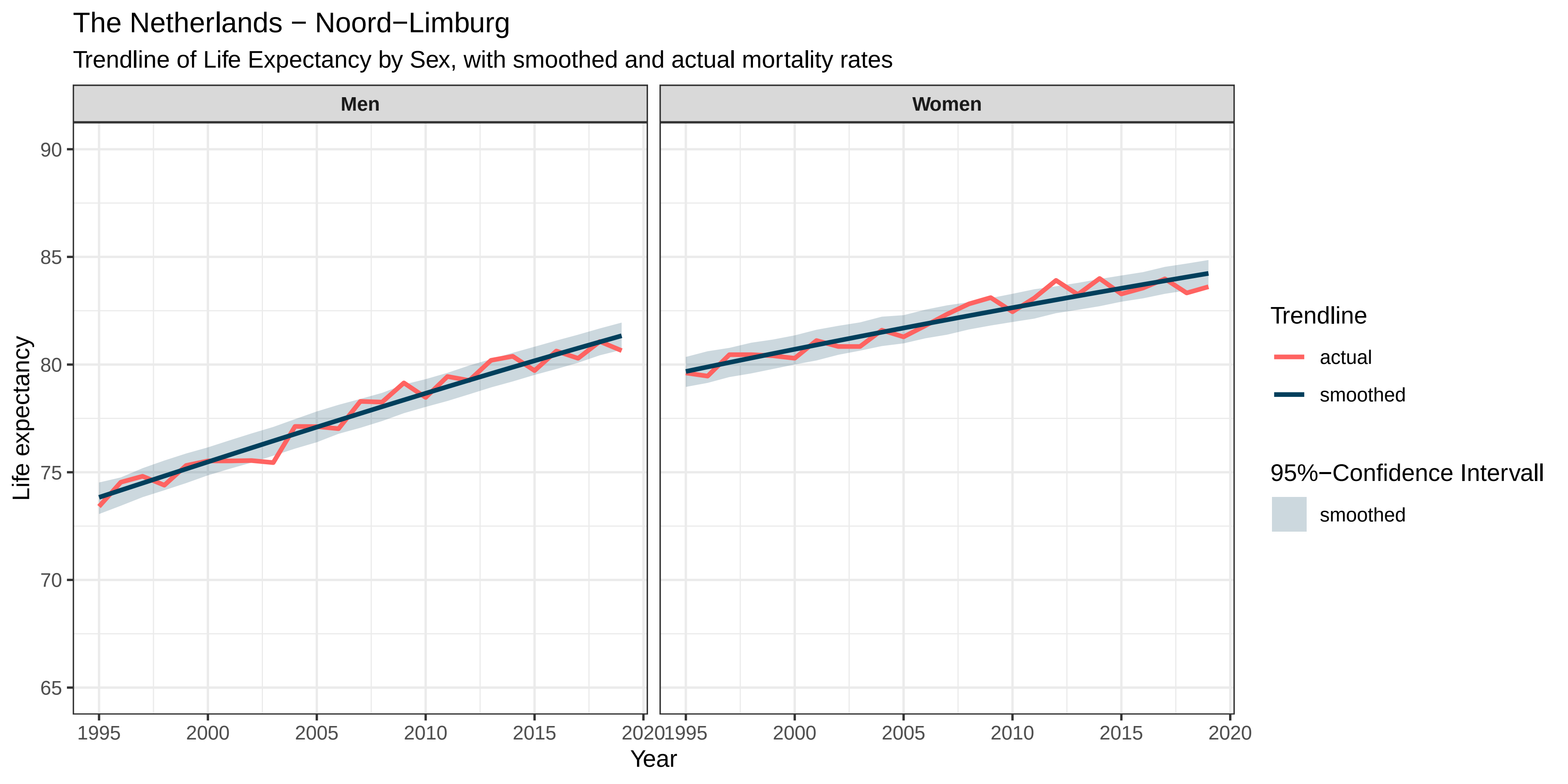
The Netherlands − Noord−Limburg
Trendline of Life Expectancy by Sex, with smoothed and actual mortality rates
Men
Women
90
85
80
75
70
65
Life expectancy
1995
2000
2005
2010
2015
2020
Year
Trendline
actual
smoothed
95%−Confidence Intervall
smoothed

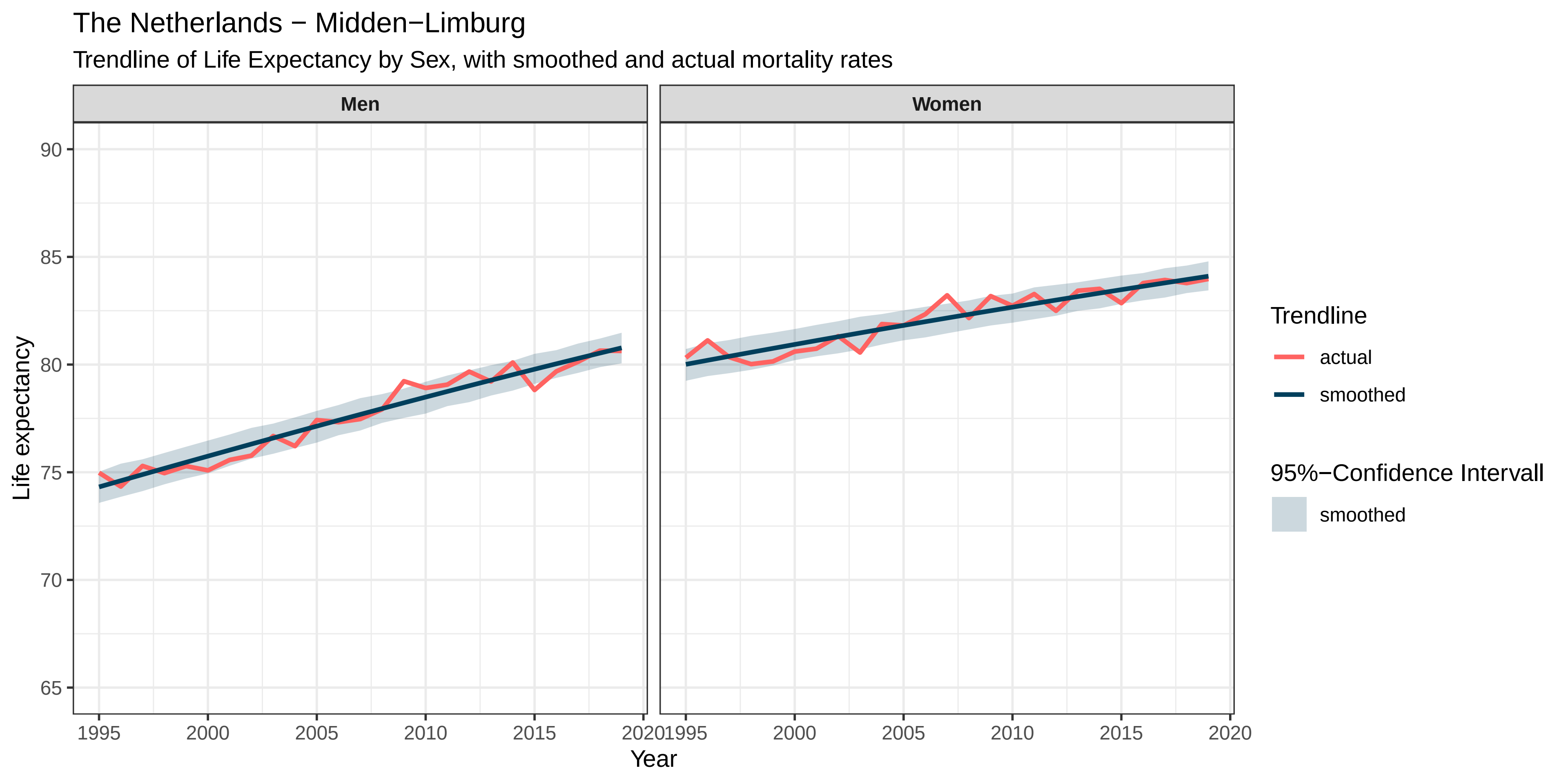
The Netherlands − Midden−Limburg
Trendline of Life Expectancy by Sex, with smoothed and actual mortality rates
Men
Women
Life expectancy
Year
90
85
80
75
70
65
1995
2000
2005
2010
2015
2020
Trendline
actual
smoothed
95%−Confidence Intervall
smoothed

The Netherlands − Zuid−Limburg

Trendline of Life Expectancy by Sex, with smoothed and actual mortality rates

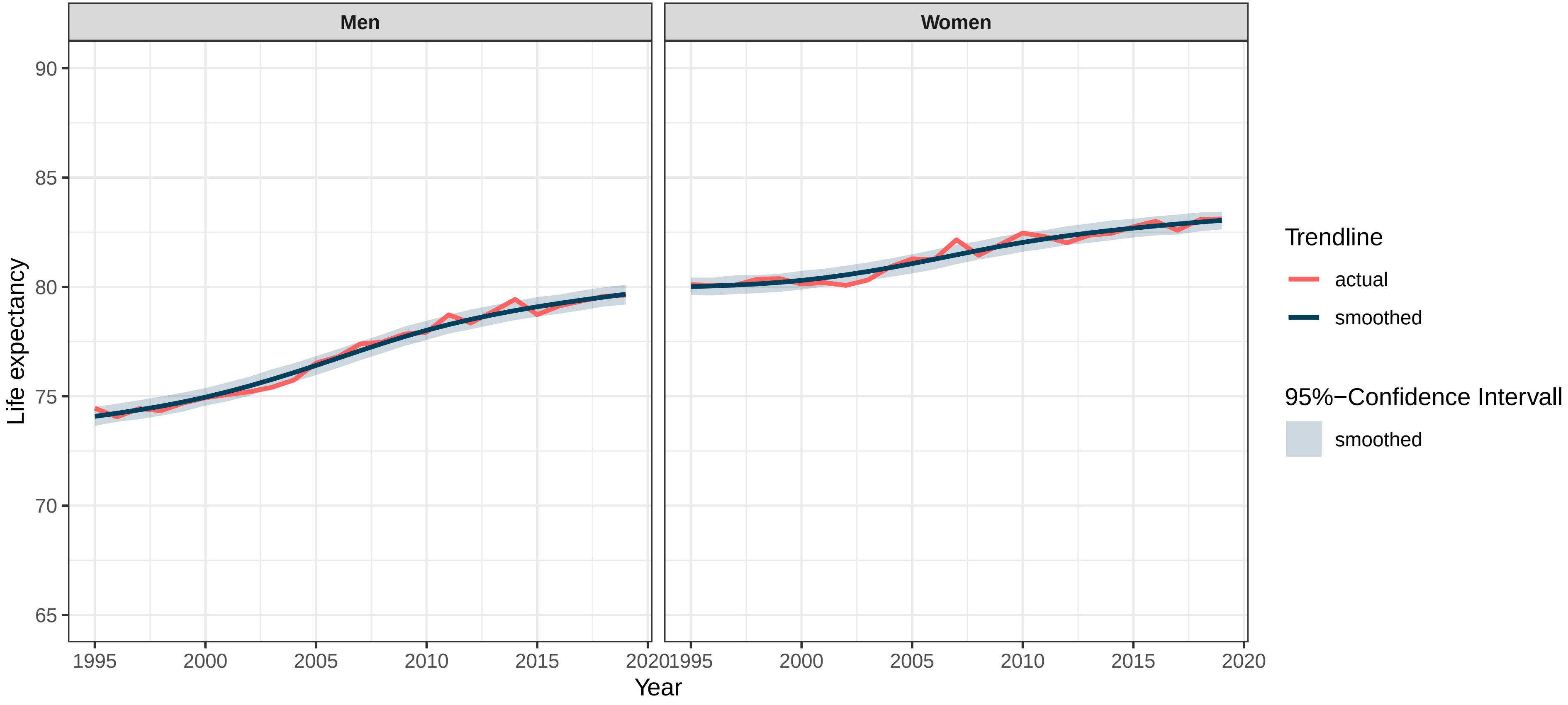

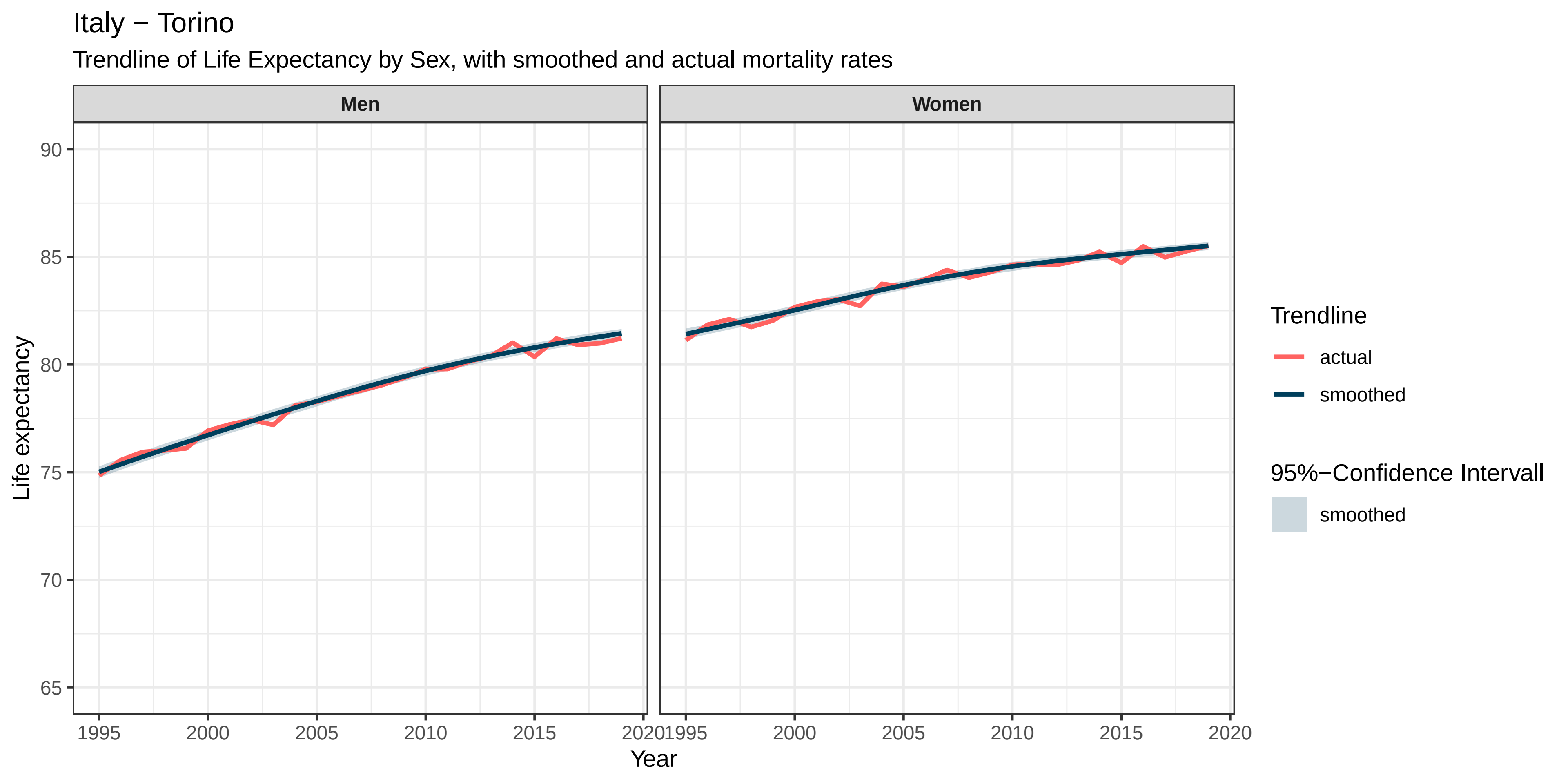

Italy − Torino
Trendline of Life Expectancy by Sex, with smoothed and actual mortality rates
Men
Women
90
85
80
75
70
65
Life expectancy
1995
2000
2005
2010
2015
2020
Year
Trendline
actual
smoothed
95%−Confidence Intervall
smoothed

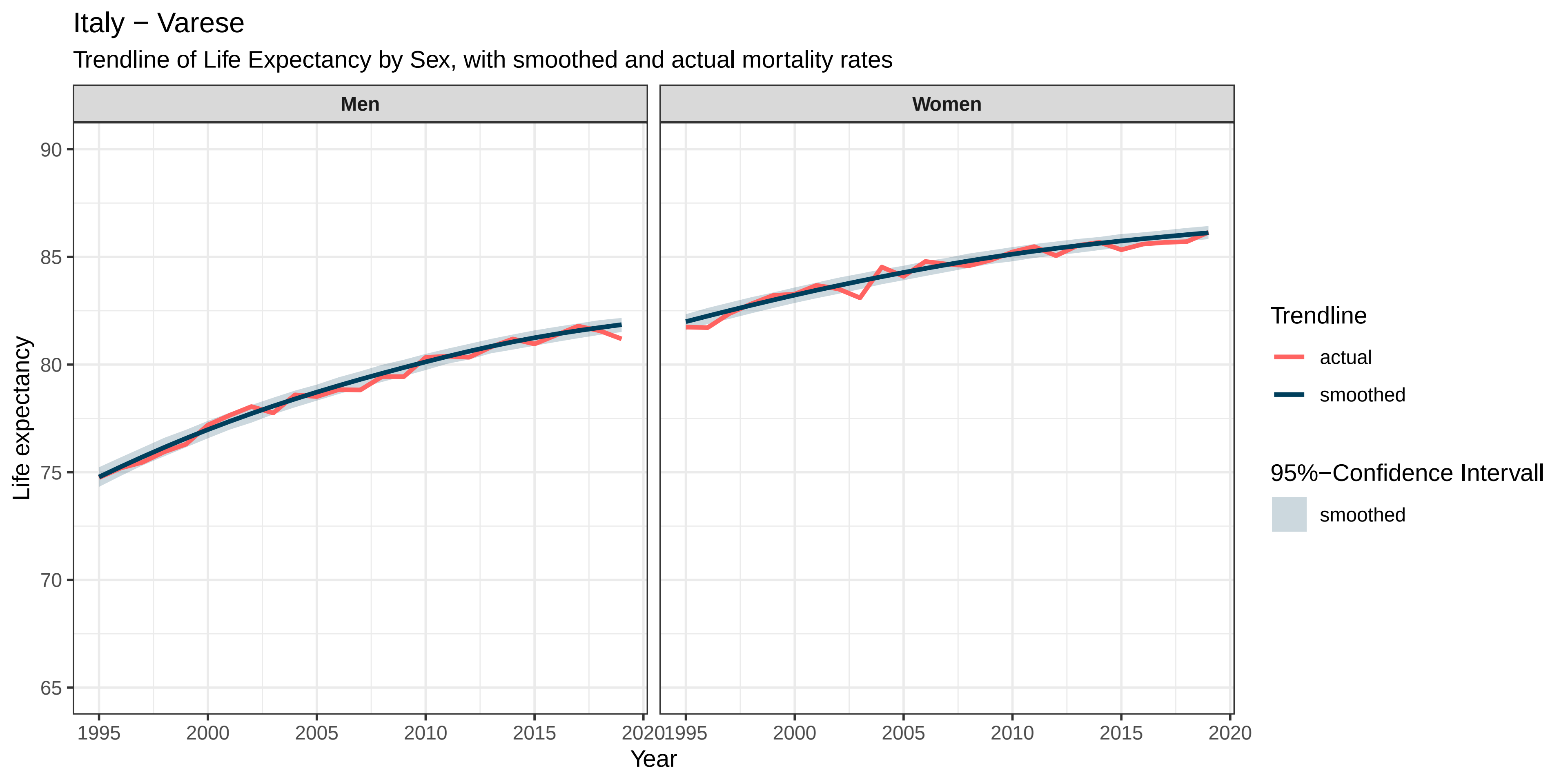

Italy − Varese
Trendline of Life Expectancy by Sex, with smoothed and actual mortality rates
Men
Women
Life expectancy
90
85
80
75
70
65
1995
2000
2005
2010
2015
2020
Year
Trendline
actual
smoothed
95%−Confidence Intervall
smoothed

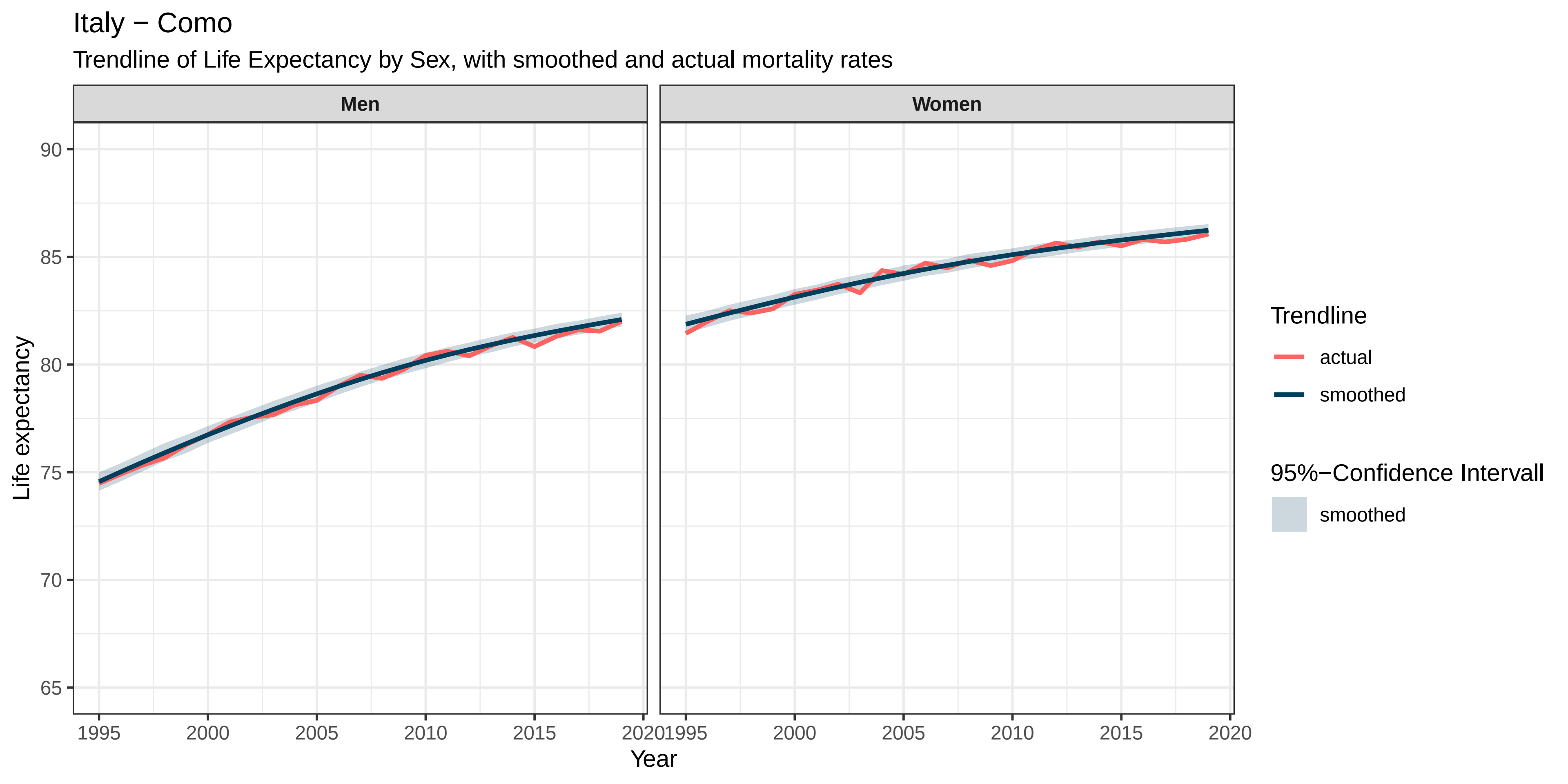

Italy − Como
Trendline of Life Expectancy by Sex, with smoothed and actual mortality rates
Men
Women
Life expectancy
90
85
80
75
70
65
1995
2000
2005
2010
2015
2020
Year
Trendline
actual
smoothed
95%−Confidence Intervall
smoothed

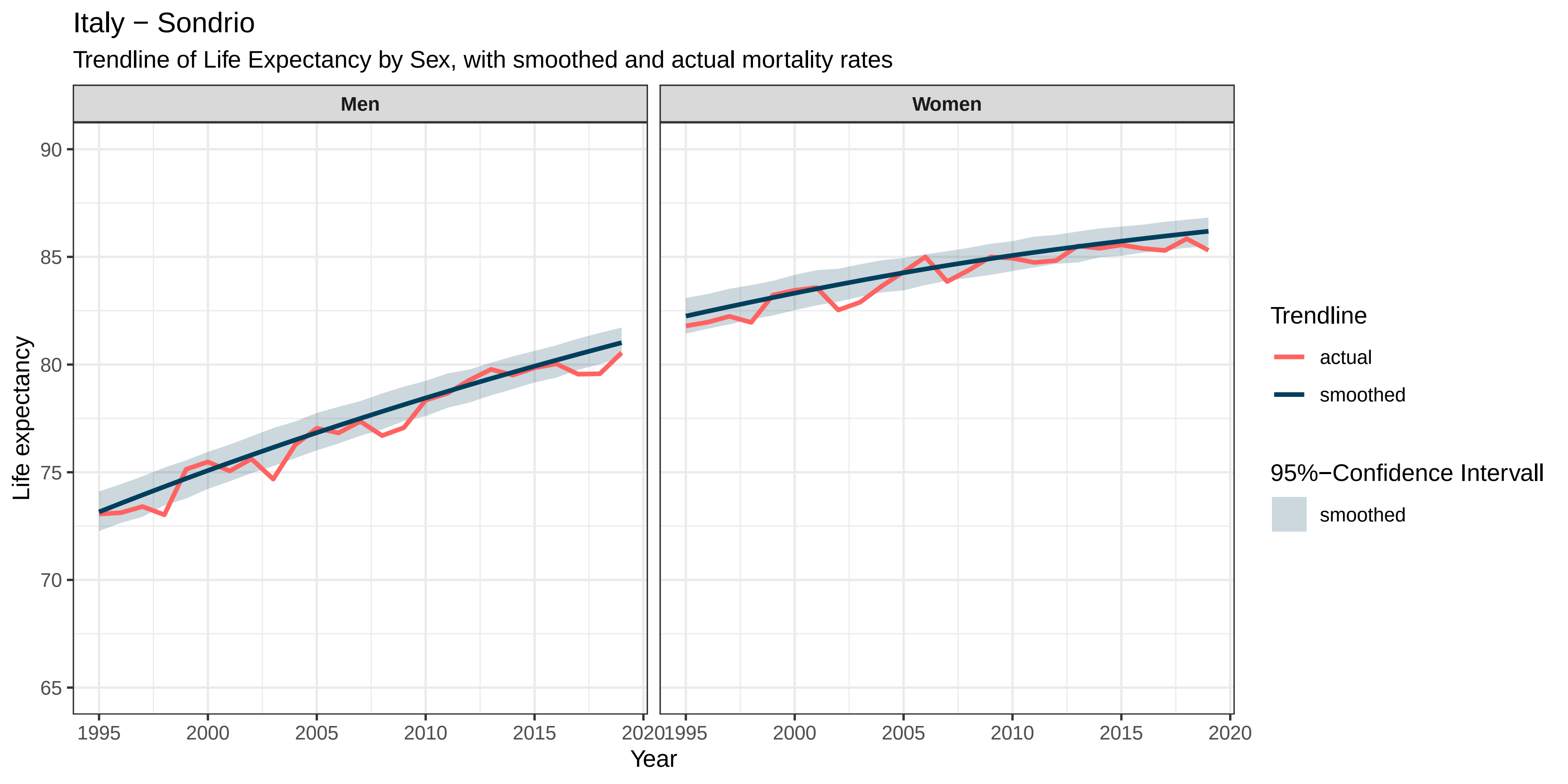
Italy − Sondrio
Trendline of Life Expectancy by Sex, with smoothed and actual mortality rates
Men
Women
90
85
80
75
70
65
1995
2000
2005
2010
2015
2020
Year
Life expectancy
Trendline
actual
smoothed
95%−Confidence Intervall
smoothed

Italy − Vercelli

Trendline of Life Expectancy by Sex, with smoothed and actual mortality rates

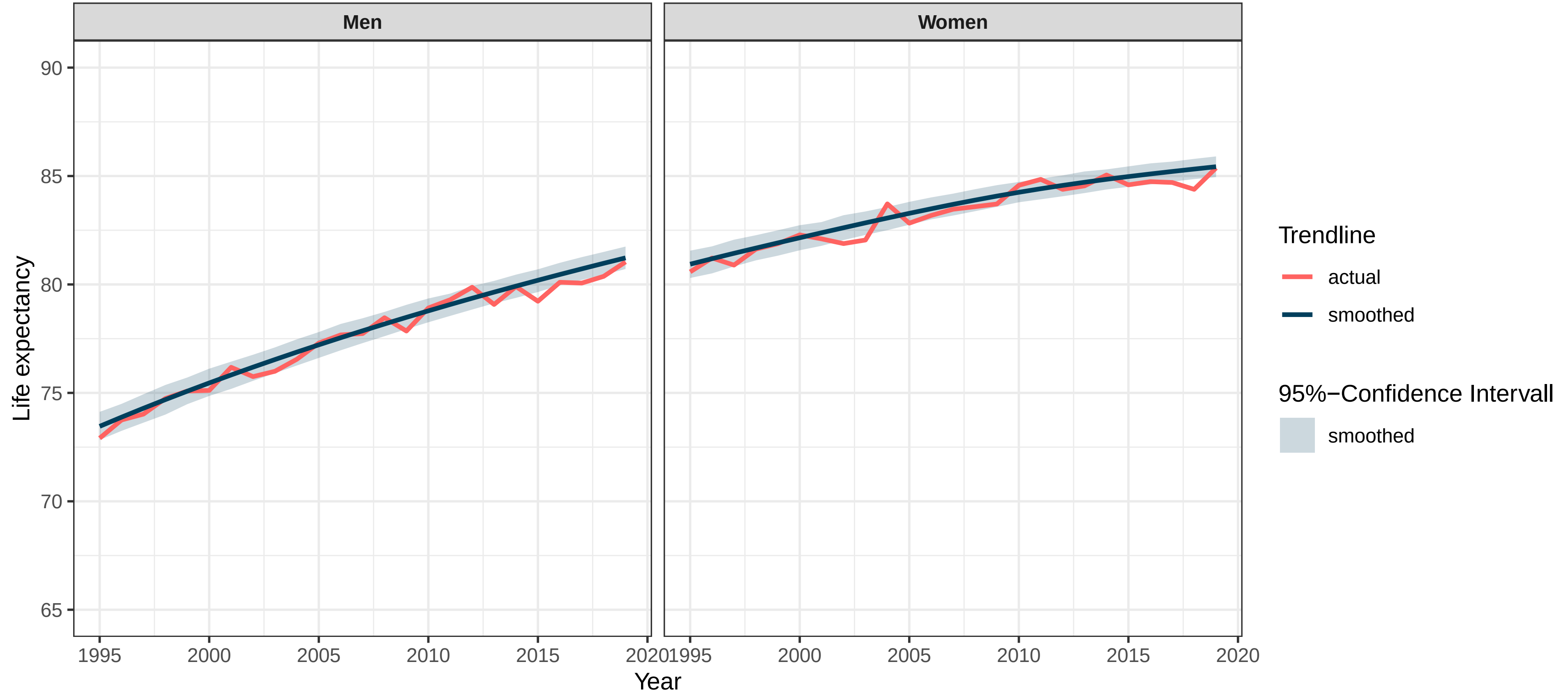

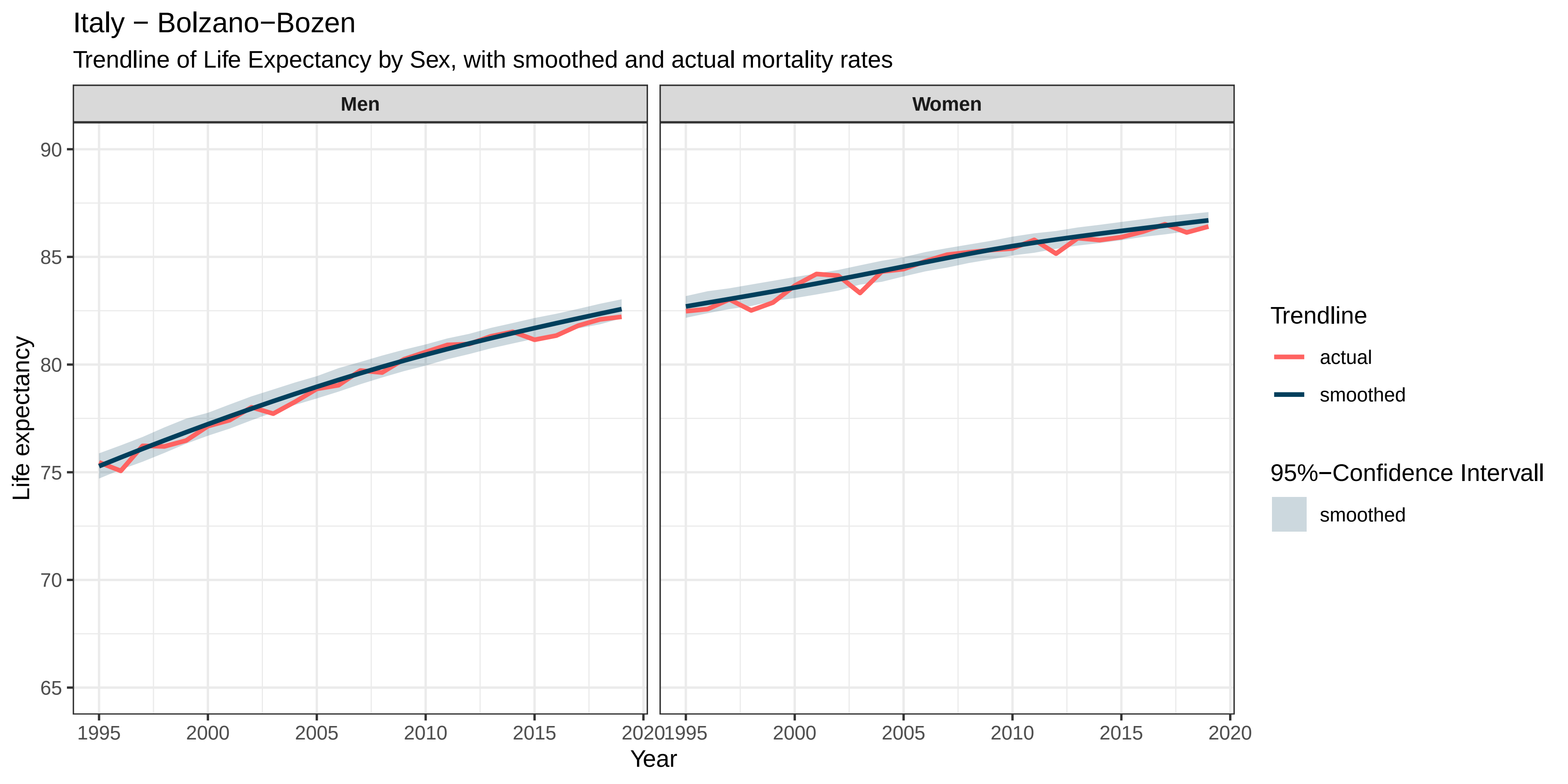

Italy − Bolzano−Bozen
Trendline of Life Expectancy by Sex, with smoothed and actual mortality rates
Men
Women
90
85
80
75
70
65
1995
2000
2005
2010
2015
2020
Life expectancy
Year
Trendline
actual
smoothed
95%−Confidence Intervall
smoothed

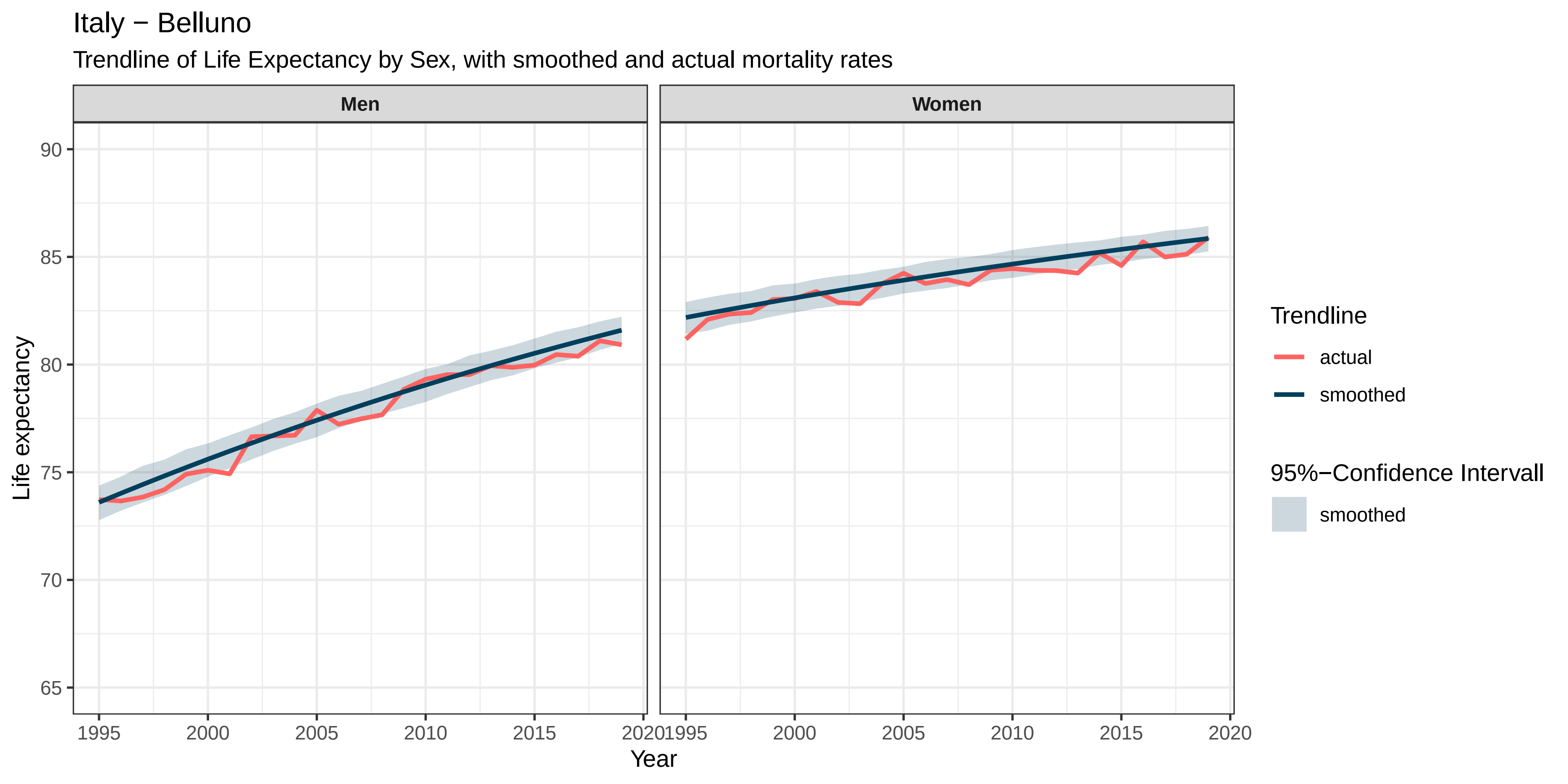
Italy − Belluno
Trendline of Life Expectancy by Sex, with smoothed and actual mortality rates
Men
Women
Life expectancy
Year
90
85
80
75
70
65
1995
2000
2005
2010
2015
2020
Trendline
actual
smoothed
95%−Confidence Intervall
smoothed

Italy − Novara

Trendline of Life Expectancy by Sex, with smoothed and actual mortality rates

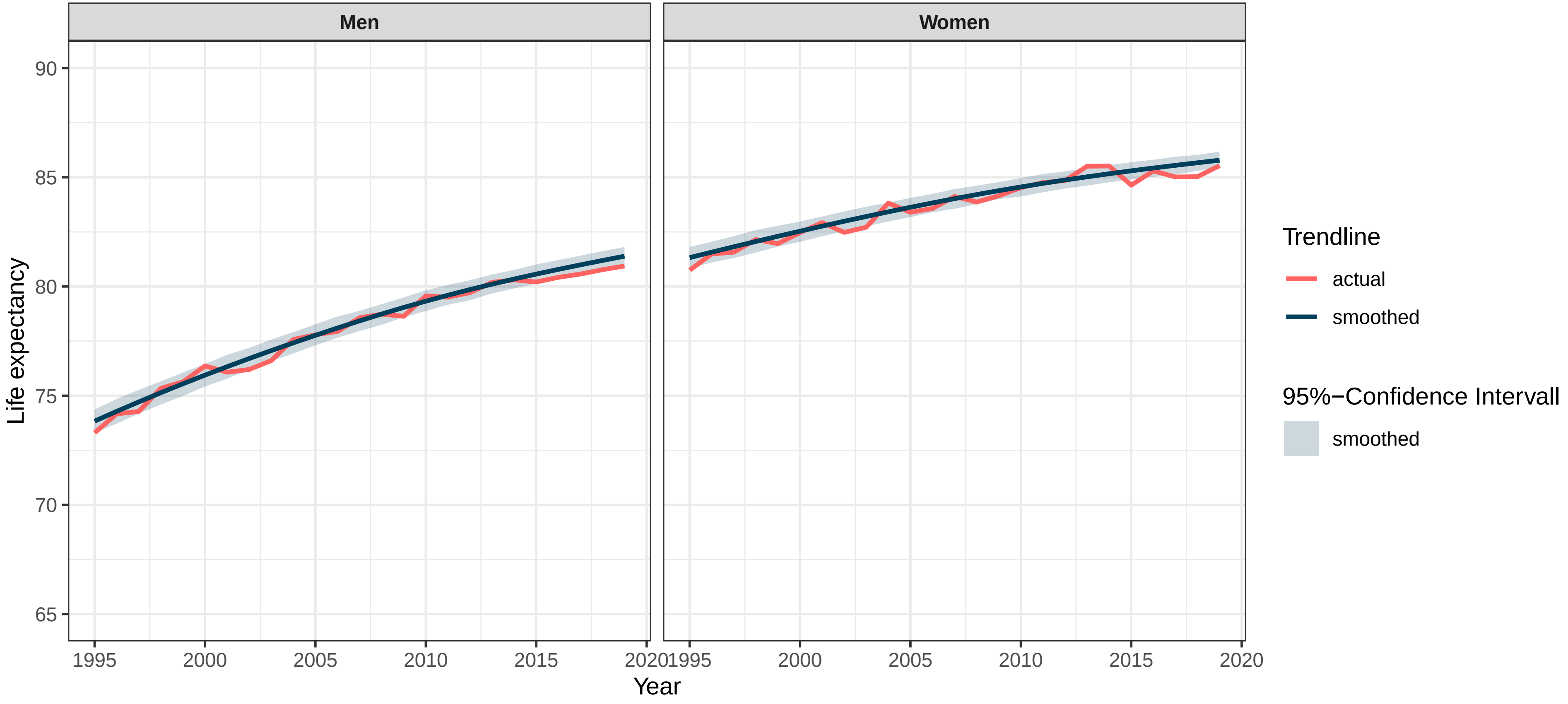

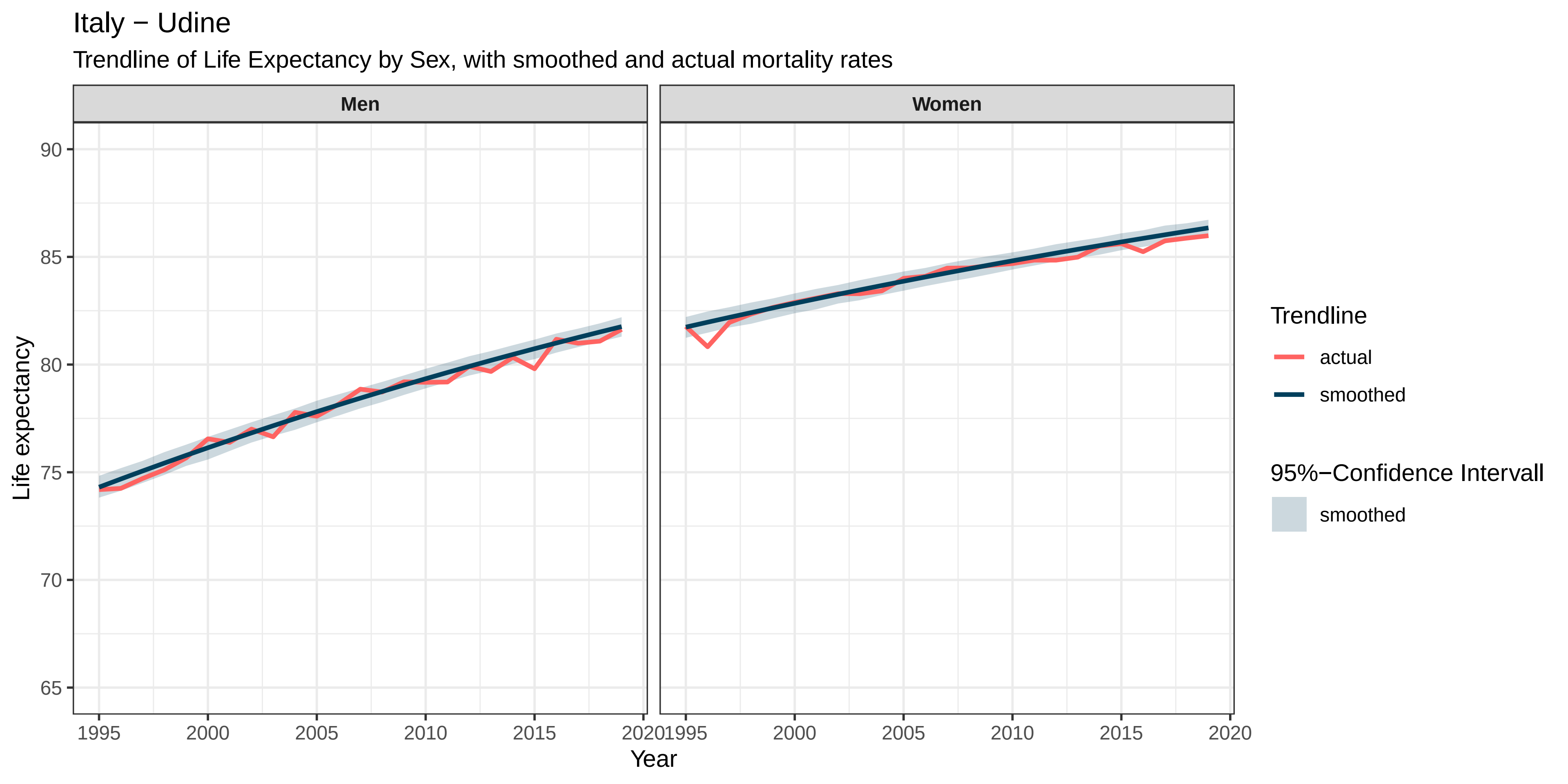

Italy − Udine
Trendline of Life Expectancy by Sex, with smoothed and actual mortality rates
Men
Women
90
85
80
75
70
65
Life expectancy
1995
2000
2005
2010
2015
2020
Year
Trendline
actual
smoothed
95%−Confidence Intervall
smoothed

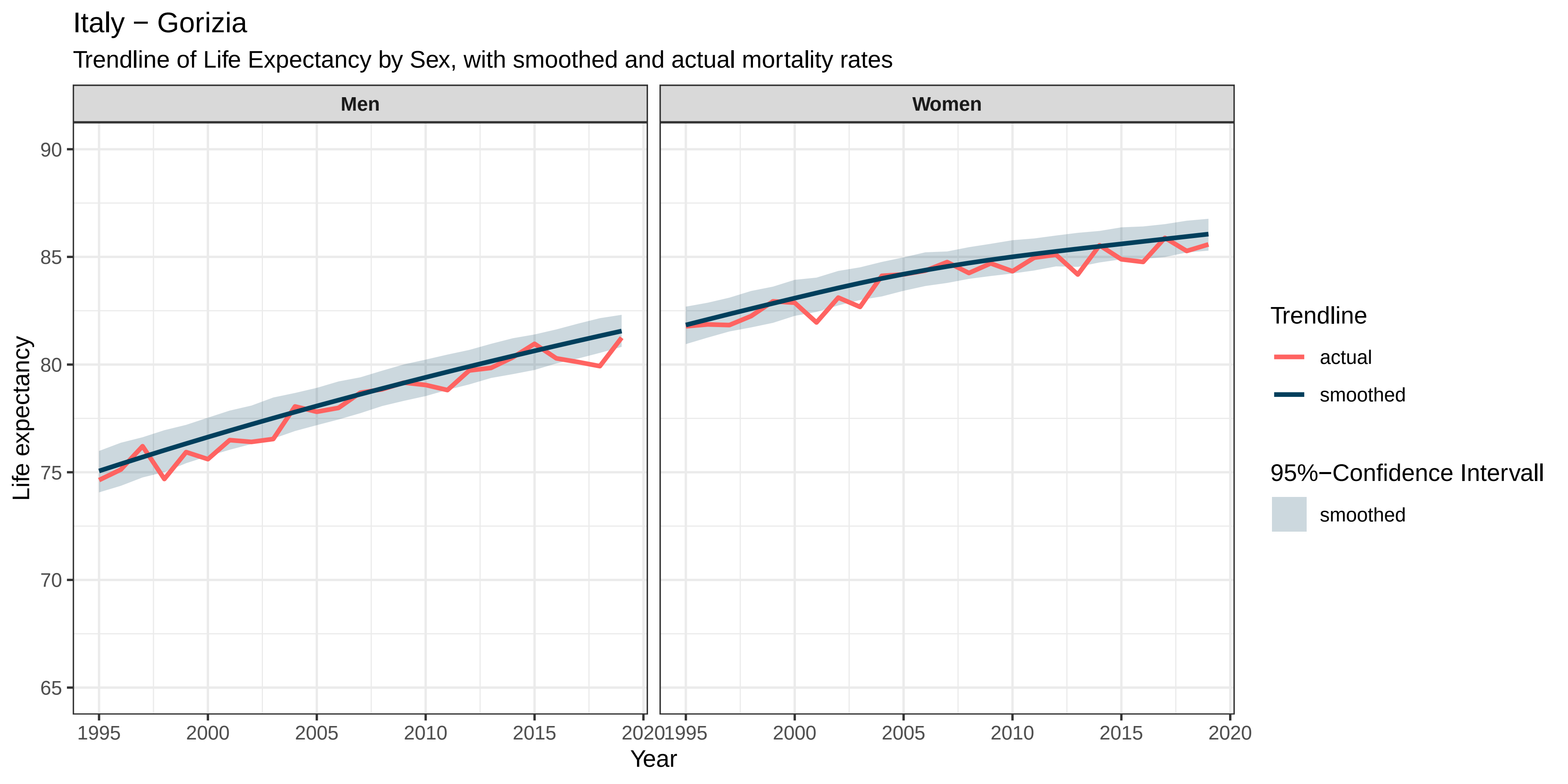

Italy − Gorizia
Trendline of Life Expectancy by Sex, with smoothed and actual mortality rates
Men
Women
90
85
80
75
70
65
Life expectancy
1995
2000
2005
2010
2015
2020
Year
Trendline
actual
smoothed
95%−Confidence Intervall
smoothed

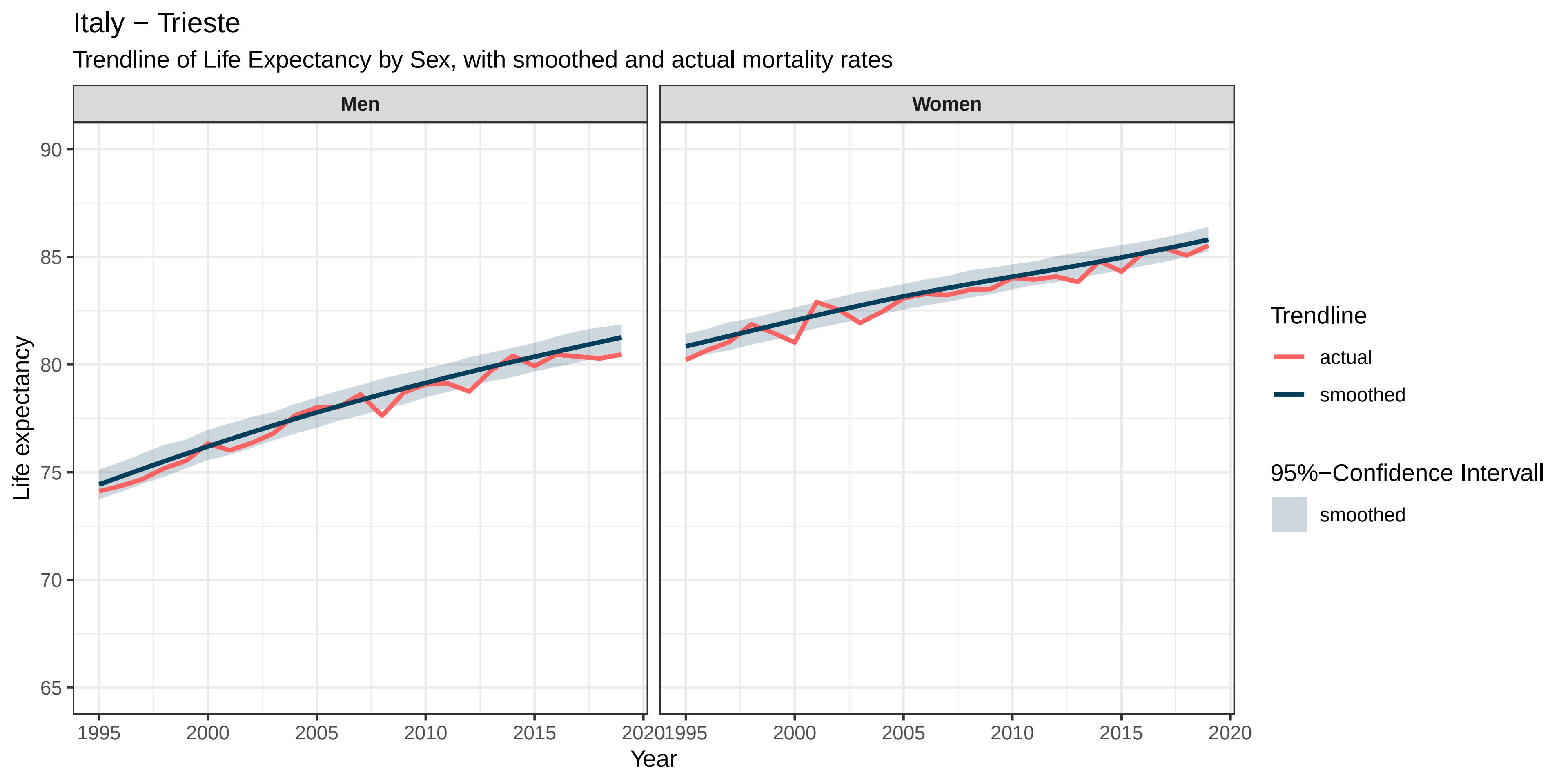
Italy − Trieste
Trendline of Life Expectancy by Sex, with smoothed and actual mortality rates
Men
Women
90
85
80
75
70
65
1995
2000
2005
2010
2015
2020
Year
Life expectancy
Trendline
actual
smoothed
95%−Confidence Intervall
smoothed

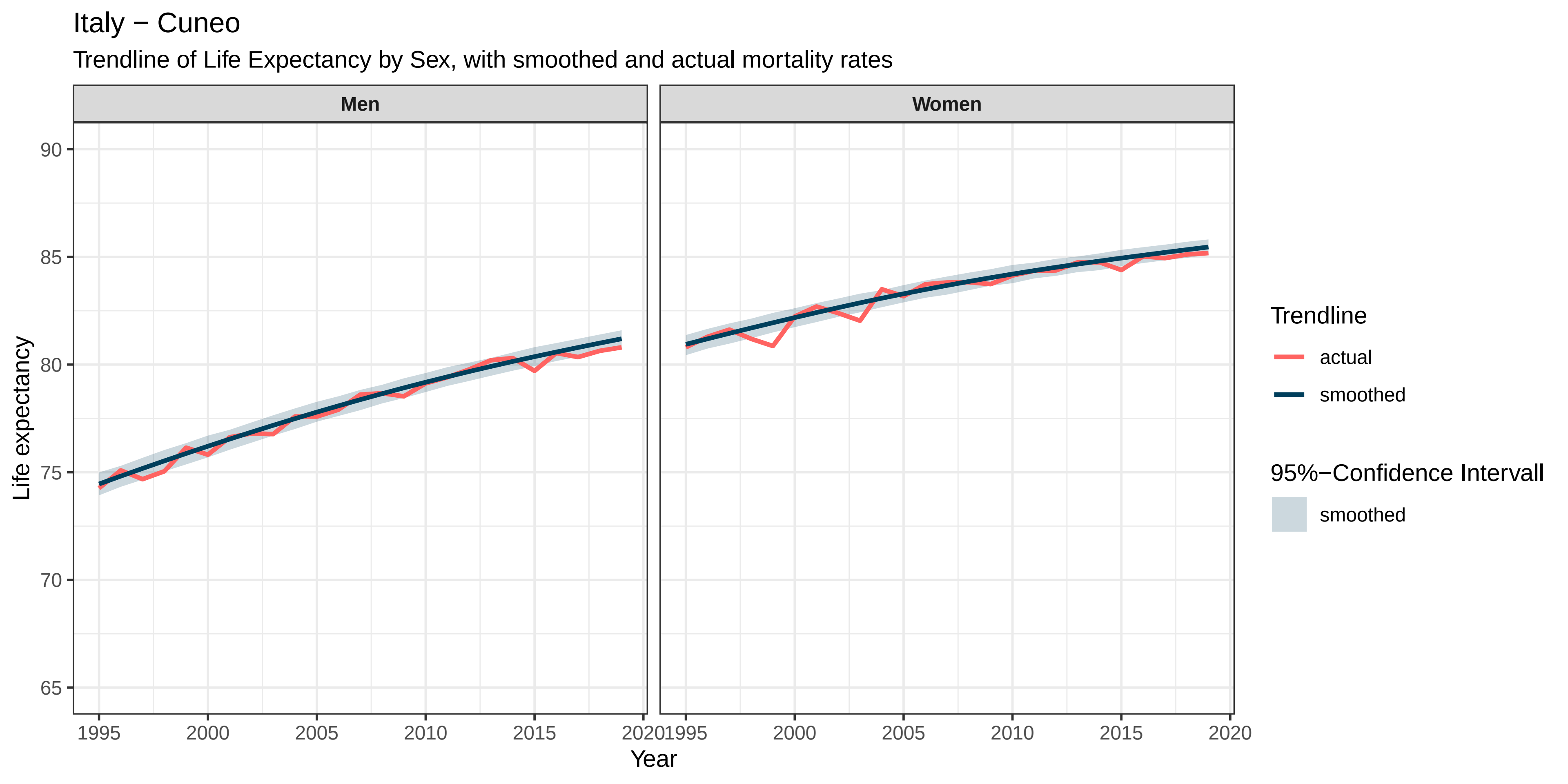

Italy − Cuneo
Trendline of Life Expectancy by Sex, with smoothed and actual mortality rates
Men
Women
Life expectancy
90
85
80
75
70
65
1995
2000
2005
2010
2015
2020
Year
Trendline
actual
smoothed
95%−Confidence Intervall
smoothed

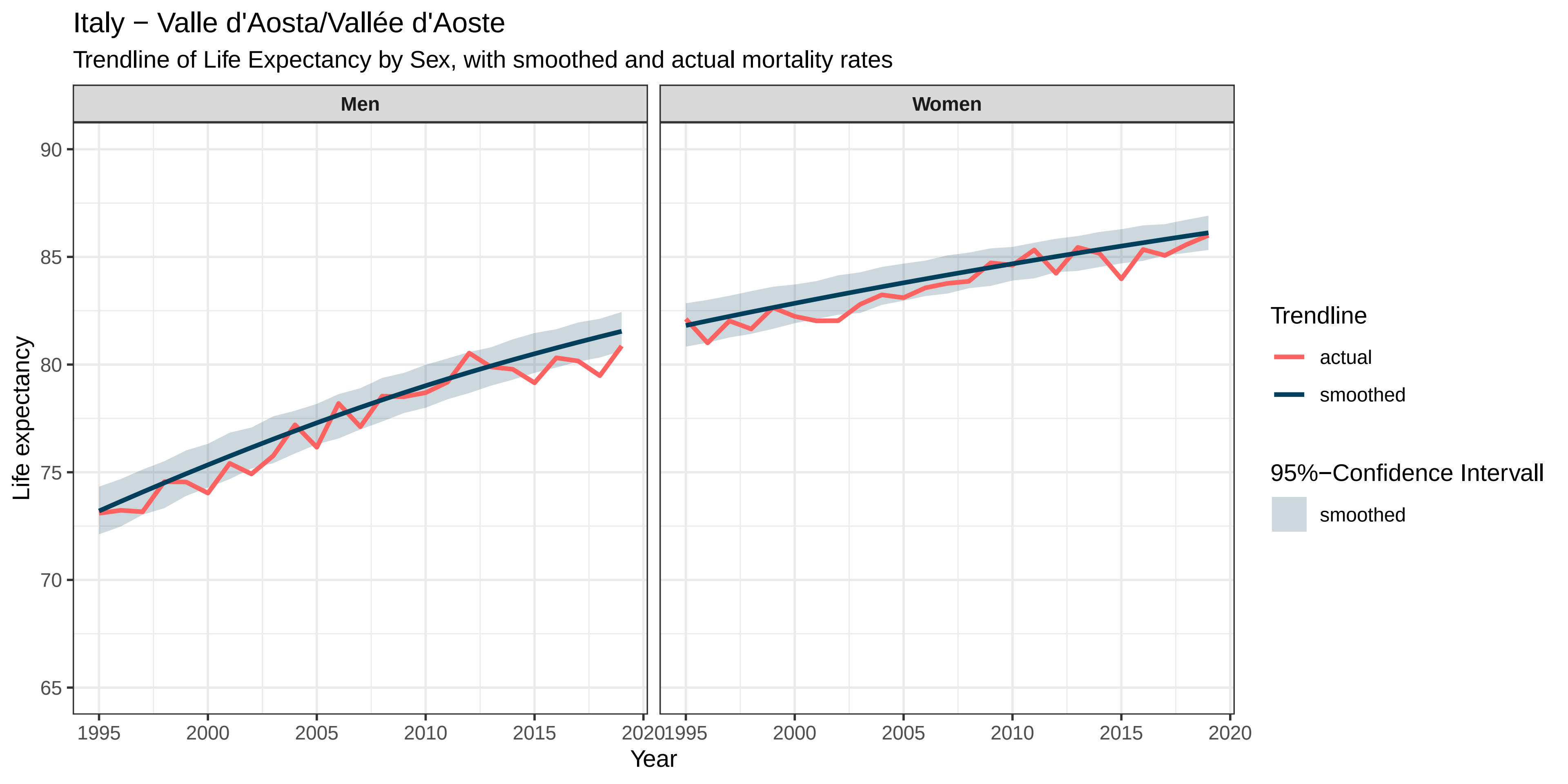

Italy − Valle d'Aosta/Vallée d'Aoste
Trendline of Life Expectancy by Sex, with smoothed and actual mortality rates
Men
Women
Life expectancy
90
85
80
75
70
65
1995
2000
2005
2010
2015
2020
Year
Trendline
actual
smoothed
95%−Confidence Intervall
smoothed

Italy − Imperia

Trendline of Life Expectancy by Sex, with smoothed and actual mortality rates

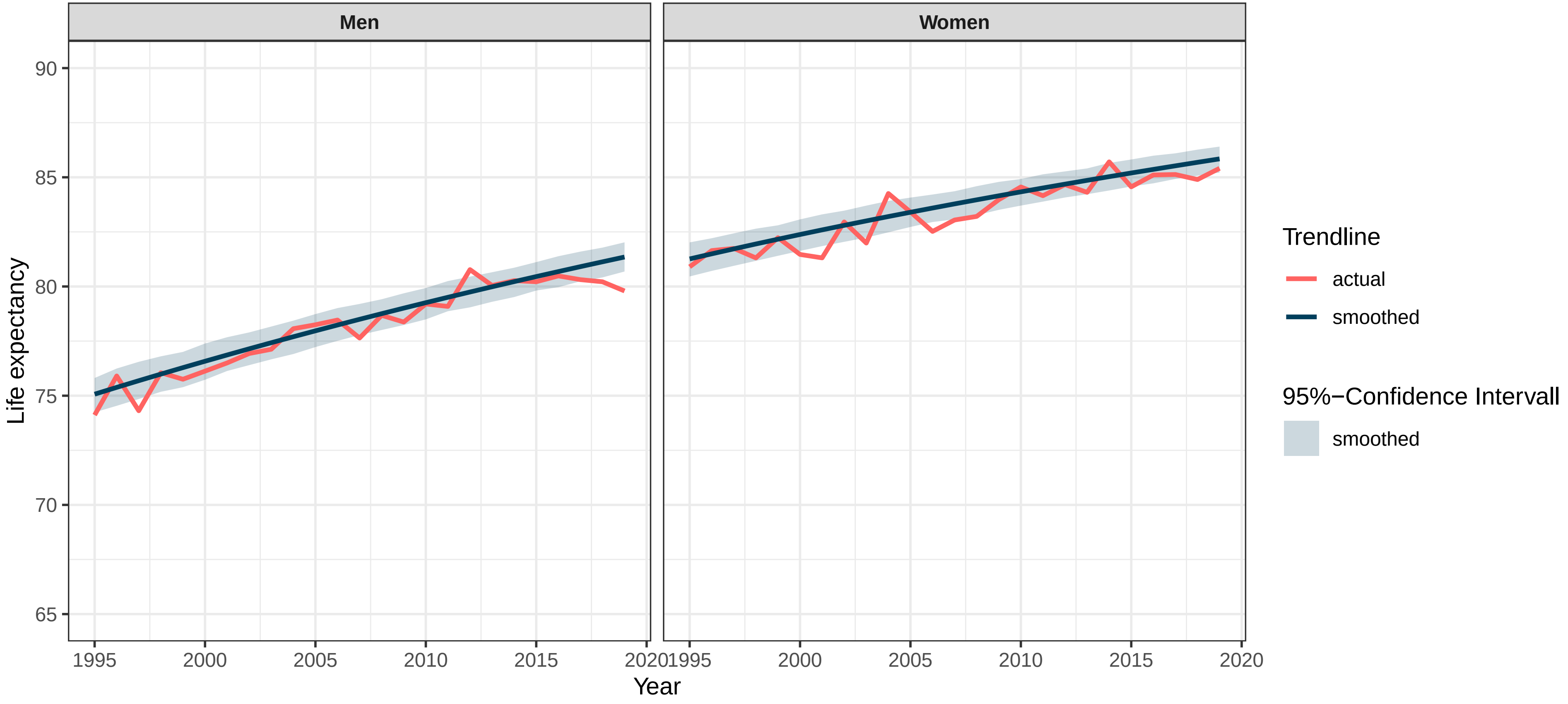

Denmark − Byen København

Trendline of Life Expectancy by Sex, with smoothed and actual mortality rates

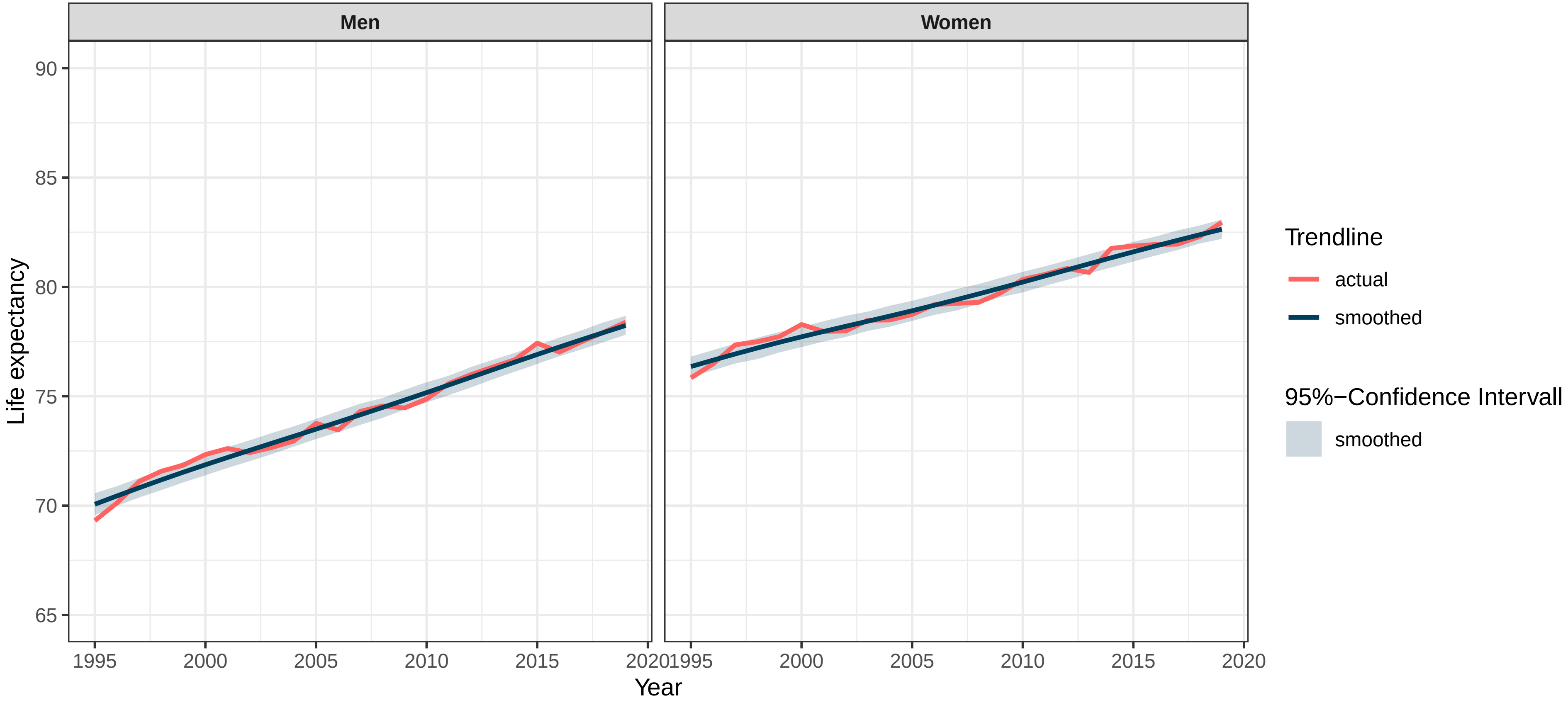

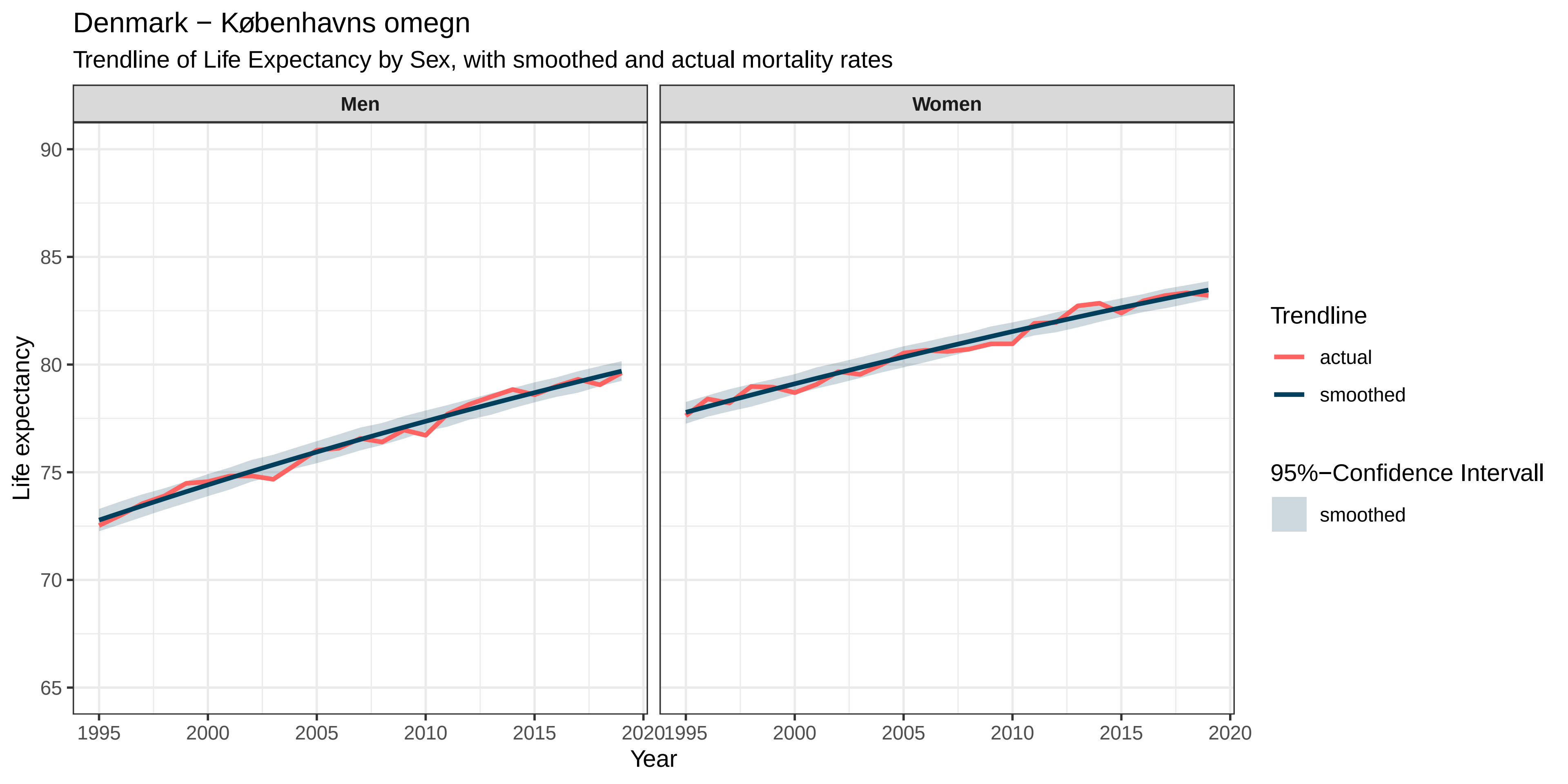

Denmark − Københavns omegn
Trendline of Life Expectancy by Sex, with smoothed and actual mortality rates
Men
Women
Life expectancy
90
85
80
75
70
65
1995
2000
2005
2010
2015
2020
Year
Trendline
actual
smoothed
95%−Confidence Intervall
smoothed

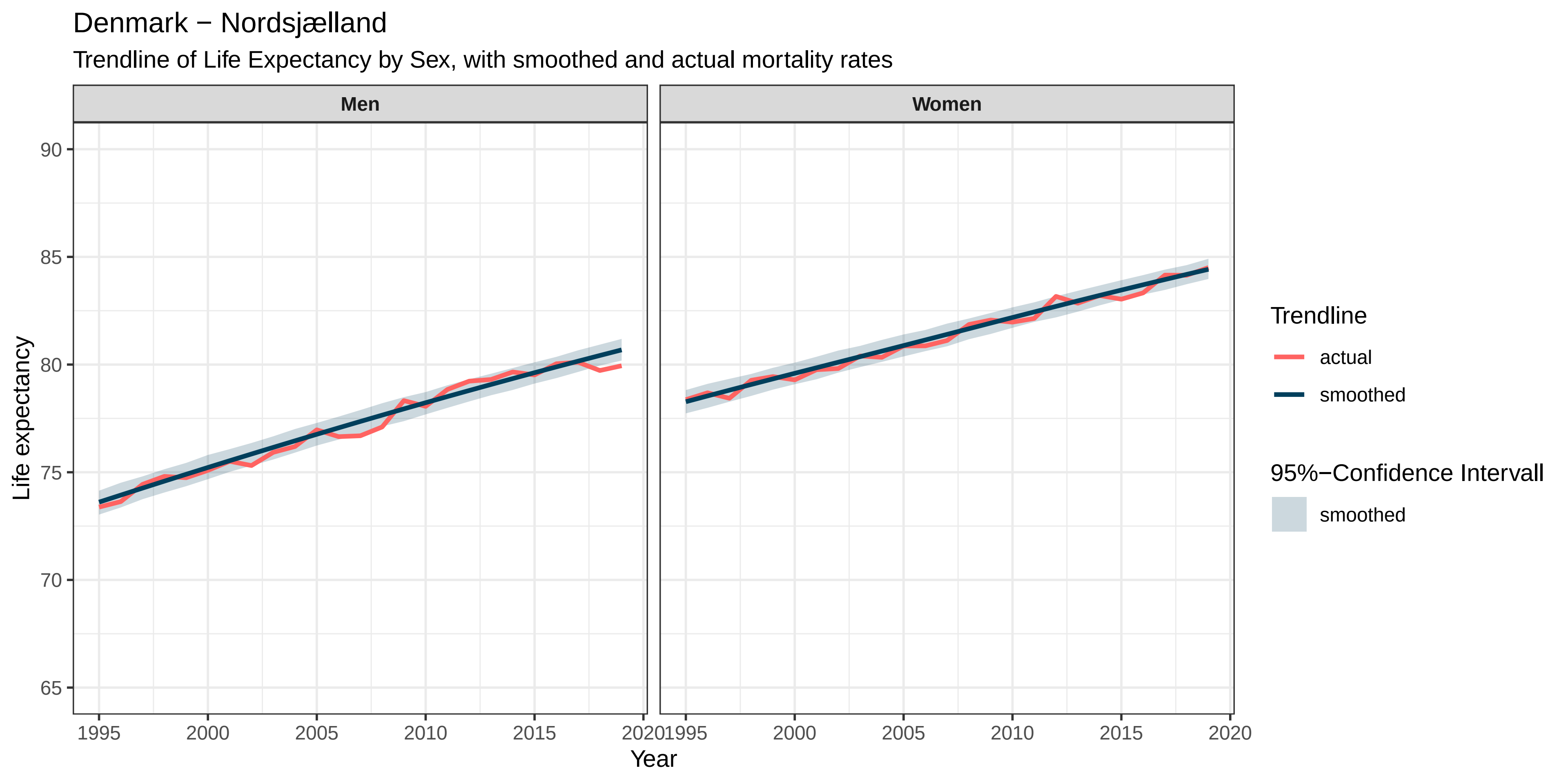

Denmark − Nordsjælland
Trendline of Life Expectancy by Sex, with smoothed and actual mortality rates
Men
Women
Life expectancy
90
85
80
75
70
65
1995
2000
2005
2010
2015
2020
Year
Trendline
actual
smoothed
95%−Confidence Intervall
smoothed

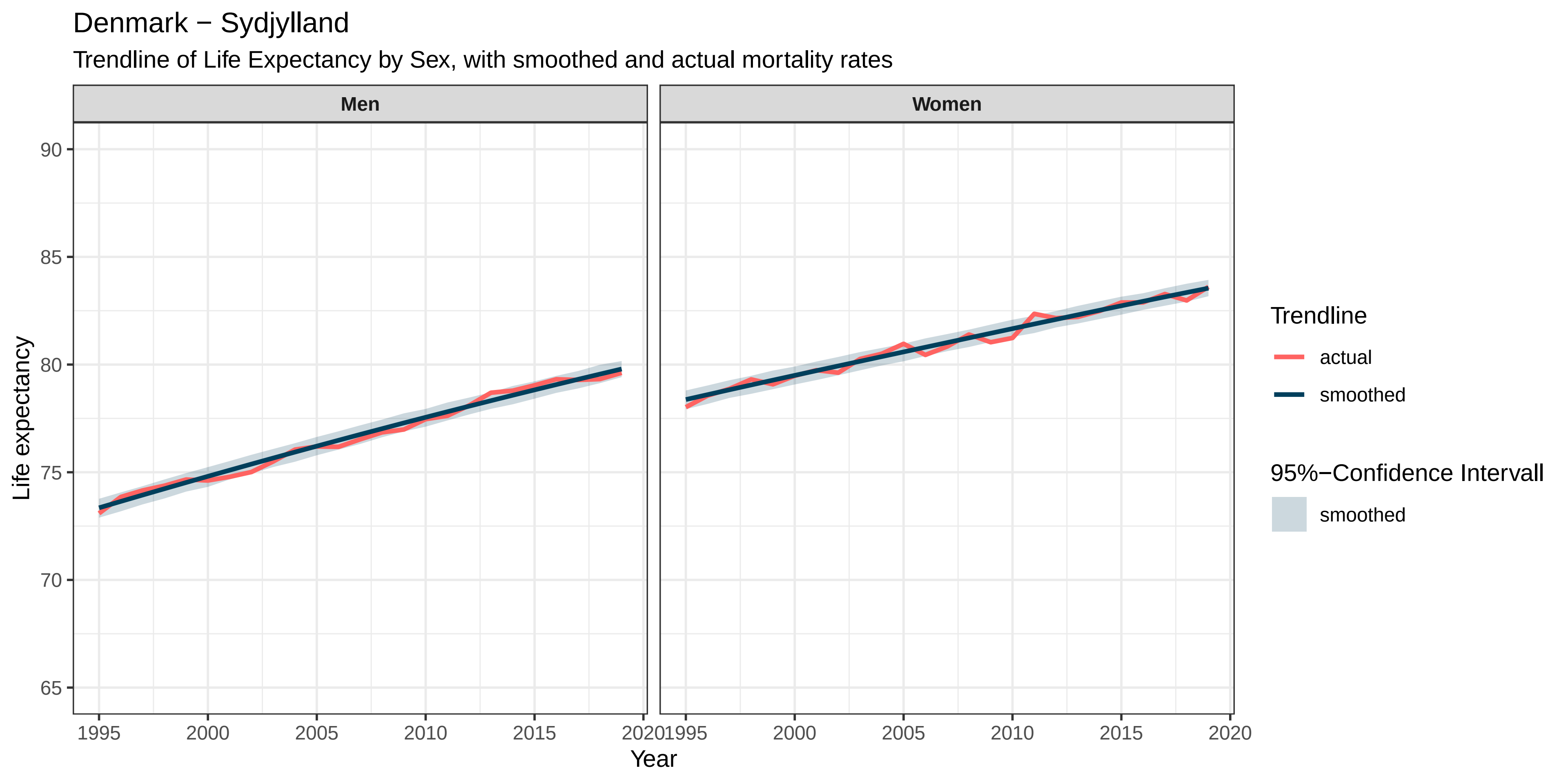

Denmark − Sydjylland
Trendline of Life Expectancy by Sex, with smoothed and actual mortality rates
Men
Women
90
85
80
75
70
65
1995
2000
2005
2010
2015
2020
Year
Life expectancy
Trendline
actual
smoothed
95%−Confidence Intervall
smoothed

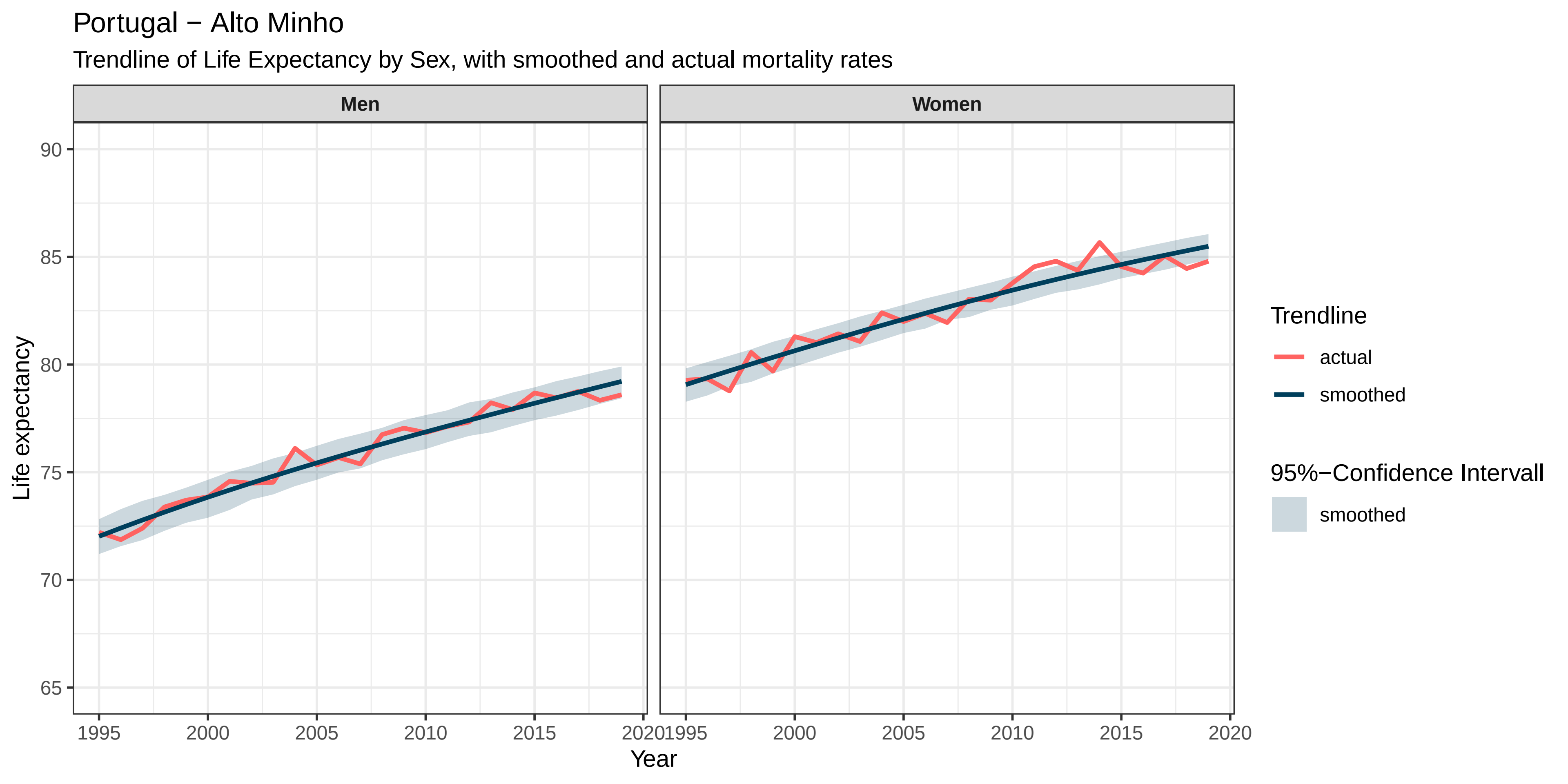

Portugal − Alto Minho
Trendline of Life Expectancy by Sex, with smoothed and actual mortality rates
Men
Women
Life expectancy
Year
90
85
80
75
70
65
1995
2000
2005
2010
2015
2020
Trendline
actual
smoothed
95%−Confidence Intervall
smoothed

Portugal − Algarve

Trendline of Life Expectancy by Sex, with smoothed and actual mortality rates

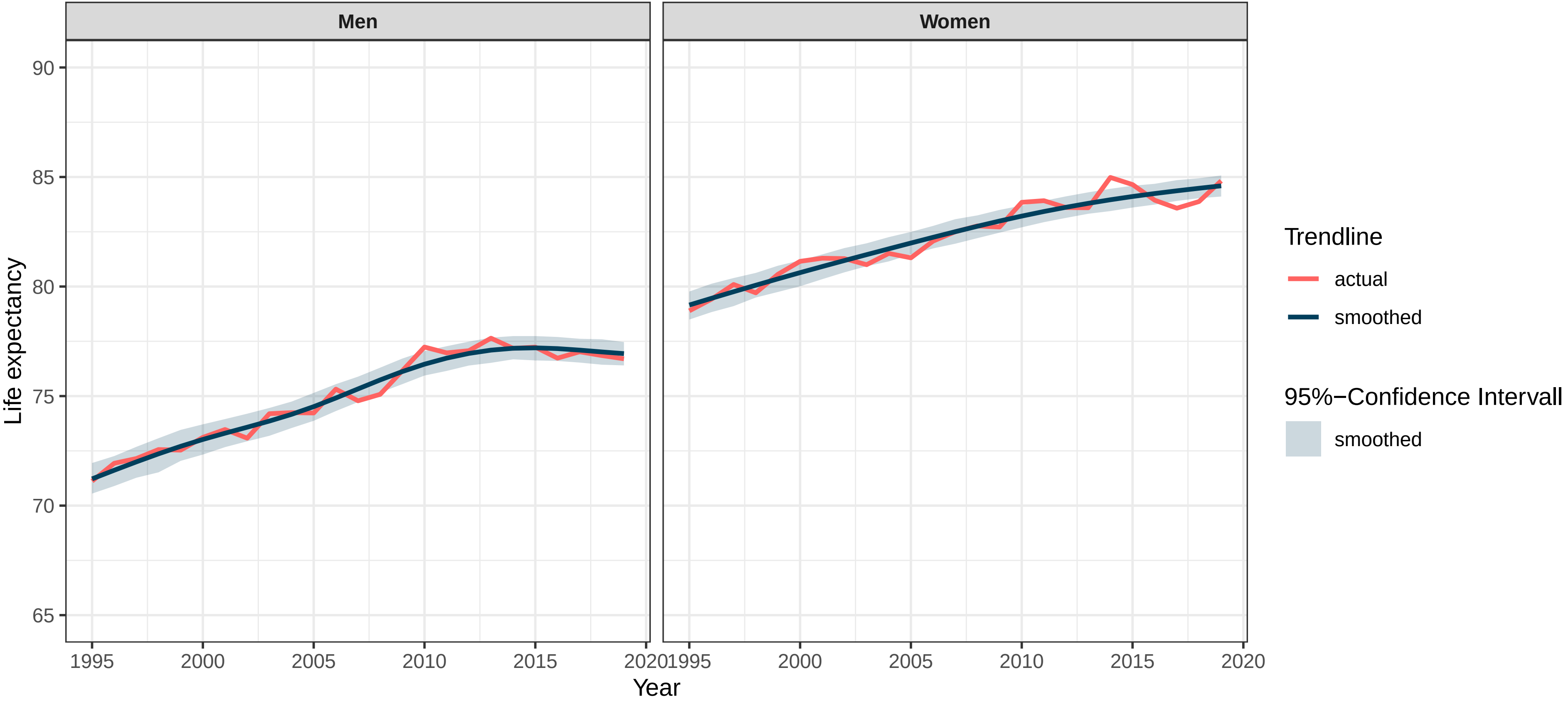

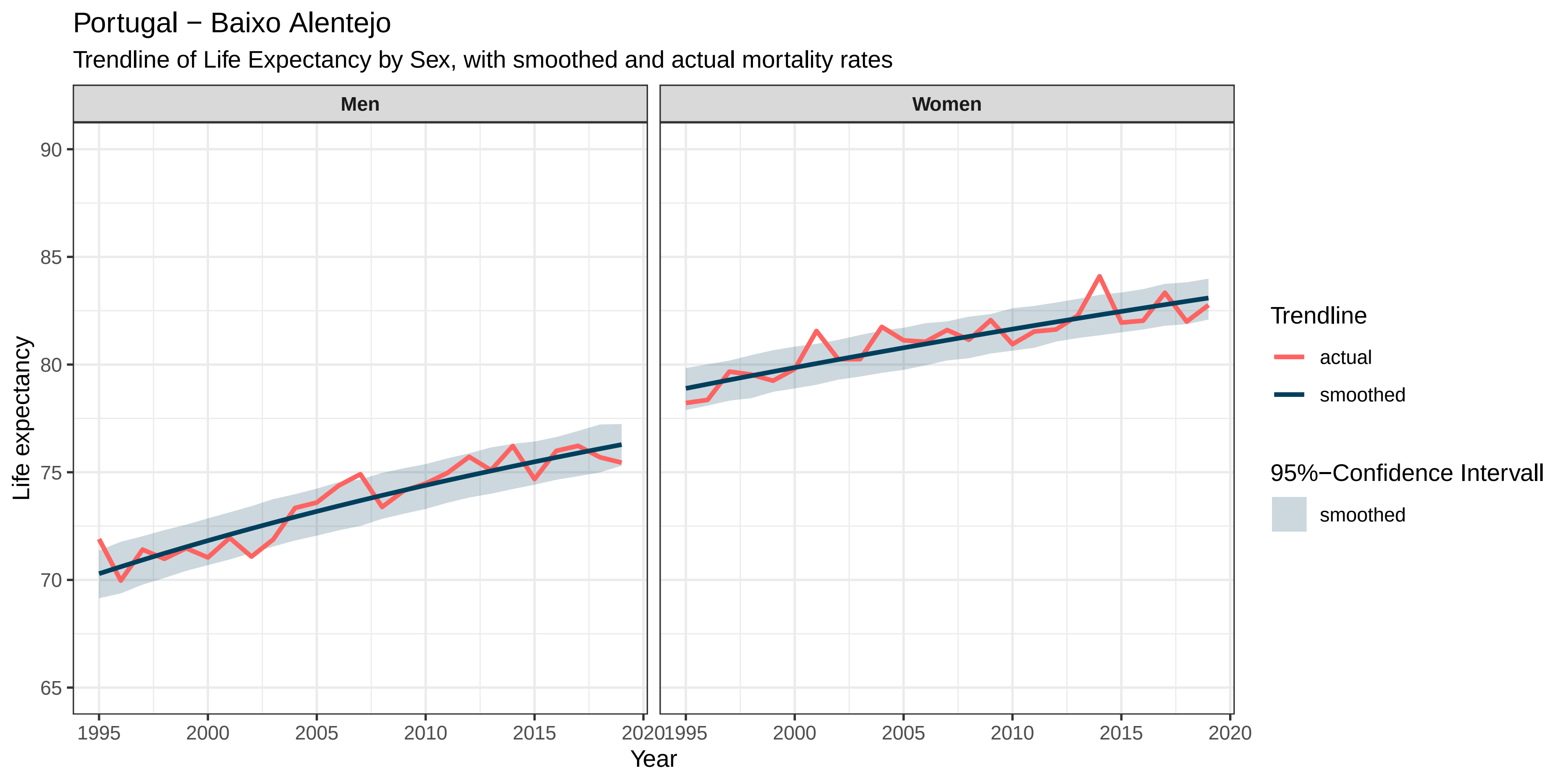
Portugal − Baixo Alentejo
Trendline of Life Expectancy by Sex, with smoothed and actual mortality rates
Men
Women
Life expectancy
Year
90
85
80
75
70
65
1995
2000
2005
2010
2015
2020
Trendline
actual
smoothed
95%−Confidence Intervall
smoothed

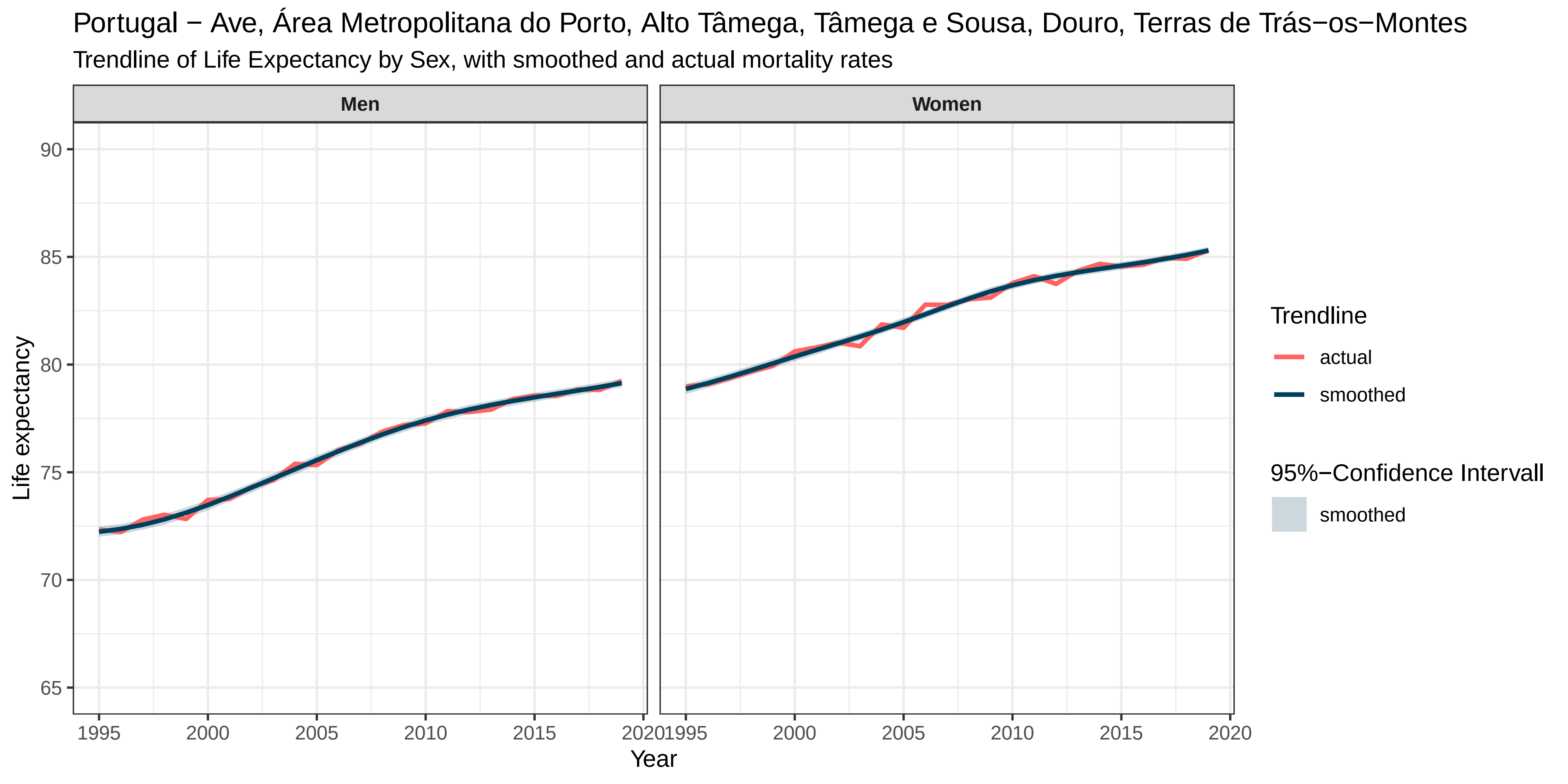
Portugal − Ave, Área Metropolitana do Porto, Alto Tâmega, Tâmega e Sousa, Douro, Terras de Trás−os−Montes
Trendline of Life Expectancy by Sex, with smoothed and actual mortality rates
Men
Women
Life expectancy
Year
90
85
80
75
70
65
1995
2000
2005
2010
2015
2020
Trendline
actual
smoothed
95%−Confidence Intervall
smoothed

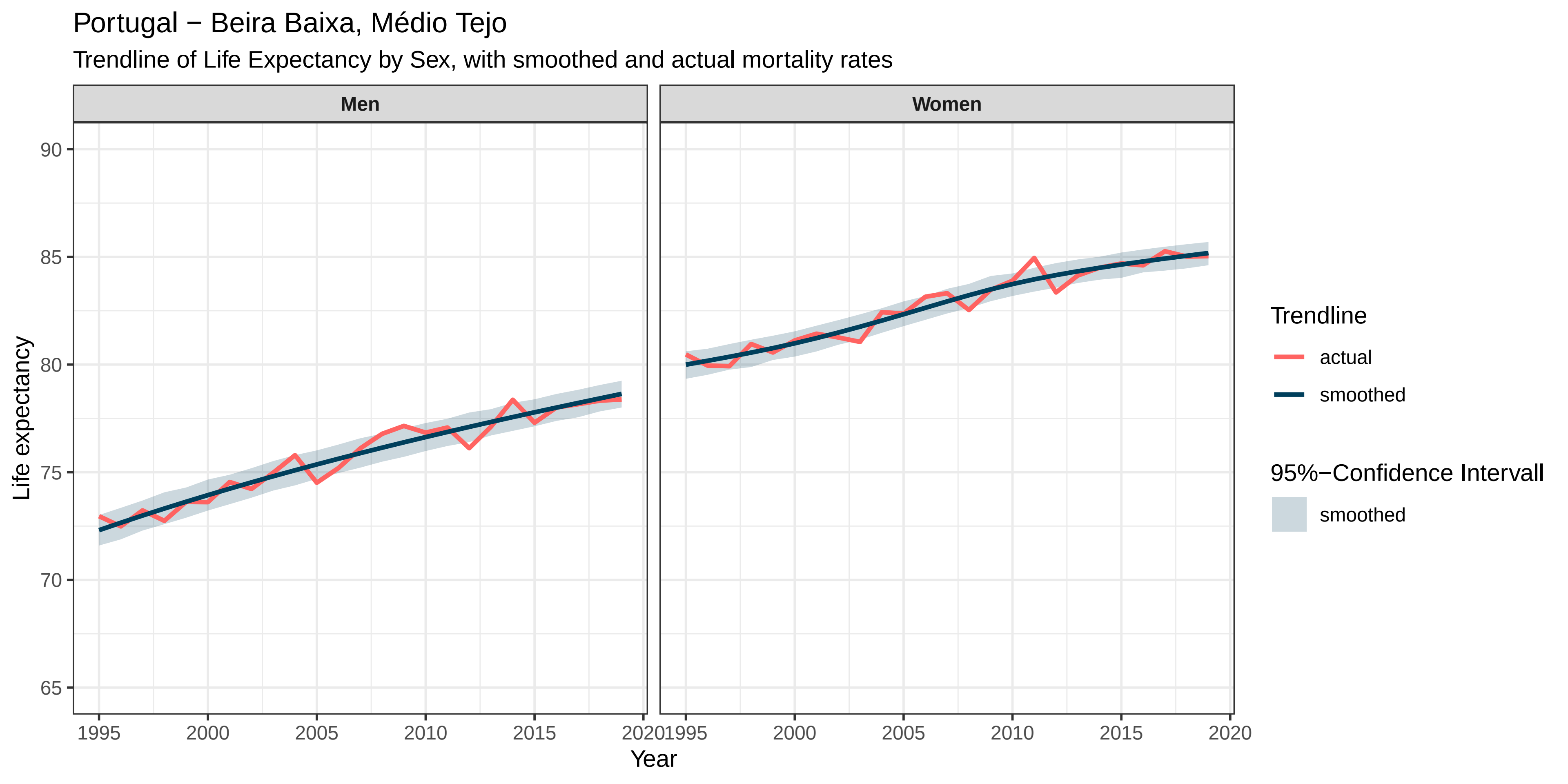
Portugal − Beira Baixa, Médio Tejo
Trendline of Life Expectancy by Sex, with smoothed and actual mortality rates
Men
Women
Life expectancy
Year
90
85
80
75
70
65
1995
2000
2005
2010
2015
2020
Trendline
actual
smoothed
95%−Confidence Intervall
smoothed

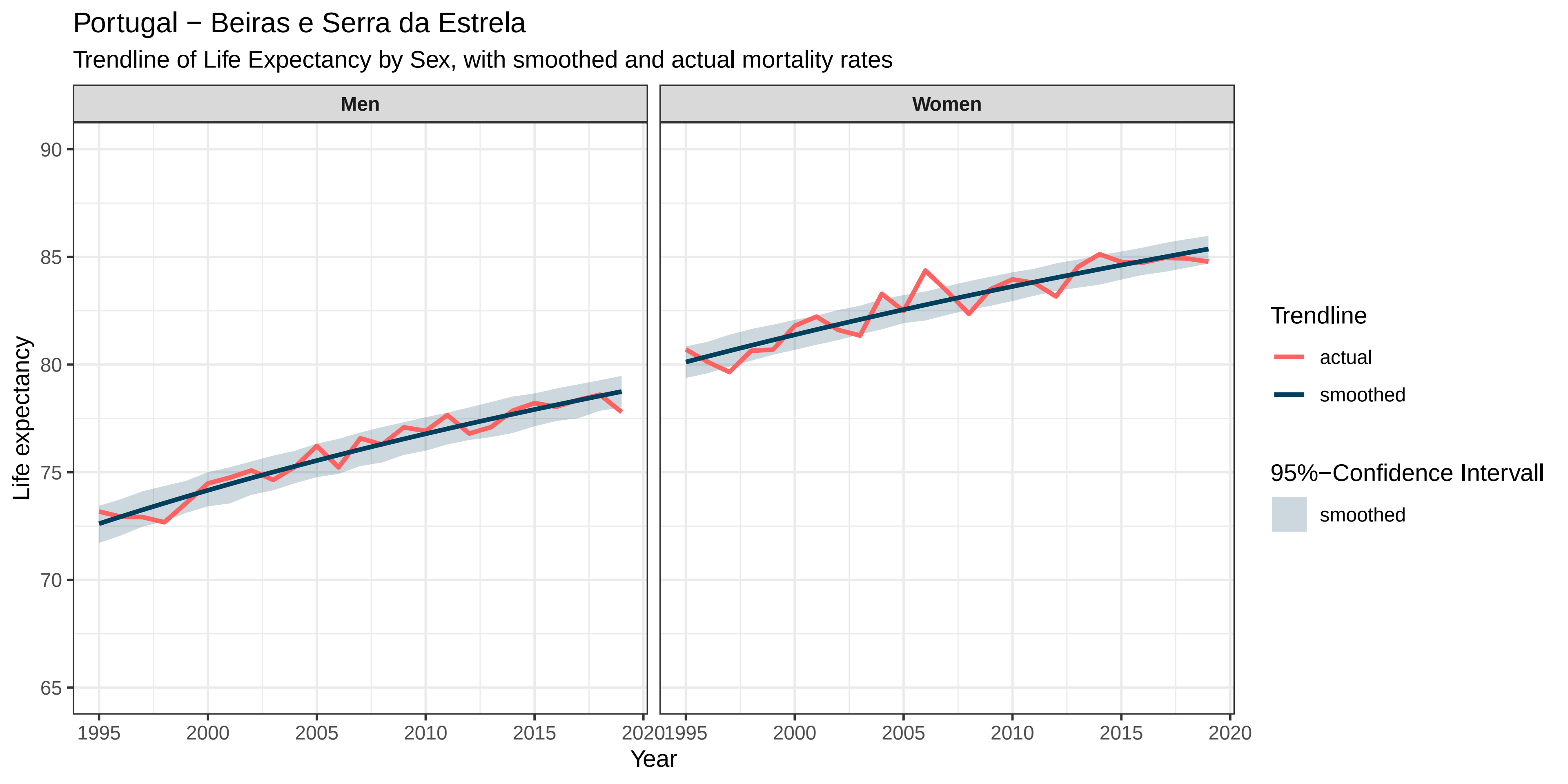

Portugal − Beiras e Serra da Estrela
Trendline of Life Expectancy by Sex, with smoothed and actual mortality rates
Men
Women
Life expectancy
90
85
80
75
70
65
1995
2000
2005
2010
2015
2020
Year
Trendline
actual
smoothed
95%−Confidence Intervall
smoothed

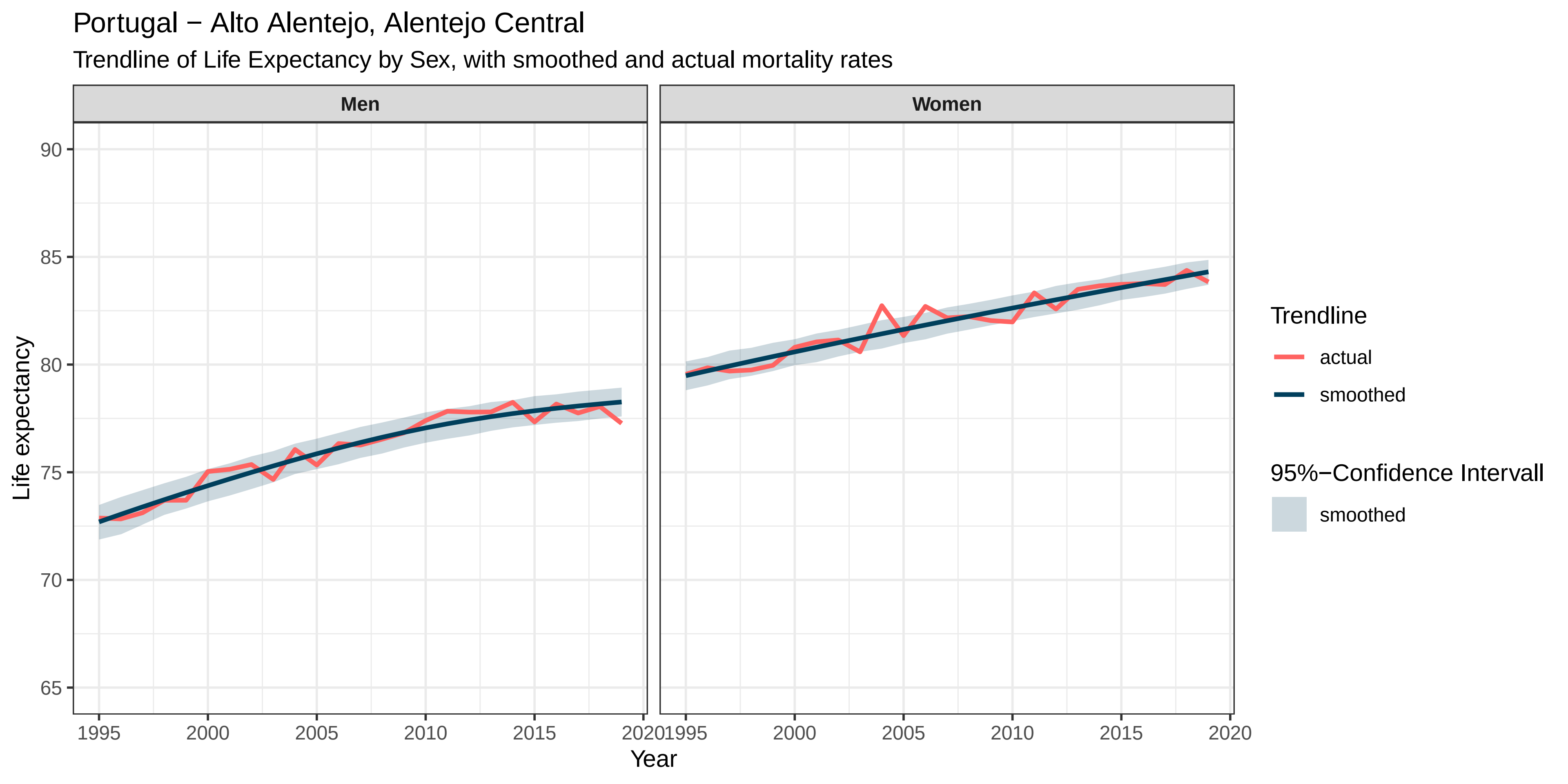

Portugal − Alto Alentejo, Alentejo Central
Trendline of Life Expectancy by Sex, with smoothed and actual mortality rates
Men
Women
Life expectancy
90
85
80
75
70
65
1995
2000
2005
2010
2015
2020
Year
Trendline
actual
smoothed
95%−Confidence Intervall
smoothed

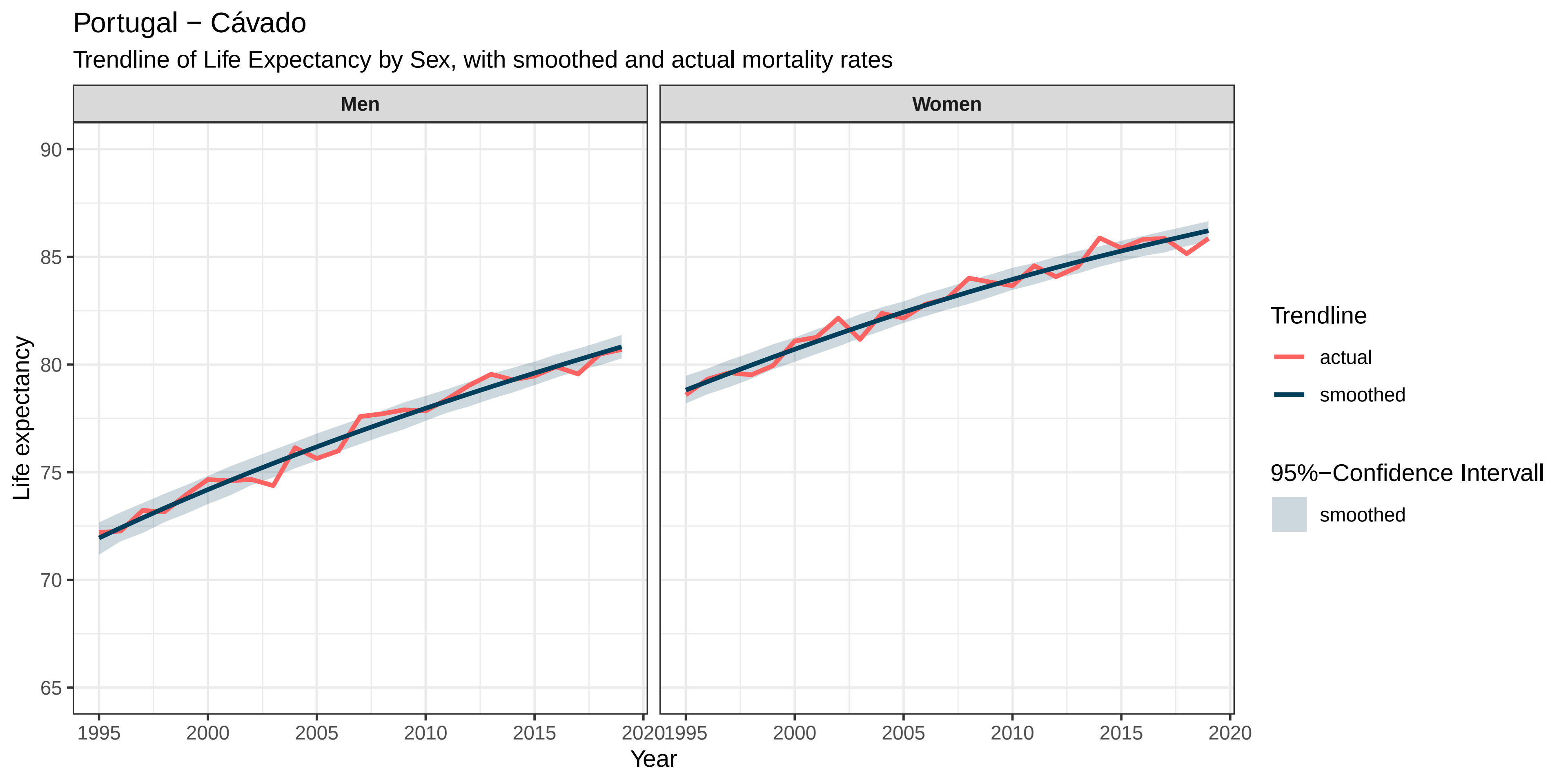
Portugal − Cávado
Trendline of Life Expectancy by Sex, with smoothed and actual mortality rates
Men
Women
Life expectancy
Year
90
85
80
75
70
65
1995
2000
2005
2010
2015
2020
Trendline
actual
smoothed
95%−Confidence Intervall
smoothed

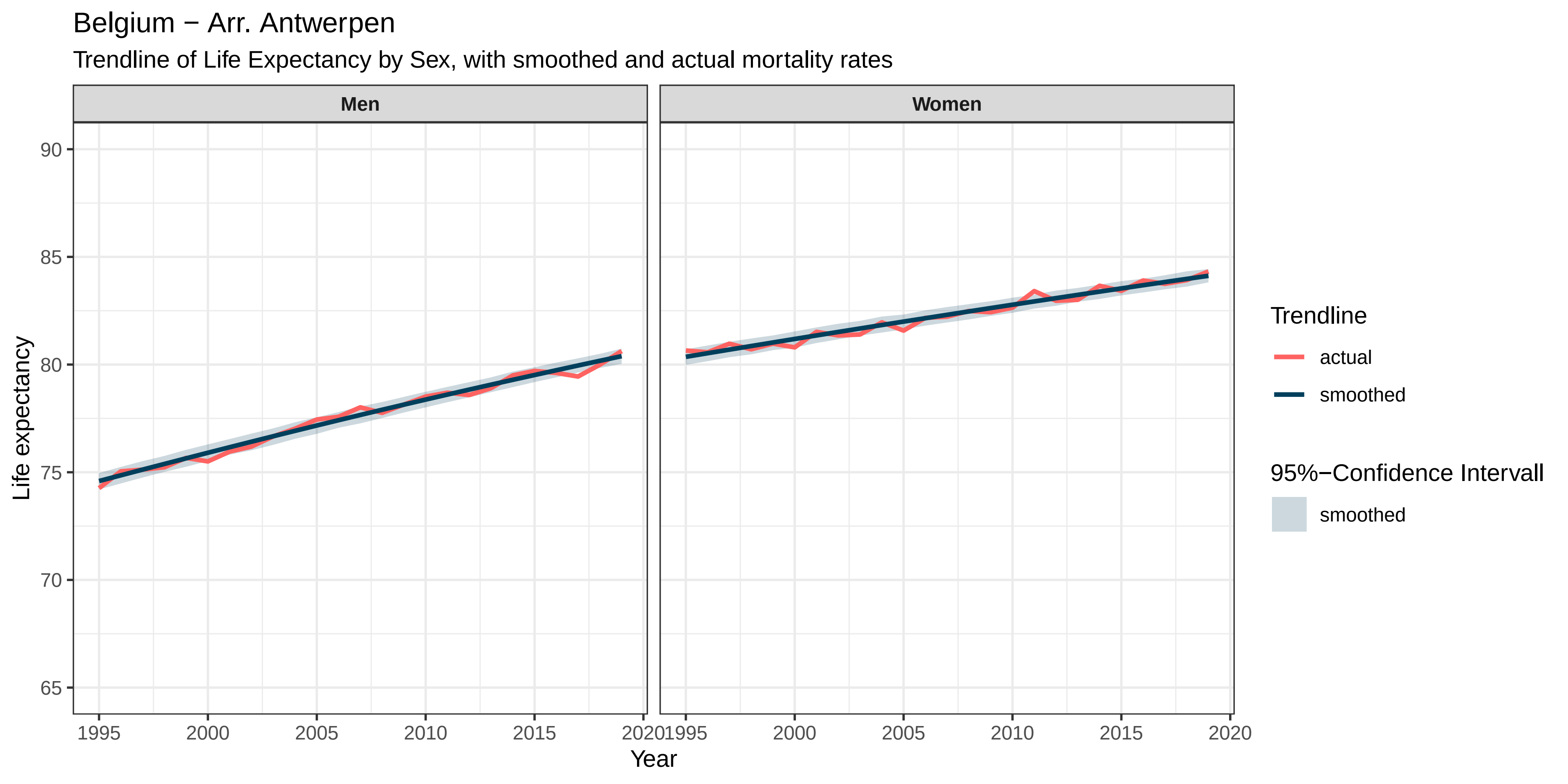
Belgium − Arr. Antwerpen
Trendline of Life Expectancy by Sex, with smoothed and actual mortality rates
Men
Women
Life expectancy
90
85
80
75
70
65
1995
2000
2005
2010
2015
2020
Year
Trendline
actual
smoothed
95%−Confidence Intervall
smoothed

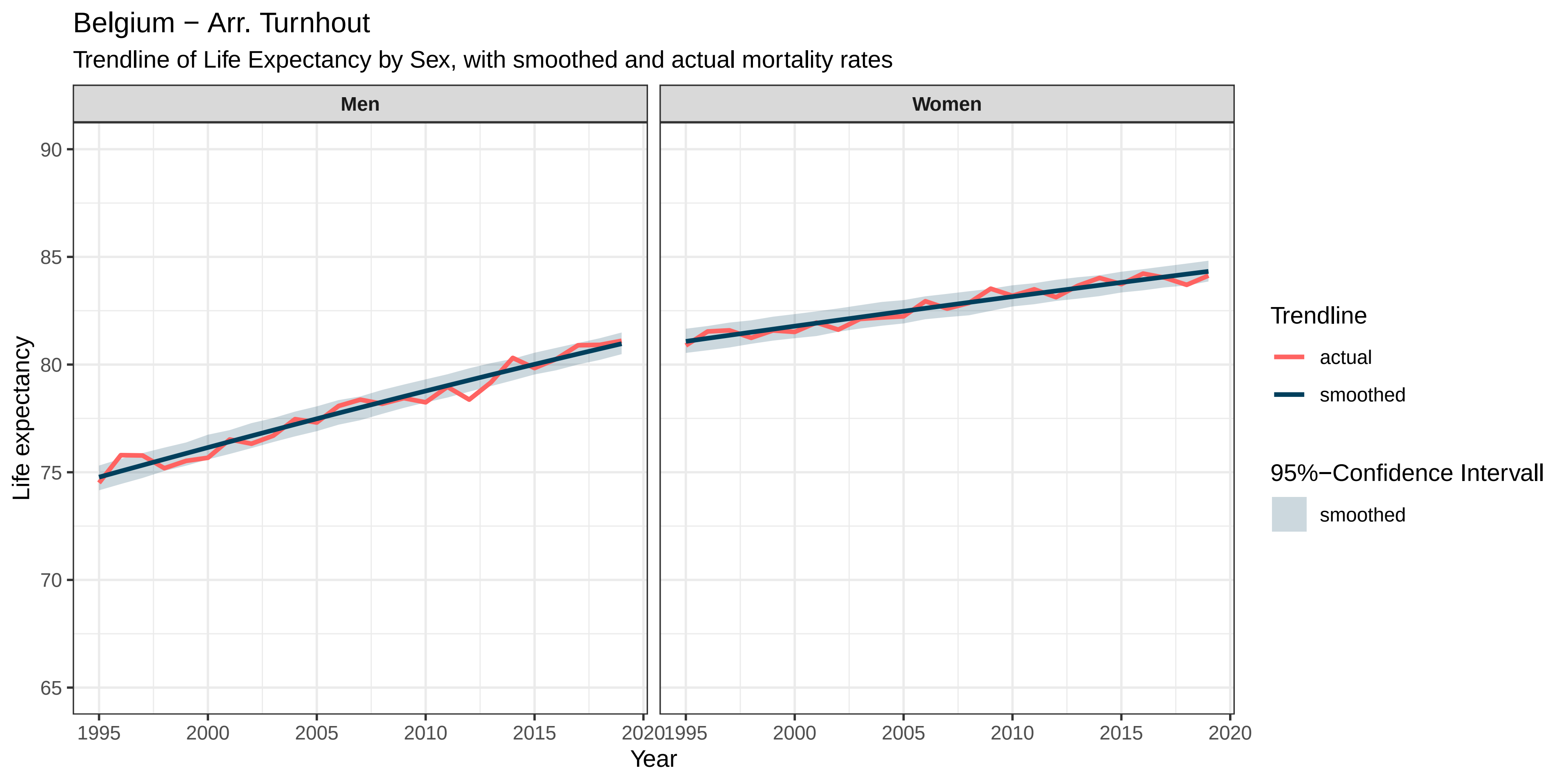
Belgium − Arr. Turnhout
Trendline of Life Expectancy by Sex, with smoothed and actual mortality rates
Men
Women
Life expectancy
Year
90
85
80
75
70
65
1995
2000
2005
2010
2015
2020
Trendline
actual
smoothed
95%−Confidence Intervall
smoothed

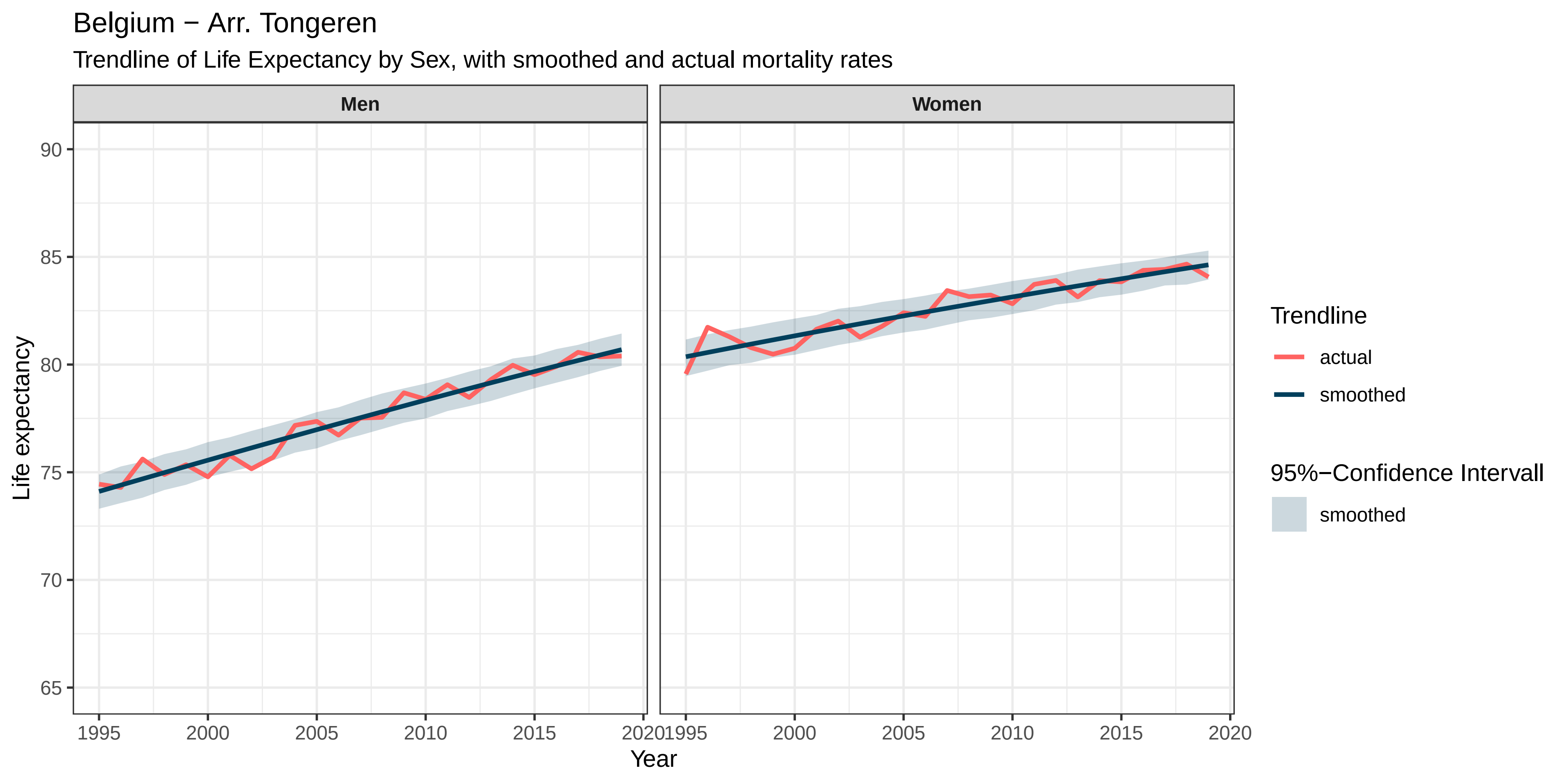
Belgium − Arr. Tongeren
Trendline of Life Expectancy by Sex, with smoothed and actual mortality rates
Men
Women
Life expectancy
90
85
80
75
70
65
1995
2000
2005
2010
2015
2020
Year
Trendline
actual
smoothed
95%−Confidence Intervall
smoothed

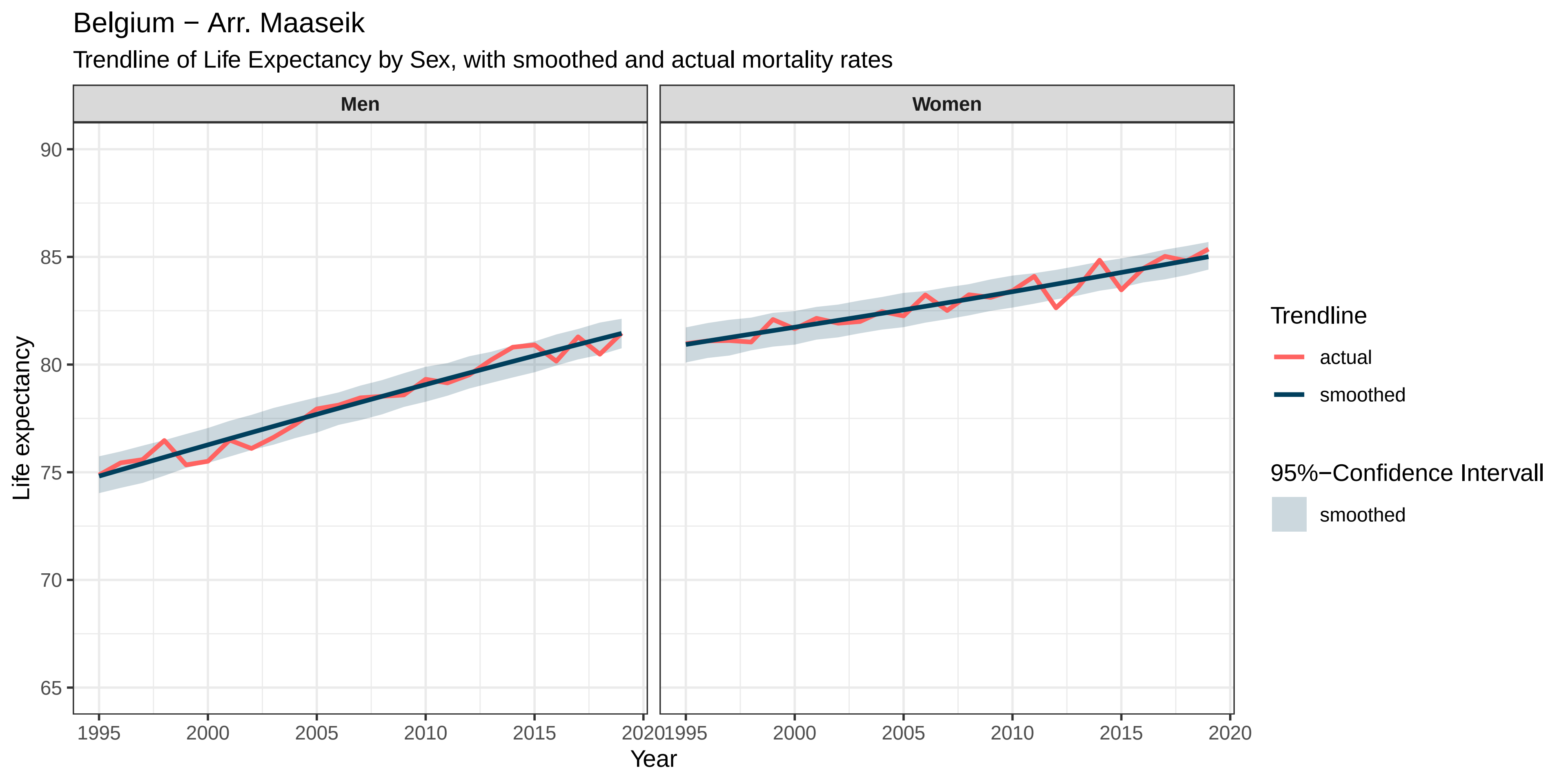
Belgium − Arr. Maaseik
Trendline of Life Expectancy by Sex, with smoothed and actual mortality rates
Men
Women
Life expectancy
90
85
80
75
70
65
1995
2000
2005
2010
2015
2020
Year
Trendline
actual
smoothed
95%−Confidence Intervall
smoothed

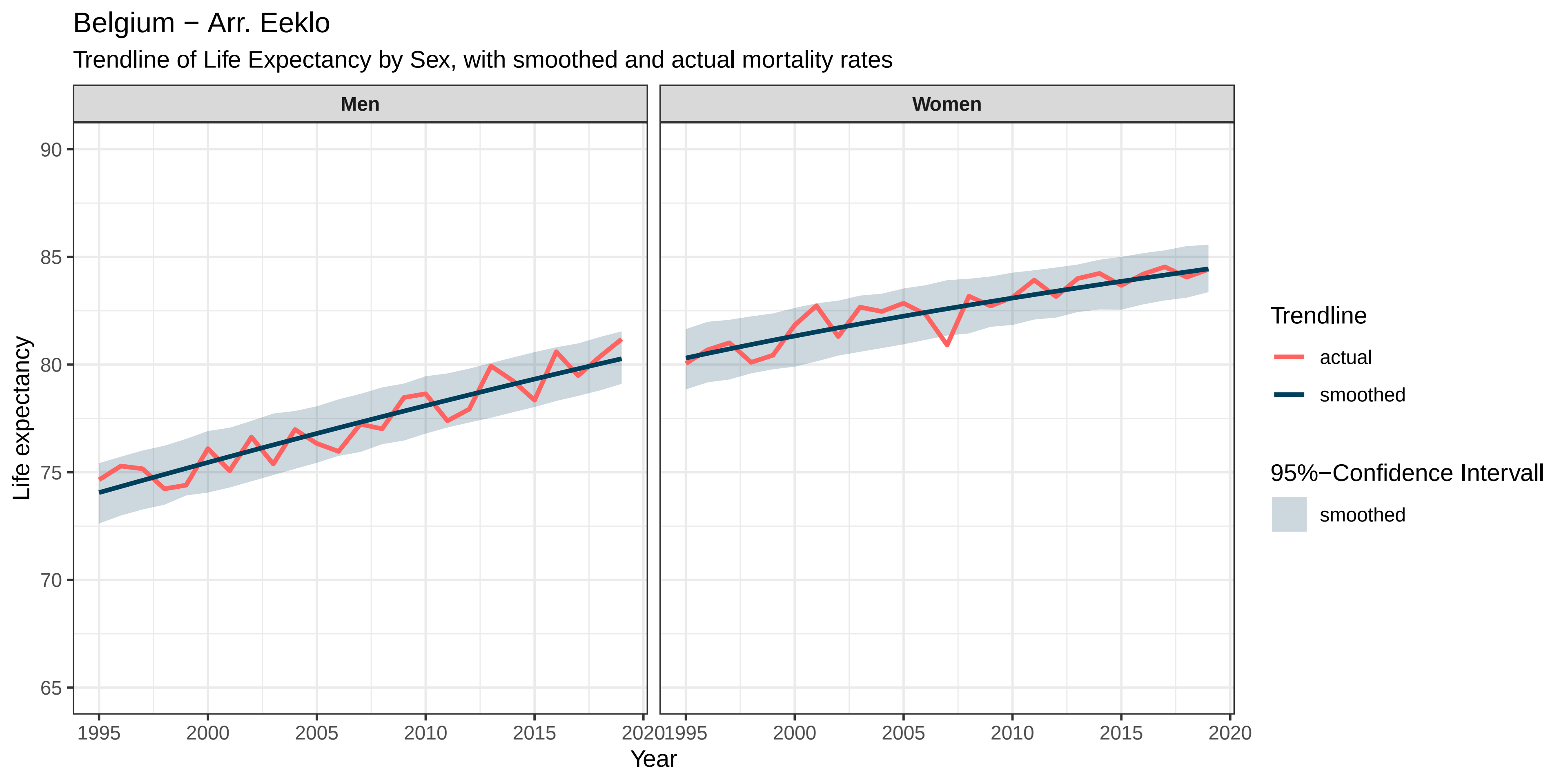
Belgium − Arr. Eeklo
Trendline of Life Expectancy by Sex, with smoothed and actual mortality rates
Men
Women
Life expectancy
Year
90
85
80
75
70
65
1995
2000
2005
2010
2015
2020
Trendline
actual
smoothed
95%−Confidence Intervall
smoothed

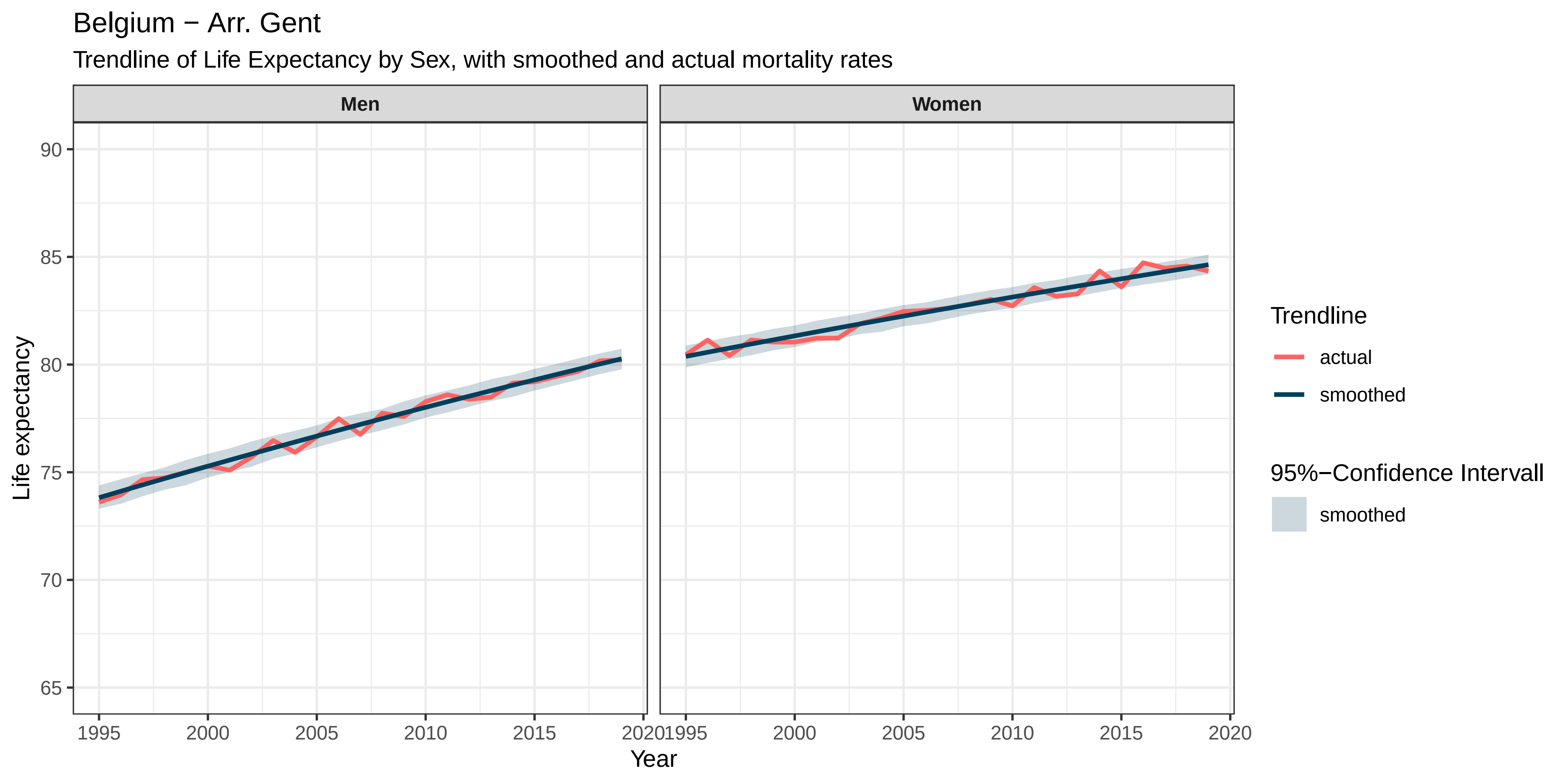

Belgium − Arr. Gent
Trendline of Life Expectancy by Sex, with smoothed and actual mortality rates
Men
Women
90
85
80
75
70
65
1995
2000
2005
2010
2015
2020
Year
Life expectancy
Trendline
actual
smoothed
95%−Confidence Intervall
smoothed

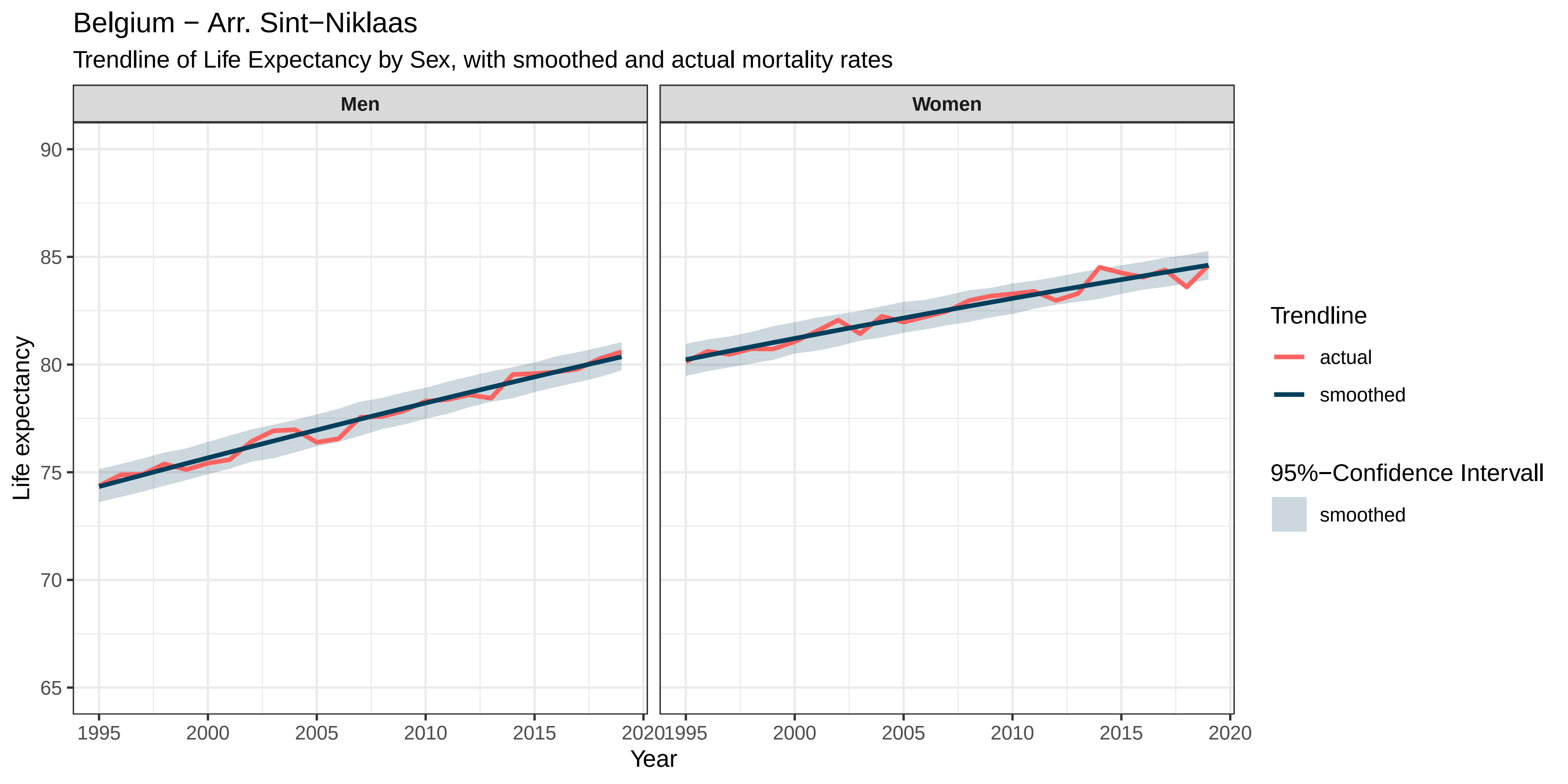

Belgium − Arr. Sint−Niklaas
Trendline of Life Expectancy by Sex, with smoothed and actual mortality rates
Men
Women
Life expectancy
Year
90
85
80
75
70
65
1995
2000
2005
2010
2015
2020
Trendline
actual
smoothed
95%−Confidence Intervall
smoothed

Belgium − Arr. Brugge

Trendline of Life Expectancy by Sex, with smoothed and actual mortality rates

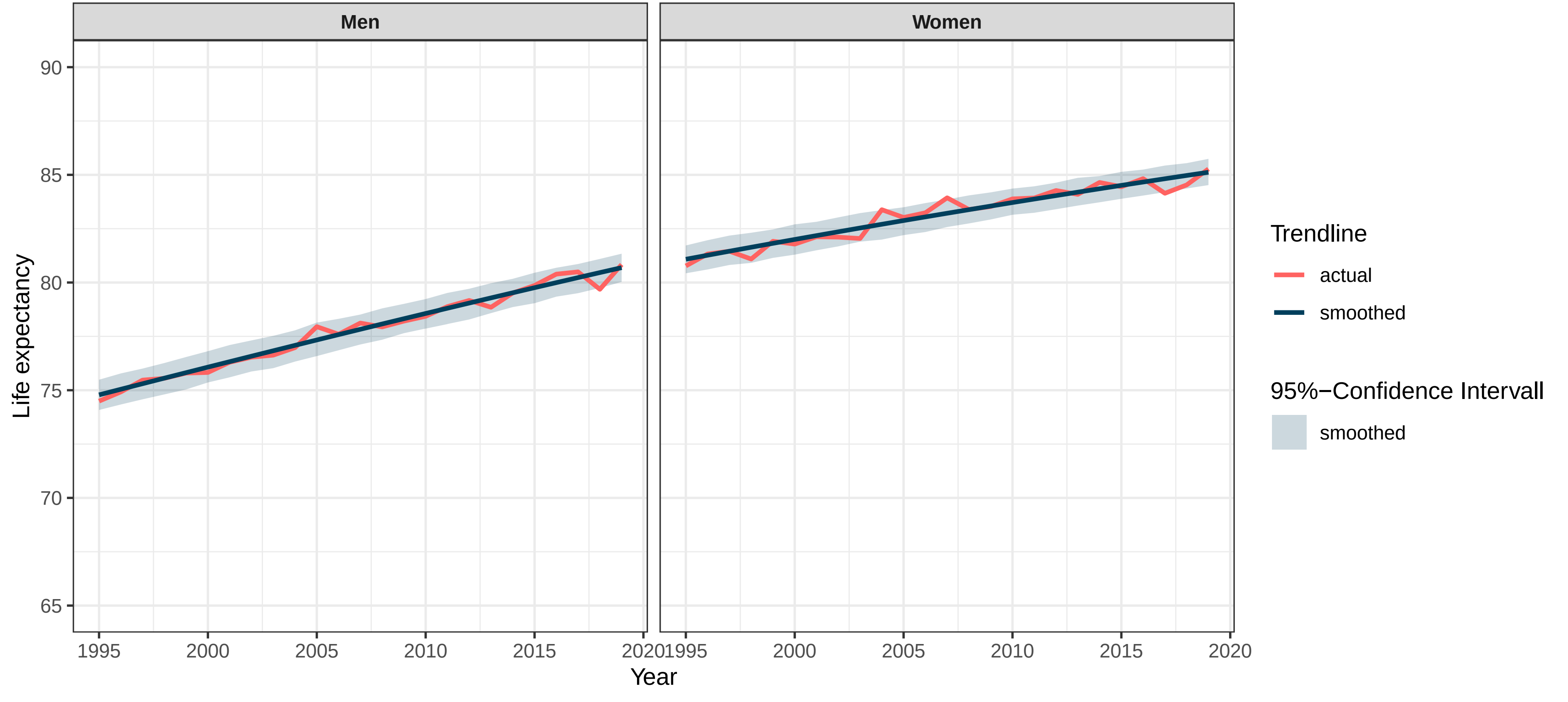

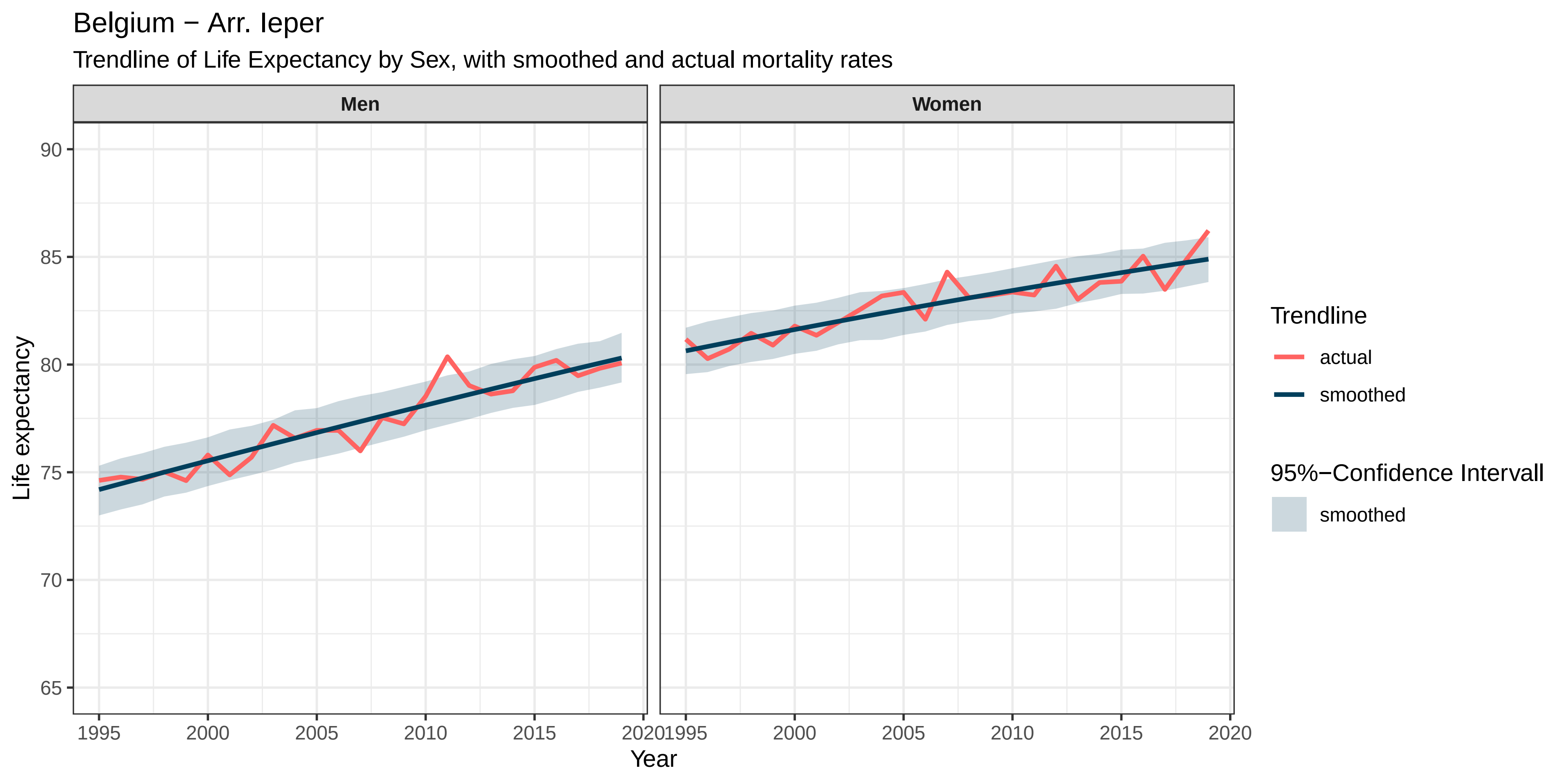

Belgium − Arr. Ieper
Trendline of Life Expectancy by Sex, with smoothed and actual mortality rates
Men
Women
Life expectancy
Year
65
70
75
80
85
90
1995
2000
2005
2010
2015
2020
Trendline
actual
smoothed
95%−Confidence Intervall
smoothed

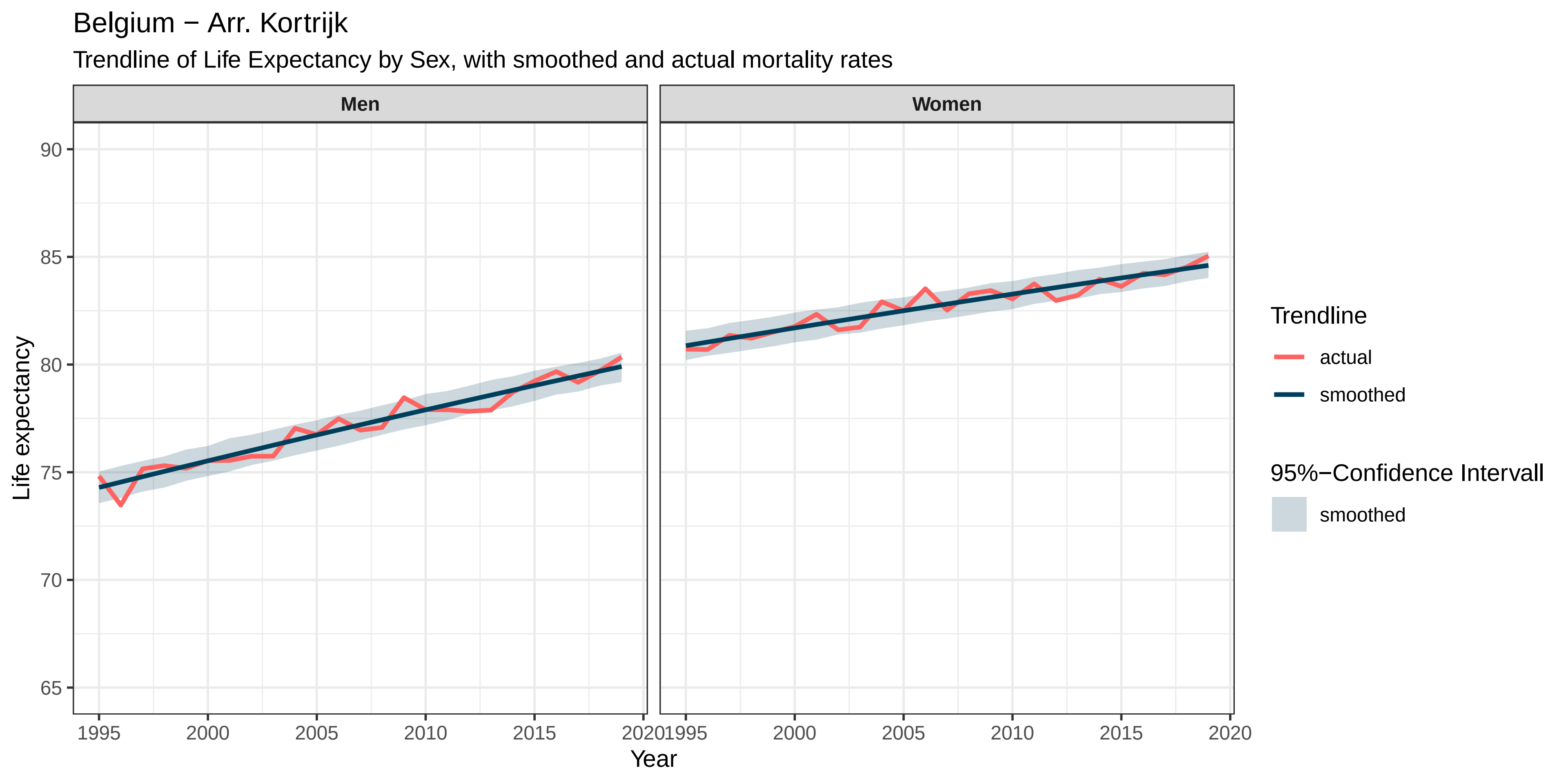
Belgium − Arr. Kortrijk
Trendline of Life Expectancy by Sex, with smoothed and actual mortality rates
Men
Women
Life expectancy
90
85
80
75
70
65
1995
2000
2005
2010
2015
2020
Year
Trendline
actual
smoothed
95%−Confidence Intervall
smoothed

Belgium − Arr. Veurne

Trendline of Life Expectancy by Sex, with smoothed and actual mortality rates

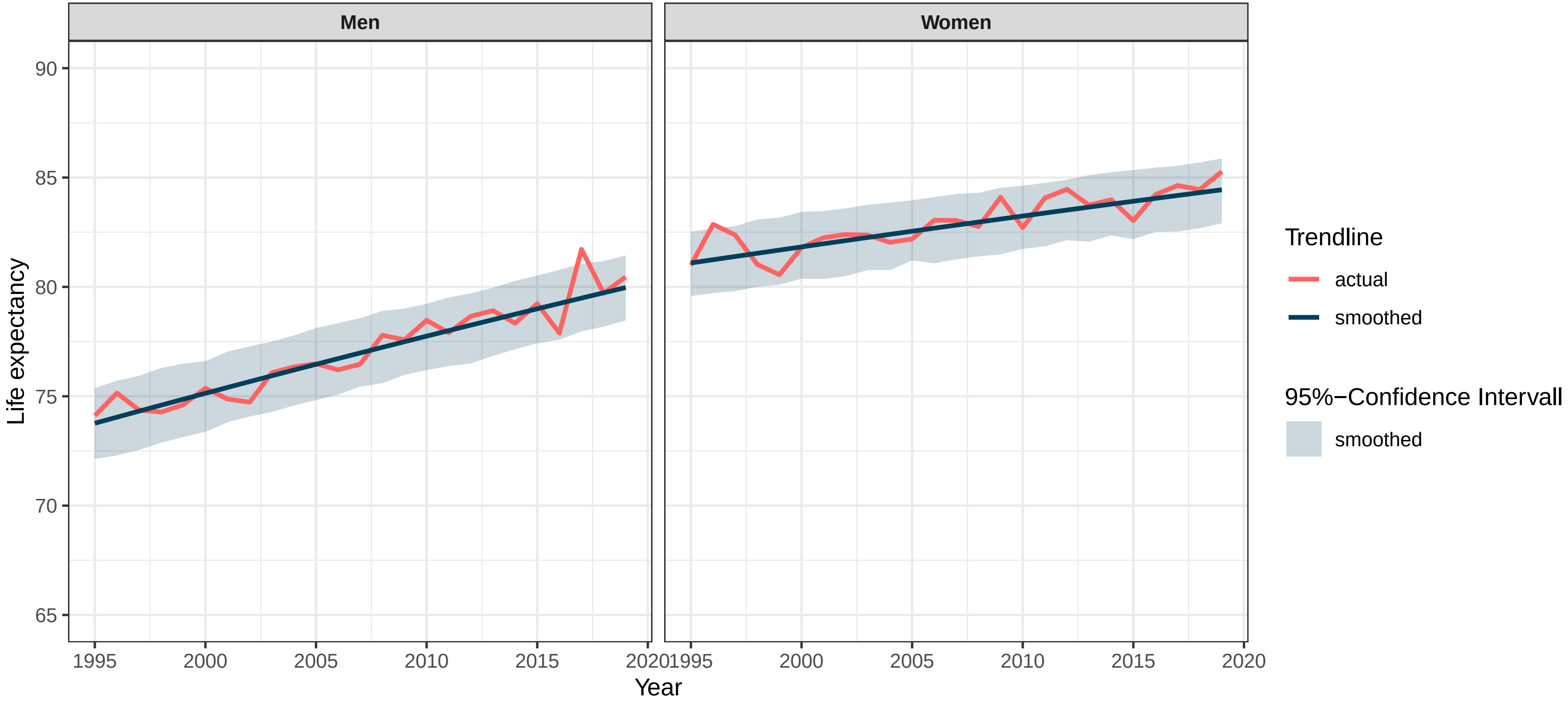

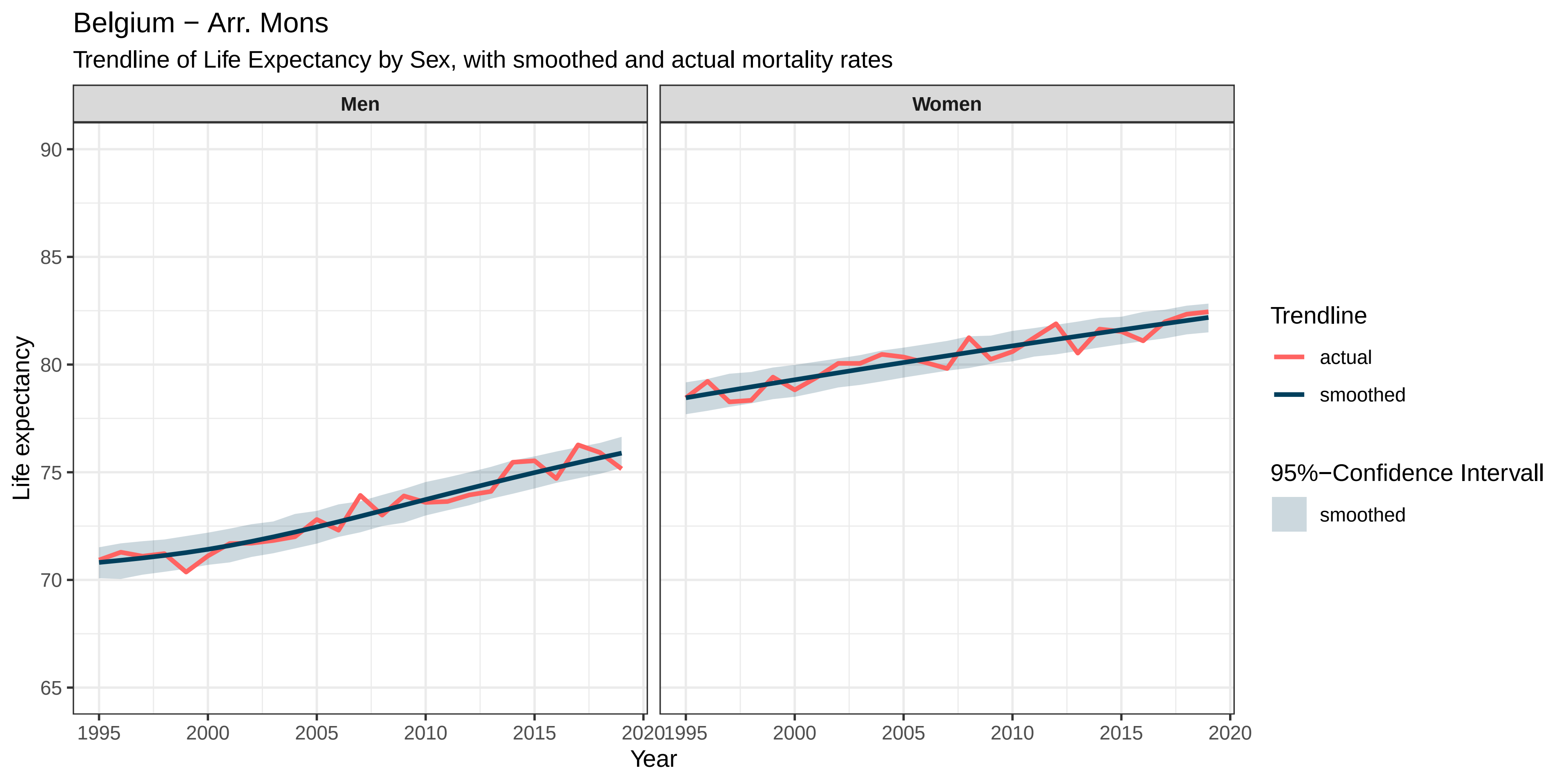
Belgium − Arr. Mons
Trendline of Life Expectancy by Sex, with smoothed and actual mortality rates
Men
Women
90
85
80
75
70
65
1995
2000
2005
2010
2015
2020
Year
Life expectancy
Trendline
actual
smoothed
95%−Confidence Intervall
smoothed

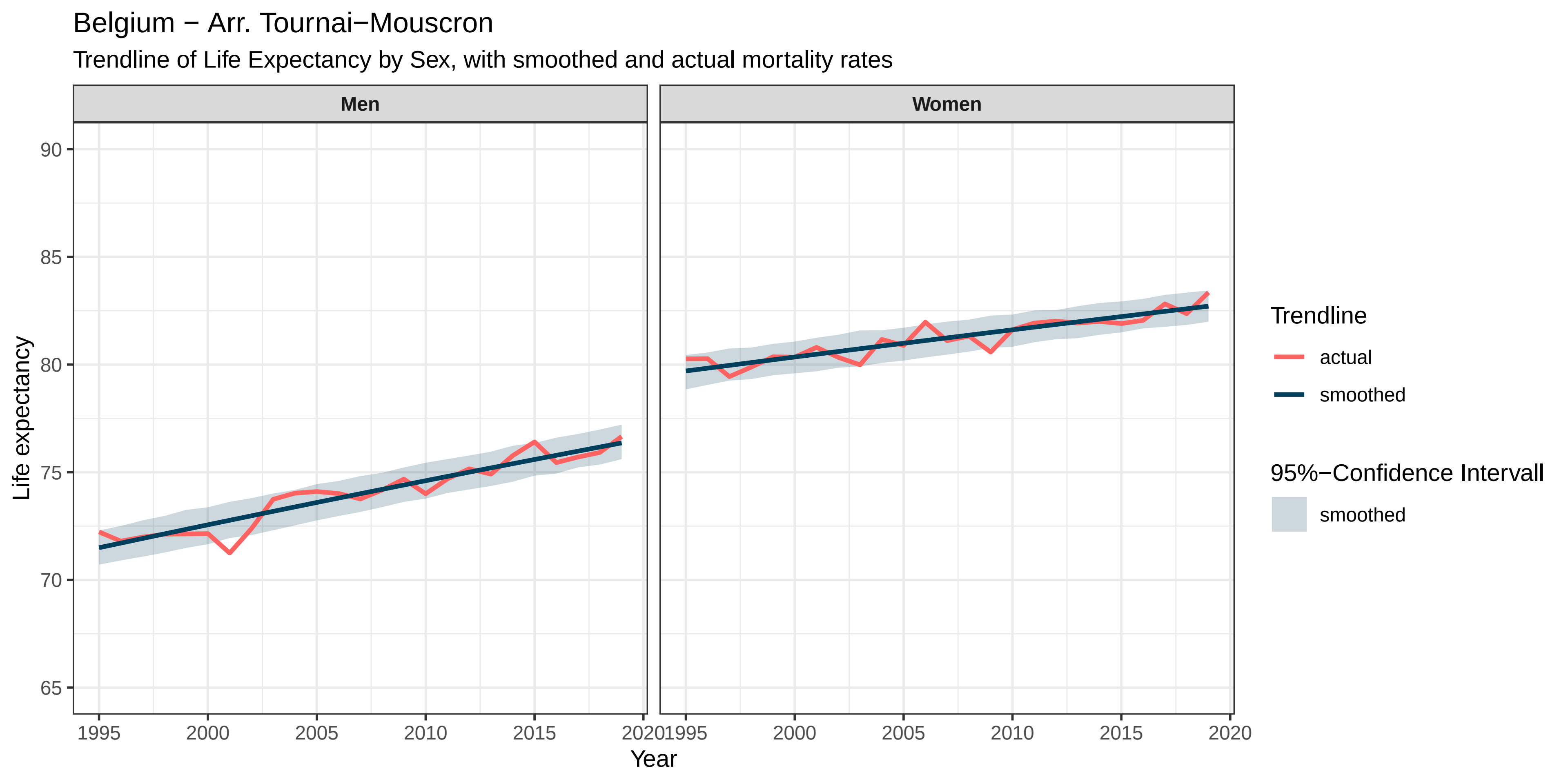

Belgium − Arr. Tournai−Mouscron
Trendline of Life Expectancy by Sex, with smoothed and actual mortality rates
Men
Women
Life expectancy
Year
90
85
80
75
70
65
1995
2000
2005
2010
2015
2020
Trendline
actual
smoothed
95%−Confidence Intervall
smoothed

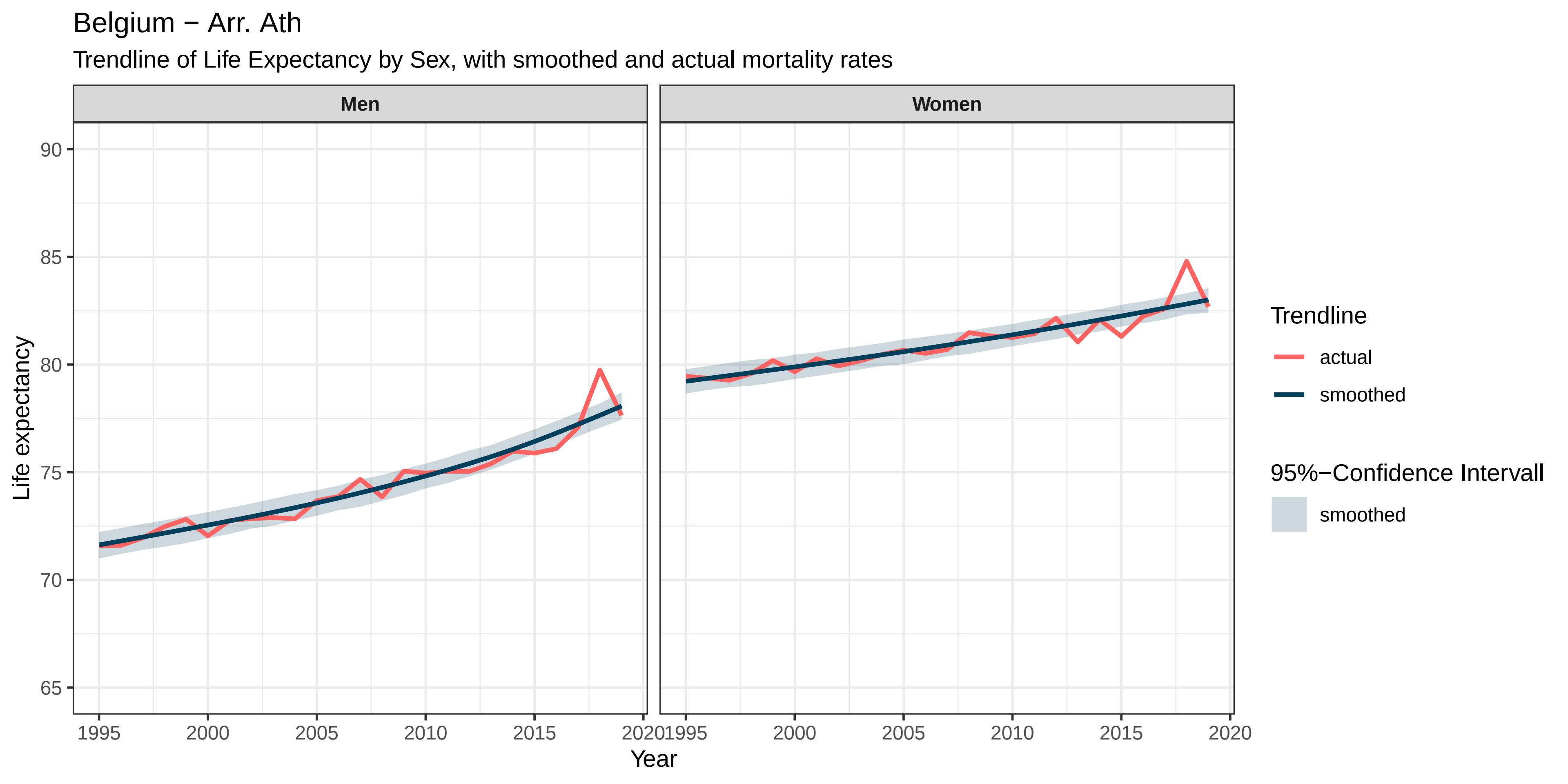

Belgium − Arr. Ath
Trendline of Life Expectancy by Sex, with smoothed and actual mortality rates
Men
Women
90
85
80
75
70
65
Life expectancy
1995
2000
2005
2010
2015
2020
Year
Trendline
actual
smoothed
95%−Confidence Intervall
smoothed

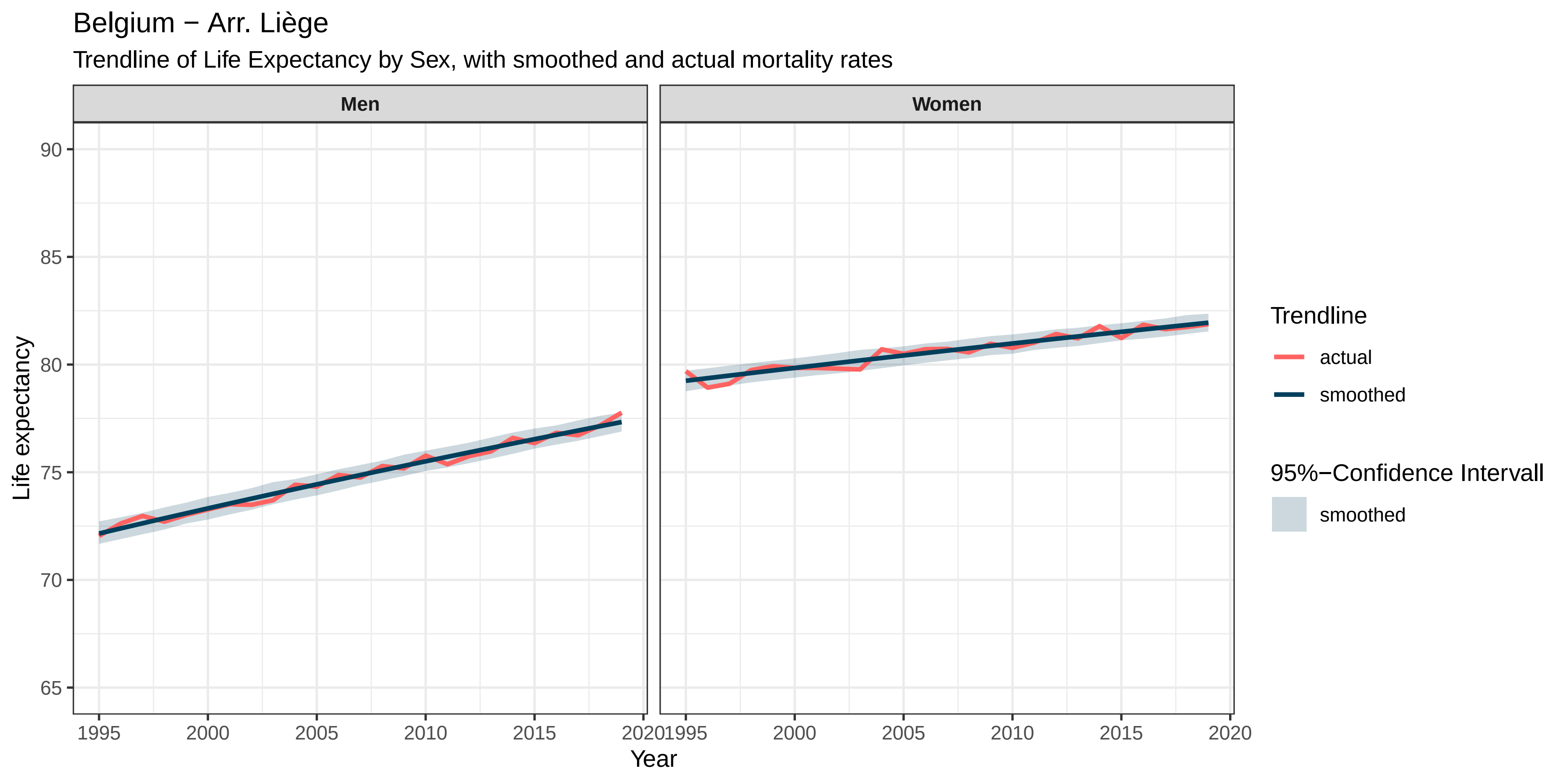

Belgium − Arr. Liège
Trendline of Life Expectancy by Sex, with smoothed and actual mortality rates
Men
Women
90
85
80
75
70
65
Life expectancy
1995
2000
2005
2010
2015
2020
Year
Trendline
actual
smoothed
95%−Confidence Intervall
smoothed

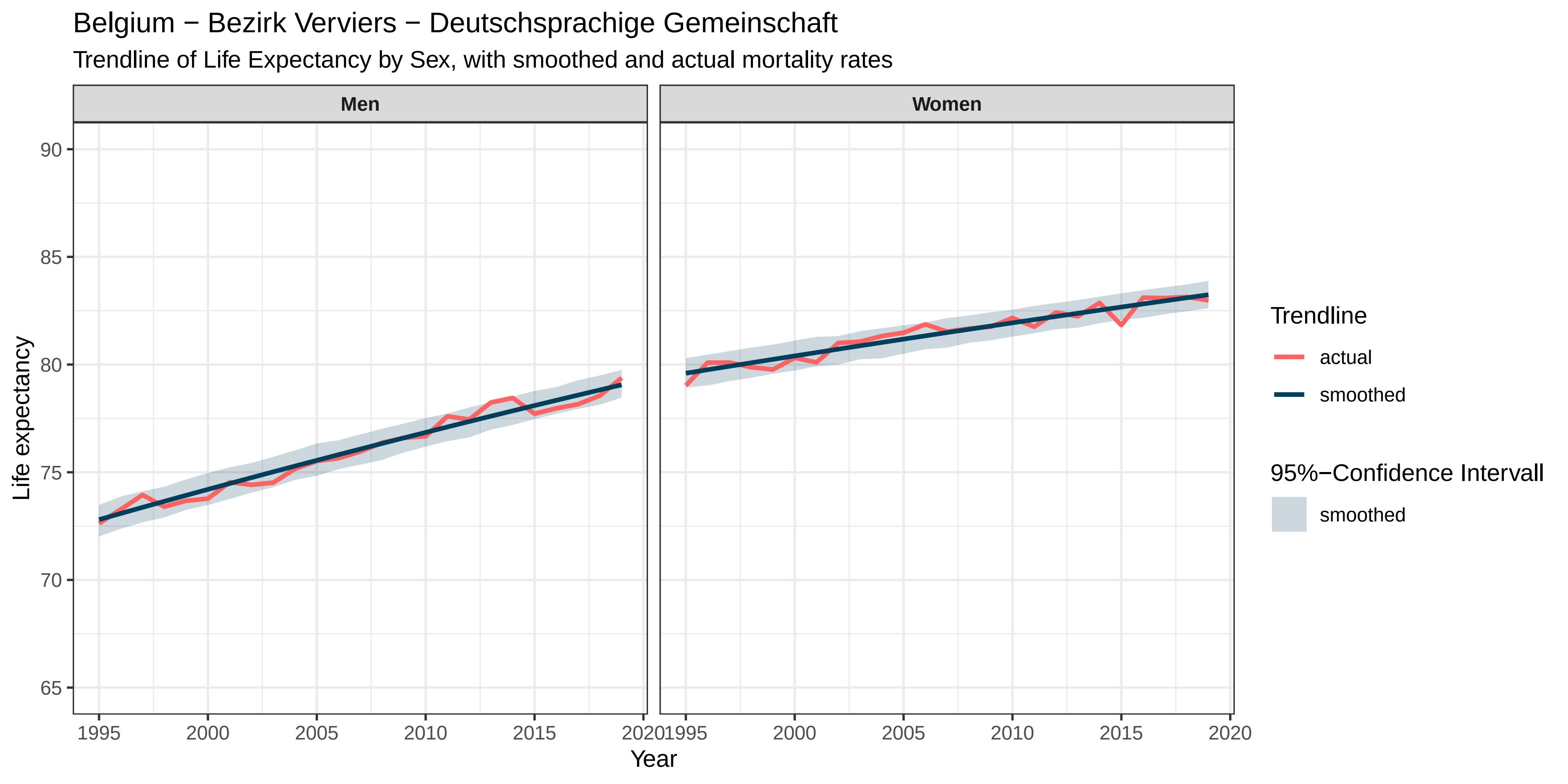
Belgium − Bezirk Verviers − Deutschsprachige Gemeinschaft
Trendline of Life Expectancy by Sex, with smoothed and actual mortality rates
Men
Women
Life expectancy
Year
90
85
80
75
70
65
1995
2000
2005
2010
2015
2020
Trendline
actual
smoothed
95%−Confidence Intervall
smoothed

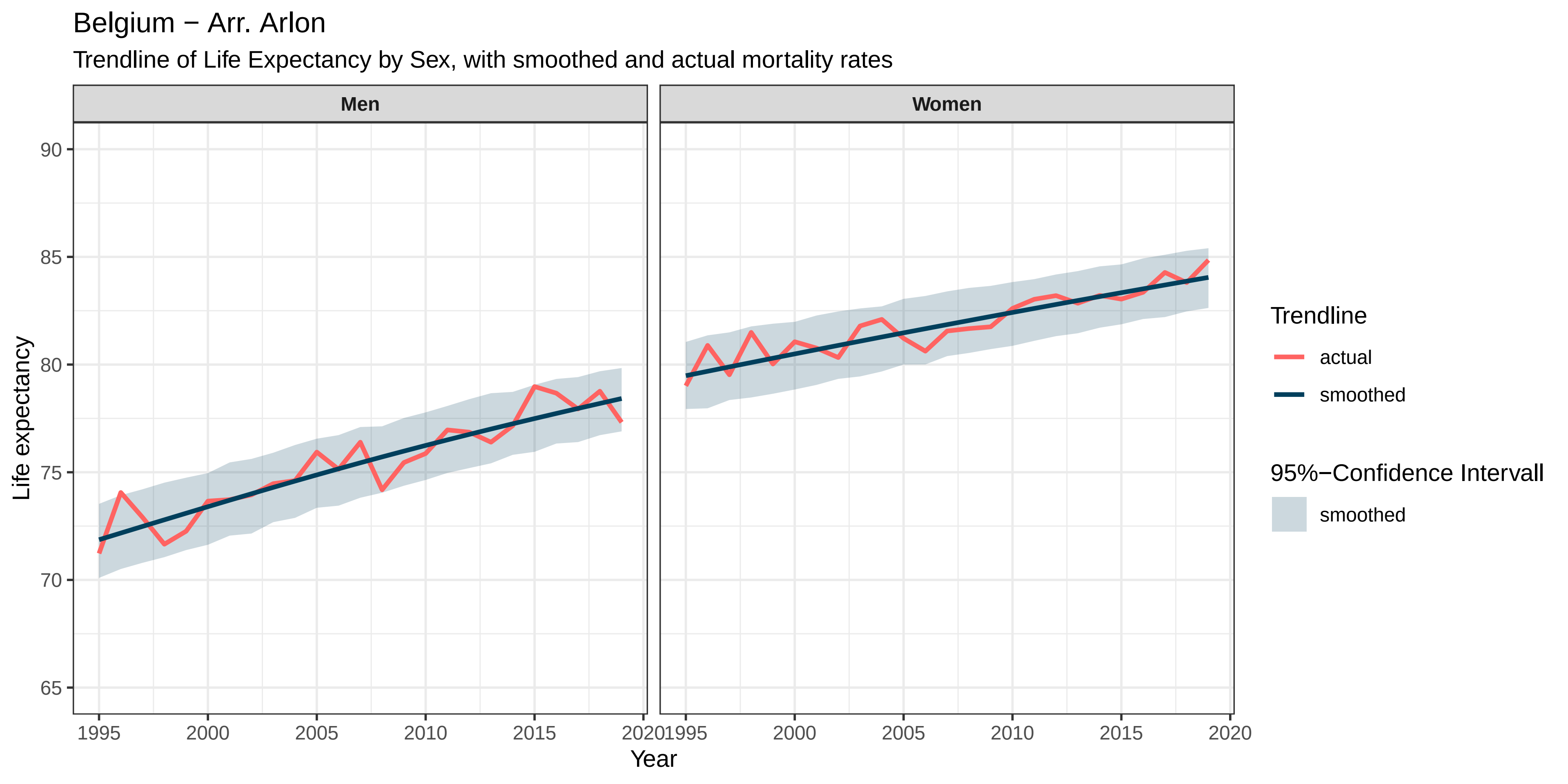

Belgium − Arr. Arlon
Trendline of Life Expectancy by Sex, with smoothed and actual mortality rates
Men
Women
90
85
80
75
70
65
Life expectancy
1995
2000
2005
2010
2015
2020
Year
Trendline
actual
smoothed
95%−Confidence Intervall
smoothed

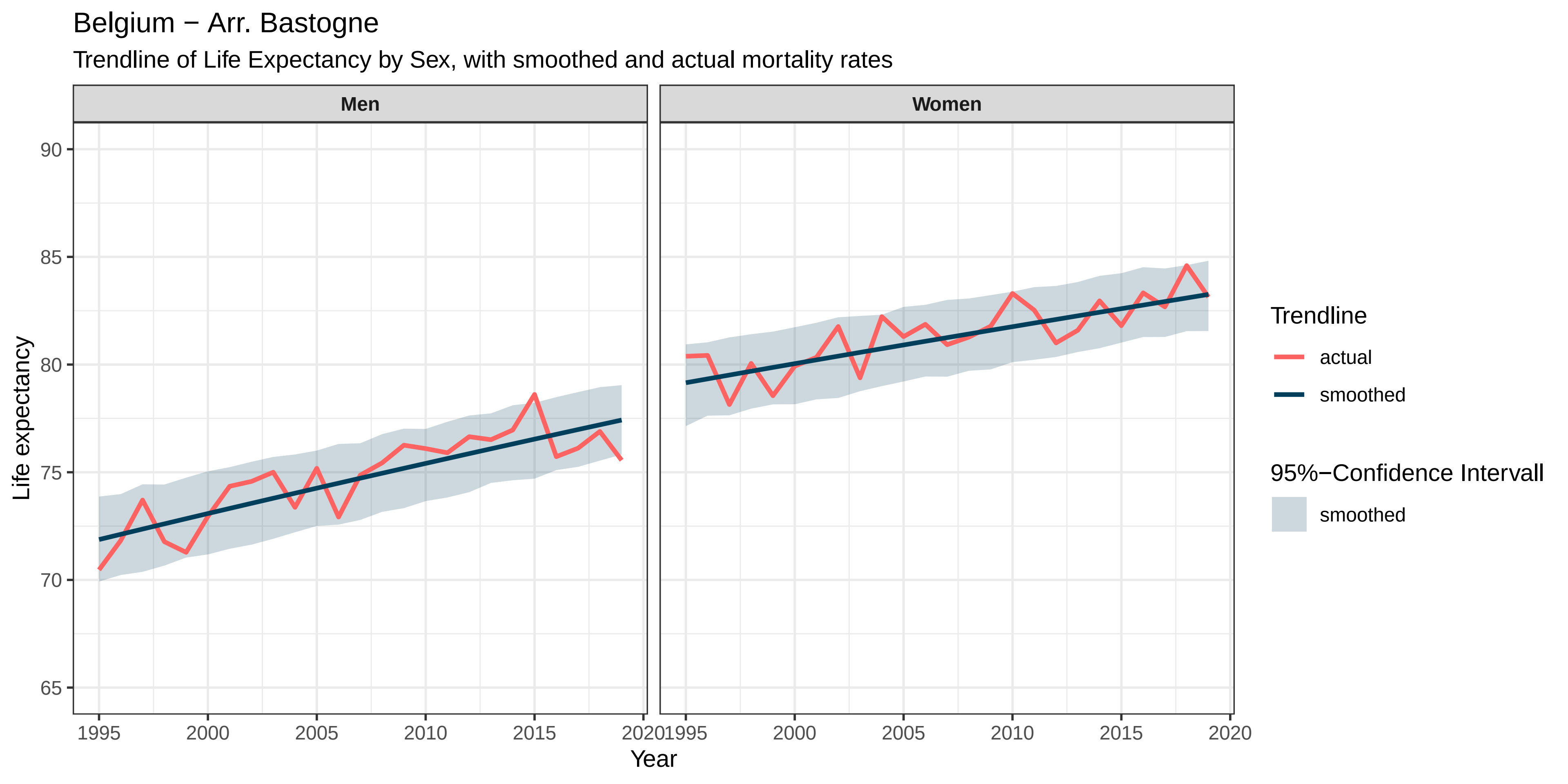

Belgium − Arr. Bastogne
Trendline of Life Expectancy by Sex, with smoothed and actual mortality rates
Men
Women
Life expectancy
90
85
80
75
70
65
1995
2000
2005
2010
2015
2020
Year
Trendline
actual
smoothed
95%−Confidence Intervall
smoothed

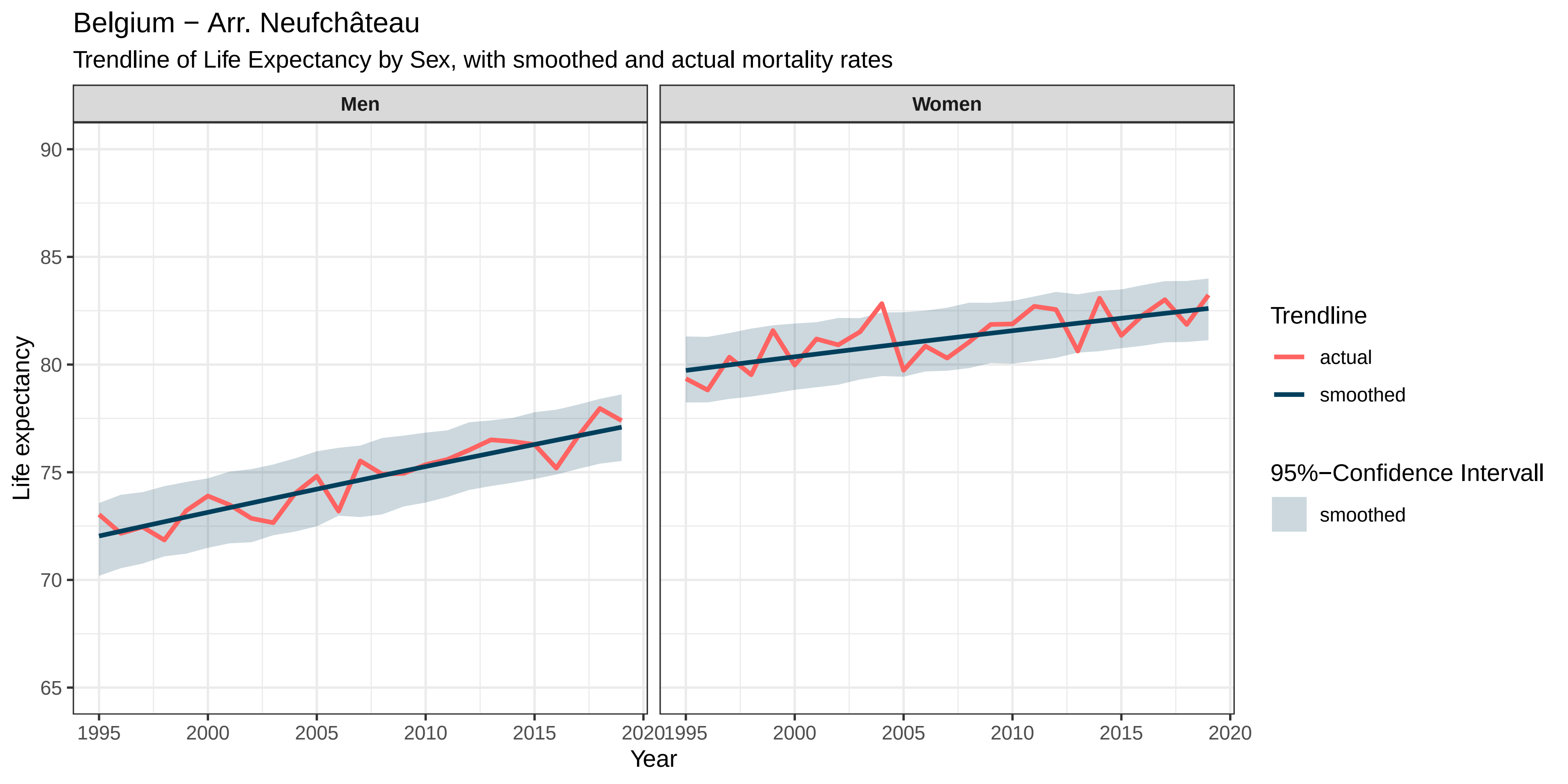

Belgium − Arr. Neufchâteau
Trendline of Life Expectancy by Sex, with smoothed and actual mortality rates
Men
Women
90
85
80
75
70
65
Life expectancy
1995
2000
2005
2010
2015
2020
Year
Trendline
actual
smoothed
95%−Confidence Intervall
smoothed

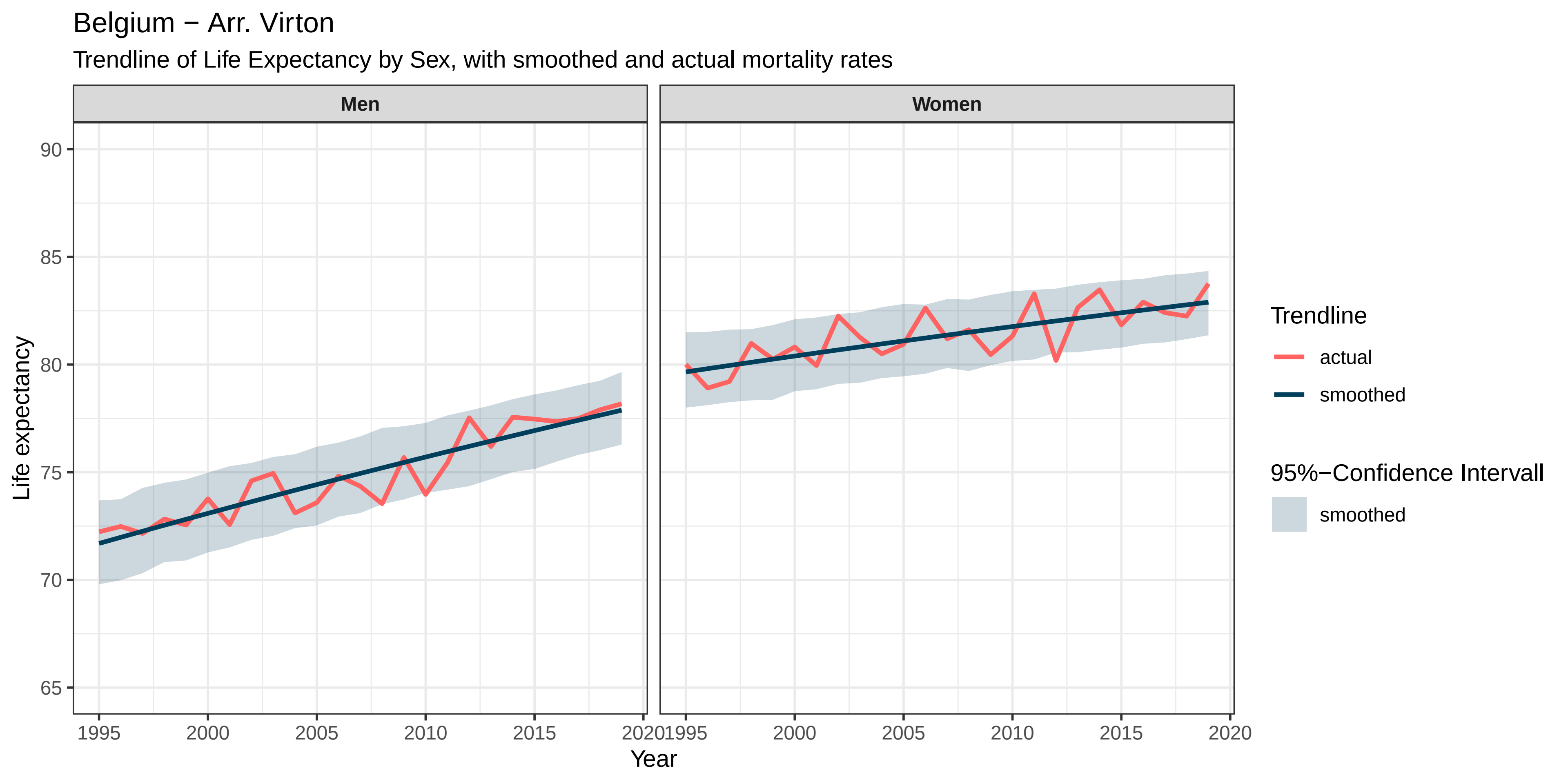

Belgium − Arr. Virton
Trendline of Life Expectancy by Sex, with smoothed and actual mortality rates
Men
Women
90
85
80
75
70
65
Life expectancy
1995
2000
2005
2010
2015
2020
Year
Trendline
actual
smoothed
95%−Confidence Intervall
smoothed

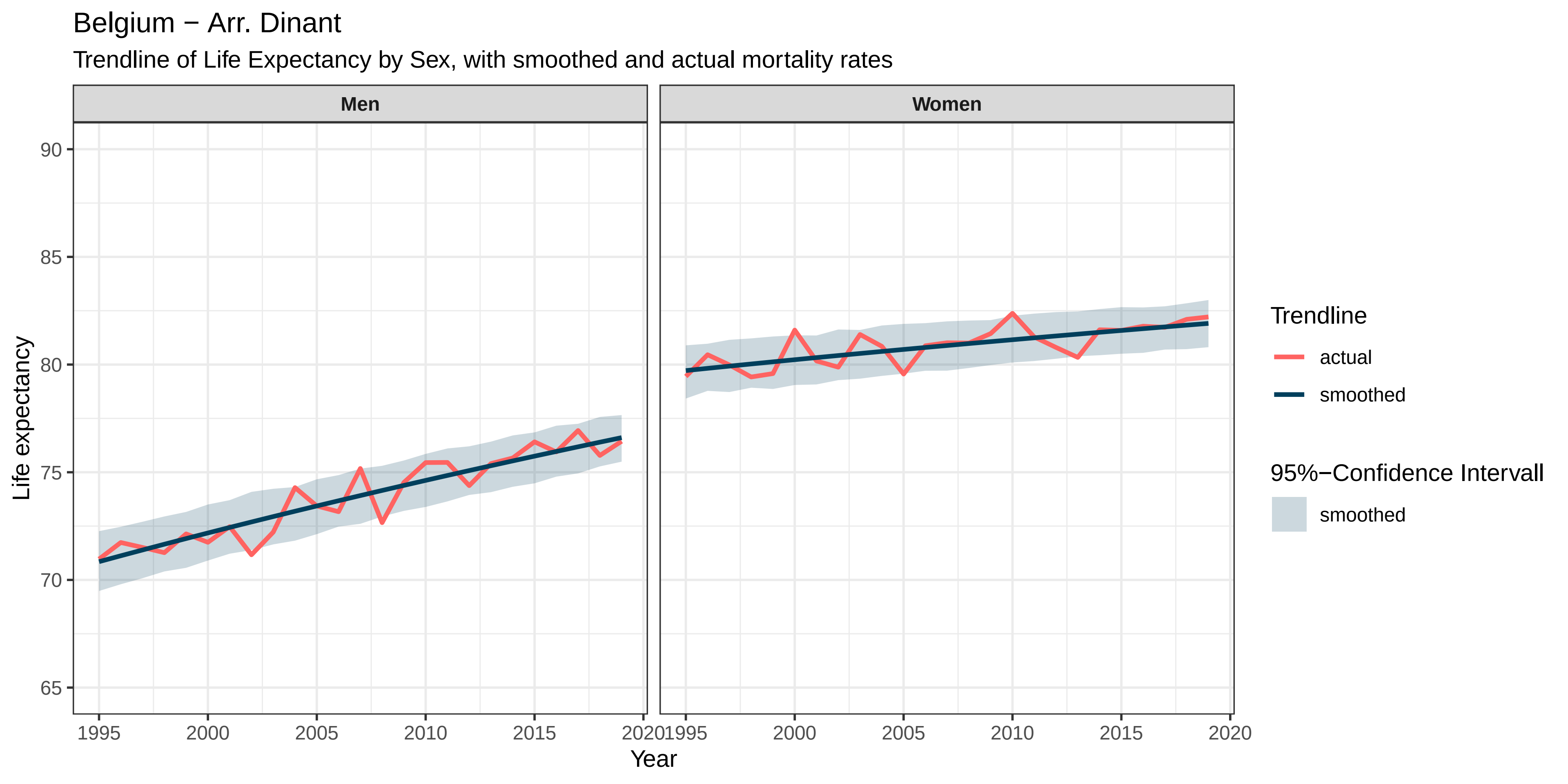

Belgium − Arr. Dinant
Trendline of Life Expectancy by Sex, with smoothed and actual mortality rates
Men
Women
Life expectancy
90
85
80
75
70
65
1995
2000
2005
2010
2015
2020
Year
Trendline
actual
smoothed
95%−Confidence Intervall
smoothed

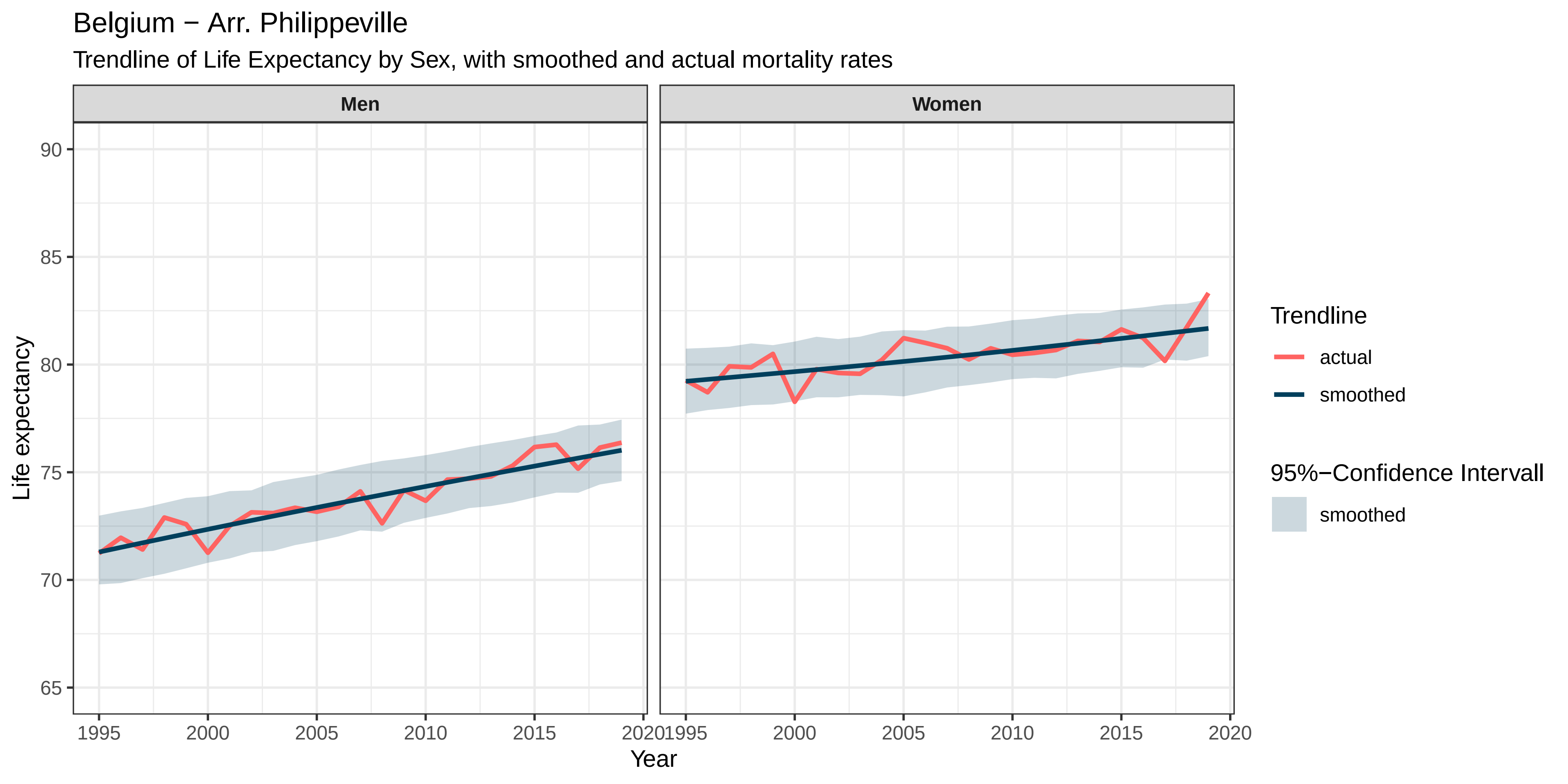

Belgium − Arr. Philippeville
Trendline of Life Expectancy by Sex, with smoothed and actual mortality rates
Men
Women
Life expectancy
90
85
80
75
70
65
1995
2000
2005
2010
2015
2020
Year
Trendline
actual
smoothed
95%−Confidence Intervall
smoothed

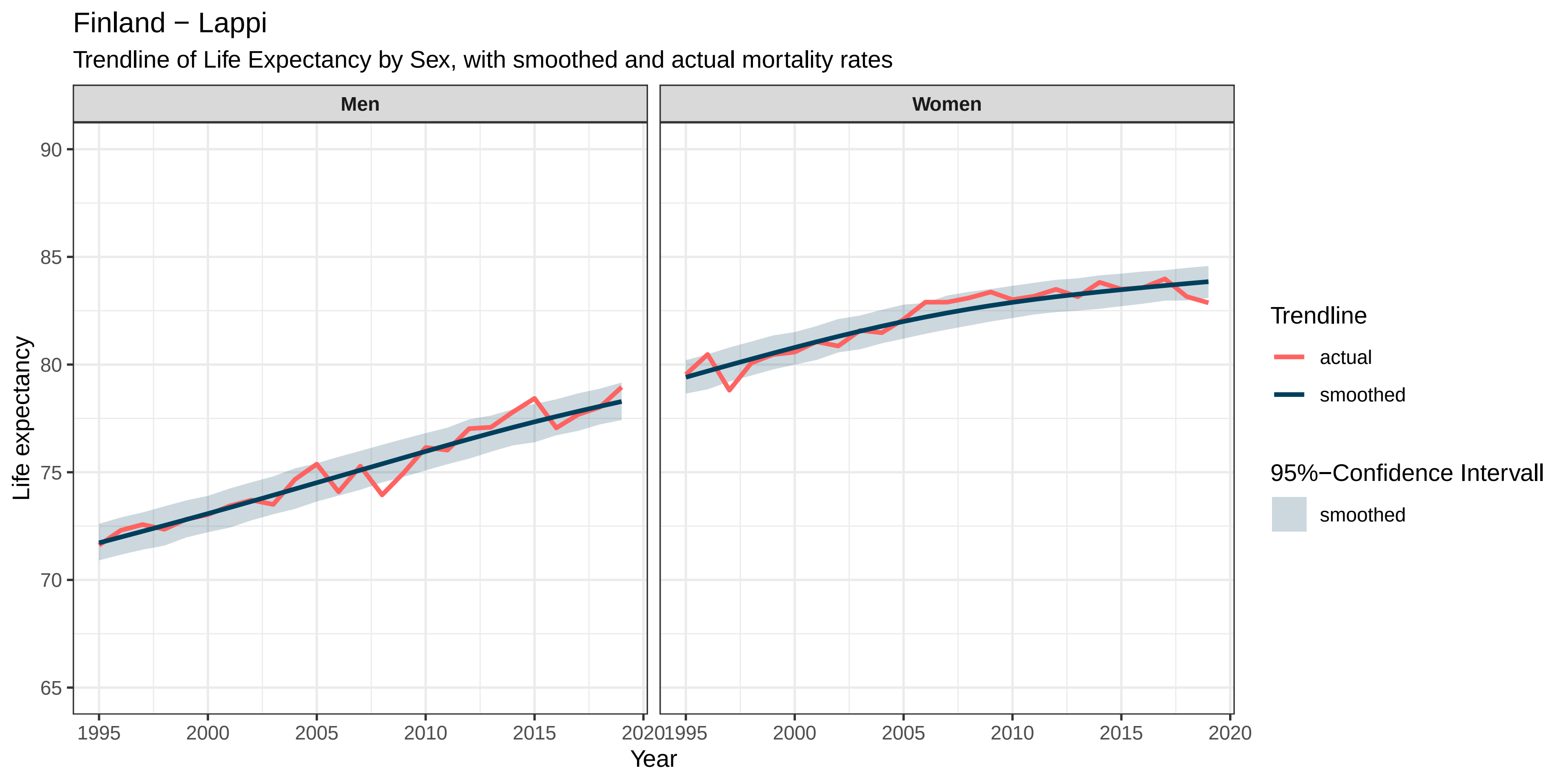
Finland − Lappi
Trendline of Life Expectancy by Sex, with smoothed and actual mortality rates
Men
Women
Life expectancy
90
85
80
75
70
65
1995
2000
2005
2010
2015
2020
Year
Trendline
actual
smoothed
95%−Confidence Intervall
smoothed

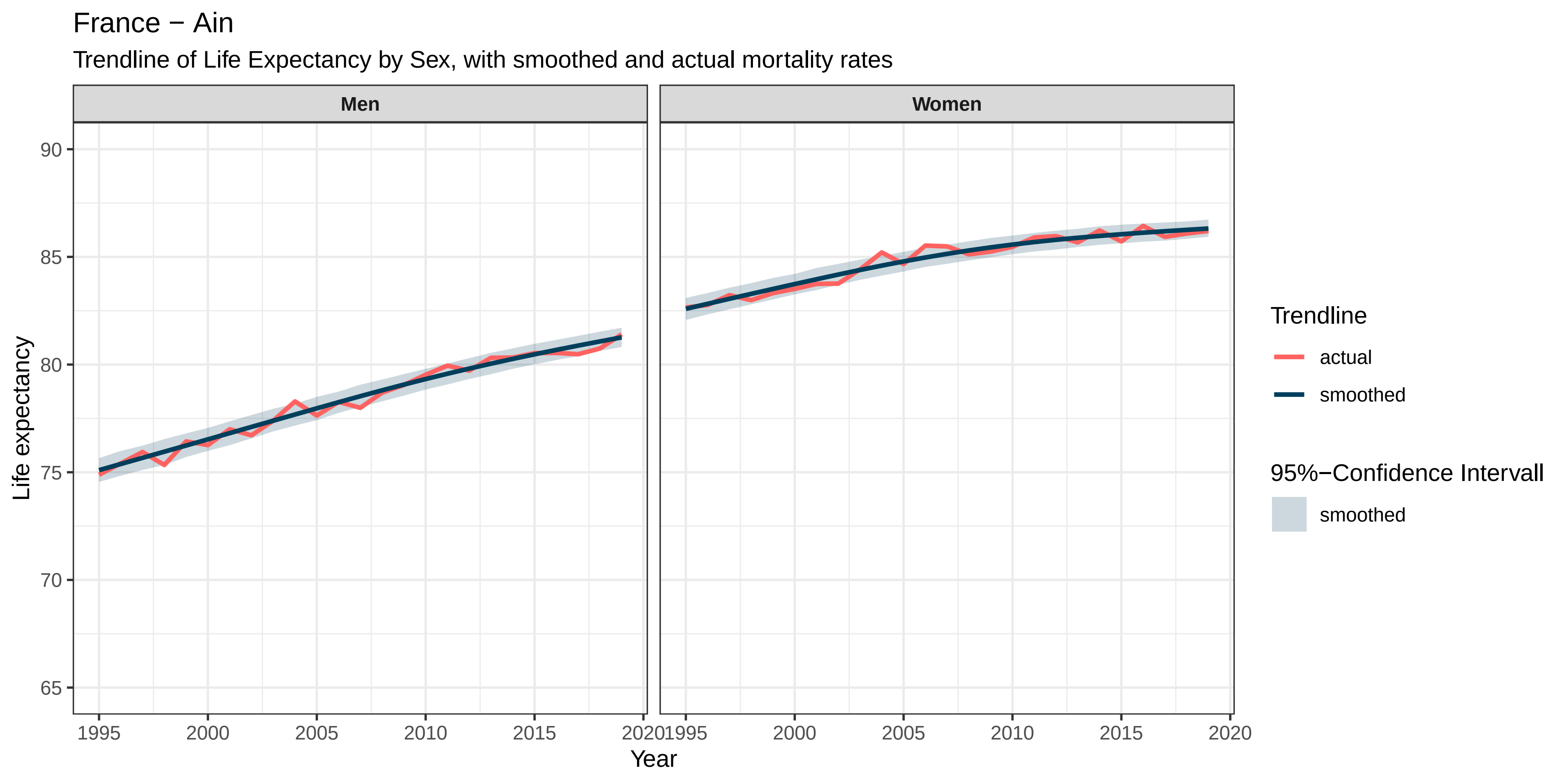

France − Ain
Trendline of Life Expectancy by Sex, with smoothed and actual mortality rates
Men
Women
Life expectancy
90
85
80
75
70
65
1995
2000
2005
2010
2015
2020
Year
Trendline
actual
smoothed
95%−Confidence Intervall
smoothed

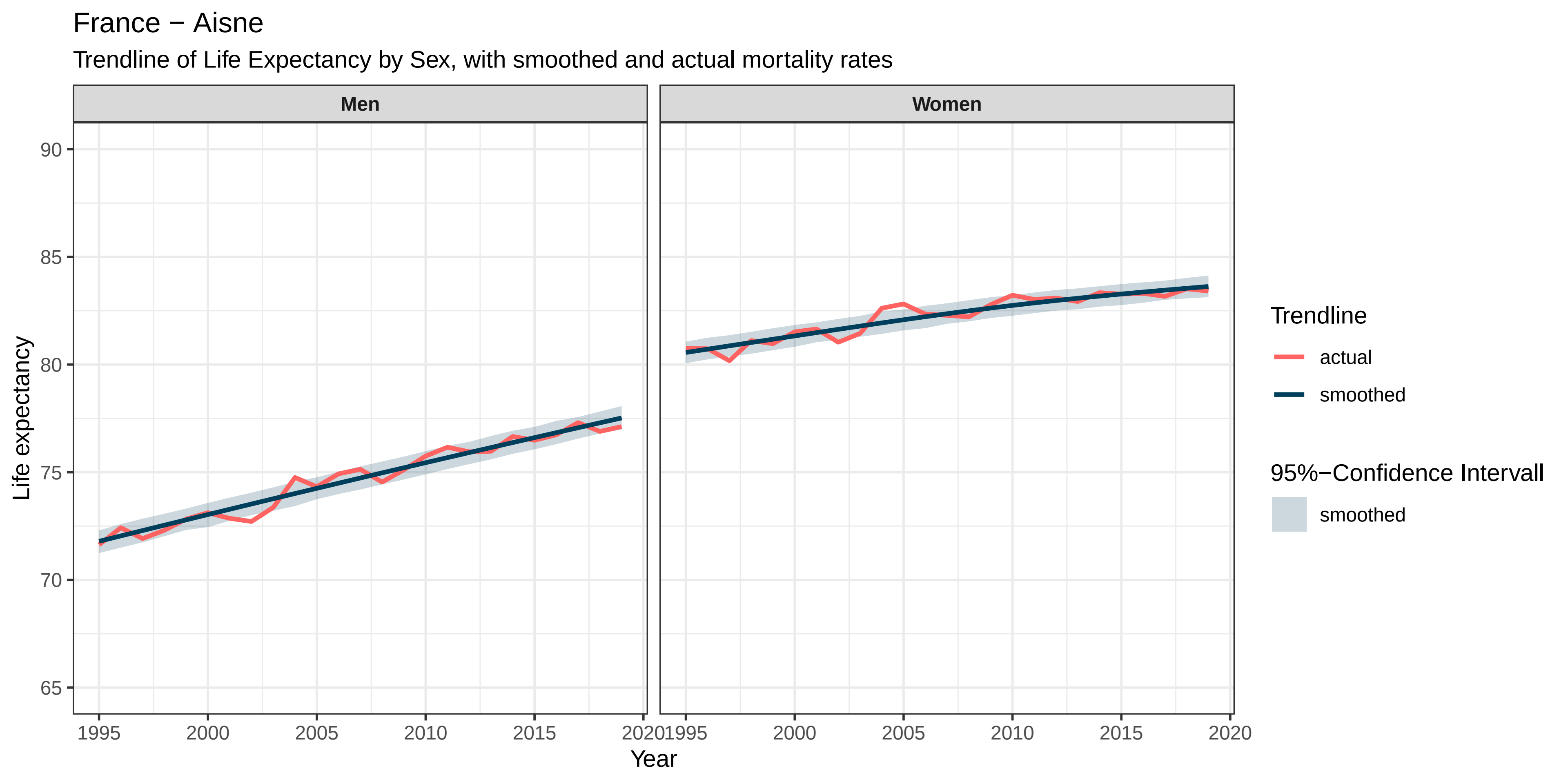
France − Aisne
Trendline of Life Expectancy by Sex, with smoothed and actual mortality rates
Men
Women
Life expectancy
90
85
80
75
70
65
1995
2000
2005
2010
2015
2020
Year
Trendline
actual
smoothed
95%−Confidence Intervall
smoothed

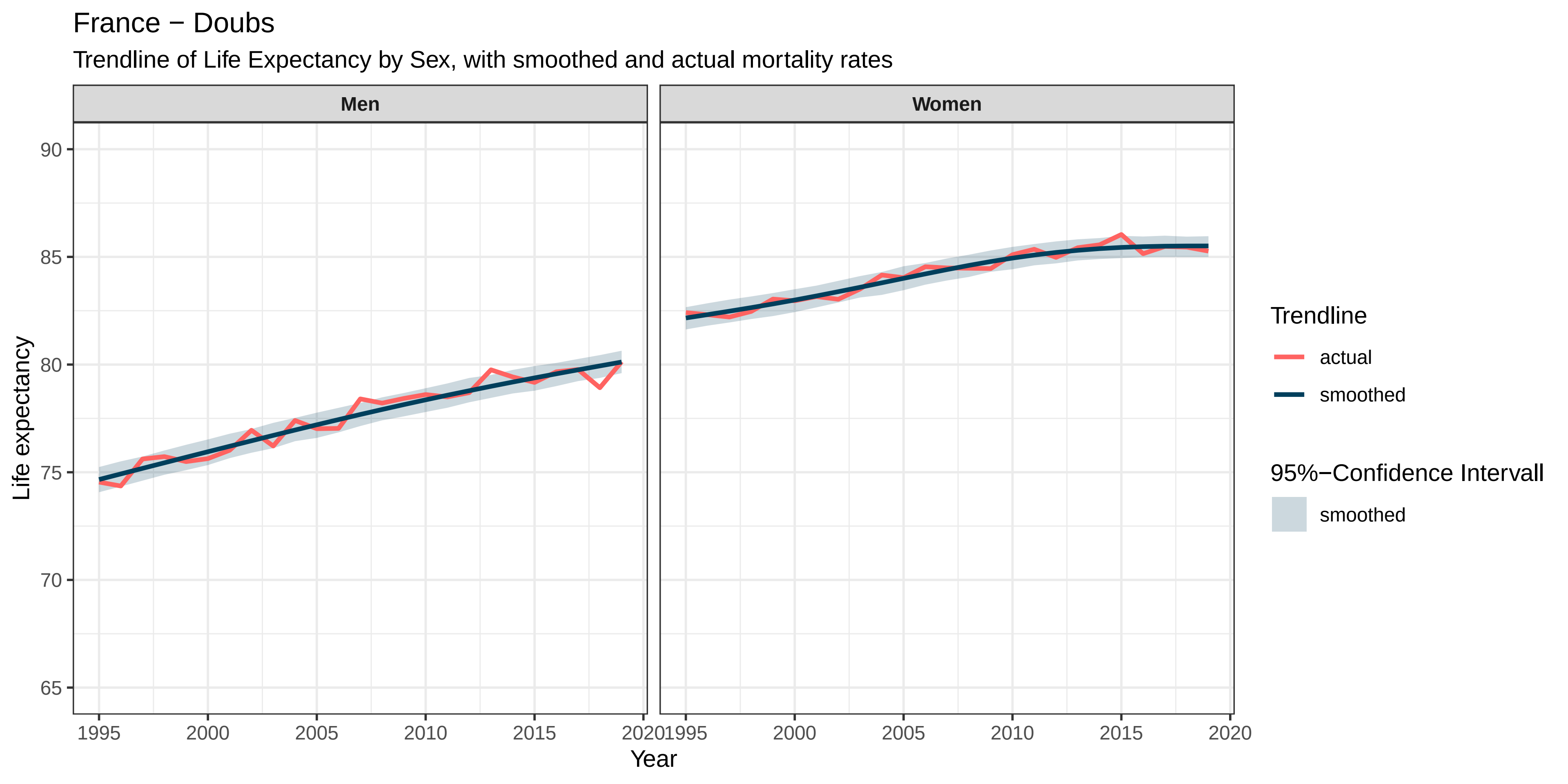
France − Doubs
Trendline of Life Expectancy by Sex, with smoothed and actual mortality rates
Men
Women
Life expectancy
90
85
80
75
70
65
1995
2000
2005
2010
2015
2020
Year
Trendline
actual
smoothed
95%−Confidence Intervall
smoothed

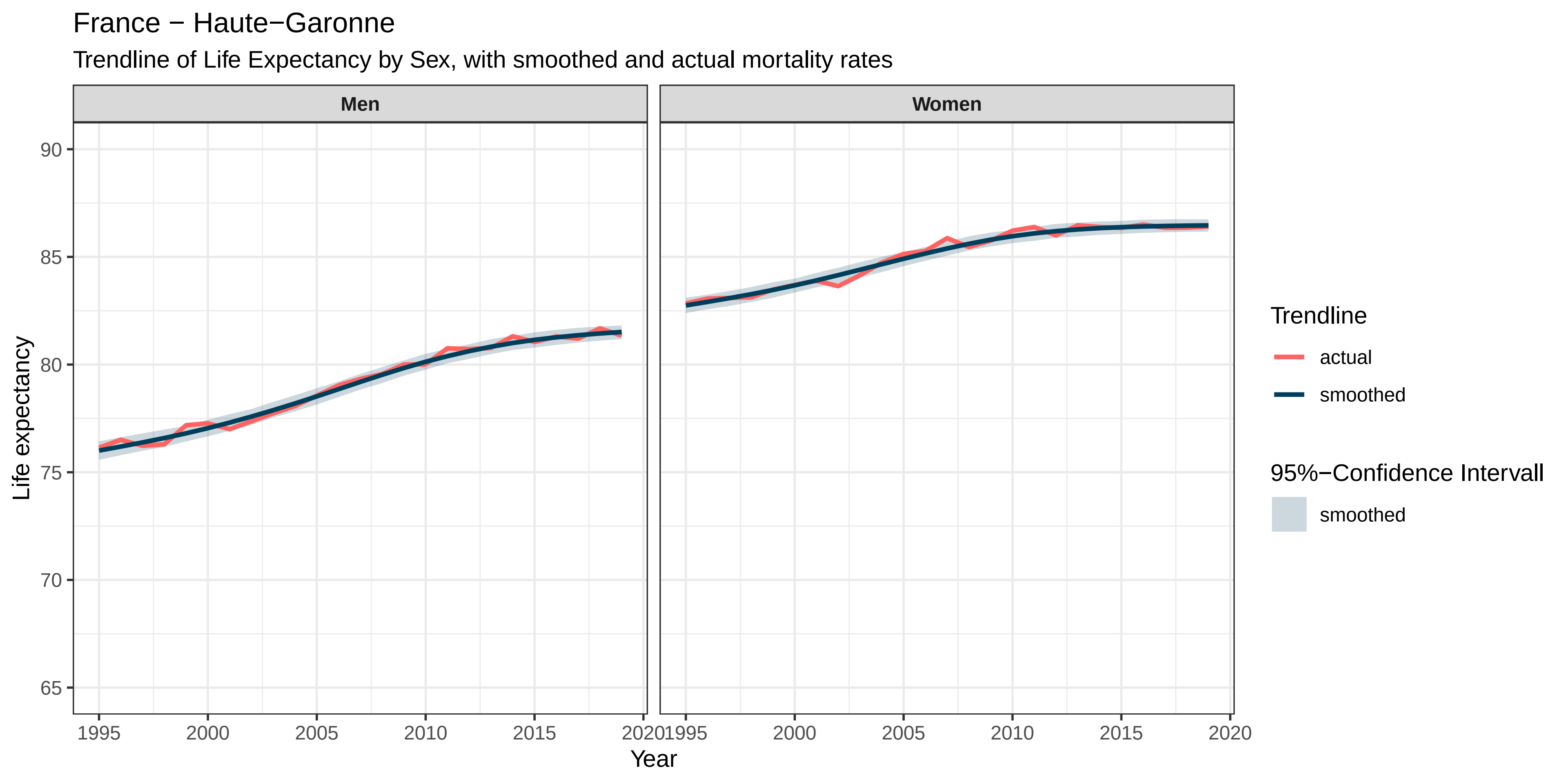
France − Haute−Garonne
Trendline of Life Expectancy by Sex, with smoothed and actual mortality rates
Men
Women
Life expectancy
90
85
80
75
70
65
1995
2000
2005
2010
2015
2020
Year
Trendline
actual
smoothed
95%−Confidence Intervall
smoothed

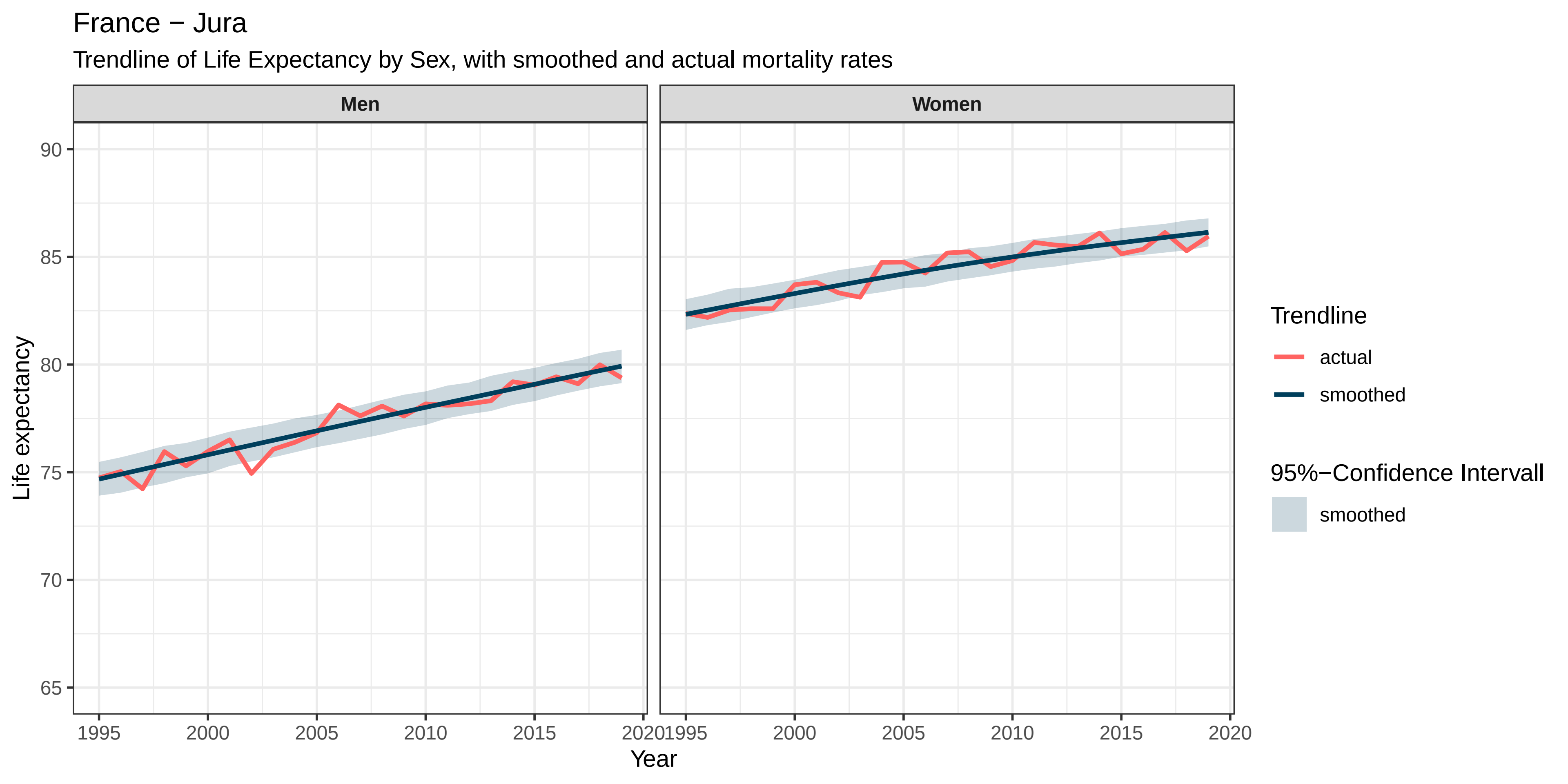
France − Jura
Trendline of Life Expectancy by Sex, with smoothed and actual mortality rates
Men
Women
Life expectancy
90
85
80
75
70
65
1995
2000
2005
2010
2015
2020
Year
Trendline
actual
smoothed
95%−Confidence Intervall
smoothed

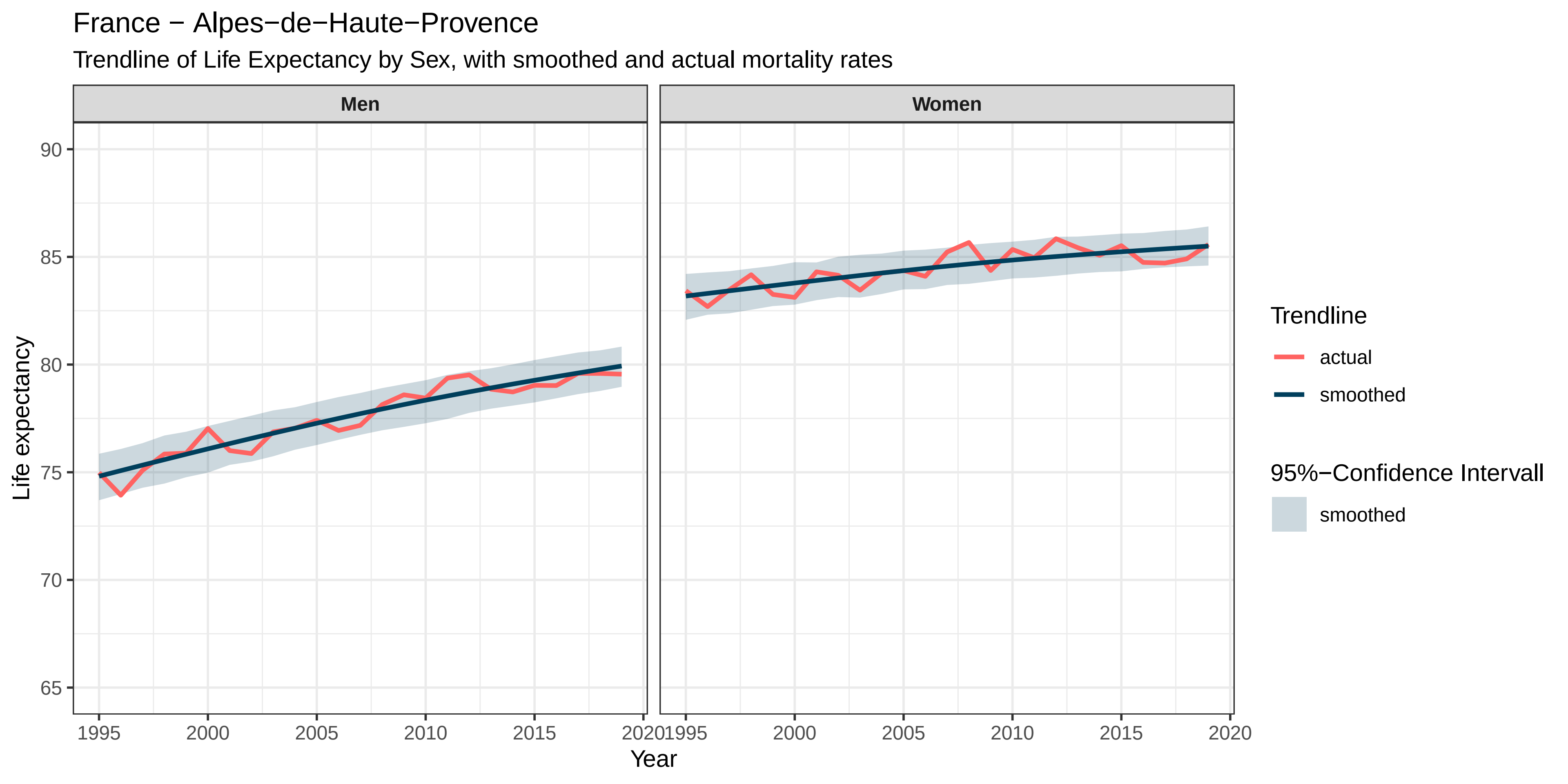

France − Alpes−de−Haute−Provence
Trendline of Life Expectancy by Sex, with smoothed and actual mortality rates
Men
Women
Life expectancy
90
85
80
75
70
65
1995
2000
2005
2010
2015
2020
Year
Trendline
actual
smoothed
95%−Confidence Intervall
smoothed

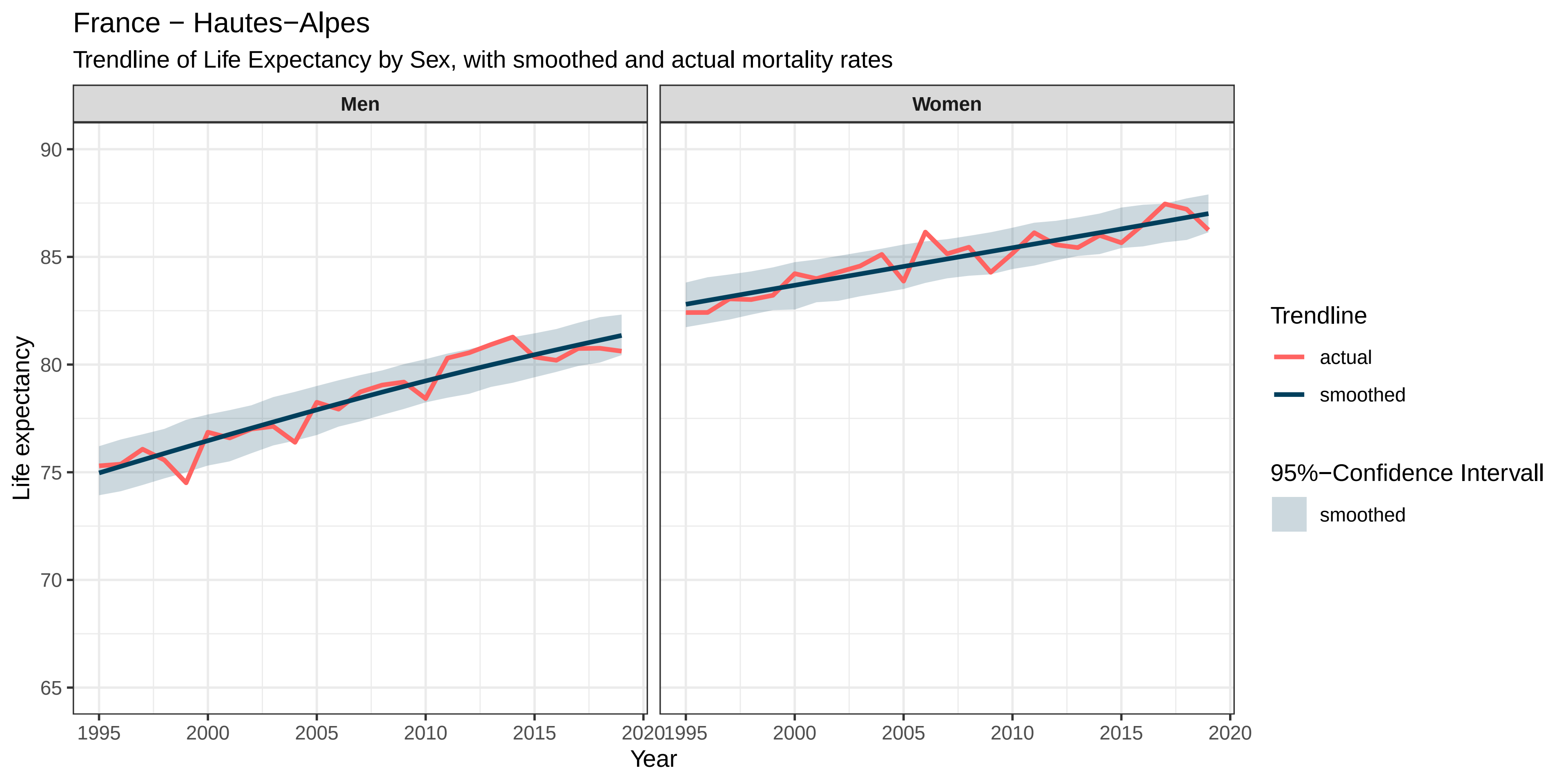

France − Hautes−Alpes
Trendline of Life Expectancy by Sex, with smoothed and actual mortality rates
Men
Women
Life expectancy
90
85
80
75
70
65
1995
2000
2005
2010
2015
2020
Year
Trendline
actual
smoothed
95%−Confidence Intervall
smoothed

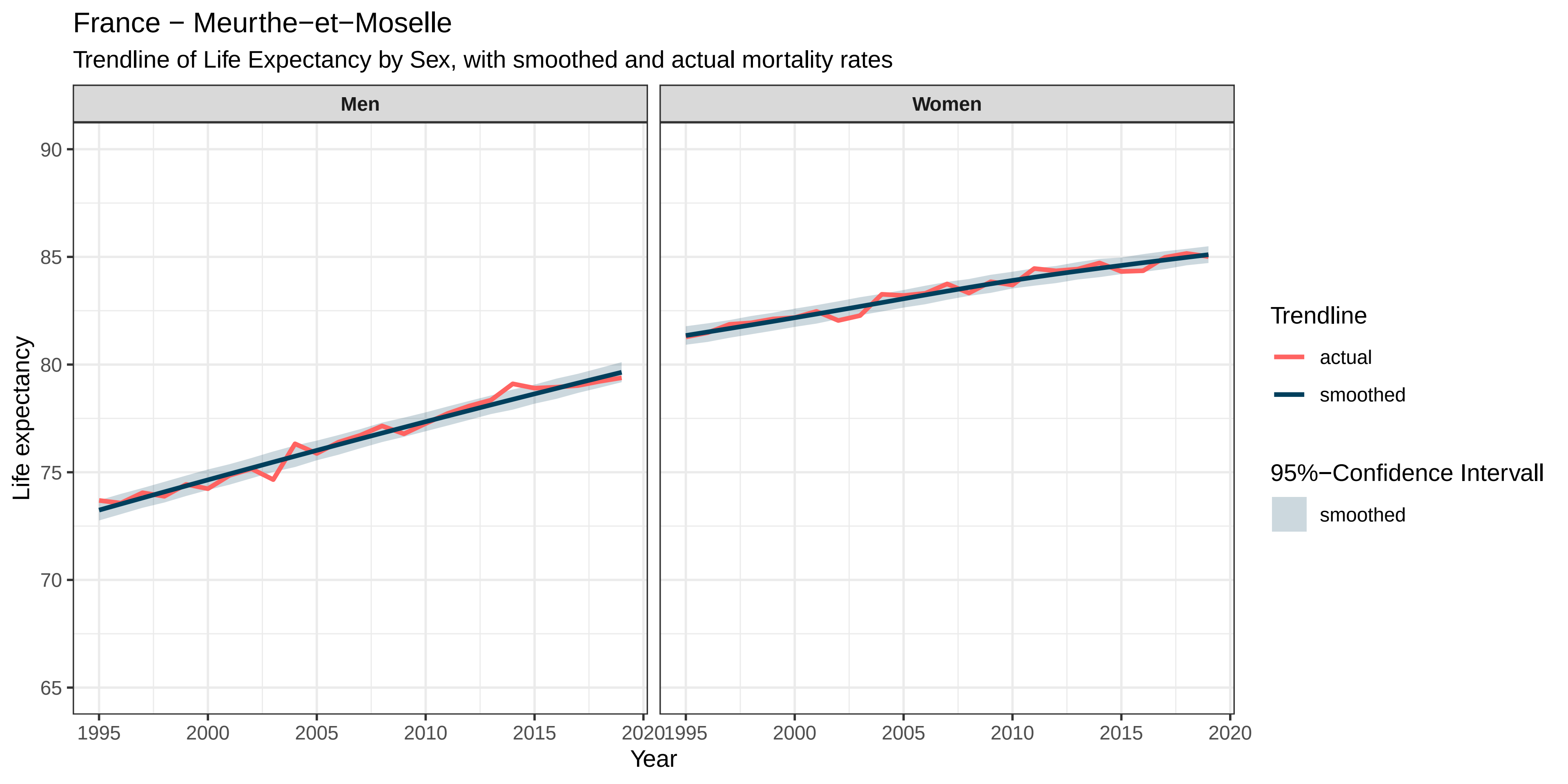

France − Meurthe−et−Moselle
Trendline of Life Expectancy by Sex, with smoothed and actual mortality rates
Men
Women
Life expectancy
Year
90
85
80
75
70
65
1995
2000
2005
2010
2015
2020
Trendline
actual
smoothed
95%−Confidence Intervall
smoothed

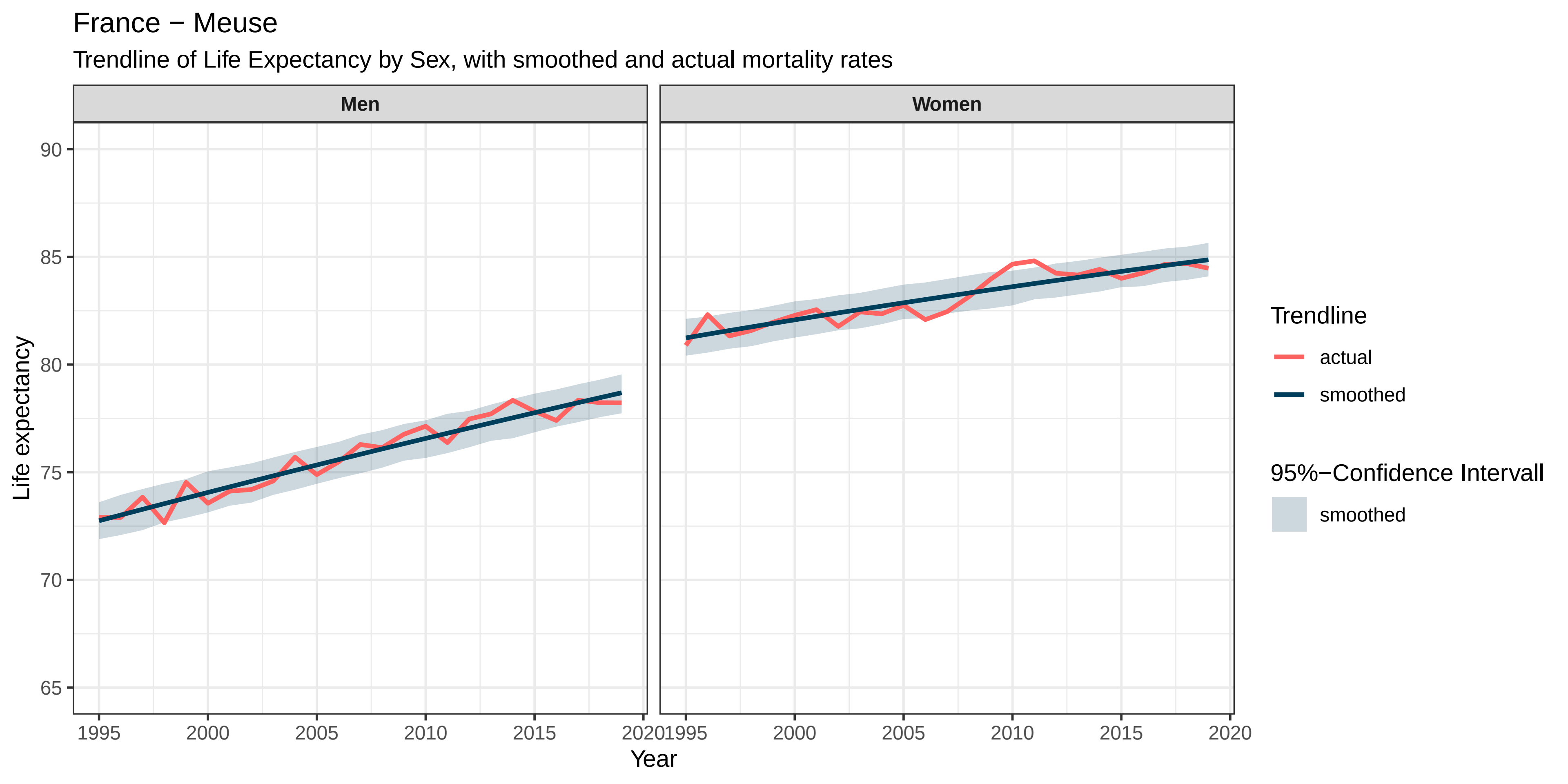
France − Meuse
Trendline of Life Expectancy by Sex, with smoothed and actual mortality rates
Men
Women
Life expectancy
Year
90
85
80
75
70
65
1995
2000
2005
2010
2015
2020
Trendline
actual
smoothed
95%−Confidence Intervall
smoothed

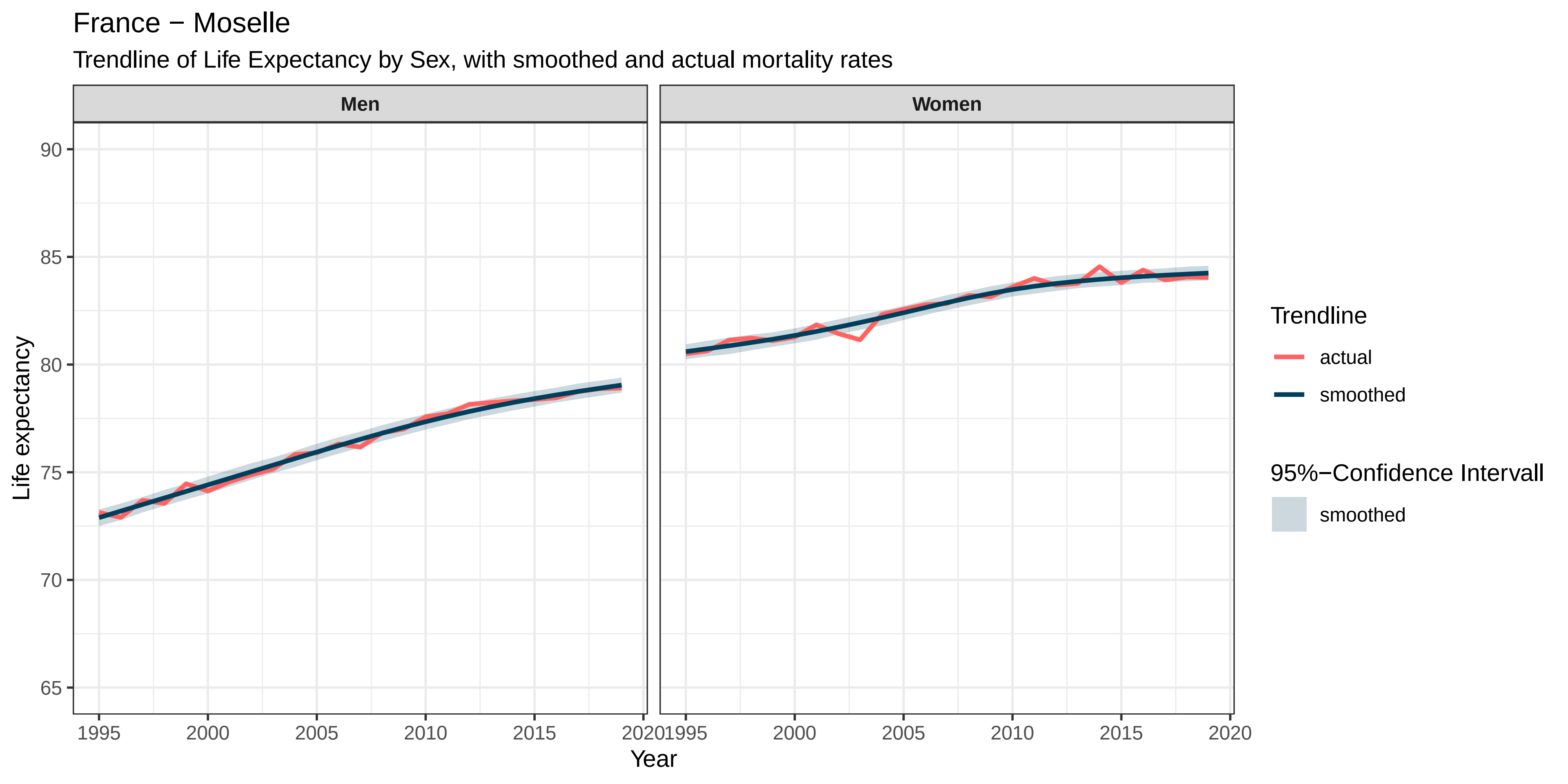
France − Moselle
Trendline of Life Expectancy by Sex, with smoothed and actual mortality rates
Men
Women
Life expectancy
Year
90
85
80
75
70
65
1995
2000
2005
2010
2015
2020
Trendline
actual
smoothed
95%−Confidence Intervall
smoothed

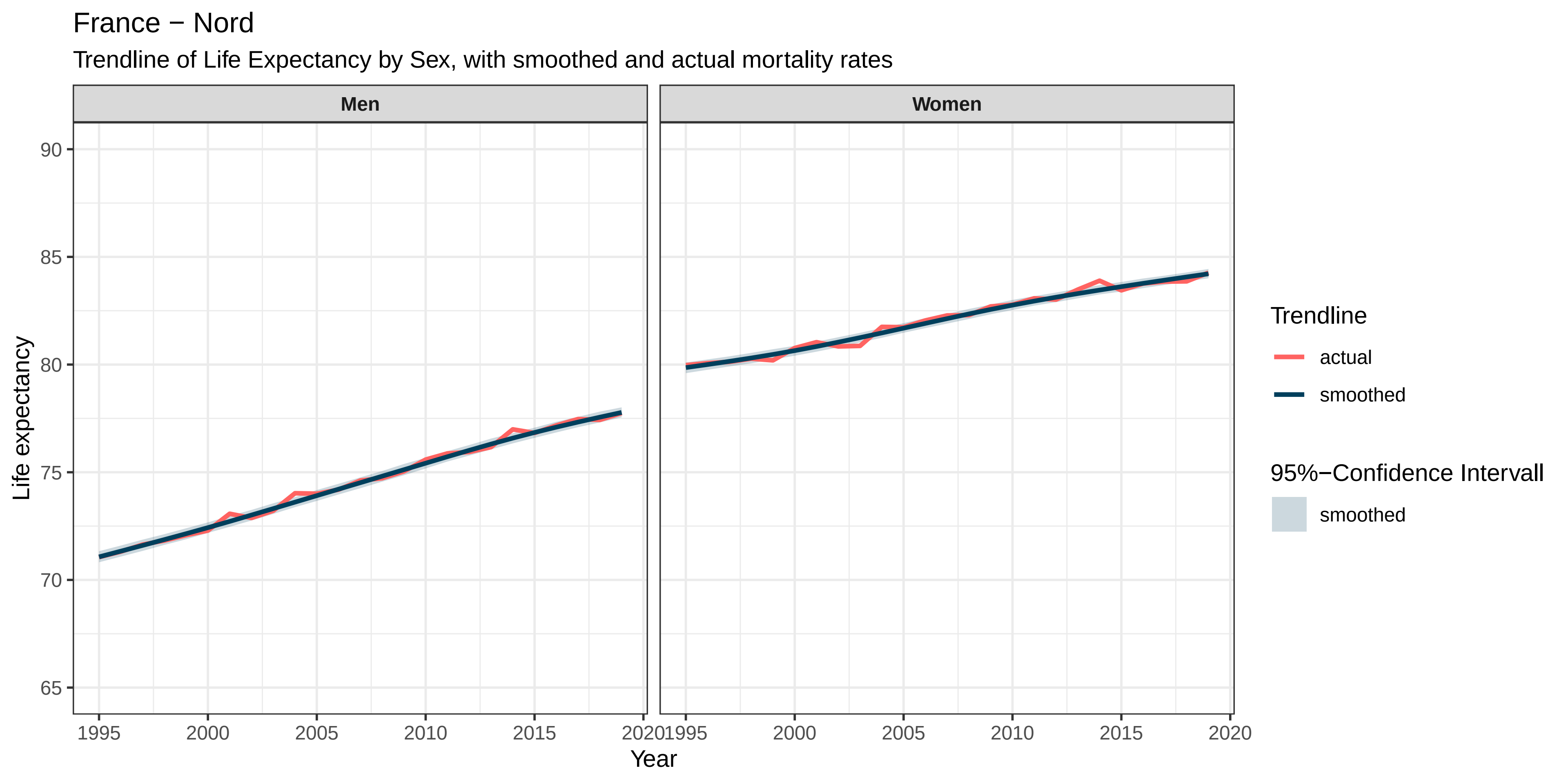

France − Nord
Trendline of Life Expectancy by Sex, with smoothed and actual mortality rates
Men
Women
90
85
80
75
70
65
Life expectancy
1995
2000
2005
2010
2015
2020
Year
Trendline
actual
smoothed
95%−Confidence Intervall
smoothed

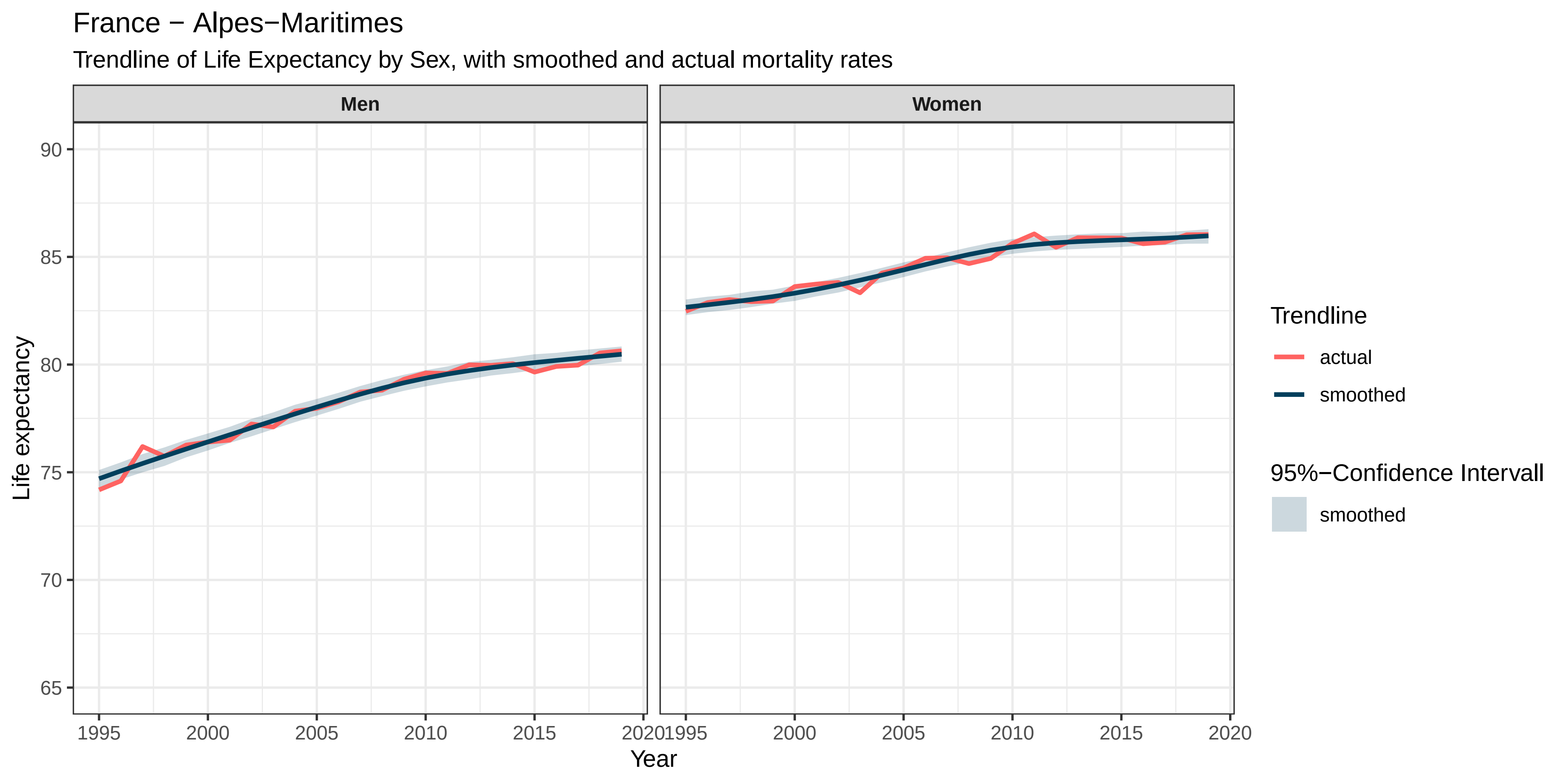
France − Alpes−Maritimes
Trendline of Life Expectancy by Sex, with smoothed and actual mortality rates
Men
Women
Life expectancy
Year
90
85
80
75
70
65
1995
2000
2005
2010
2015
2020
Trendline
actual
smoothed
95%−Confidence Intervall
smoothed

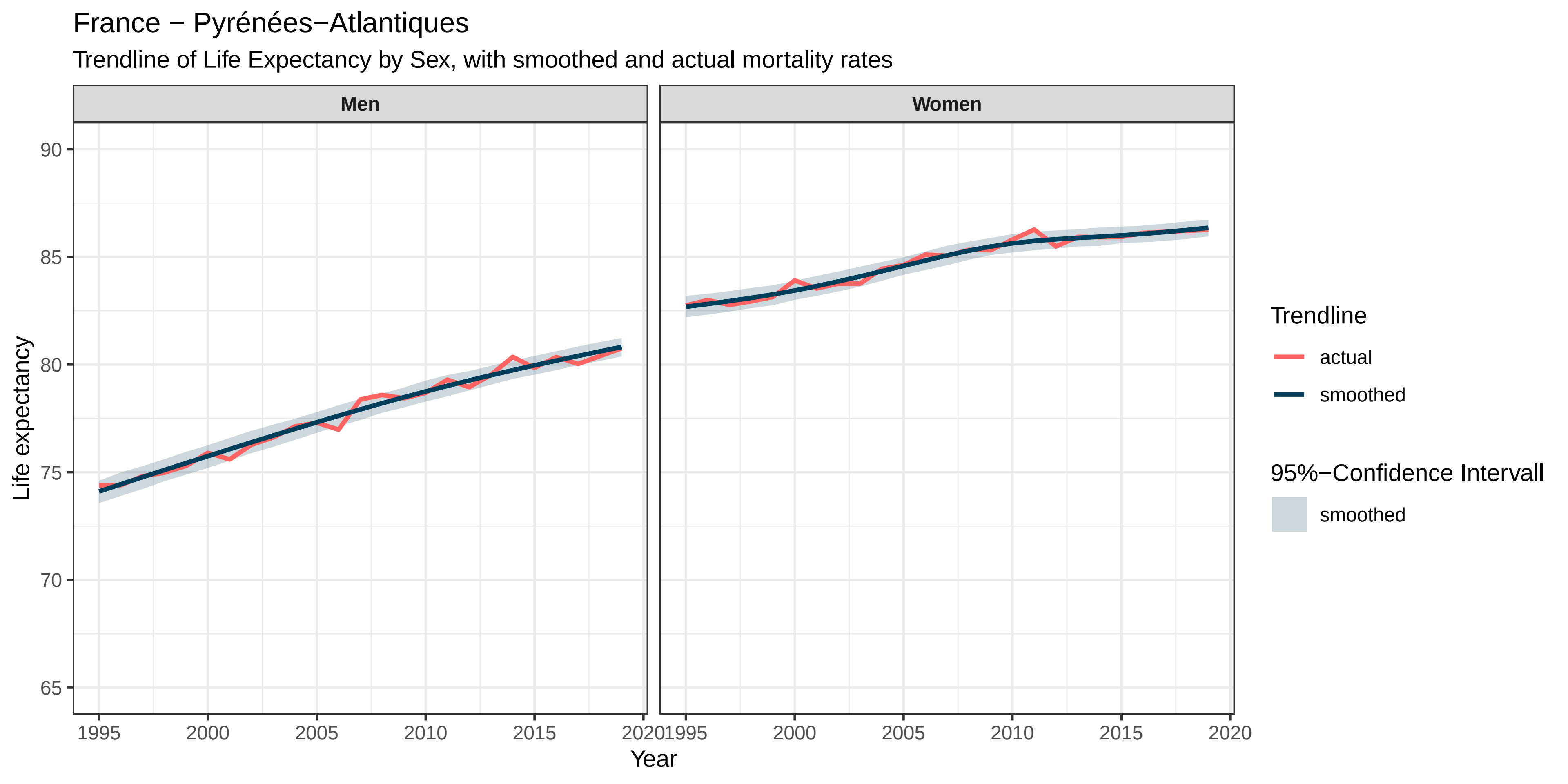
France − Pyrénées−Atlantiques
Trendline of Life Expectancy by Sex, with smoothed and actual mortality rates
Men
Women
Life expectancy
90
85
80
75
70
65
1995
2000
2005
2010
2015
2020
Year
Trendline
actual
smoothed
95%−Confidence Intervall
smoothed

France − Hautes−Pyrénées

Trendline of Life Expectancy by Sex, with smoothed and actual mortality rates

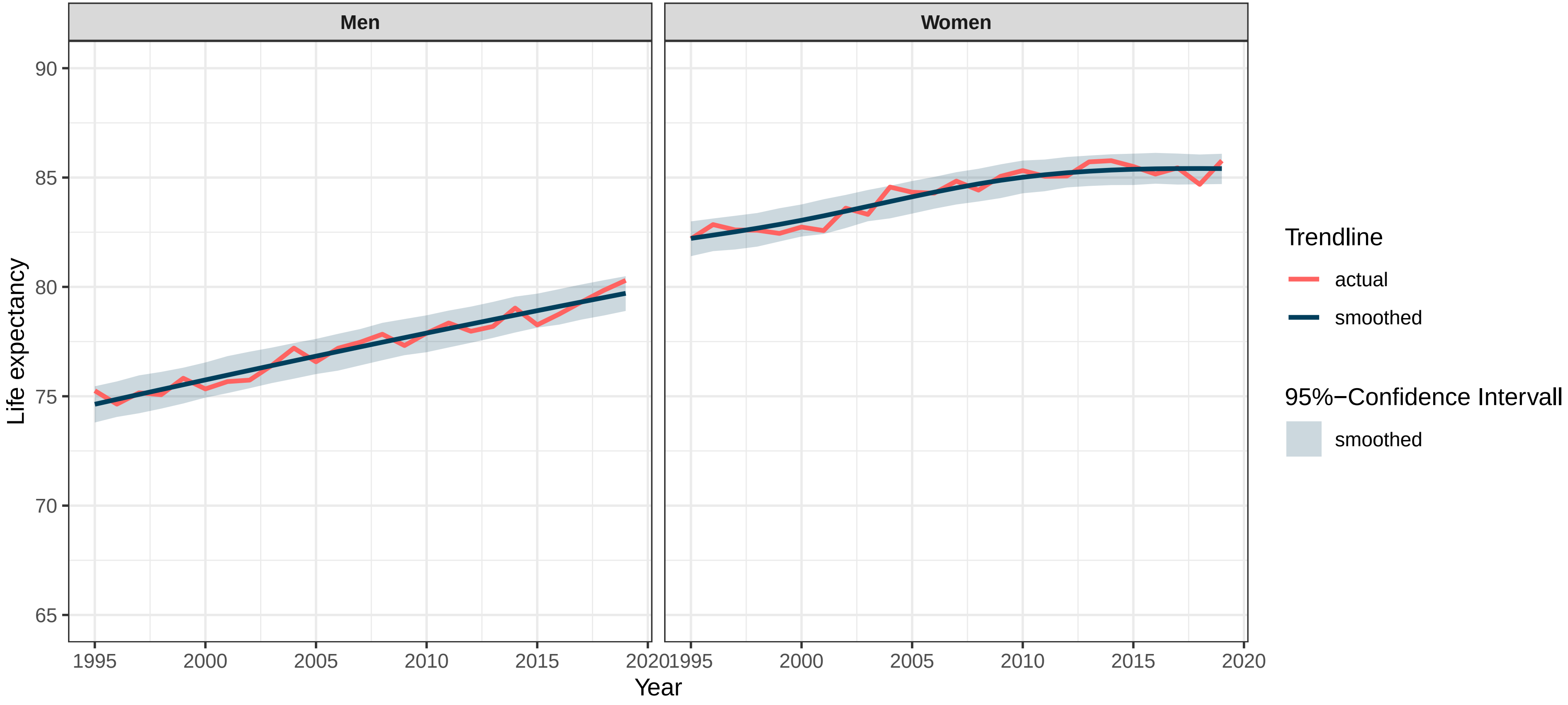

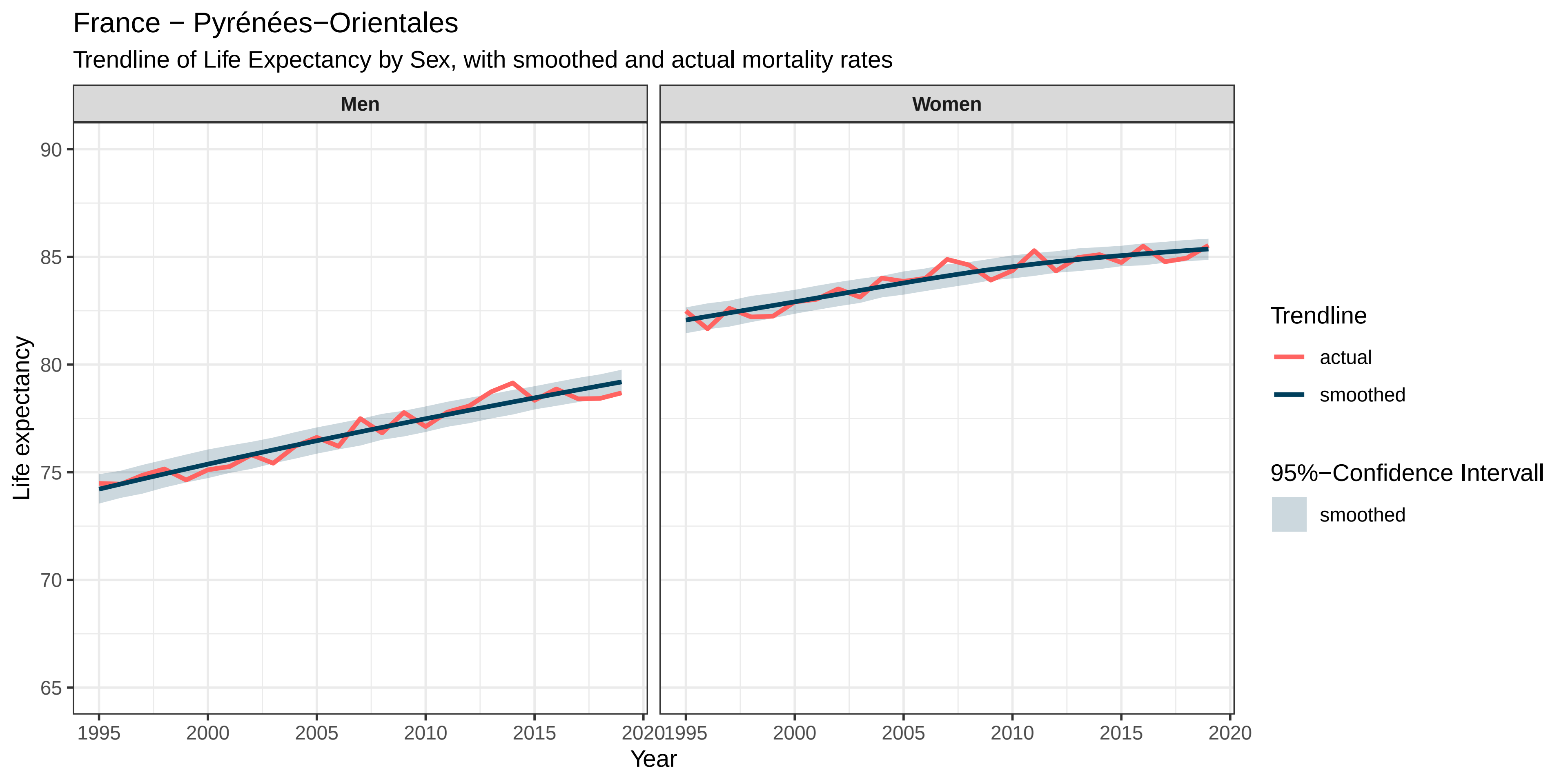

France − Pyrénées−Orientales
Trendline of Life Expectancy by Sex, with smoothed and actual mortality rates
Men
Women
Life expectancy
90
85
80
75
70
65
1995
2000
2005
2010
2015
2020
Year
Trendline
actual
smoothed
95%−Confidence Intervall
smoothed

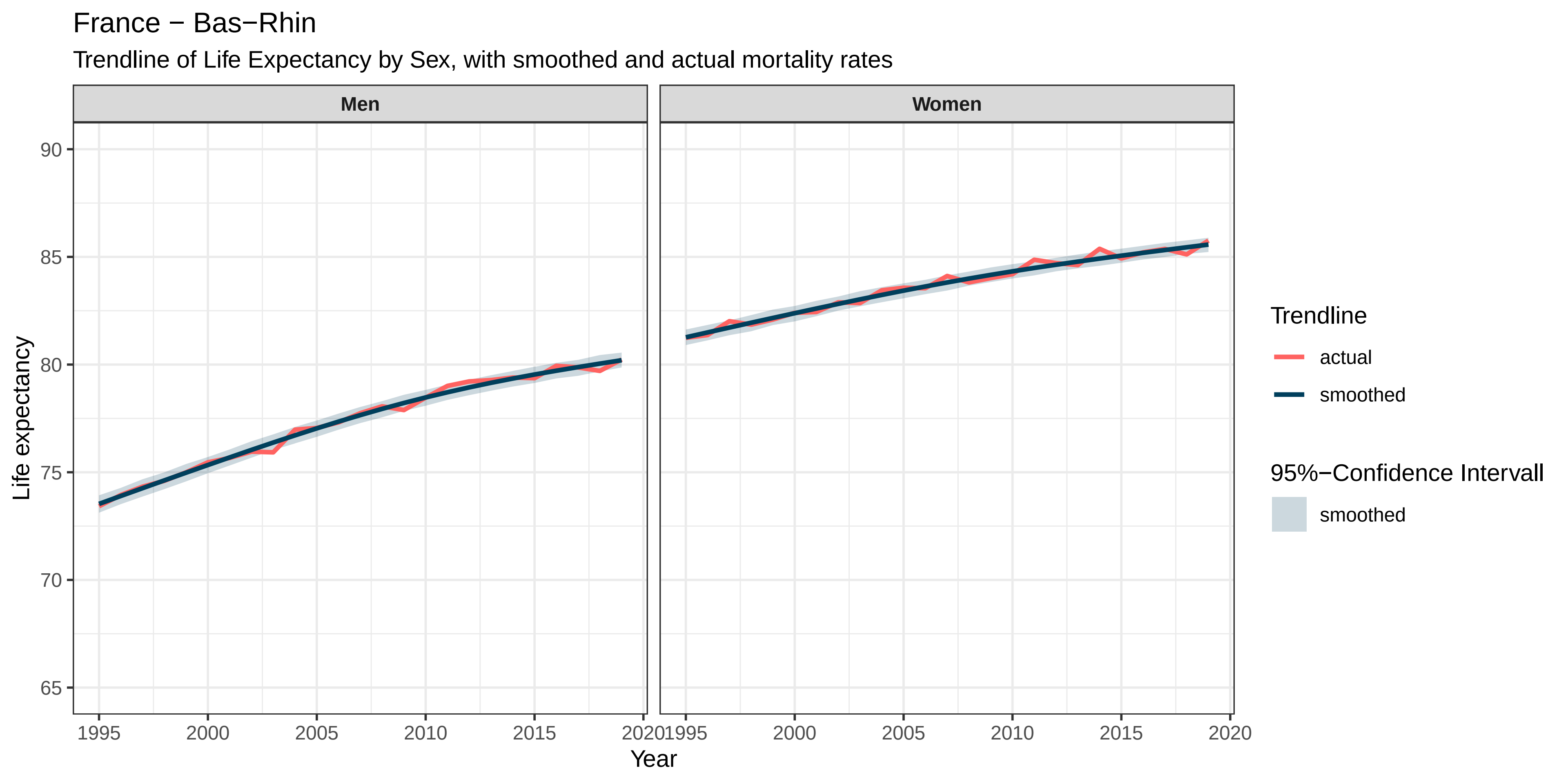
France − Bas−Rhin
Trendline of Life Expectancy by Sex, with smoothed and actual mortality rates
Men
Women
90
85
80
75
70
65
Life expectancy
1995
2000
2005
2010
2015
2020
Year
Trendline
actual
smoothed
95%−Confidence Intervall
smoothed

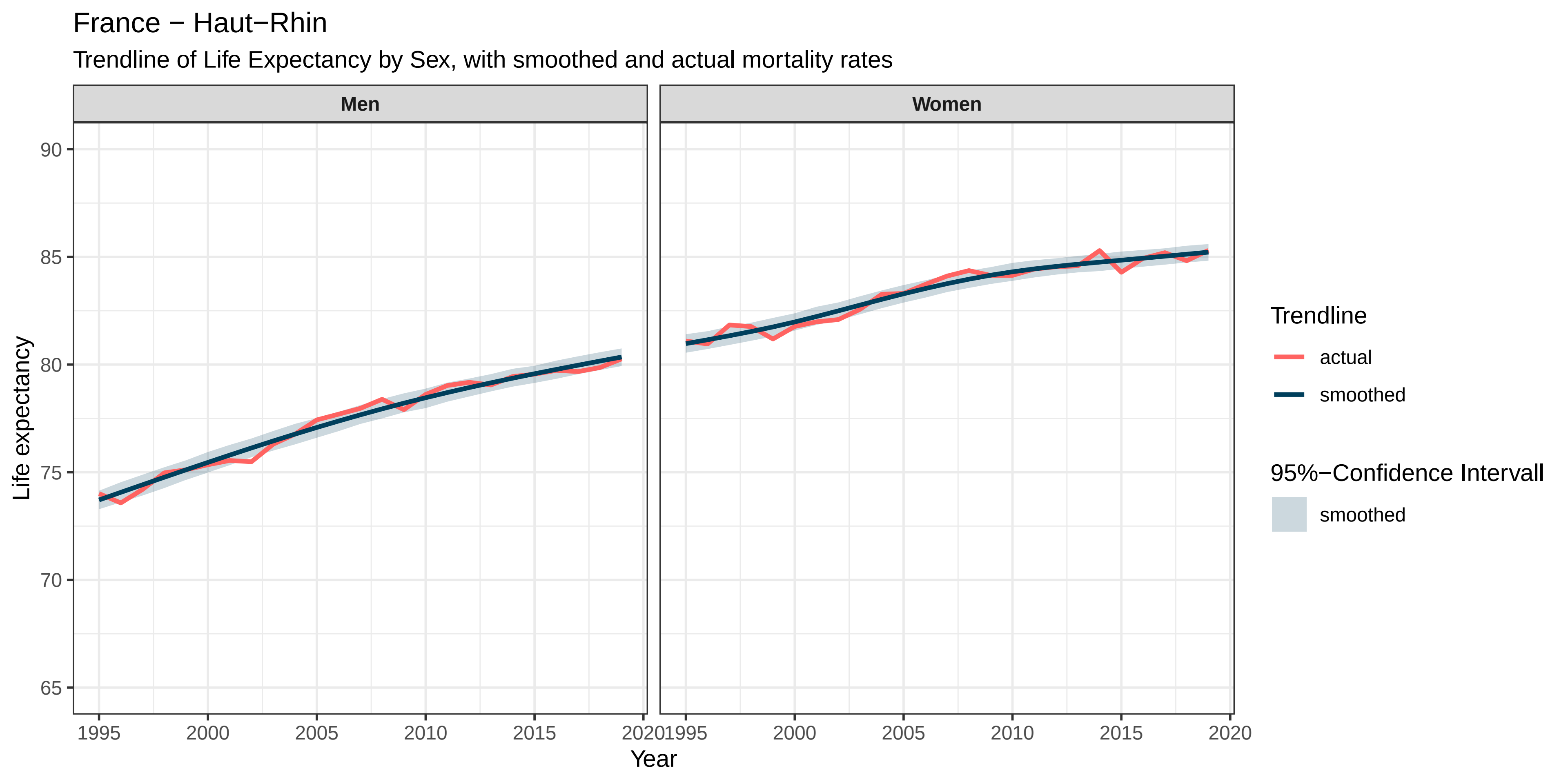
France − Haut−Rhin
Trendline of Life Expectancy by Sex, with smoothed and actual mortality rates
Men
Women
Life expectancy
90
85
80
75
70
65
1995
2000
2005
2010
2015
2020
Year
Trendline
actual
smoothed
95%−Confidence Intervall
smoothed

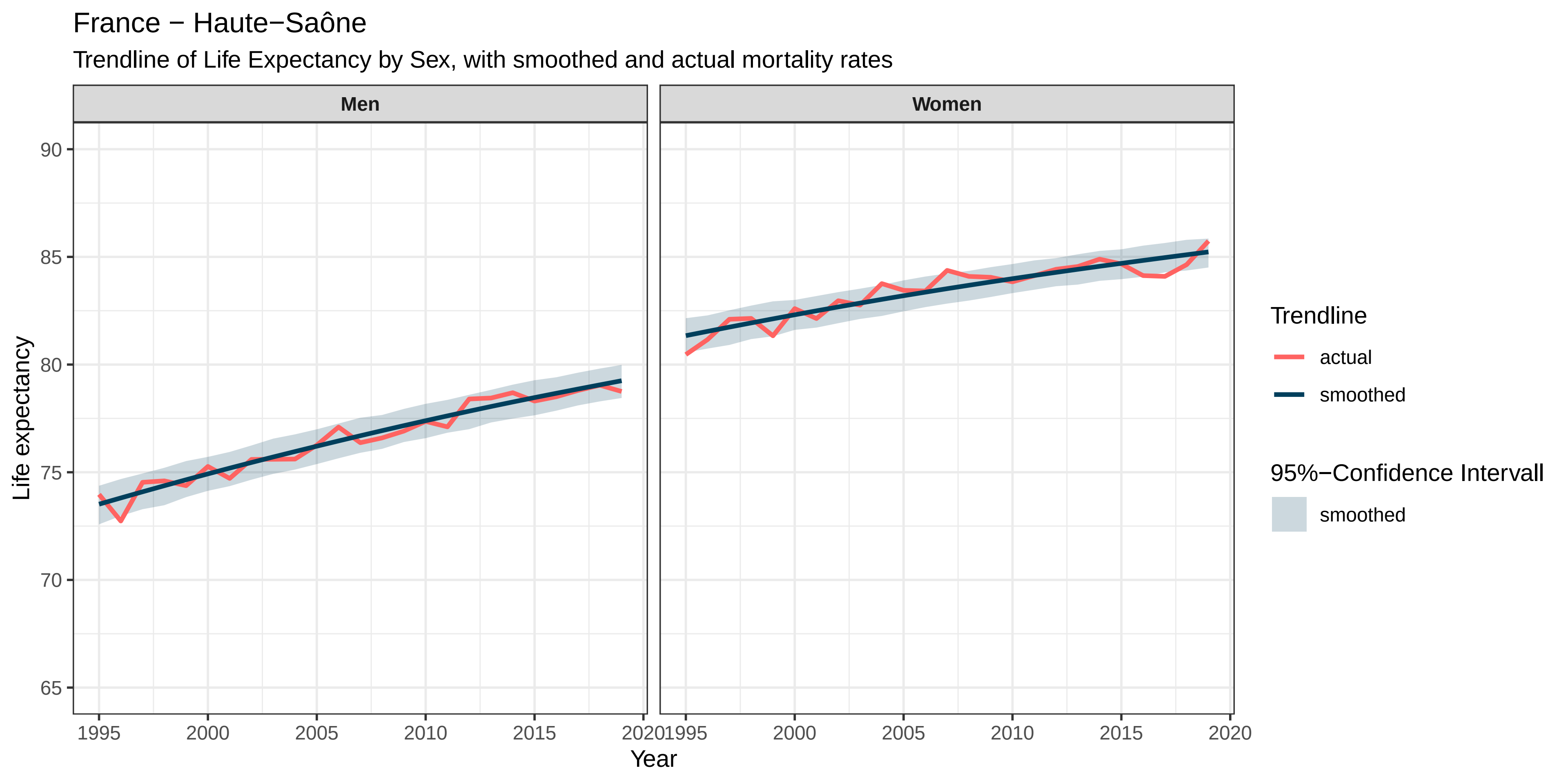
France − Haute−Saône
Trendline of Life Expectancy by Sex, with smoothed and actual mortality rates
Men
Women
Life expectancy
65
70
75
80
85
90
1995
2000
2005
2010
2015
2020
Year
Trendline
actual
smoothed
95%−Confidence Intervall
smoothed

France − Savoie

Trendline of Life Expectancy by Sex, with smoothed and actual mortality rates

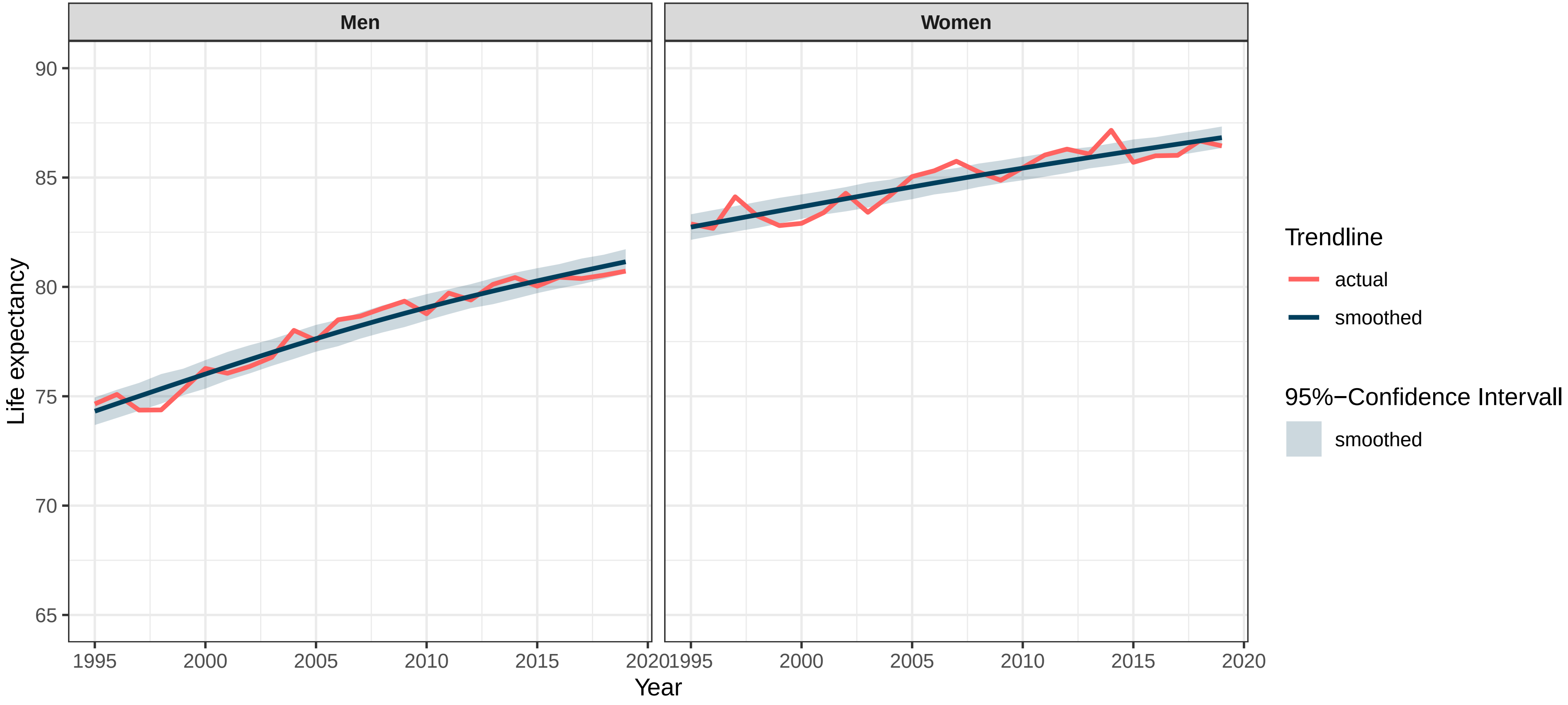

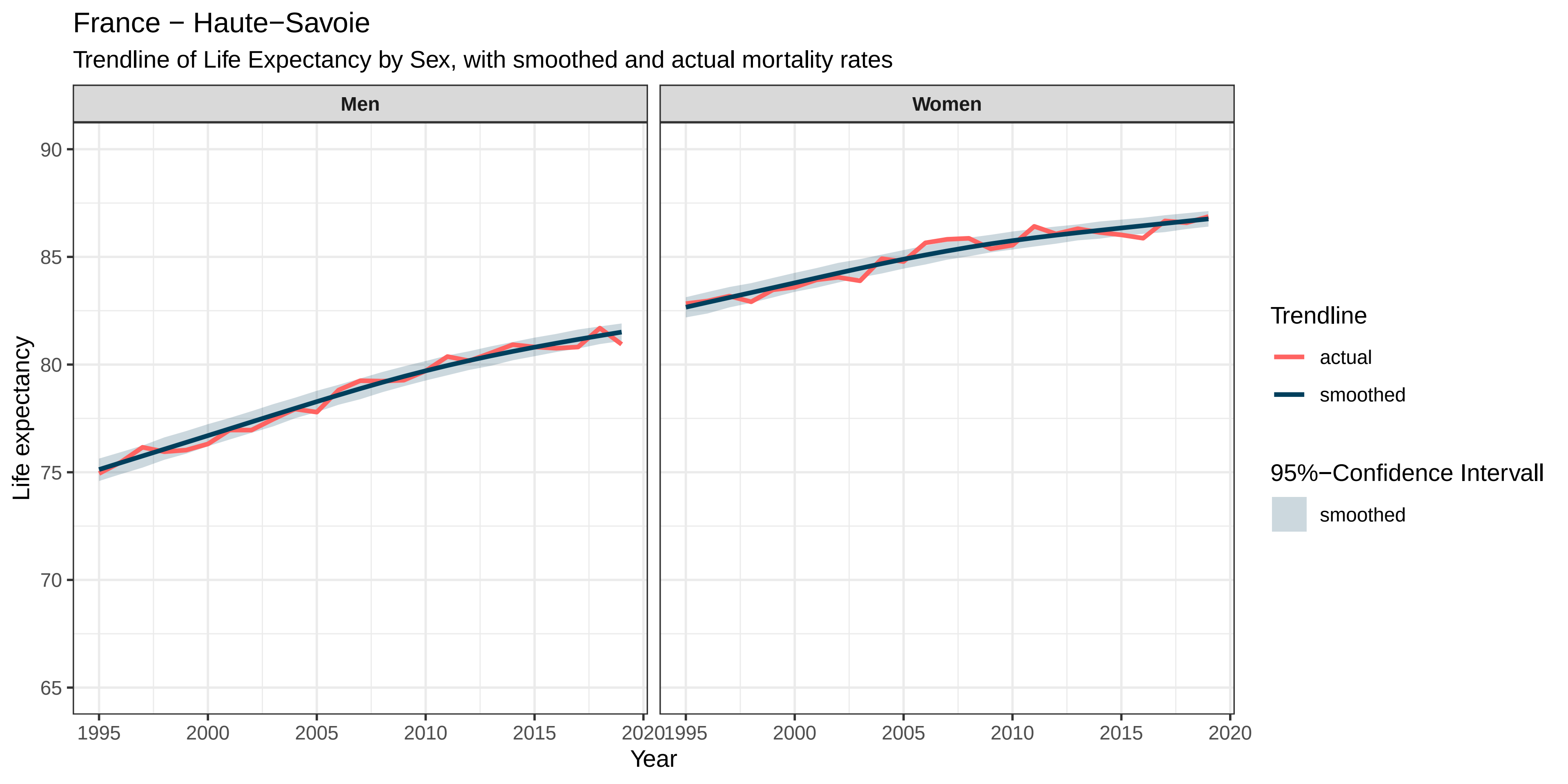
France − Haute−Savoie
Trendline of Life Expectancy by Sex, with smoothed and actual mortality rates
Men
Women
90
85
80
75
70
65
Life expectancy
1995
2000
2005
2010
2015
2020
Year
Trendline
actual
smoothed
95%−Confidence Intervall
smoothed

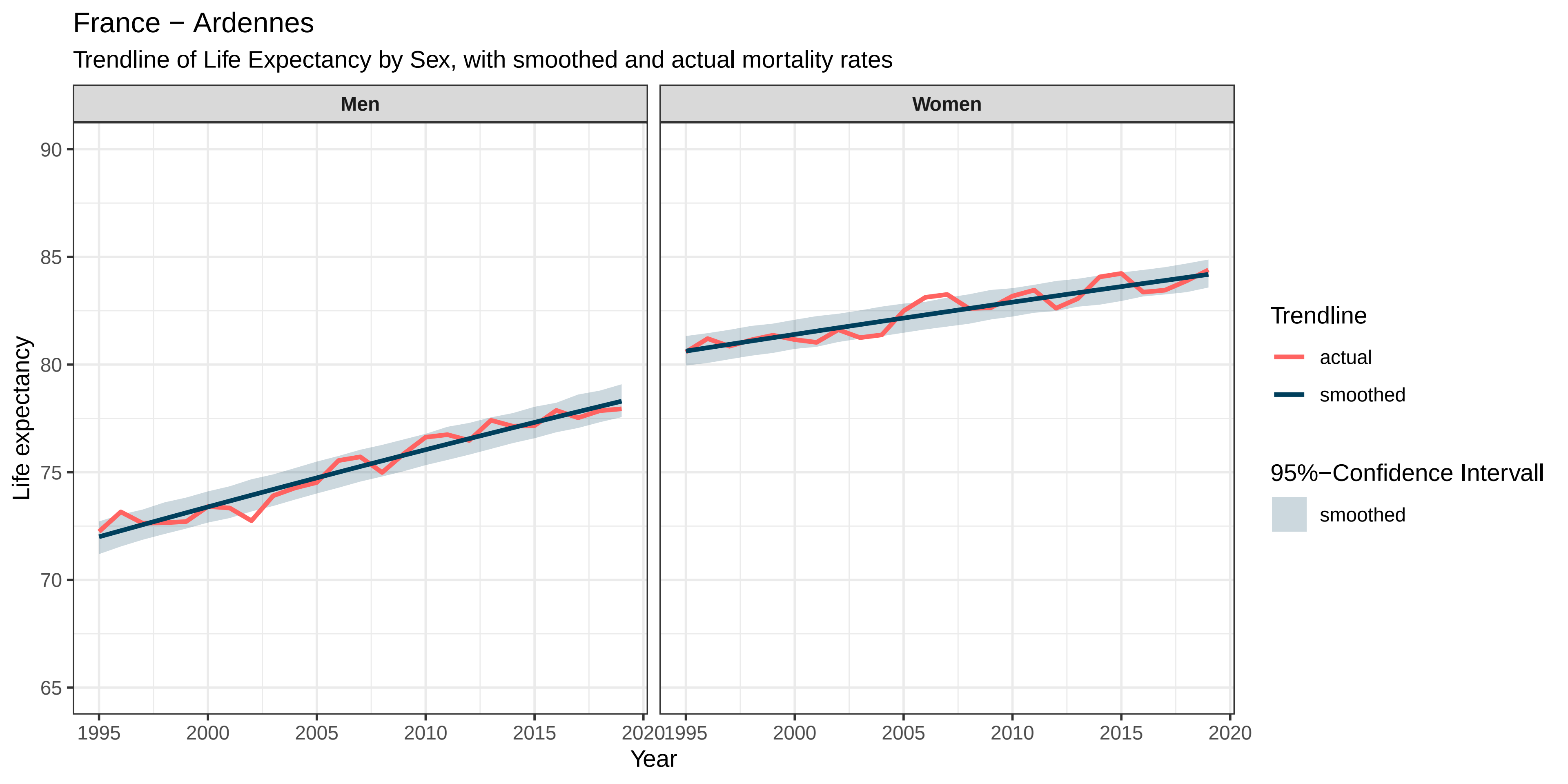
France − Ardennes
Trendline of Life Expectancy by Sex, with smoothed and actual mortality rates
Men
Women
Life expectancy
90
85
80
75
70
65
1995
2000
2005
2010
2015
2020
Year
Trendline
actual
smoothed
95%−Confidence Intervall
smoothed

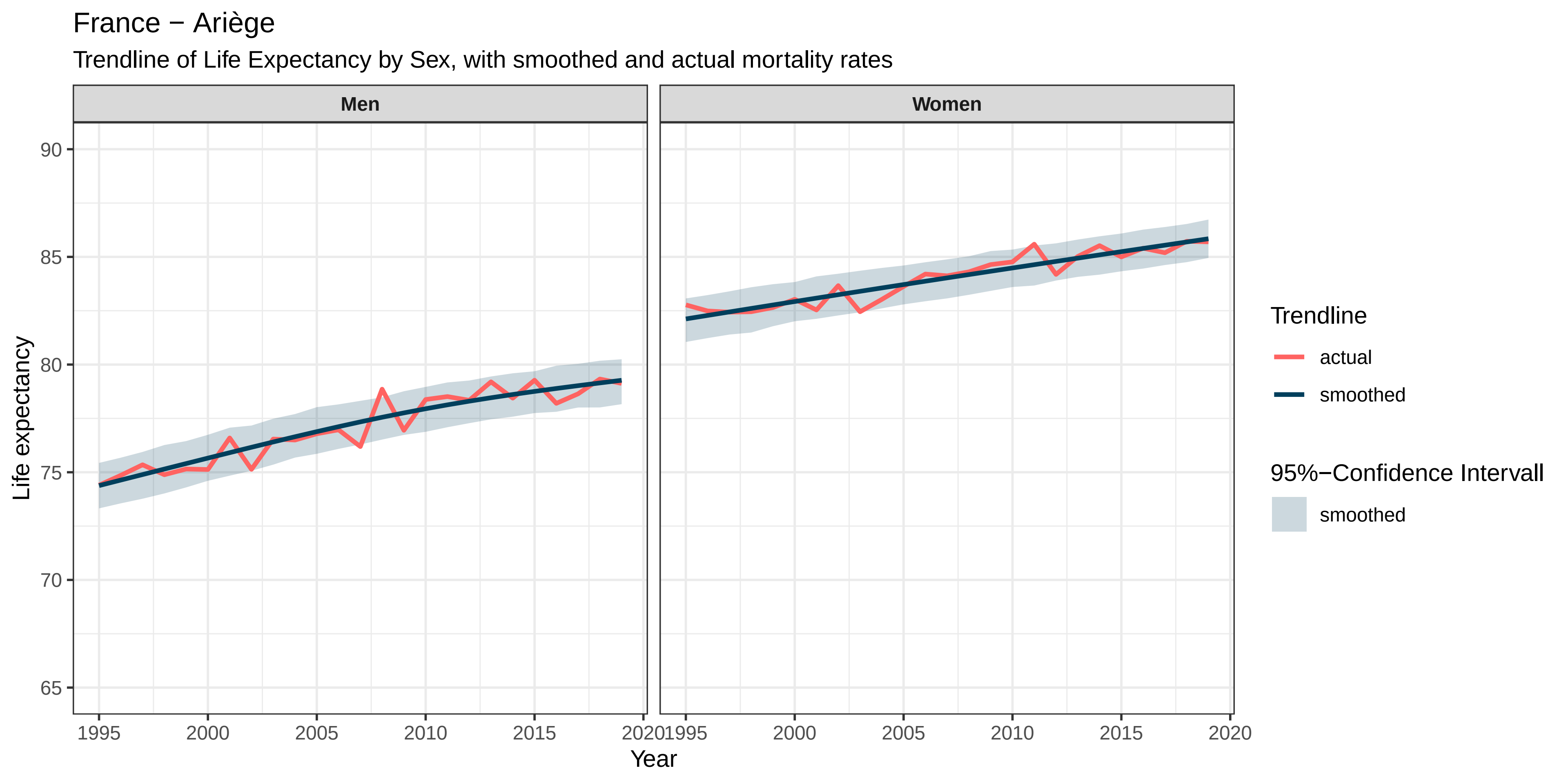
France − Ariège
Trendline of Life Expectancy by Sex, with smoothed and actual mortality rates
Men
Women
Life expectancy
90
85
80
75
70
65
1995
2000
2005
2010
2015
2020
Year
Trendline
actual
smoothed
95%−Confidence Intervall
smoothed

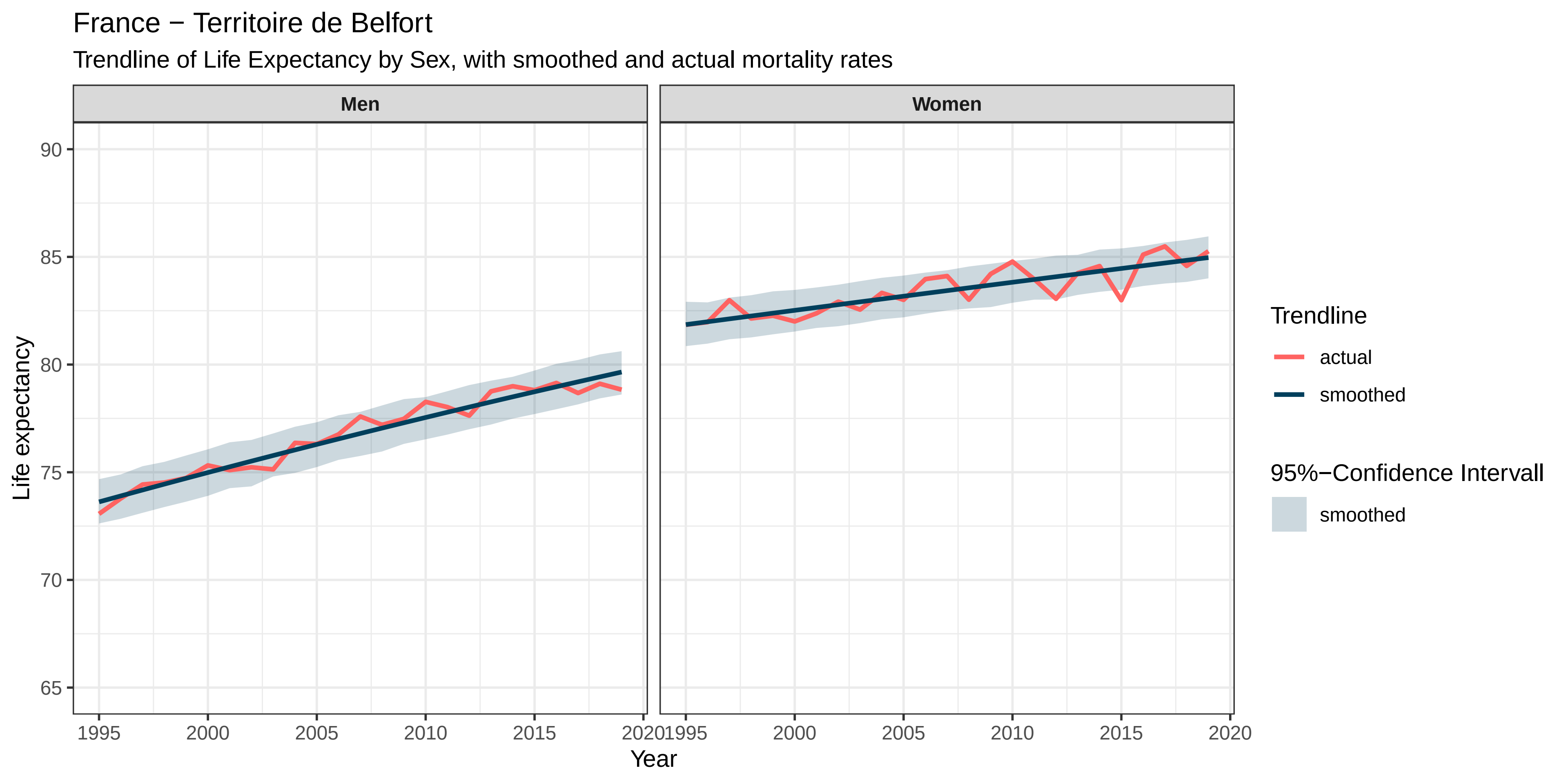

France − Territoire de Belfort
Trendline of Life Expectancy by Sex, with smoothed and actual mortality rates
Men
Women
Life expectancy
90
85
80
75
70
65
1995
2000
2005
2010
2015
2020
Year
Trendline
actual
smoothed
95%−Confidence Intervall
smoothed

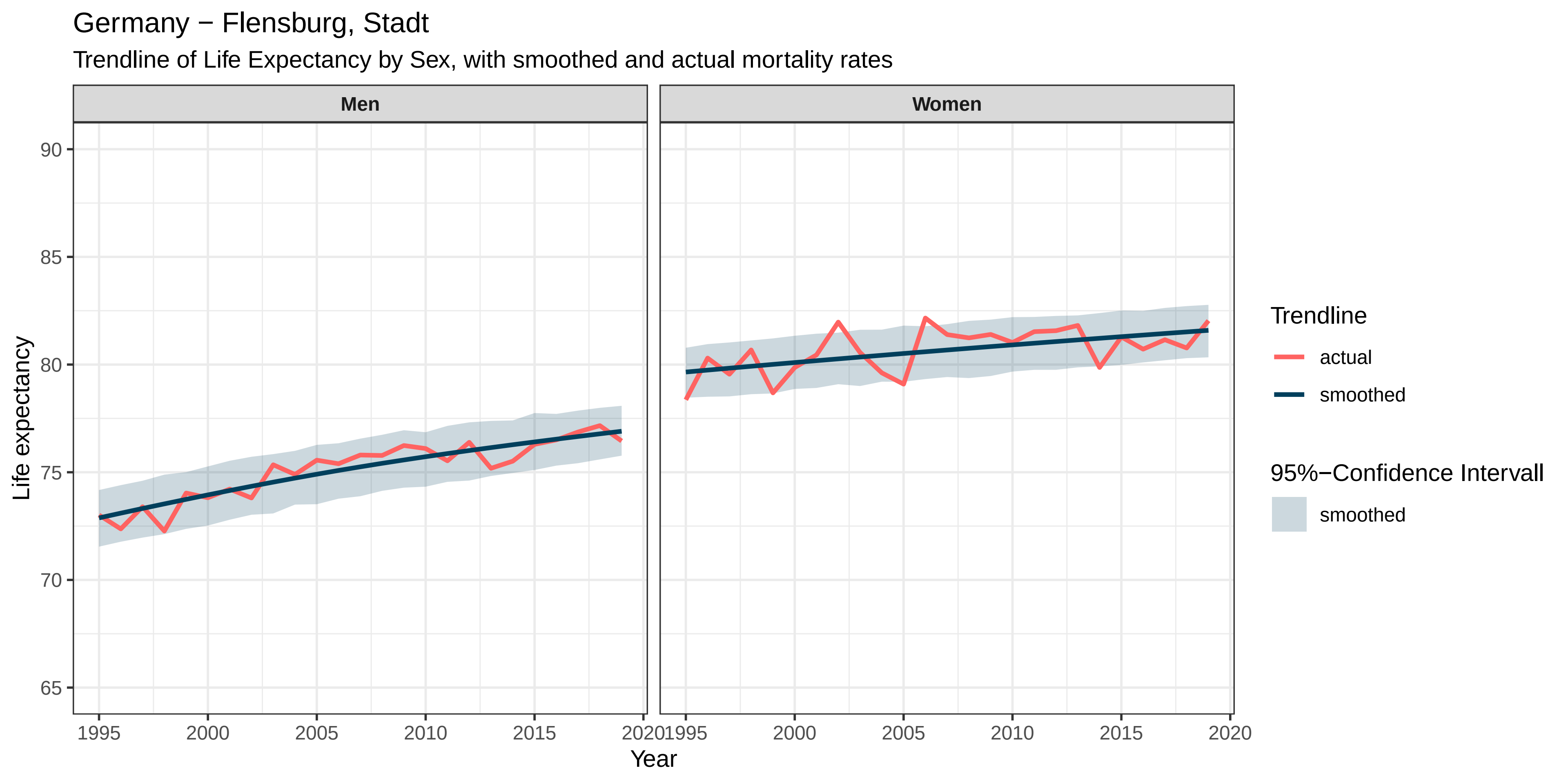

Germany − Flensburg, Stadt
Trendline of Life Expectancy by Sex, with smoothed and actual mortality rates
Men
Women
Life expectancy
90
85
80
75
70
65
1995
2000
2005
2010
2015
2020
Year
Trendline
actual
smoothed
95%−Confidence Intervall
smoothed

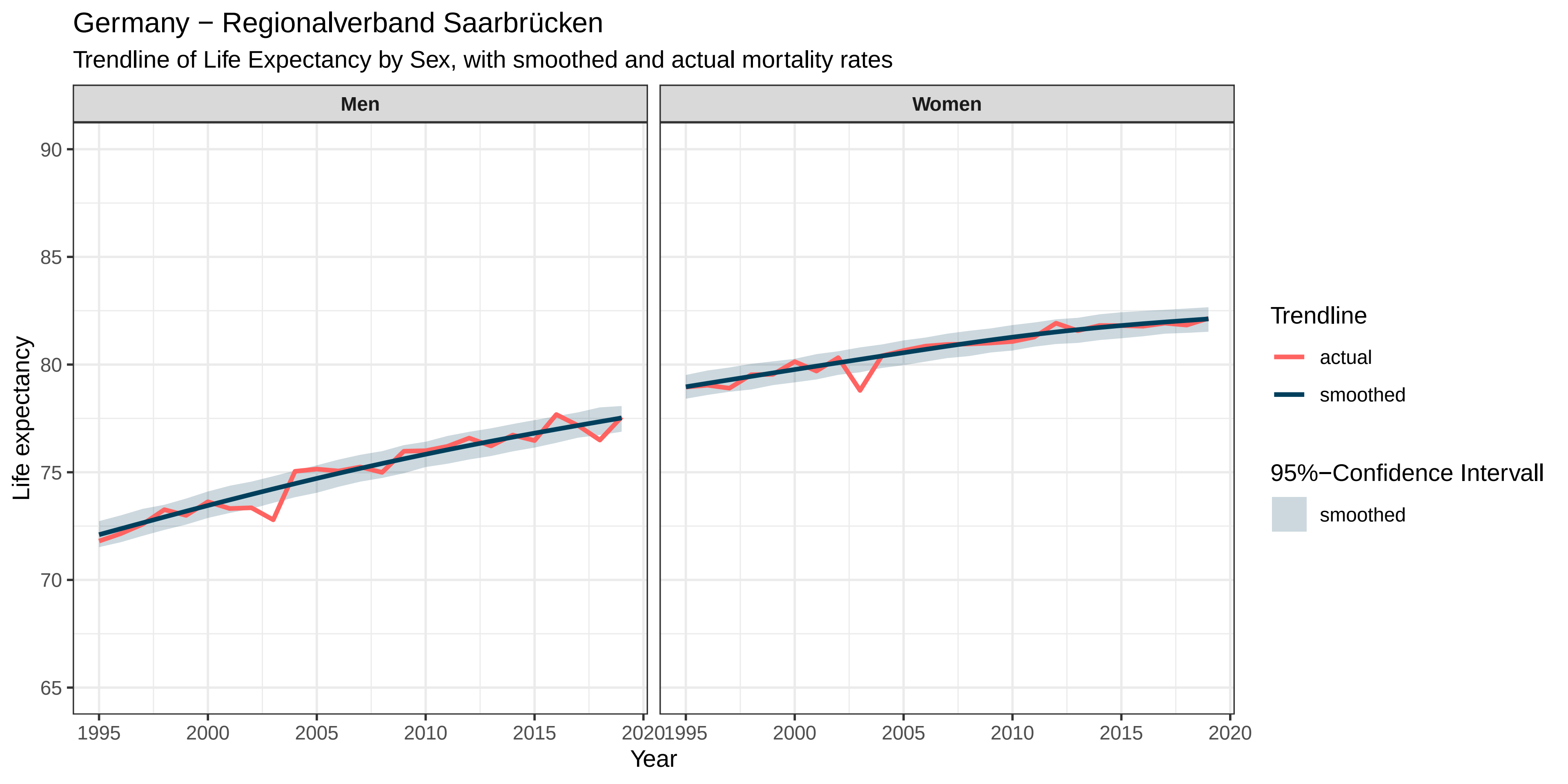

Germany − Regionalverband Saarbrücken
Trendline of Life Expectancy by Sex, with smoothed and actual mortality rates
Men
Women
Life expectancy
90
85
80
75
70
65
1995
2000
2005
2010
2015
2020
Year
Trendline
actual
smoothed
95%−Confidence Intervall
smoothed

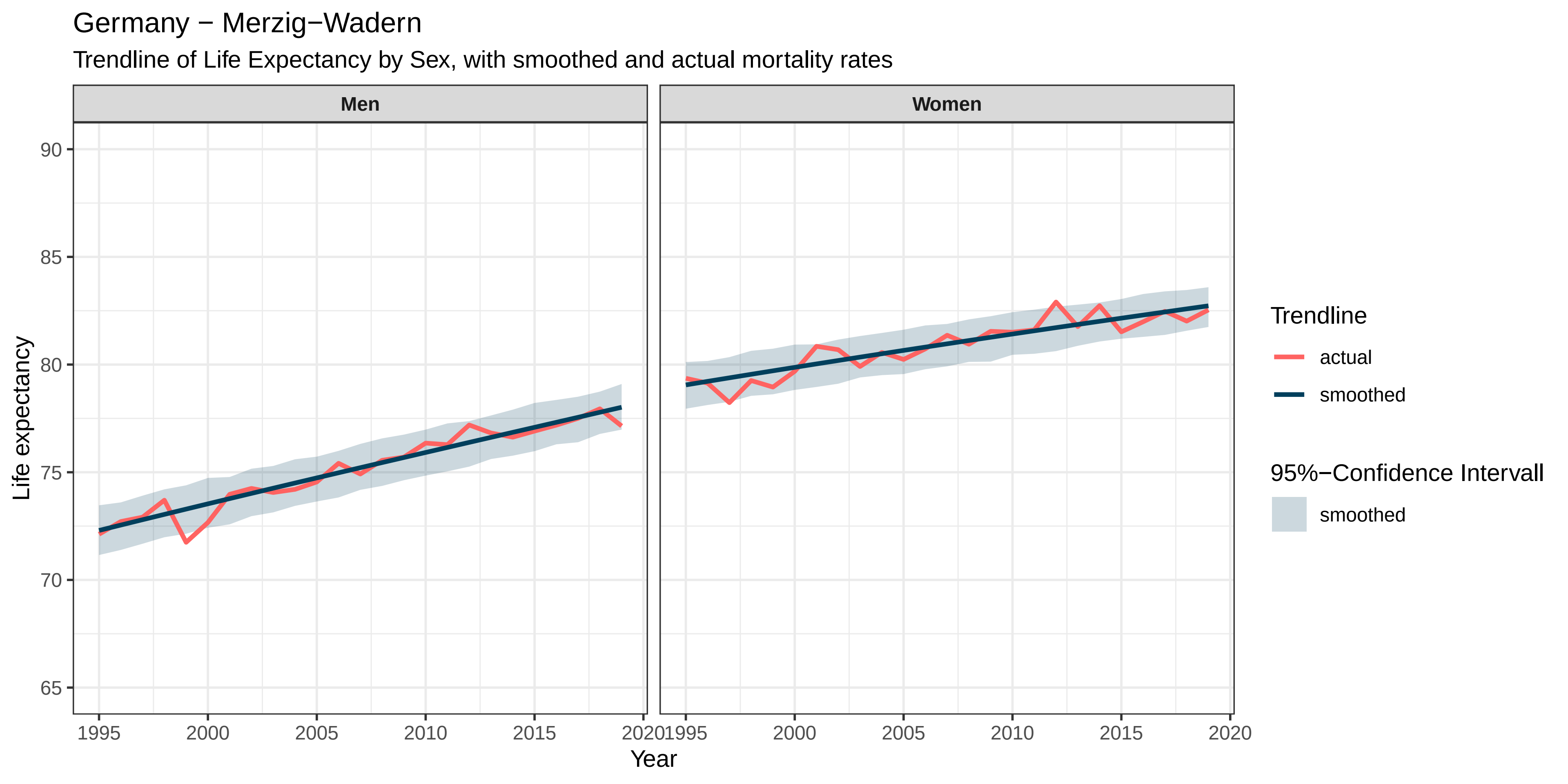

Germany − Merzig−Wadern
Trendline of Life Expectancy by Sex, with smoothed and actual mortality rates
Men
Women
90
85
80
75
70
65
1995
2000
2005
2010
2015
2020
Year
Life expectancy
Trendline
actual
smoothed
95%−Confidence Intervall
smoothed

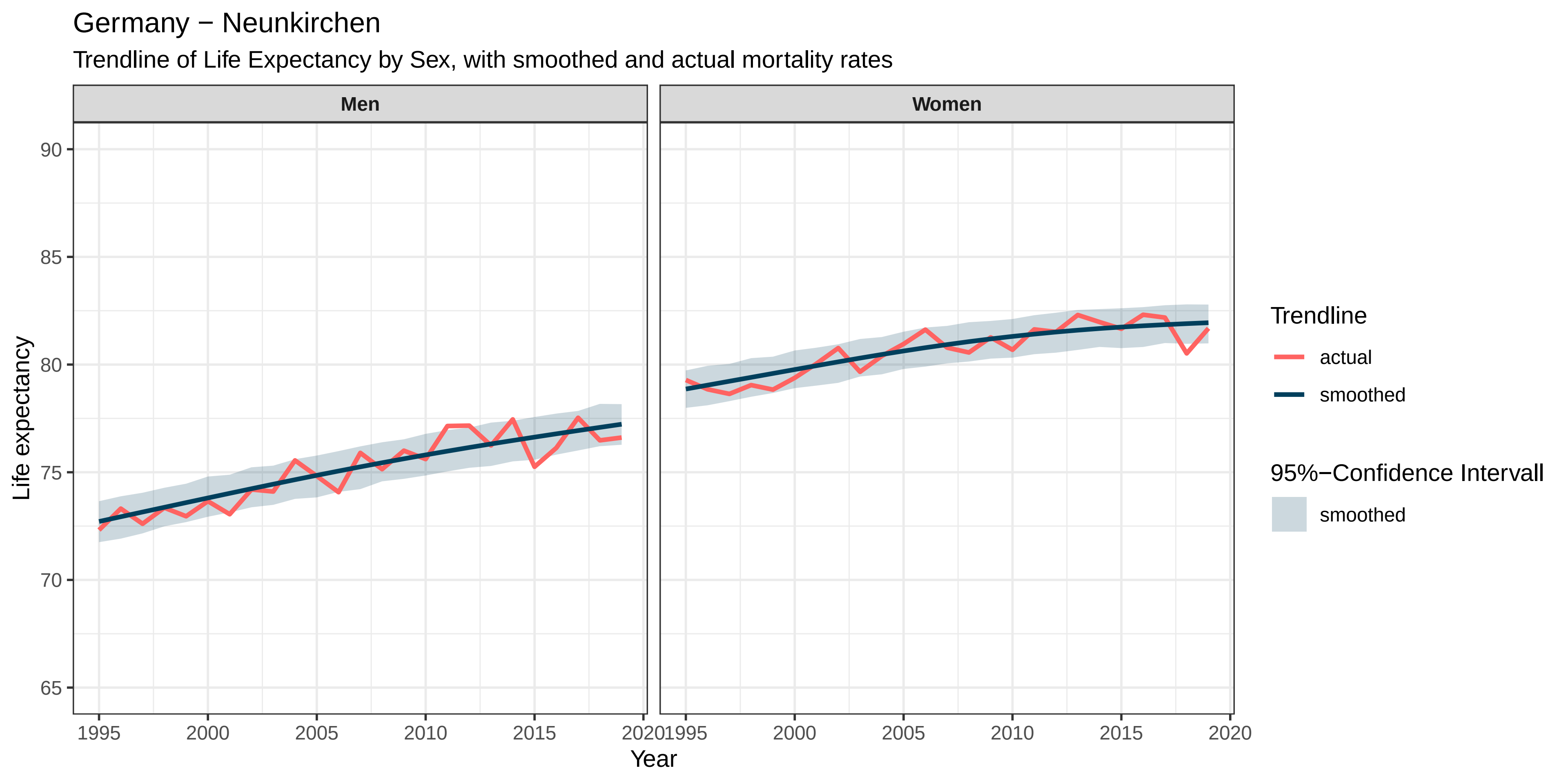
Germany − Neunkirchen
Trendline of Life Expectancy by Sex, with smoothed and actual mortality rates
Men
Women
Life expectancy
90
85
80
75
70
65
1995
2000
2005
2010
2015
2020
Year
Trendline
actual
smoothed
95%−Confidence Intervall
smoothed

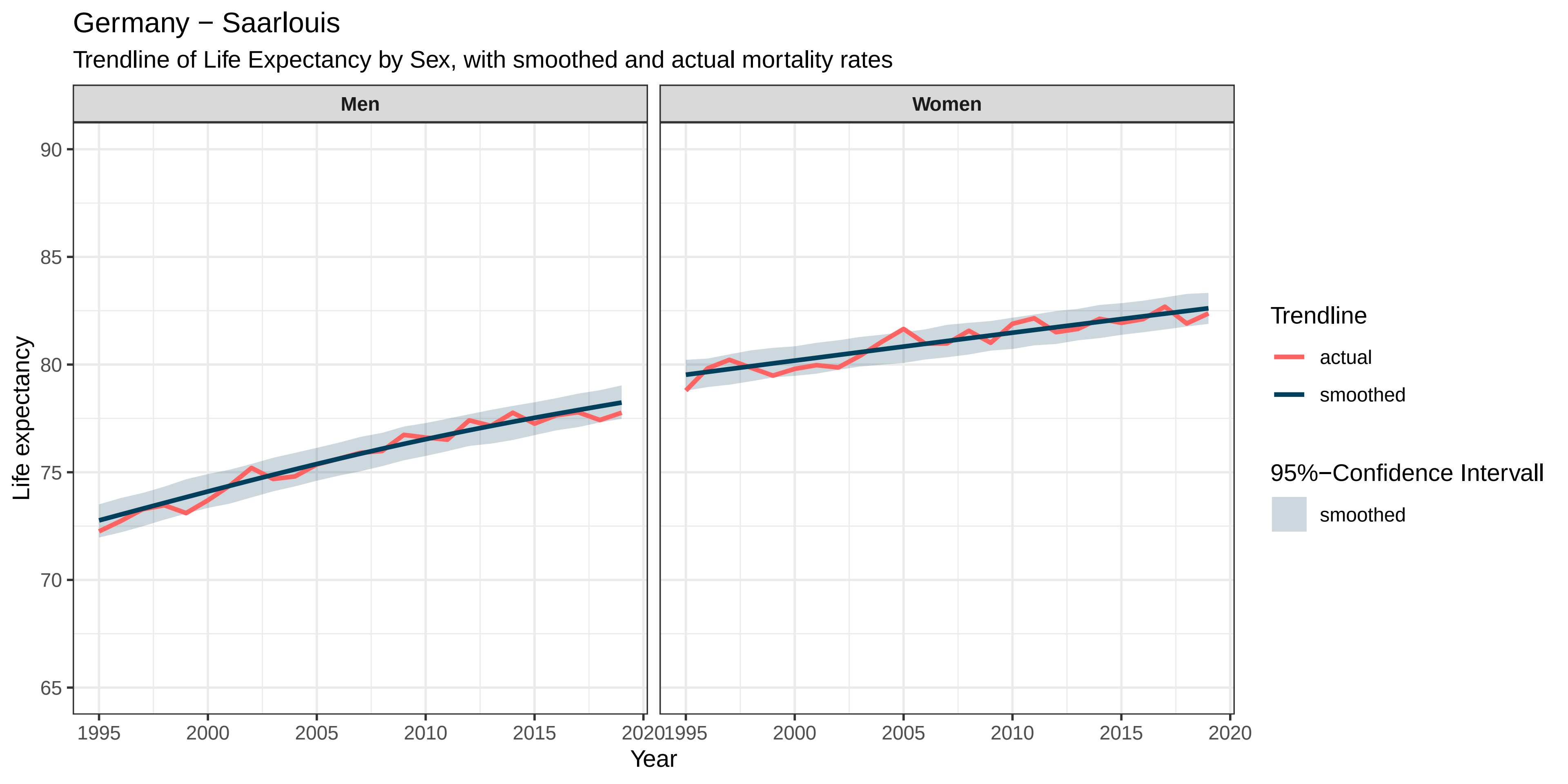

Germany − Saarlouis
Trendline of Life Expectancy by Sex, with smoothed and actual mortality rates
Men
Women
90
85
80
75
70
65
Life expectancy
1995
2000
2005
2010
2015
2020
Year
Trendline
actual
smoothed
95%−Confidence Intervall
smoothed

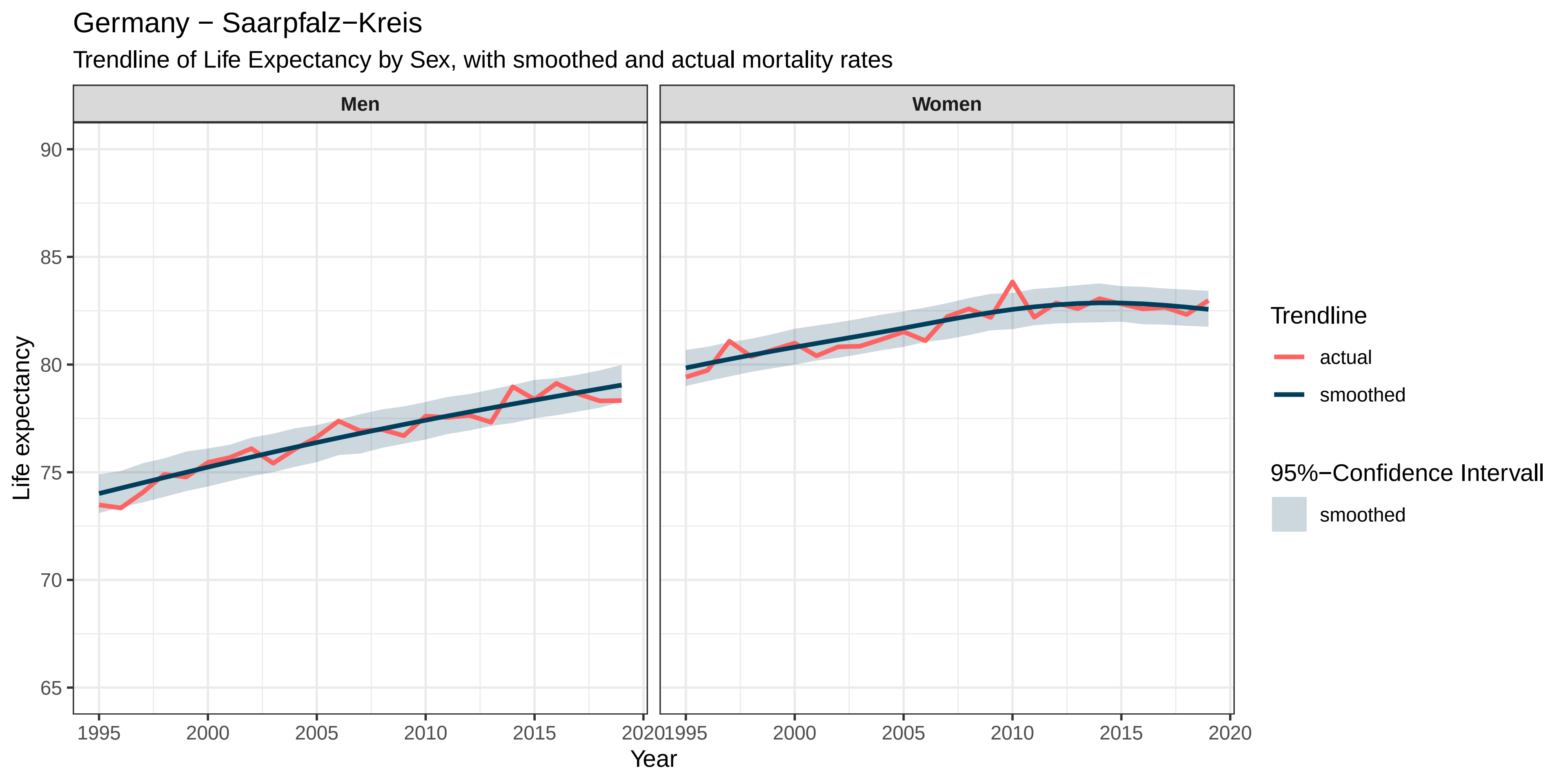
Germany − Saarpfalz−Kreis
Trendline of Life Expectancy by Sex, with smoothed and actual mortality rates
Men
Women
90
85
80
75
70
65
Life expectancy
1995
2000
2005
2010
2015
2020
Year
Trendline
actual
smoothed
95%−Confidence Intervall
smoothed

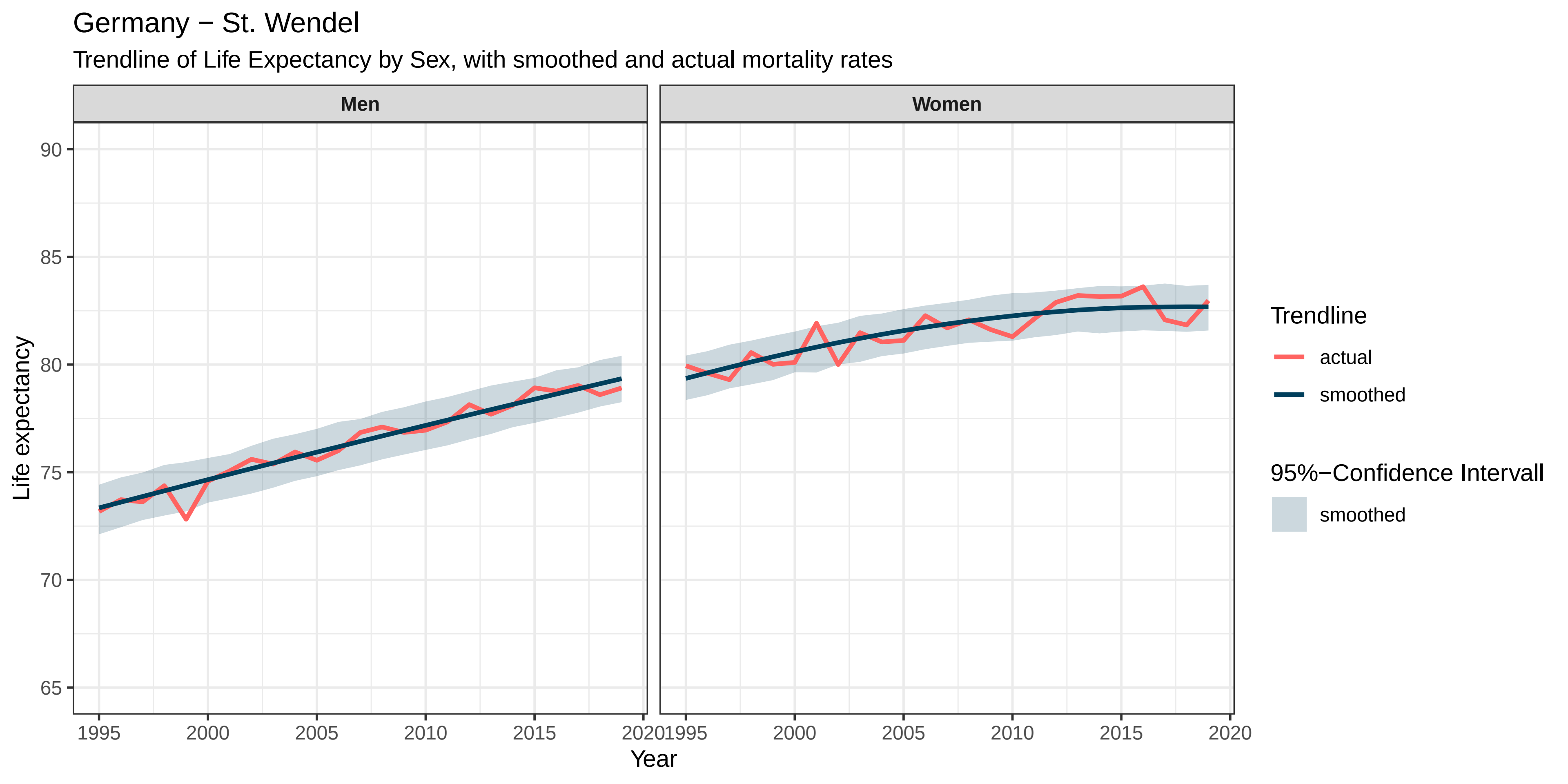
Germany − St. Wendel
Trendline of Life Expectancy by Sex, with smoothed and actual mortality rates
Men
Women
Life expectancy
Year
90
85
80
75
70
65
1995
2000
2005
2010
2015
2020
Trendline
actual
smoothed
95%−Confidence Intervall
smoothed

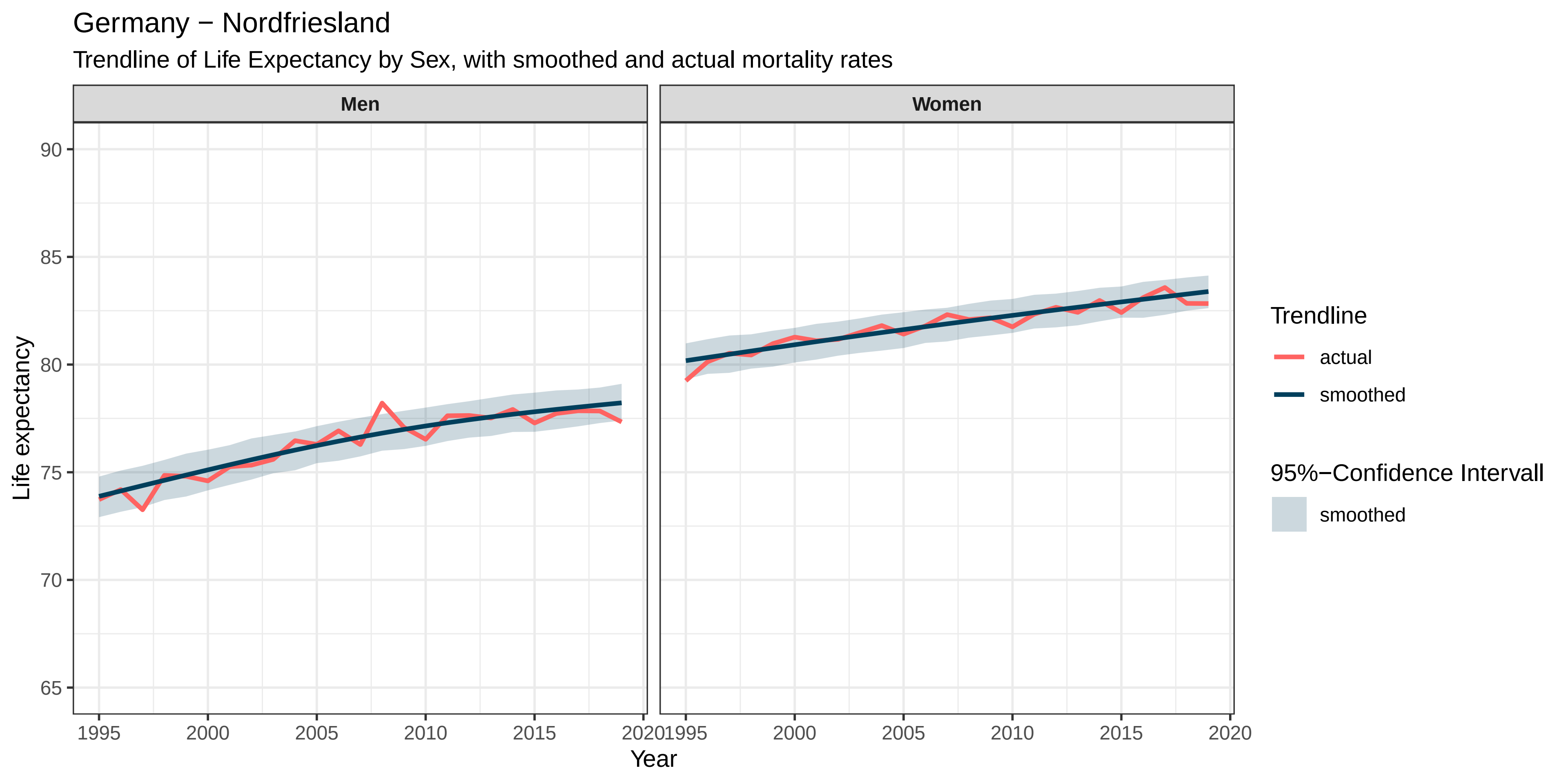

Germany − Nordfriesland
Trendline of Life Expectancy by Sex, with smoothed and actual mortality rates
Men
Women
90
85
80
75
70
65
Life expectancy
1995
2000
2005
2010
2015
2020
Year
Trendline
actual
smoothed
95%−Confidence Intervall
smoothed

Germany − Schleswig−Flensburg

Trendline of Life Expectancy by Sex, with smoothed and actual mortality rates

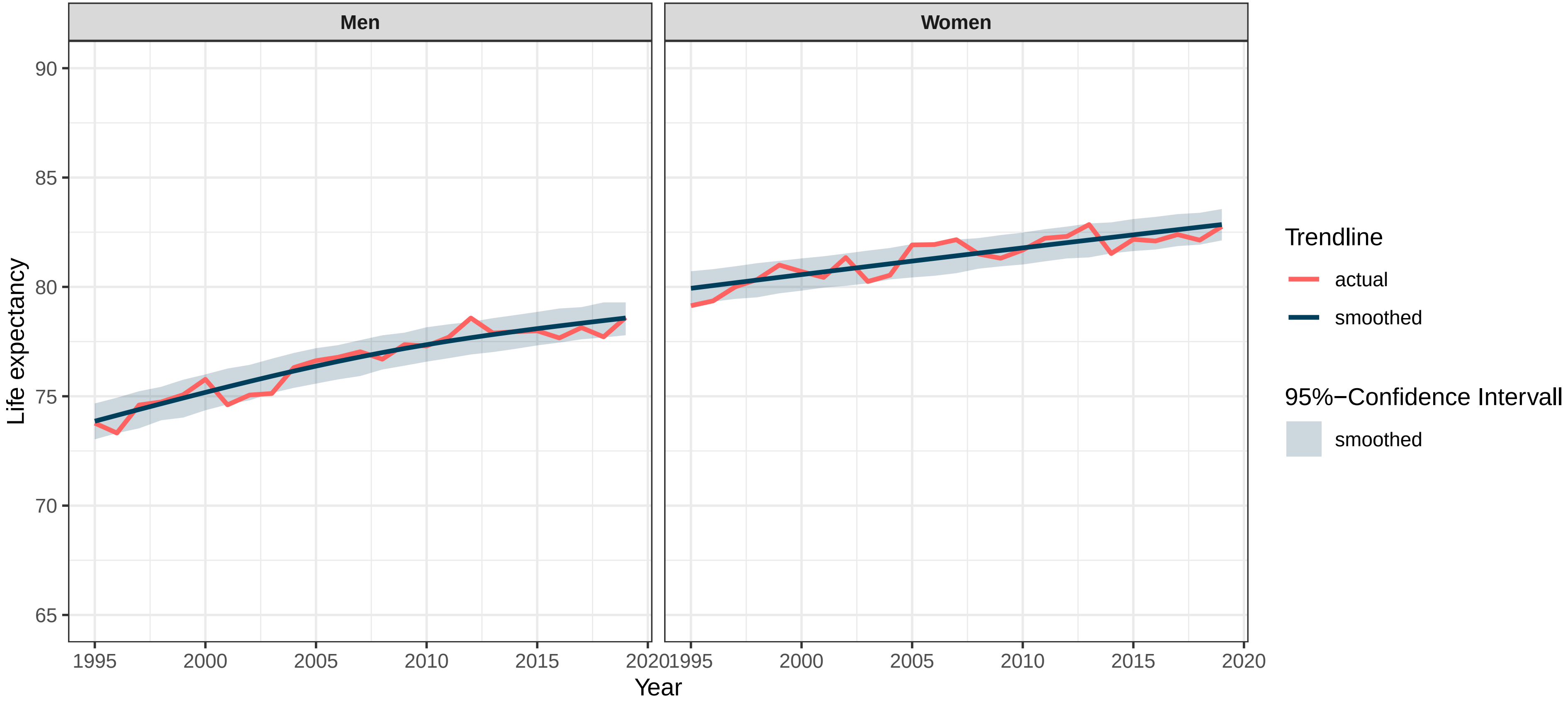

Germany − Cottbus, Stadt

Trendline of Life Expectancy by Sex, with smoothed and actual mortality rates

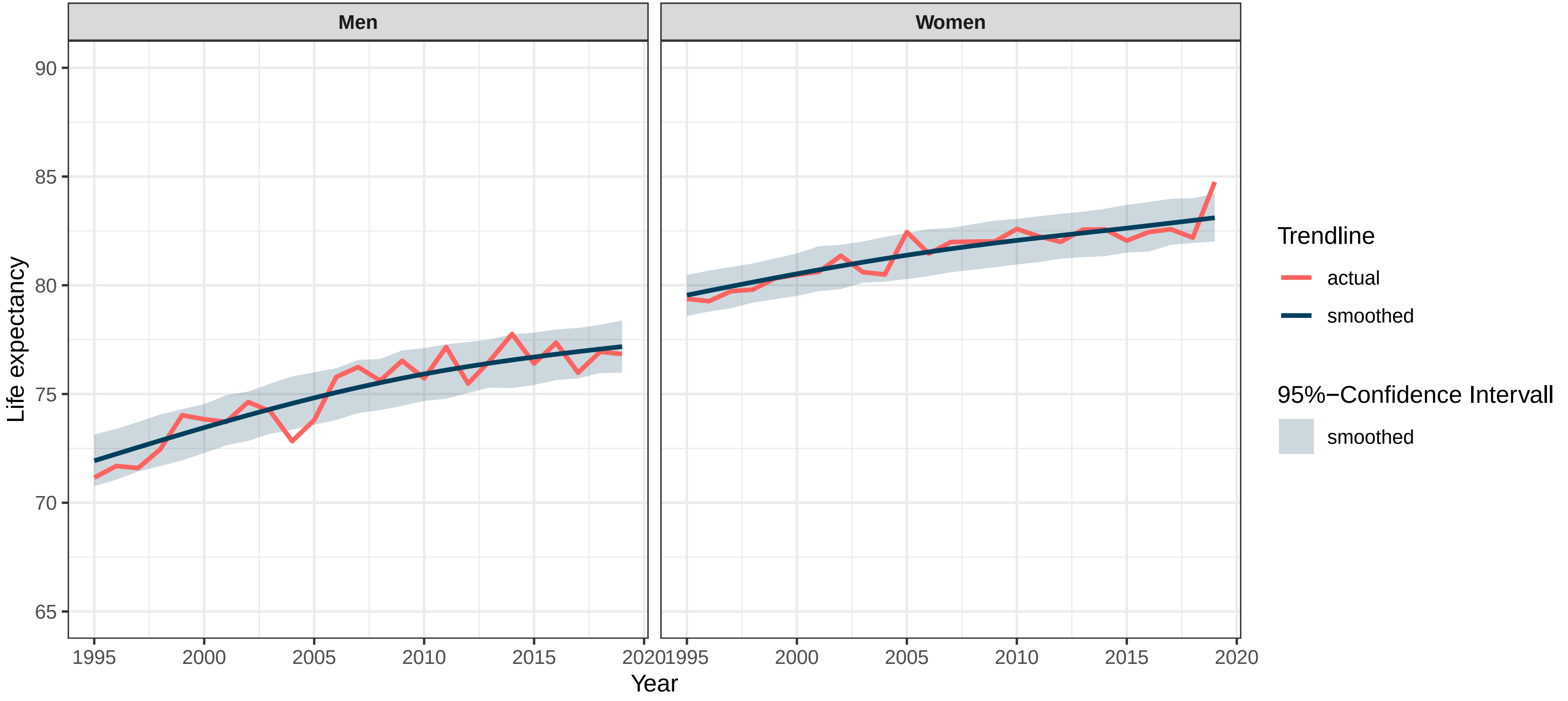

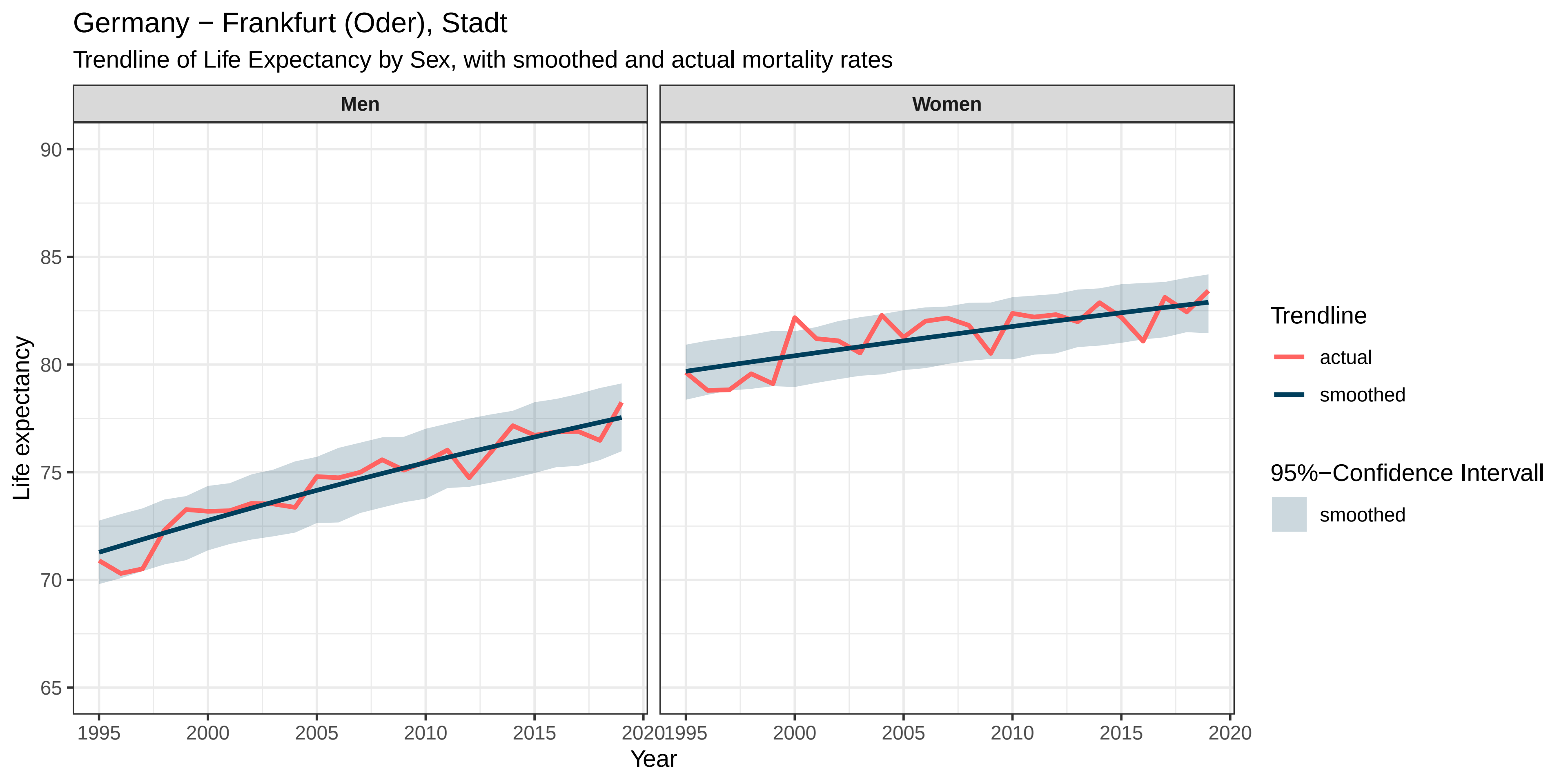
Germany − Frankfurt (Oder), Stadt
Trendline of Life Expectancy by Sex, with smoothed and actual mortality rates
Men
Women
Life expectancy
Year
65
70
75
80
85
90
1995
2000
2005
2010
2015
2020
Trendline
actual
smoothed
95%−Confidence Intervall
smoothed

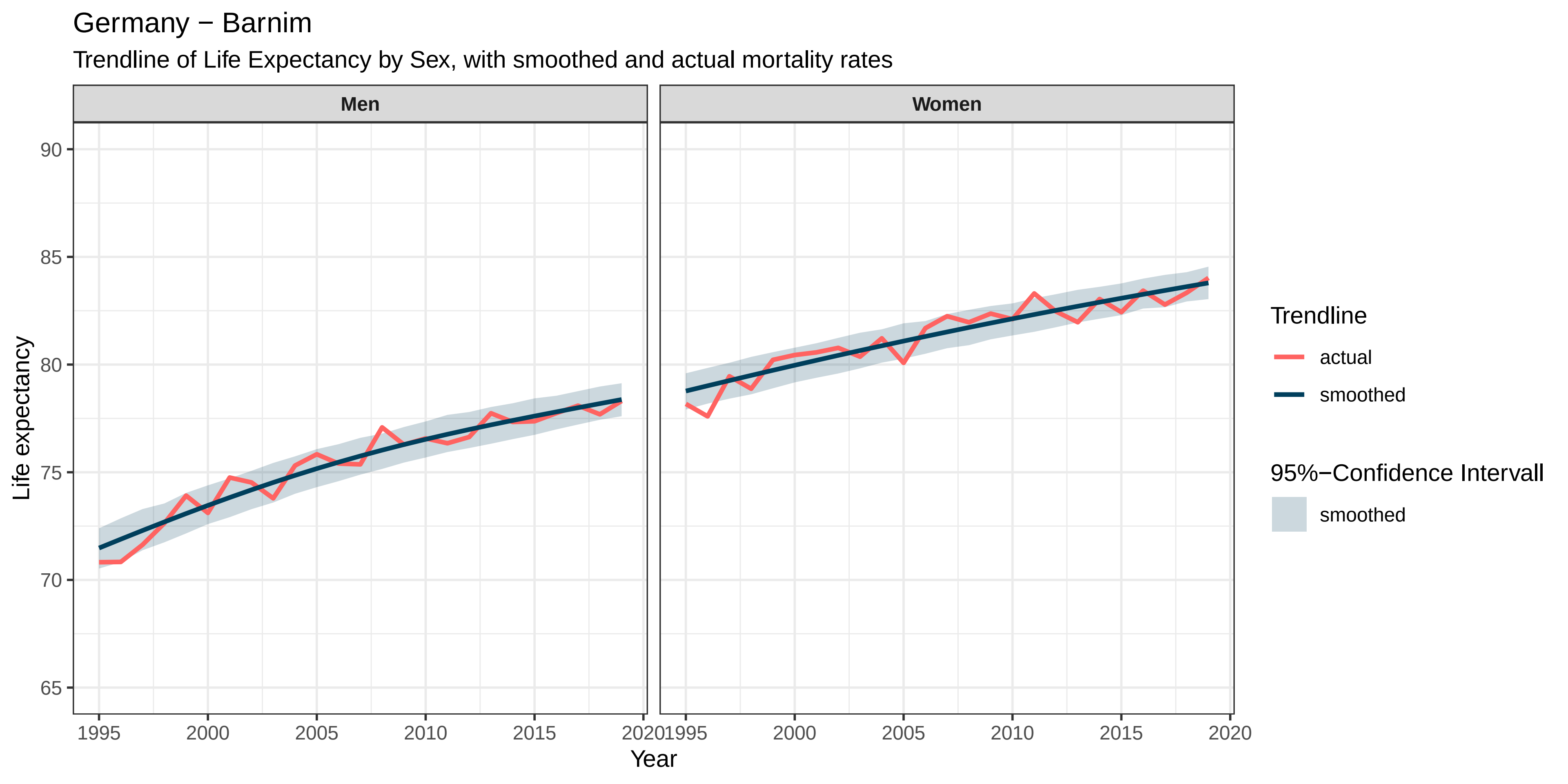
Germany − Barnim
Trendline of Life Expectancy by Sex, with smoothed and actual mortality rates
Men
Women
90
85
80
75
70
65
Life expectancy
1995
2000
2005
2010
2015
2020
Year
Trendline
actual
smoothed
95%−Confidence Intervall
smoothed

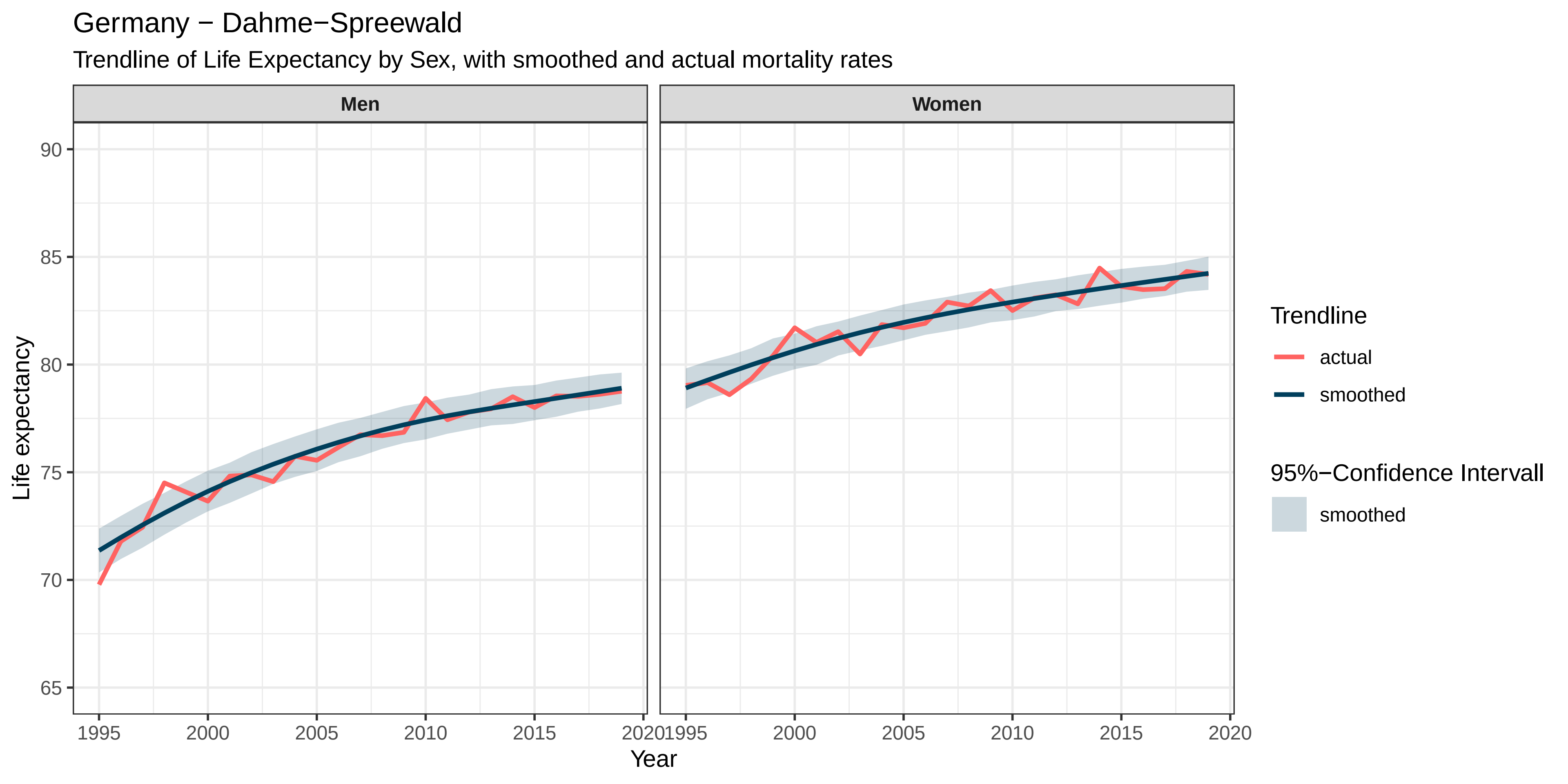
Germany − Dahme−Spreewald
Trendline of Life Expectancy by Sex, with smoothed and actual mortality rates
Men
Women
Life expectancy
Year
90
85
80
75
70
65
1995
2000
2005
2010
2015
2020
Trendline
actual
smoothed
95%−Confidence Intervall
smoothed

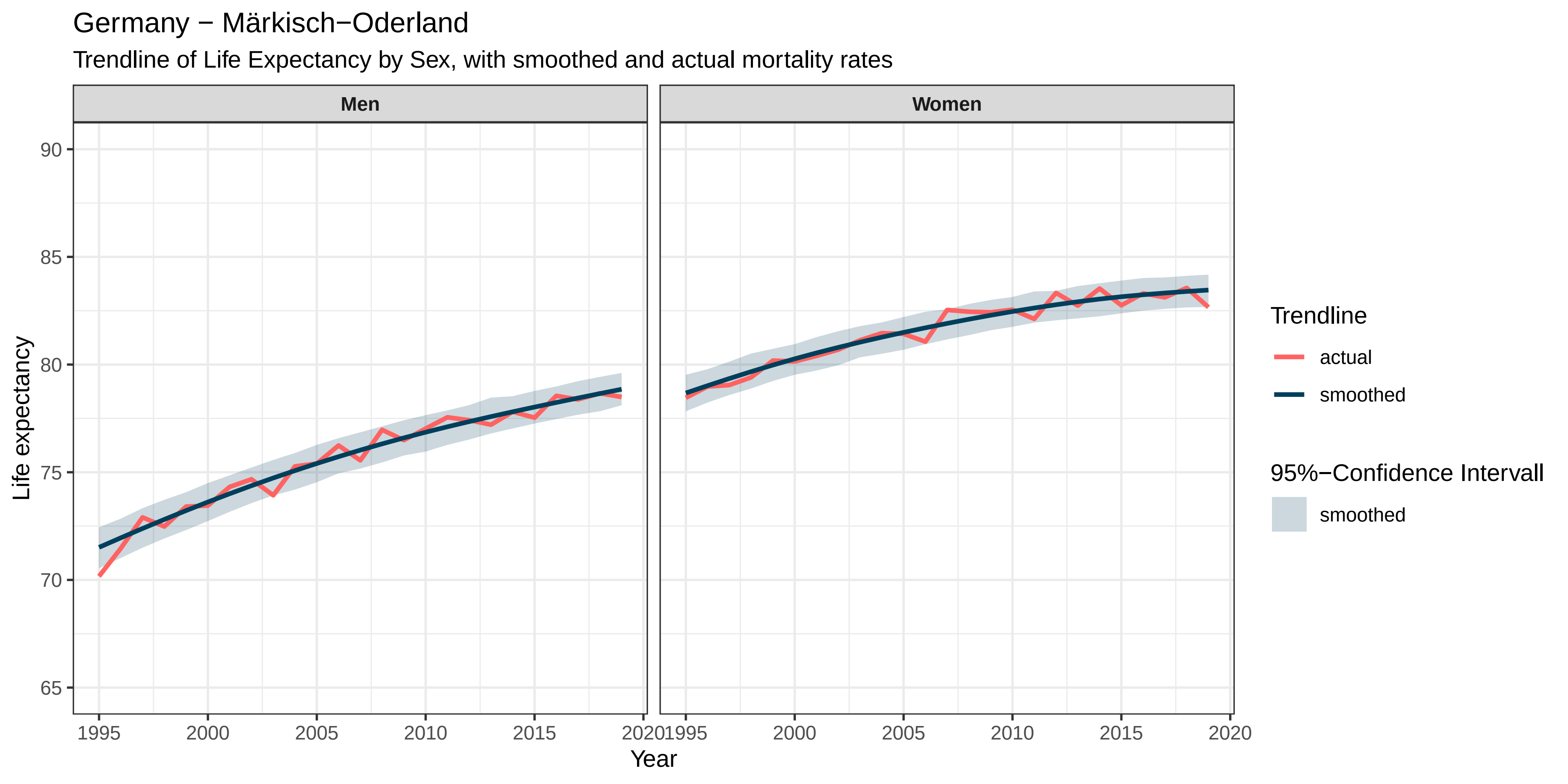

Germany − Märkisch−Oderland
Trendline of Life Expectancy by Sex, with smoothed and actual mortality rates
Men
Women
Life expectancy
90
85
80
75
70
65
1995
2000
2005
2010
2015
2020
Year
Trendline
actual
smoothed
95%−Confidence Intervall
smoothed

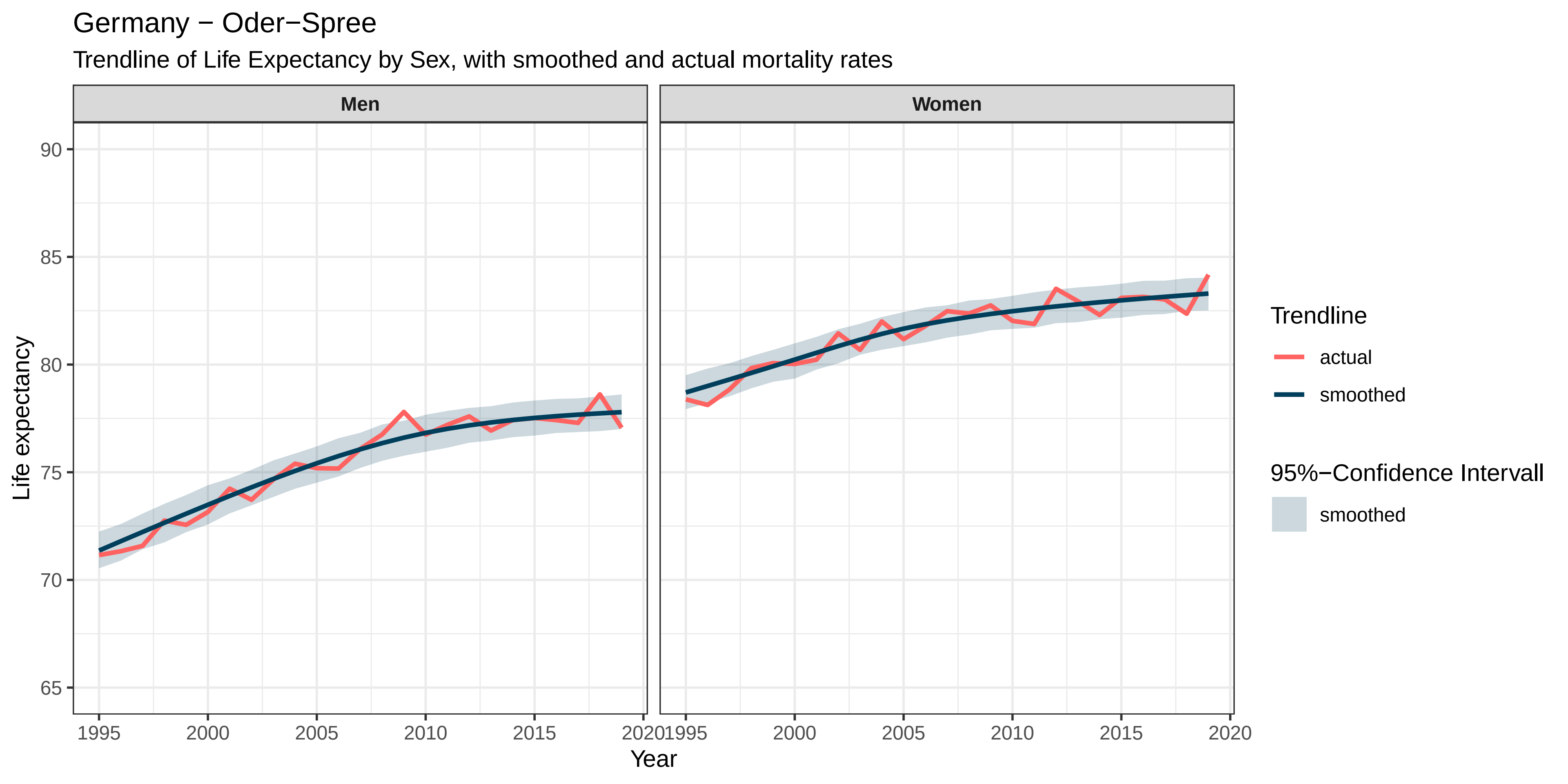
Germany − Oder−Spree
Trendline of Life Expectancy by Sex, with smoothed and actual mortality rates
Men
Women
90
85
80
75
70
65
Life expectancy
1995
2000
2005
2010
2015
2020
Year
Trendline
actual
smoothed
95%−Confidence Intervall
smoothed

Germany − Spree−Neiße

Trendline of Life Expectancy by Sex, with smoothed and actual mortality rates

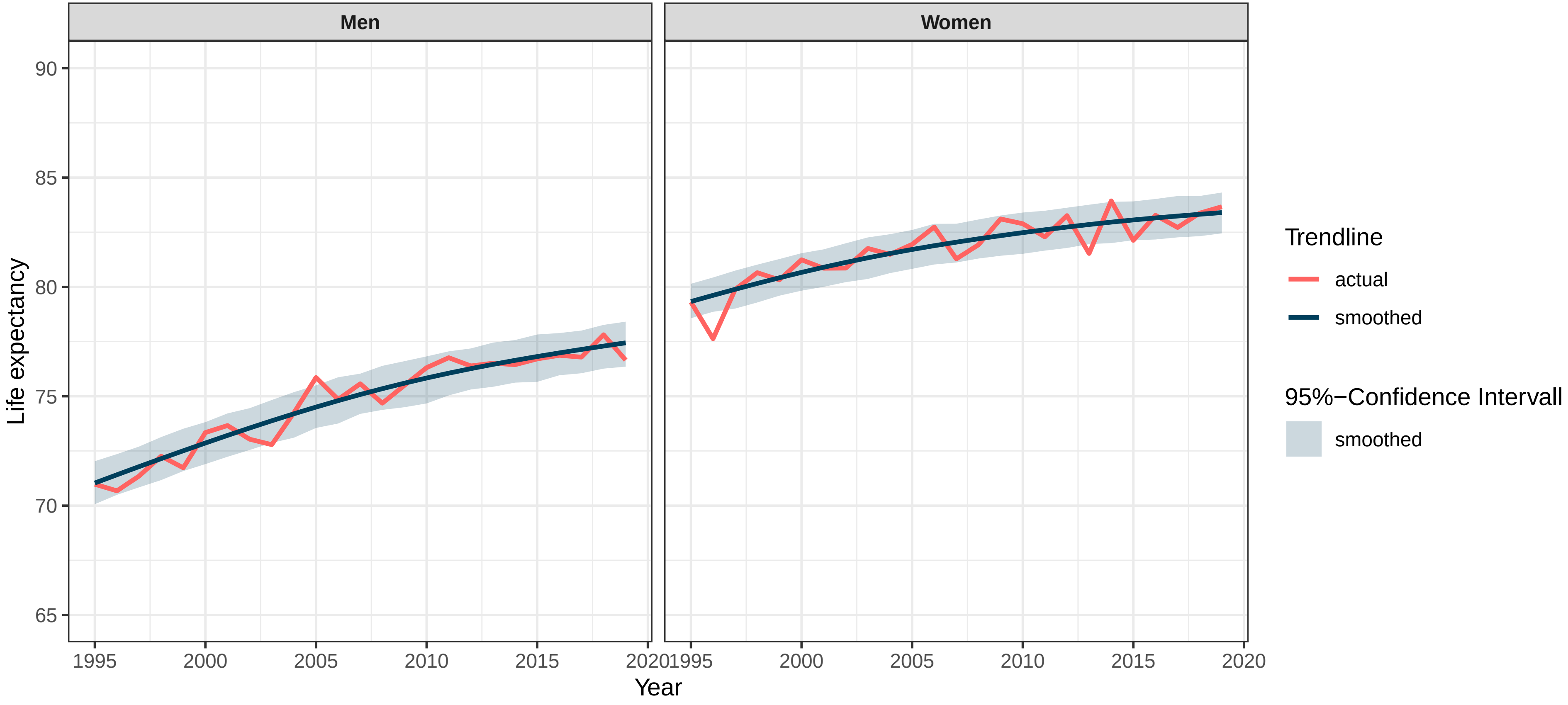

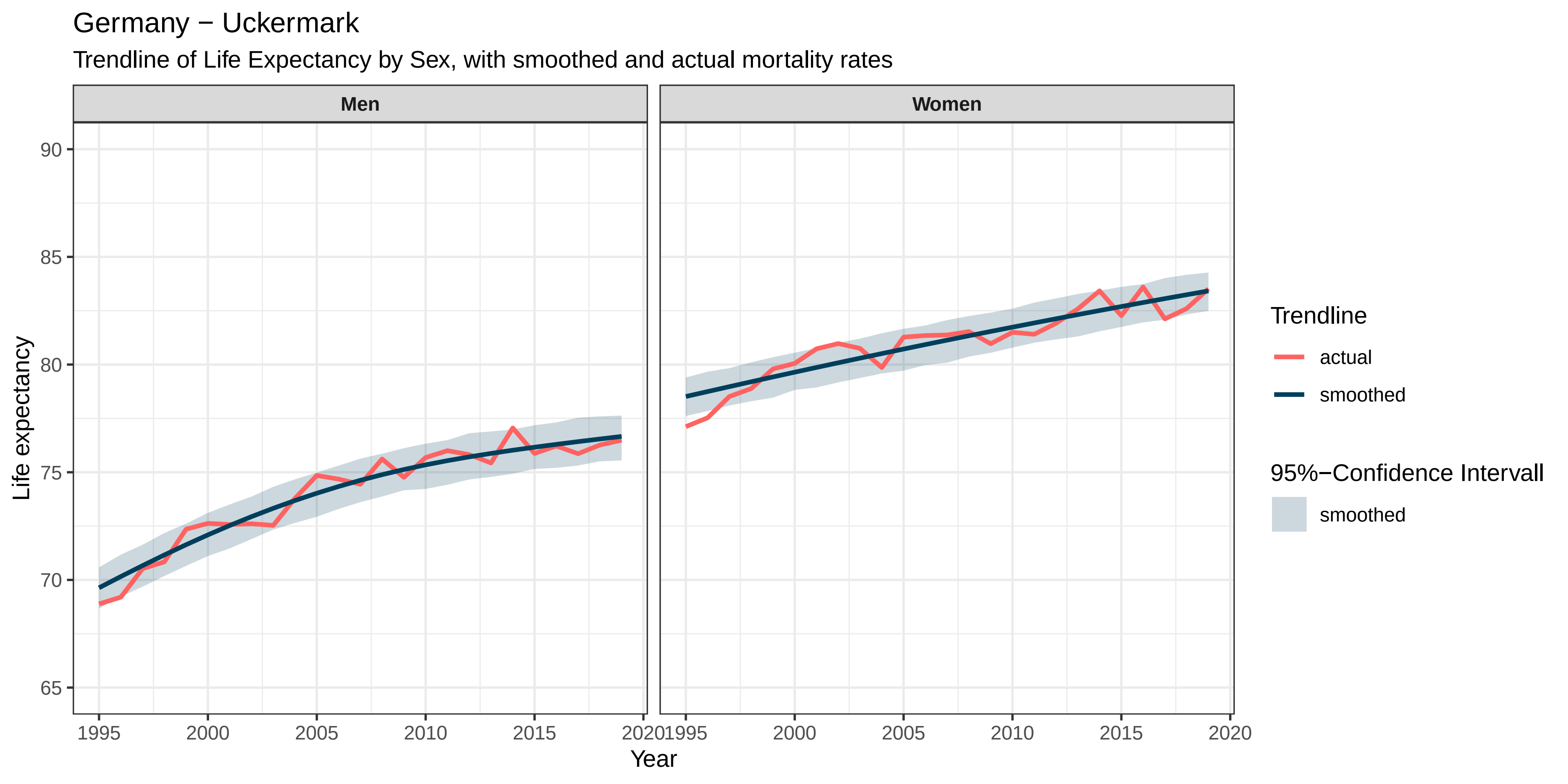

Germany − Uckermark
Trendline of Life Expectancy by Sex, with smoothed and actual mortality rates
Men
Women
Life expectancy
90
85
80
75
70
65
1995
2000
2005
2010
2015
2020
Year
Trendline
actual
smoothed
95%−Confidence Intervall
smoothed

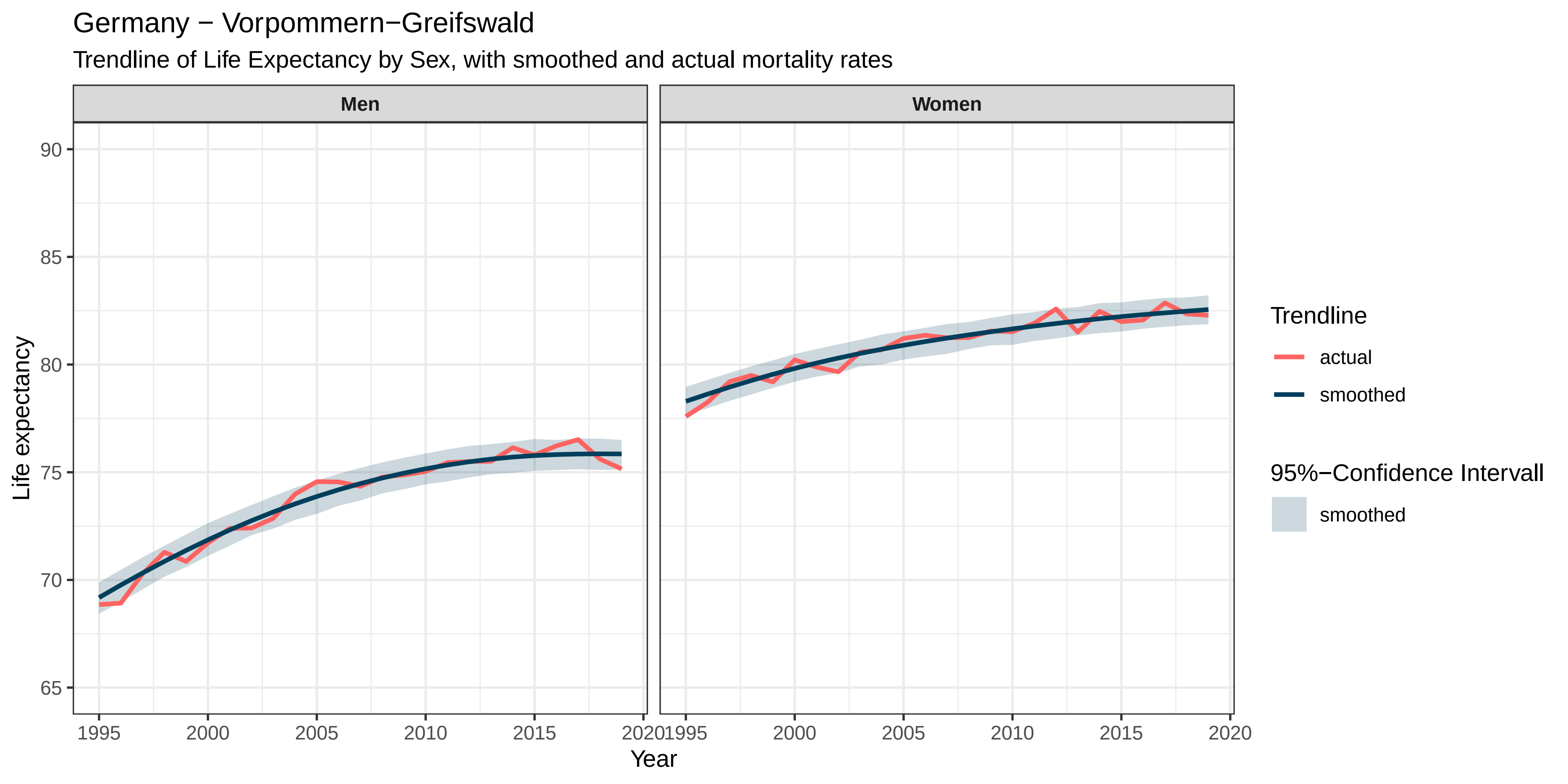

Germany − Vorpommern−Greifswald
Trendline of Life Expectancy by Sex, with smoothed and actual mortality rates
Men
Women
Life expectancy
90
85
80
75
70
65
1995
2000
2005
2010
2015
2020
Year
Trendline
actual
smoothed
95%−Confidence Intervall
smoothed

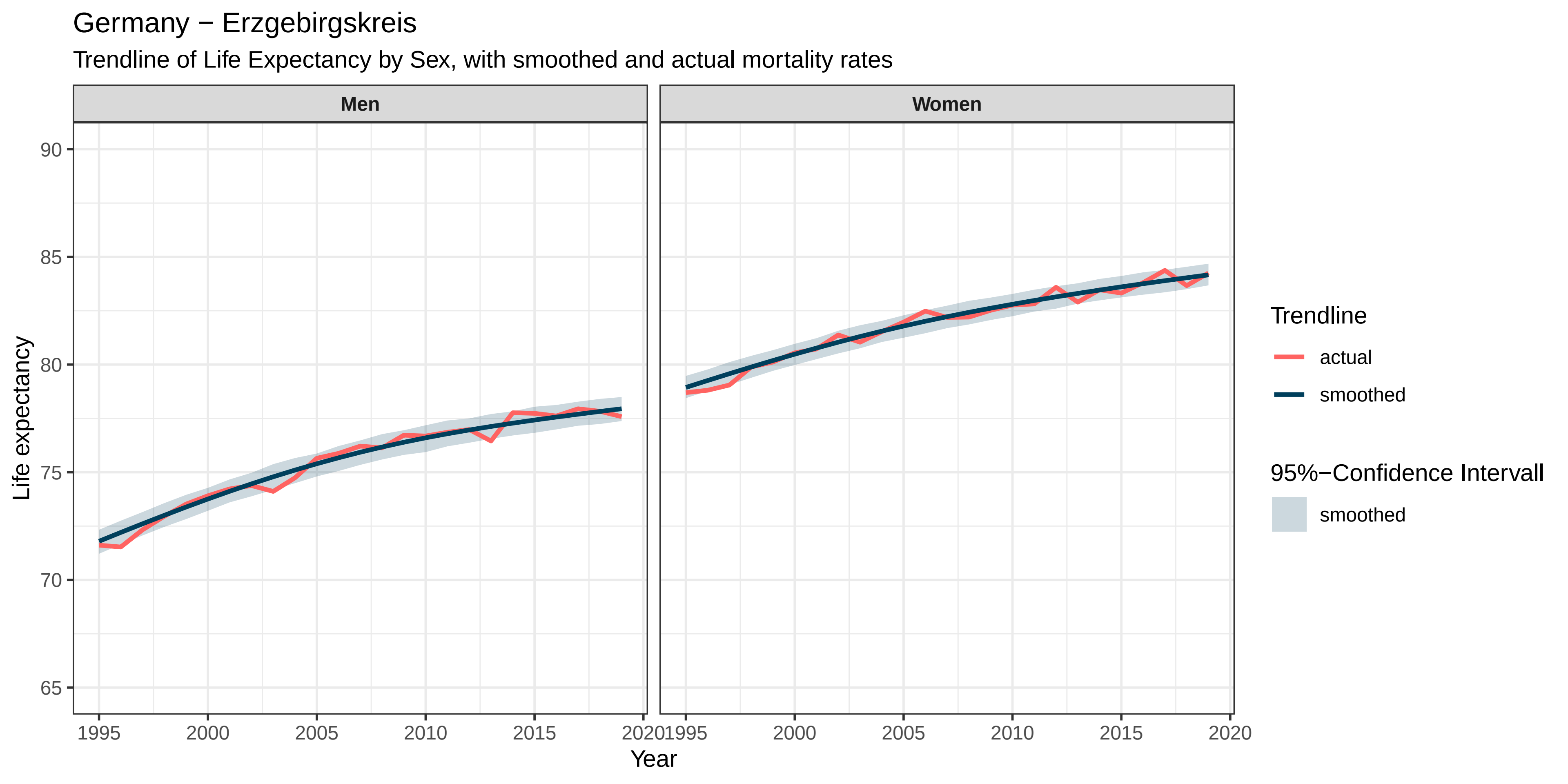

Germany − Erzgebirgskreis
Trendline of Life Expectancy by Sex, with smoothed and actual mortality rates
Men
Women
90
85
80
75
70
65
Life expectancy
1995
2000
2005
2010
2015
2020
Year
Trendline
actual
smoothed
95%−Confidence Intervall
smoothed

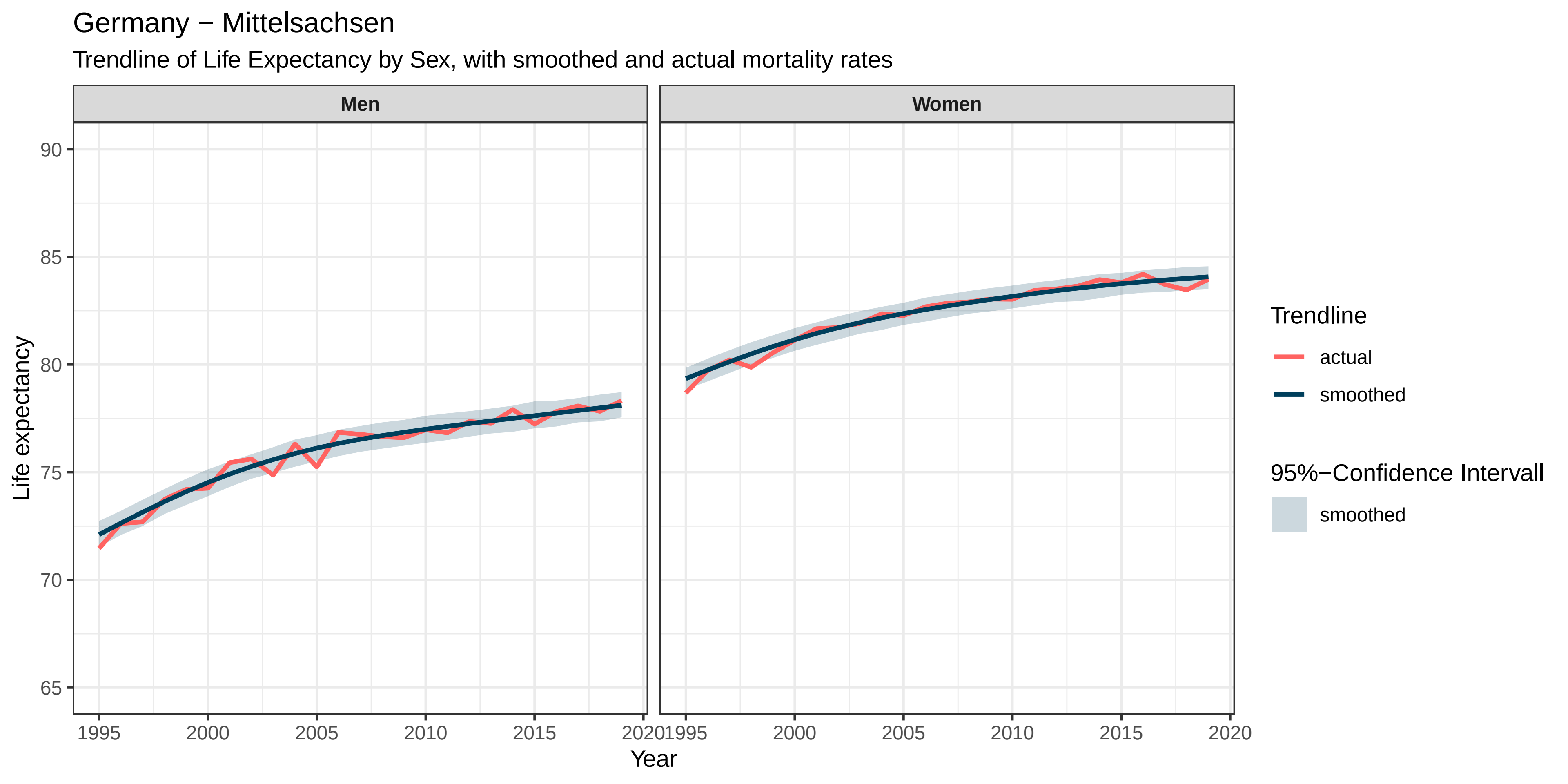

Germany − Mittelsachsen
Trendline of Life Expectancy by Sex, with smoothed and actual mortality rates
Men
Women
Life expectancy
90
85
80
75
70
65
1995
2000
2005
2010
2015
2020
Year
Trendline
actual
smoothed
95%−Confidence Intervall
smoothed

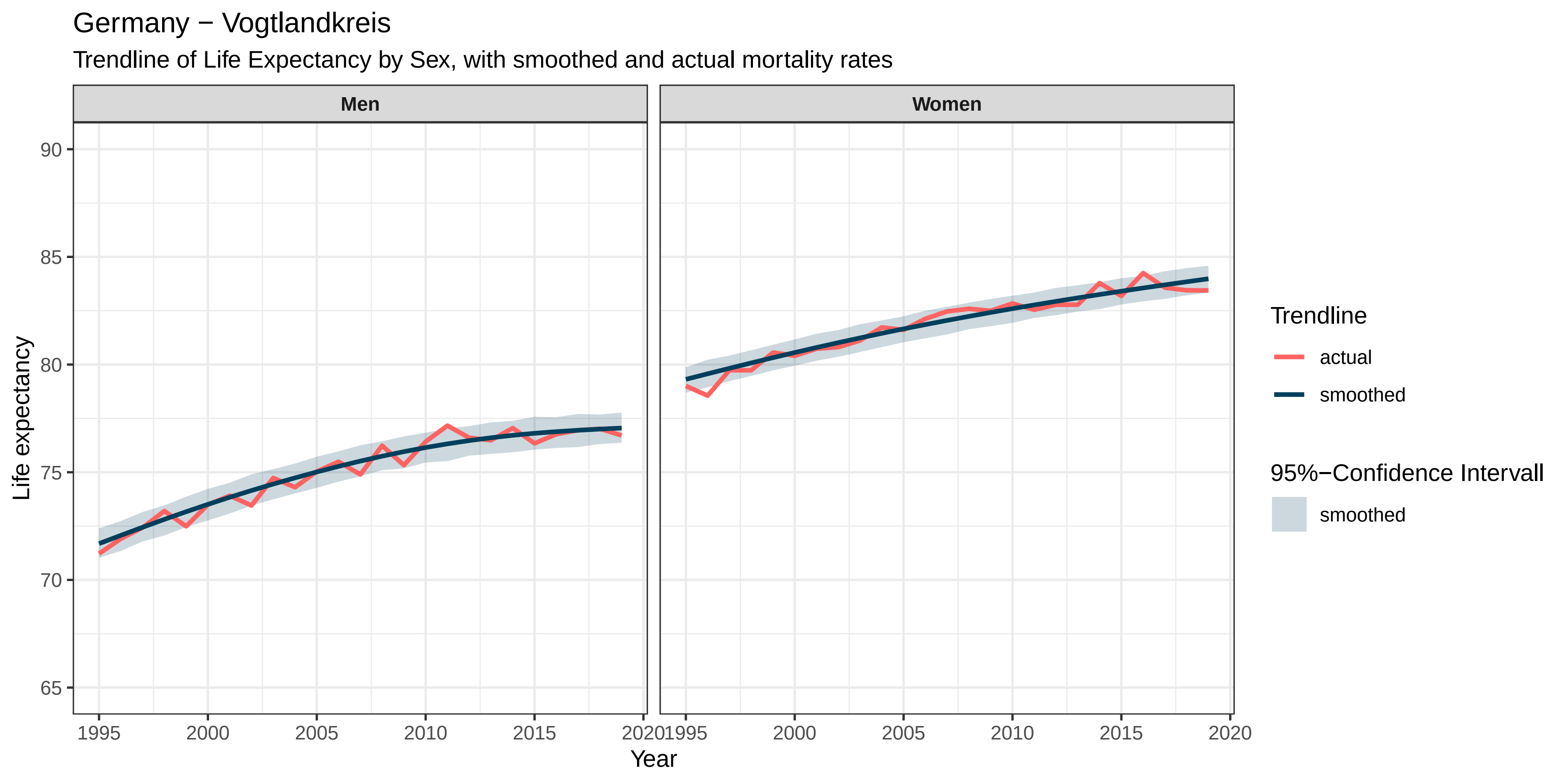

Germany − Vogtlandkreis
Trendline of Life Expectancy by Sex, with smoothed and actual mortality rates
Men
Women
Life expectancy
90
85
80
75
70
65
1995
2000
2005
2010
2015
2020
Year
Trendline
actual
smoothed
95%−Confidence Intervall
smoothed

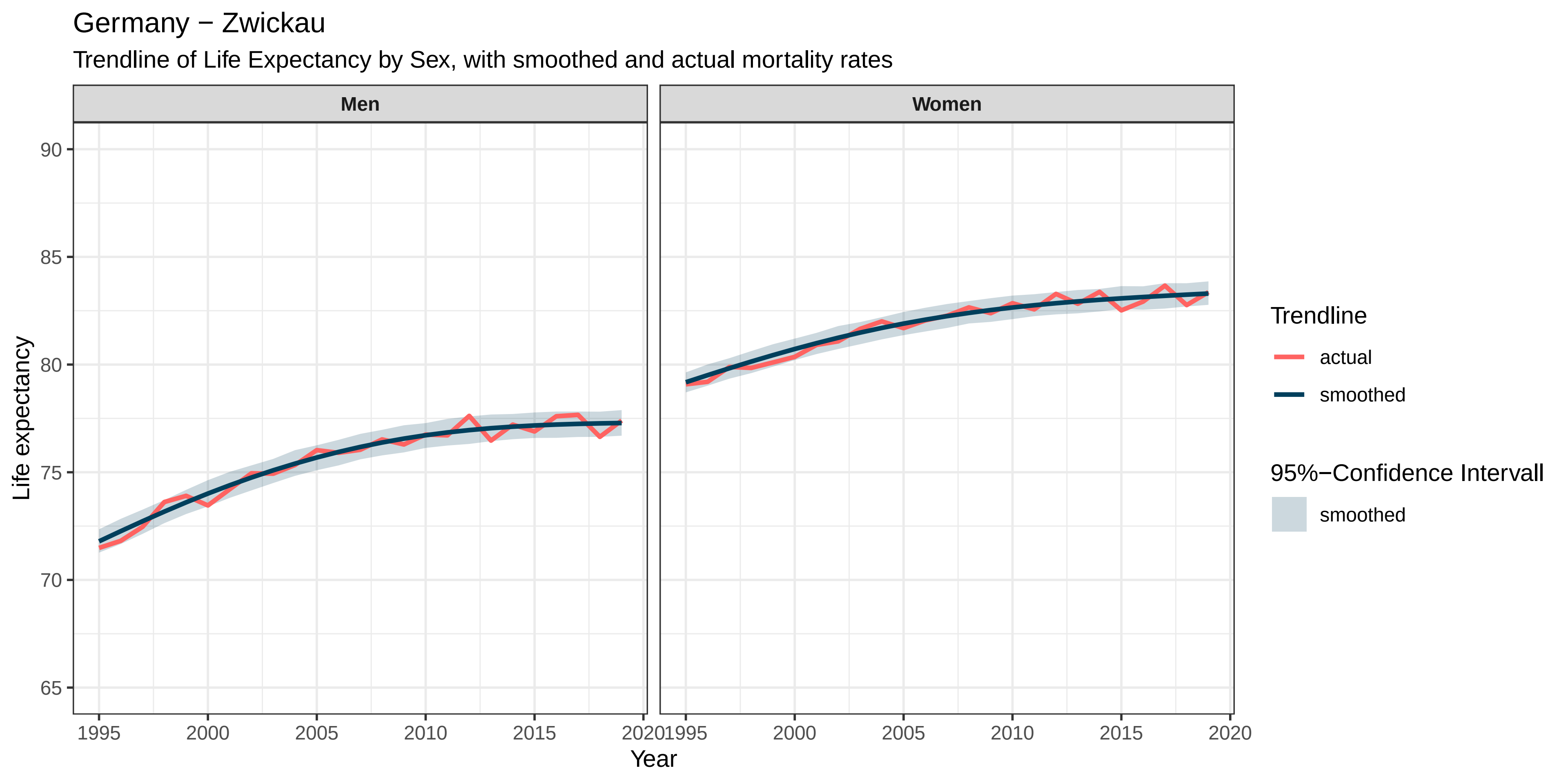

Germany − Zwickau
Trendline of Life Expectancy by Sex, with smoothed and actual mortality rates
Men
Women
90
85
80
75
70
65
Life expectancy
1995
2000
2005
2010
2015
2020
Year
Trendline
actual
smoothed
95%−Confidence Intervall
smoothed

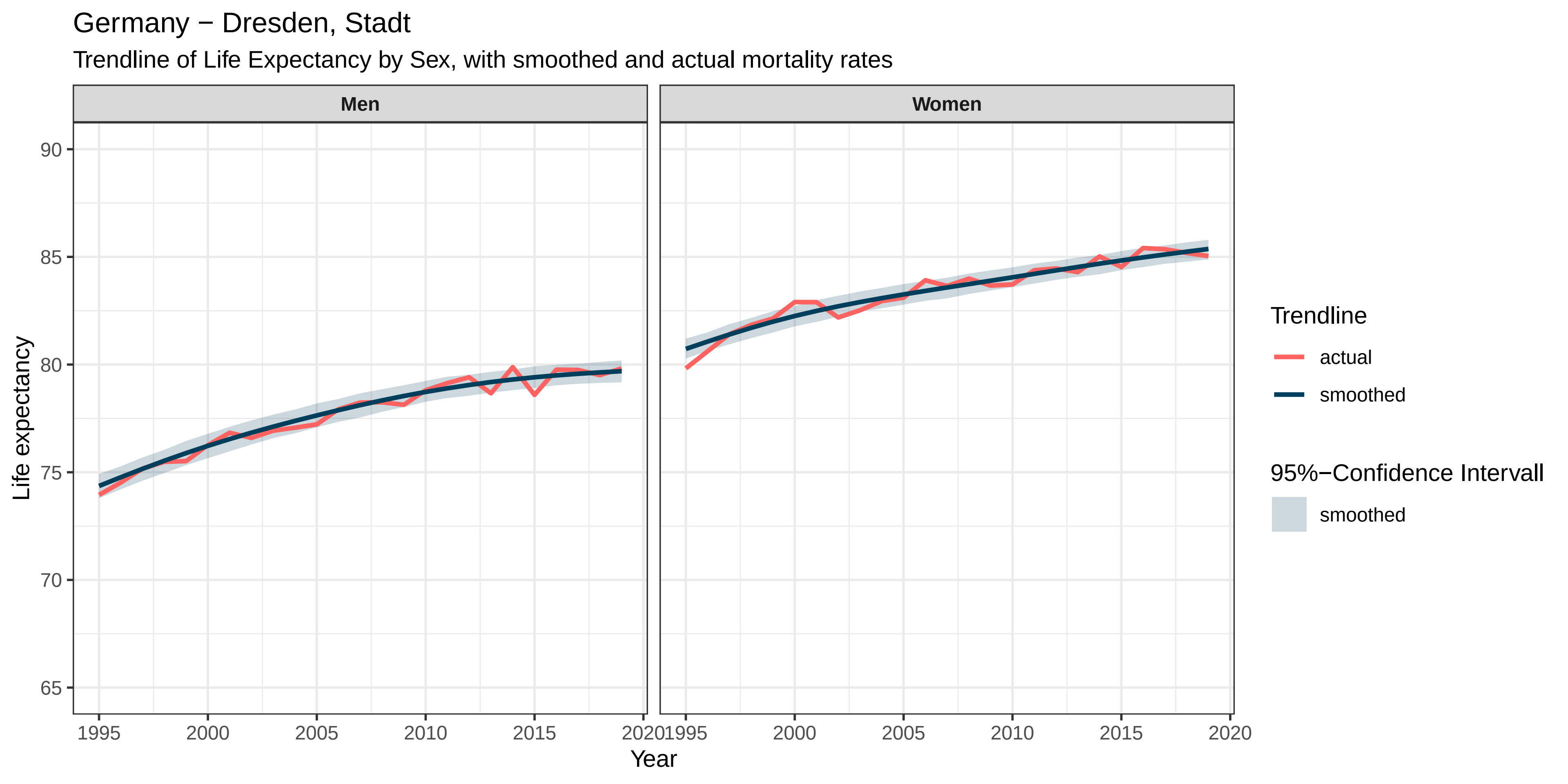

Germany − Dresden, Stadt
Trendline of Life Expectancy by Sex, with smoothed and actual mortality rates
Men
Women
90
85
80
75
70
65
1995
2000
2005
2010
2015
2020
Year
Life expectancy
Trendline
actual
smoothed
95%−Confidence Intervall
smoothed

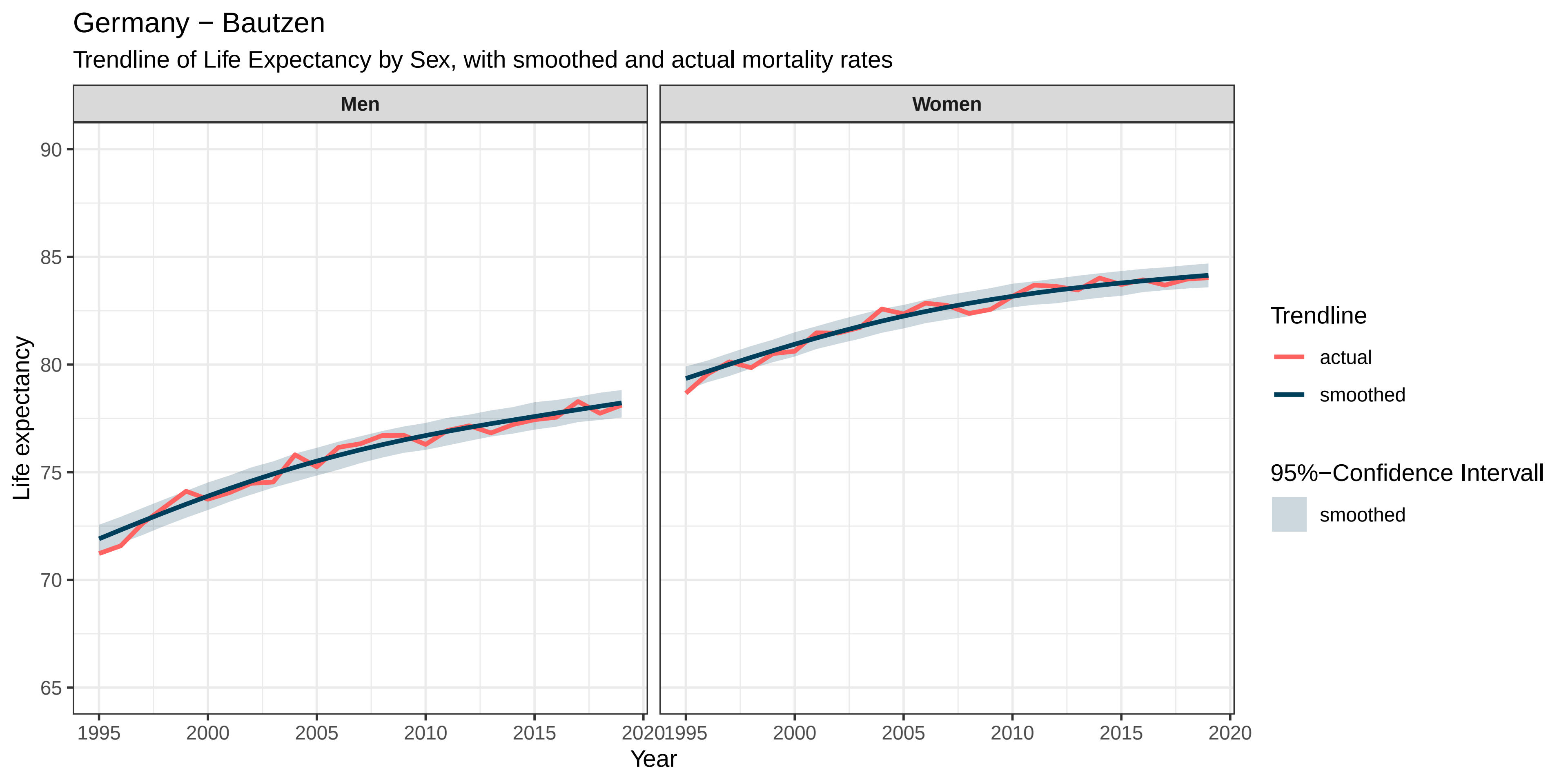

Germany − Bautzen
Trendline of Life Expectancy by Sex, with smoothed and actual mortality rates
Men
Women
90
85
80
75
70
65
1995
2000
2005
2010
2015
2020
Year
Life expectancy
Trendline
actual
smoothed
95%−Confidence Intervall
smoothed

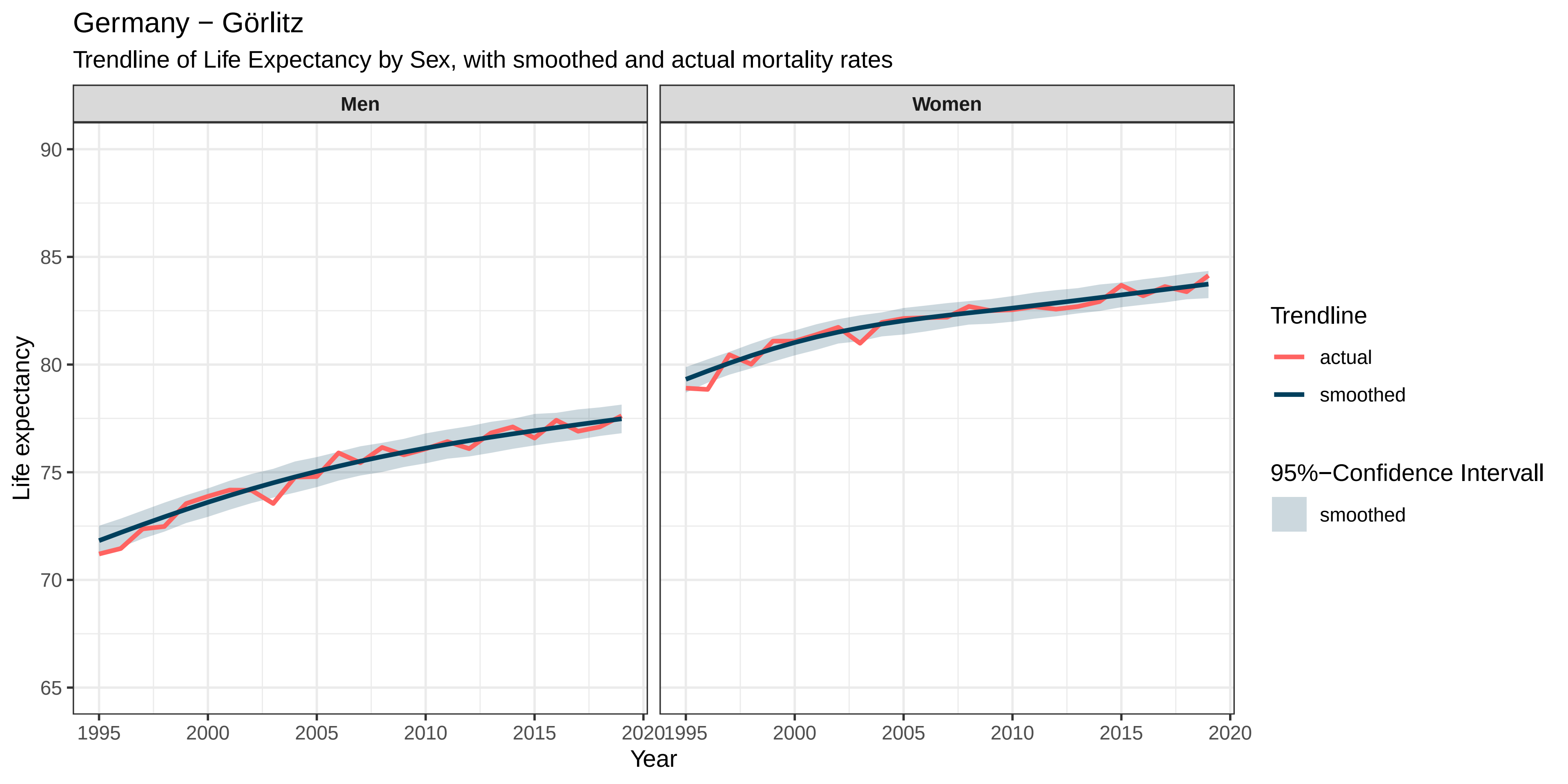

Germany − Görlitz
Trendline of Life Expectancy by Sex, with smoothed and actual mortality rates
Men
Women
Life expectancy
90
85
80
75
70
65
1995
2000
2005
2010
2015
2020
Year
Trendline
actual
smoothed
95%−Confidence Intervall
smoothed

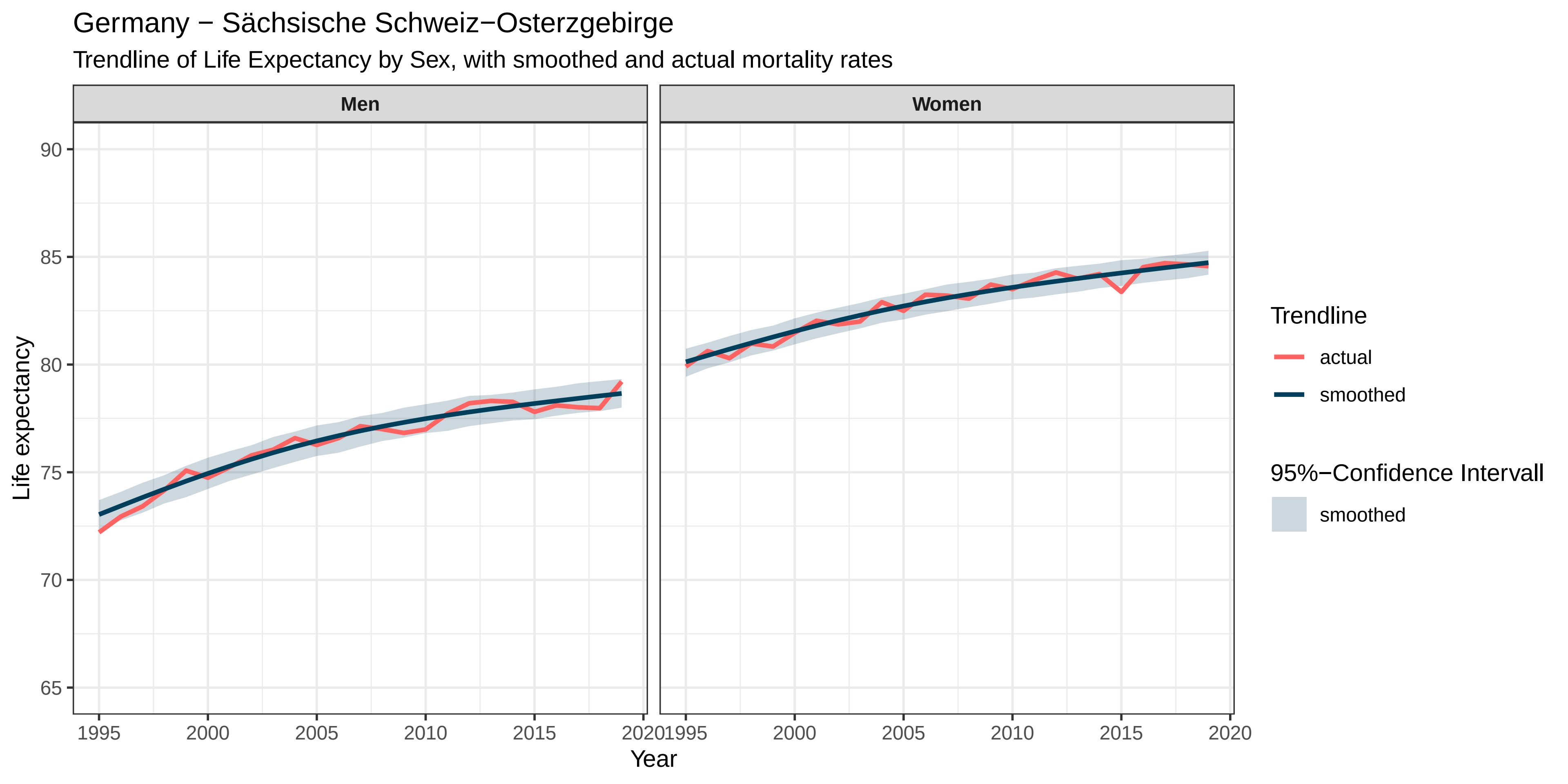
Germany − Sächsische Schweiz−Osterzgebirge
Trendline of Life Expectancy by Sex, with smoothed and actual mortality rates
Men
Women
Life expectancy
90
85
80
75
70
65
1995
2000
2005
2010
2015
2020
Year
Trendline
actual
smoothed
95%−Confidence Intervall
smoothed

Germany − Saale−Orla−Kreis

Trendline of Life Expectancy by Sex, with smoothed and actual mortality rates

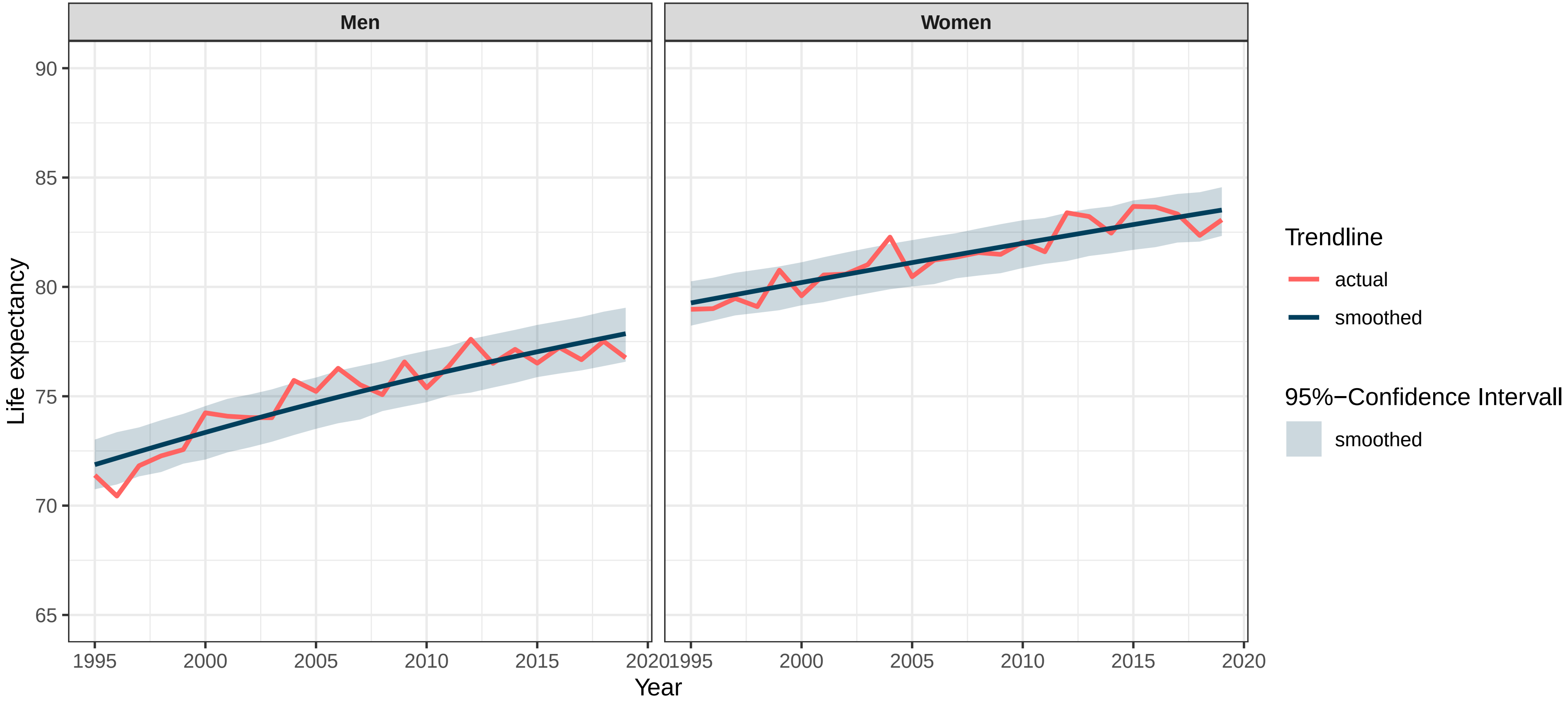

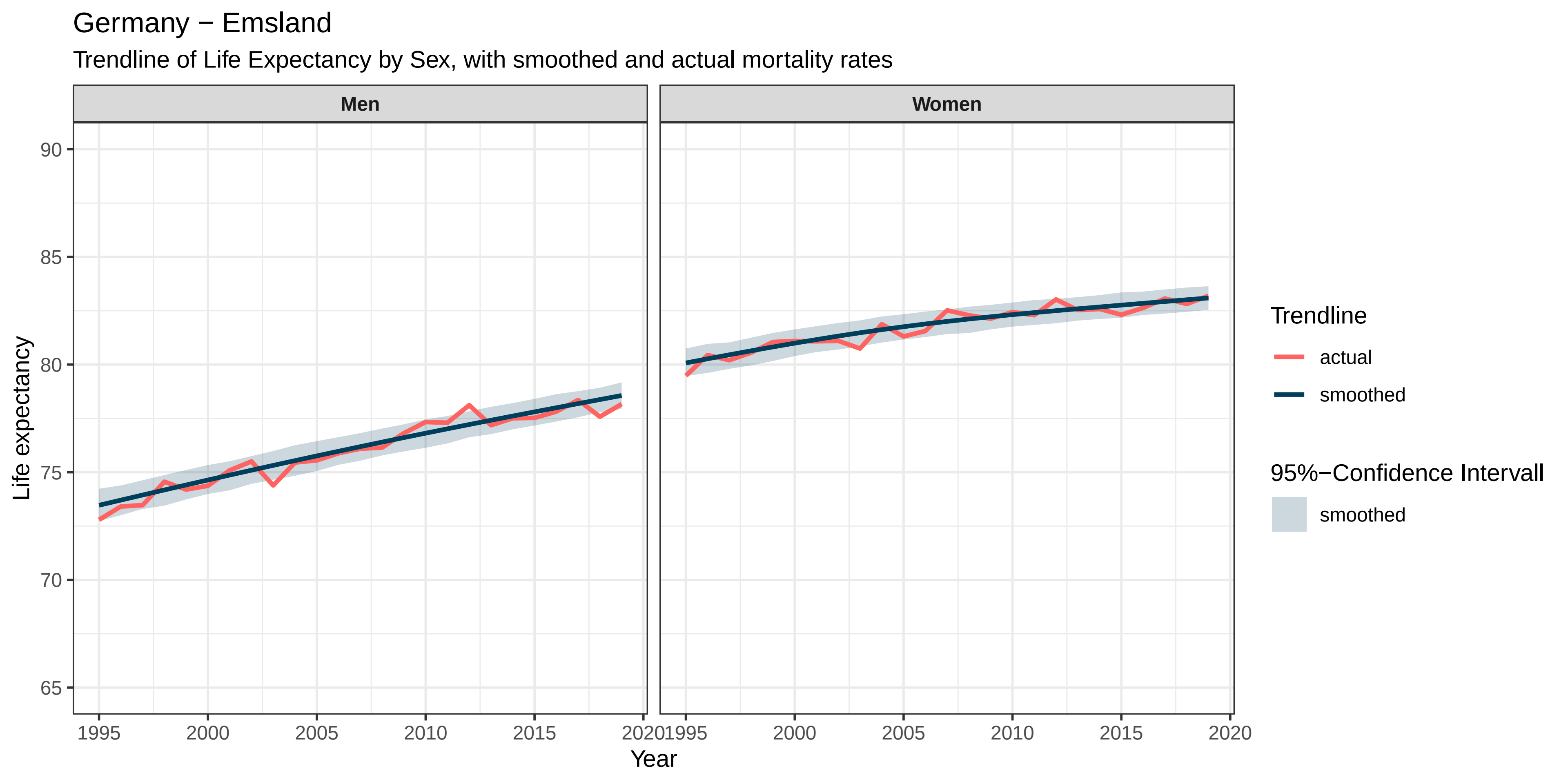
Germany − Emsland
Trendline of Life Expectancy by Sex, with smoothed and actual mortality rates
Men
Women
Life expectancy
90
85
80
75
70
65
1995
2000
2005
2010
2015
2020
Year
Trendline
actual
smoothed
95%−Confidence Intervall
smoothed

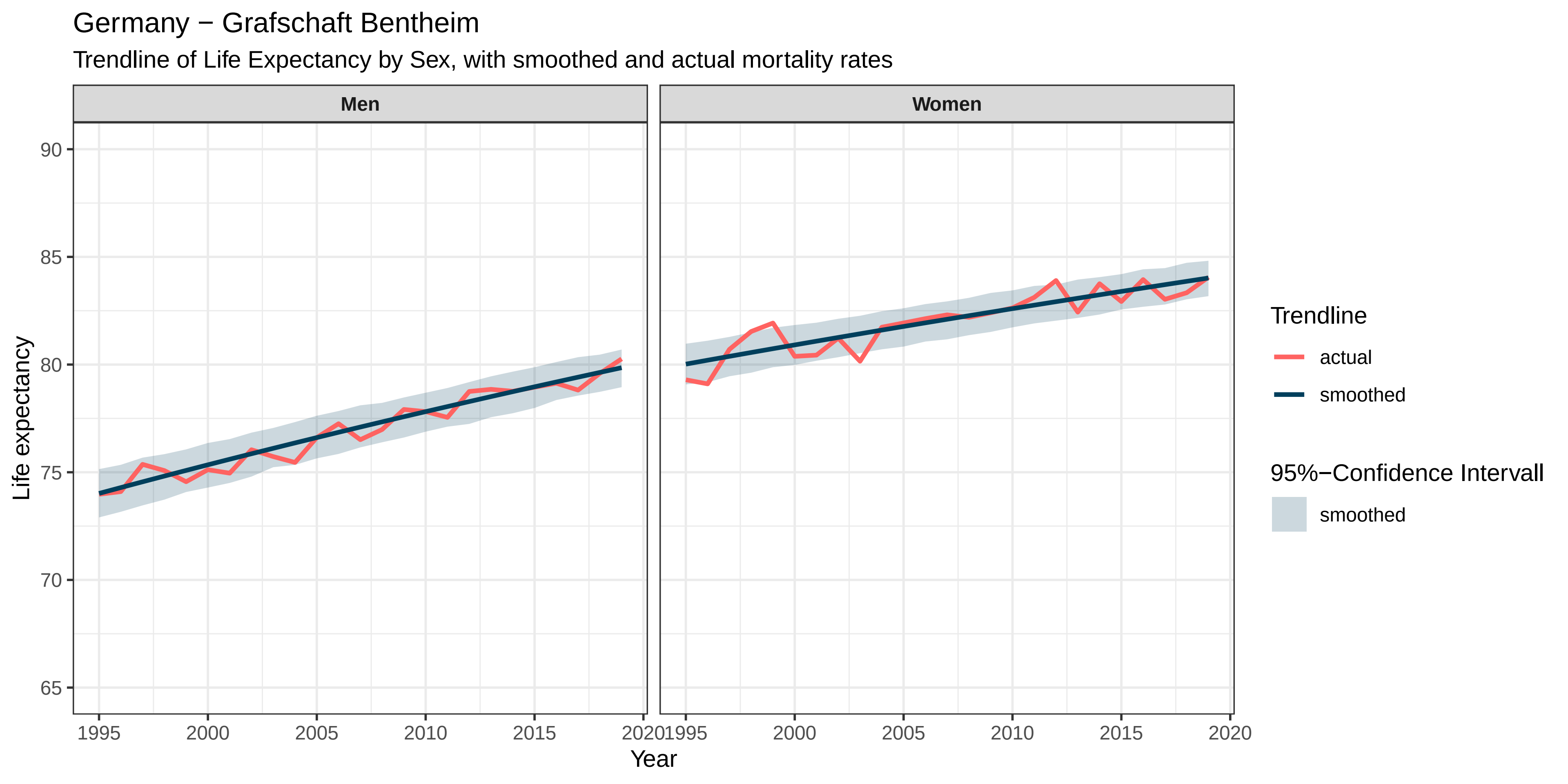

Germany − Grafschaft Bentheim
Trendline of Life Expectancy by Sex, with smoothed and actual mortality rates
Men
Women
90
85
80
75
70
65
1995
2000
2005
2010
2015
2020
Life expectancy
Year
Trendline
actual
smoothed
95%−Confidence Intervall
smoothed

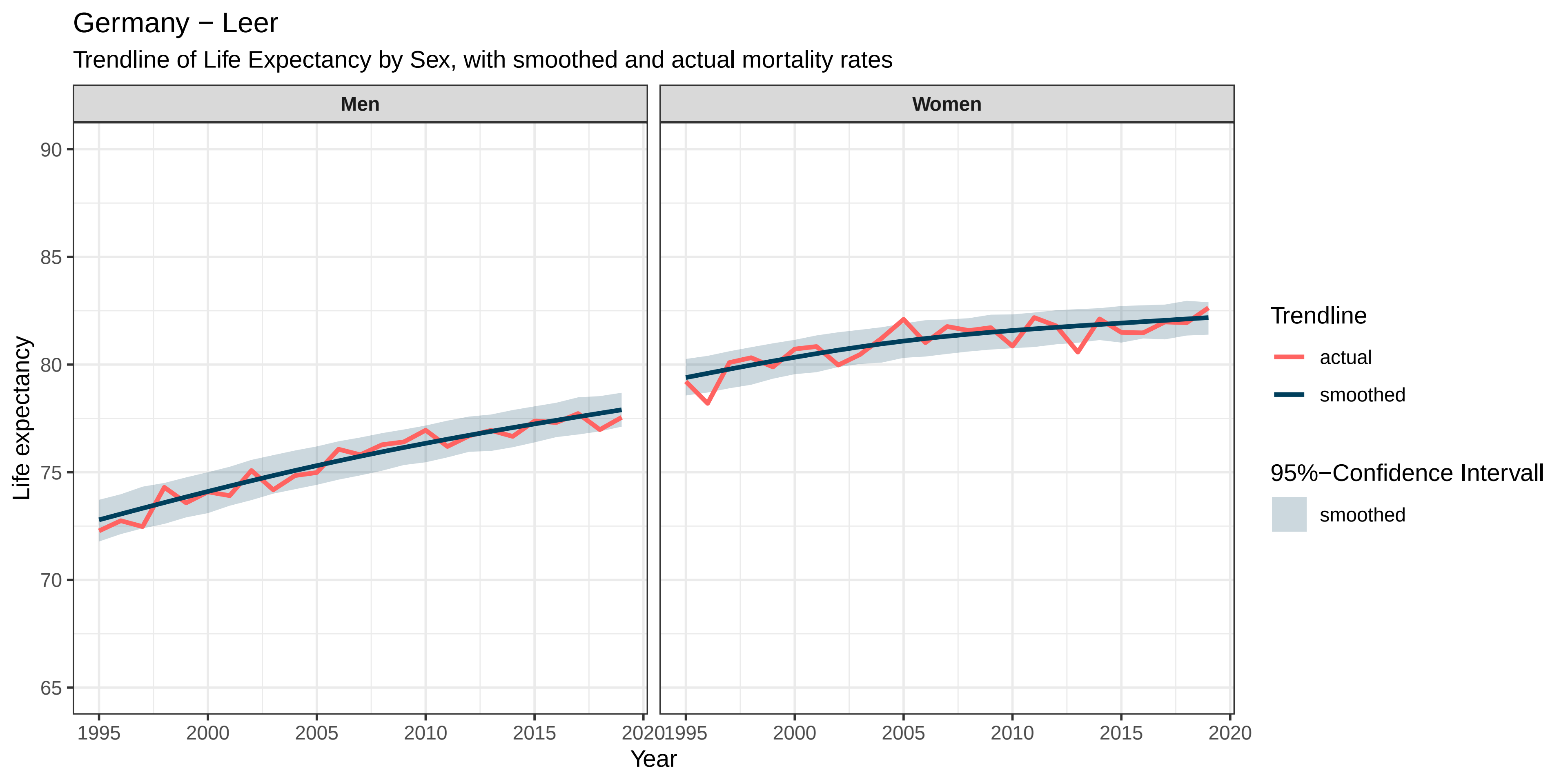

Germany − Leer
Trendline of Life Expectancy by Sex, with smoothed and actual mortality rates
Men
Women
Life expectancy
Year
90
85
80
75
70
65
1995
2000
2005
2010
2015
2020
Trendline
actual
smoothed
95%−Confidence Intervall
smoothed

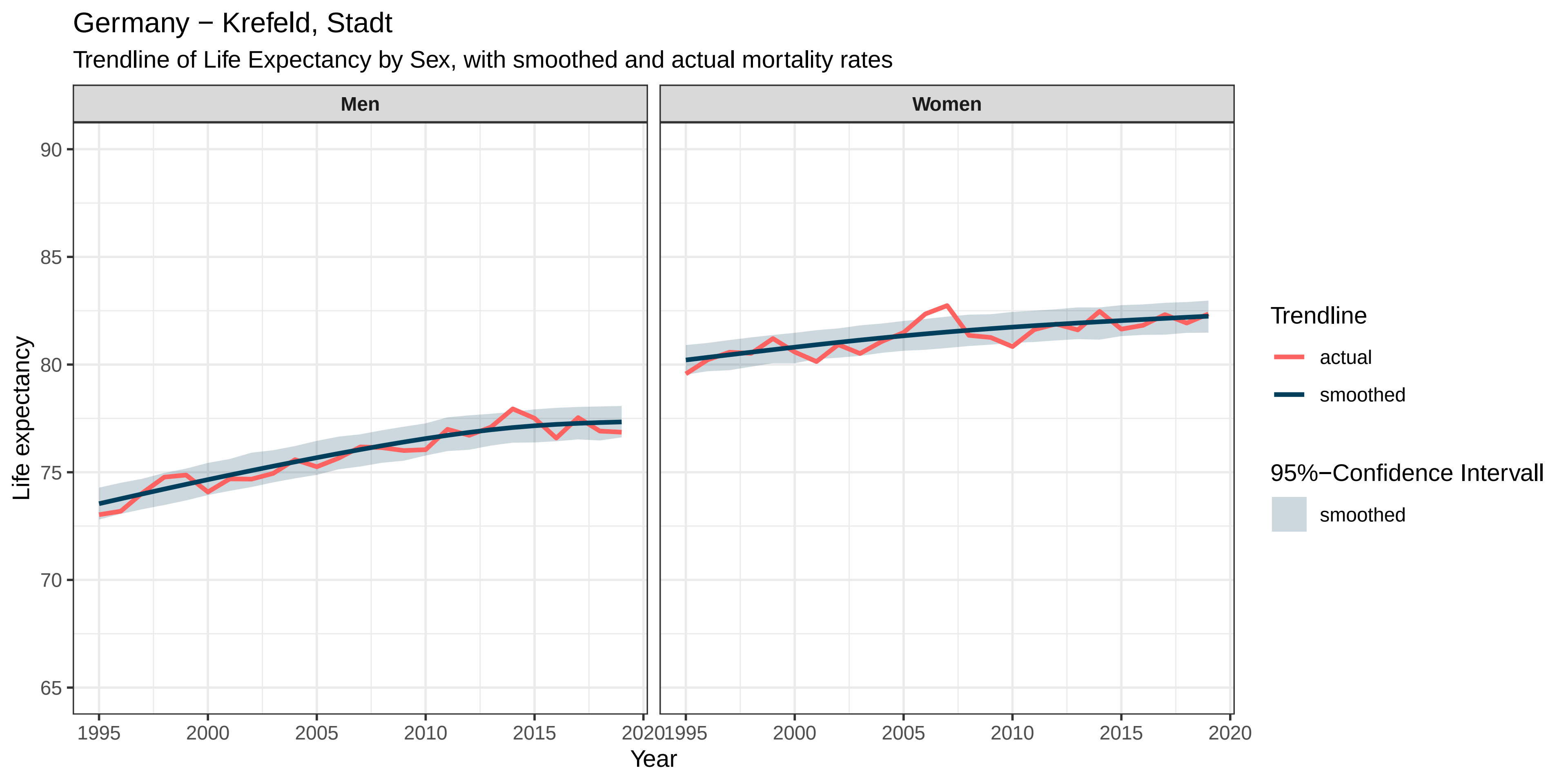
Germany − Krefeld, Stadt
Trendline of Life Expectancy by Sex, with smoothed and actual mortality rates
Men
Women
Life expectancy
Year
90
85
80
75
70
65
1995
2000
2005
2010
2015
2020
Trendline
actual
smoothed
95%−Confidence Intervall
smoothed

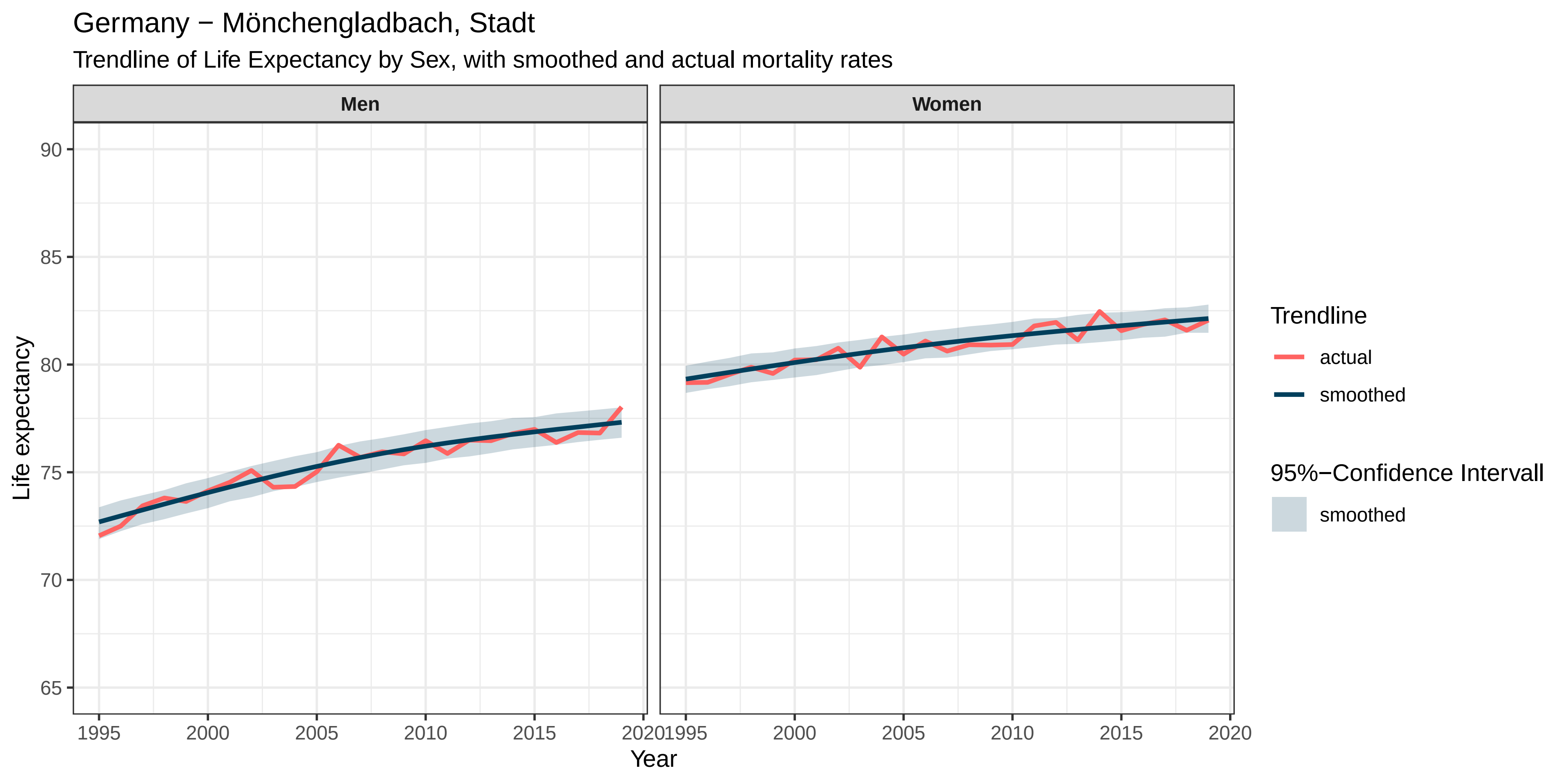

Germany − Mönchengladbach, Stadt
Trendline of Life Expectancy by Sex, with smoothed and actual mortality rates
Men
Women
Life expectancy
90
85
80
75
70
65
1995
2000
2005
2010
2015
2020
Year
Trendline
actual
smoothed
95%−Confidence Intervall
smoothed

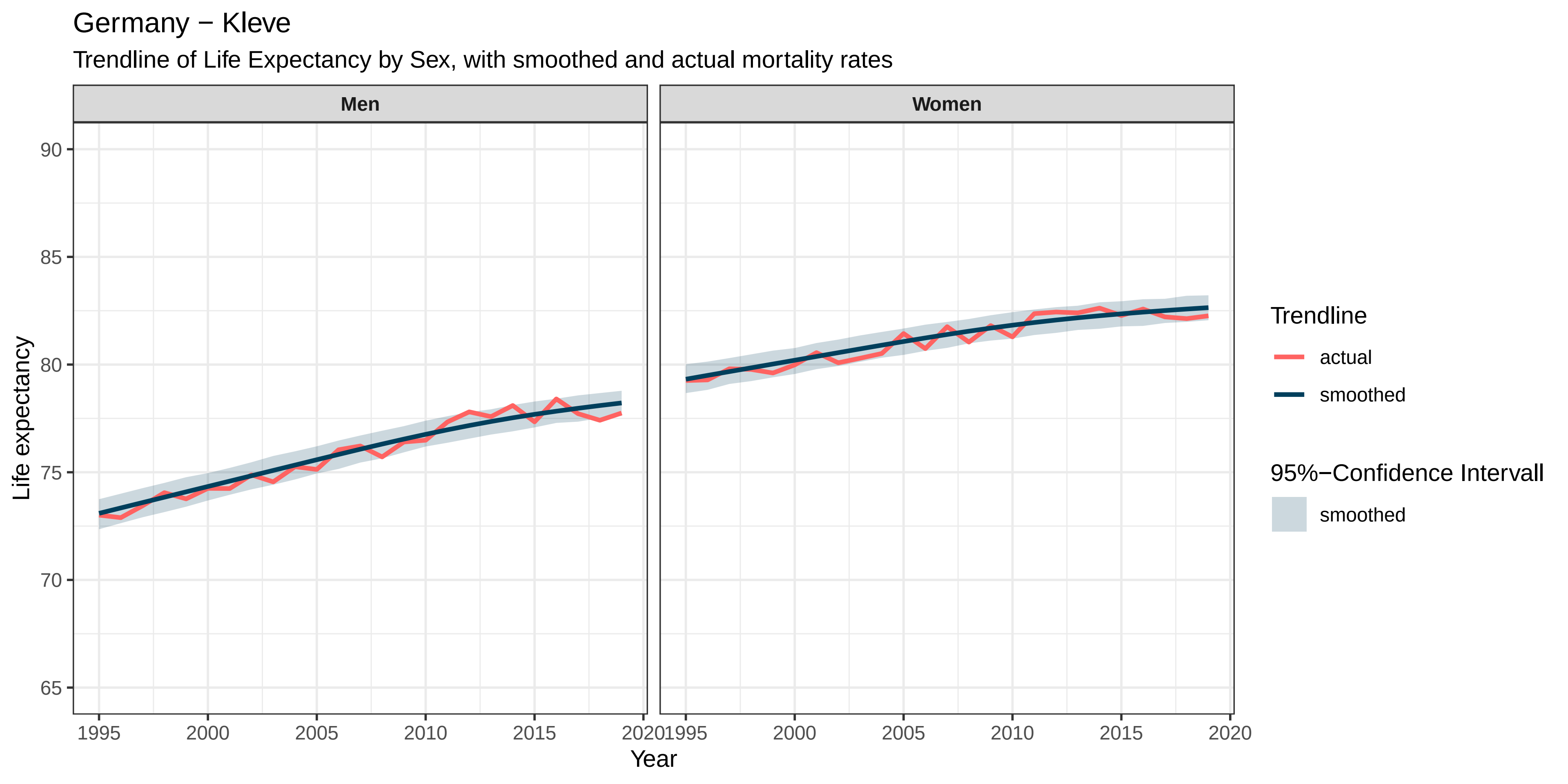

Germany − Kleve
Trendline of Life Expectancy by Sex, with smoothed and actual mortality rates
Men
Women
Life expectancy
90
85
80
75
70
65
1995
2000
2005
2010
2015
2020
Year
Trendline
actual
smoothed
95%−Confidence Intervall
smoothed

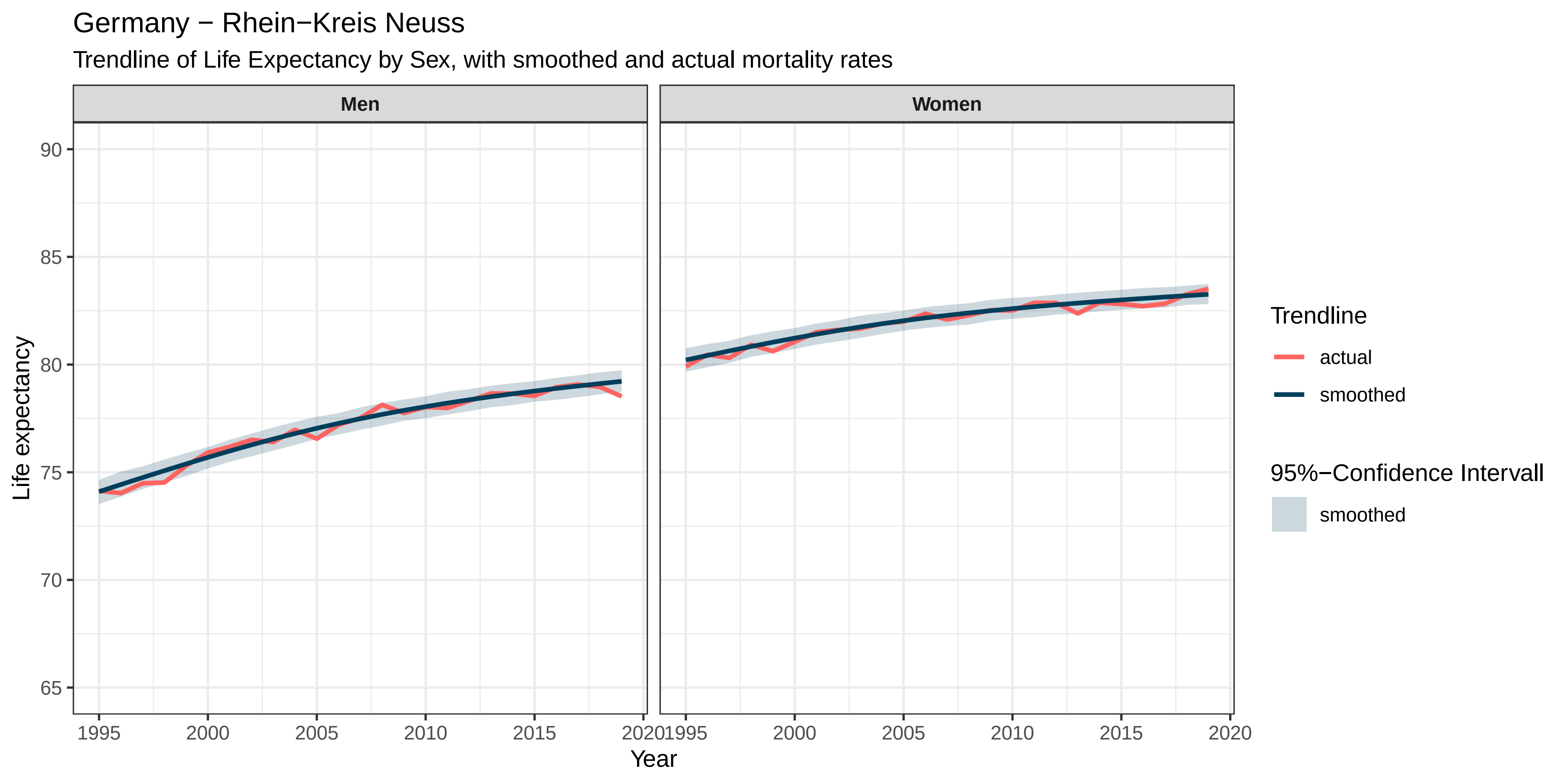

Germany − Rhein−Kreis Neuss
Trendline of Life Expectancy by Sex, with smoothed and actual mortality rates
Men
Women
Life expectancy
Year
90
85
80
75
70
65
1995
2000
2005
2010
2015
2020
Trendline
actual
smoothed
95%−Confidence Intervall
smoothed

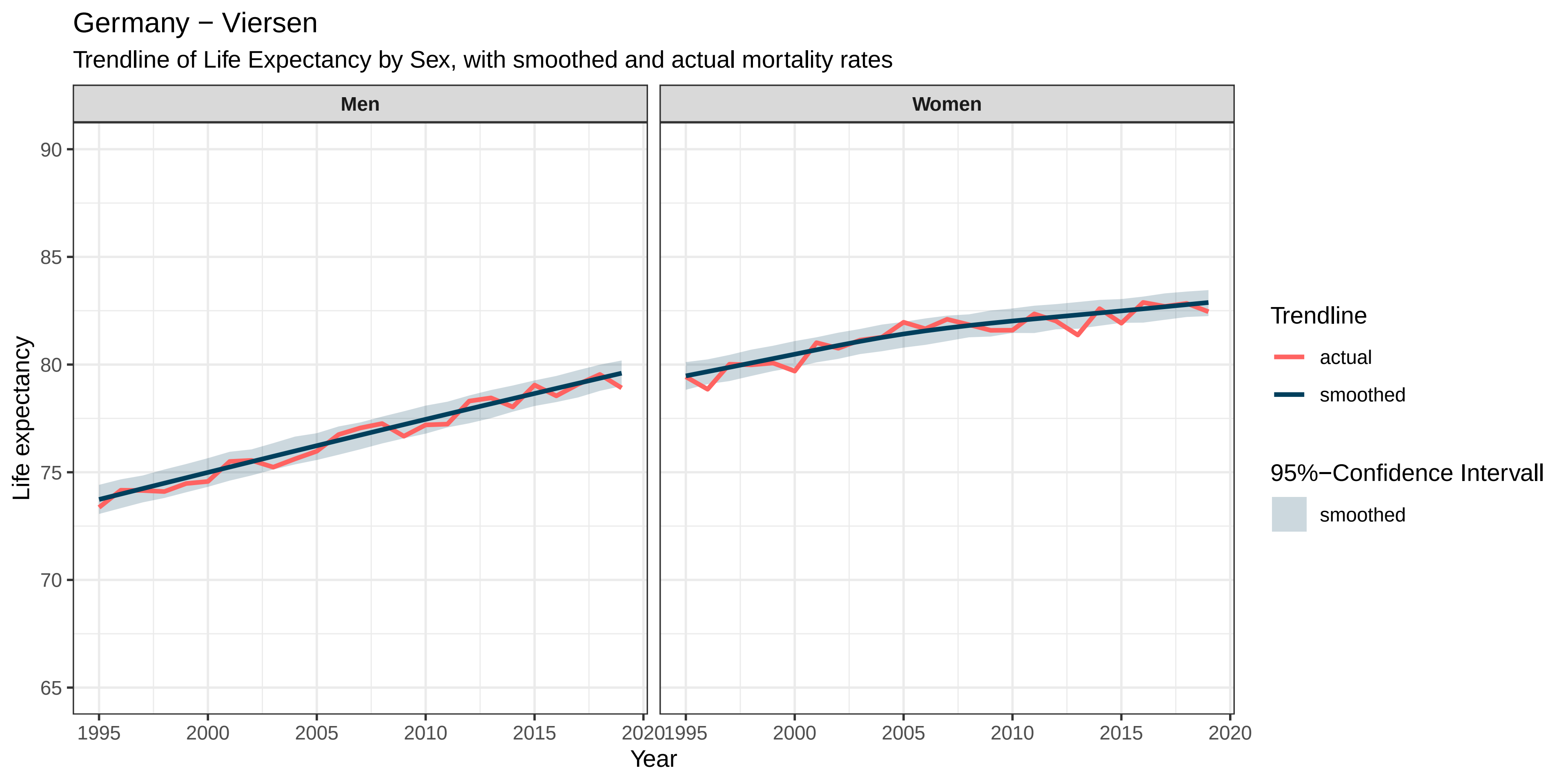

Germany − Viersen
Trendline of Life Expectancy by Sex, with smoothed and actual mortality rates
Men
Women
Life expectancy
90
85
80
75
70
65
1995
2000
2005
2010
2015
2020
Year
Trendline
actual
smoothed
95%−Confidence Intervall
smoothed

Germany − Wesel

Trendline of Life Expectancy by Sex, with smoothed and actual mortality rates

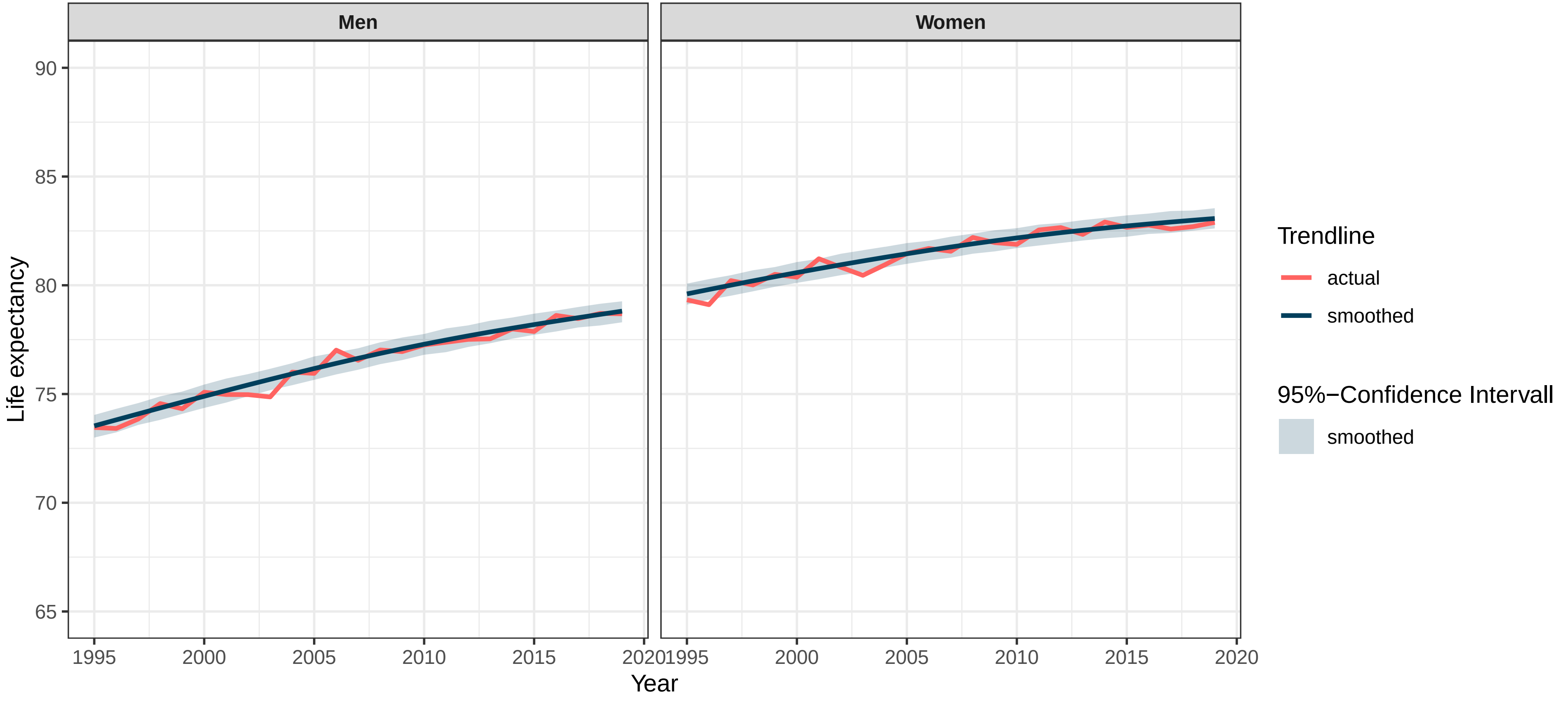

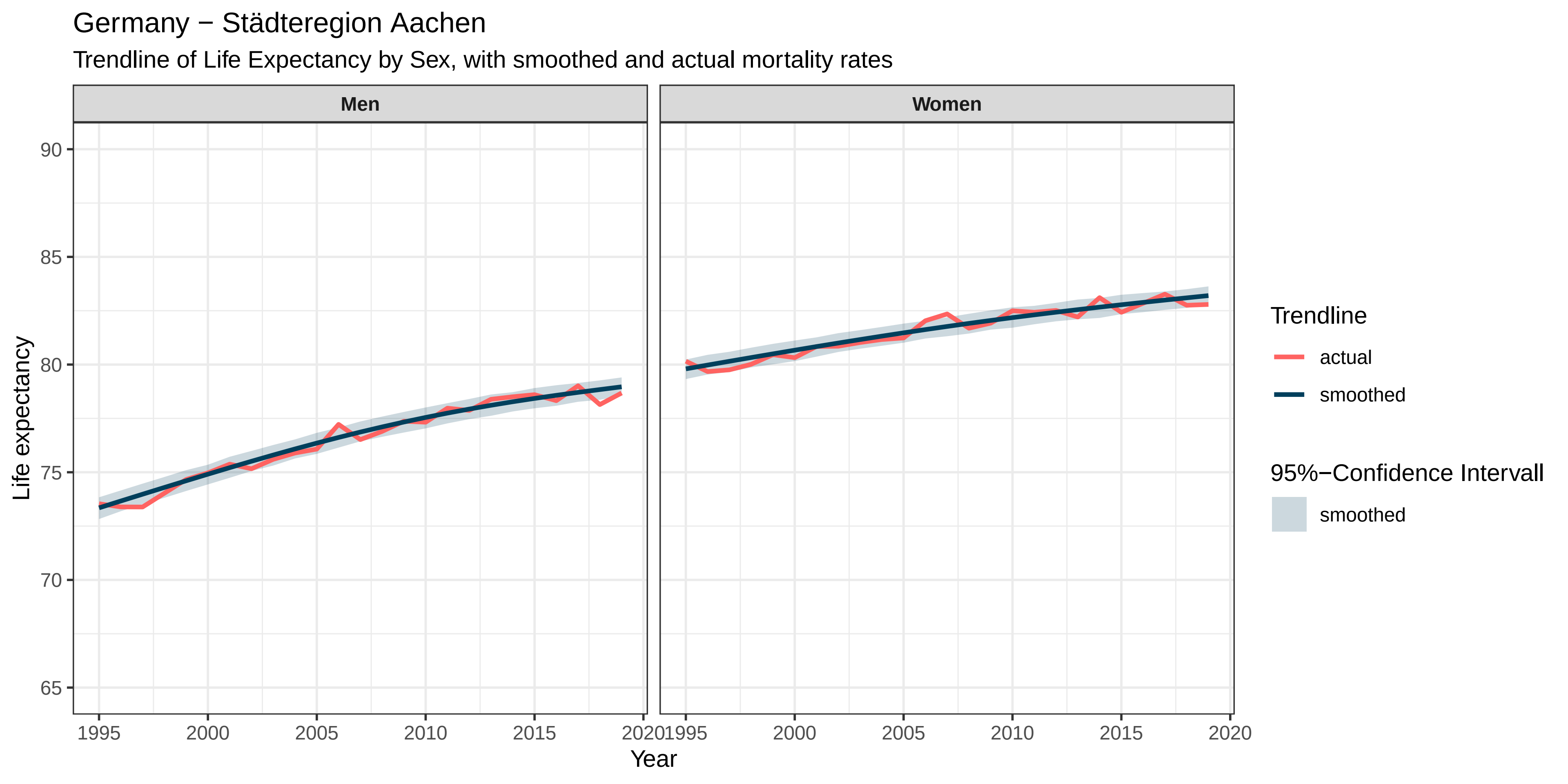
Germany − Städteregion Aachen
Trendline of Life Expectancy by Sex, with smoothed and actual mortality rates
Men
Women
Life expectancy
Year
90
85
80
75
70
65
1995
2000
2005
2010
2015
2020
Trendline
actual
smoothed
95%−Confidence Intervall
smoothed

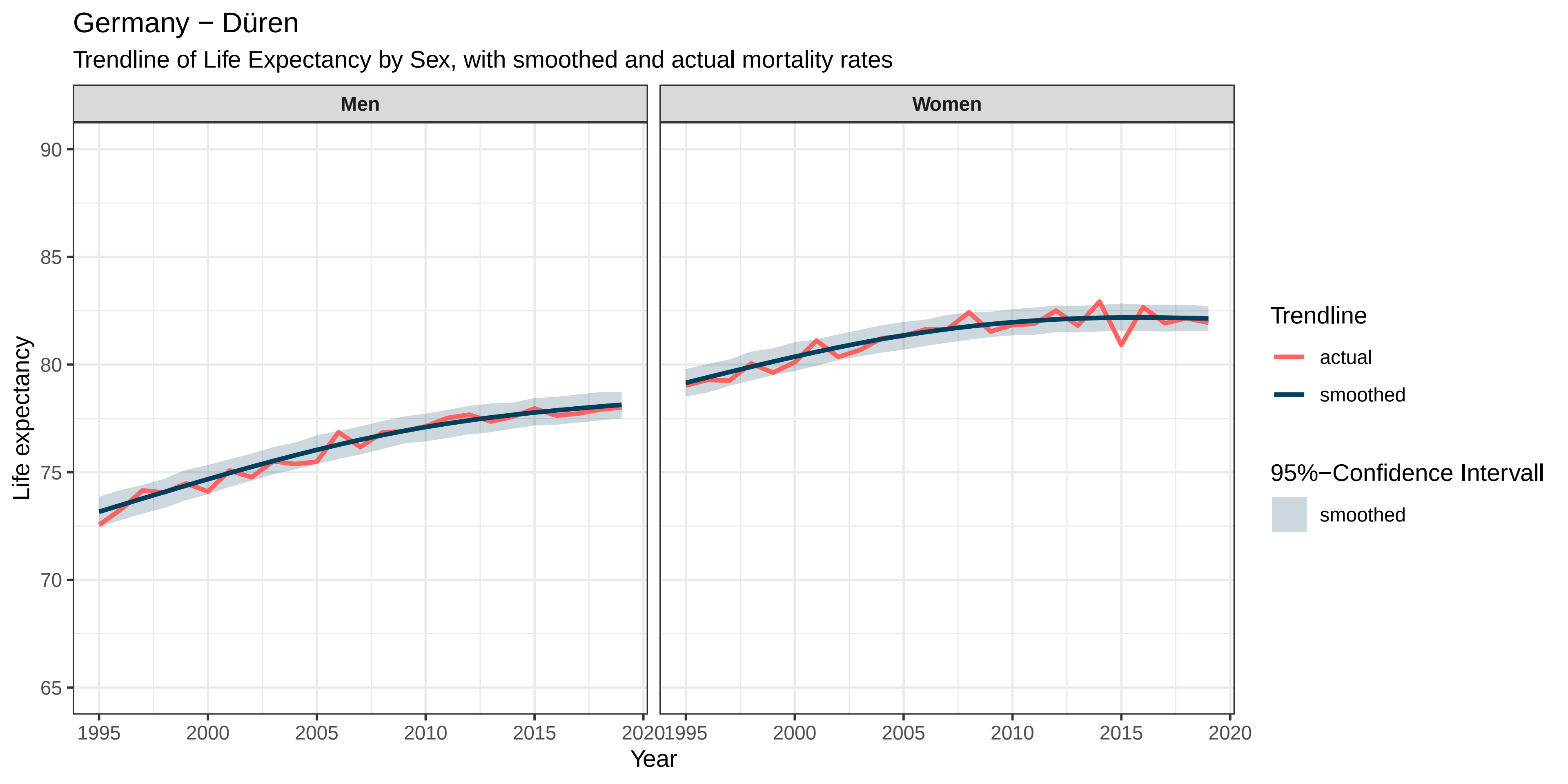
Germany − Düren
Trendline of Life Expectancy by Sex, with smoothed and actual mortality rates
Men
Women
90
85
80
75
70
65
Life expectancy
1995
2000
2005
2010
2015
2020
Year
Trendline
actual
smoothed
95%−Confidence Intervall
smoothed

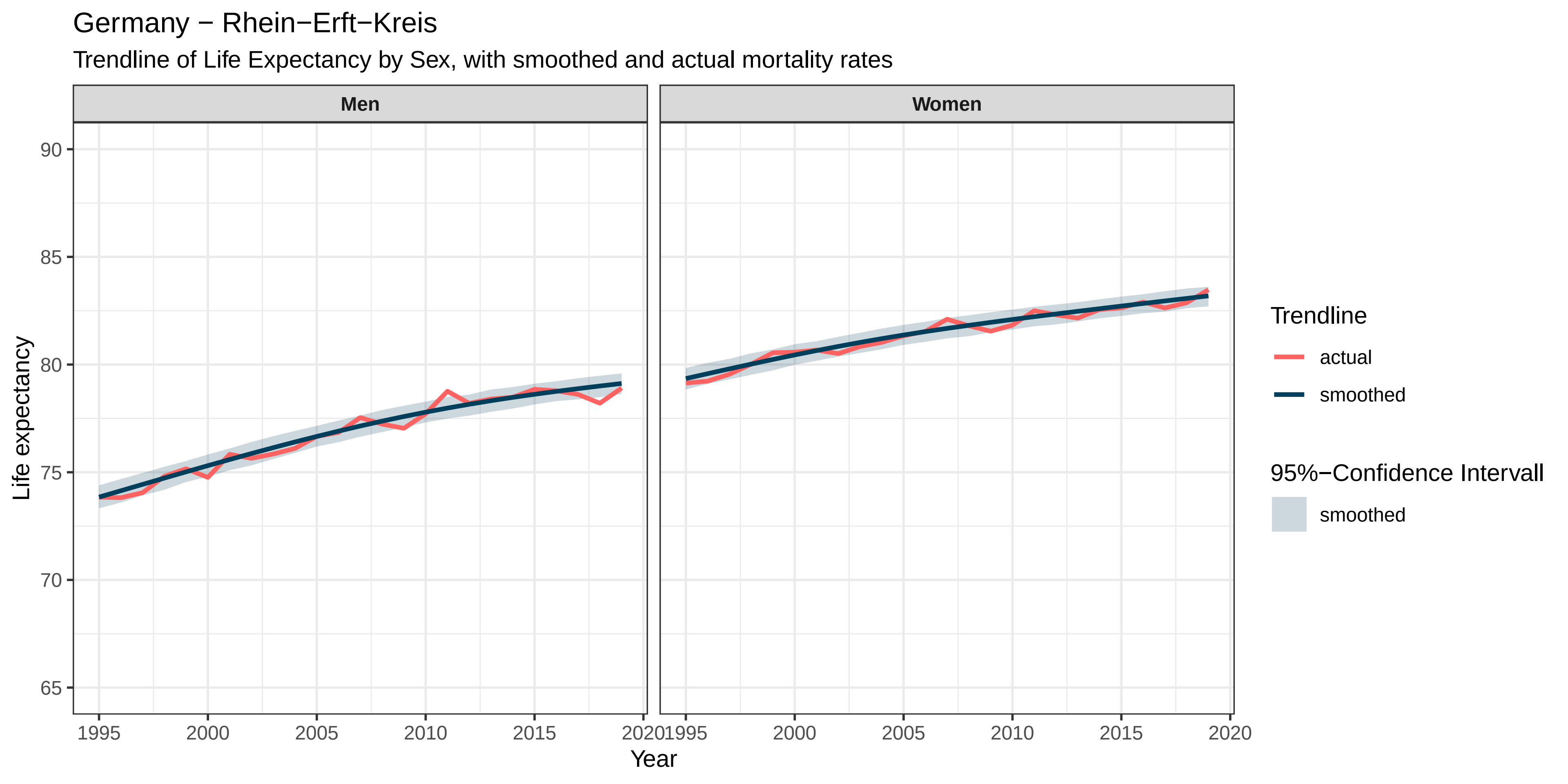

Germany − Rhein−Erft−Kreis
Trendline of Life Expectancy by Sex, with smoothed and actual mortality rates
Men
Women
90
85
80
75
70
65
Life expectancy
1995
2000
2005
2010
2015
2020
Year
Trendline
actual
smoothed
95%−Confidence Intervall
smoothed

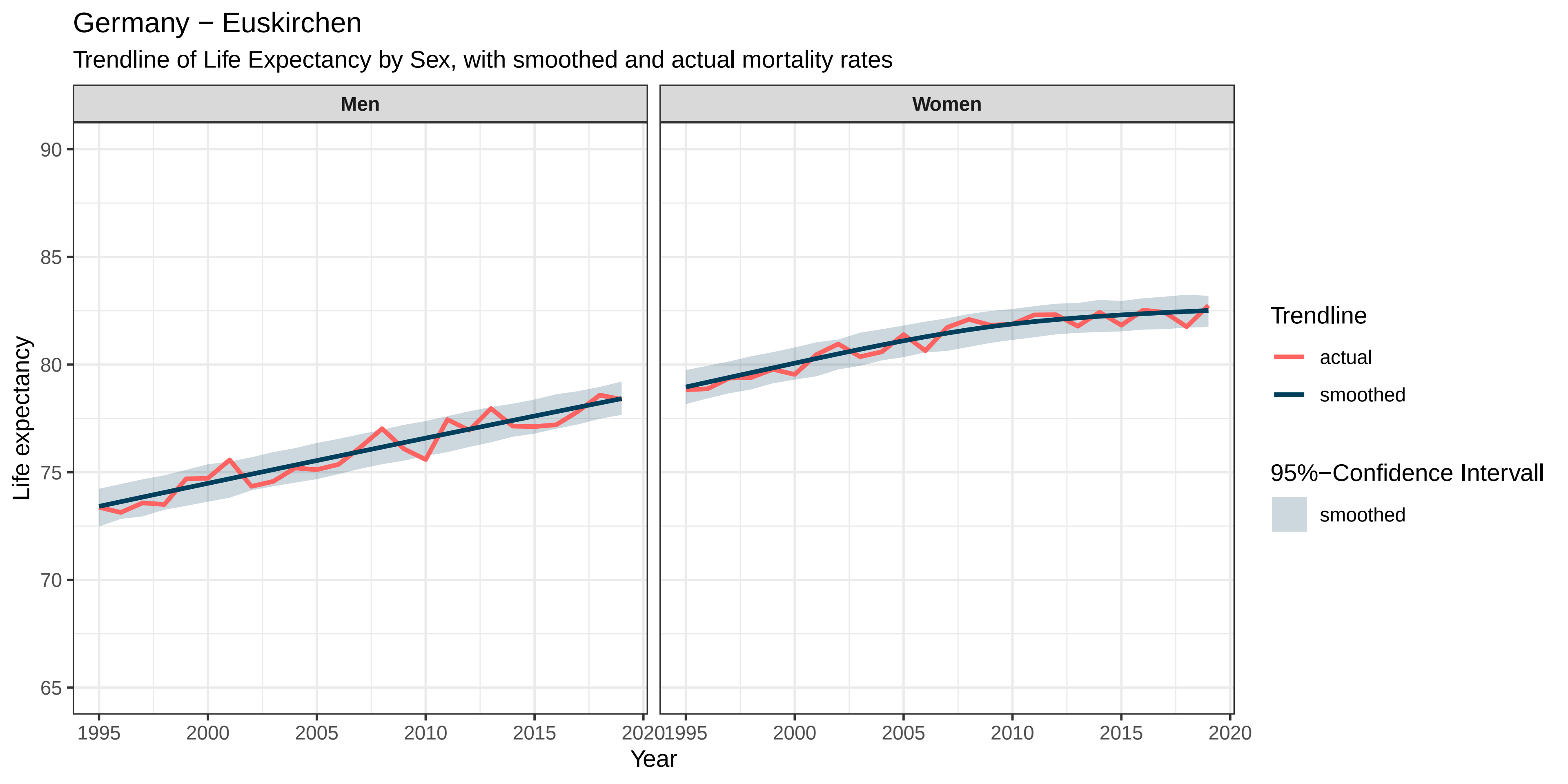

Germany − Euskirchen
Trendline of Life Expectancy by Sex, with smoothed and actual mortality rates
Men
Women
90
85
80
75
70
65
Life expectancy
1995
2000
2005
2010
2015
2020
Year
Trendline
actual
smoothed
95%−Confidence Intervall
smoothed

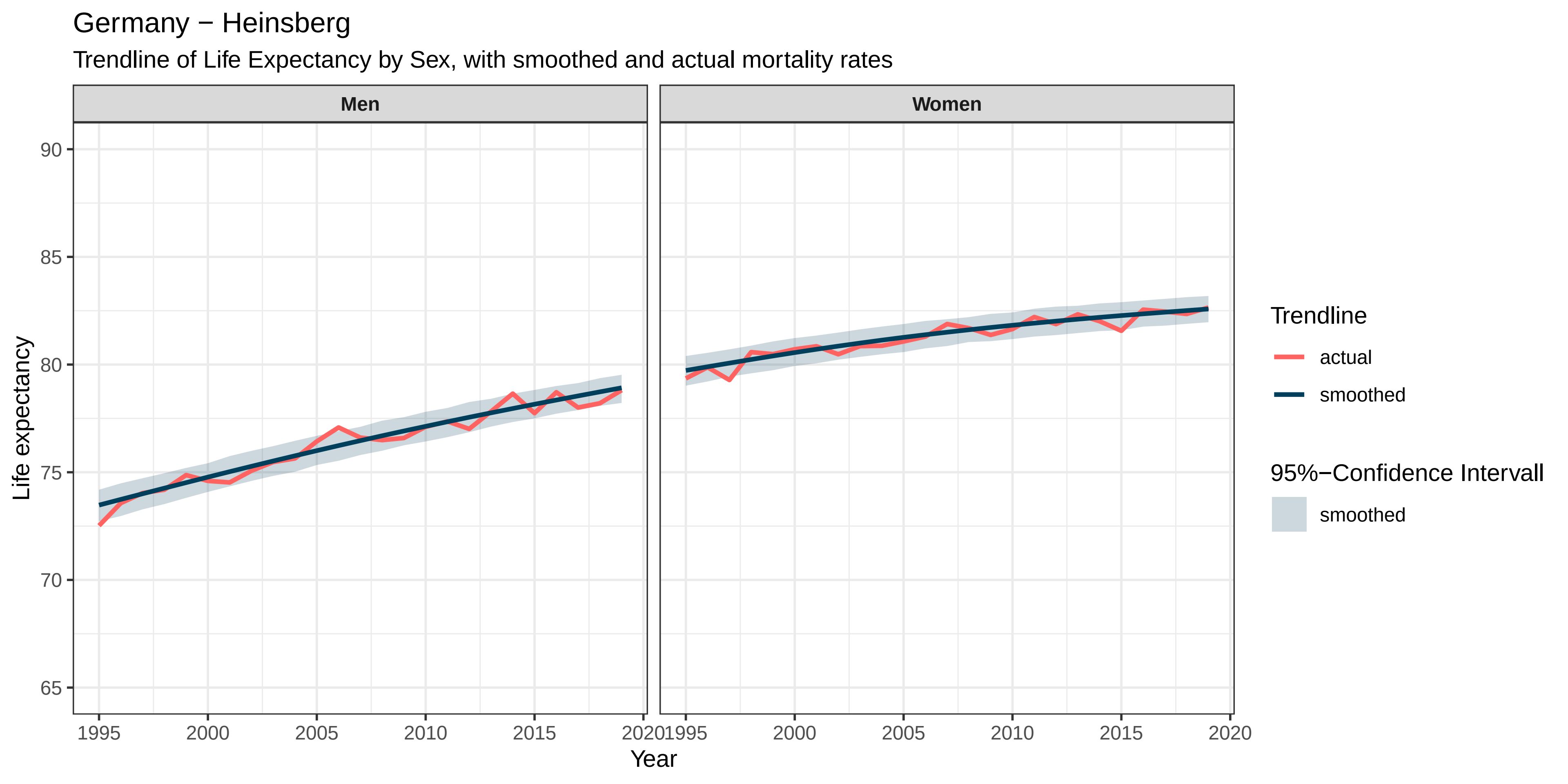
Germany − Heinsberg
Trendline of Life Expectancy by Sex, with smoothed and actual mortality rates
Men
Women
Life expectancy
90
85
80
75
70
65
1995
2000
2005
2010
2015
2020
Year
Trendline
actual
smoothed
95%−Confidence Intervall
smoothed

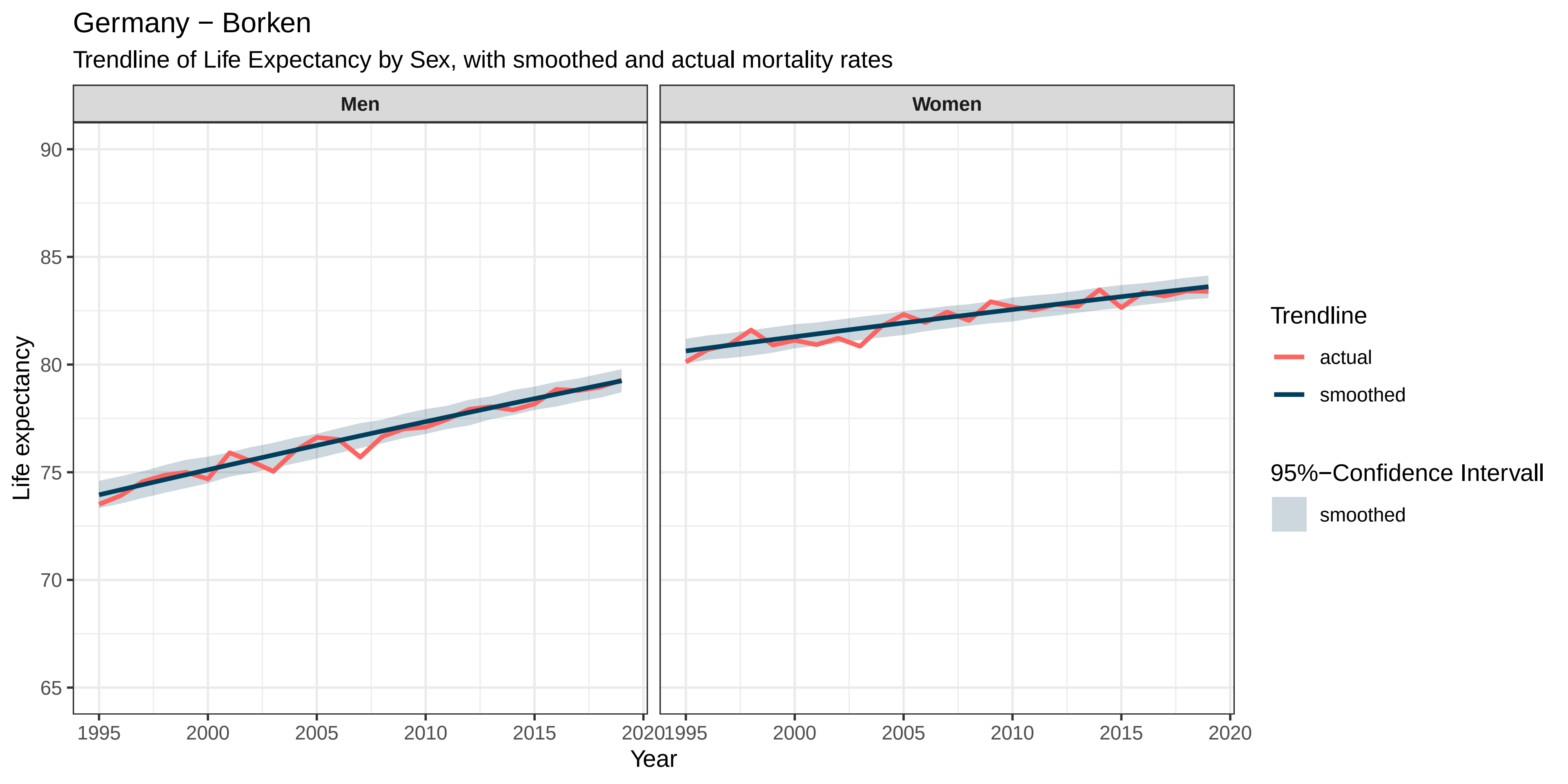
Germany − Borken
Trendline of Life Expectancy by Sex, with smoothed and actual mortality rates
Men
Women
Life expectancy
90
85
80
75
70
65
1995
2000
2005
2010
2015
2020
Year
Trendline
actual
smoothed
95%−Confidence Intervall
smoothed

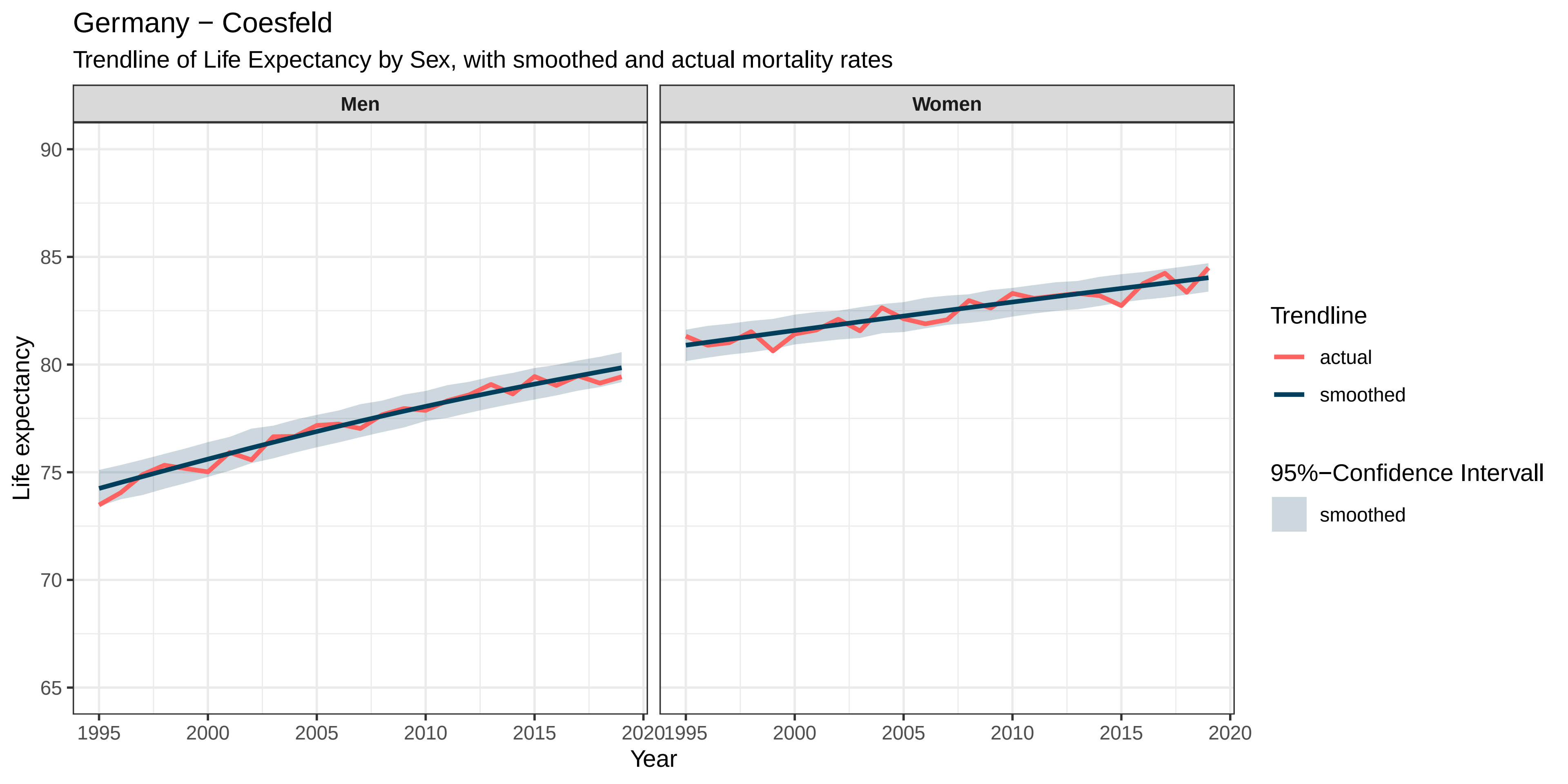

Germany − Coesfeld
Trendline of Life Expectancy by Sex, with smoothed and actual mortality rates
Men
Women
Life expectancy
90
85
80
75
70
65
1995
2000
2005
2010
2015
2020
Year
Trendline
actual
smoothed
95%−Confidence Intervall
smoothed

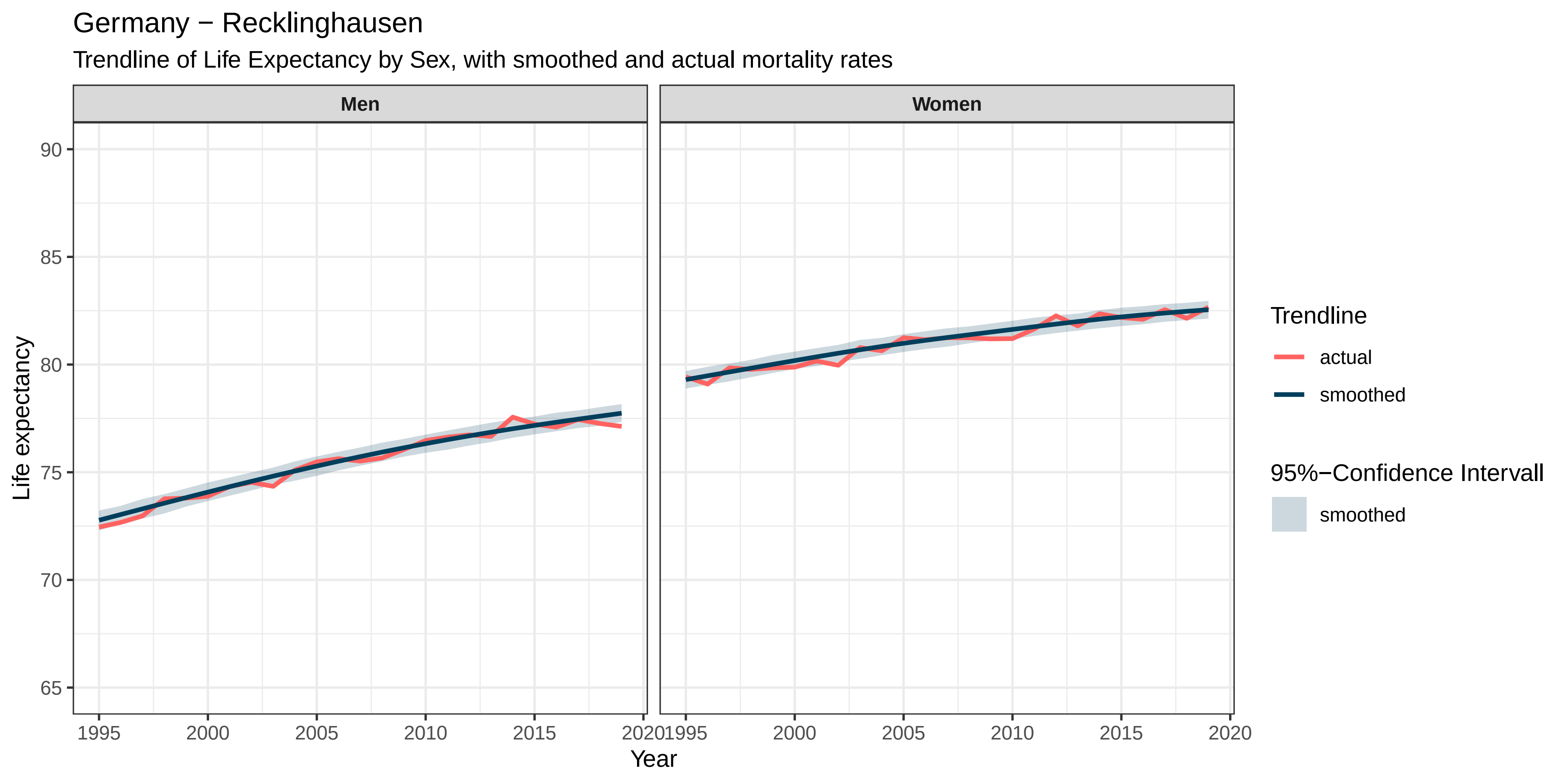
Germany − Recklinghausen
Trendline of Life Expectancy by Sex, with smoothed and actual mortality rates
Men
Women
Life expectancy
Year
90
85
80
75
70
65
1995
2000
2005
2010
2015
2020
Trendline
actual
smoothed
95%−Confidence Intervall
smoothed

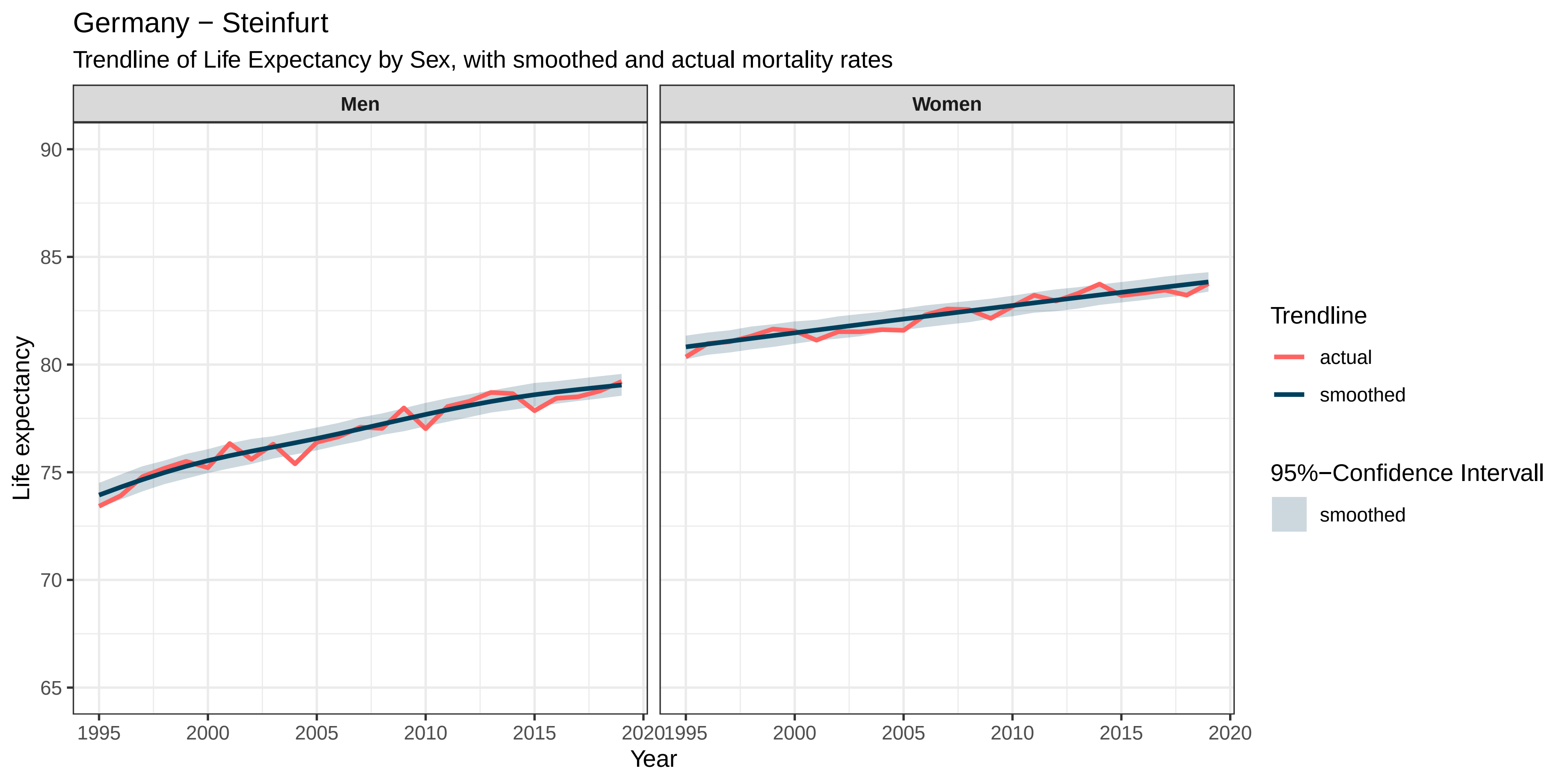
Germany − Steinfurt
Trendline of Life Expectancy by Sex, with smoothed and actual mortality rates
Men
Women
90
85
80
75
70
65
Life expectancy
1995
2000
2005
2010
2015
2020
Year
Trendline
actual
smoothed
95%−Confidence Intervall
smoothed

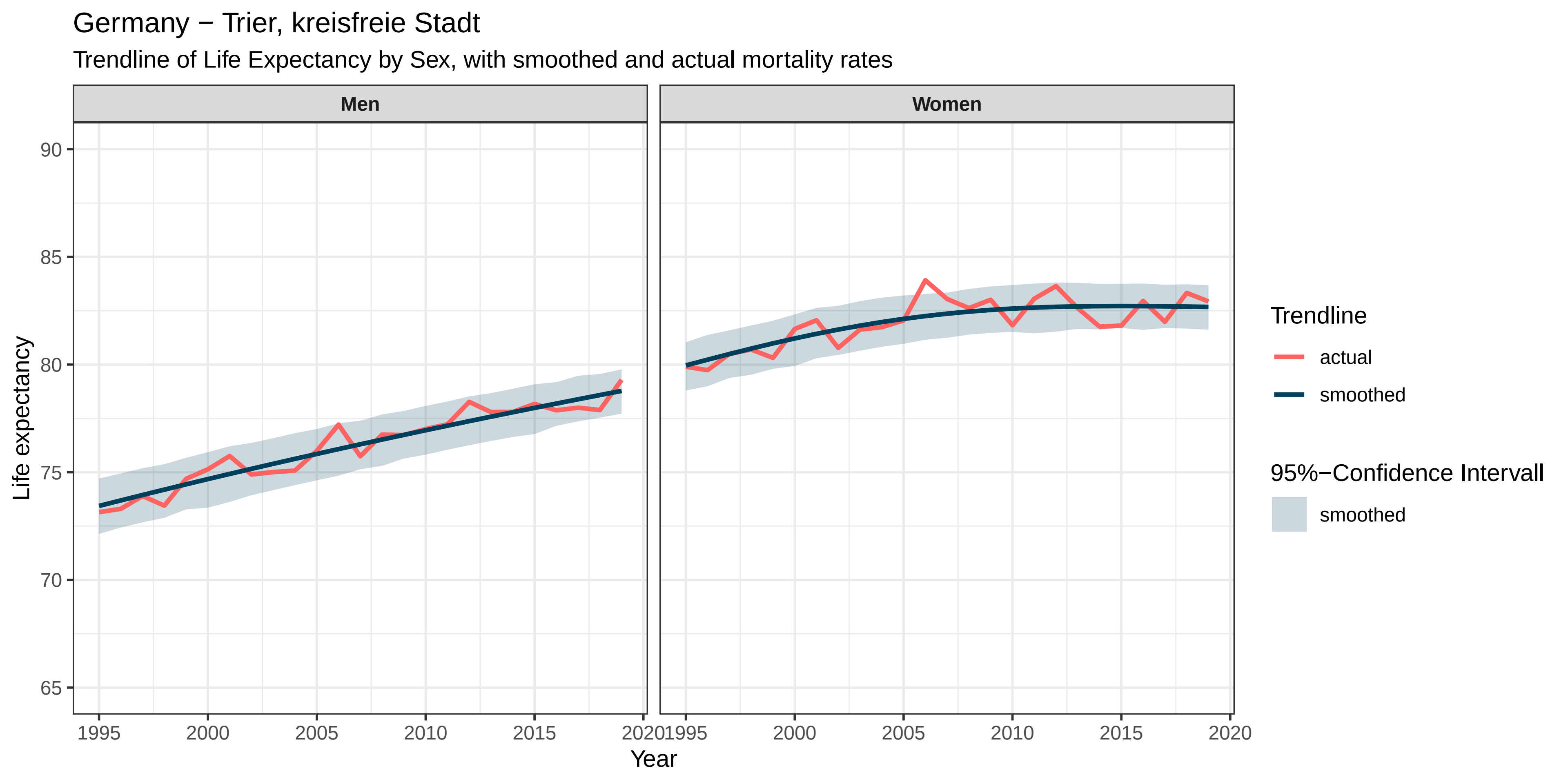

Germany − Trier, kreisfreie Stadt
Trendline of Life Expectancy by Sex, with smoothed and actual mortality rates
Men
Women
Life expectancy
65
70
75
80
85
90
1995
2000
2005
2010
2015
2020
Year
Trendline
actual
smoothed
95%−Confidence Intervall
smoothed

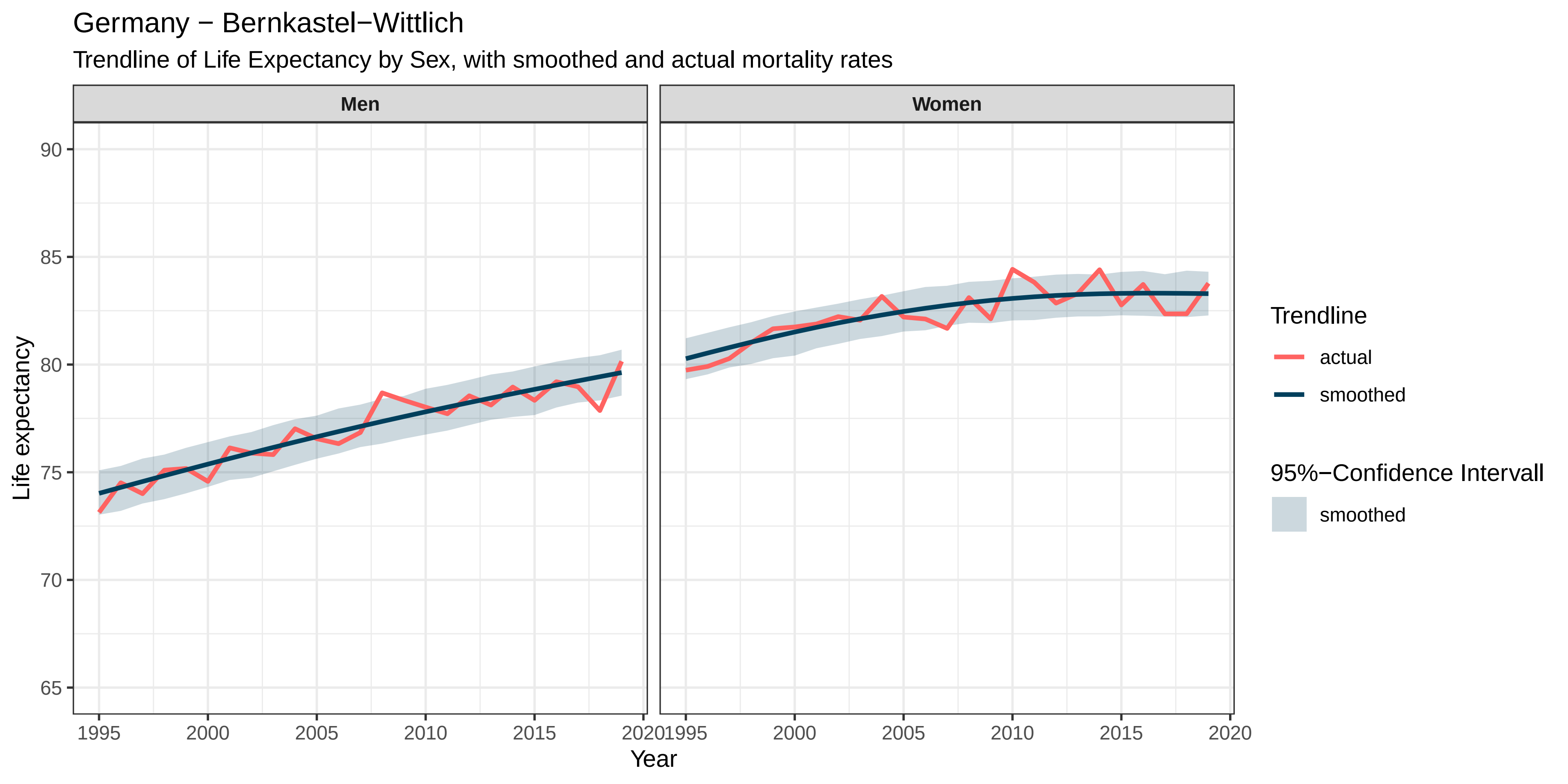

Germany − Bernkastel−Wittlich
Trendline of Life Expectancy by Sex, with smoothed and actual mortality rates
Men
Women
90
85
80
75
70
65
Life expectancy
1995
2000
2005
2010
2015
2020
Year
Trendline
actual
smoothed
95%−Confidence Intervall
smoothed

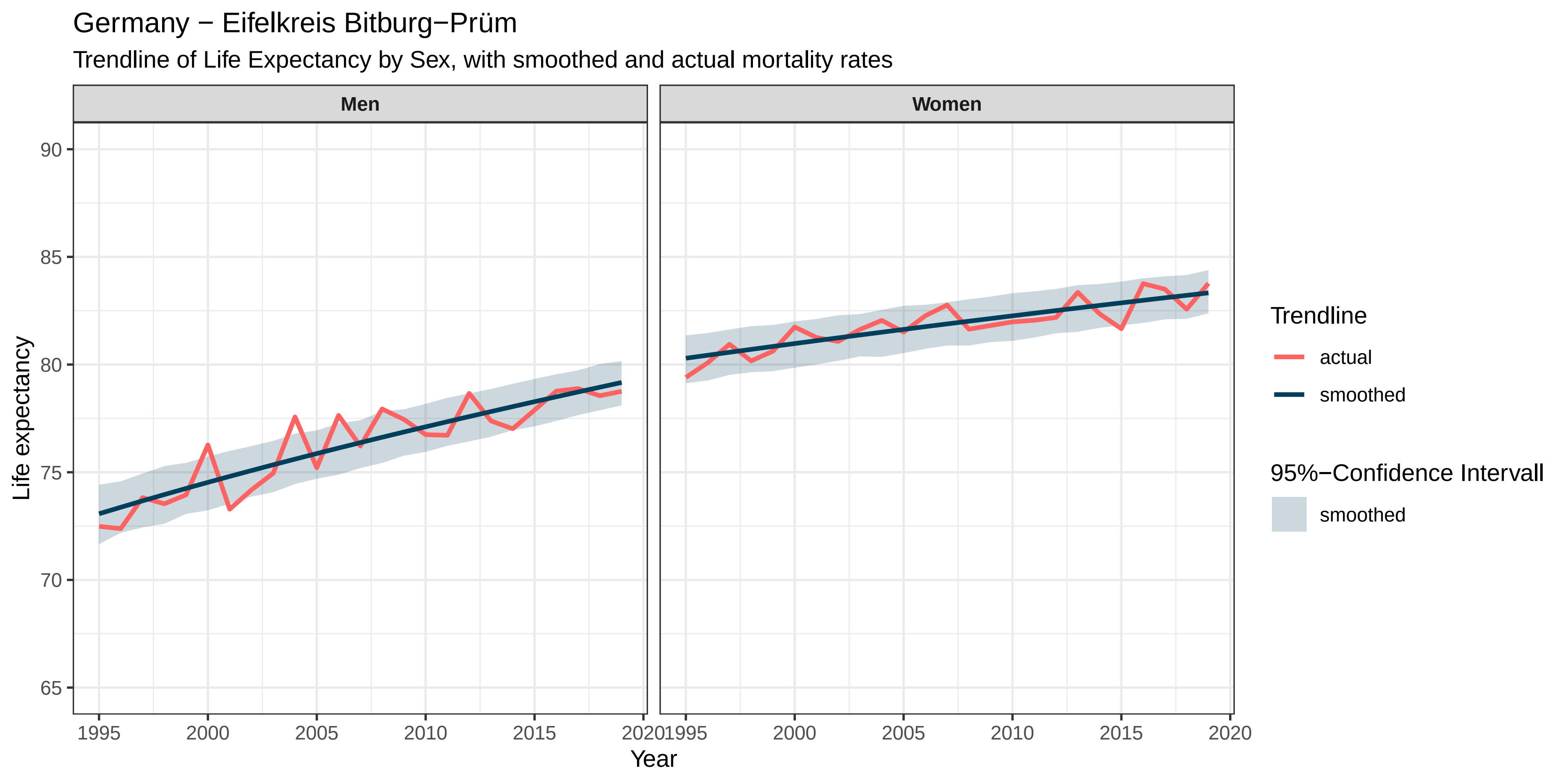
Germany − Eifelkreis Bitburg−Prüm
Trendline of Life Expectancy by Sex, with smoothed and actual mortality rates
Men
Women
Life expectancy
90
85
80
75
70
65
1995
2000
2005
2010
2015
2020
Year
Trendline
actual
smoothed
95%−Confidence Intervall
smoothed

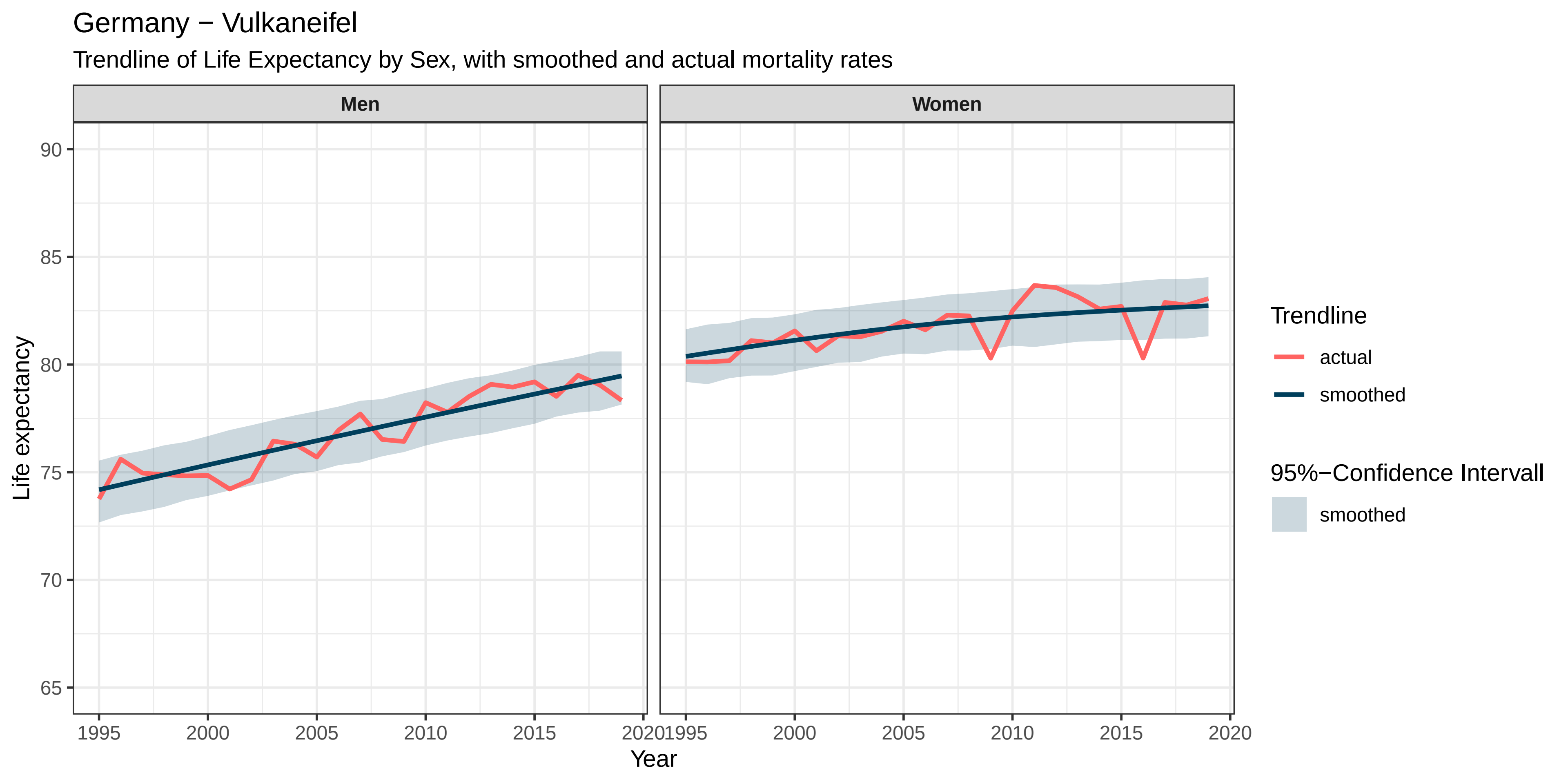

Germany − Vulkaneifel
Trendline of Life Expectancy by Sex, with smoothed and actual mortality rates
Men
Women
90
85
80
75
70
65
1995
2000
2005
2010
2015
2020
Year
Life expectancy
Trendline
actual
smoothed
95%−Confidence Intervall
smoothed

Germany − Trier−Saarburg

Trendline of Life Expectancy by Sex, with smoothed and actual mortality rates

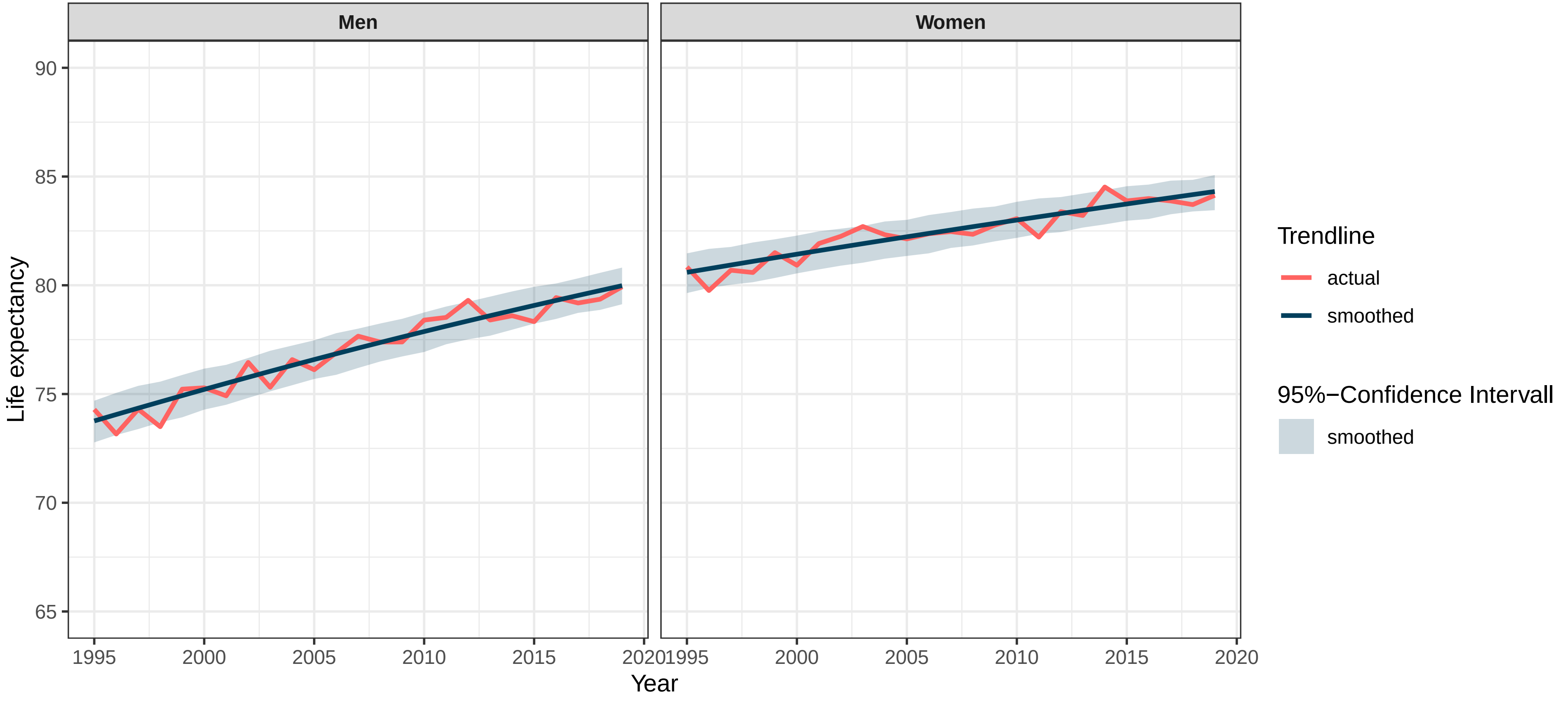

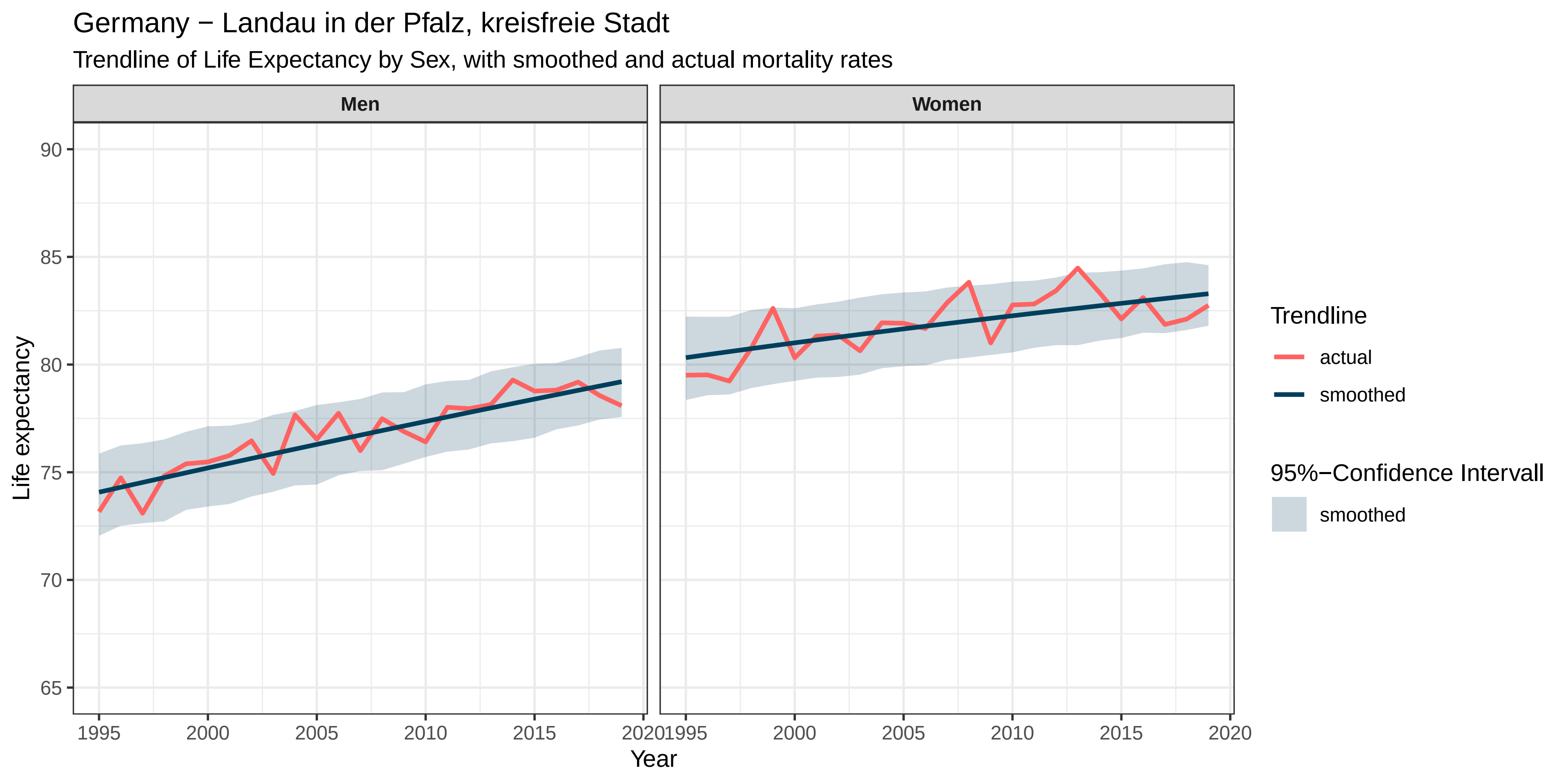

Germany − Landau in der Pfalz, kreisfreie Stadt
Trendline of Life Expectancy by Sex, with smoothed and actual mortality rates
Men
Women
Life expectancy
90
85
80
75
70
65
1995
2000
2005
2010
2015
2020
Year
Trendline
actual
smoothed
95%−Confidence Intervall
smoothed

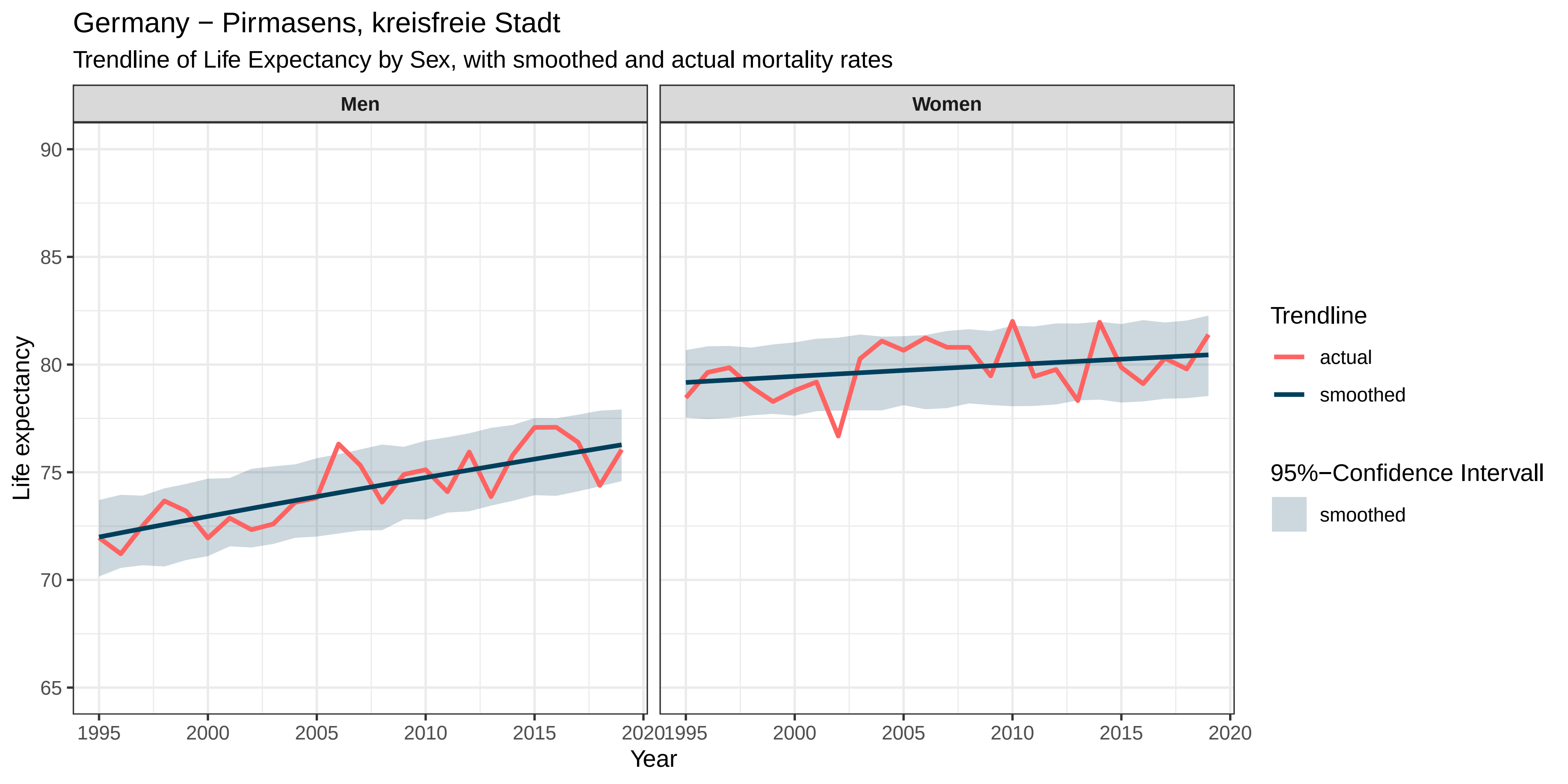
Germany − Pirmasens, kreisfreie Stadt
Trendline of Life Expectancy by Sex, with smoothed and actual mortality rates
Men
Women
Life expectancy
Year
65
70
75
80
85
90
1995
2000
2005
2010
2015
2020
Trendline
actual
smoothed
95%−Confidence Intervall
smoothed

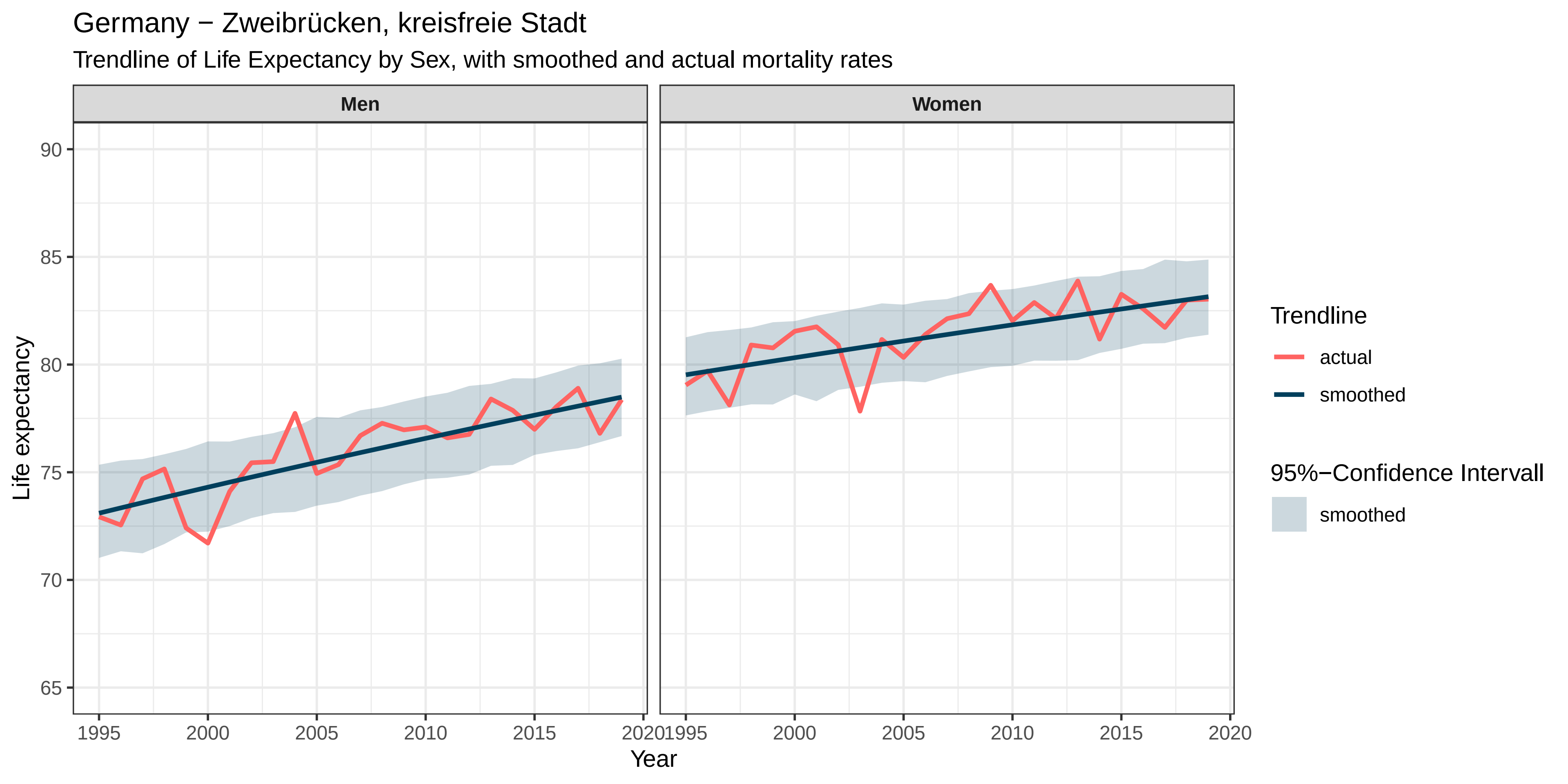

Germany − Zweibrücken, kreisfreie Stadt
Trendline of Life Expectancy by Sex, with smoothed and actual mortality rates
Men
Women
90
85
80
75
70
65
Life expectancy
1995
2000
2005
2010
2015
2020
Year
Trendline
actual
smoothed
95%−Confidence Intervall
smoothed

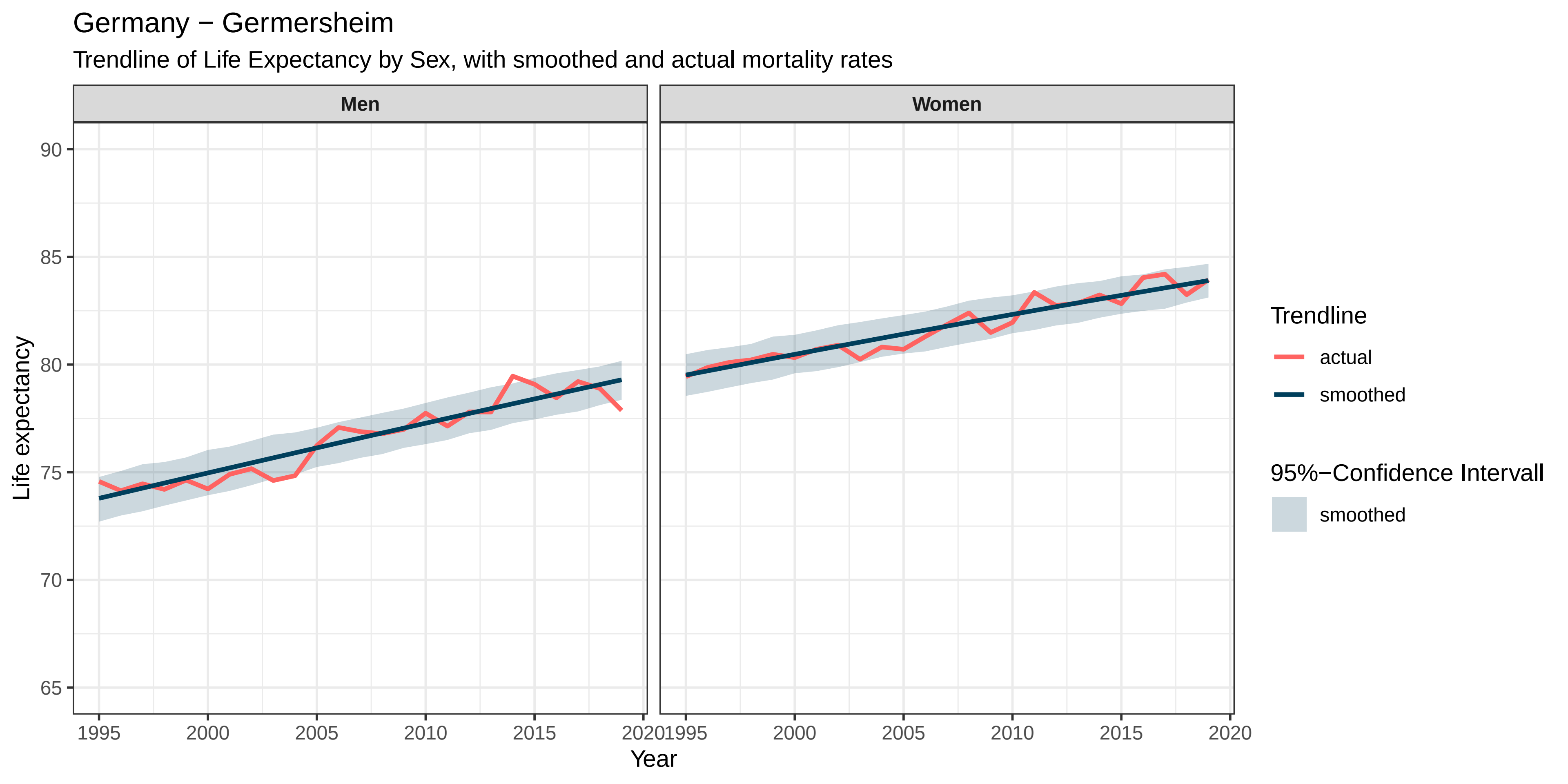

Germany − Germersheim
Trendline of Life Expectancy by Sex, with smoothed and actual mortality rates
Men
Women
Life expectancy
90
85
80
75
70
65
1995
2000
2005
2010
2015
2020
Year
Trendline
actual
smoothed
95%−Confidence Intervall
smoothed

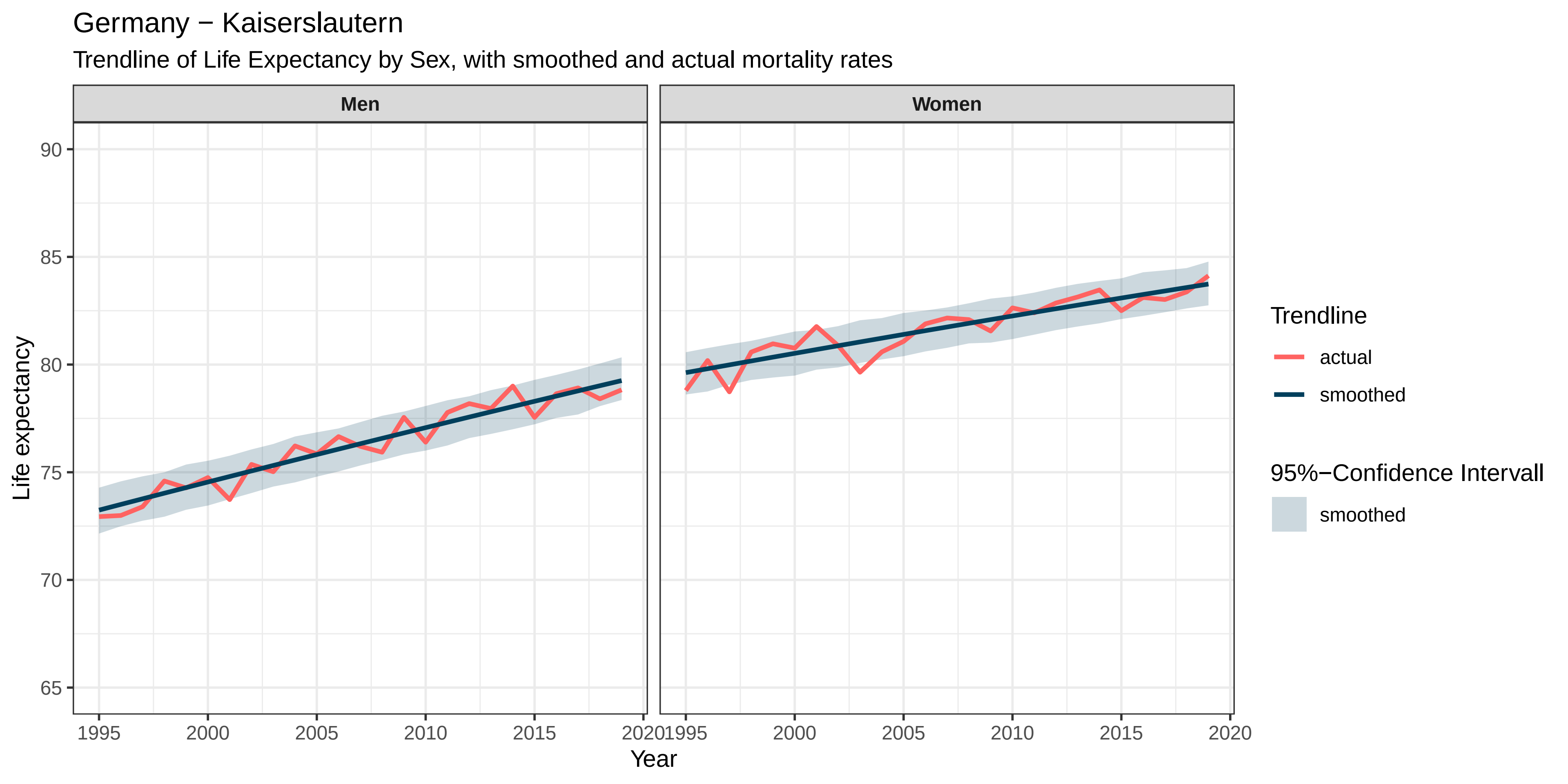
Germany − Kaiserslautern
Trendline of Life Expectancy by Sex, with smoothed and actual mortality rates
Men
Women
Life expectancy
Year
Trendline
actual
smoothed
95%−Confidence Intervall
smoothed

Germany − Kusel

Trendline of Life Expectancy by Sex, with smoothed and actual mortality rates

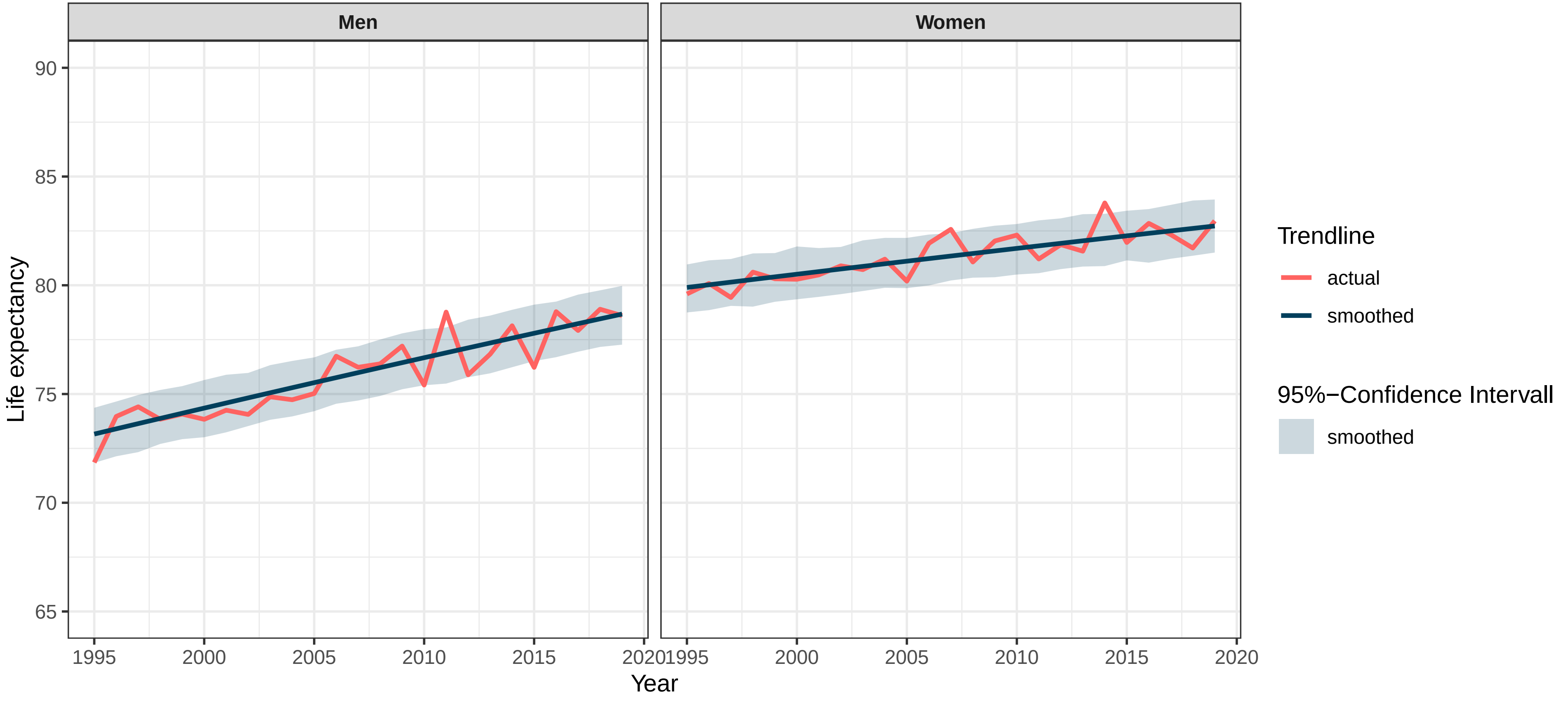

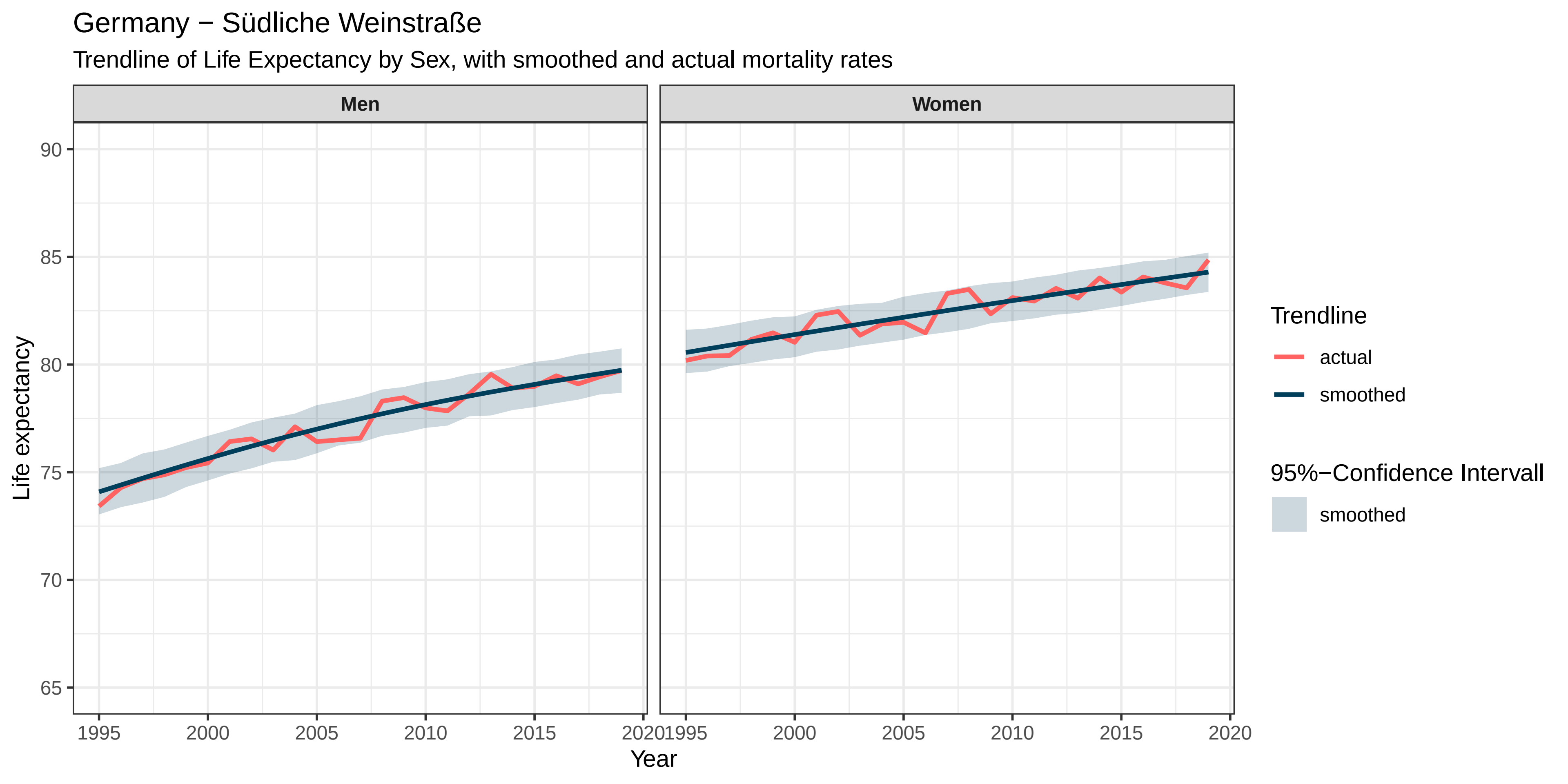
Germany − Südliche Weinstraße
Trendline of Life Expectancy by Sex, with smoothed and actual mortality rates
Men
Women
Life expectancy
Year
90
85
80
75
70
65
1995
2000
2005
2010
2015
2020
Trendline
actual
smoothed
95%−Confidence Intervall
smoothed

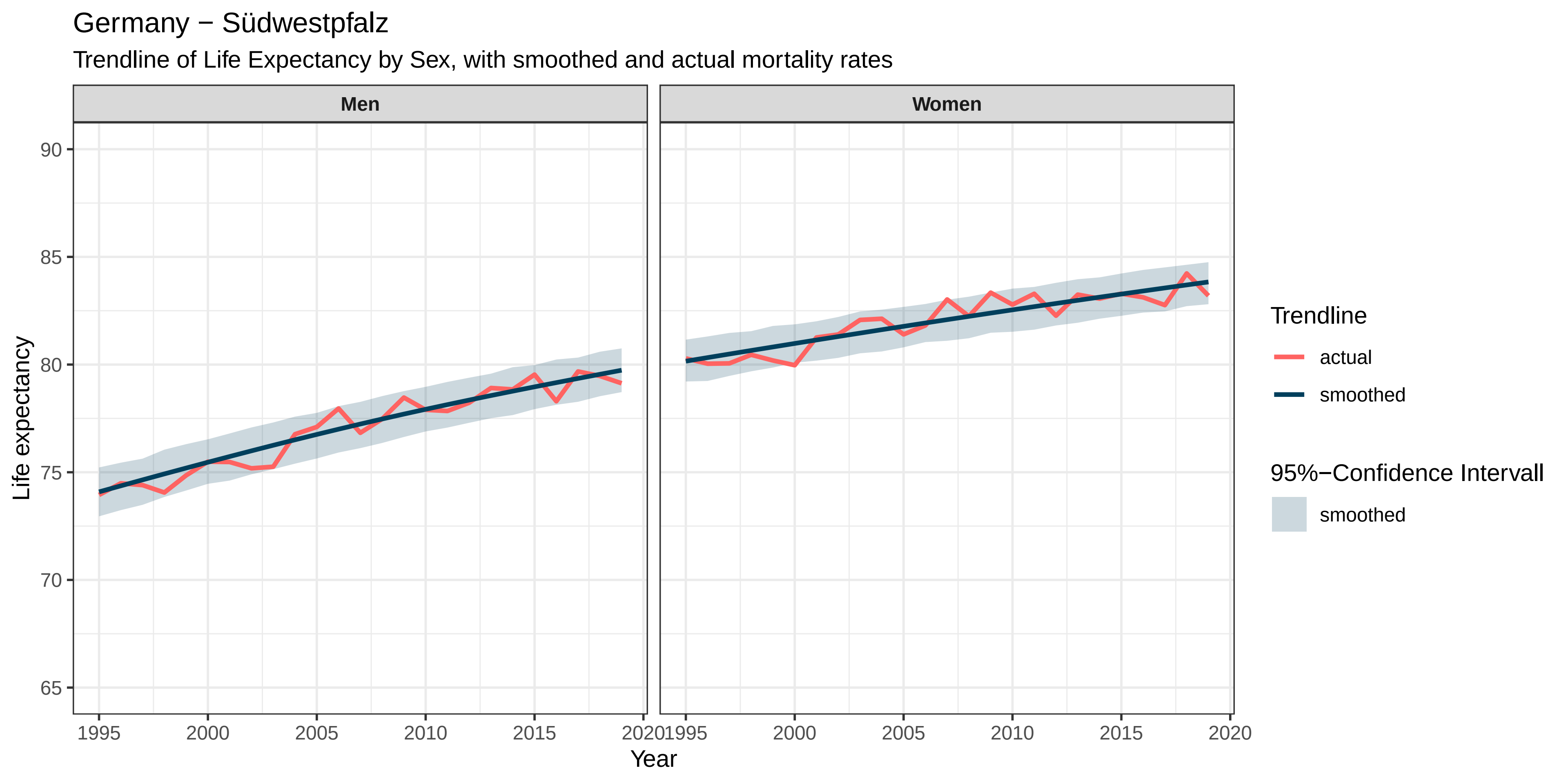

Germany − Südwestpfalz
Trendline of Life Expectancy by Sex, with smoothed and actual mortality rates
Men
Women
Life expectancy
90
85
80
75
70
65
1995
2000
2005
2010
2015
2020
Year
Trendline
actual
smoothed
95%−Confidence Intervall
smoothed

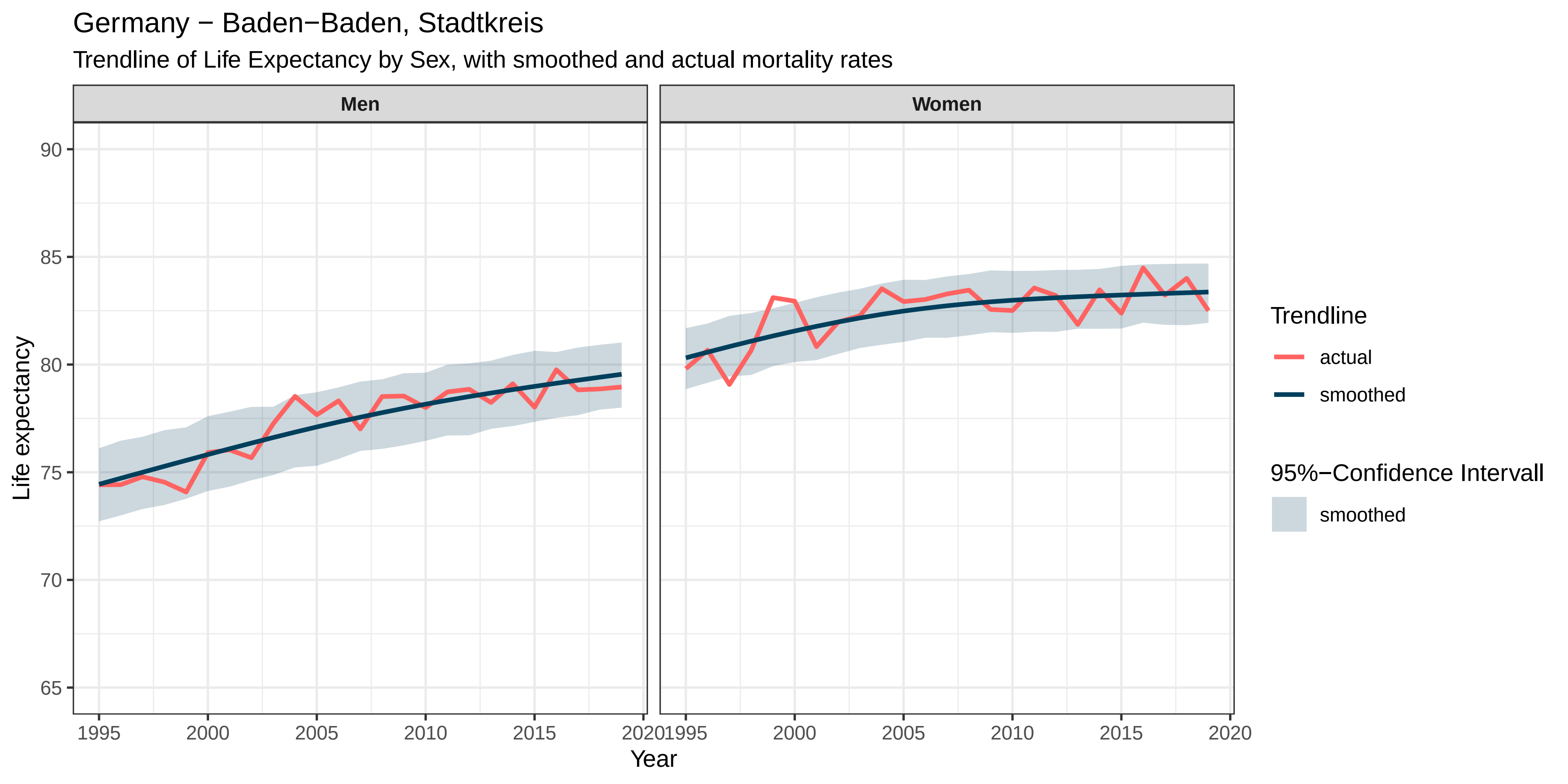

Germany − Baden−Baden, Stadtkreis
Trendline of Life Expectancy by Sex, with smoothed and actual mortality rates
Men
Women
Life expectancy
90
85
80
75
70
65
1995
2000
2005
2010
2015
2020
Year
Trendline
actual
smoothed
95%−Confidence Intervall
smoothed

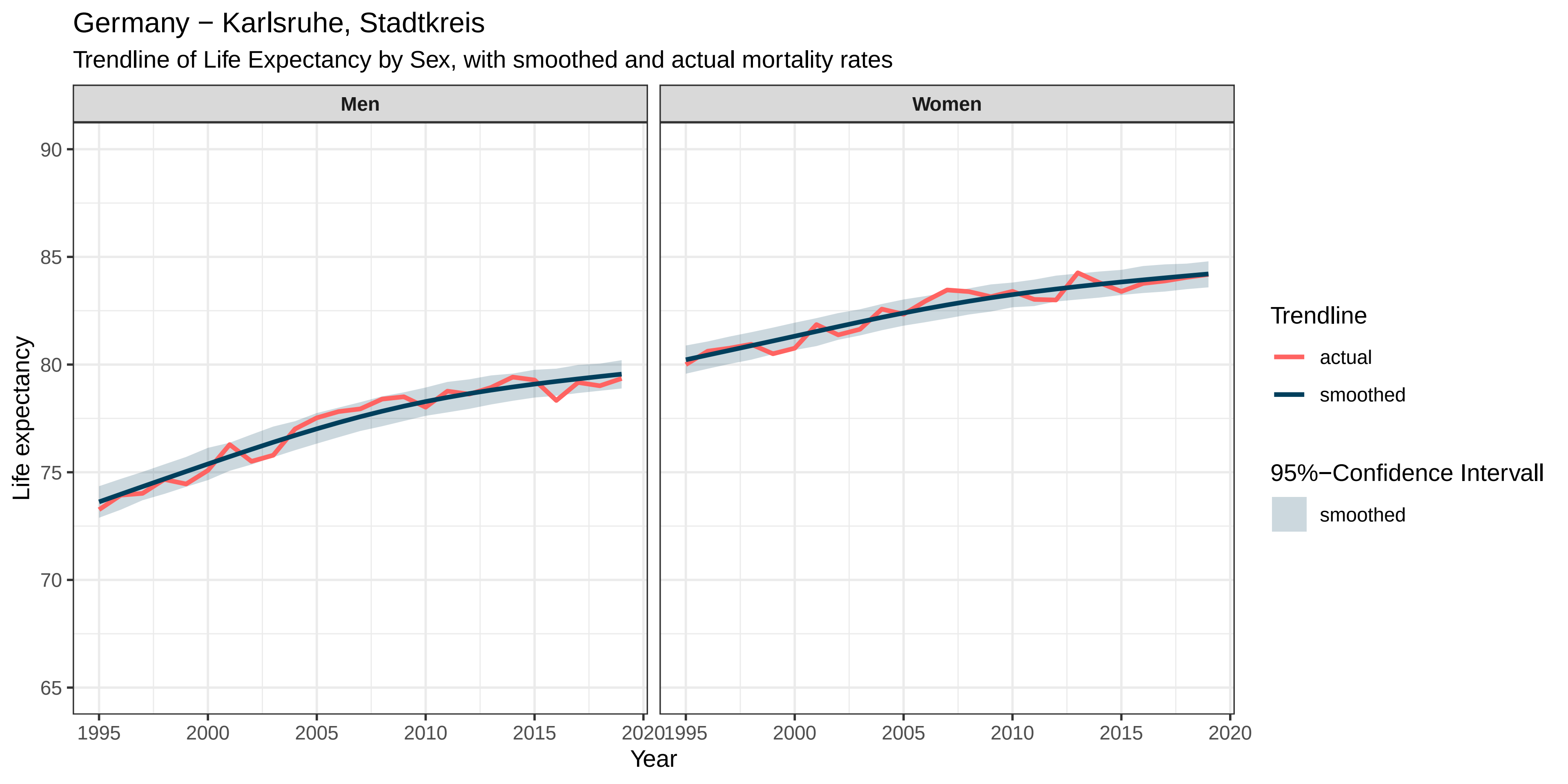

Germany − Karlsruhe, Stadtkreis
Trendline of Life Expectancy by Sex, with smoothed and actual mortality rates
Men
Women
Life expectancy
90
85
80
75
70
65
1995
2000
2005
2010
2015
2020
Year
Trendline
actual
smoothed
95%−Confidence Intervall
smoothed

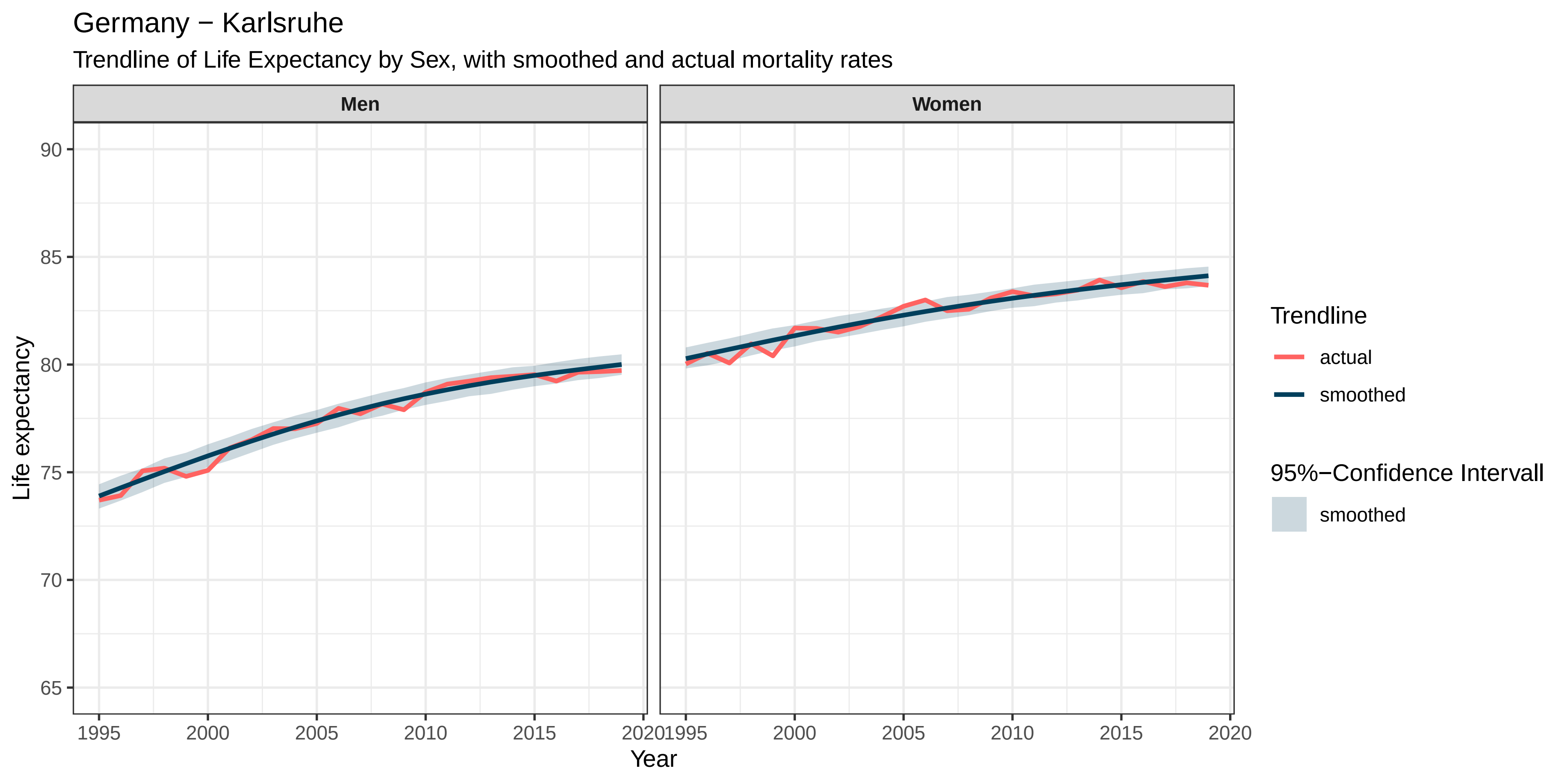

Germany − Karlsruhe
Trendline of Life Expectancy by Sex, with smoothed and actual mortality rates
Men
Women
Life expectancy
Year
90
85
80
75
70
65
1995
2000
2005
2010
2015
2020
Trendline
actual
smoothed
95%−Confidence Intervall
smoothed

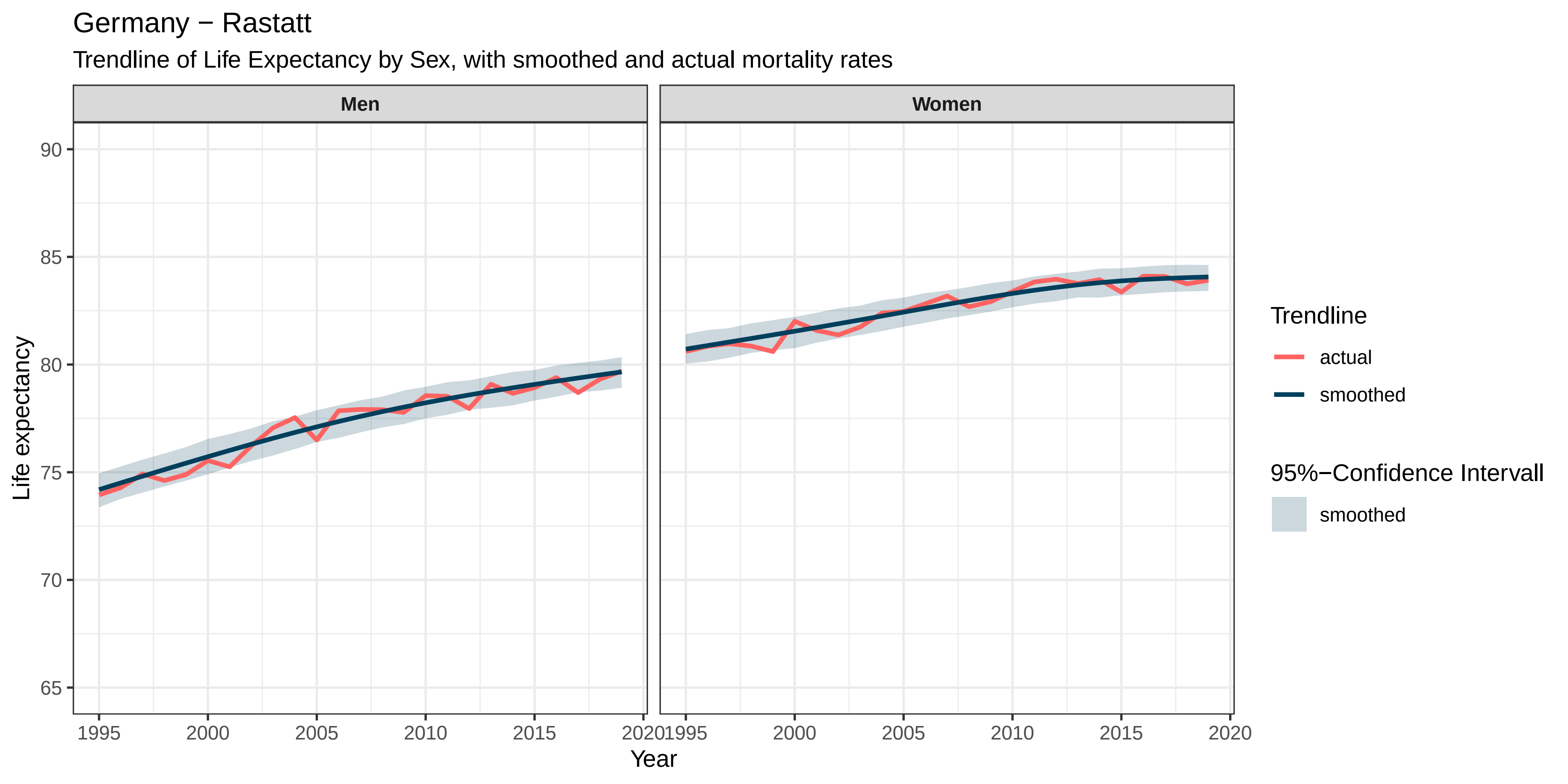

Germany − Rastatt
Trendline of Life Expectancy by Sex, with smoothed and actual mortality rates
Men
Women
Life expectancy
90
85
80
75
70
65
1995
2000
2005
2010
2015
2020
Year
Trendline
actual
smoothed
95%−Confidence Intervall
smoothed

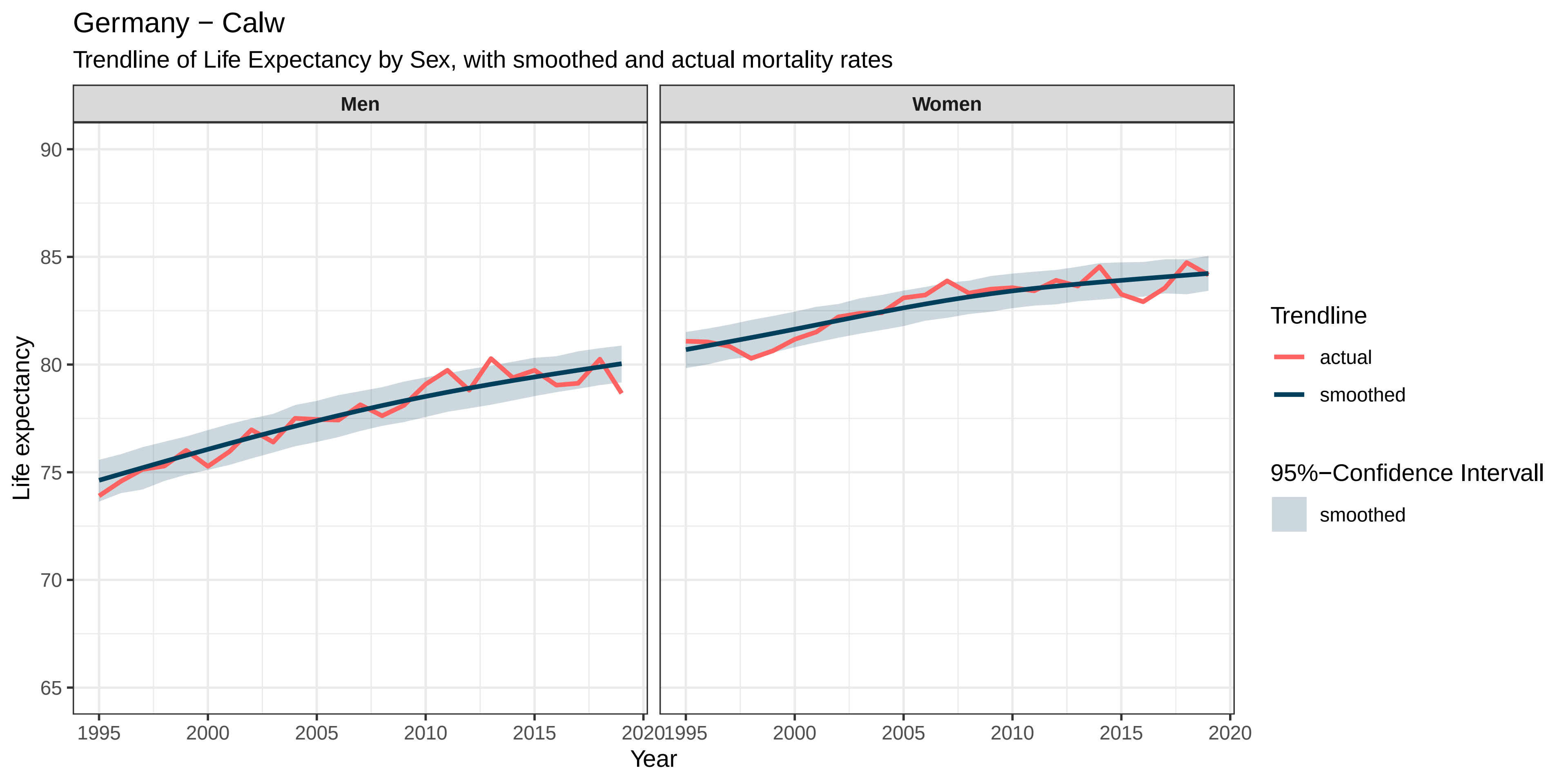

Germany − Calw
Trendline of Life Expectancy by Sex, with smoothed and actual mortality rates
Men
Women
Life expectancy
Year
90
85
80
75
70
65
1995
2000
2005
2010
2015
2020
Trendline
actual
smoothed
95%−Confidence Intervall
smoothed

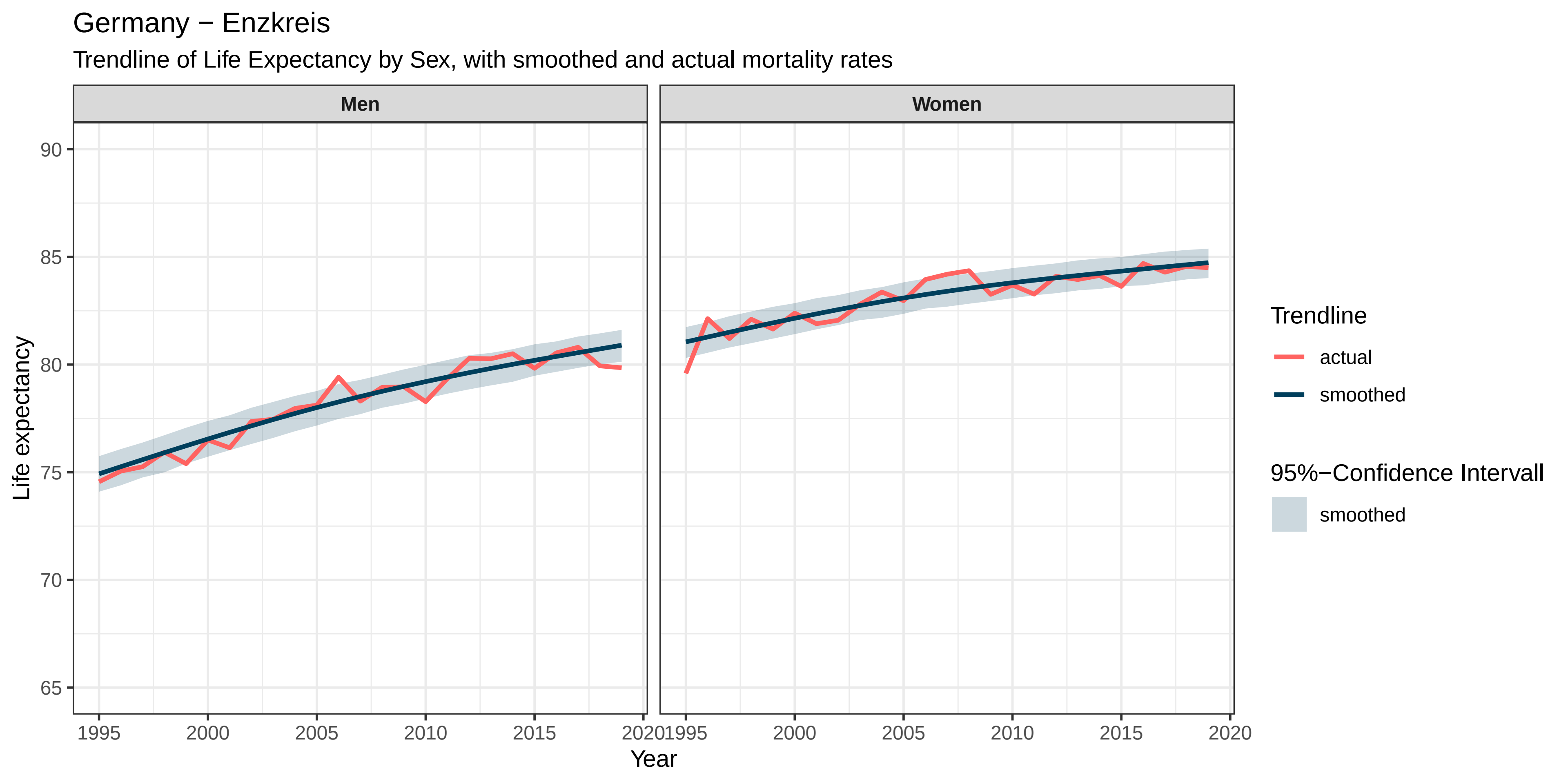
Germany − Enzkreis
Trendline of Life Expectancy by Sex, with smoothed and actual mortality rates
Men
Women
Life expectancy
90
85
80
75
70
65
1995
2000
2005
2010
2015
2020
Year
Trendline
actual
smoothed
95%−Confidence Intervall
smoothed

Germany − Freudenstadt

Trendline of Life Expectancy by Sex, with smoothed and actual mortality rates

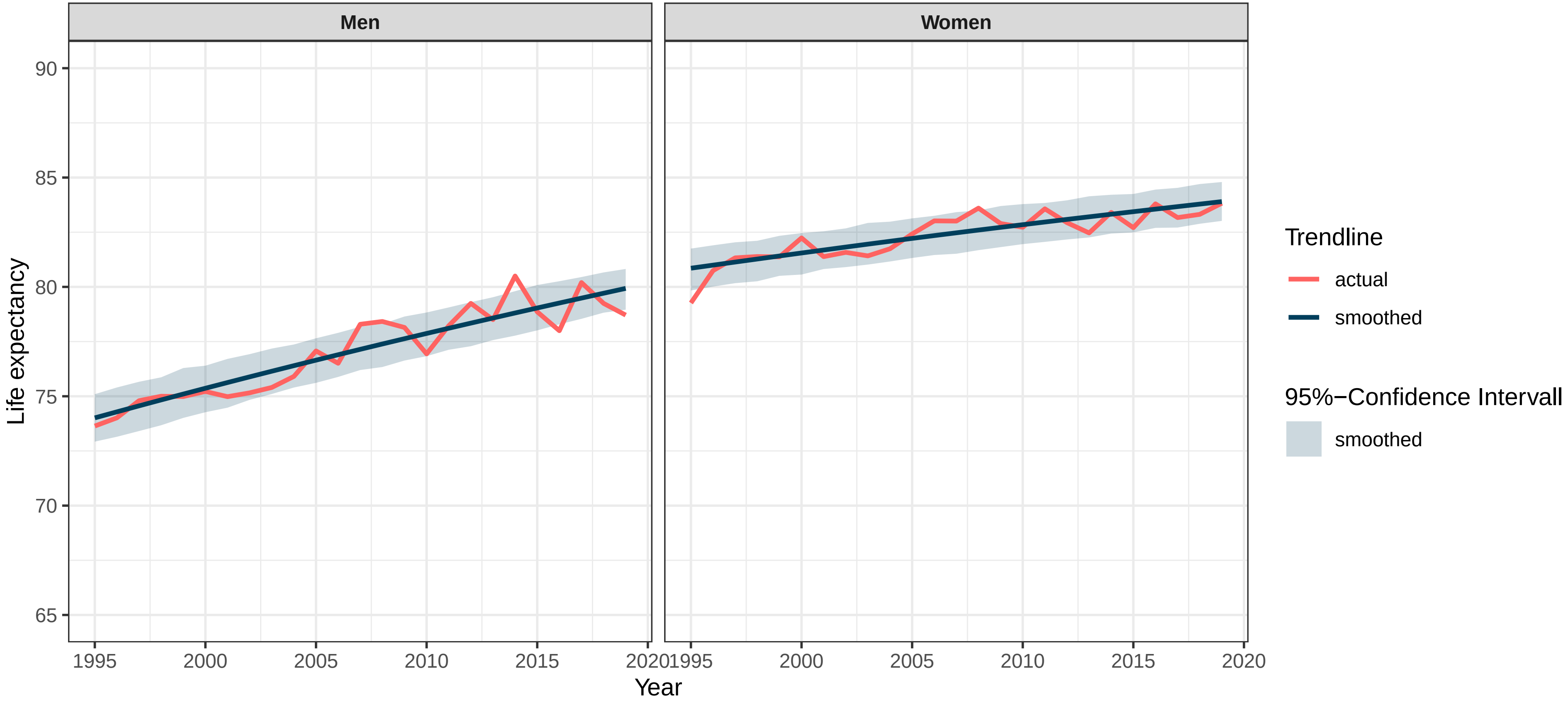

Germany − Freiburg im Breisgau, Stadtkreis

Trendline of Life Expectancy by Sex, with smoothed and actual mortality rates

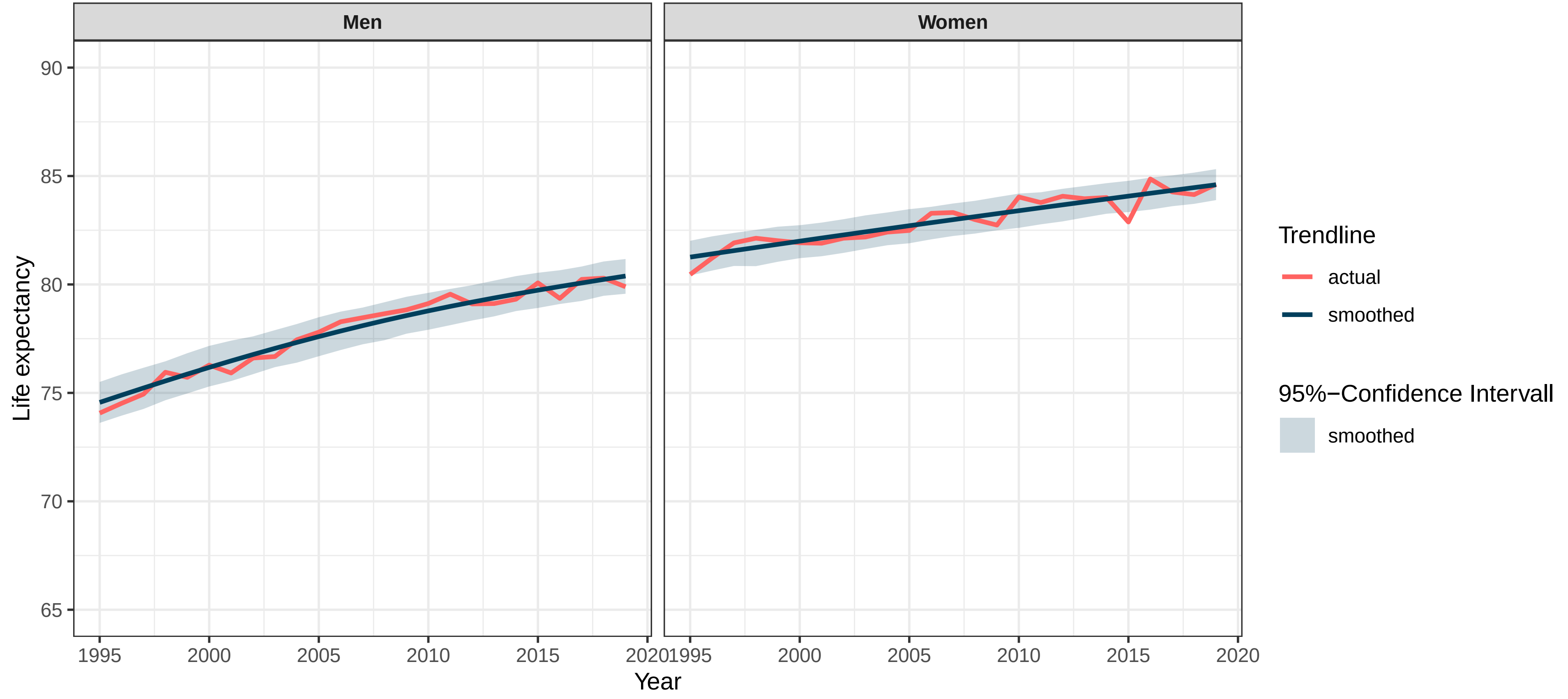

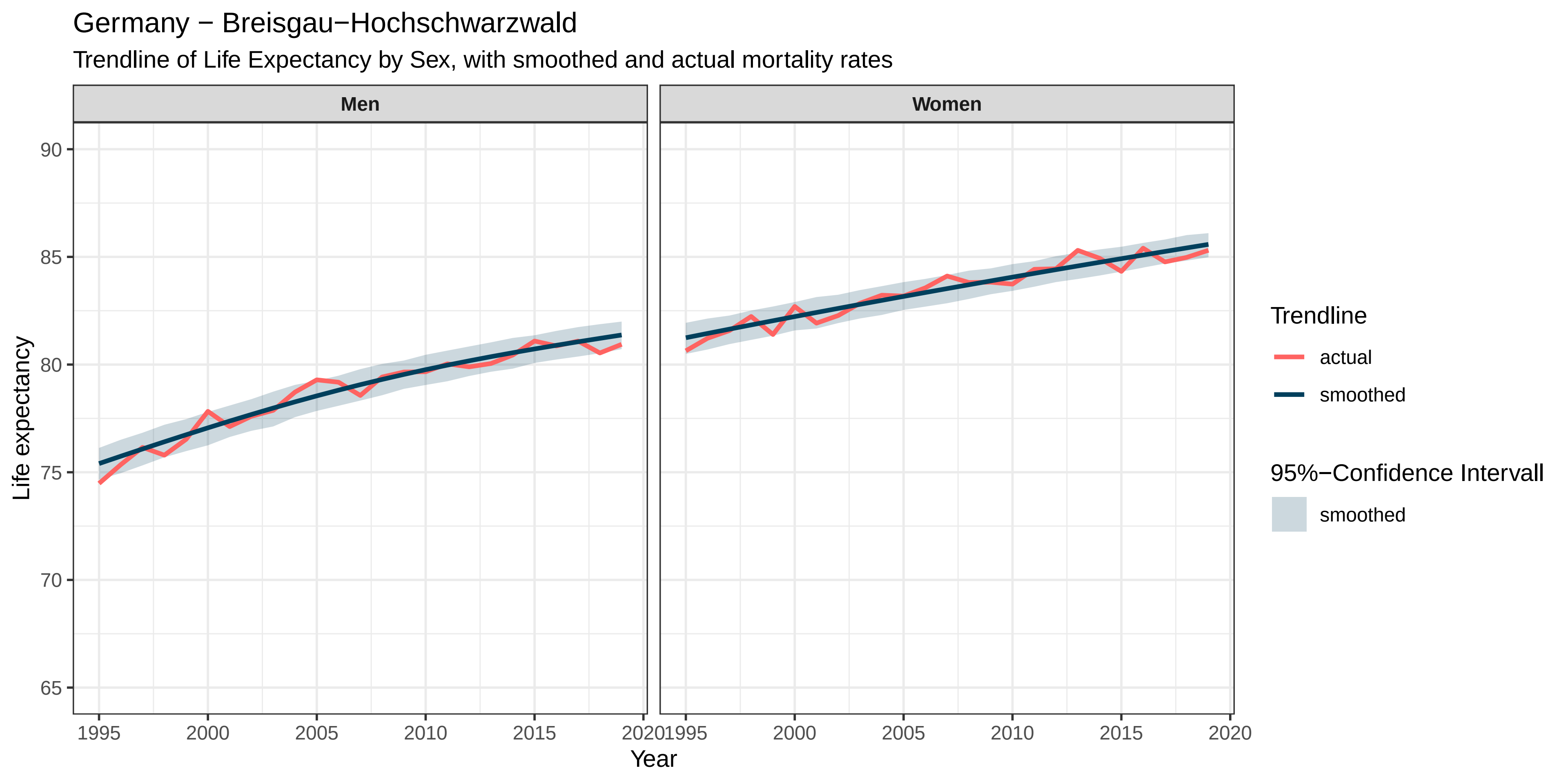
Germany − Breisgau−Hochschwarzwald
Trendline of Life Expectancy by Sex, with smoothed and actual mortality rates
Men
Women
Life expectancy
Year
90
85
80
75
70
65
1995
2000
2005
2010
2015
2020
Trendline
actual
smoothed
95%−Confidence Intervall
smoothed

Germany − Emmendingen

Trendline of Life Expectancy by Sex, with smoothed and actual mortality rates

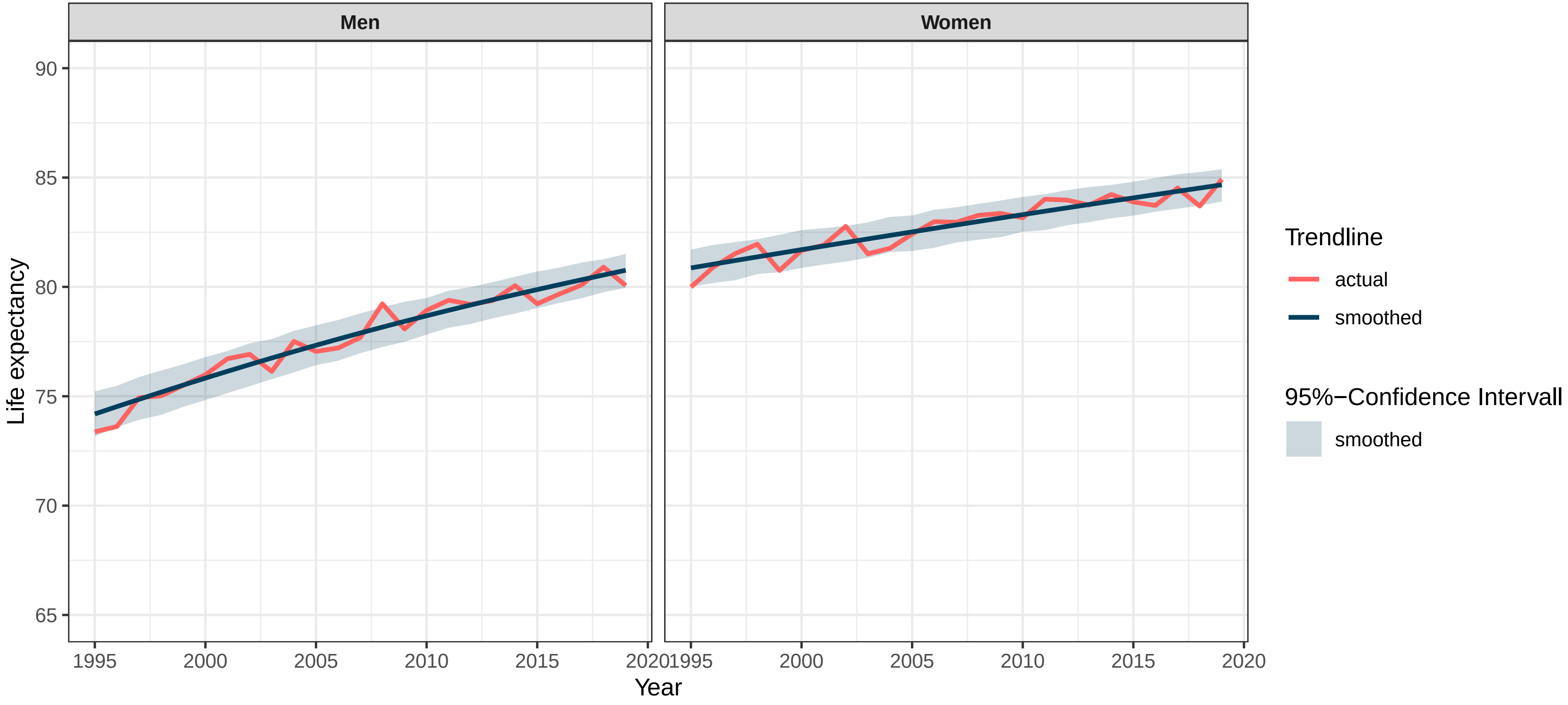

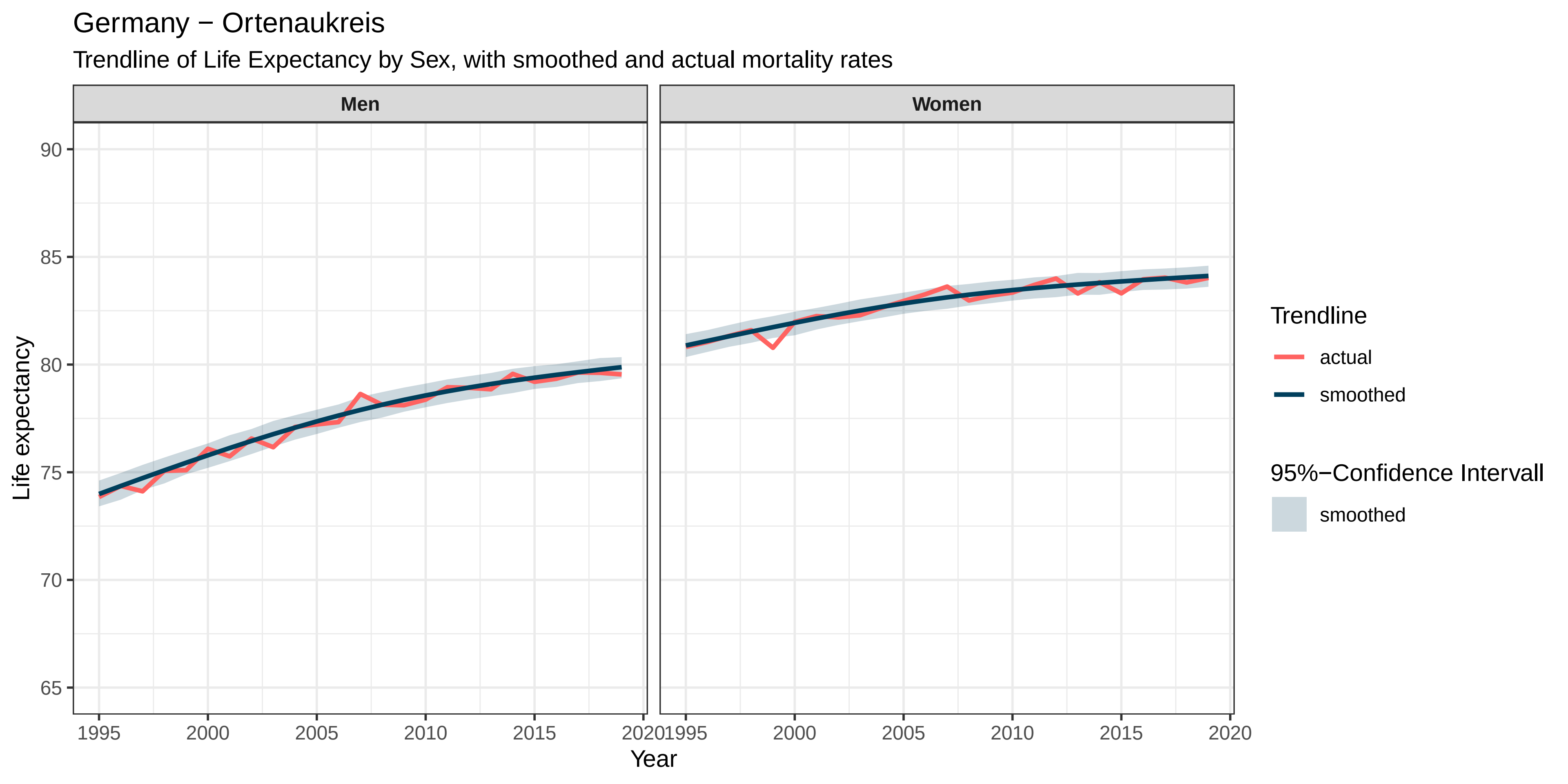
Germany − Ortenaukreis
Trendline of Life Expectancy by Sex, with smoothed and actual mortality rates
Men
Women
90
85
80
75
70
65
1995
2000
2005
2010
2015
2020
Year
Life expectancy
Trendline
actual
smoothed
95%−Confidence Intervall
smoothed

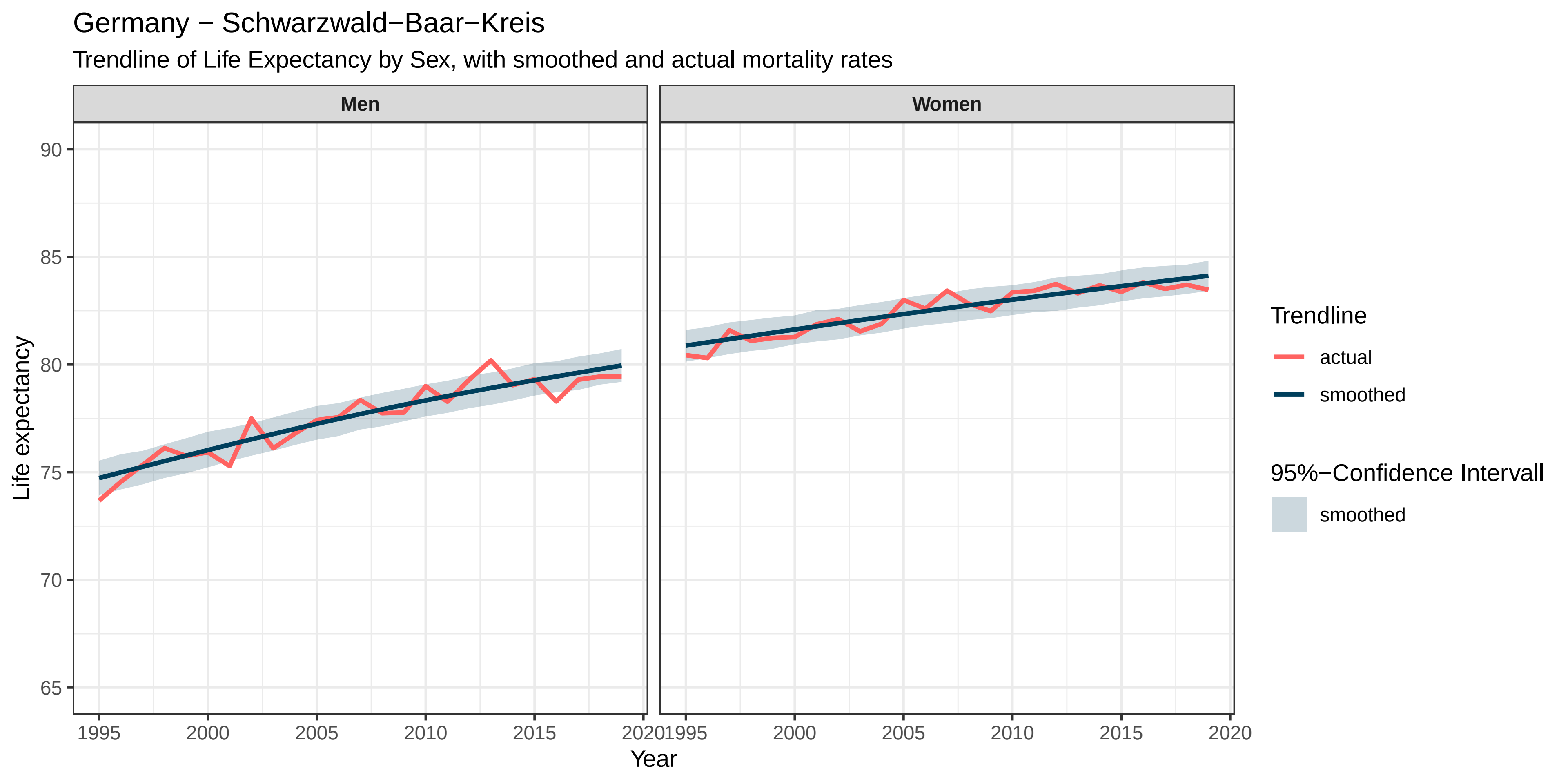

Germany − Schwarzwald−Baar−Kreis
Trendline of Life Expectancy by Sex, with smoothed and actual mortality rates
Men
Women
90
85
80
75
70
65
Life expectancy
1995
2000
2005
2010
2015
2020
Year
Trendline
actual
smoothed
95%−Confidence Intervall
smoothed

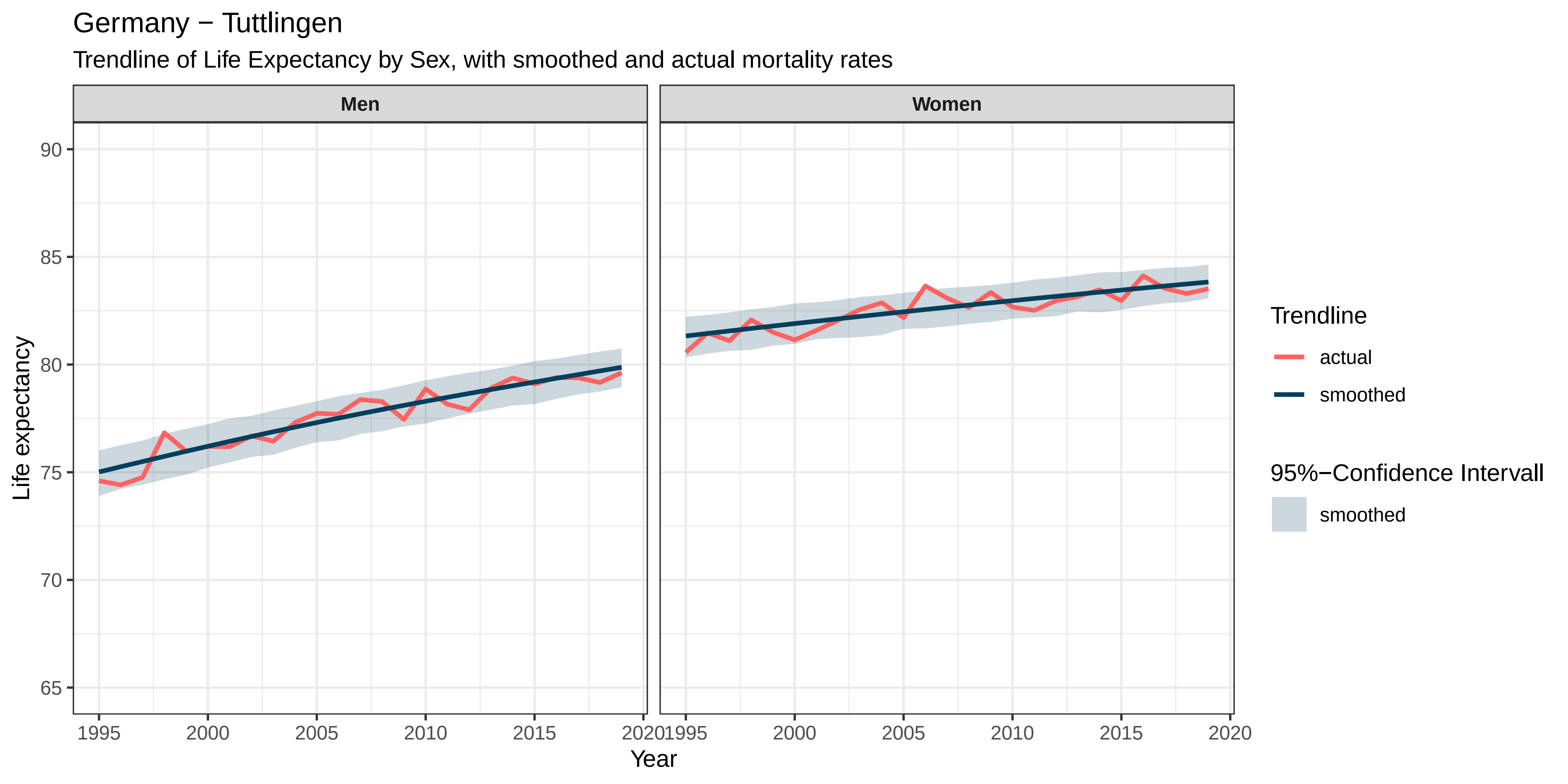
Germany − Tuttlingen
Trendline of Life Expectancy by Sex, with smoothed and actual mortality rates
Men
Women
Life expectancy
Year
90
85
80
75
70
65
1995
2000
2005
2010
2015
2020
Trendline
actual
smoothed
95%−Confidence Intervall
smoothed

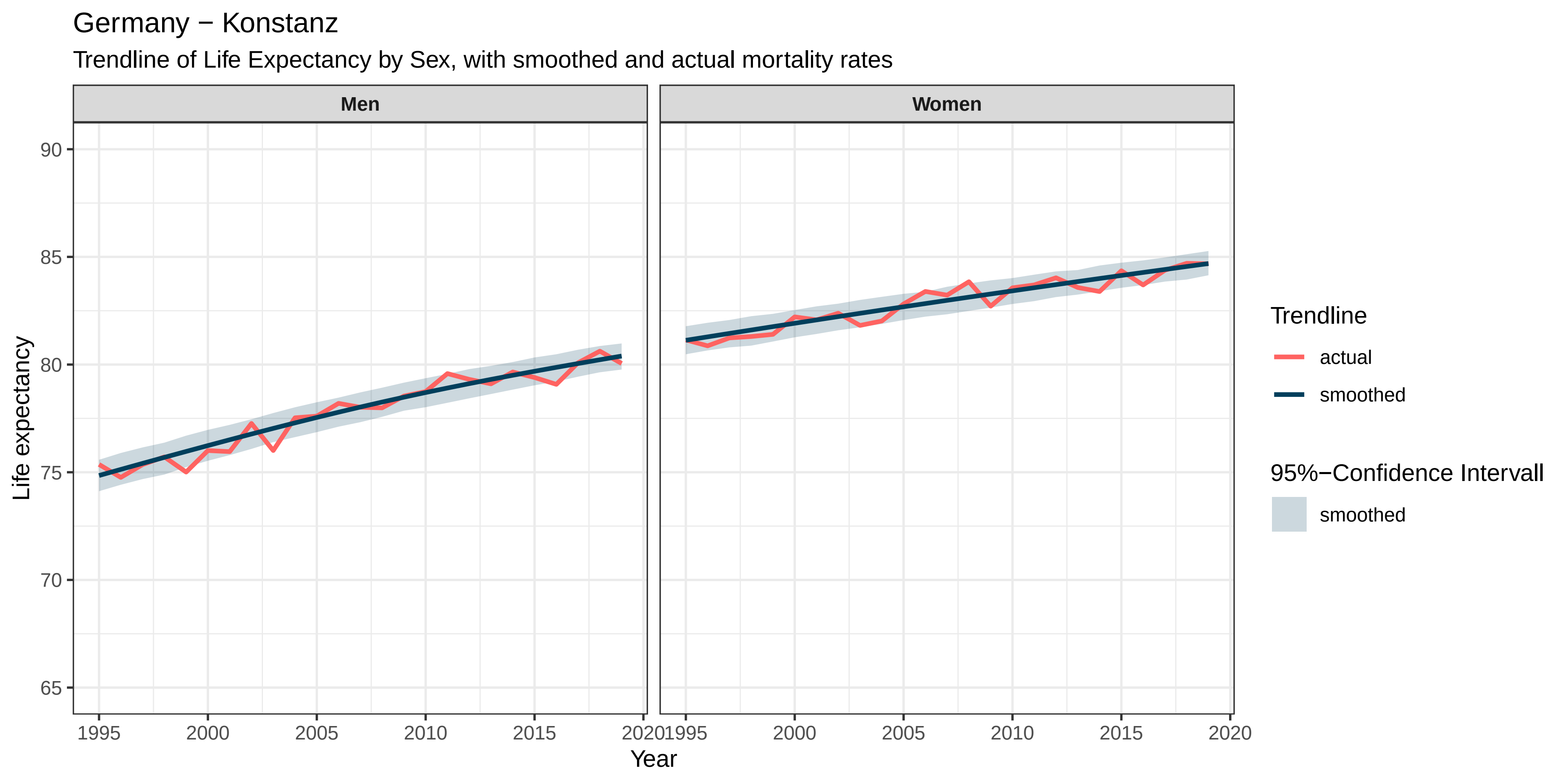
Germany − Konstanz
Trendline of Life Expectancy by Sex, with smoothed and actual mortality rates
Men
Women
Life expectancy
90
85
80
75
70
65
1995
2000
2005
2010
2015
2020
Year
Trendline
actual
smoothed
95%−Confidence Intervall
smoothed

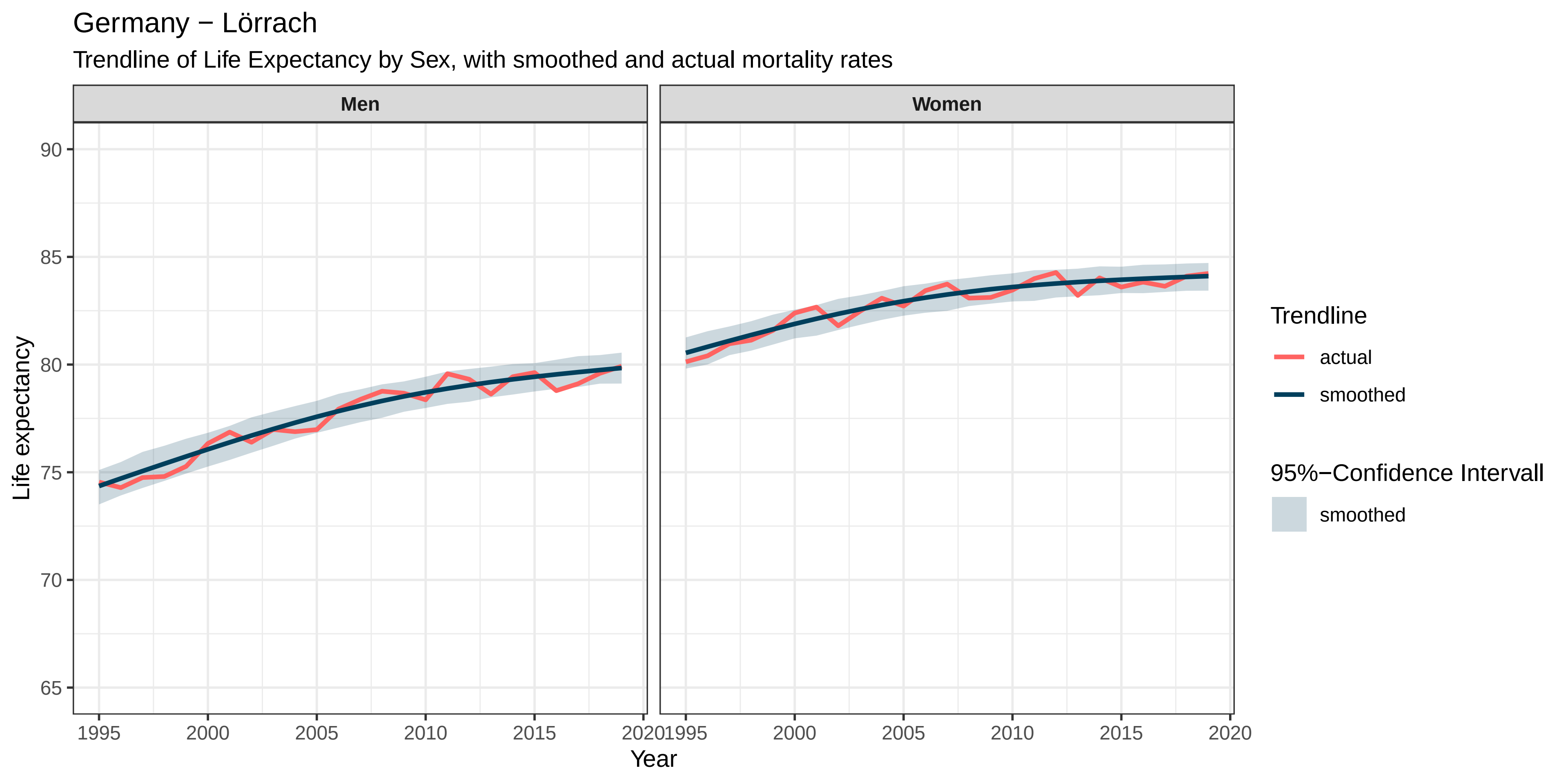

Germany − Lörrach
Trendline of Life Expectancy by Sex, with smoothed and actual mortality rates
Men
Women
Life expectancy
Year
90
85
80
75
70
65
1995
2000
2005
2010
2015
2020
Trendline
actual
smoothed
95%−Confidence Intervall
smoothed

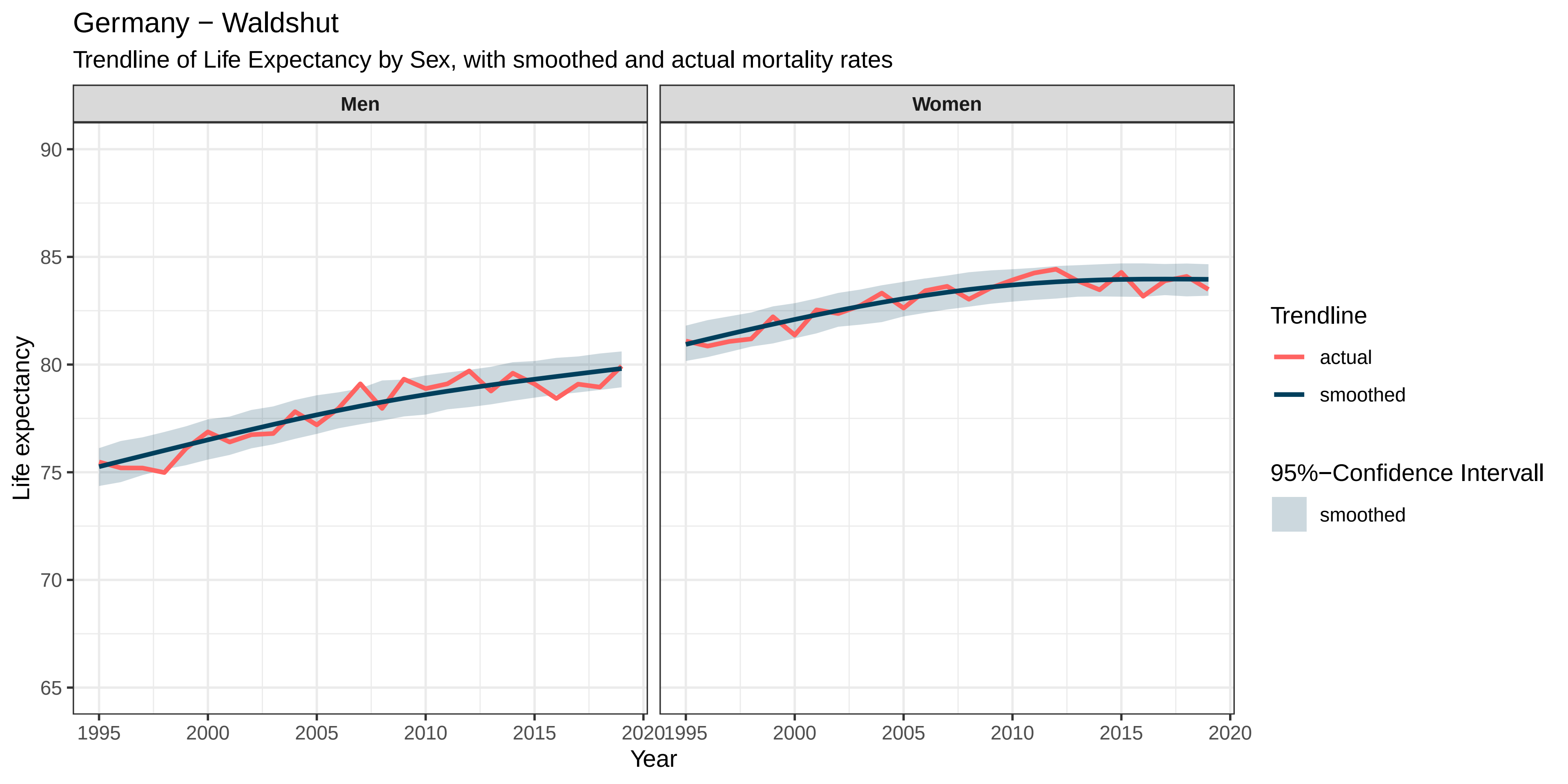

Germany − Waldshut
Trendline of Life Expectancy by Sex, with smoothed and actual mortality rates
Men
Women
Life expectancy
90
85
80
75
70
65
1995
2000
2005
2010
2015
2020
Year
Trendline
actual
smoothed
95%−Confidence Intervall
smoothed

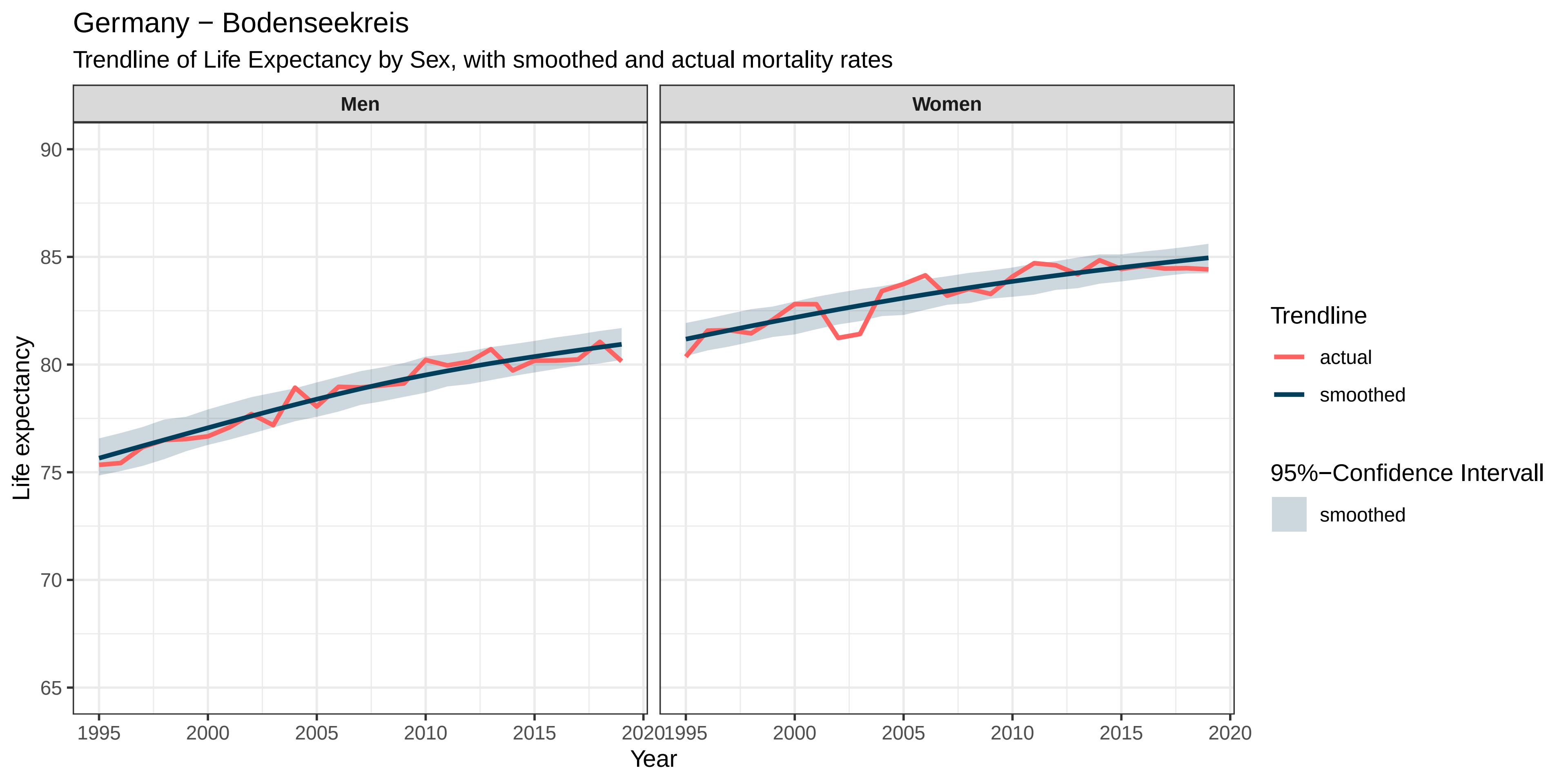
Germany − Bodenseekreis
Trendline of Life Expectancy by Sex, with smoothed and actual mortality rates
Men
Women
Life expectancy
90
85
80
75
70
65
1995
2000
2005
2010
2015
2020
Year
Trendline
actual
smoothed
95%−Confidence Intervall
smoothed

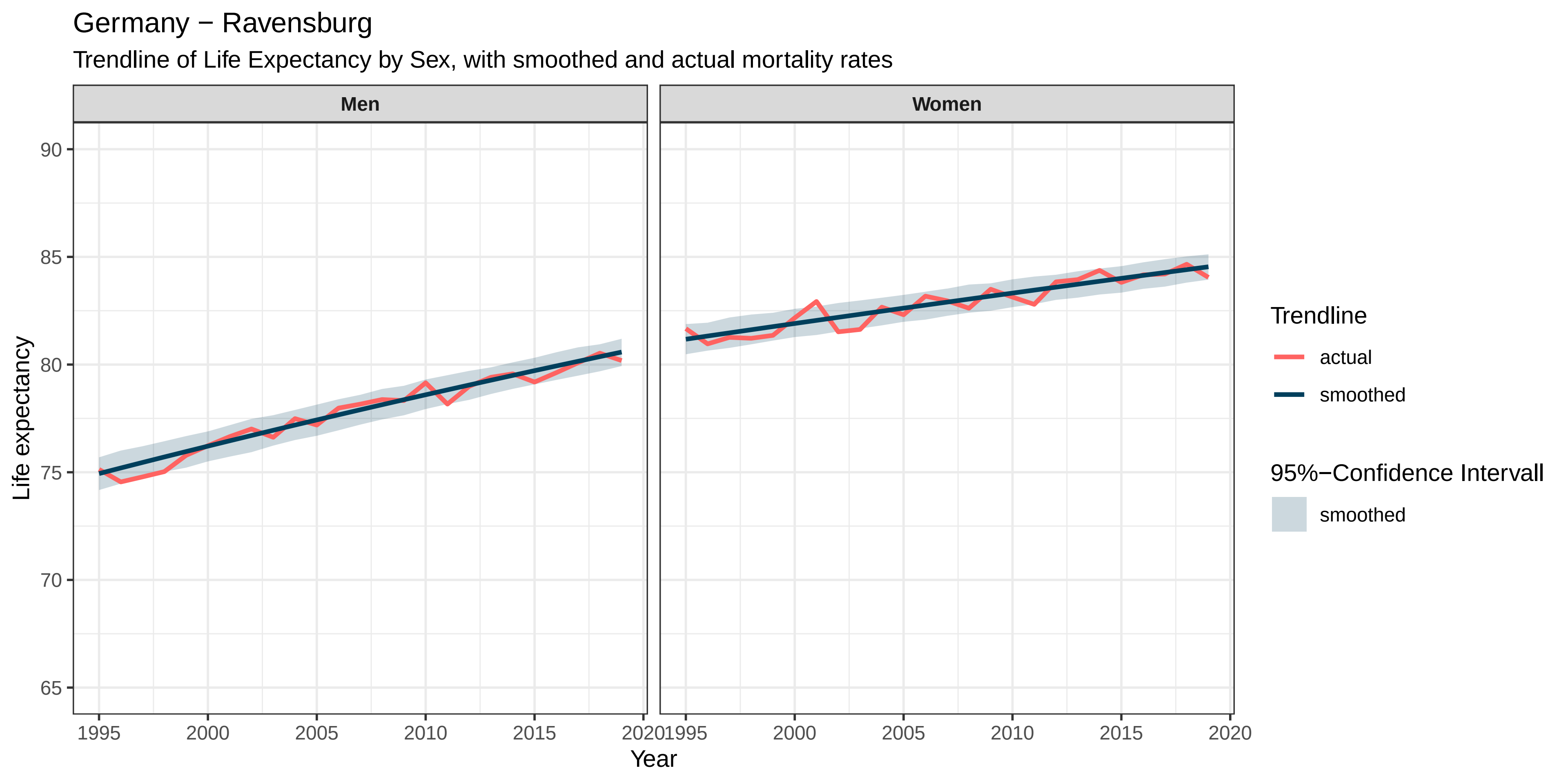
Germany − Ravensburg
Trendline of Life Expectancy by Sex, with smoothed and actual mortality rates
Men
Women
Life expectancy
90
85
80
75
70
65
1995
2000
2005
2010
2015
2020
Year
Trendline
actual
smoothed
95%−Confidence Intervall
smoothed

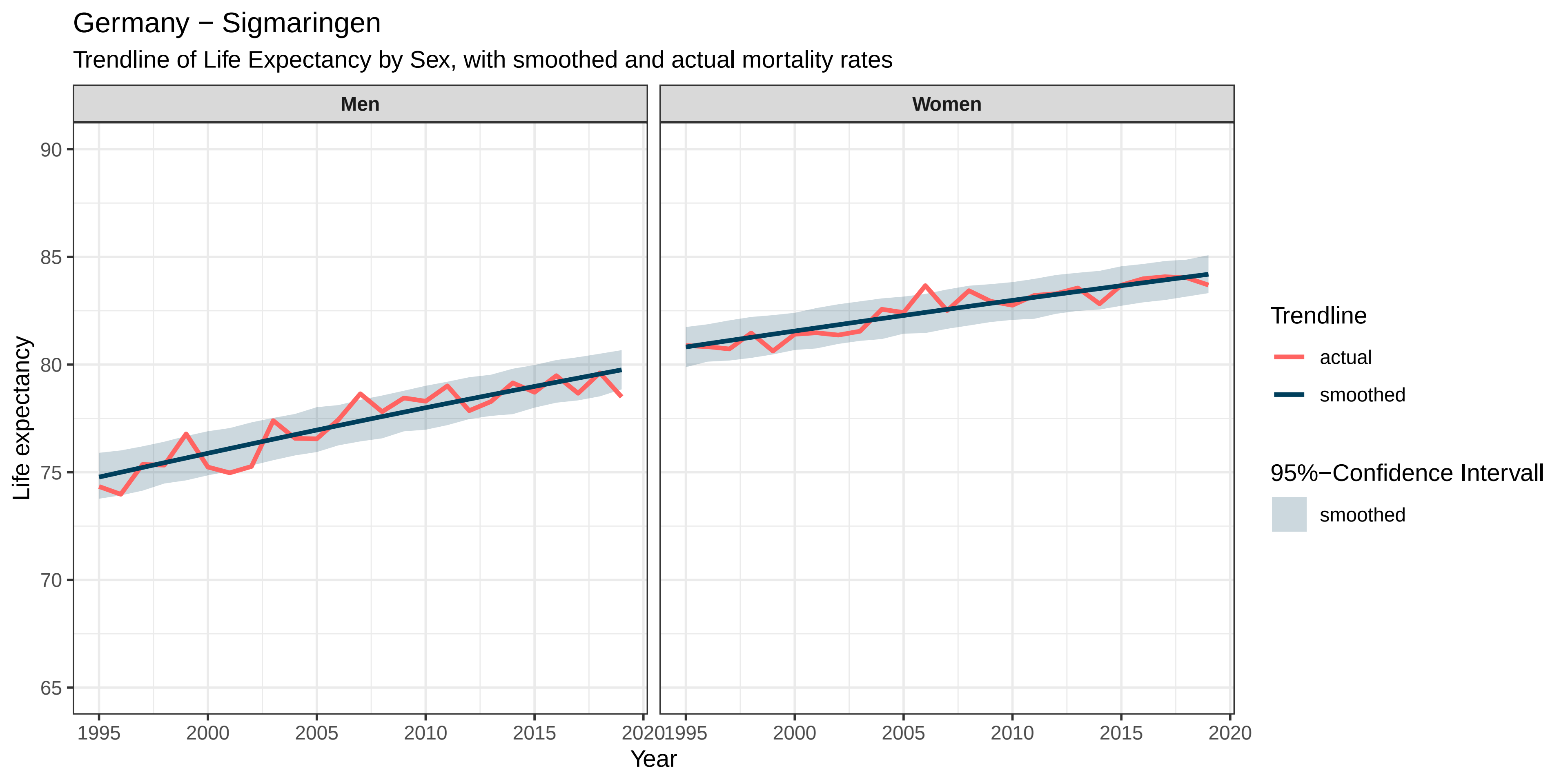
Germany − Sigmaringen
Trendline of Life Expectancy by Sex, with smoothed and actual mortality rates
Men
Women
Life expectancy
Year
90
85
80
75
70
65
1995
2000
2005
2010
2015
2020
Trendline
actual
smoothed
95%−Confidence Intervall
smoothed

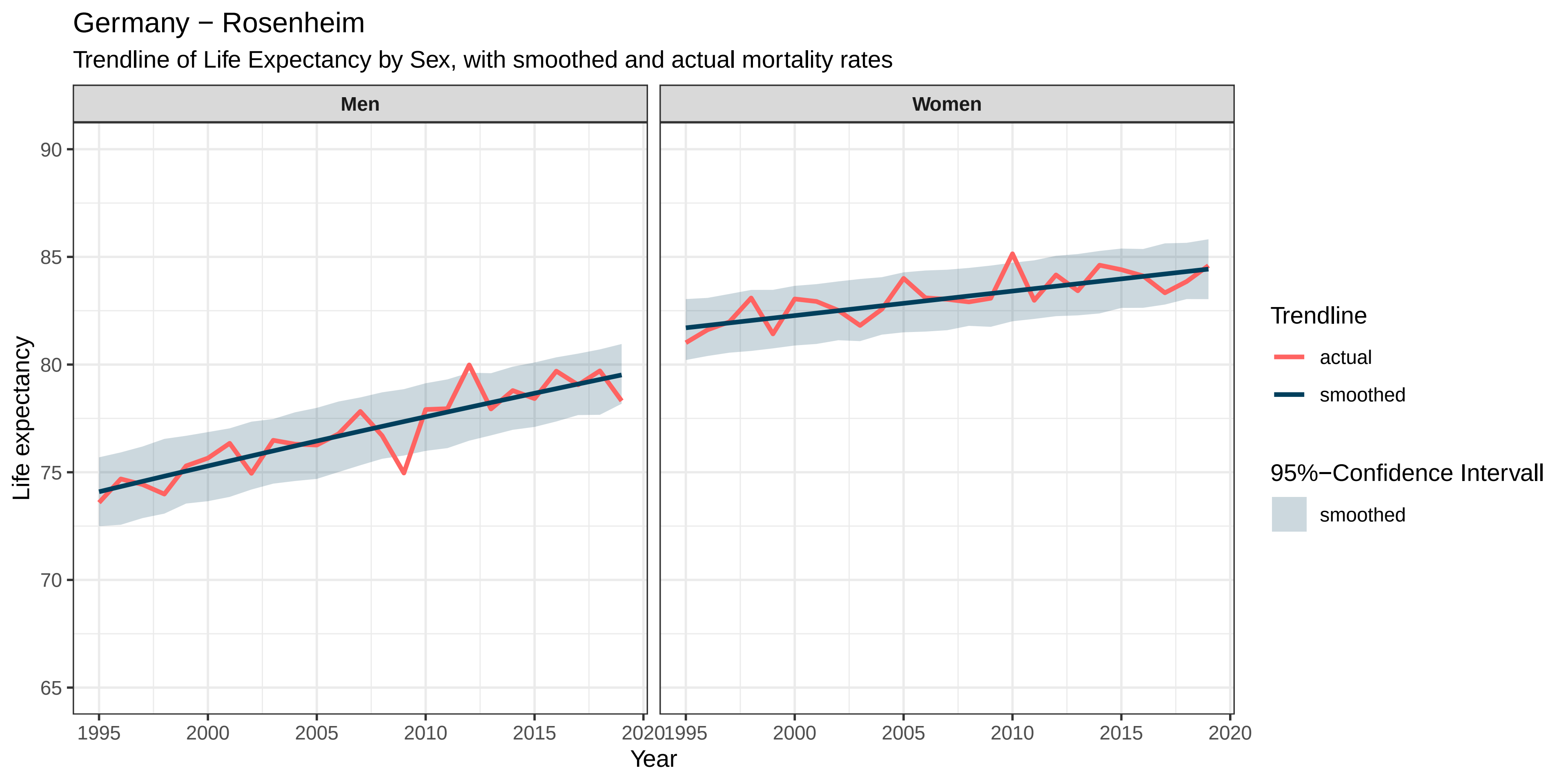

Germany − Rosenheim
Trendline of Life Expectancy by Sex, with smoothed and actual mortality rates
Men
Women
Life expectancy
90
85
80
75
70
65
1995
2000
2005
2010
2015
2020
Year
Trendline
actual
smoothed
95%−Confidence Intervall
smoothed

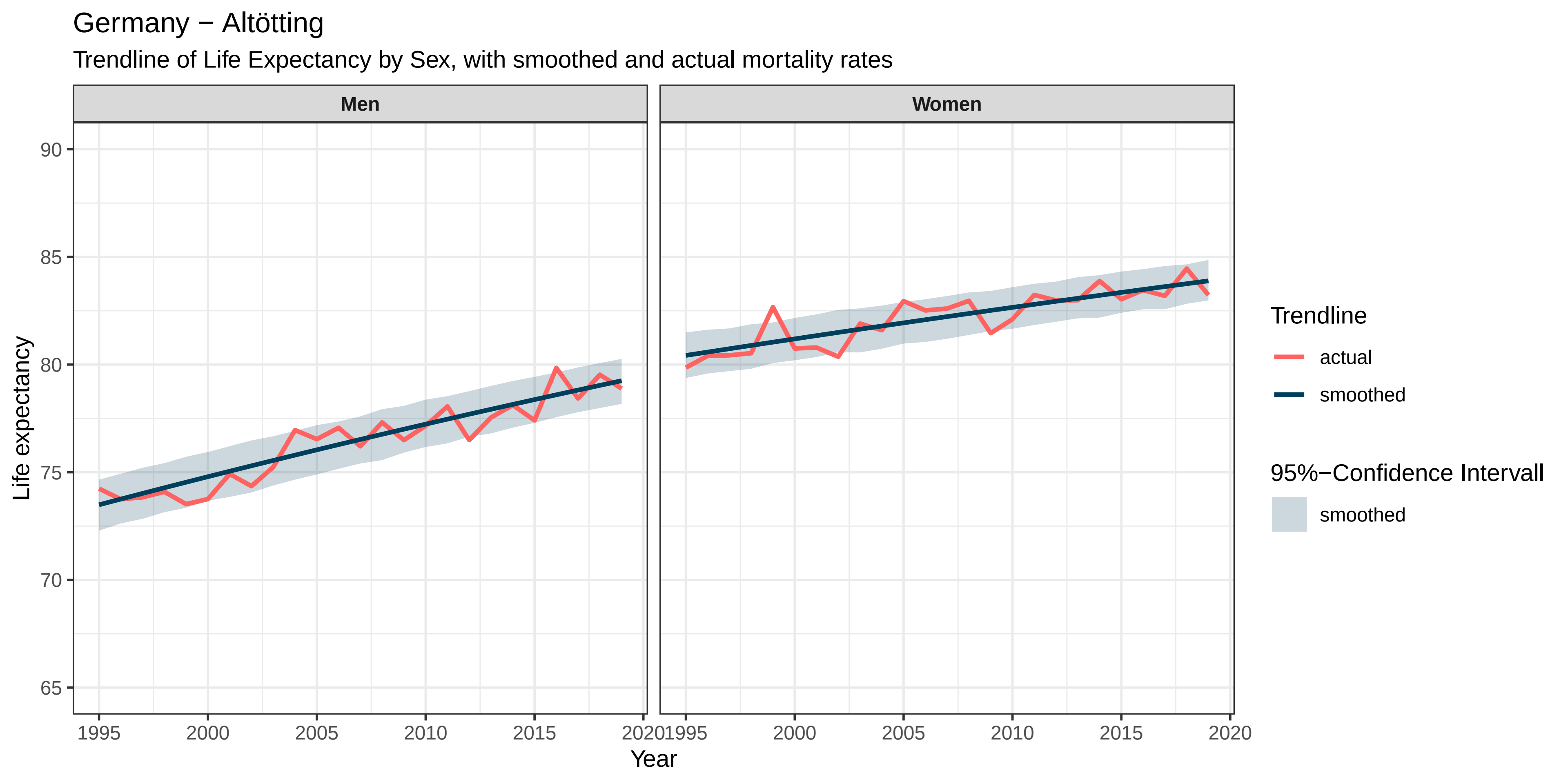
Germany − Altötting
Trendline of Life Expectancy by Sex, with smoothed and actual mortality rates
Men
Women
Life expectancy
90
85
80
75
70
65
1995
2000
2005
2010
2015
2020
Year
Trendline
actual
smoothed
95%−Confidence Intervall
smoothed

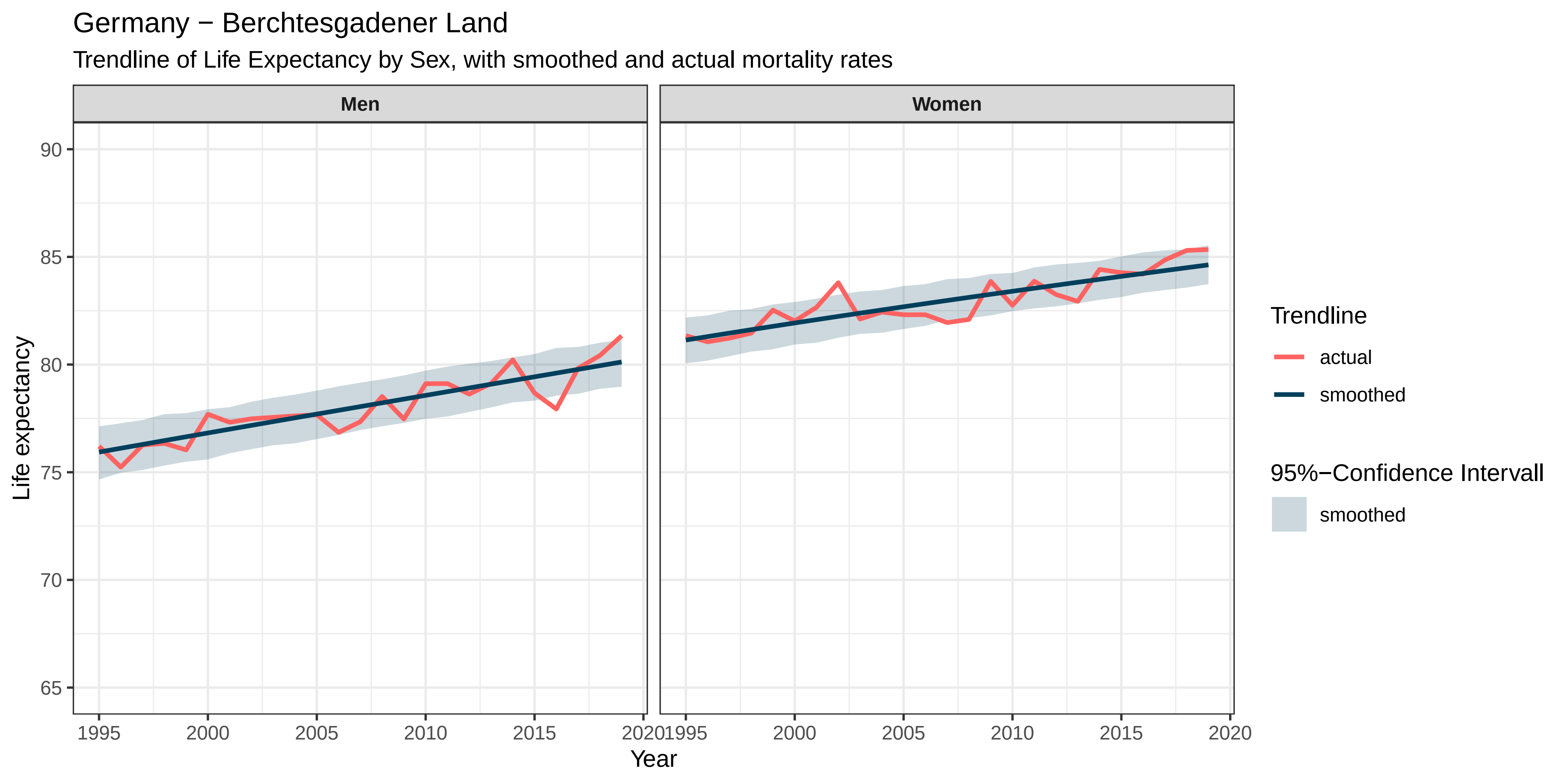

Germany − Berchtesgadener Land
Trendline of Life Expectancy by Sex, with smoothed and actual mortality rates
Men
Women
Life expectancy
90
85
80
75
70
65
1995
2000
2005
2010
2015
2020
Year
Trendline
actual
smoothed
95%−Confidence Intervall
smoothed

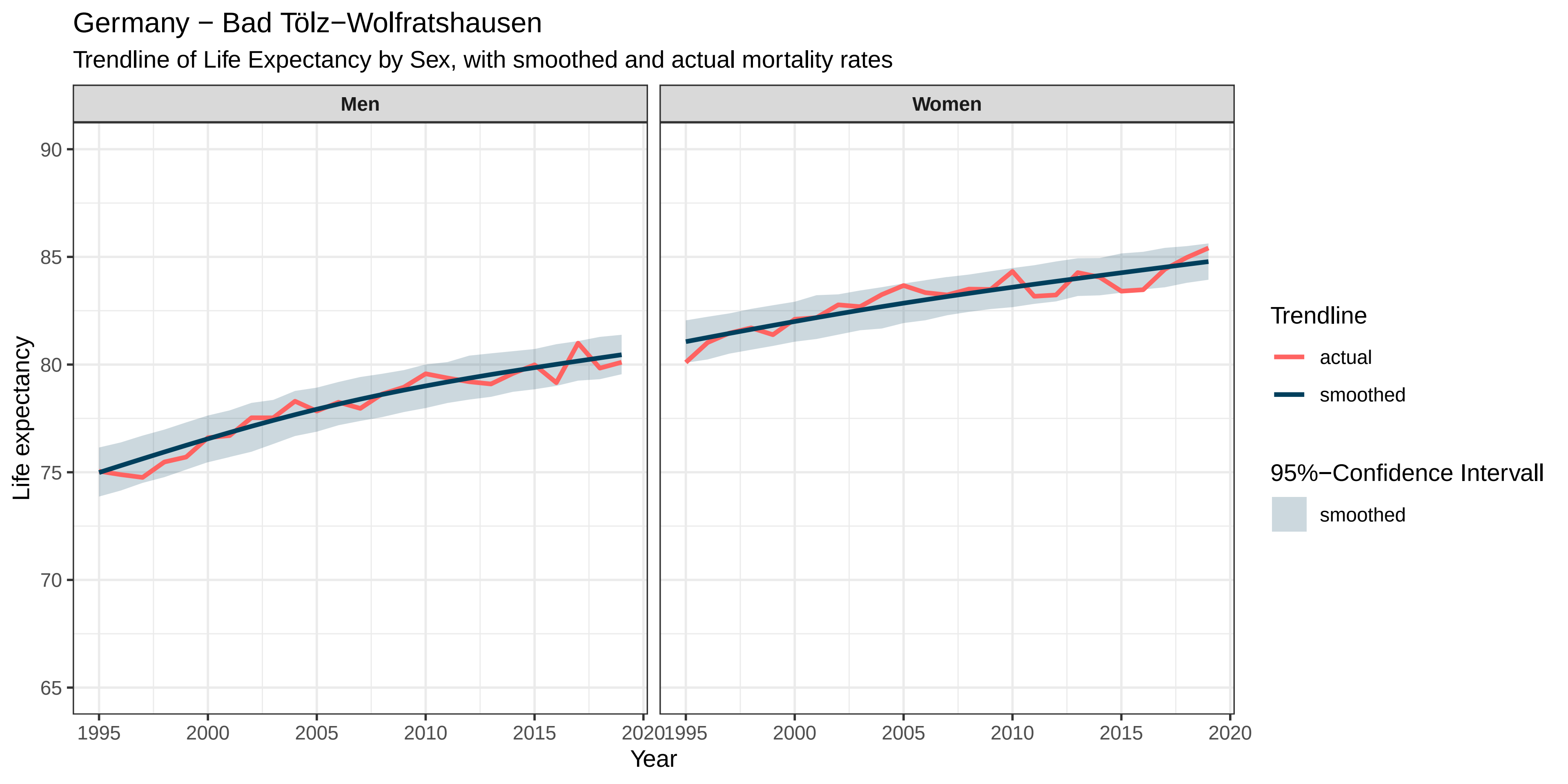

Germany − Bad Tölz−Wolfratshausen
Trendline of Life Expectancy by Sex, with smoothed and actual mortality rates
Men
Women
Life expectancy
Year
90
85
80
75
70
65
1995
2000
2005
2010
2015
2020
Trendline
actual
smoothed
95%−Confidence Intervall
smoothed

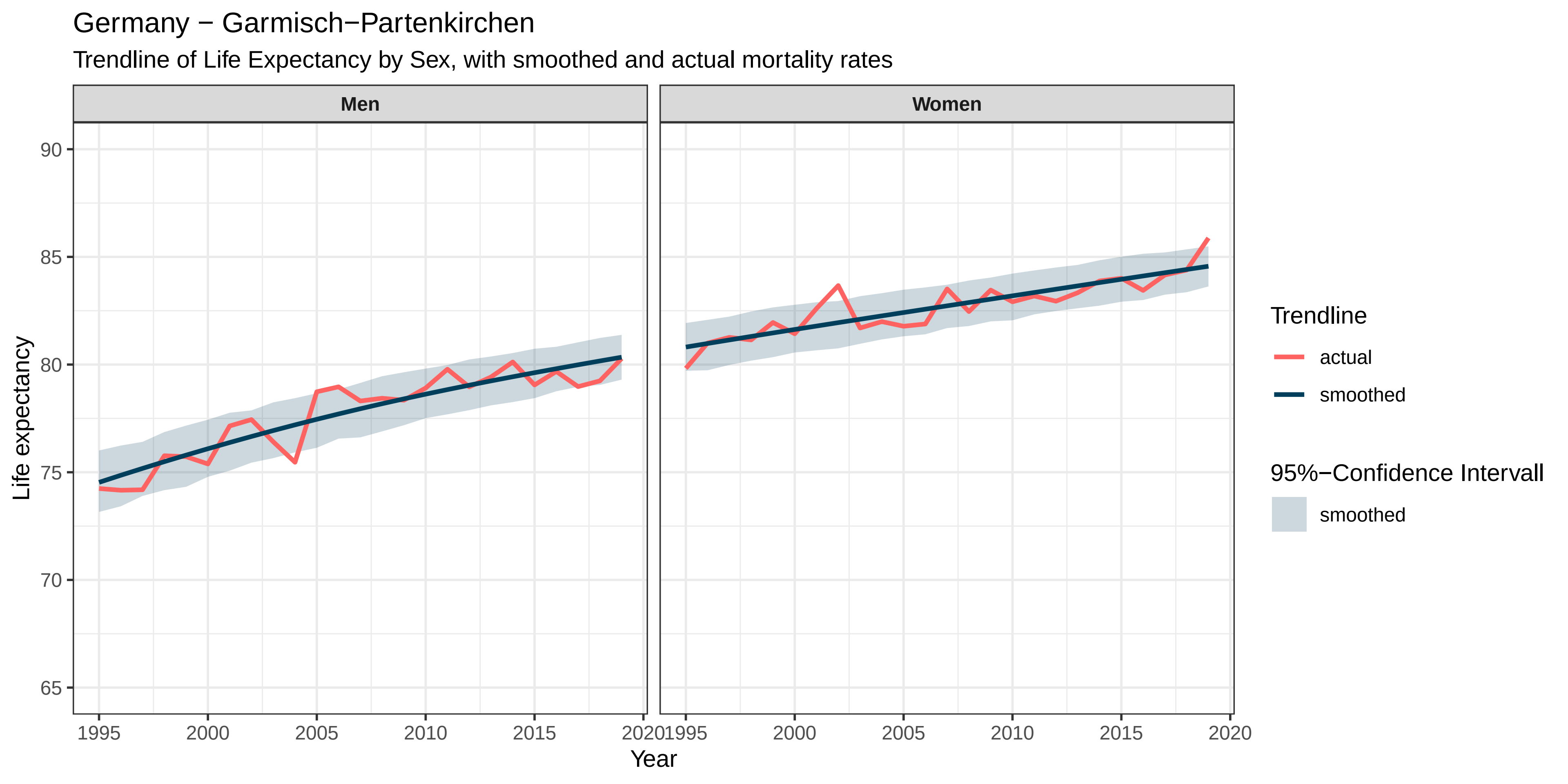
Germany − Garmisch−Partenkirchen
Trendline of Life Expectancy by Sex, with smoothed and actual mortality rates
Men
Women
Life expectancy
Year
Trendline
actual
smoothed
95%−Confidence Intervall
smoothed

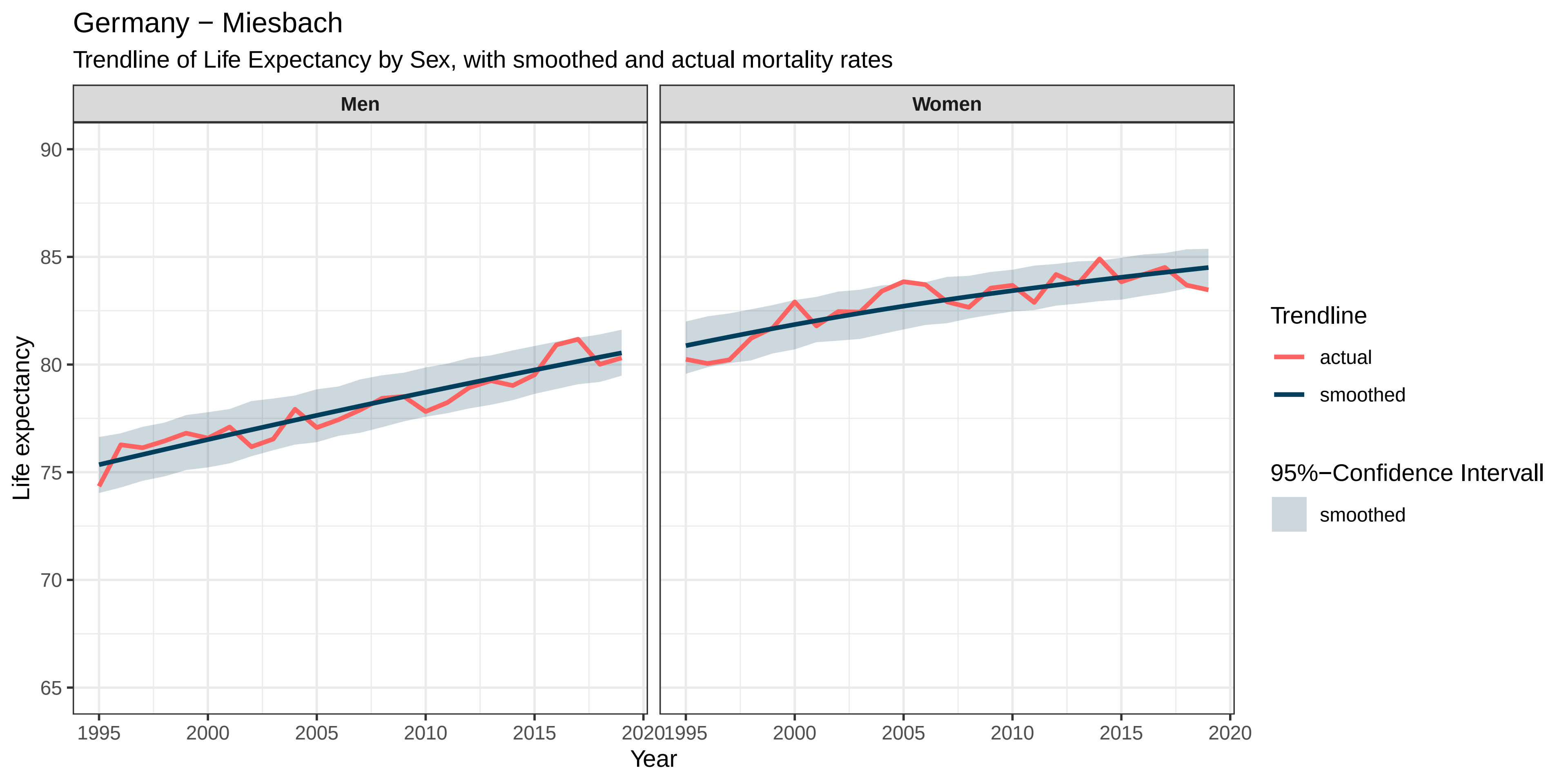

Germany − Miesbach
Trendline of Life Expectancy by Sex, with smoothed and actual mortality rates
Men
Women
Life expectancy
90
85
80
75
70
65
1995
2000
2005
2010
2015
2020
Year
Trendline
actual
smoothed
95%−Confidence Intervall
smoothed

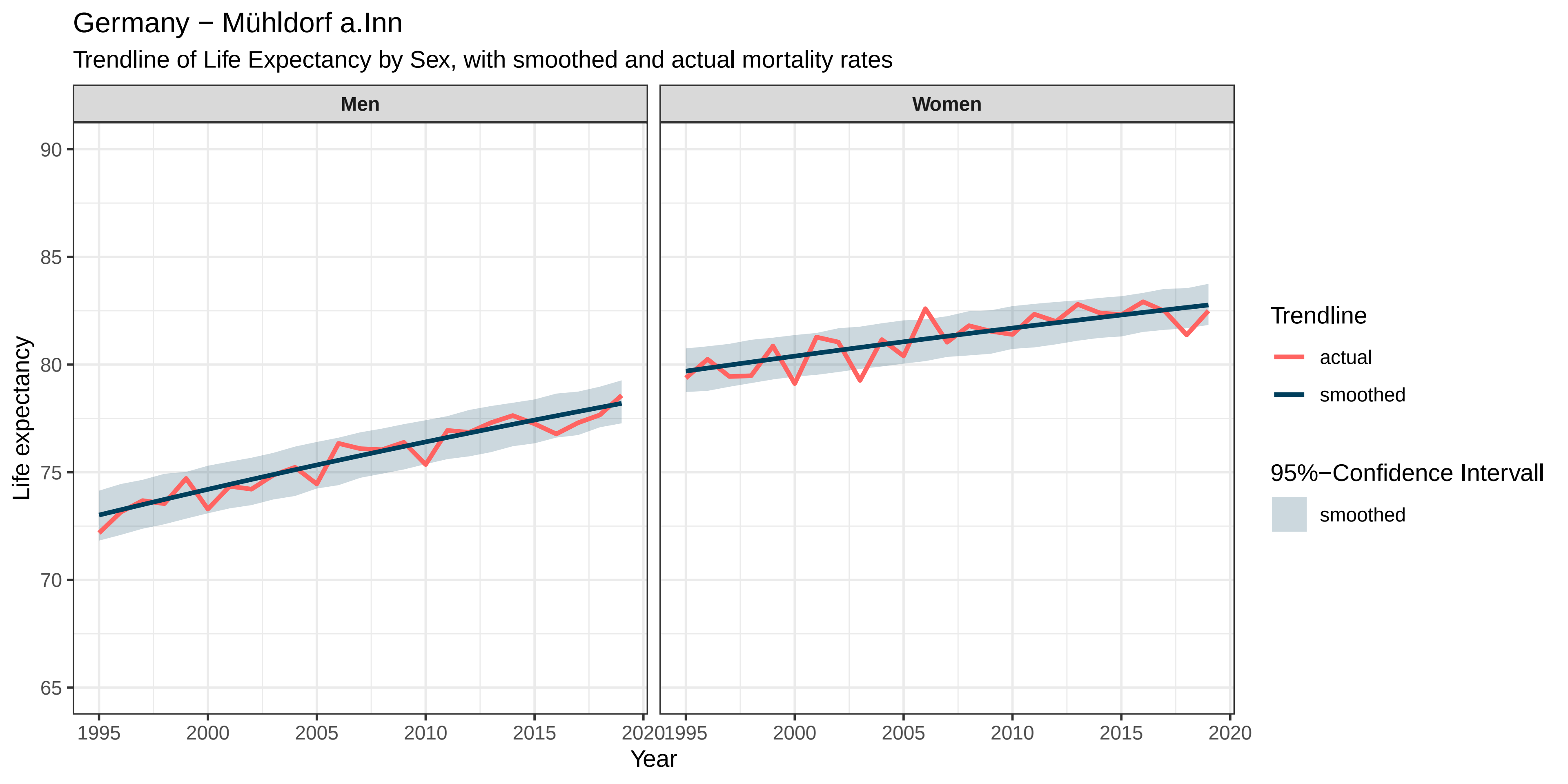

Germany − Mühldorf a.Inn
Trendline of Life Expectancy by Sex, with smoothed and actual mortality rates
Men
Women
Life expectancy
90
85
80
75
70
65
1995
2000
2005
2010
2015
2020
Year
Trendline
actual
smoothed
95%−Confidence Intervall
smoothed

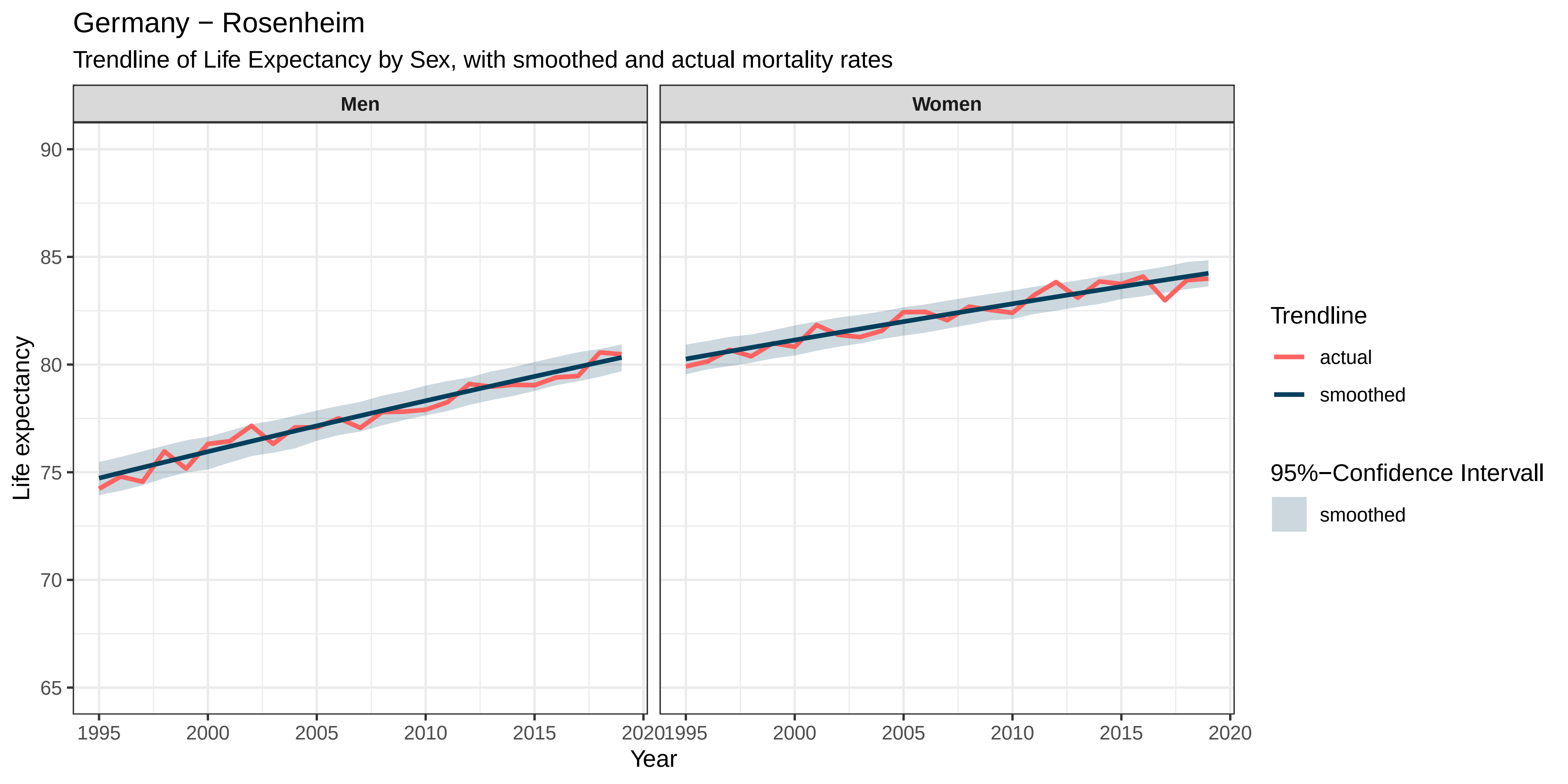
Germany − Rosenheim
Trendline of Life Expectancy by Sex, with smoothed and actual mortality rates
Men
Women
Life expectancy
90
85
80
75
70
65
1995
2000
2005
2010
2015
2020
Year
Trendline
actual
smoothed
95%−Confidence Intervall
smoothed

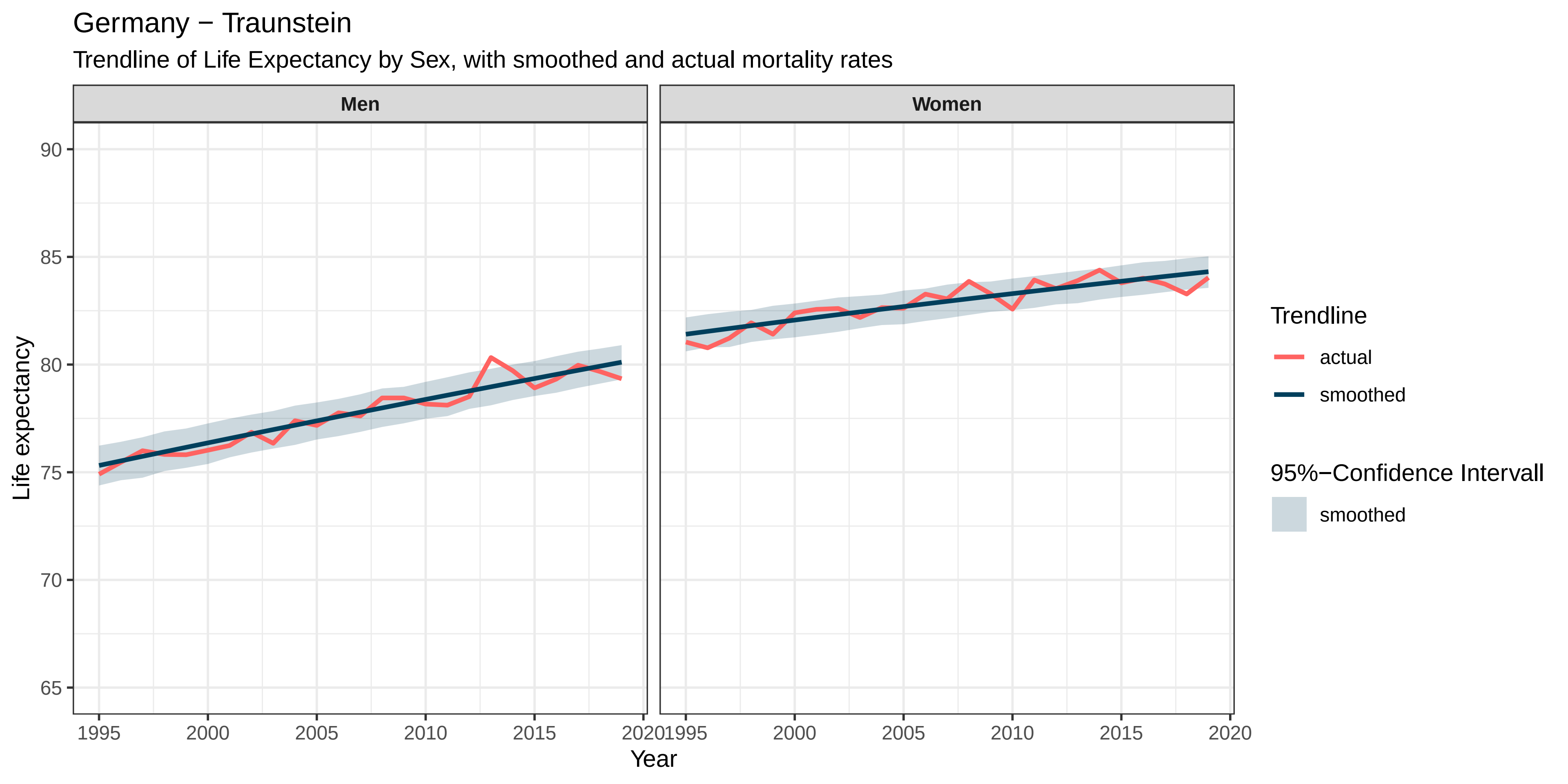

Germany − Traunstein
Trendline of Life Expectancy by Sex, with smoothed and actual mortality rates
Men
Women
Life expectancy
90
85
80
75
70
65
1995
2000
2005
2010
2015
2020
Year
Trendline
actual
smoothed
95%−Confidence Intervall
smoothed

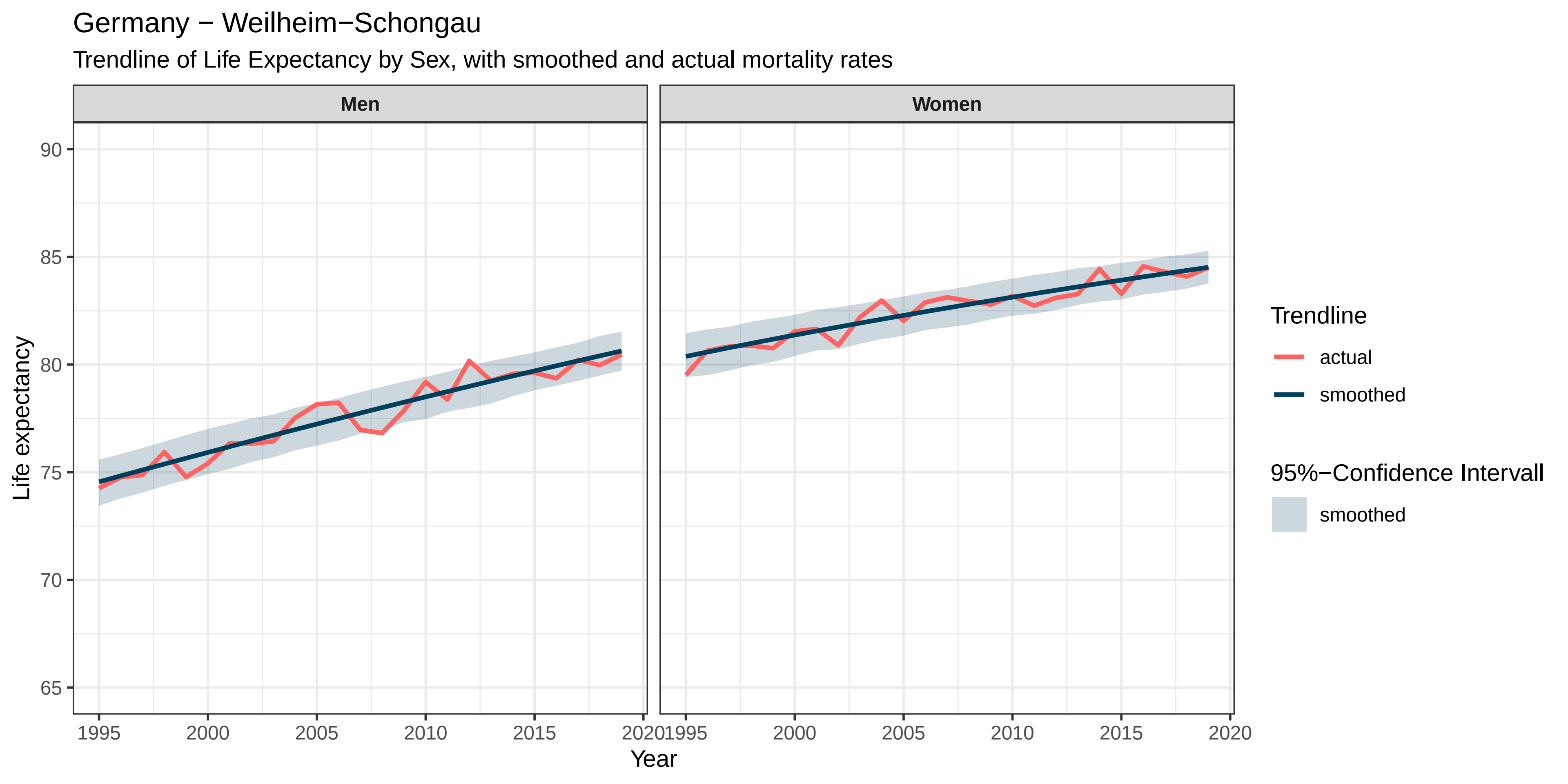

Germany − Weilheim−Schongau
Trendline of Life Expectancy by Sex, with smoothed and actual mortality rates
Men
Women
Life expectancy
90
85
80
75
70
65
1995
2000
2005
2010
2015
2020
Year
Trendline
actual
smoothed
95%−Confidence Intervall
smoothed

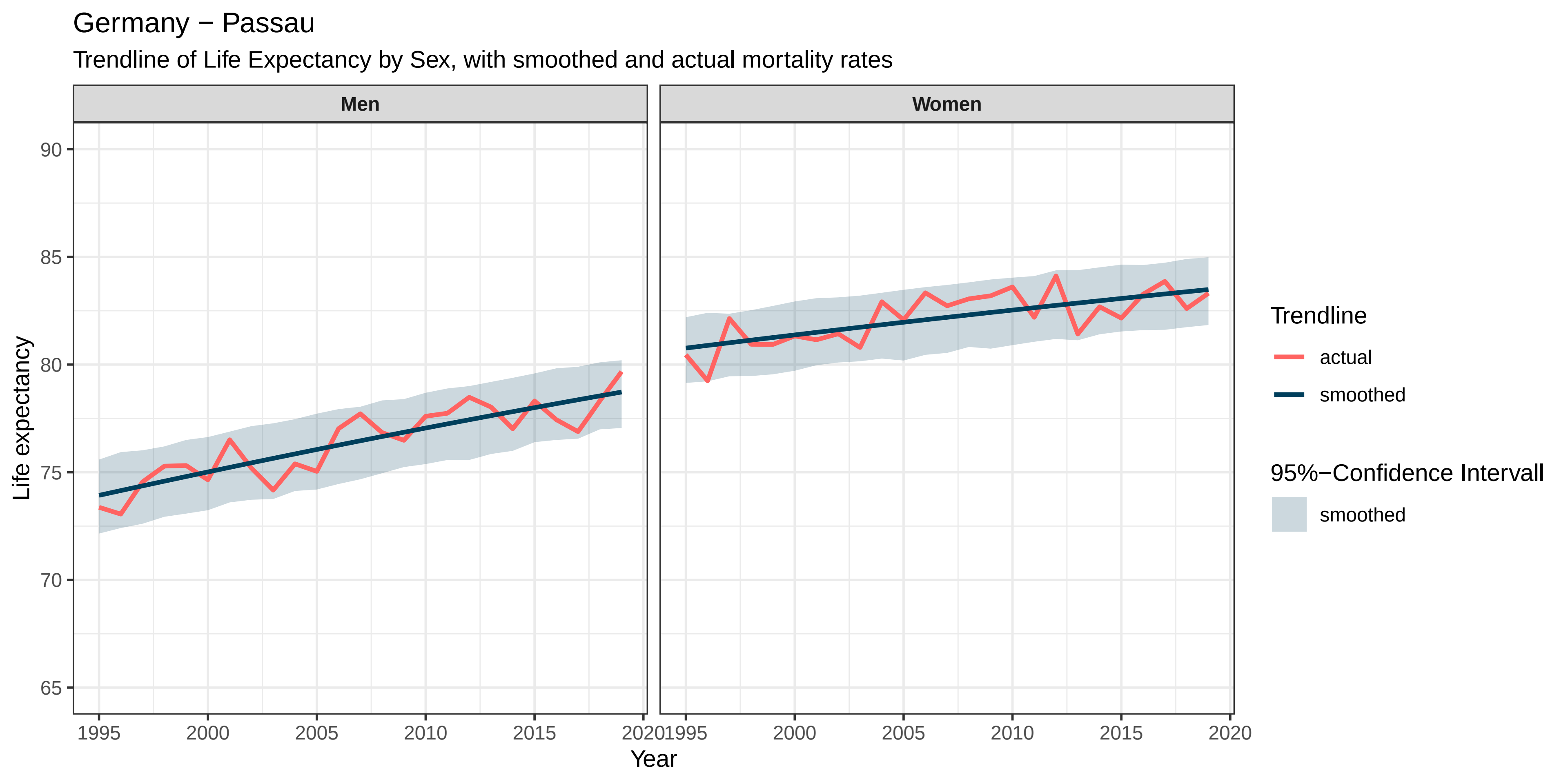

Germany − Passau
Trendline of Life Expectancy by Sex, with smoothed and actual mortality rates
Men
Women
Life expectancy
90
85
80
75
70
65
1995
2000
2005
2010
2015
2020
Year
Trendline
actual
smoothed
95%−Confidence Intervall
smoothed

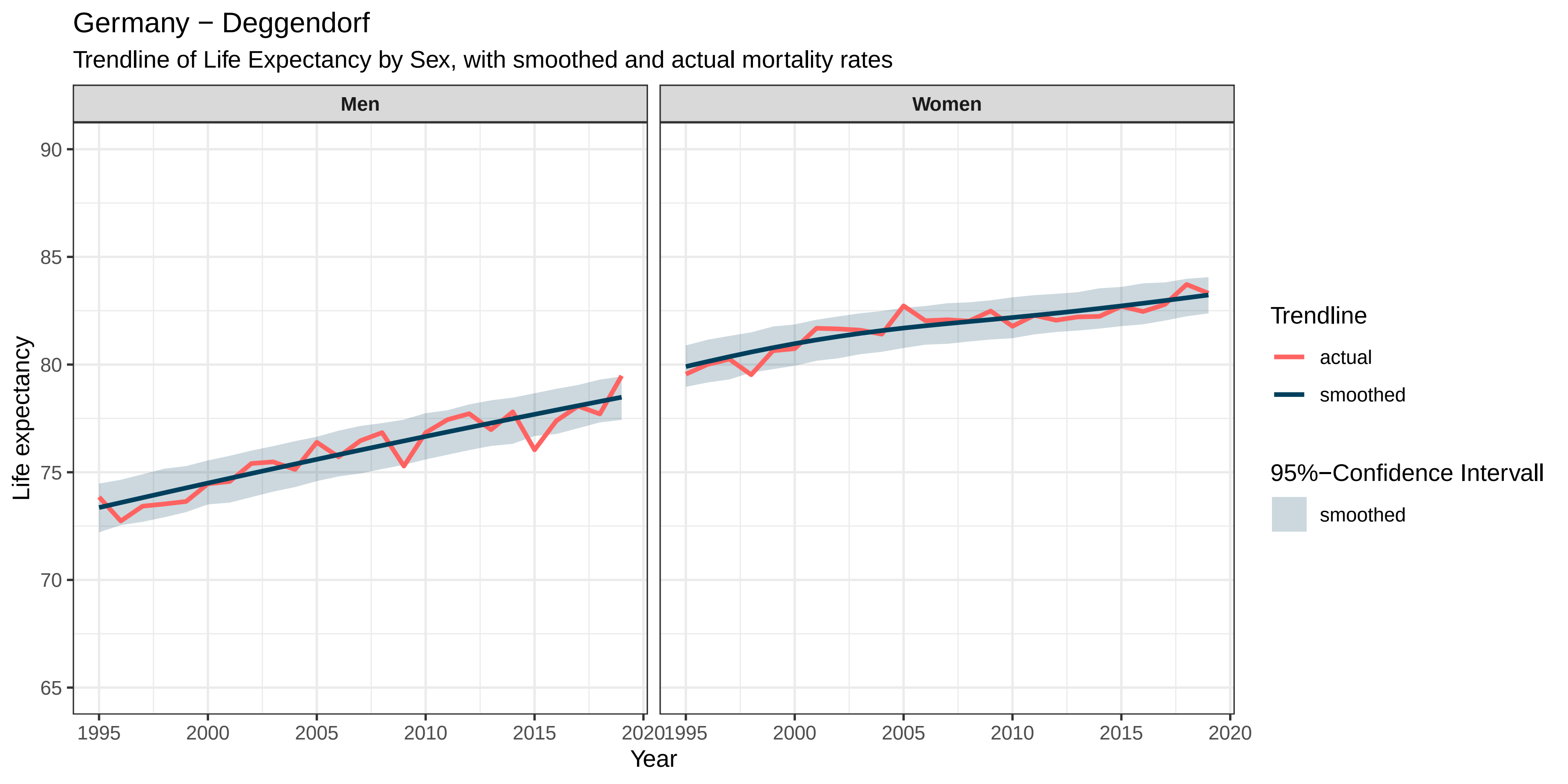
Germany − Deggendorf
Trendline of Life Expectancy by Sex, with smoothed and actual mortality rates
Men
Women
Life expectancy
90
85
80
75
70
65
1995
2000
2005
2010
2015
2020
Year
Trendline
actual
smoothed
95%−Confidence Intervall
smoothed

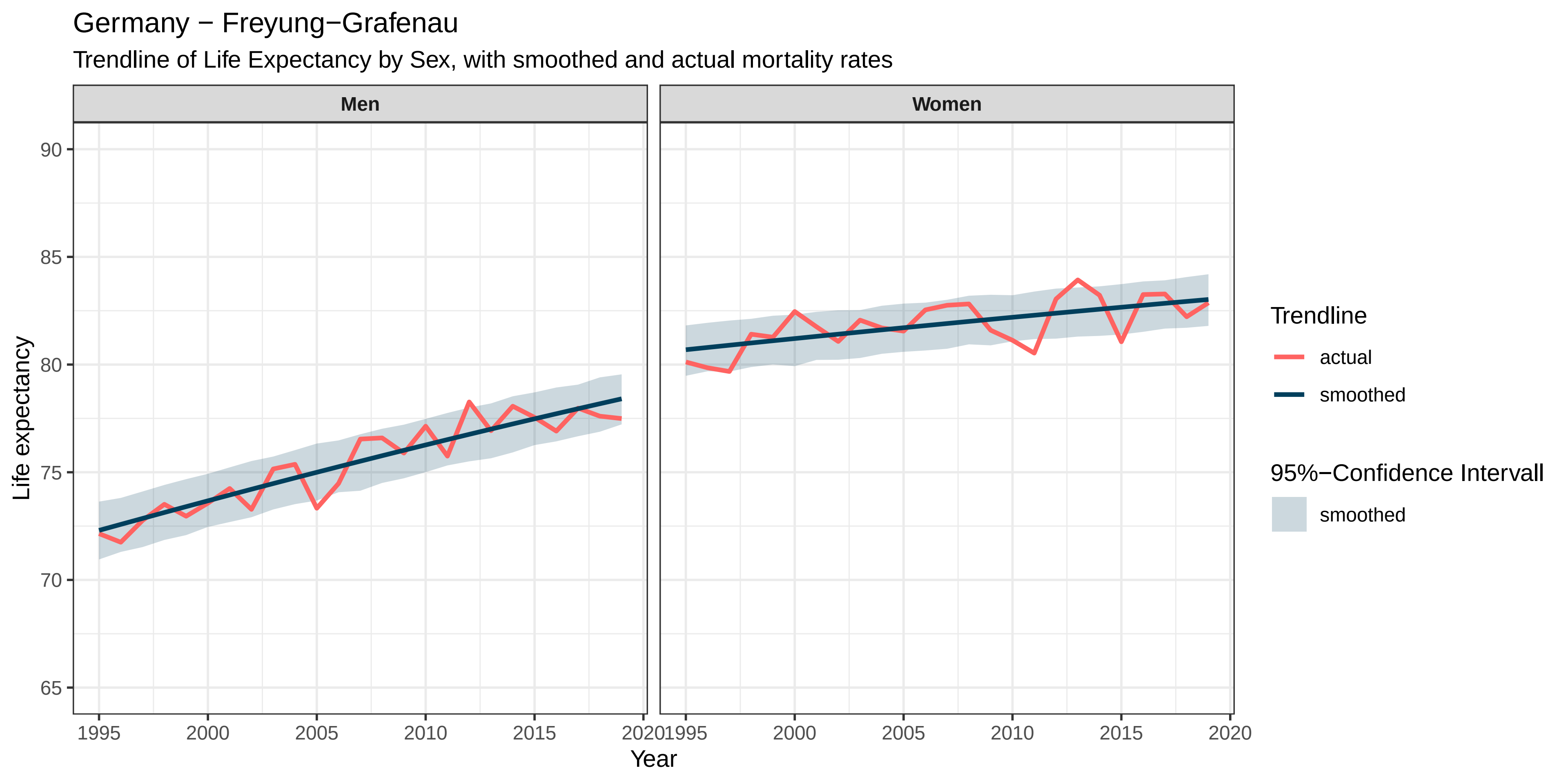

Germany − Freyung−Grafenau
Trendline of Life Expectancy by Sex, with smoothed and actual mortality rates
Men
Women
Life expectancy
Year
65
70
75
80
85
90
1995
2000
2005
2010
2015
2020
Trendline
actual
smoothed
95%−Confidence Intervall
smoothed

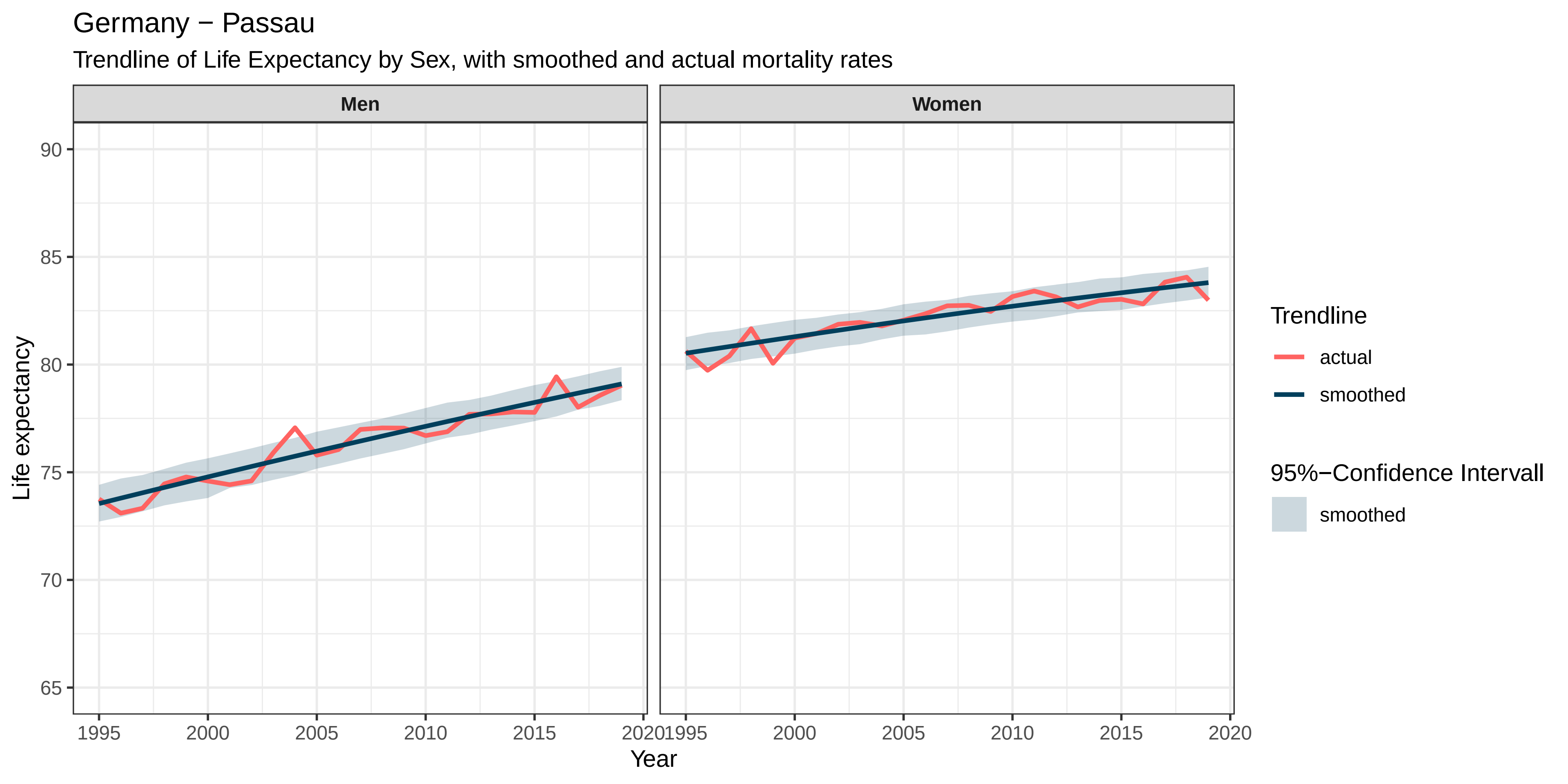
Germany − Passau
Trendline of Life Expectancy by Sex, with smoothed and actual mortality rates
Men
Women
Life expectancy
Year
90
85
80
75
70
65
1995
2000
2005
2010
2015
2020
Trendline
actual
smoothed
95%−Confidence Intervall
smoothed

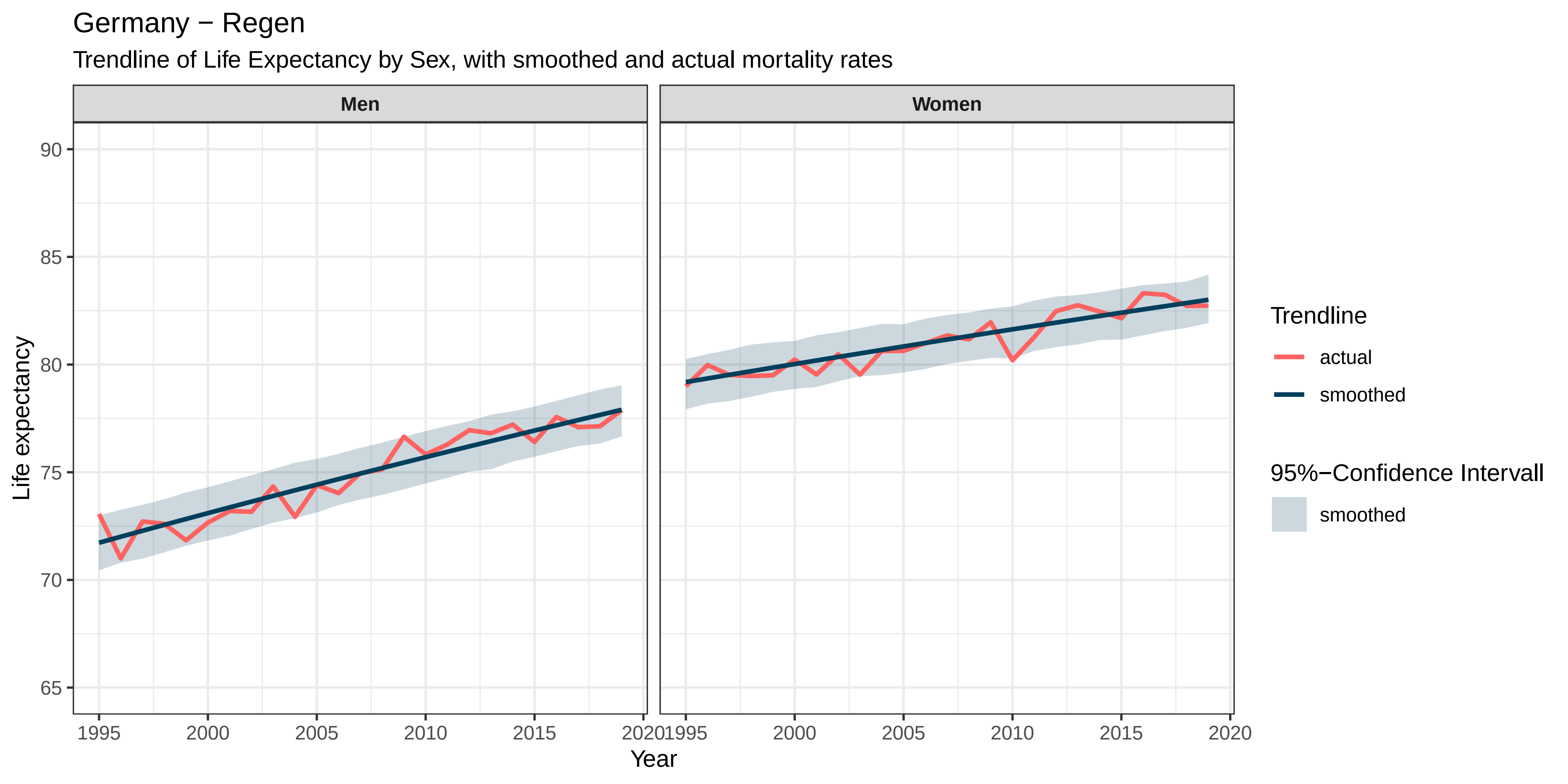

Germany − Regen
Trendline of Life Expectancy by Sex, with smoothed and actual mortality rates
Men
Women
90
85
80
75
70
65
Life expectancy
1995
2000
2005
2010
2015
2020
Year
Trendline
actual
smoothed
95%−Confidence Intervall
smoothed

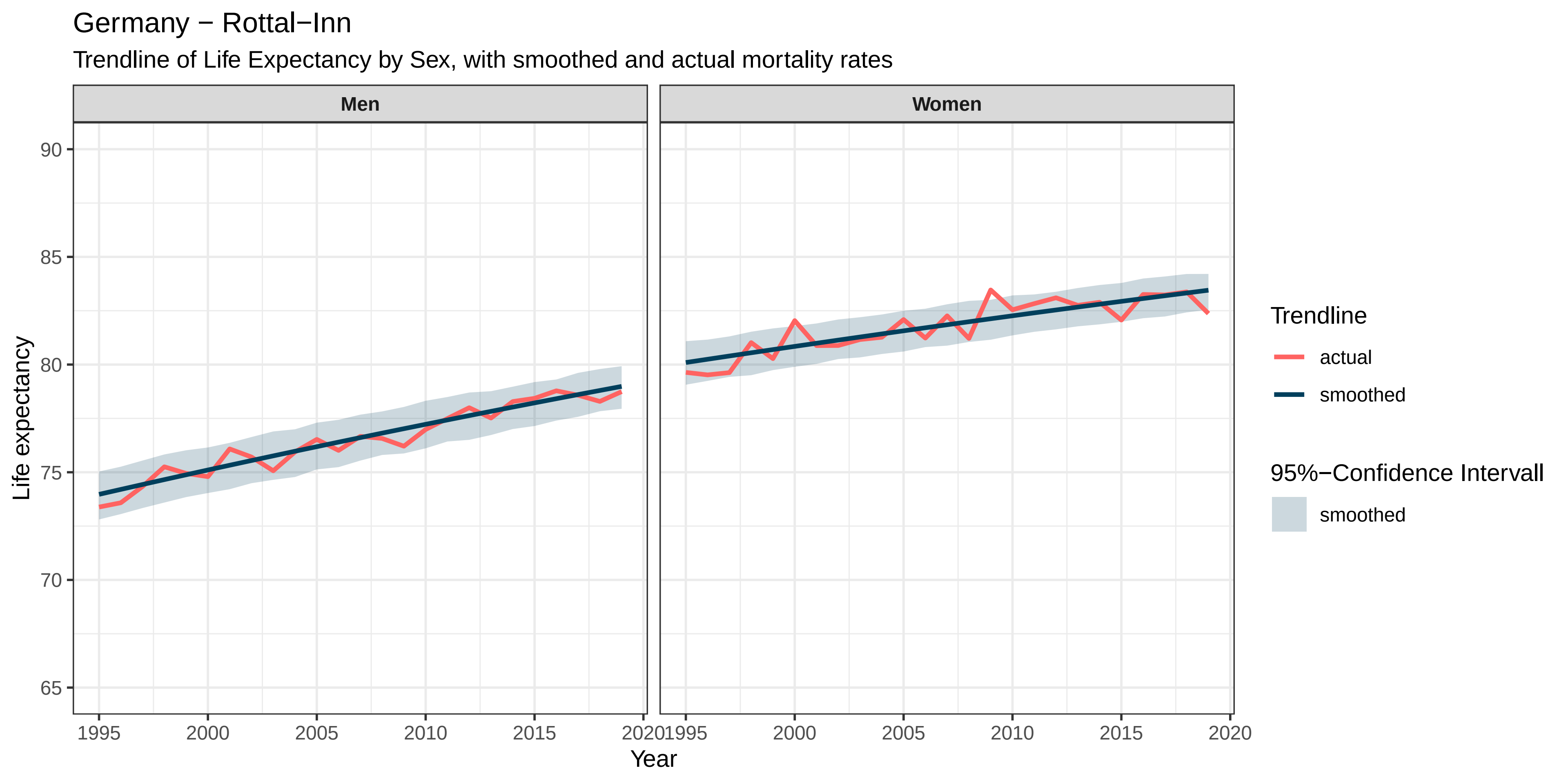
Germany − Rottal−Inn
Trendline of Life Expectancy by Sex, with smoothed and actual mortality rates
Men
Women
Life expectancy
90
85
80
75
70
65
1995
2000
2005
2010
2015
2020
Year
Trendline
actual
smoothed
95%−Confidence Intervall
smoothed

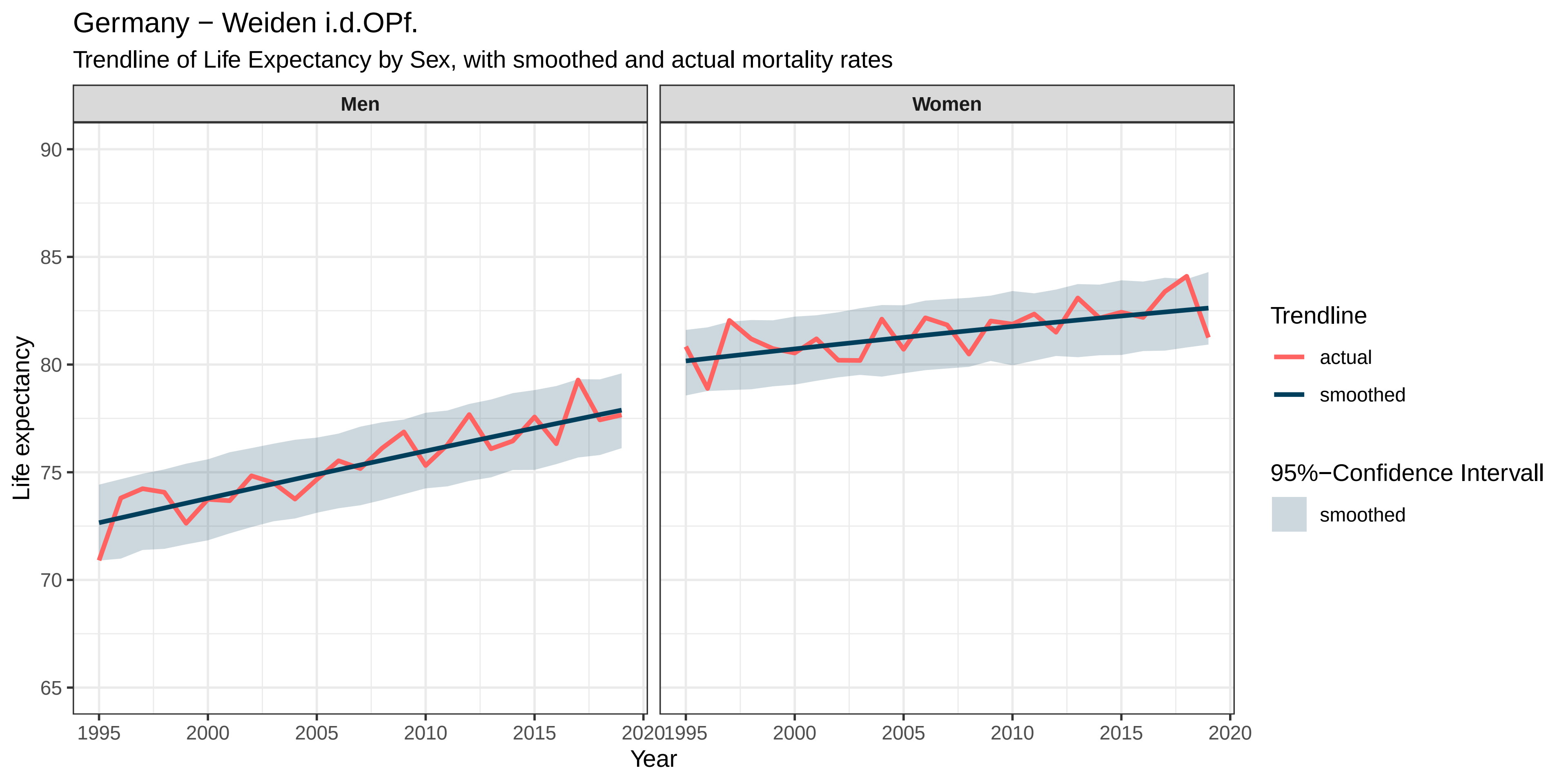

Germany − Weiden i.d.OPf.
Trendline of Life Expectancy by Sex, with smoothed and actual mortality rates
Men
Women
90
85
80
75
70
65
Life expectancy
1995
2000
2005
2010
2015
2020
Year
Trendline
actual
smoothed
95%−Confidence Intervall
smoothed

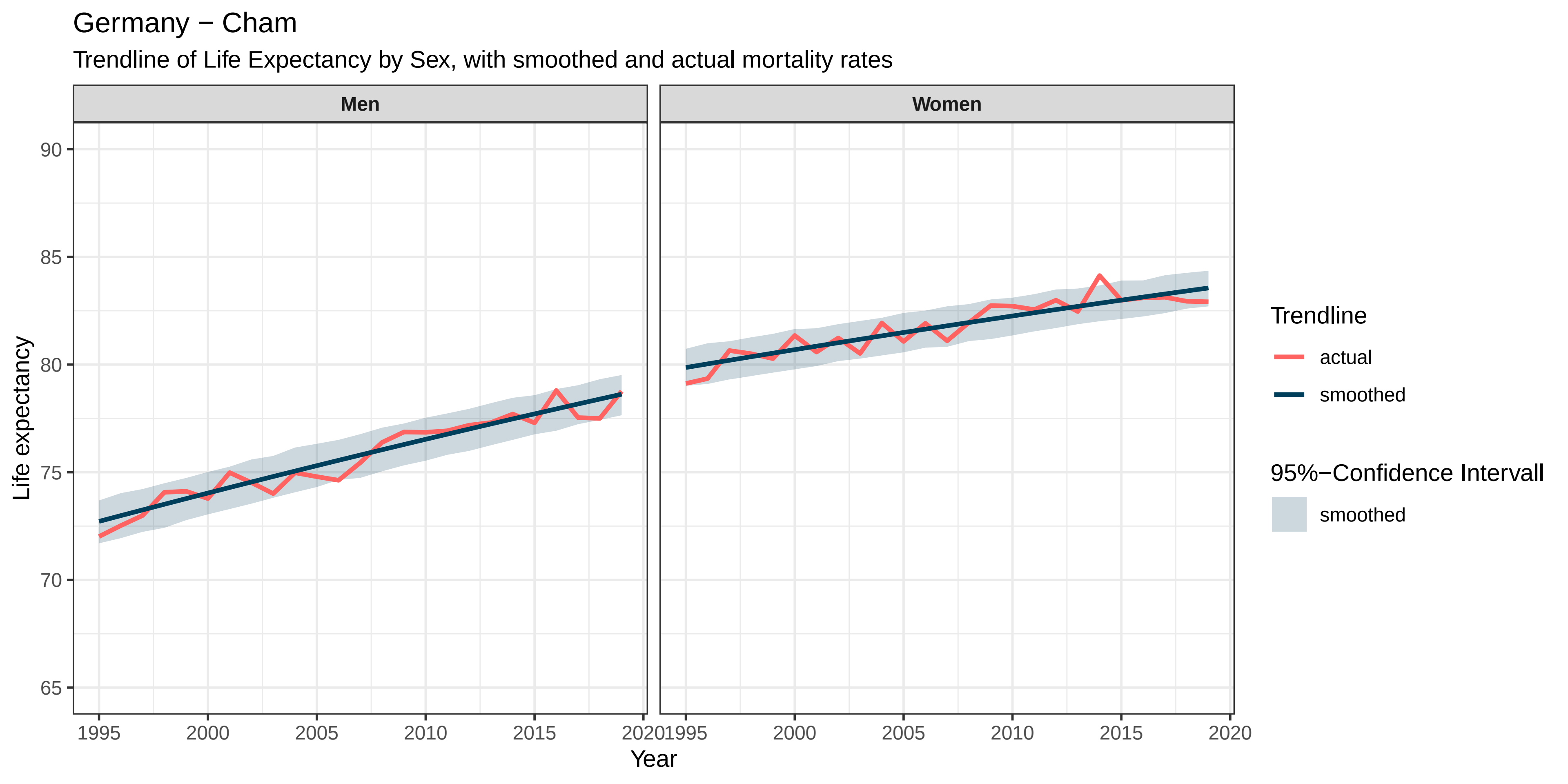
Germany − Cham
Trendline of Life Expectancy by Sex, with smoothed and actual mortality rates
Men
Women
Life expectancy
Year
90
85
80
75
70
65
1995
2000
2005
2010
2015
2020
Trendline
actual
smoothed
95%−Confidence Intervall
smoothed

Germany − Neustadt a.d.Waldnaab

Trendline of Life Expectancy by Sex, with smoothed and actual mortality rates

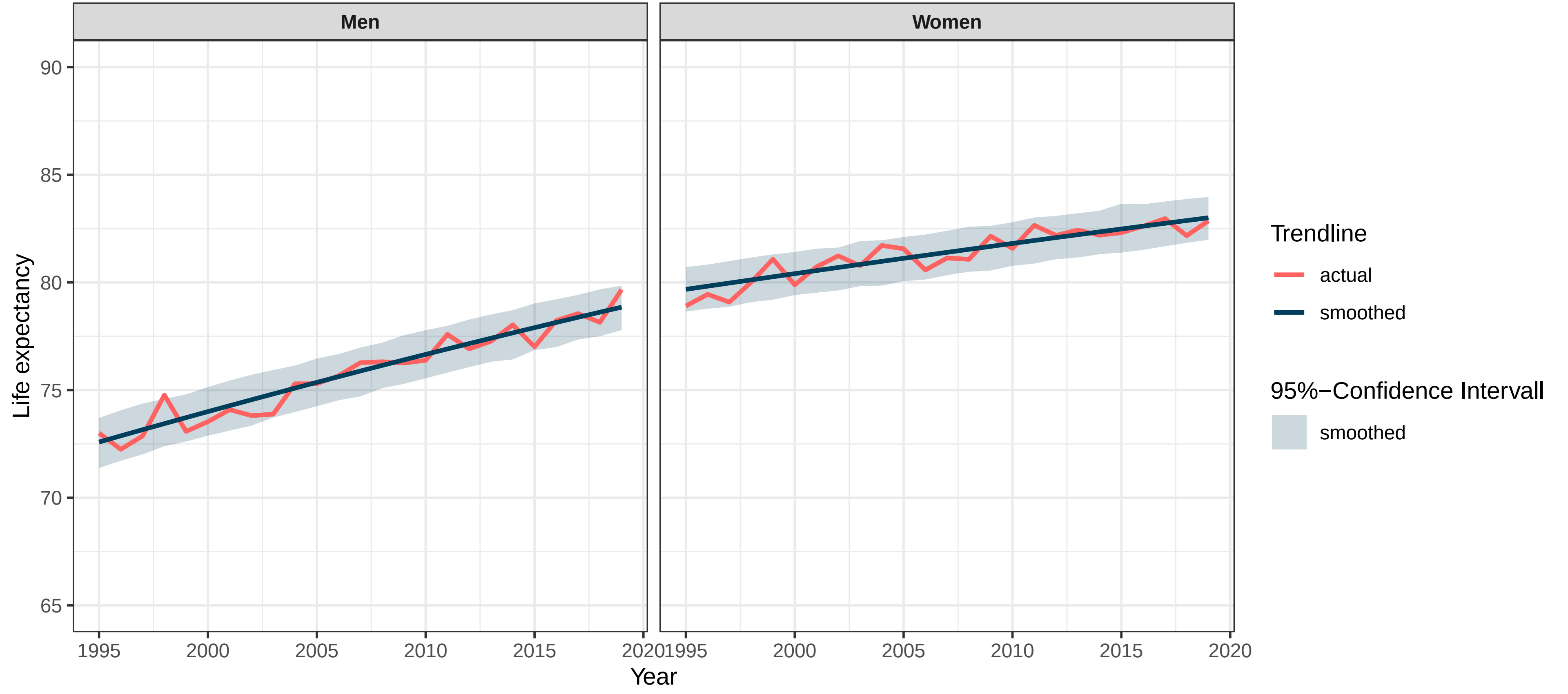

Germany − Schwandorf

Trendline of Life Expectancy by Sex, with smoothed and actual mortality rates

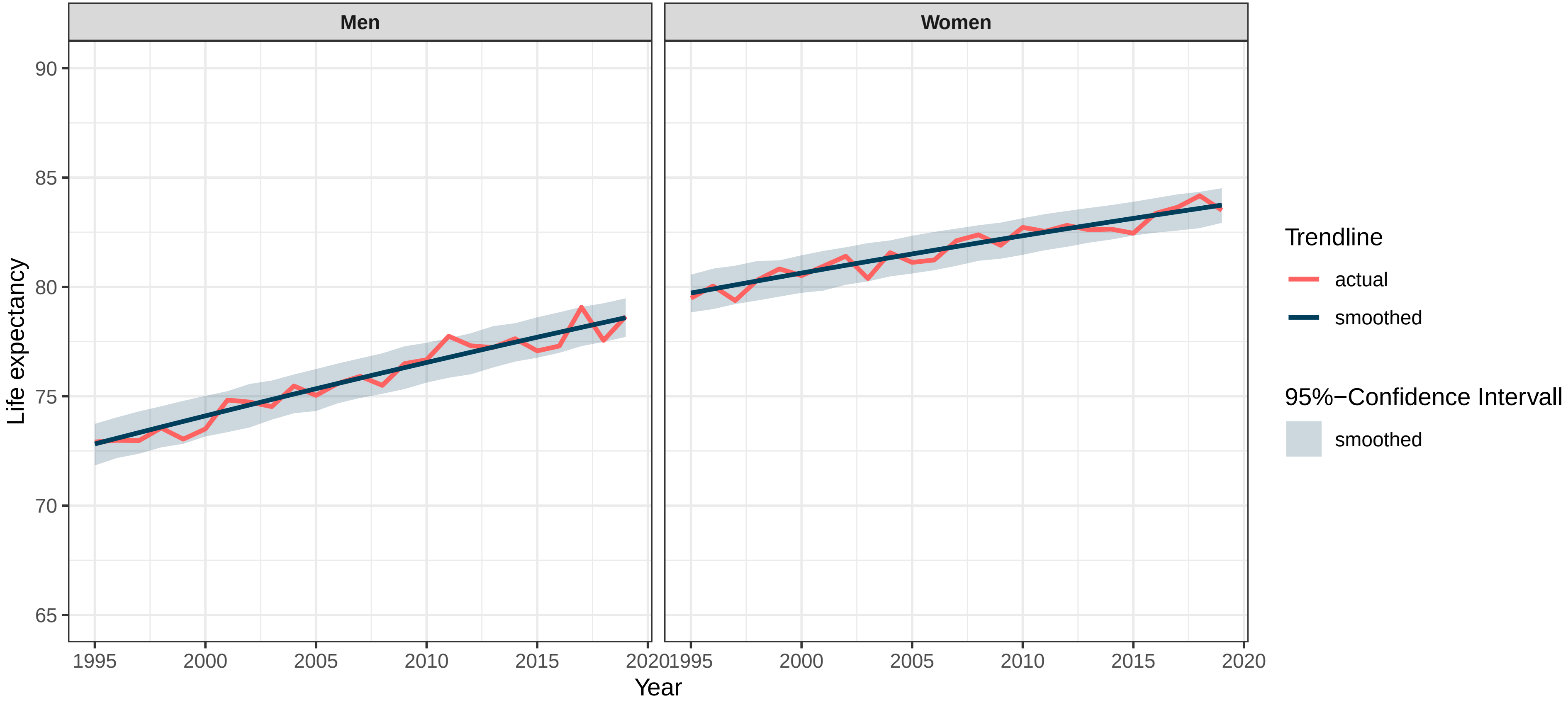

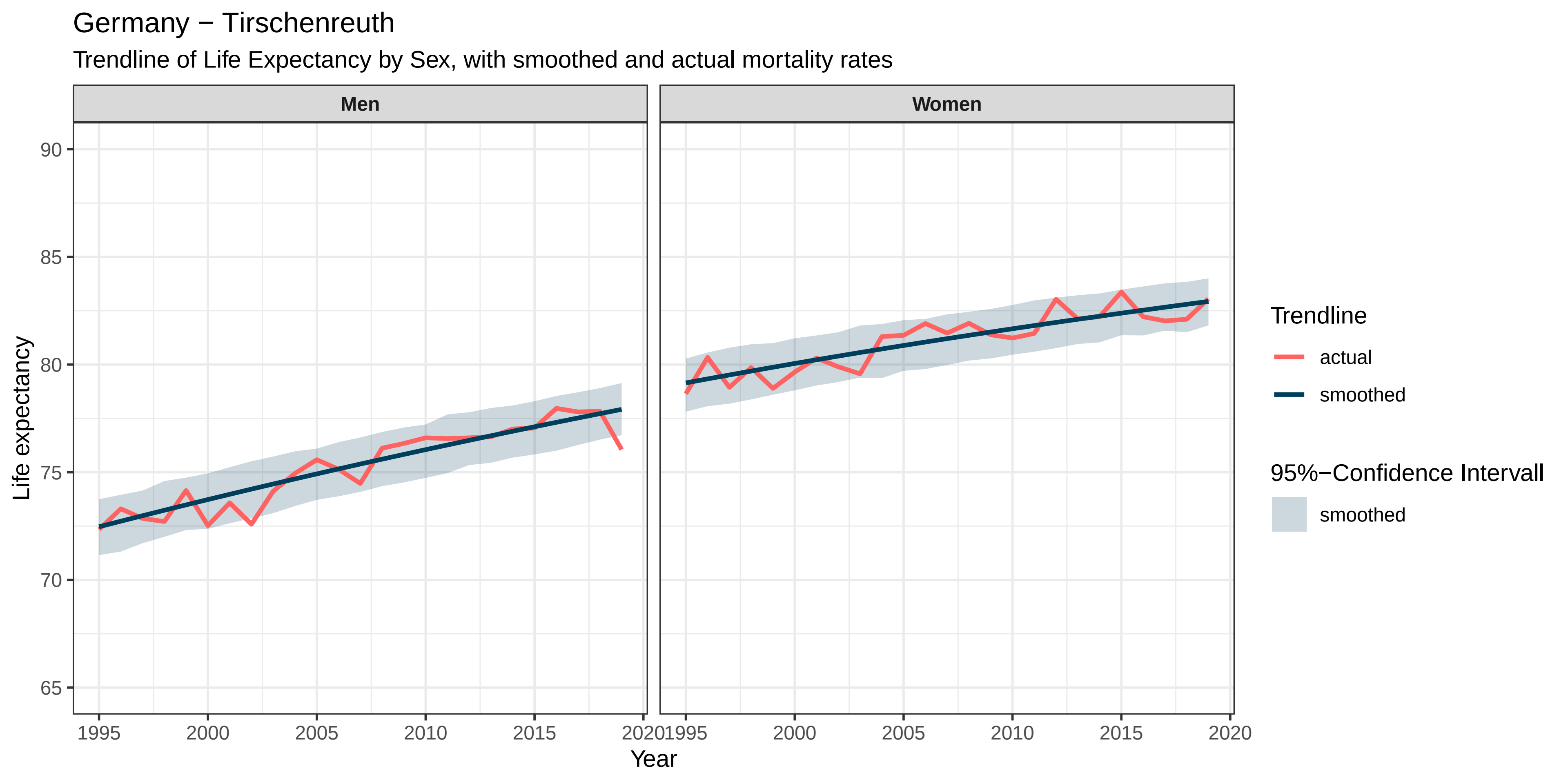

Germany − Tirschenreuth
Trendline of Life Expectancy by Sex, with smoothed and actual mortality rates
Men
Women
90
85
80
75
70
65
Life expectancy
1995
2000
2005
2010
2015
2020
Year
Trendline
actual
smoothed
95%−Confidence Intervall
smoothed

Germany − Hof

Trendline of Life Expectancy by Sex, with smoothed and actual mortality rates

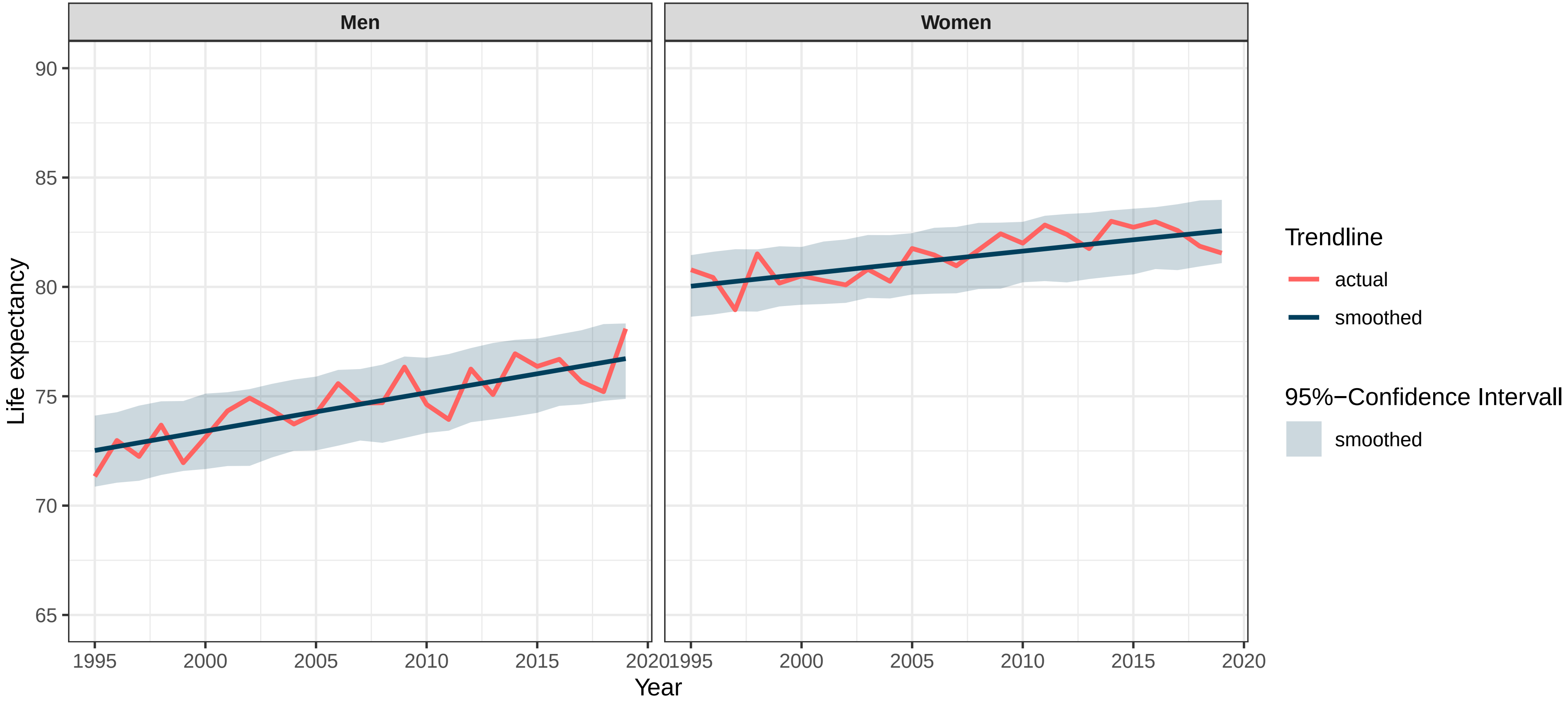

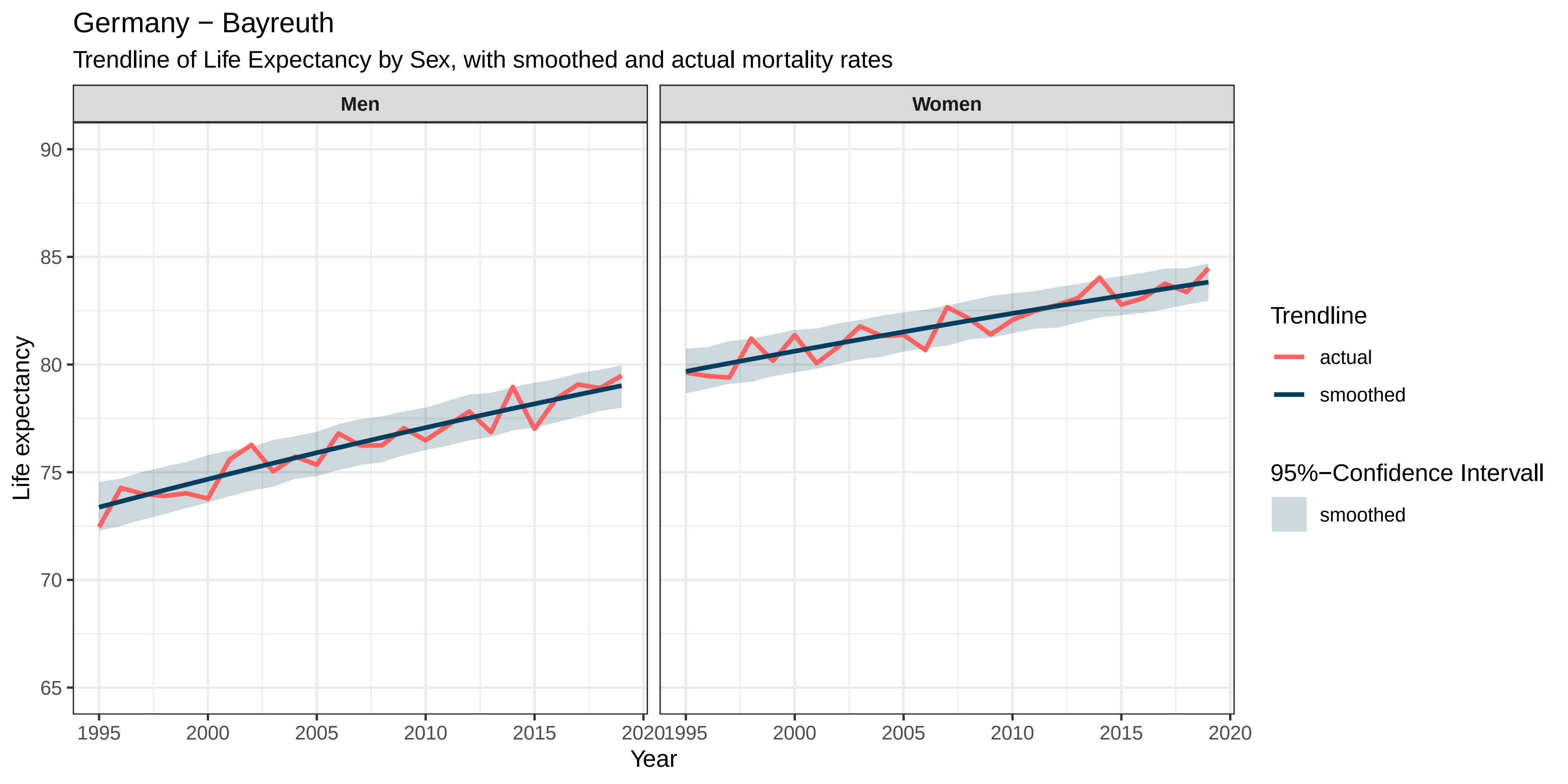

Germany − Bayreuth
Trendline of Life Expectancy by Sex, with smoothed and actual mortality rates
Men
Women
Life expectancy
Year
90
85
80
75
70
65
1995
2000
2005
2010
2015
2020
Trendline
actual
smoothed
95%−Confidence Intervall
smoothed

Germany − Hof

Trendline of Life Expectancy by Sex, with smoothed and actual mortality rates

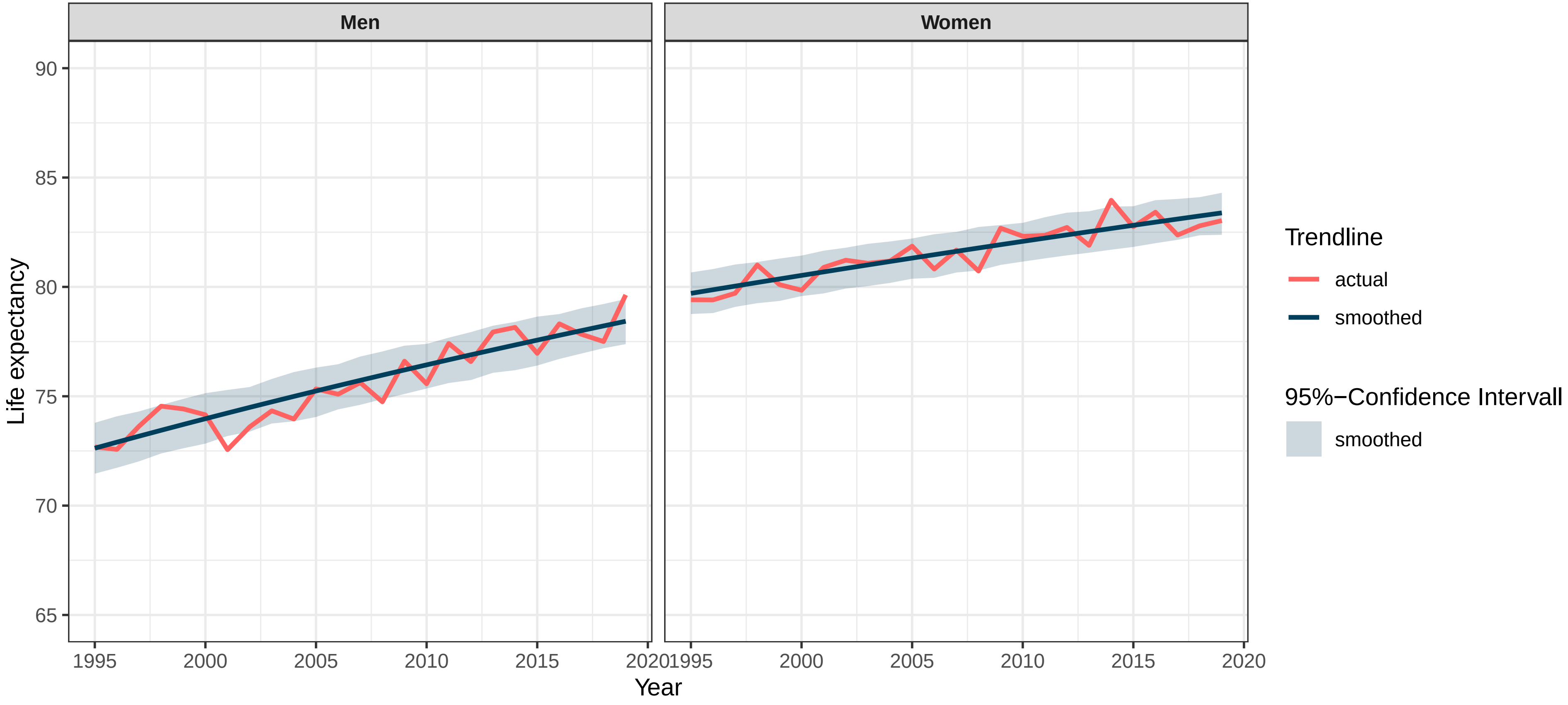

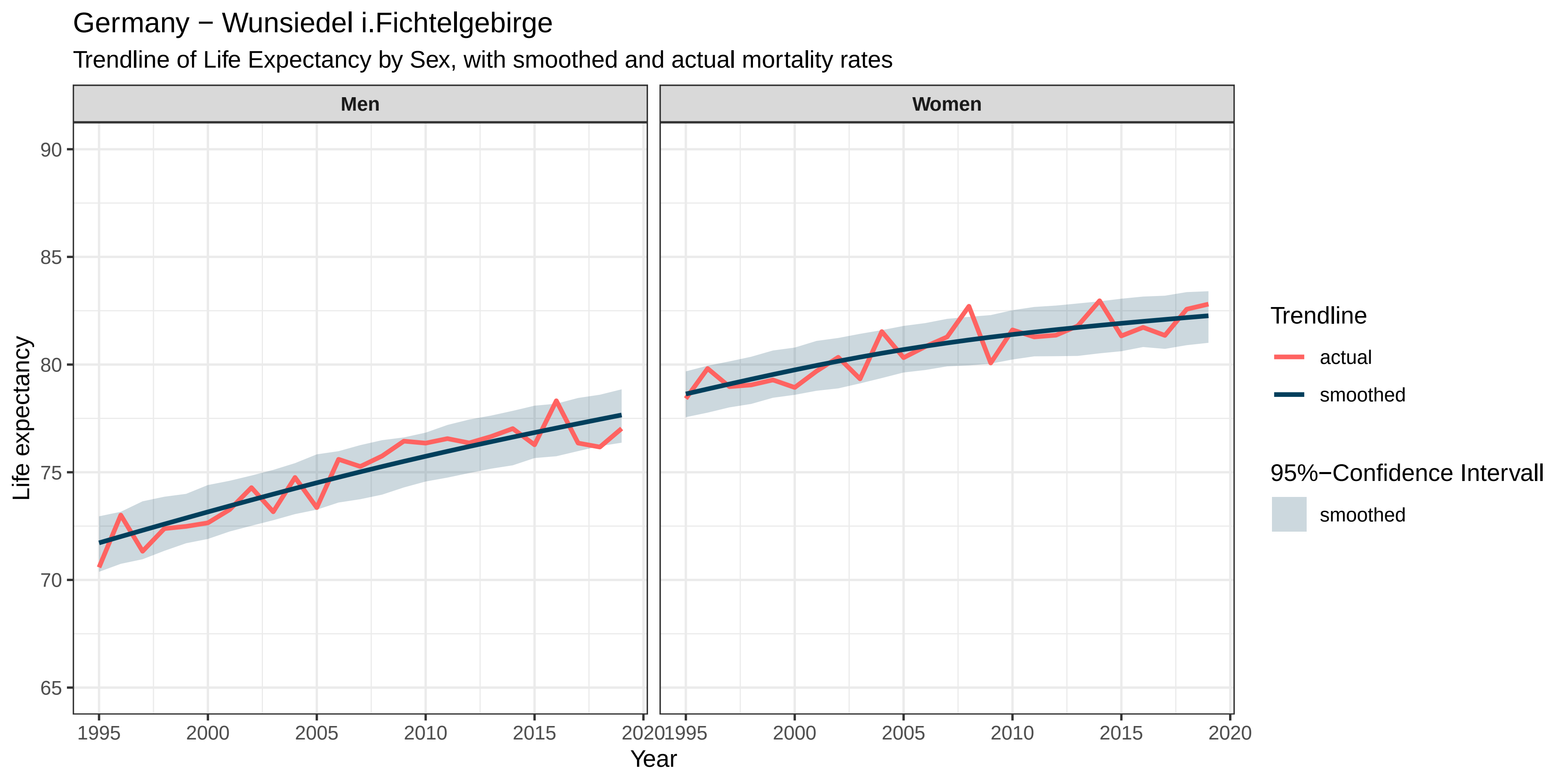

Germany − Wunsiedel i.Fichtelgebirge
Trendline of Life Expectancy by Sex, with smoothed and actual mortality rates
Men
Women
Life expectancy
90
85
80
75
70
65
1995
2000
2005
2010
2015
2020
Year
Trendline
actual
smoothed
95%−Confidence Intervall
smoothed

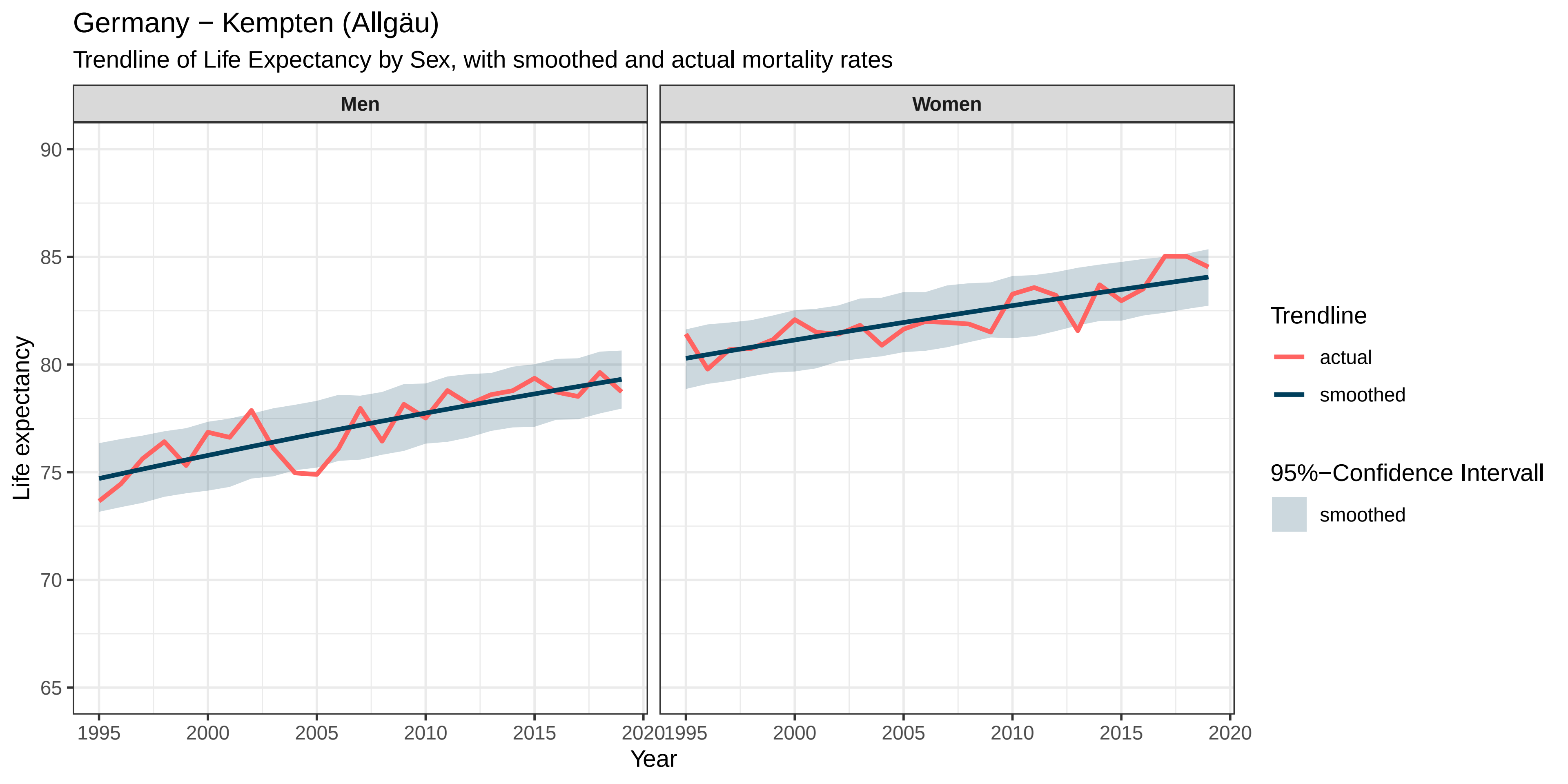
Germany − Kempten (Allgäu)
Trendline of Life Expectancy by Sex, with smoothed and actual mortality rates
Men
Women
Life expectancy
90
85
80
75
70
65
1995
2000
2005
2010
2015
2020
Year
Trendline
actual
smoothed
95%−Confidence Intervall
smoothed

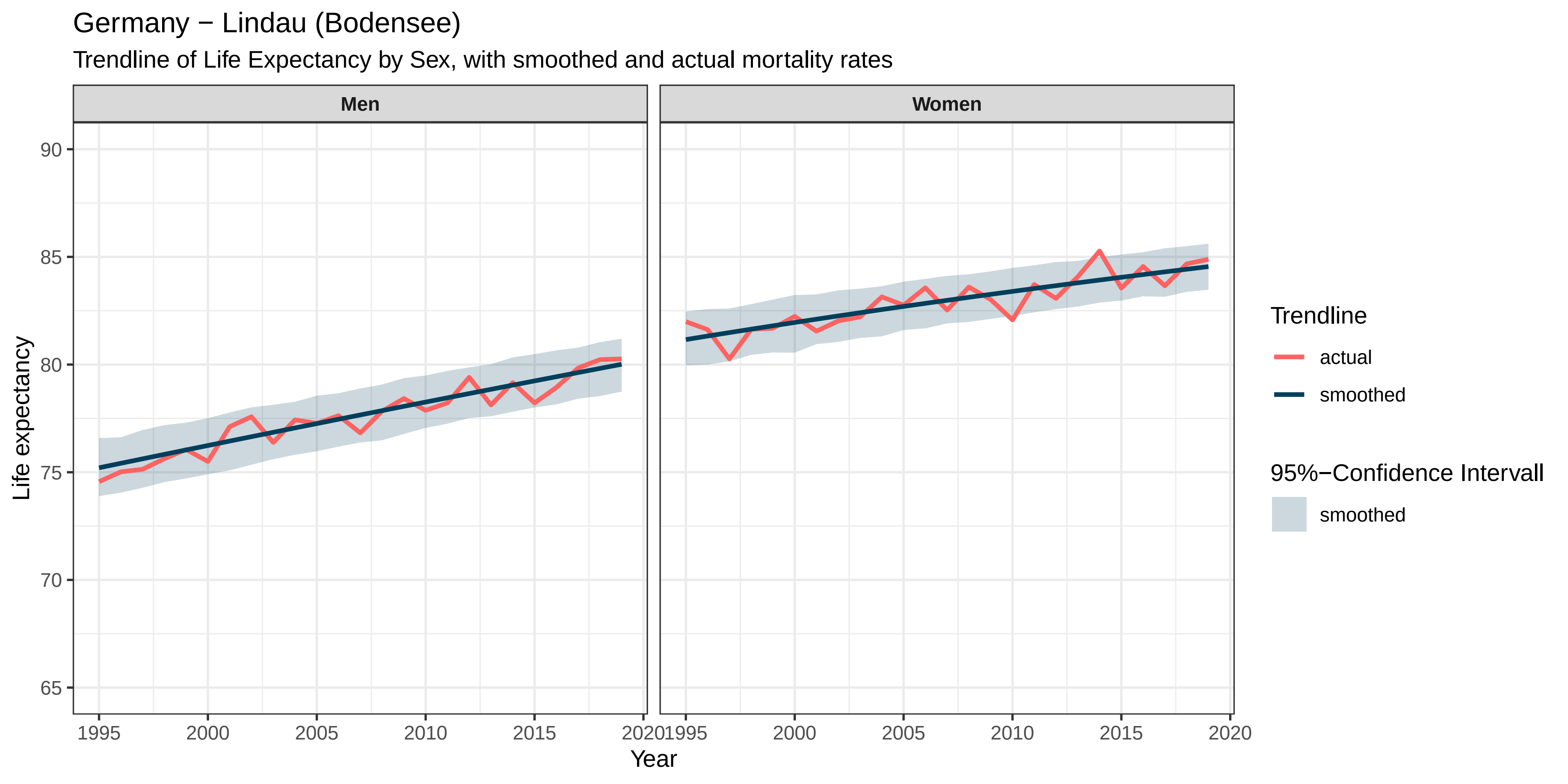

Germany − Lindau (Bodensee)
Trendline of Life Expectancy by Sex, with smoothed and actual mortality rates
Men
Women
90
85
80
75
70
65
Life expectancy
1995
2000
2005
2010
2015
2020
Year
Trendline
actual
smoothed
95%−Confidence Intervall
smoothed

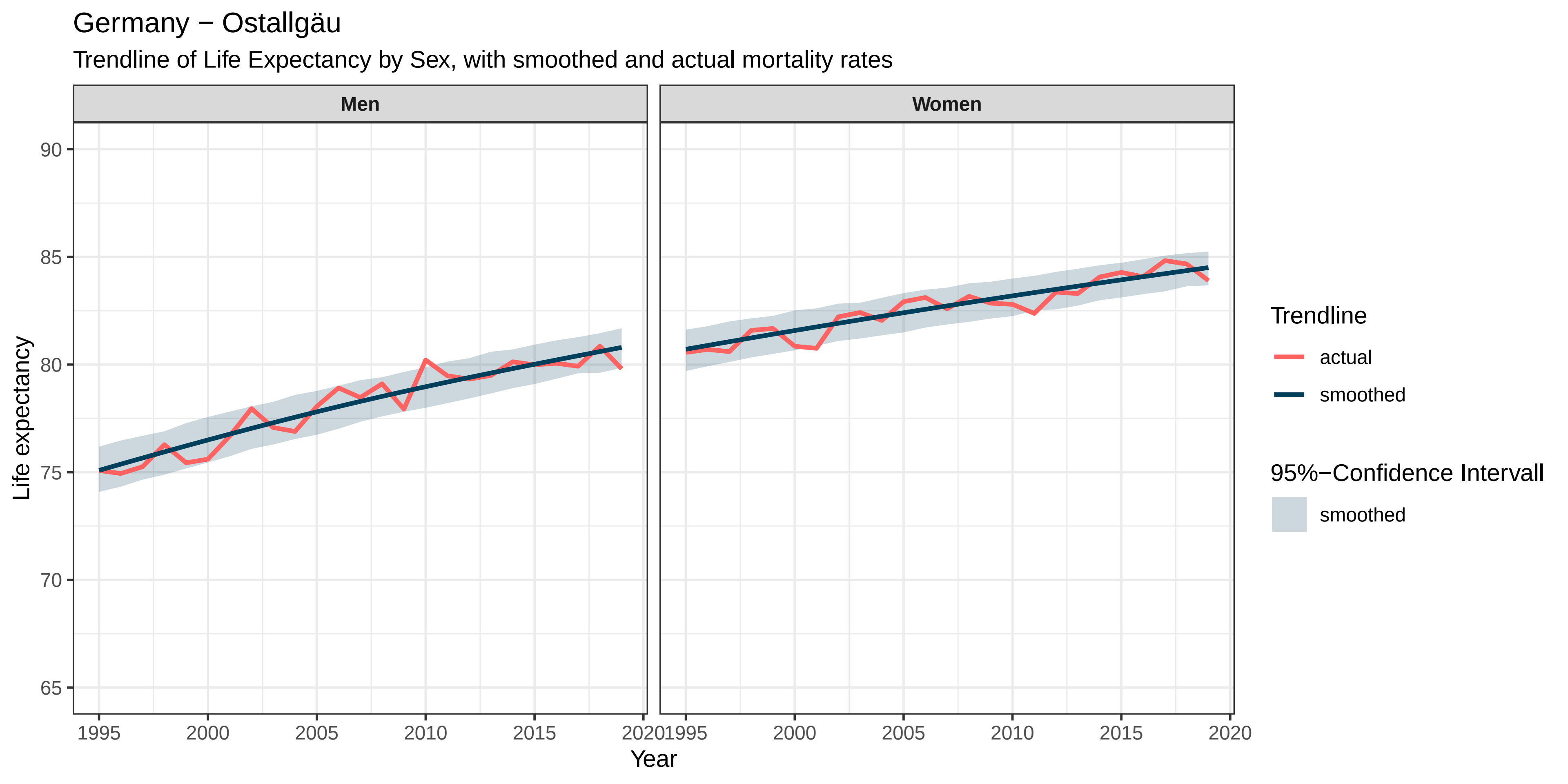

Germany − Ostallgäu
Trendline of Life Expectancy by Sex, with smoothed and actual mortality rates
Men
Women
Life expectancy
90
85
80
75
70
65
1995
2000
2005
2010
2015
2020
Year
Trendline
actual
smoothed
95%−Confidence Intervall
smoothed

Germany − Oberallgäu

Trendline of Life Expectancy by Sex, with smoothed and actual mortality rates

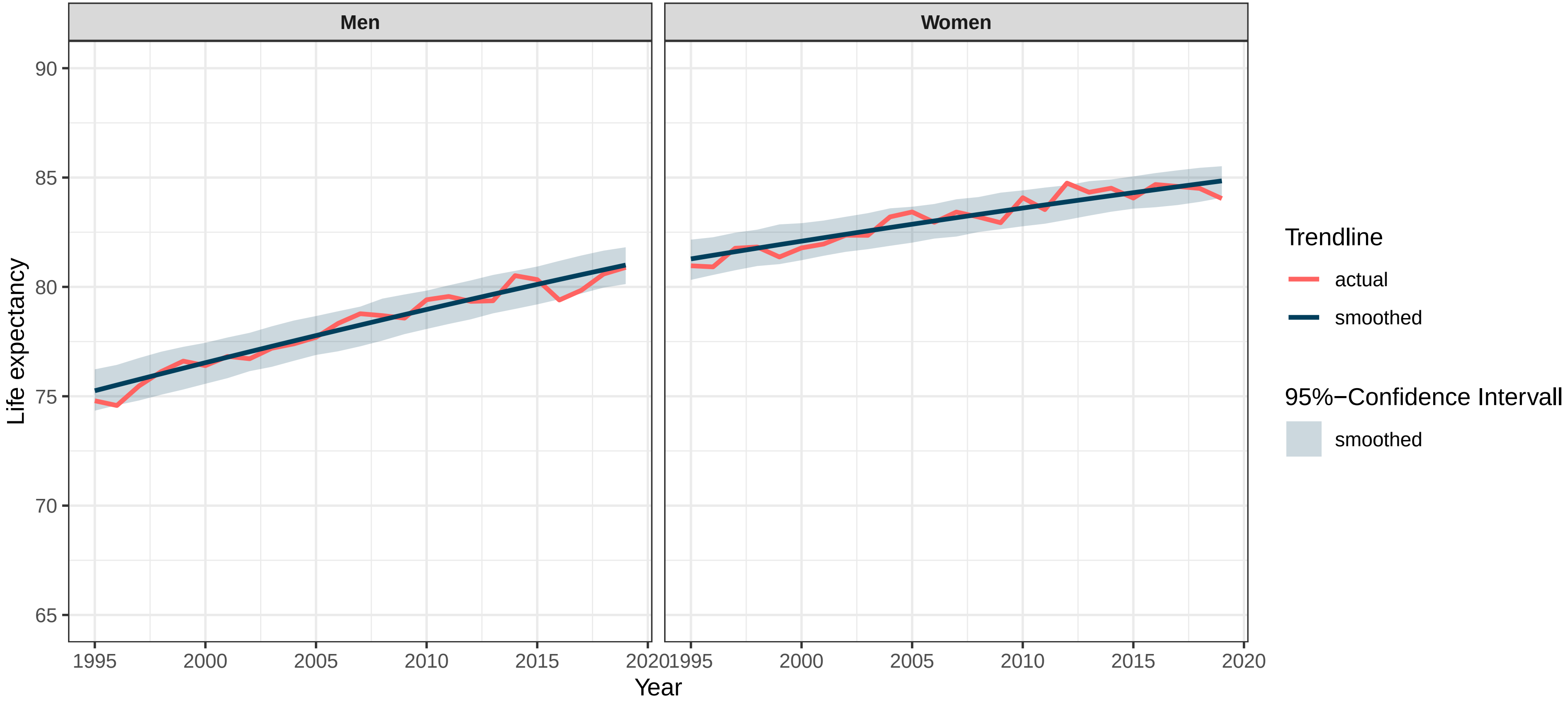

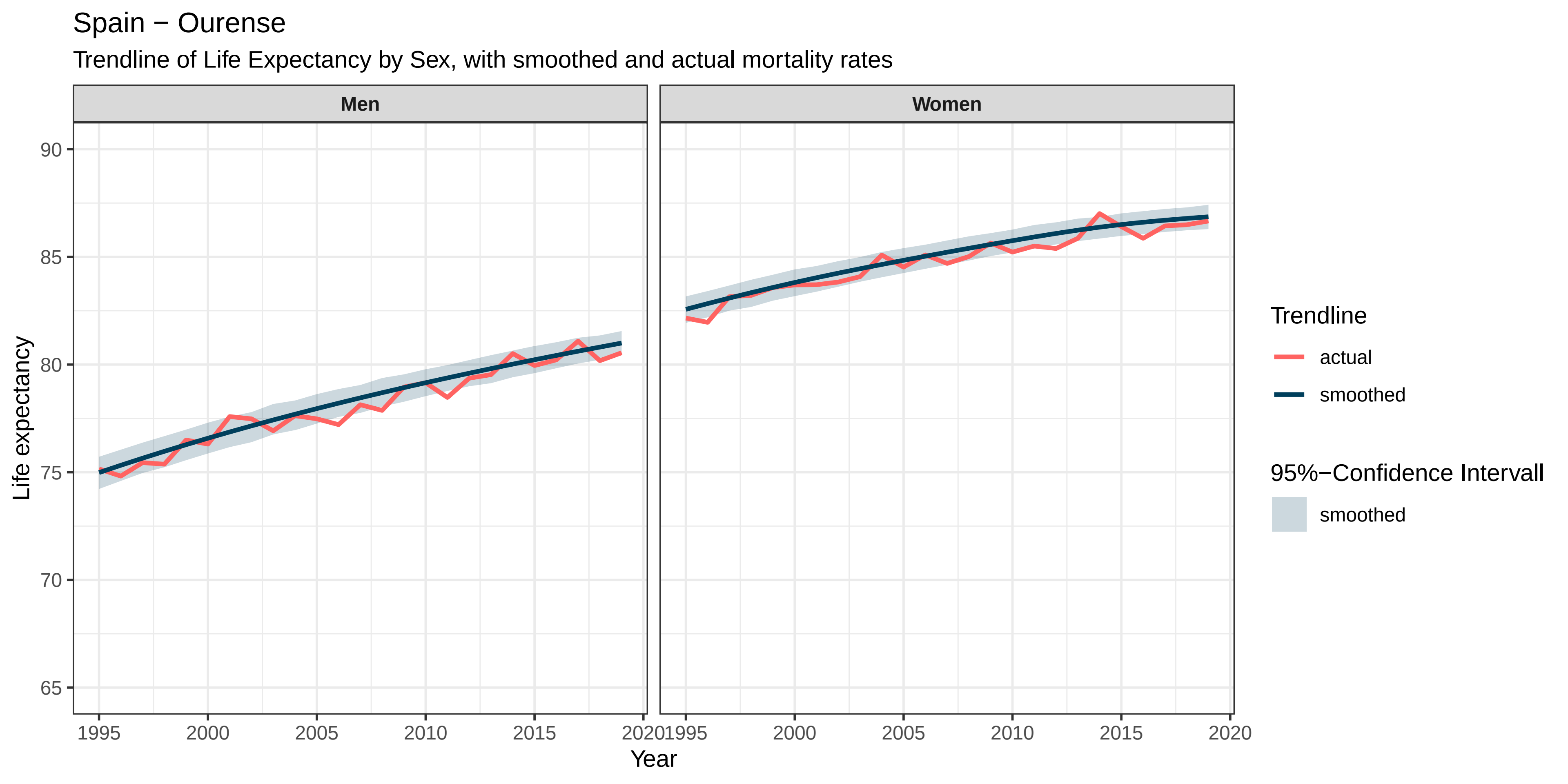

Spain − Ourense
Trendline of Life Expectancy by Sex, with smoothed and actual mortality rates
Men
Women
Life expectancy
90
85
80
75
70
65
1995
2000
2005
2010
2015
2020
Year
Trendline
actual
smoothed
95%−Confidence Intervall
smoothed

Spain − Pontevedra

Trendline of Life Expectancy by Sex, with smoothed and actual mortality rates

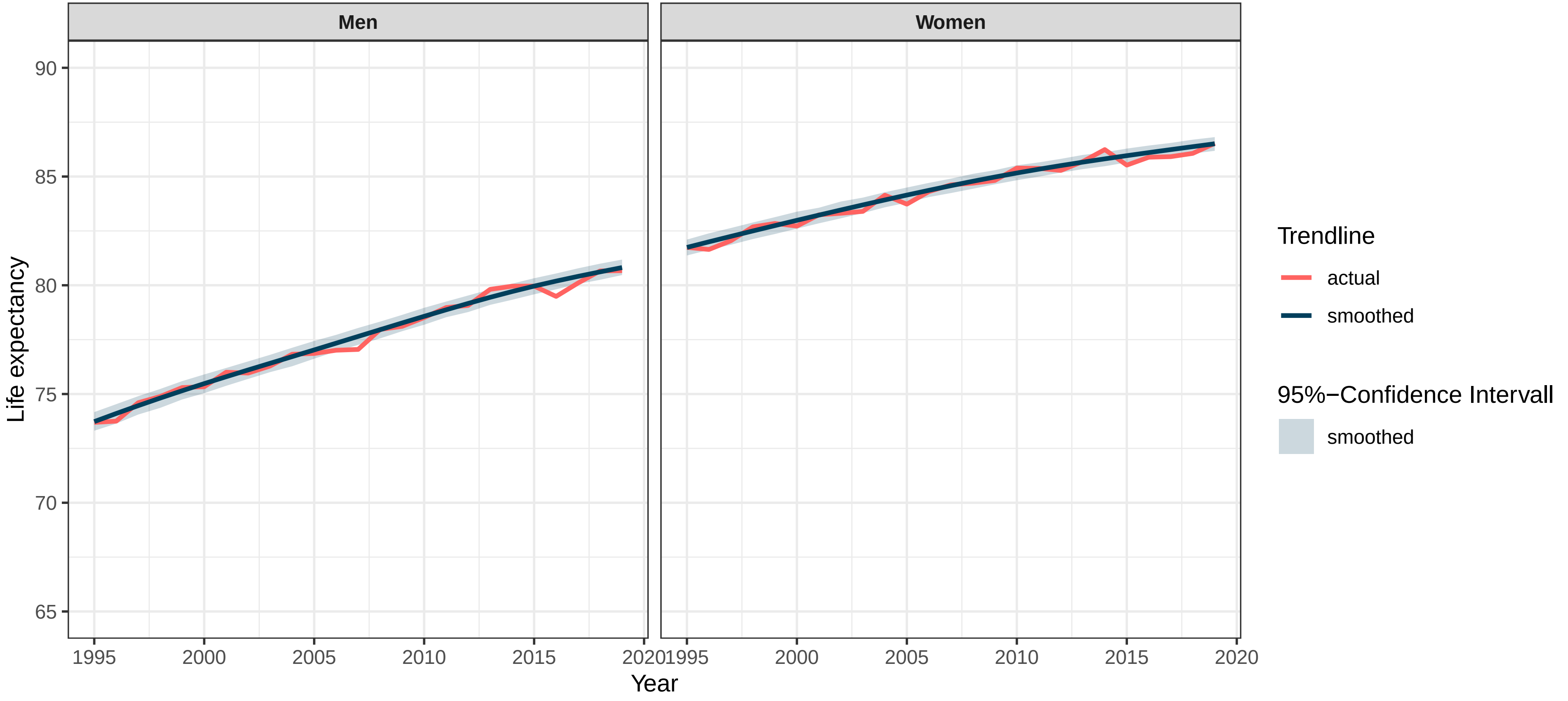

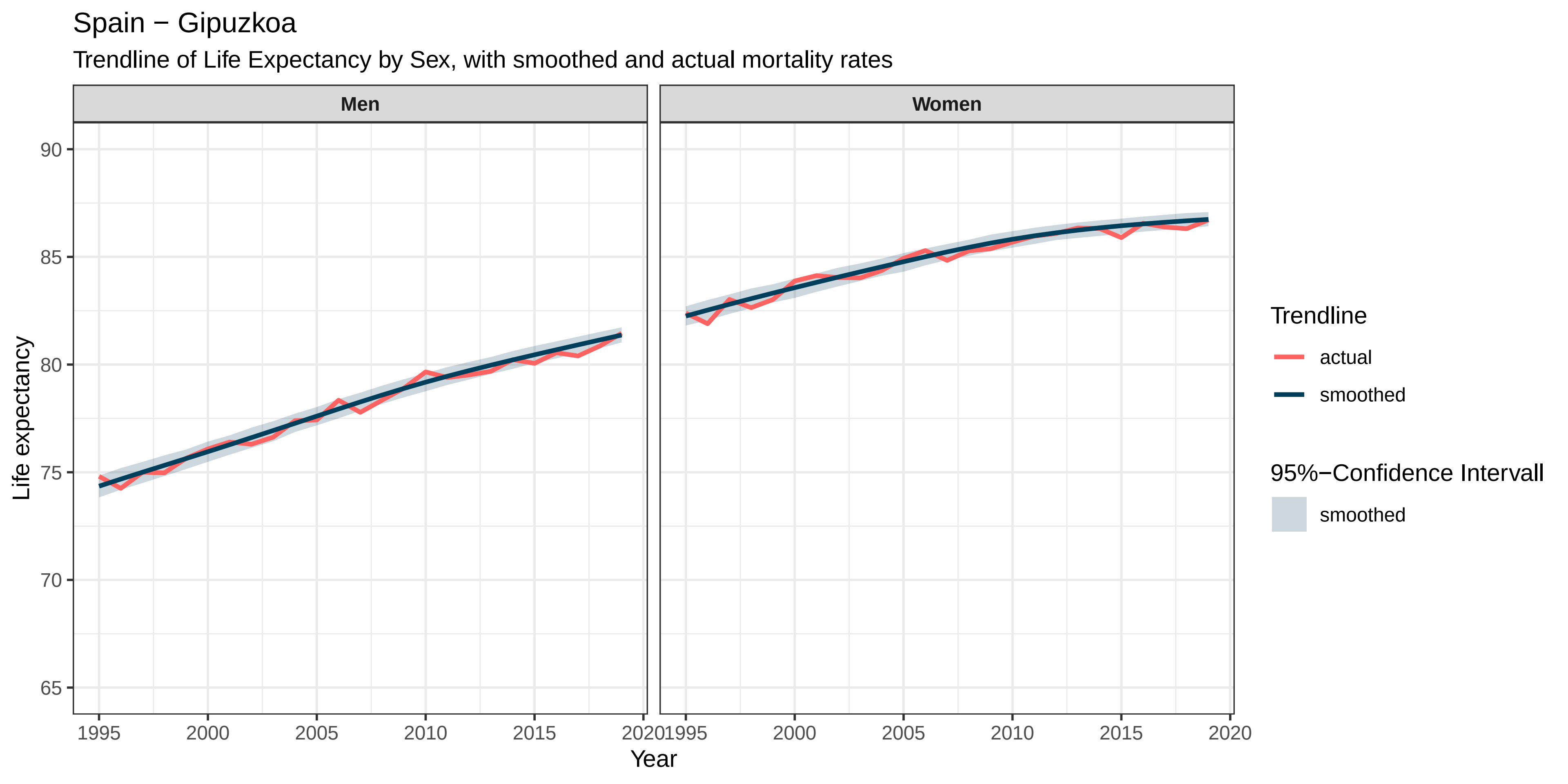

Spain − Gipuzkoa
Trendline of Life Expectancy by Sex, with smoothed and actual mortality rates
Men
Women
90
85
80
75
70
65
Life expectancy
1995
2000
2005
2010
2015
2020
Year
Trendline
actual
smoothed
95%−Confidence Intervall
smoothed

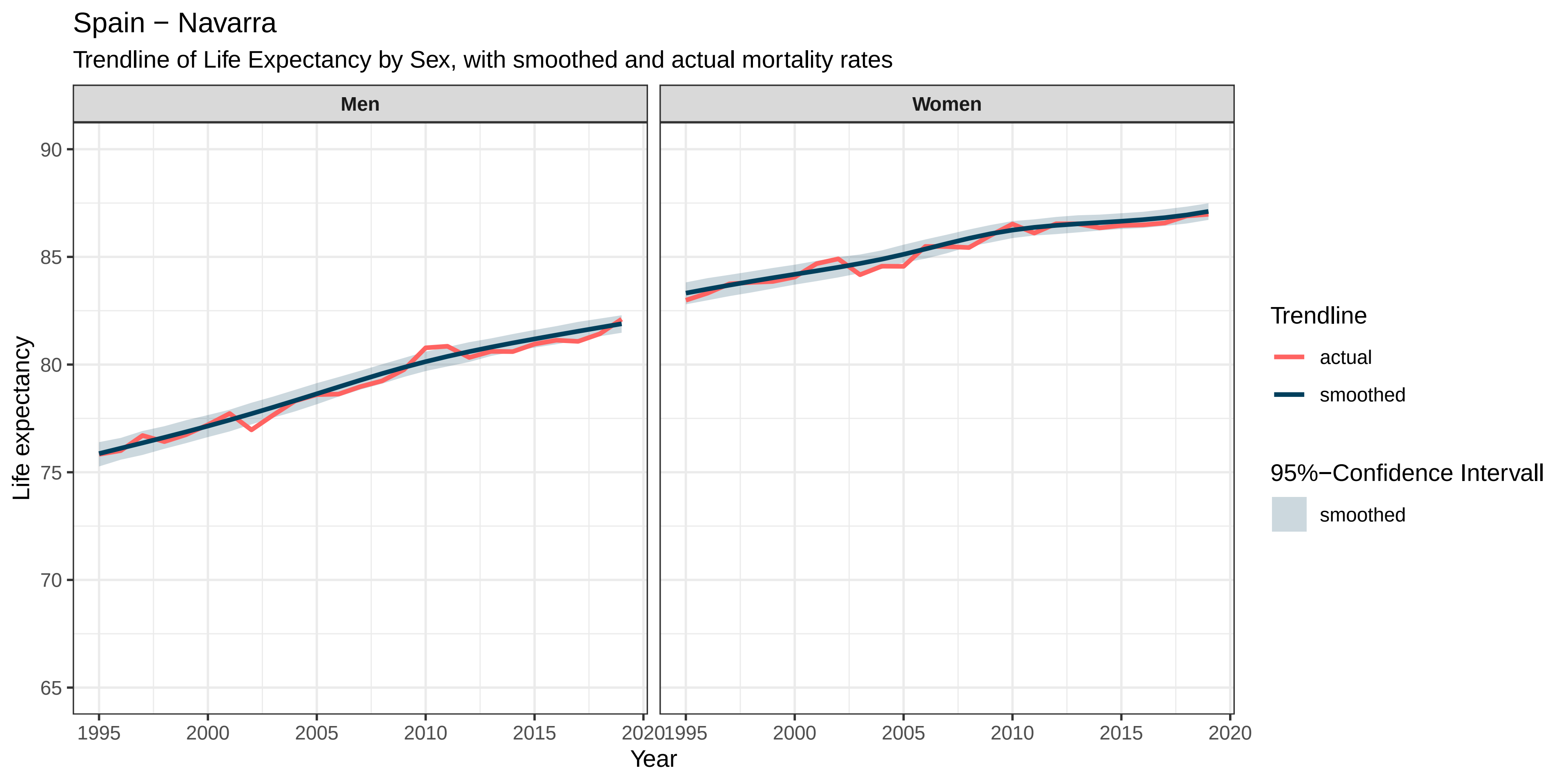
Spain − Navarra
Trendline of Life Expectancy by Sex, with smoothed and actual mortality rates
Men
Women
Life expectancy
Year
90
85
80
75
70
65
1995
2000
2005
2010
2015
2020
Trendline
actual
smoothed
95%−Confidence Intervall
smoothed

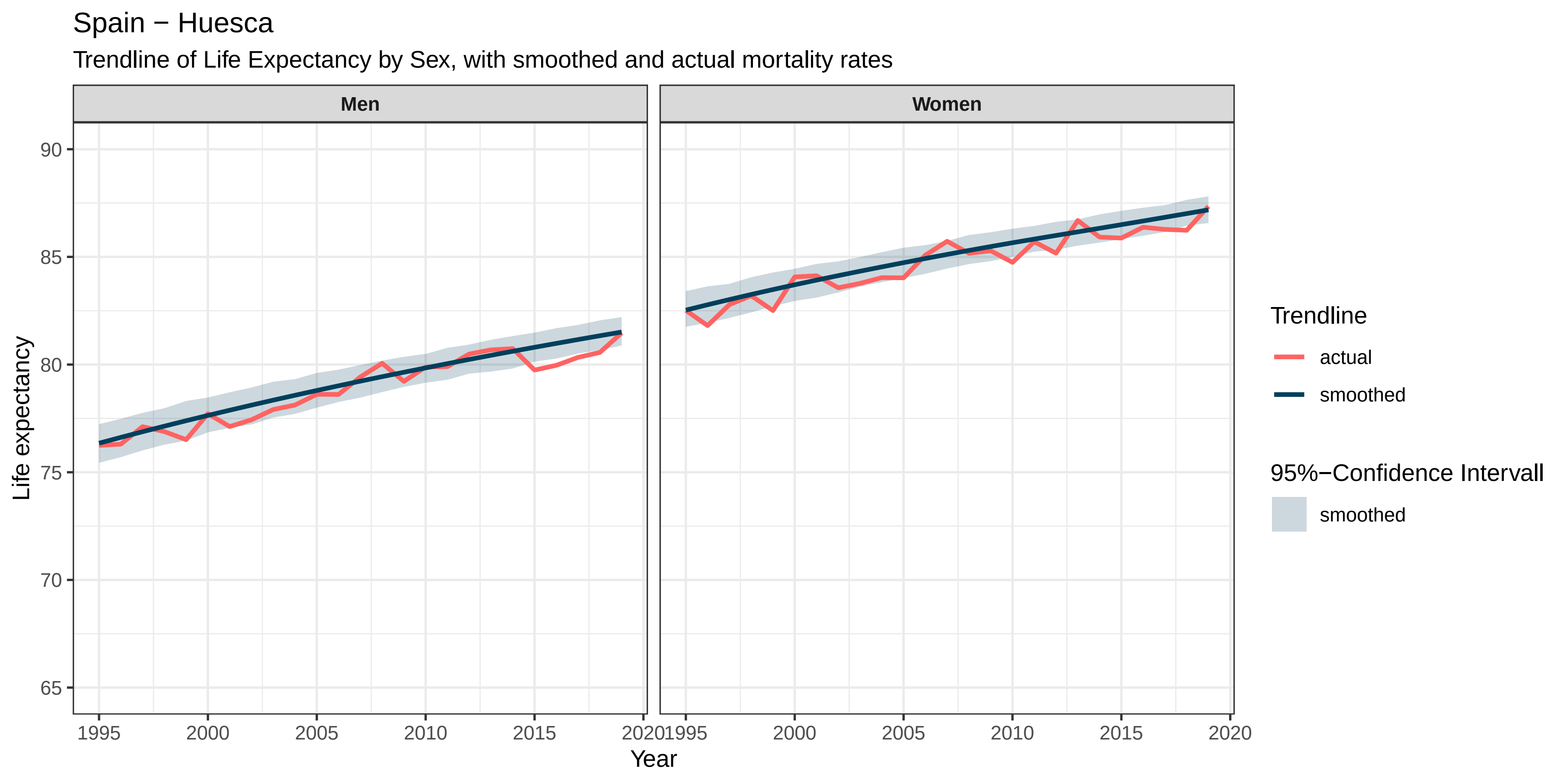

Spain − Huesca
Trendline of Life Expectancy by Sex, with smoothed and actual mortality rates
Men
Women
90
85
80
75
70
65
Life expectancy
1995
2000
2005
2010
2015
2020
Year
Trendline
actual
smoothed
95%−Confidence Intervall
smoothed

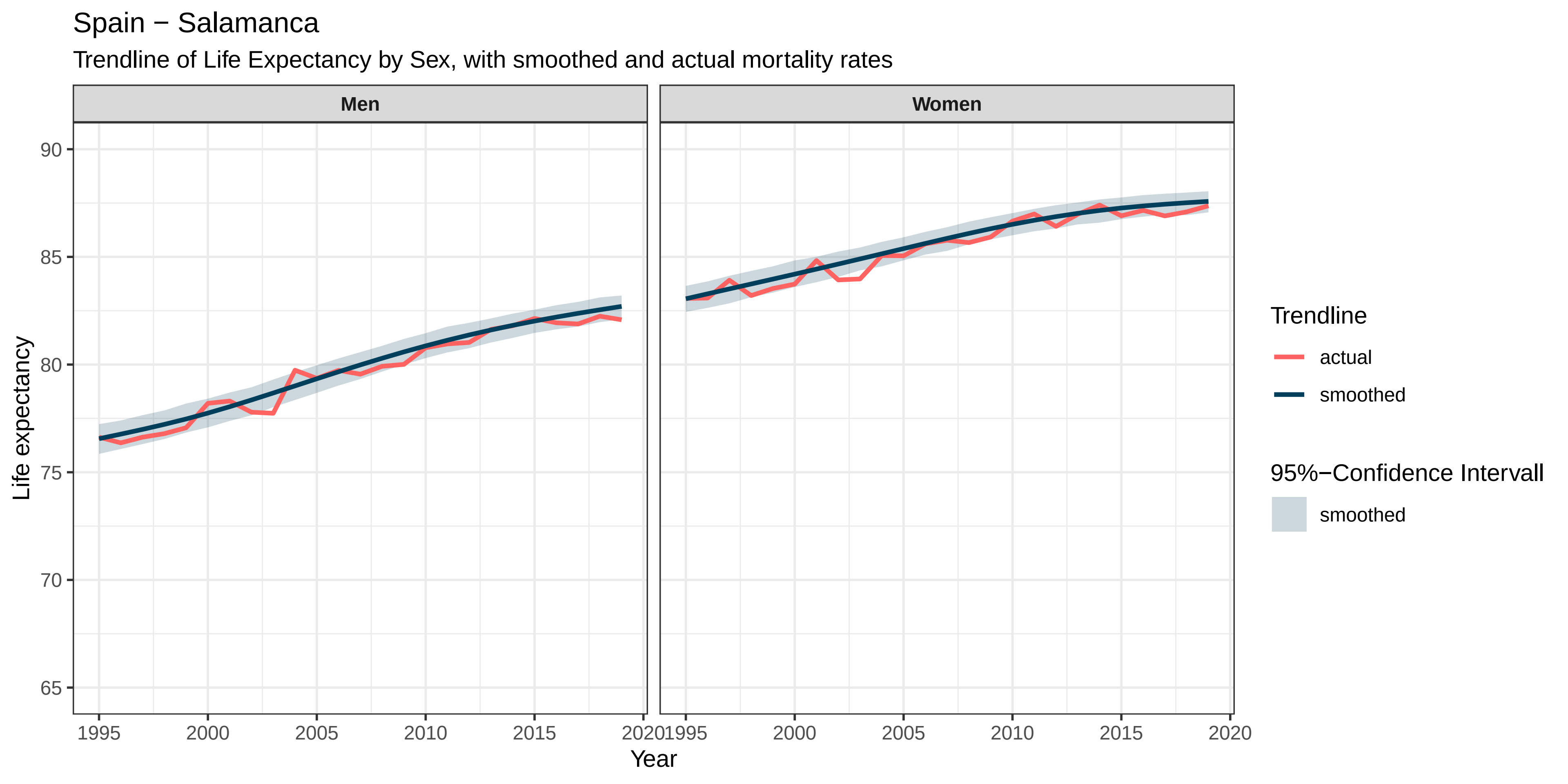
Spain − Salamanca
Trendline of Life Expectancy by Sex, with smoothed and actual mortality rates
Men
Women
Life expectancy
Year
90
85
80
75
70
65
1995
2000
2005
2010
2015
2020
Trendline
actual
smoothed
95%−Confidence Intervall
smoothed

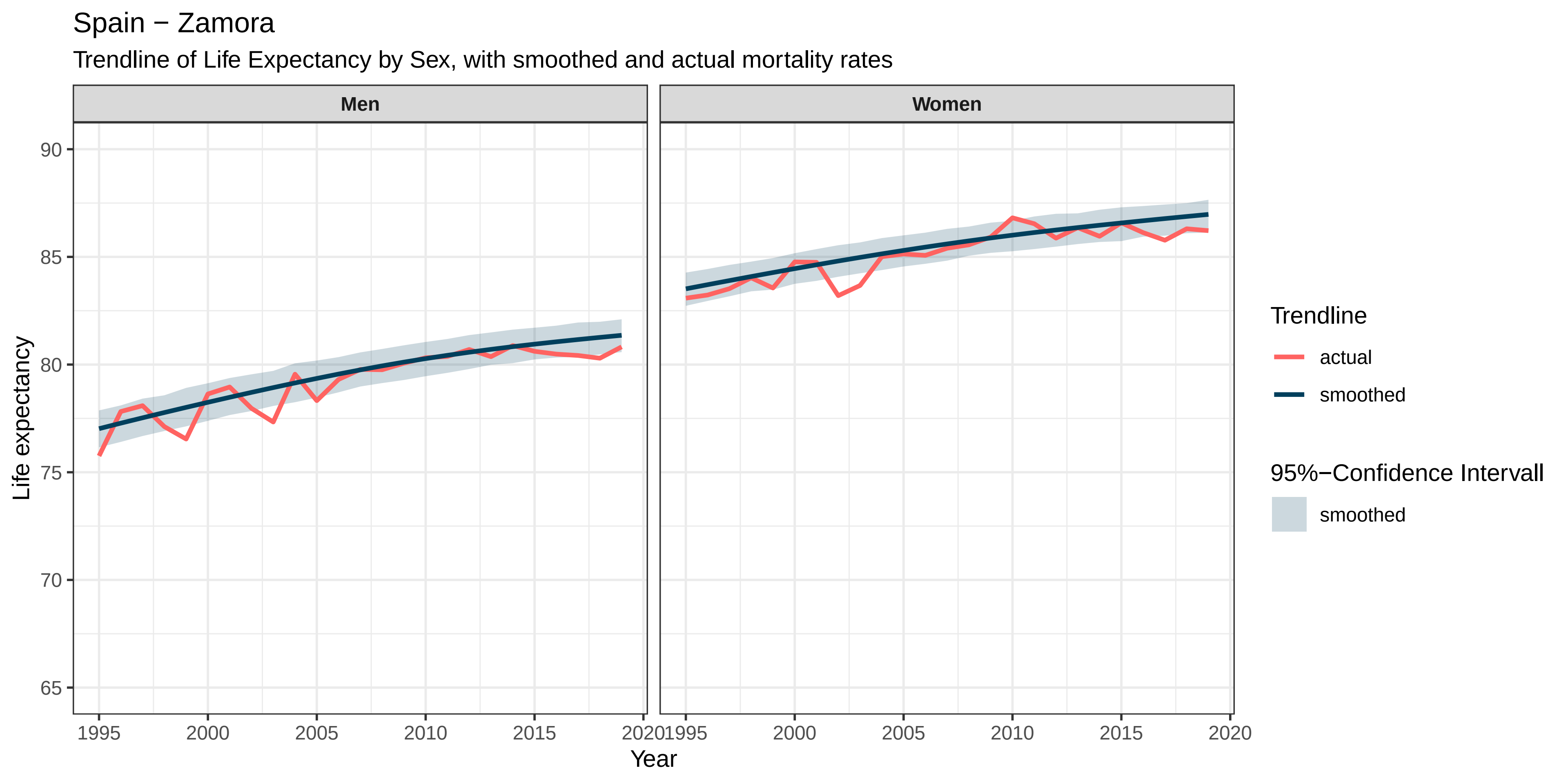

Spain − Zamora
Trendline of Life Expectancy by Sex, with smoothed and actual mortality rates
Men
Women
Life expectancy
Year
90
85
80
75
70
65
1995
2000
2005
2010
2015
2020
Trendline
actual
smoothed
95%−Confidence Intervall
smoothed

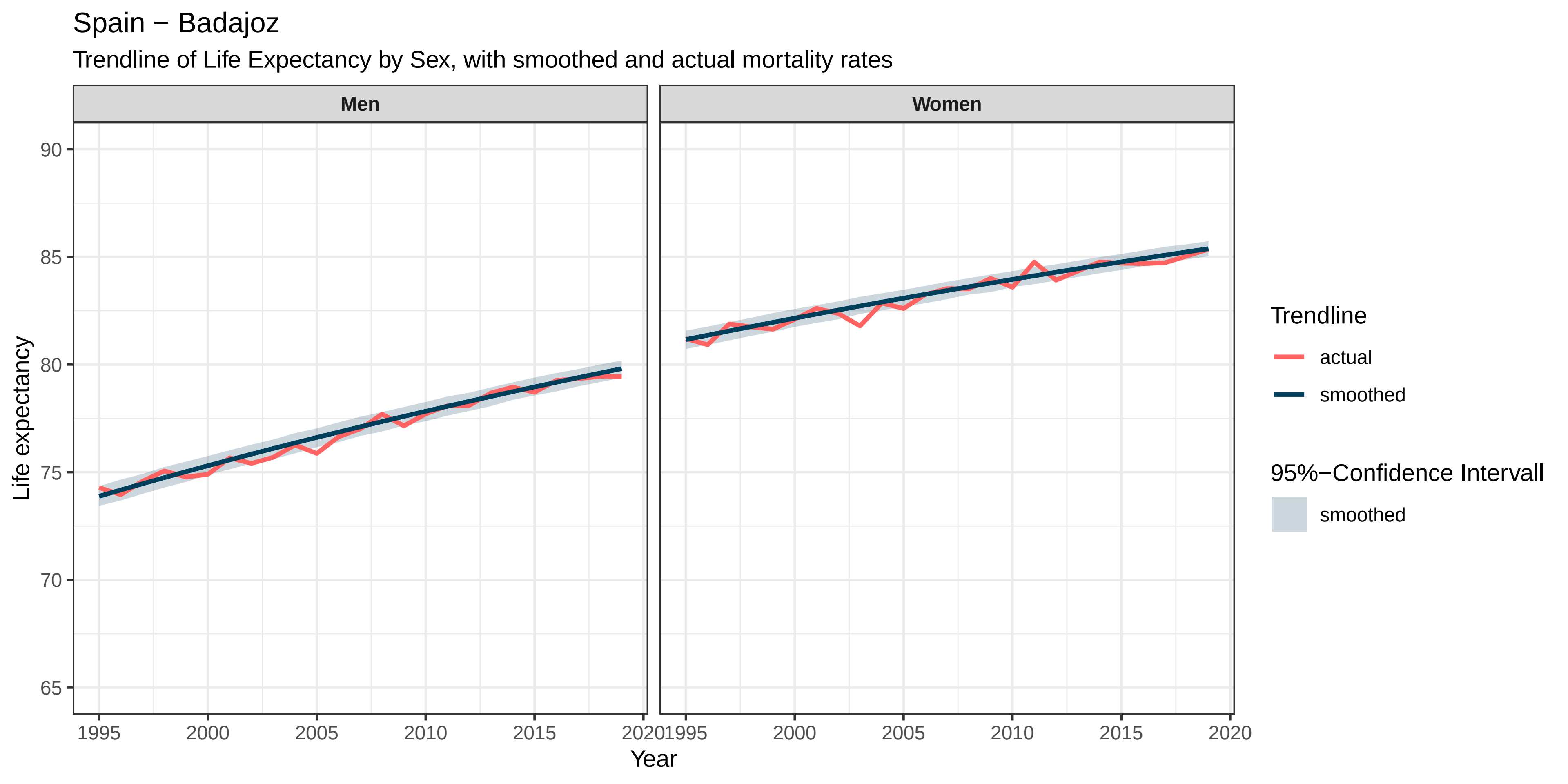

Spain − Badajoz
Trendline of Life Expectancy by Sex, with smoothed and actual mortality rates
Men
Women
Life expectancy
90
85
80
75
70
65
1995
2000
2005
2010
2015
2020
Year
Trendline
actual
smoothed
95%−Confidence Intervall
smoothed

Spain − Cáceres

Trendline of Life Expectancy by Sex, with smoothed and actual mortality rates

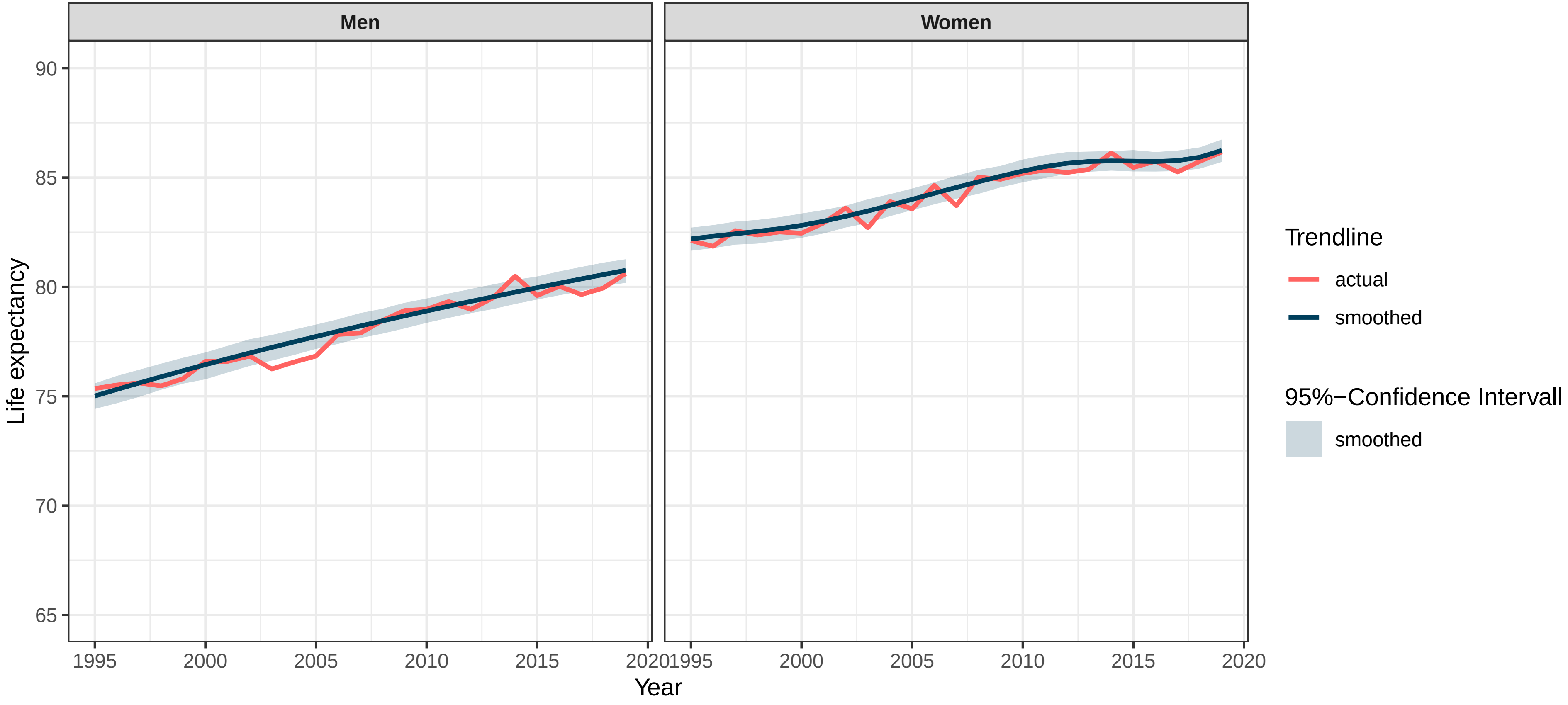

Spain − Girona

Trendline of Life Expectancy by Sex, with smoothed and actual mortality rates

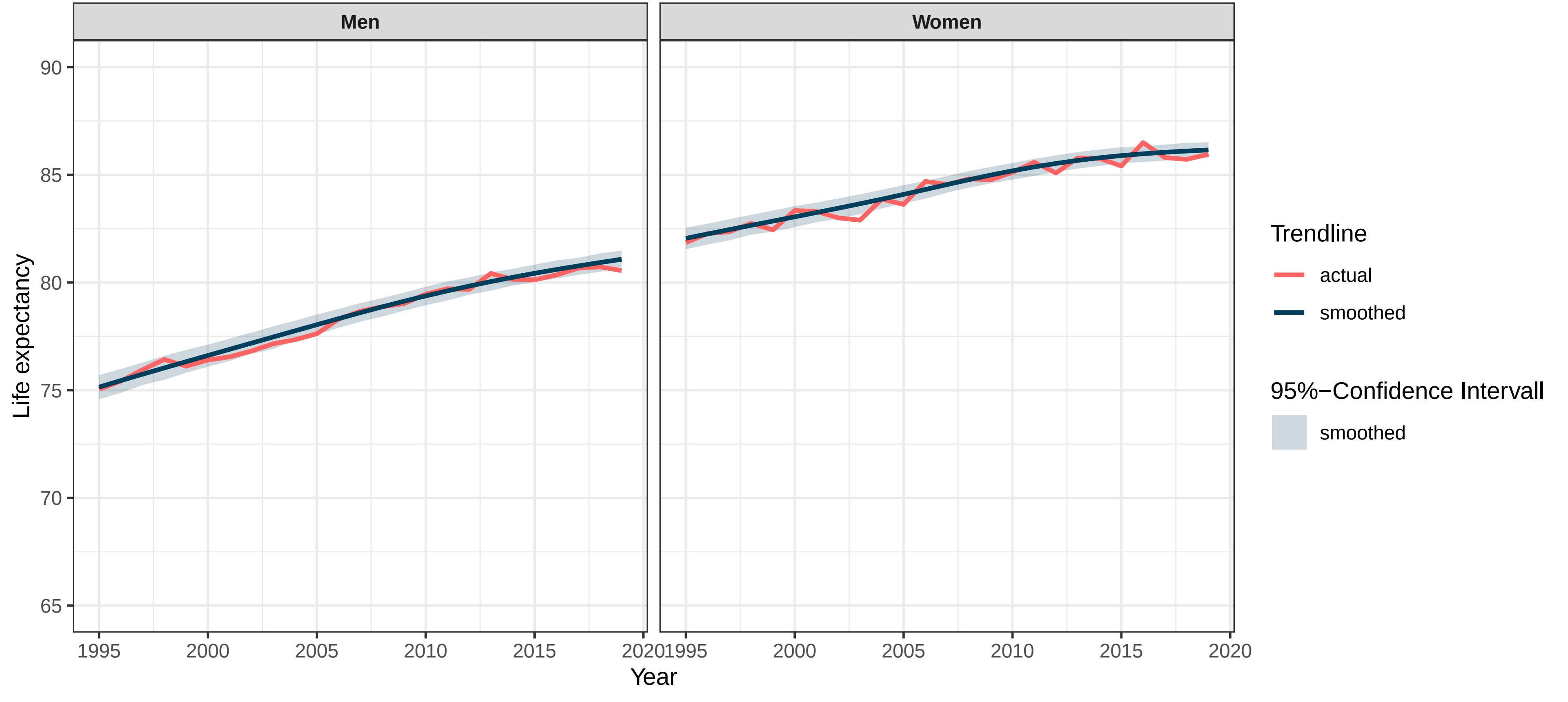

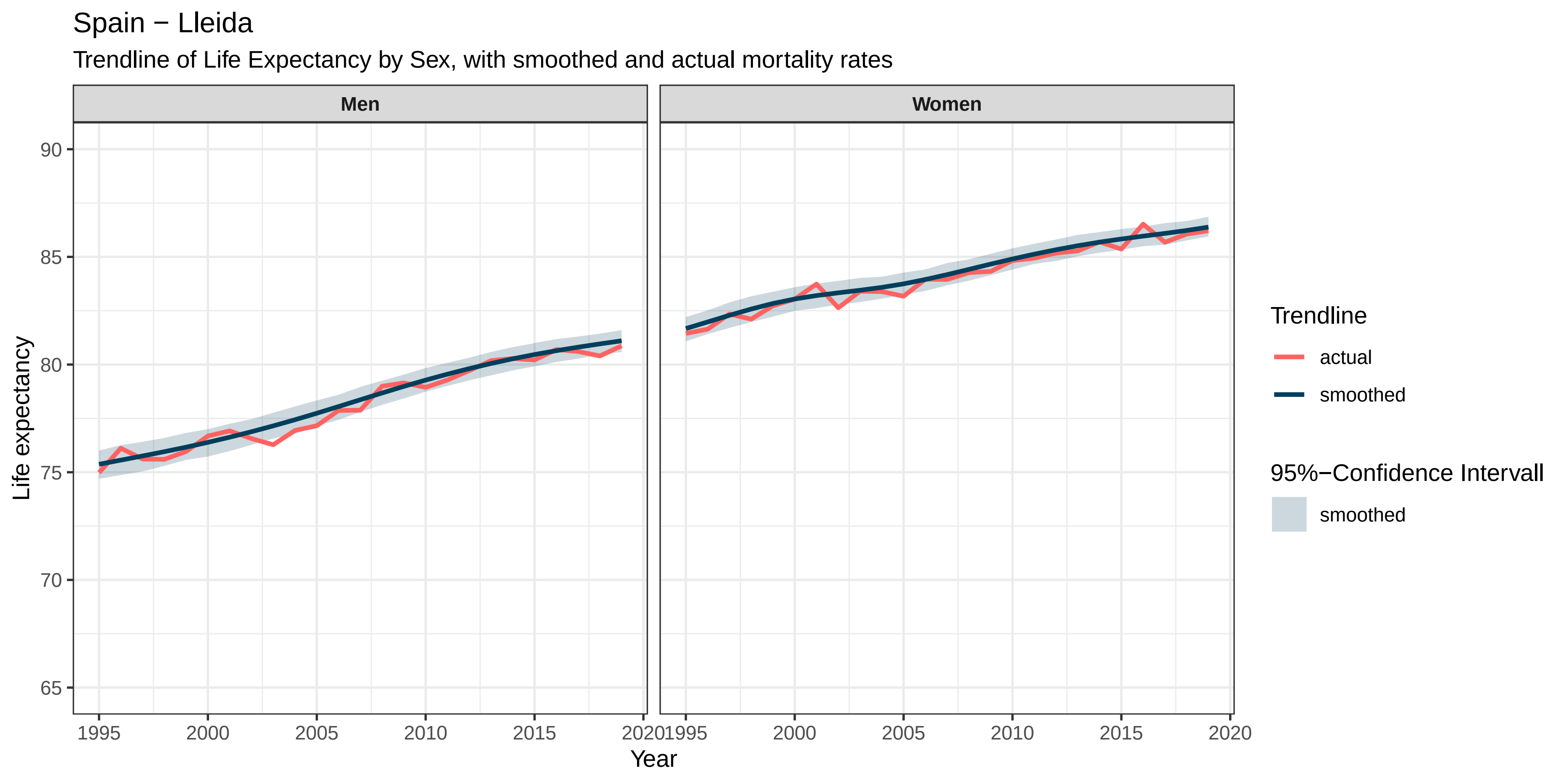

Spain − Lleida
Trendline of Life Expectancy by Sex, with smoothed and actual mortality rates
Men
Women
90
85
80
75
70
65
Life expectancy
1995
2000
2005
2010
2015
2020
Year
Trendline
actual
smoothed
95%−Confidence Intervall
smoothed

Spain − Huelva

Trendline of Life Expectancy by Sex, with smoothed and actual mortality rates

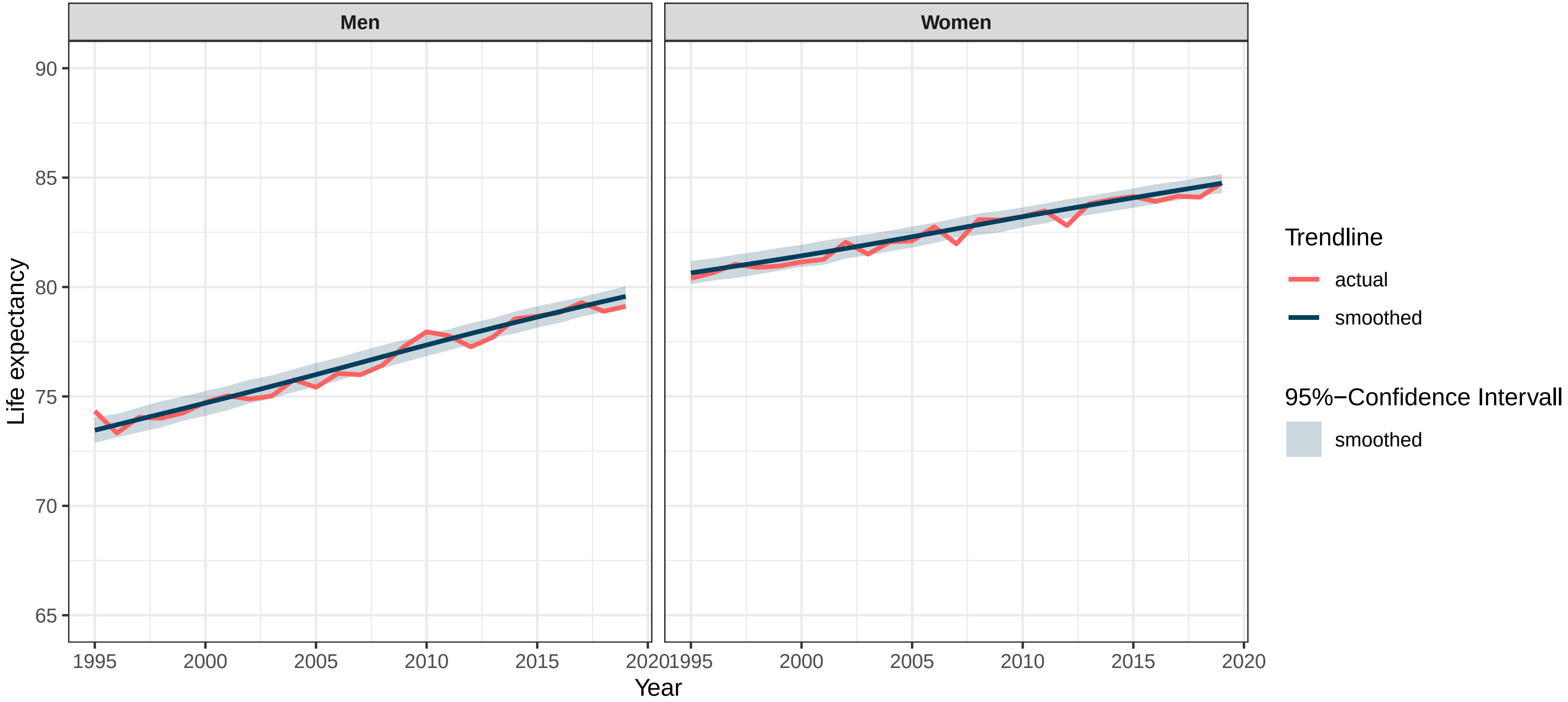

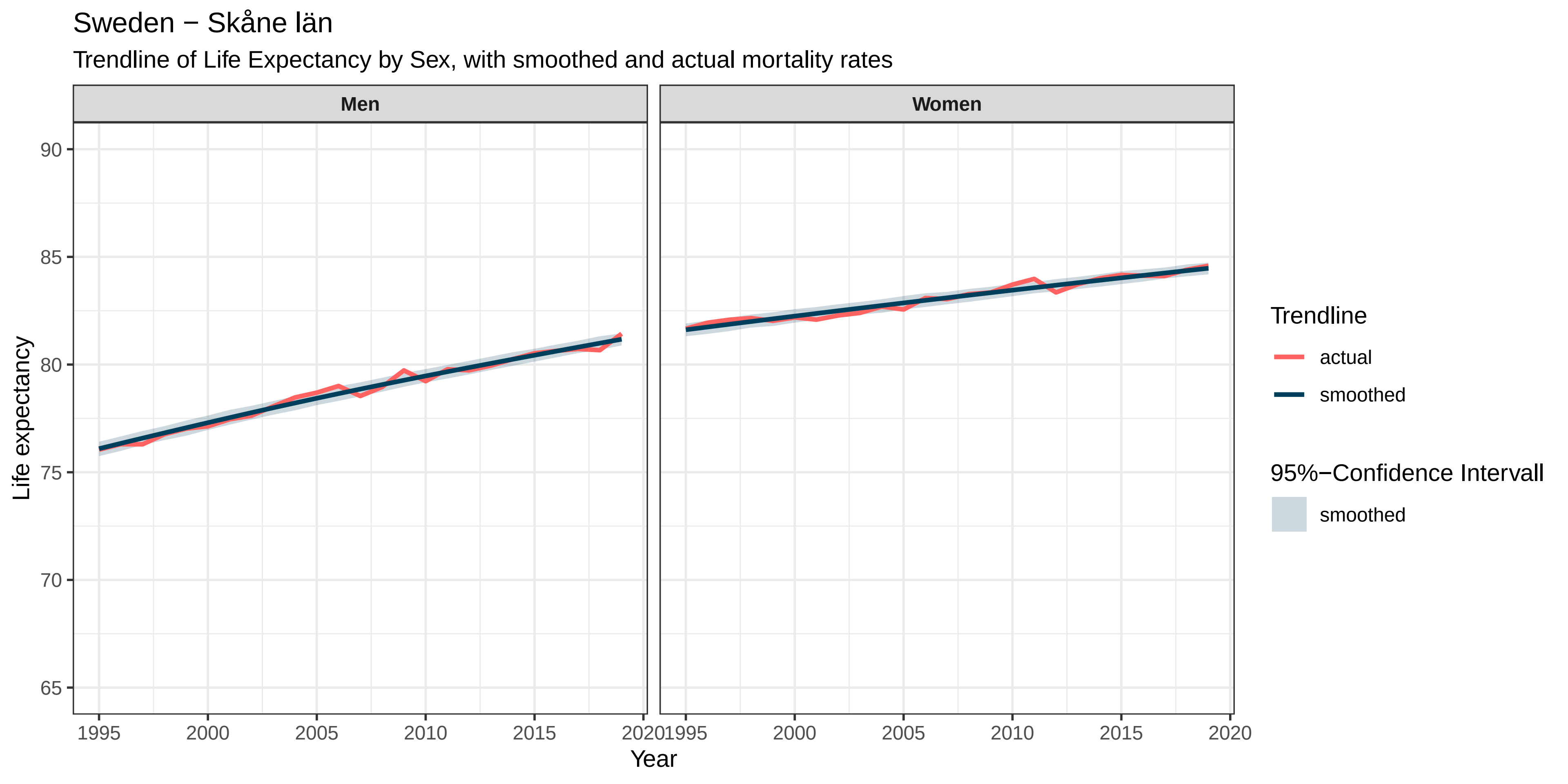

Sweden − Skåne län
Trendline of Life Expectancy by Sex, with smoothed and actual mortality rates
Men
Women
Life expectancy
90
85
80
75
70
65
1995
2000
2005
2010
2015
2020
Year
Trendline
actual
smoothed
95%−Confidence Intervall
smoothed

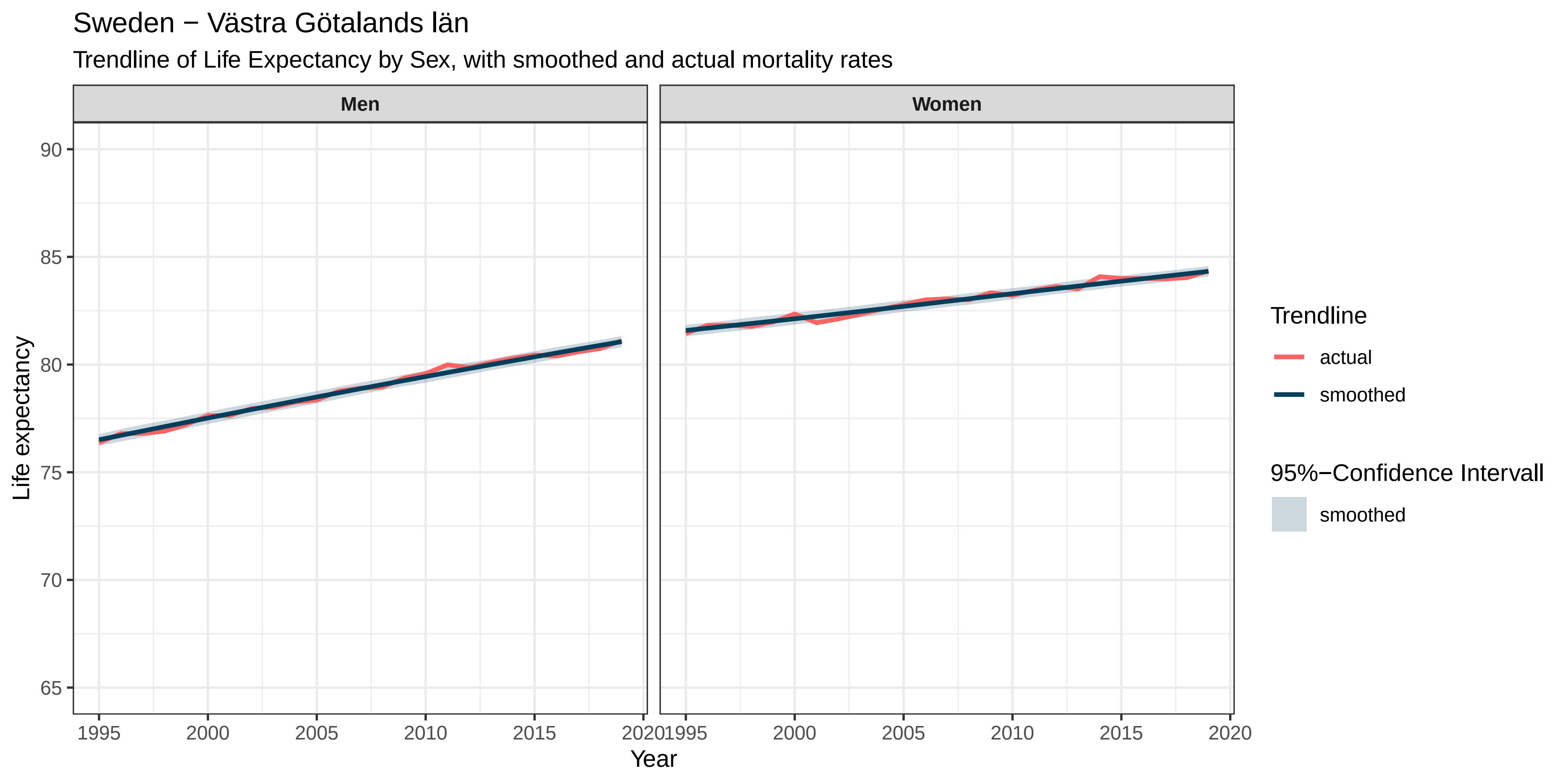
Sweden − Västra Götalands län
Trendline of Life Expectancy by Sex, with smoothed and actual mortality rates
Men
Women
Life expectancy
90
85
80
75
70
65
1995
2000
2005
2010
2015
2020
Year
Trendline
actual
smoothed
95%−Confidence Intervall
smoothed

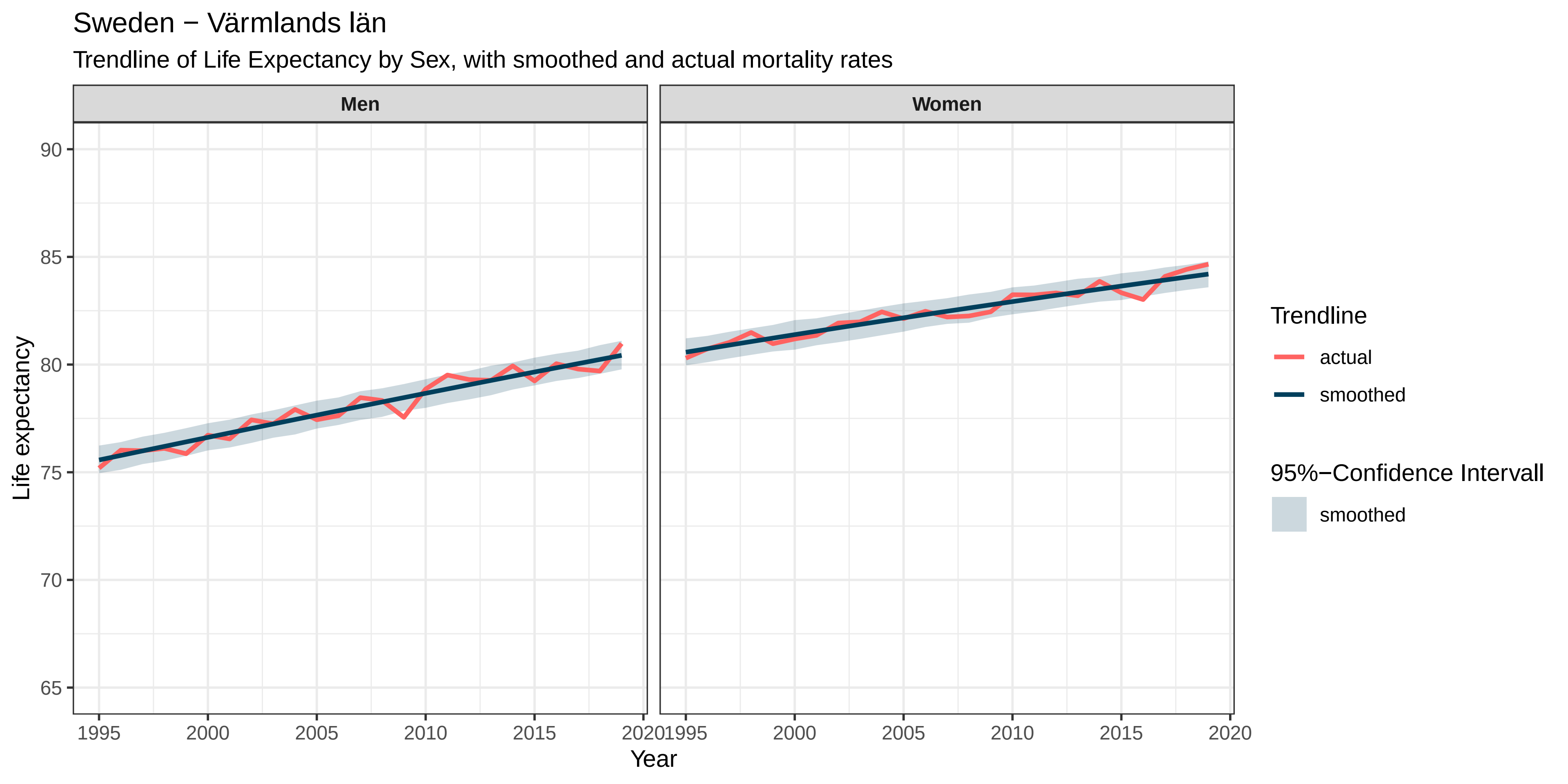
Sweden − Värmlands län
Trendline of Life Expectancy by Sex, with smoothed and actual mortality rates
Men
Women
Life expectancy
90
85
80
75
70
65
1995
2000
2005
2010
2015
2020
Year
Trendline
actual
smoothed
95%−Confidence Intervall
smoothed

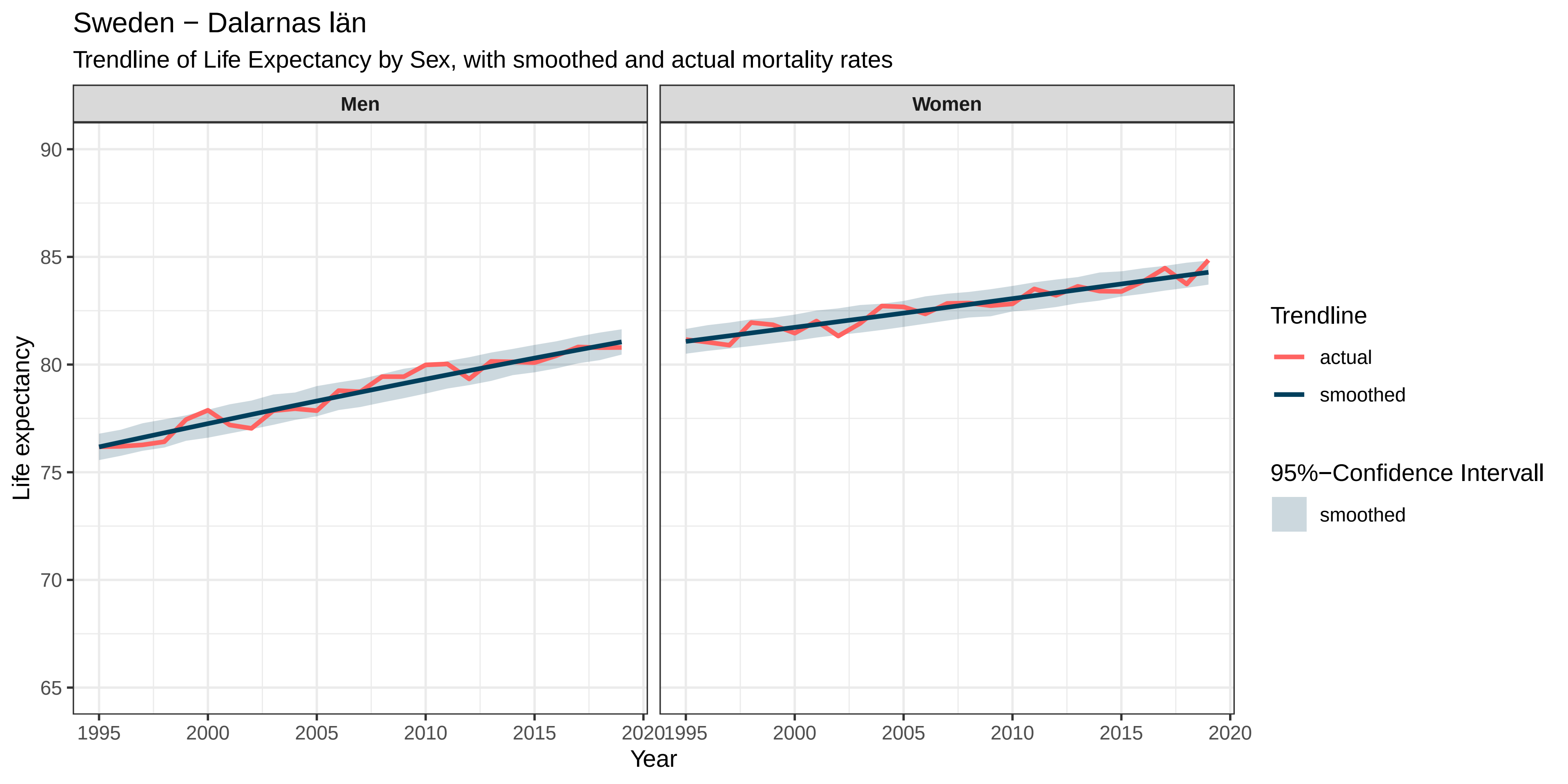
Sweden − Dalarnas län
Trendline of Life Expectancy by Sex, with smoothed and actual mortality rates
Men
Women
Life expectancy
90
85
80
75
70
65
1995
2000
2005
2010
2015
2020
Year
Trendline
actual
smoothed
95%−Confidence Intervall
smoothed

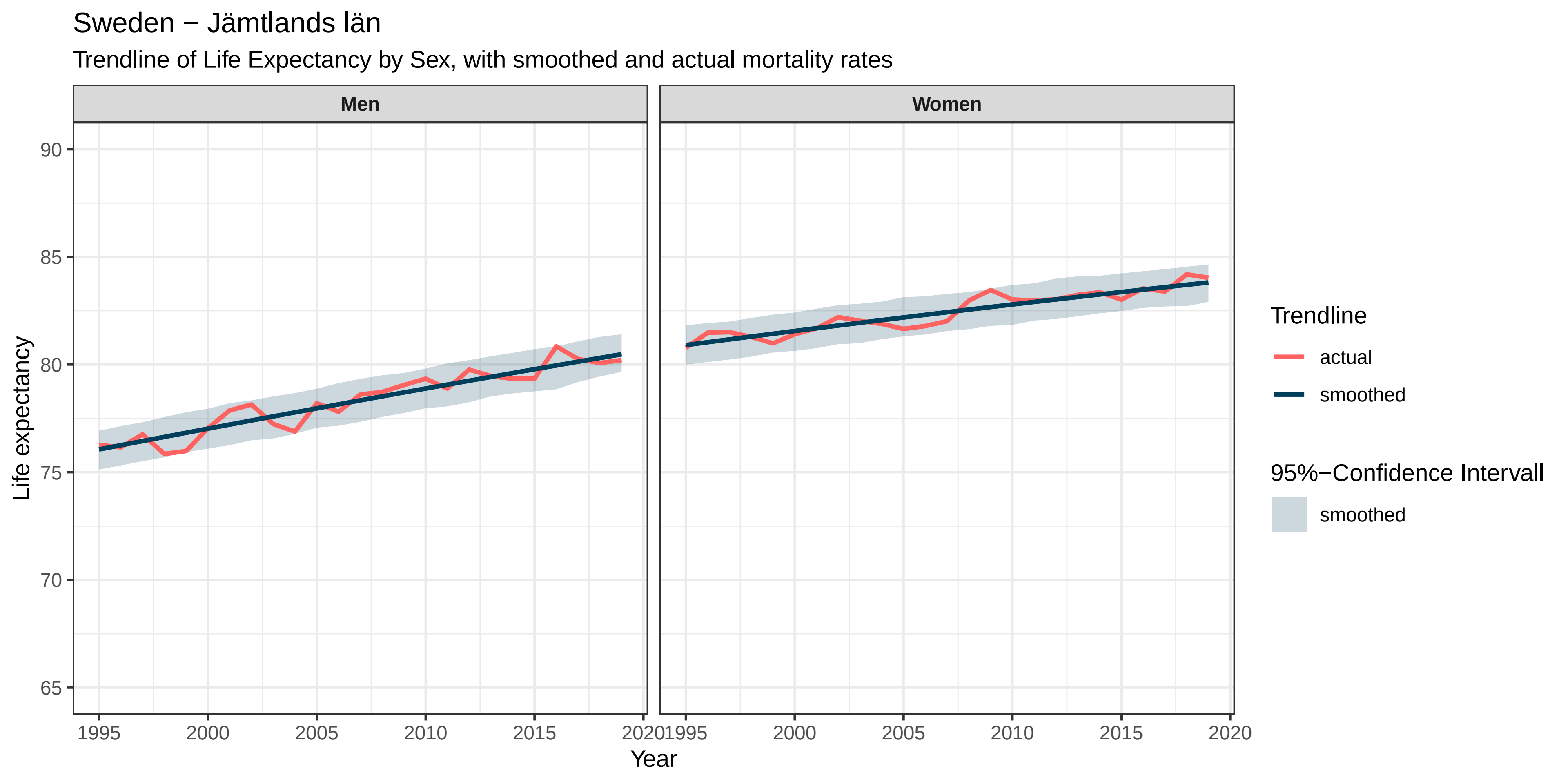

Sweden − Jämtlands län
Trendline of Life Expectancy by Sex, with smoothed and actual mortality rates
Men
Women
Life expectancy
90
85
80
75
70
65
1995
2000
2005
2010
2015
2020
Year
Trendline
actual
smoothed
95%−Confidence Intervall
smoothed

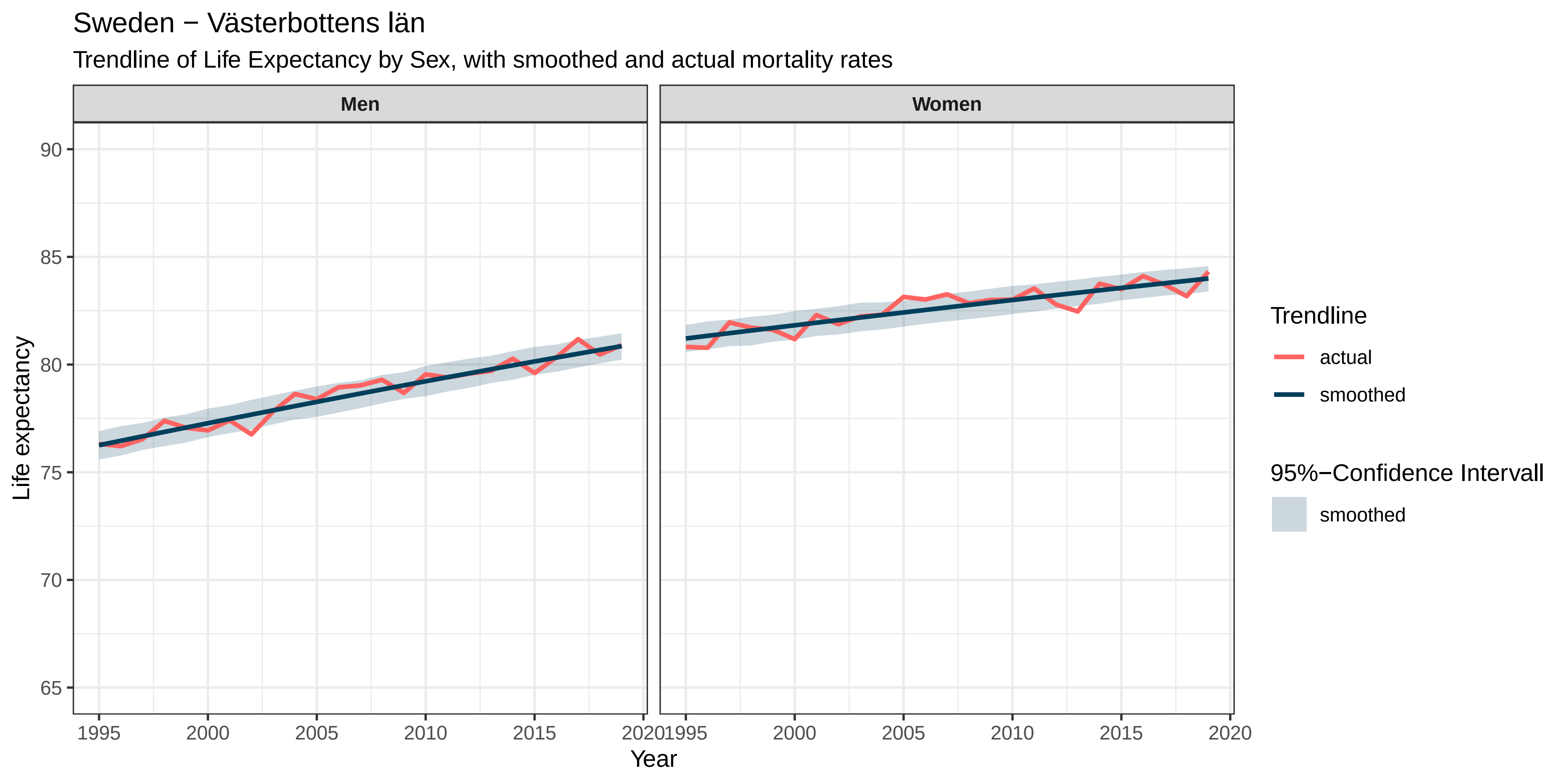

Sweden − Västerbottens län
Trendline of Life Expectancy by Sex, with smoothed and actual mortality rates
Men
Women
90
85
80
75
70
65
Life expectancy
1995
2000
2005
2010
2015
2020
Year
Trendline
actual
smoothed
95%−Confidence Intervall
smoothed

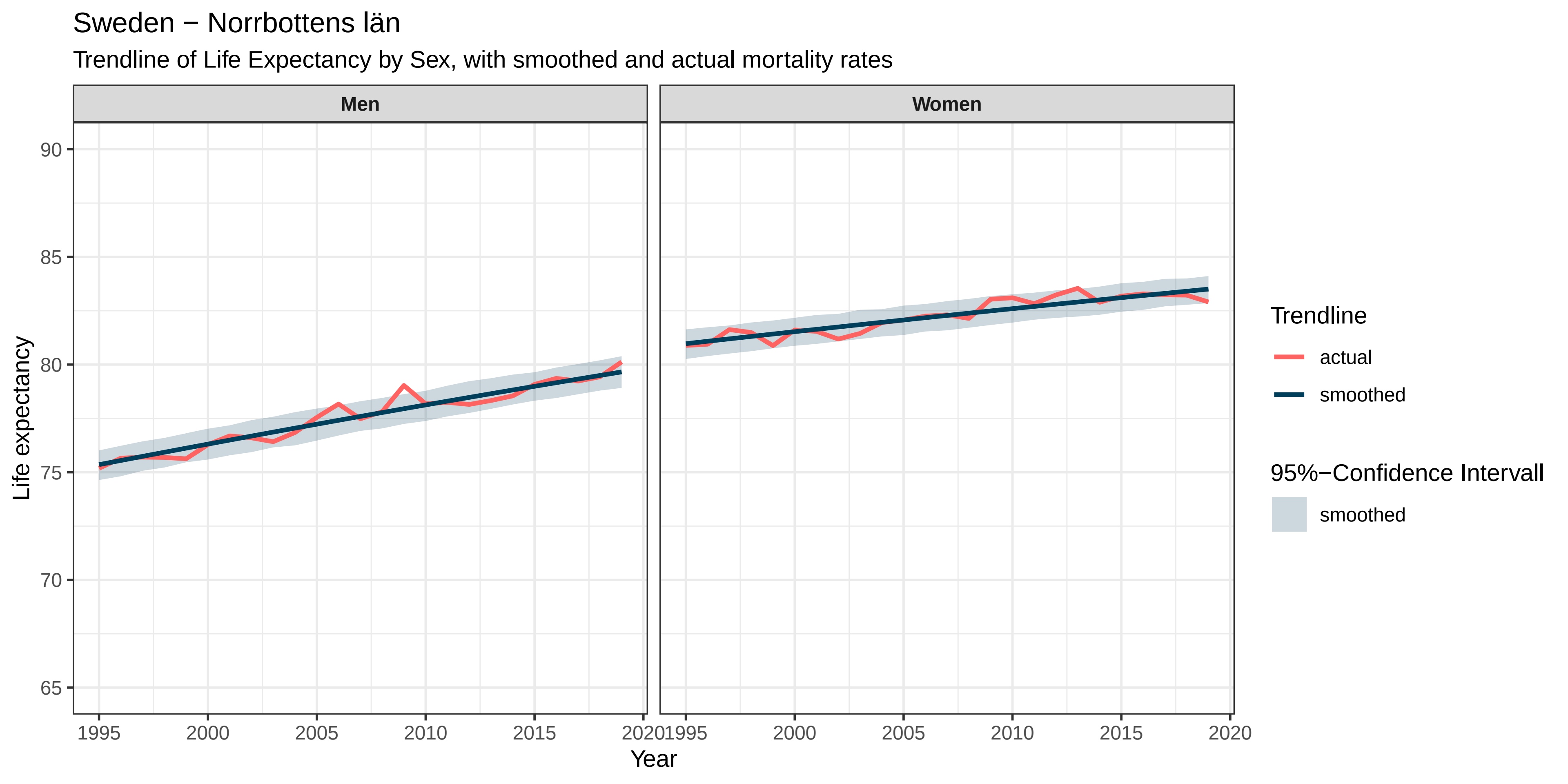
Sweden − Norrbottens län
Trendline of Life Expectancy by Sex, with smoothed and actual mortality rates
Men
Women
Life expectancy
90
85
80
75
70
65
1995
2000
2005
2010
2015
2020
Year
Trendline
actual
smoothed
95%−Confidence Intervall
smoothed
