# Supplementary material for: Getting closer to each other? Convergence and divergence patterns of life expectancy in 277 border regions of Western Europe 1995–2019
Source: Eur J Epidemiol. 2025 Jul 19;40(9):1031–43. doi: 10.1007/s10654-025-01279-w (PMC12537618; doi:10.1007/s10654-025-01279-w)
Supplement: Supplementary file 5 — Supplementary Material 5 [file 10654_2025_1279_MOESM5_ESM.pdf]

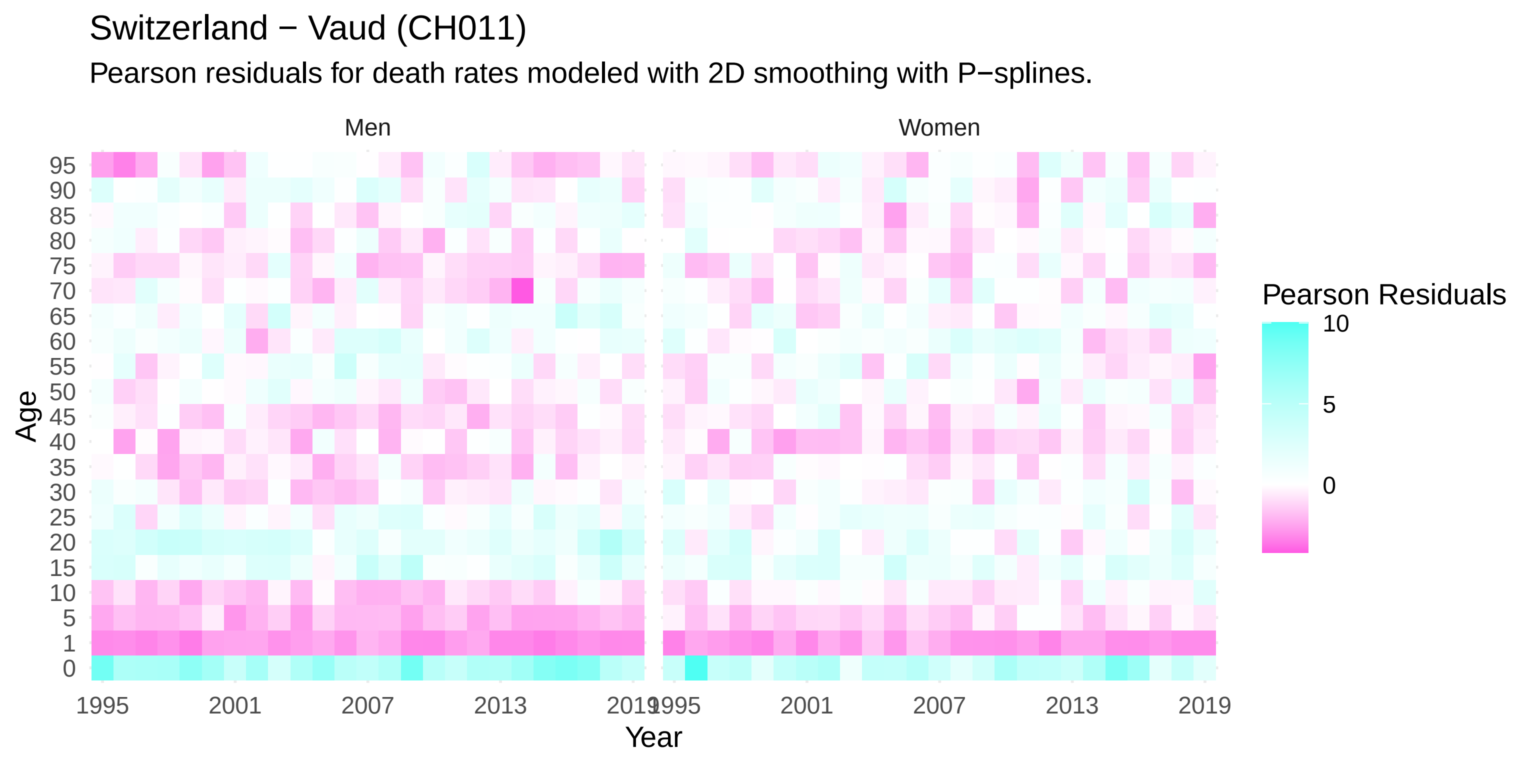

Switzerland − Vaud (CH011)
Pearson residuals for death rates modeled with 2D smoothing with P−splines.
Men
Women
Age
95
90
85
80
75
70
65
60
55
50
45
40
35
30
25
20
15
10
5
1
0
1995
2001
2007
2013
2019
Year
Pearson Residuals
10
5
0

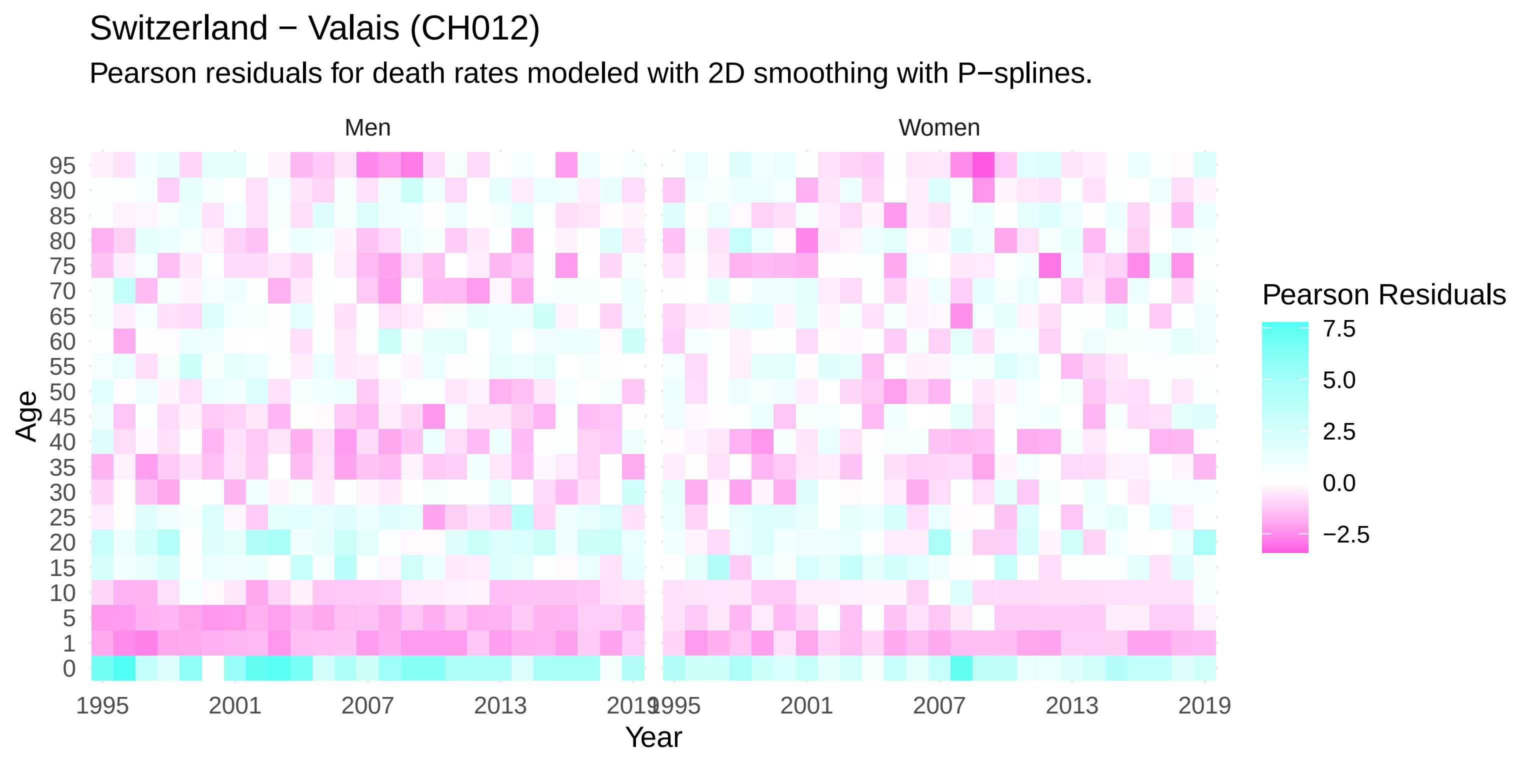

Switzerland − Valais (CH012)
Pearson residuals for death rates modeled with 2D smoothing with P−splines.
Men
Women
Age
Year
Pearson Residuals
7.5
5.0
2.5
0.0
−2.5
95
90
85
80
75
70
65
60
55
50
45
40
35
30
25
20
15
10
5
1
0
1995
2001
2007
2013
2019

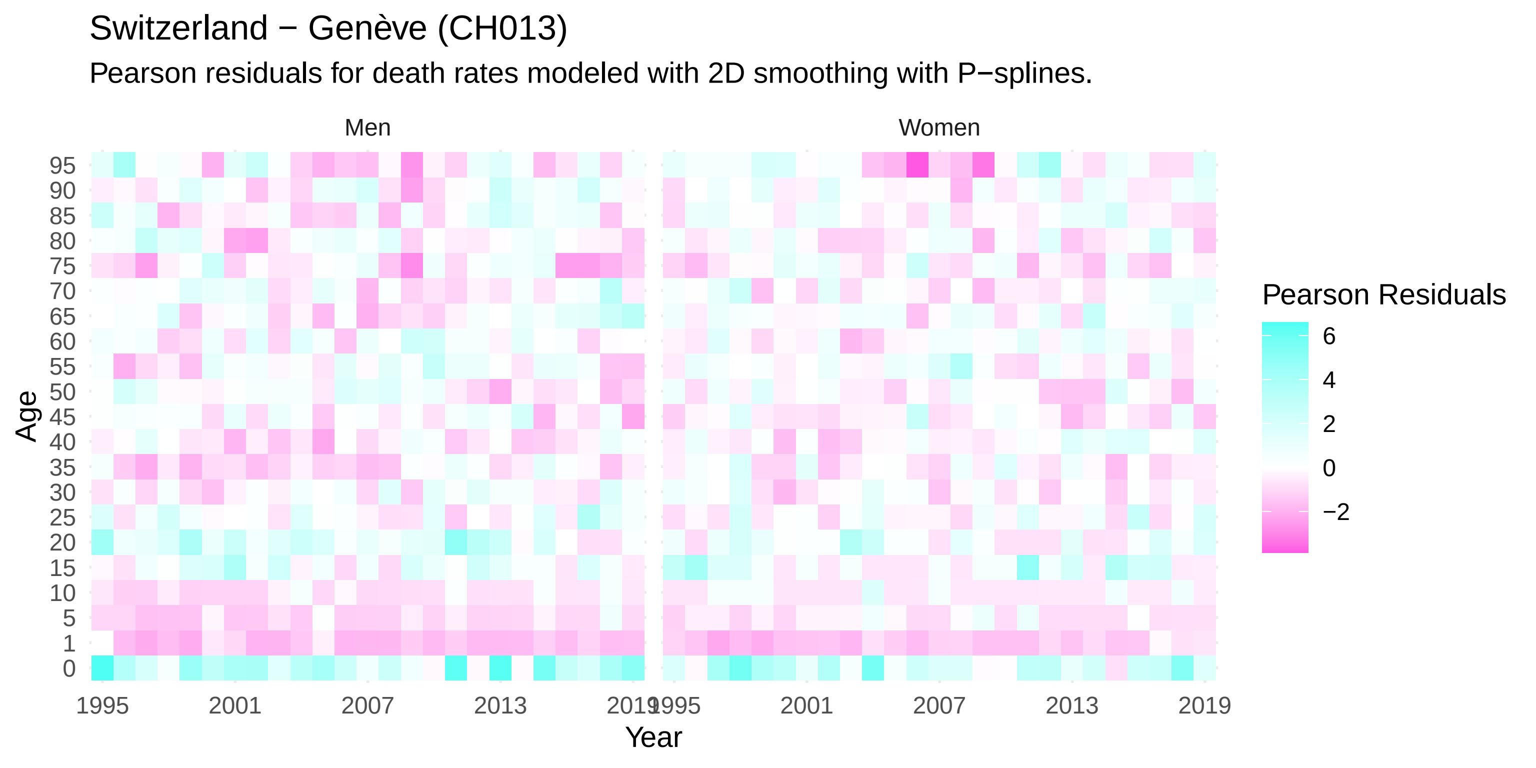
Switzerland − Genève (CH013)
Pearson residuals for death rates modeled with 2D smoothing with P−splines.
Men
Women
Age
95
90
85
80
75
70
65
60
55
50
45
40
35
30
25
20
15
10
5
1
0
1995
2001
2007
2013
2019
Year
Pearson Residuals
6
4
2
0
−2

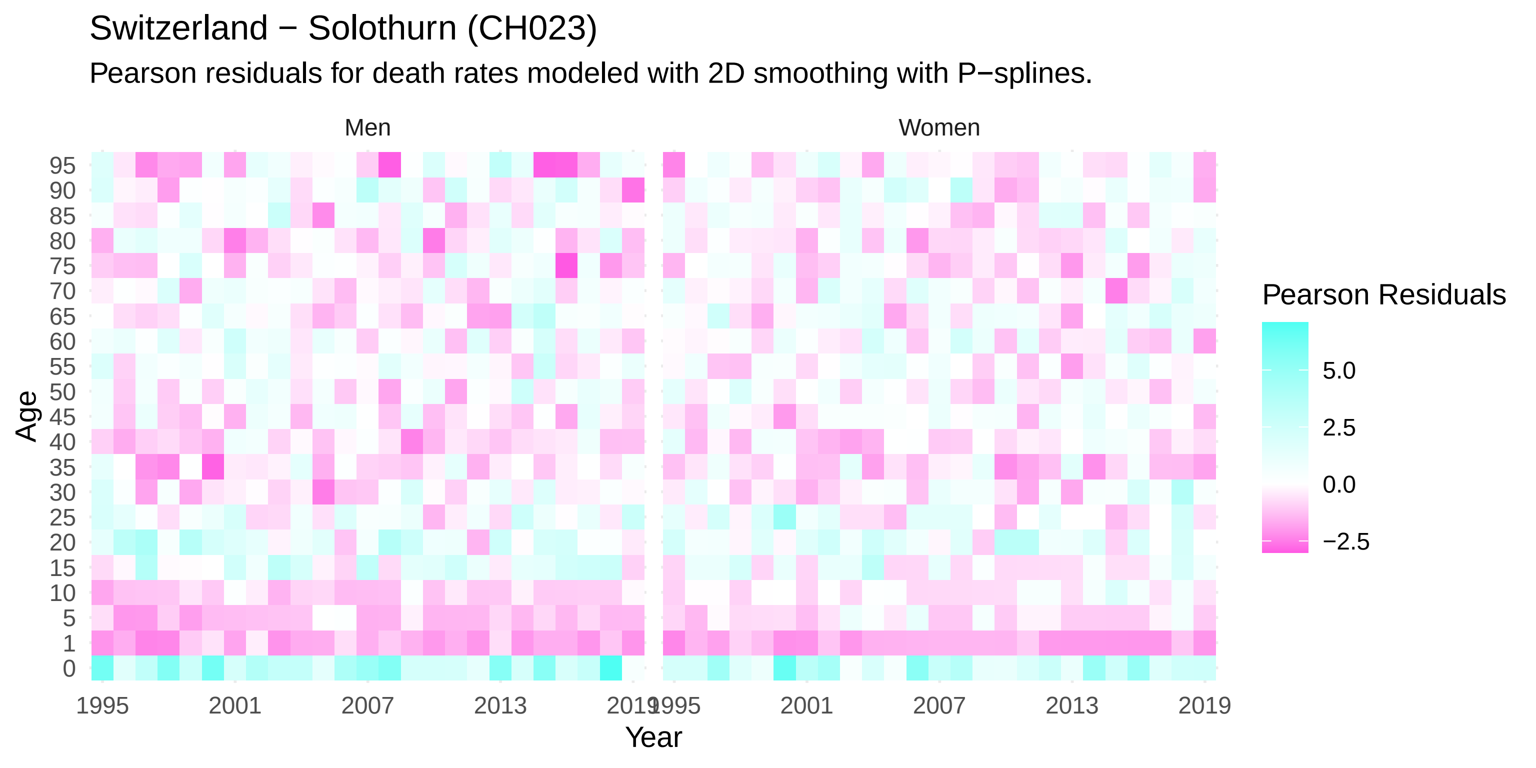
Switzerland − Solothurn (CH023)
Pearson residuals for death rates modeled with 2D smoothing with P−splines.
Men
Women
Age
95
90
85
80
75
70
65
60
55
50
45
40
35
30
25
20
15
10
5
1
0
1995
2001
2007
2013
2019
Year
Pearson Residuals
5.0
2.5
0.0
−2.5

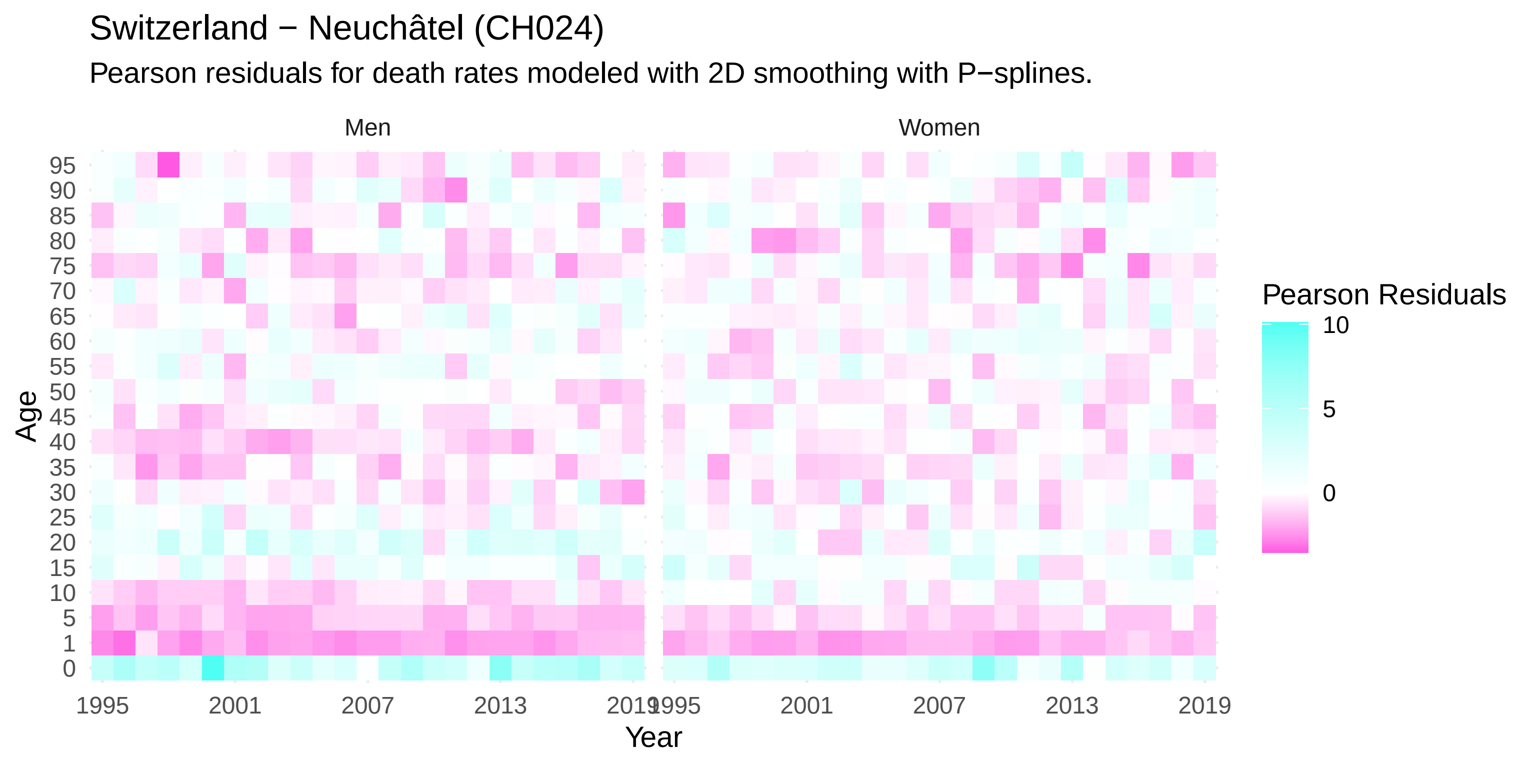
Switzerland − Neuchâtel (CH024)
Pearson residuals for death rates modeled with 2D smoothing with P−splines.
Men
Women
Age
95
90
85
80
75
70
65
60
55
50
45
40
35
30
25
20
15
10
5
1
0
1995
2001
2007
2013
2019
Year
Pearson Residuals
10
5
0

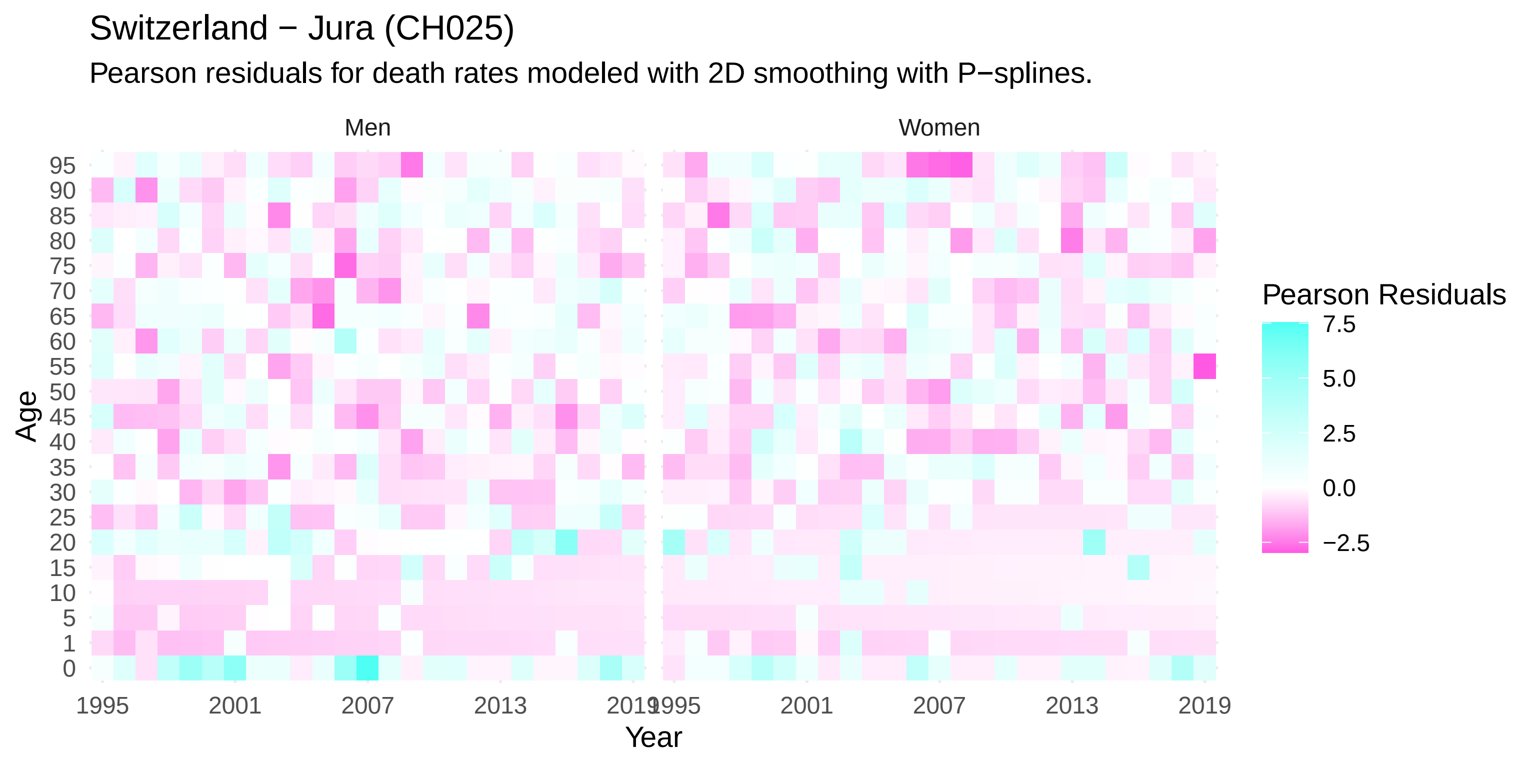

Switzerland − Jura (CH025)
Pearson residuals for death rates modeled with 2D smoothing with P−splines.
Men
Women
Age
95
90
85
80
75
70
65
60
55
50
45
40
35
30
25
20
15
10
5
1
0
1995
2001
2007
2013
2019
Year
Pearson Residuals
7.5
5.0
2.5
0.0
−2.5

Switzerland − Basel−Stadt (CH031)

Pearson residuals for death rates modeled with 2D smoothing with P−splines.

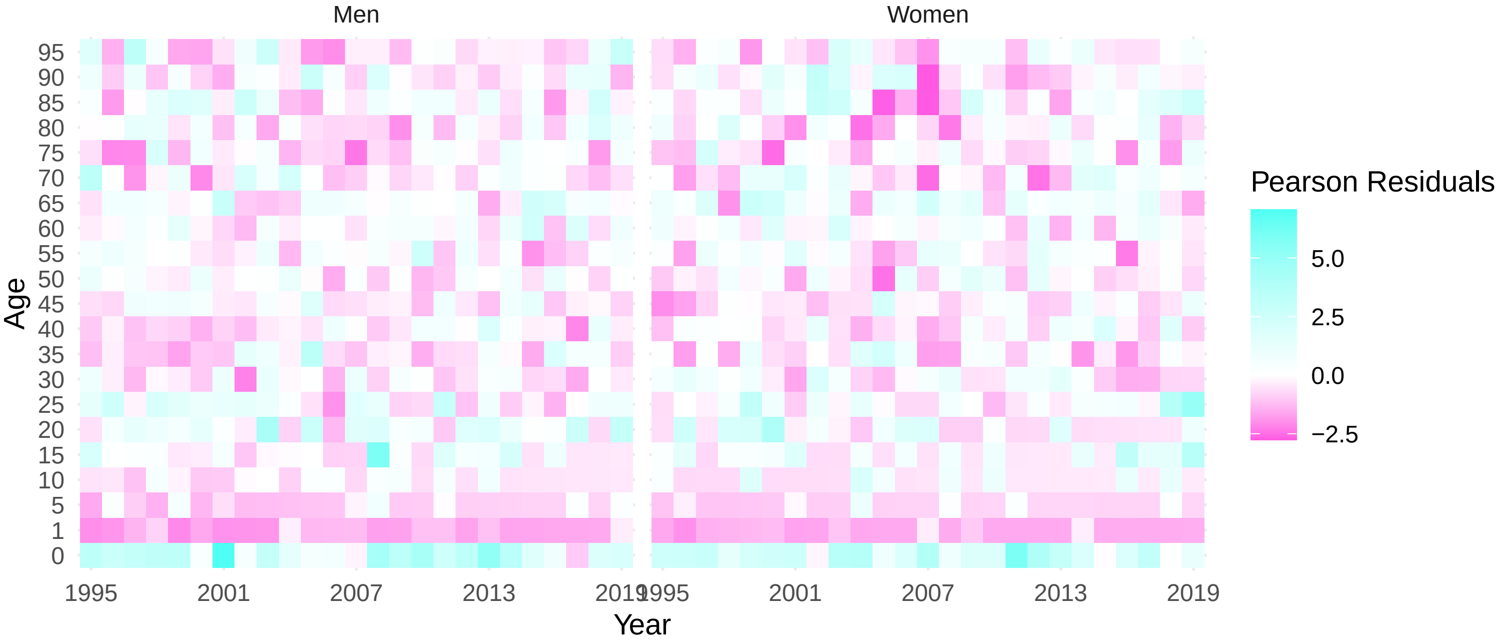

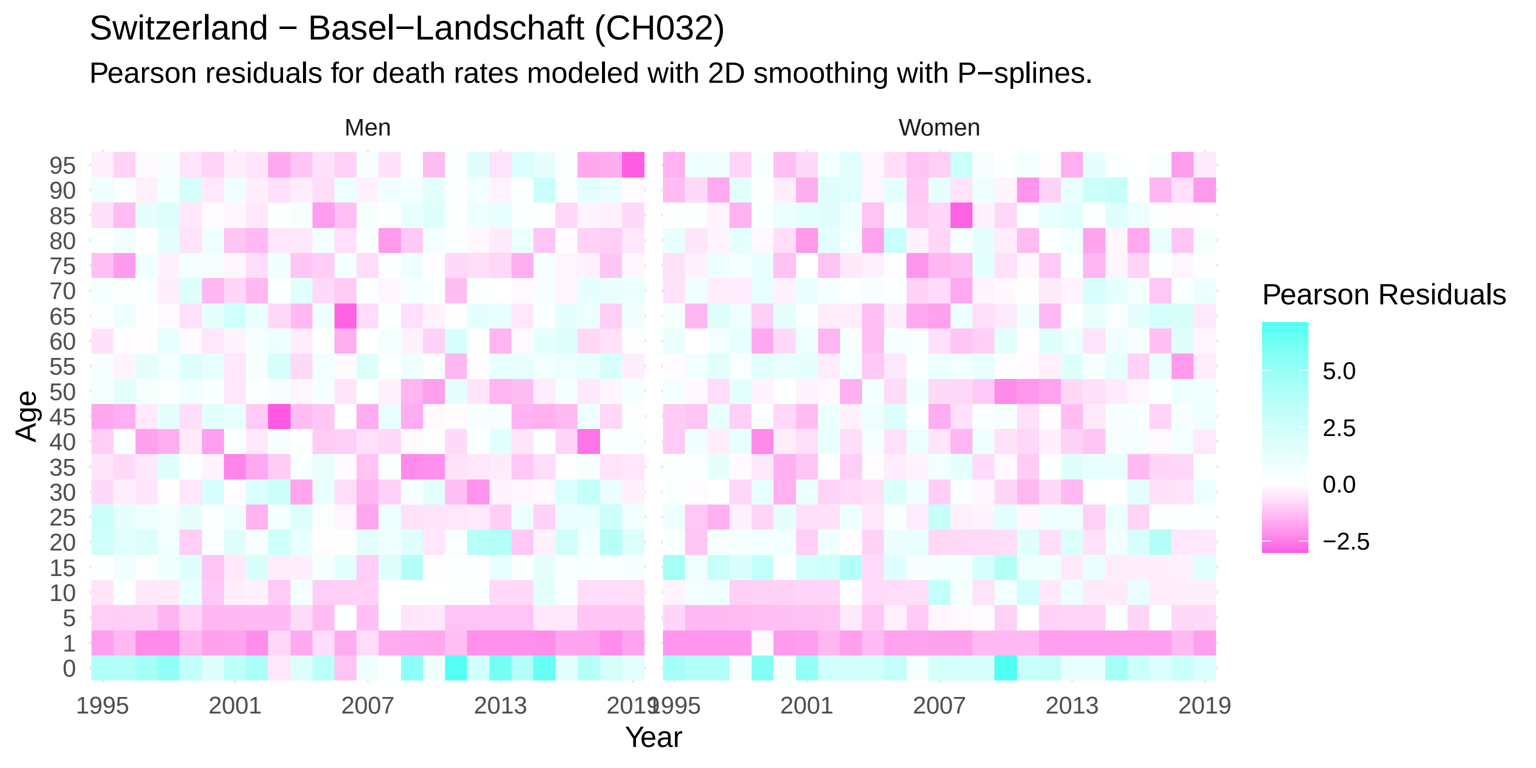
Switzerland − Basel−Landschaft (CH032)
Pearson residuals for death rates modeled with 2D smoothing with P−splines.
Men
Women
Age
95
90
85
80
75
70
65
60
55
50
45
40
35
30
25
20
15
10
5
1
0
1995
2001
2007
2013
2019
Year
Pearson Residuals
5.0
2.5
0.0
−2.5

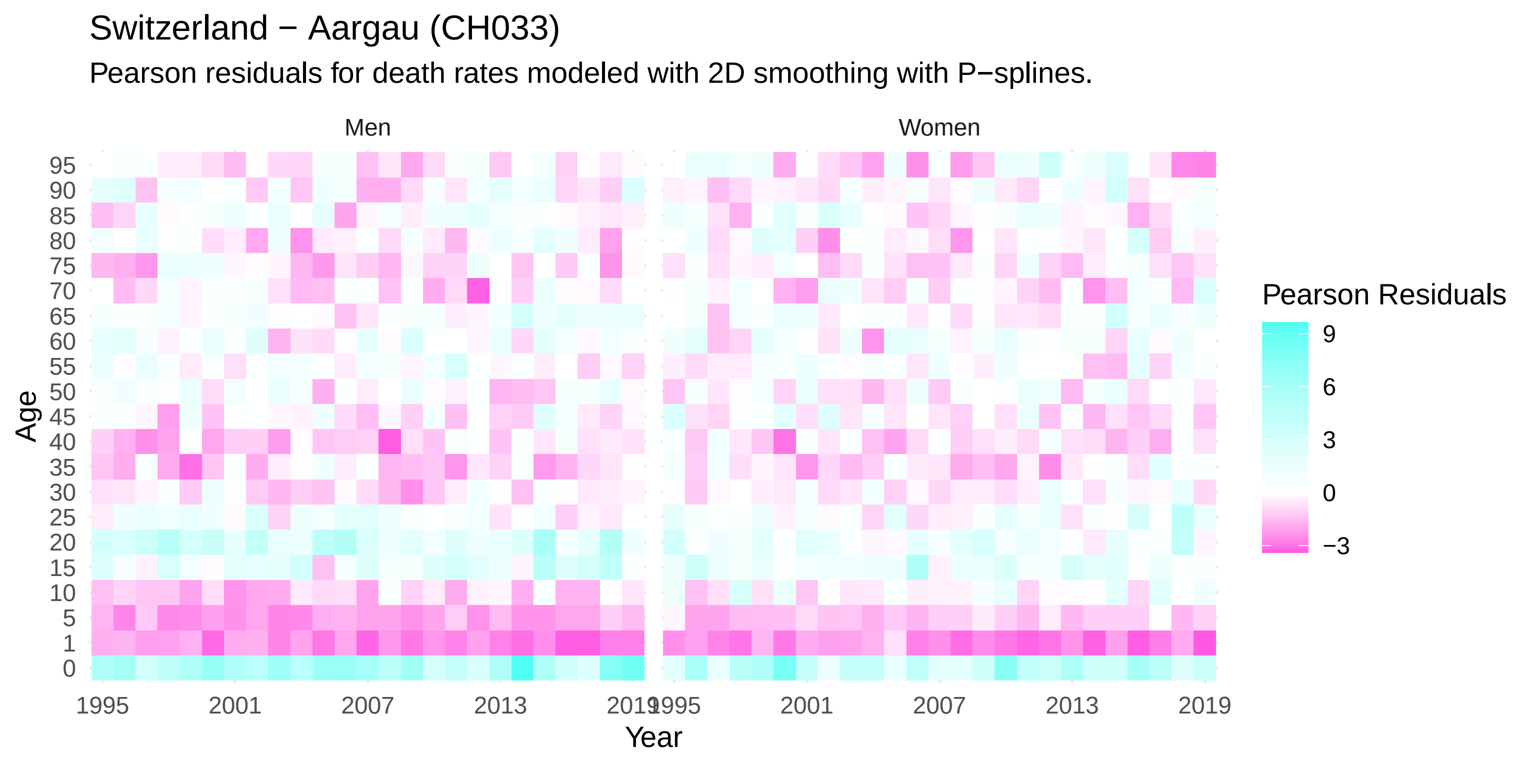

Switzerland − Aargau (CH033)
Pearson residuals for death rates modeled with 2D smoothing with P−splines.
Men
Women
Age
95
90
85
80
75
70
65
60
55
50
45
40
35
30
25
20
15
10
5
1
0
1995
2001
2007
2013
2019
Year
Pearson Residuals
9
6
3
0
−3

Switzerland − Zürich (CH040)

Pearson residuals for death rates modeled with 2D smoothing with P−splines.

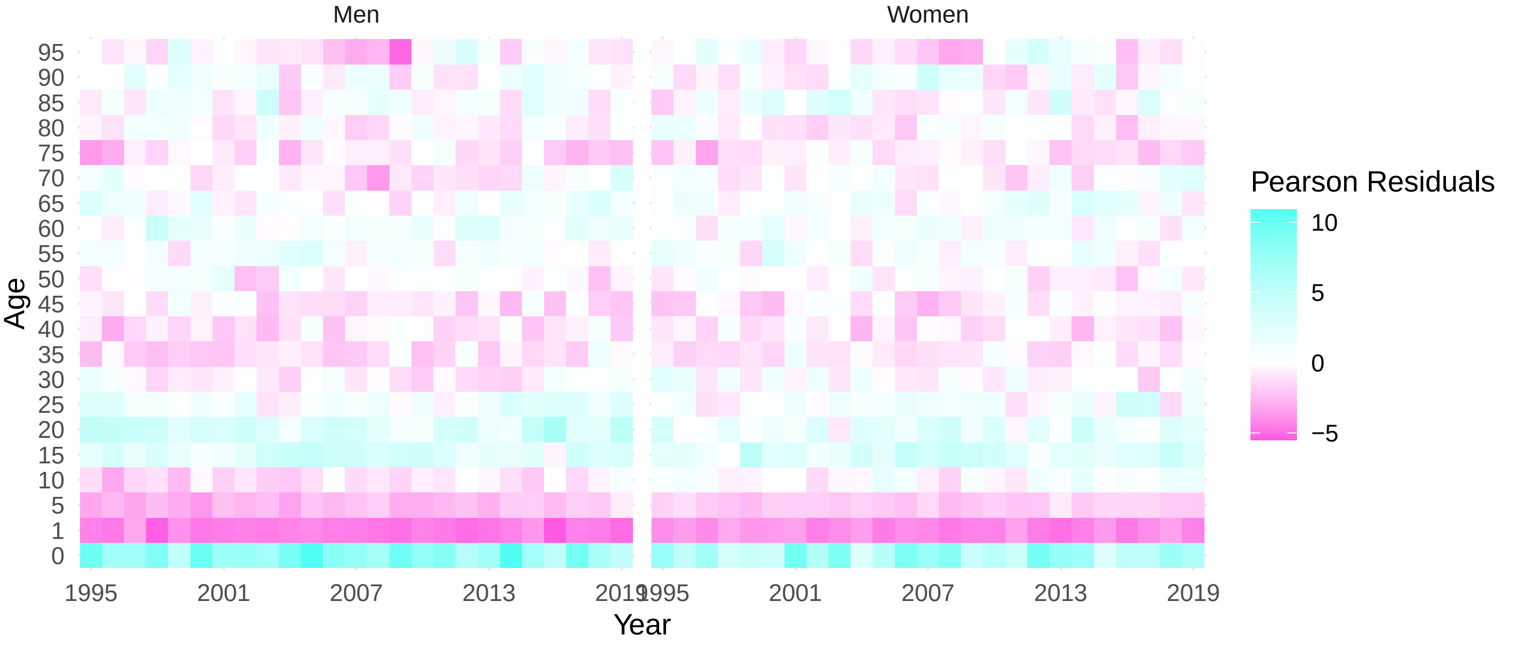

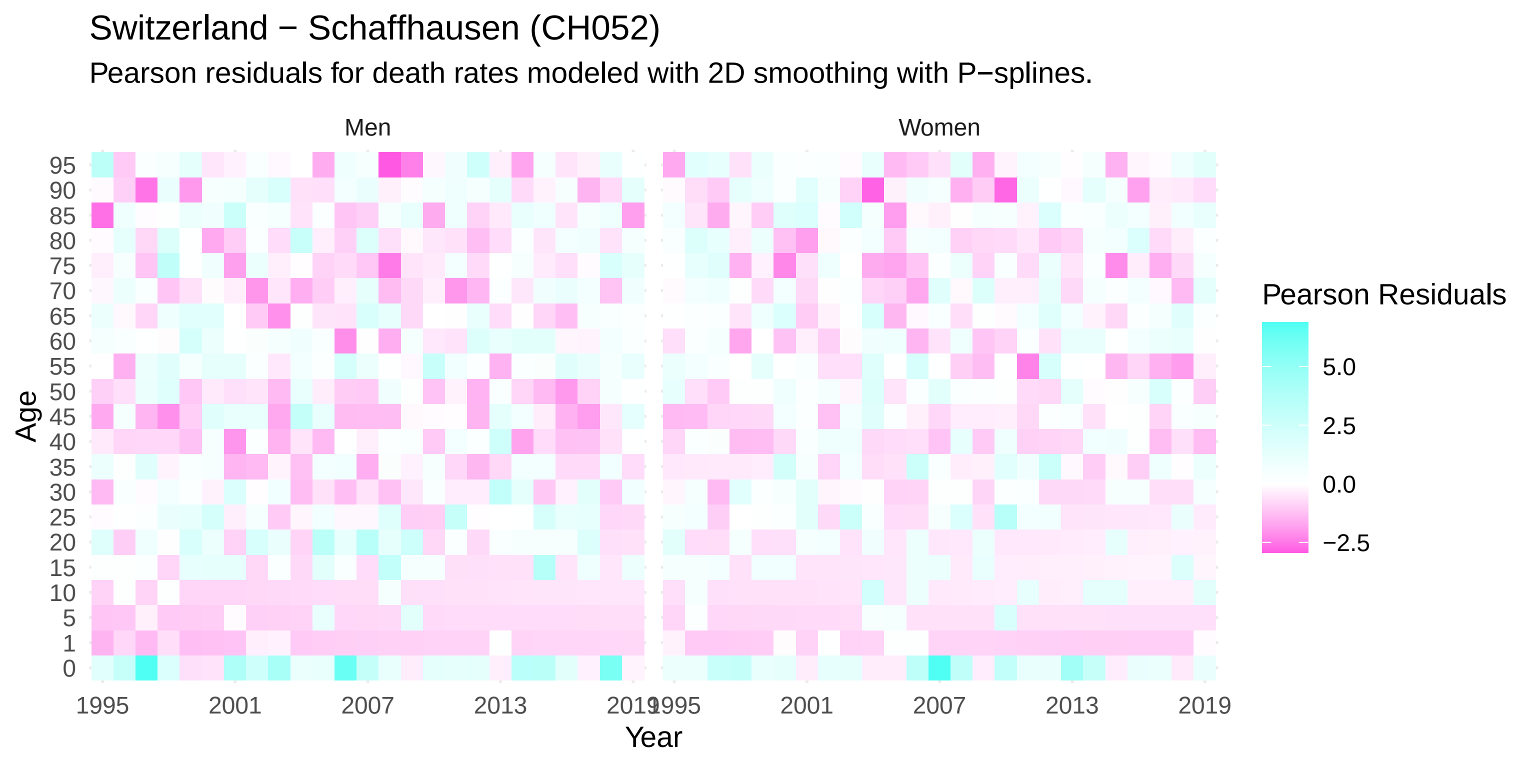
Switzerland − Schaffhausen (CH052)
Pearson residuals for death rates modeled with 2D smoothing with P−splines.
Men
Women
Age
95
90
85
80
75
70
65
60
55
50
45
40
35
30
25
20
15
10
5
1
0
1995
2001
2007
2013
2019
Year
Pearson Residuals
5.0
2.5
0.0
−2.5

Switzerland − Appenzell Ausserrhoden (CH053)

Pearson residuals for death rates modeled with 2D smoothing with P−splines.

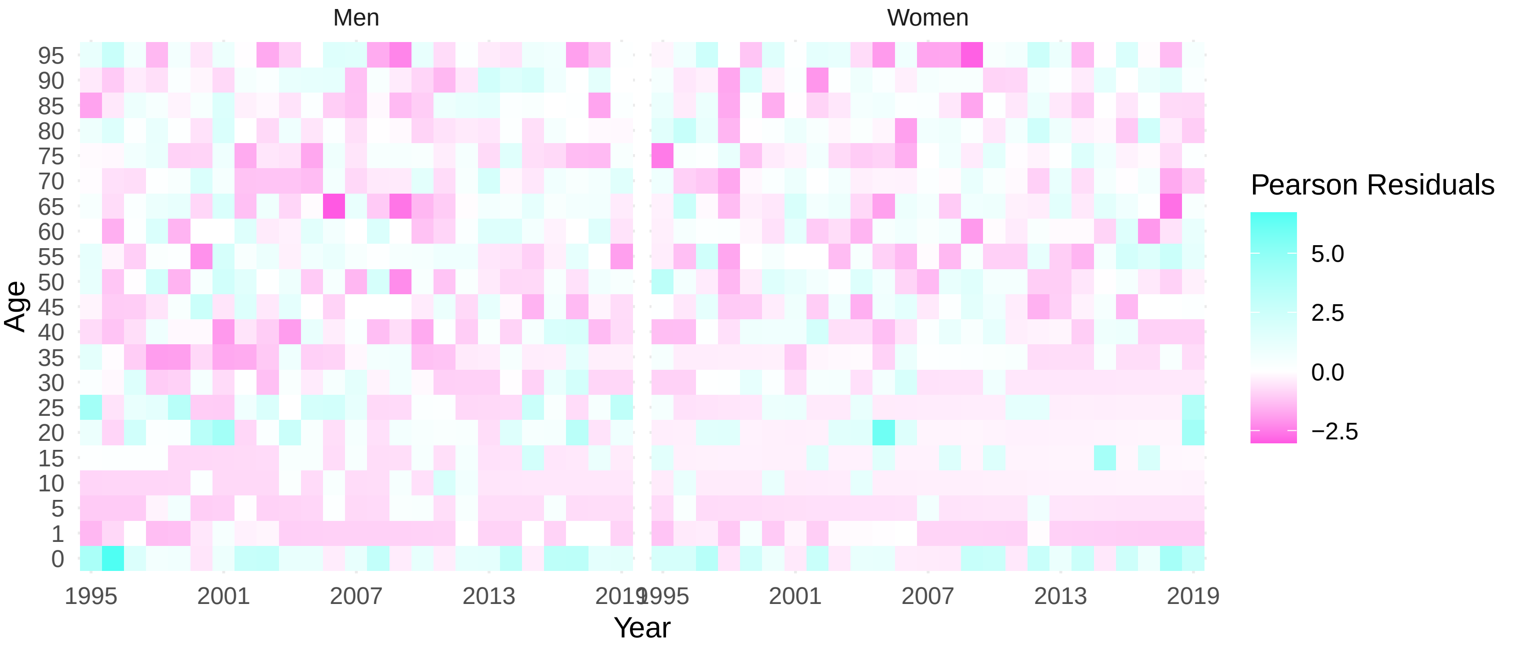

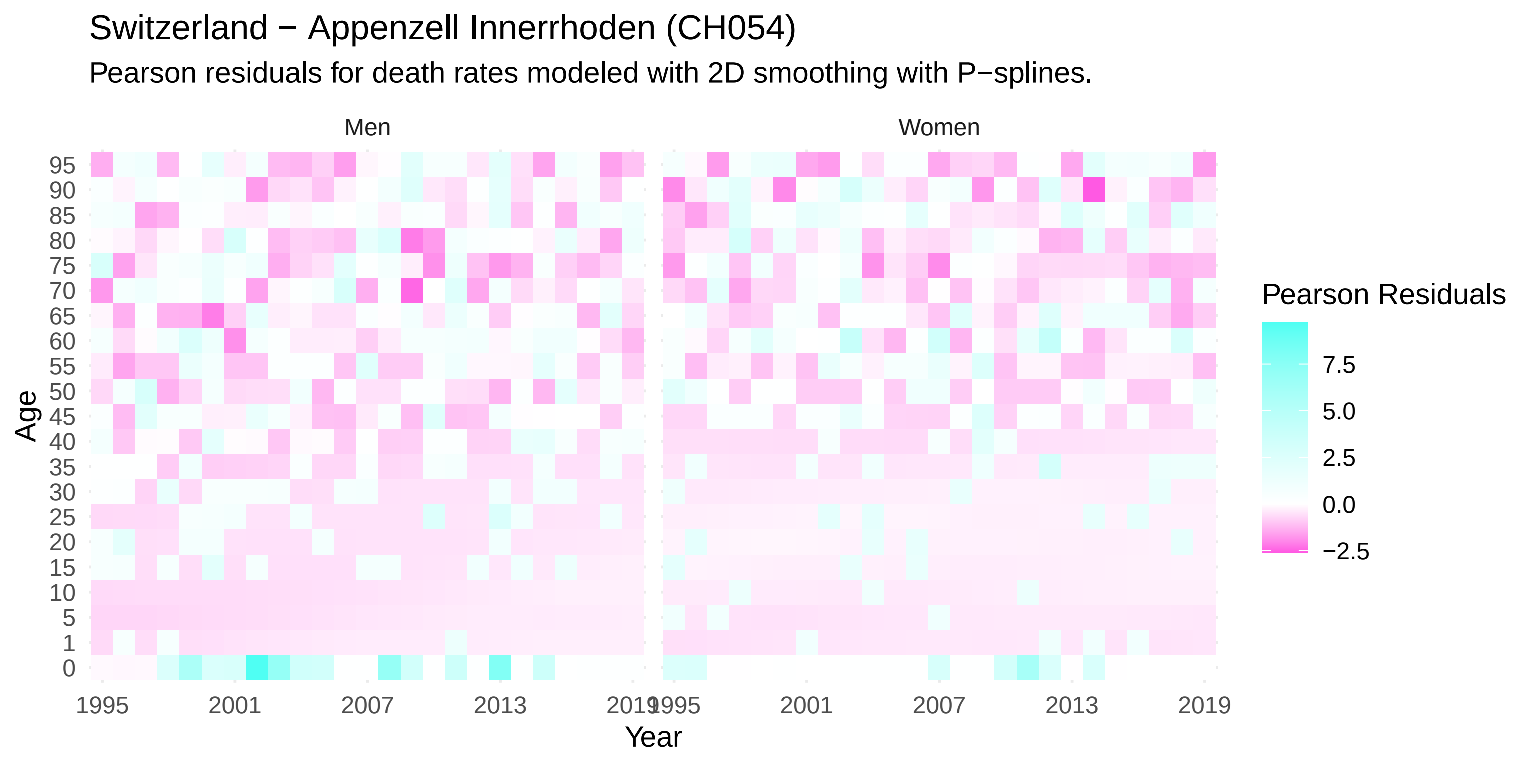
Switzerland − Appenzell Innerrhoden (CH054)
Pearson residuals for death rates modeled with 2D smoothing with P−splines.
Men
Women
Age
95
90
85
80
75
70
65
60
55
50
45
40
35
30
25
20
15
10
5
1
0
1995
2001
2007
2013
2019
Year
Pearson Residuals
7.5
5.0
2.5
0.0
−2.5

Switzerland − St. Gallen (CH055)

Pearson residuals for death rates modeled with 2D smoothing with P−splines.

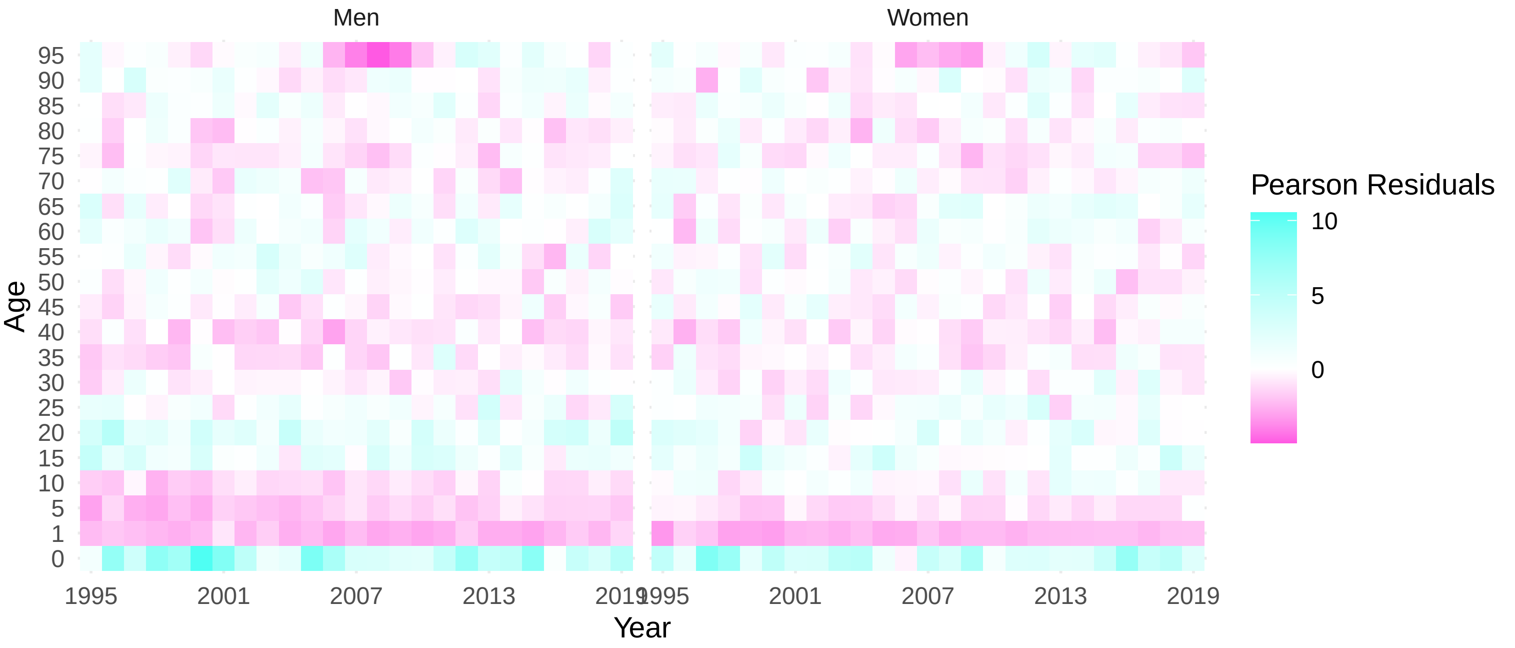

Switzerland − Graubünden (CH056)

Pearson residuals for death rates modeled with 2D smoothing with P−splines.

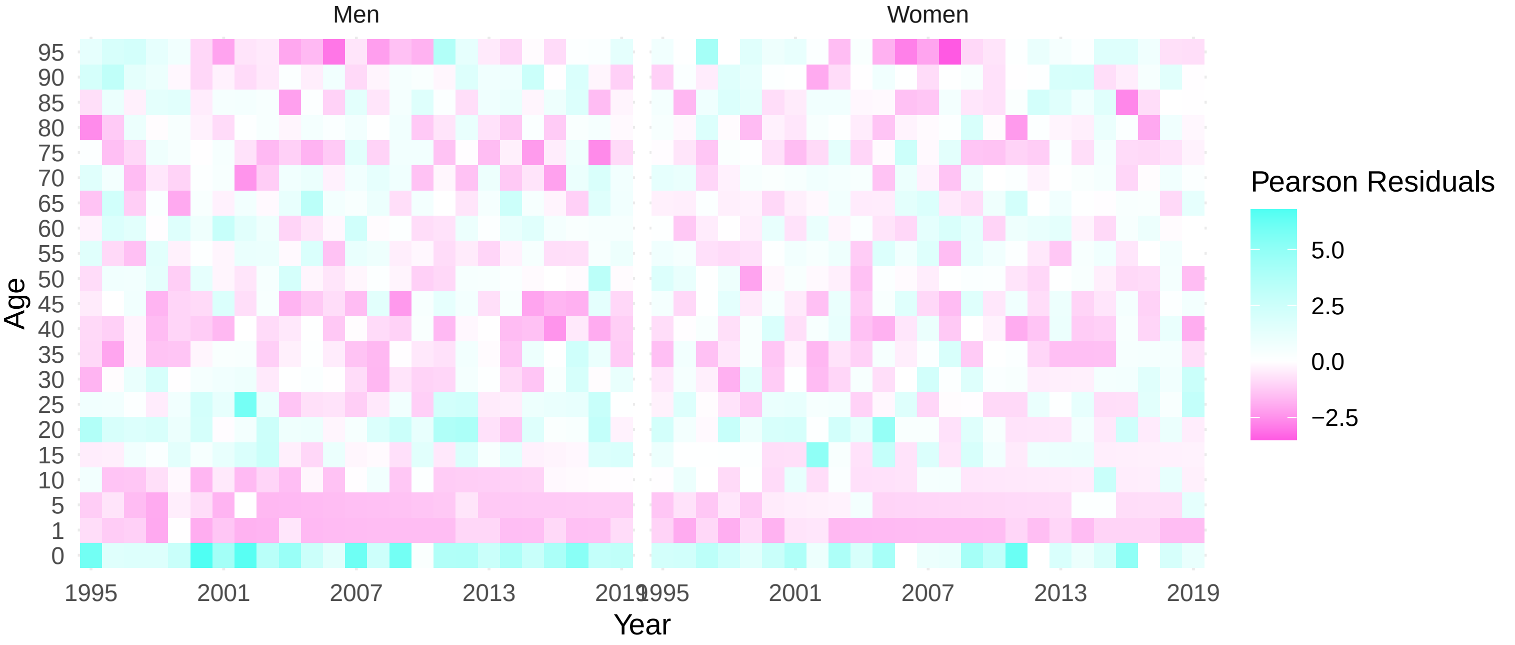

Switzerland − Thurgau (CH057)

Pearson residuals for death rates modeled with 2D smoothing with P−splines.

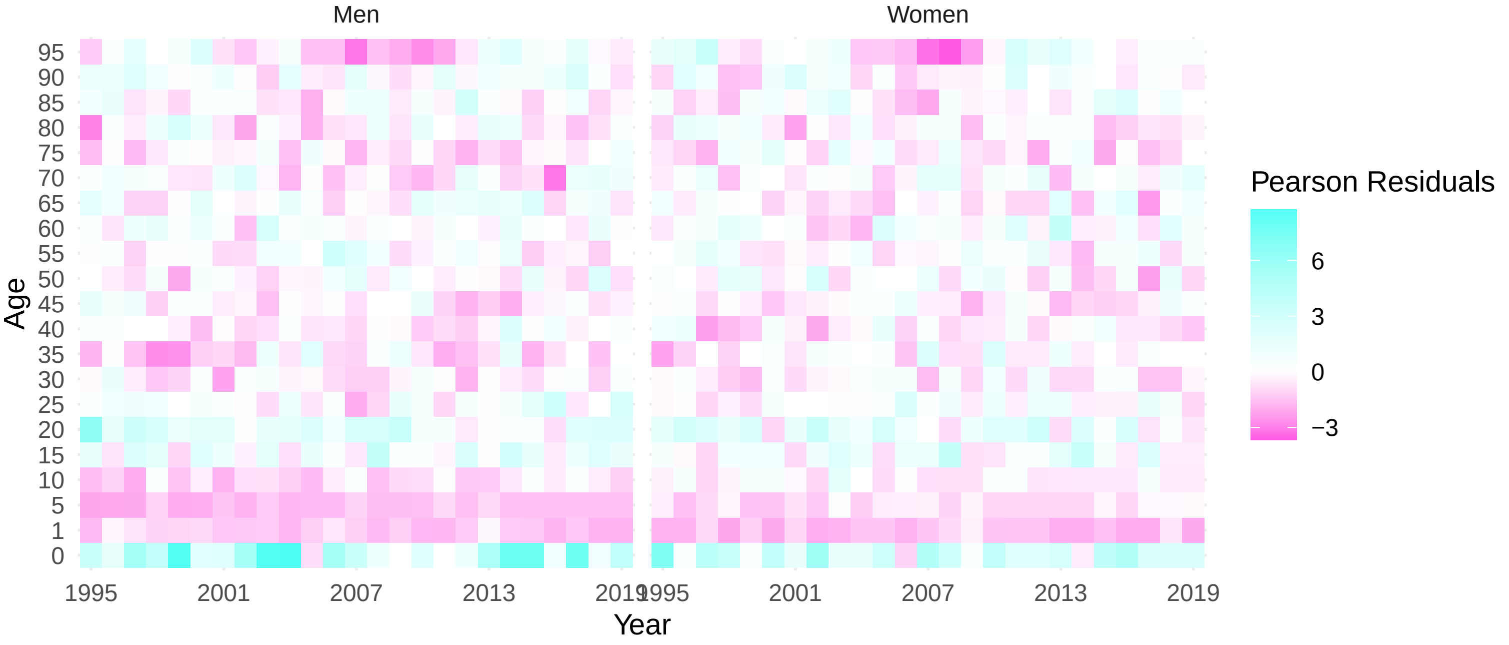

Switzerland − Ticino (CH070)

Pearson residuals for death rates modeled with 2D smoothing with P−splines.

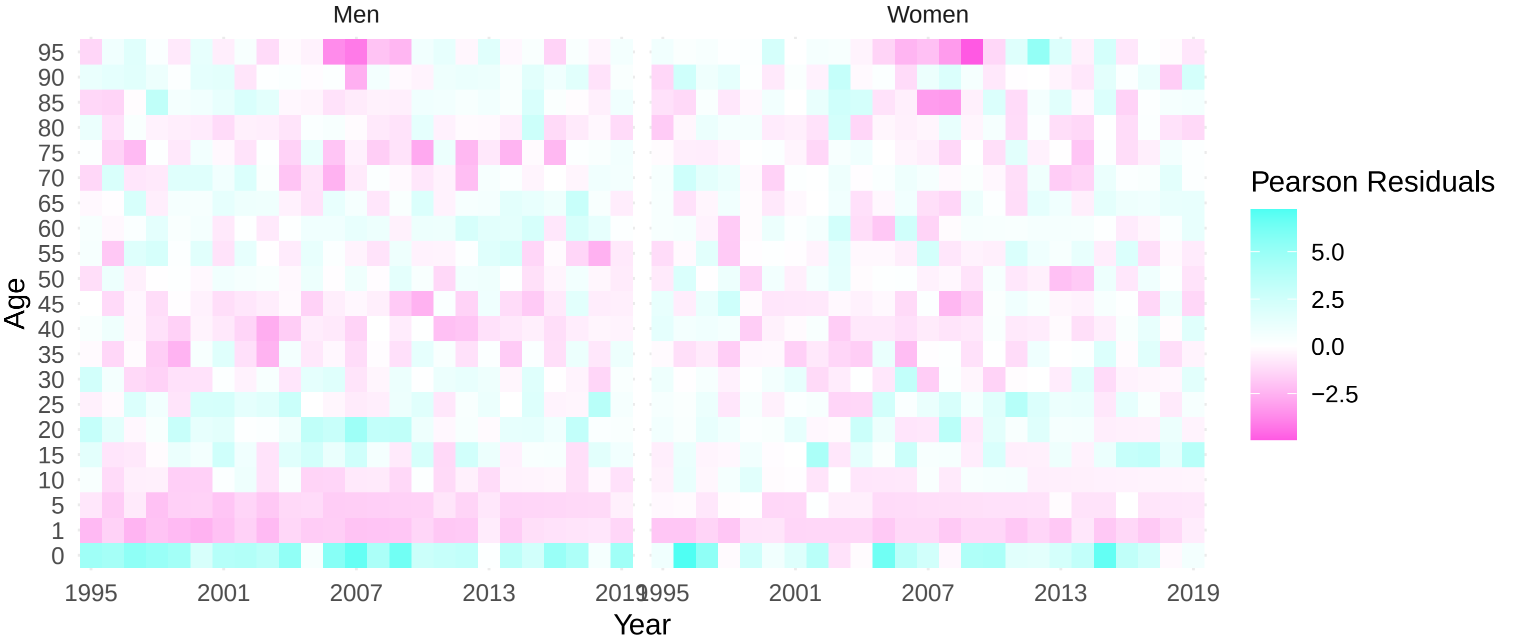

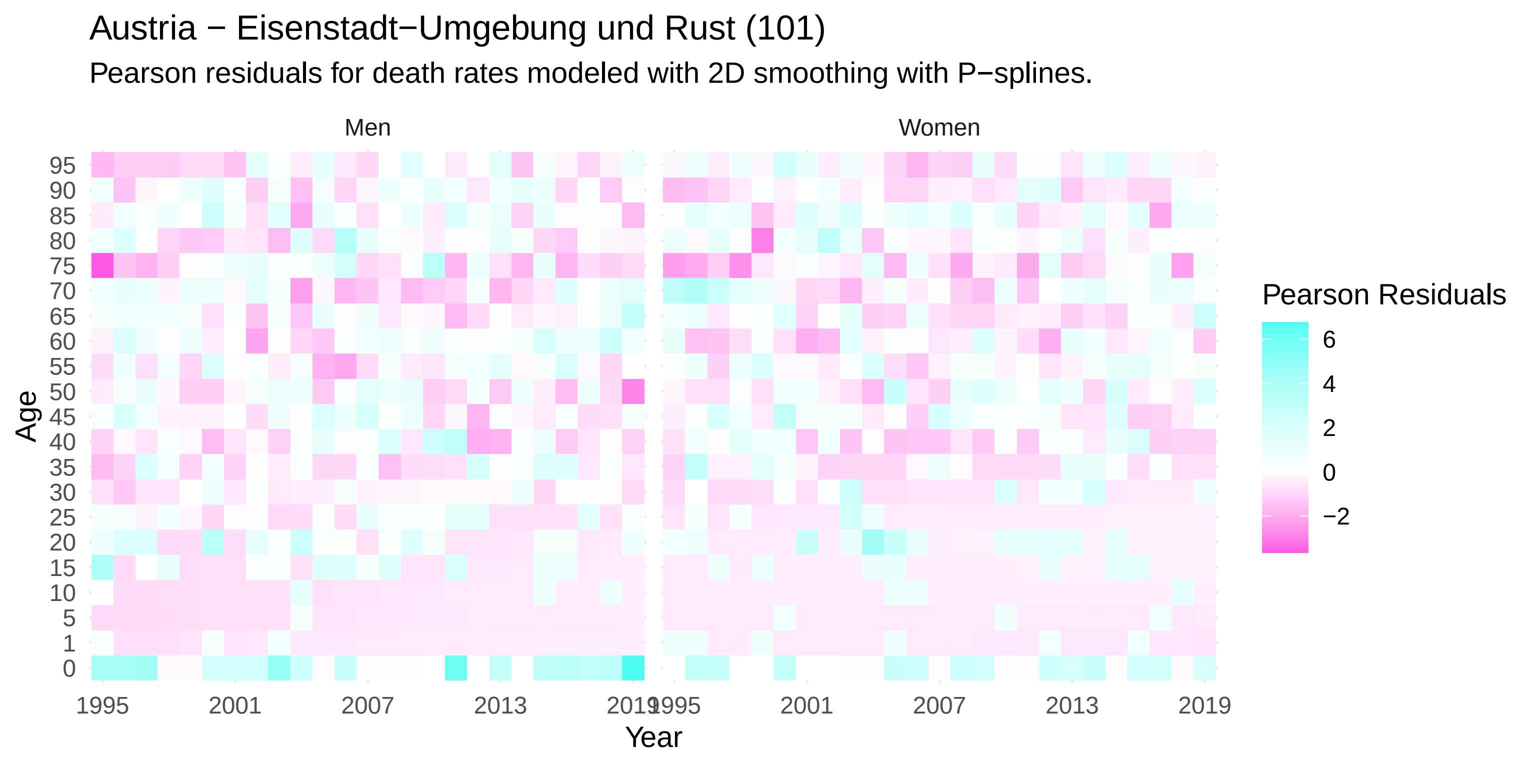

Austria − Eisenstadt−Umgebung und Rust (101)
Pearson residuals for death rates modeled with 2D smoothing with P−splines.
Men
Women
Age
95
90
85
80
75
70
65
60
55
50
45
40
35
30
25
20
15
10
5
1
0
1995
2001
2007
2013
2019
Year
Pearson Residuals
6
4
2
0
−2

Austria − Güssing (104)

Pearson residuals for death rates modeled with 2D smoothing with P−splines.

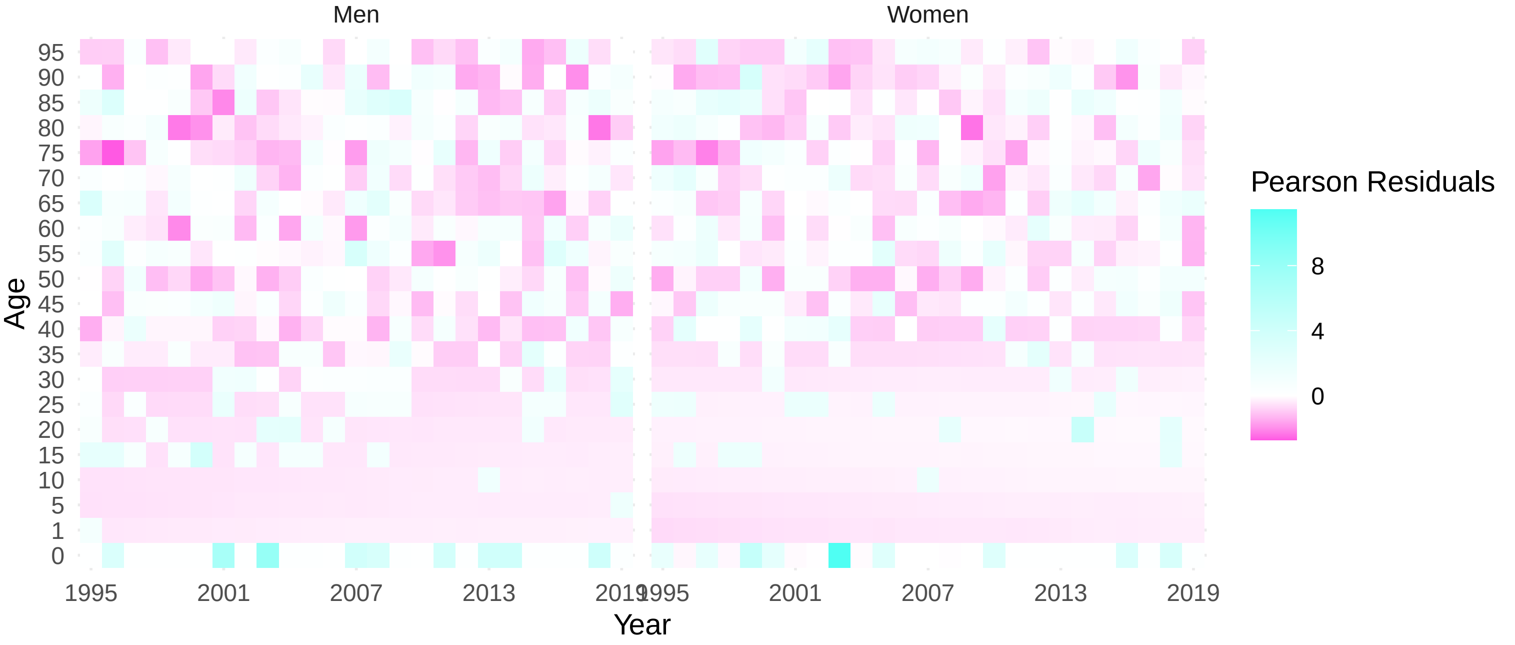

Austria − Jennersdorf (105)

Pearson residuals for death rates modeled with 2D smoothing with P−splines.

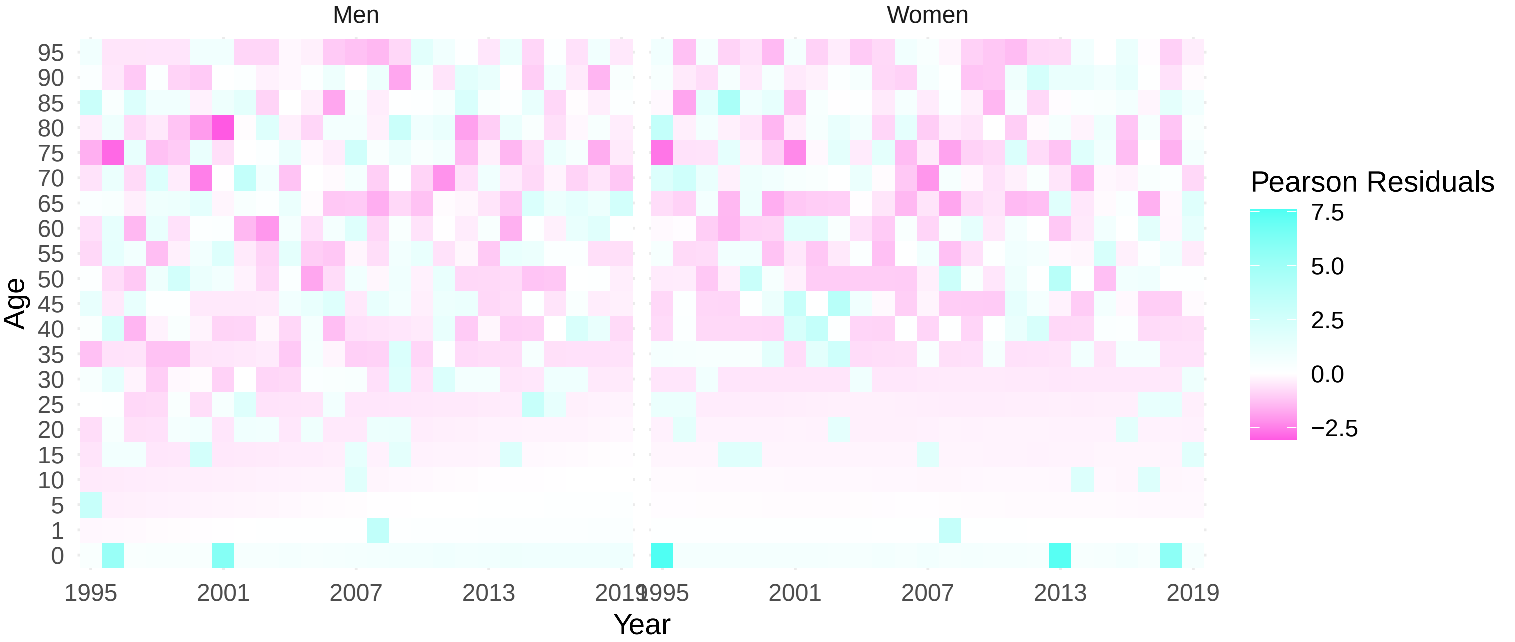

Austria − Mattersburg (106)

Pearson residuals for death rates modeled with 2D smoothing with P−splines.

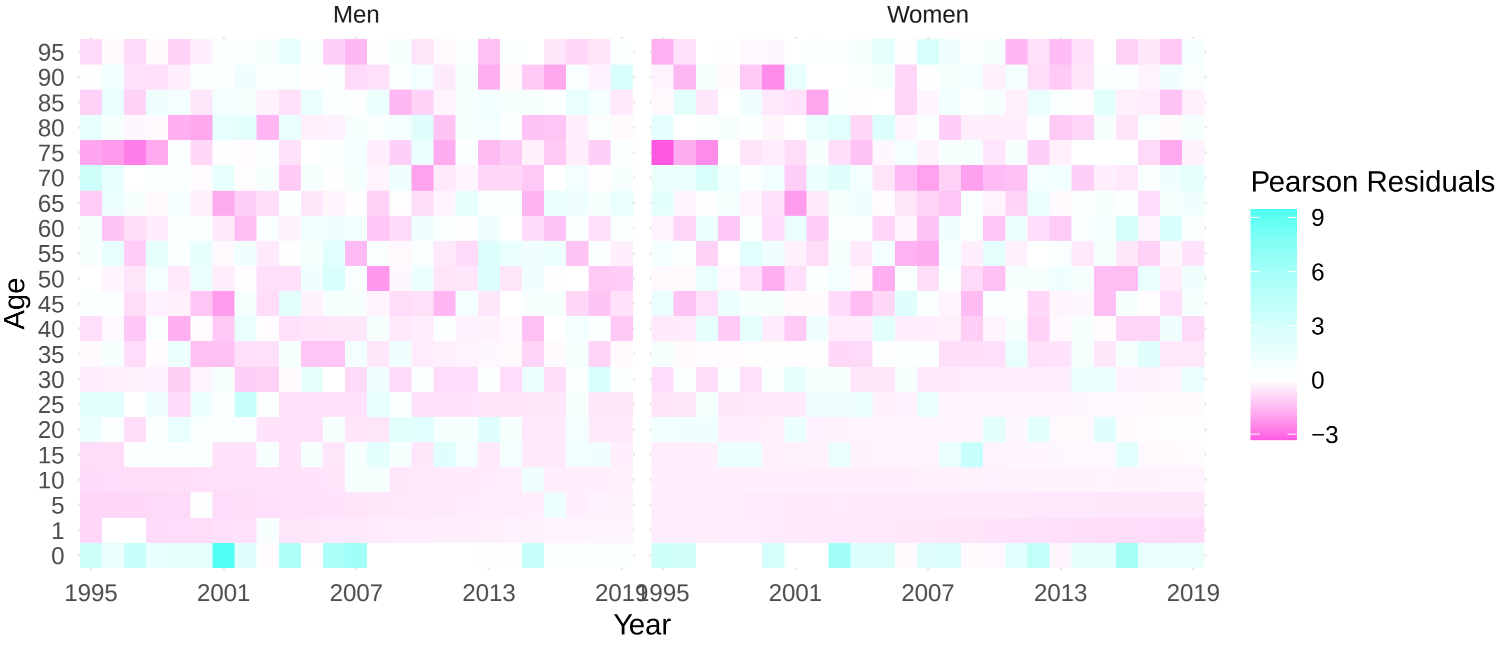

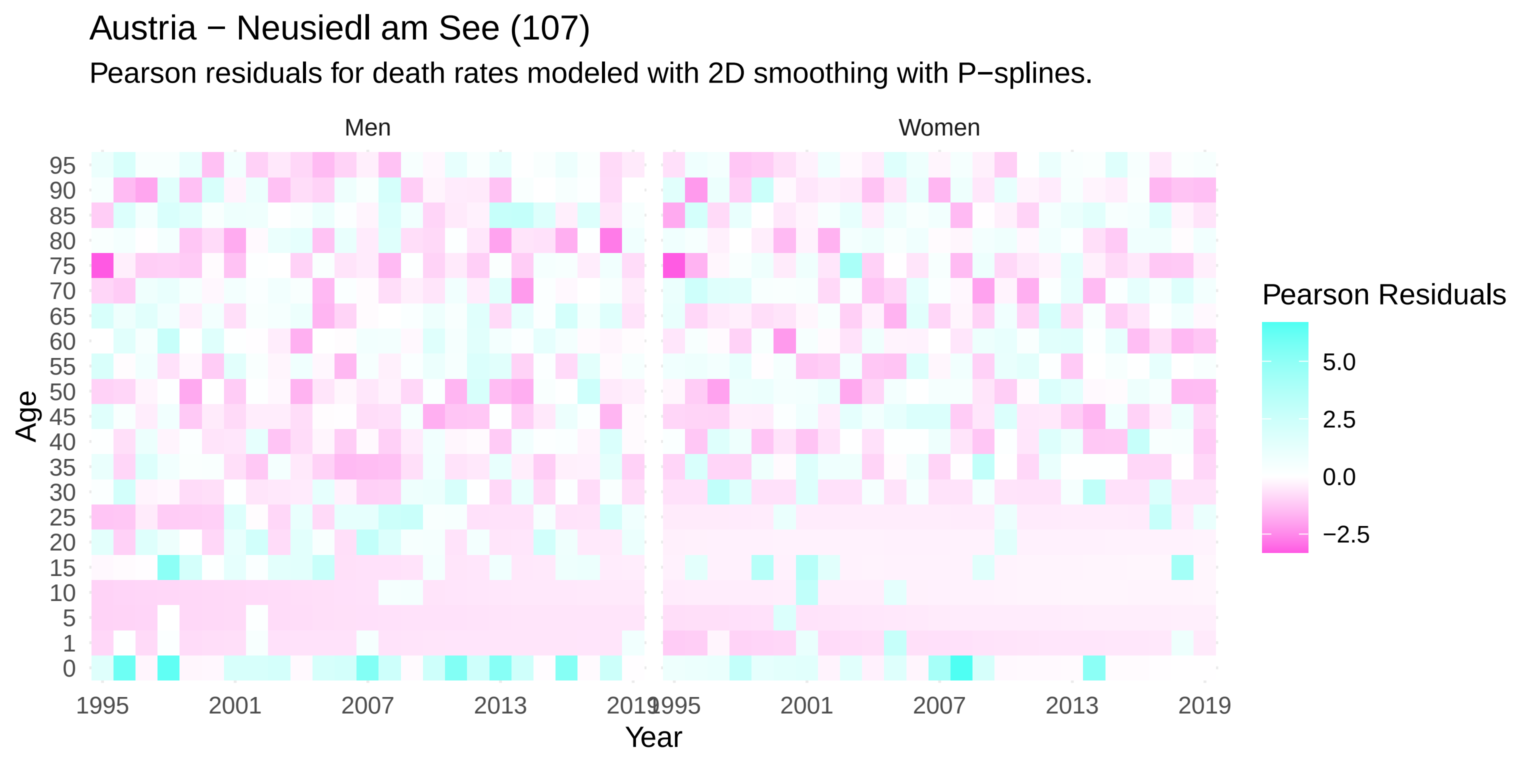

Austria − Neusiedl am See (107)
Pearson residuals for death rates modeled with 2D smoothing with P−splines.
Men
Women
Age
95
90
85
80
75
70
65
60
55
50
45
40
35
30
25
20
15
10
5
1
0
1995
2001
2007
2013
2019
Year
Pearson Residuals
5.0
2.5
0.0
−2.5

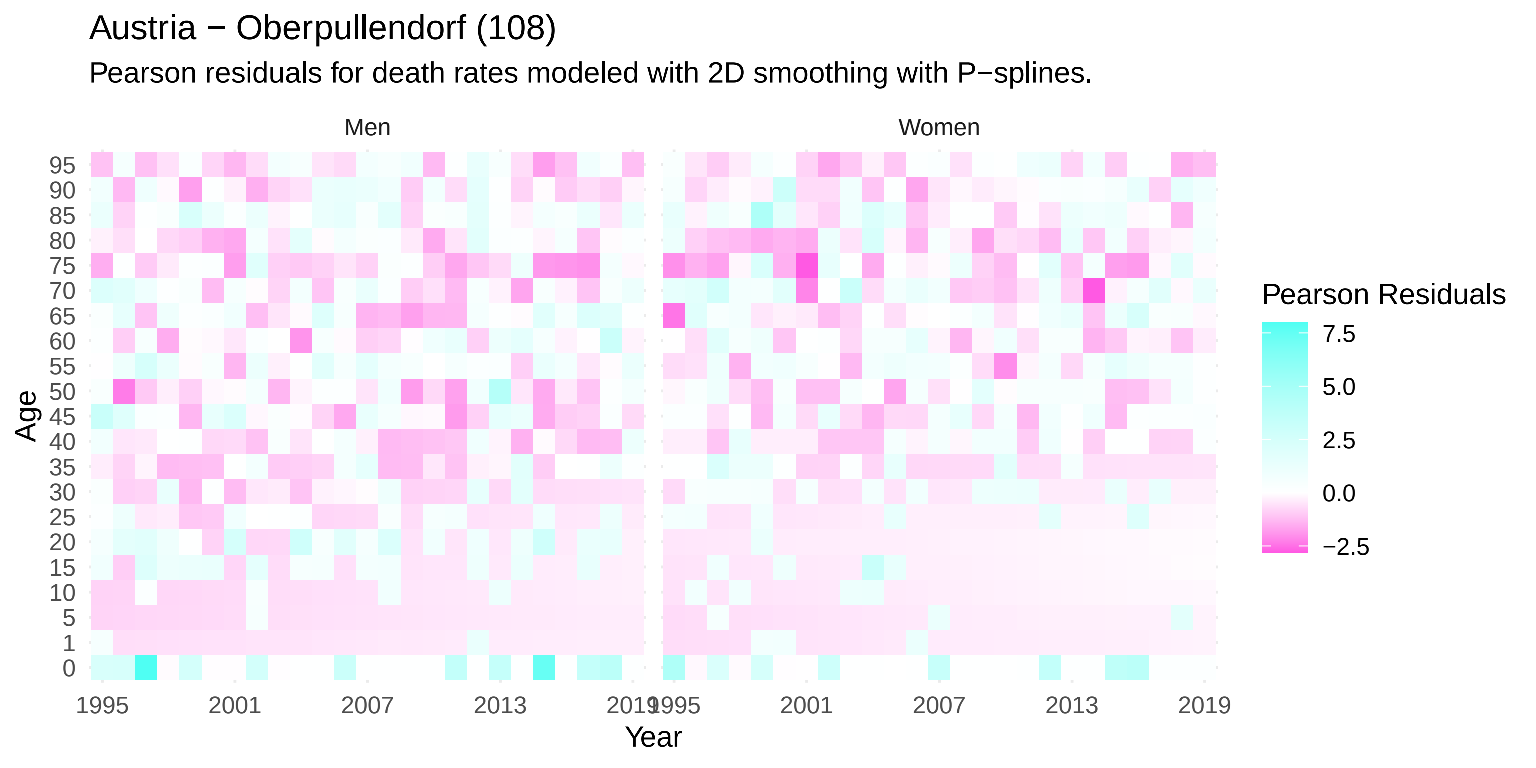

Austria − Oberpullendorf (108)
Pearson residuals for death rates modeled with 2D smoothing with P−splines.
Men
Women
Pearson Residuals
7.5
5.0
2.5
0.0
−2.5
Age
95
90
85
80
75
70
65
60
55
50
45
40
35
30
25
20
15
10
5
1
0
1995
2001
2007
2013
2019
Year

Austria − Oberwart (109)

Pearson residuals for death rates modeled with 2D smoothing with P−splines.

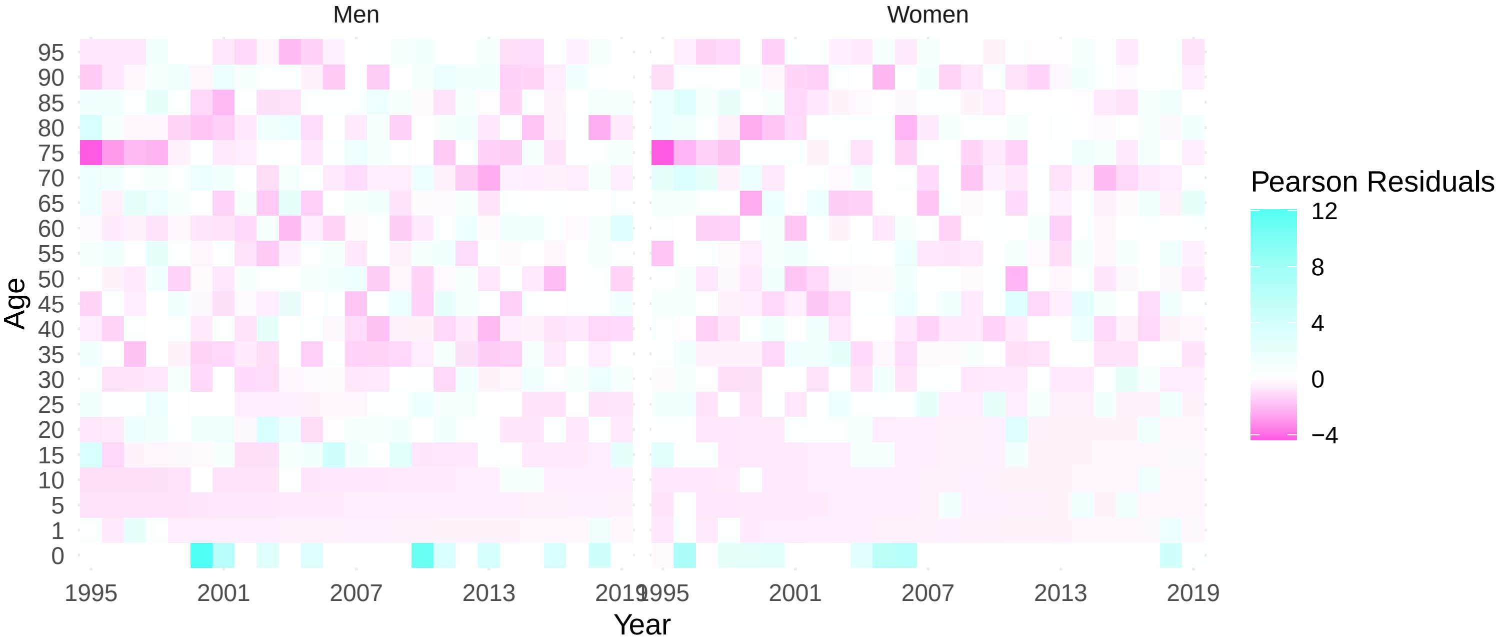

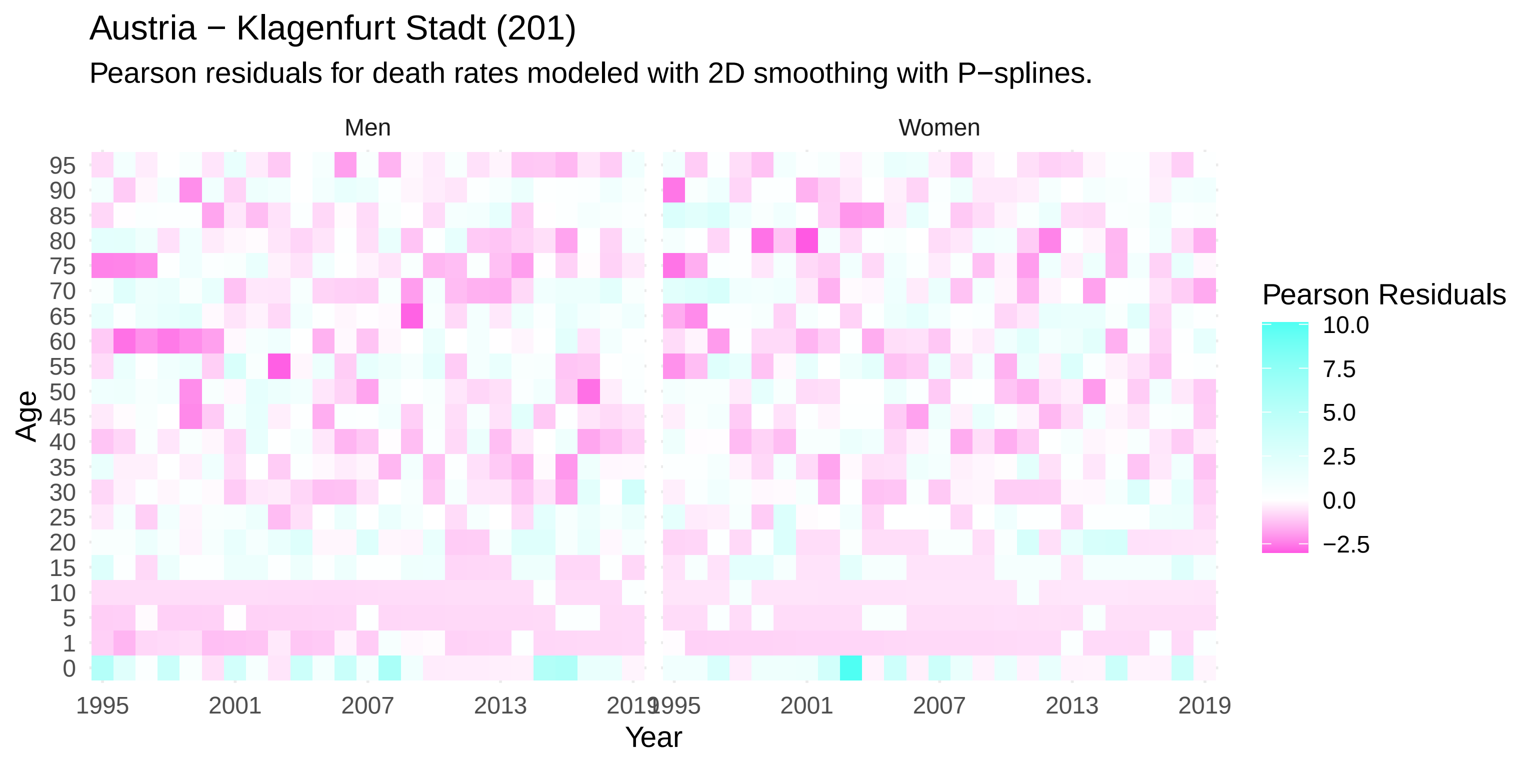

Austria − Klagenfurt Stadt (201)
Pearson residuals for death rates modeled with 2D smoothing with P−splines.
Men
Women
Age
95
90
85
80
75
70
65
60
55
50
45
40
35
30
25
20
15
10
5
1
0
1995
2001
2007
2013
2019
Year
Pearson Residuals
10.0
7.5
5.0
2.5
0.0
−2.5

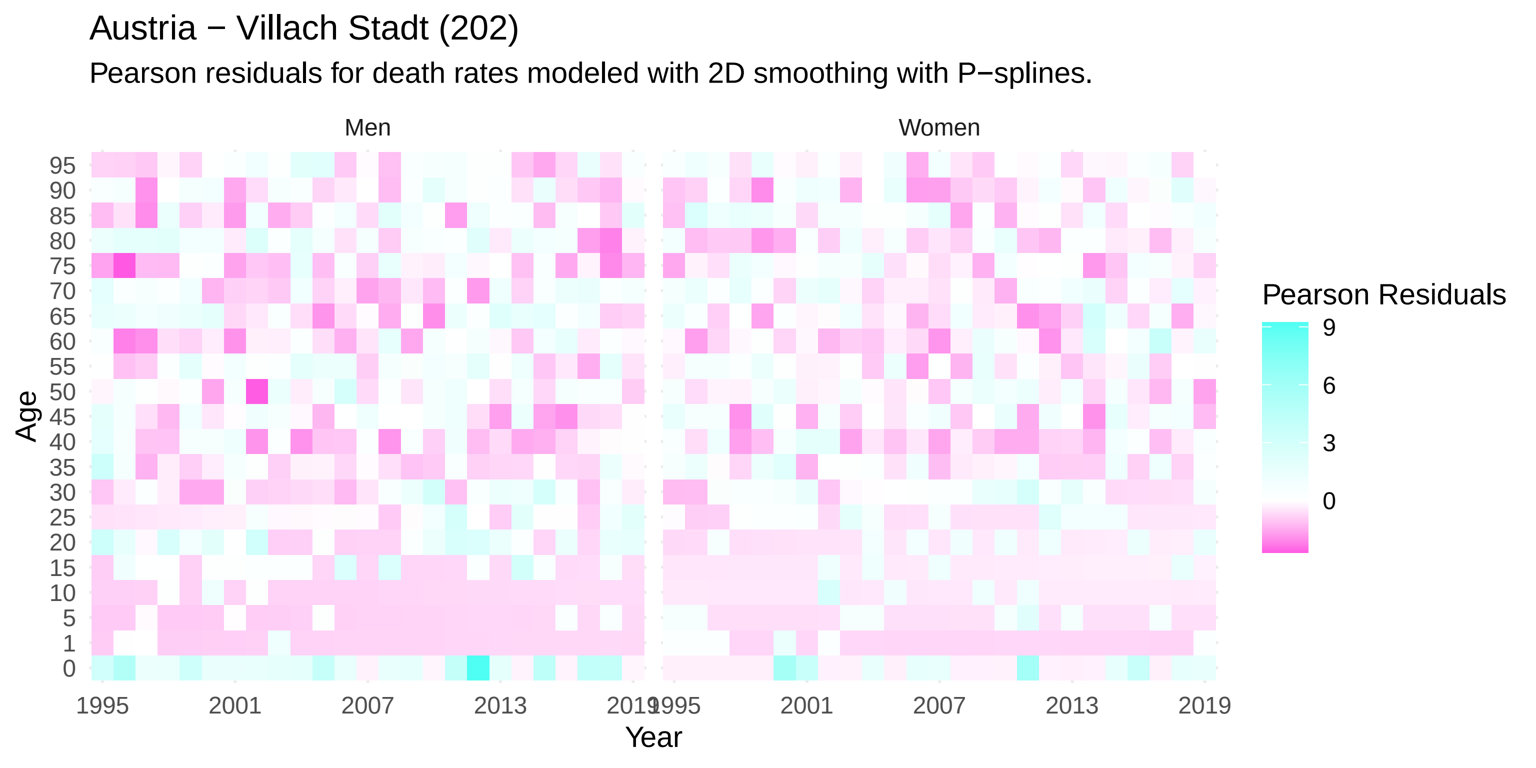

Austria − Villach Stadt (202)
Pearson residuals for death rates modeled with 2D smoothing with P−splines.
Men
Women
Age
95
90
85
80
75
70
65
60
55
50
45
40
35
30
25
20
15
10
5
1
0
1995
2001
2007
2013
2019
Year
Pearson Residuals
9
6
3
0

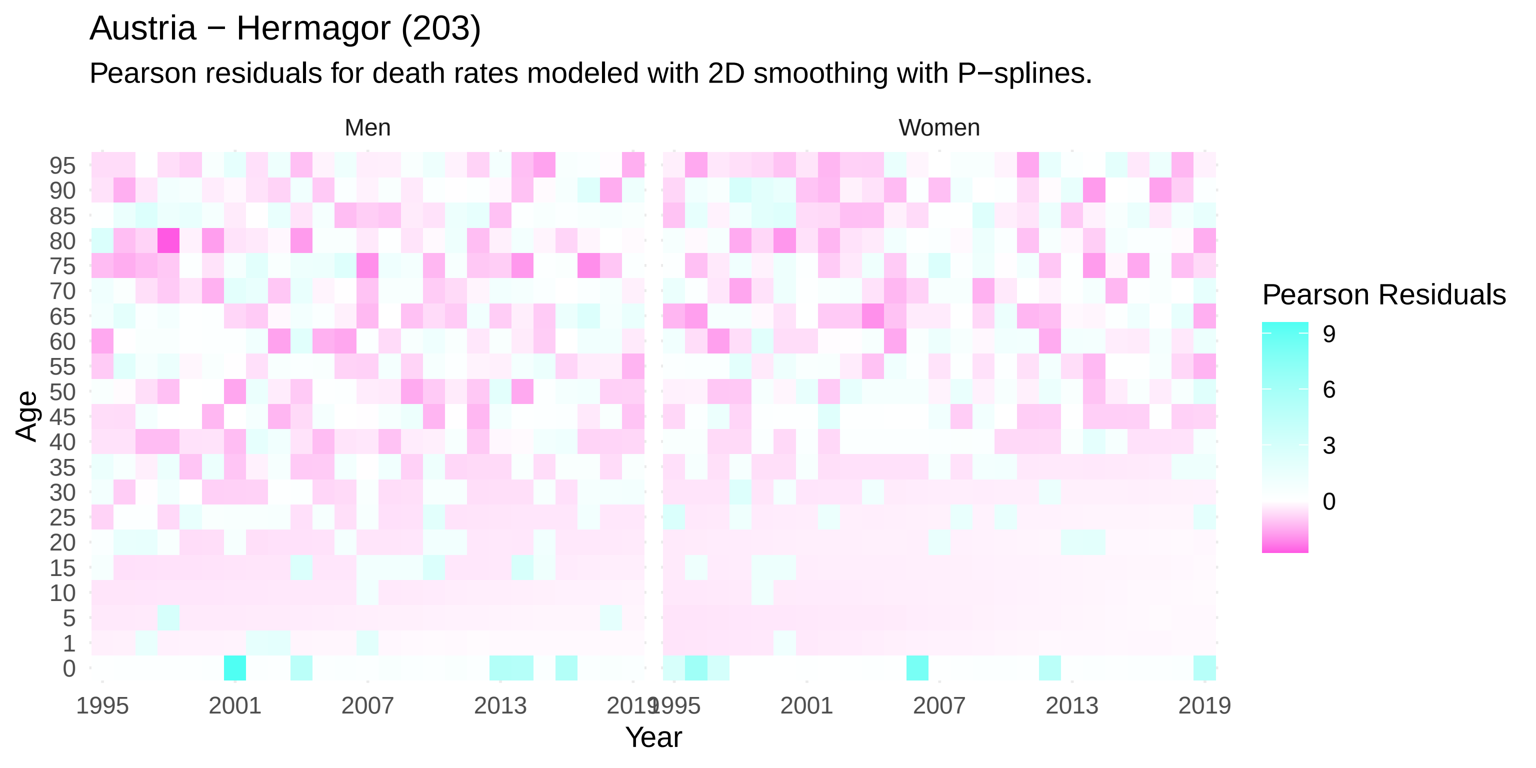

Austria − Hermagor (203)
Pearson residuals for death rates modeled with 2D smoothing with P−splines.
Men
Women
Age
95
90
85
80
75
70
65
60
55
50
45
40
35
30
25
20
15
10
5
1
0
1995
2001
2007
2013
2019
Year
Pearson Residuals
9
6
3
0

Austria − Klagenfurt Land (204)

Pearson residuals for death rates modeled with 2D smoothing with P−splines.

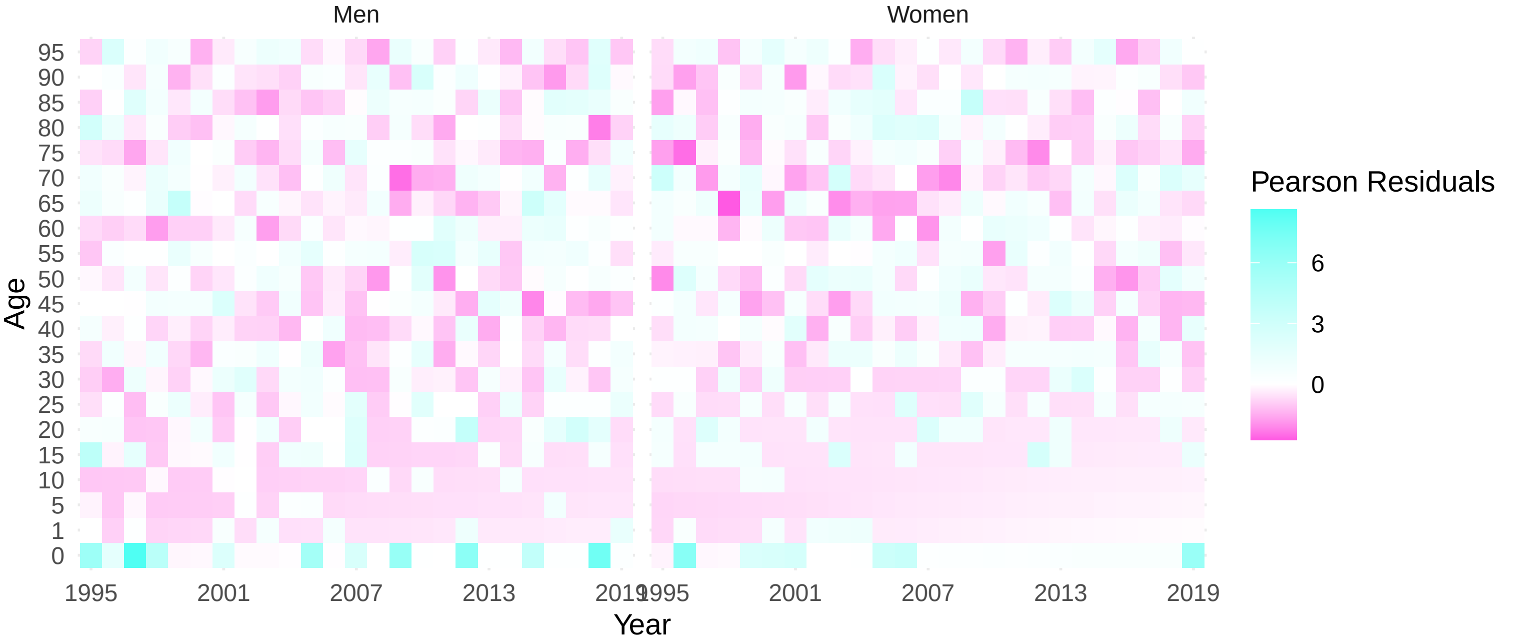

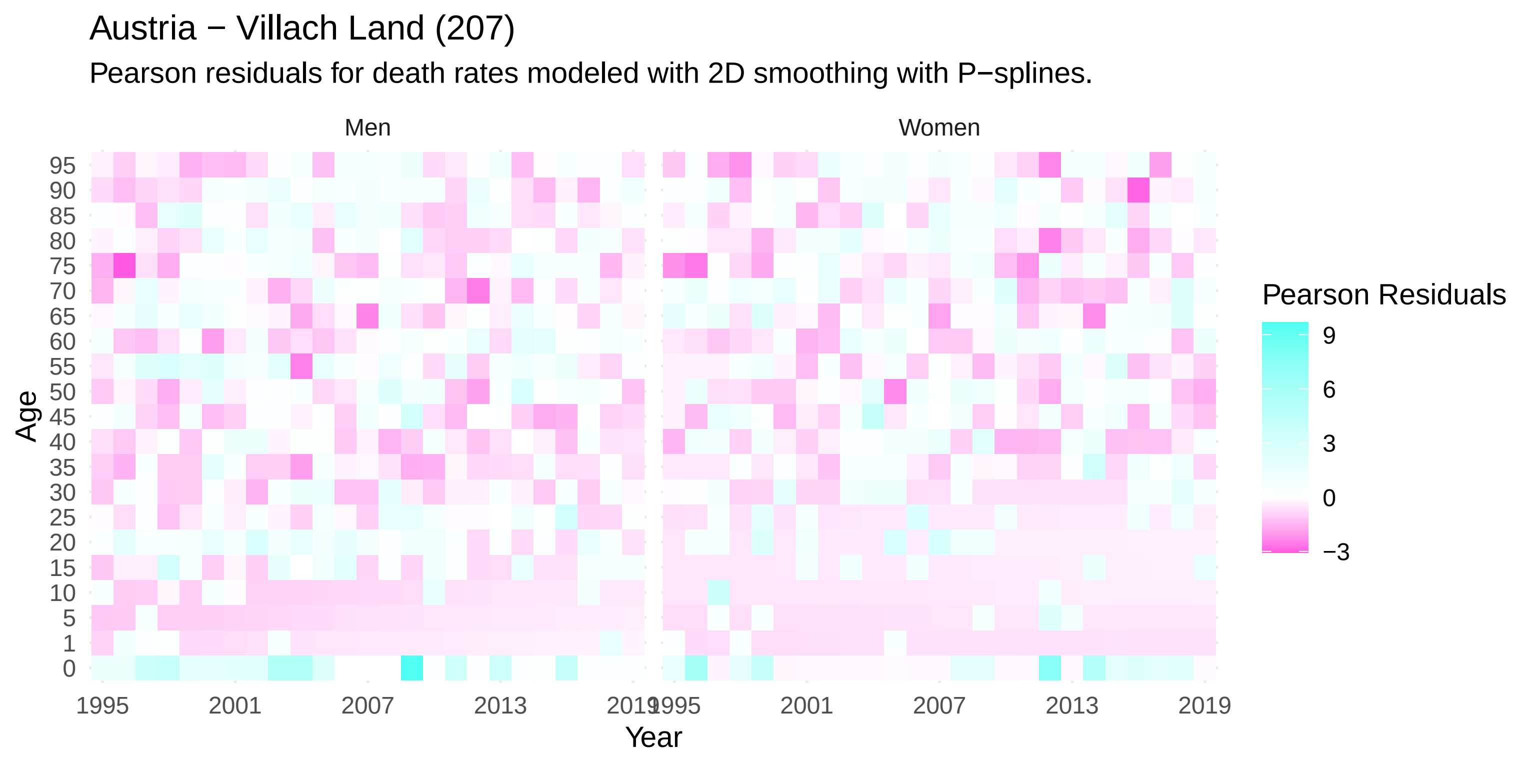

Austria − Villach Land (207)
Pearson residuals for death rates modeled with 2D smoothing with P−splines.
Men
Women
Age
95
90
85
80
75
70
65
60
55
50
45
40
35
30
25
20
15
10
5
1
0
1995
2001
2007
2013
2019
Year
Pearson Residuals
9
6
3
0
−3

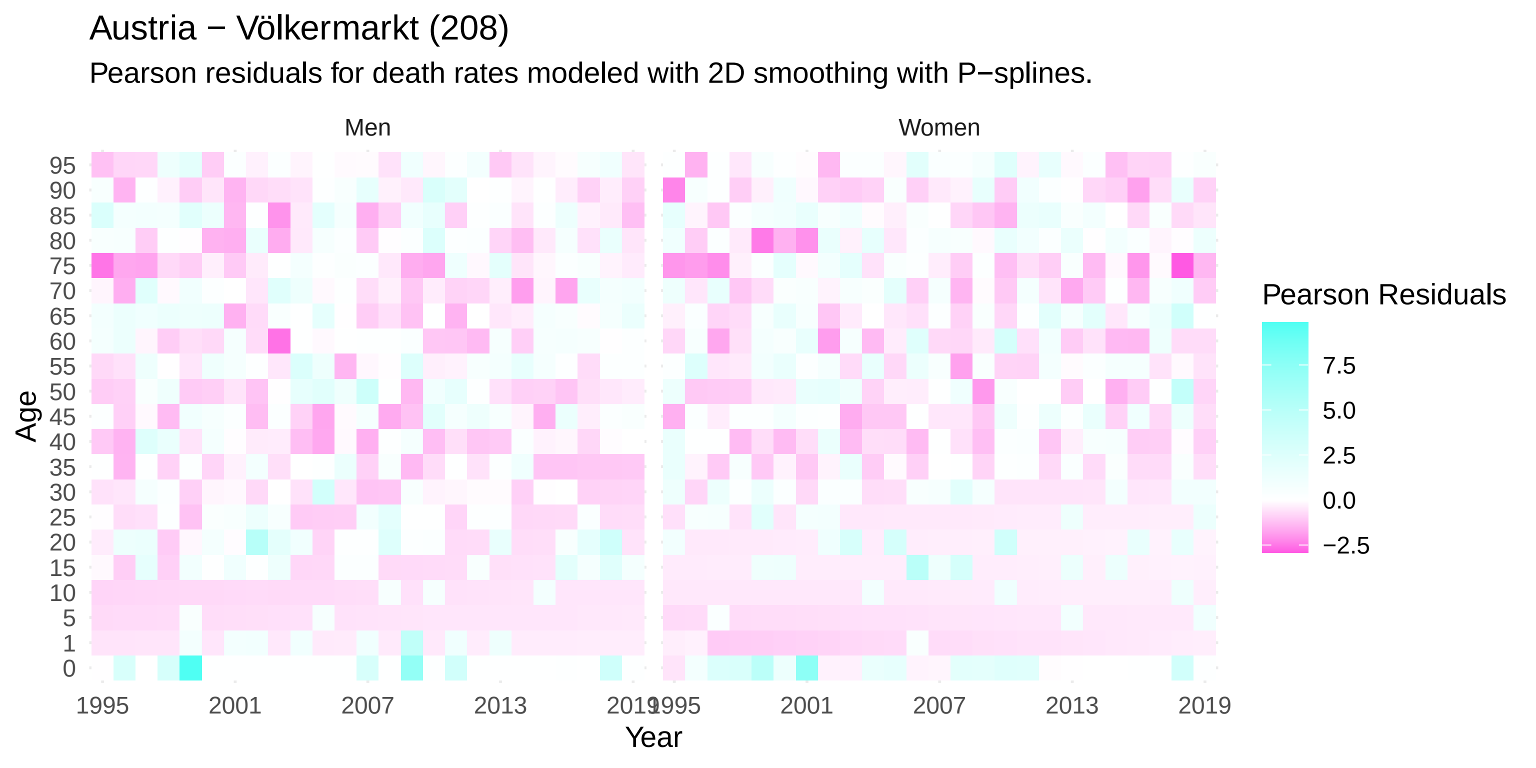

Austria − Völkermarkt (208)
Pearson residuals for death rates modeled with 2D smoothing with P−splines.
Men
Women
Age
95
90
85
80
75
70
65
60
55
50
45
40
35
30
25
20
15
10
5
1
0
1995
2001
2007
2013
2019
Year
Pearson Residuals
7.5
5.0
2.5
0.0
−2.5

Austria − Wolfsberg (209)

Pearson residuals for death rates modeled with 2D smoothing with P−splines.

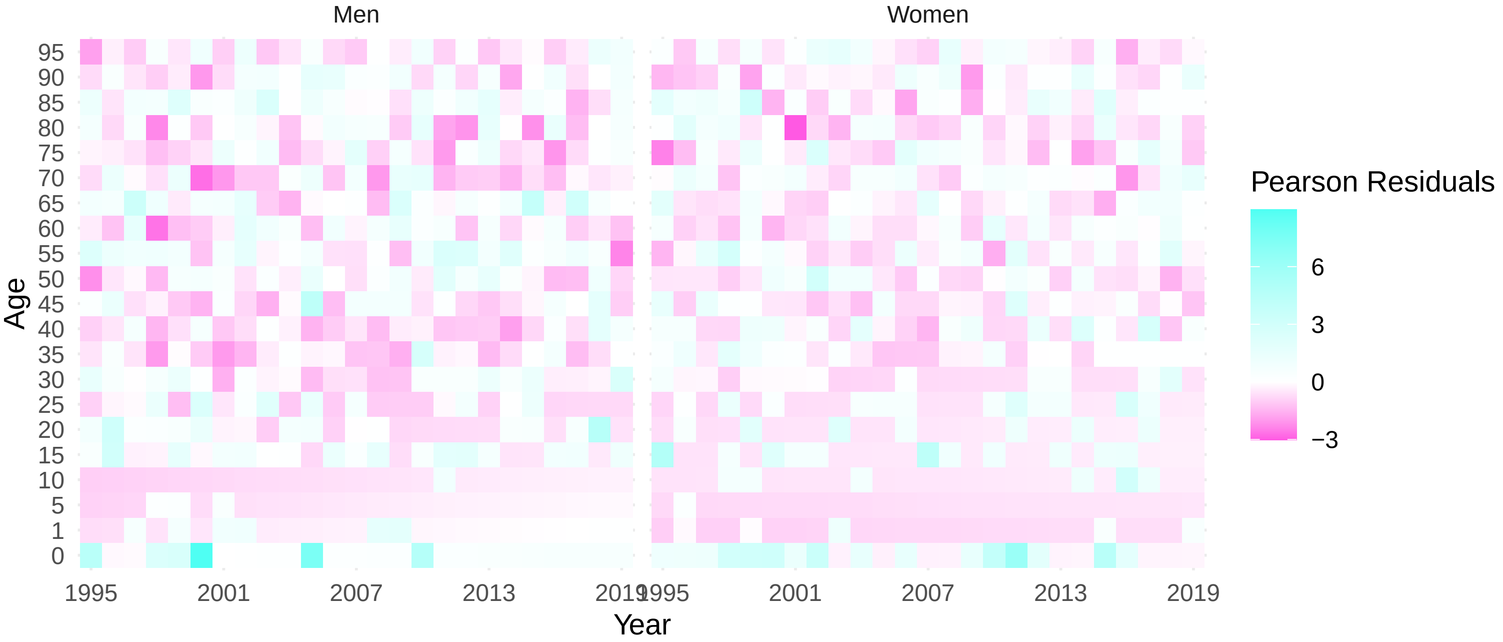

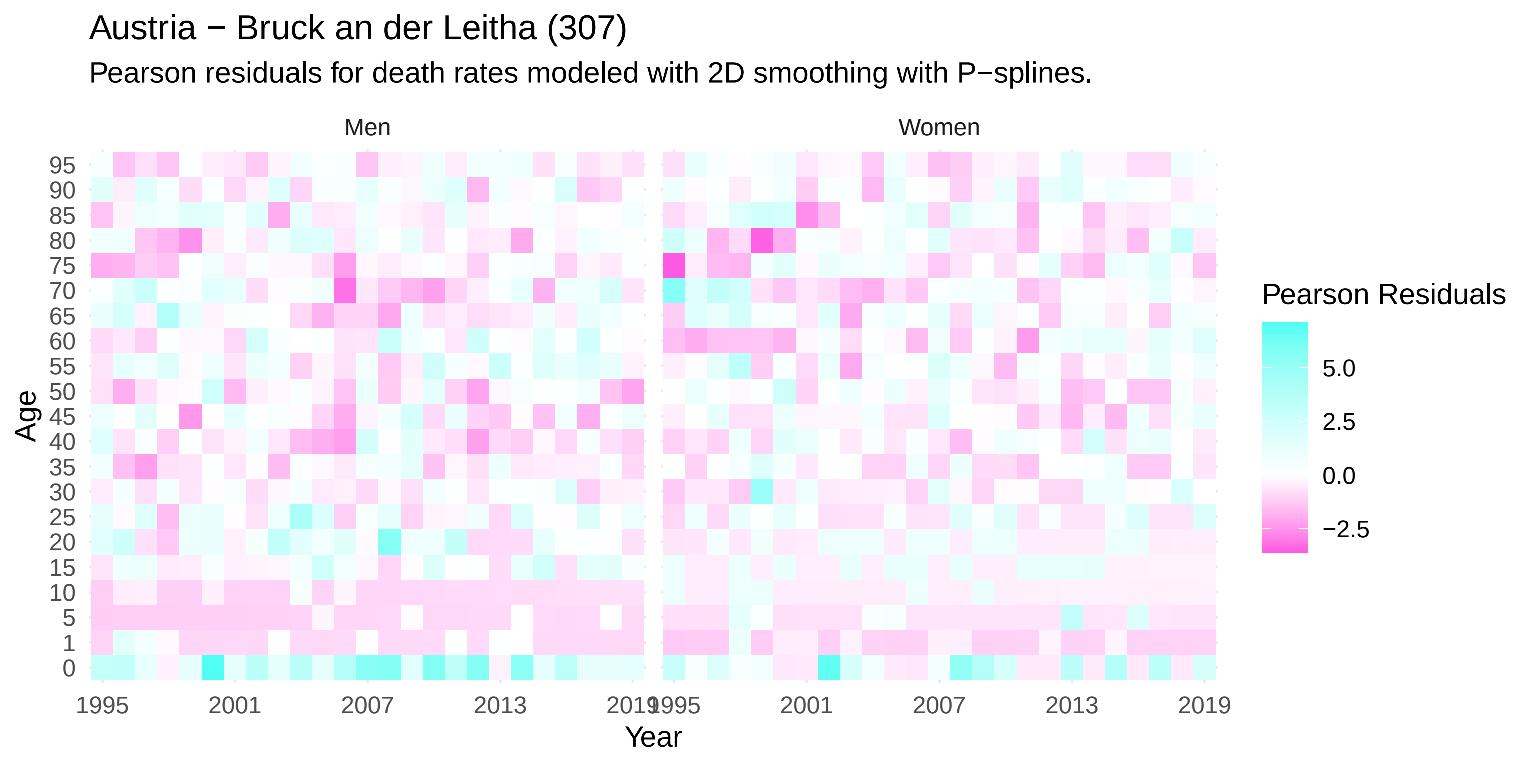

Austria − Bruck an der Leitha (307)
Pearson residuals for death rates modeled with 2D smoothing with P−splines.
Men
Women
Age
95
90
85
80
75
70
65
60
55
50
45
40
35
30
25
20
15
10
5
1
0
1995
2001
2007
2013
2019
Year
Pearson Residuals
5.0
2.5
0.0
−2.5

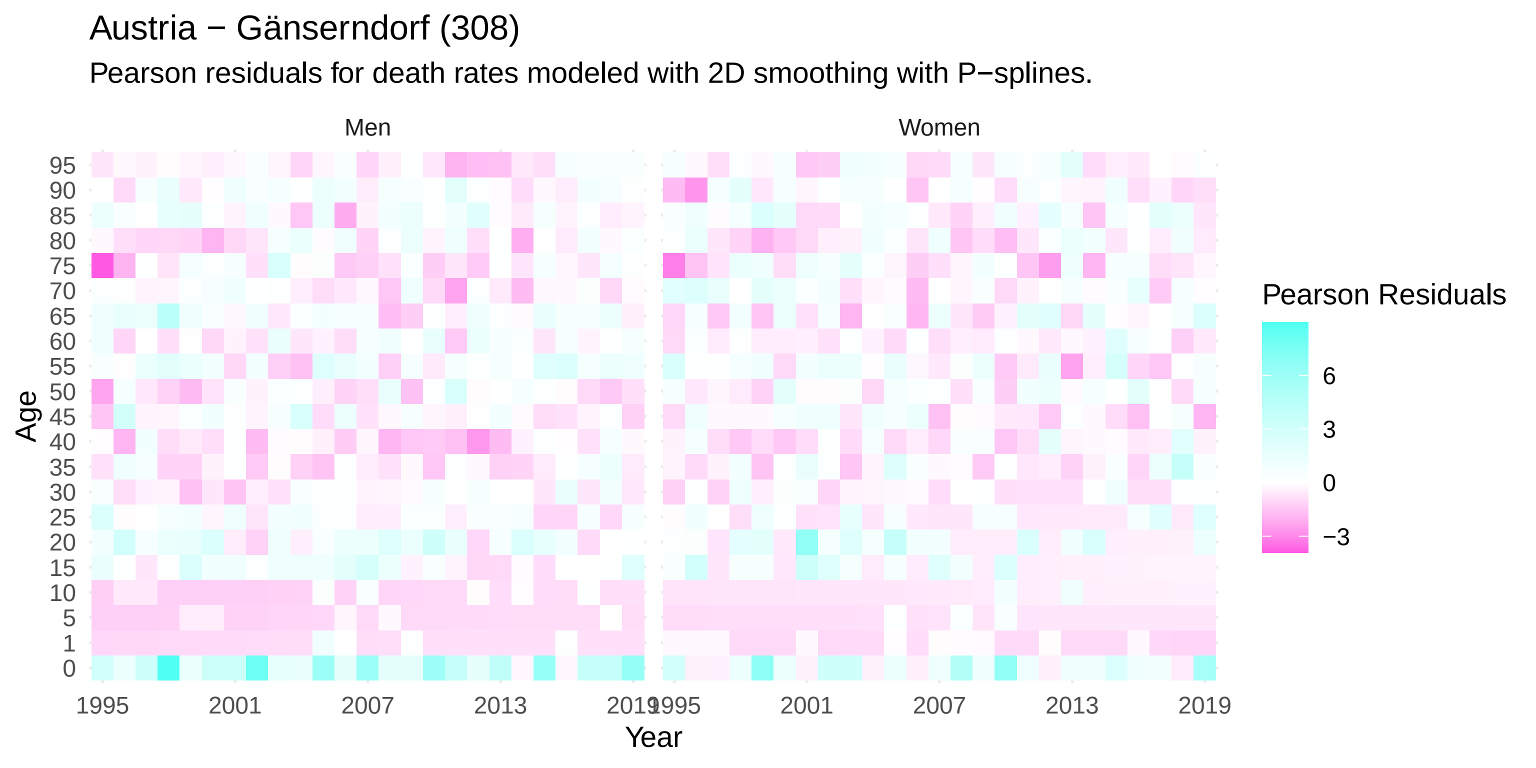

Austria − Gänserndorf (308)
Pearson residuals for death rates modeled with 2D smoothing with P−splines.
Men
Women
Age
95
90
85
80
75
70
65
60
55
50
45
40
35
30
25
20
15
10
5
1
0
1995
2001
2007
2013
2019
Year
Pearson Residuals
6
3
0
−3

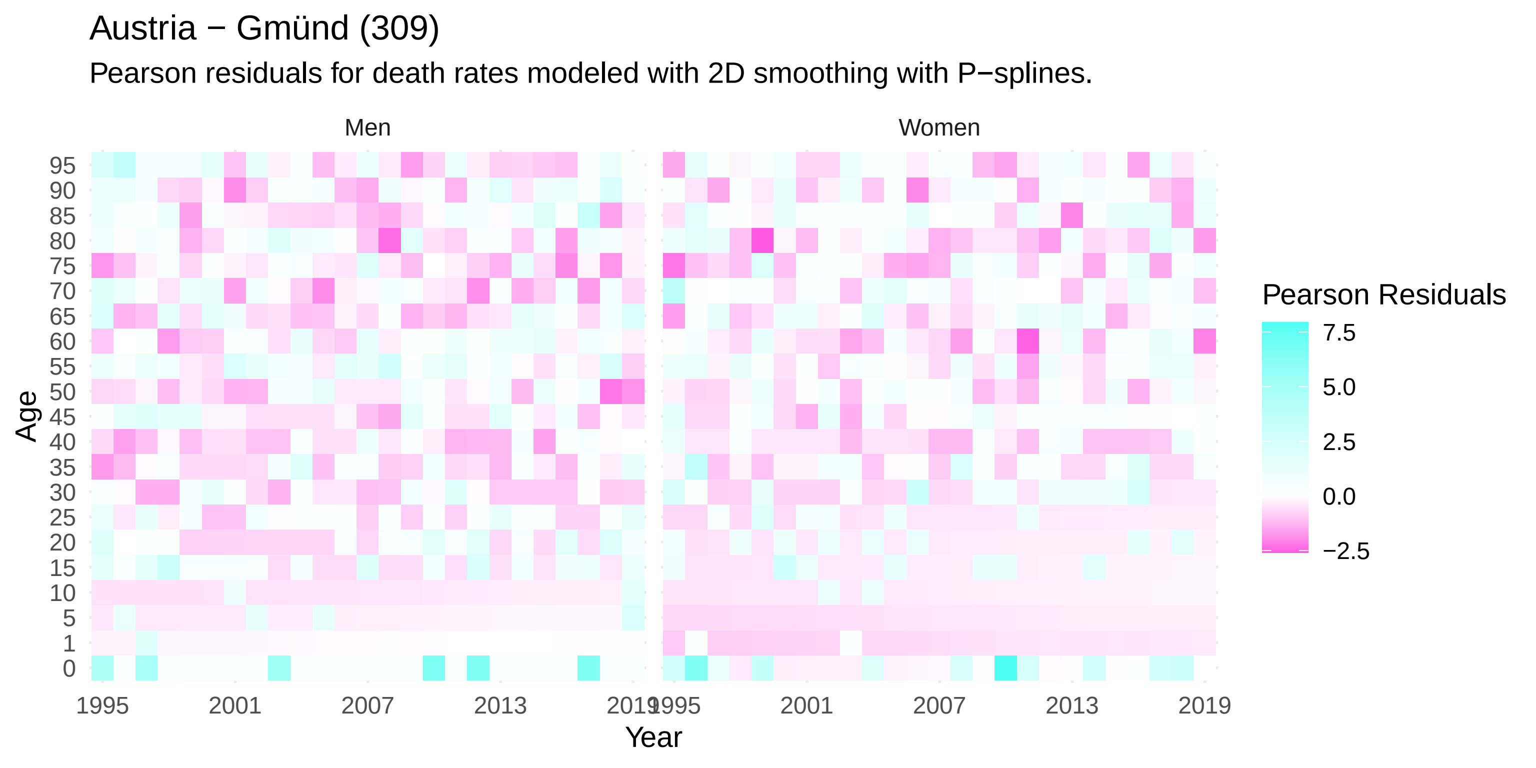

Austria − Gmünd (309)
Pearson residuals for death rates modeled with 2D smoothing with P−splines.
Men
Women
Age
95
90
85
80
75
70
65
60
55
50
45
40
35
30
25
20
15
10
5
1
0
1995
2001
2007
2013
2019
Year
Pearson Residuals
7.5
5.0
2.5
0.0
−2.5

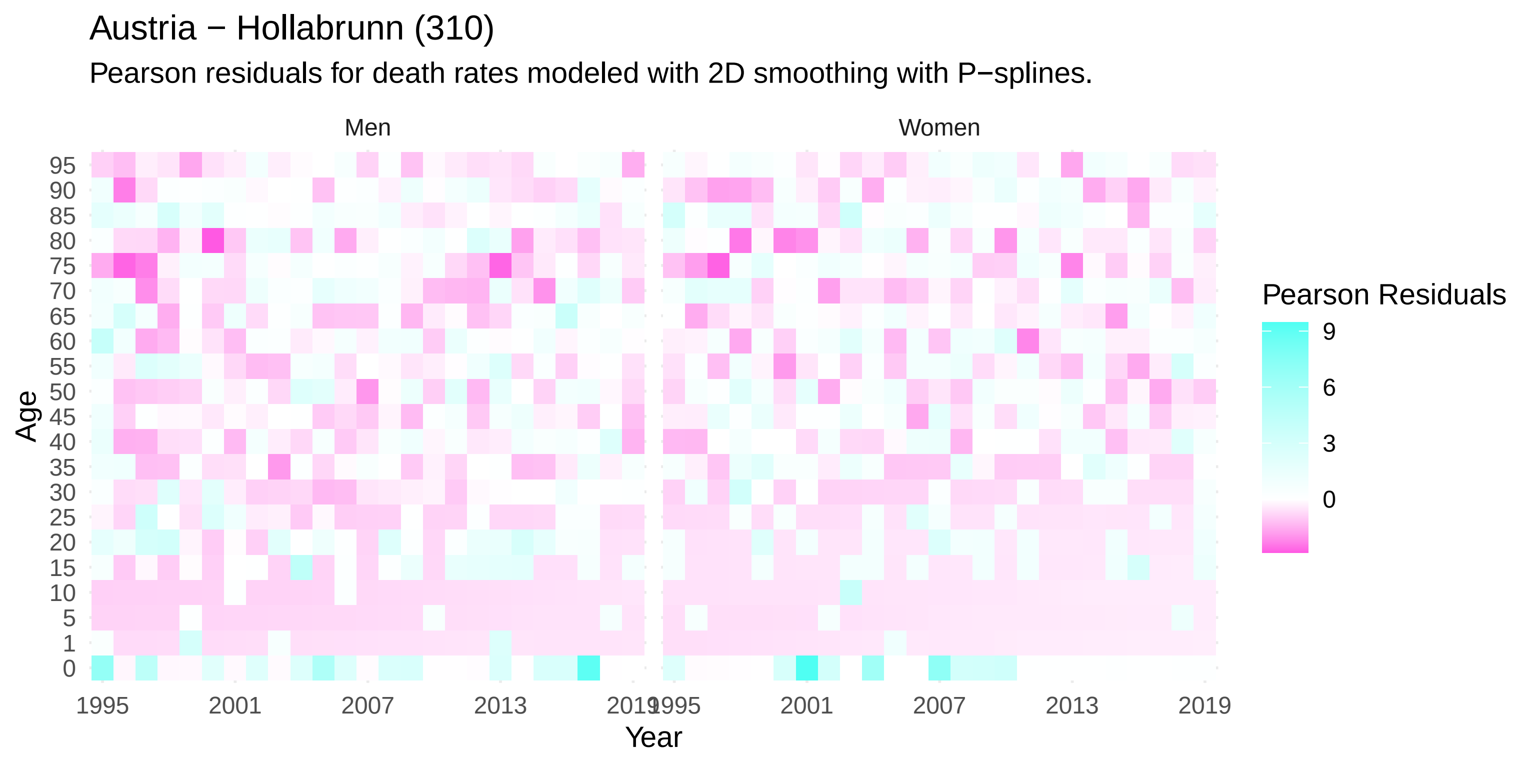

Austria − Hollabrunn (310)
Pearson residuals for death rates modeled with 2D smoothing with P−splines.
Men
Women
Age
95
90
85
80
75
70
65
60
55
50
45
40
35
30
25
20
15
10
5
1
0
1995
2001
2007
2013
2019
Year
Pearson Residuals
9
6
3
0

Austria − Horn (311)

Pearson residuals for death rates modeled with 2D smoothing with P−splines.

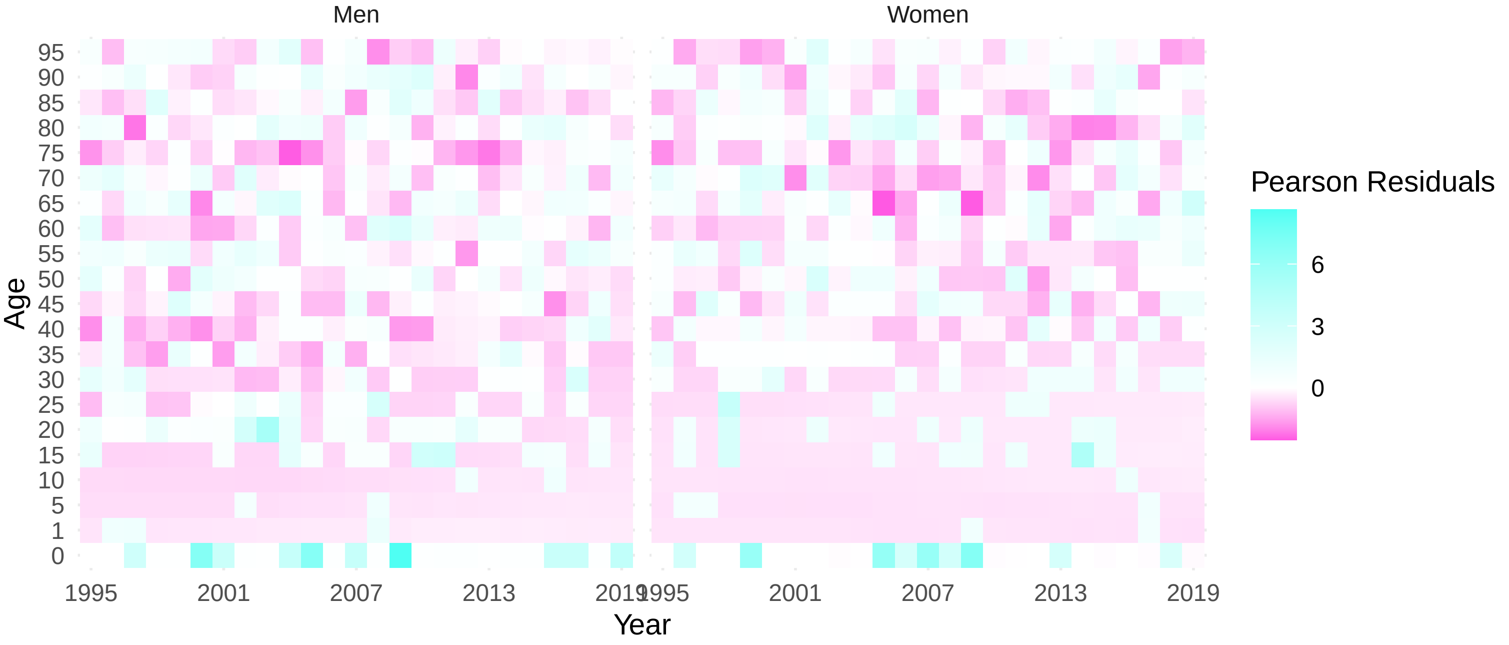

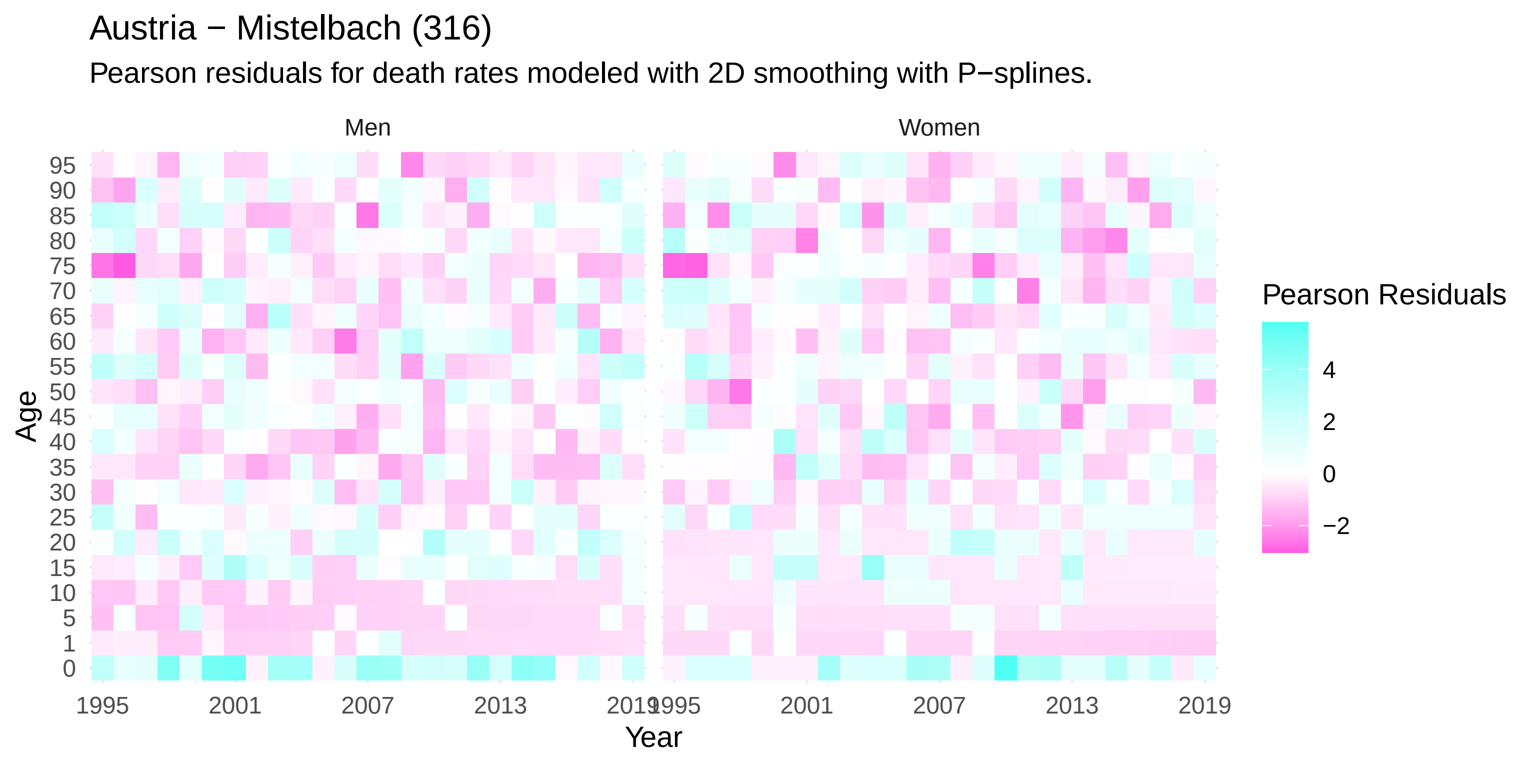

Austria − Mistelbach (316)
Pearson residuals for death rates modeled with 2D smoothing with P−splines.
Men
Women
Age
95
90
85
80
75
70
65
60
55
50
45
40
35
30
25
20
15
10
5
1
0
1995
2001
2007
2013
2019
Year
Pearson Residuals
4
2
0
−2

Austria − Waidhofen an der Thaya (322)

Pearson residuals for death rates modeled with 2D smoothing with P−splines.

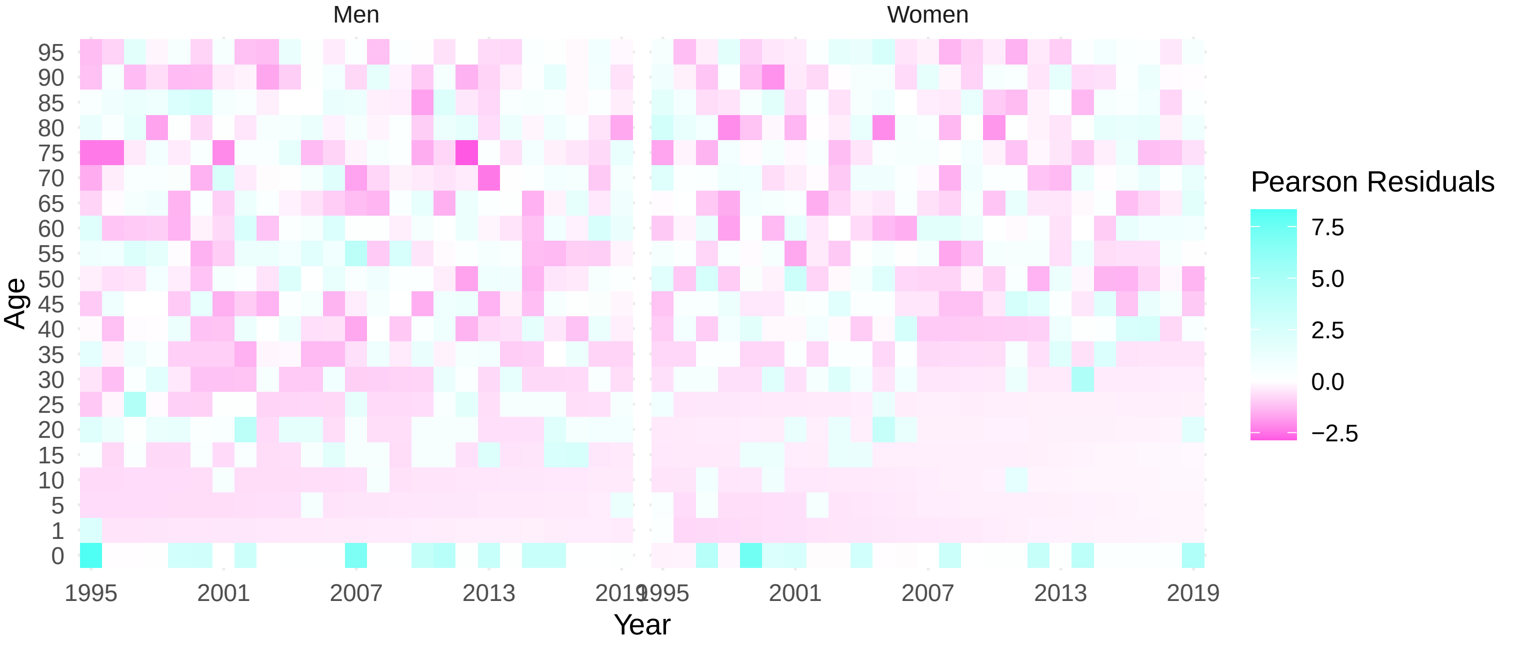

Austria − Braunau am Inn (404)

Pearson residuals for death rates modeled with 2D smoothing with P−splines.

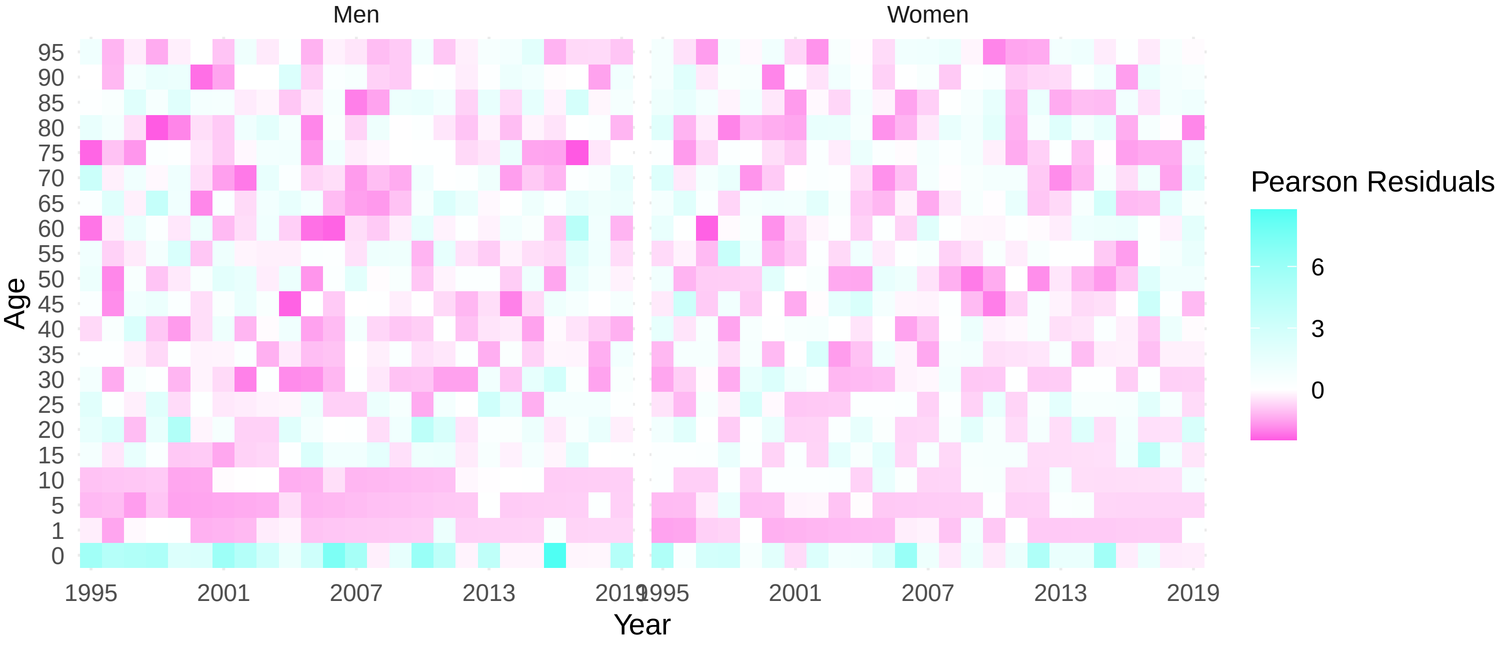

Austria − Freistadt (406)

Pearson residuals for death rates modeled with 2D smoothing with P−splines.

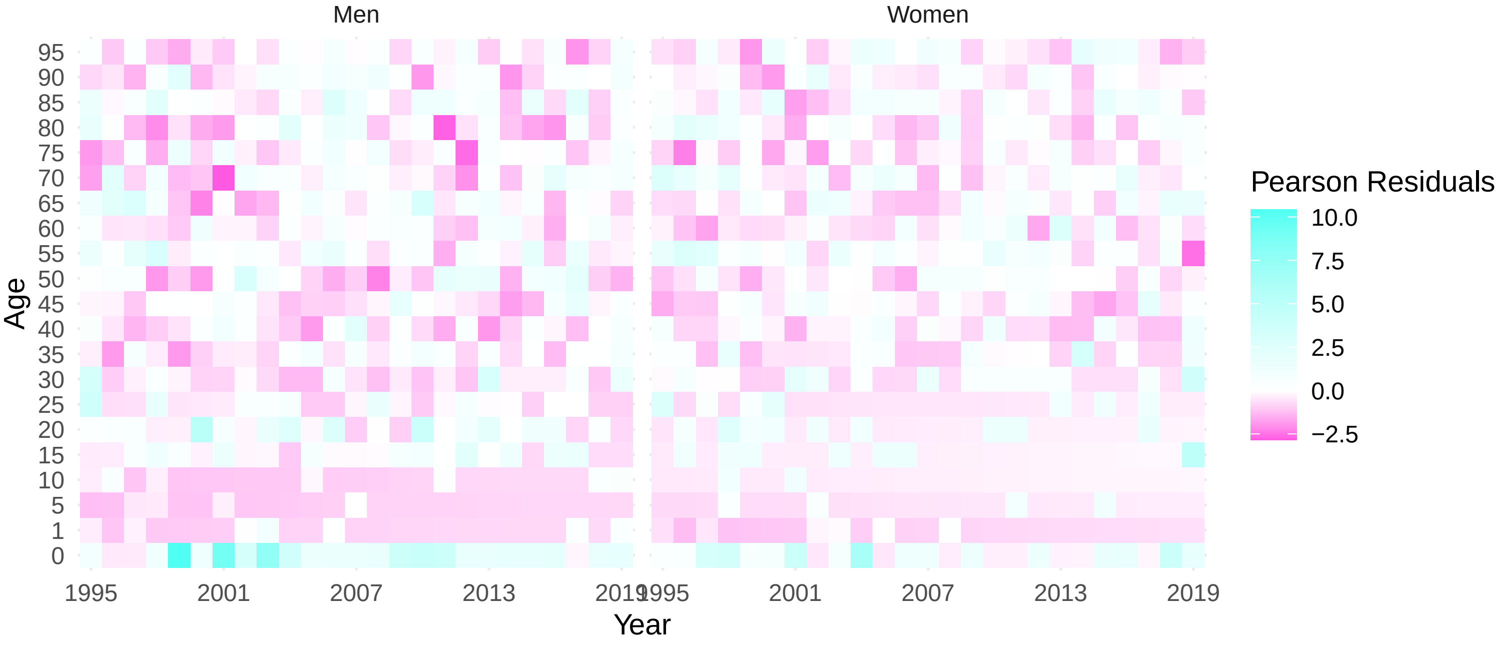

Austria − Ried im Innkreis (412)

Pearson residuals for death rates modeled with 2D smoothing with P−splines.

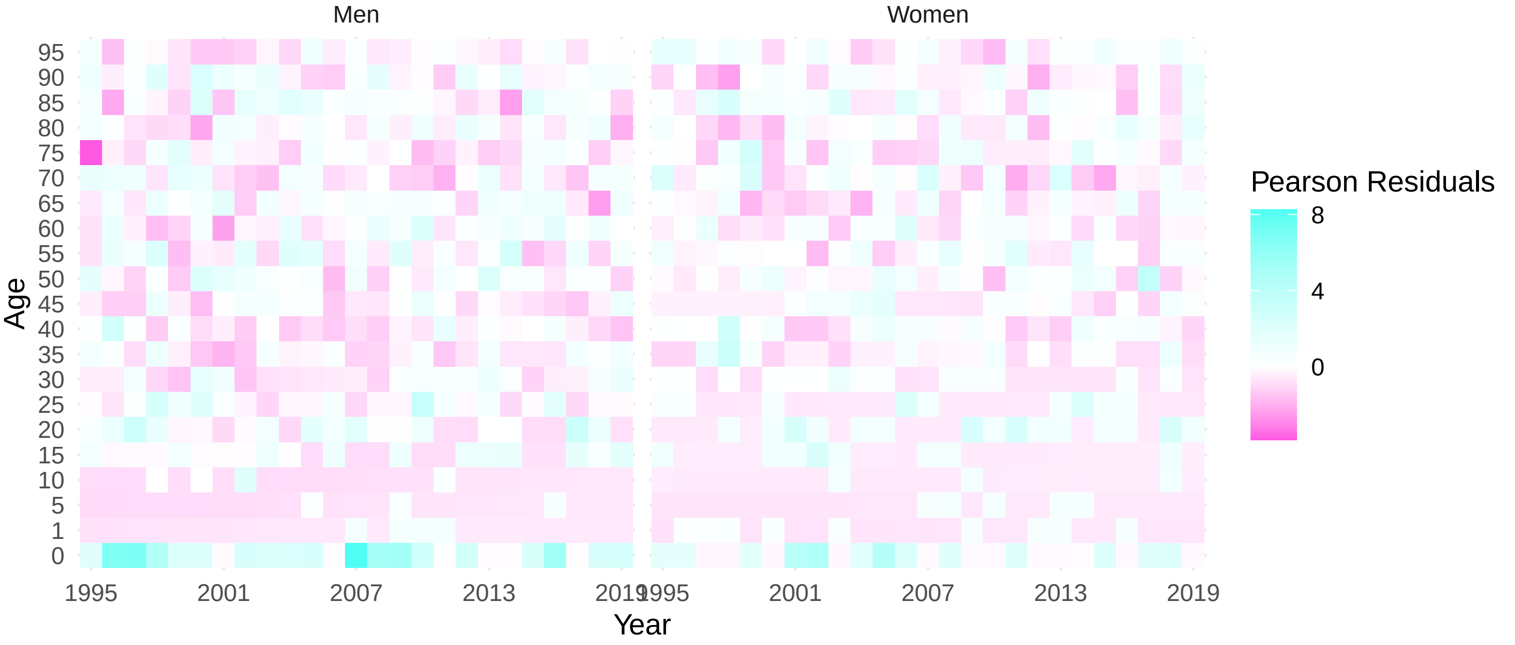

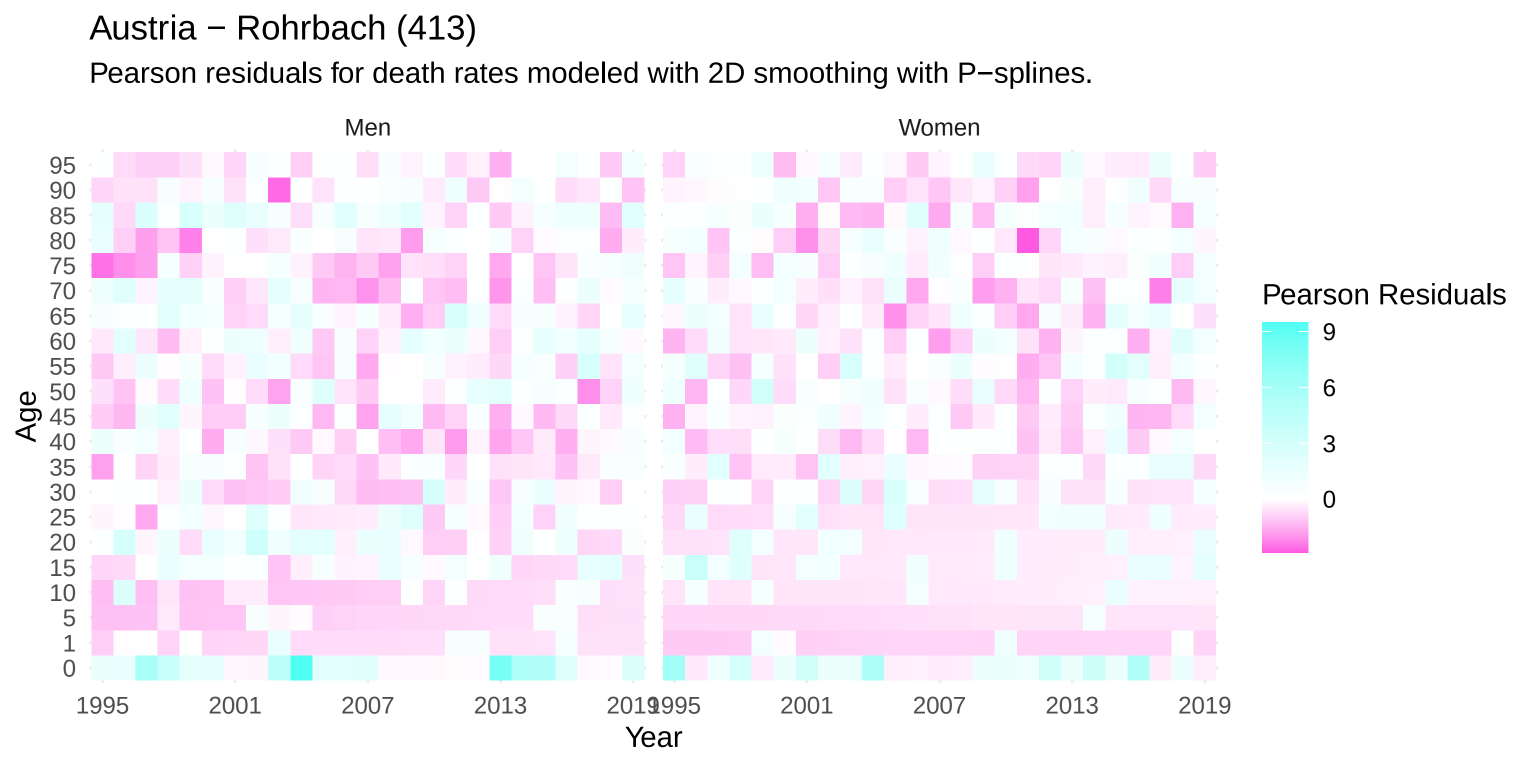
Austria − Rohrbach (413)
Pearson residuals for death rates modeled with 2D smoothing with P−splines.
Men
Women
Age
Year
Pearson Residuals
9
6
3
0
95
90
85
80
75
70
65
60
55
50
45
40
35
30
25
20
15
10
5
1
0
1995
2001
2007
2013
2019

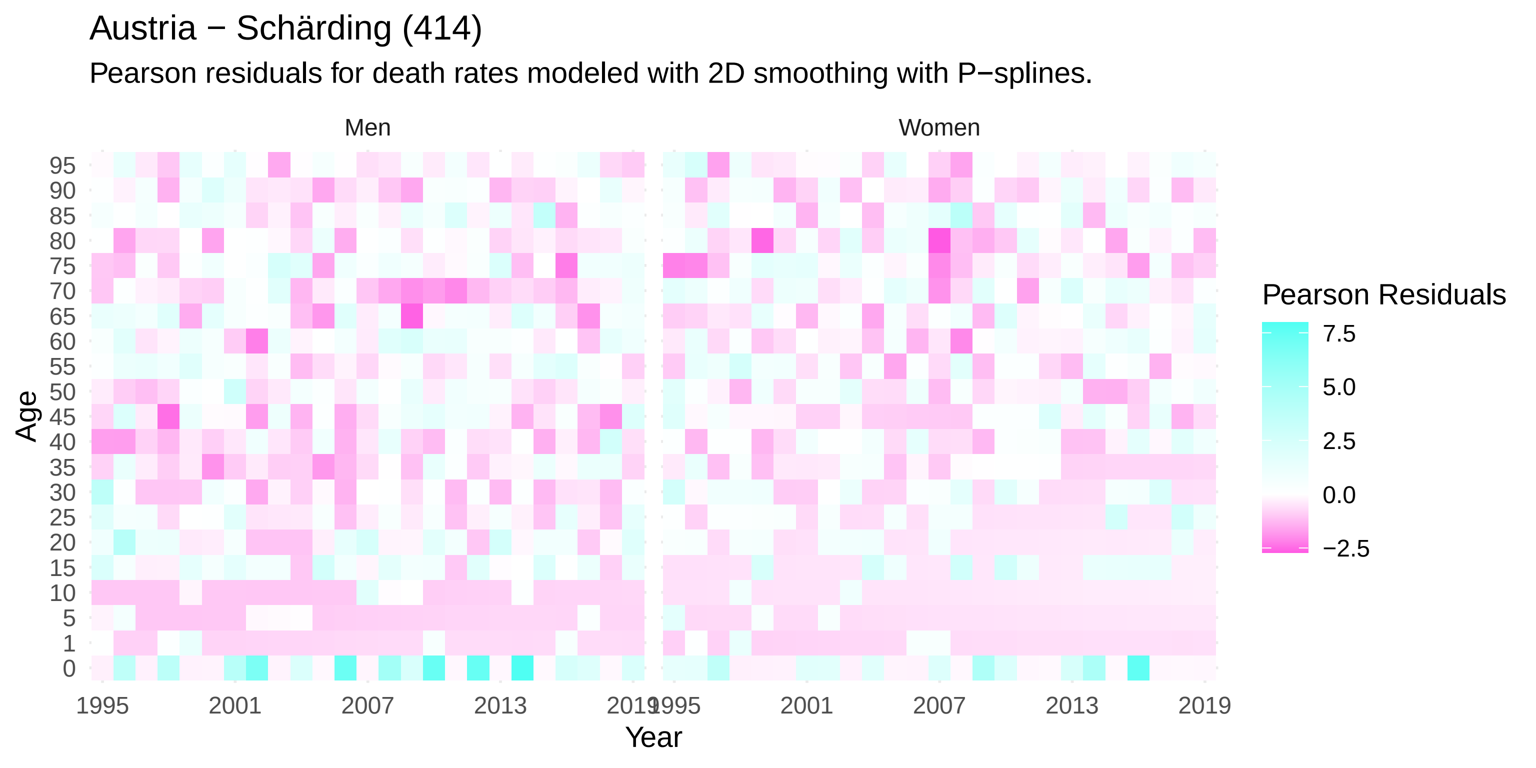

Austria − Schärding (414)
Pearson residuals for death rates modeled with 2D smoothing with P−splines.
Men
Women
Age
95
90
85
80
75
70
65
60
55
50
45
40
35
30
25
20
15
10
5
1
0
1995
2001
2007
2013
2019
Year
Pearson Residuals
7.5
5.0
2.5
0.0
−2.5

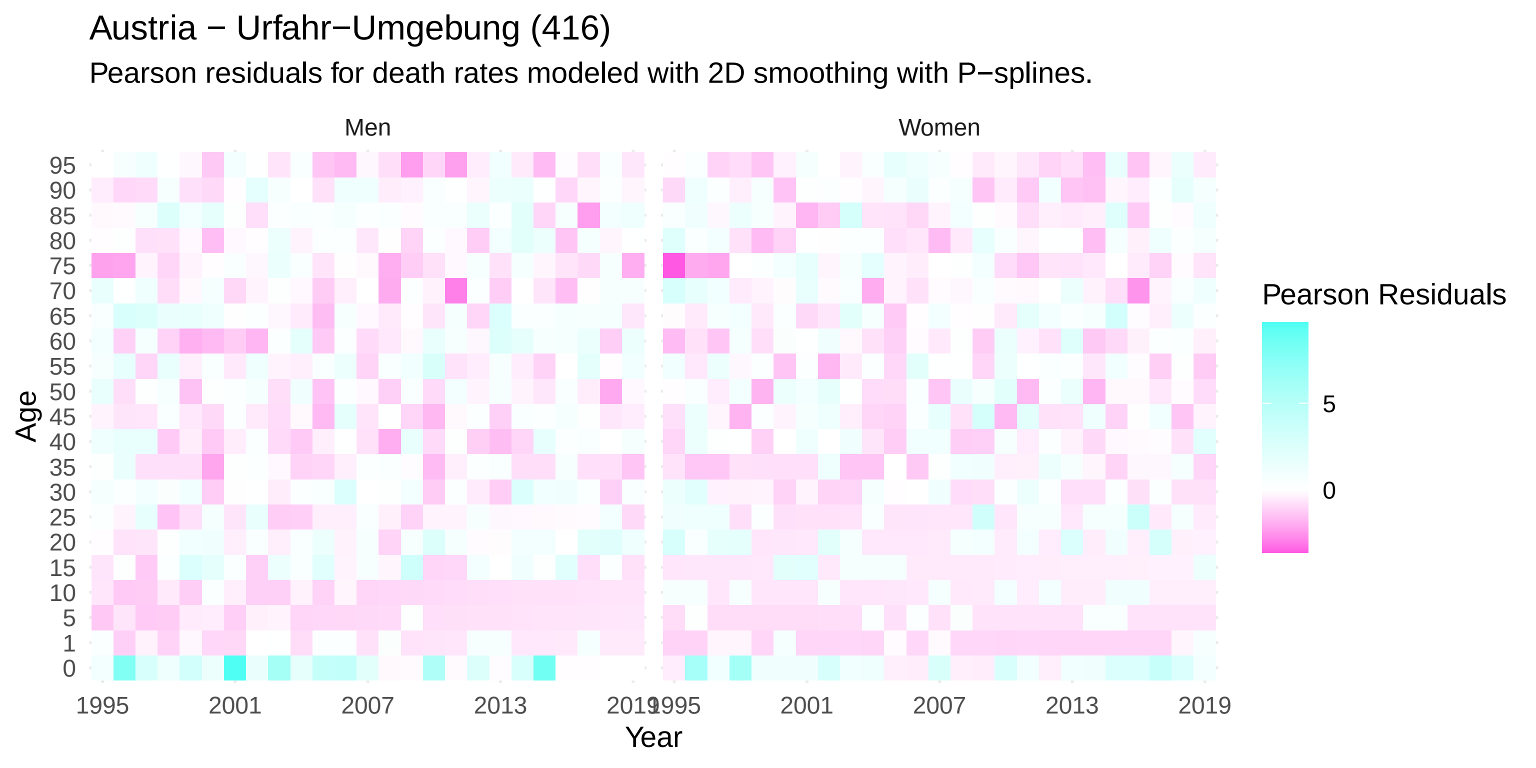

Austria − Urfahr−Umgebung (416)
Pearson residuals for death rates modeled with 2D smoothing with P−splines.
Men
Women
Age
95
90
85
80
75
70
65
60
55
50
45
40
35
30
25
20
15
10
5
1
0
1995
2001
2007
2013
2019
Year
Pearson Residuals
5
0

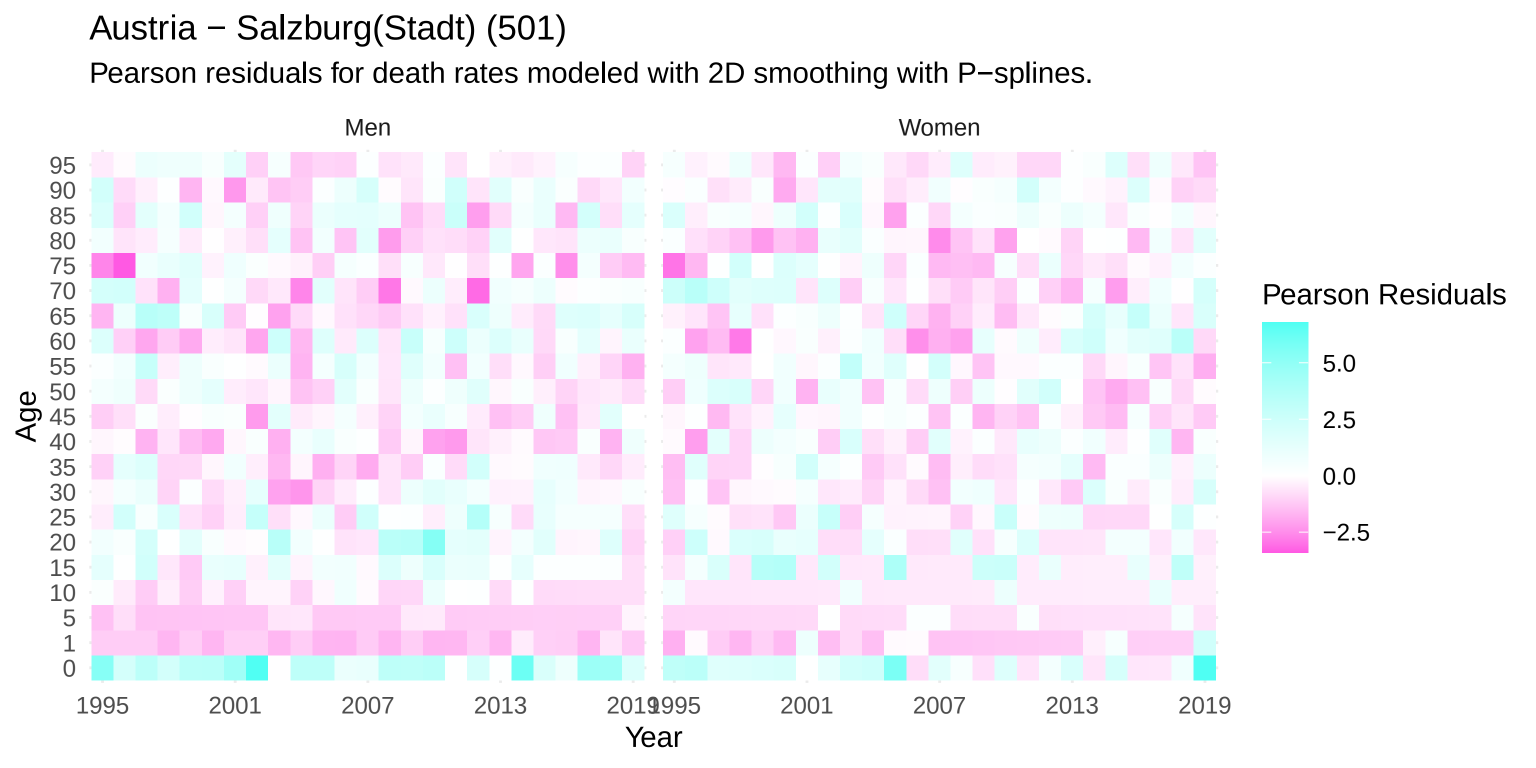

Austria − Salzburg(Stadt) (501)
Pearson residuals for death rates modeled with 2D smoothing with P−splines.
Men
Women
Age
95
90
85
80
75
70
65
60
55
50
45
40
35
30
25
20
15
10
5
1
0
1995
2001
2007
2013
2019
Year
Pearson Residuals
5.0
2.5
0.0
−2.5

Austria − Hallein (502)

Pearson residuals for death rates modeled with 2D smoothing with P−splines.

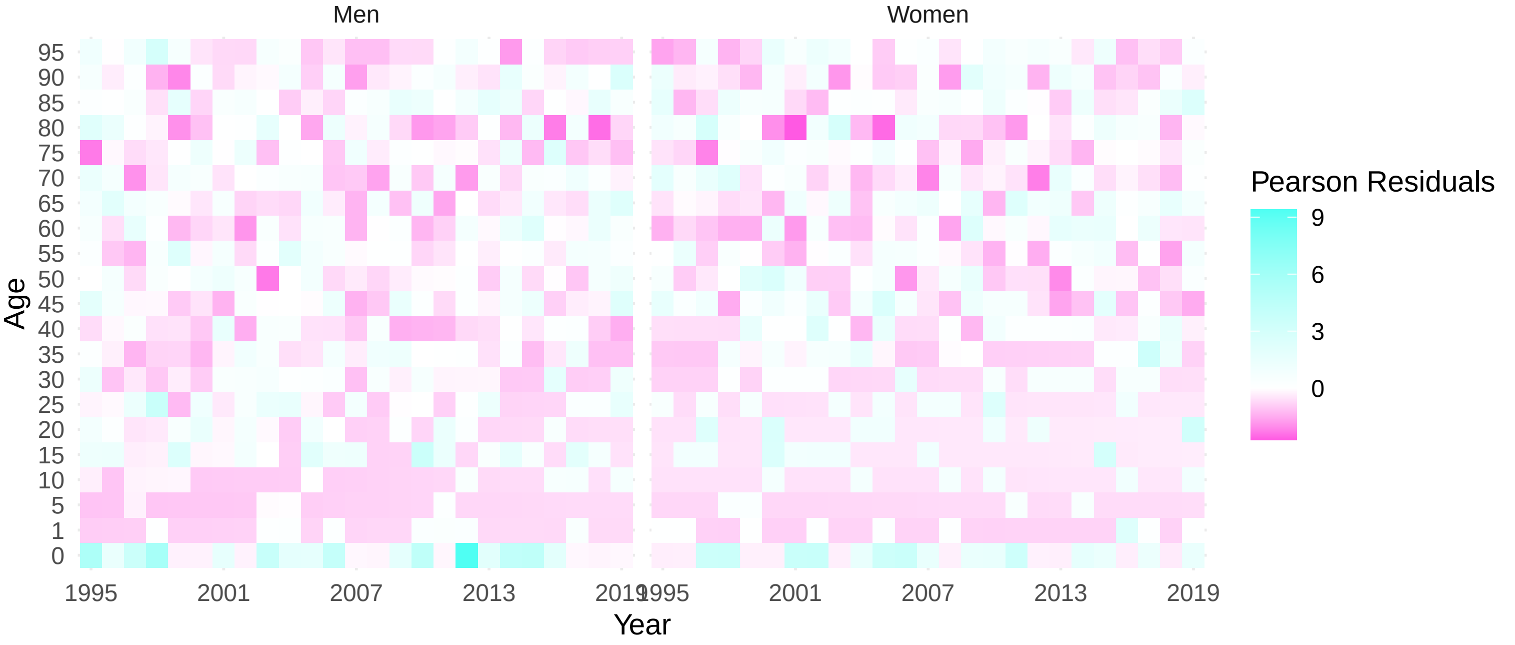

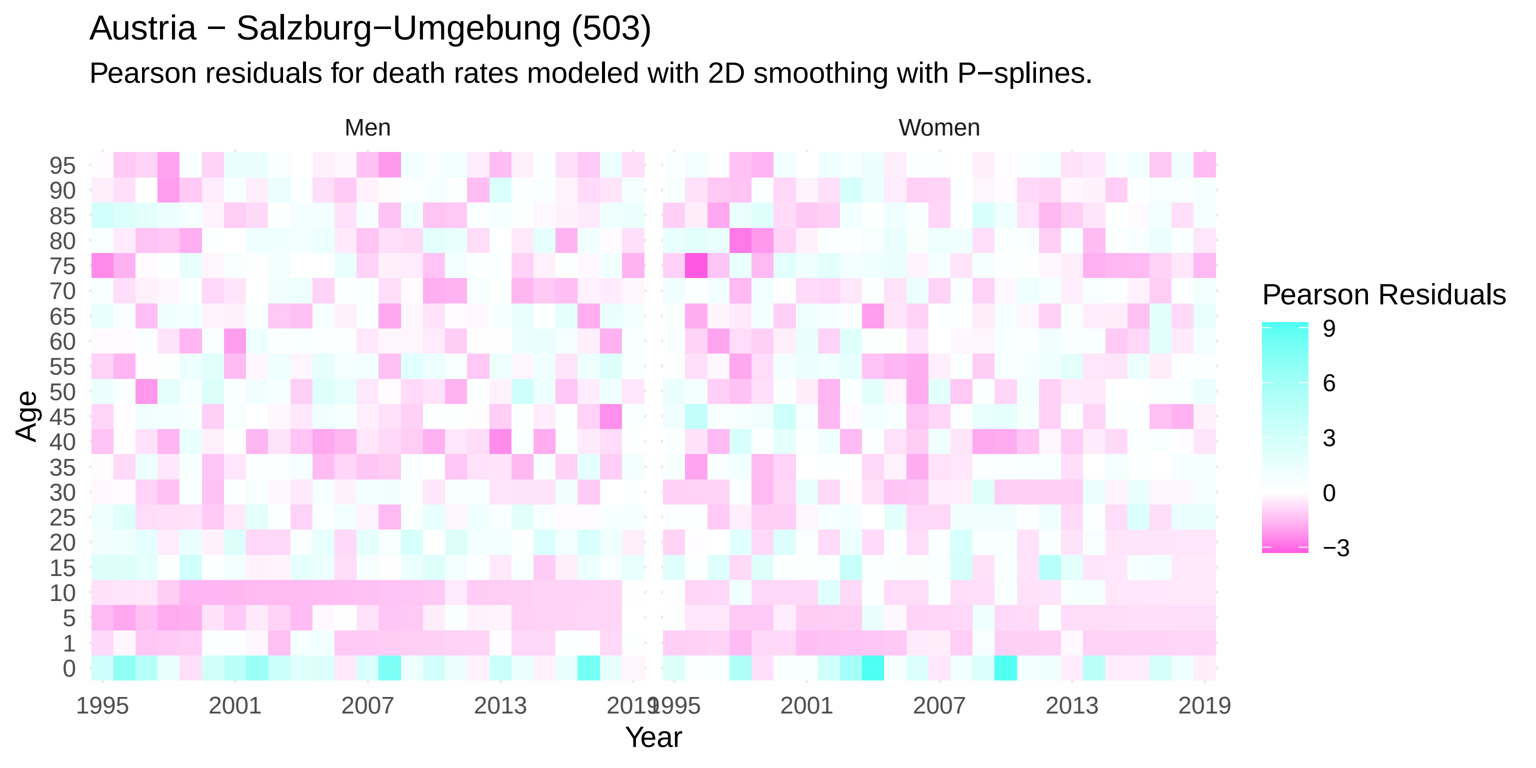

Austria − Salzburg−Umgebung (503)
Pearson residuals for death rates modeled with 2D smoothing with P−splines.
Men
Women
Age
95
90
85
80
75
70
65
60
55
50
45
40
35
30
25
20
15
10
5
1
0
1995
2001
2007
2013
2019
Year
Pearson Residuals
9
6
3
0
−3

Austria − Sankt Johann im Pongau (504)

Pearson residuals for death rates modeled with 2D smoothing with P−splines.

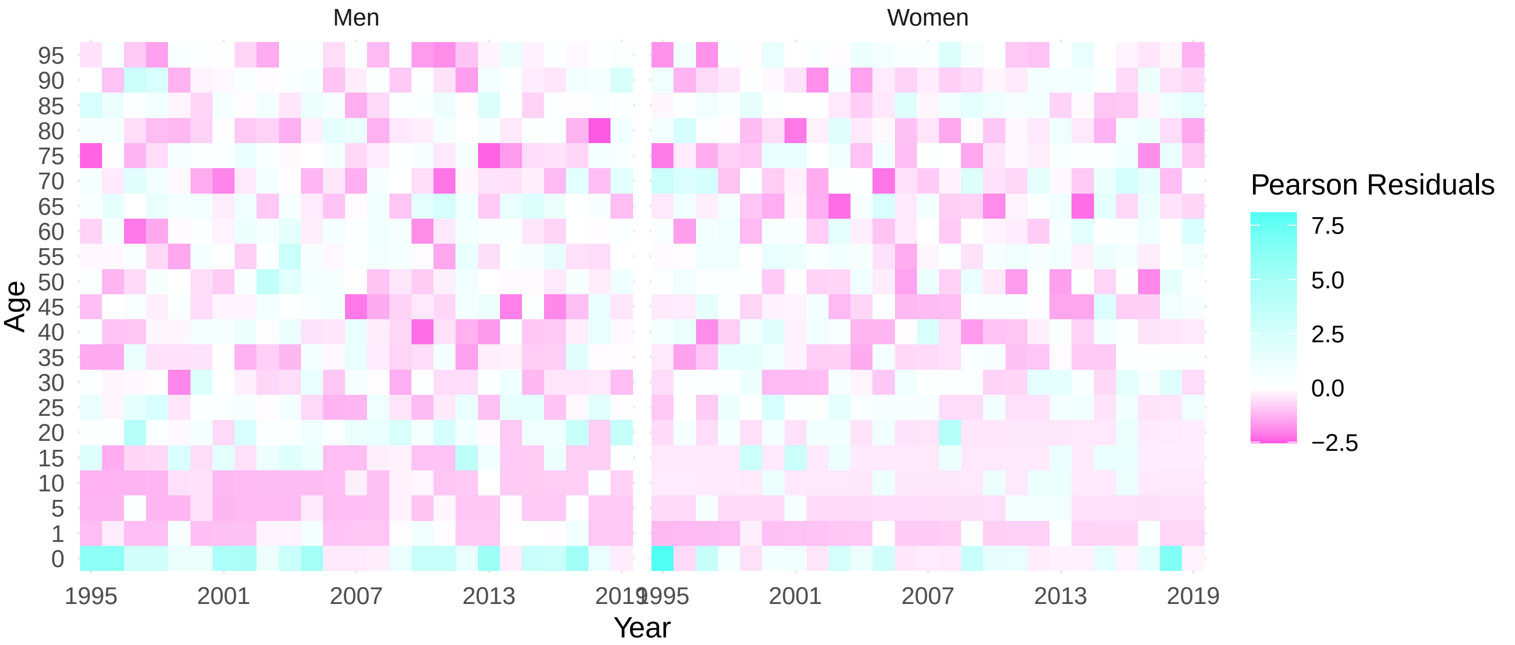

Austria − Zell am See (506)

Pearson residuals for death rates modeled with 2D smoothing with P−splines.

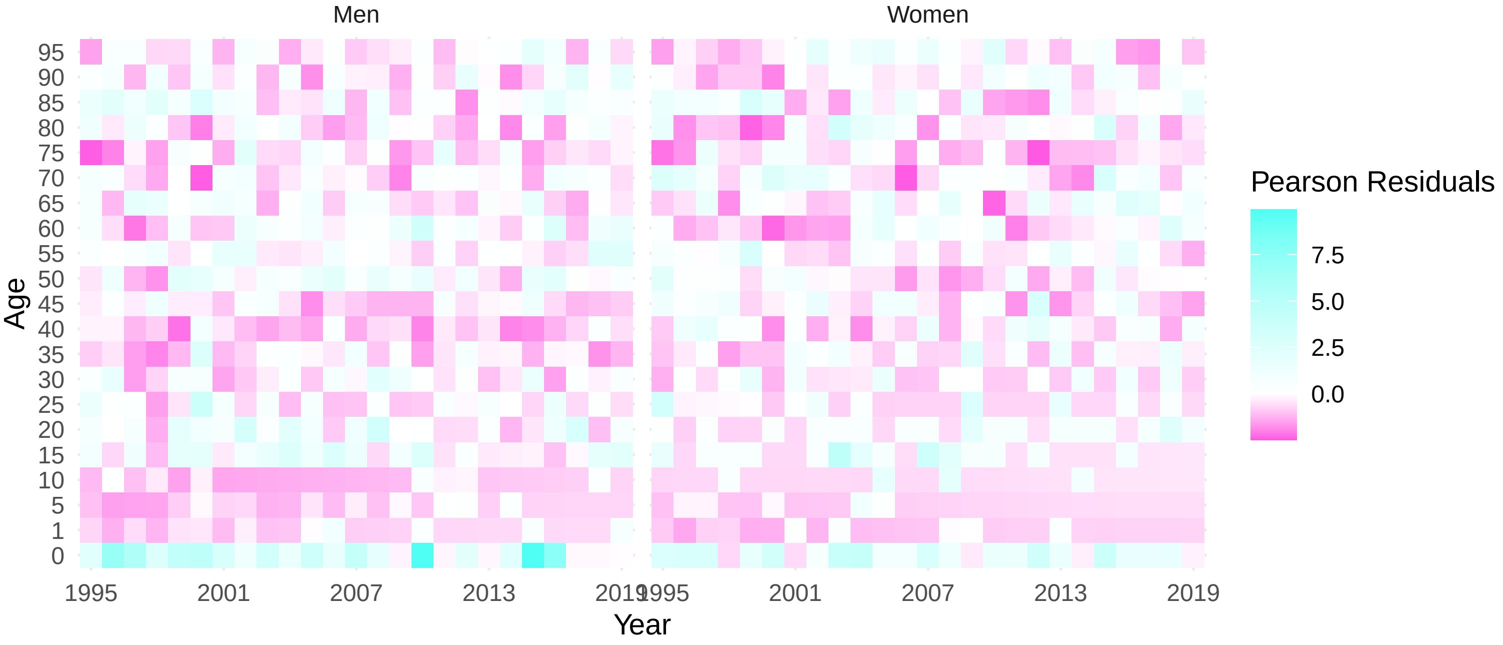

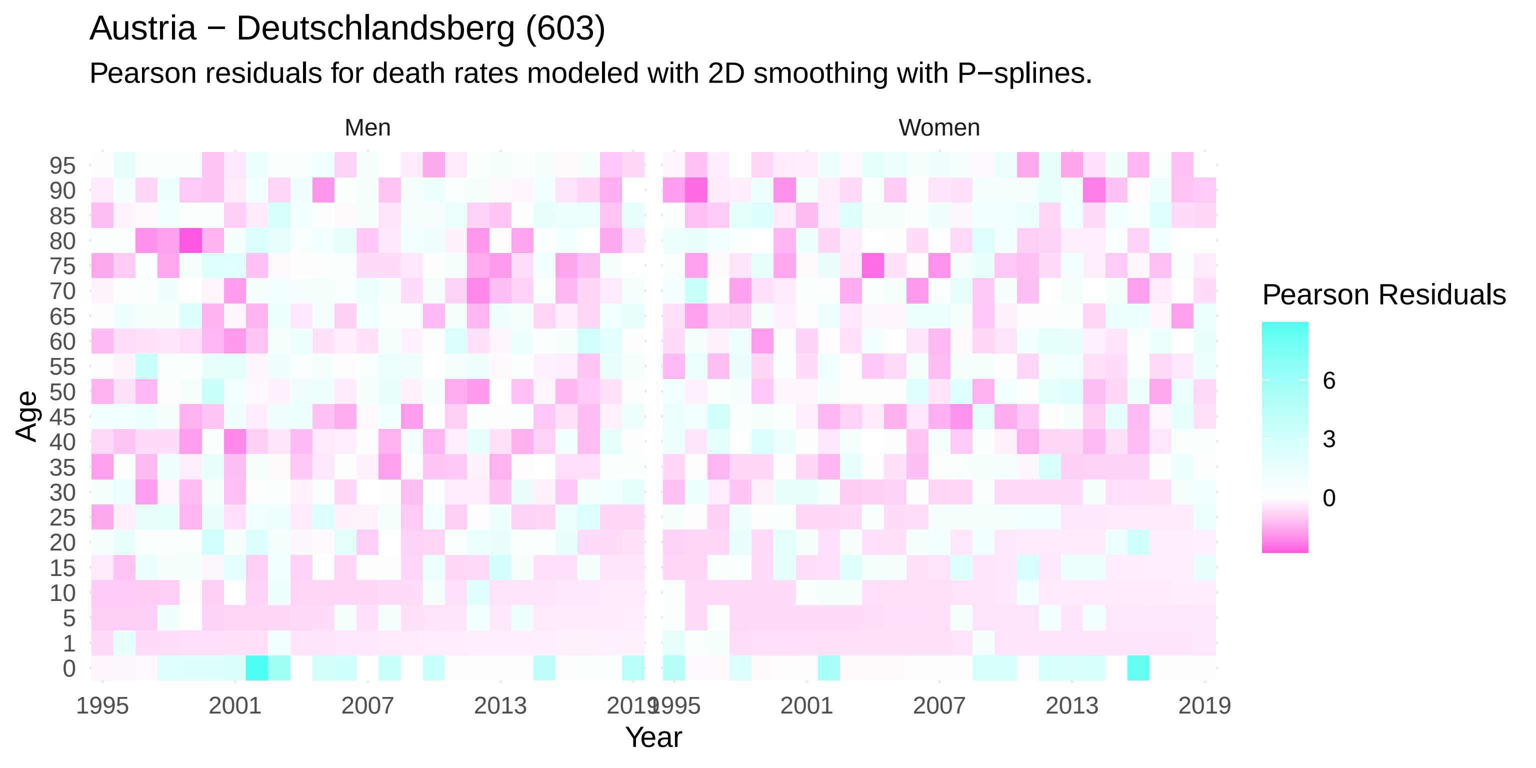
Austria − Deutschlandsberg (603)
Pearson residuals for death rates modeled with 2D smoothing with P−splines.
Men
Women
Age
95
90
85
80
75
70
65
60
55
50
45
40
35
30
25
20
15
10
5
1
0
1995
2001
2007
2013
2019
Year
Pearson Residuals
6
3
0

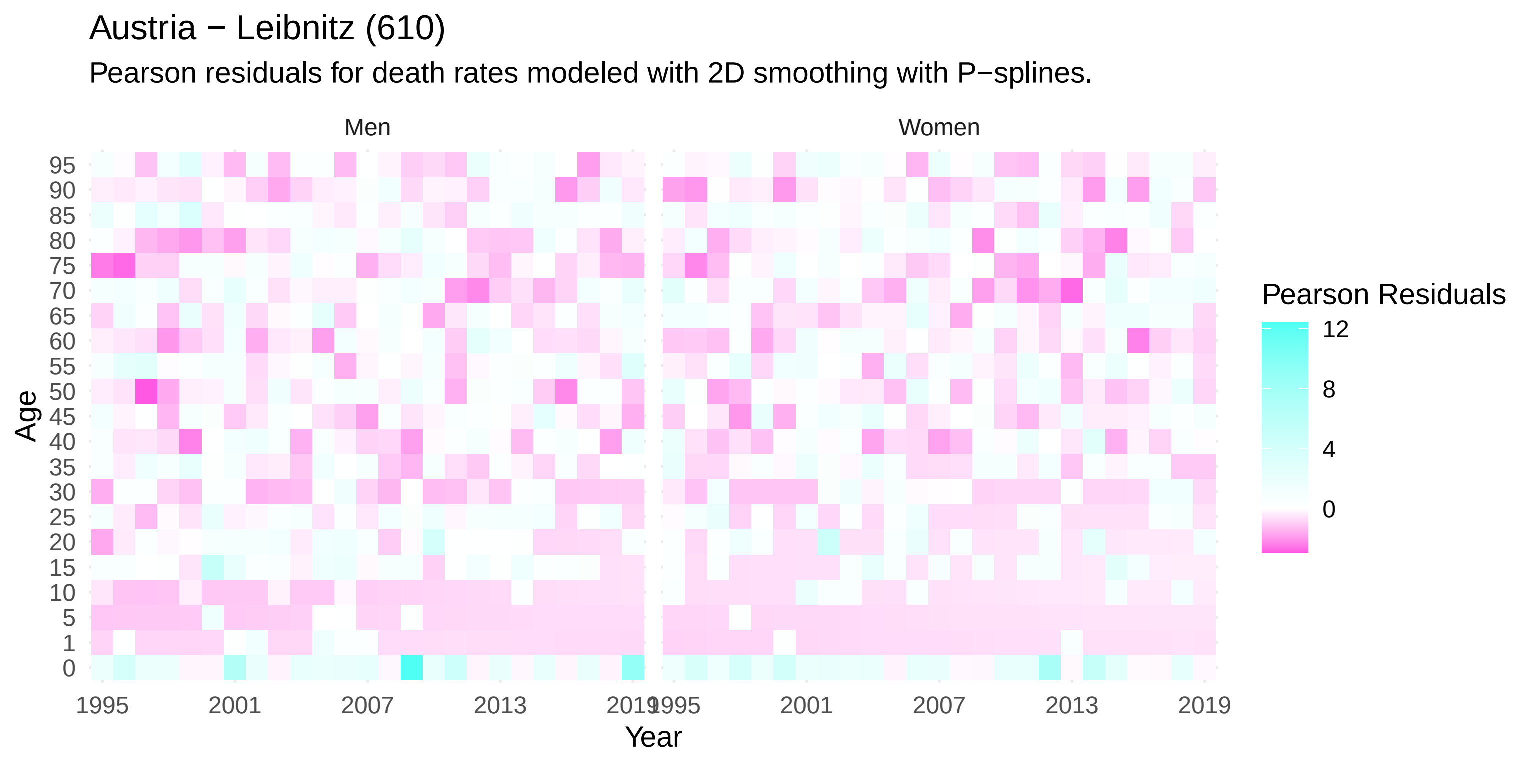

Austria − Leibnitz (610)
Pearson residuals for death rates modeled with 2D smoothing with P−splines.
Men
Women
Age
95
90
85
80
75
70
65
60
55
50
45
40
35
30
25
20
15
10
5
1
0
1995
2001
2007
2013
2019
Year
Pearson Residuals
12
8
4
0

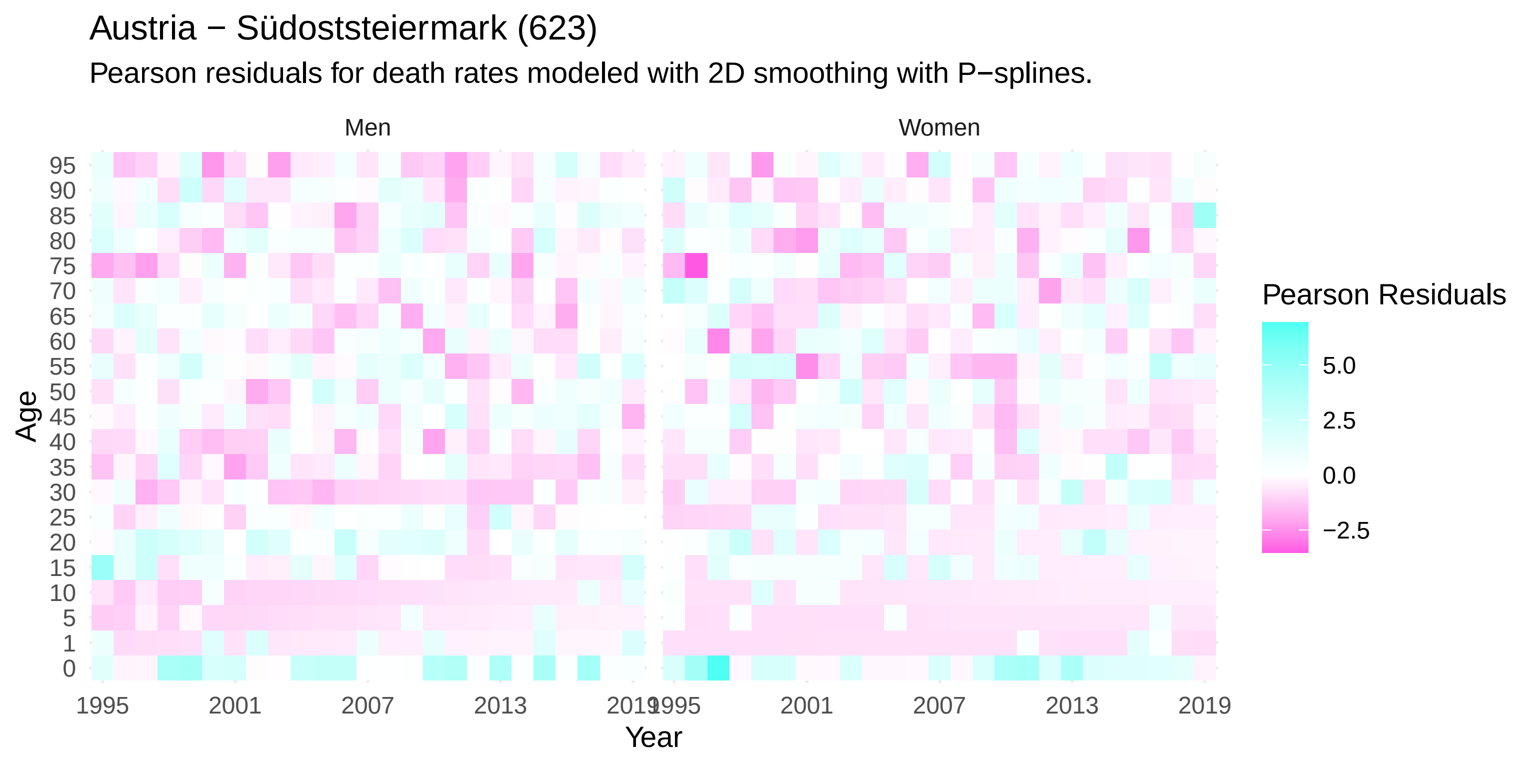

Austria − Südoststeiermark (623)
Pearson residuals for death rates modeled with 2D smoothing with P−splines.
Men
Women
Age
95
90
85
80
75
70
65
60
55
50
45
40
35
30
25
20
15
10
5
1
0
1995
2001
2007
2013
2019
Year
Pearson Residuals
5.0
2.5
0.0
−2.5

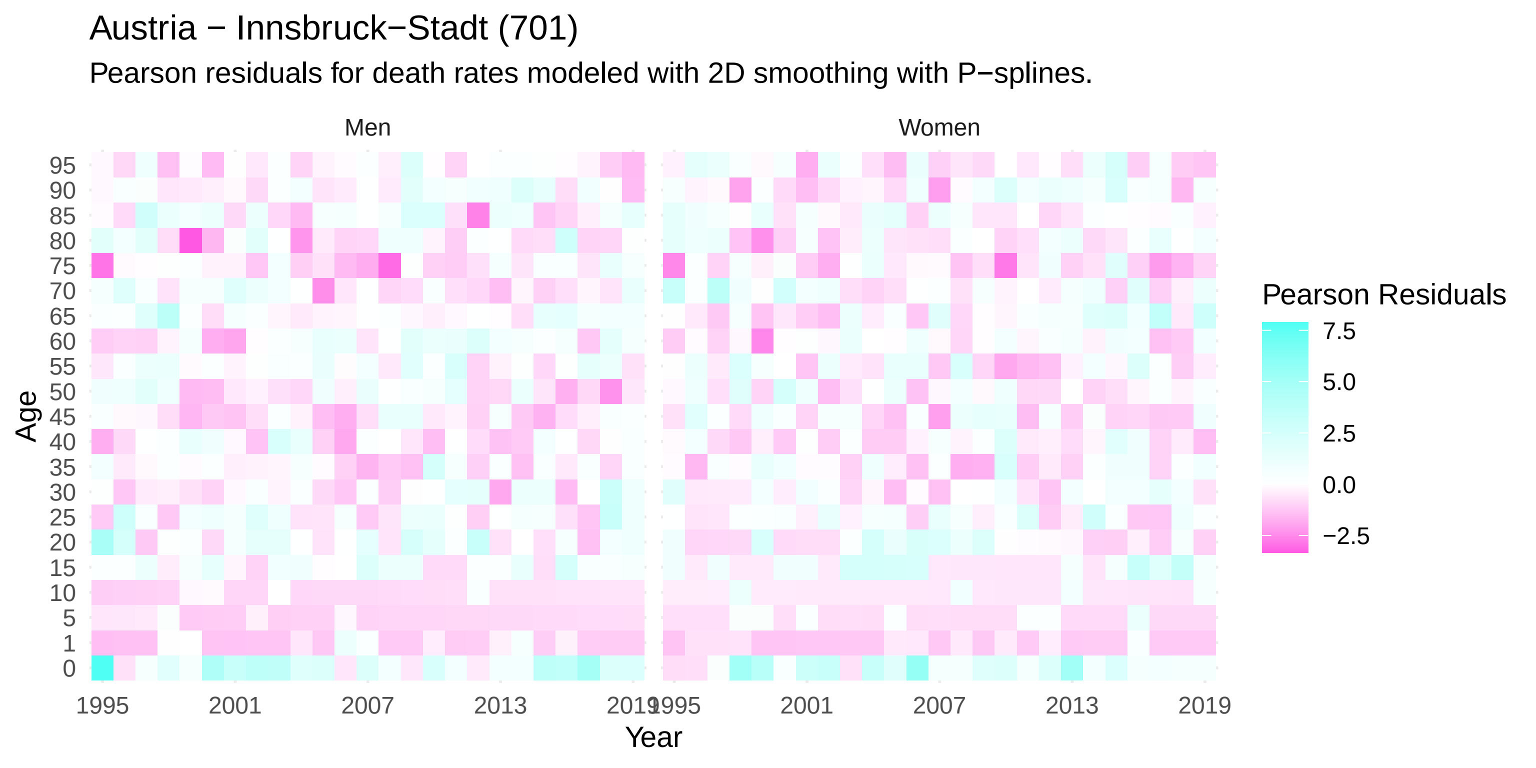

Austria − Innsbruck−Stadt (701)
Pearson residuals for death rates modeled with 2D smoothing with P−splines.
Men
Women
Age
95
90
85
80
75
70
65
60
55
50
45
40
35
30
25
20
15
10
5
1
0
1995
2001
2007
2013
2019
Year
Pearson Residuals
7.5
5.0
2.5
0.0
−2.5

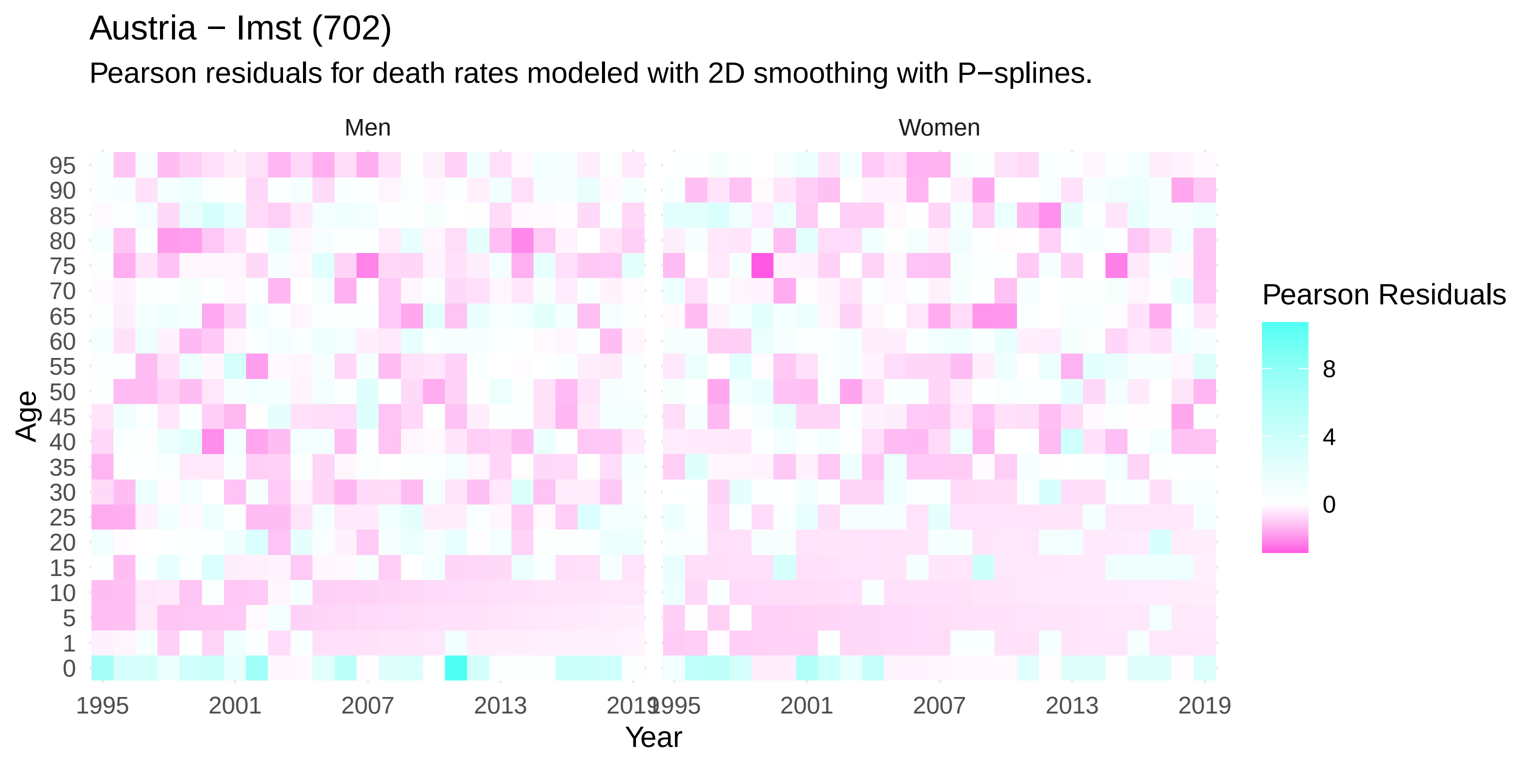

Austria − Imst (702)
Pearson residuals for death rates modeled with 2D smoothing with P−splines.
Men
Women
Age
95
90
85
80
75
70
65
60
55
50
45
40
35
30
25
20
15
10
5
1
0
1995
2001
2007
2013
2019
Year
Pearson Residuals
8
4
0

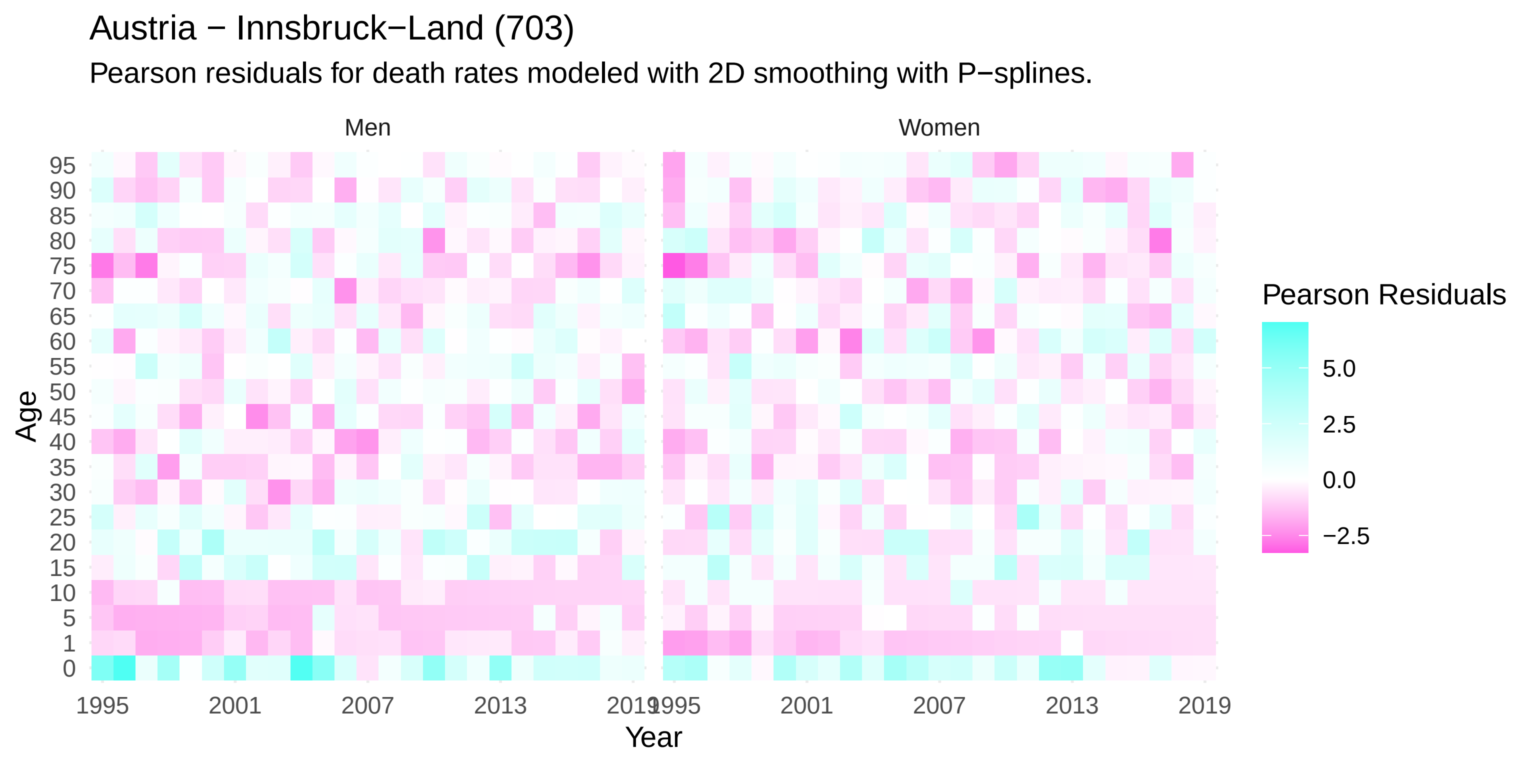

Austria − Innsbruck−Land (703)
Pearson residuals for death rates modeled with 2D smoothing with P−splines.
Men
Women
Age
95
90
85
80
75
70
65
60
55
50
45
40
35
30
25
20
15
10
5
1
0
1995
2001
2007
2013
2019
Year
Pearson Residuals
5.0
2.5
0.0
−2.5

Austria − Kitzbühel (704)

Pearson residuals for death rates modeled with 2D smoothing with P−splines.

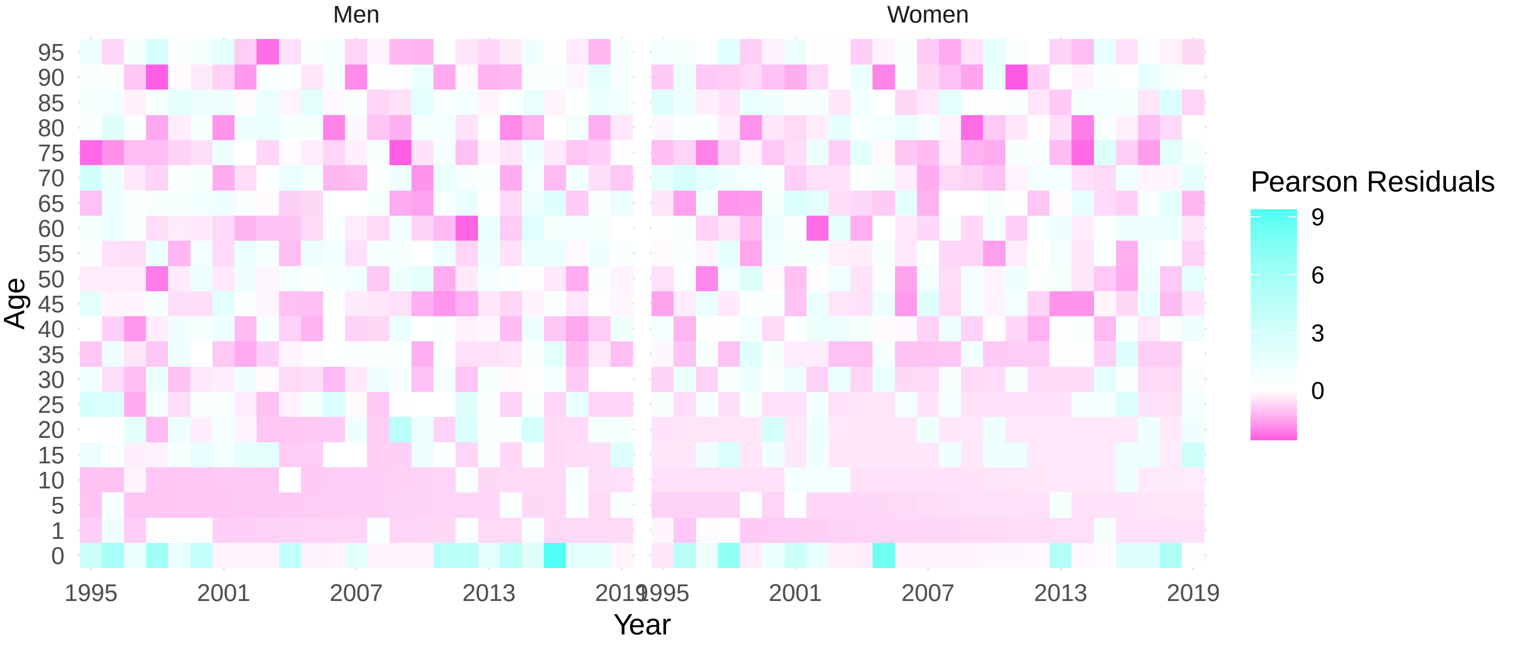

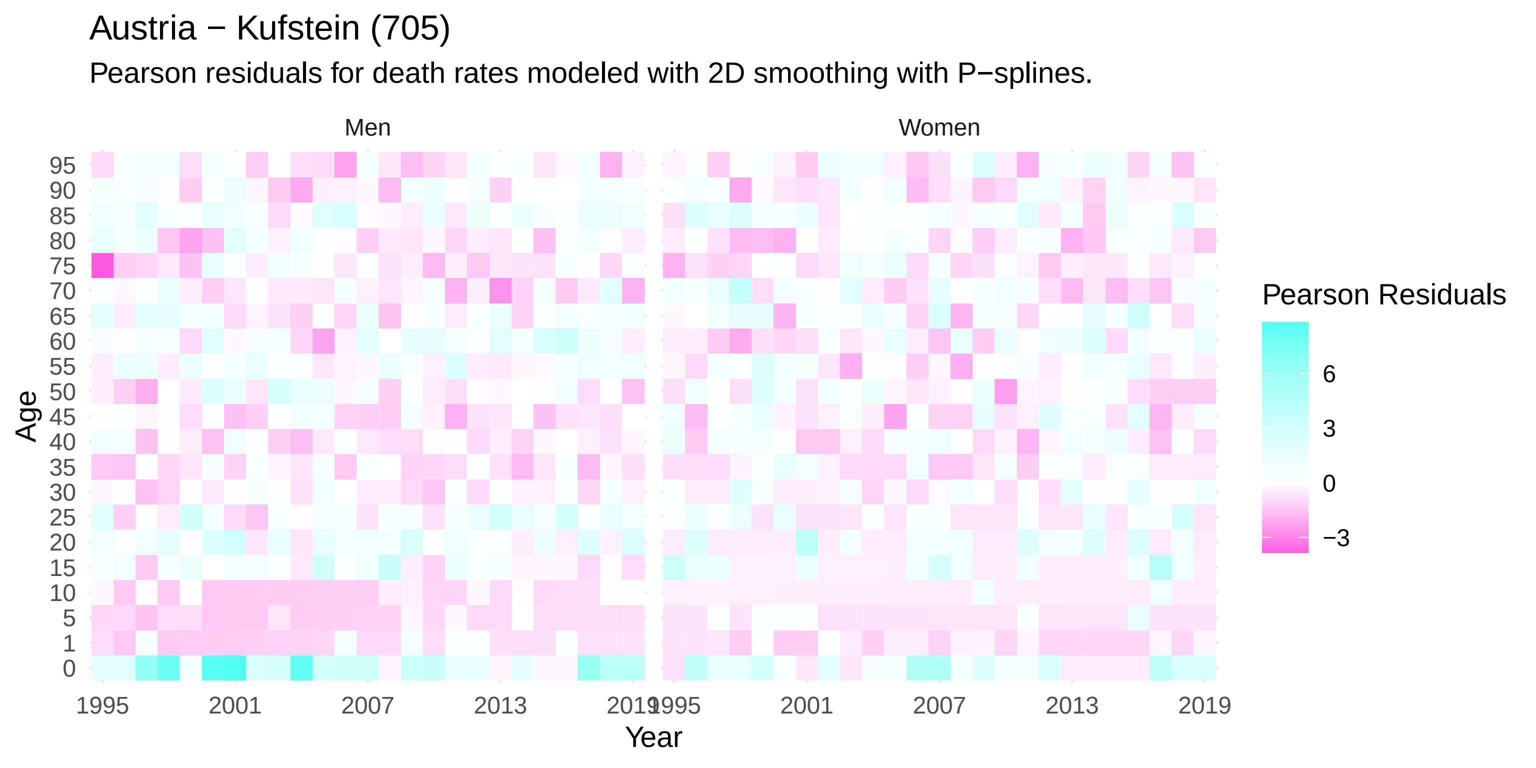

Austria − Kufstein (705)
Pearson residuals for death rates modeled with 2D smoothing with P−splines.
Men
Women
Age
95
90
85
80
75
70
65
60
55
50
45
40
35
30
25
20
15
10
5
1
0
1995
2001
2007
2013
2019
Year
Pearson Residuals
6
3
0
−3

Austria − Landeck (706)

Pearson residuals for death rates modeled with 2D smoothing with P−splines.

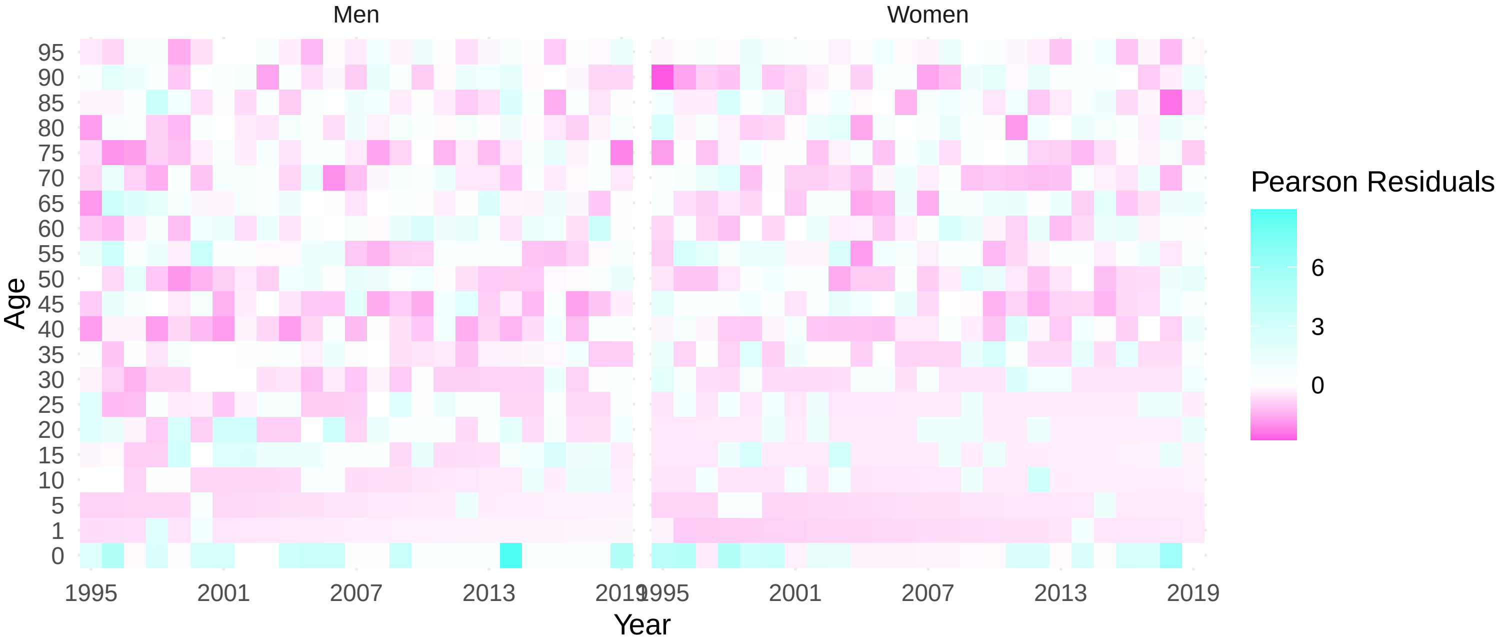

Austria − Lienz (707)

Pearson residuals for death rates modeled with 2D smoothing with P−splines.

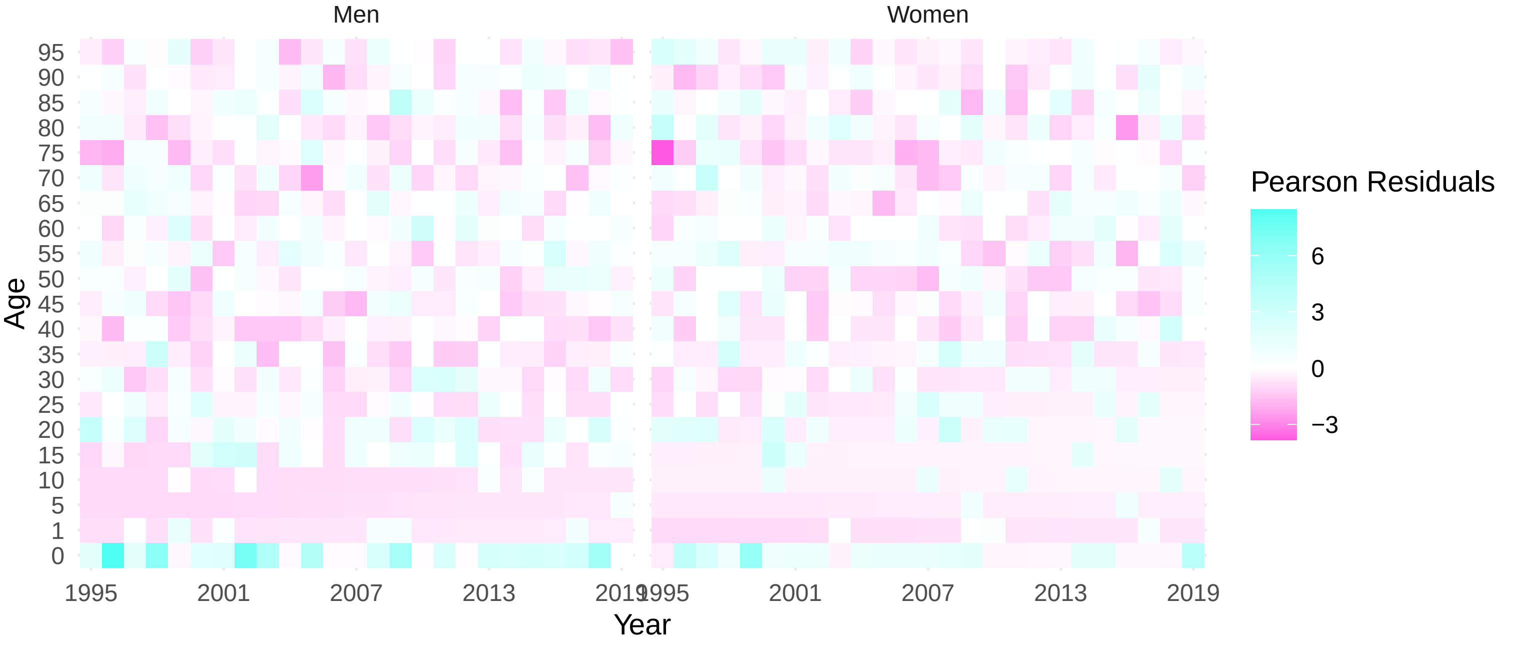

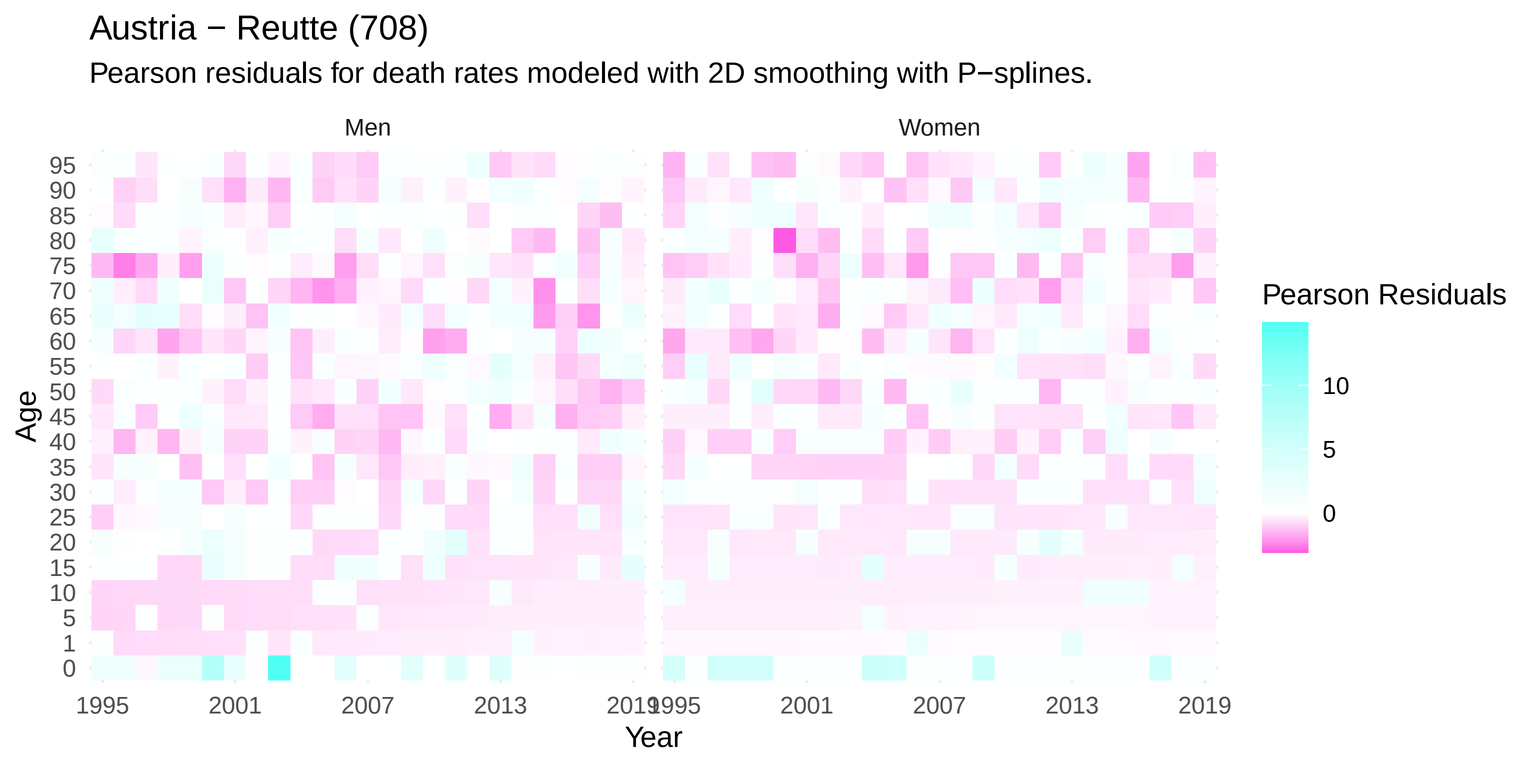

Austria − Reutte (708)
Pearson residuals for death rates modeled with 2D smoothing with P−splines.
Men
Women
Age
95
90
85
80
75
70
65
60
55
50
45
40
35
30
25
20
15
10
5
1
0
1995
2001
2007
2013
2019
Year
Pearson Residuals
10
5
0

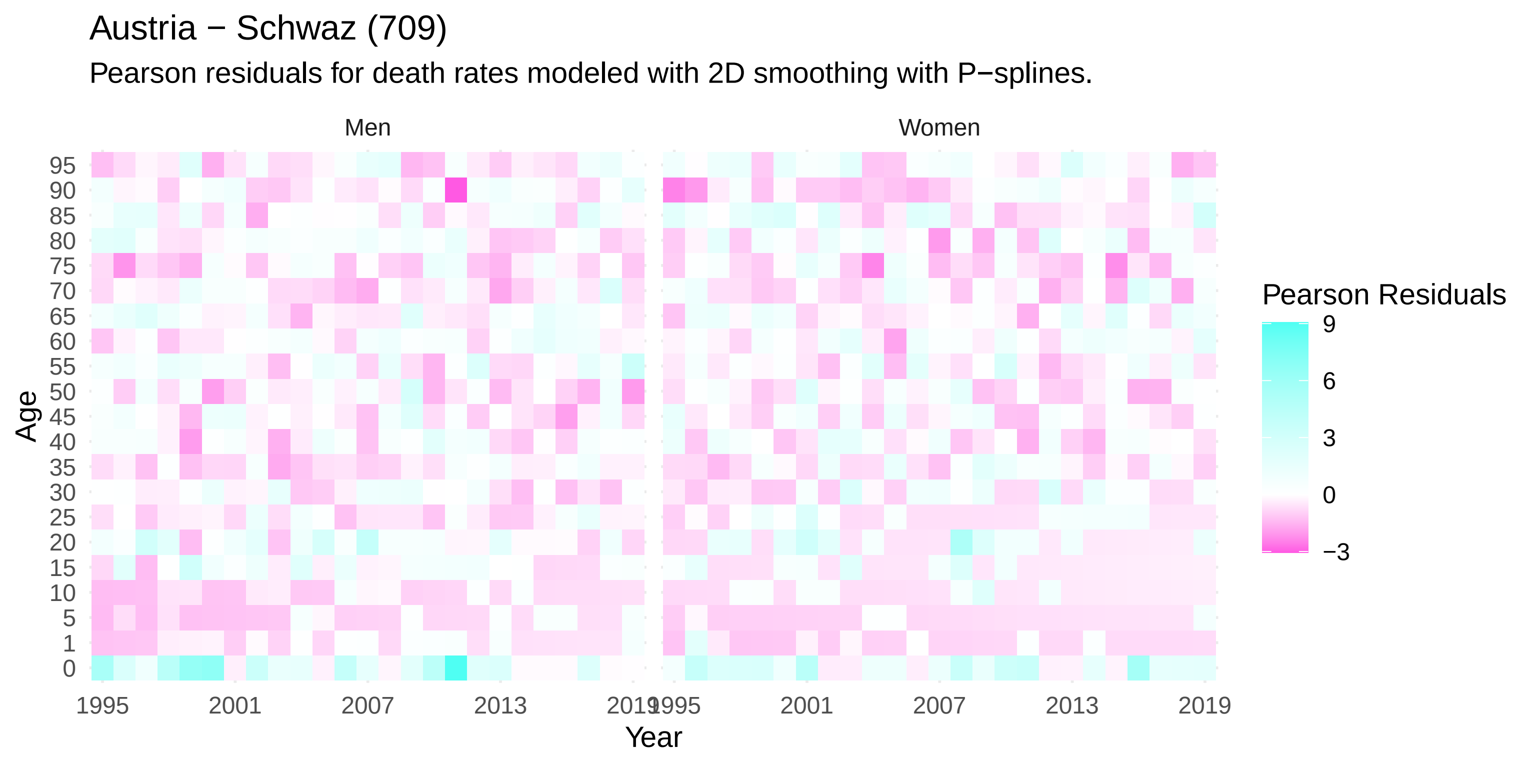

Austria − Schwaz (709)
Pearson residuals for death rates modeled with 2D smoothing with P−splines.
Men
Women
Age
95
90
85
80
75
70
65
60
55
50
45
40
35
30
25
20
15
10
5
1
0
1995
2001
2007
2013
2019
Year
Pearson Residuals
9
6
3
0
−3

Austria − Bludenz (801)

Pearson residuals for death rates modeled with 2D smoothing with P−splines.

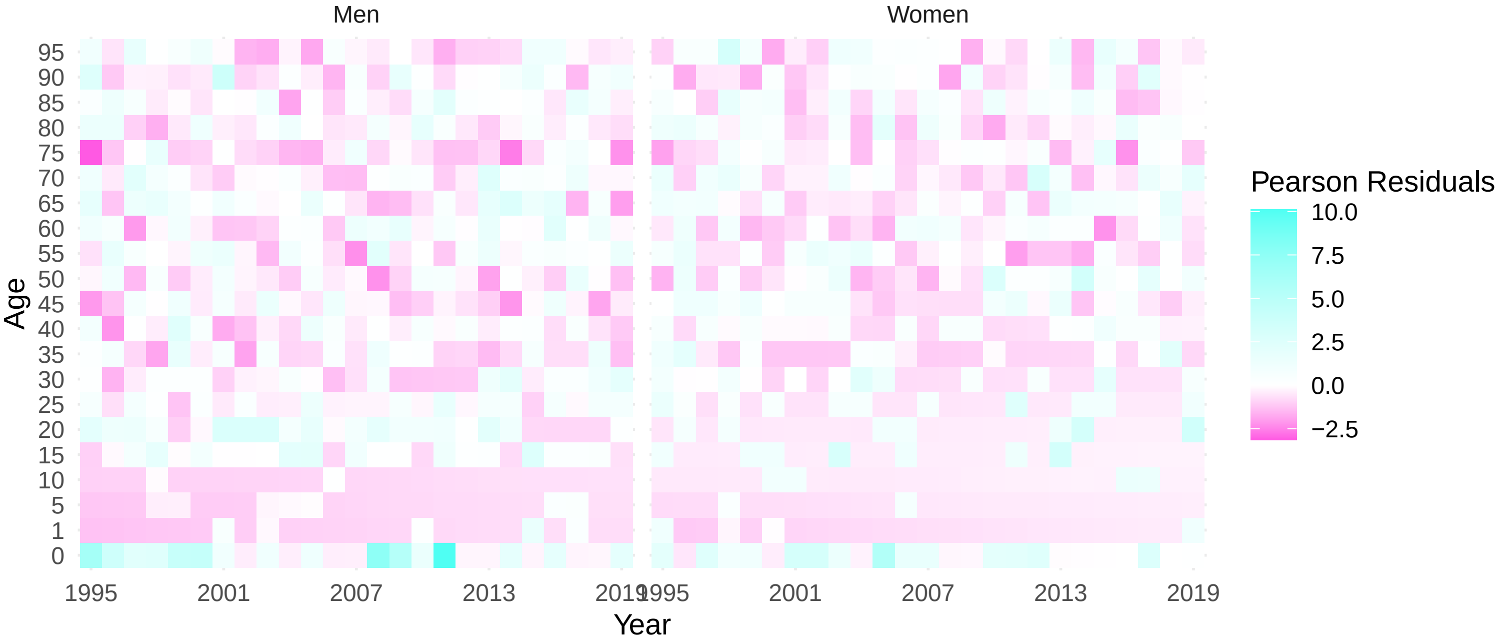

Austria − Bregenz (802)

Pearson residuals for death rates modeled with 2D smoothing with P−splines.

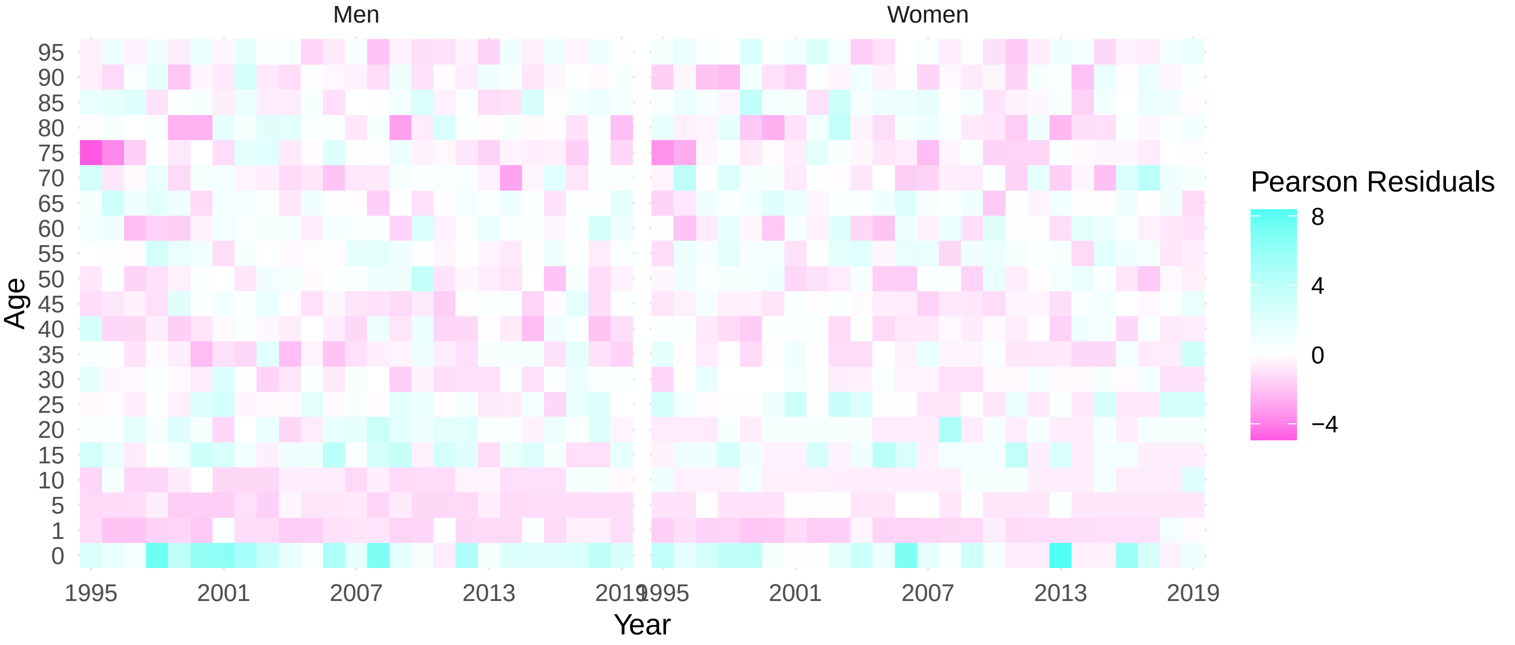

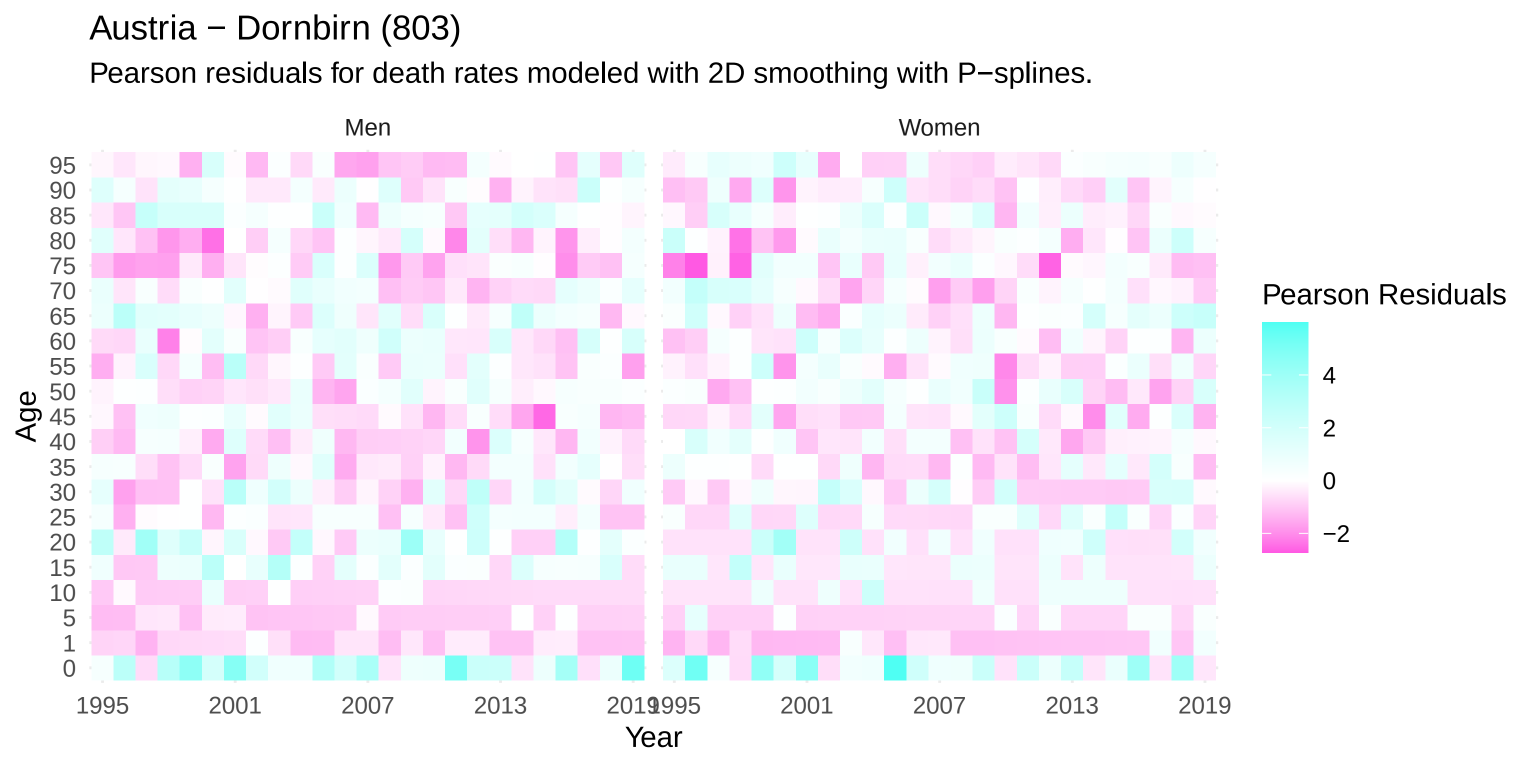

Austria − Dornbirn (803)
Pearson residuals for death rates modeled with 2D smoothing with P−splines.
Men
Women
Age
95
90
85
80
75
70
65
60
55
50
45
40
35
30
25
20
15
10
5
1
0
1995
2001
2007
2013
2019
Year
Pearson Residuals
4
2
0
−2

Austria − Feldkirch (804)

Pearson residuals for death rates modeled with 2D smoothing with P−splines.

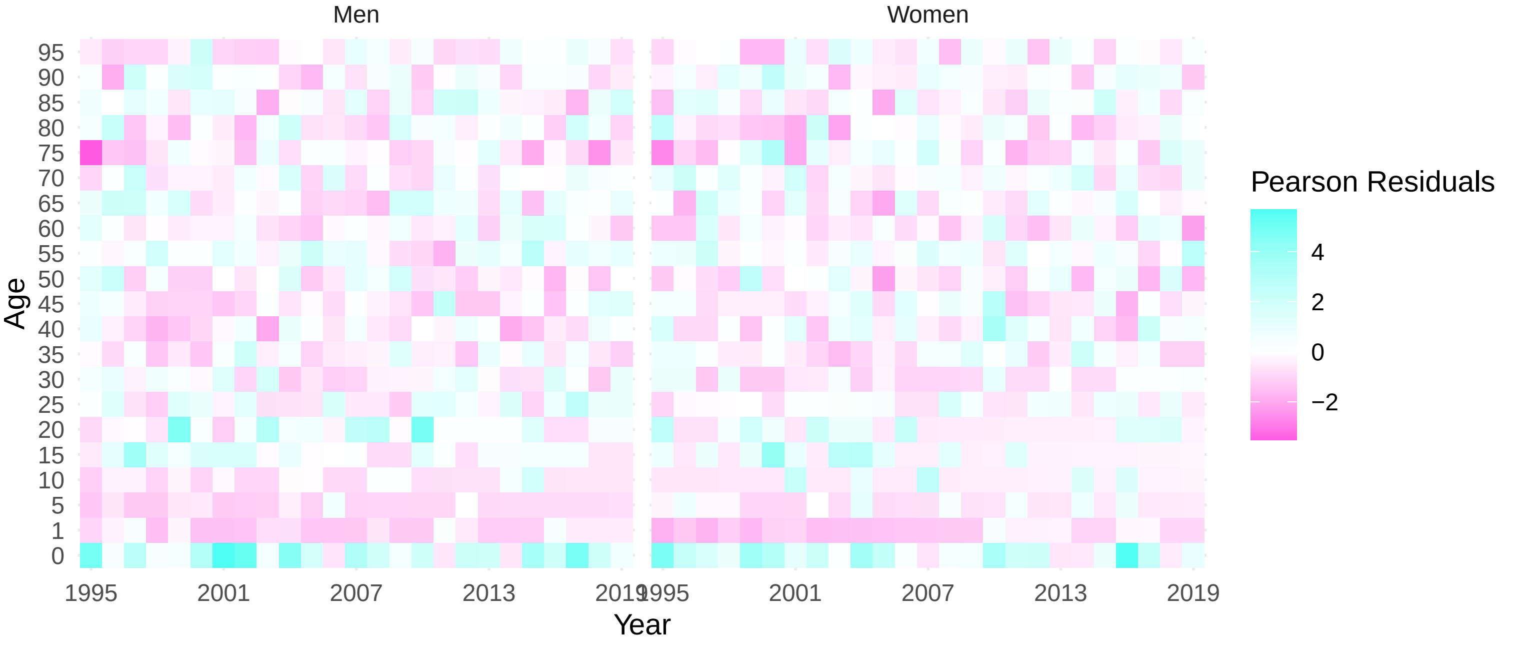

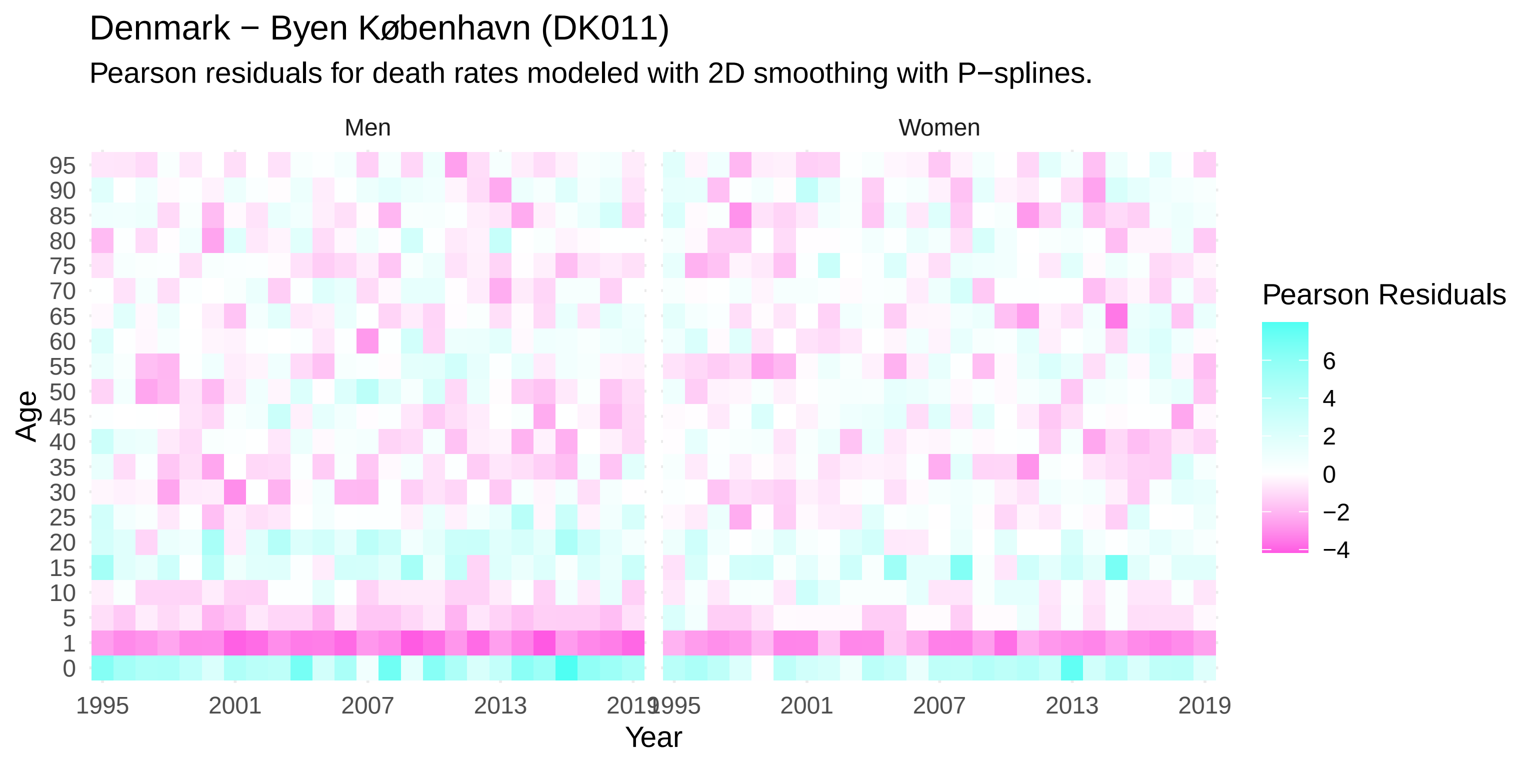
Denmark − Byen København (DK011)
Pearson residuals for death rates modeled with 2D smoothing with P−splines.
Men
Women
Age
95
90
85
80
75
70
65
60
55
50
45
40
35
30
25
20
15
10
5
1
0
1995
2001
2007
2013
2019
Year
Pearson Residuals
6
4
2
0
−2
−4

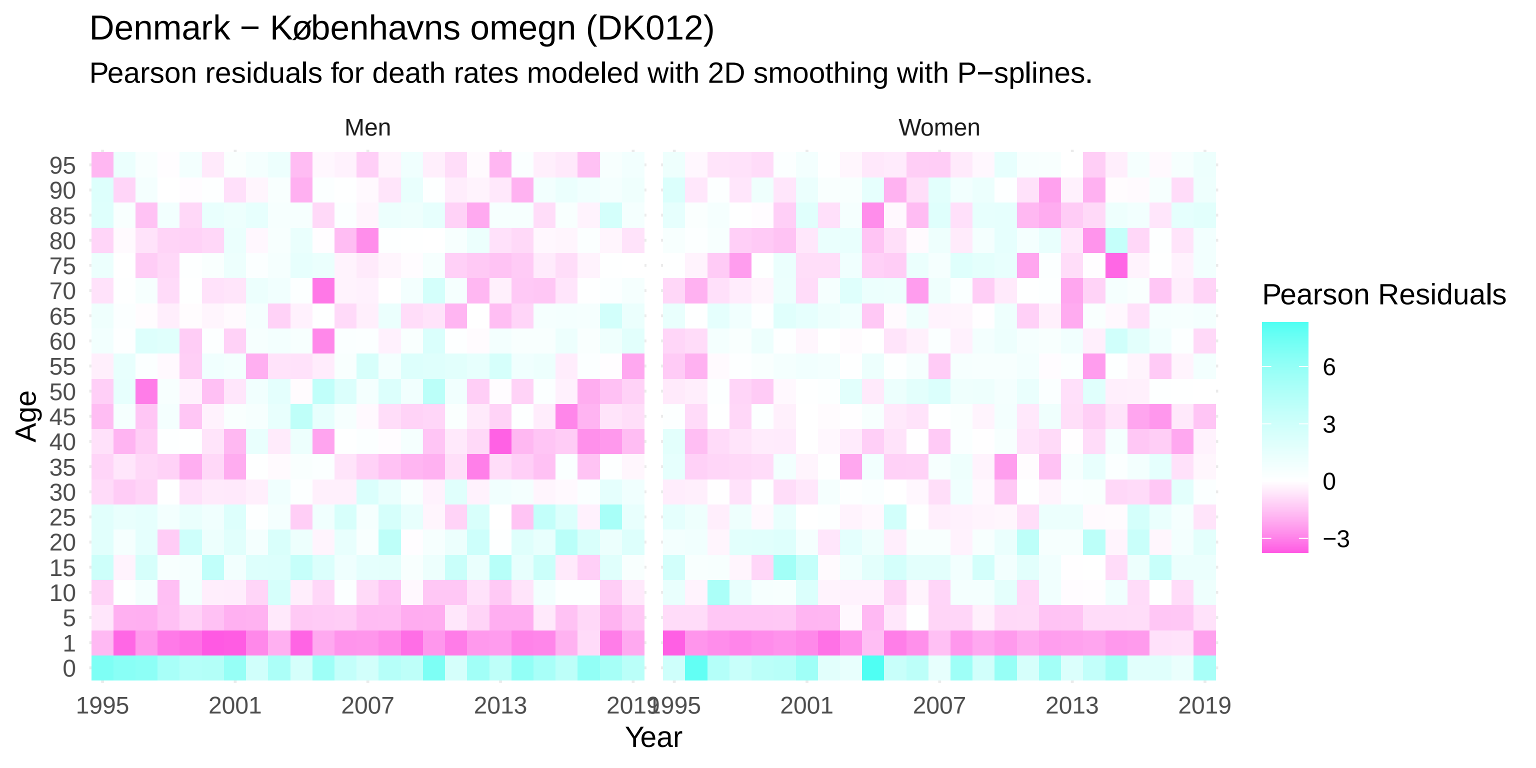
Denmark − Københavns omegn (DK012)
Pearson residuals for death rates modeled with 2D smoothing with P−splines.
Men
Women
Age
95
90
85
80
75
70
65
60
55
50
45
40
35
30
25
20
15
10
5
1
0
1995
2001
2007
2013
2019
Year
Pearson Residuals
6
3
0
−3

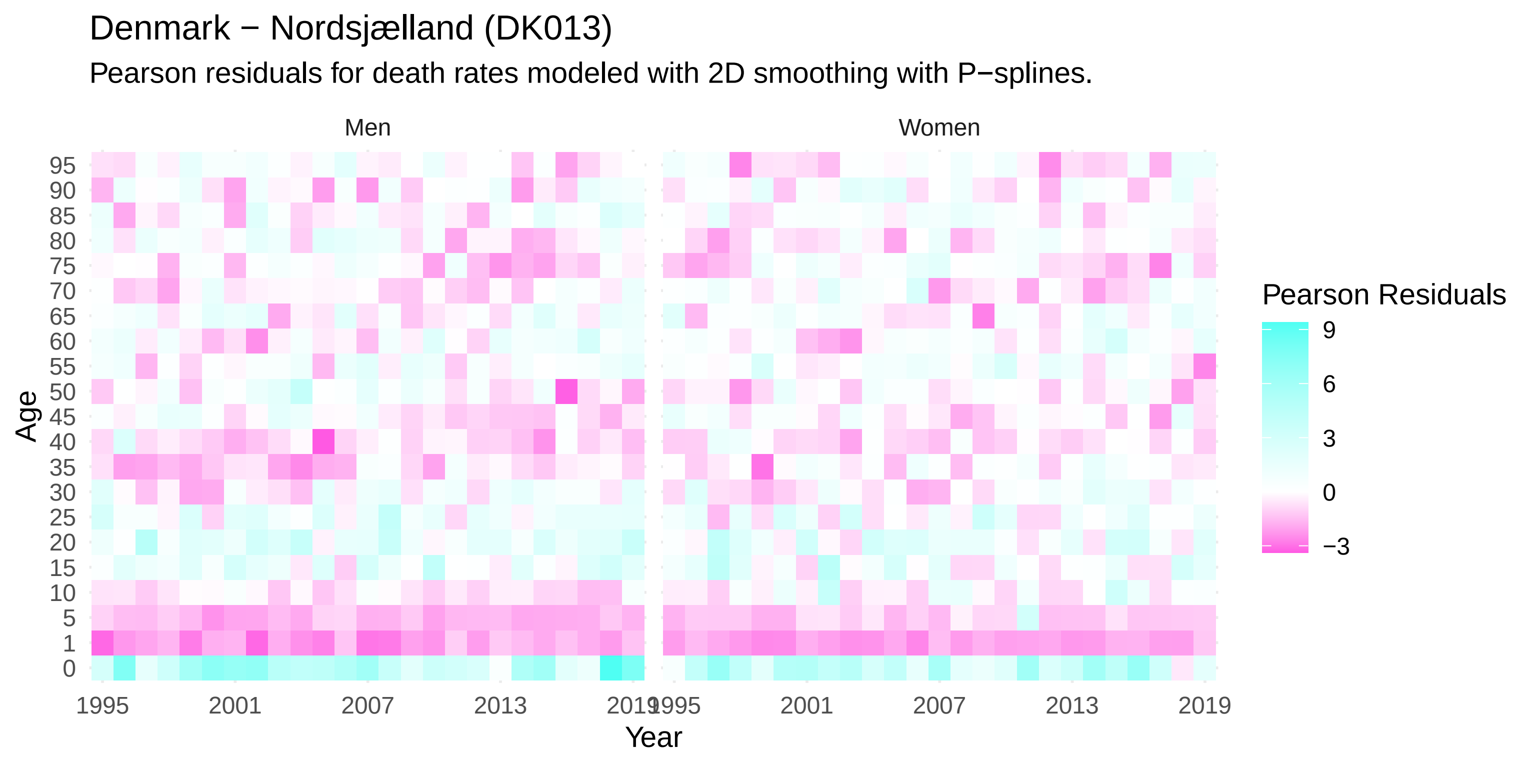

Denmark − Nordsjælland (DK013)
Pearson residuals for death rates modeled with 2D smoothing with P−splines.
Men
Women
Age
95
90
85
80
75
70
65
60
55
50
45
40
35
30
25
20
15
10
5
1
0
1995
2001
2007
2013
2019
Year
Pearson Residuals
9
6
3
0
−3

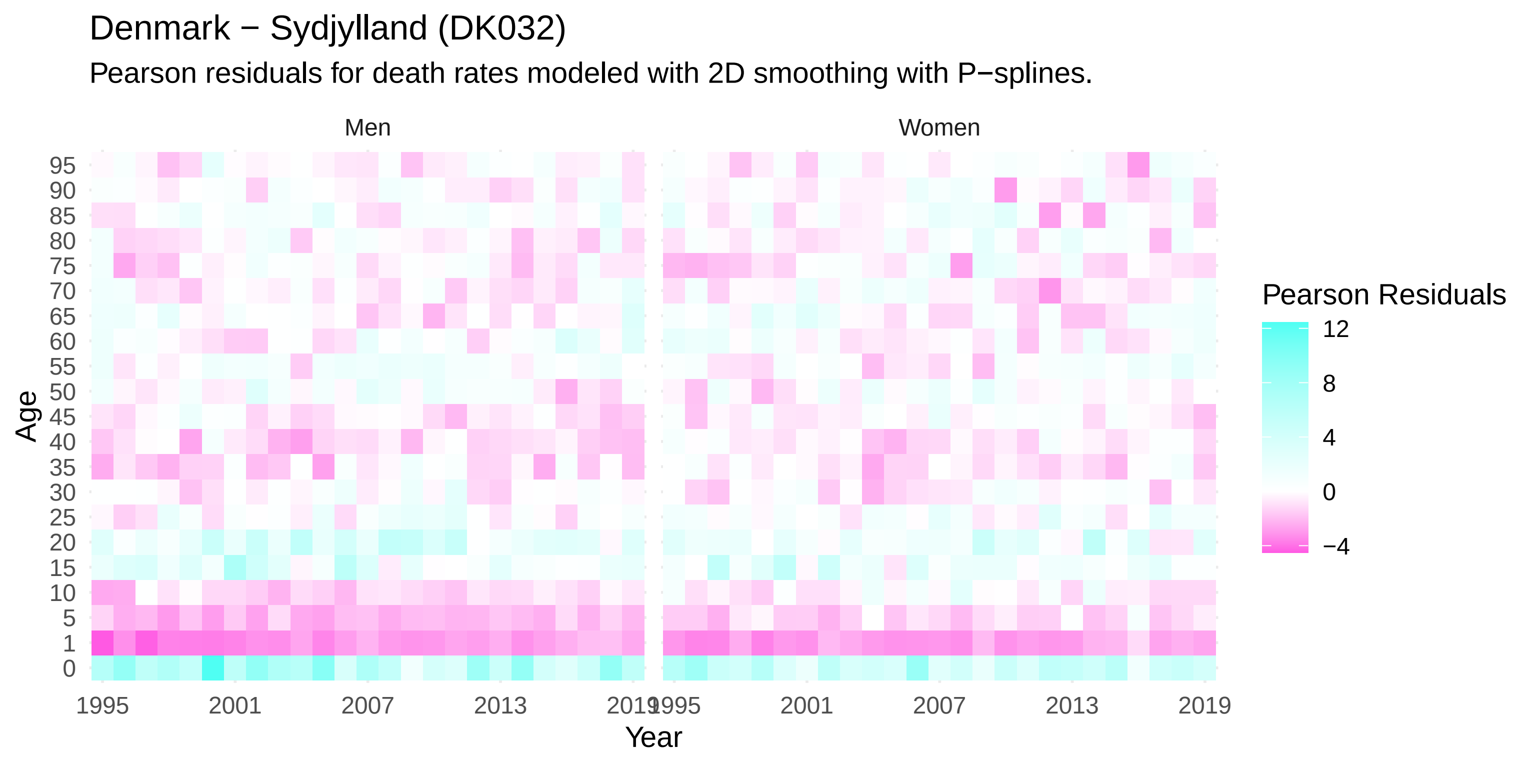

Denmark − Sydjylland (DK032)
Pearson residuals for death rates modeled with 2D smoothing with P−splines.
Men
Women
Age
95
90
85
80
75
70
65
60
55
50
45
40
35
30
25
20
15
10
5
1
0
1995
2001
2007
2013
2019
Year
Pearson Residuals
12
8
4
0
−4

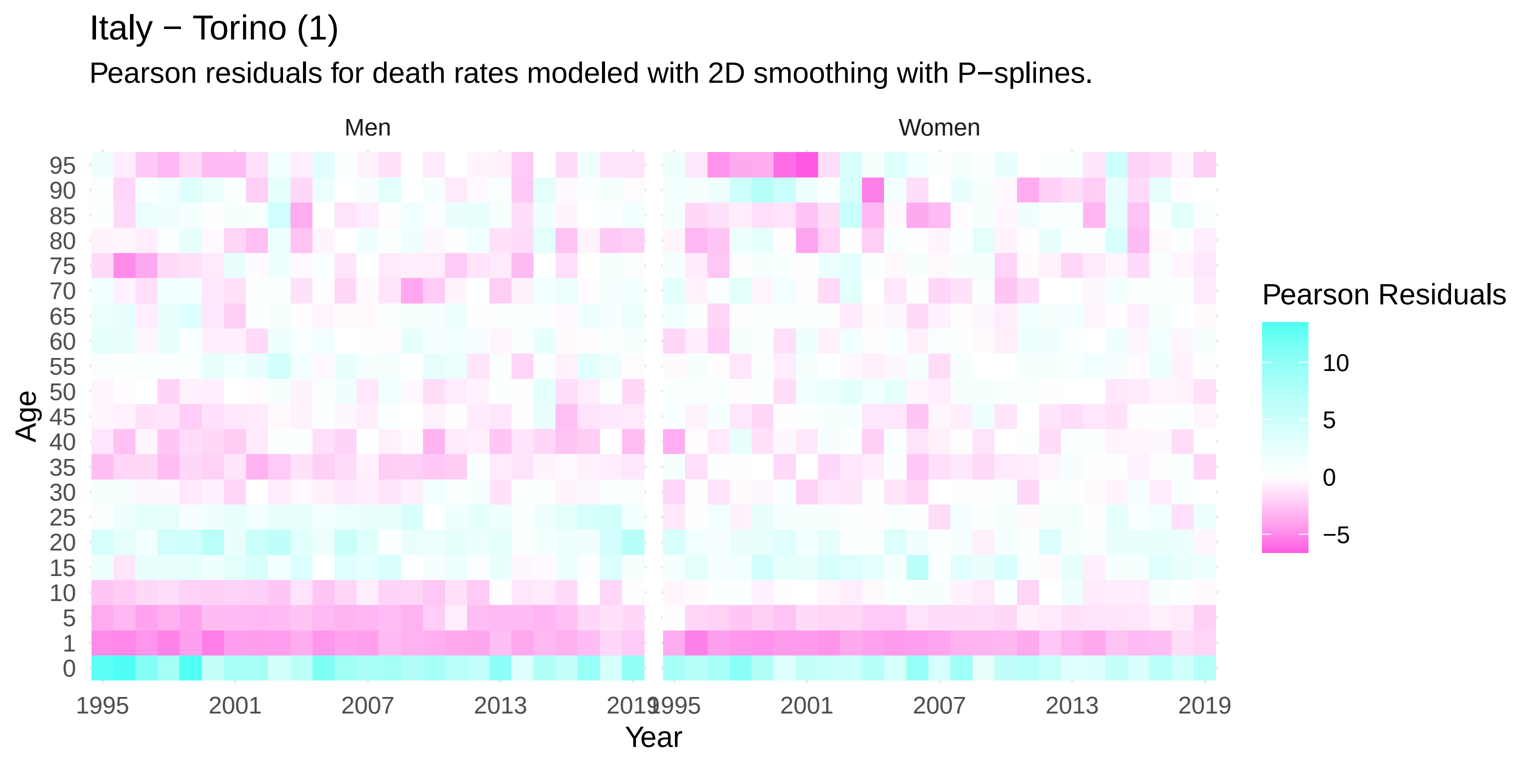
Italy − Torino (1)
Pearson residuals for death rates modeled with 2D smoothing with P−splines.
Men
Women
Age
Year
Pearson Residuals
10
5
0
−5
95
90
85
80
75
70
65
60
55
50
45
40
35
30
25
20
15
10
5
1
0
1995
2001
2007
2013
2019

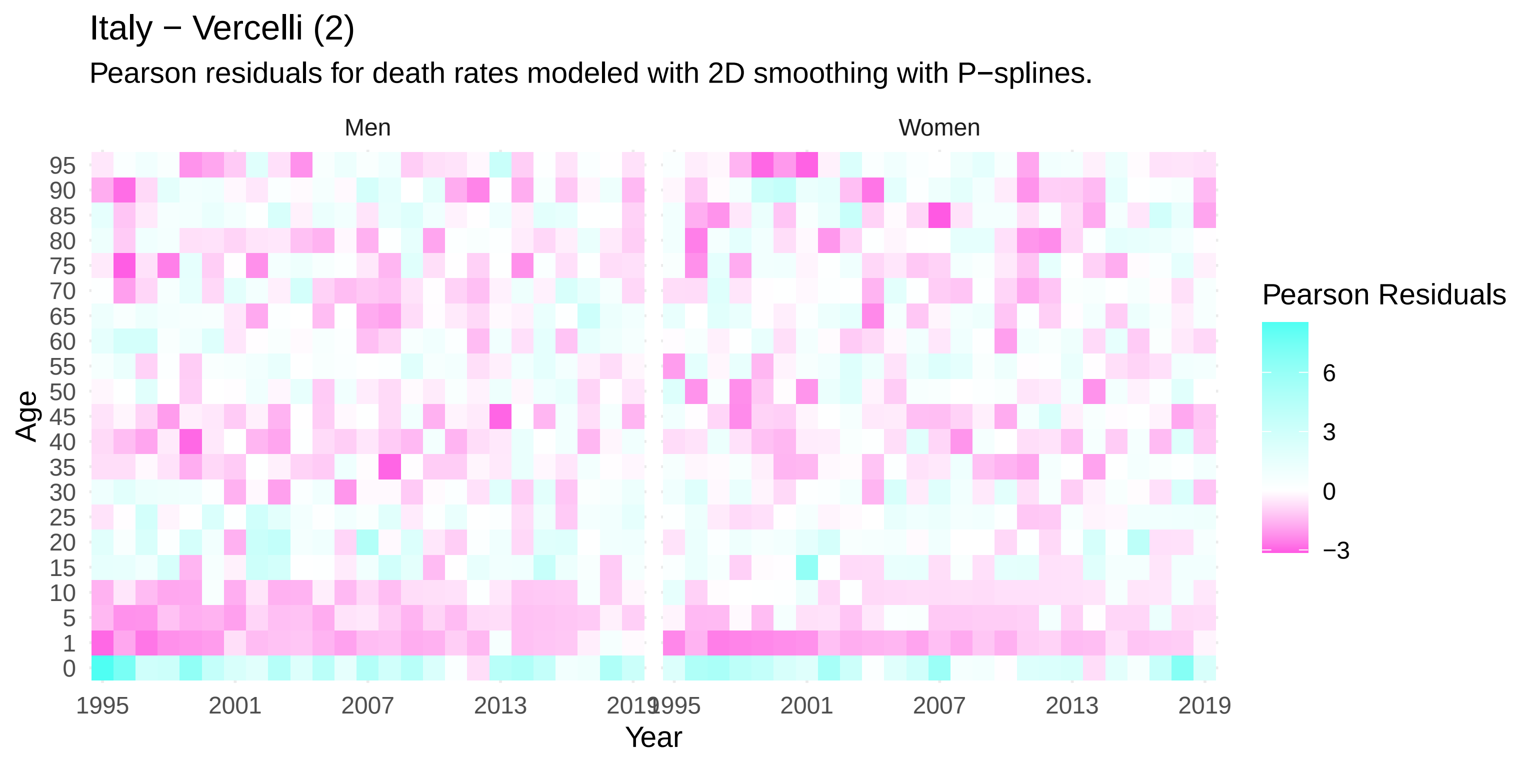

Italy − Vercelli (2)
Pearson residuals for death rates modeled with 2D smoothing with P−splines.
Men
Women
Age
95
90
85
80
75
70
65
60
55
50
45
40
35
30
25
20
15
10
5
1
0
1995
2001
2007
2013
2019
Year
Pearson Residuals
6
3
0
−3

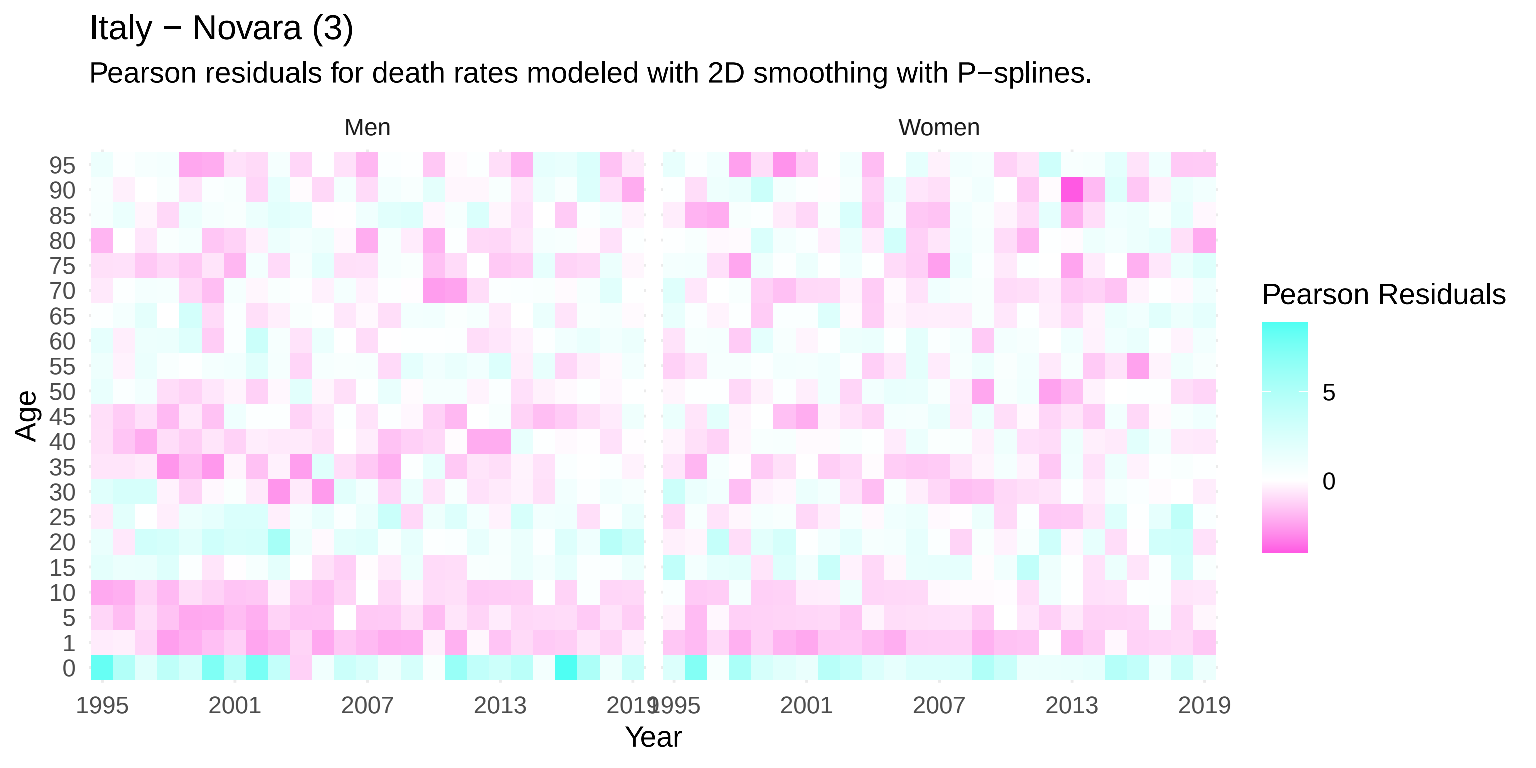

Italy − Novara (3)
Pearson residuals for death rates modeled with 2D smoothing with P−splines.
Men
Women
Age
95
90
85
80
75
70
65
60
55
50
45
40
35
30
25
20
15
10
5
1
0
1995
2001
2007
2013
2019
Year
Pearson Residuals
5
0

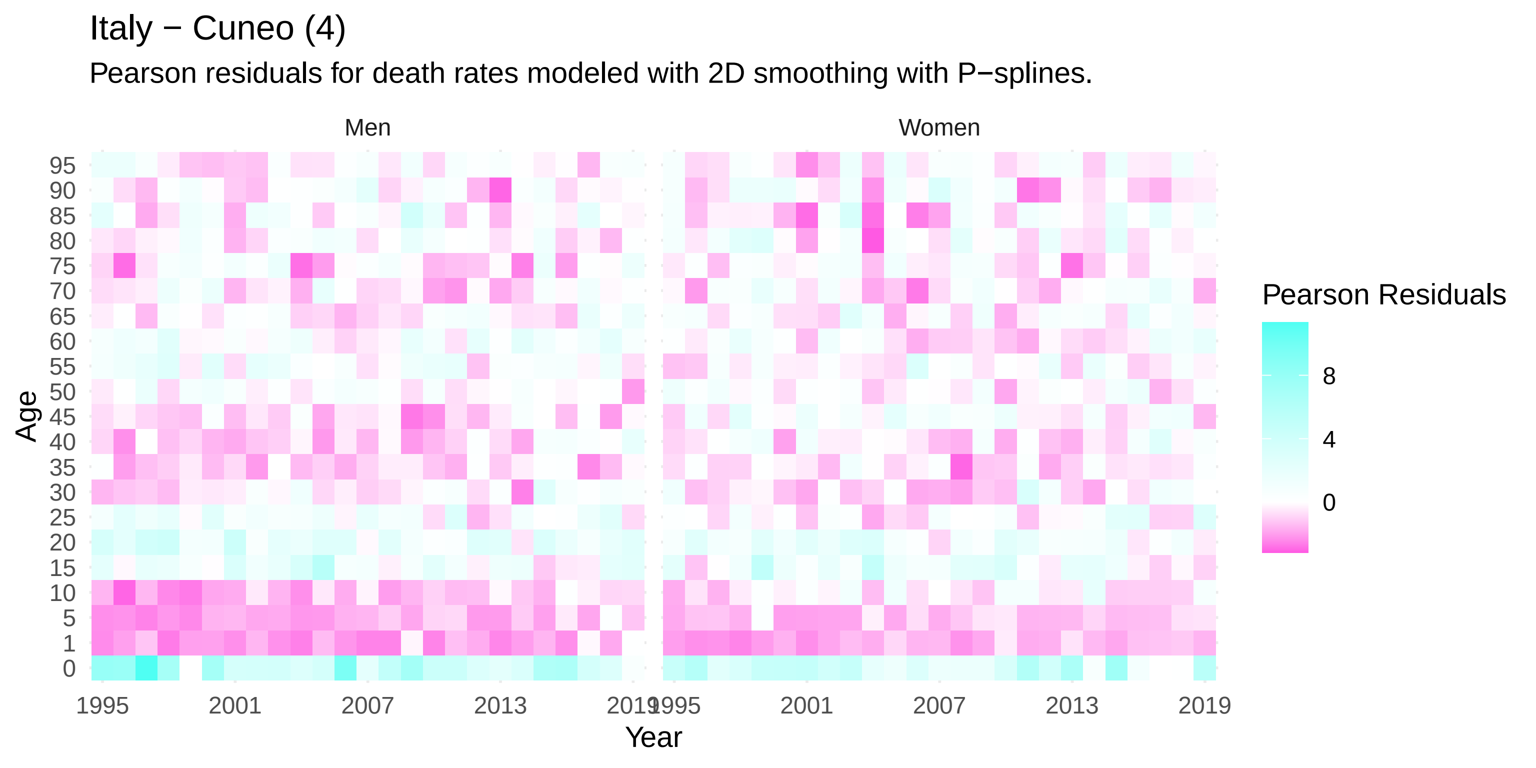
Italy − Cuneo (4)
Pearson residuals for death rates modeled with 2D smoothing with P−splines.
Men
Women
Age
95
90
85
80
75
70
65
60
55
50
45
40
35
30
25
20
15
10
5
1
0
1995
2001
2007
2013
2019
Year
Pearson Residuals
8
4
0

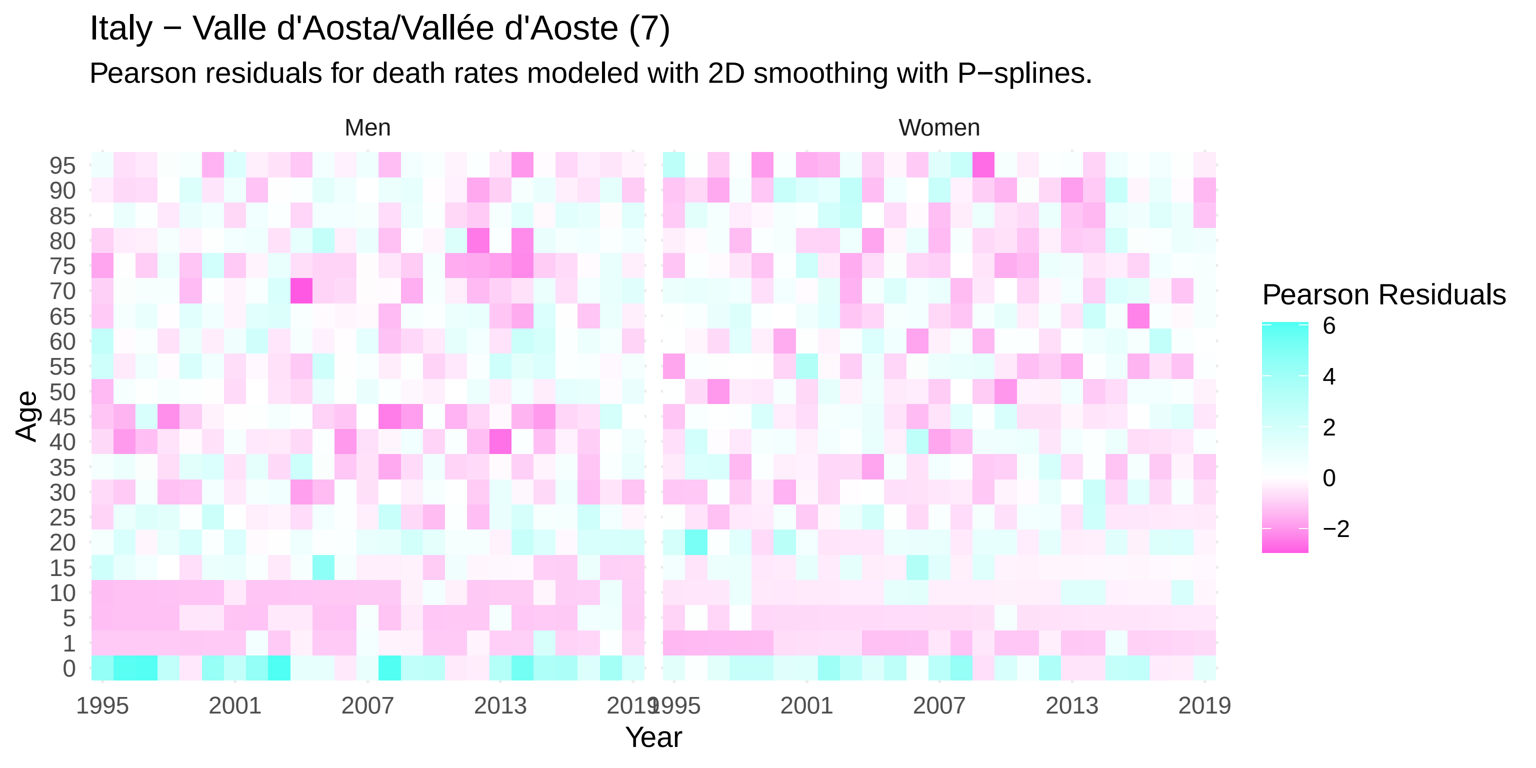

Italy − Valle d'Aosta/Vallée d'Aoste (7)
Pearson residuals for death rates modeled with 2D smoothing with P−splines.
Men
Women
Age
95
90
85
80
75
70
65
60
55
50
45
40
35
30
25
20
15
10
5
1
0
1995
2001
2007
2013
2019
Year
Pearson Residuals
6
4
2
0
−2

Italy − Imperia (8)

Pearson residuals for death rates modeled with 2D smoothing with P−splines.

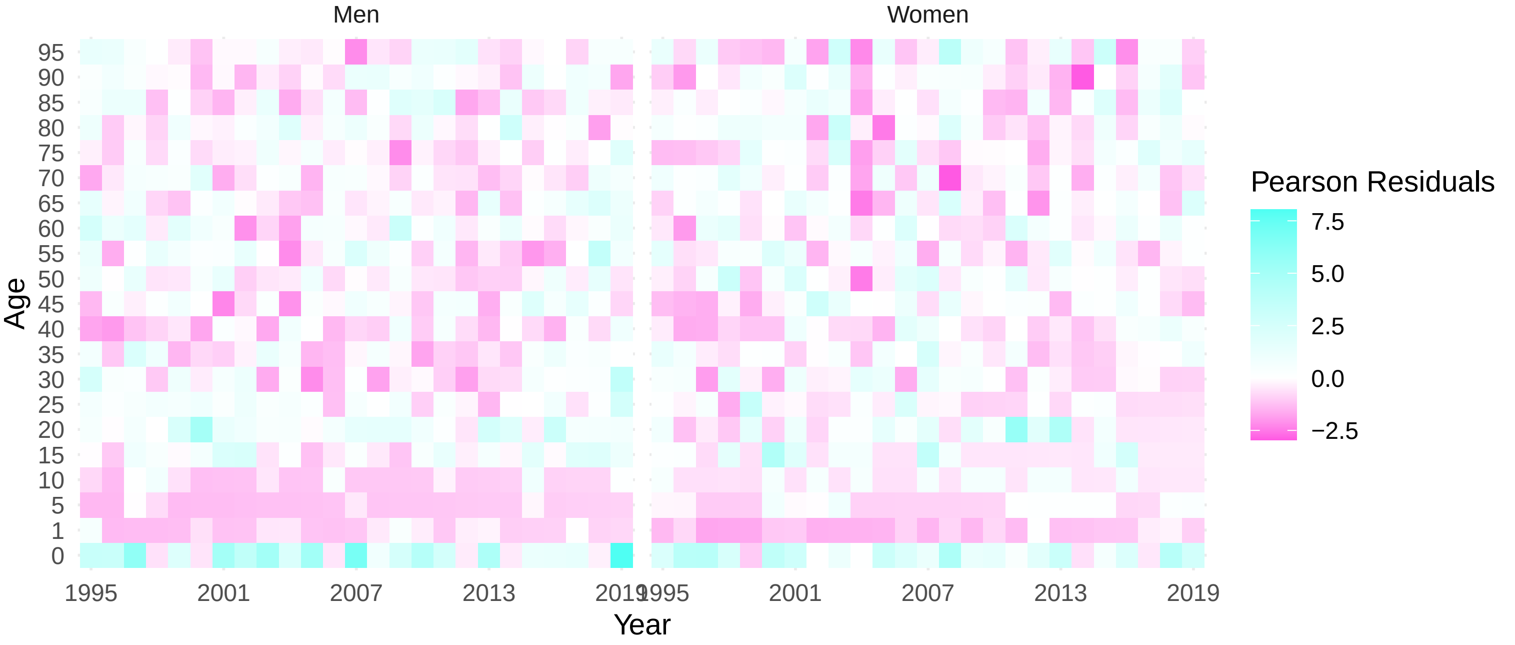

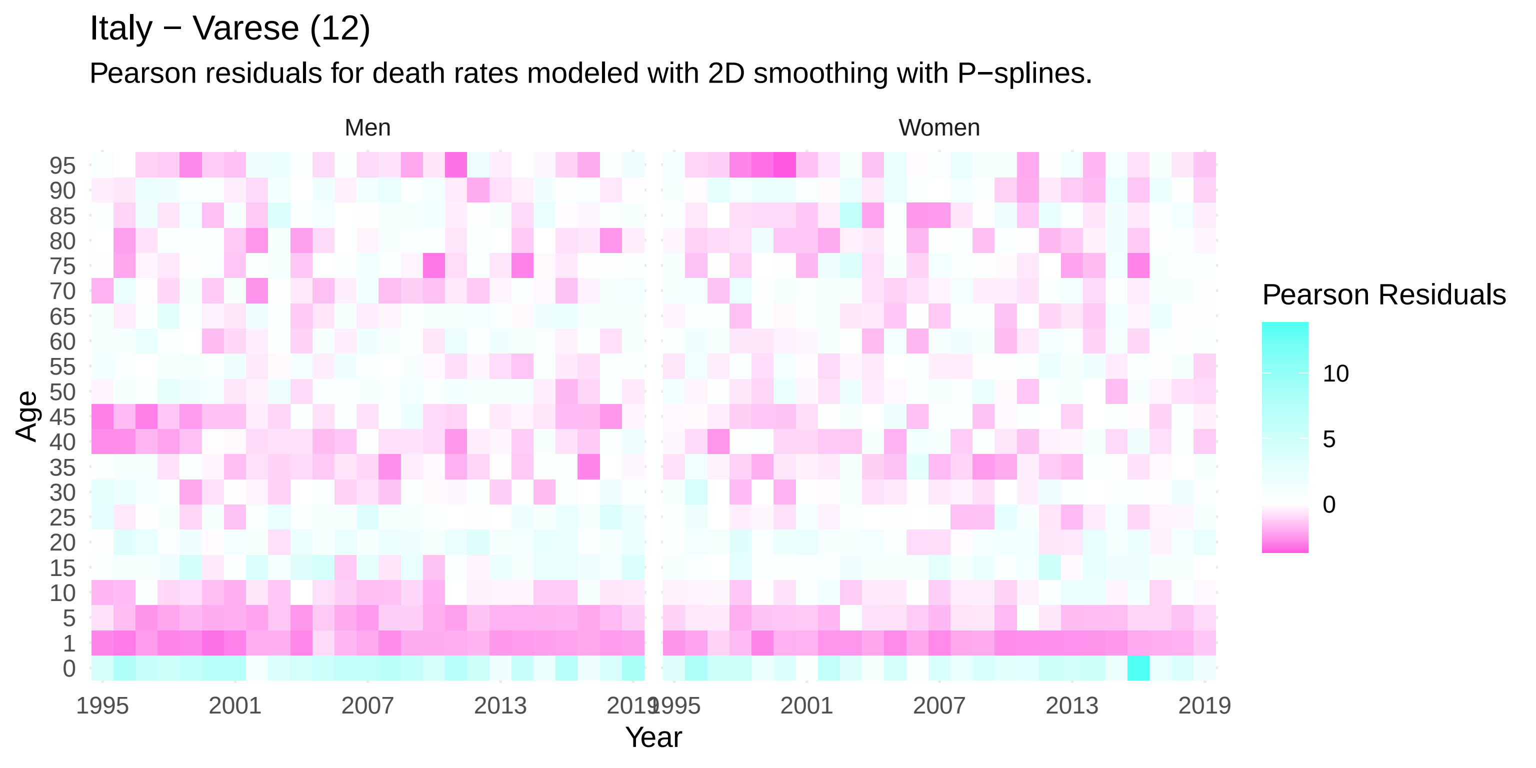

Italy − Varese (12)
Pearson residuals for death rates modeled with 2D smoothing with P−splines.
Men
Women
Age
95
90
85
80
75
70
65
60
55
50
45
40
35
30
25
20
15
10
5
1
0
1995
2001
2007
2013
2019
Year
Pearson Residuals
10
5
0

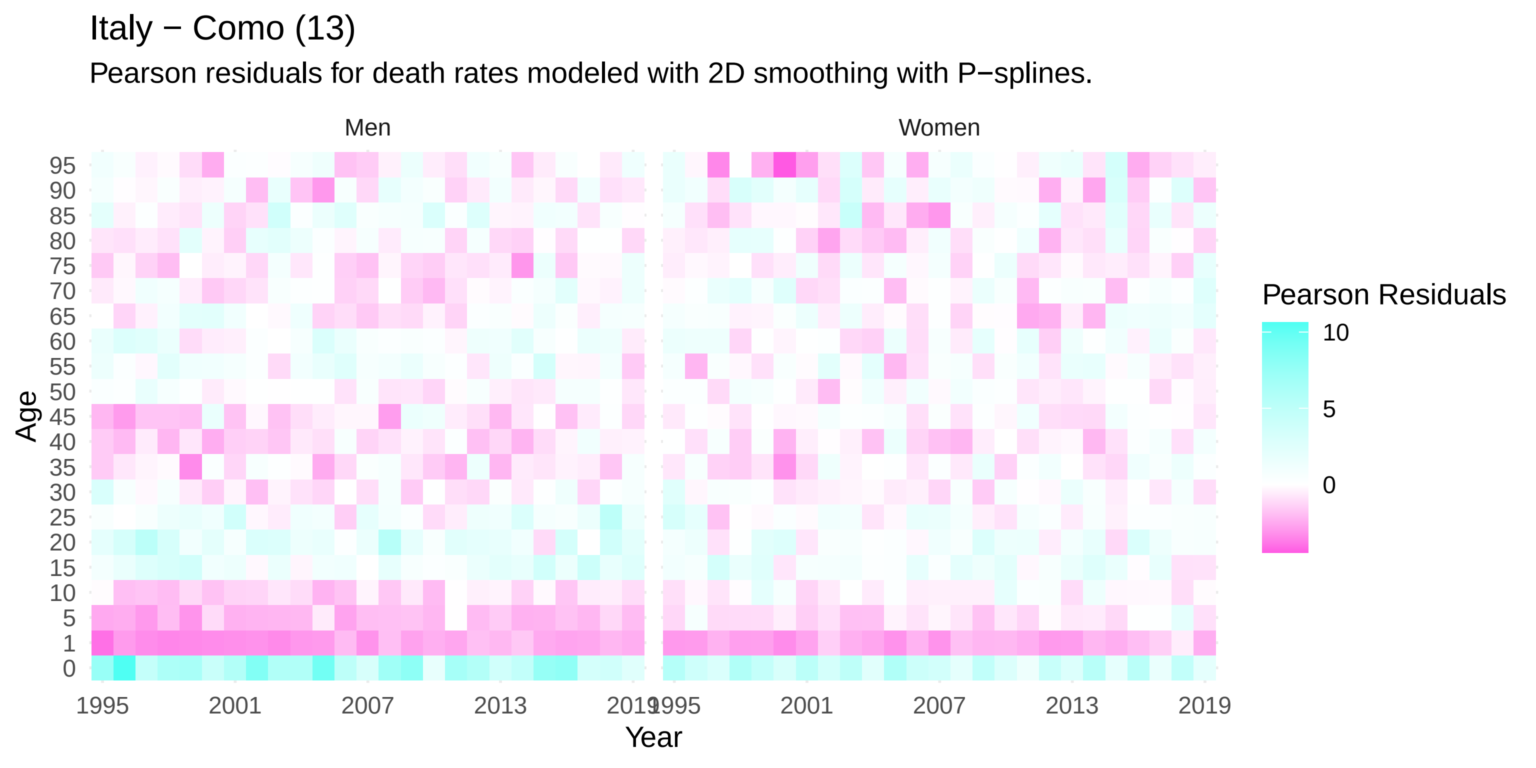
Italy − Como (13)
Pearson residuals for death rates modeled with 2D smoothing with P−splines.
Men
Women
Age
95
90
85
80
75
70
65
60
55
50
45
40
35
30
25
20
15
10
5
1
0
1995
2001
2007
2013
2019
Year
Pearson Residuals
10
5
0

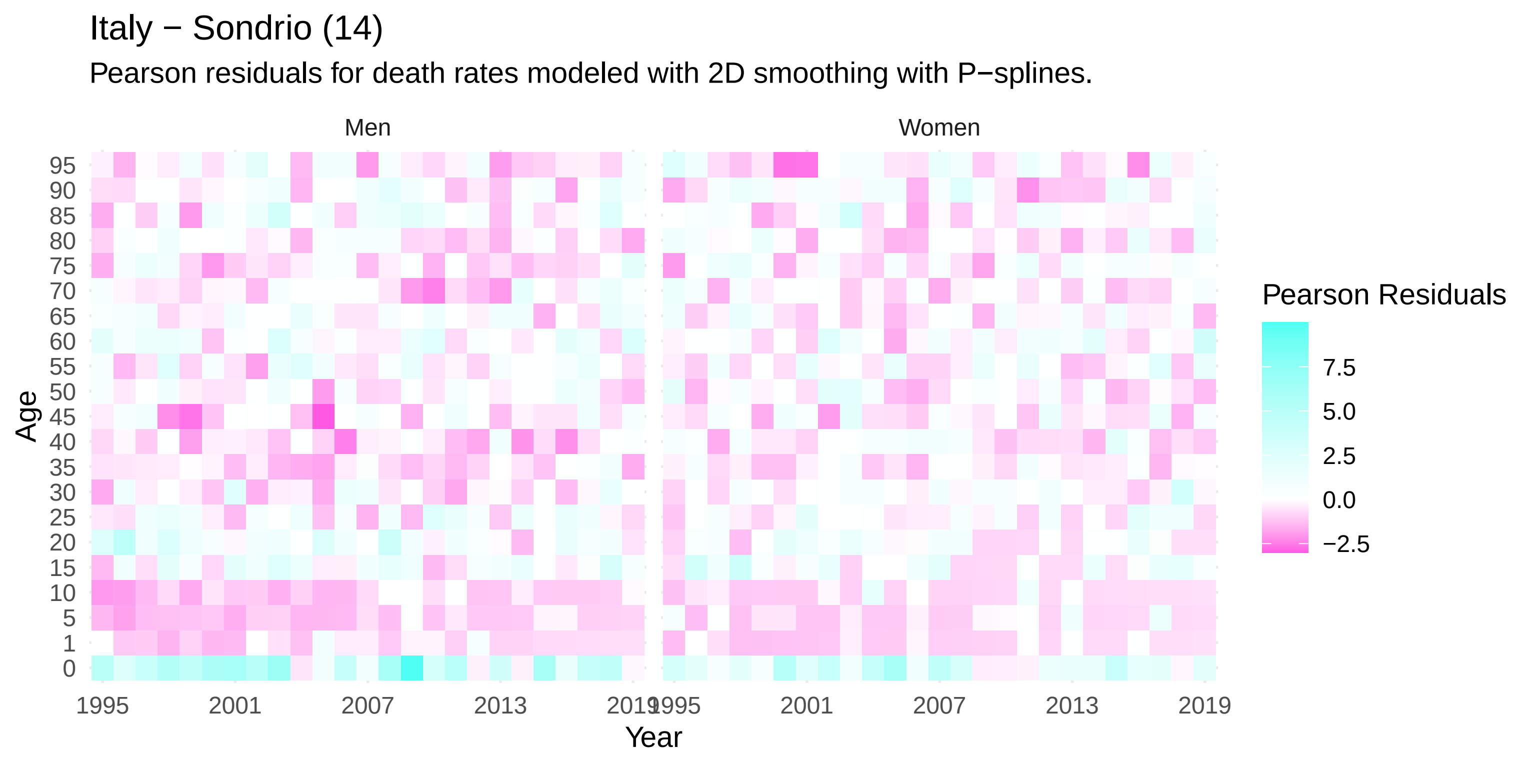
Italy − Sondrio (14)
Pearson residuals for death rates modeled with 2D smoothing with P−splines.
Men
Women
Age
95
90
85
80
75
70
65
60
55
50
45
40
35
30
25
20
15
10
5
1
0
1995
2001
2007
2013
2019
Year
Pearson Residuals
7.5
5.0
2.5
0.0
−2.5

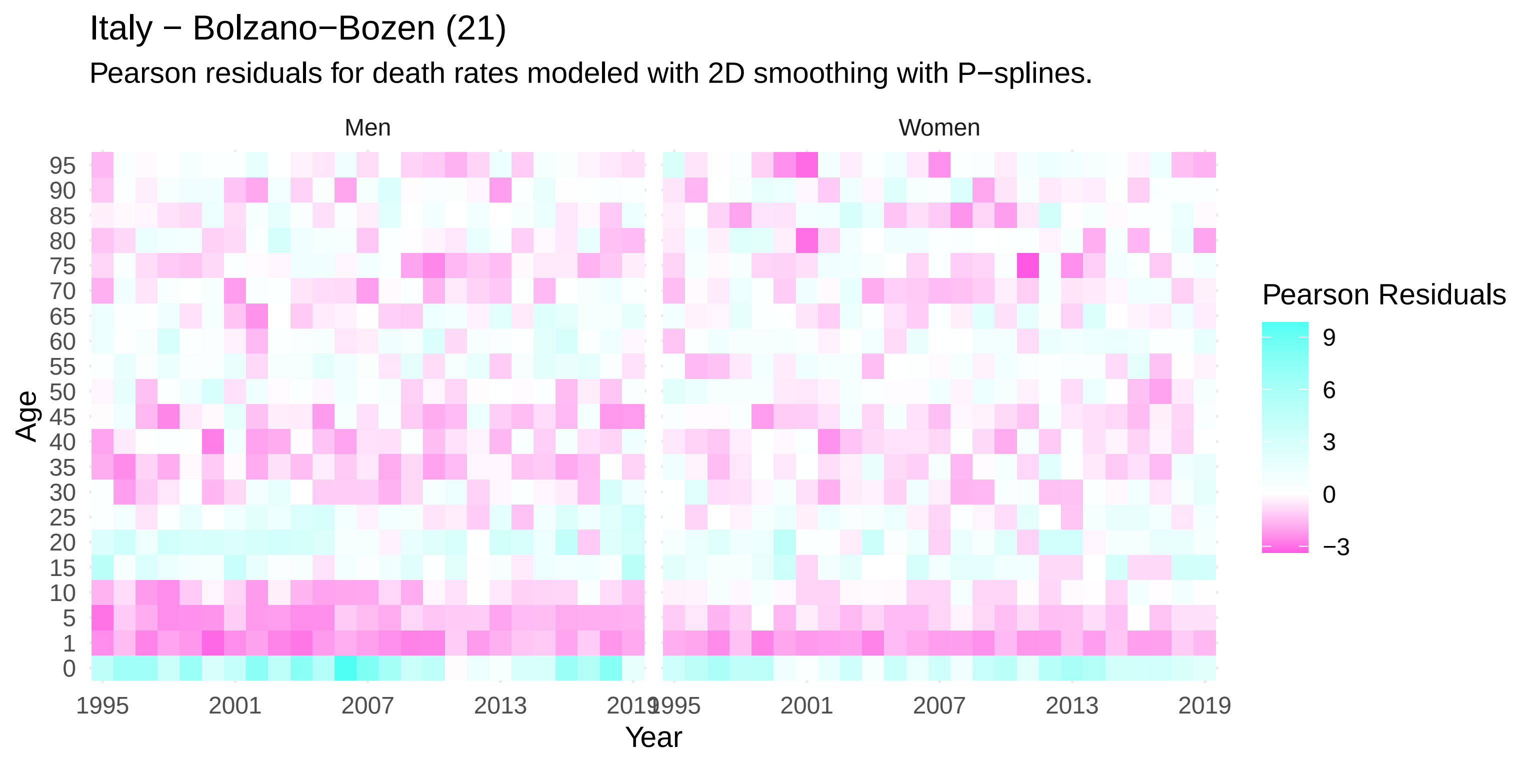

Italy − Bolzano−Bozen (21)
Pearson residuals for death rates modeled with 2D smoothing with P−splines.
Men
Women
Age
95
90
85
80
75
70
65
60
55
50
45
40
35
30
25
20
15
10
5
1
0
1995
2001
2007
2013
2019
Year
Pearson Residuals
9
6
3
0
−3

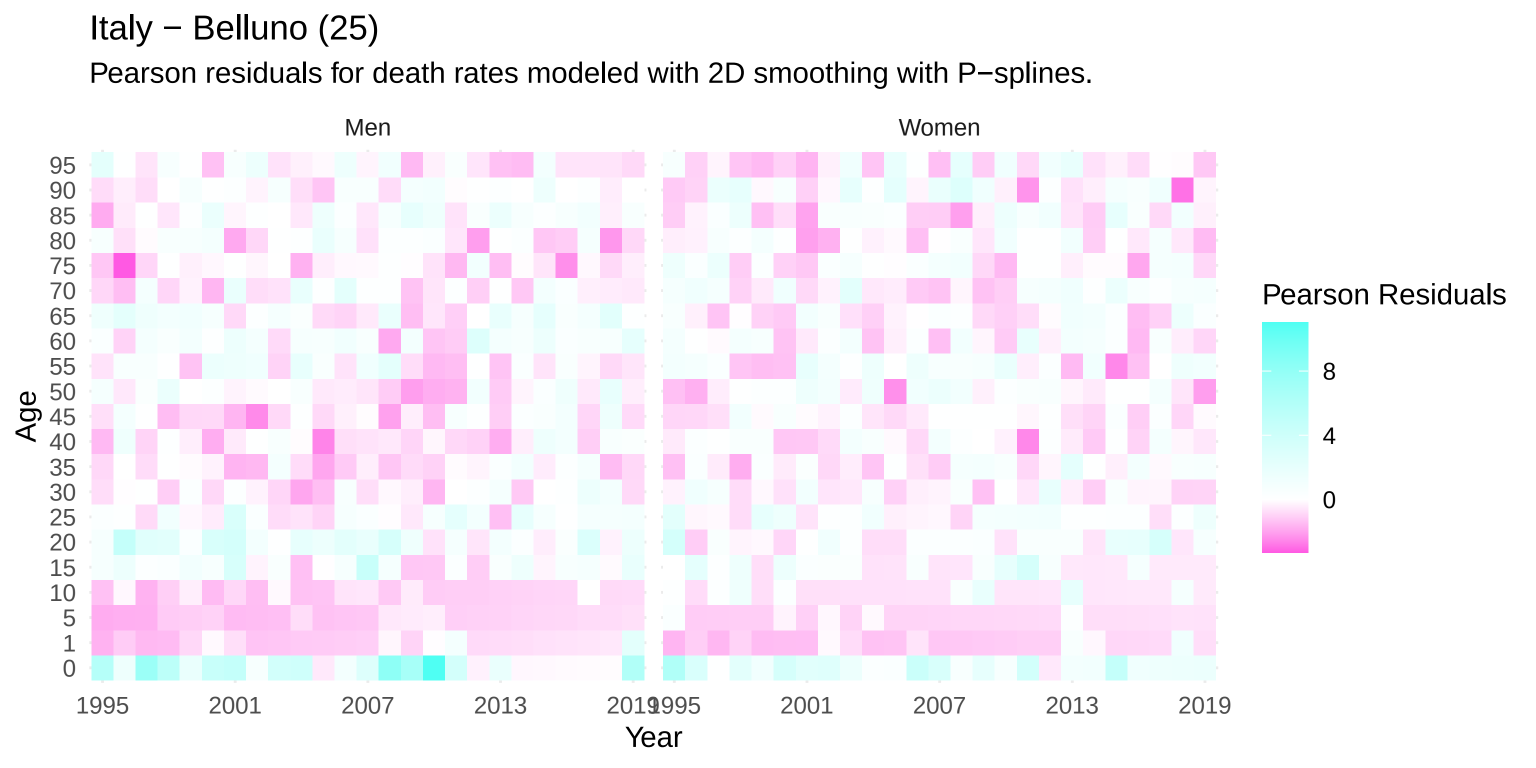
Italy − Belluno (25)
Pearson residuals for death rates modeled with 2D smoothing with P−splines.
Men
Women
Age
95
90
85
80
75
70
65
60
55
50
45
40
35
30
25
20
15
10
5
1
0
1995
2001
2007
2013
2019
Year
Pearson Residuals
8
4
0

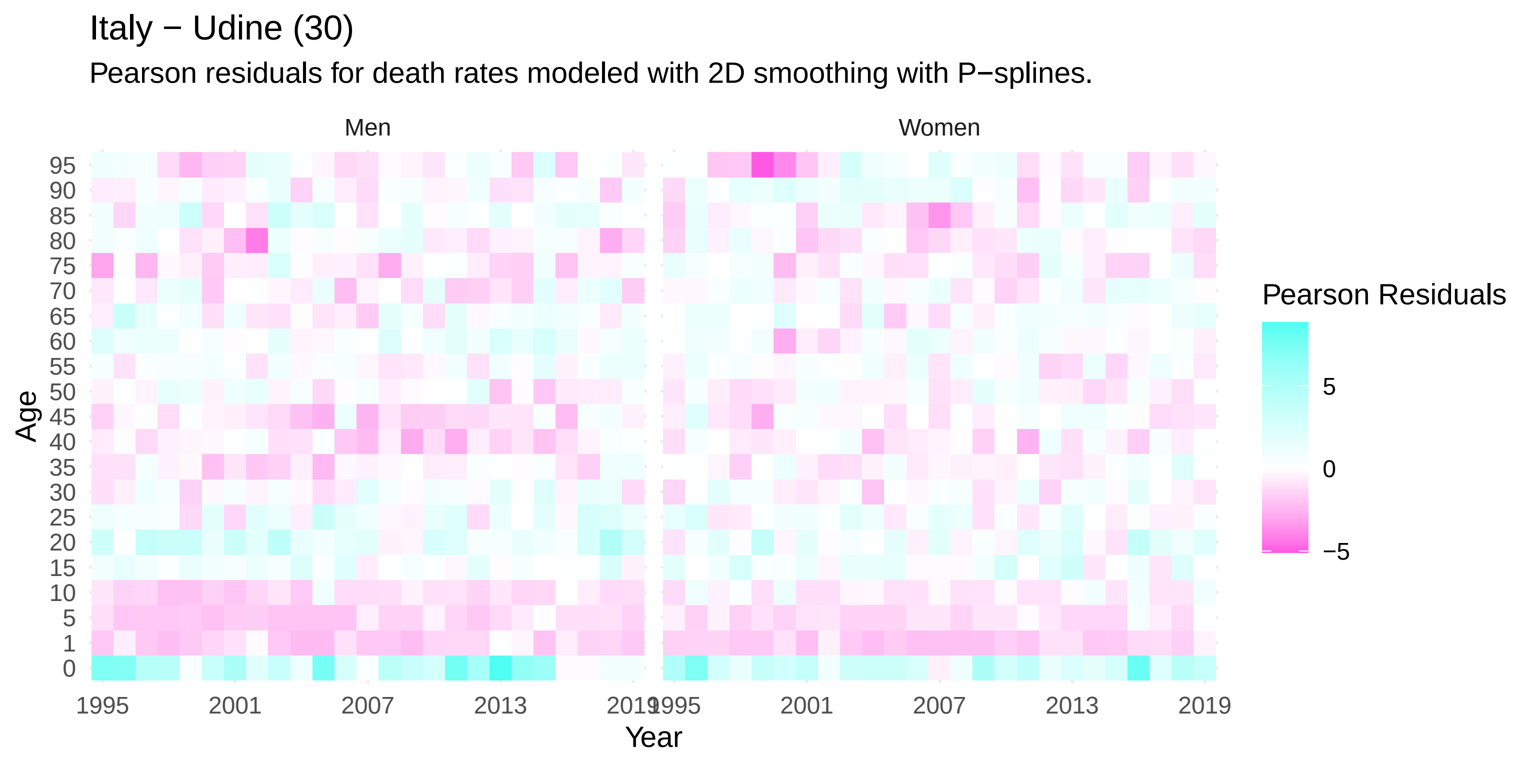

Italy − Udine (30)
Pearson residuals for death rates modeled with 2D smoothing with P−splines.
Men
Women
Age
95
90
85
80
75
70
65
60
55
50
45
40
35
30
25
20
15
10
5
1
0
1995
2001
2007
2013
2019
Year
Pearson Residuals
5
0
−5

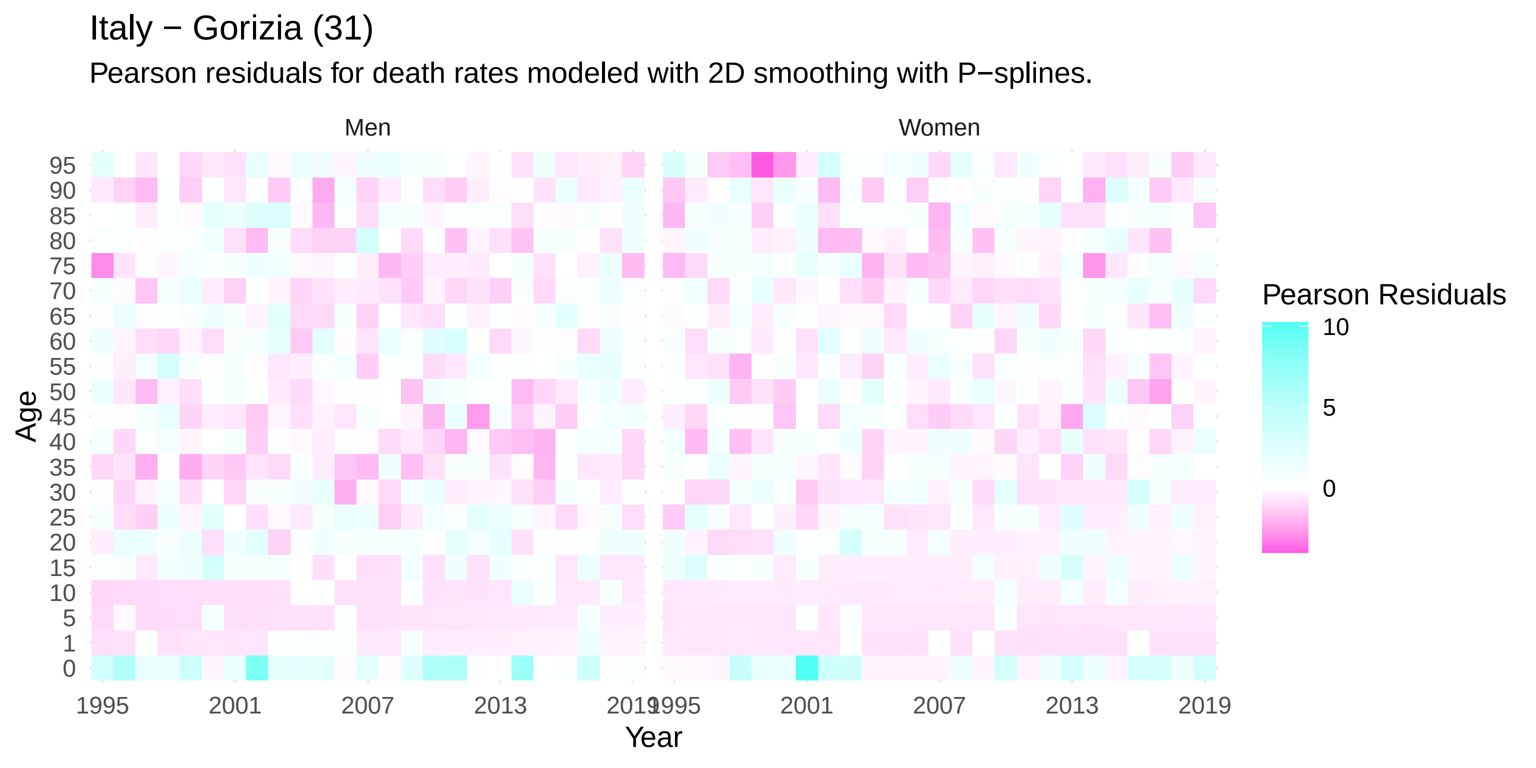

Italy − Gorizia (31)
Pearson residuals for death rates modeled with 2D smoothing with P−splines.
Men
Women
Age
95
90
85
80
75
70
65
60
55
50
45
40
35
30
25
20
15
10
5
1
0
1995
2001
2007
2013
2019
Year
Pearson Residuals
10
5
0

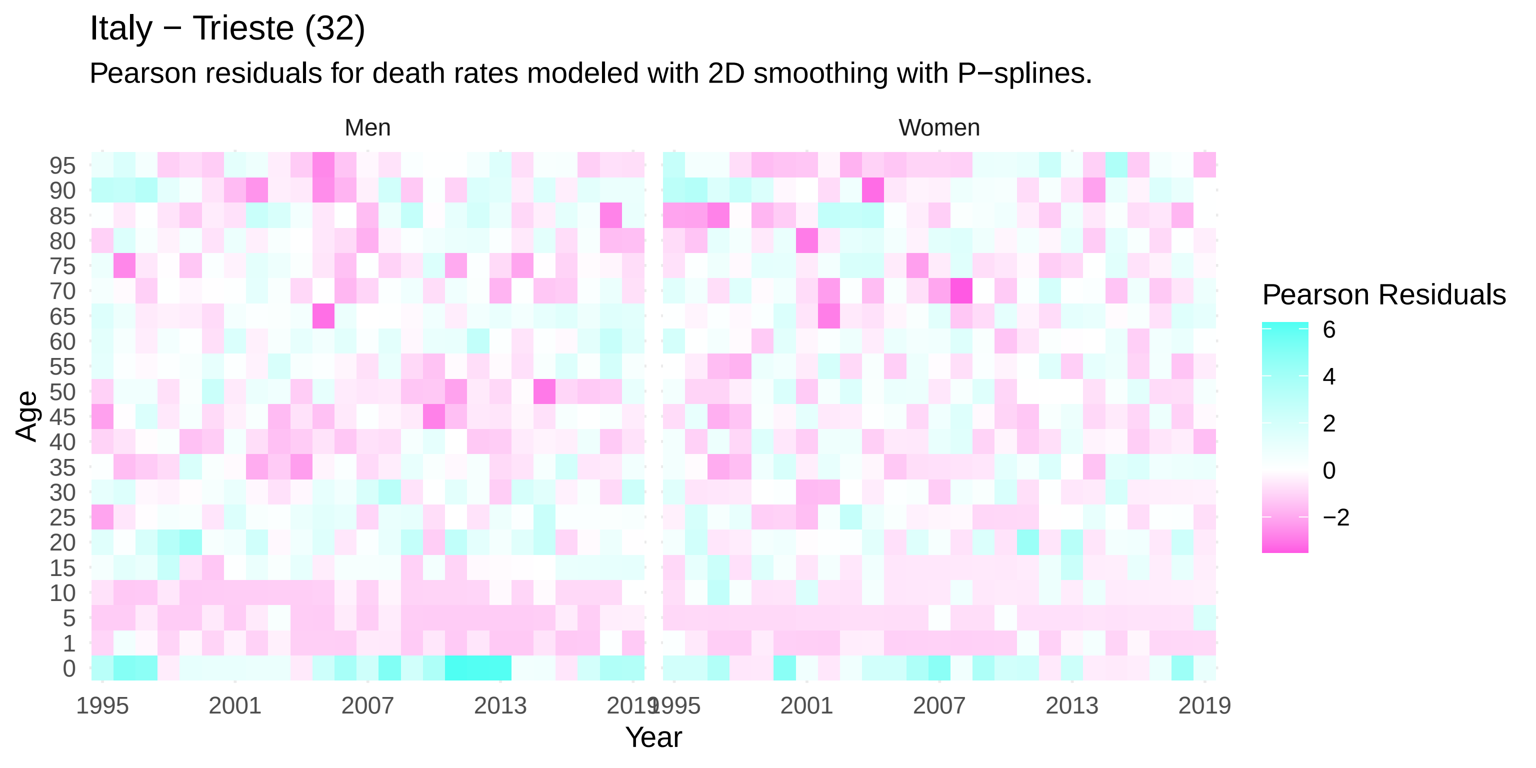

Italy − Trieste (32)
Pearson residuals for death rates modeled with 2D smoothing with P−splines.
Men
Women
Age
95
90
85
80
75
70
65
60
55
50
45
40
35
30
25
20
15
10
5
1
0
1995
2001
2007
2013
2019
Year
Pearson Residuals
6
4
2
0
−2

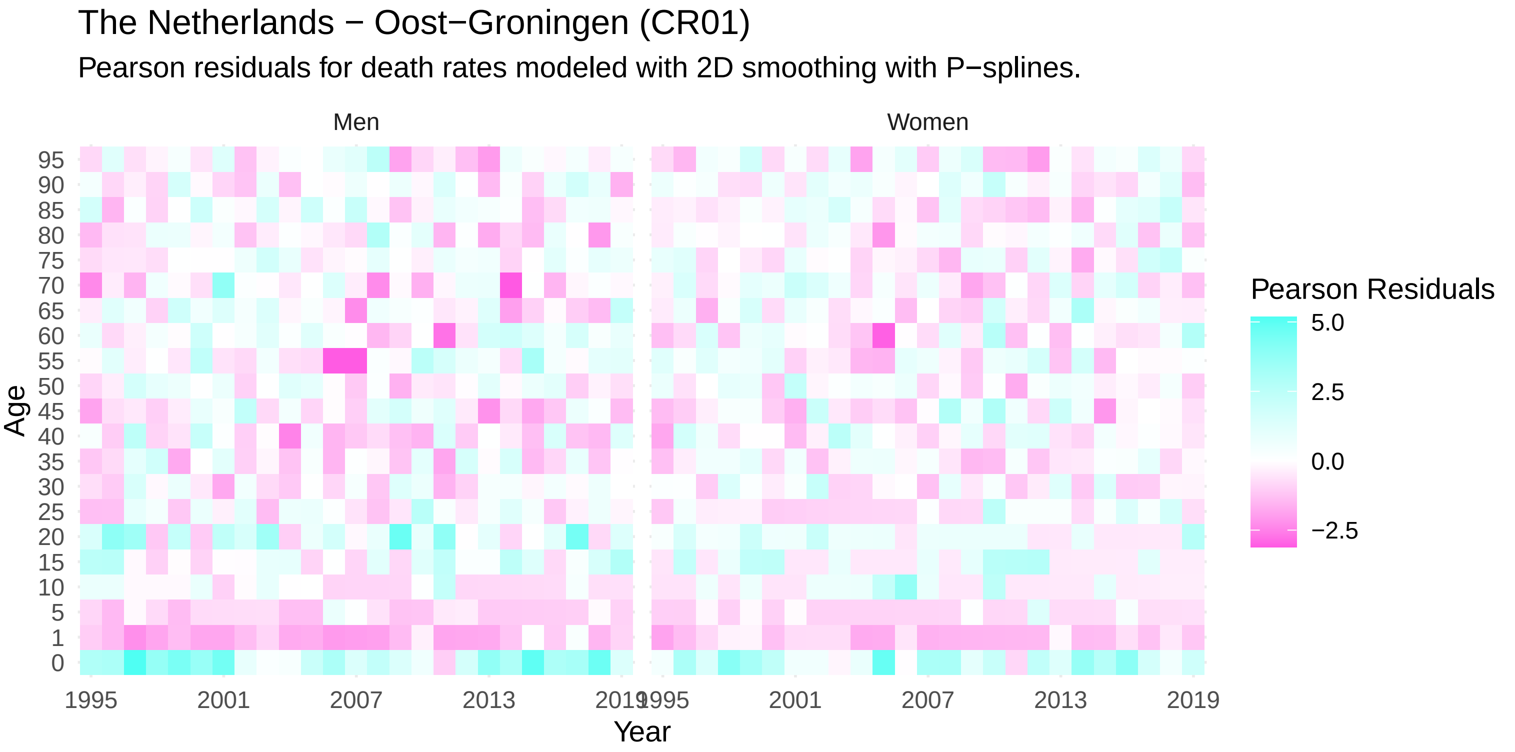
The Netherlands − Oost−Groningen (CR01)
Pearson residuals for death rates modeled with 2D smoothing with P−splines.
Men
Women
Age
95
90
85
80
75
70
65
60
55
50
45
40
35
30
25
20
15
10
5
1
0
1995
2001
2007
2013
2019
Year
Pearson Residuals
5.0
2.5
0.0
−2.5

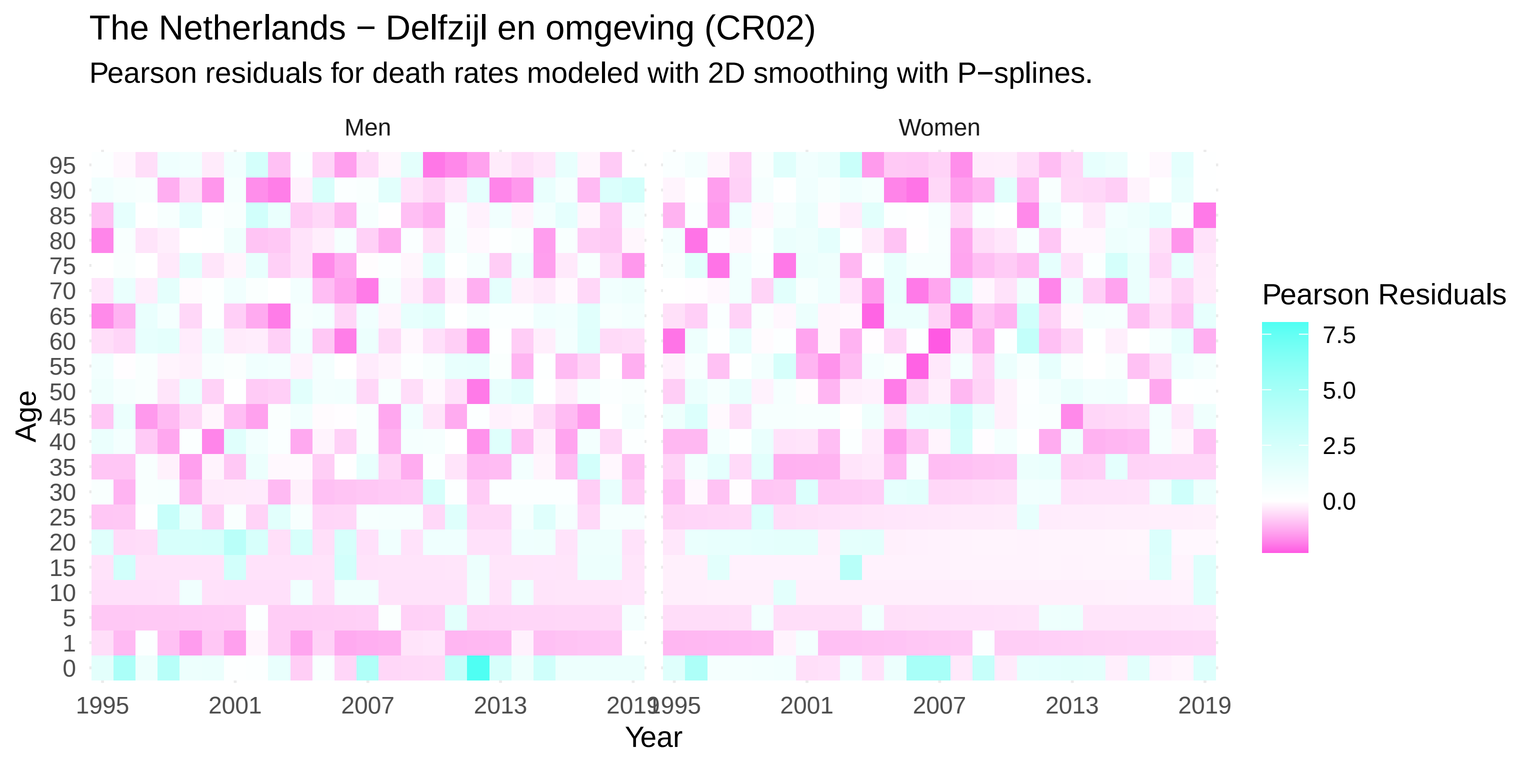
The Netherlands − Delfzijl en omgeving (CR02)
Pearson residuals for death rates modeled with 2D smoothing with P−splines.
Men
Women
Age
95
90
85
80
75
70
65
60
55
50
45
40
35
30
25
20
15
10
5
1
0
1995
2001
2007
2013
2019
Year
Pearson Residuals
7.5
5.0
2.5
0.0

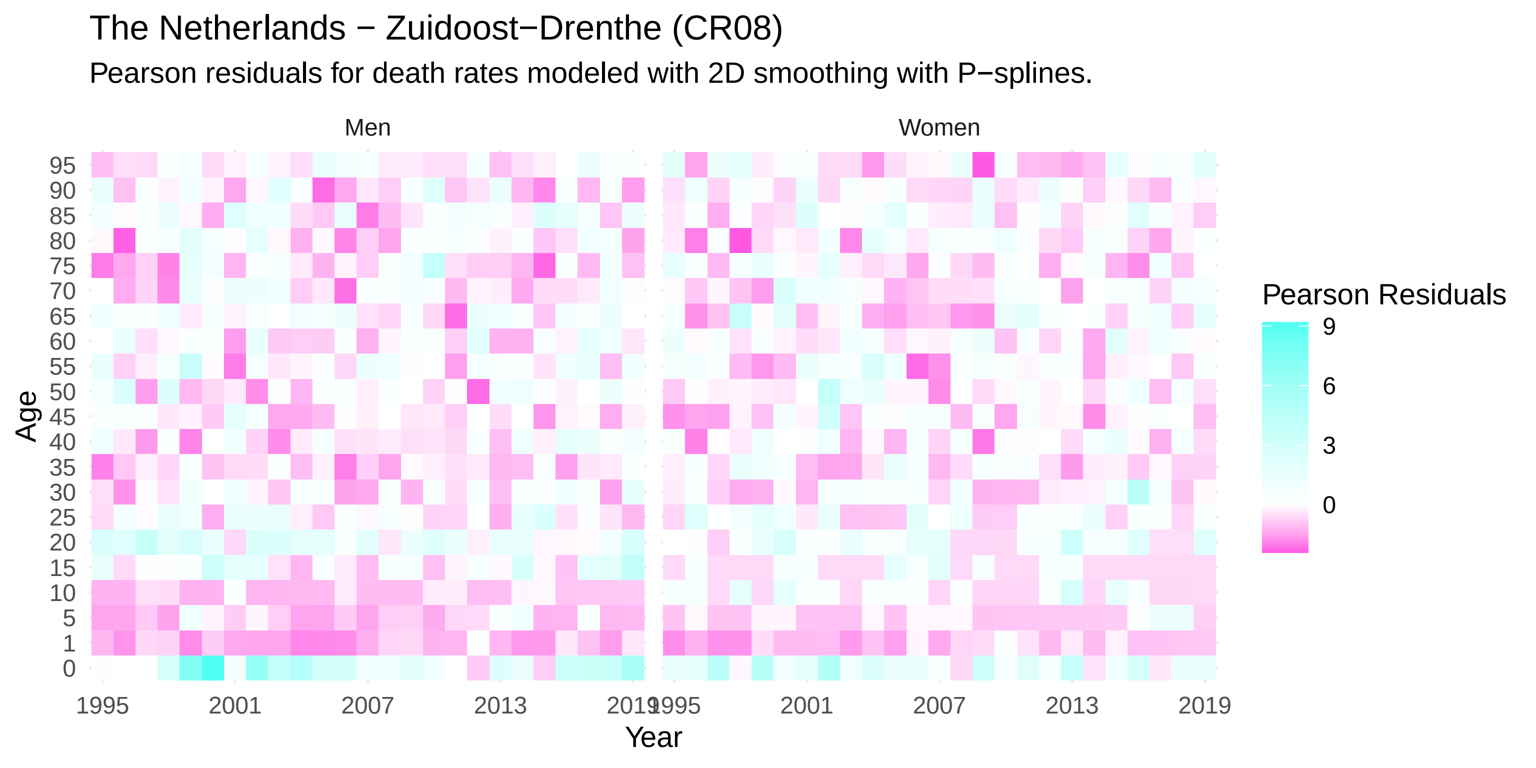

The Netherlands − Zuidoost−Drenthe (CR08)
Pearson residuals for death rates modeled with 2D smoothing with P−splines.
Men
Women
Age
95
90
85
80
75
70
65
60
55
50
45
40
35
30
25
20
15
10
5
1
0
1995
2001
2007
2013
2019
Year
Pearson Residuals
9
6
3
0

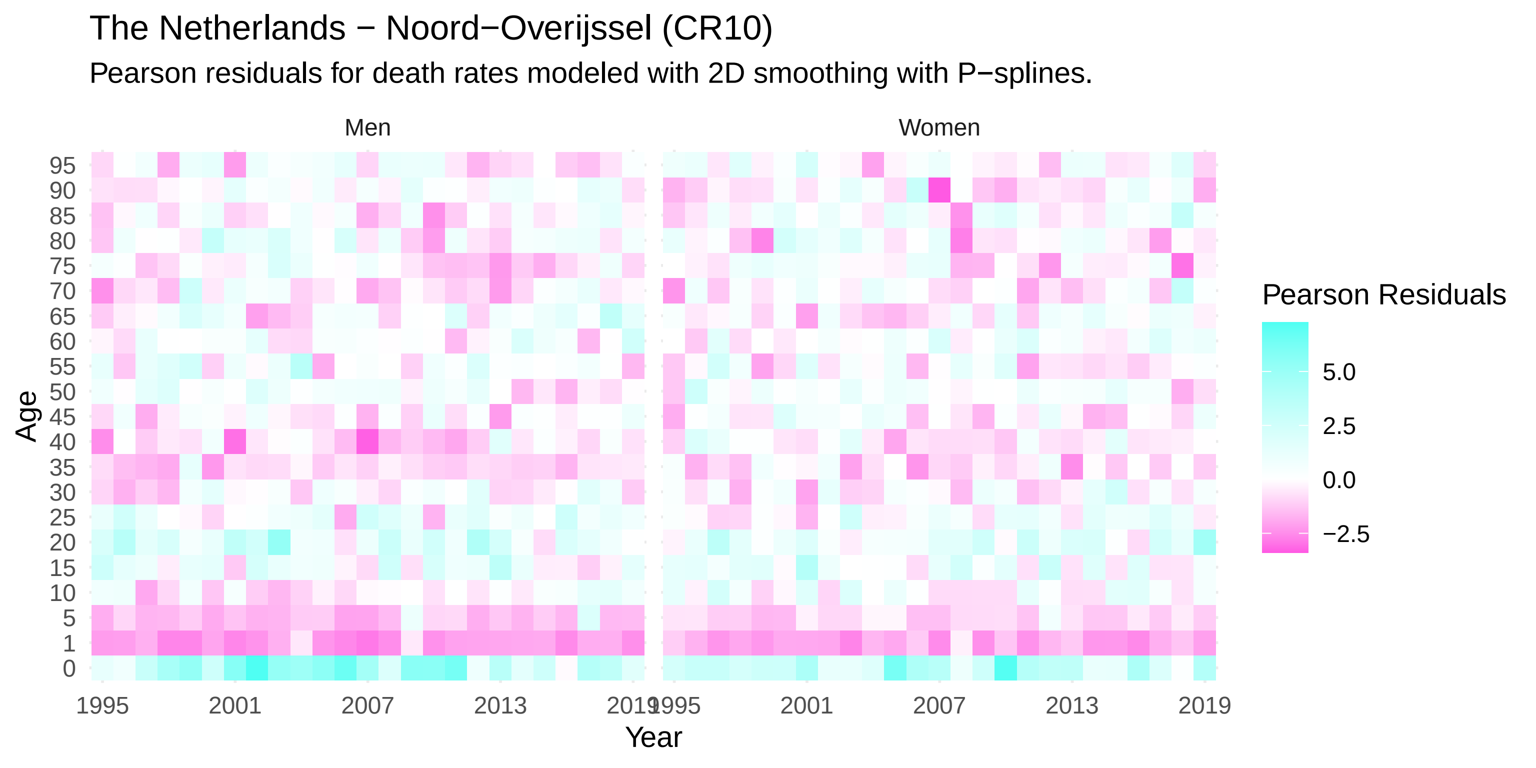

The Netherlands − Noord−Overijssel (CR10)
Pearson residuals for death rates modeled with 2D smoothing with P−splines.
Men
Women
Age
95
90
85
80
75
70
65
60
55
50
45
40
35
30
25
20
15
10
5
1
0
1995
2001
2007
2013
2019
Year
Pearson Residuals
5.0
2.5
0.0
−2.5

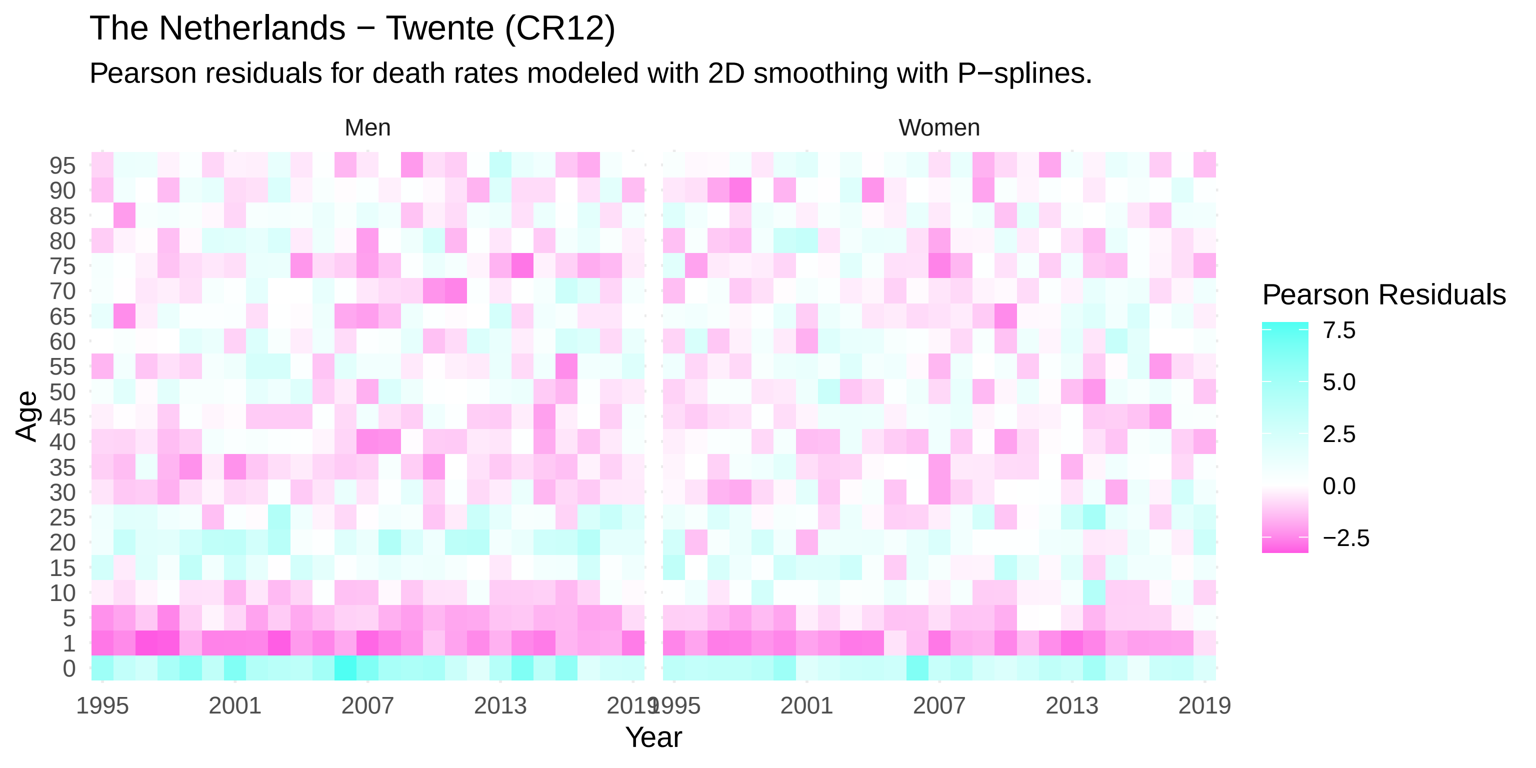

The Netherlands − Twente (CR12)
Pearson residuals for death rates modeled with 2D smoothing with P−splines.
Men
Women
Age
95
90
85
80
75
70
65
60
55
50
45
40
35
30
25
20
15
10
5
1
0
1995
2001
2007
2013
2019
Year
Pearson Residuals
7.5
5.0
2.5
0.0
−2.5

The Netherlands − Achterhoek (CR14)

Pearson residuals for death rates modeled with 2D smoothing with P−splines.

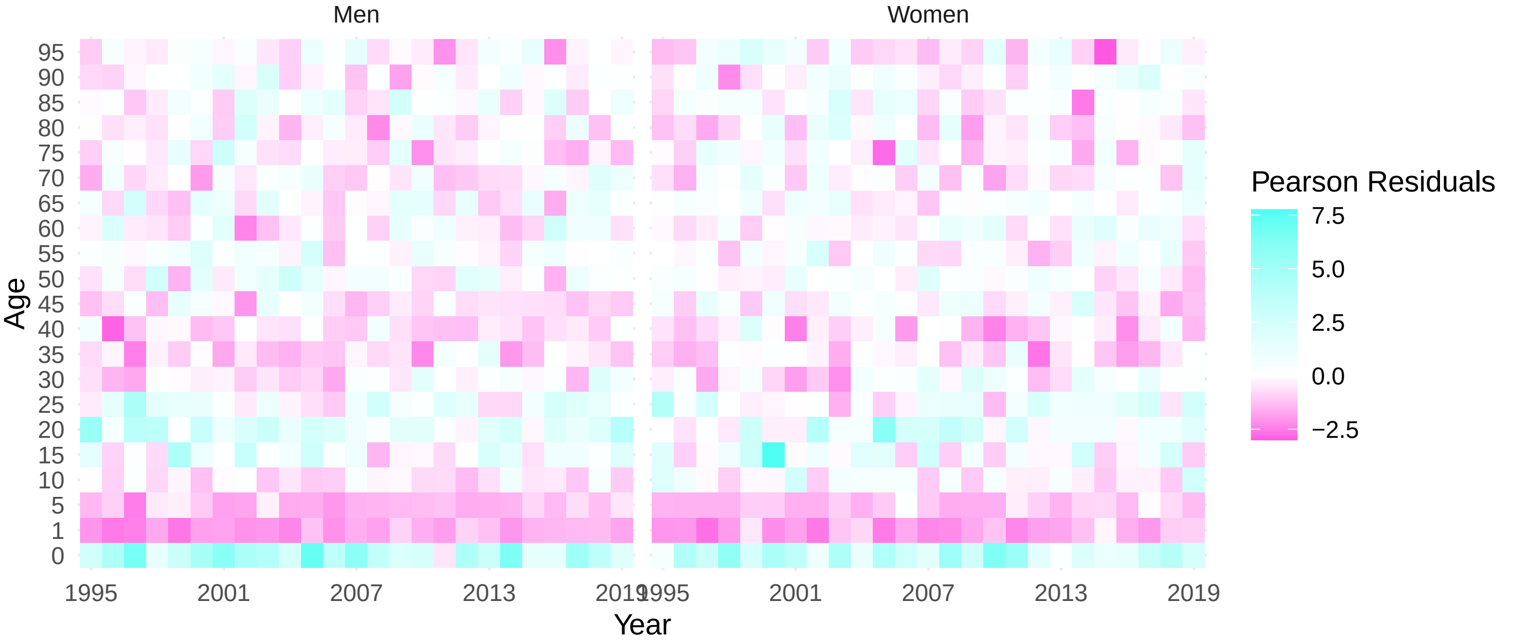

The Netherlands − Arnhem/Nijmegen (CR15)

Pearson residuals for death rates modeled with 2D smoothing with P−splines.

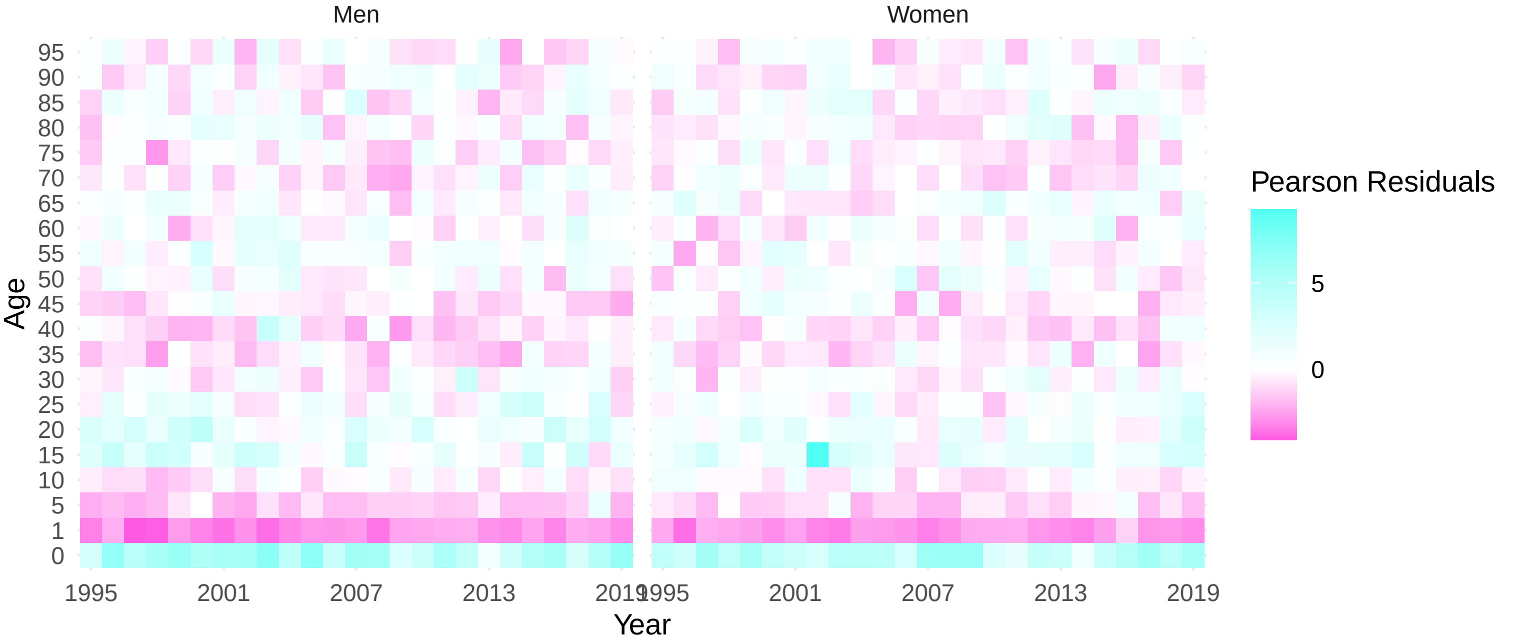

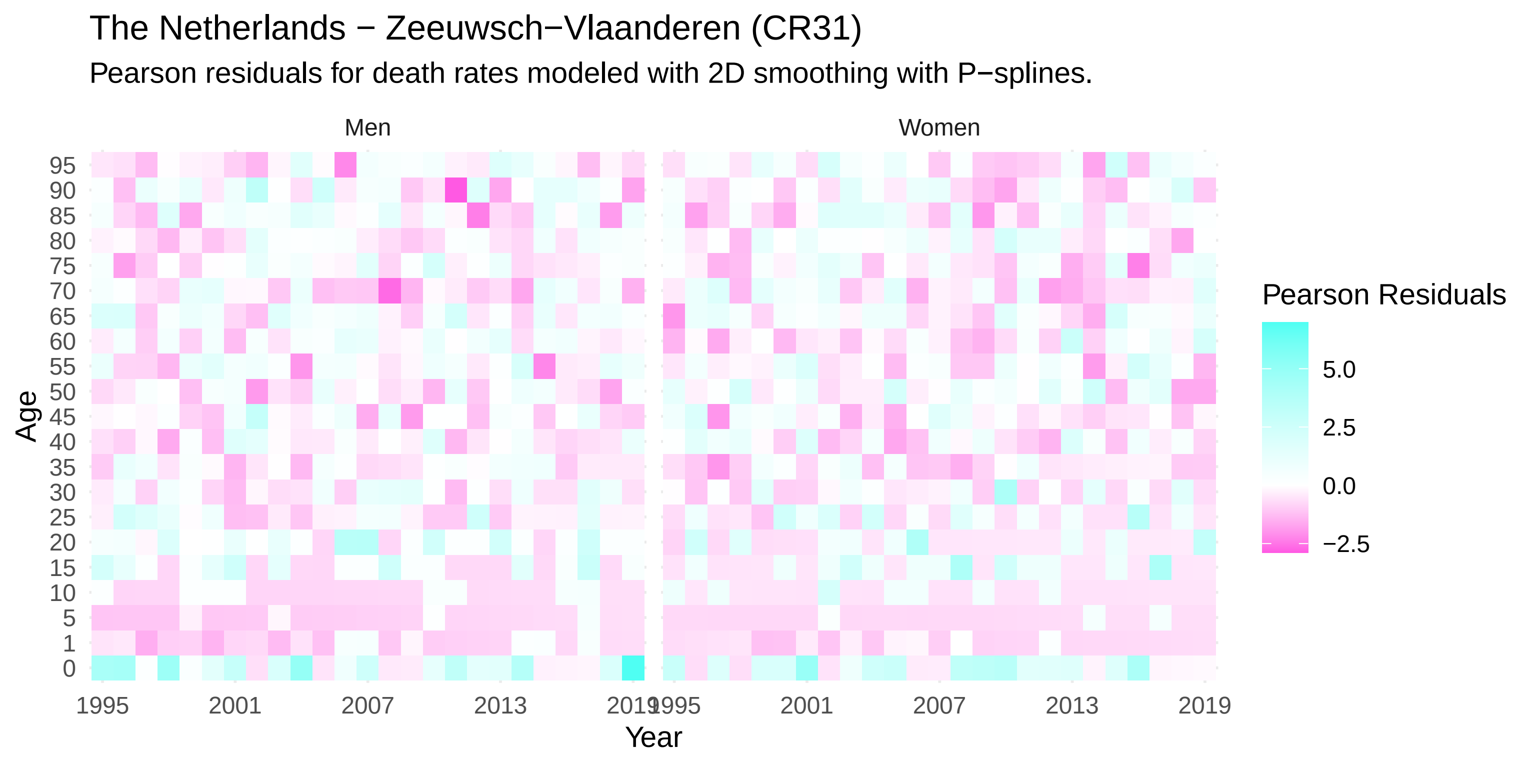

The Netherlands − Zeeuwsch−Vlaanderen (CR31)
Pearson residuals for death rates modeled with 2D smoothing with P−splines.
Men
Women
Age
95
90
85
80
75
70
65
60
55
50
45
40
35
30
25
20
15
10
5
1
0
1995
2001
2007
2013
2019
Year
Pearson Residuals
5.0
2.5
0.0
−2.5

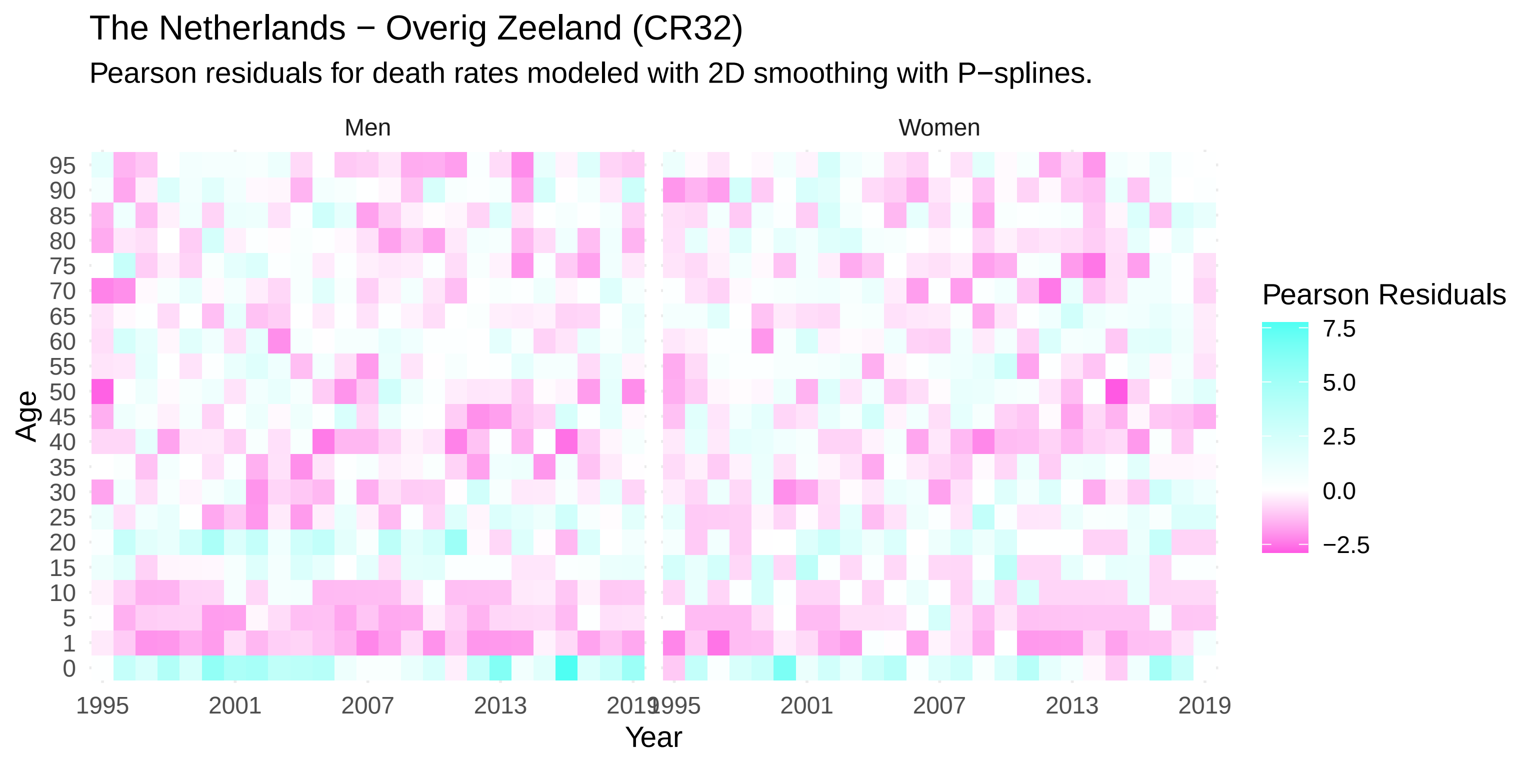

The Netherlands − Overig Zeeland (CR32)
Pearson residuals for death rates modeled with 2D smoothing with P−splines.
Men
Women
Age
95
90
85
80
75
70
65
60
55
50
45
40
35
30
25
20
15
10
5
1
0
1995
2001
2007
2013
2019
Year
Pearson Residuals
7.5
5.0
2.5
0.0
−2.5

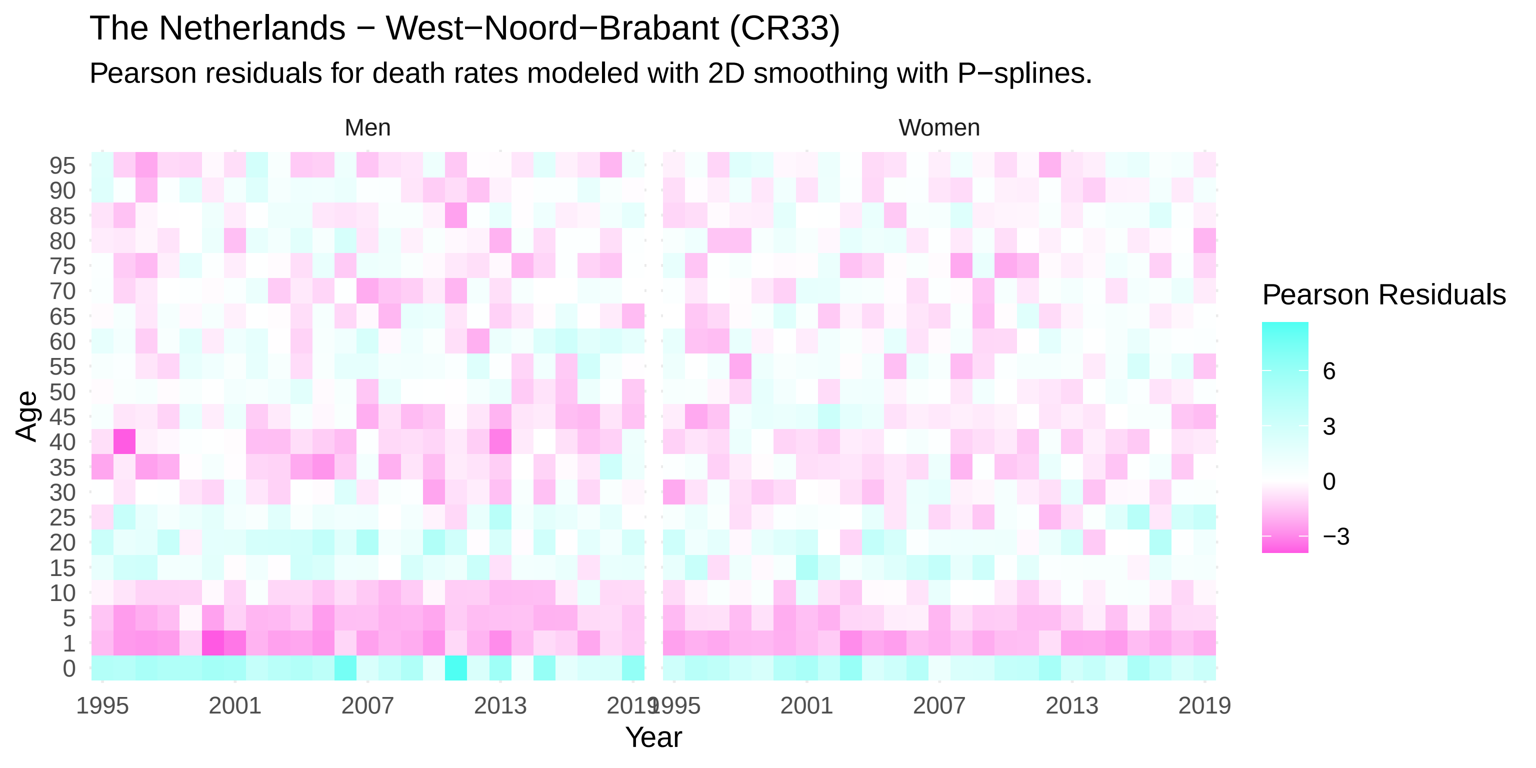

The Netherlands − West−Noord−Brabant (CR33)
Pearson residuals for death rates modeled with 2D smoothing with P−splines.
Men
Women
Age
95
90
85
80
75
70
65
60
55
50
45
40
35
30
25
20
15
10
5
1
0
1995
2001
2007
2013
2019
Year
Pearson Residuals
6
3
0
−3

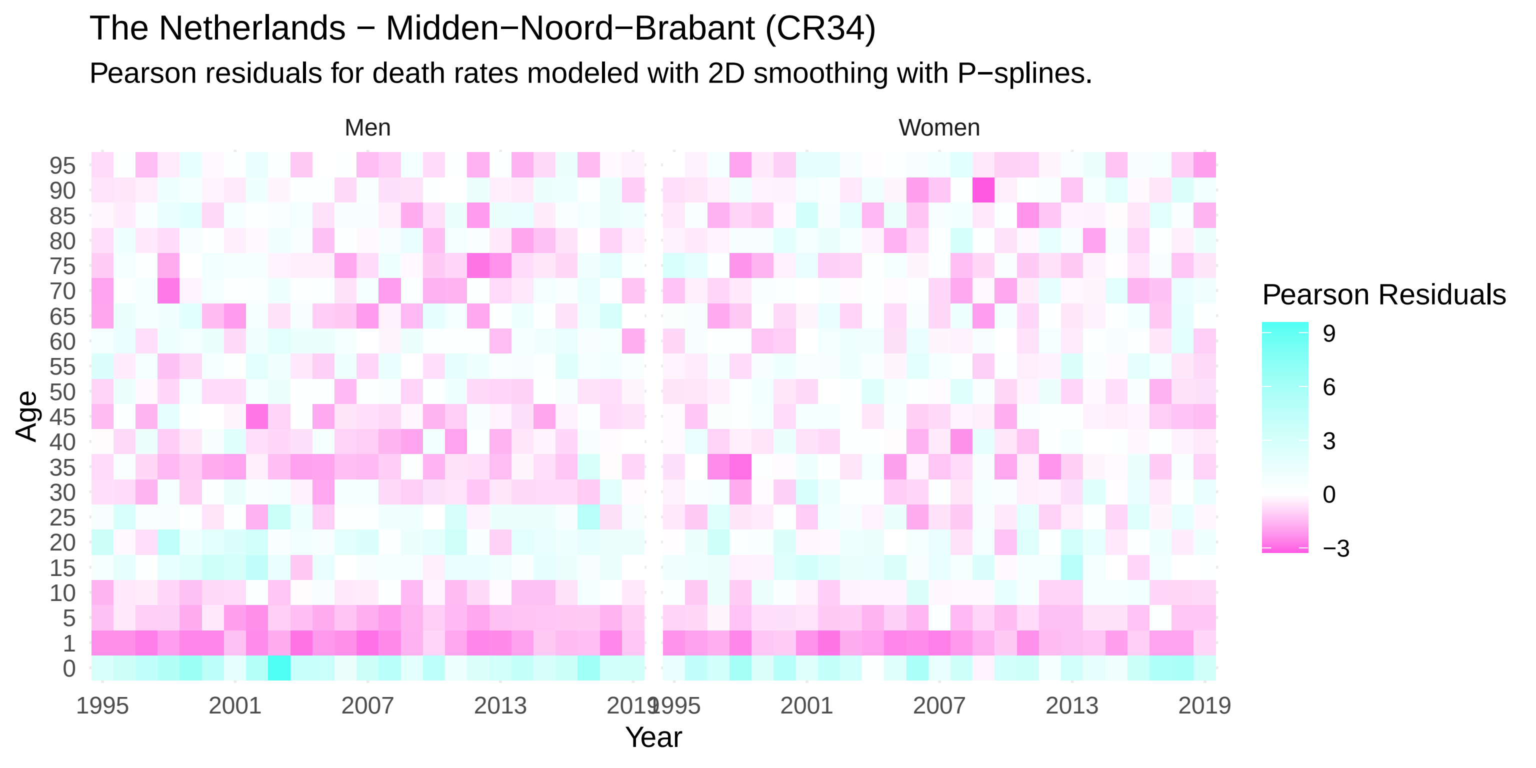

The Netherlands − Midden−Noord−Brabant (CR34)
Pearson residuals for death rates modeled with 2D smoothing with P−splines.
Men
Women
Age
95
90
85
80
75
70
65
60
55
50
45
40
35
30
25
20
15
10
5
1
0
1995
2001
2007
2013
2019
Year
Pearson Residuals
9
6
3
0
−3

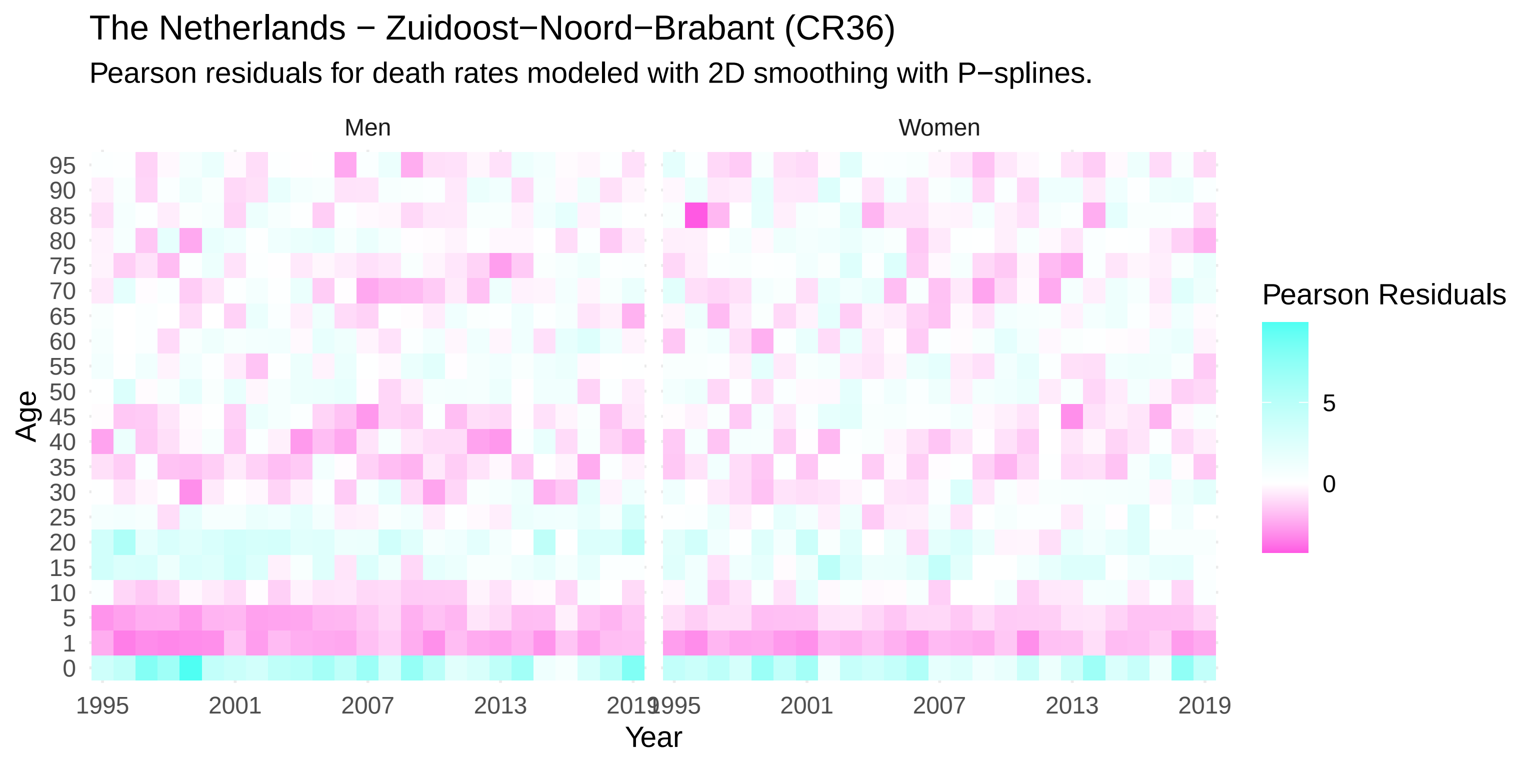
The Netherlands − Zuidoost−Noord−Brabant (CR36)
Pearson residuals for death rates modeled with 2D smoothing with P−splines.
Men
Women
Age
95
90
85
80
75
70
65
60
55
50
45
40
35
30
25
20
15
10
5
1
0
1995
2001
2007
2013
2019
Year
Pearson Residuals
5
0

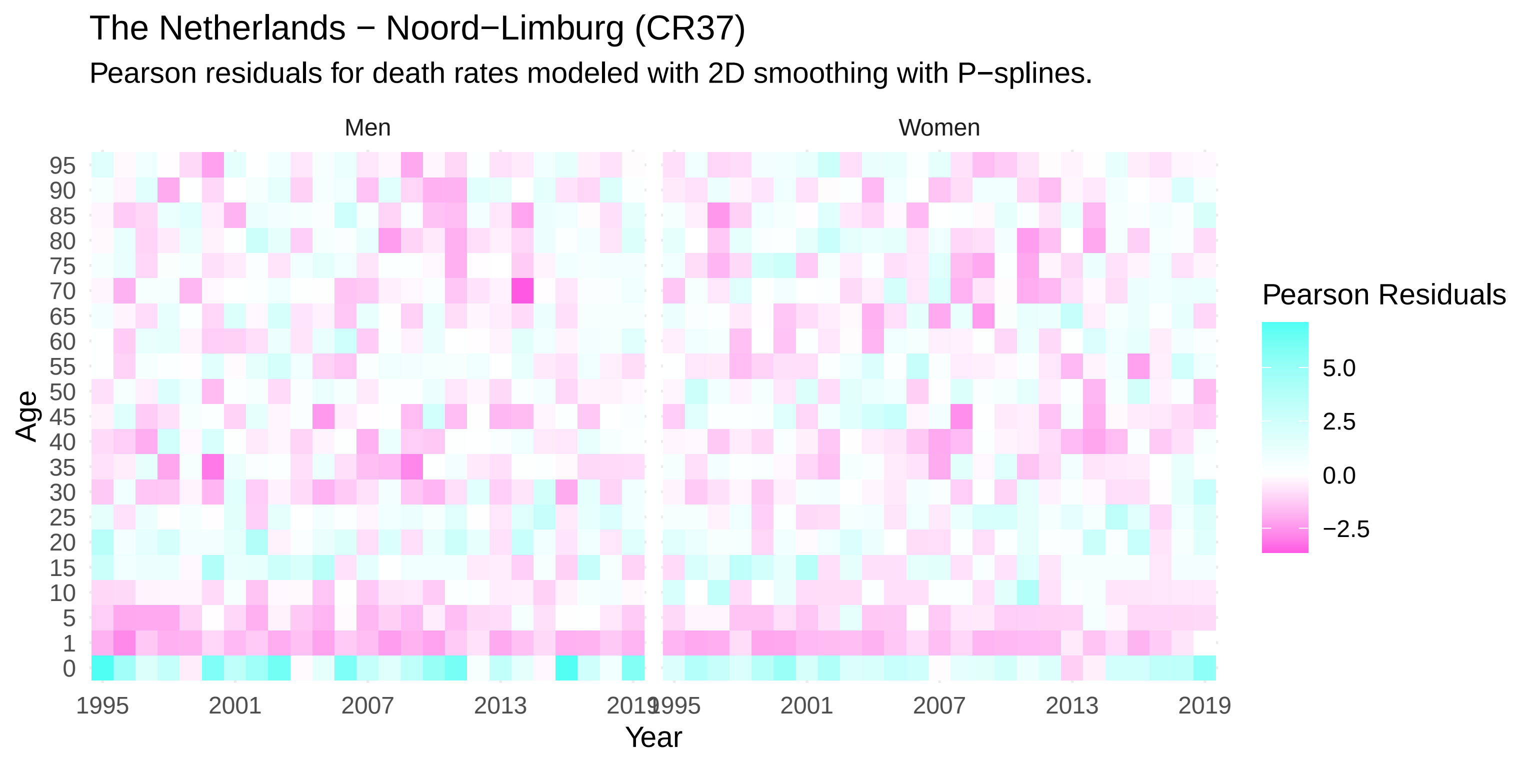

The Netherlands − Noord−Limburg (CR37)
Pearson residuals for death rates modeled with 2D smoothing with P−splines.
Men
Women
Age
95
90
85
80
75
70
65
60
55
50
45
40
35
30
25
20
15
10
5
1
0
1995
2001
2007
2013
2019
Year
Pearson Residuals
5.0
2.5
0.0
−2.5

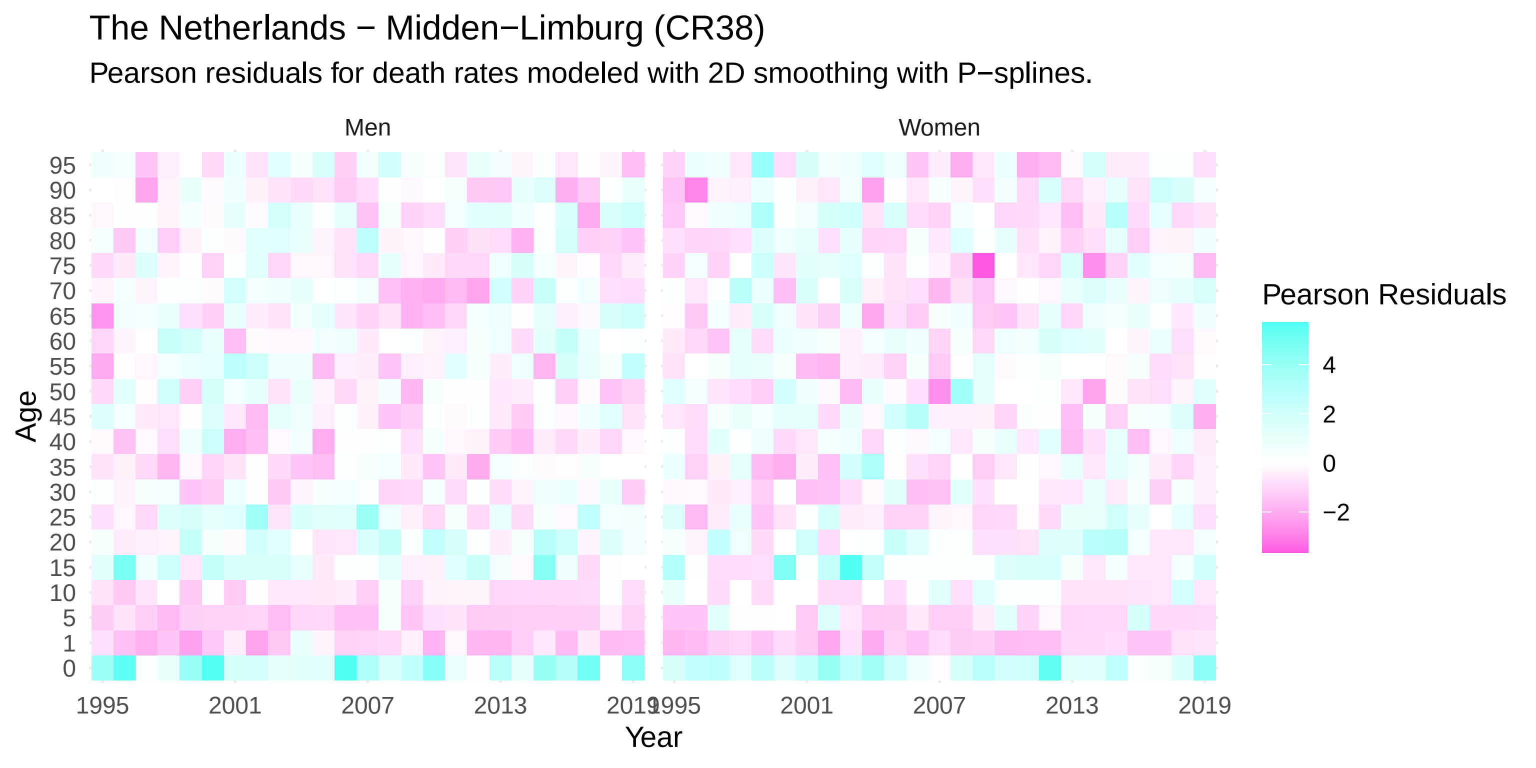

The Netherlands − Midden−Limburg (CR38)
Pearson residuals for death rates modeled with 2D smoothing with P−splines.
Men
Women
Age
95
90
85
80
75
70
65
60
55
50
45
40
35
30
25
20
15
10
5
1
0
1995
2001
2007
2013
2019
Year
Pearson Residuals
4
2
0
−2

The Netherlands − Zuid−Limburg (CR39)

Pearson residuals for death rates modeled with 2D smoothing with P−splines.

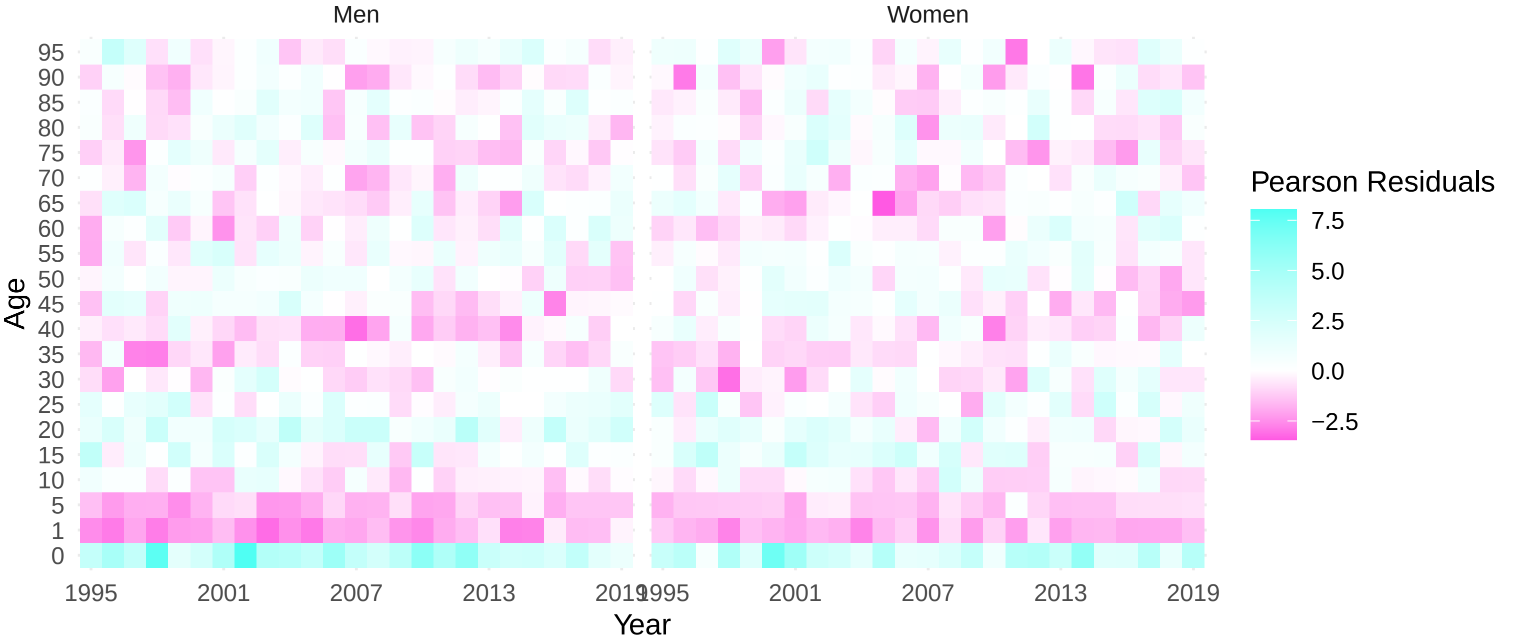

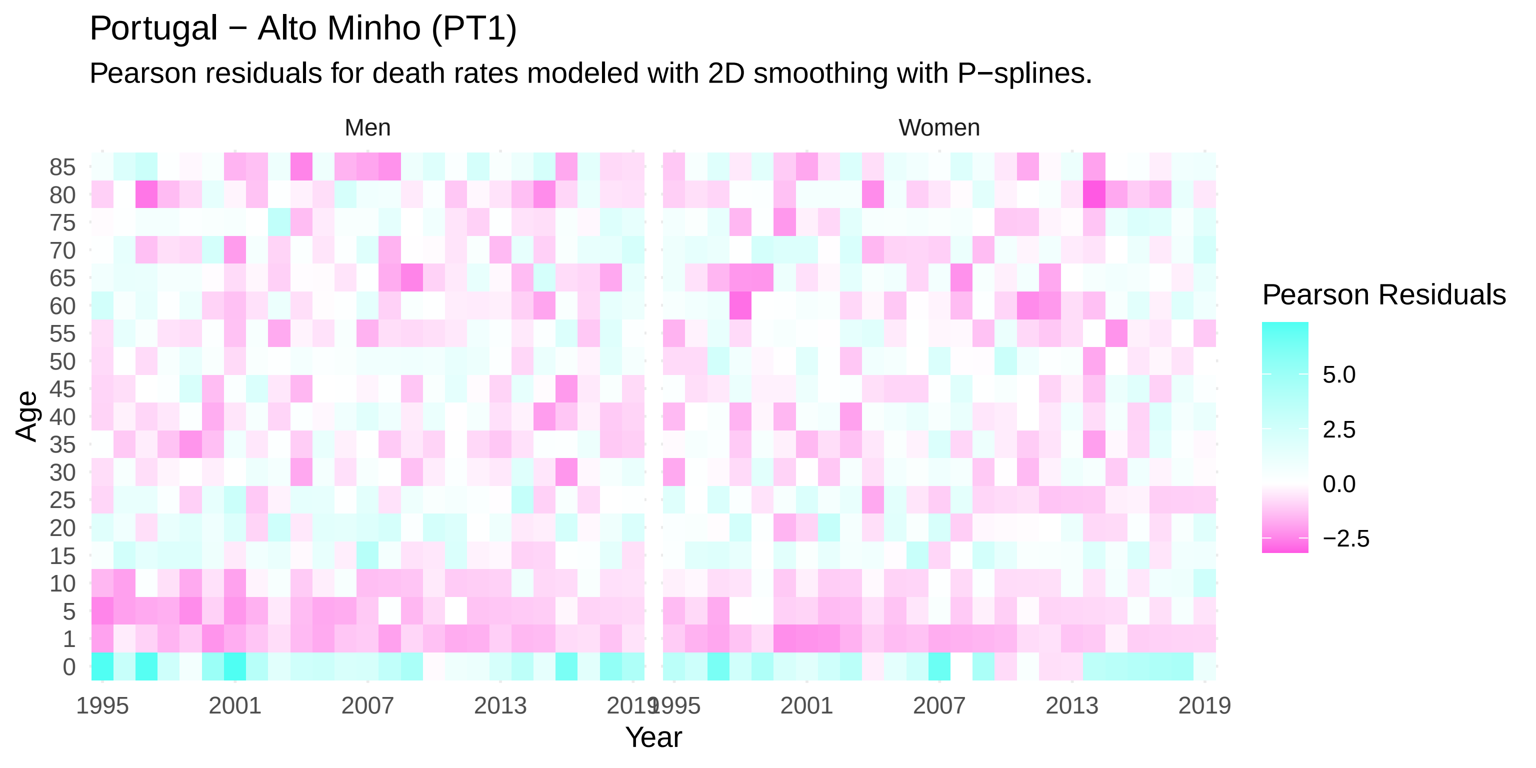

Portugal − Alto Minho (PT1)
Pearson residuals for death rates modeled with 2D smoothing with P−splines.
Men
Women
Age
85
80
75
70
65
60
55
50
45
40
35
30
25
20
15
10
5
1
0
1995
2001
2007
2013
2019
Year
Pearson Residuals
5.0
2.5
0.0
−2.5

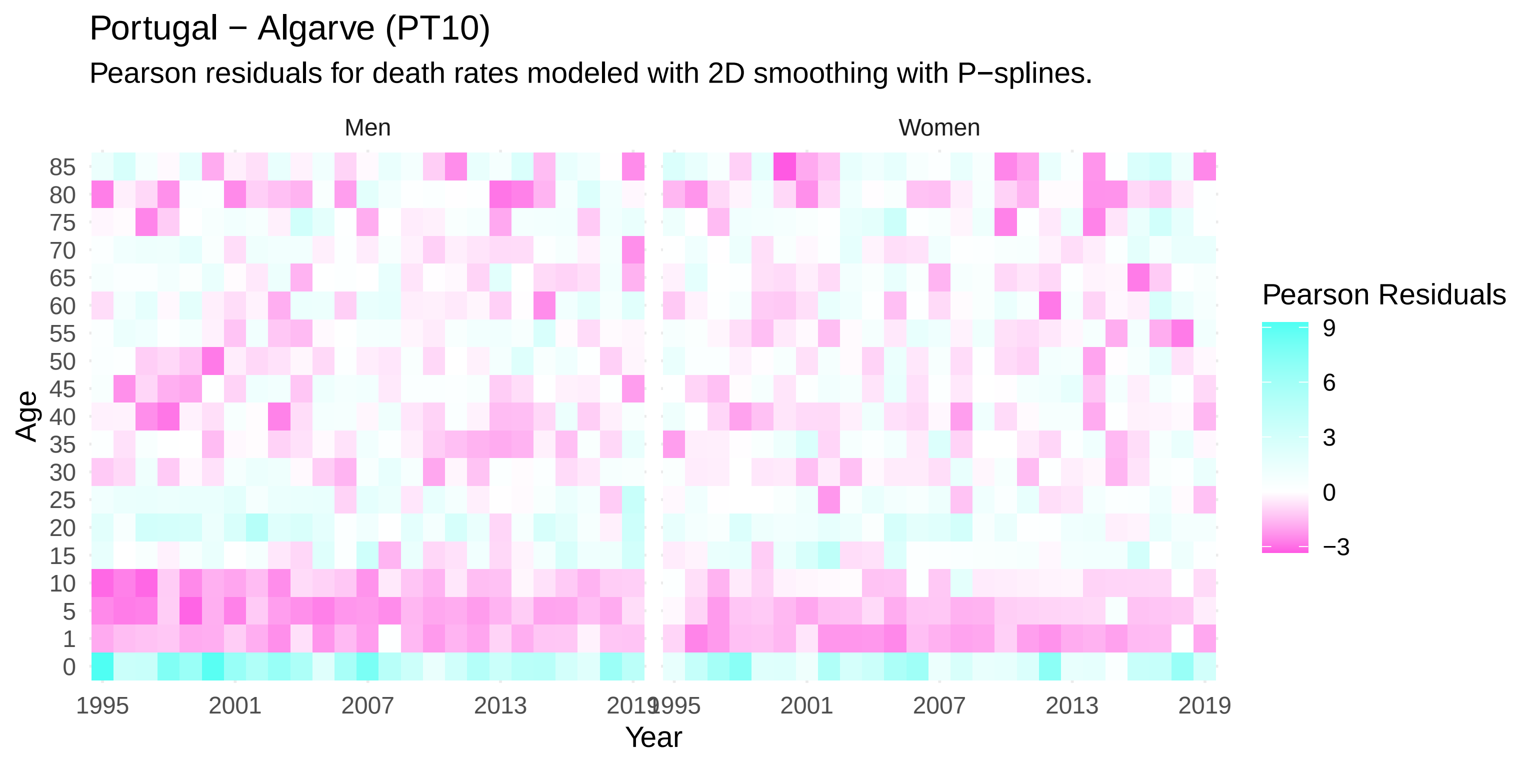

Portugal − Algarve (PT10)
Pearson residuals for death rates modeled with 2D smoothing with P−splines.
Men
Women
Age
Year
85
80
75
70
65
60
55
50
45
40
35
30
25
20
15
10
5
1
0
1995
2001
2007
2013
2019
Pearson Residuals
9
6
3
0
−3

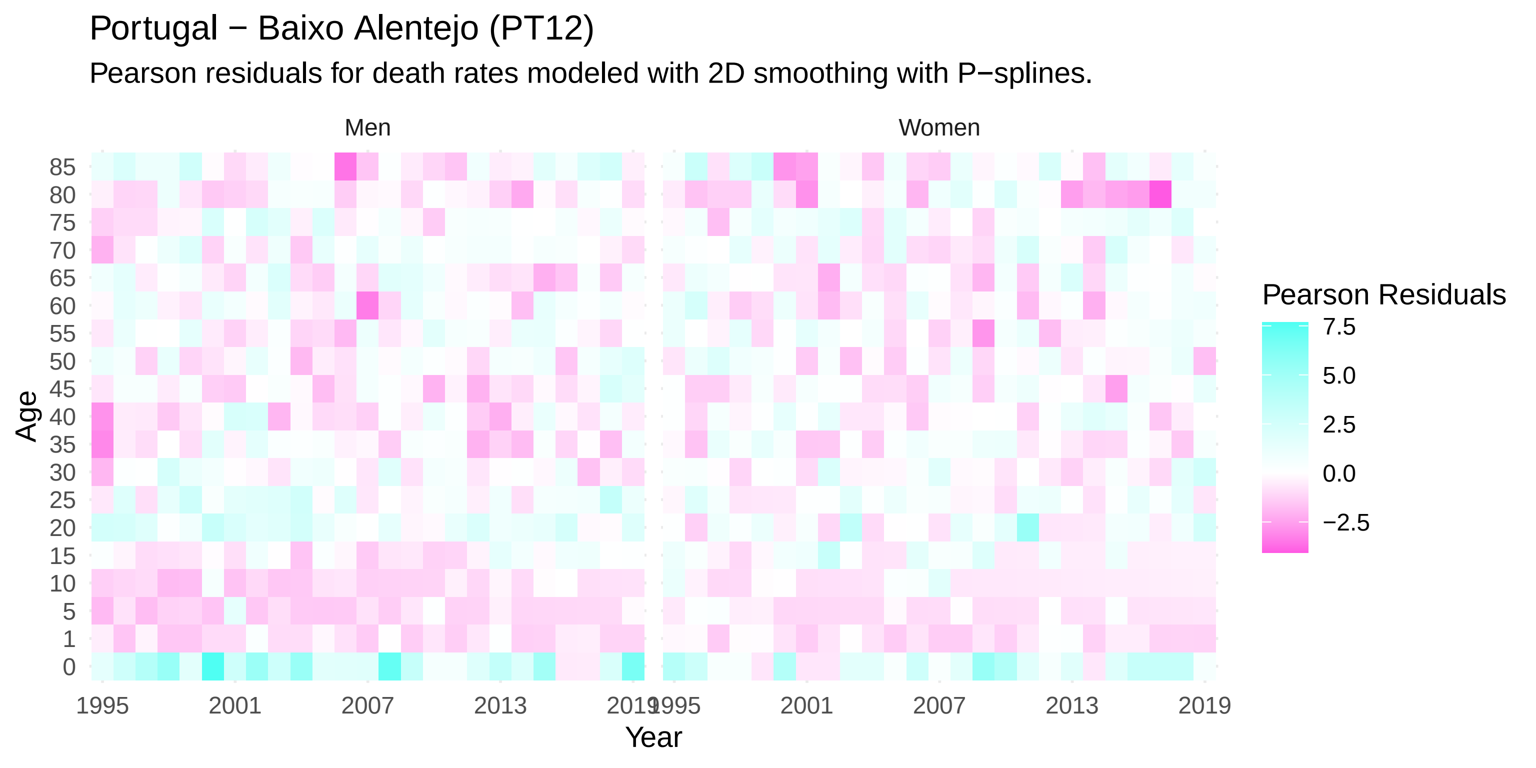

Portugal − Baixo Alentejo (PT12)
Pearson residuals for death rates modeled with 2D smoothing with P−splines.
Men
Women
Age
85
80
75
70
65
60
55
50
45
40
35
30
25
20
15
10
5
1
0
1995
2001
2007
2013
2019
Year
Pearson Residuals
7.5
5.0
2.5
0.0
−2.5

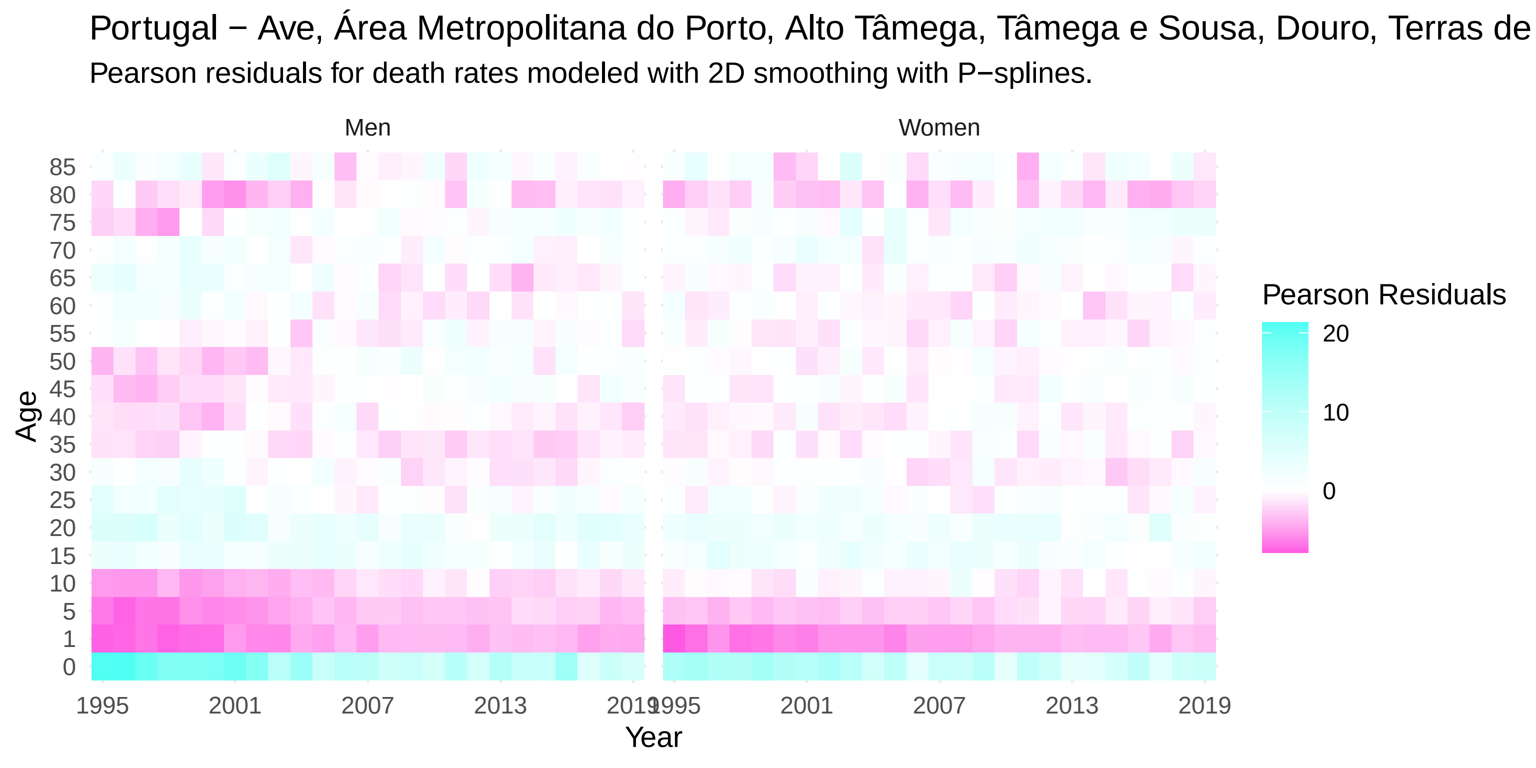

Portugal − Ave, Área Metropolitana do Porto, Alto Tâmega, Tâmega e Sousa, Douro, Terras de
Pearson residuals for death rates modeled with 2D smoothing with P−splines.
Men
Women
Age
85
80
75
70
65
60
55
50
45
40
35
30
25
20
15
10
5
1
0
1995
2001
2007
2013
2019
Year
Pearson Residuals
20
10
0

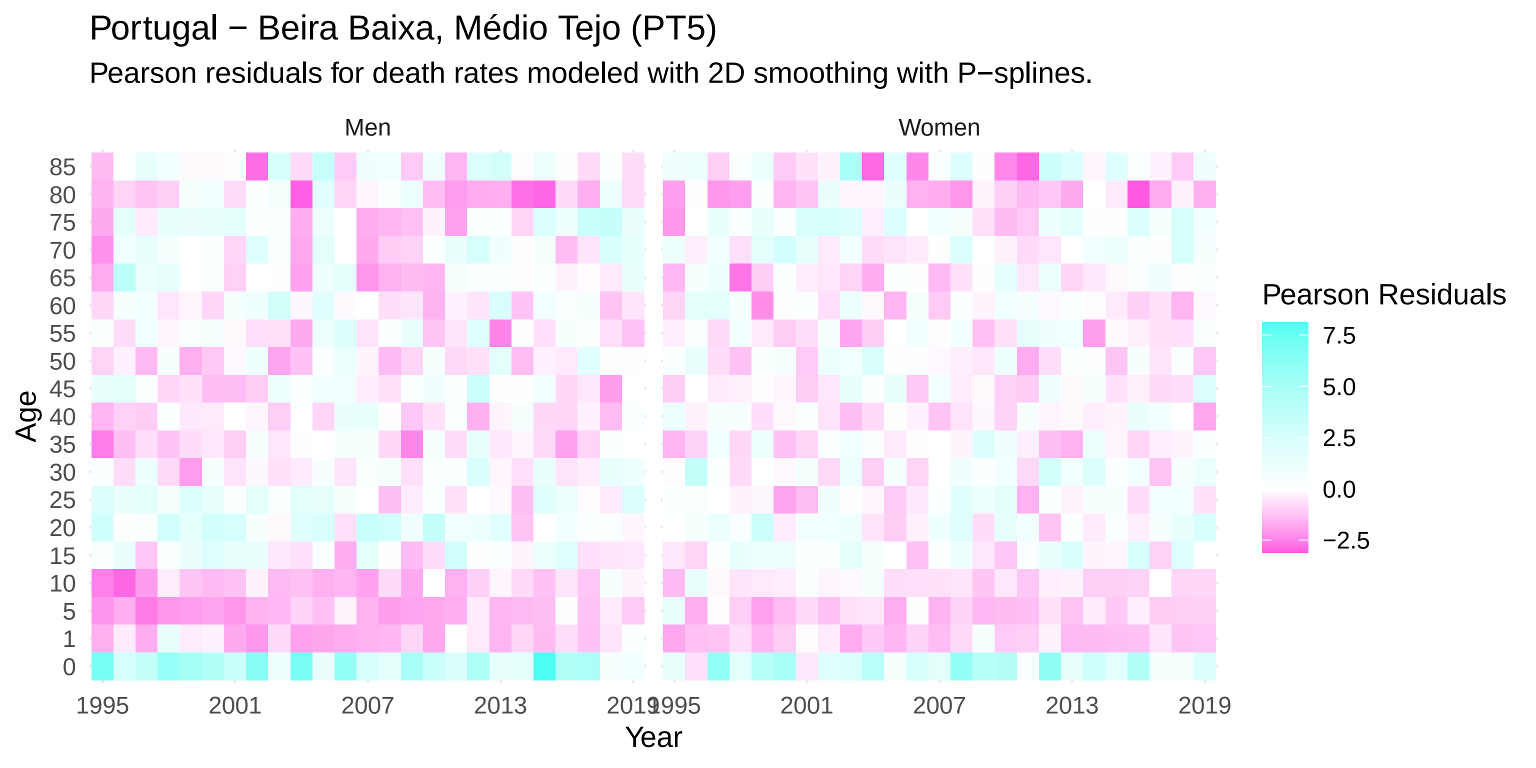

Portugal − Beira Baixa, Médio Tejo (PT5)
Pearson residuals for death rates modeled with 2D smoothing with P−splines.
Men
Women
Age
85
80
75
70
65
60
55
50
45
40
35
30
25
20
15
10
5
1
0
1995
2001
2007
2013
2019
Year
Pearson Residuals
7.5
5.0
2.5
0.0
−2.5

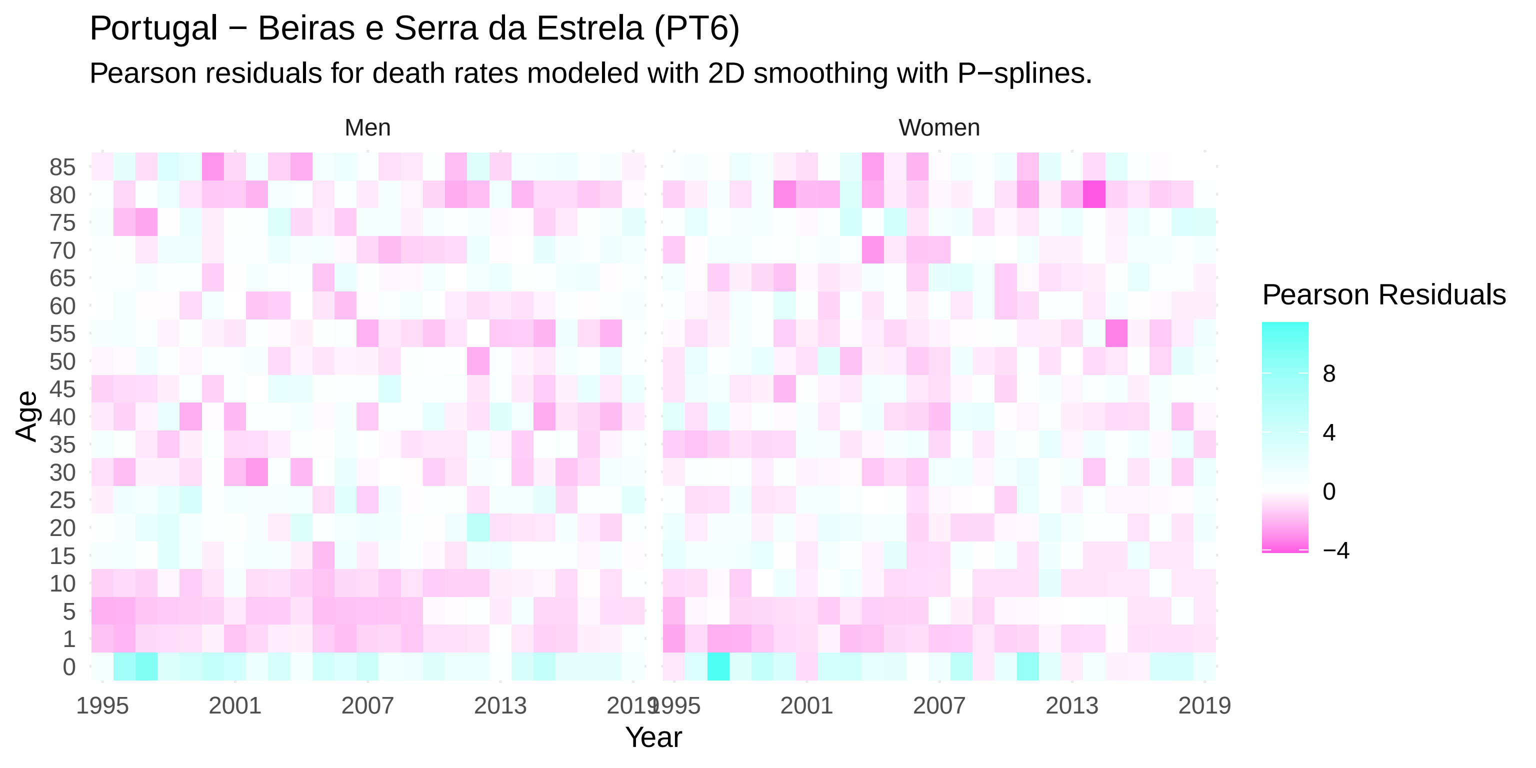
Portugal − Beiras e Serra da Estrela (PT6)
Pearson residuals for death rates modeled with 2D smoothing with P−splines.
Men
Women
Age
85
80
75
70
65
60
55
50
45
40
35
30
25
20
15
10
5
1
0
1995
2001
2007
2013
2019
Year
Pearson Residuals
8
4
0
−4

Portugal − Alto Alentejo, Alentejo Central (PT8)

Pearson residuals for death rates modeled with 2D smoothing with P−splines.

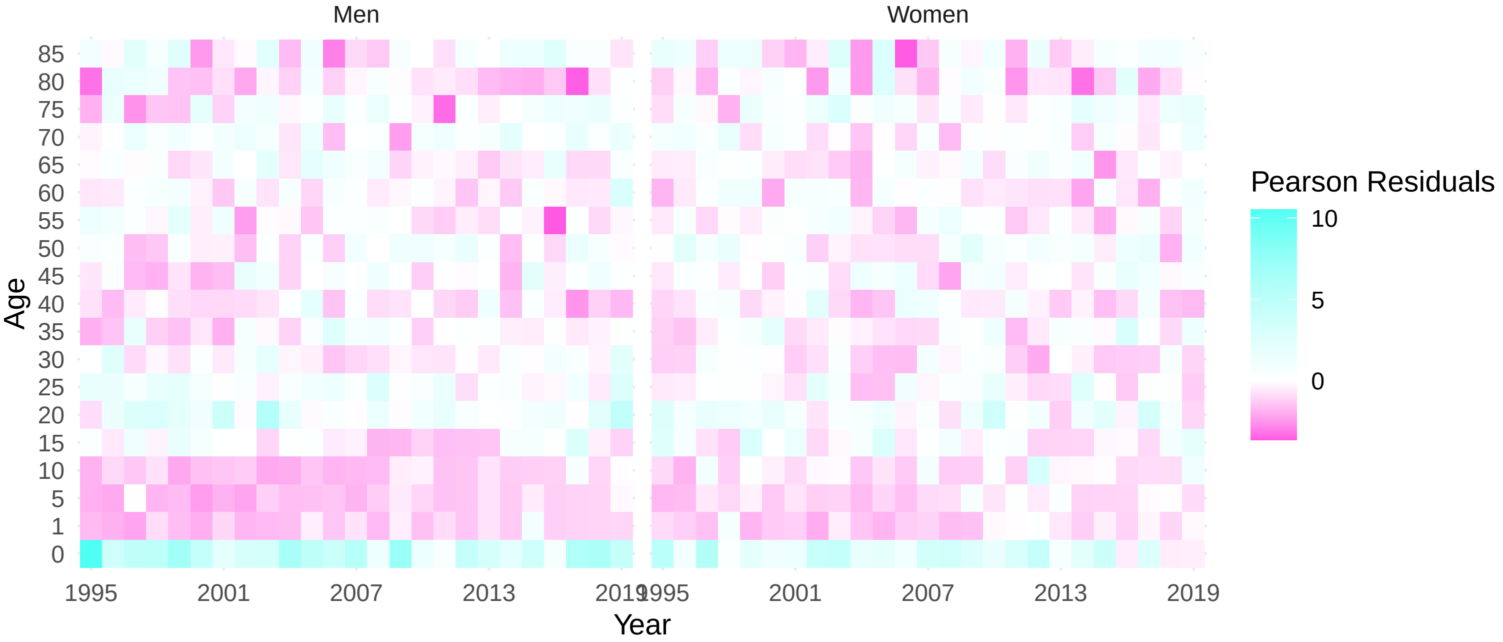

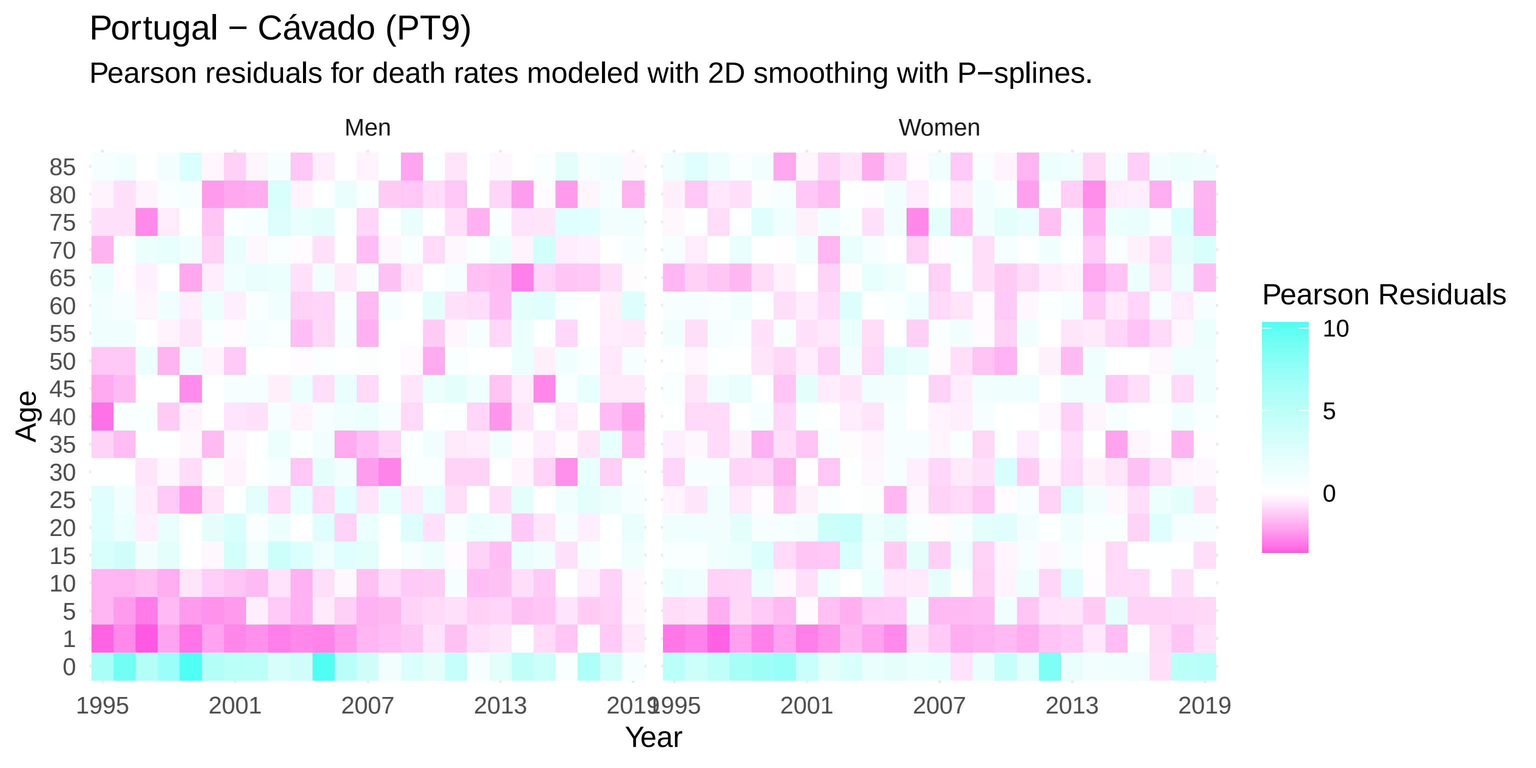

Portugal − Cávado (PT9)
Pearson residuals for death rates modeled with 2D smoothing with P−splines.
Men
Women
Age
85
80
75
70
65
60
55
50
45
40
35
30
25
20
15
10
5
1
0
1995
2001
2007
2013
2019
Year
Pearson Residuals
10
5
0

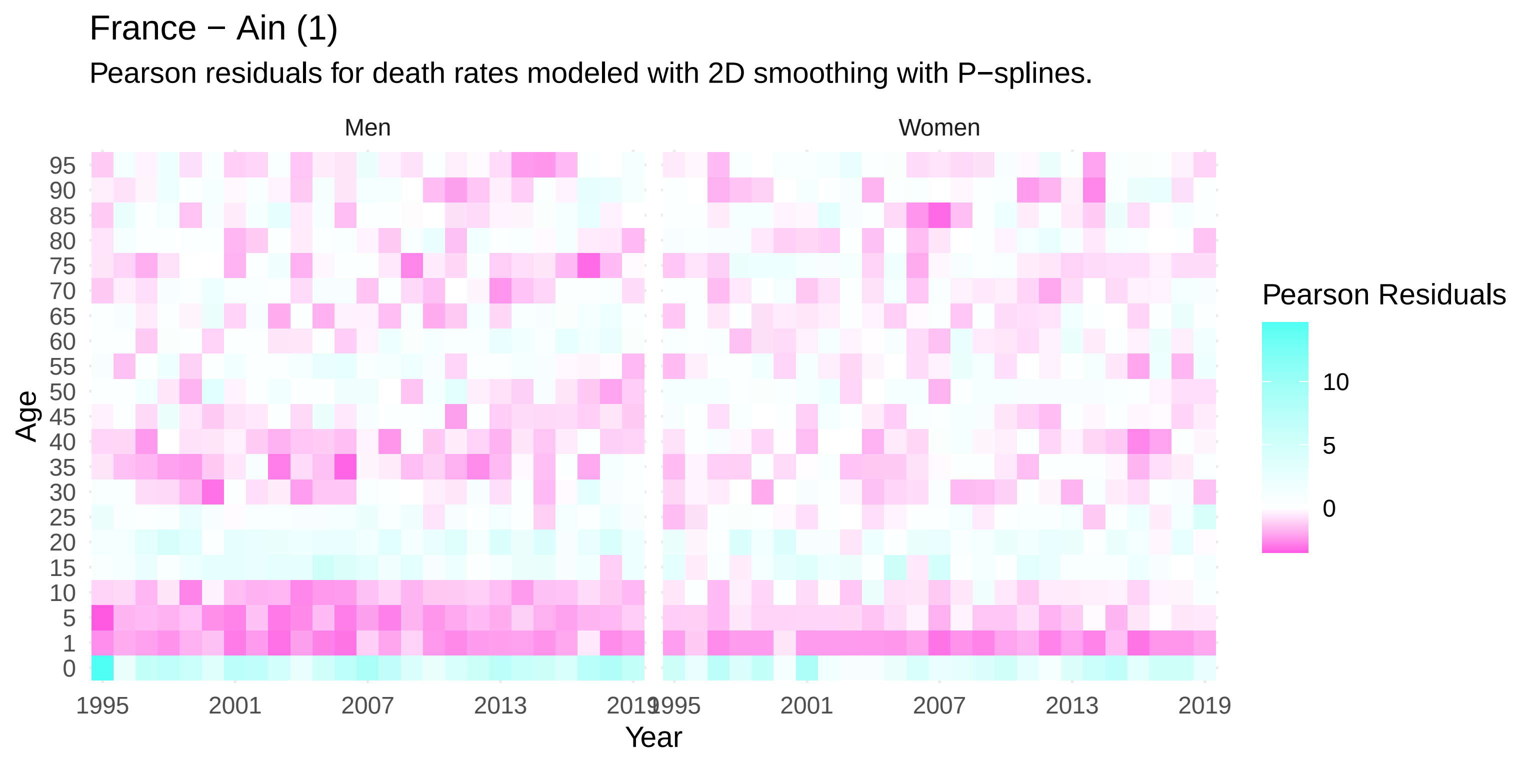
France − Ain (1)
Pearson residuals for death rates modeled with 2D smoothing with P−splines.
Men
Women
Age
95
90
85
80
75
70
65
60
55
50
45
40
35
30
25
20
15
10
5
1
0
1995
2001
2007
2013
2019
Year
Pearson Residuals
10
5
0

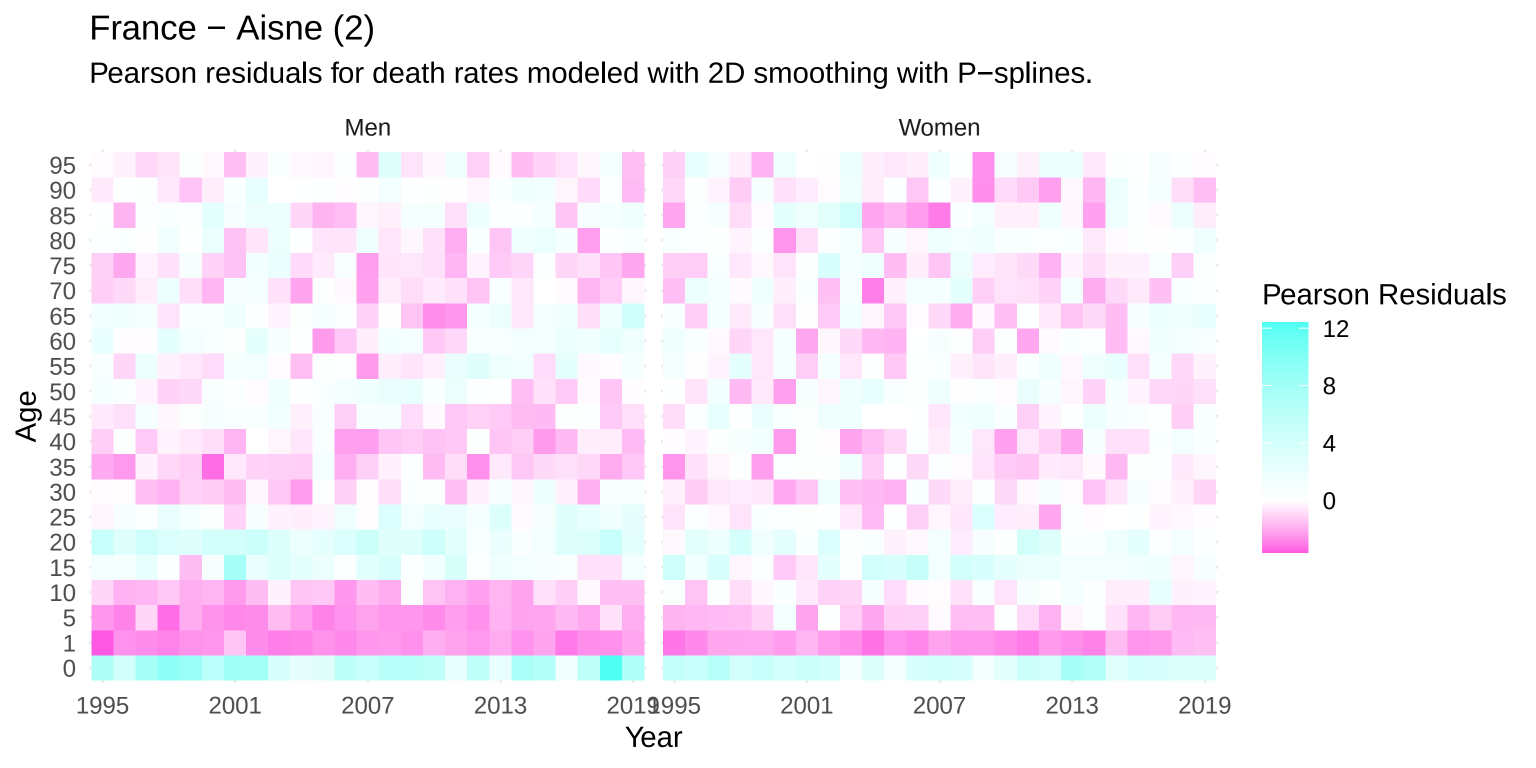

France − Aisne (2)
Pearson residuals for death rates modeled with 2D smoothing with P−splines.
Men
Women
Age
95
90
85
80
75
70
65
60
55
50
45
40
35
30
25
20
15
10
5
1
0
1995
2001
2007
2013
2019
Year
Pearson Residuals
12
8
4
0

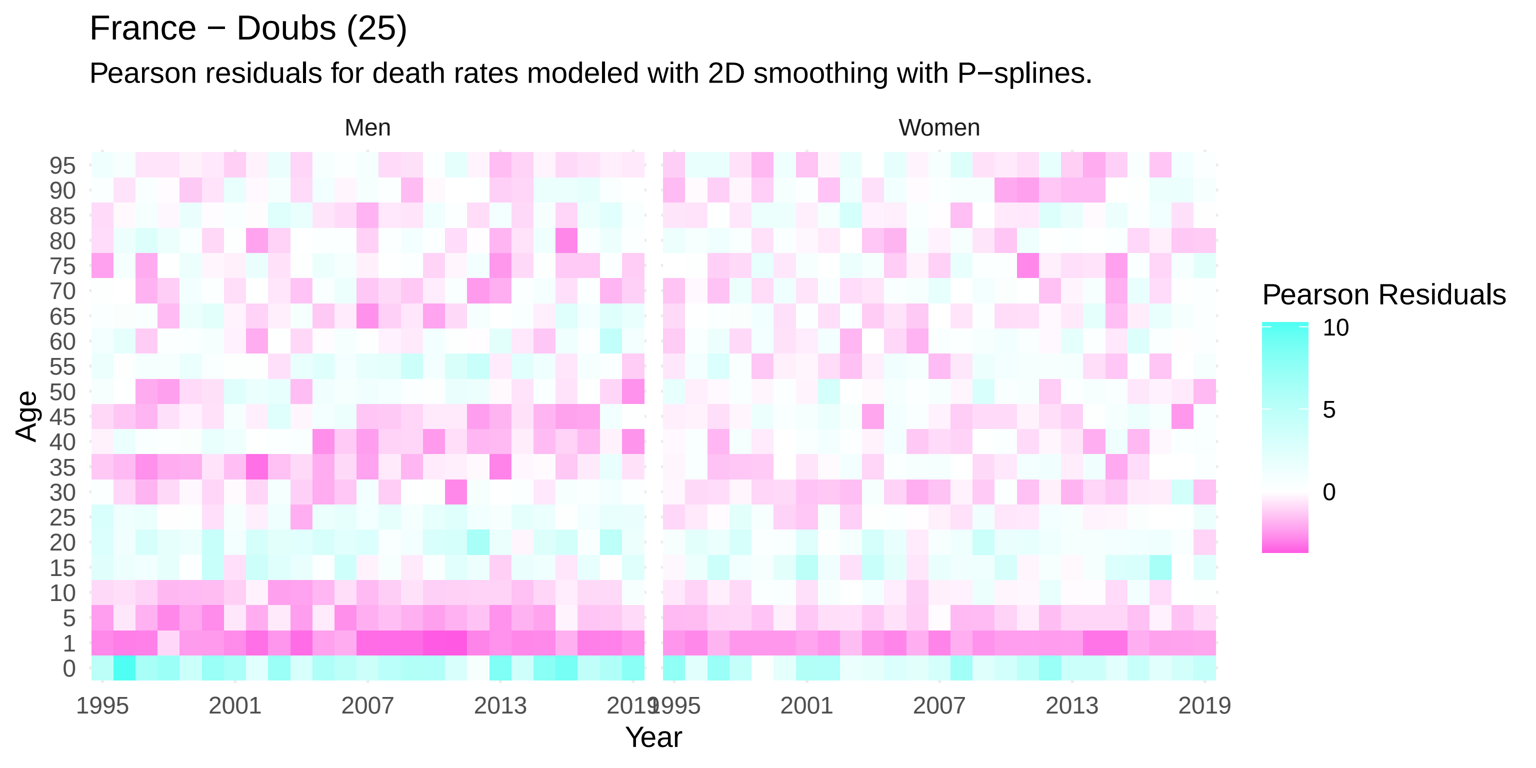
France − Doubs (25)
Pearson residuals for death rates modeled with 2D smoothing with P−splines.
Men
Women
Age
95
90
85
80
75
70
65
60
55
50
45
40
35
30
25
20
15
10
5
1
0
1995
2001
2007
2013
2019
Year
Pearson Residuals
10
5
0

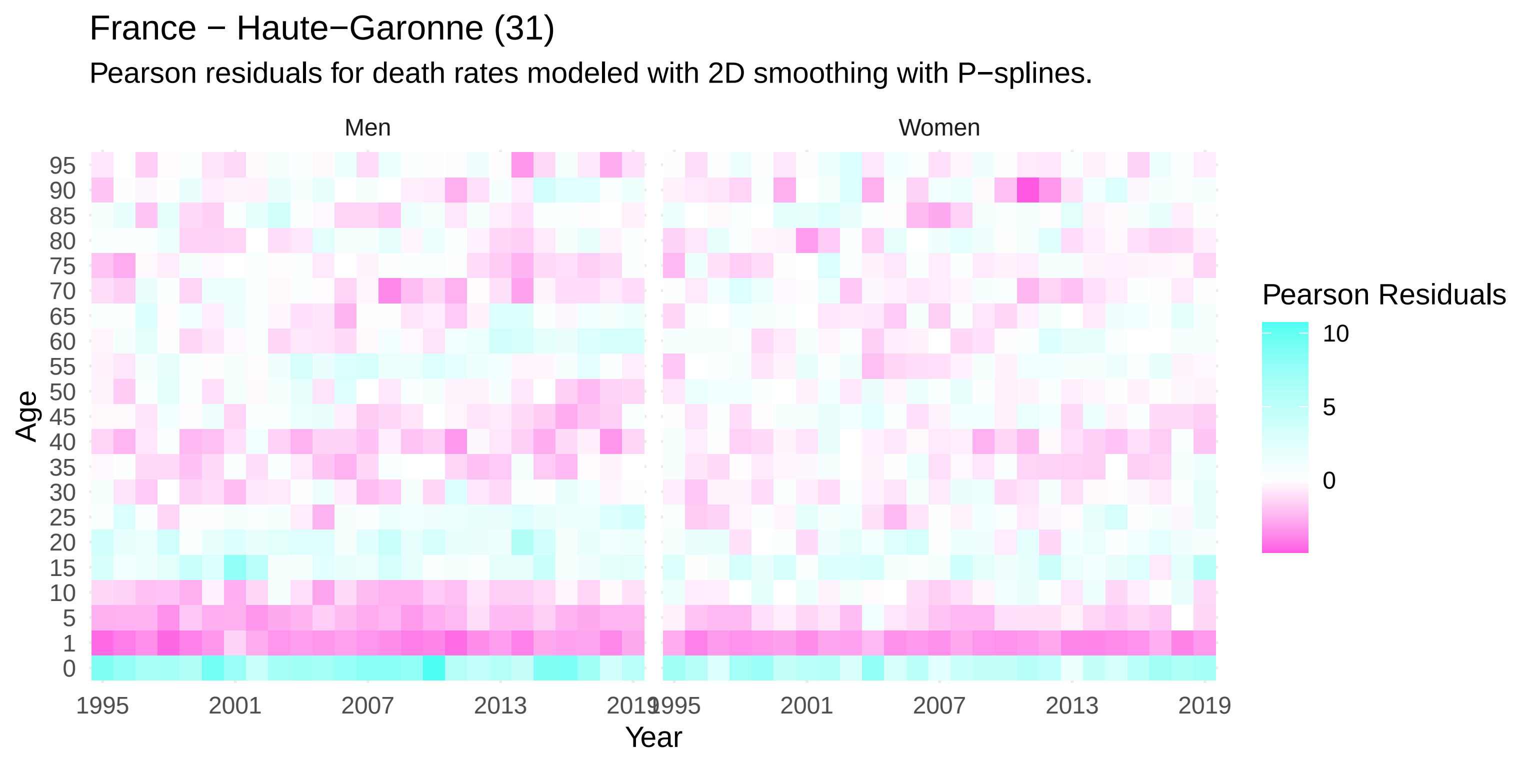
France − Haute−Garonne (31)
Pearson residuals for death rates modeled with 2D smoothing with P−splines.
Men
Women
Age
95
90
85
80
75
70
65
60
55
50
45
40
35
30
25
20
15
10
5
1
0
1995
2001
2007
2013
2019
Year
Pearson Residuals
10
5
0

France − Jura (39)

Pearson residuals for death rates modeled with 2D smoothing with P−splines.

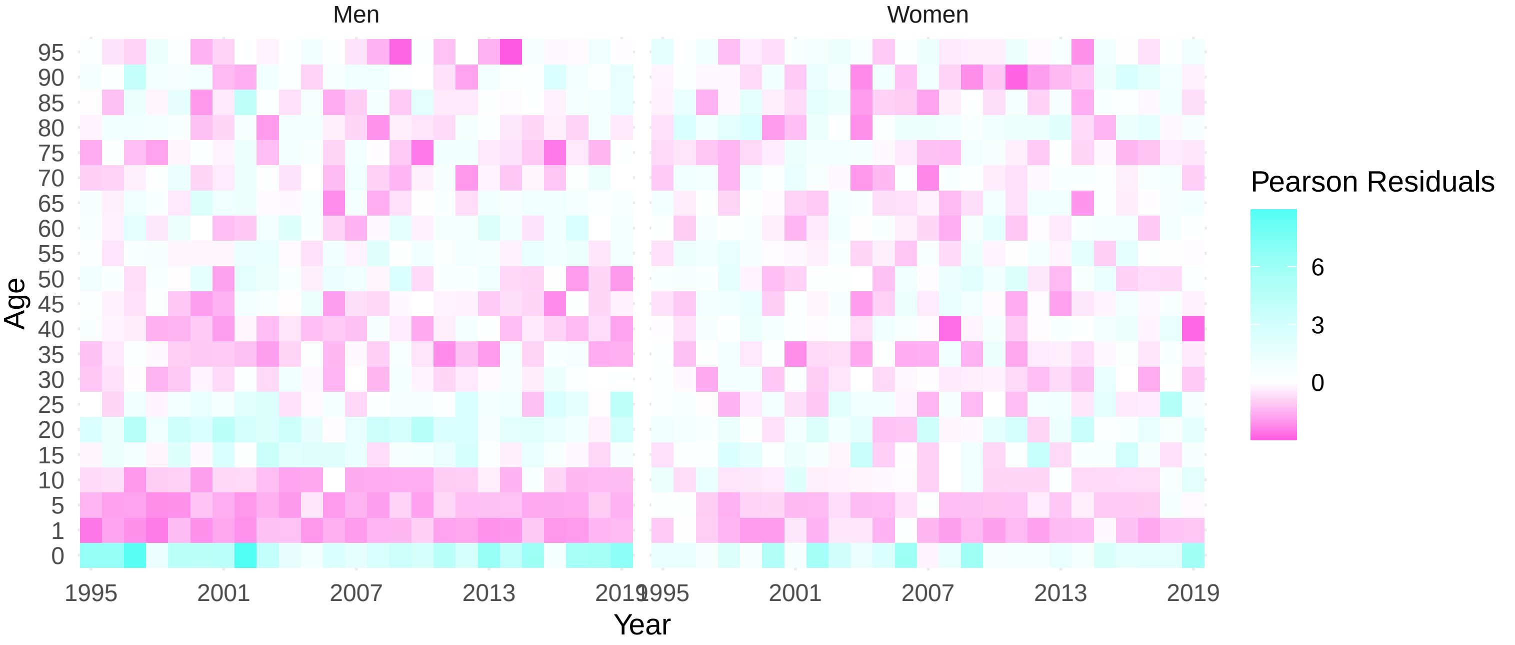

France − Alpes−de−Haute−Provence (4)

Pearson residuals for death rates modeled with 2D smoothing with P−splines.

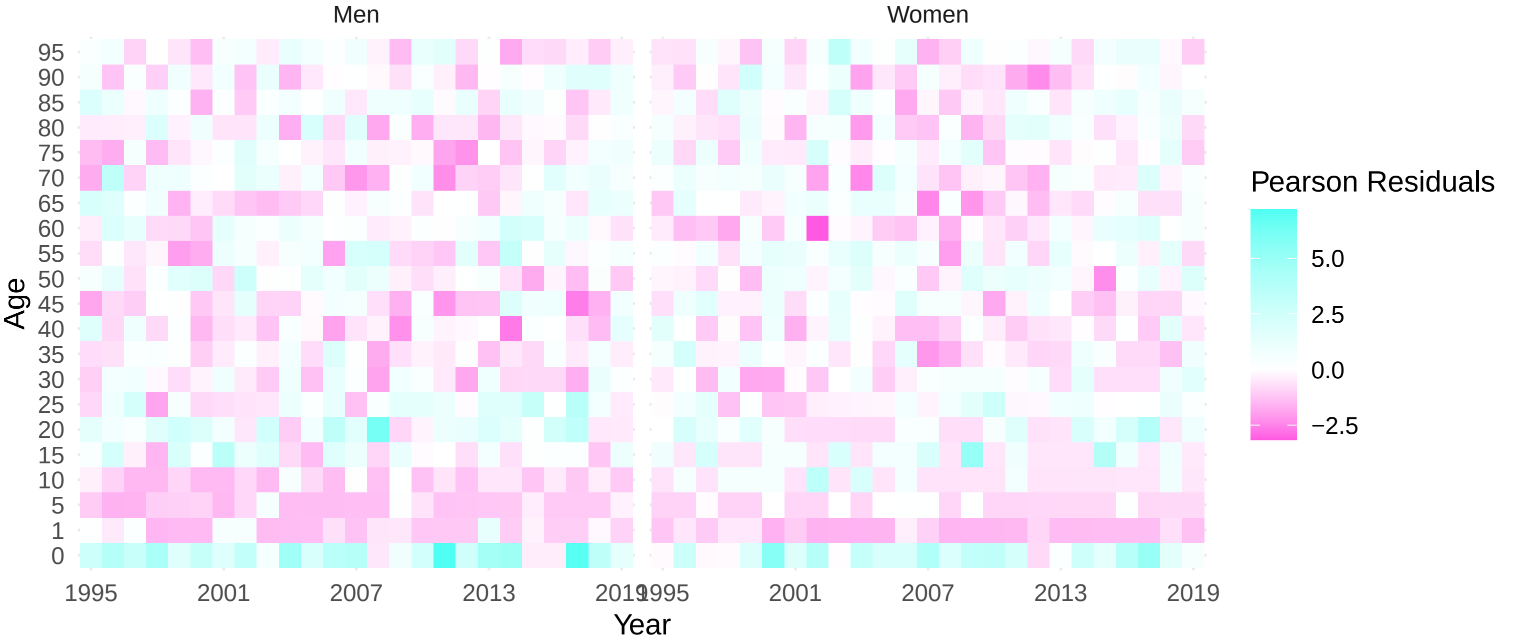

France − Hautes−Alpes (5)

Pearson residuals for death rates modeled with 2D smoothing with P−splines.

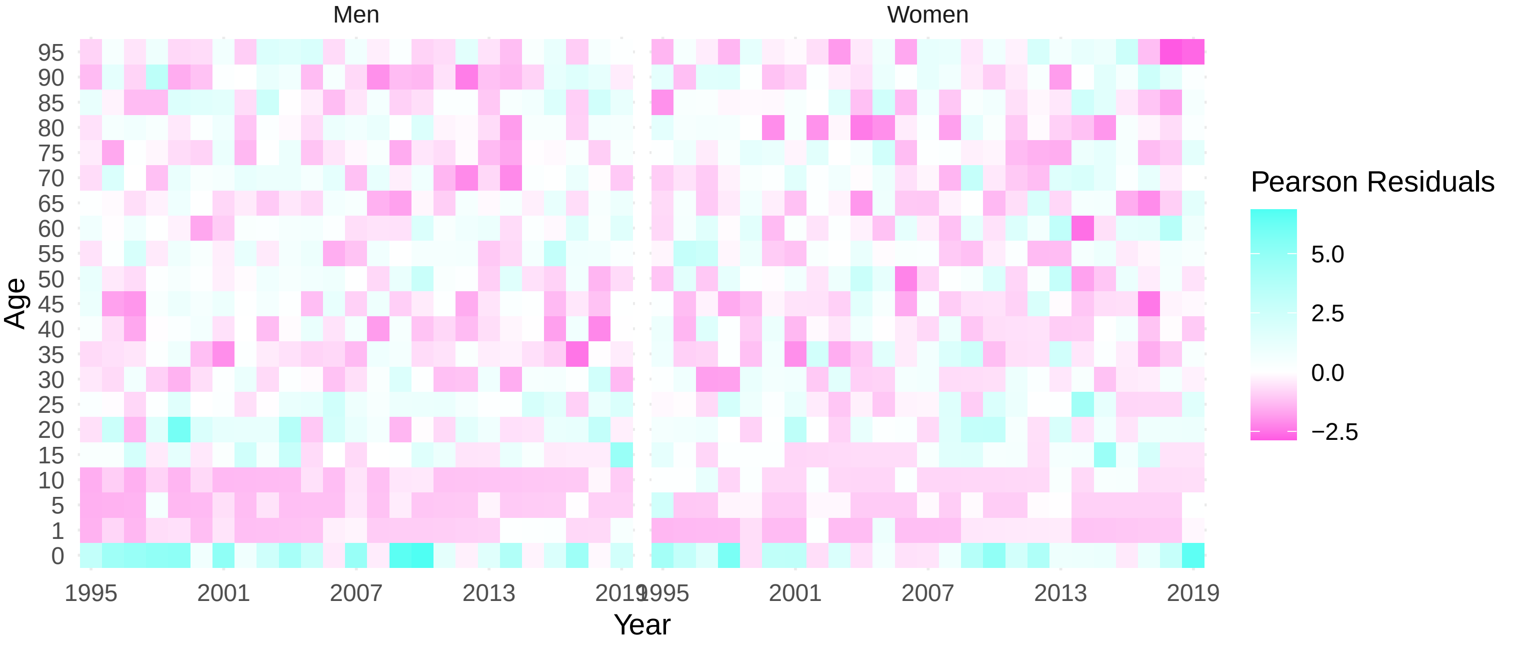

France − Meurthe−et−Moselle (54)

Pearson residuals for death rates modeled with 2D smoothing with P−splines.

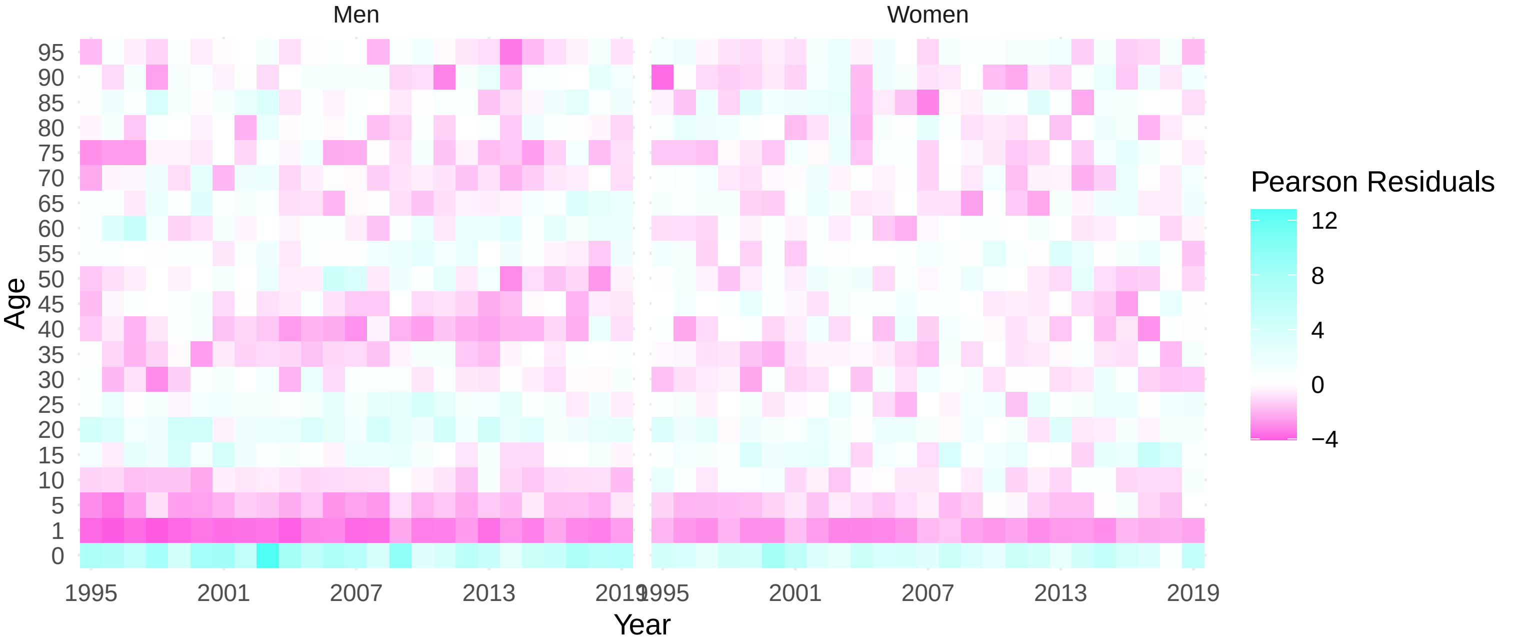

France − Meuse (55)

Pearson residuals for death rates modeled with 2D smoothing with P−splines.

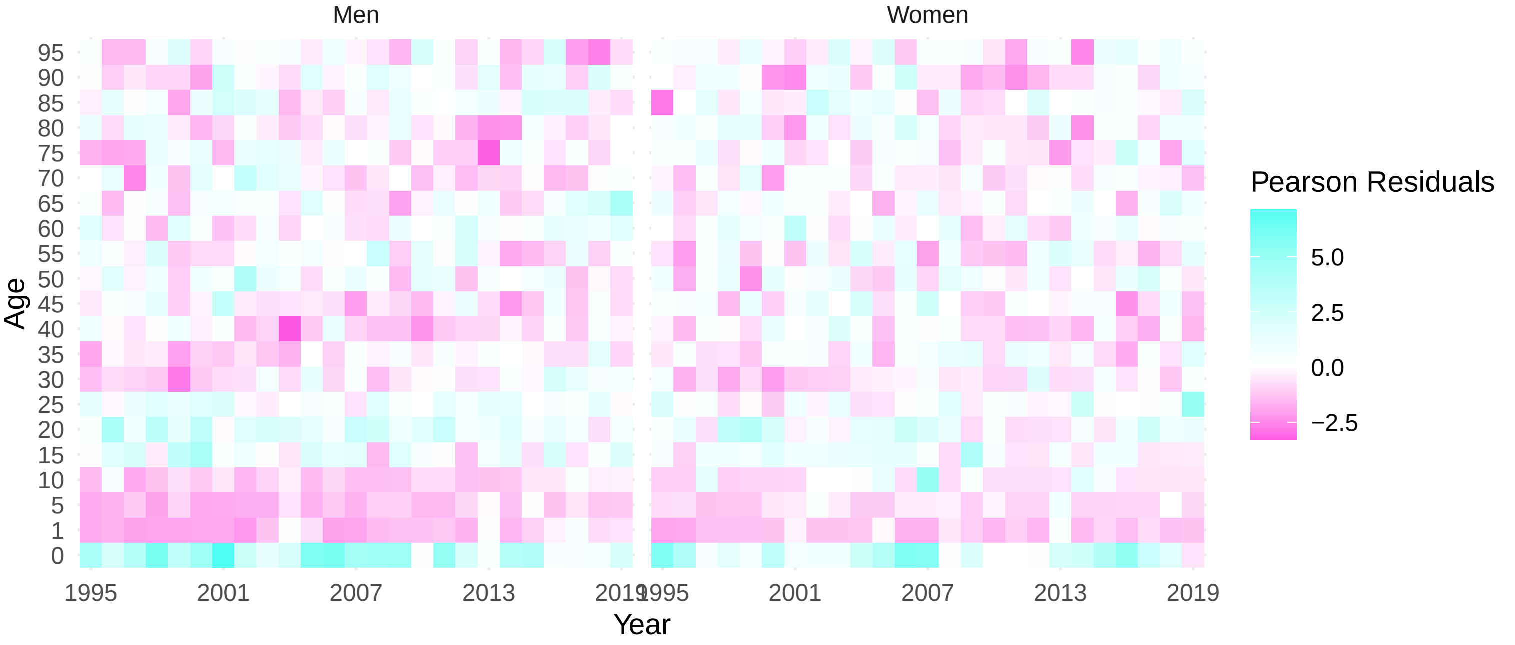

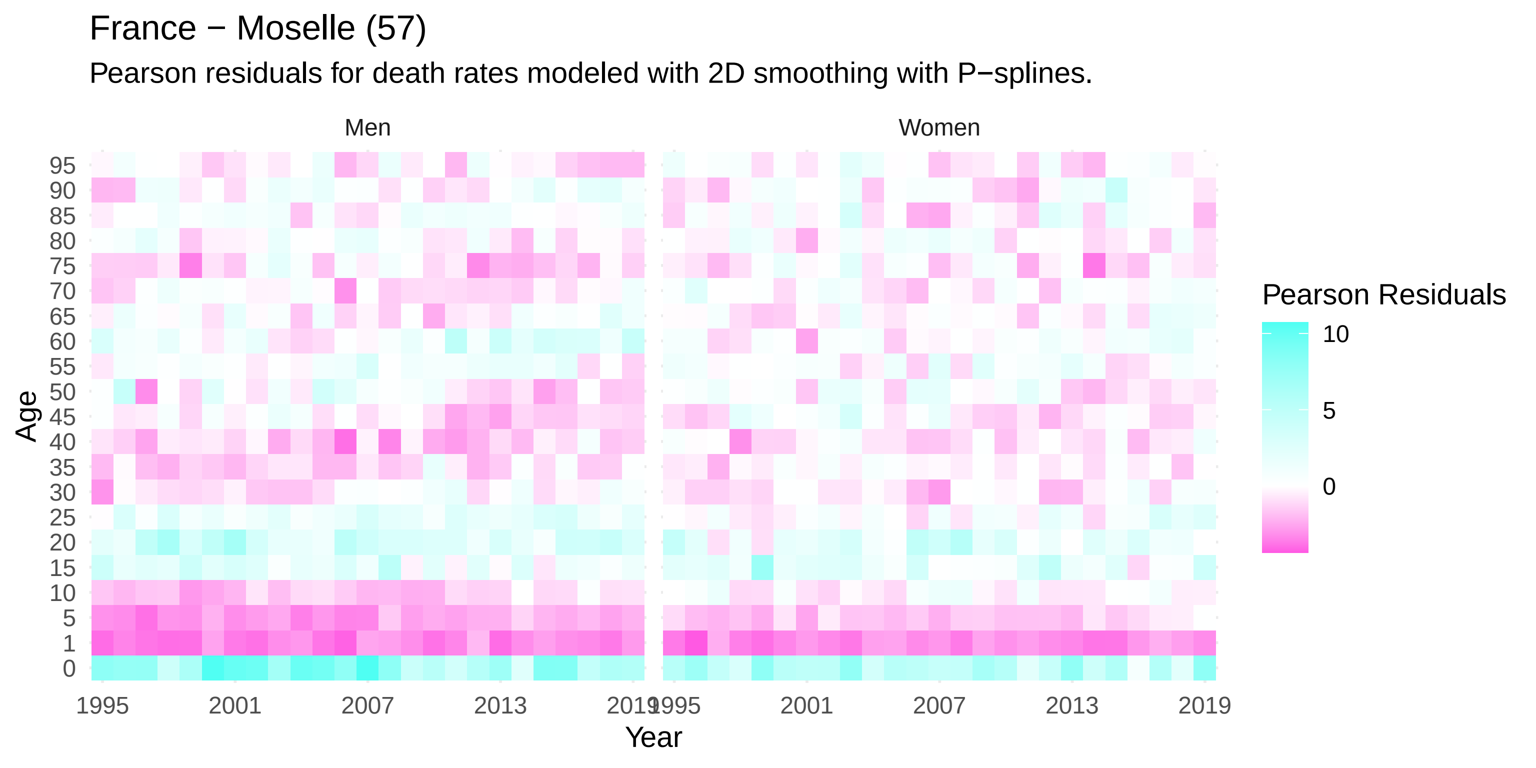

France − Moselle (57)
Pearson residuals for death rates modeled with 2D smoothing with P−splines.
Men
Women
Age
95
90
85
80
75
70
65
60
55
50
45
40
35
30
25
20
15
10
5
1
0
1995
2001
2007
2013
2019
Year
Pearson Residuals
10
5
0

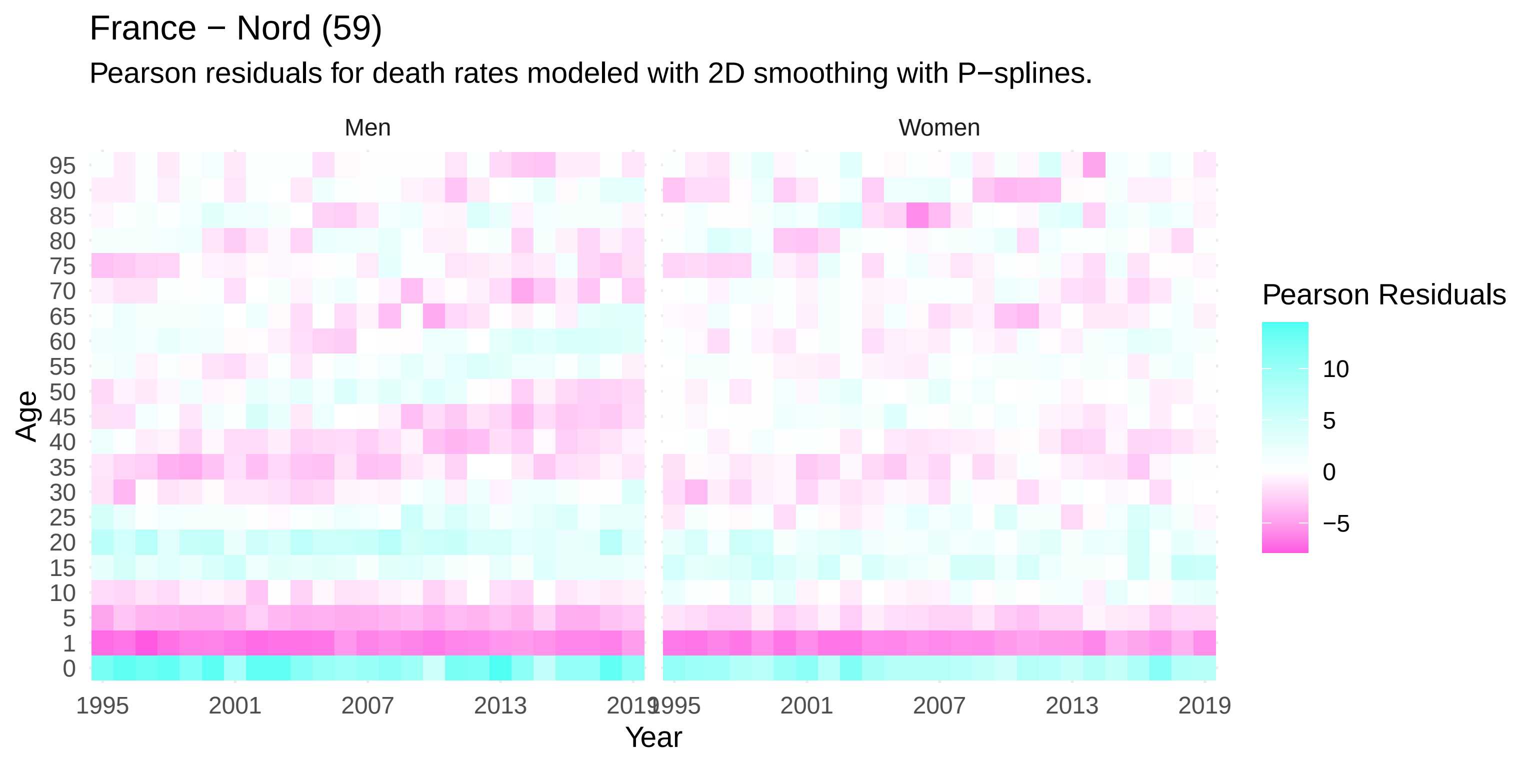
France − Nord (59)
Pearson residuals for death rates modeled with 2D smoothing with P−splines.
Men
Women
Age
95
90
85
80
75
70
65
60
55
50
45
40
35
30
25
20
15
10
5
1
0
1995
2001
2007
2013
2019
Year
Pearson Residuals
10
5
0
−5

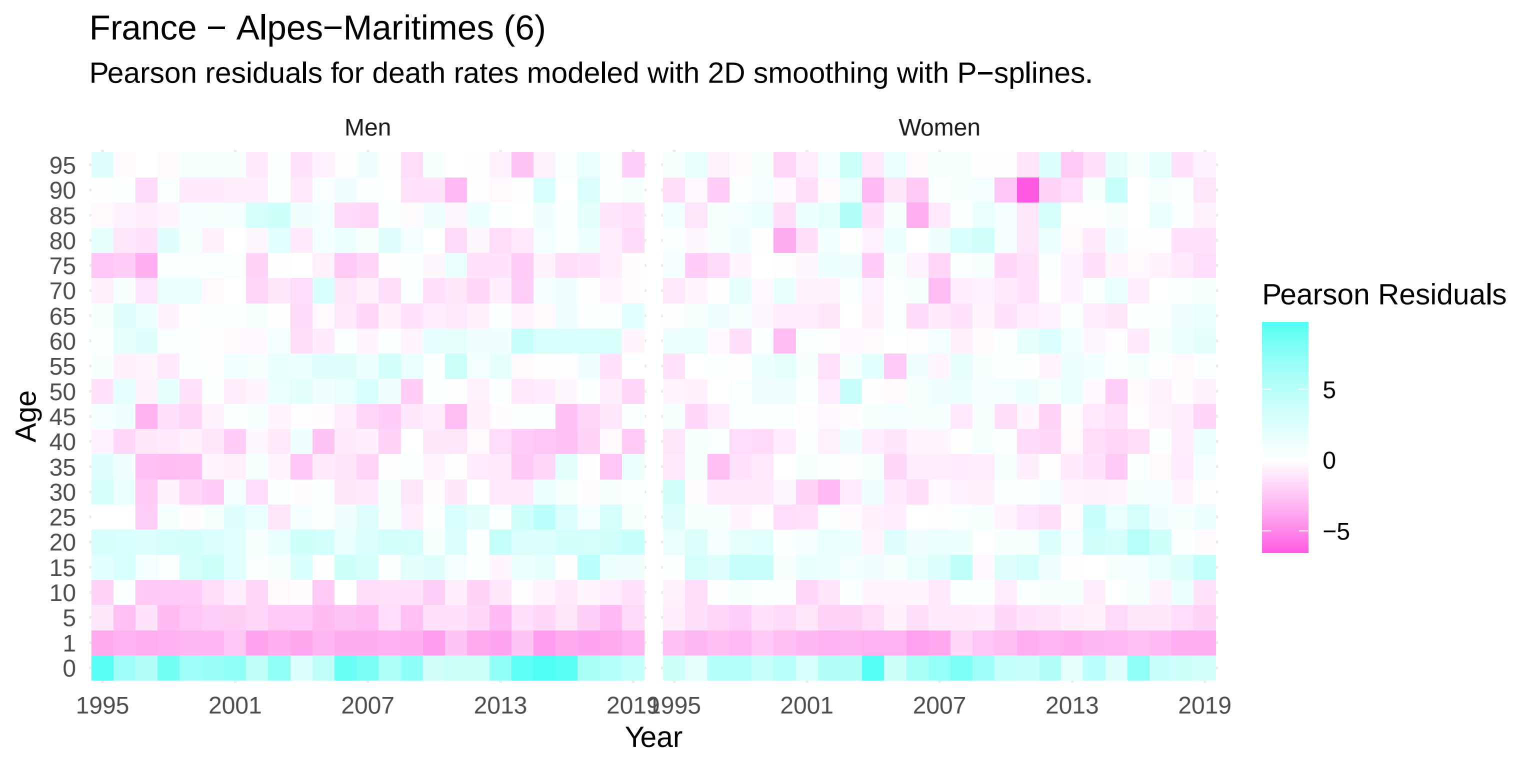
France − Alpes−Maritimes (6)
Pearson residuals for death rates modeled with 2D smoothing with P−splines.
Men
Women
Age
Year
95
90
85
80
75
70
65
60
55
50
45
40
35
30
25
20
15
10
5
1
0
1995
2001
2007
2013
2019
Pearson Residuals
5
0
−5

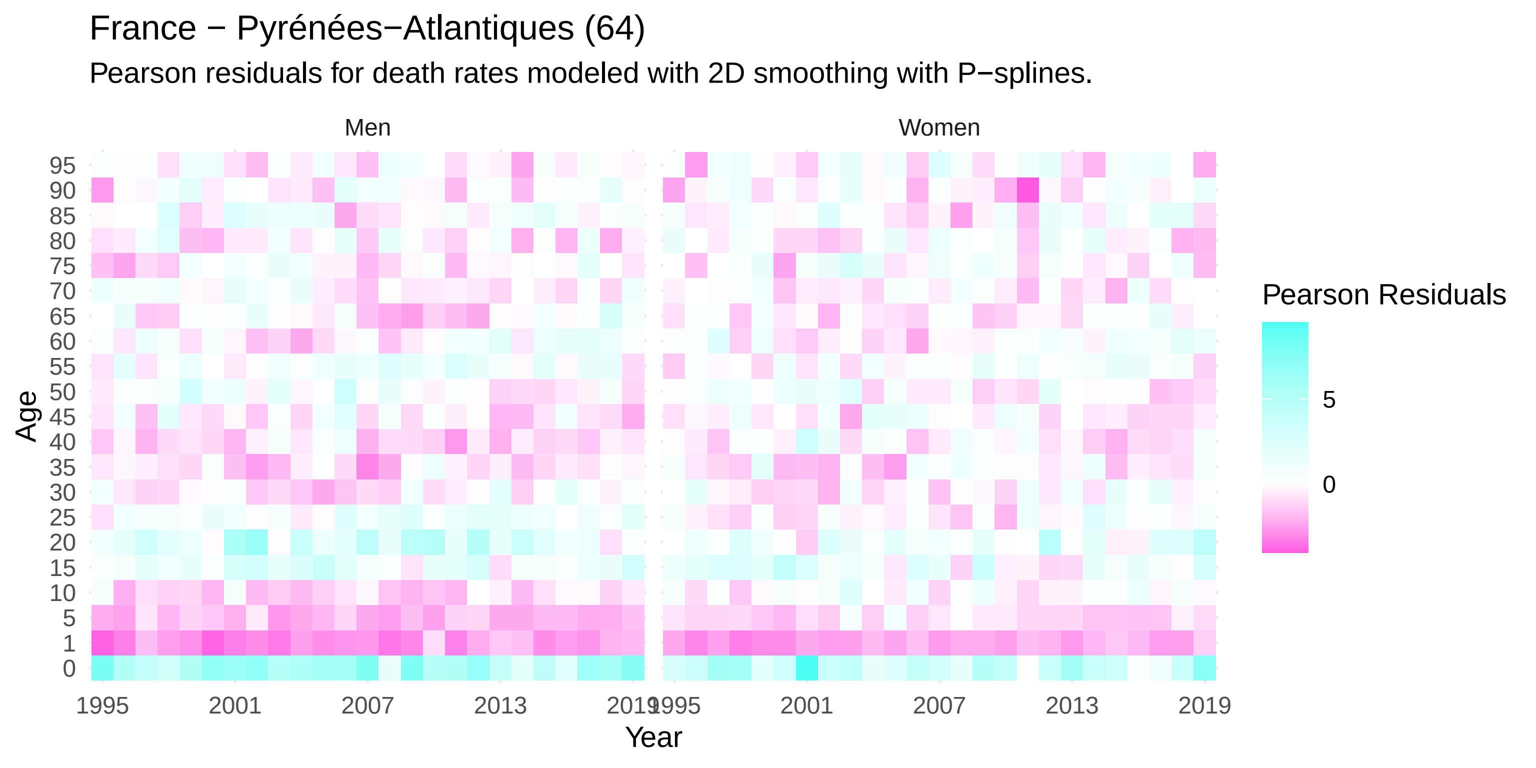

France − Pyrénées−Atlantiques (64)
Pearson residuals for death rates modeled with 2D smoothing with P−splines.
Men
Women
Age
95
90
85
80
75
70
65
60
55
50
45
40
35
30
25
20
15
10
5
1
0
1995
2001
2007
2013
2019
Year
Pearson Residuals
5
0

France − Hautes−Pyrénées (65)

Pearson residuals for death rates modeled with 2D smoothing with P−splines.

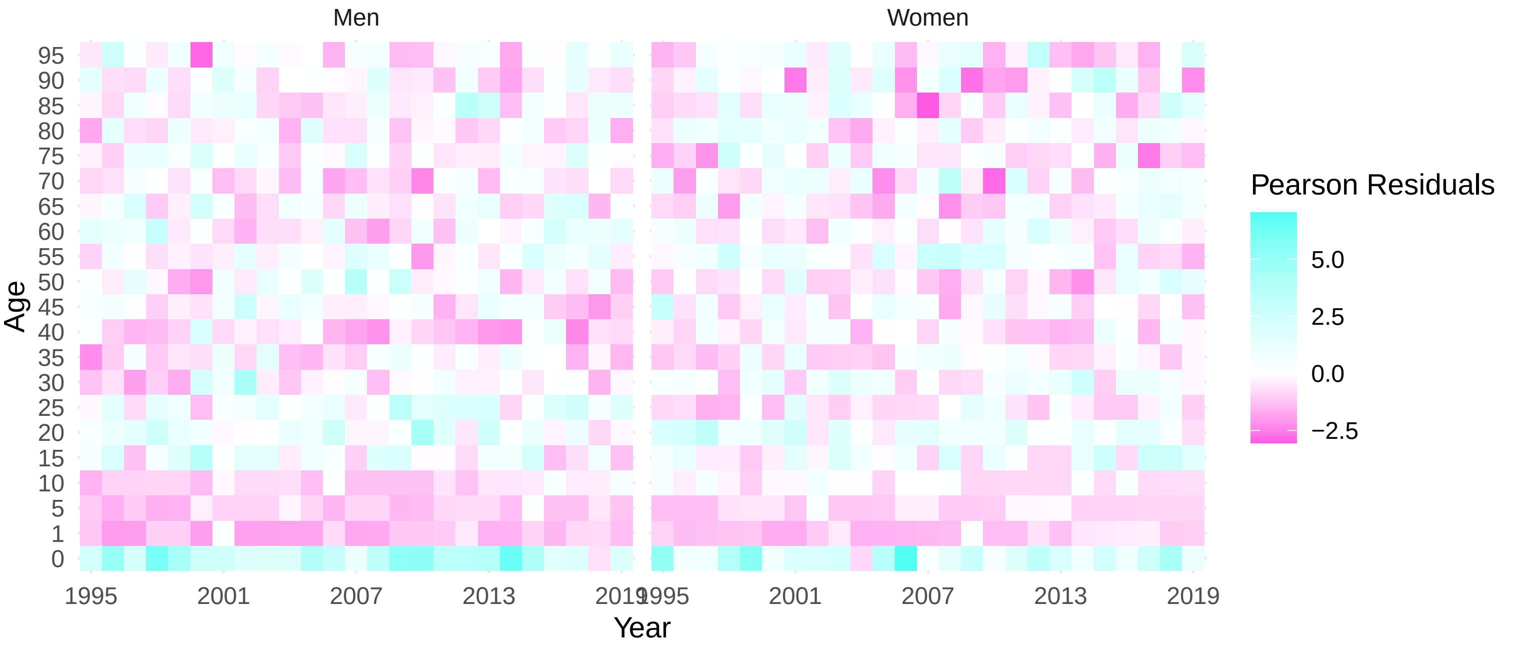

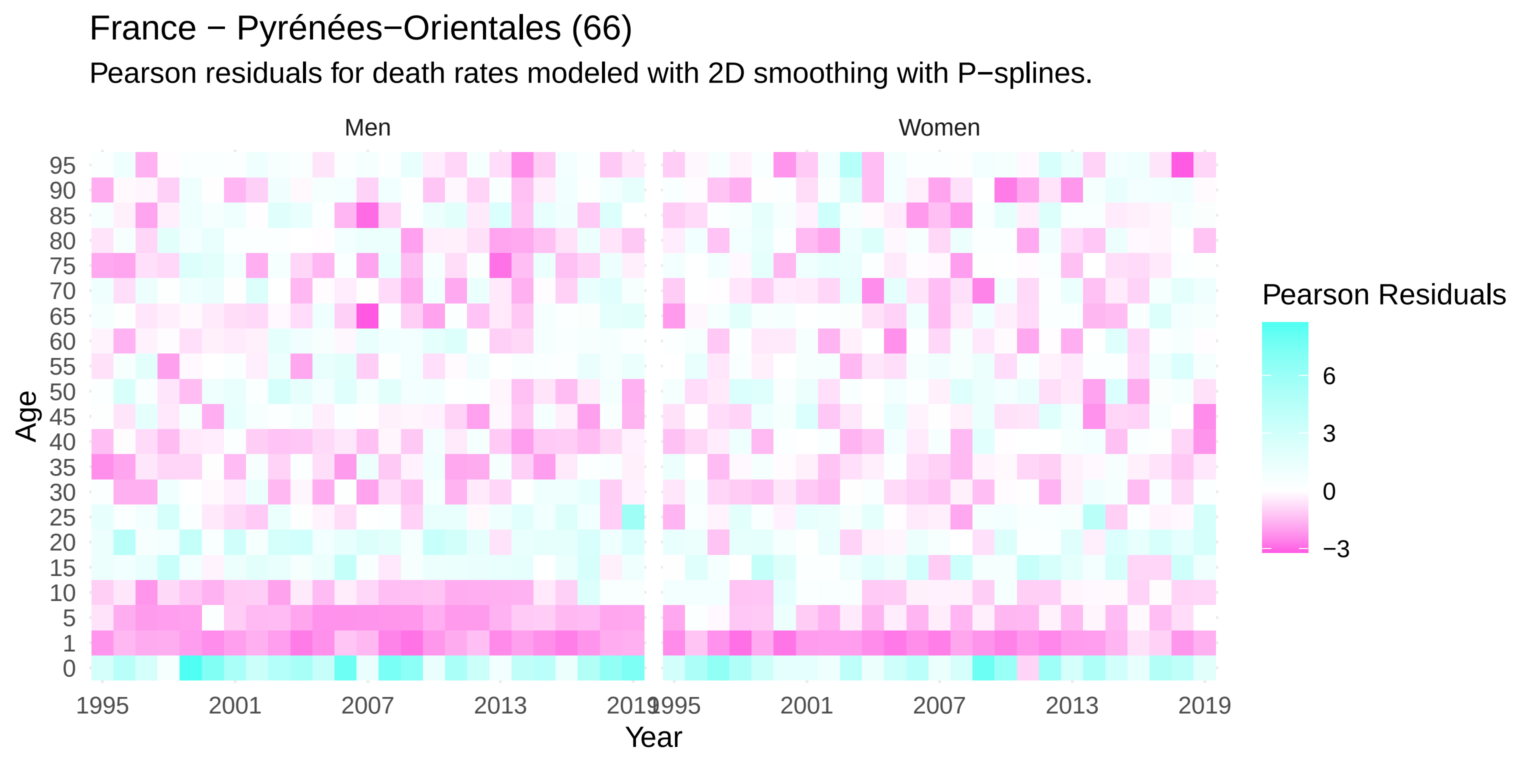
France − Pyrénées−Orientales (66)
Pearson residuals for death rates modeled with 2D smoothing with P−splines.
Men
Women
Age
95
90
85
80
75
70
65
60
55
50
45
40
35
30
25
20
15
10
5
1
0
1995
2001
2007
2013
2019
Year
Pearson Residuals
6
3
0
−3

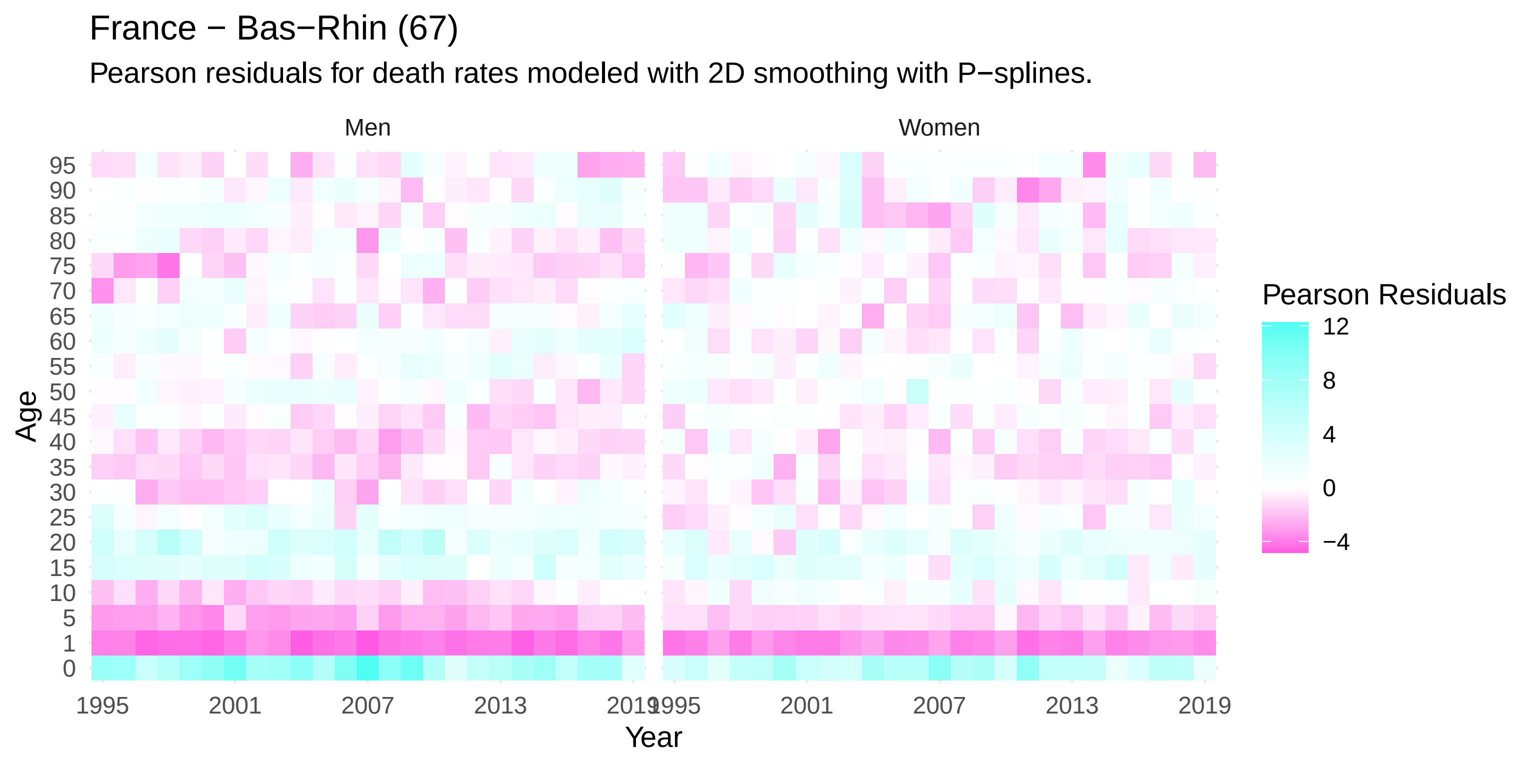

France − Bas−Rhin (67)
Pearson residuals for death rates modeled with 2D smoothing with P−splines.
Men
Women
Age
95
90
85
80
75
70
65
60
55
50
45
40
35
30
25
20
15
10
5
1
0
1995
2001
2007
2013
2019
Year
Pearson Residuals
12
8
4
0
−4

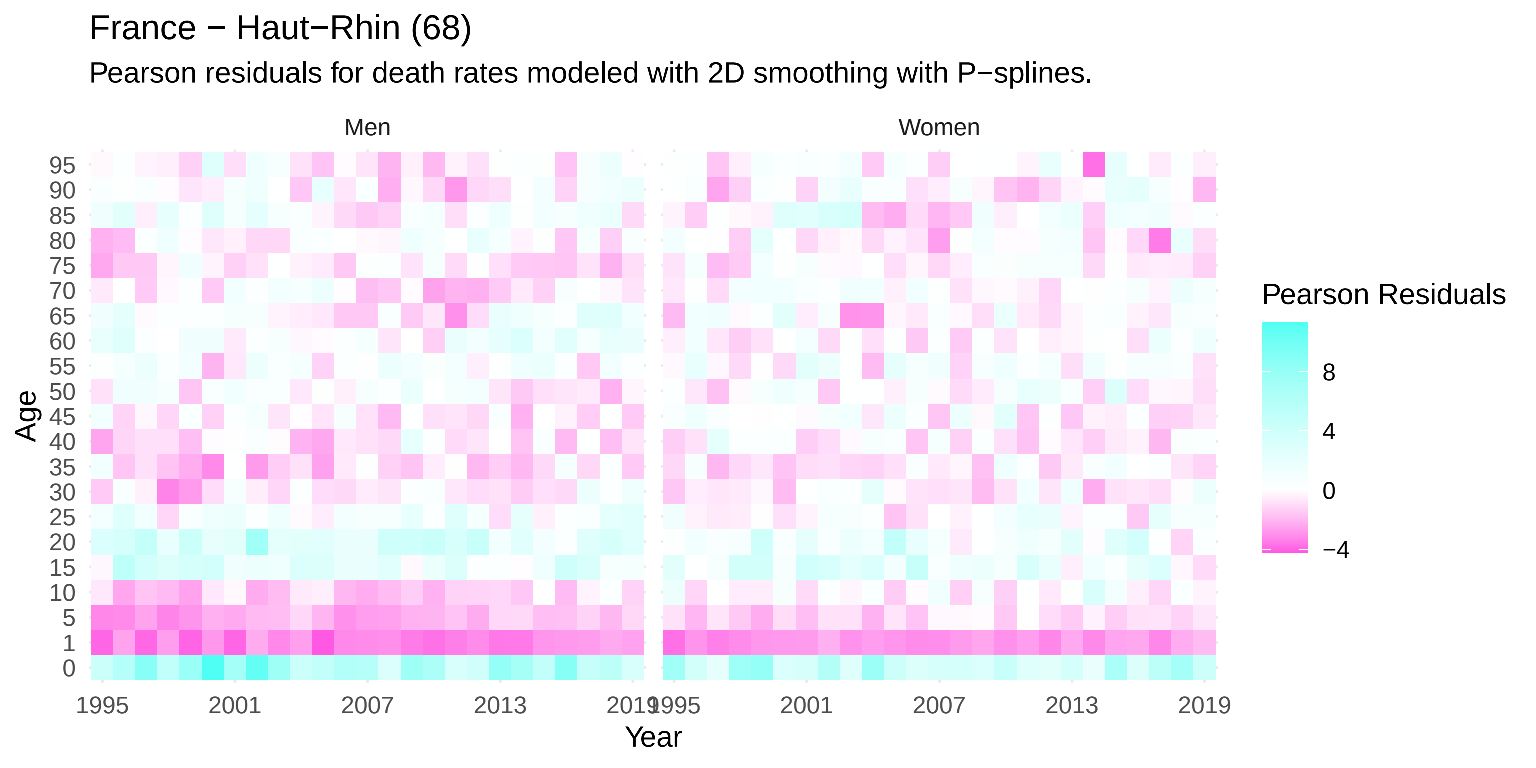

France − Haut−Rhin (68)
Pearson residuals for death rates modeled with 2D smoothing with P−splines.
Men
Women
Age
95
90
85
80
75
70
65
60
55
50
45
40
35
30
25
20
15
10
5
1
0
1995
2001
2007
2013
2019
Year
Pearson Residuals
8
4
0
−4

France − Haute−Saône (70)

Pearson residuals for death rates modeled with 2D smoothing with P−splines.

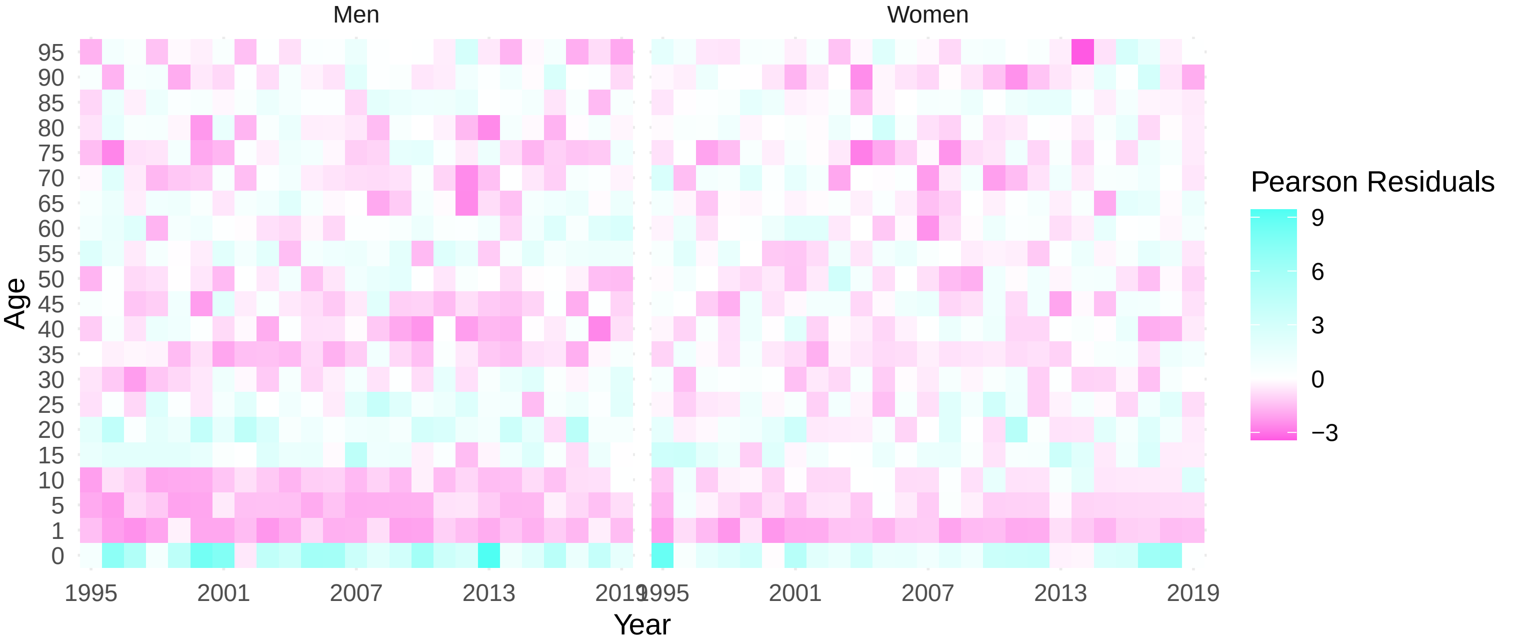

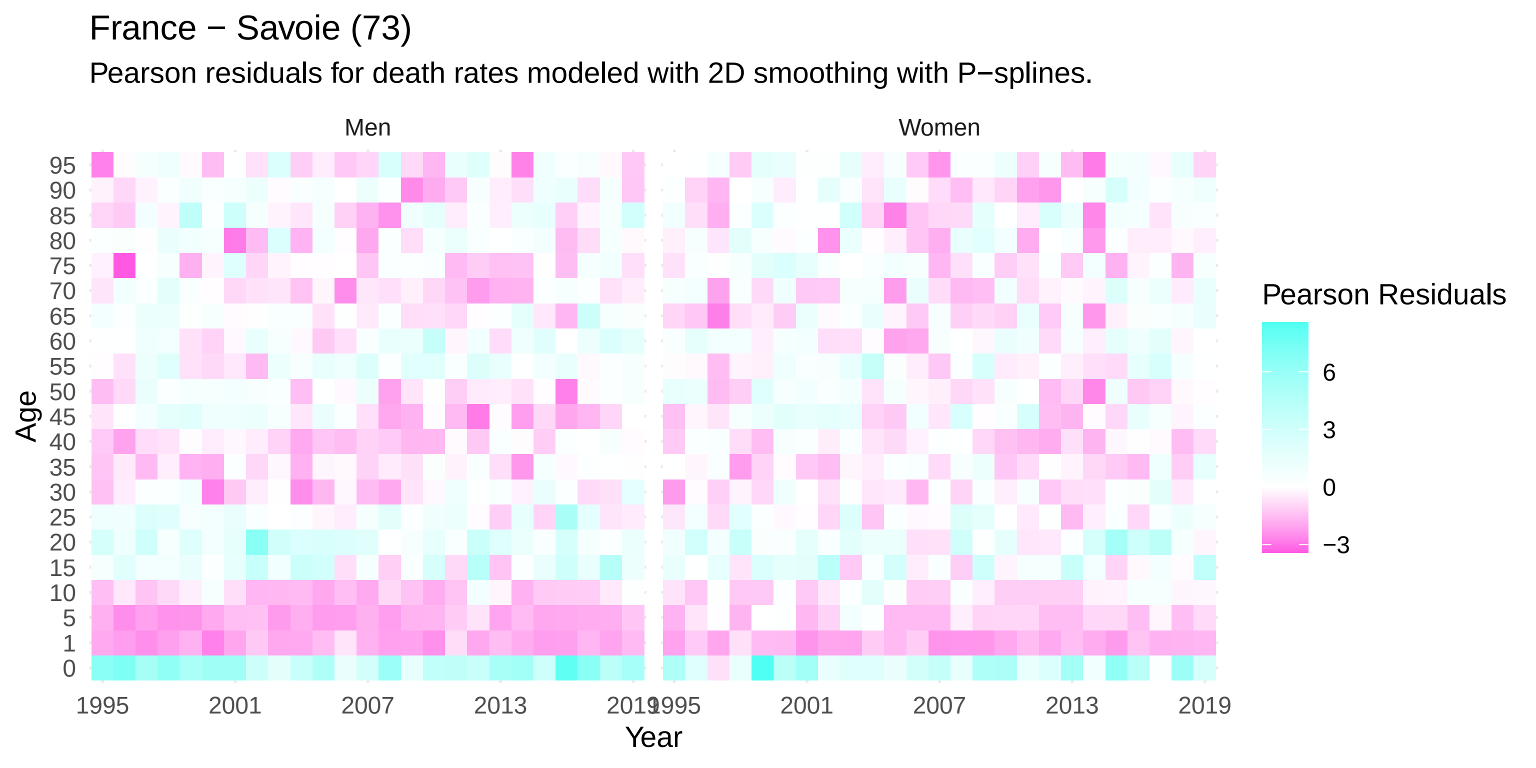
France − Savoie (73)
Pearson residuals for death rates modeled with 2D smoothing with P−splines.
Men
Women
Age
95
90
85
80
75
70
65
60
55
50
45
40
35
30
25
20
15
10
5
1
0
1995
2001
2007
2013
2019
Year
Pearson Residuals
6
3
0
−3

France − Haute−Savoie (74)

Pearson residuals for death rates modeled with 2D smoothing with P−splines.

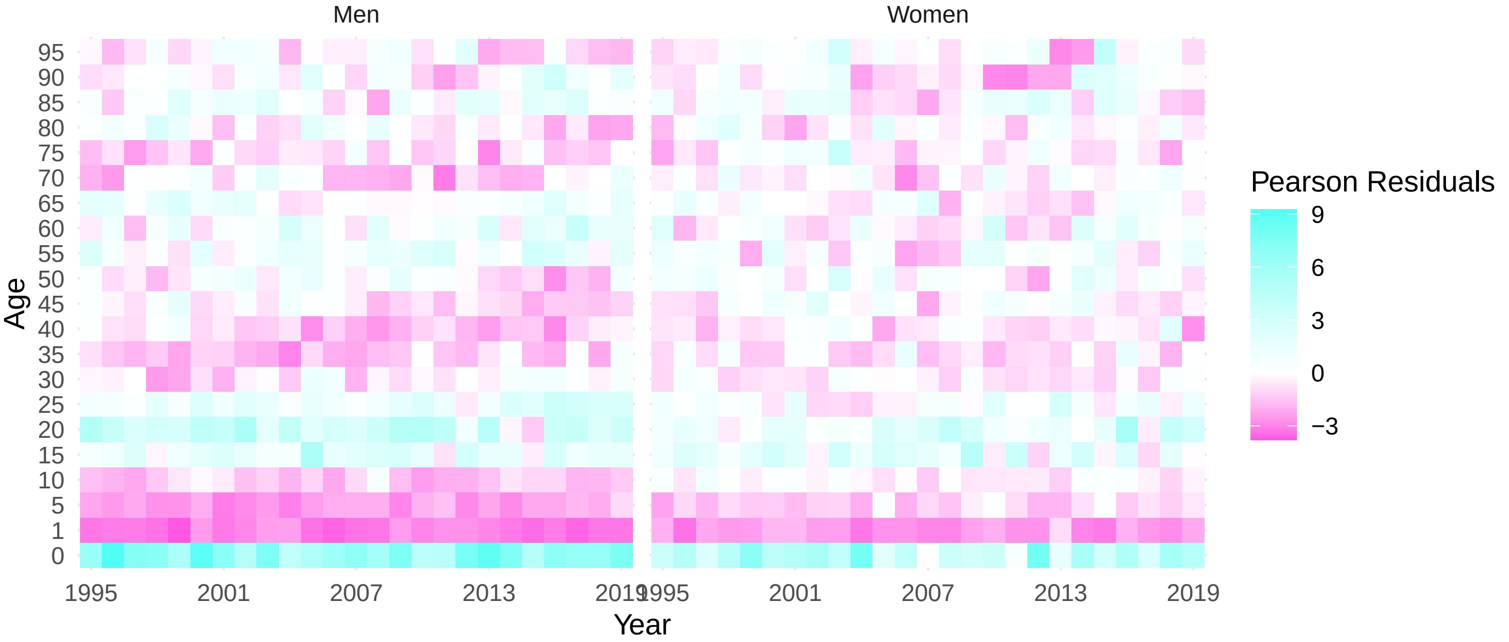

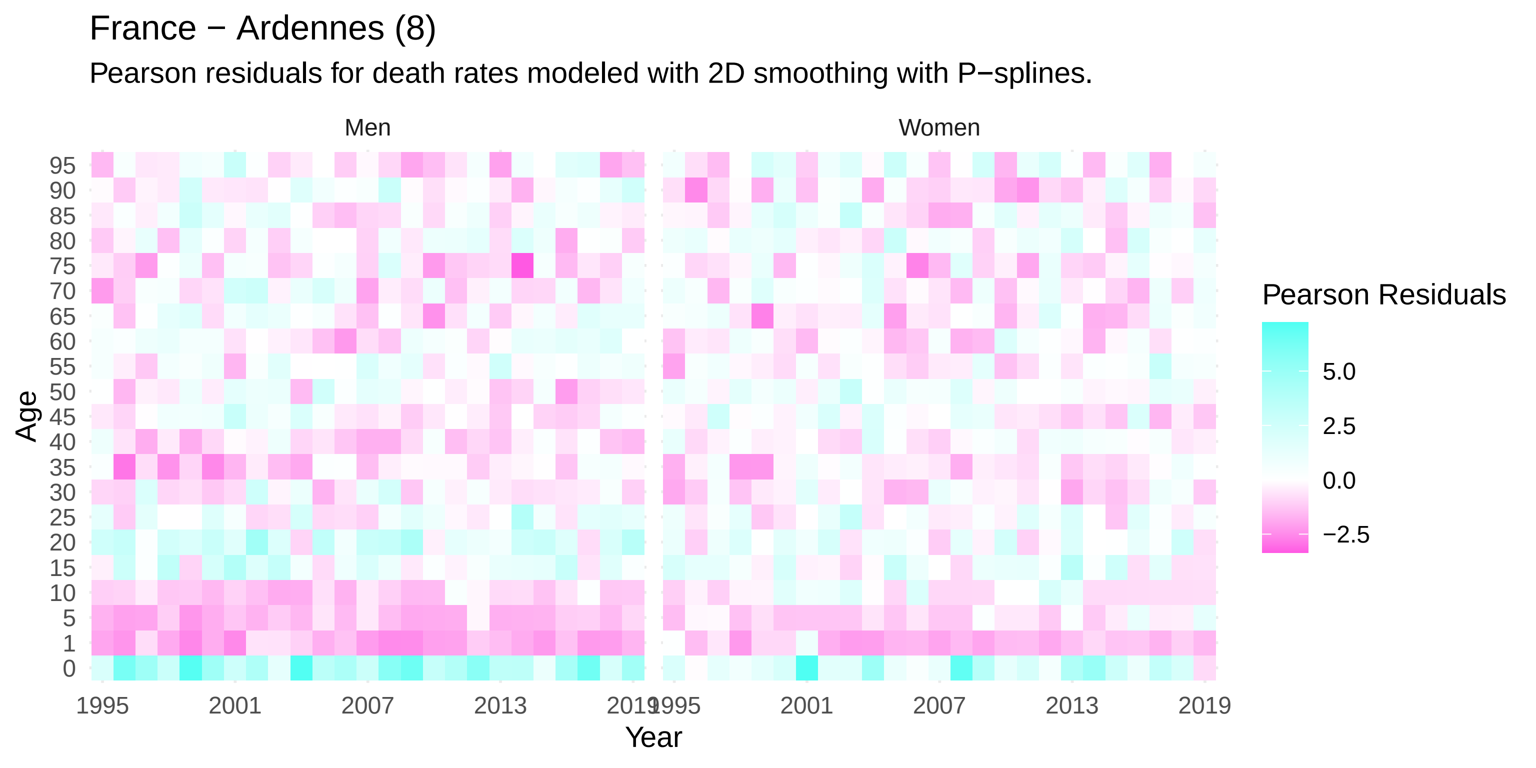
France − Ardennes (8)
Pearson residuals for death rates modeled with 2D smoothing with P−splines.
Men
Women
Age
Year
Pearson Residuals
5.0
2.5
0.0
−2.5
95
90
85
80
75
70
65
60
55
50
45
40
35
30
25
20
15
10
5
1
0
1995
2001
2007
2013
2019

France − Ariège (9)

Pearson residuals for death rates modeled with 2D smoothing with P−splines.

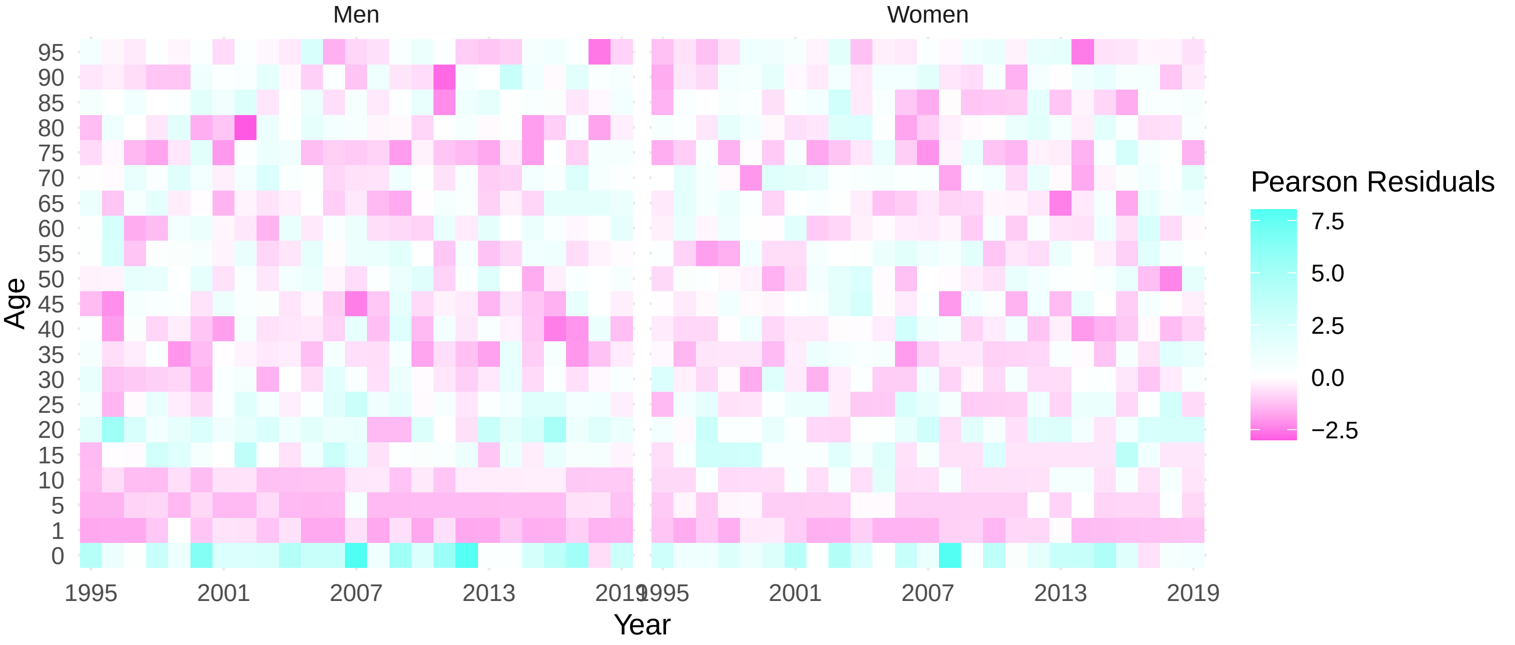

France − Territoire de Belfort (90)

Pearson residuals for death rates modeled with 2D smoothing with P−splines.

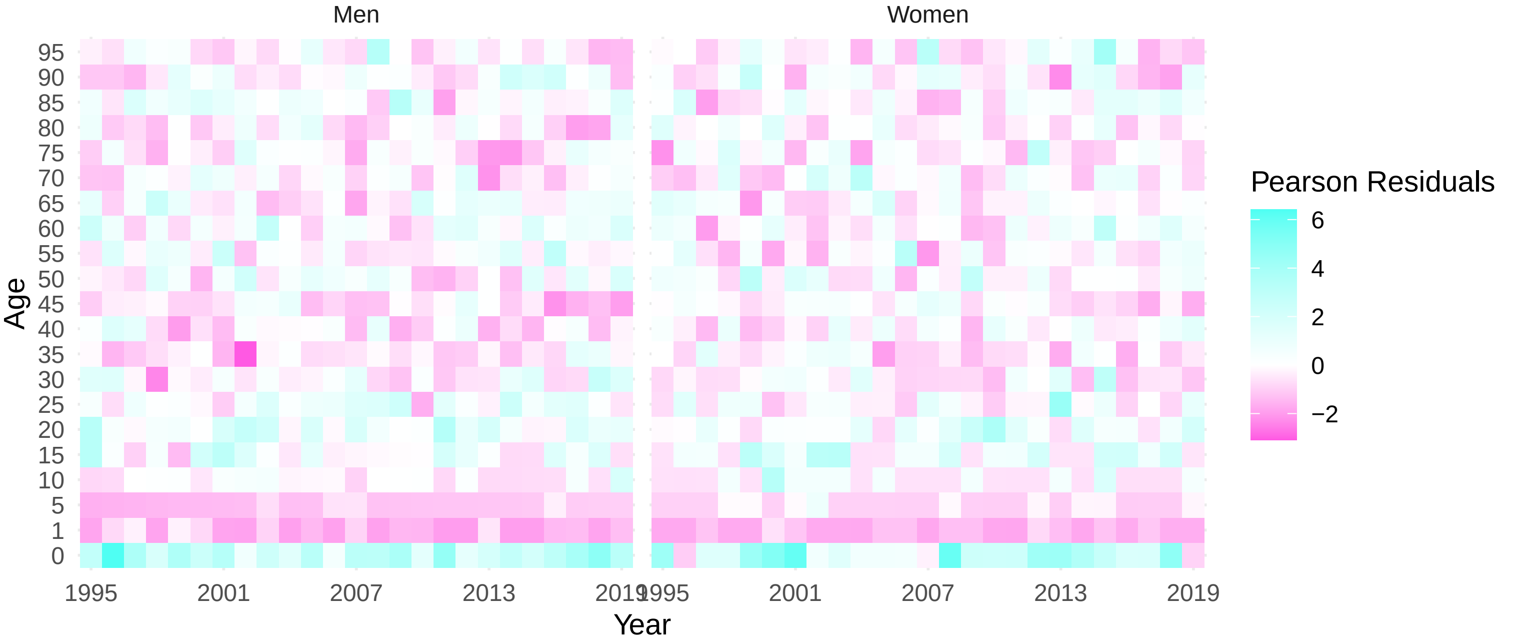

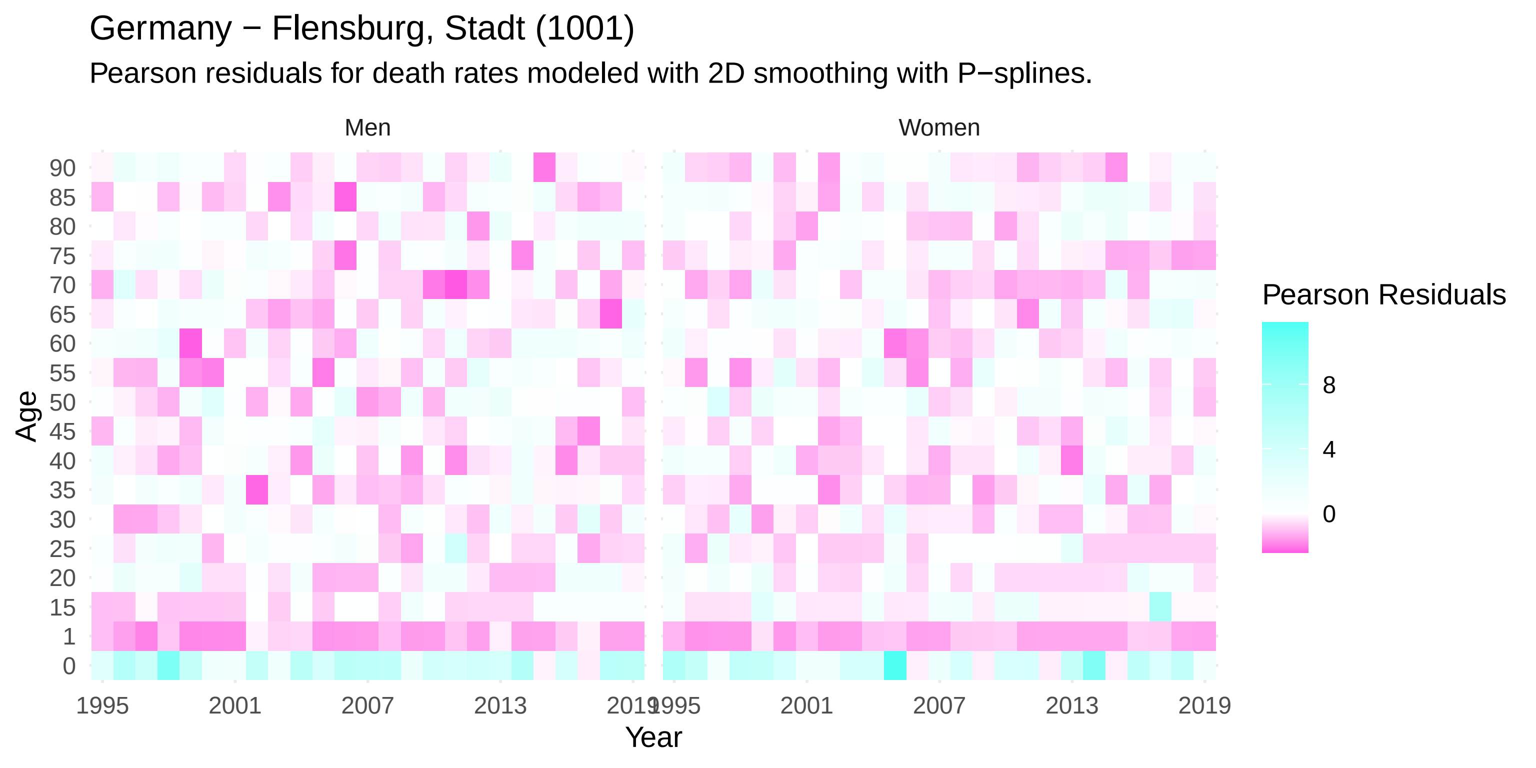

Germany − Flensburg, Stadt (1001)
Pearson residuals for death rates modeled with 2D smoothing with P−splines.
Men
Women
Age
90
85
80
75
70
65
60
55
50
45
40
35
30
25
20
15
1
0
1995
2001
2007
2013
2019
Year
Pearson Residuals
8
4
0

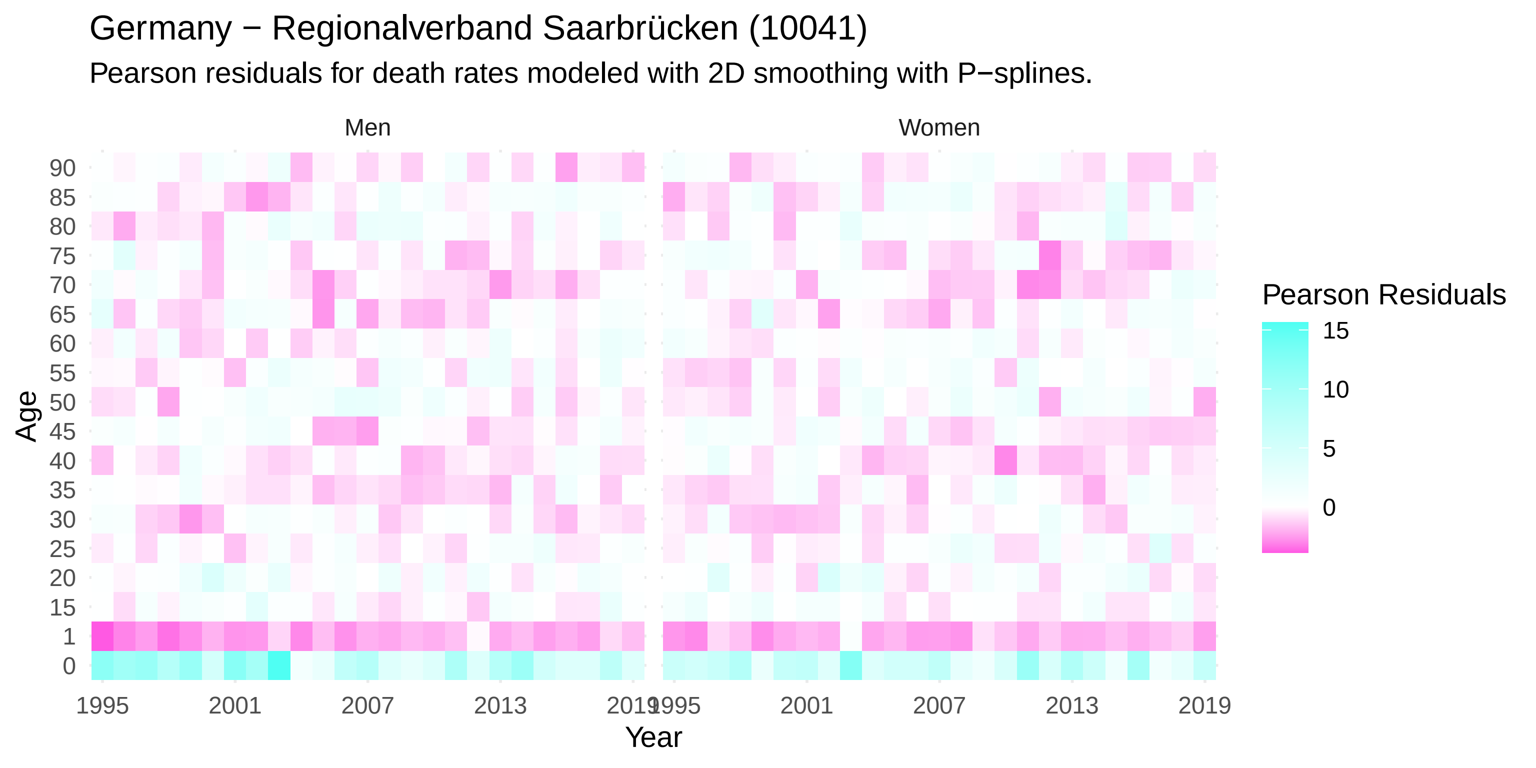

Germany − Regionalverband Saarbrücken (10041)
Pearson residuals for death rates modeled with 2D smoothing with P−splines.
Men
Women
Age
90
85
80
75
70
65
60
55
50
45
40
35
30
25
20
15
1
0
1995
2001
2007
2013
2019
Year
Pearson Residuals
15
10
5
0

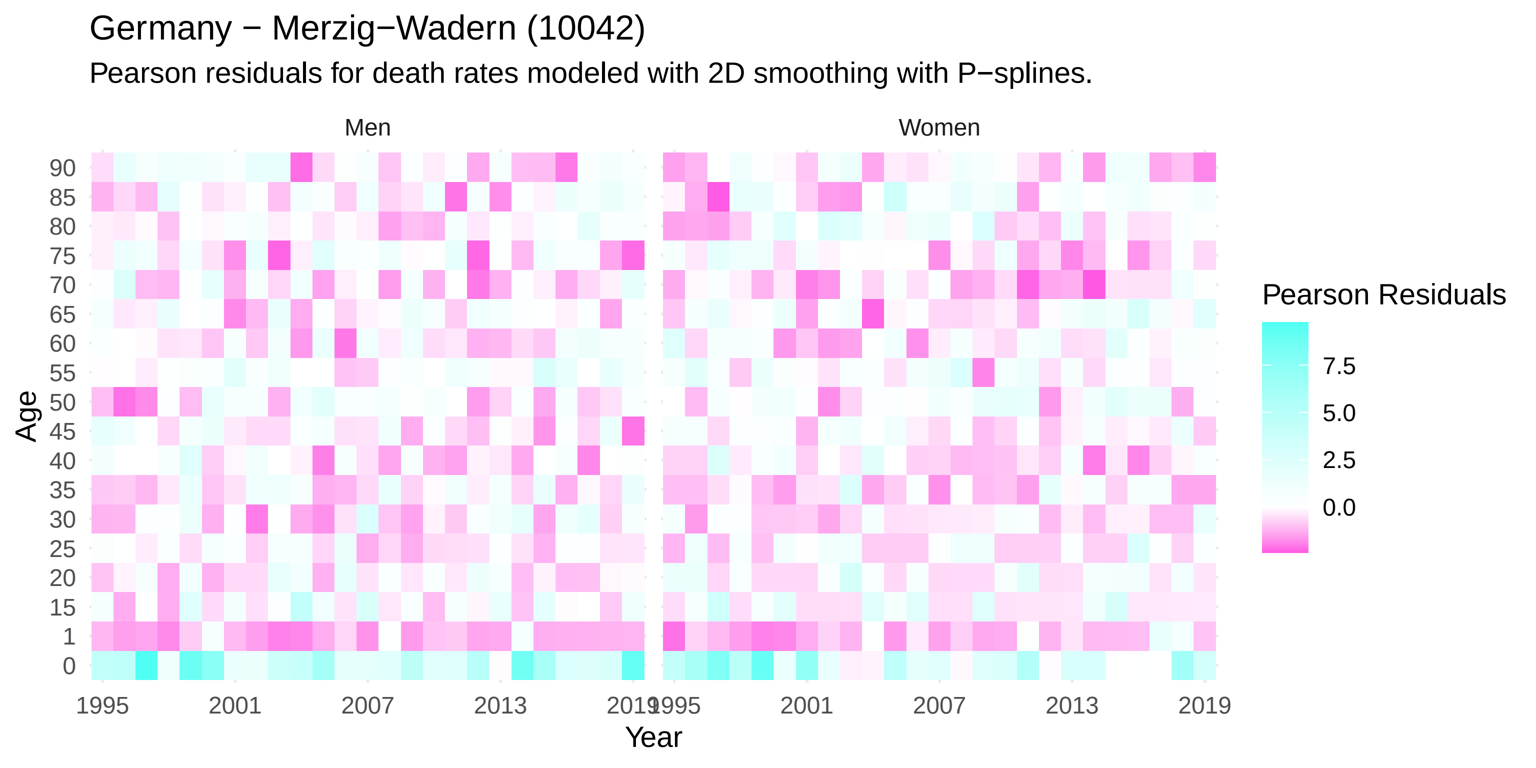

Germany − Merzig−Wadern (10042)
Pearson residuals for death rates modeled with 2D smoothing with P−splines.
Men
Women
Age
90
85
80
75
70
65
60
55
50
45
40
35
30
25
20
15
1
0
1995
2001
2007
2013
2019
Year
Pearson Residuals
7.5
5.0
2.5
0.0

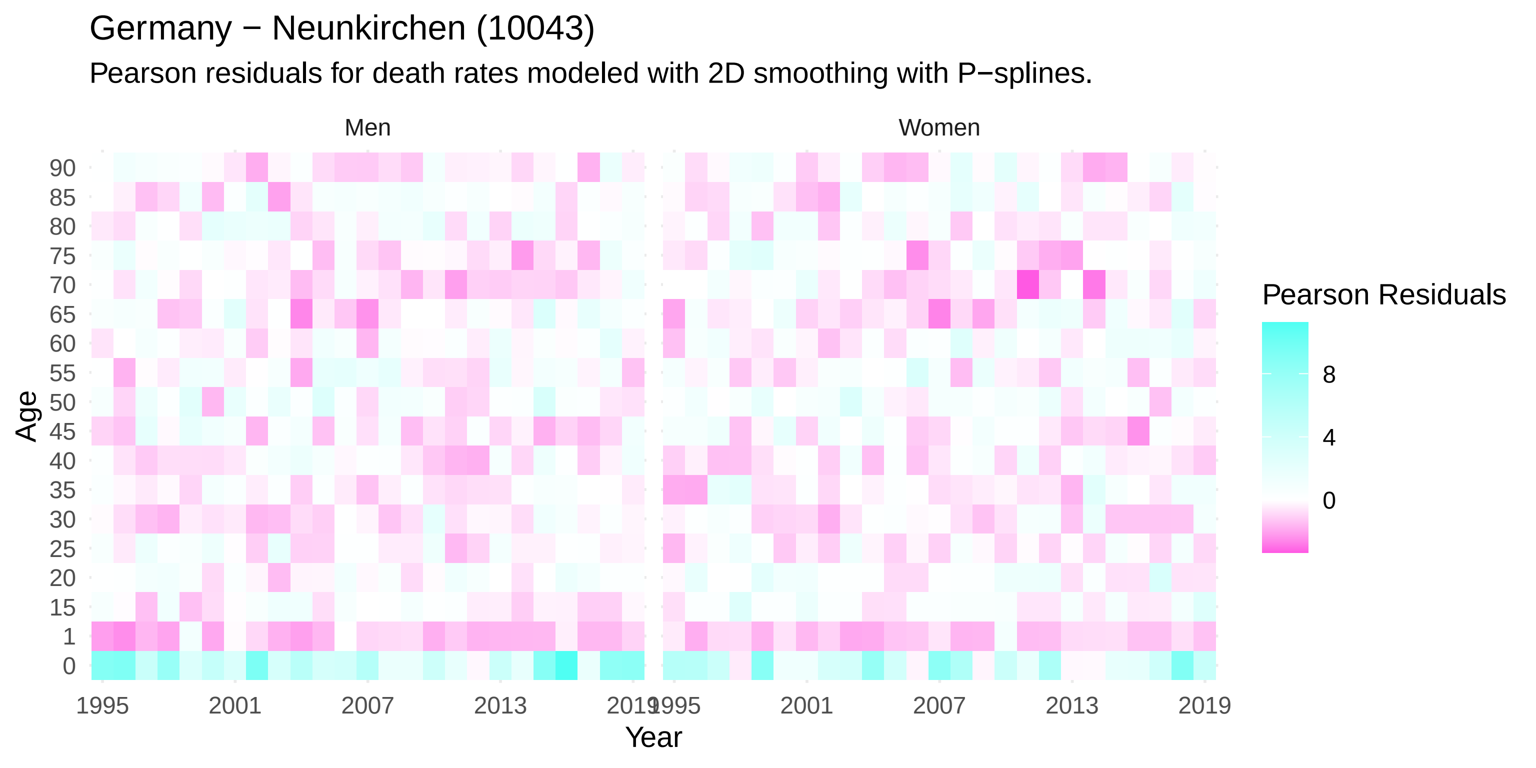

Germany − Neunkirchen (10043)
Pearson residuals for death rates modeled with 2D smoothing with P−splines.
Men
Women
Age
90
85
80
75
70
65
60
55
50
45
40
35
30
25
20
15
1
0
1995
2001
2007
2013
2019
Year
Pearson Residuals
8
4
0

Germany − Saarlouis (10044)

Pearson residuals for death rates modeled with 2D smoothing with P−splines.

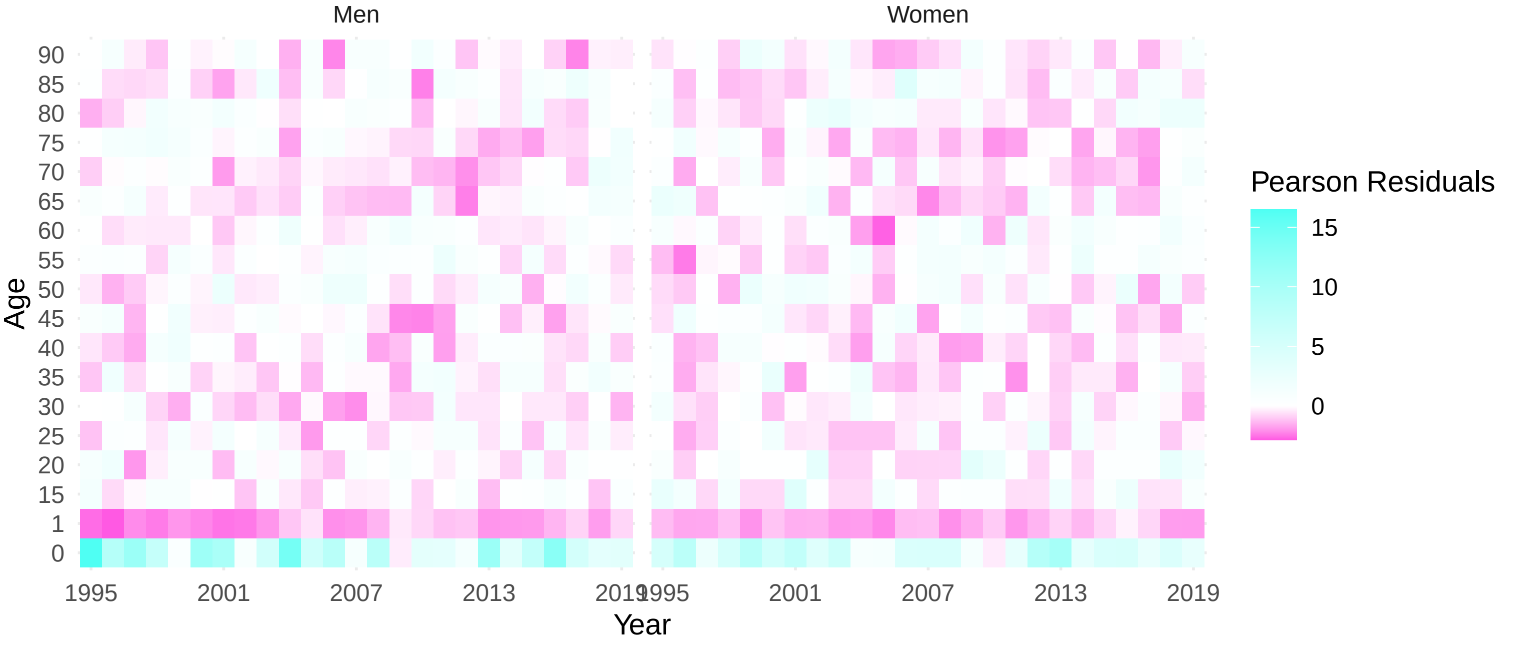

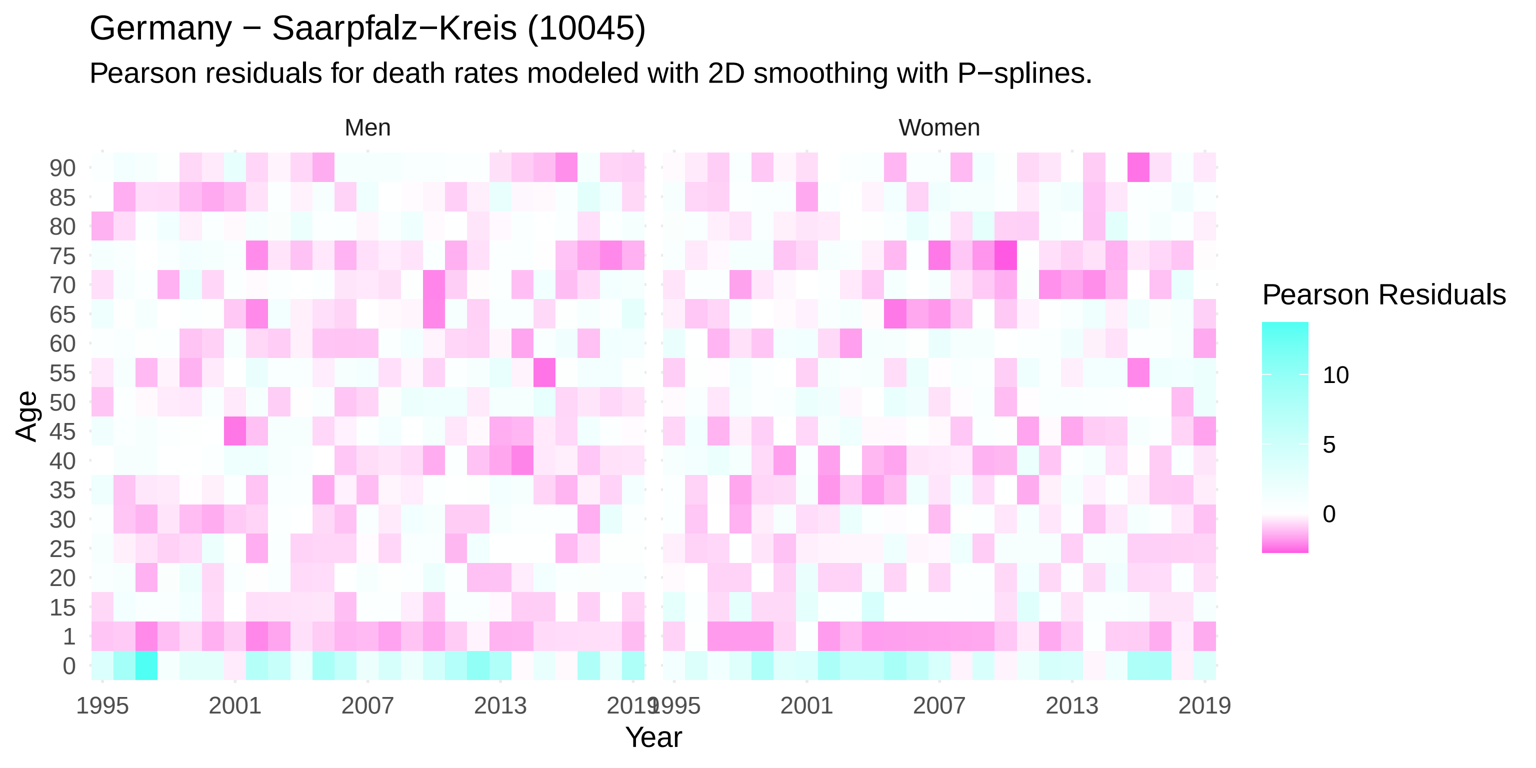

Germany − Saarpfalz−Kreis (10045)
Pearson residuals for death rates modeled with 2D smoothing with P−splines.
Men
Women
Age
90
85
80
75
70
65
60
55
50
45
40
35
30
25
20
15
1
0
1995
2001
2007
2013
2019
Year
Pearson Residuals
10
5
0

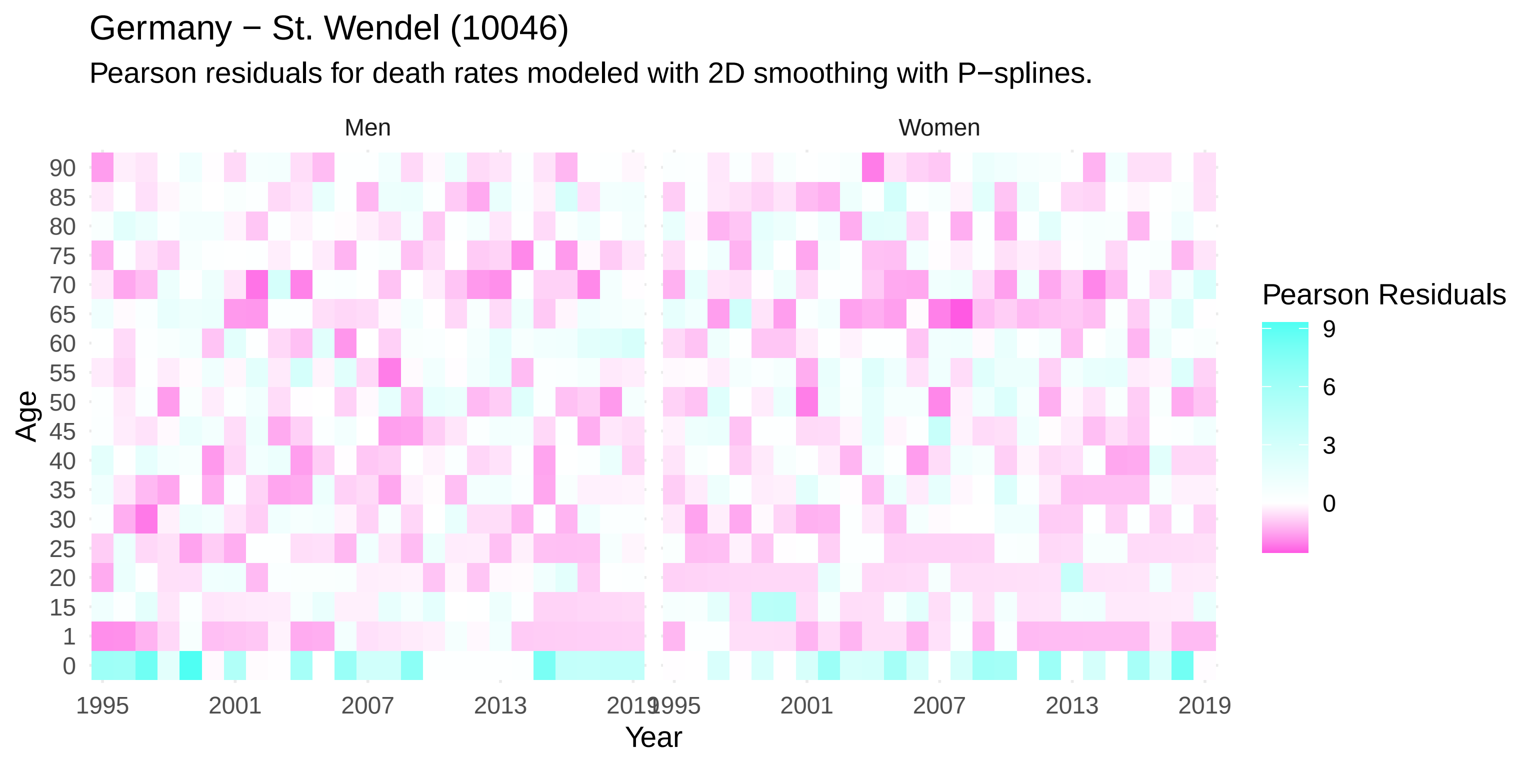

Germany − St. Wendel (10046)
Pearson residuals for death rates modeled with 2D smoothing with P−splines.
Men
Women
Age
90
85
80
75
70
65
60
55
50
45
40
35
30
25
20
15
1
0
1995
2001
2007
2013
2019
Year
Pearson Residuals
9
6
3
0

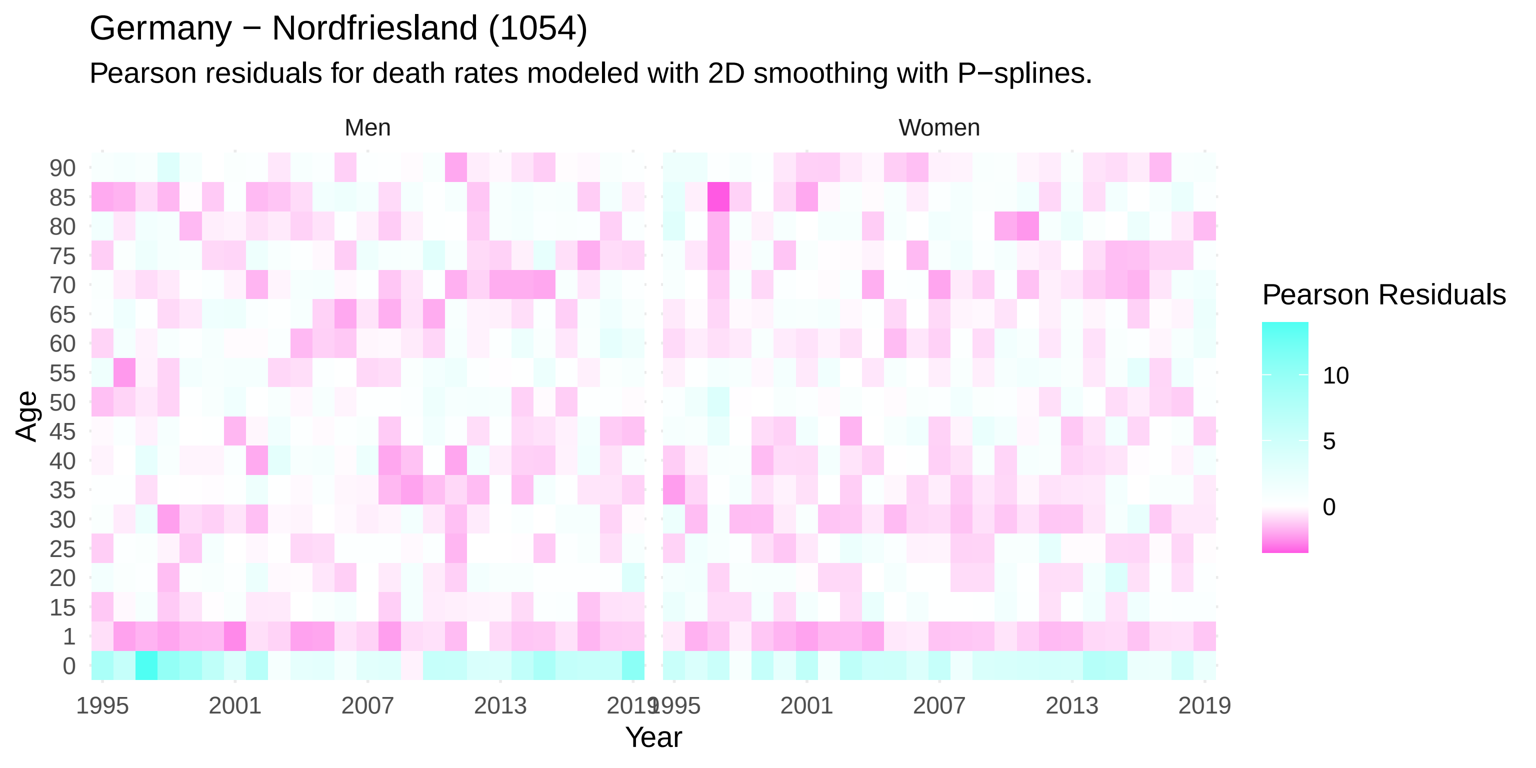

Germany − Nordfriesland (1054)
Pearson residuals for death rates modeled with 2D smoothing with P−splines.
Men
Women
Age
90
85
80
75
70
65
60
55
50
45
40
35
30
25
20
15
1
0
1995
2001
2007
2013
2019
Year
Pearson Residuals
10
5
0

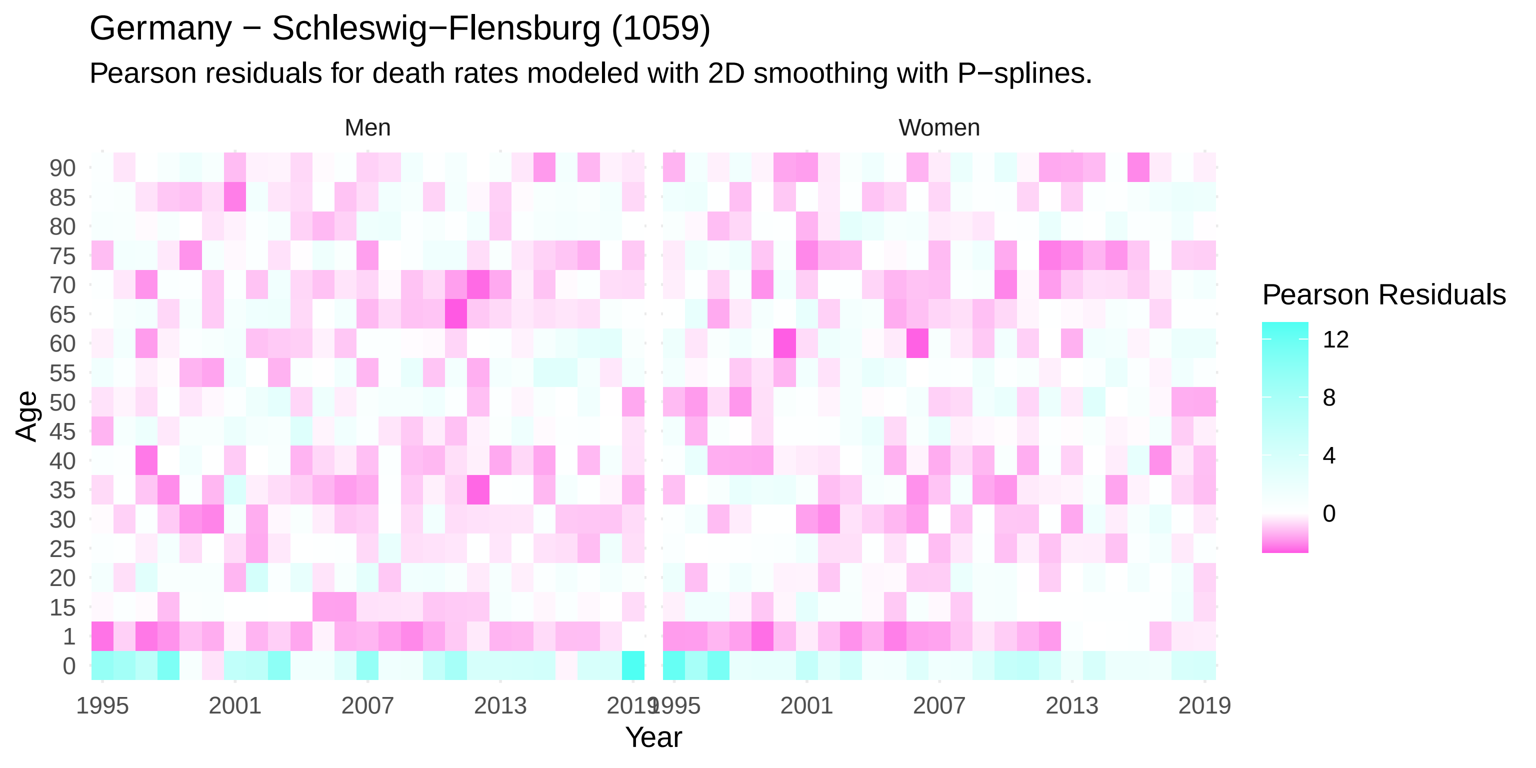

Germany − Schleswig−Flensburg (1059)
Pearson residuals for death rates modeled with 2D smoothing with P−splines.
Men
Women
Age
90
85
80
75
70
65
60
55
50
45
40
35
30
25
20
15
1
0
1995
2001
2007
2013
2019
Year
Pearson Residuals
12
8
4
0

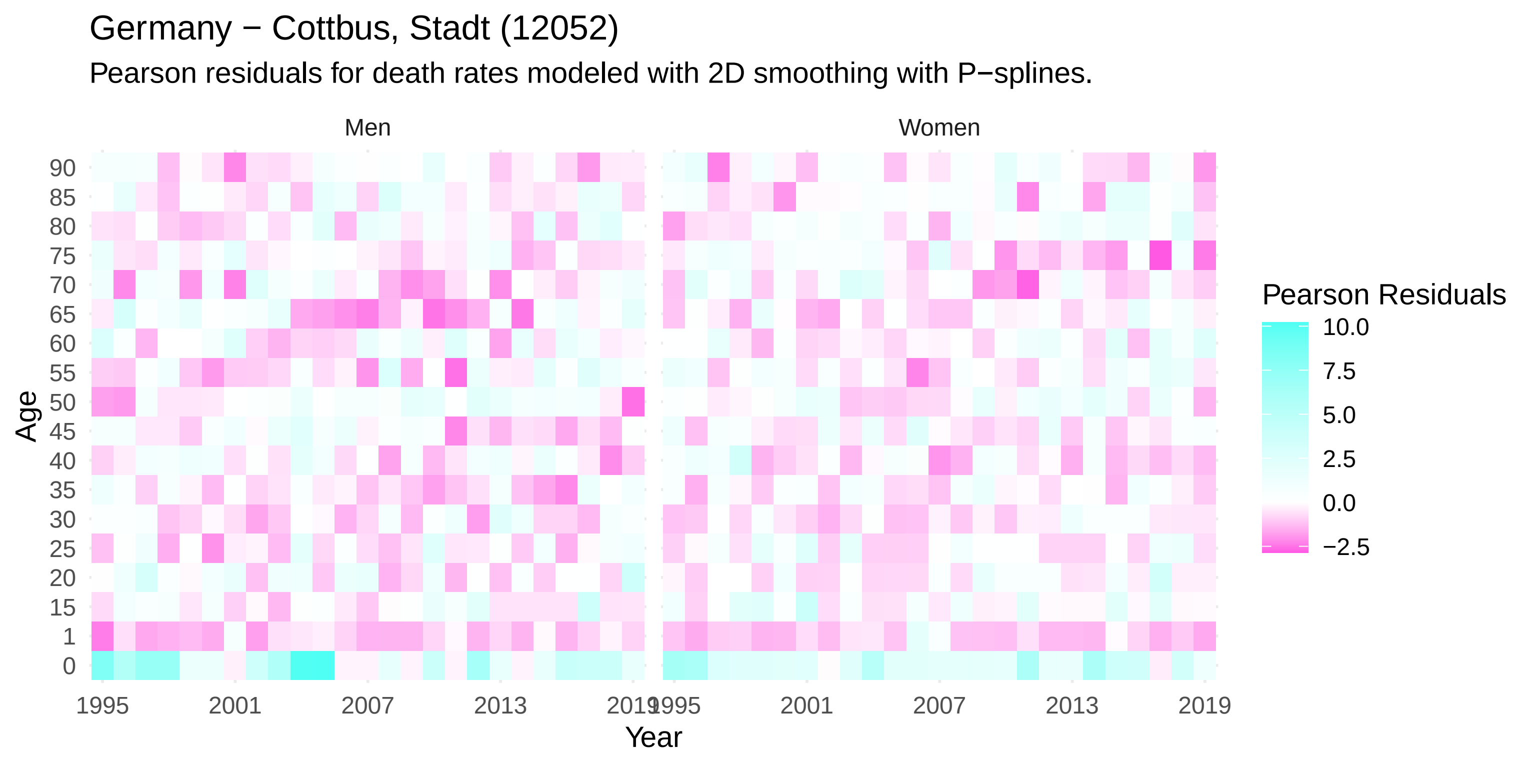
Germany − Cottbus, Stadt (12052)
Pearson residuals for death rates modeled with 2D smoothing with P−splines.
Men
Women
Age
90
85
80
75
70
65
60
55
50
45
40
35
30
25
20
15
1
0
1995
2001
2007
2013
2019
Year
Pearson Residuals
10.0
7.5
5.0
2.5
0.0
−2.5

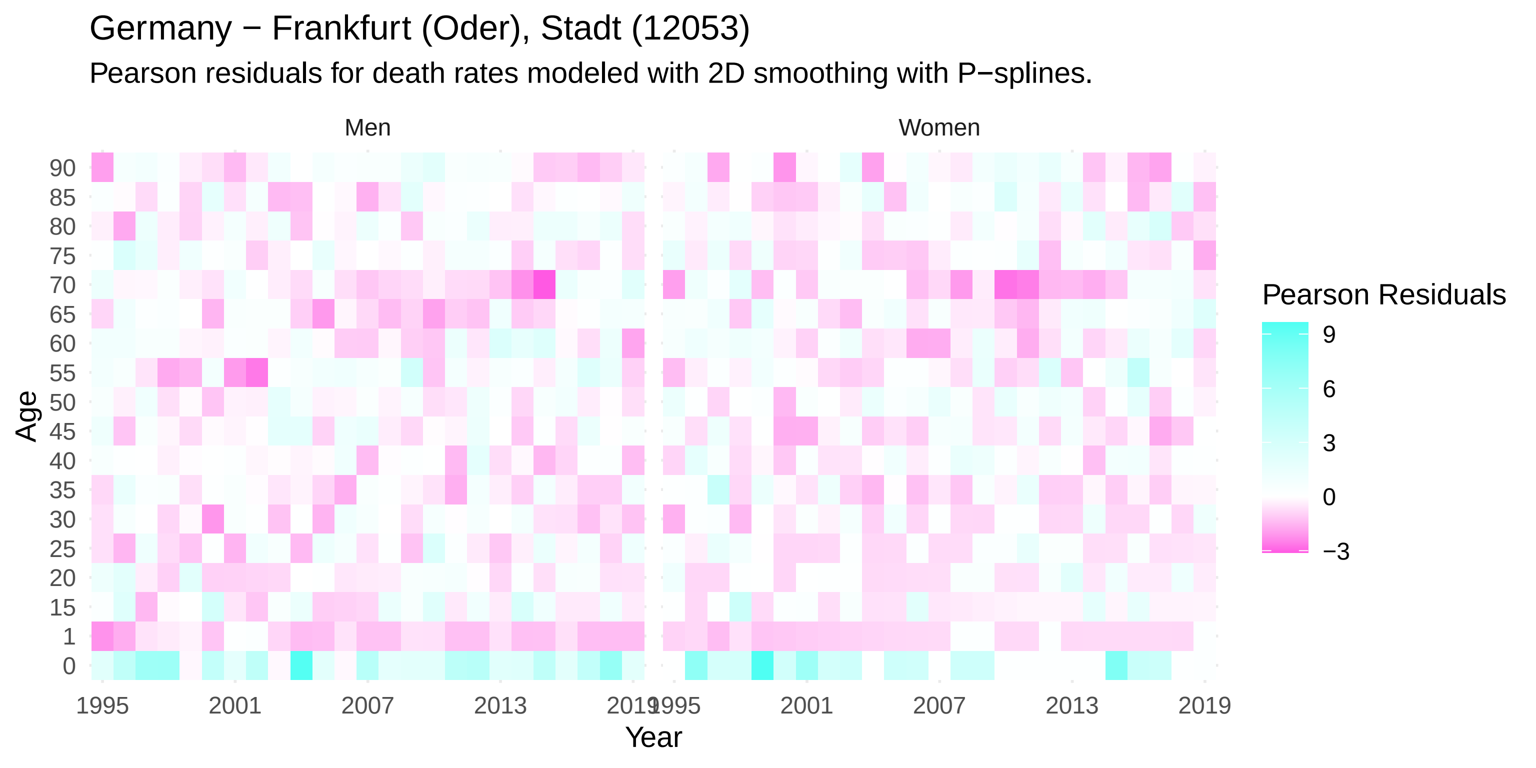
Germany − Frankfurt (Oder), Stadt (12053)
Pearson residuals for death rates modeled with 2D smoothing with P−splines.
Men
Women
Age
90
85
80
75
70
65
60
55
50
45
40
35
30
25
20
15
1
0
1995
2001
2007
2013
2019
Year
Pearson Residuals
9
6
3
0
−3

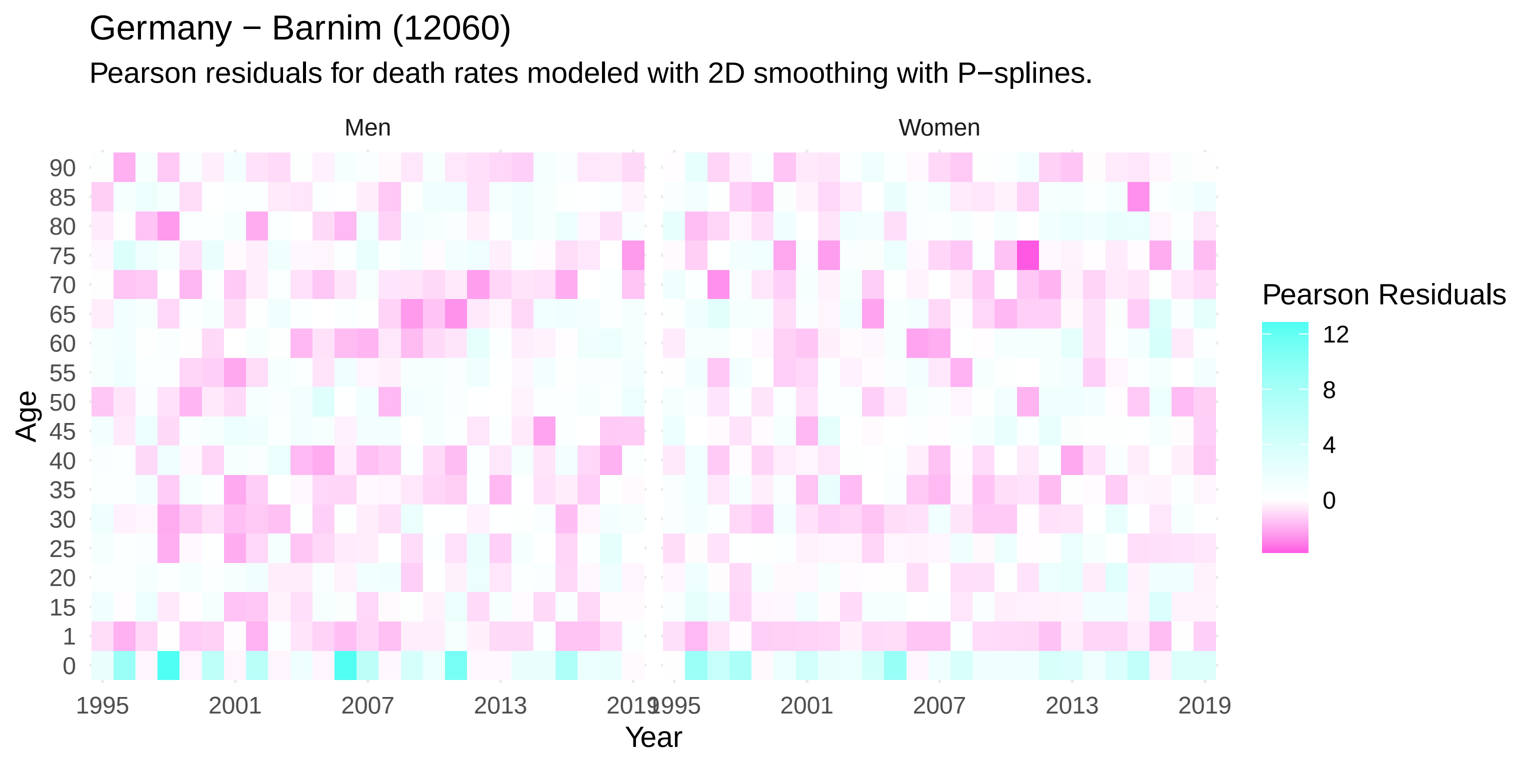

Germany − Barnim (12060)
Pearson residuals for death rates modeled with 2D smoothing with P−splines.
Men
Women
Age
90
85
80
75
70
65
60
55
50
45
40
35
30
25
20
15
1
0
1995
2001
2007
2013
2019
Year
Pearson Residuals
12
8
4
0

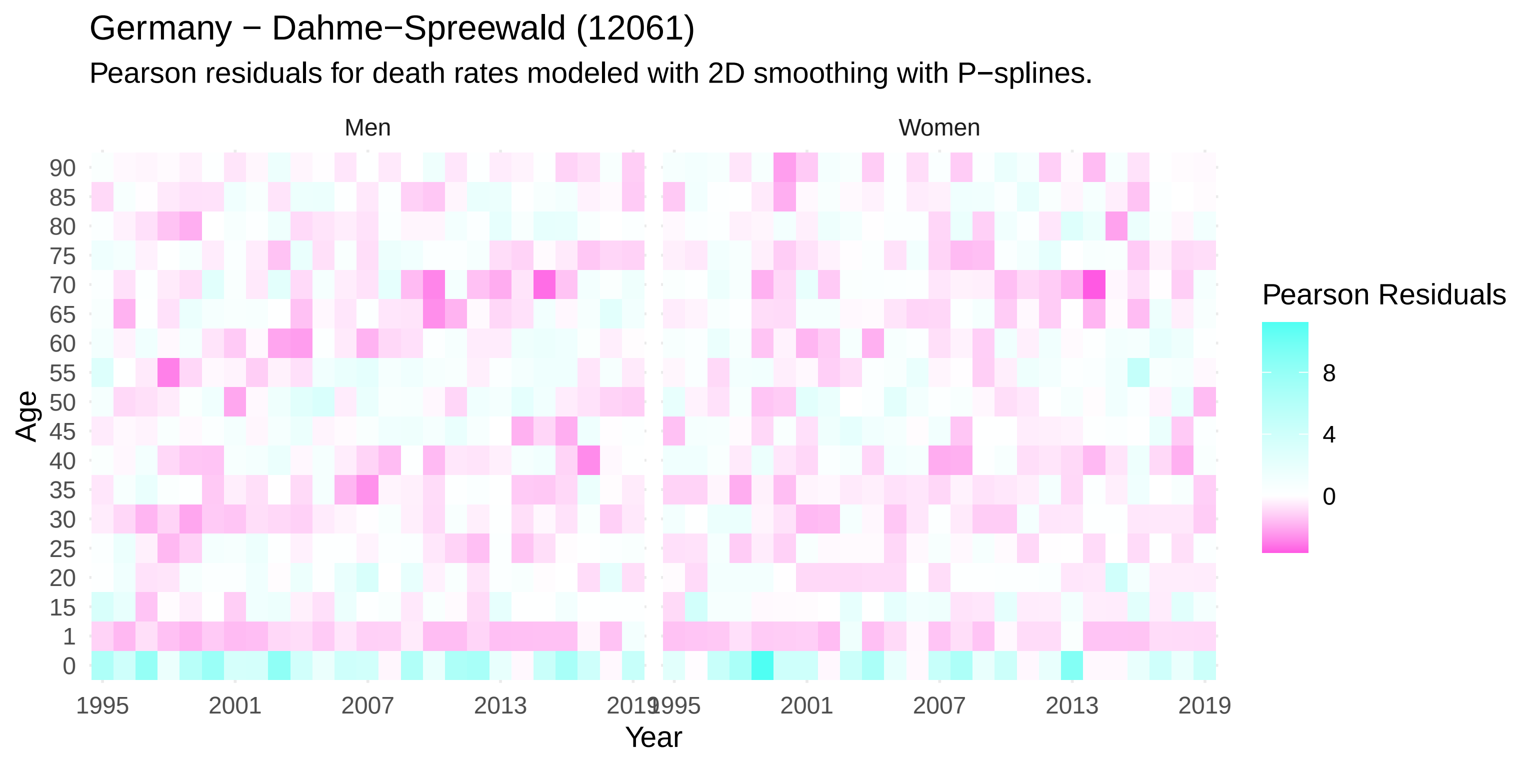

Germany − Dahme−Spreewald (12061)
Pearson residuals for death rates modeled with 2D smoothing with P−splines.
Men
Women
Age
90
85
80
75
70
65
60
55
50
45
40
35
30
25
20
15
1
0
1995
2001
2007
2013
2019
Year
Pearson Residuals
8
4
0

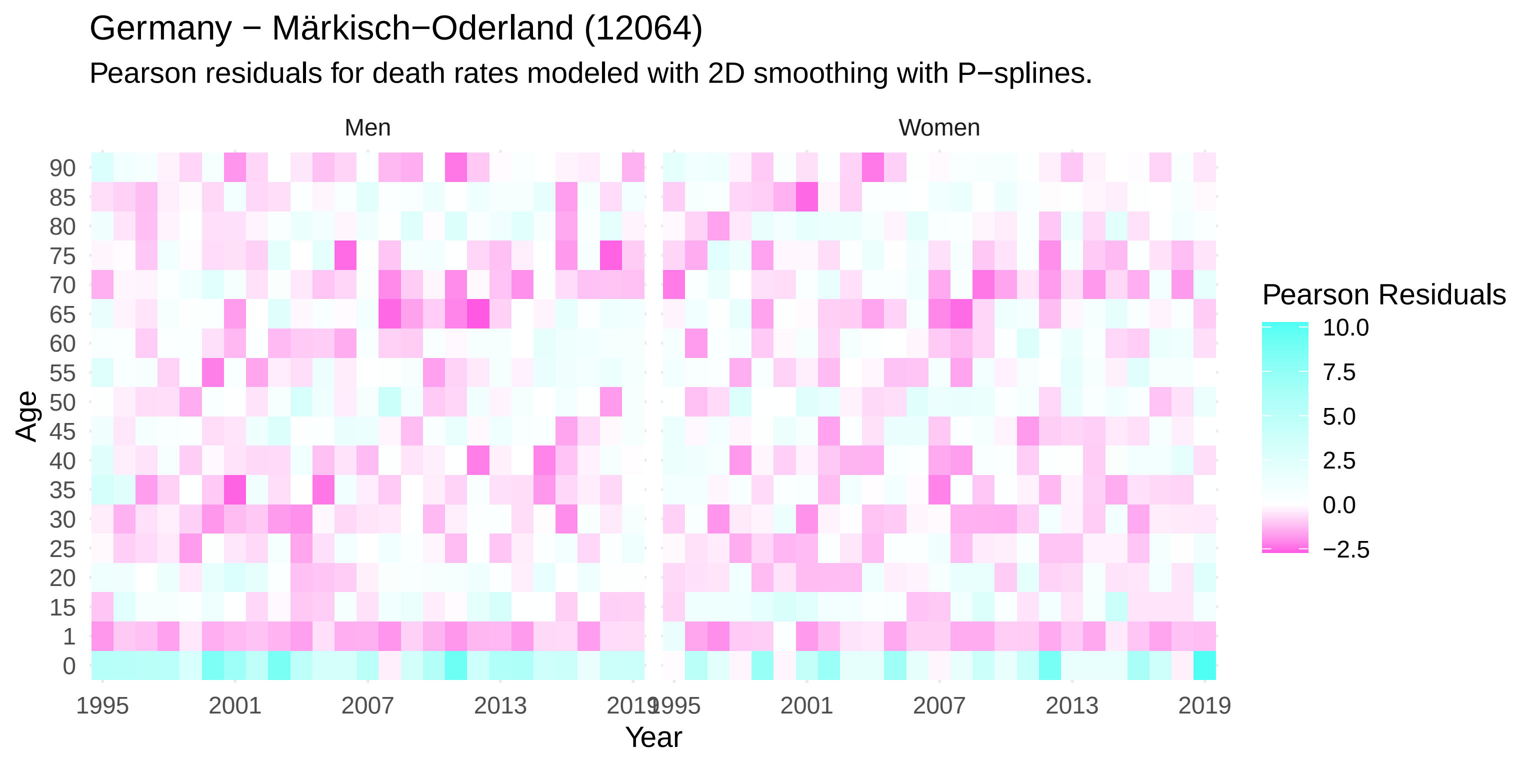

Germany − Märkisch−Oderland (12064)
Pearson residuals for death rates modeled with 2D smoothing with P−splines.
Men
Women
Age
90
85
80
75
70
65
60
55
50
45
40
35
30
25
20
15
1
0
1995
2001
2007
2013
2019
Year
Pearson Residuals
10.0
7.5
5.0
2.5
0.0
−2.5

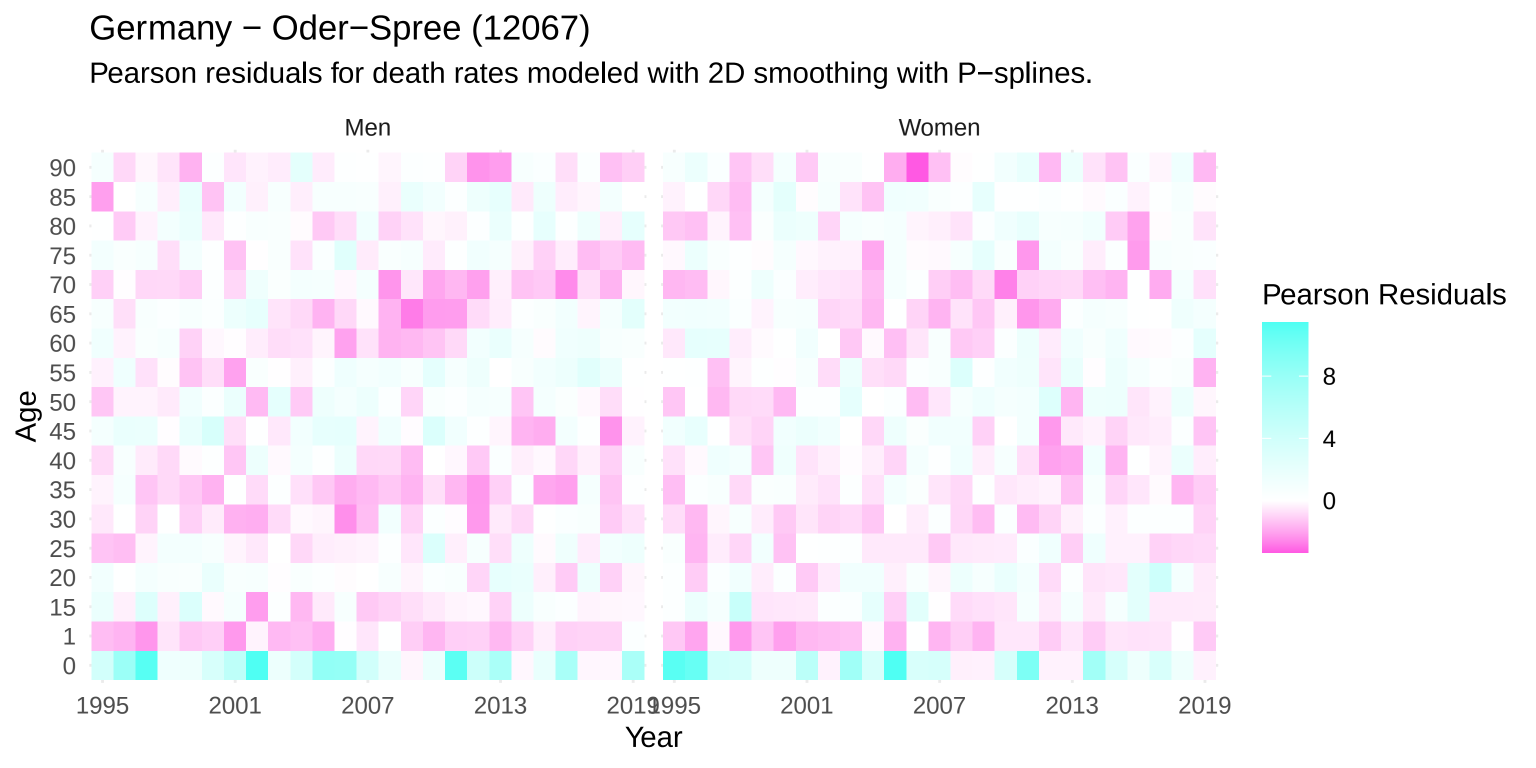

Germany − Oder−Spree (12067)
Pearson residuals for death rates modeled with 2D smoothing with P−splines.
Men
Women
Age
90
85
80
75
70
65
60
55
50
45
40
35
30
25
20
15
1
0
1995
2001
2007
2013
2019
Year
Pearson Residuals
8
4
0

Germany − Spree−Neiße (12071)

Pearson residuals for death rates modeled with 2D smoothing with P−splines.

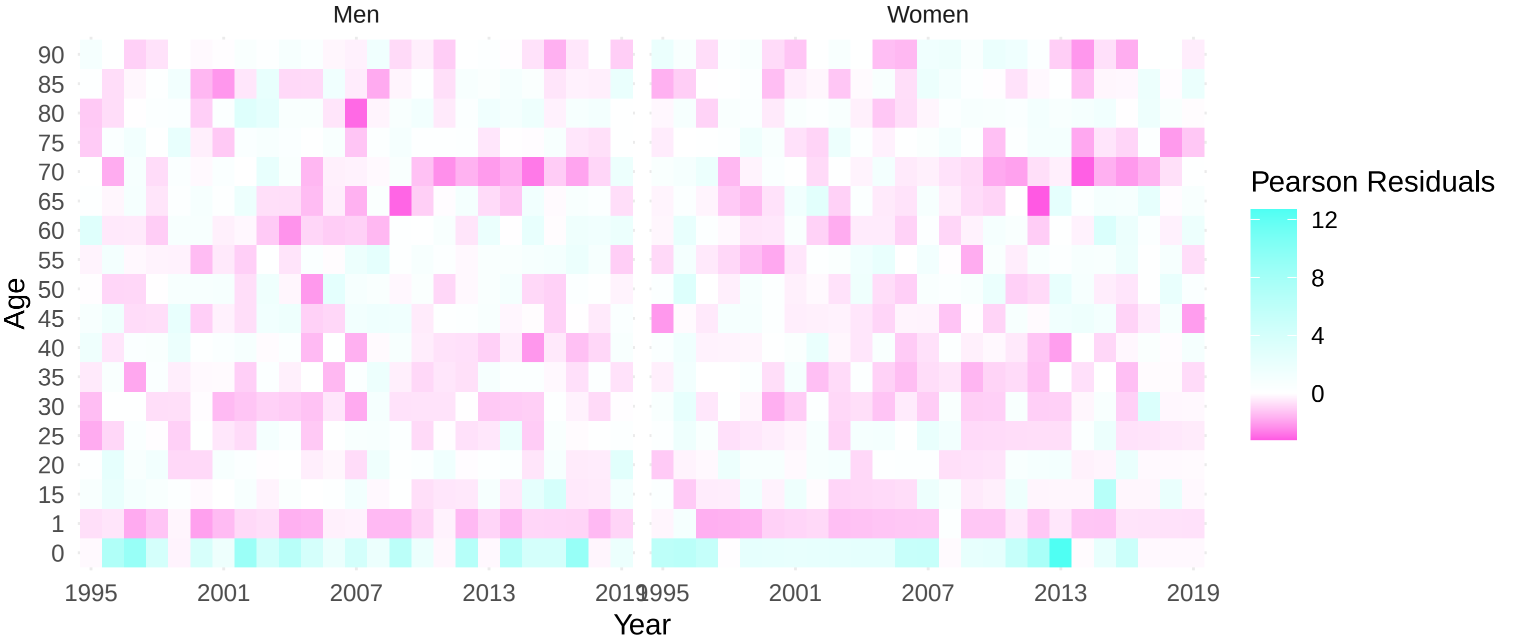

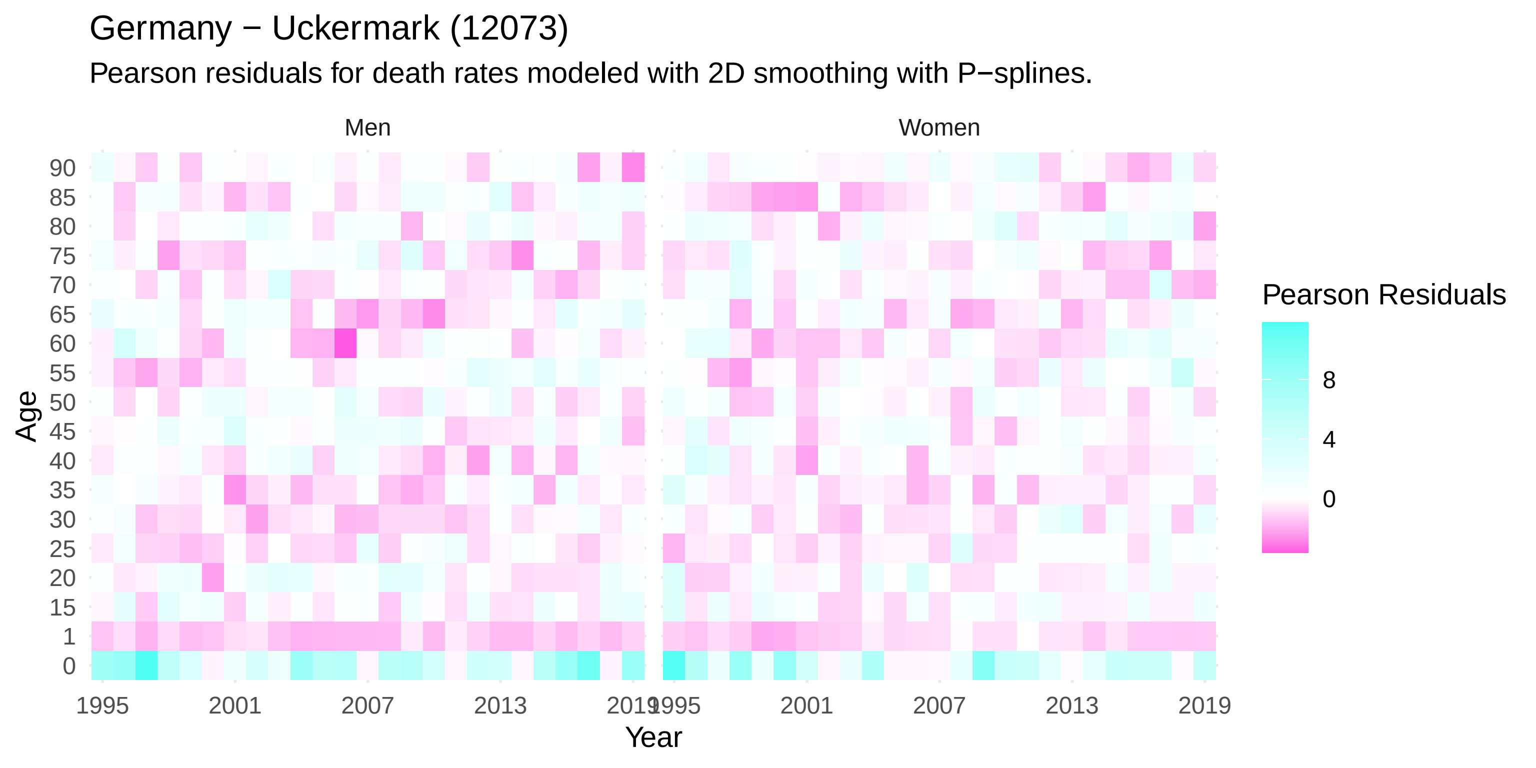

Germany − Uckermark (12073)
Pearson residuals for death rates modeled with 2D smoothing with P−splines.
Men
Women
Age
90
85
80
75
70
65
60
55
50
45
40
35
30
25
20
15
1
0
1995
2001
2007
2013
2019
Year
Pearson Residuals
8
4
0

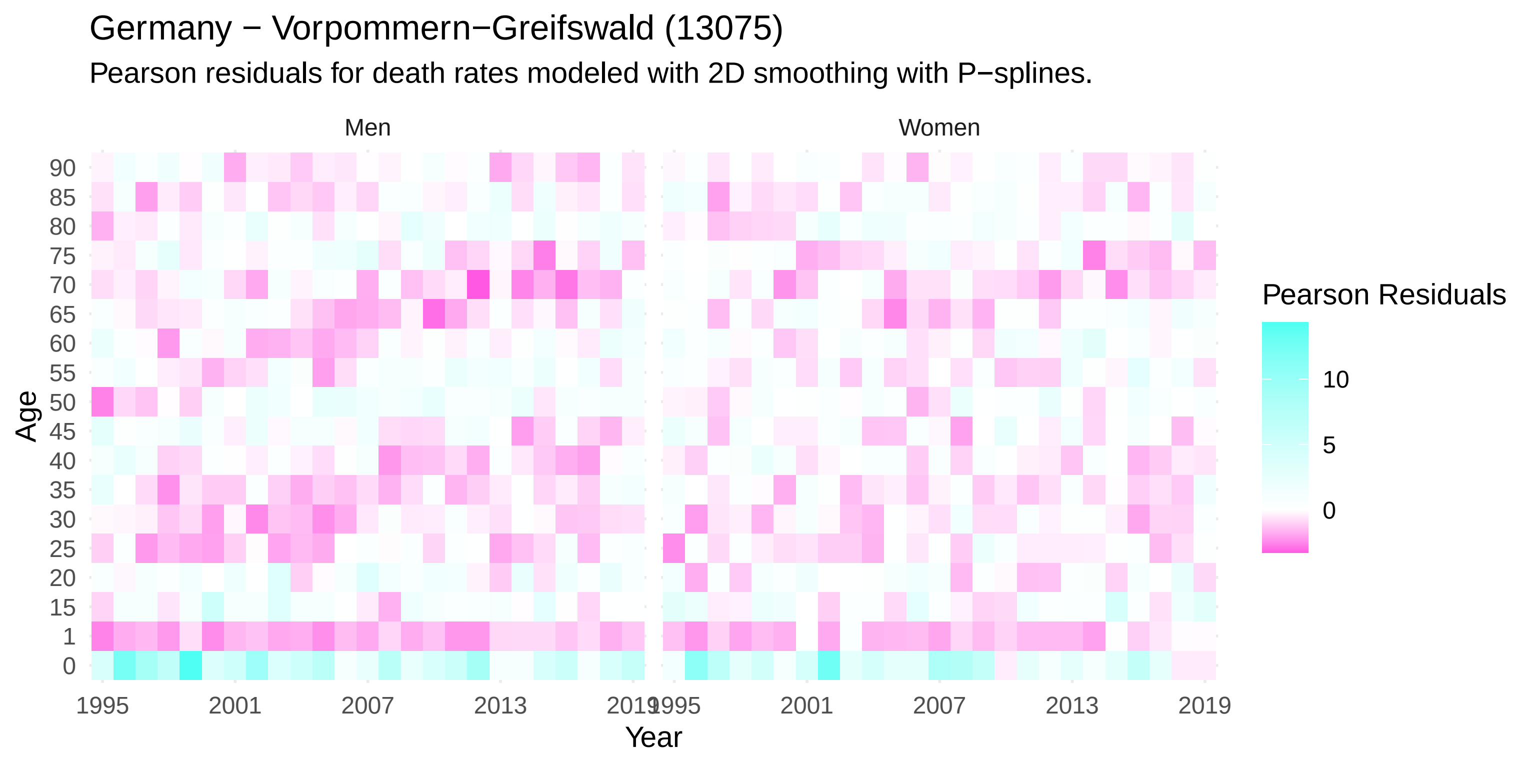
Germany − Vorpommern−Greifswald (13075)
Pearson residuals for death rates modeled with 2D smoothing with P−splines.
Men
Women
Age
90
85
80
75
70
65
60
55
50
45
40
35
30
25
20
15
1
0
1995
2001
2007
2013
2019
Year
Pearson Residuals
10
5
0

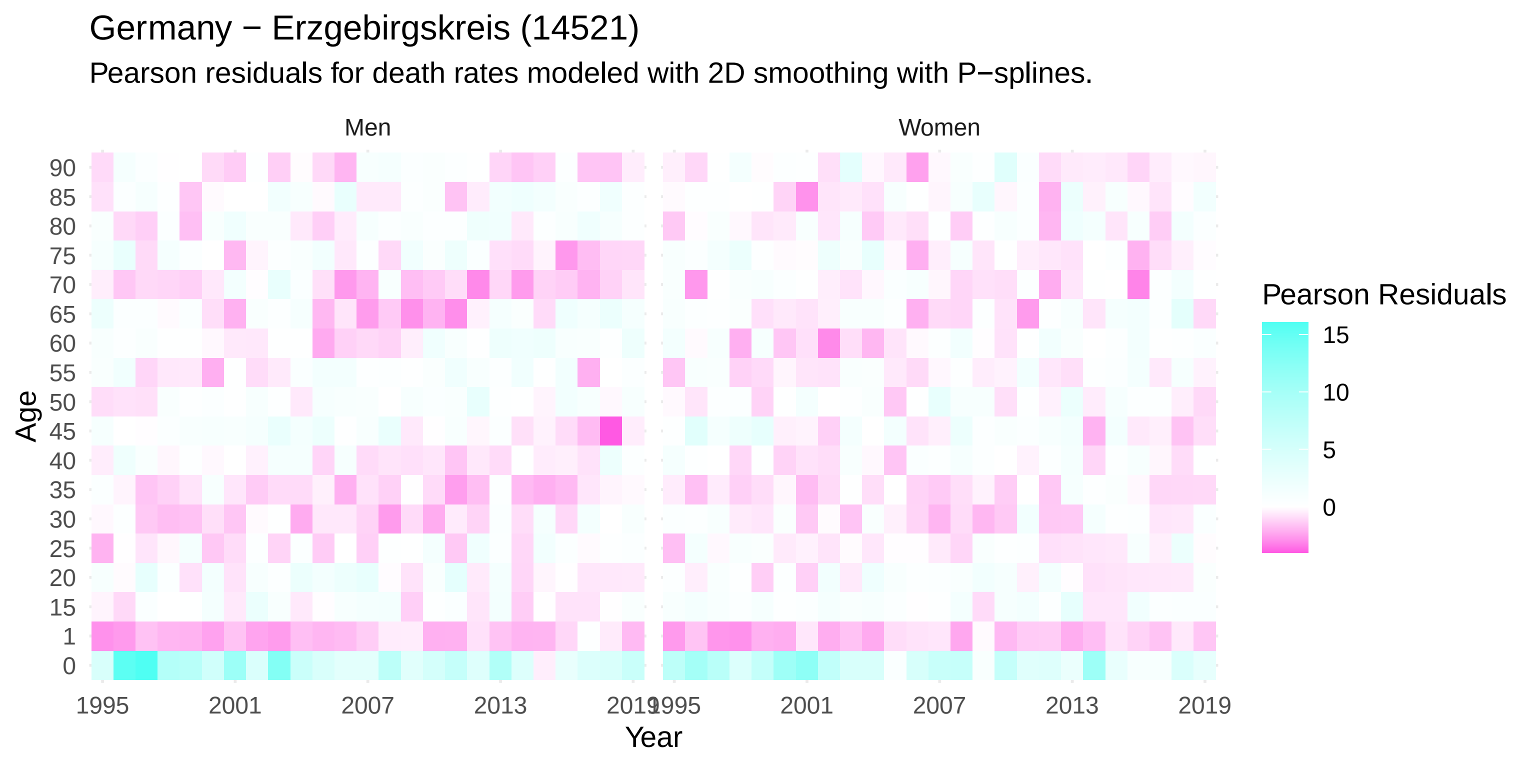

Germany − Erzgebirgskreis (14521)
Pearson residuals for death rates modeled with 2D smoothing with P−splines.
Men
Women
Age
90
85
80
75
70
65
60
55
50
45
40
35
30
25
20
15
1
0
1995
2001
2007
2013
2019
Year
Pearson Residuals
15
10
5
0

Germany − Mittelsachsen (14522)

Pearson residuals for death rates modeled with 2D smoothing with P−splines.

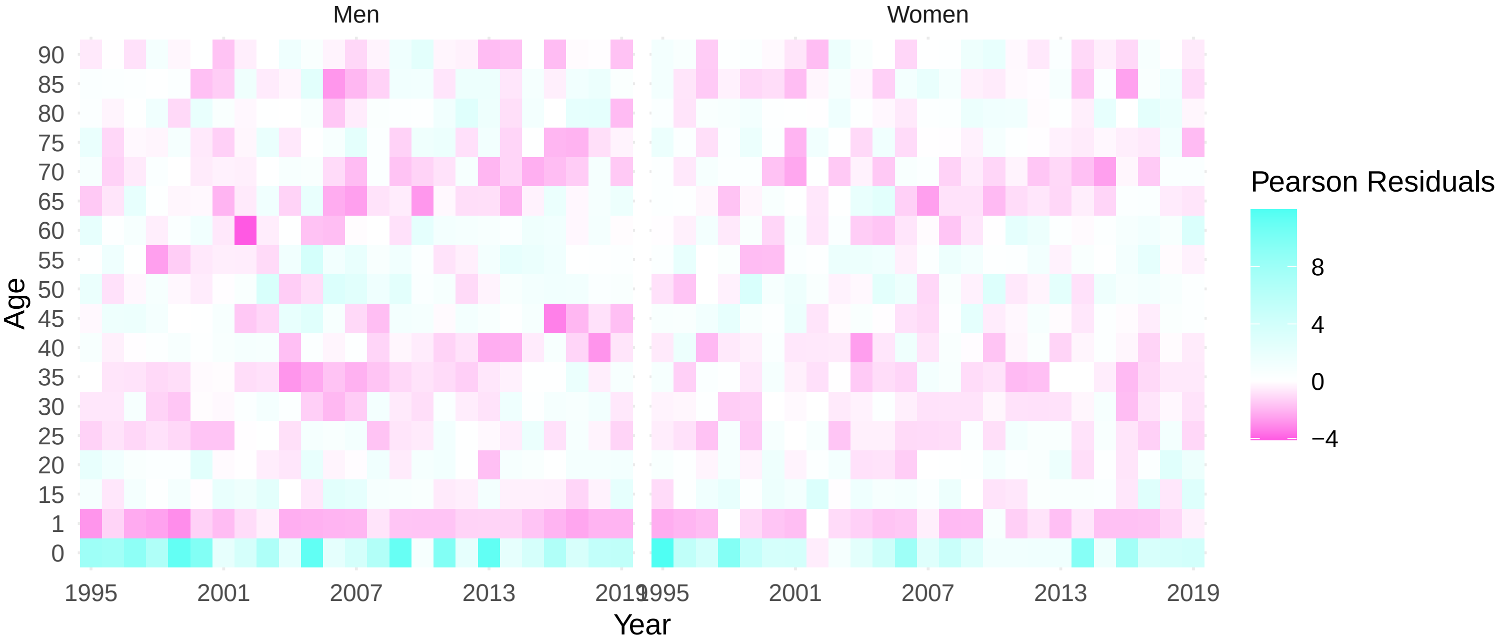

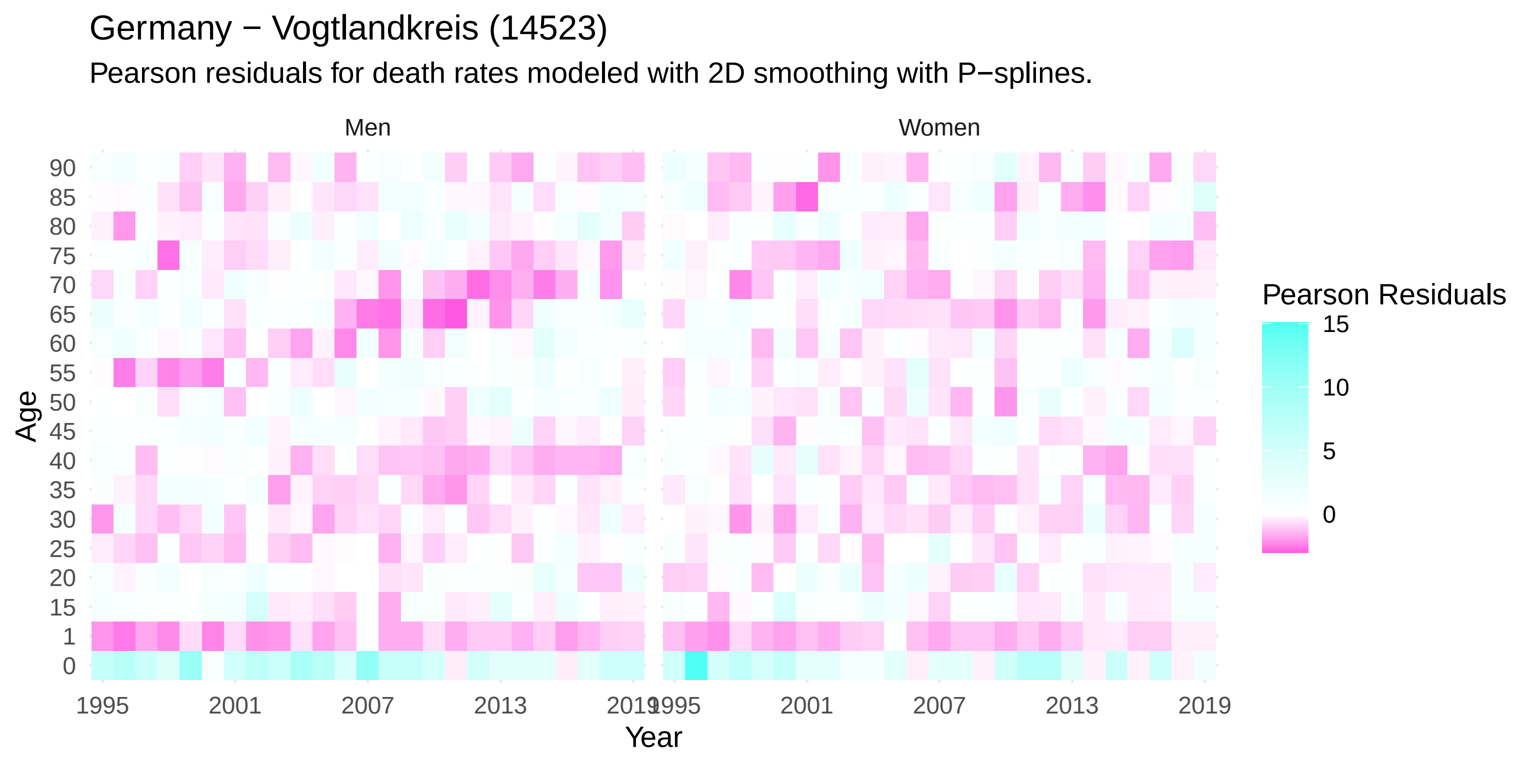

Germany − Vogtlandkreis (14523)
Pearson residuals for death rates modeled with 2D smoothing with P−splines.
Men
Women
Age
90
85
80
75
70
65
60
55
50
45
40
35
30
25
20
15
1
0
1995
2001
2007
2013
2019
Year
Pearson Residuals
15
10
5
0

Germany − Zwickau (14524)

Pearson residuals for death rates modeled with 2D smoothing with P−splines.

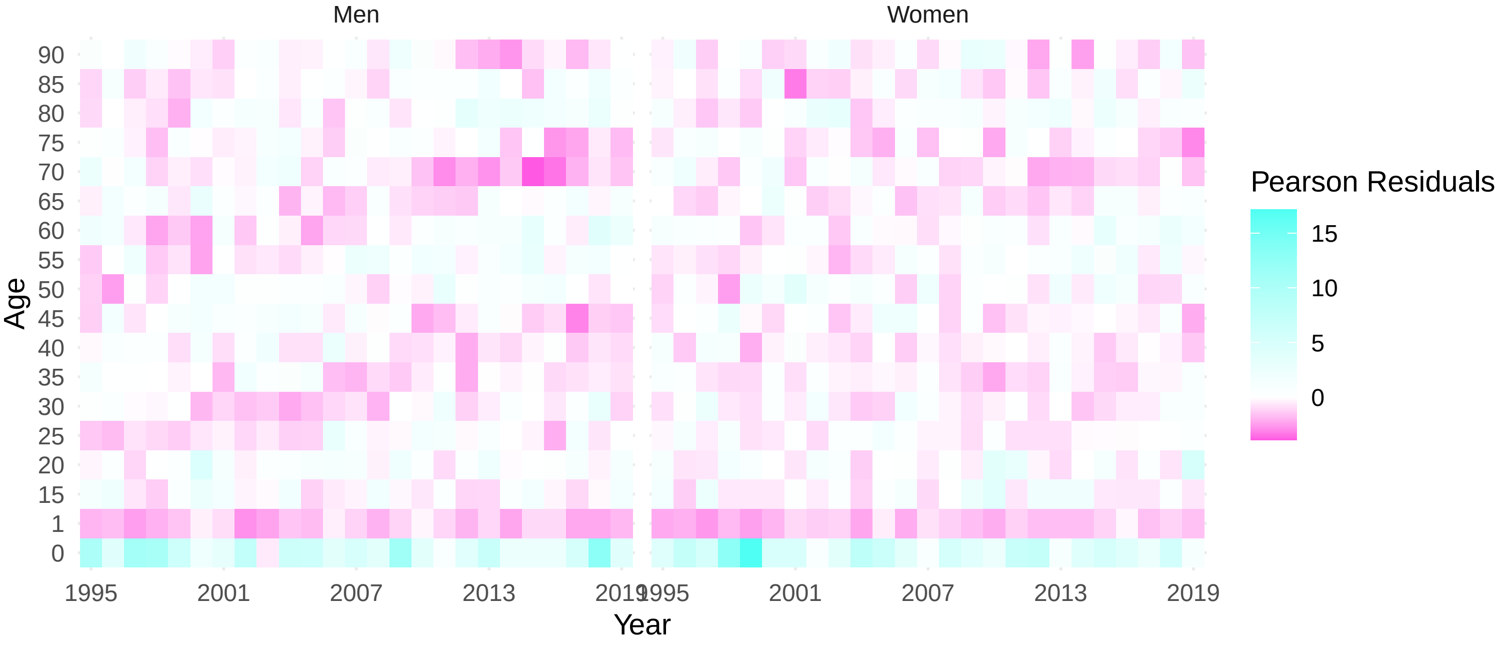

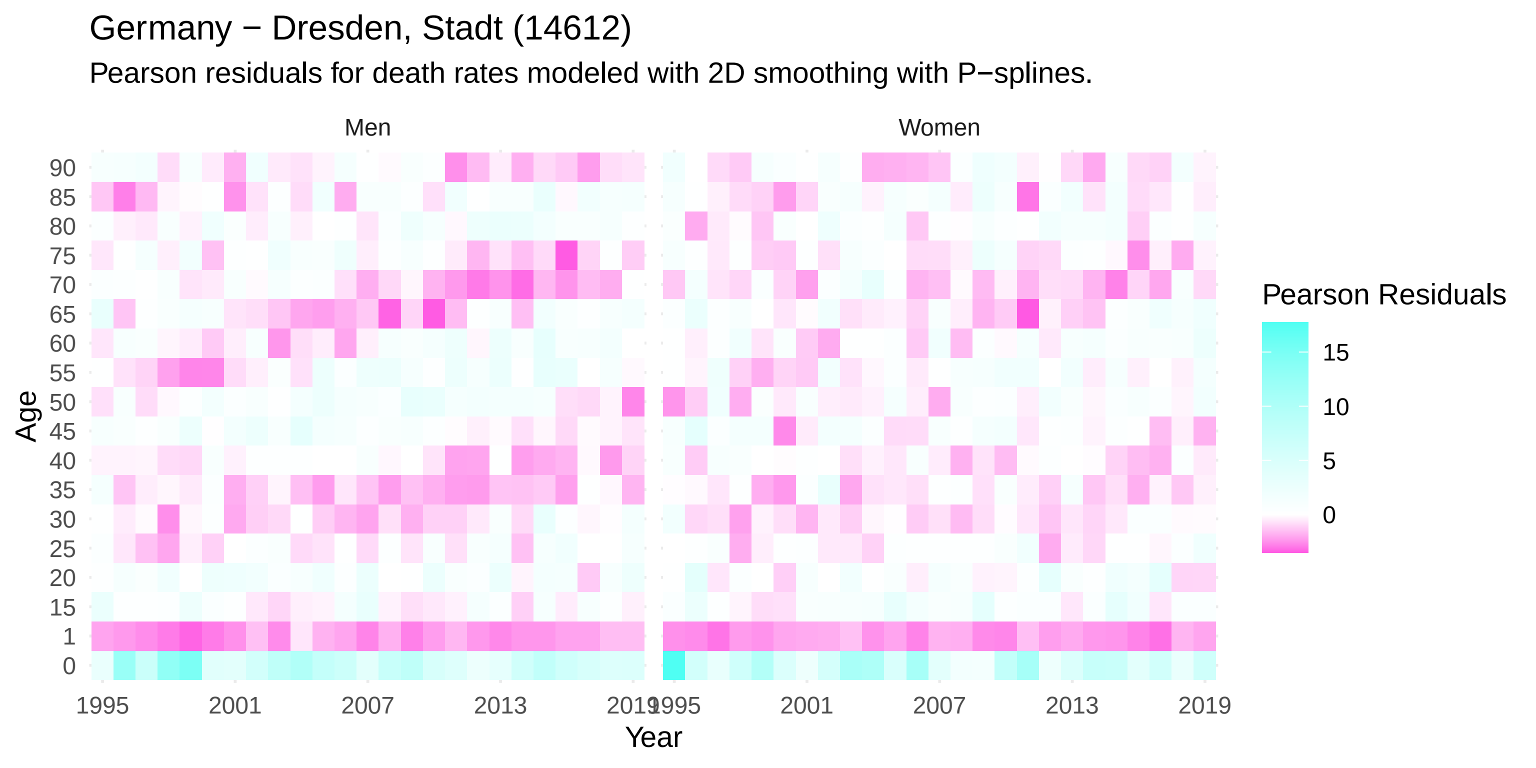

Germany − Dresden, Stadt (14612)
Pearson residuals for death rates modeled with 2D smoothing with P−splines.
Men
Women
Age
90
85
80
75
70
65
60
55
50
45
40
35
30
25
20
15
1
0
1995
2001
2007
2013
2019
Year
Pearson Residuals
15
10
5
0

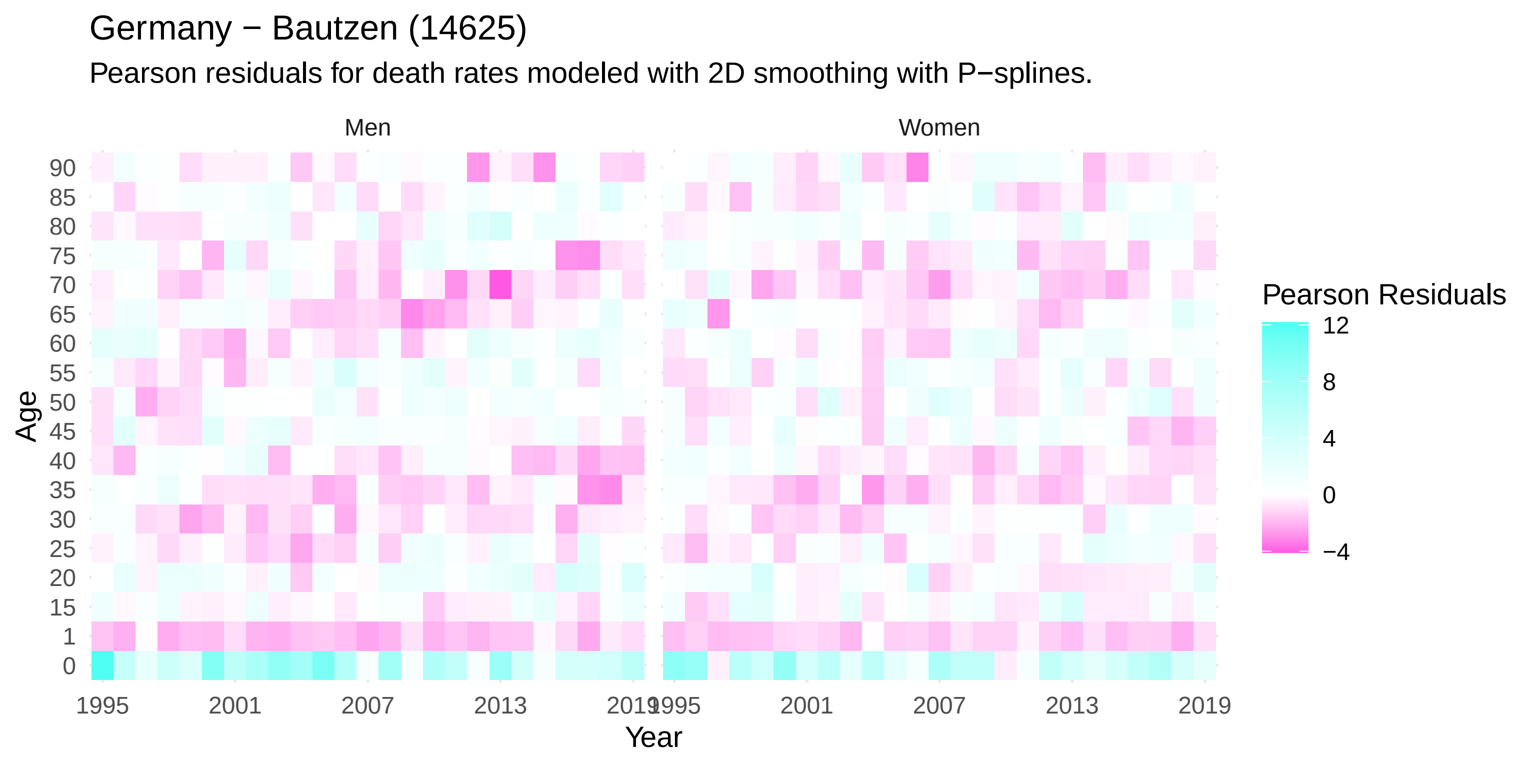

Germany − Bautzen (14625)
Pearson residuals for death rates modeled with 2D smoothing with P−splines.
Men
Women
Age
90
85
80
75
70
65
60
55
50
45
40
35
30
25
20
15
1
0
1995
2001
2007
2013
2019
Year
Pearson Residuals
12
8
4
0
−4

Germany − Görlitz (14626)

Pearson residuals for death rates modeled with 2D smoothing with P−splines.

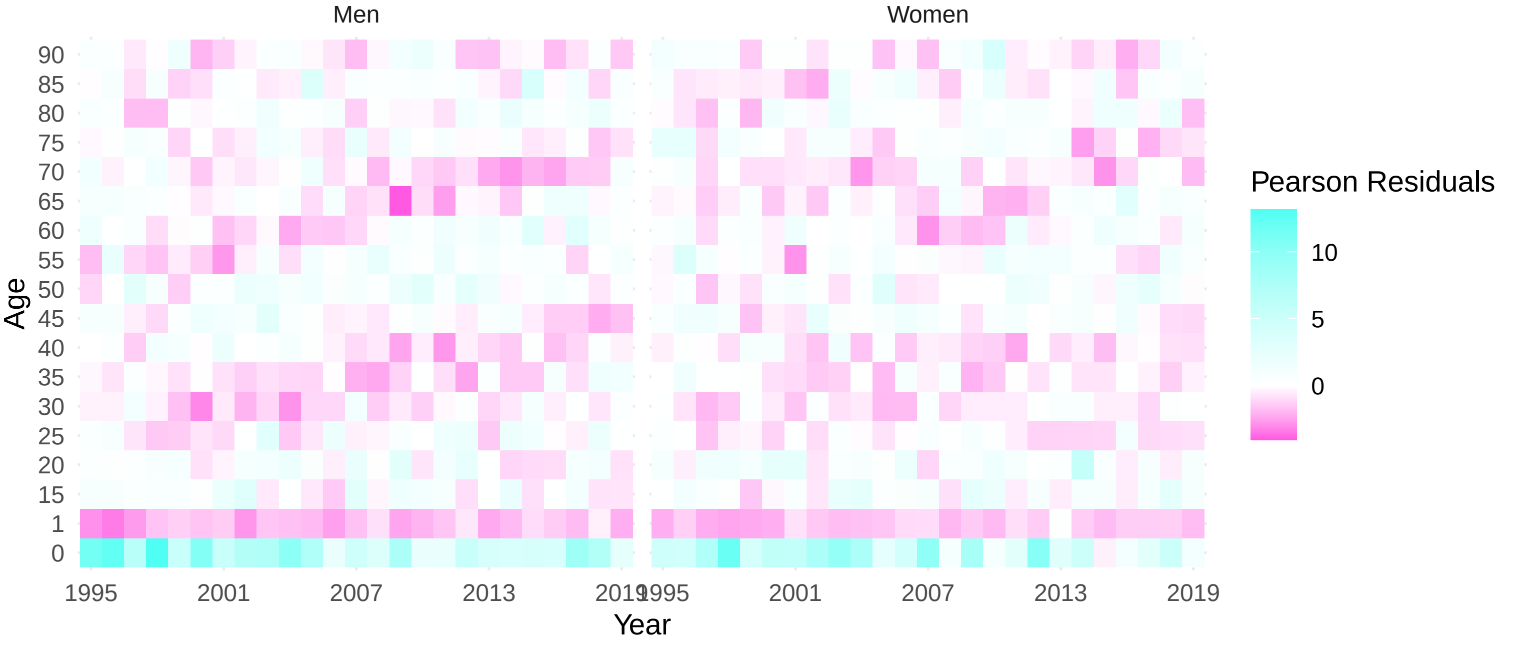

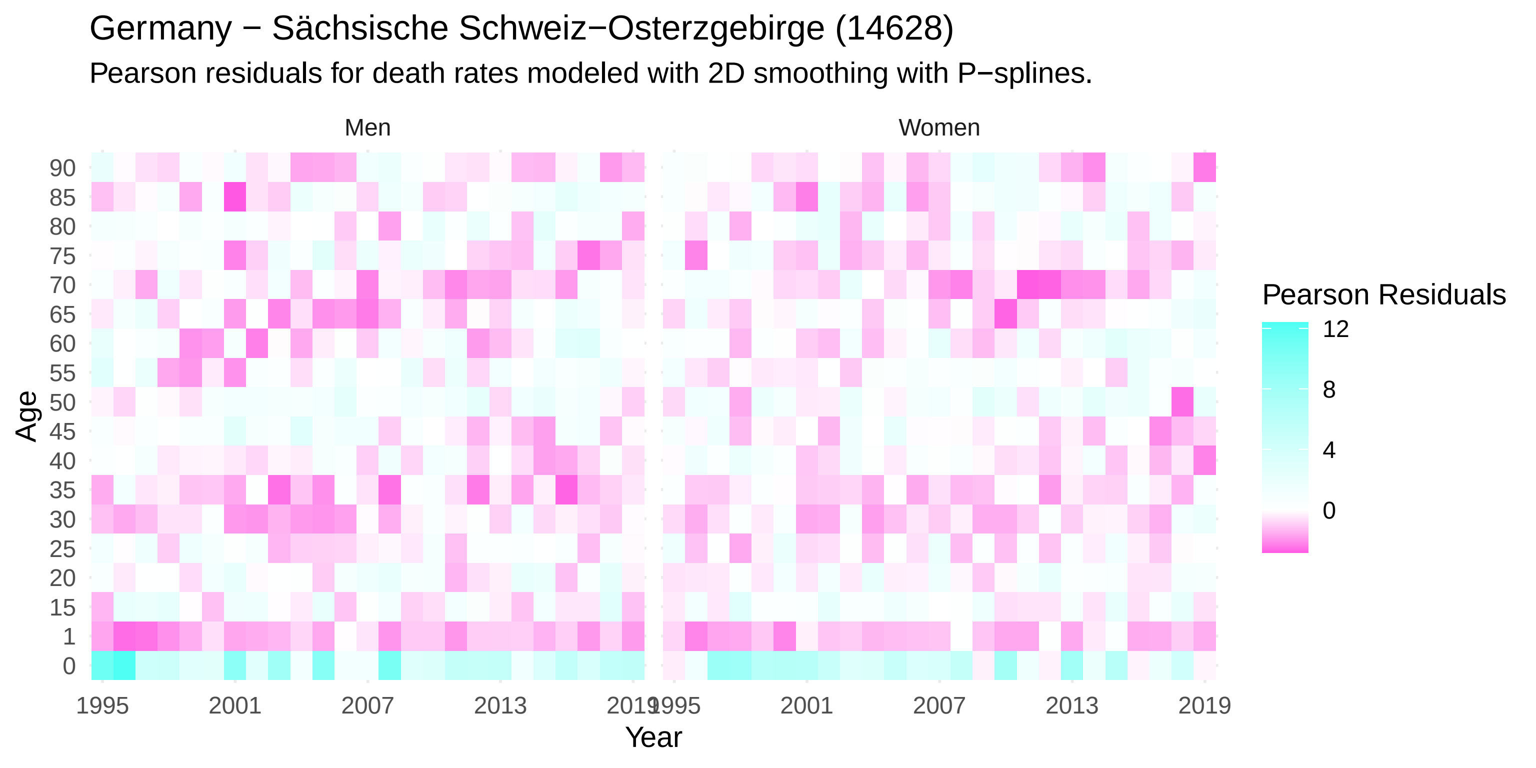

Germany − Sächsische Schweiz−Osterzgebirge (14628)
Pearson residuals for death rates modeled with 2D smoothing with P−splines.
Men
Women
Age
90
85
80
75
70
65
60
55
50
45
40
35
30
25
20
15
1
0
1995
2001
2007
2013
2019
Year
Pearson Residuals
12
8
4
0

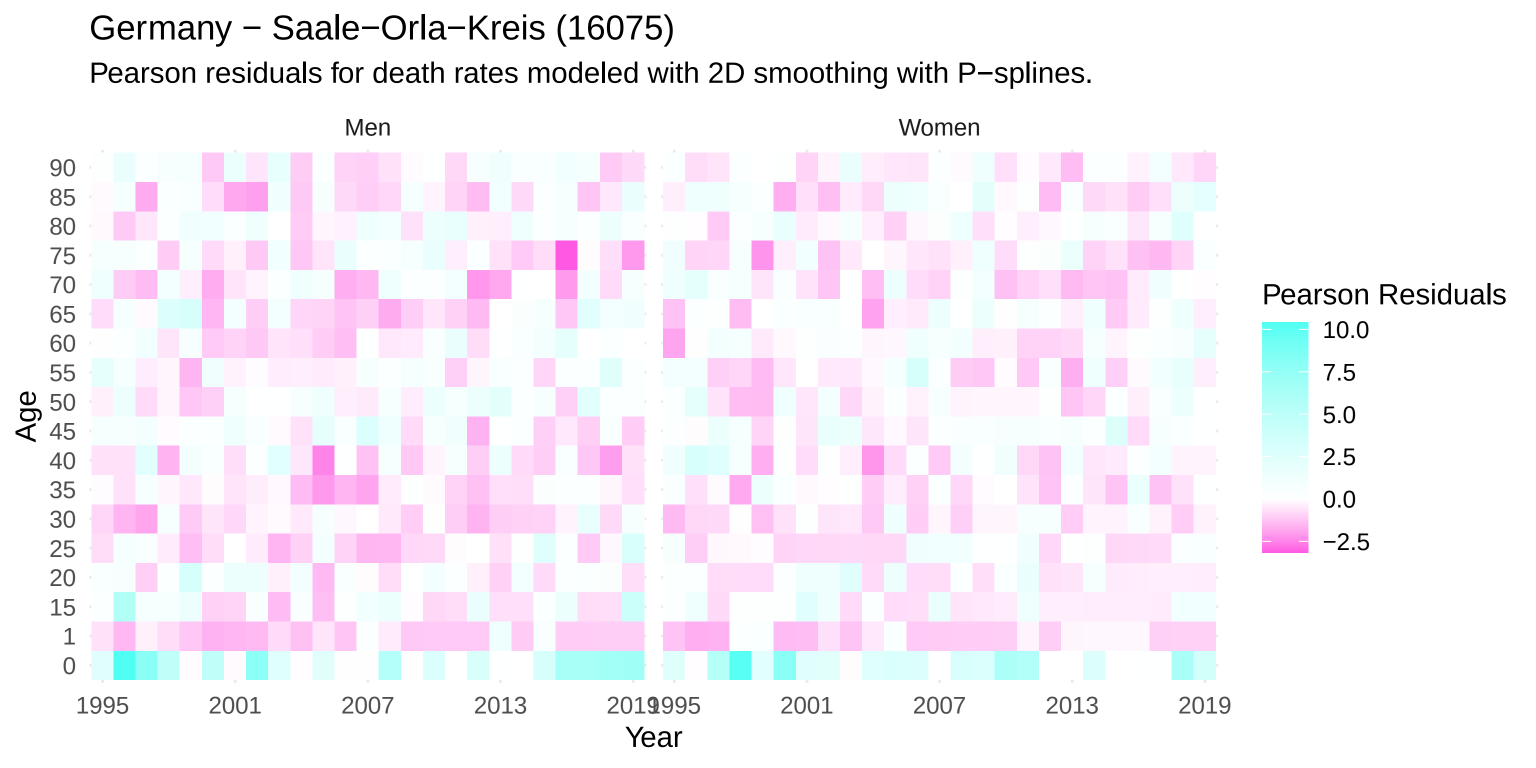

Germany − Saale−Orla−Kreis (16075)
Pearson residuals for death rates modeled with 2D smoothing with P−splines.
Men
Women
Age
90
85
80
75
70
65
60
55
50
45
40
35
30
25
20
15
1
0
1995
2001
2007
2013
2019
Year
Pearson Residuals
10.0
7.5
5.0
2.5
0.0
−2.5

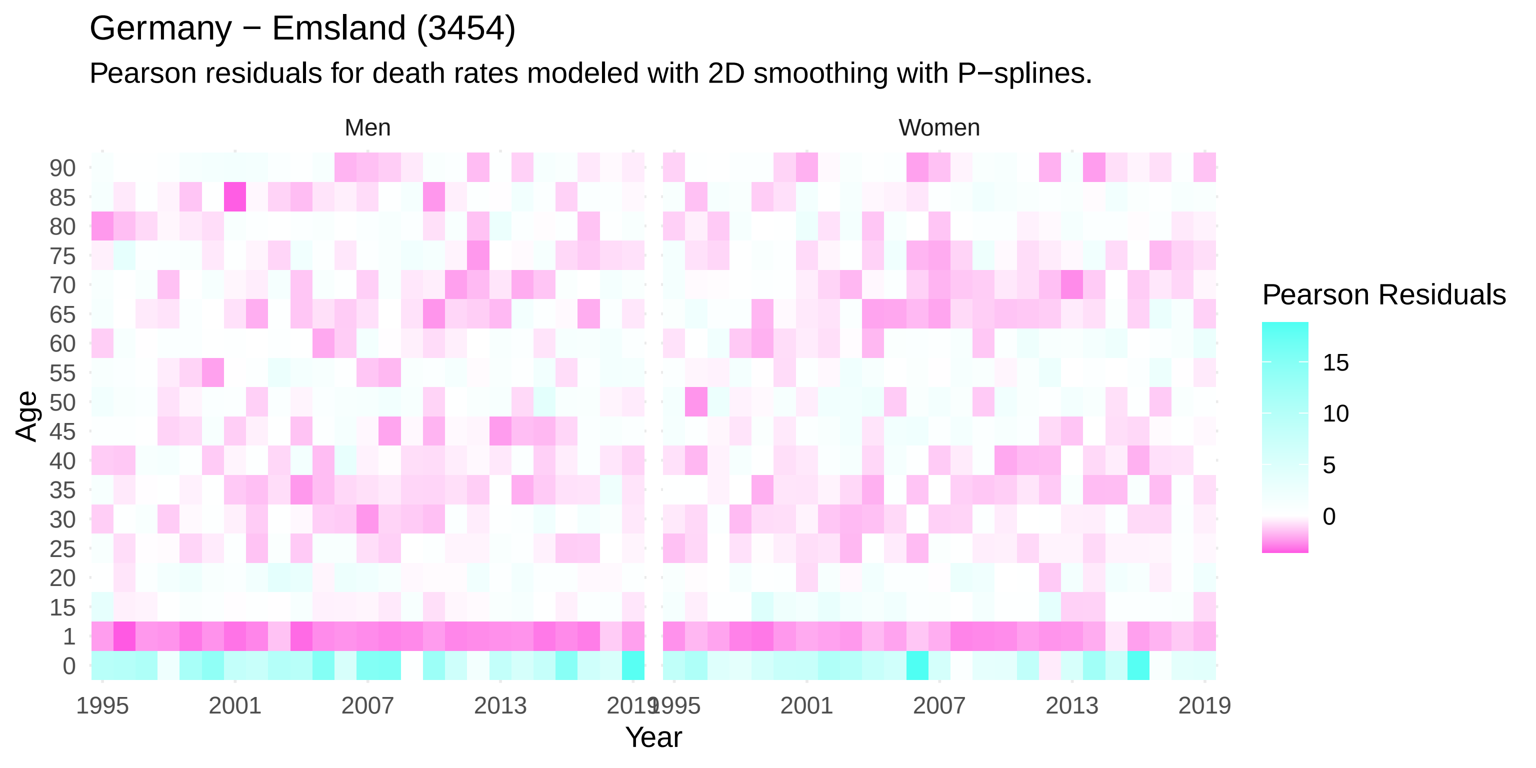
Germany − Emsland (3454)
Pearson residuals for death rates modeled with 2D smoothing with P−splines.
Men
Women
Age
90
85
80
75
70
65
60
55
50
45
40
35
30
25
20
15
1
0
Year
1995
2001
2007
2013
2019
Pearson Residuals
15
10
5
0

Germany − Grafschaft Bentheim (3456)

Pearson residuals for death rates modeled with 2D smoothing with P−splines.

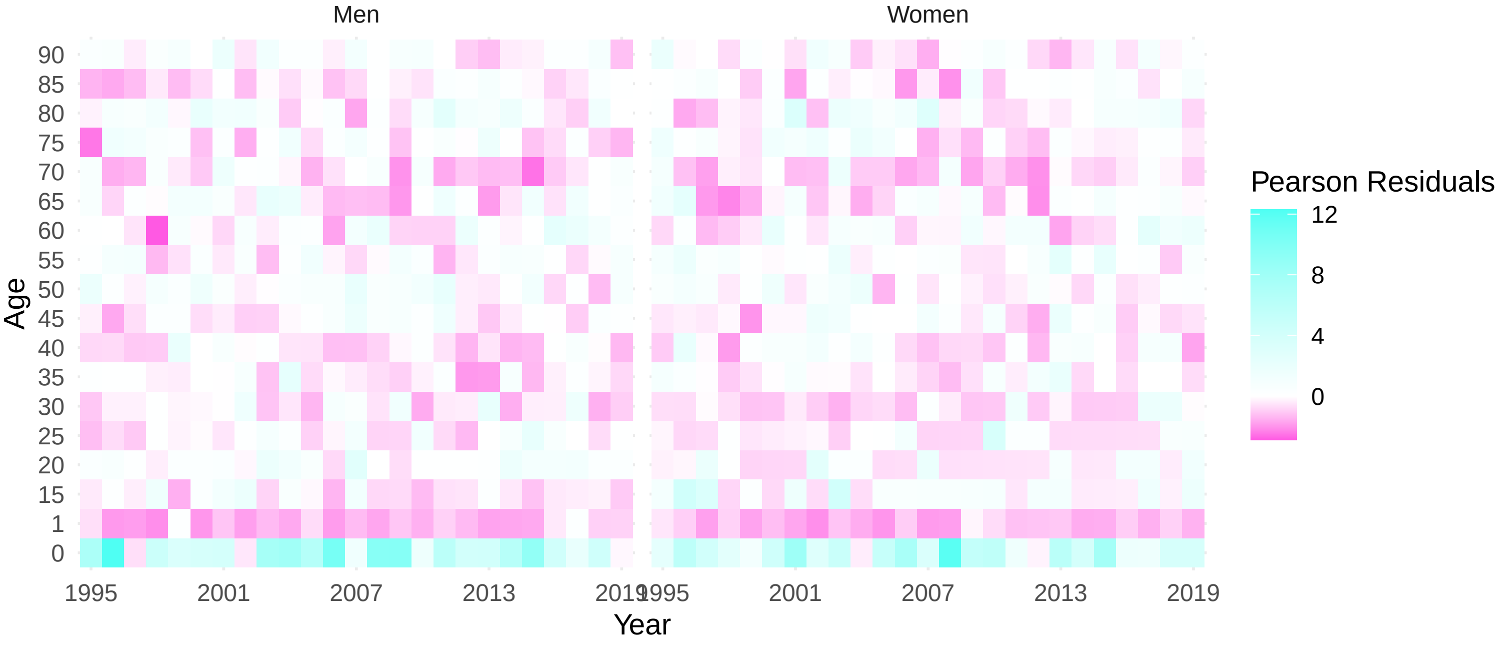

Germany − Leer (3457)

Pearson residuals for death rates modeled with 2D smoothing with P−splines.

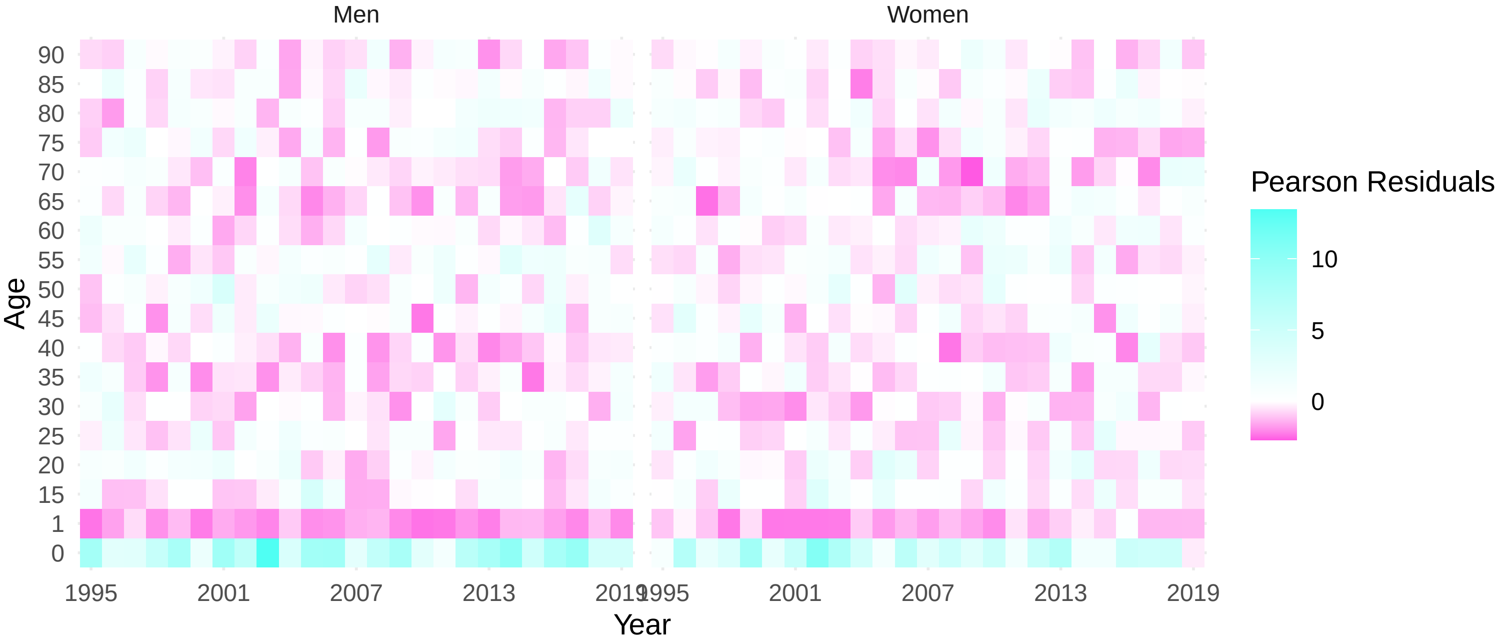

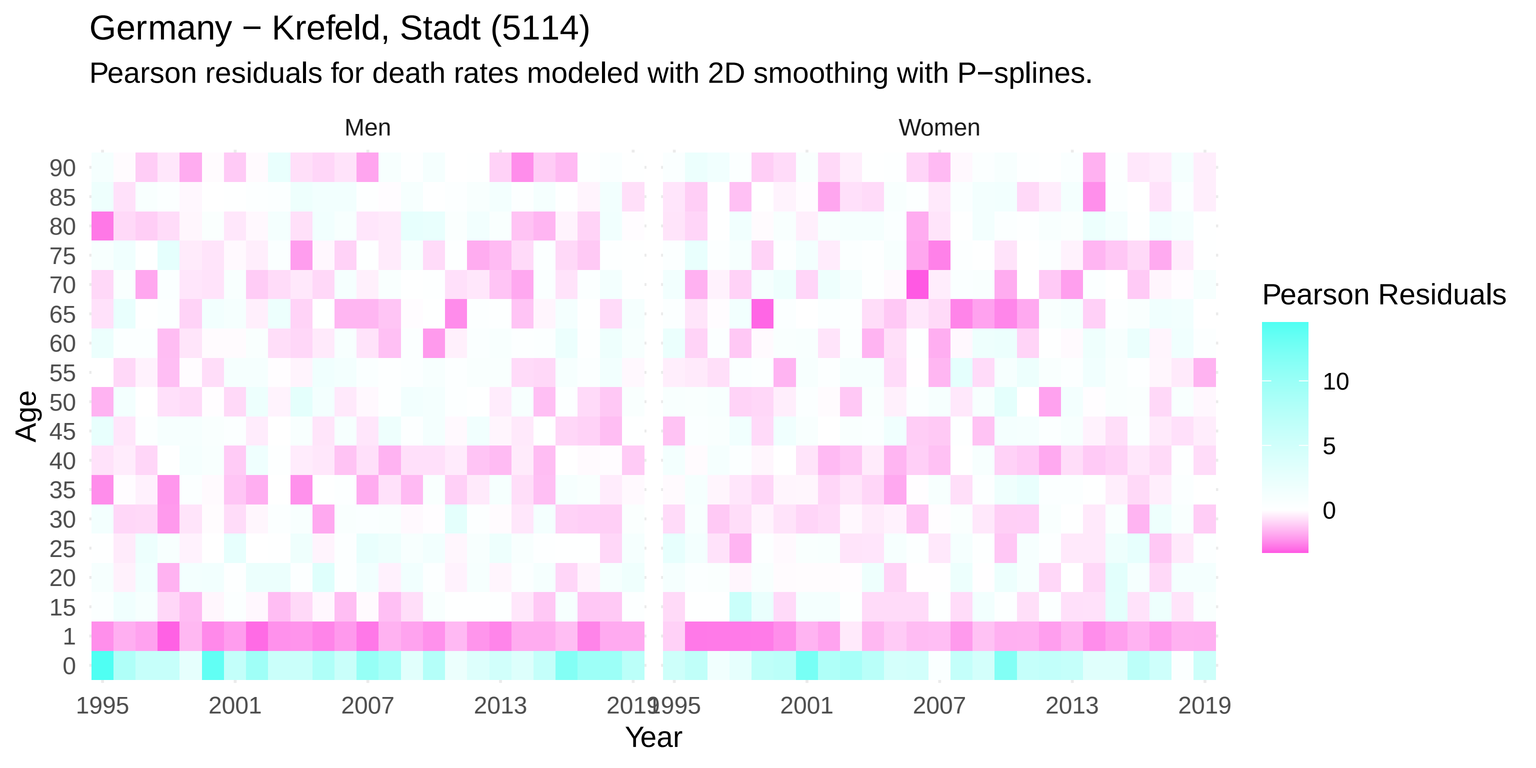

Germany − Krefeld, Stadt (5114)
Pearson residuals for death rates modeled with 2D smoothing with P−splines.
Men
Women
Age
90
85
80
75
70
65
60
55
50
45
40
35
30
25
20
15
1
0
1995
2001
2007
2013
2019
Year
Pearson Residuals
10
5
0

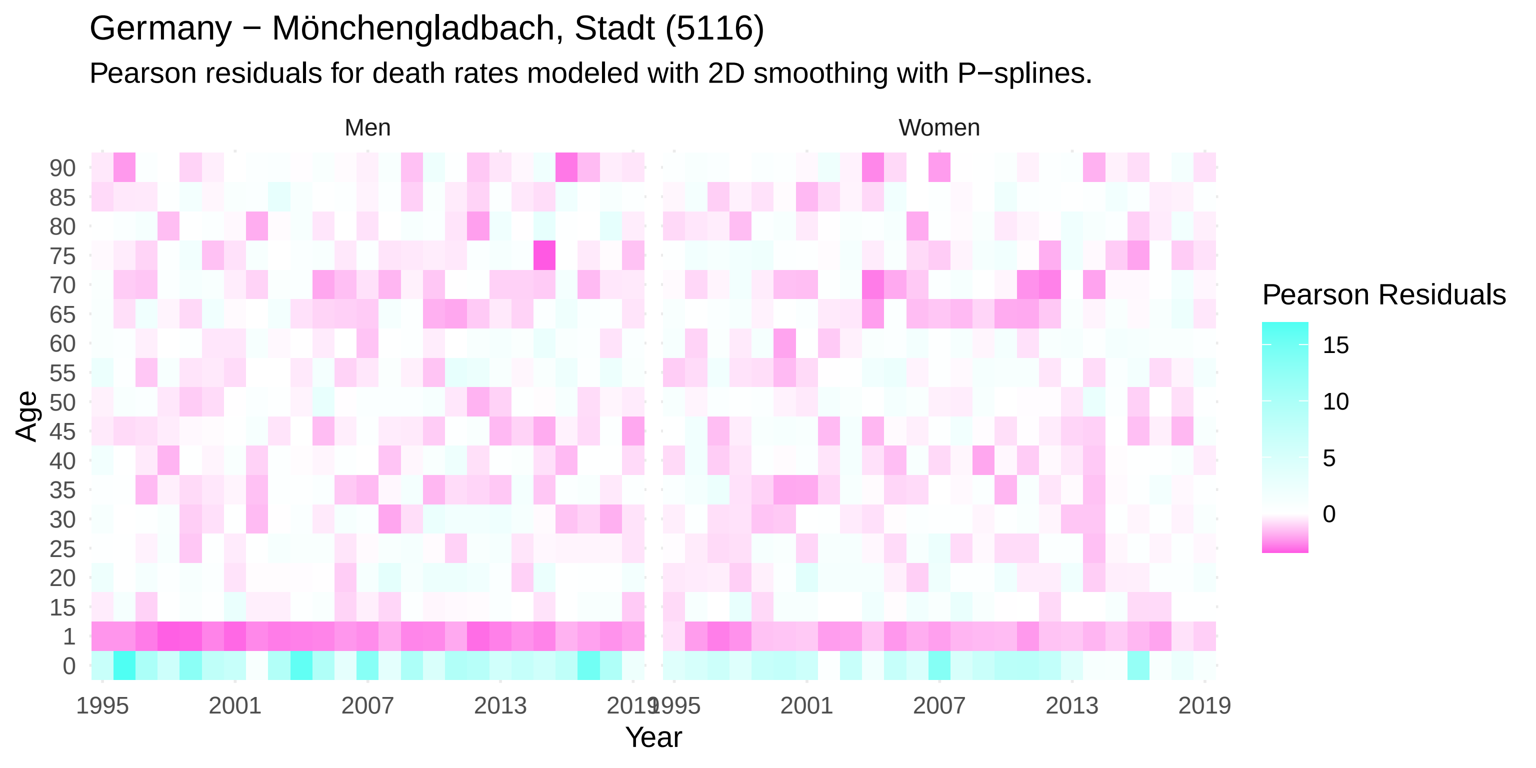

Germany − Mönchengladbach, Stadt (5116)
Pearson residuals for death rates modeled with 2D smoothing with P−splines.
Men
Women
Age
90
85
80
75
70
65
60
55
50
45
40
35
30
25
20
15
1
0
1995
2001
2007
2013
2019
Year
Pearson Residuals
15
10
5
0

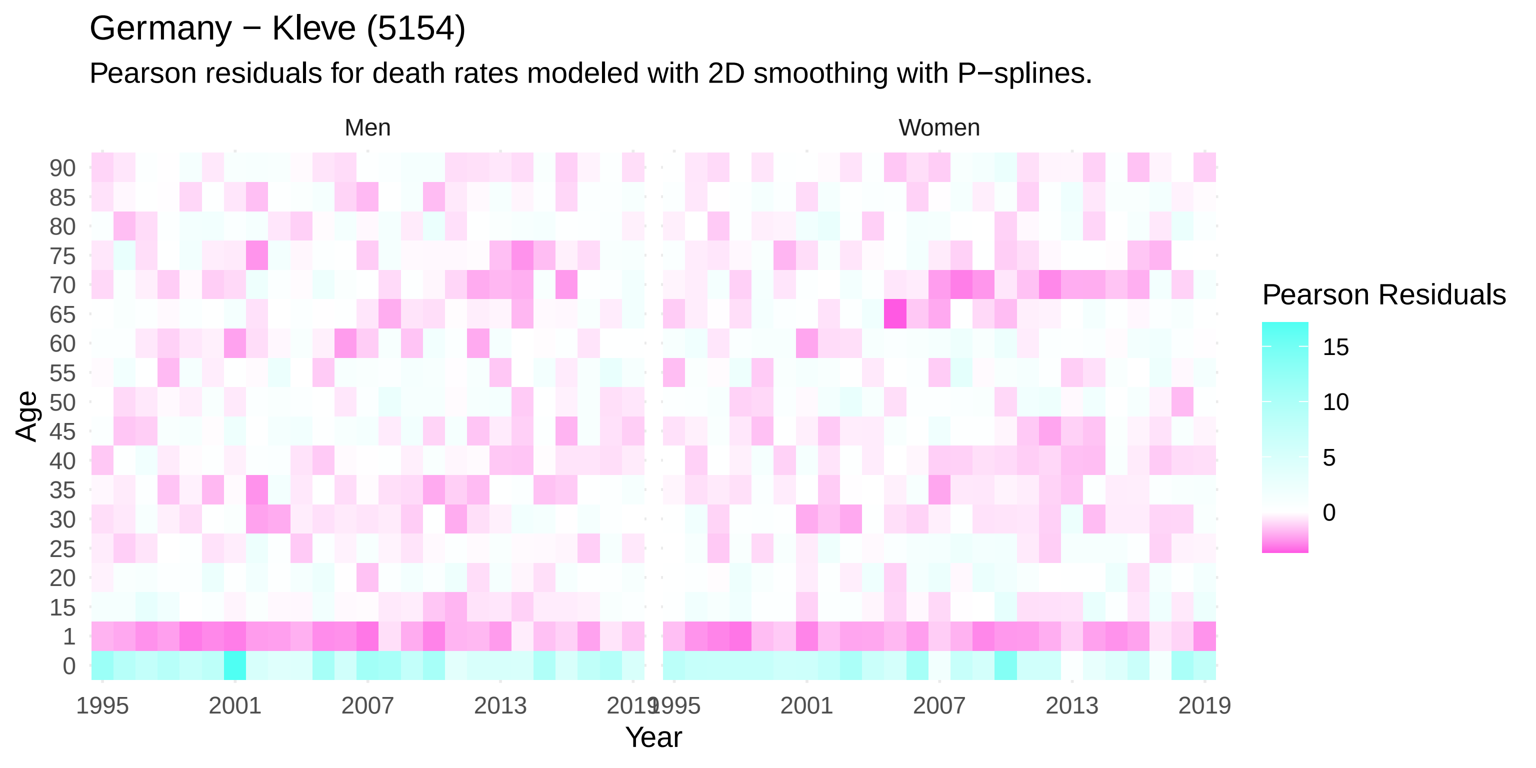
Germany − Kleve (5154)
Pearson residuals for death rates modeled with 2D smoothing with P−splines.
Men
Women
Age
90
85
80
75
70
65
60
55
50
45
40
35
30
25
20
15
1
0
1995
2001
2007
2013
2019
Year
Pearson Residuals
15
10
5
0

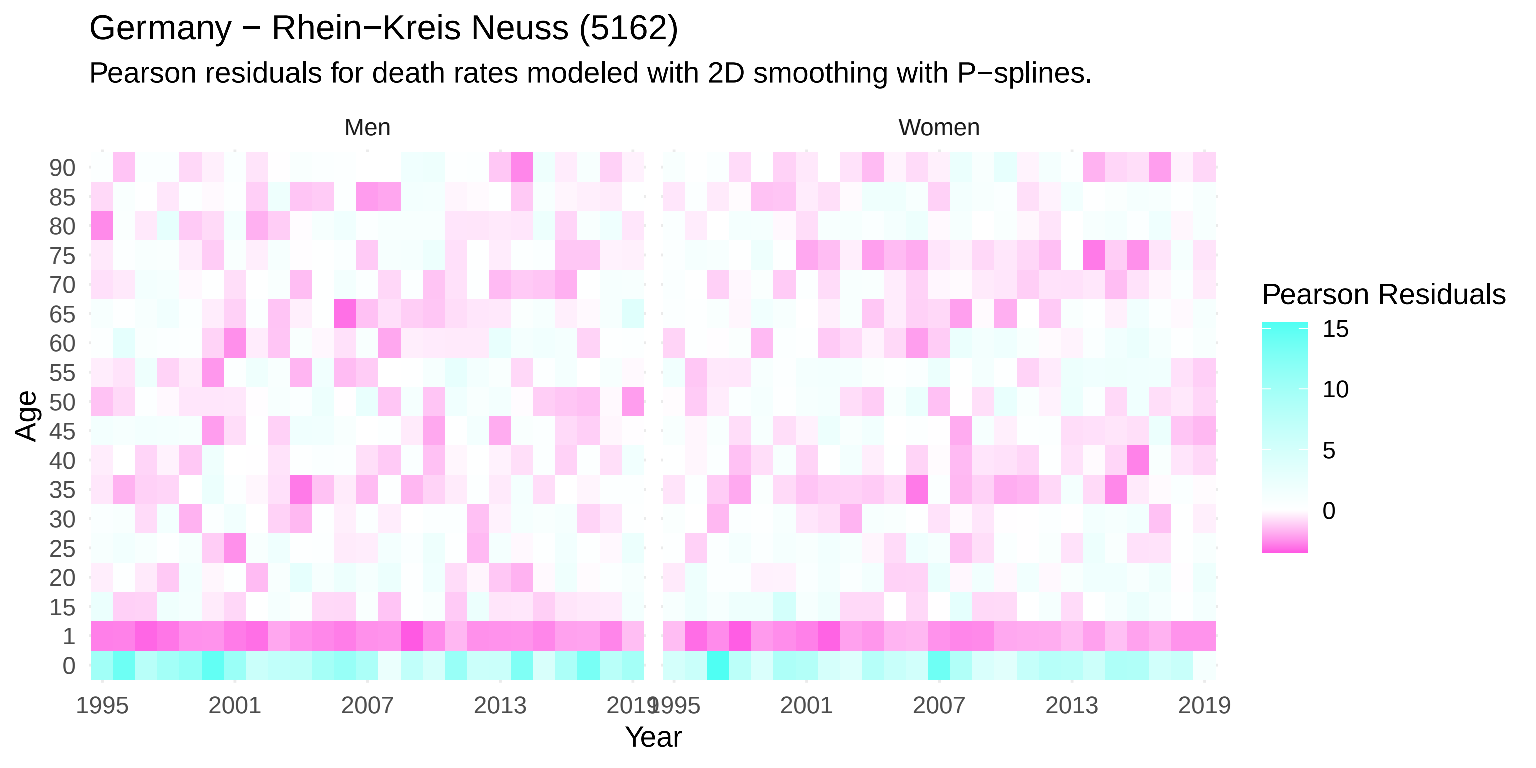

Germany − Rhein−Kreis Neuss (5162)
Pearson residuals for death rates modeled with 2D smoothing with P−splines.
Men
Women
Age
90
85
80
75
70
65
60
55
50
45
40
35
30
25
20
15
1
0
1995
2001
2007
2013
2019
Year
Pearson Residuals
15
10
5
0

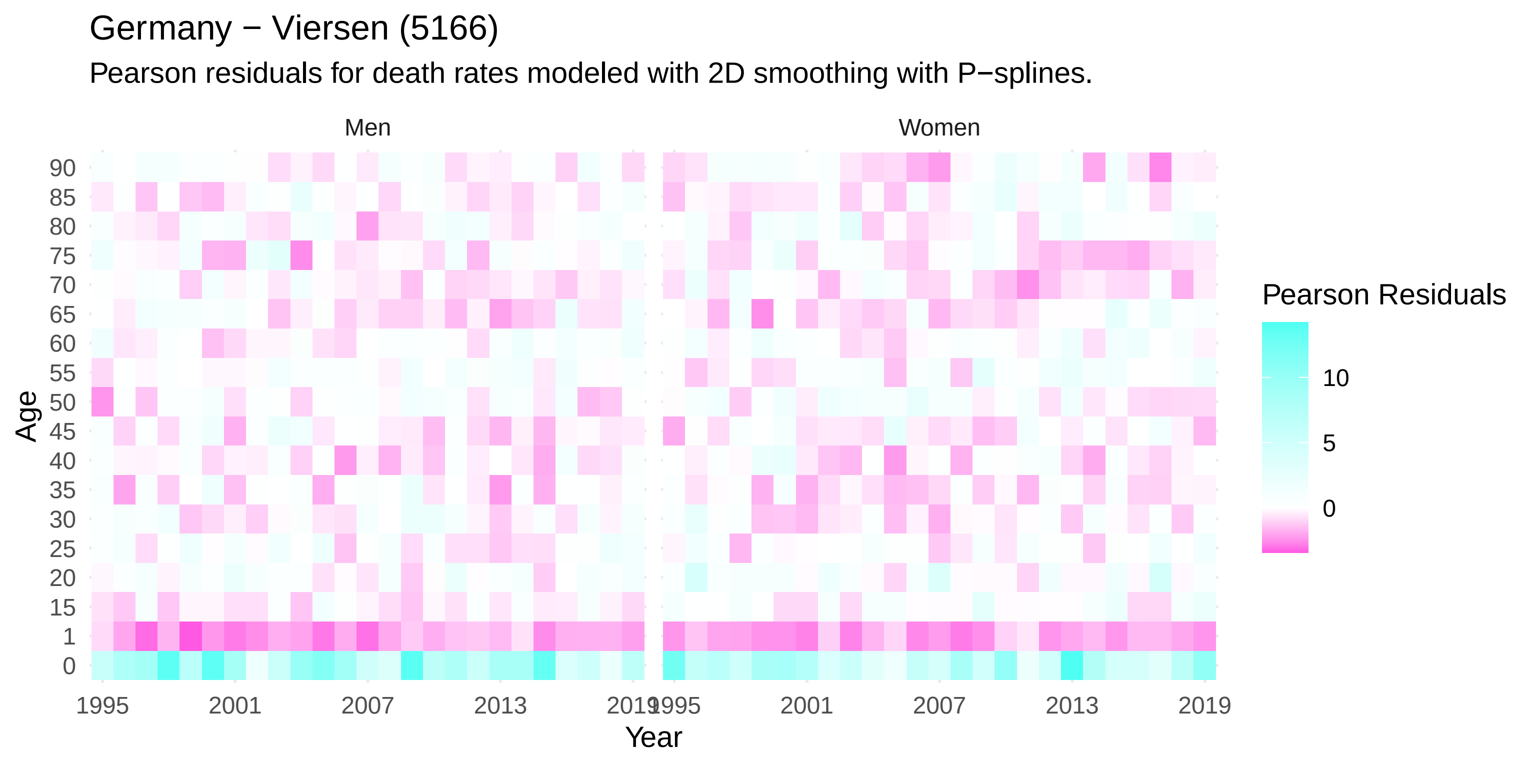

Germany − Viersen (5166)
Pearson residuals for death rates modeled with 2D smoothing with P−splines.
Men
Women
Age
90
85
80
75
70
65
60
55
50
45
40
35
30
25
20
15
1
0
1995
2001
2007
2013
2019
Year
Pearson Residuals
10
5
0

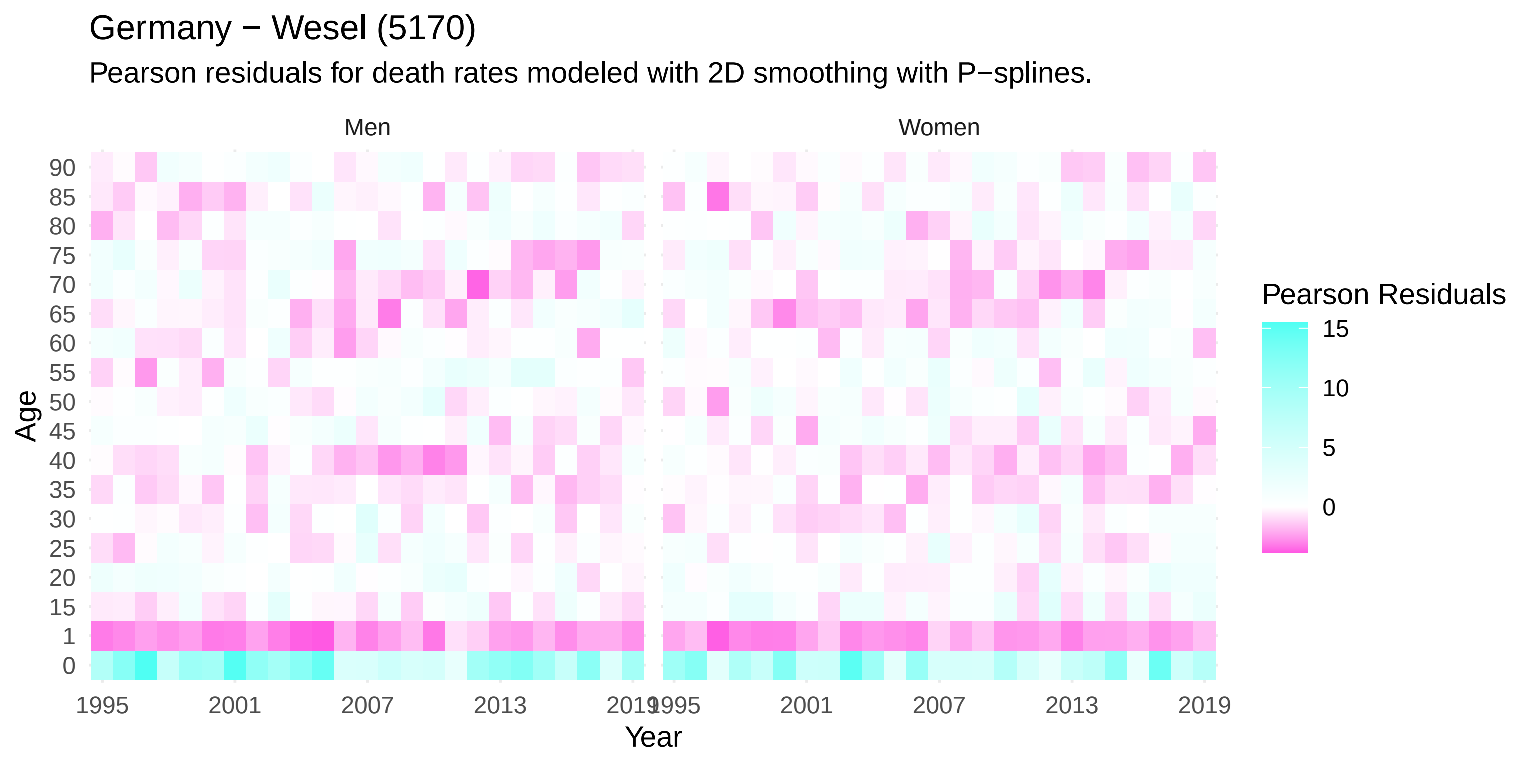

Germany − Wesel (5170)
Pearson residuals for death rates modeled with 2D smoothing with P−splines.
Men
Women
Age
90
85
80
75
70
65
60
55
50
45
40
35
30
25
20
15
1
0
1995
2001
2007
2013
2019
Year
Pearson Residuals
15
10
5
0

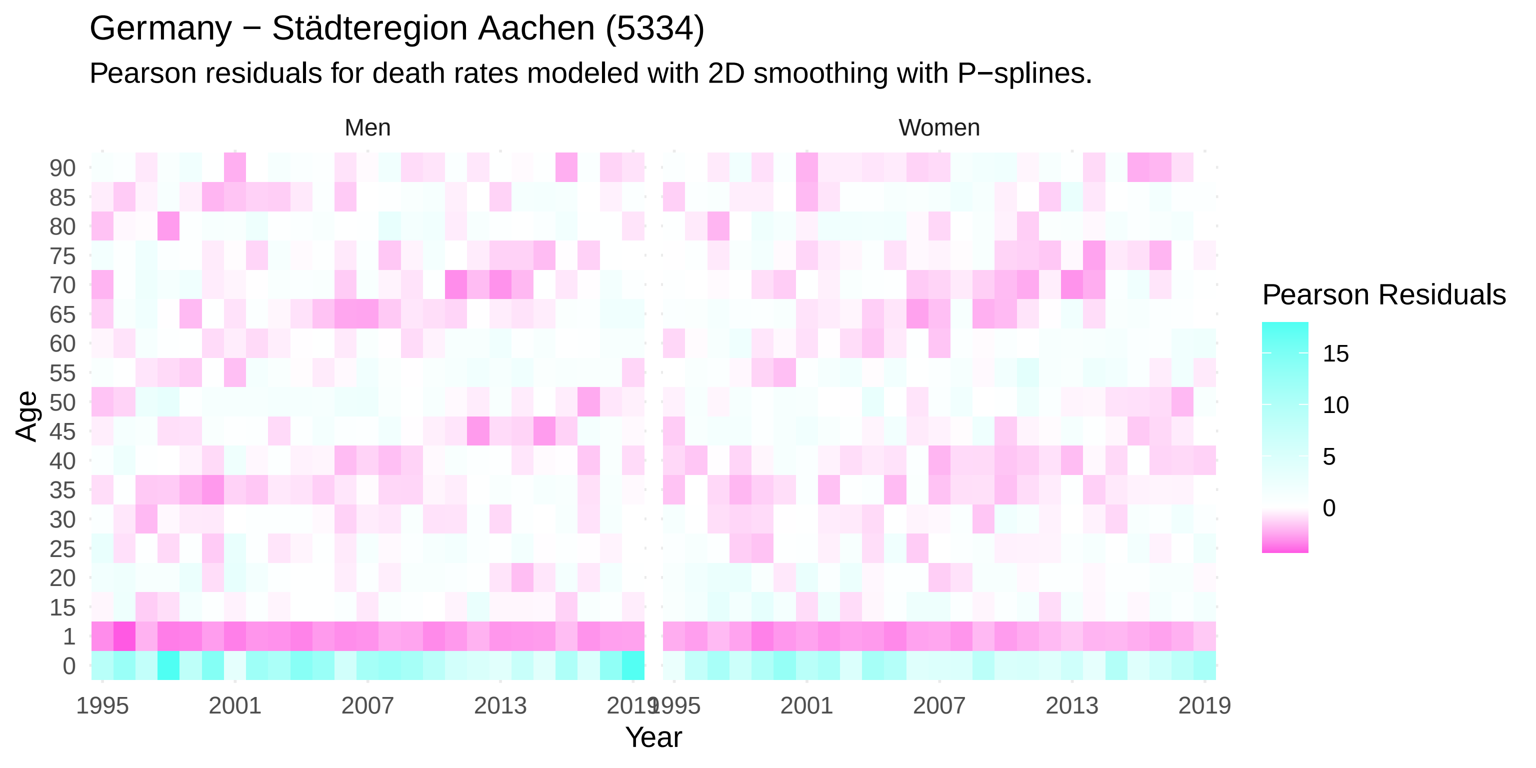

Germany − Städteregion Aachen (5334)
Pearson residuals for death rates modeled with 2D smoothing with P−splines.
Men
Women
Age
90
85
80
75
70
65
60
55
50
45
40
35
30
25
20
15
1
0
1995
2001
2007
2013
2019
Year
Pearson Residuals
15
10
5
0

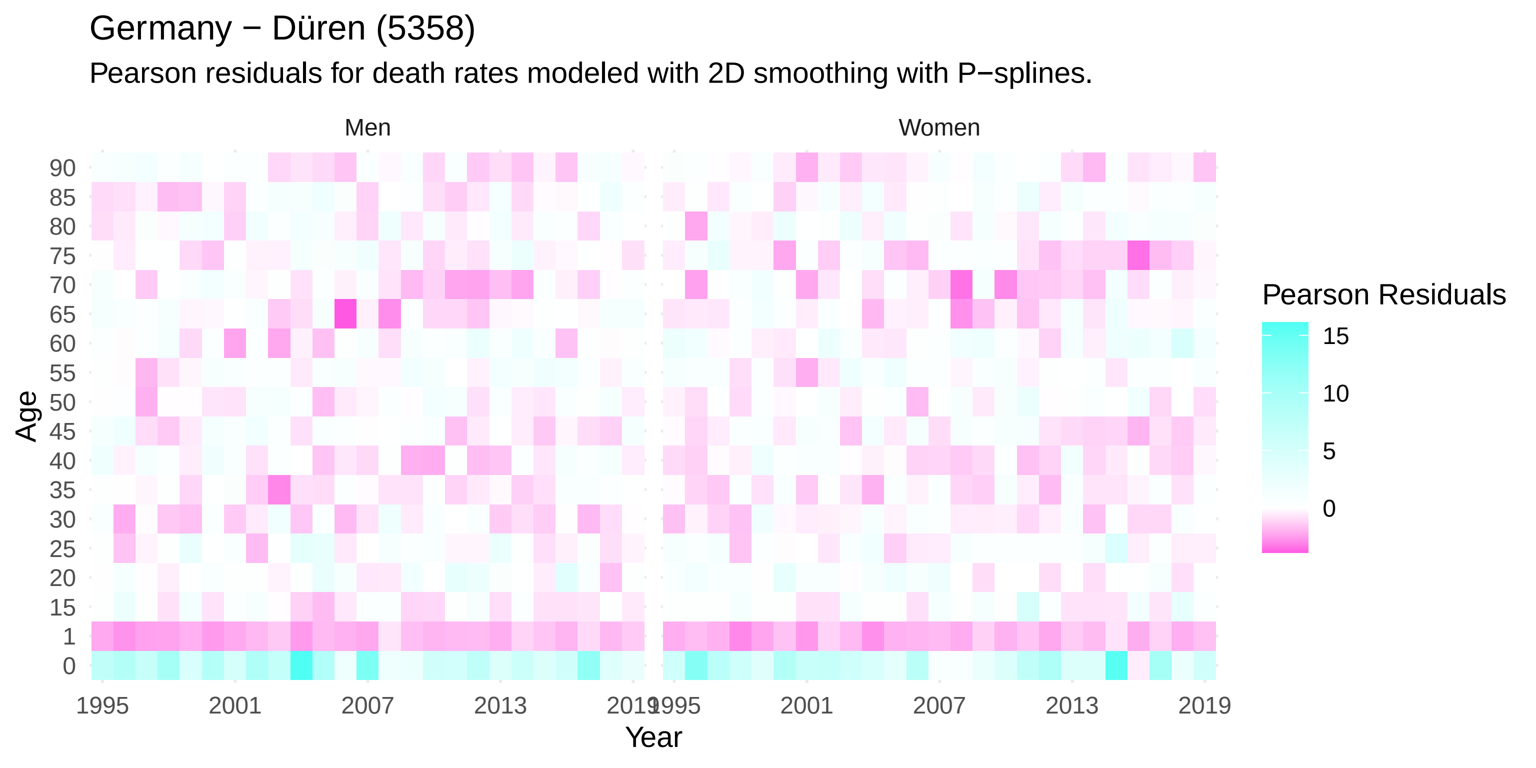
Germany − Düren (5358)
Pearson residuals for death rates modeled with 2D smoothing with P−splines.
Men
Women
Age
90
85
80
75
70
65
60
55
50
45
40
35
30
25
20
15
1
0
1995
2001
2007
2013
2019
Year
Pearson Residuals
15
10
5
0

Germany − Rhein−Erft−Kreis (5362)

Pearson residuals for death rates modeled with 2D smoothing with P−splines.

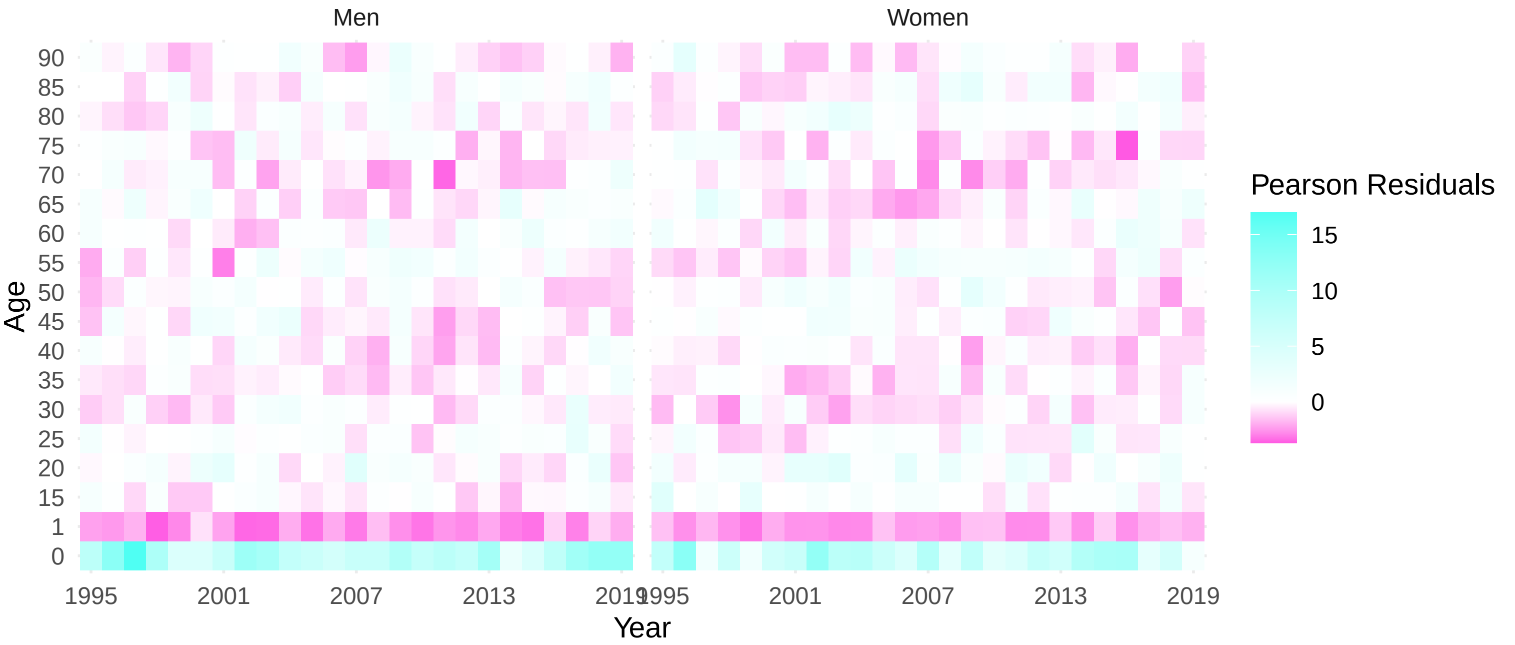

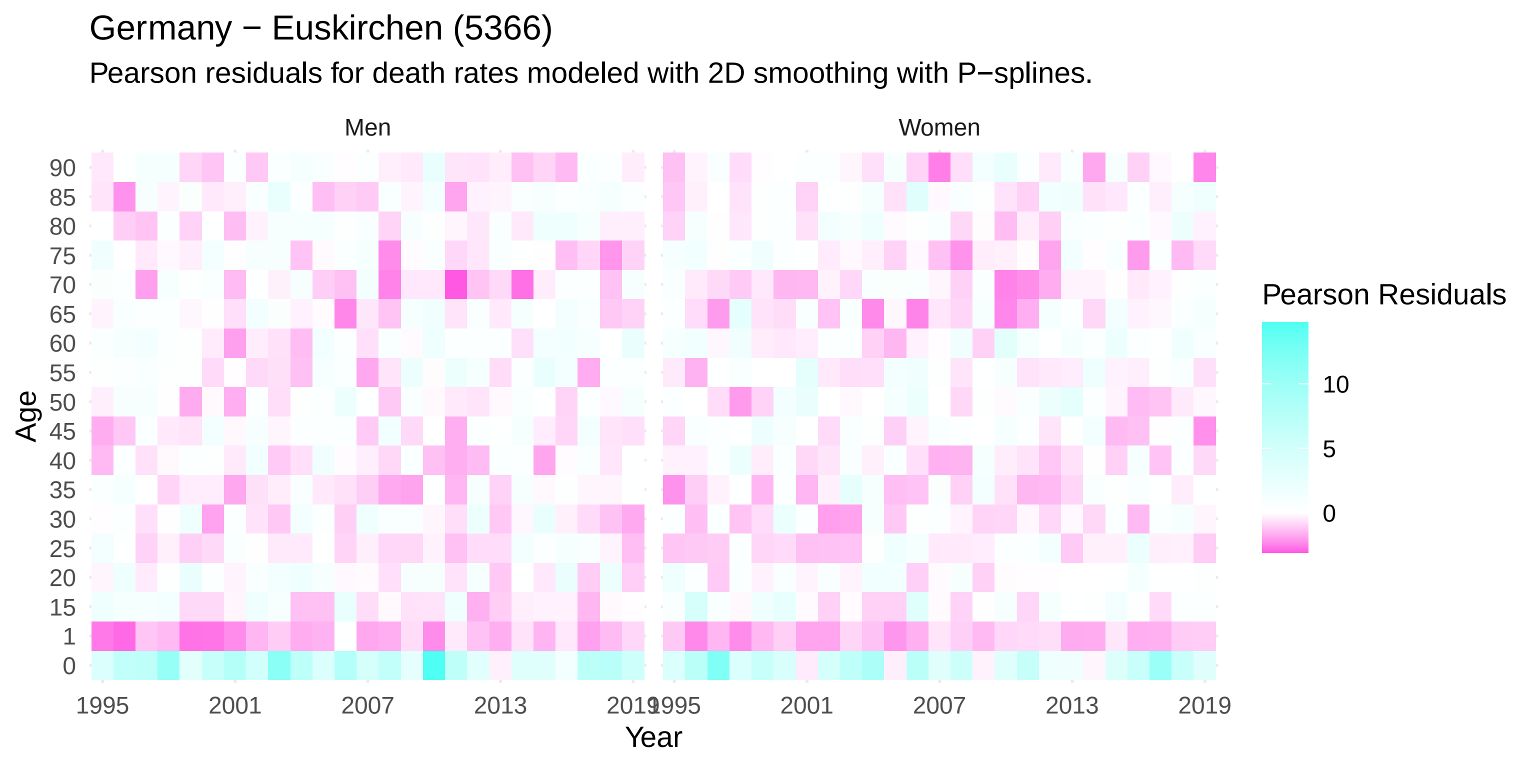

Germany − Euskirchen (5366)
Pearson residuals for death rates modeled with 2D smoothing with P−splines.
Men
Women
Age
90
85
80
75
70
65
60
55
50
45
40
35
30
25
20
15
1
0
1995
2001
2007
2013
2019
Year
Pearson Residuals
10
5
0

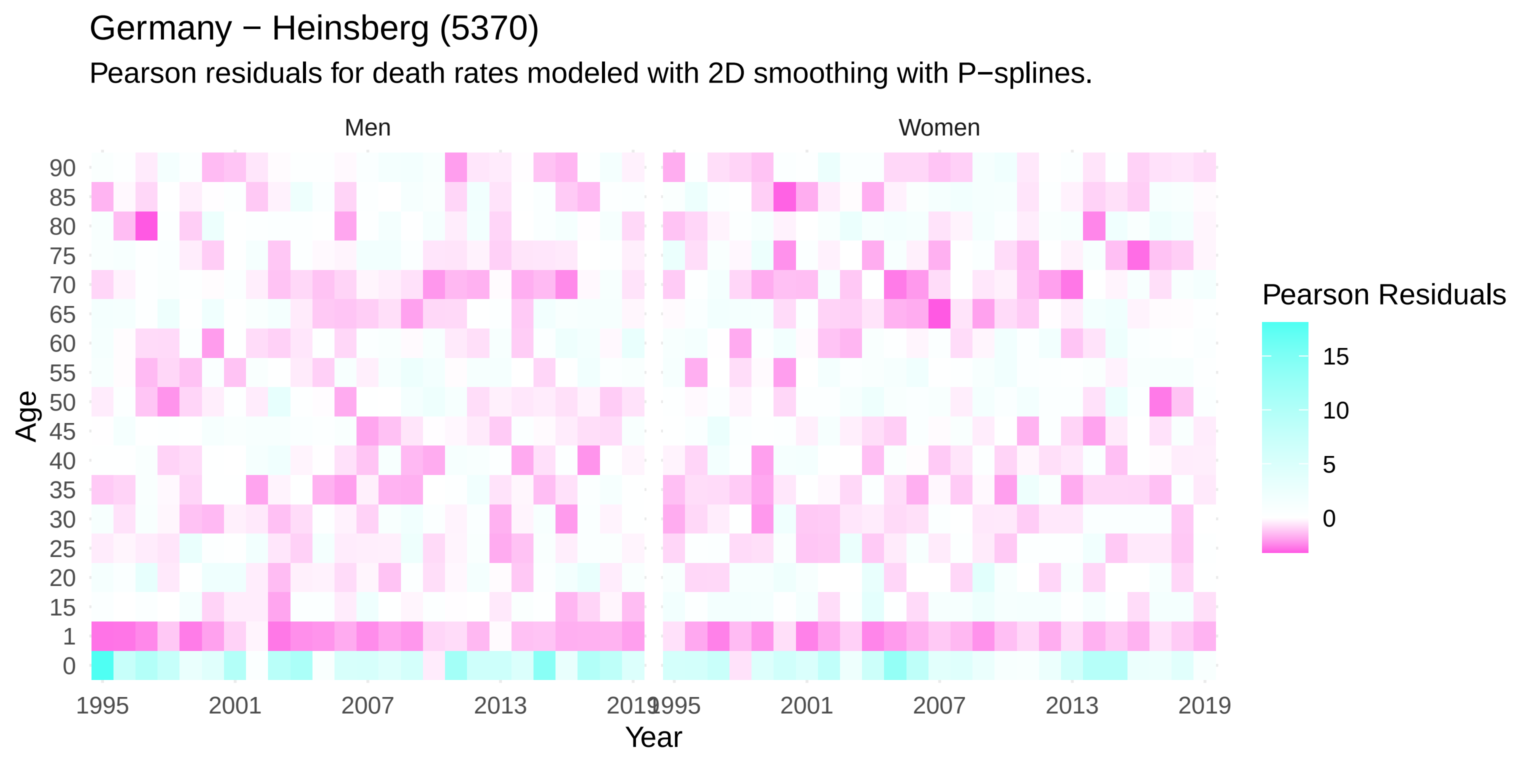

Germany − Heinsberg (5370)
Pearson residuals for death rates modeled with 2D smoothing with P−splines.
Men
Women
Age
90
85
80
75
70
65
60
55
50
45
40
35
30
25
20
15
1
0
1995
2001
2007
2013
2019
Year
Pearson Residuals
15
10
5
0

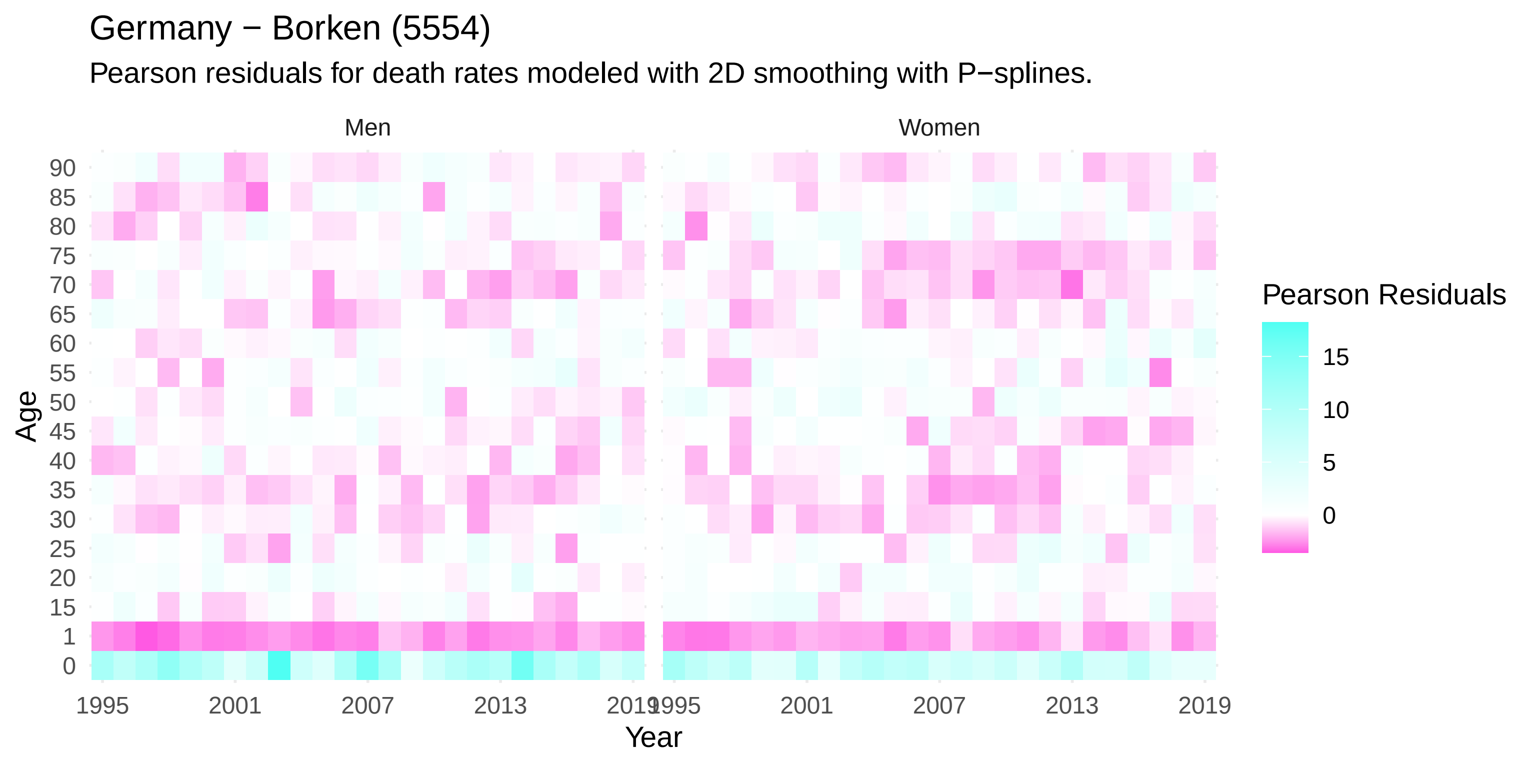
Germany − Borken (5554)
Pearson residuals for death rates modeled with 2D smoothing with P−splines.
Men
Women
Age
90
85
80
75
70
65
60
55
50
45
40
35
30
25
20
15
1
0
1995
2001
2007
2013
2019
Year
Pearson Residuals
15
10
5
0

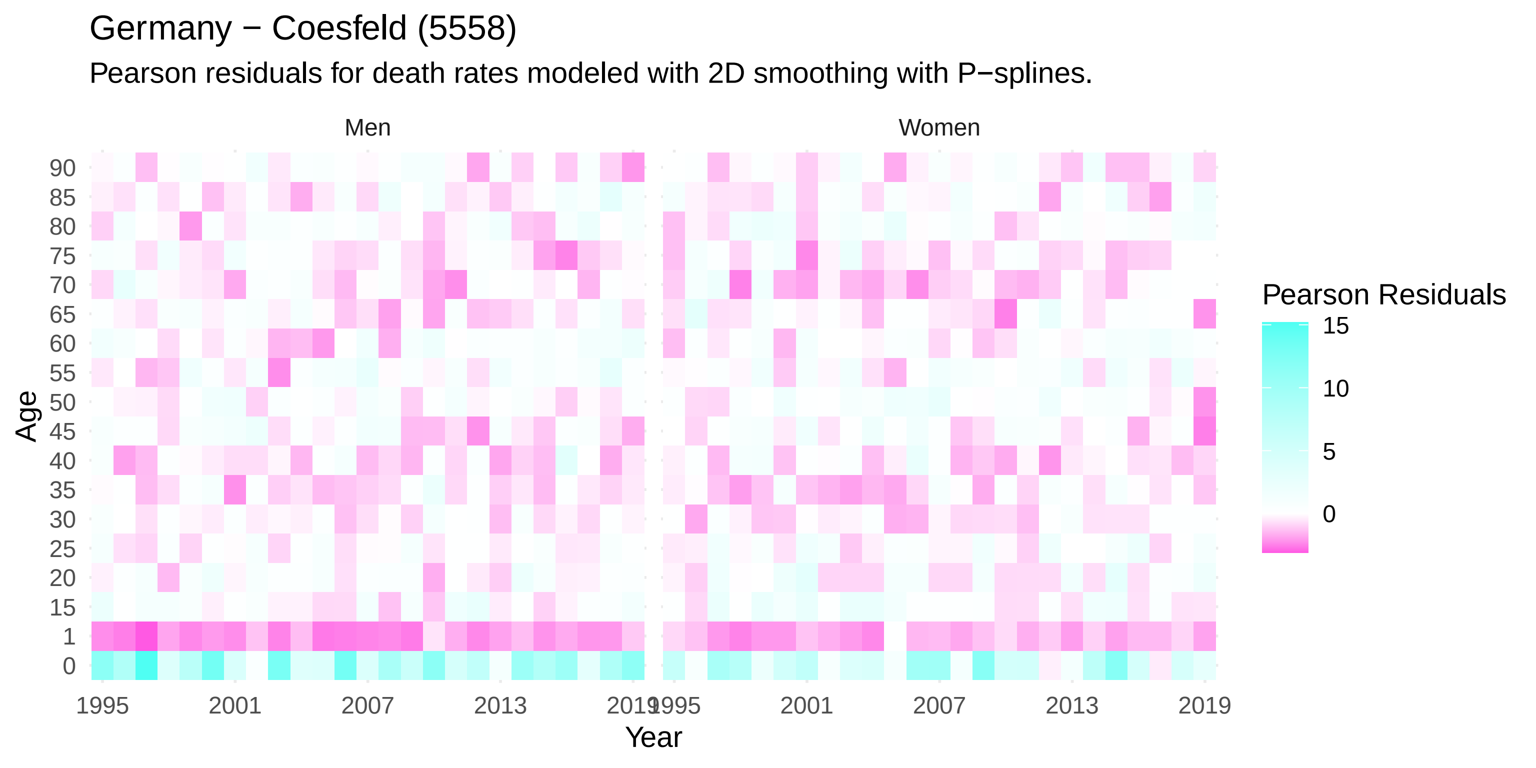

Germany − Coesfeld (5558)
Pearson residuals for death rates modeled with 2D smoothing with P−splines.
Men
Women
Age
90
85
80
75
70
65
60
55
50
45
40
35
30
25
20
15
1
0
1995
2001
2007
2013
2019
Year
Pearson Residuals
15
10
5
0

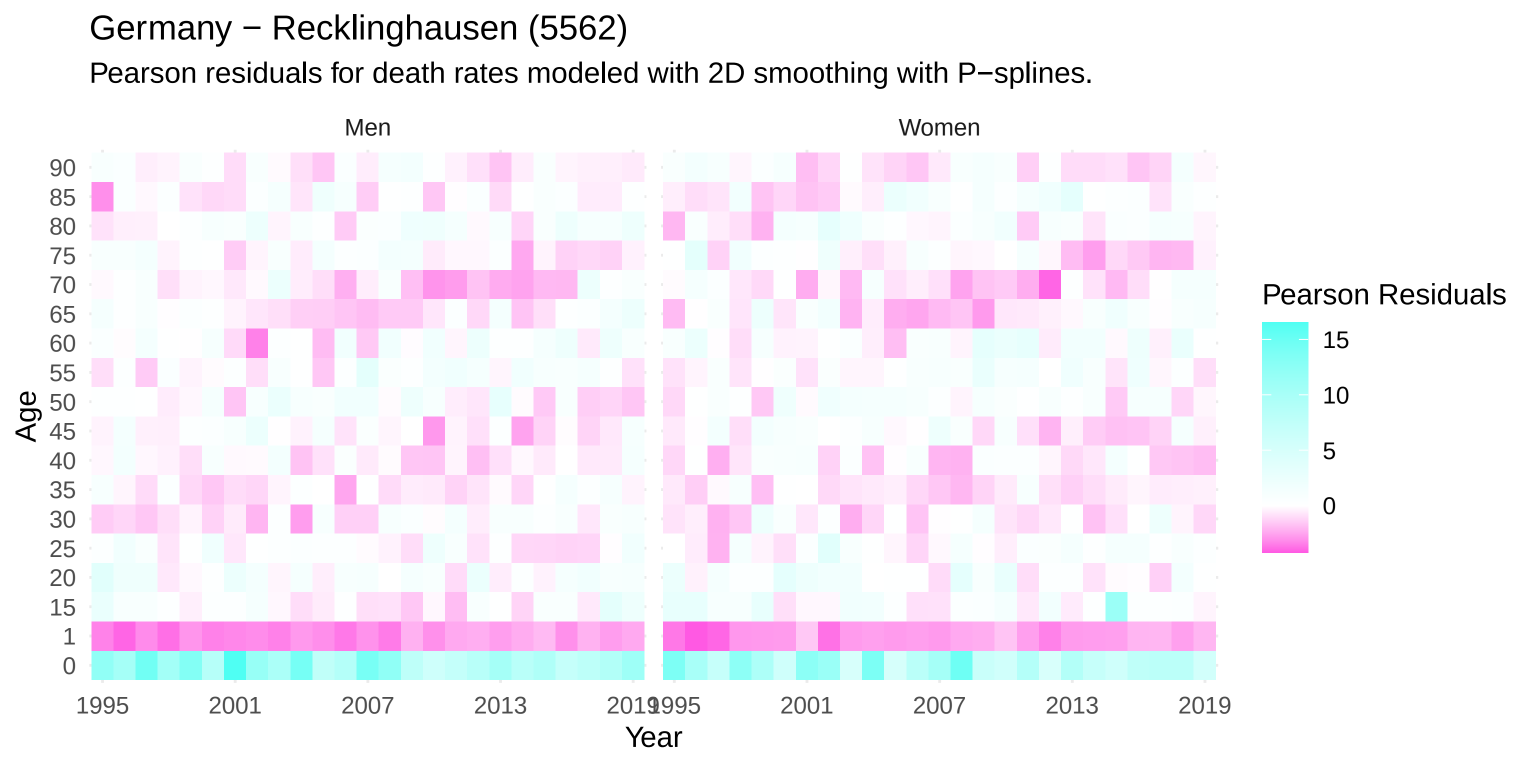

Germany − Recklinghausen (5562)
Pearson residuals for death rates modeled with 2D smoothing with P−splines.
Men
Women
Age
90
85
80
75
70
65
60
55
50
45
40
35
30
25
20
15
1
0
1995
2001
2007
2013
2019
Year
Pearson Residuals
15
10
5
0

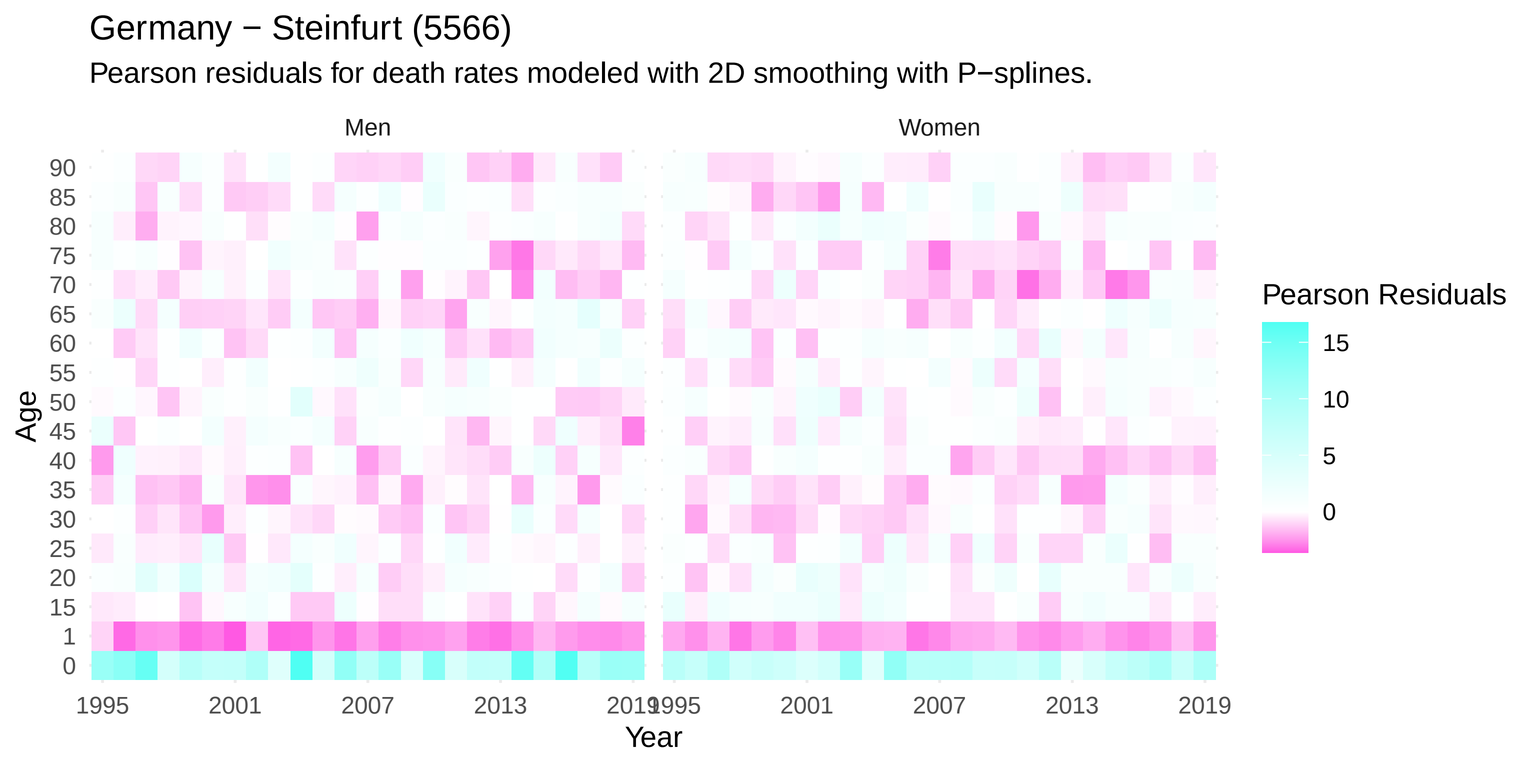

Germany − Steinfurt (5566)
Pearson residuals for death rates modeled with 2D smoothing with P−splines.
Men
Women
Age
Year
90
85
80
75
70
65
60
55
50
45
40
35
30
25
20
15
1
0
1995
2001
2007
2013
2019
Pearson Residuals
15
10
5
0

Germany − Trier, kreisfreie Stadt (7211)

Pearson residuals for death rates modeled with 2D smoothing with P−splines.

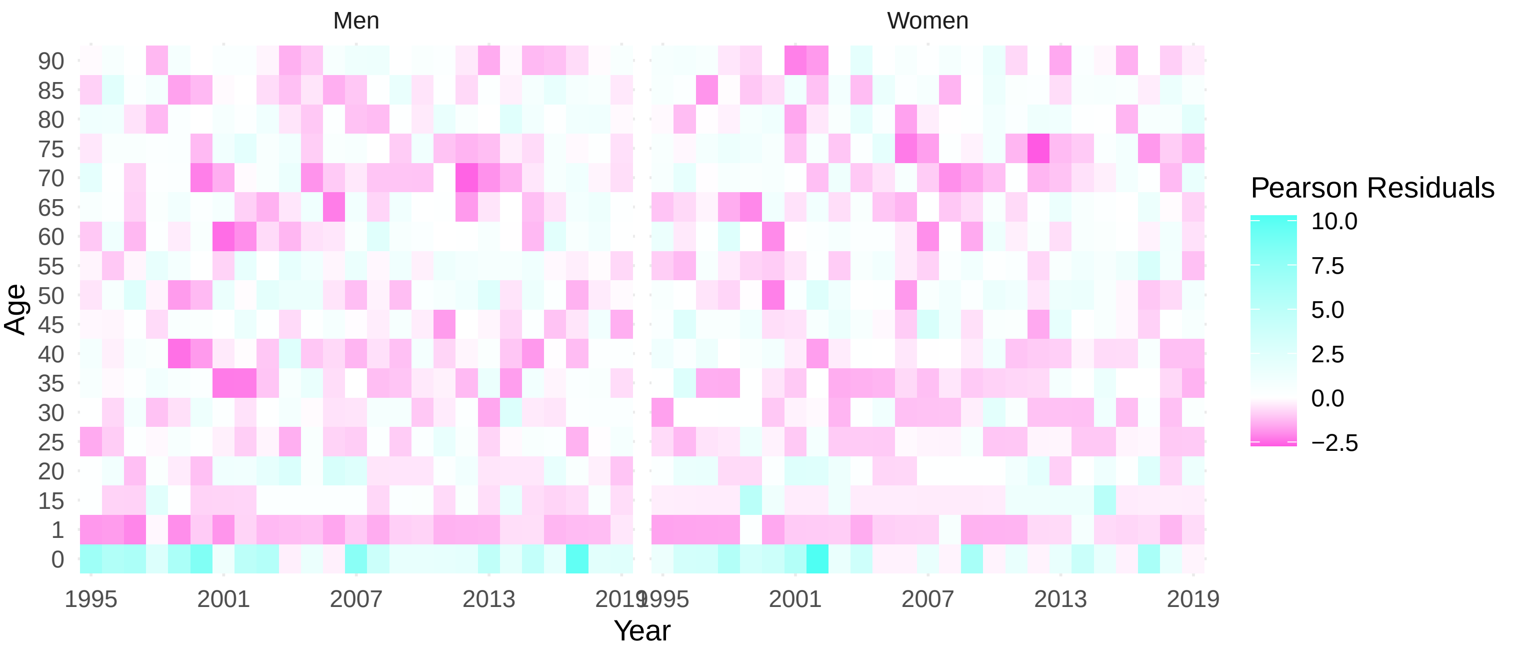

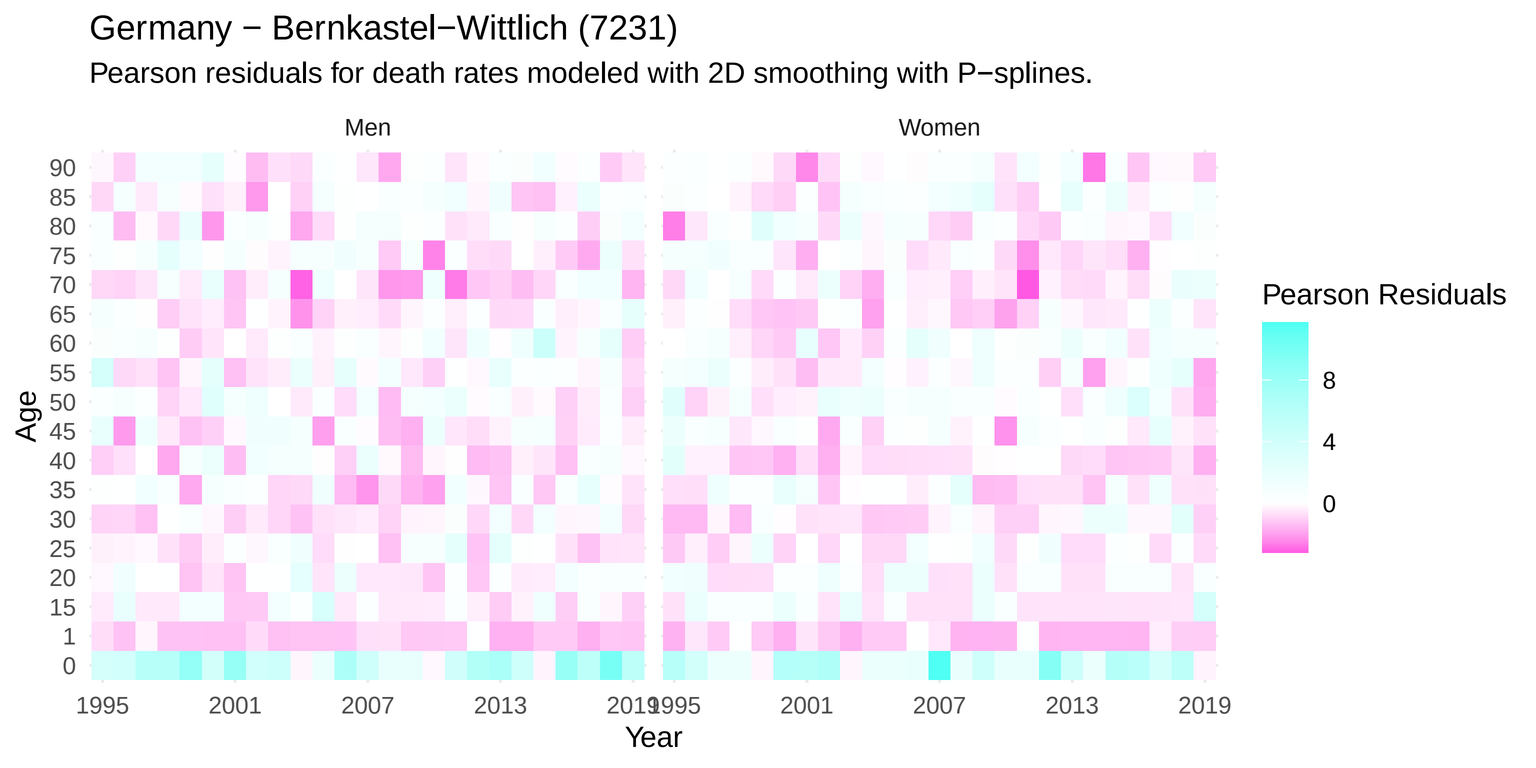

Germany − Bernkastel−Wittlich (7231)
Pearson residuals for death rates modeled with 2D smoothing with P−splines.
Men
Women
Age
90
85
80
75
70
65
60
55
50
45
40
35
30
25
20
15
1
0
1995
2001
2007
2013
2019
Year
Pearson Residuals
8
4
0

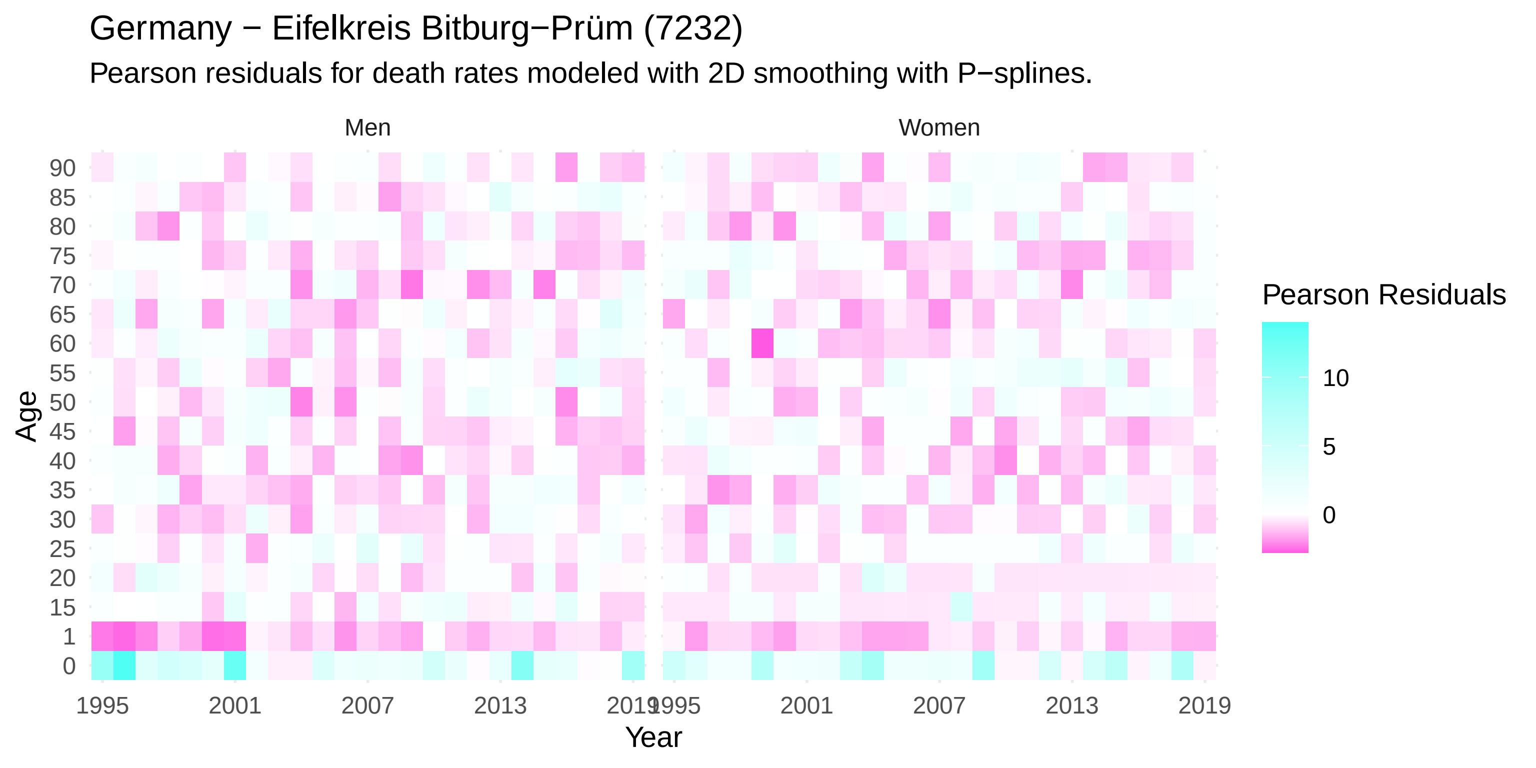

Germany − Eifelkreis Bitburg−Prüm (7232)
Pearson residuals for death rates modeled with 2D smoothing with P−splines.
Men
Women
Age
90
85
80
75
70
65
60
55
50
45
40
35
30
25
20
15
1
0
1995
2001
2007
2013
2019
Year
Pearson Residuals
10
5
0

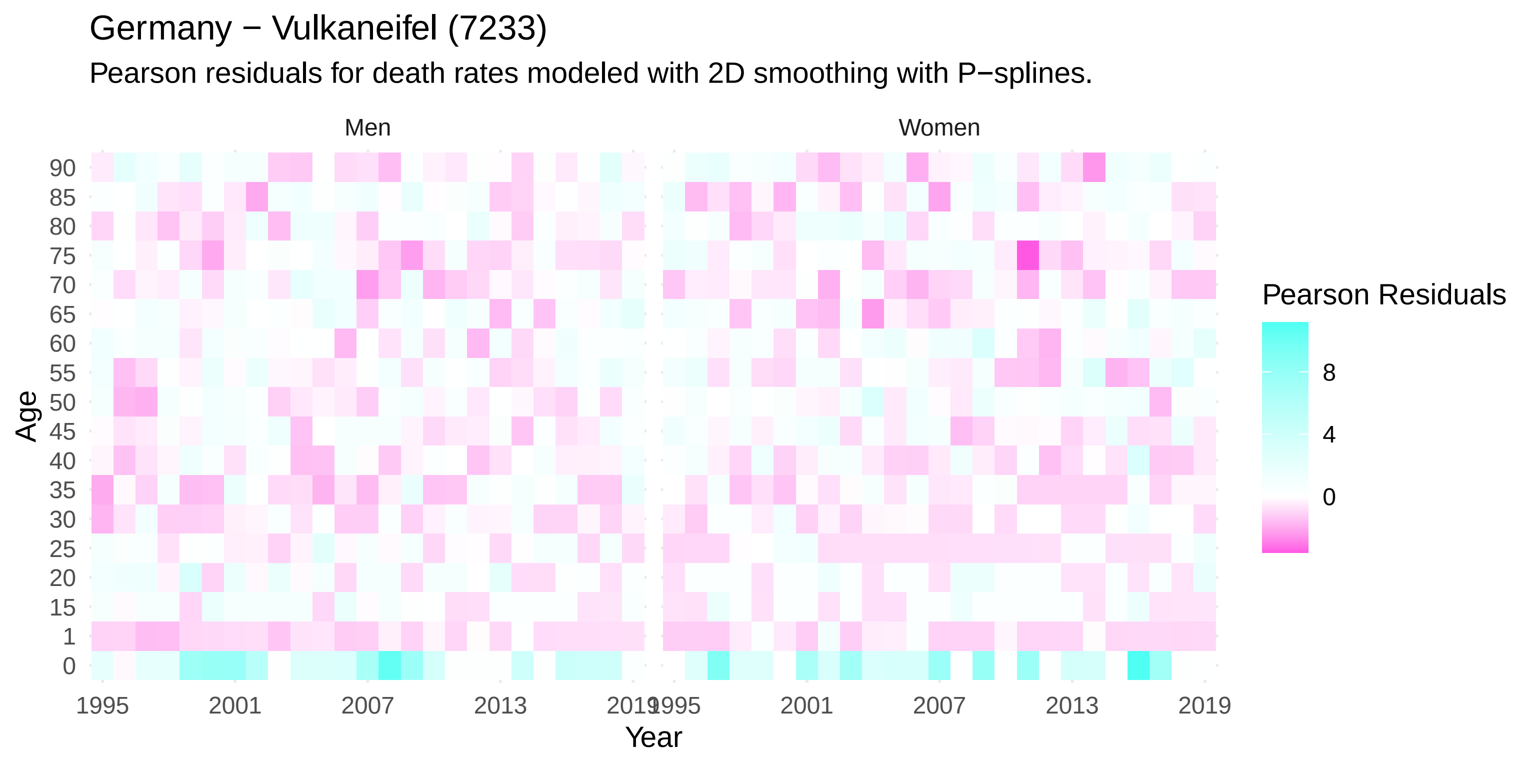

Germany − Vulkaneifel (7233)
Pearson residuals for death rates modeled with 2D smoothing with P−splines.
Men
Women
Age
90
85
80
75
70
65
60
55
50
45
40
35
30
25
20
15
1
0
1995
2001
2007
2013
2019
Year
Pearson Residuals
8
4
0

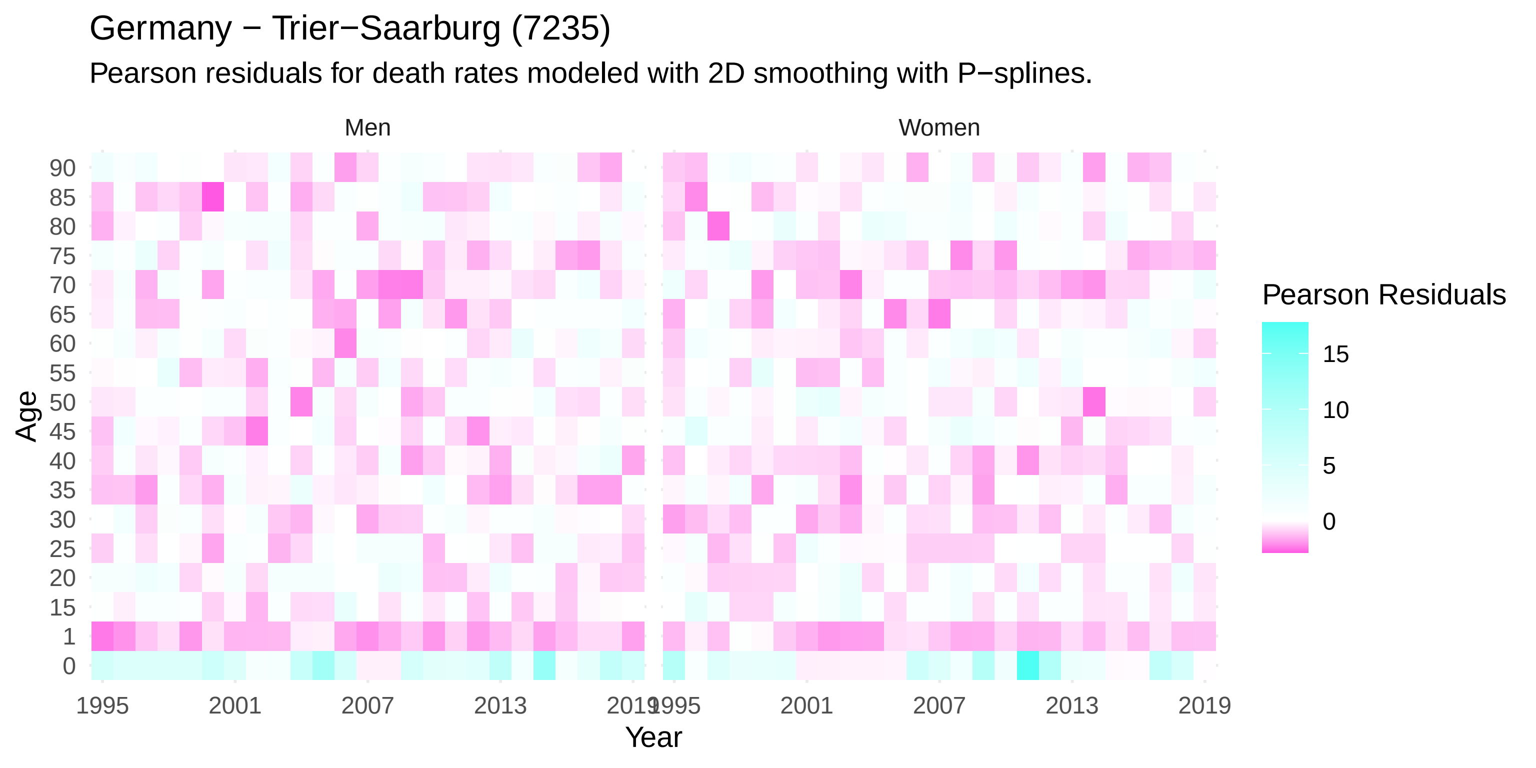
Germany − Trier−Saarburg (7235)
Pearson residuals for death rates modeled with 2D smoothing with P−splines.
Men
Women
Age
90
85
80
75
70
65
60
55
50
45
40
35
30
25
20
15
1
0
1995
2001
2007
2013
2019
Year
Pearson Residuals
15
10
5
0

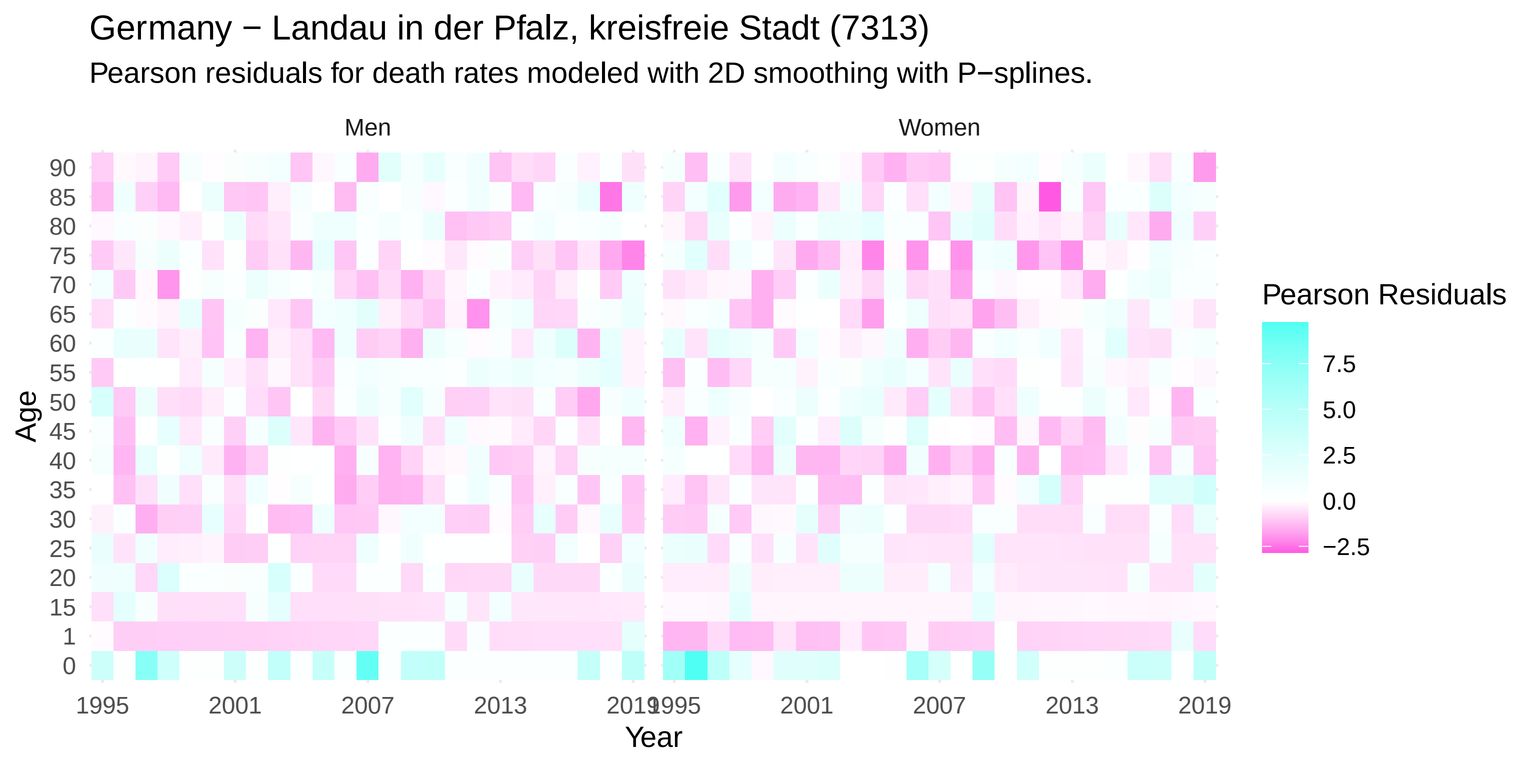
Germany − Landau in der Pfalz, kreisfreie Stadt (7313)
Pearson residuals for death rates modeled with 2D smoothing with P−splines.
Men
Women
Age
90
85
80
75
70
65
60
55
50
45
40
35
30
25
20
15
1
0
1995
2001
2007
2013
2019
Year
Pearson Residuals
7.5
5.0
2.5
0.0
−2.5

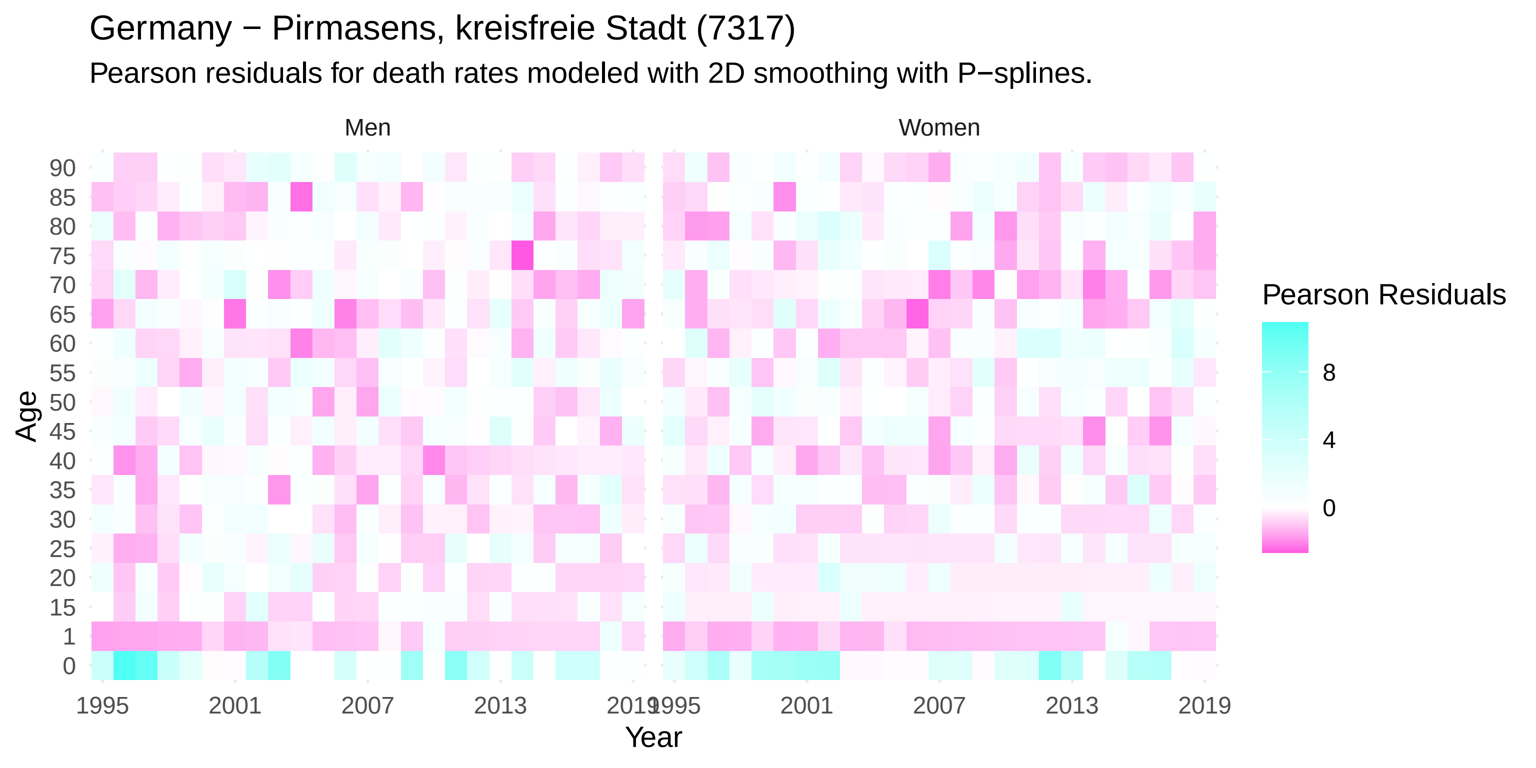
Germany − Pirmasens, kreisfreie Stadt (7317)
Pearson residuals for death rates modeled with 2D smoothing with P−splines.
Men
Women
Age
90
85
80
75
70
65
60
55
50
45
40
35
30
25
20
15
1
0
1995
2001
2007
2013
2019
Year
Pearson Residuals
8
4
0

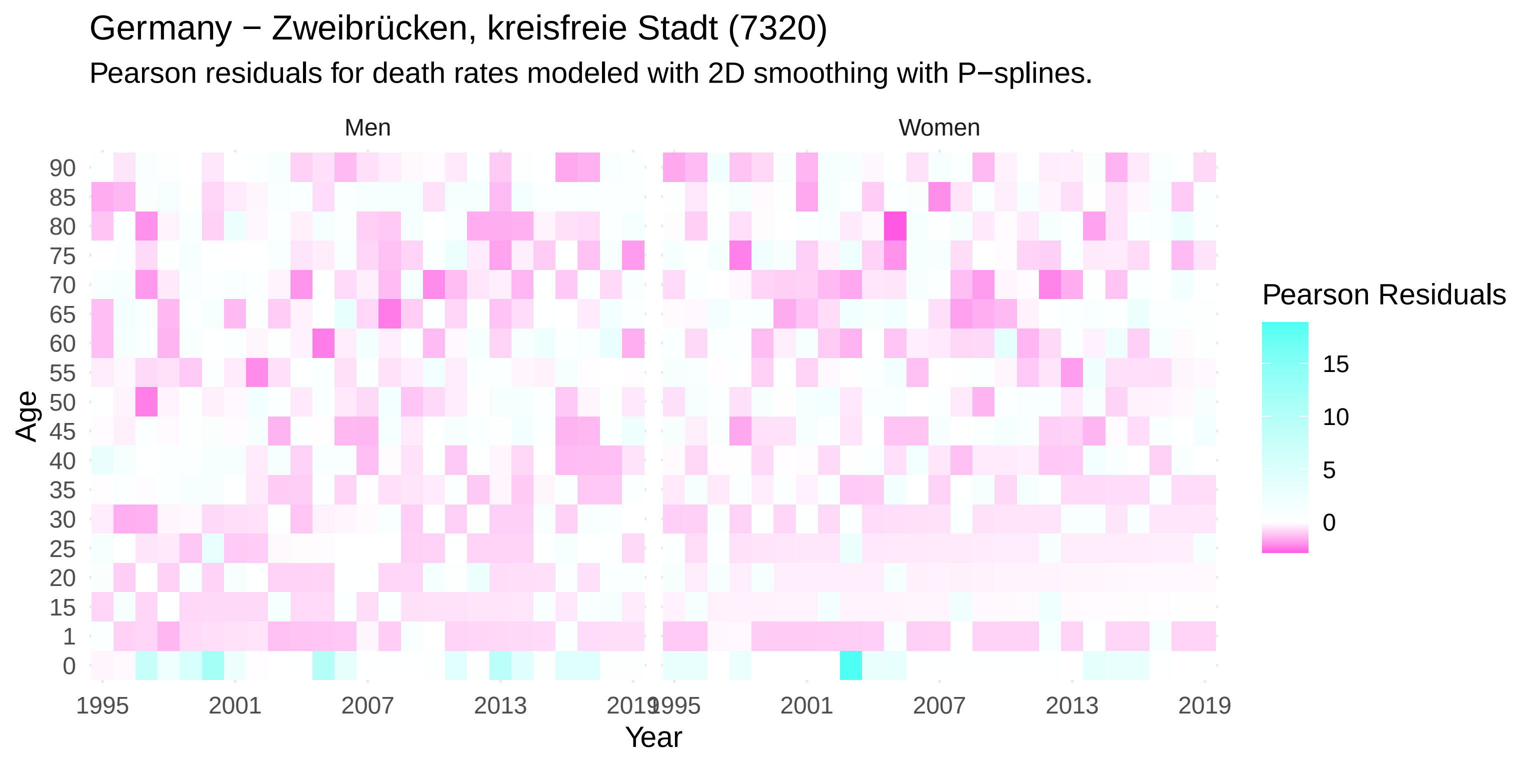

Germany − Zweibrücken, kreisfreie Stadt (7320)
Pearson residuals for death rates modeled with 2D smoothing with P−splines.
Men
Women
Age
90
85
80
75
70
65
60
55
50
45
40
35
30
25
20
15
1
0
1995
2001
2007
2013
2019
Year
Pearson Residuals
15
10
5
0

Germany − Germersheim (7334)

Pearson residuals for death rates modeled with 2D smoothing with P−splines.

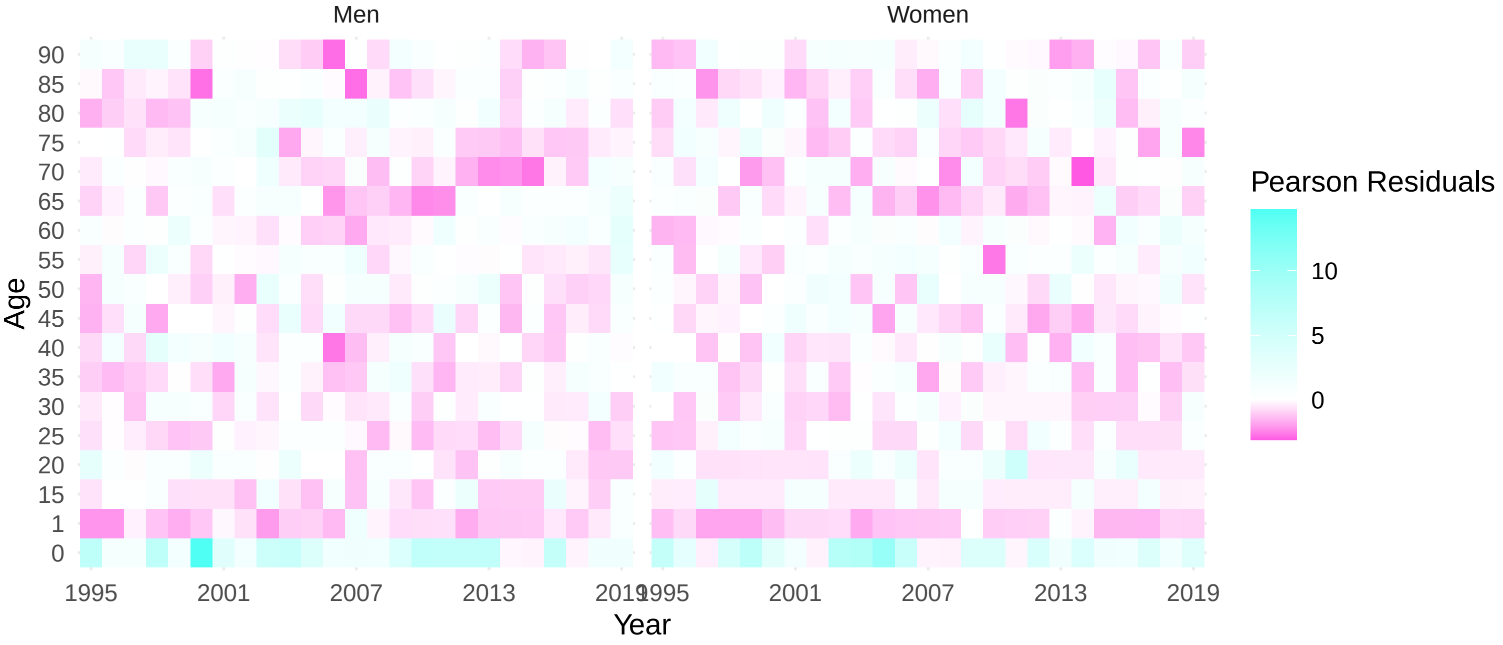

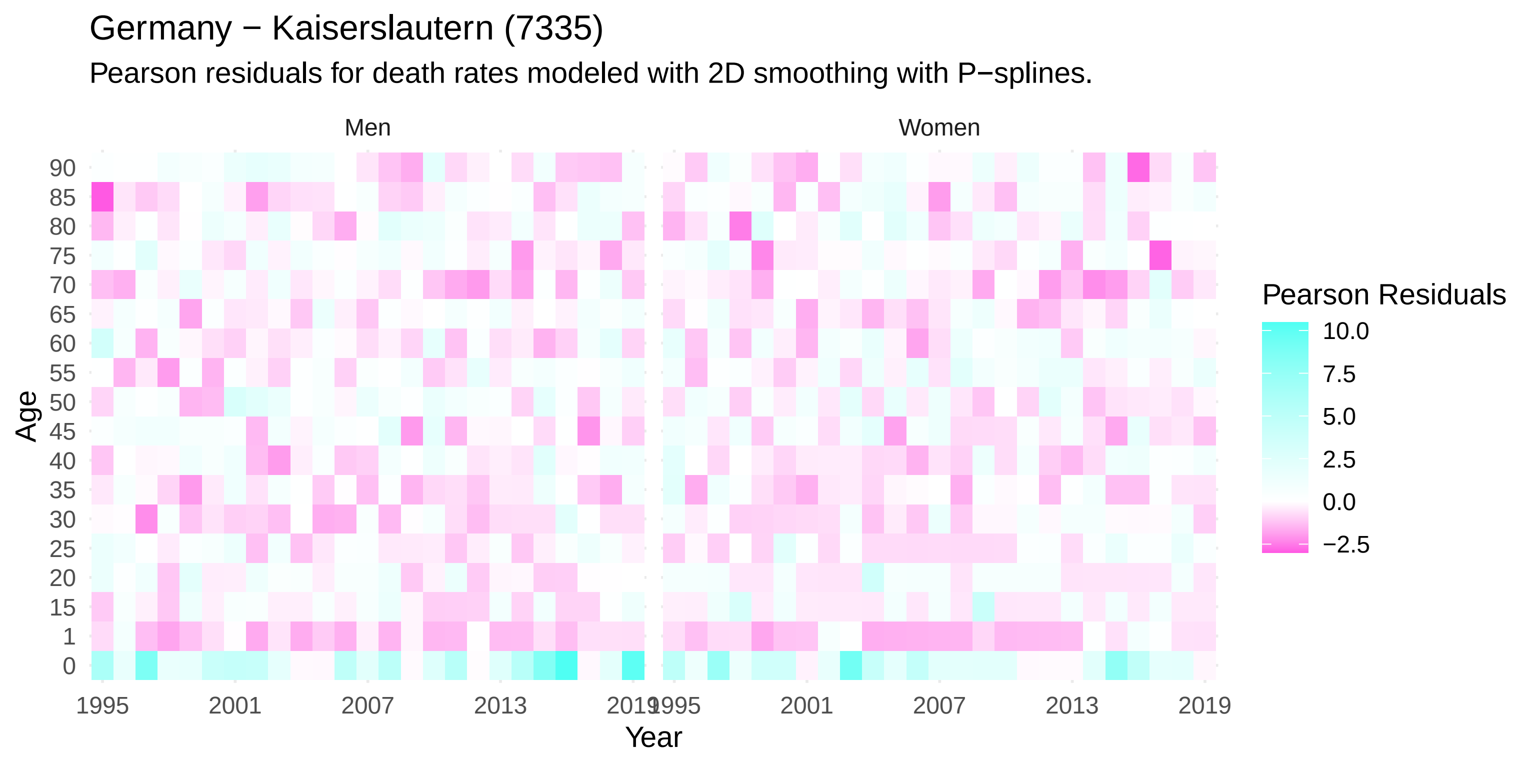
Germany − Kaiserslautern (7335)
Pearson residuals for death rates modeled with 2D smoothing with P−splines.
Men
Women
Age
90
85
80
75
70
65
60
55
50
45
40
35
30
25
20
15
1
0
1995
2001
2007
2013
2019
Year
Pearson Residuals
10.0
7.5
5.0
2.5
0.0
−2.5

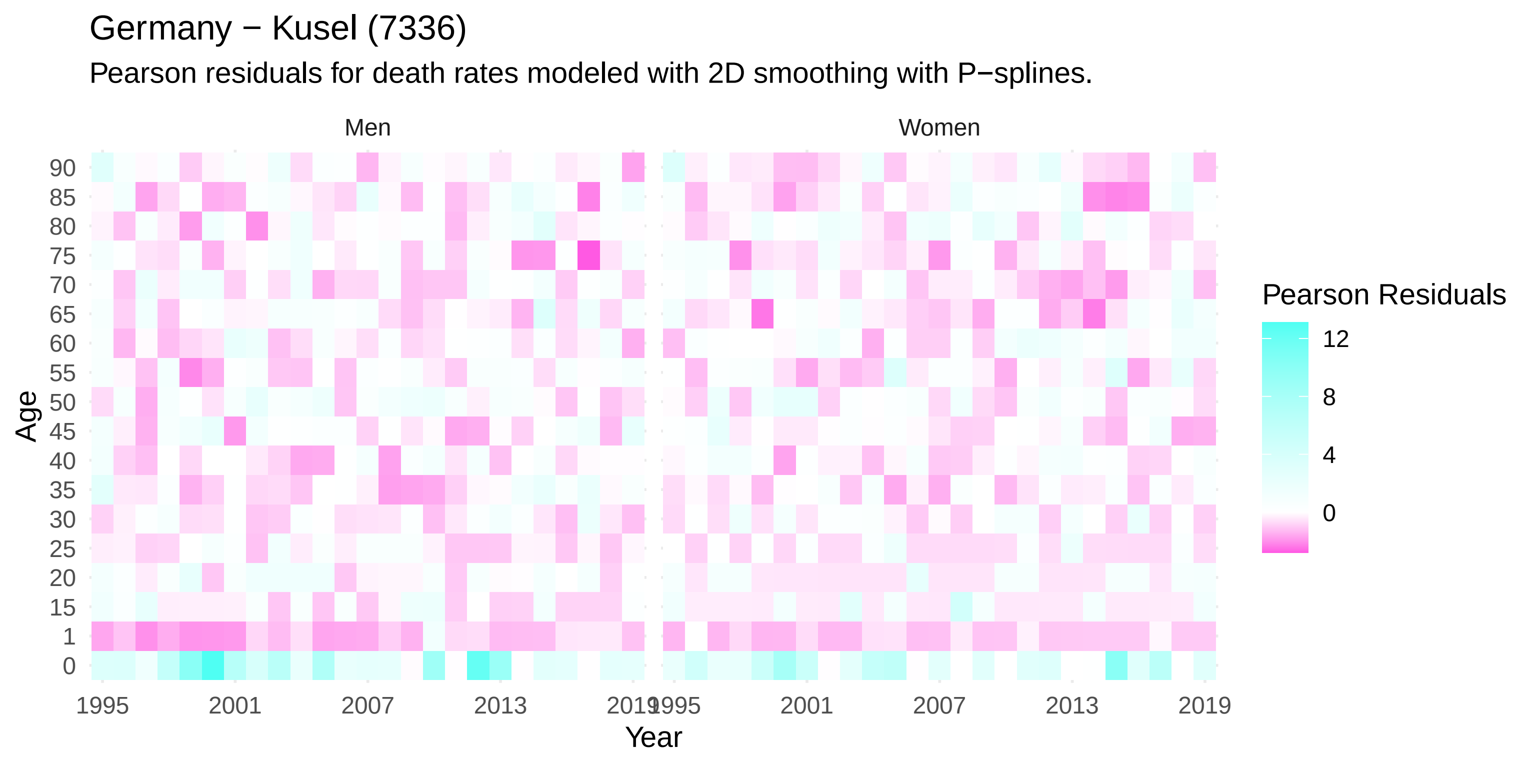

Germany − Kusel (7336)
Pearson residuals for death rates modeled with 2D smoothing with P−splines.
Men
Women
Age
90
85
80
75
70
65
60
55
50
45
40
35
30
25
20
15
1
0
1995
2001
2007
2013
2019
Year
Pearson Residuals
12
8
4
0

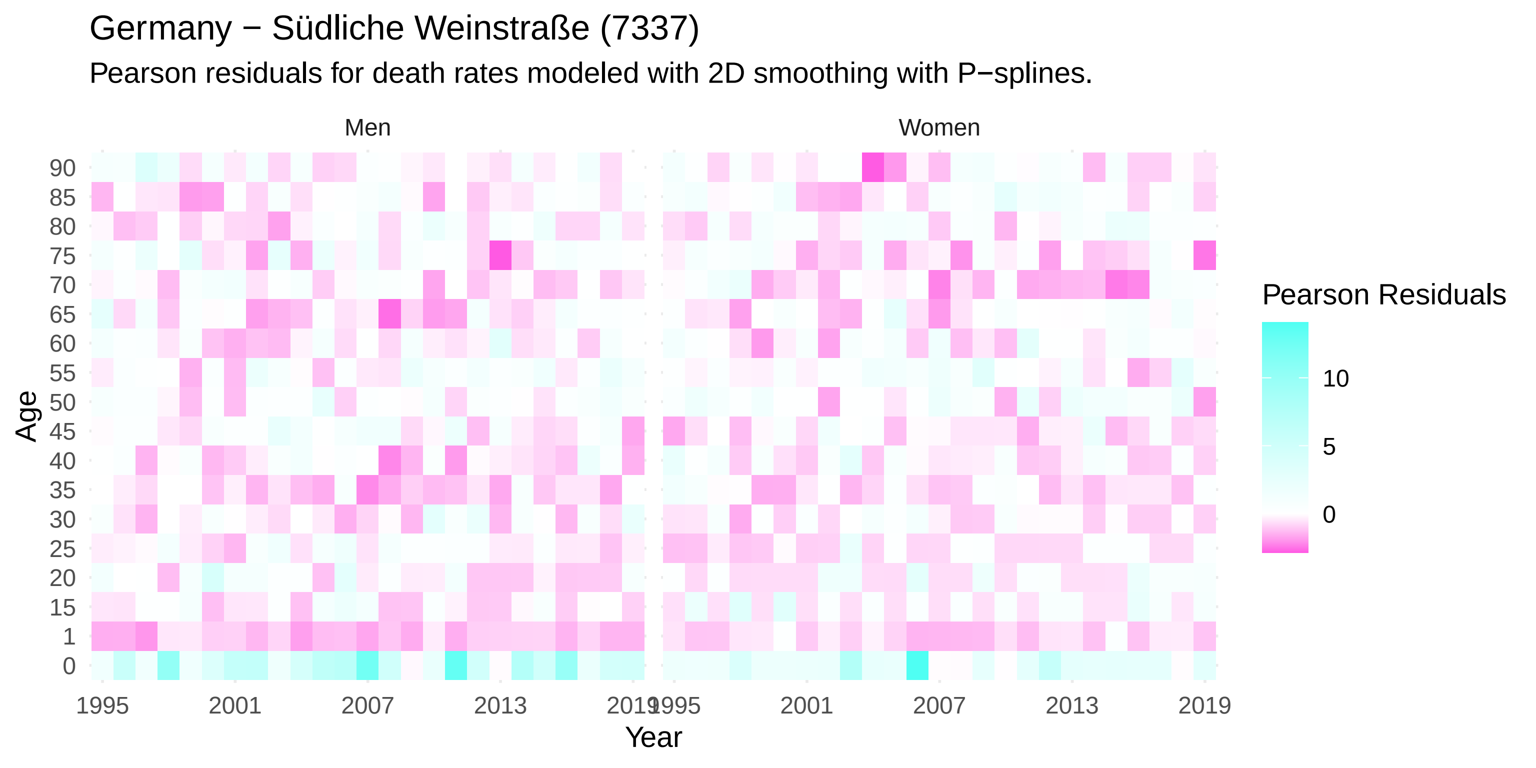

Germany − Südliche Weinstraße (7337)
Pearson residuals for death rates modeled with 2D smoothing with P−splines.
Men
Women
Age
90
85
80
75
70
65
60
55
50
45
40
35
30
25
20
15
1
0
1995
2001
2007
2013
2019
Year
Pearson Residuals
10
5
0

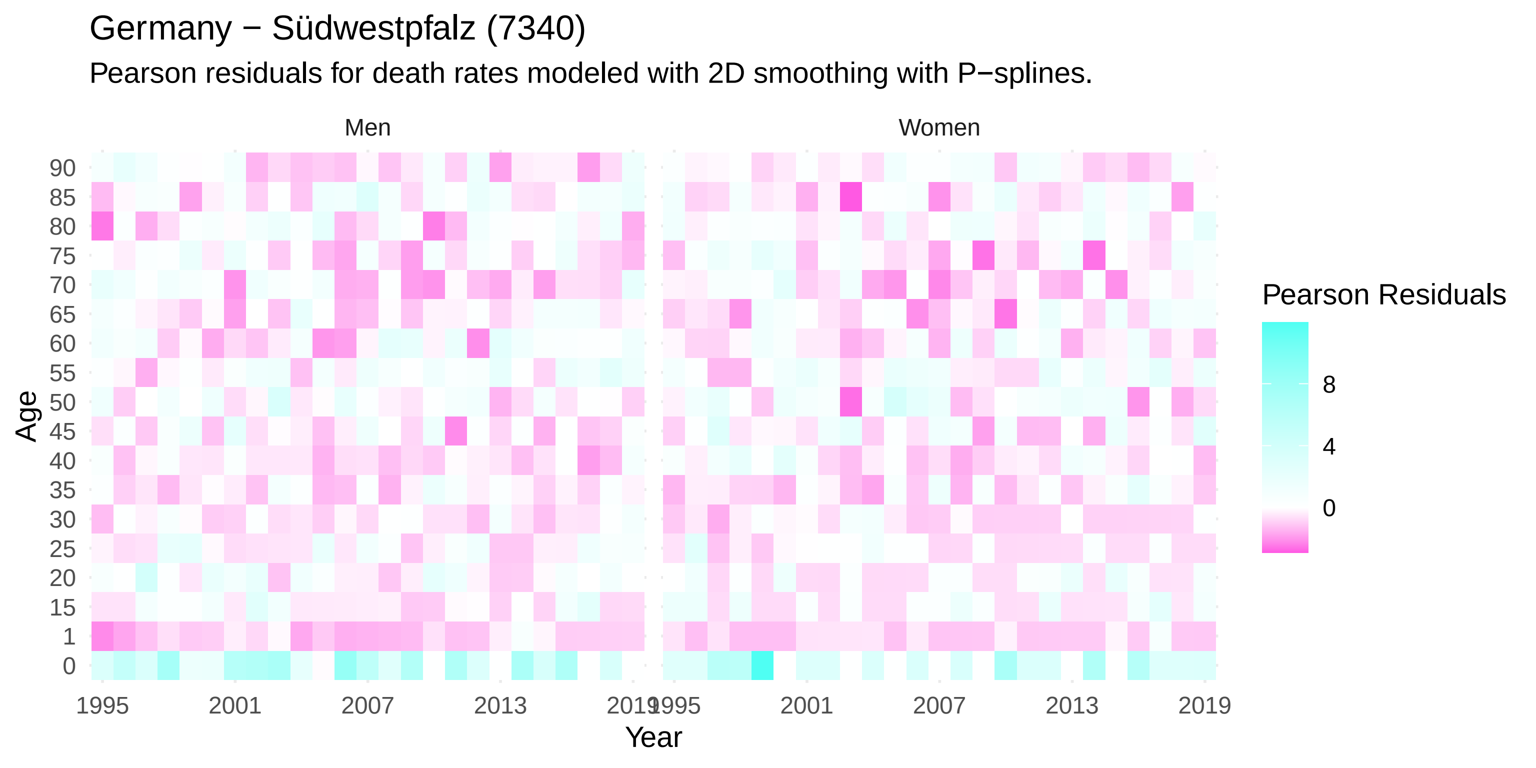

Germany − Südwestpfalz (7340)
Pearson residuals for death rates modeled with 2D smoothing with P−splines.
Men
Women
Age
90
85
80
75
70
65
60
55
50
45
40
35
30
25
20
15
1
0
1995
2001
2007
2013
2019
Year
Pearson Residuals
8
4
0

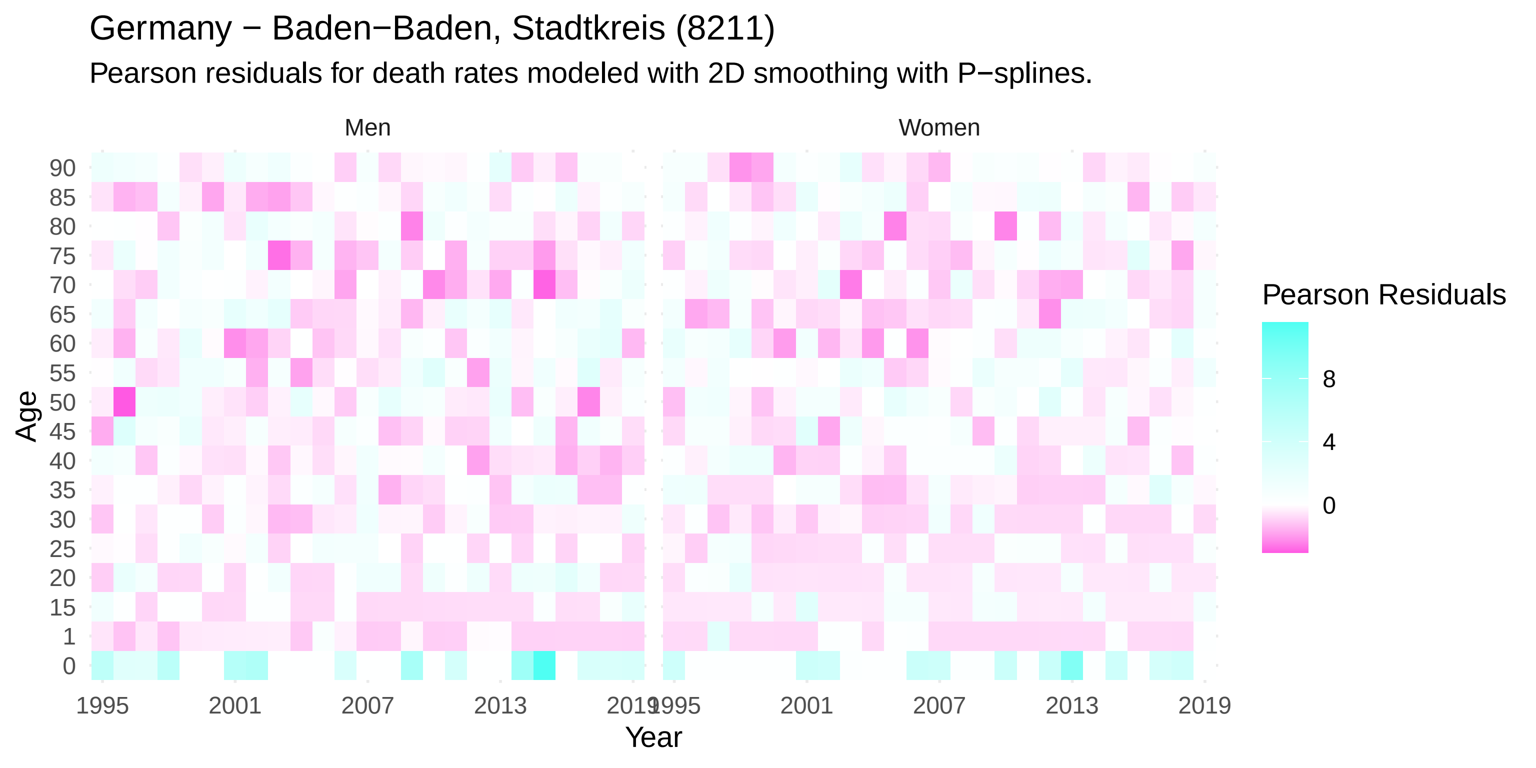

Germany − Baden−Baden, Stadtkreis (8211)
Pearson residuals for death rates modeled with 2D smoothing with P−splines.
Men
Women
Age
90
85
80
75
70
65
60
55
50
45
40
35
30
25
20
15
1
0
1995
2001
2007
2013
2019
Year
Pearson Residuals
8
4
0

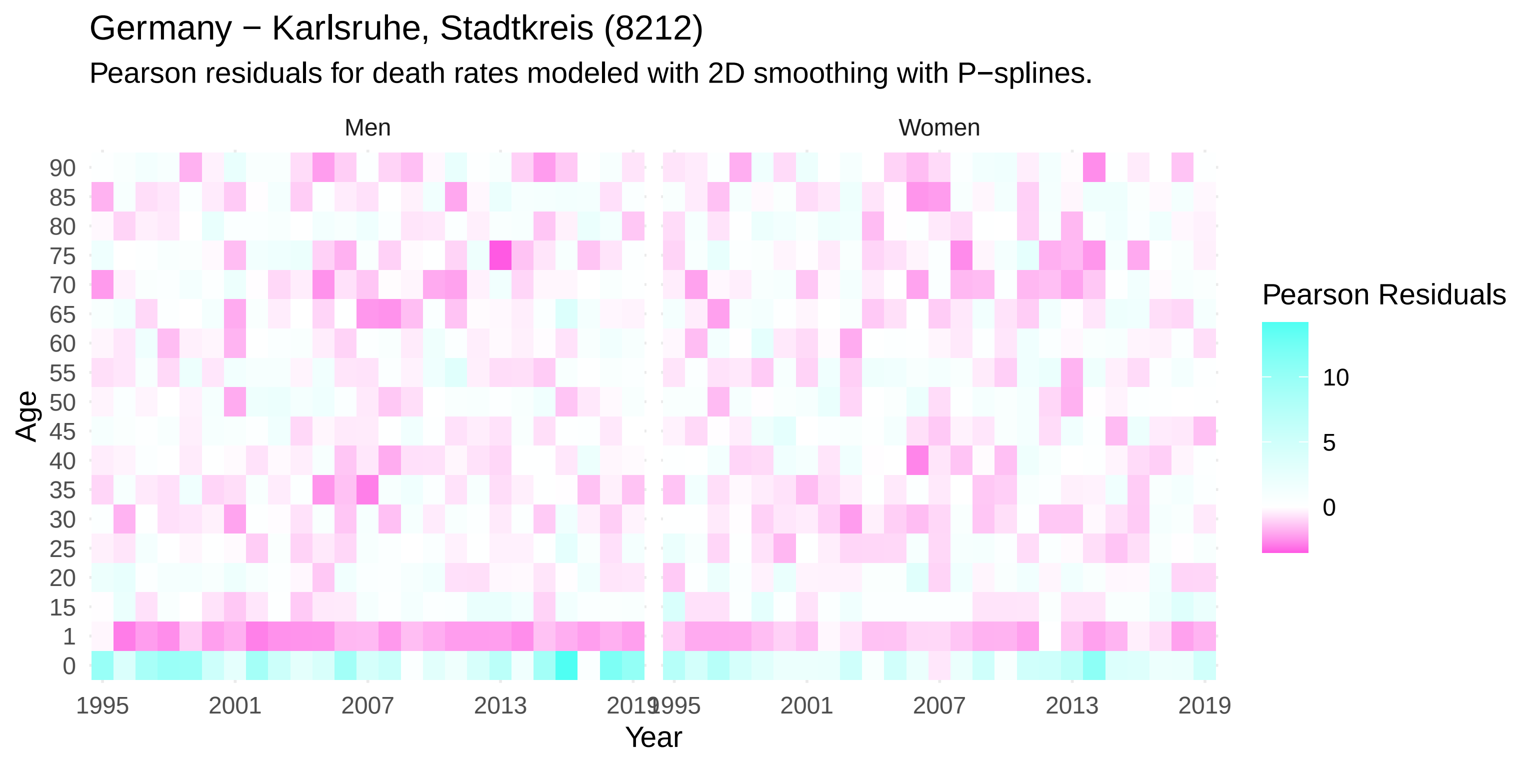

Germany − Karlsruhe, Stadtkreis (8212)
Pearson residuals for death rates modeled with 2D smoothing with P−splines.
Men
Women
Age
90
85
80
75
70
65
60
55
50
45
40
35
30
25
20
15
1
0
1995
2001
2007
2013
2019
Year
Pearson Residuals
10
5
0

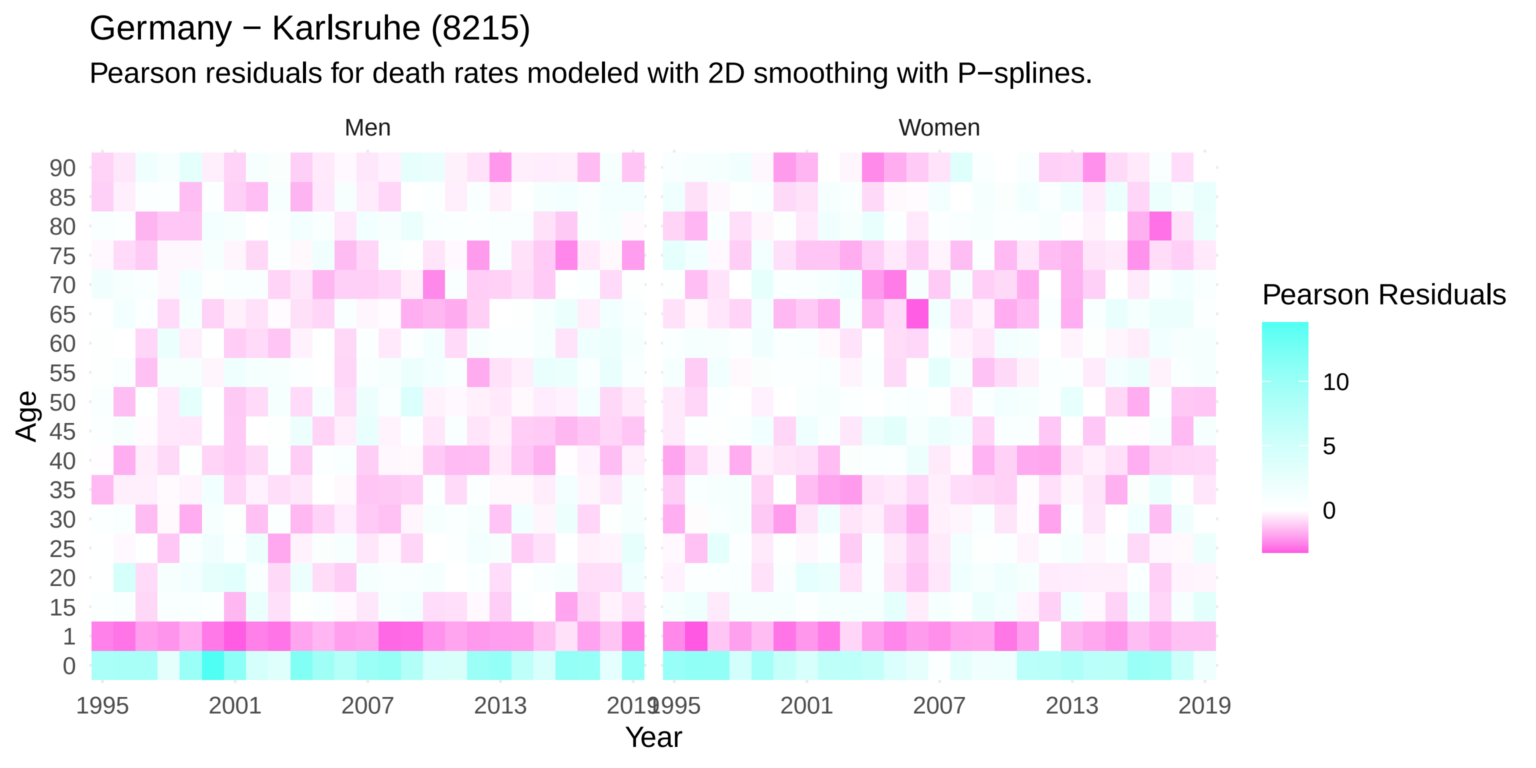
Germany − Karlsruhe (8215)
Pearson residuals for death rates modeled with 2D smoothing with P−splines.
Men
Women
Age
90
85
80
75
70
65
60
55
50
45
40
35
30
25
20
15
1
0
1995
2001
2007
2013
2019
Year
Pearson Residuals
10
5
0

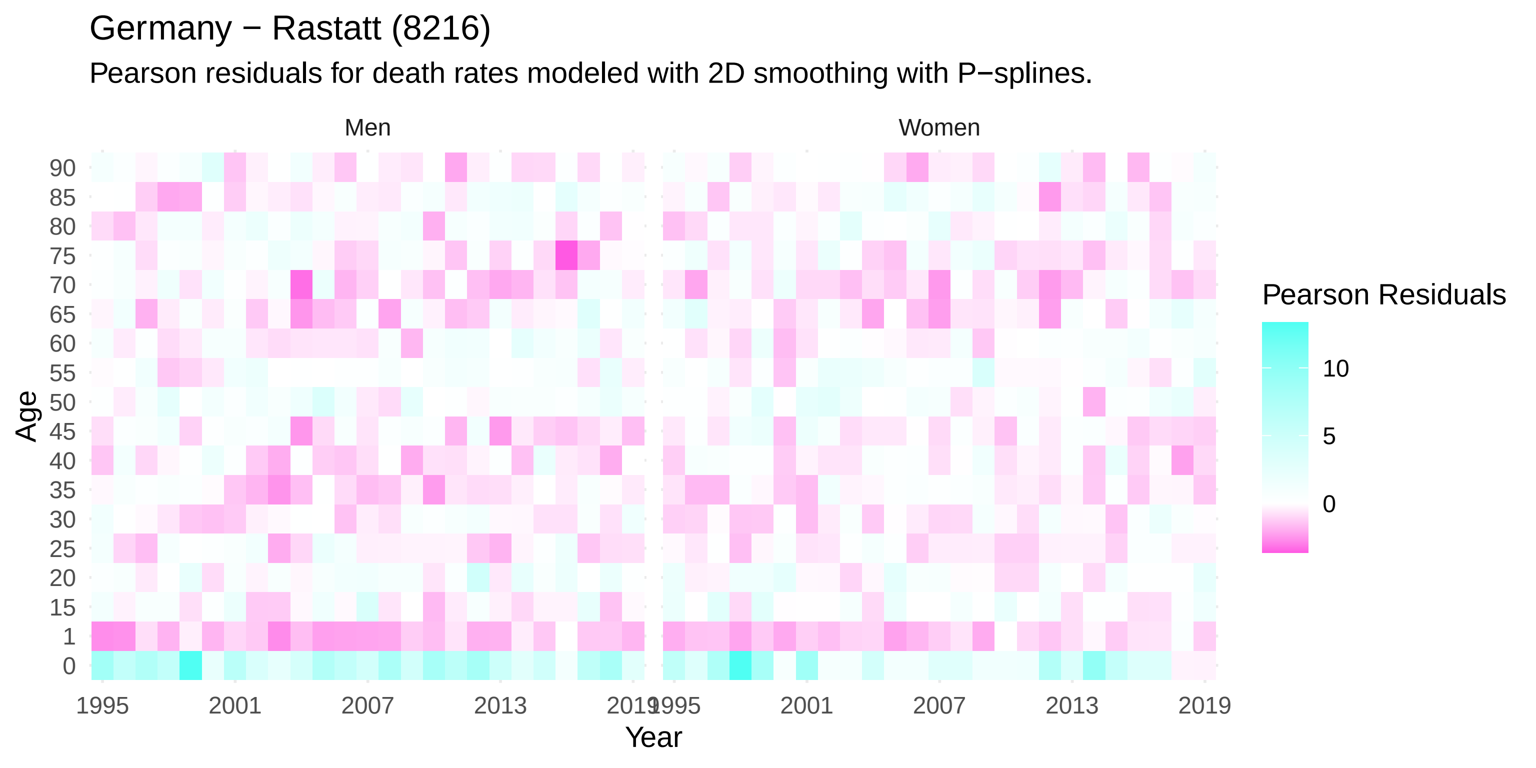

Germany − Rastatt (8216)
Pearson residuals for death rates modeled with 2D smoothing with P−splines.
Men
Women
Age
90
85
80
75
70
65
60
55
50
45
40
35
30
25
20
15
1
0
1995
2001
2007
2013
2019
Year
Pearson Residuals
10
5
0

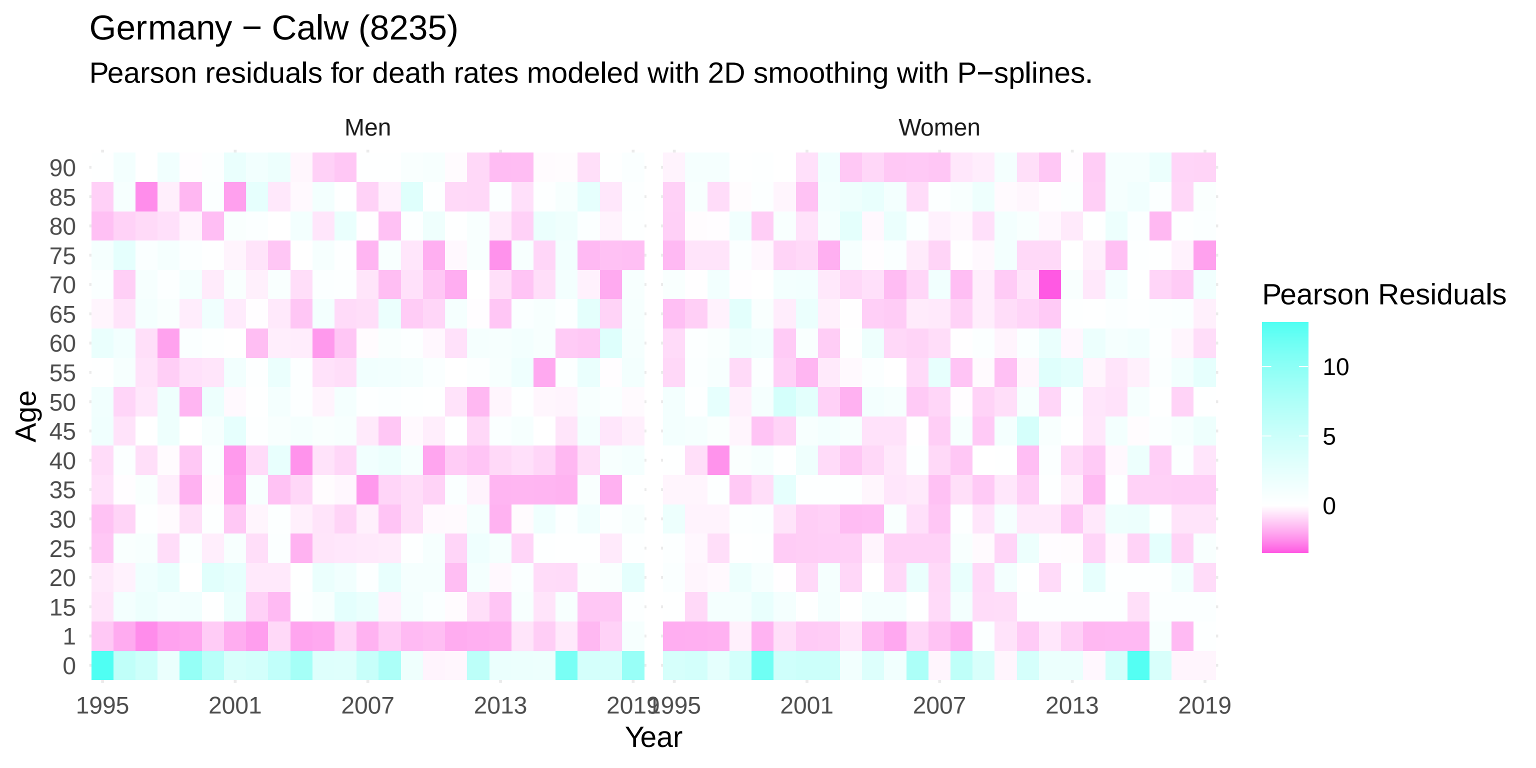

Germany − Calw (8235)
Pearson residuals for death rates modeled with 2D smoothing with P−splines.
Men
Women
Age
90
85
80
75
70
65
60
55
50
45
40
35
30
25
20
15
1
0
1995
2001
2007
2013
2019
Year
Pearson Residuals
10
5
0

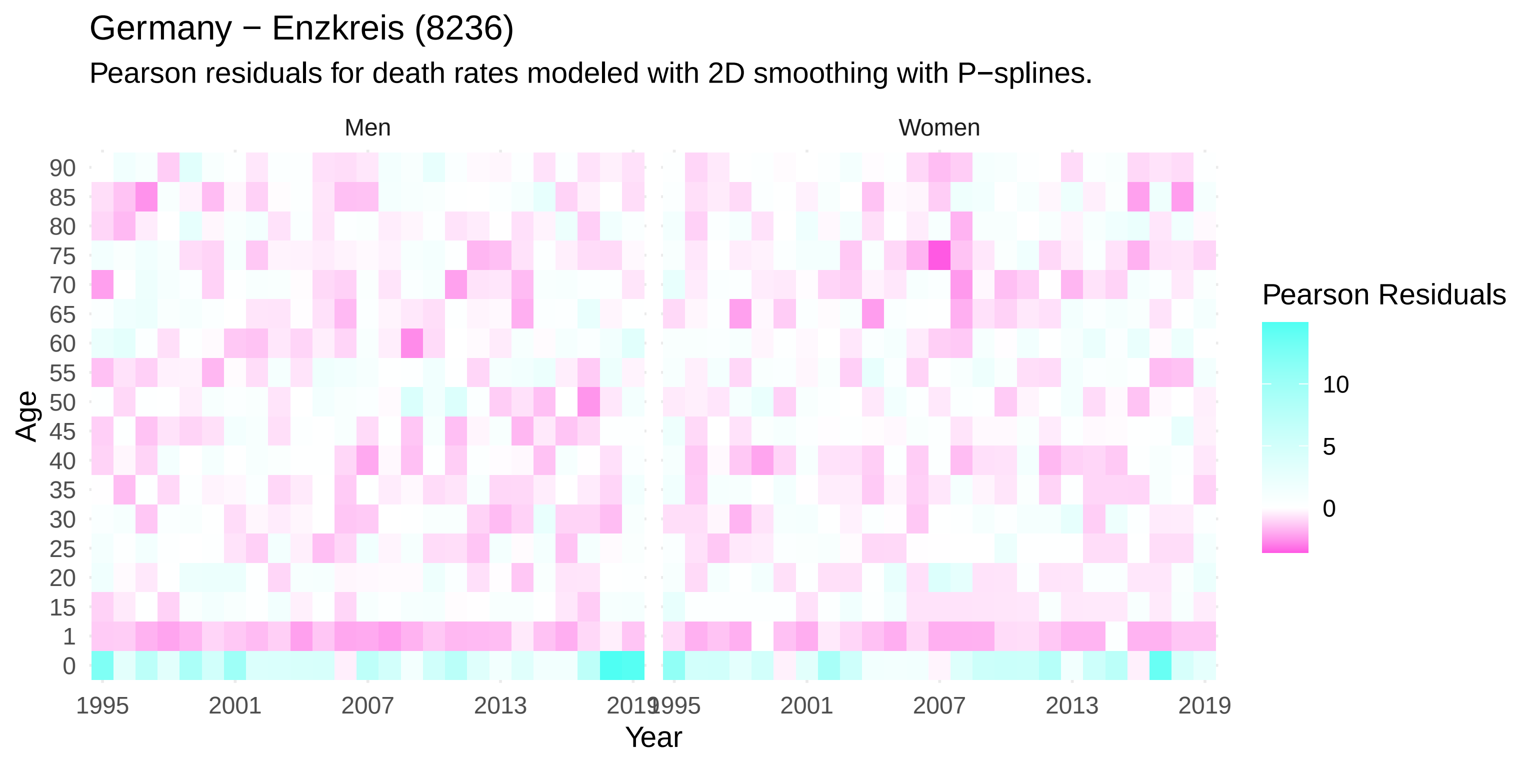
Germany − Enzkreis (8236)
Pearson residuals for death rates modeled with 2D smoothing with P−splines.
Men
Women
Age
90
85
80
75
70
65
60
55
50
45
40
35
30
25
20
15
1
0
1995
2001
2007
2013
2019
Year
Pearson Residuals
10
5
0

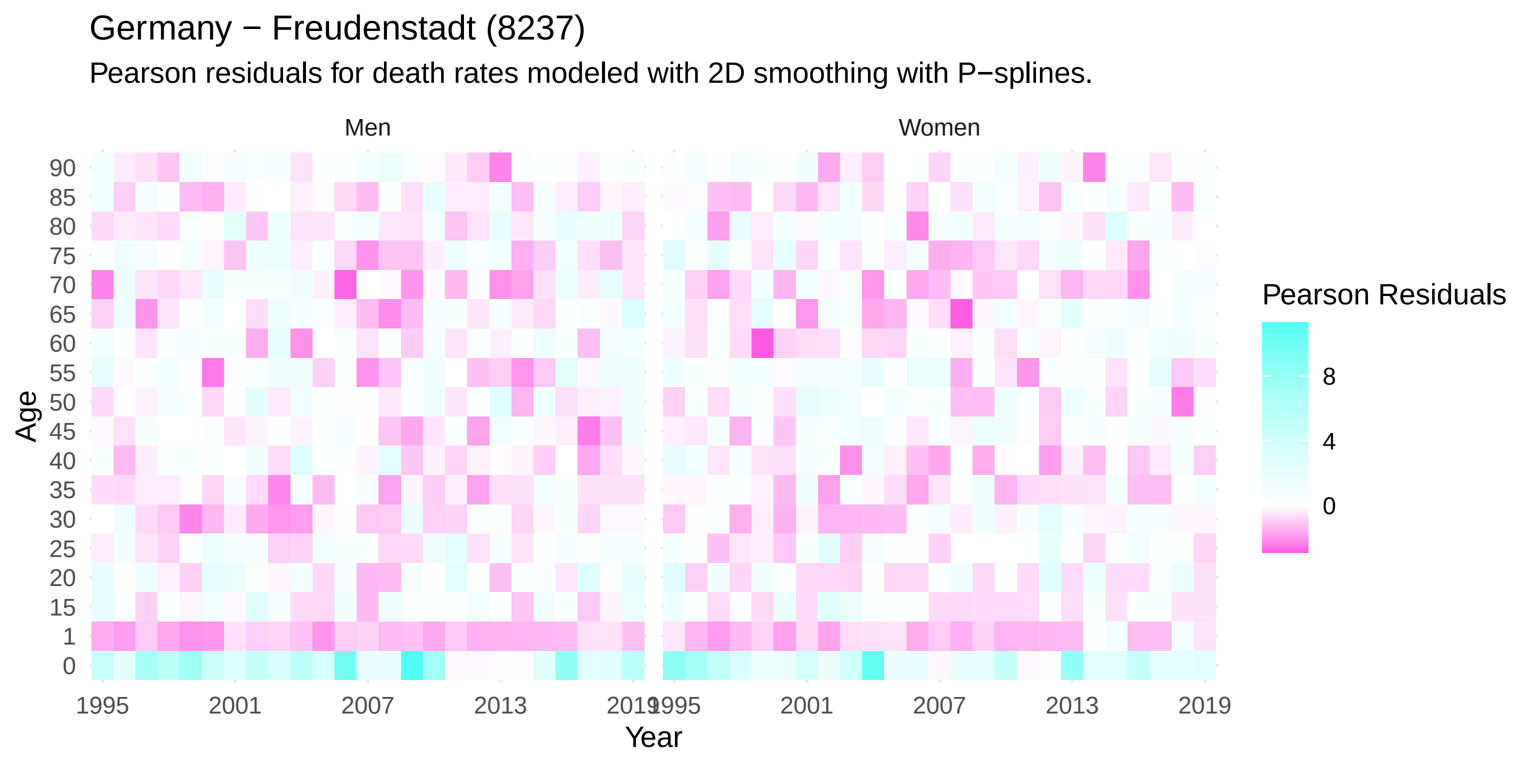
Germany − Freudenstadt (8237)
Pearson residuals for death rates modeled with 2D smoothing with P−splines.
Men
Women
Age
90
85
80
75
70
65
60
55
50
45
40
35
30
25
20
15
1
0
1995
2001
2007
2013
2019
Year
Pearson Residuals
8
4
0

Germany − Freiburg im Breisgau, Stadtkreis (8311)

Pearson residuals for death rates modeled with 2D smoothing with P−splines.

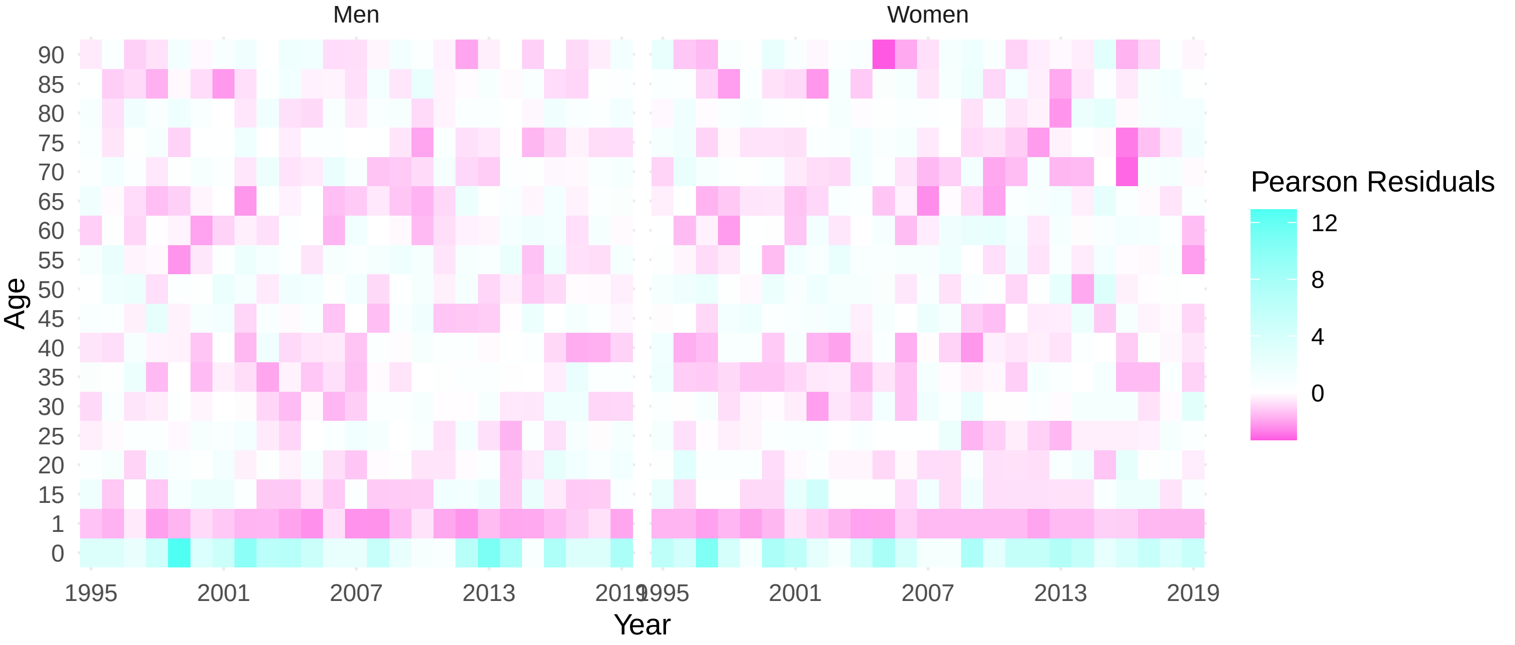

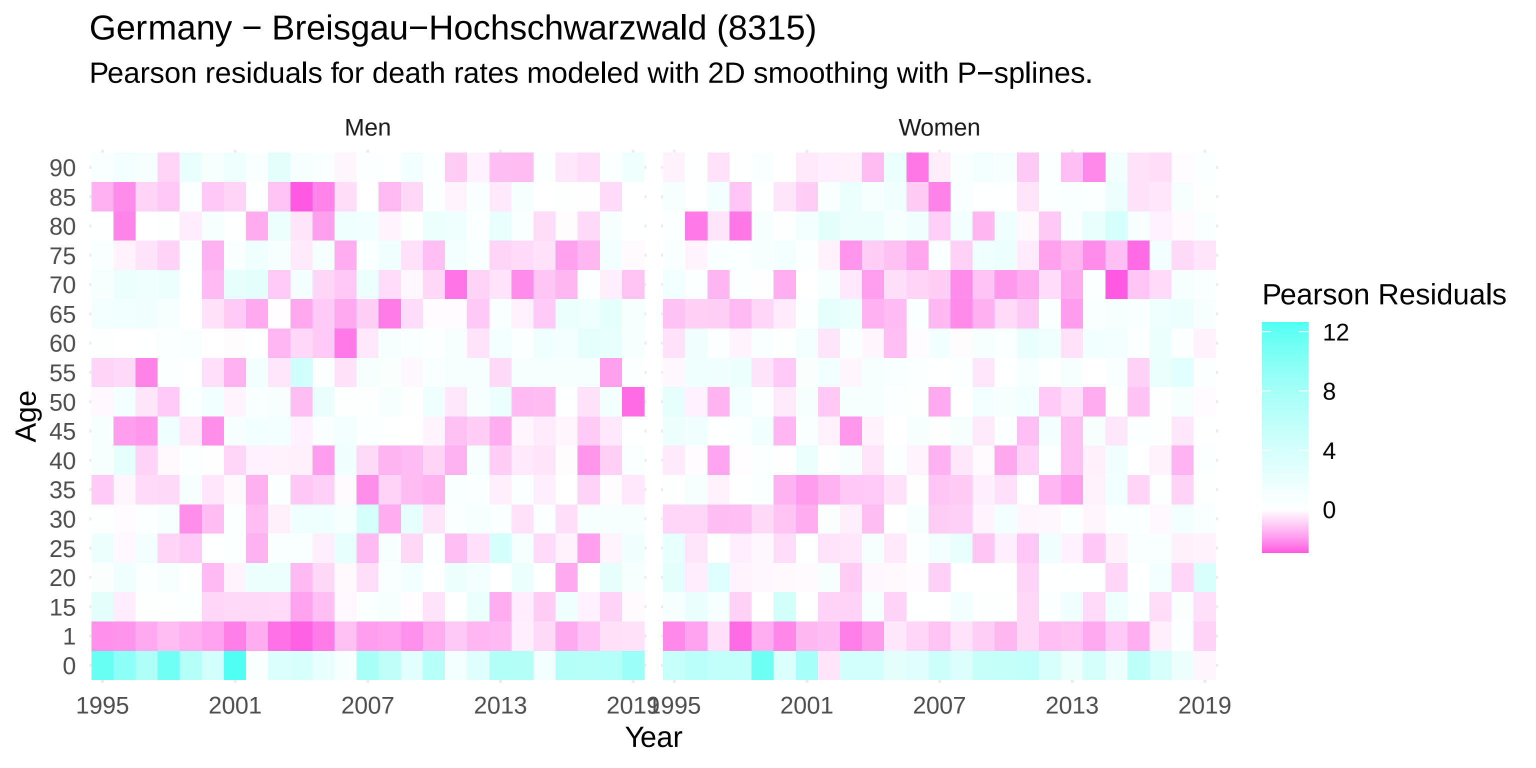
Germany − Breisgau−Hochschwarzwald (8315)
Pearson residuals for death rates modeled with 2D smoothing with P−splines.
Men
Women
Age
90
85
80
75
70
65
60
55
50
45
40
35
30
25
20
15
1
0
1995
2001
2007
2013
2019
Year
Pearson Residuals
12
8
4
0

Germany − Emmendingen (8316)

Pearson residuals for death rates modeled with 2D smoothing with P−splines.

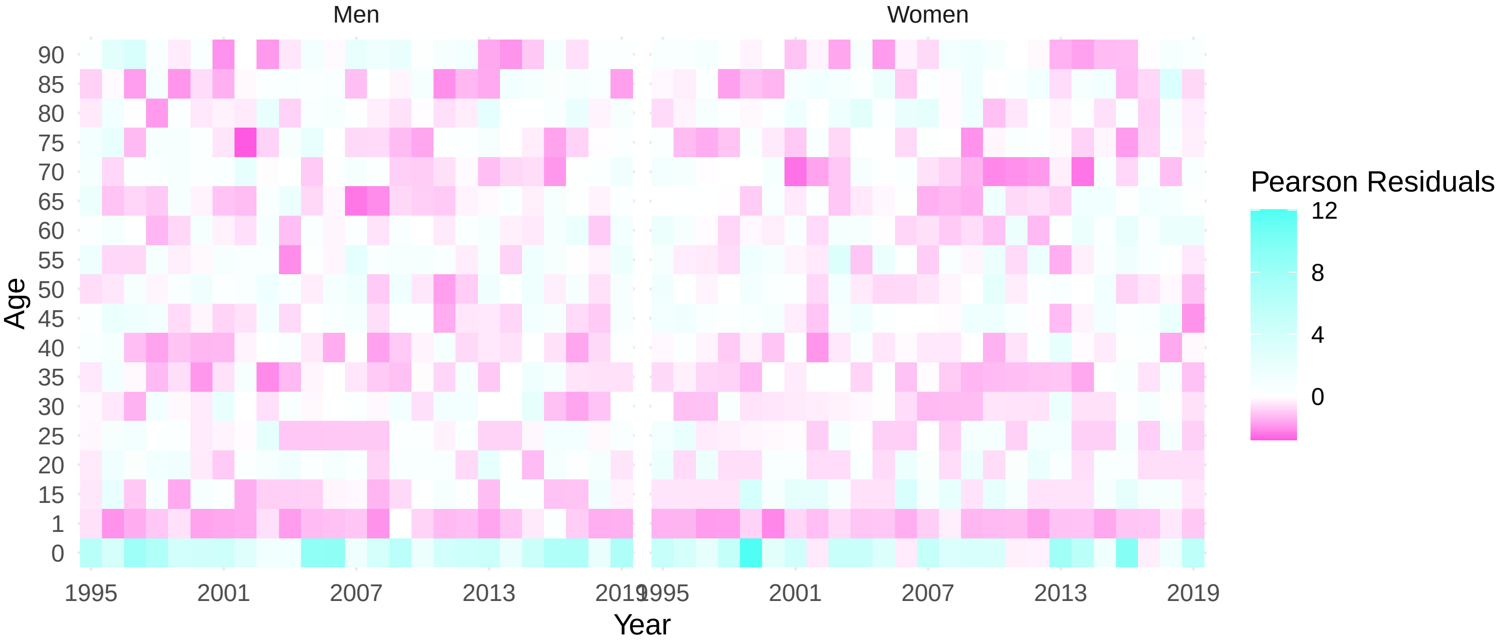

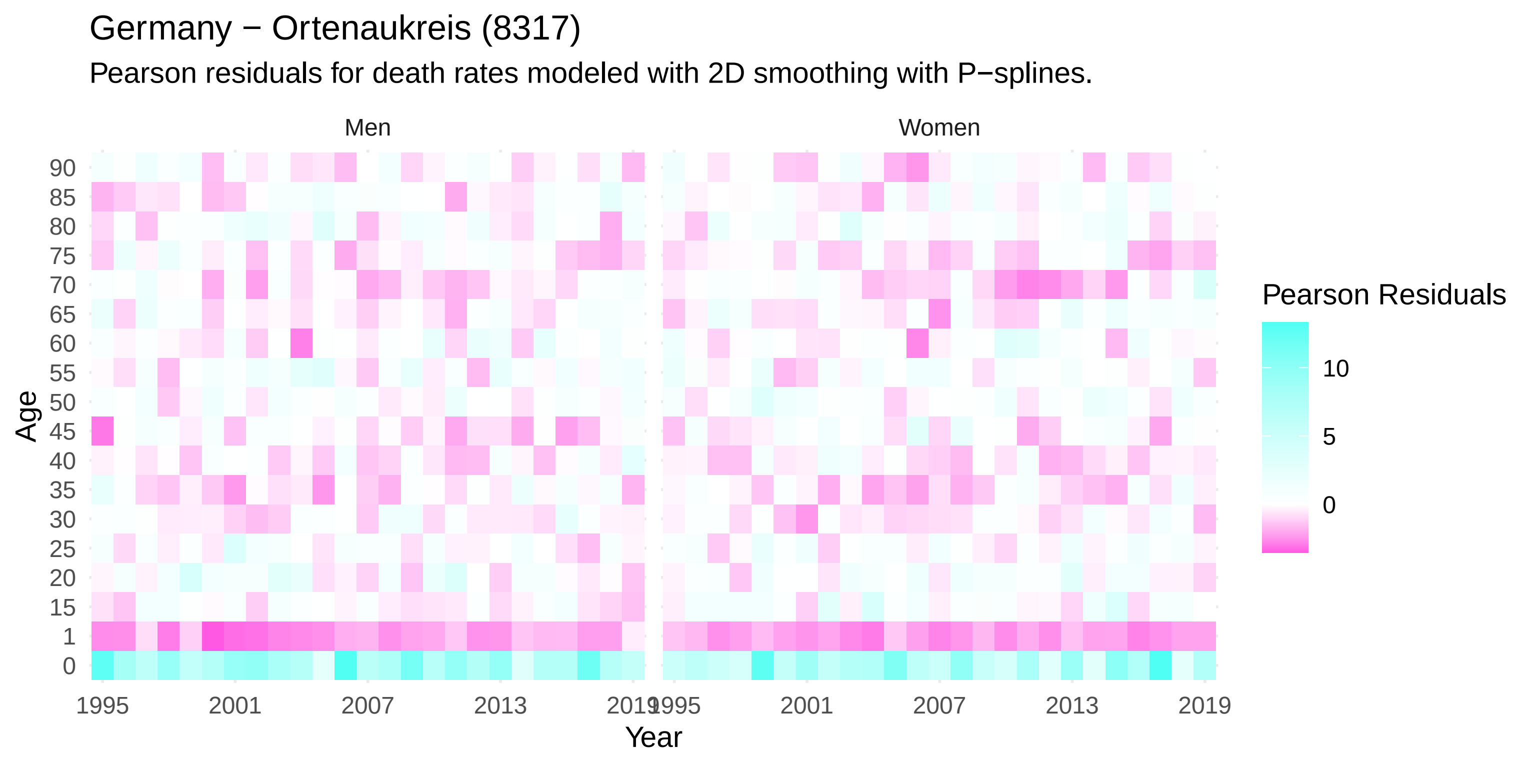

Germany − Ortenaukreis (8317)
Pearson residuals for death rates modeled with 2D smoothing with P−splines.
Men
Women
Age
90
85
80
75
70
65
60
55
50
45
40
35
30
25
20
15
1
0
1995
2001
2007
2013
2019
Year
Pearson Residuals
10
5
0

Germany − Schwarzwald−Baar−Kreis (8326)

Pearson residuals for death rates modeled with 2D smoothing with P−splines.

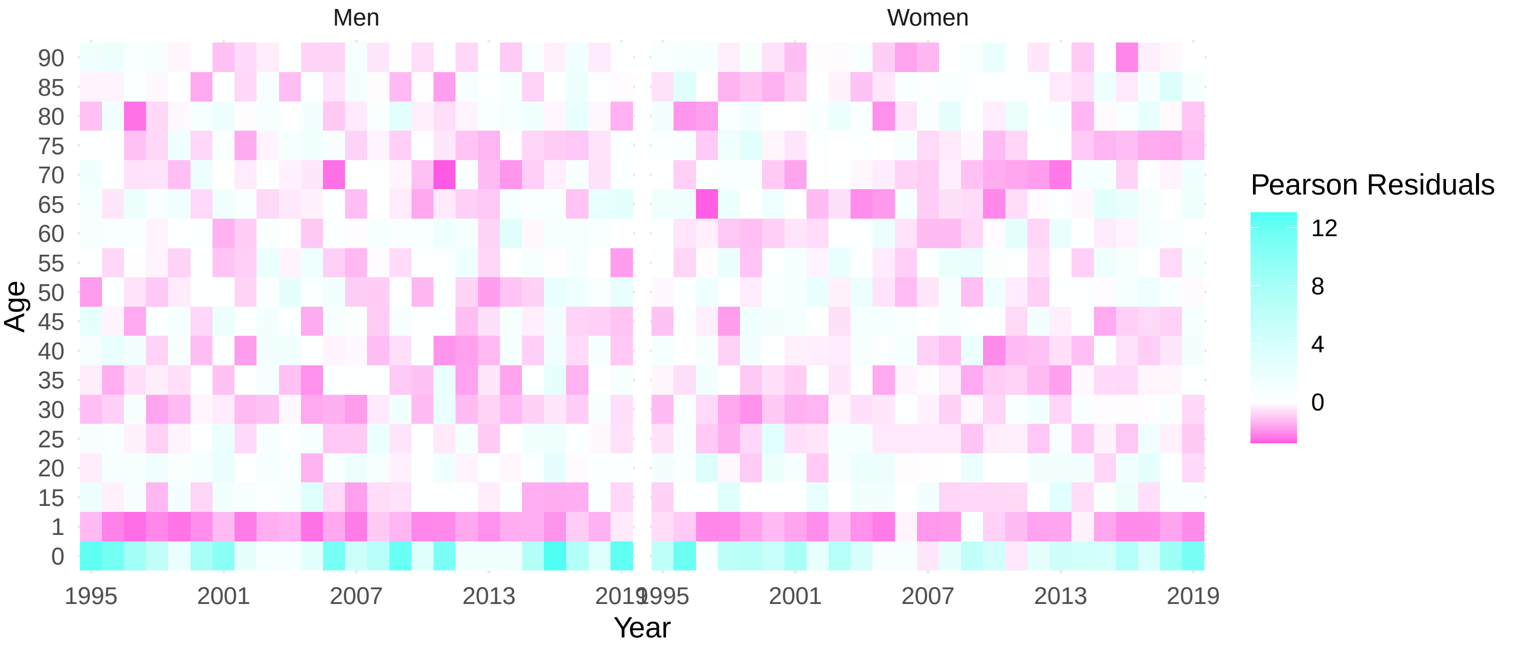

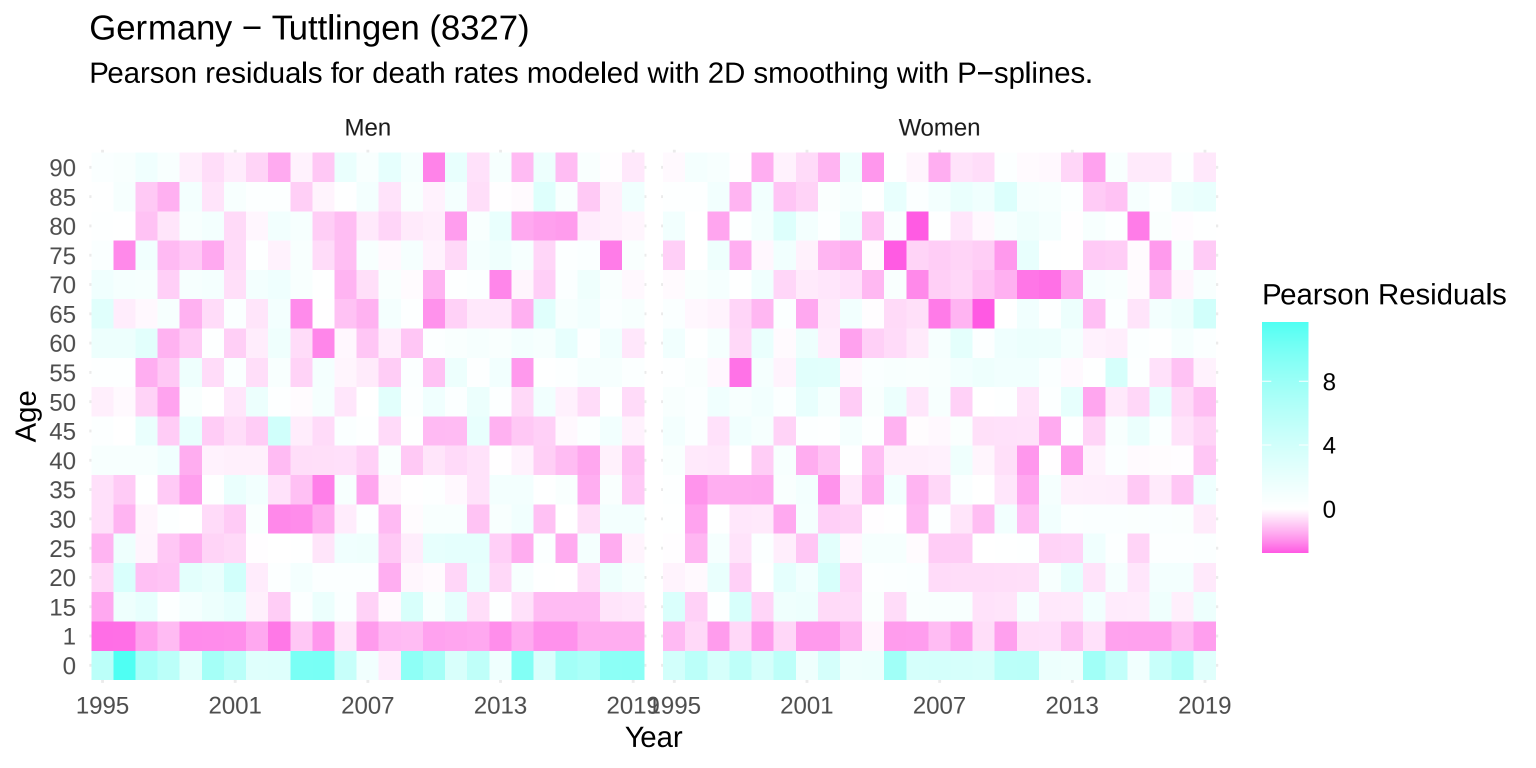
Germany − Tuttlingen (8327)
Pearson residuals for death rates modeled with 2D smoothing with P−splines.
Men
Women
Age
90
85
80
75
70
65
60
55
50
45
40
35
30
25
20
15
1
0
1995
2001
2007
2013
2019
Year
Pearson Residuals
8
4
0

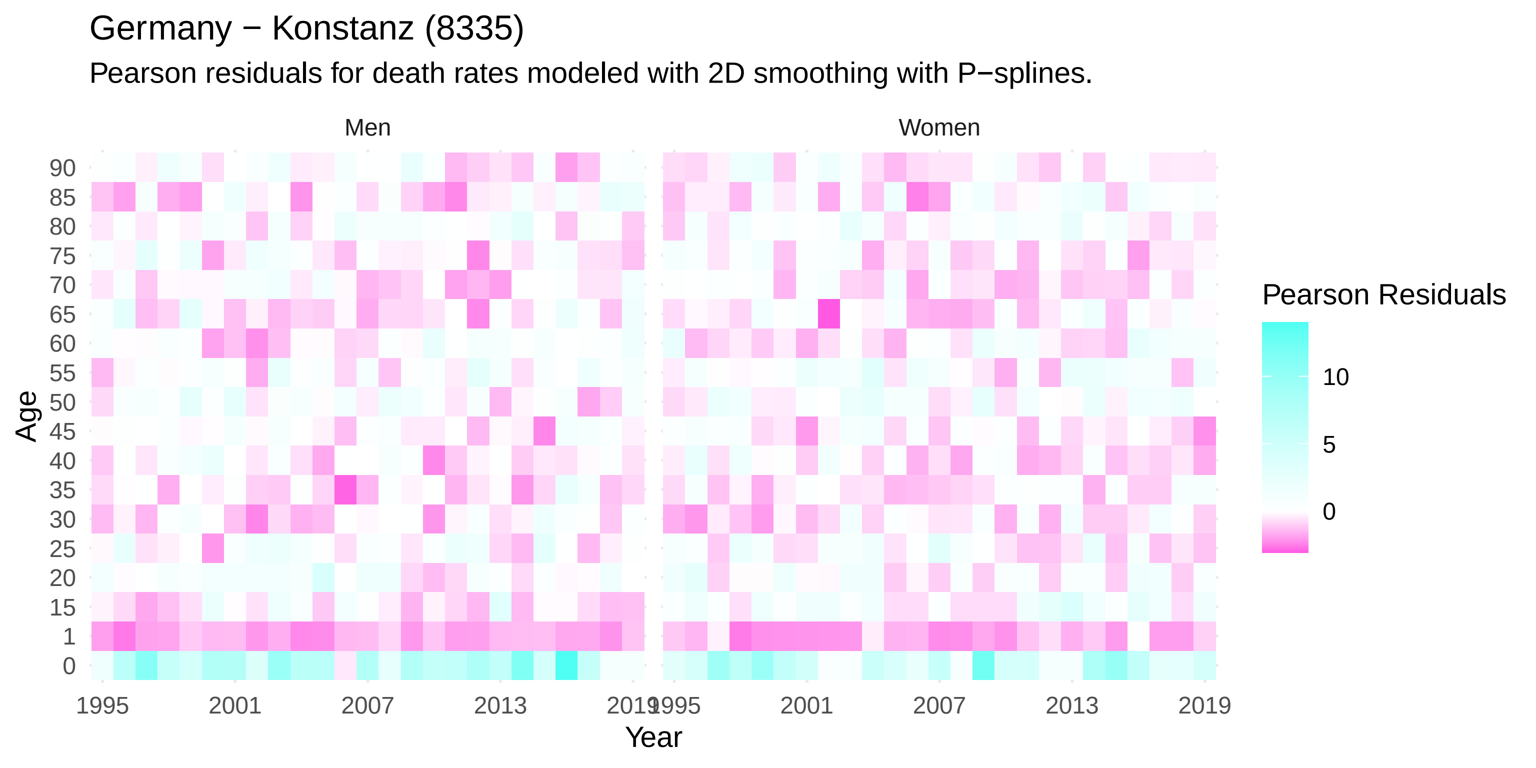

Germany − Konstanz (8335)
Pearson residuals for death rates modeled with 2D smoothing with P−splines.
Men
Women
Age
90
85
80
75
70
65
60
55
50
45
40
35
30
25
20
15
1
0
1995
2001
2007
2013
2019
Year
Pearson Residuals
10
5
0

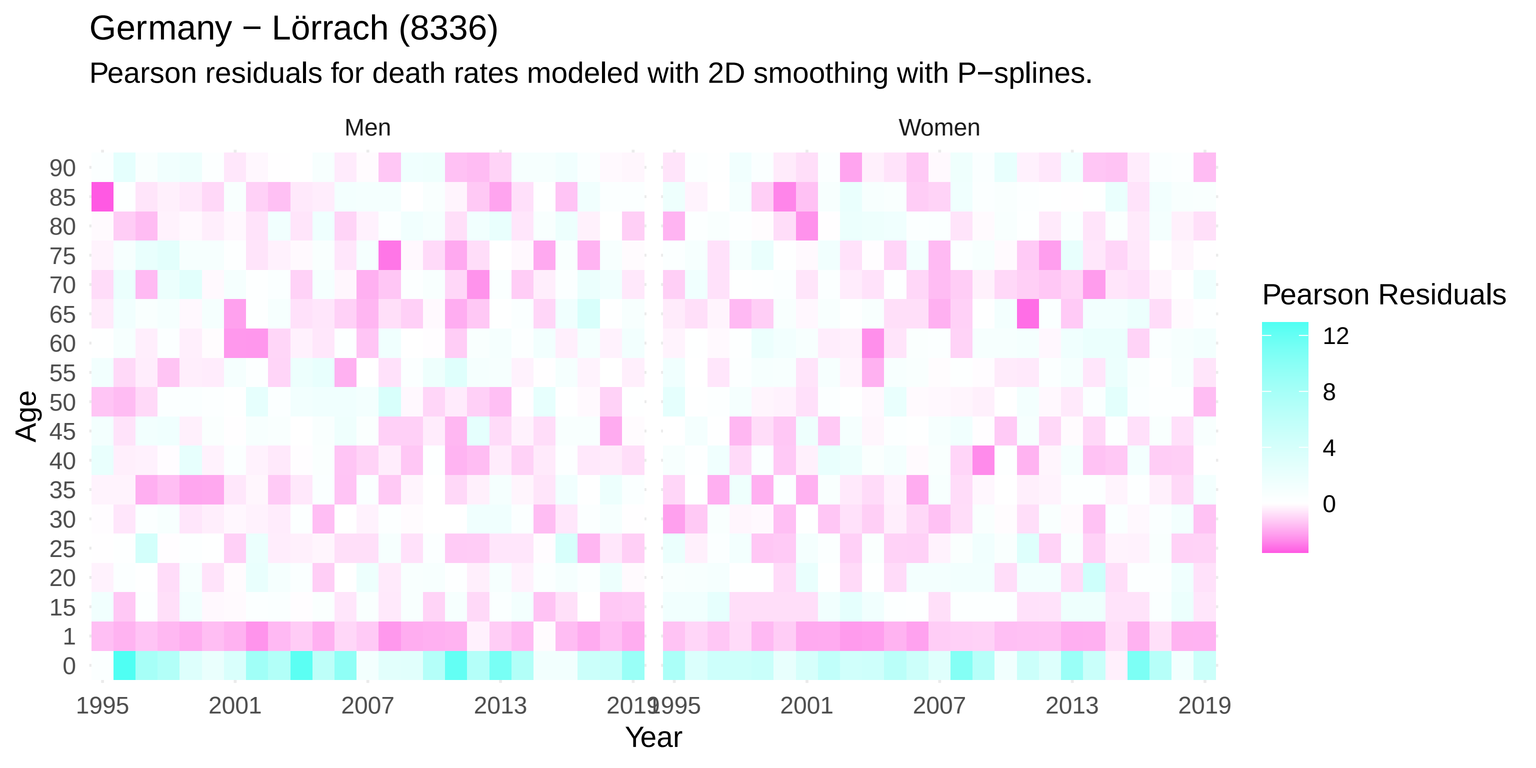

Germany − Lörrach (8336)
Pearson residuals for death rates modeled with 2D smoothing with P−splines.
Men
Women
Age
90
85
80
75
70
65
60
55
50
45
40
35
30
25
20
15
1
0
1995
2001
2007
2013
2019
Year
Pearson Residuals
12
8
4
0

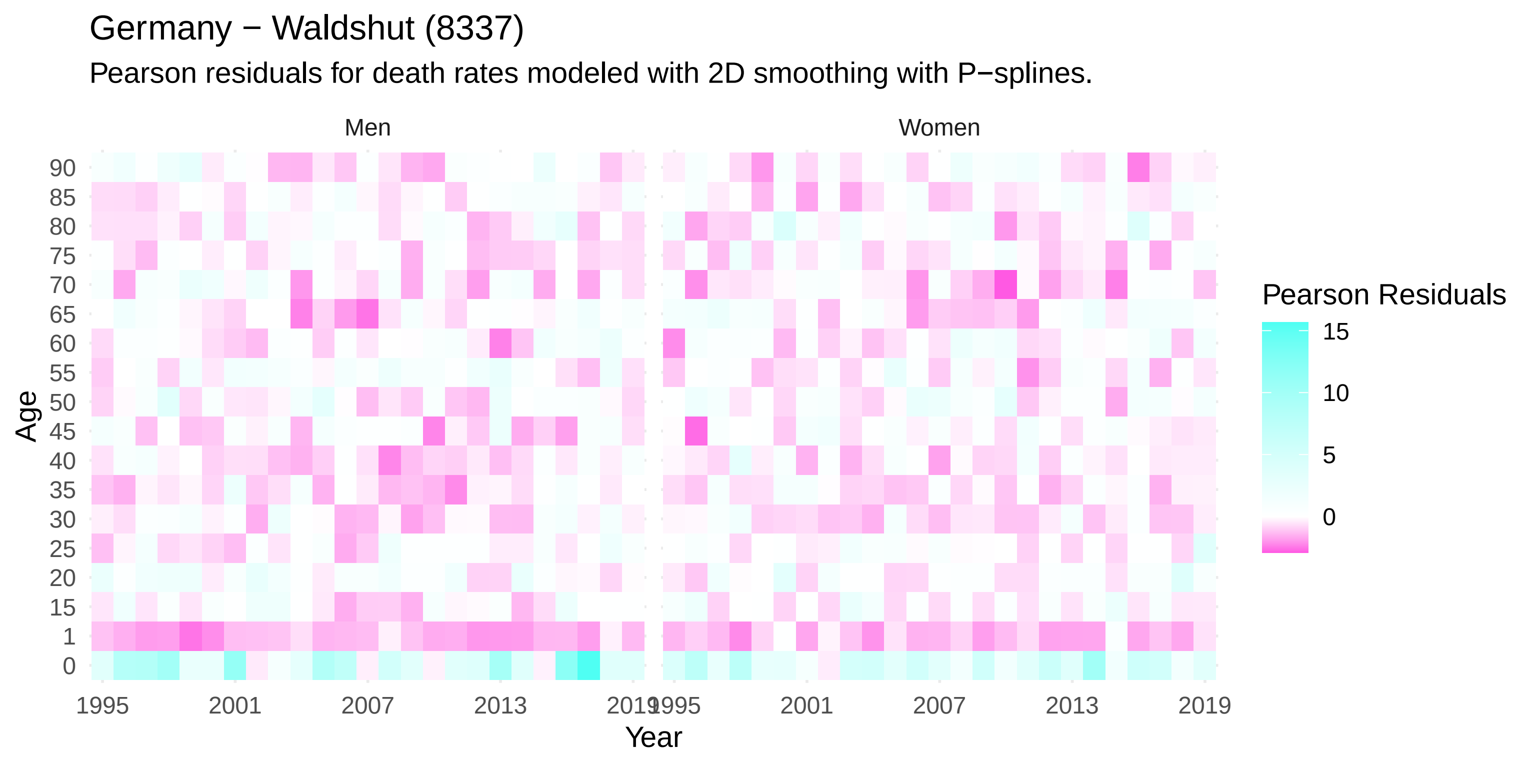

Germany − Waldshut (8337)
Pearson residuals for death rates modeled with 2D smoothing with P−splines.
Men
Women
Age
90
85
80
75
70
65
60
55
50
45
40
35
30
25
20
15
1
0
1995
2001
2007
2013
2019
Year
Pearson Residuals
15
10
5
0

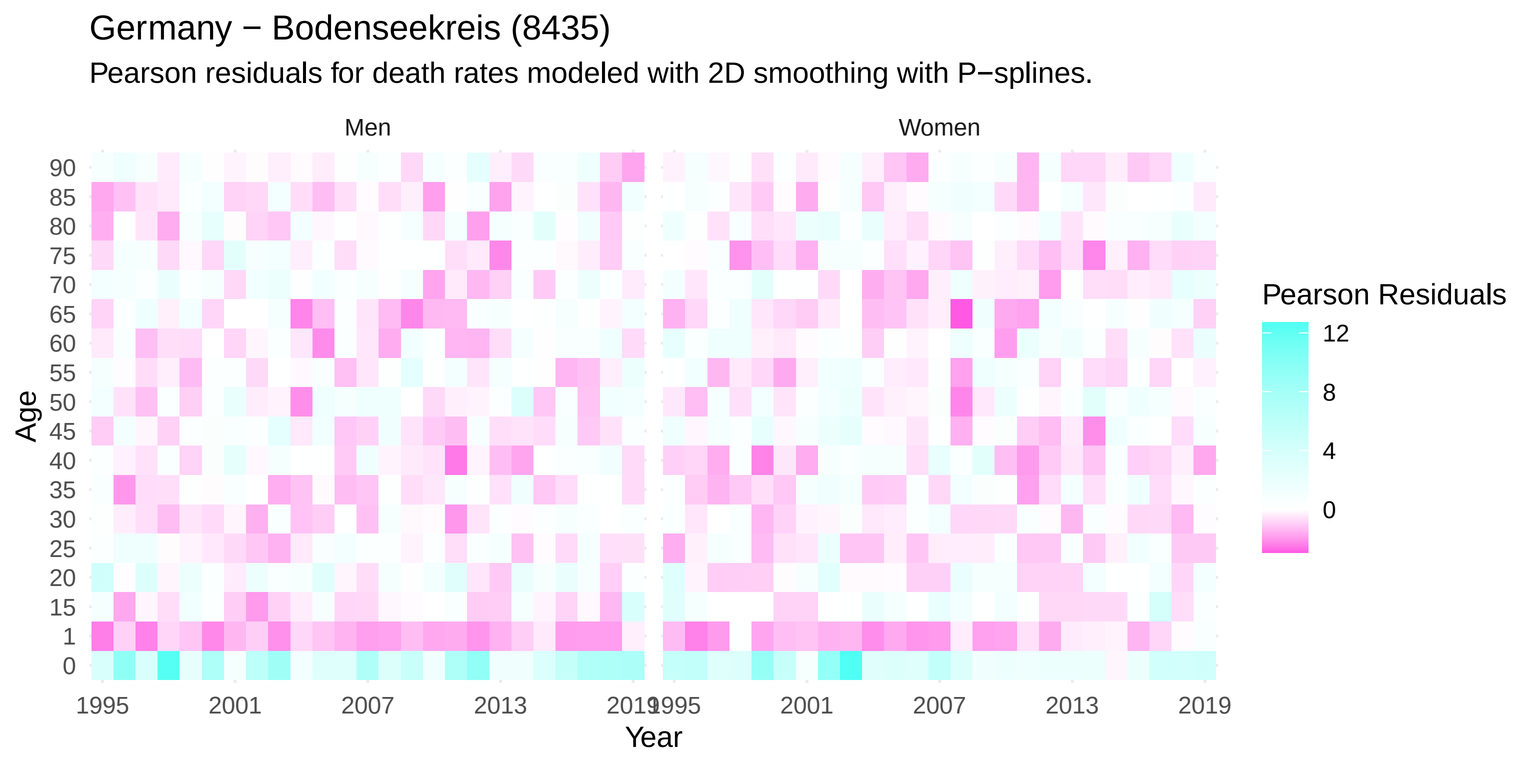

Germany − Bodenseekreis (8435)
Pearson residuals for death rates modeled with 2D smoothing with P−splines.
Men
Women
Age
90
85
80
75
70
65
60
55
50
45
40
35
30
25
20
15
1
0
1995
2001
2007
2013
2019
Year
Pearson Residuals
12
8
4
0

Germany − Ravensburg (8436)

Pearson residuals for death rates modeled with 2D smoothing with P−splines.

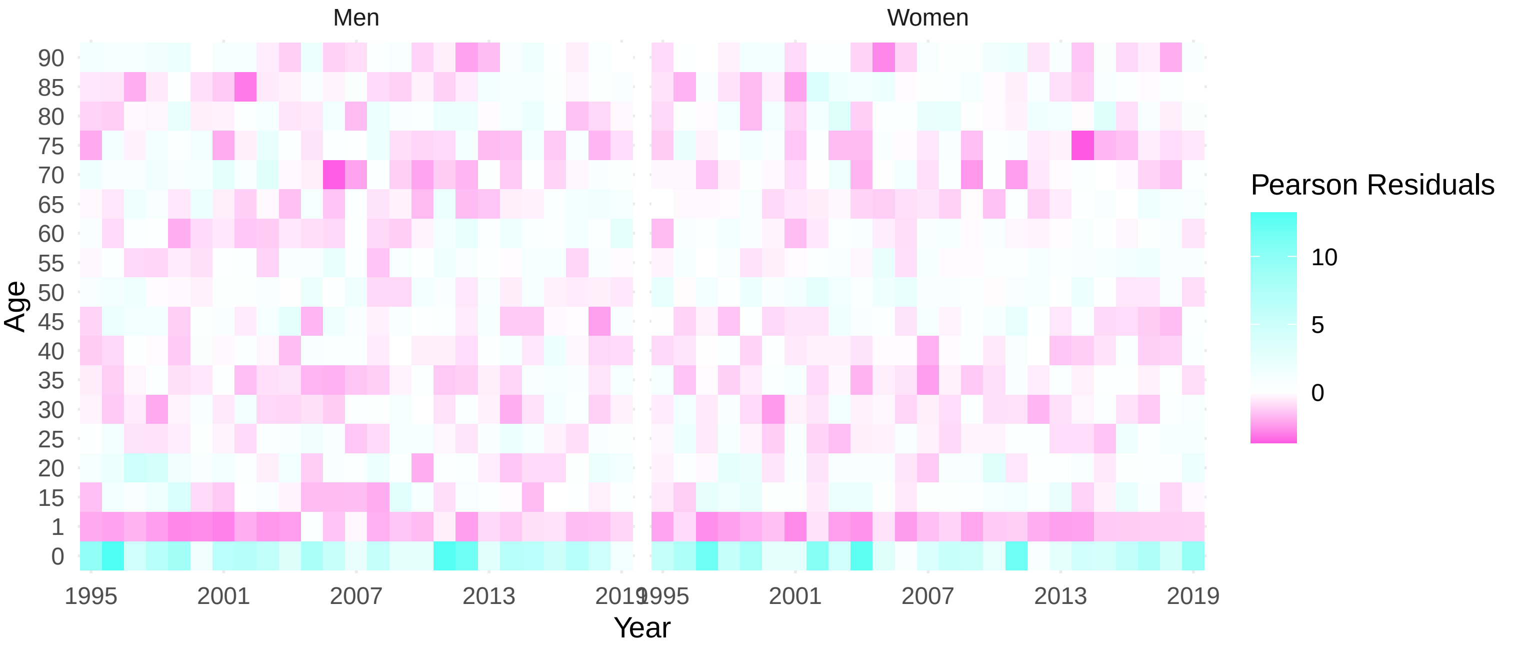

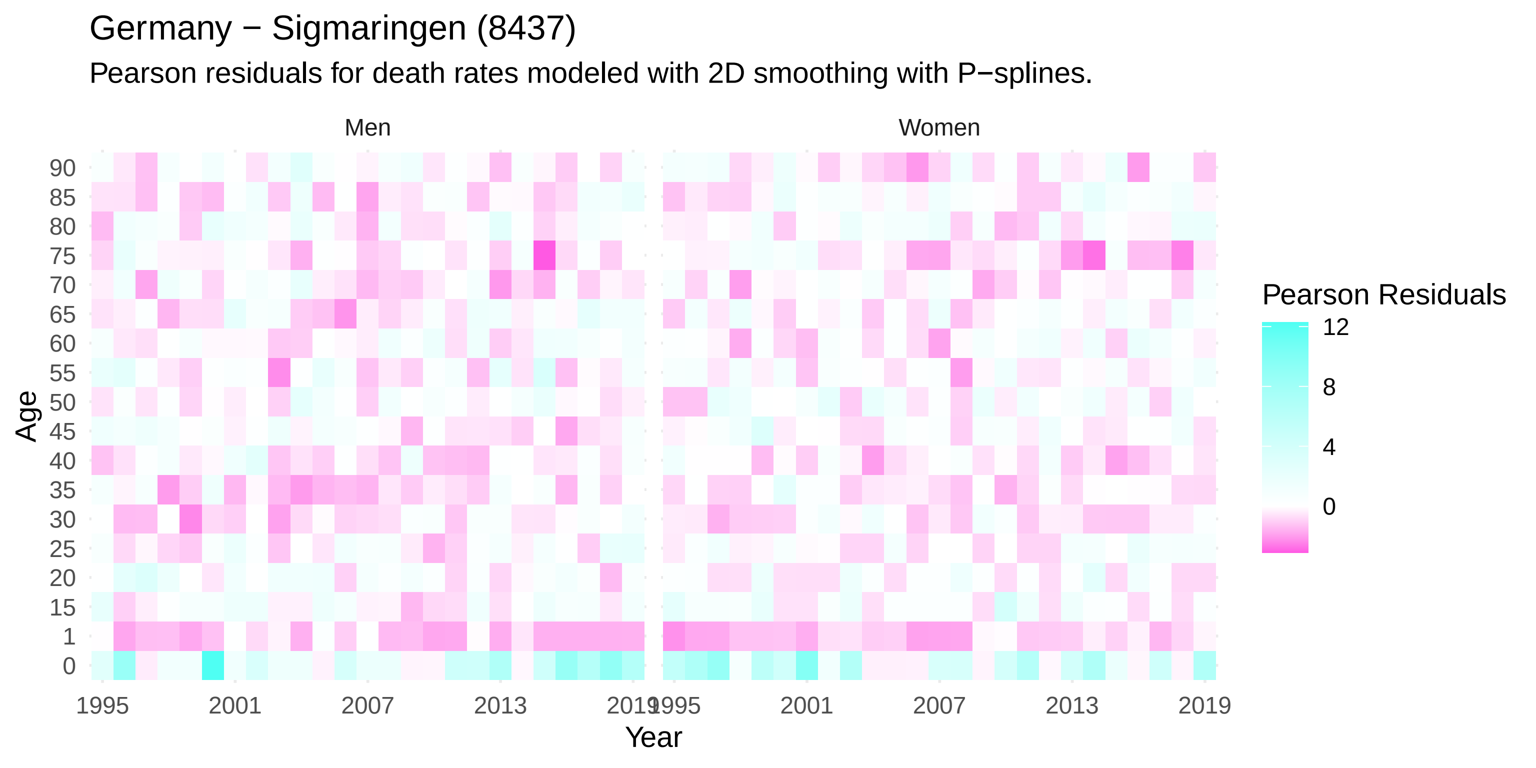
Germany − Sigmaringen (8437)
Pearson residuals for death rates modeled with 2D smoothing with P−splines.
Men
Women
Age
90
85
80
75
70
65
60
55
50
45
40
35
30
25
20
15
1
0
1995
2001
2007
2013
2019
Year
Pearson Residuals
12
8
4
0

Germany − Rosenheim (9163)

Pearson residuals for death rates modeled with 2D smoothing with P−splines.

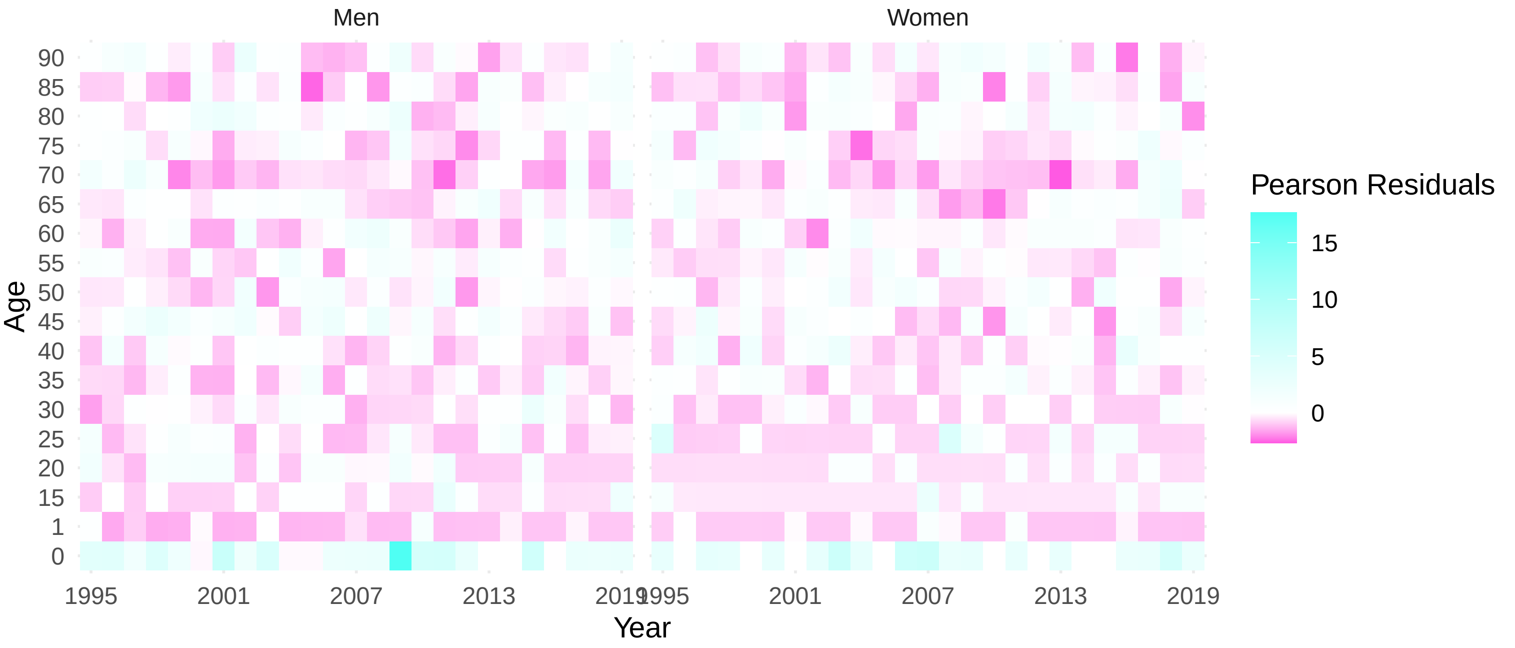

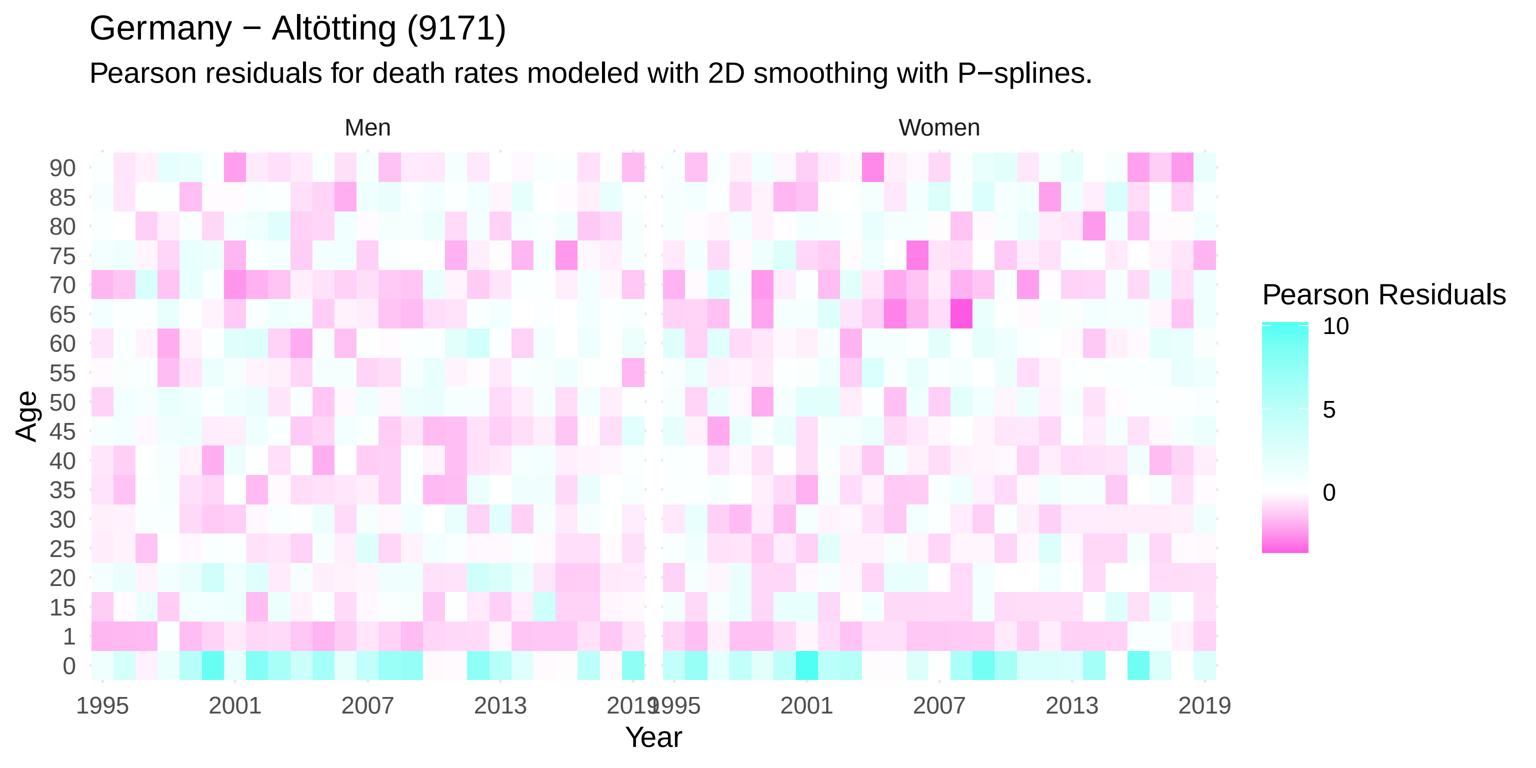
Germany − Altötting (9171)
Pearson residuals for death rates modeled with 2D smoothing with P−splines.
Men
Women
Age
90
85
80
75
70
65
60
55
50
45
40
35
30
25
20
15
1
0
1995
2001
2007
2013
2019
Year
Pearson Residuals
10
5
0

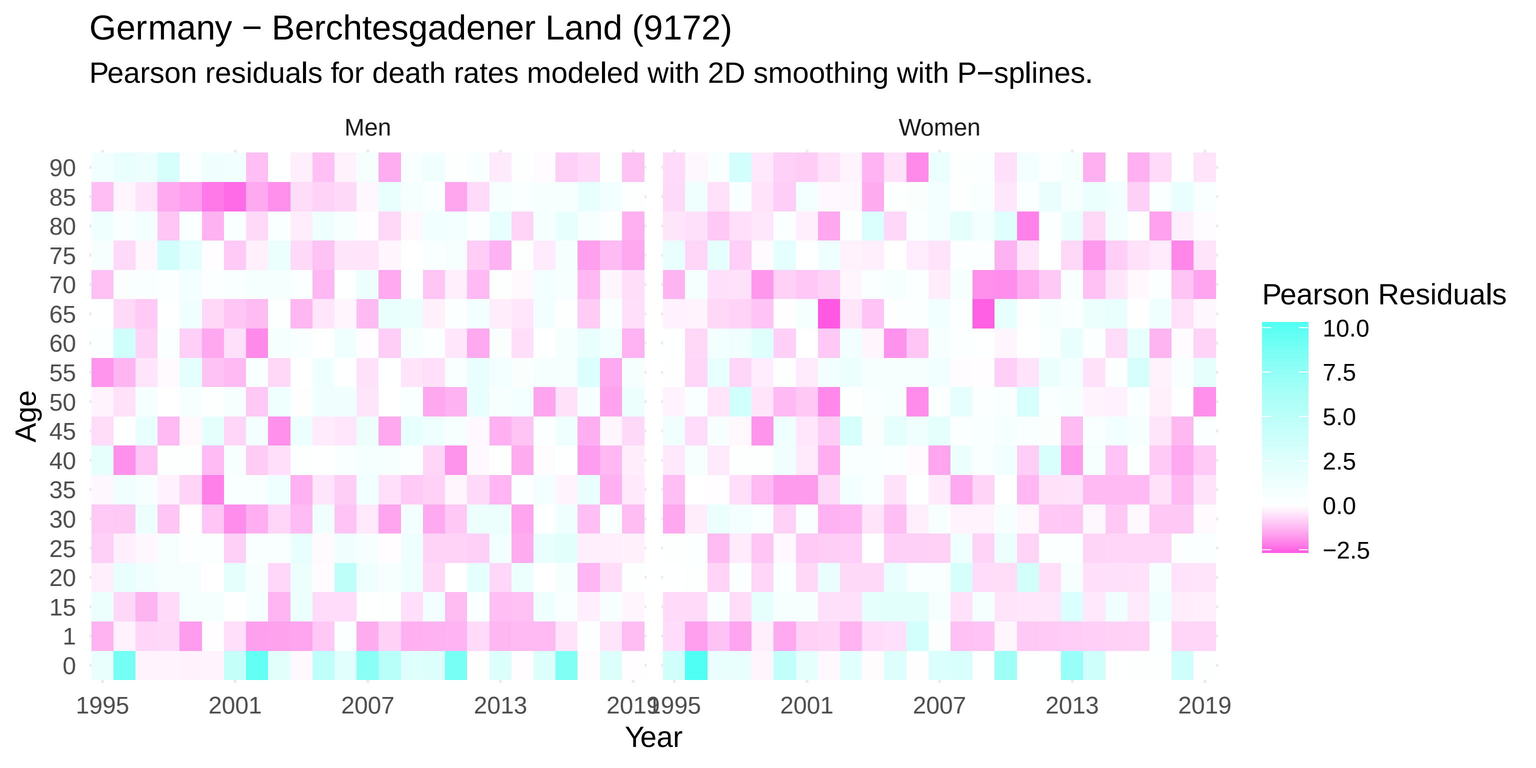

Germany − Berchtesgadener Land (9172)
Pearson residuals for death rates modeled with 2D smoothing with P−splines.
Men
Women
Age
90
85
80
75
70
65
60
55
50
45
40
35
30
25
20
15
1
0
1995
2001
2007
2013
2019
Year
Pearson Residuals
10.0
7.5
5.0
2.5
0.0
−2.5

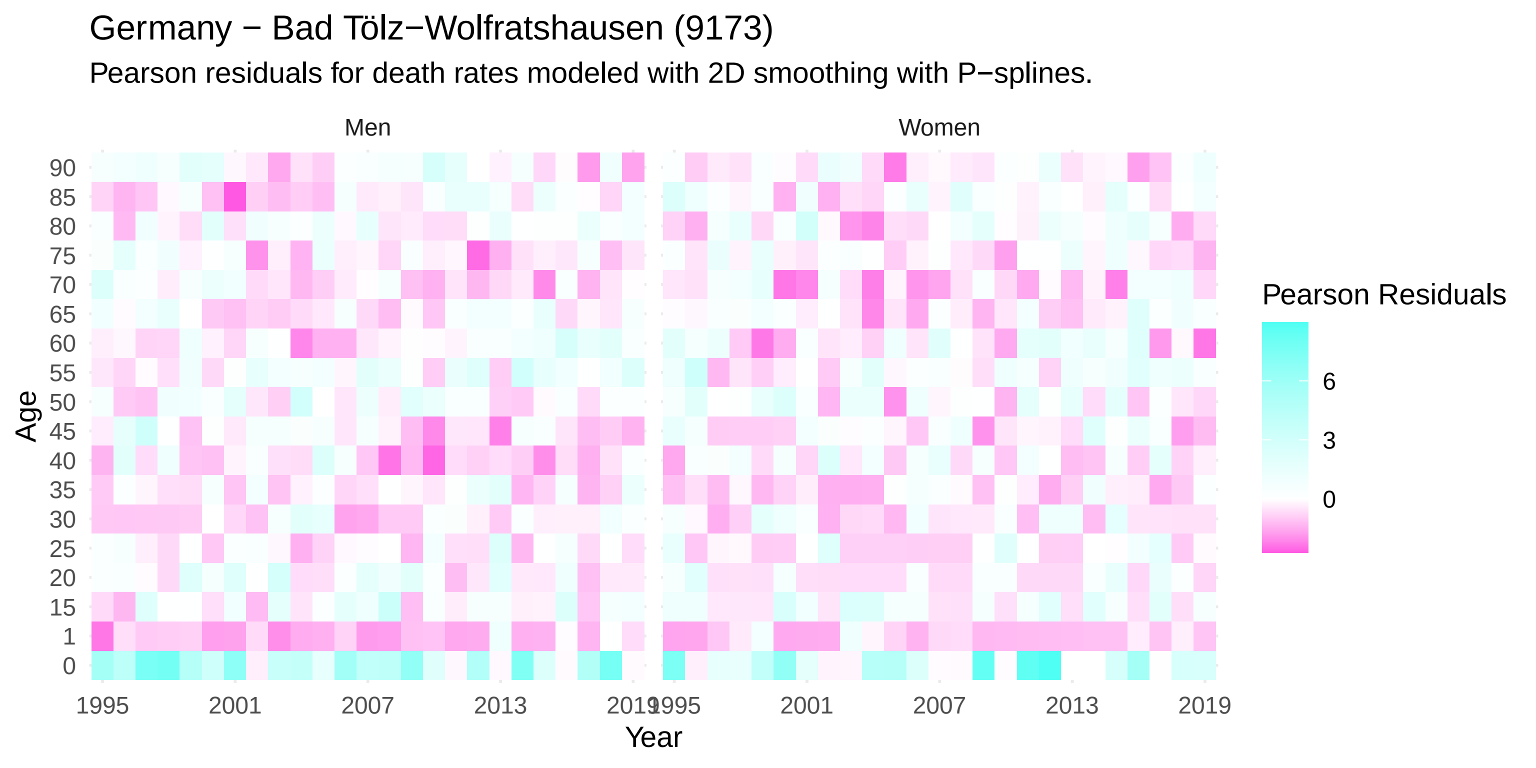
Germany − Bad Tölz−Wolfratshausen (9173)
Pearson residuals for death rates modeled with 2D smoothing with P−splines.
Men
Women
Age
90
85
80
75
70
65
60
55
50
45
40
35
30
25
20
15
1
0
1995
2001
2007
2013
2019
Year
Pearson Residuals
6
3
0

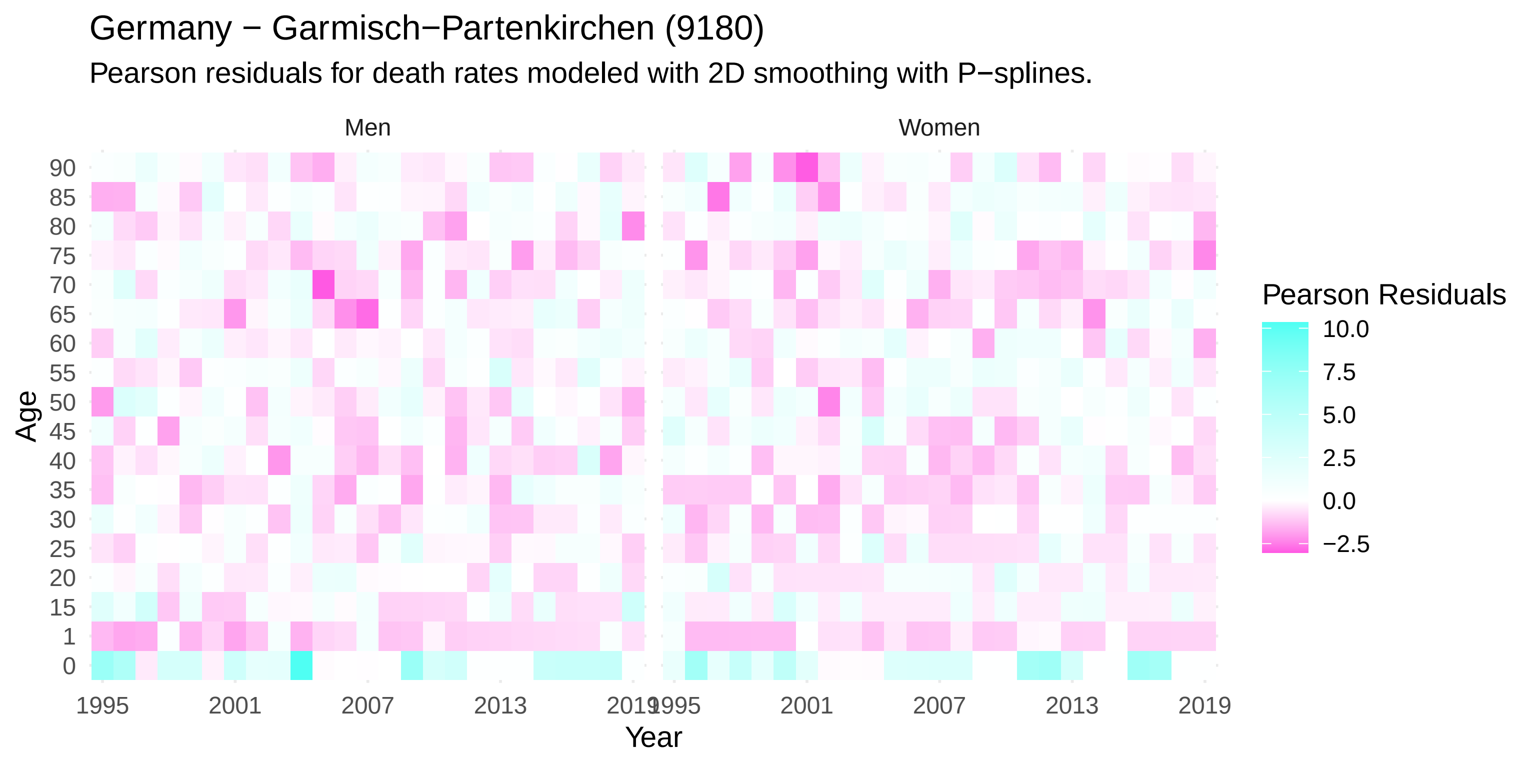

Germany − Garmisch−Partenkirchen (9180)
Pearson residuals for death rates modeled with 2D smoothing with P−splines.
Men
Women
Age
90
85
80
75
70
65
60
55
50
45
40
35
30
25
20
15
1
0
1995
2001
2007
2013
2019
Year
Pearson Residuals
10.0
7.5
5.0
2.5
0.0
−2.5

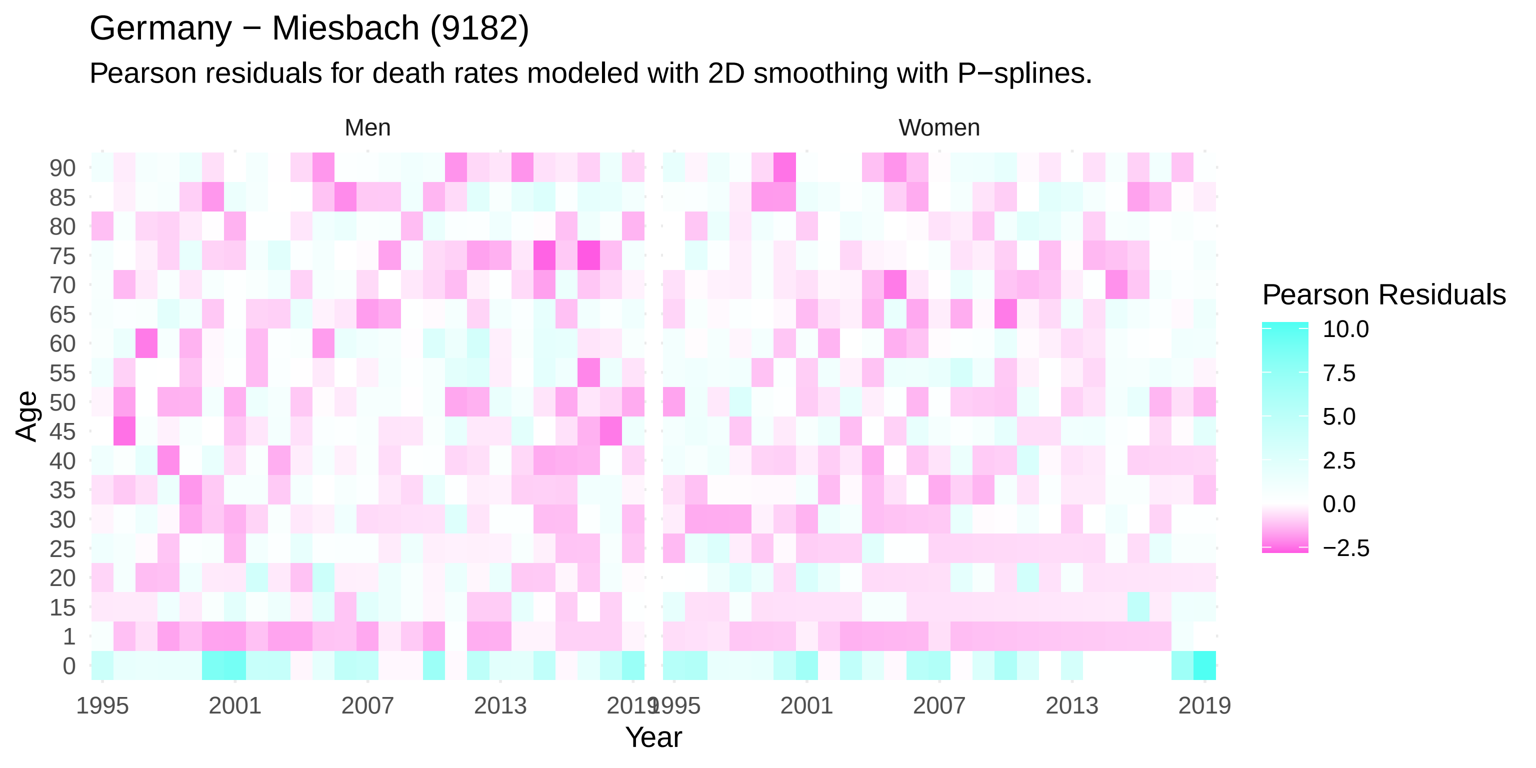

Germany − Miesbach (9182)
Pearson residuals for death rates modeled with 2D smoothing with P−splines.
Men
Women
Age
90
85
80
75
70
65
60
55
50
45
40
35
30
25
20
15
1
0
1995
2001
2007
2013
2019
Year
Pearson Residuals
10.0
7.5
5.0
2.5
0.0
−2.5

Germany − Mühldorf a.Inn (9183)

Pearson residuals for death rates modeled with 2D smoothing with P−splines.

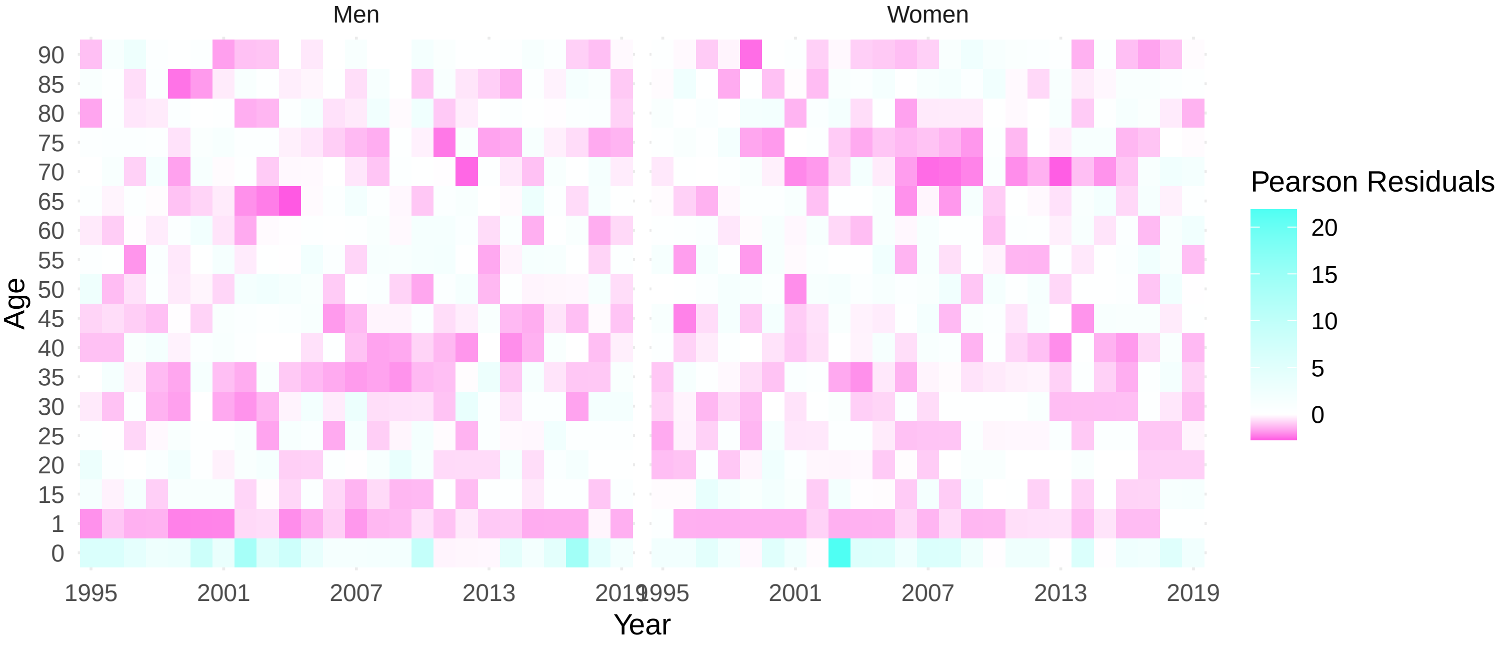

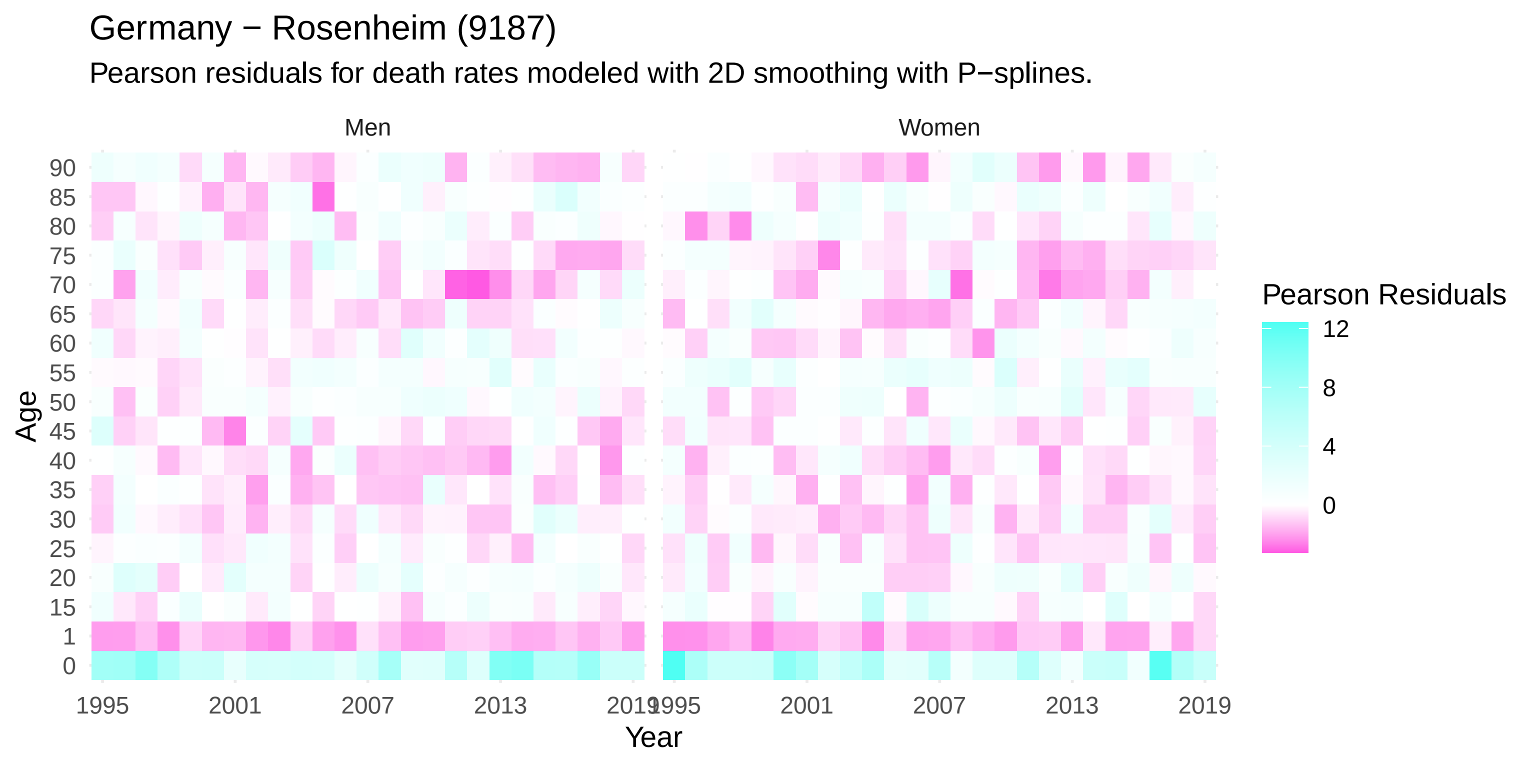

Germany − Rosenheim (9187)
Pearson residuals for death rates modeled with 2D smoothing with P−splines.
Men
Women
Age
Year
Pearson Residuals
12
8
4
0
90
85
80
75
70
65
60
55
50
45
40
35
30
25
20
15
1
0
1995
2001
2007
2013
2019

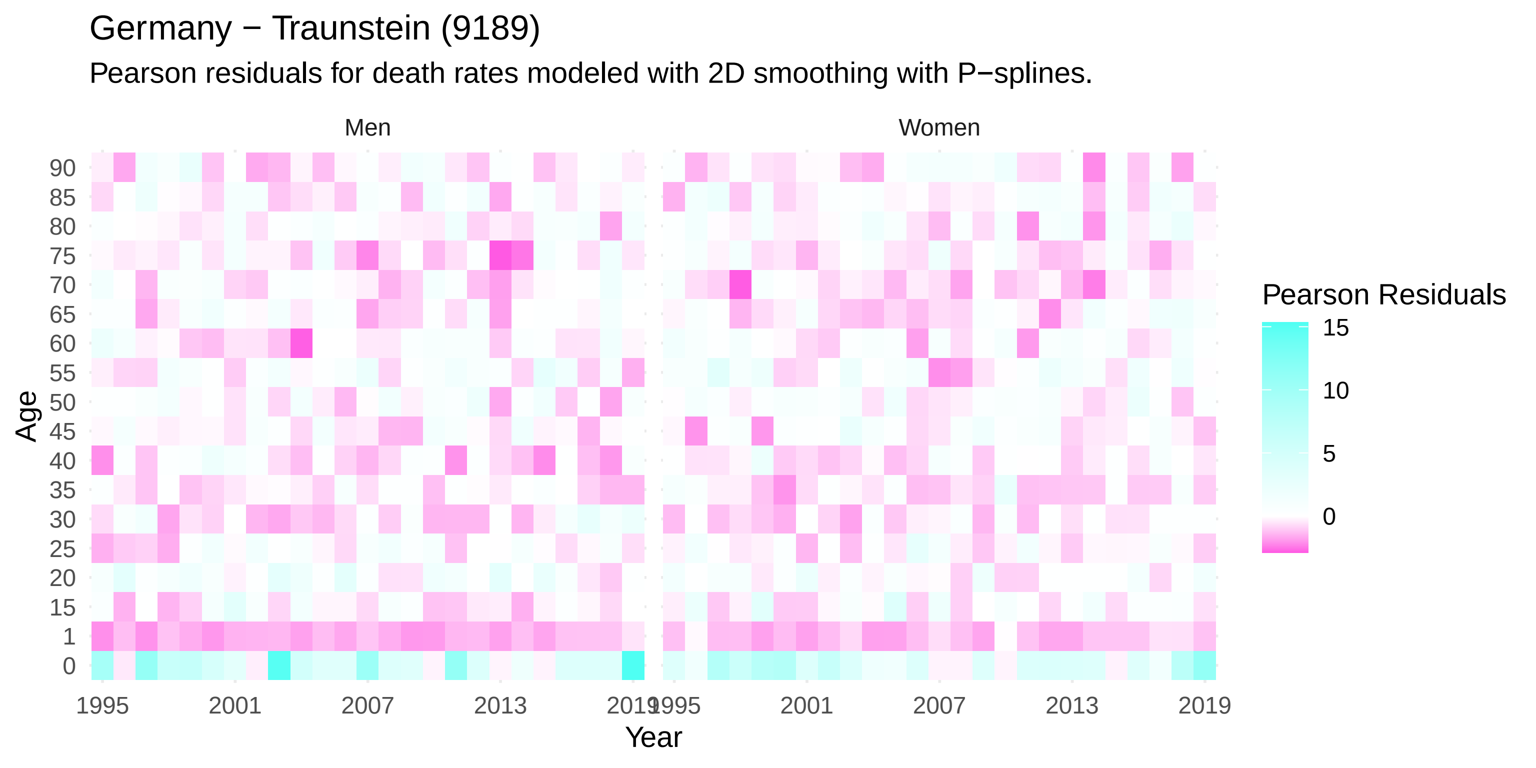

Germany − Traunstein (9189)
Pearson residuals for death rates modeled with 2D smoothing with P−splines.
Men
Women
Age
90
85
80
75
70
65
60
55
50
45
40
35
30
25
20
15
1
0
1995
2001
2007
2013
2019
Year
Pearson Residuals
15
10
5
0

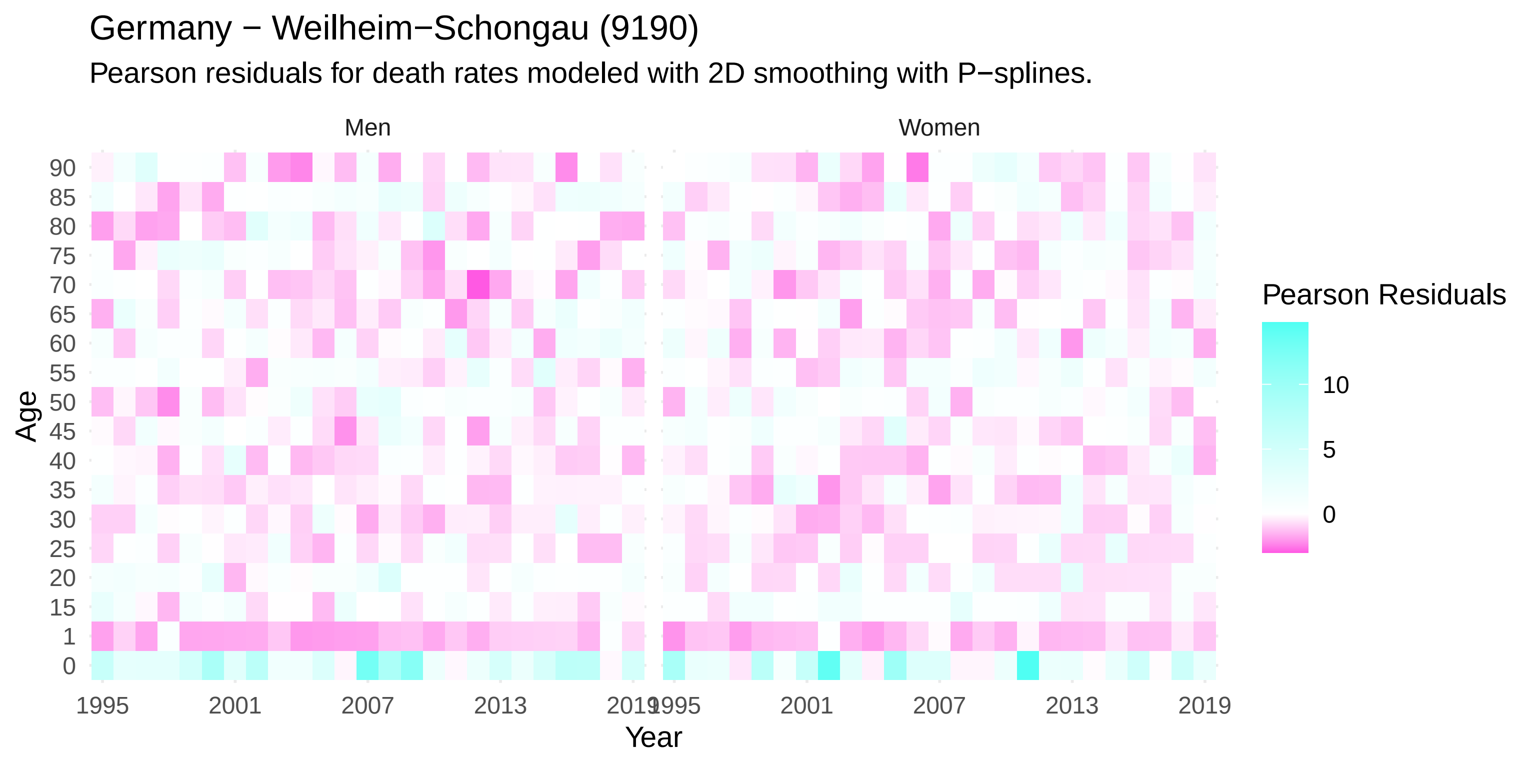

Germany − Weilheim−Schongau (9190)
Pearson residuals for death rates modeled with 2D smoothing with P−splines.
Men
Women
Age
90
85
80
75
70
65
60
55
50
45
40
35
30
25
20
15
1
0
1995
2001
2007
2013
2019
Year
Pearson Residuals
10
5
0

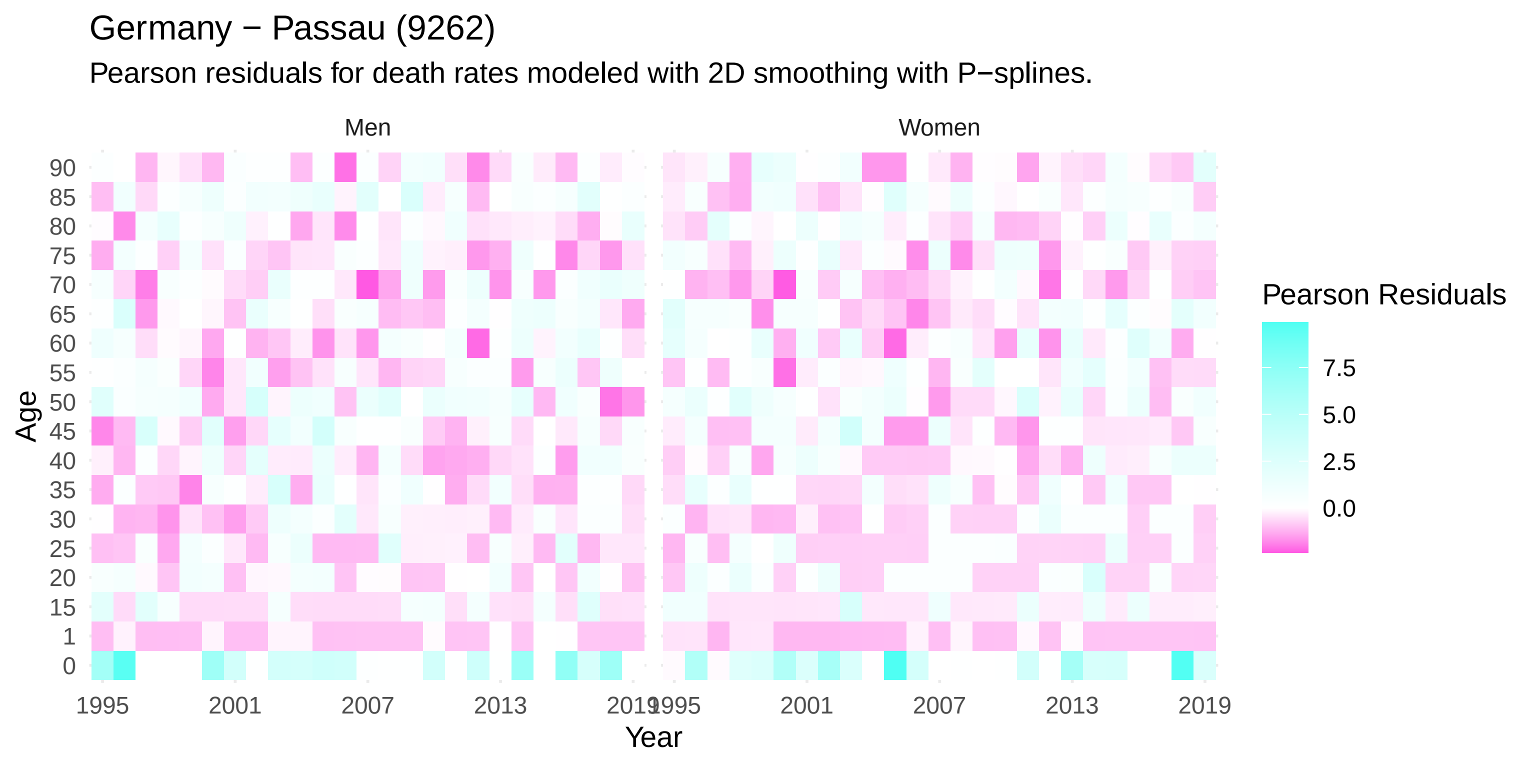

Germany − Passau (9262)
Pearson residuals for death rates modeled with 2D smoothing with P−splines.
Men
Women
Age
90
85
80
75
70
65
60
55
50
45
40
35
30
25
20
15
1
0
1995
2001
2007
2013
2019
Year
Pearson Residuals
7.5
5.0
2.5
0.0

Germany − Deggendorf (9271)

Pearson residuals for death rates modeled with 2D smoothing with P−splines.

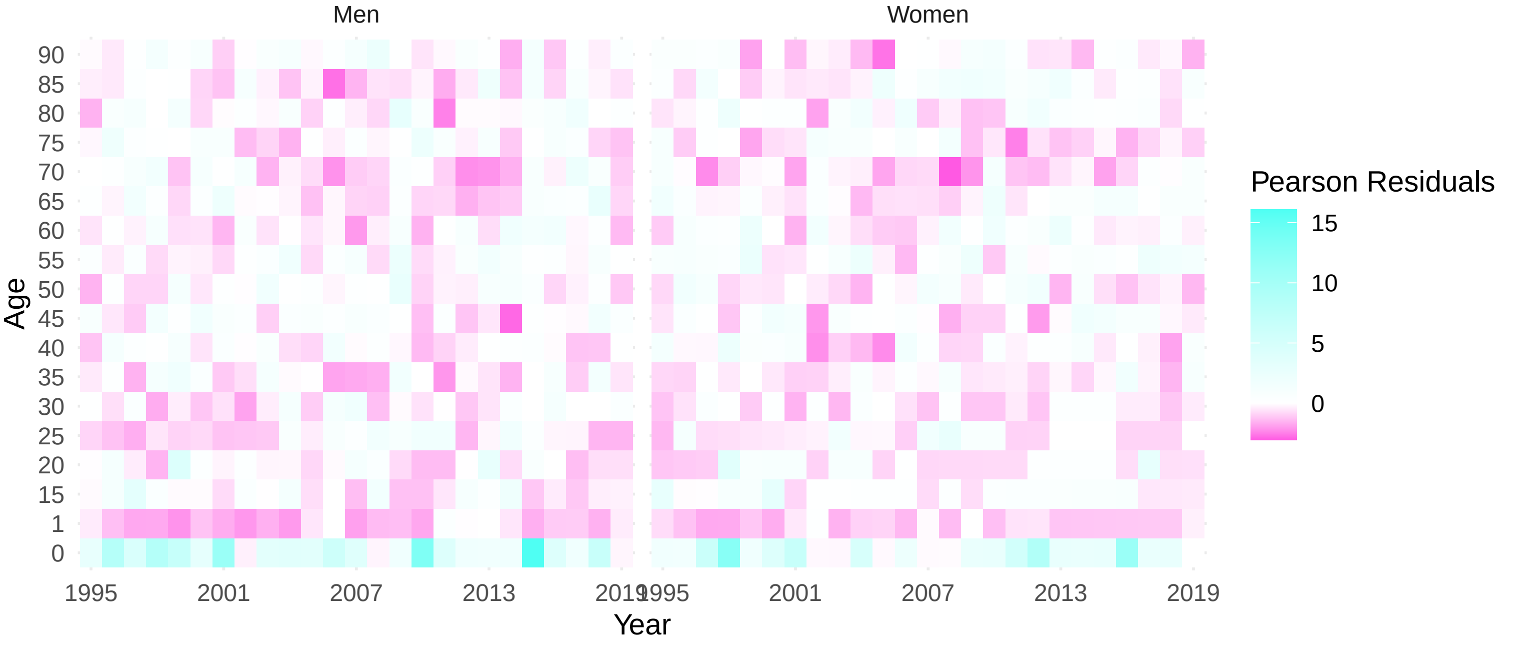

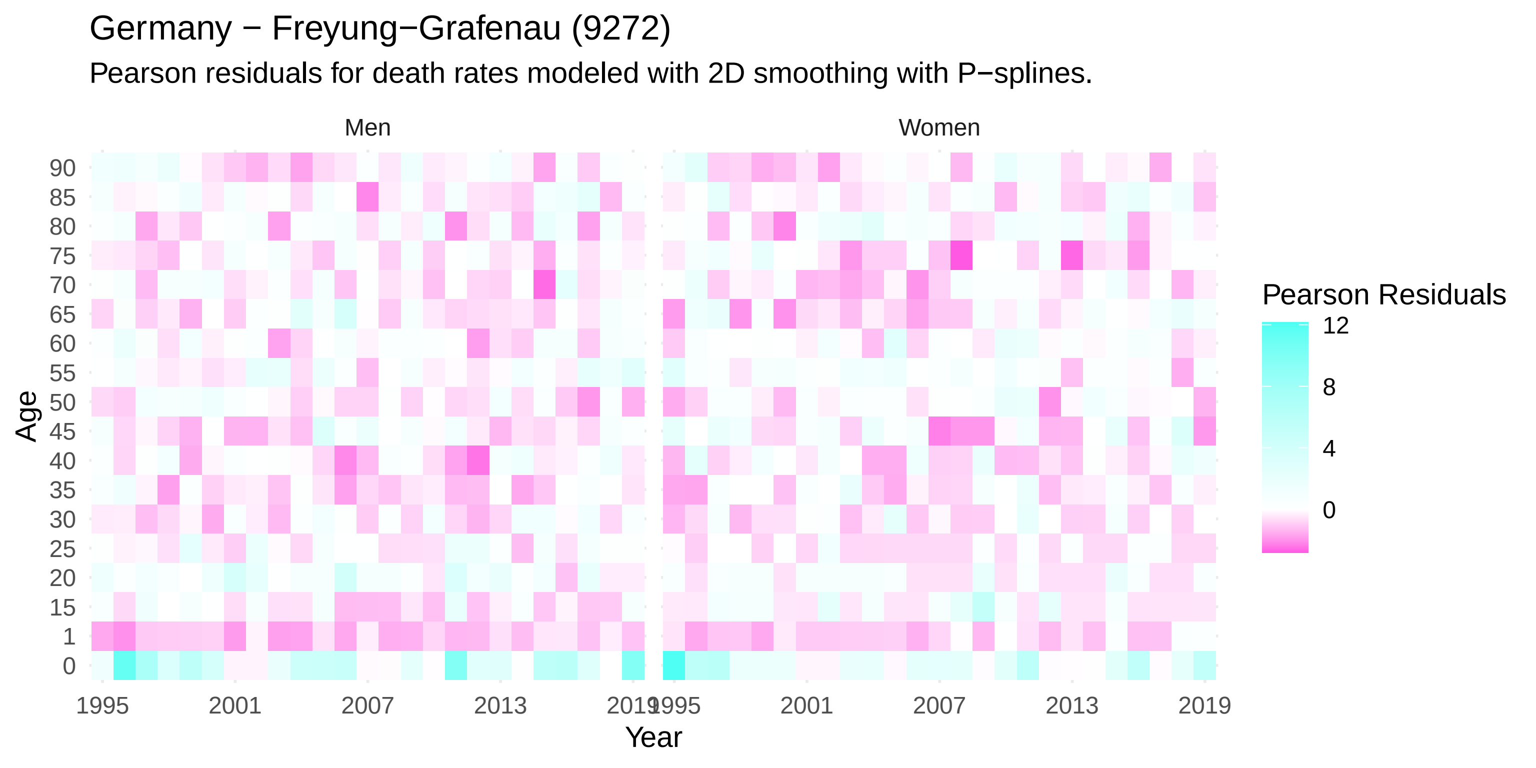
Germany − Freyung−Grafenau (9272)
Pearson residuals for death rates modeled with 2D smoothing with P−splines.
Men
Women
Age
Year
Pearson Residuals
12
8
4
0
90
85
80
75
70
65
60
55
50
45
40
35
30
25
20
15
1
0
1995
2001
2007
2013
2019

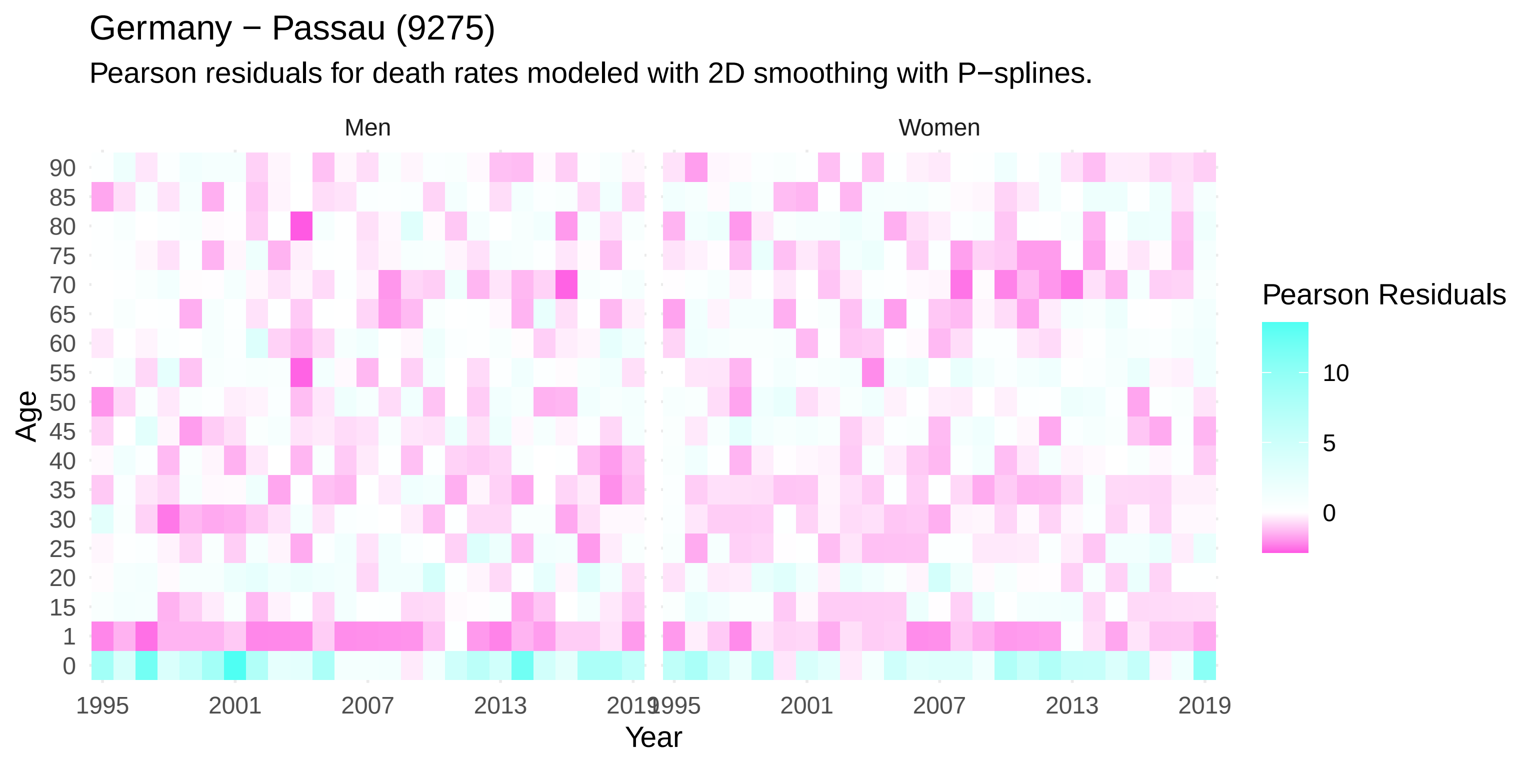
Germany − Passau (9275)
Pearson residuals for death rates modeled with 2D smoothing with P−splines.
Men
Women
Age
90
85
80
75
70
65
60
55
50
45
40
35
30
25
20
15
1
0
1995
2001
2007
2013
2019
Year
Pearson Residuals
10
5
0

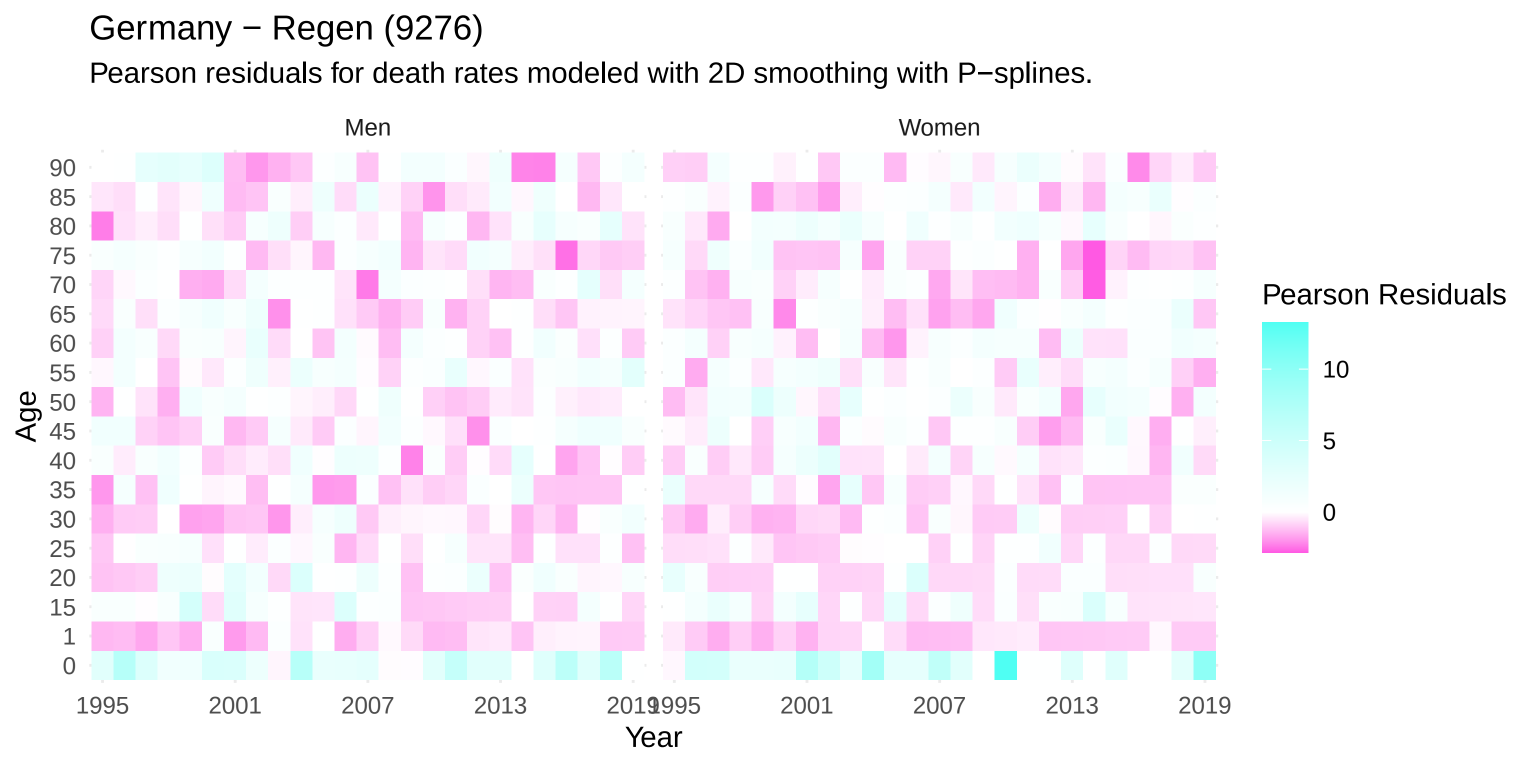

Germany − Regen (9276)
Pearson residuals for death rates modeled with 2D smoothing with P−splines.
Men
Women
Age
90
85
80
75
70
65
60
55
50
45
40
35
30
25
20
15
1
0
1995
2001
2007
2013
2019
Year
Pearson Residuals
10
5
0

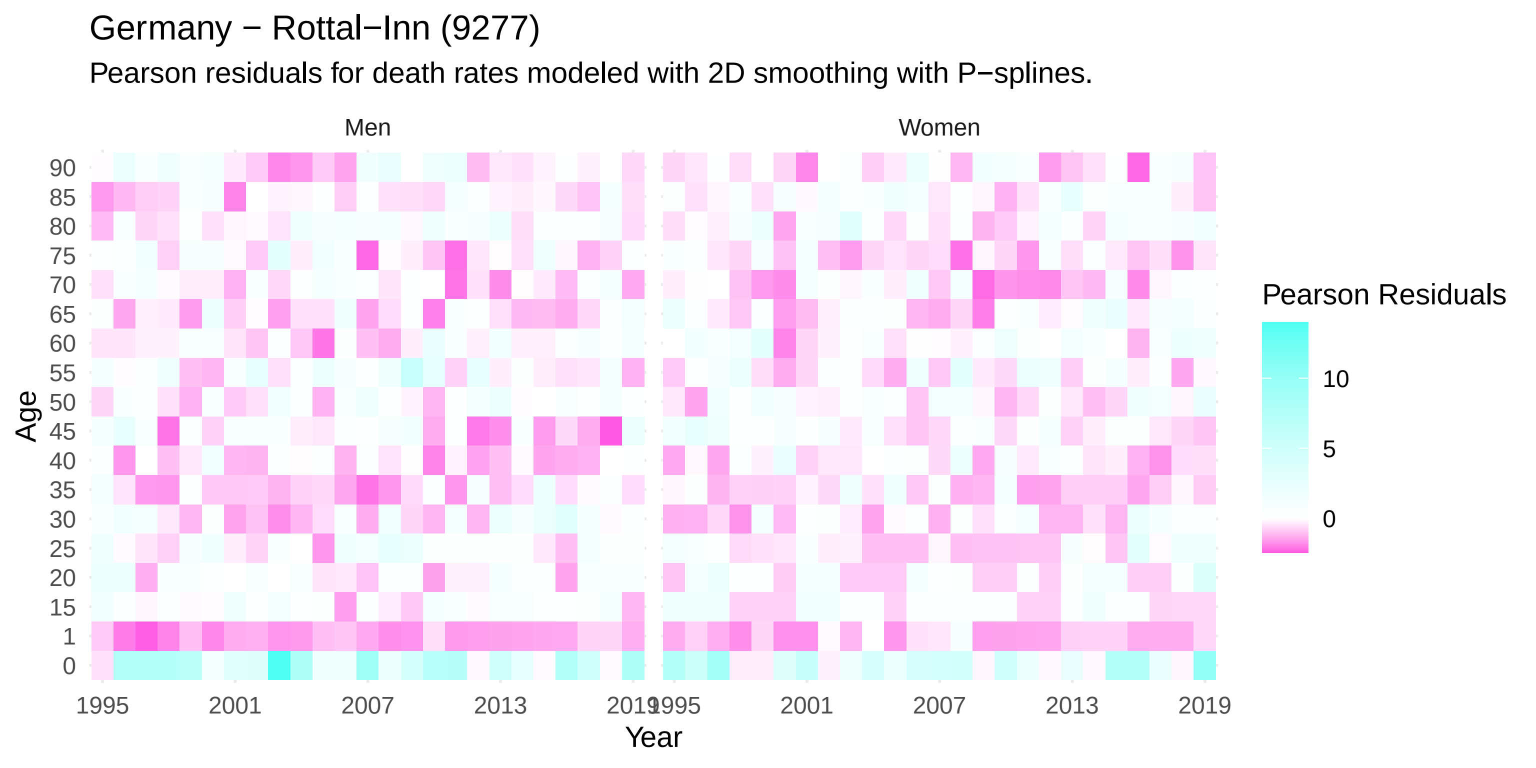
Germany − Rottal−Inn (9277)
Pearson residuals for death rates modeled with 2D smoothing with P−splines.
Men
Women
Age
90
85
80
75
70
65
60
55
50
45
40
35
30
25
20
15
1
0
1995
2001
2007
2013
2019
Year
Pearson Residuals
10
5
0

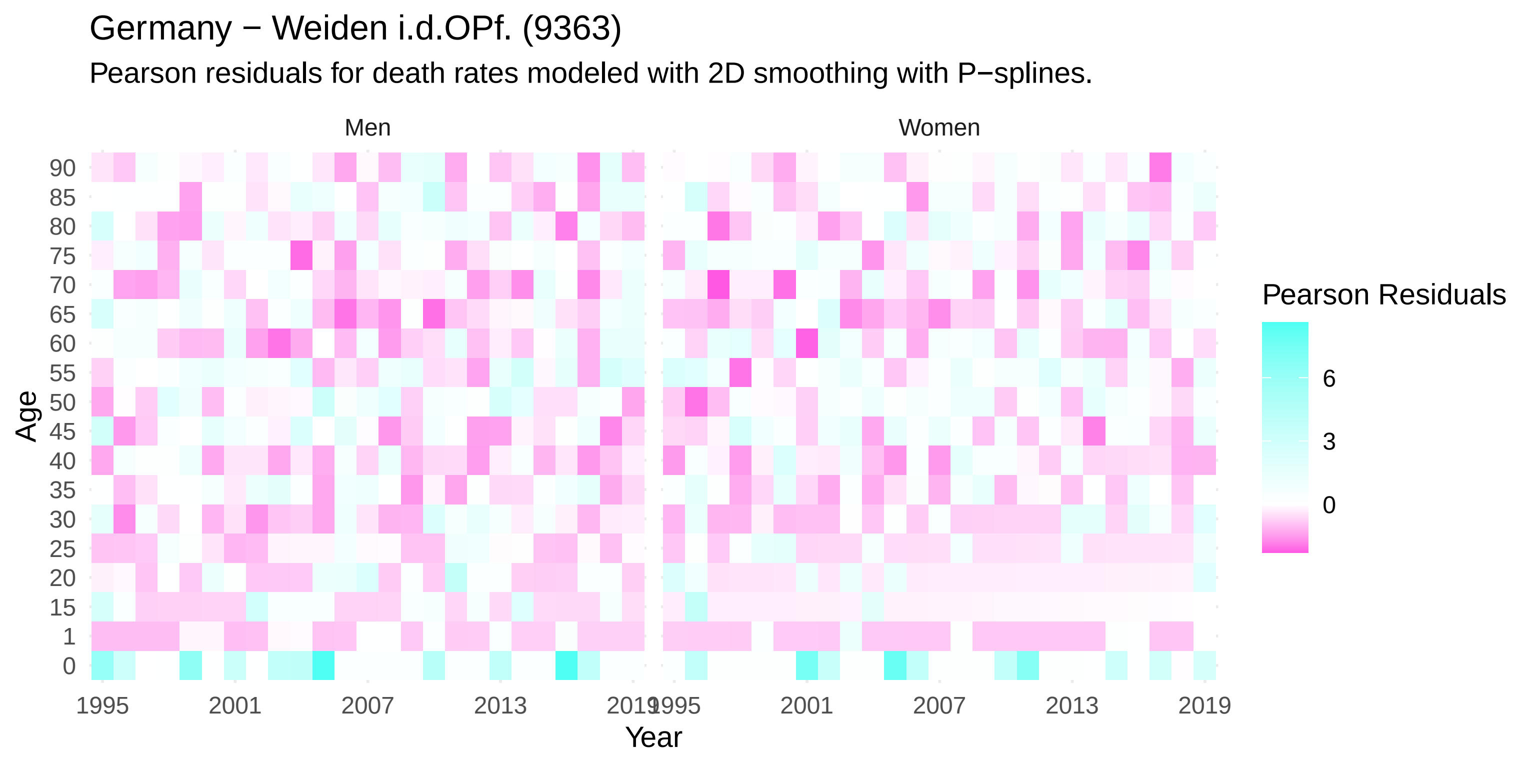

Germany − Weiden i.d.OPf. (9363)
Pearson residuals for death rates modeled with 2D smoothing with P−splines.
Men
Women
Age
90
85
80
75
70
65
60
55
50
45
40
35
30
25
20
15
1
0
1995
2001
2007
2013
2019
Year
Pearson Residuals
6
3
0

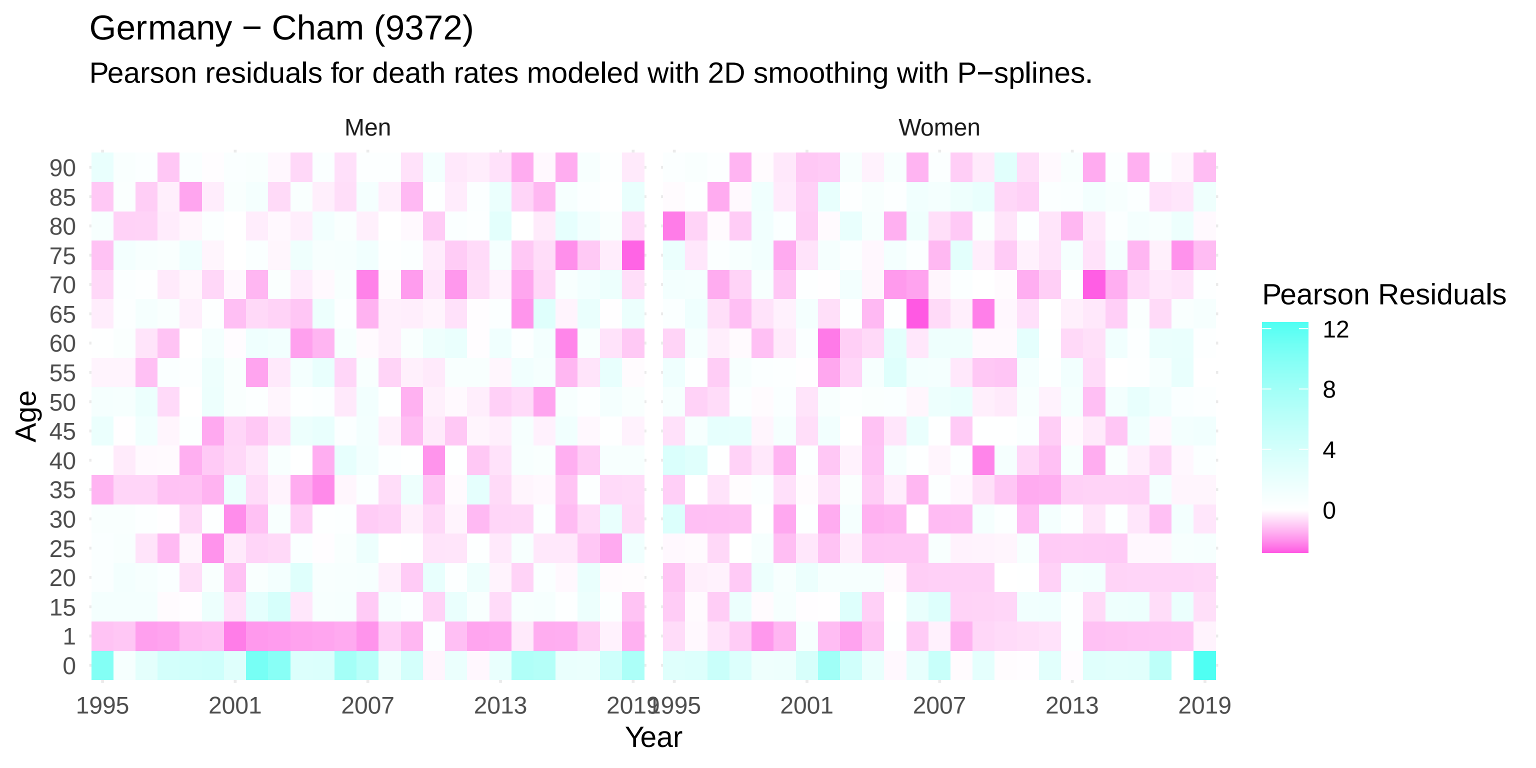

Germany − Cham (9372)
Pearson residuals for death rates modeled with 2D smoothing with P−splines.
Men
Women
Age
90
85
80
75
70
65
60
55
50
45
40
35
30
25
20
15
1
0
1995
2001
2007
2013
2019
Year
Pearson Residuals
12
8
4
0

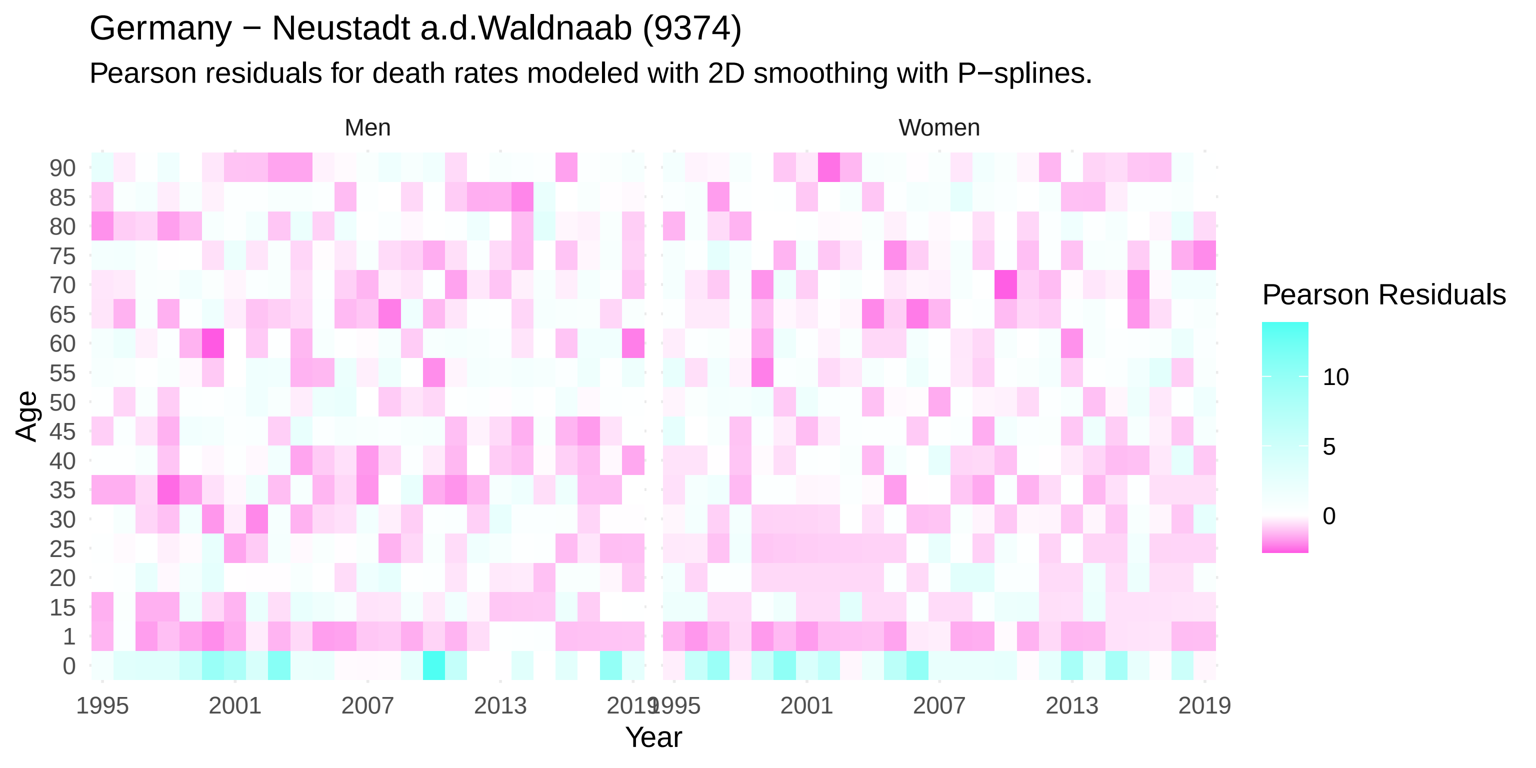

Germany − Neustadt a.d.Waldnaab (9374)
Pearson residuals for death rates modeled with 2D smoothing with P−splines.
Men
Women
Age
90
85
80
75
70
65
60
55
50
45
40
35
30
25
20
15
1
0
1995
2001
2007
2013
2019
Year
Pearson Residuals
10
5
0

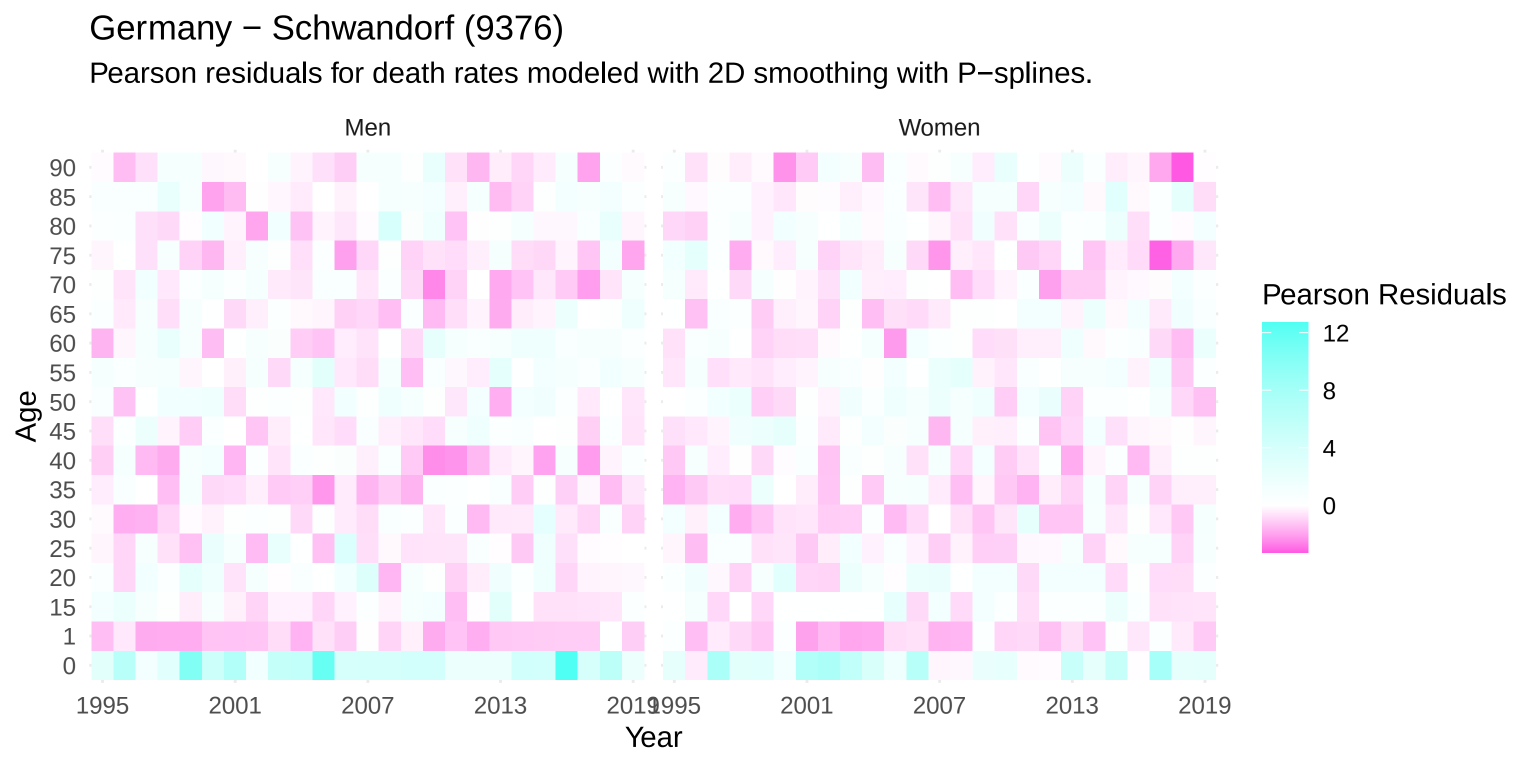
Germany − Schwandorf (9376)
Pearson residuals for death rates modeled with 2D smoothing with P−splines.
Men
Women
Age
90
85
80
75
70
65
60
55
50
45
40
35
30
25
20
15
1
0
1995
2001
2007
2013
2019
Year
Pearson Residuals
12
8
4
0

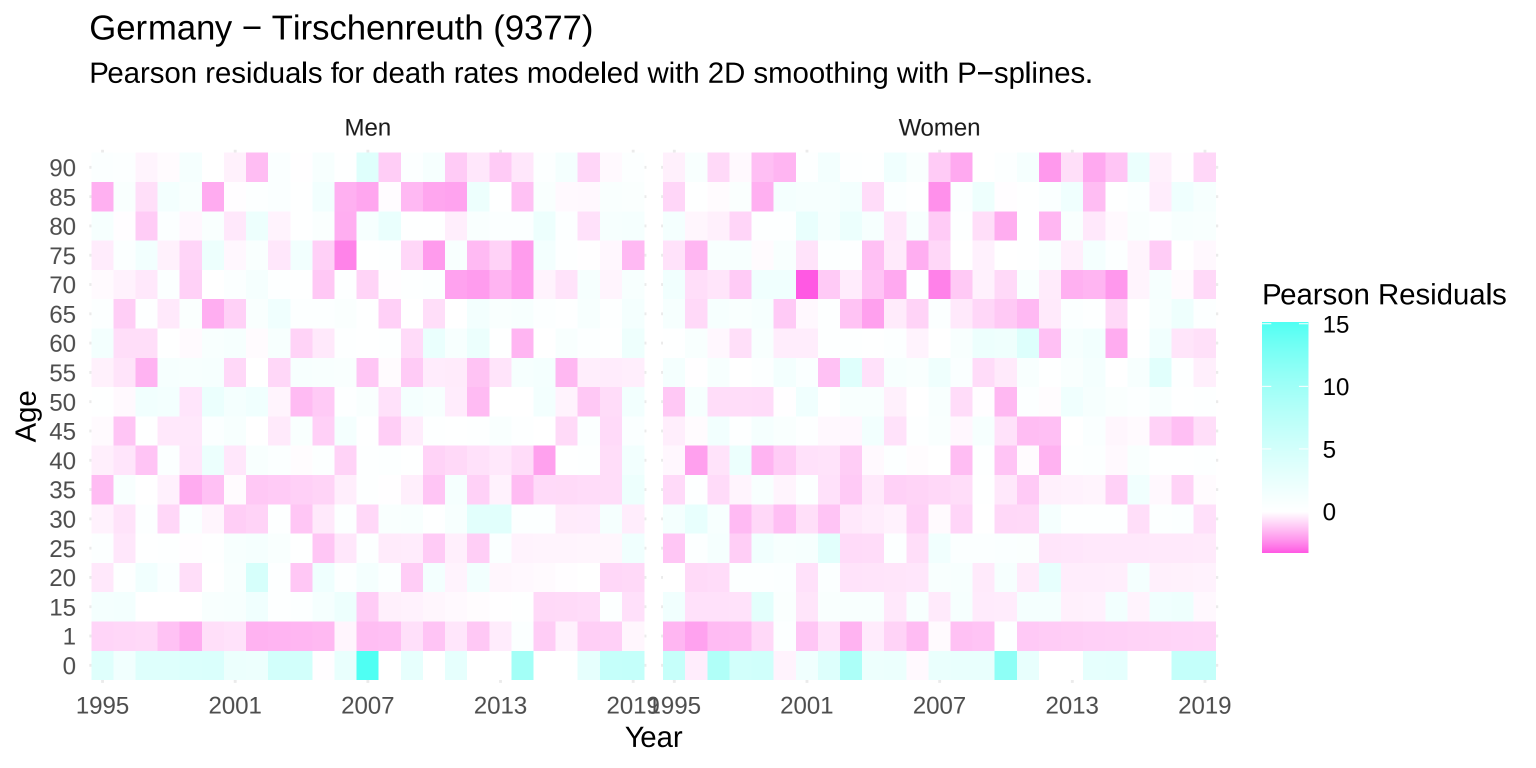
Germany − Tirschenreuth (9377)
Pearson residuals for death rates modeled with 2D smoothing with P−splines.
Men
Women
Age
90
85
80
75
70
65
60
55
50
45
40
35
30
25
20
15
1
0
1995
2001
2007
2013
2019
1995
2001
2007
2013
2019
Year
Pearson Residuals
15
10
5
0

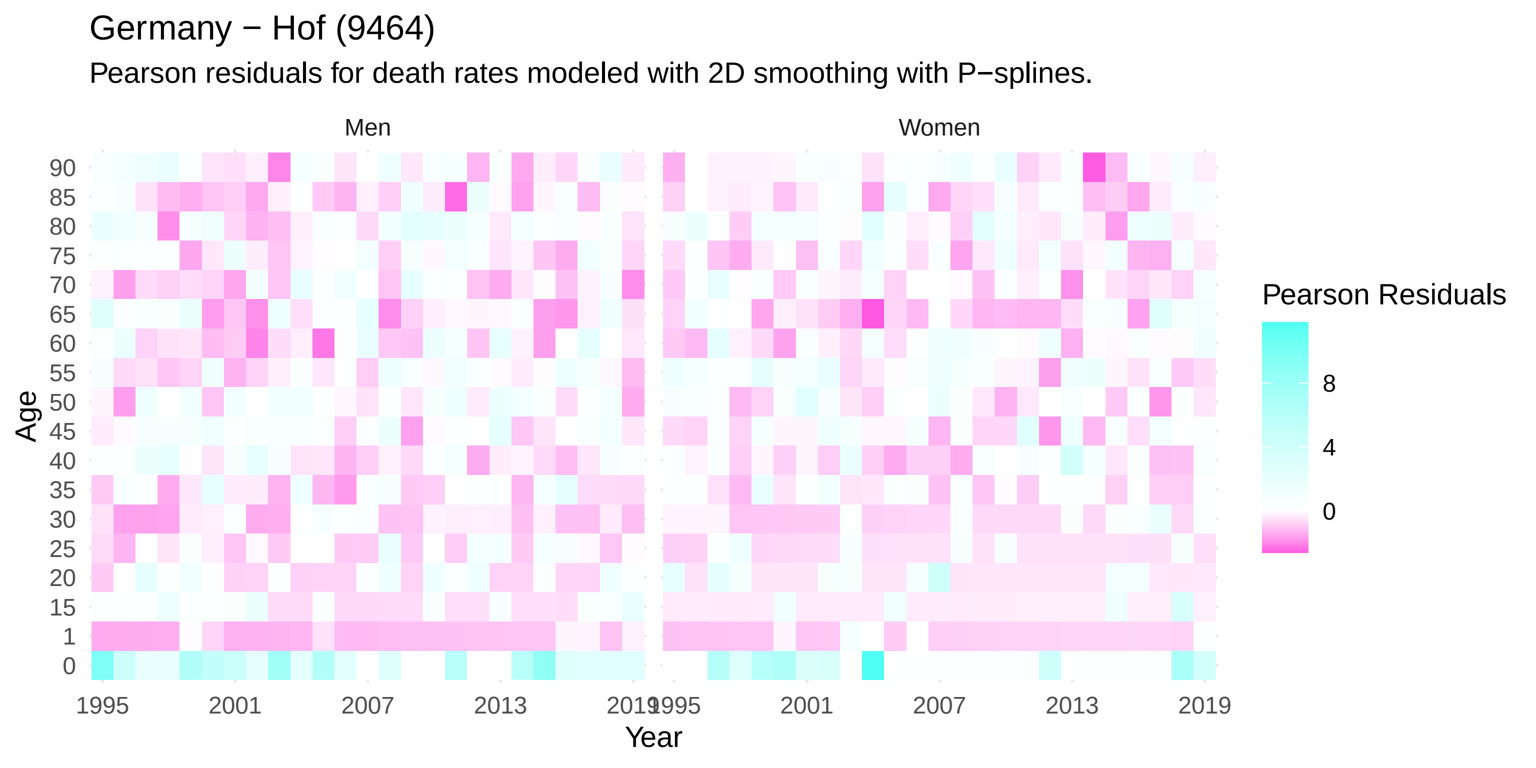

Germany − Hof (9464)
Pearson residuals for death rates modeled with 2D smoothing with P−splines.
Men
Women
Age
90
85
80
75
70
65
60
55
50
45
40
35
30
25
20
15
1
0
Year
1995
2001
2007
2013
2019
Pearson Residuals
8
4
0

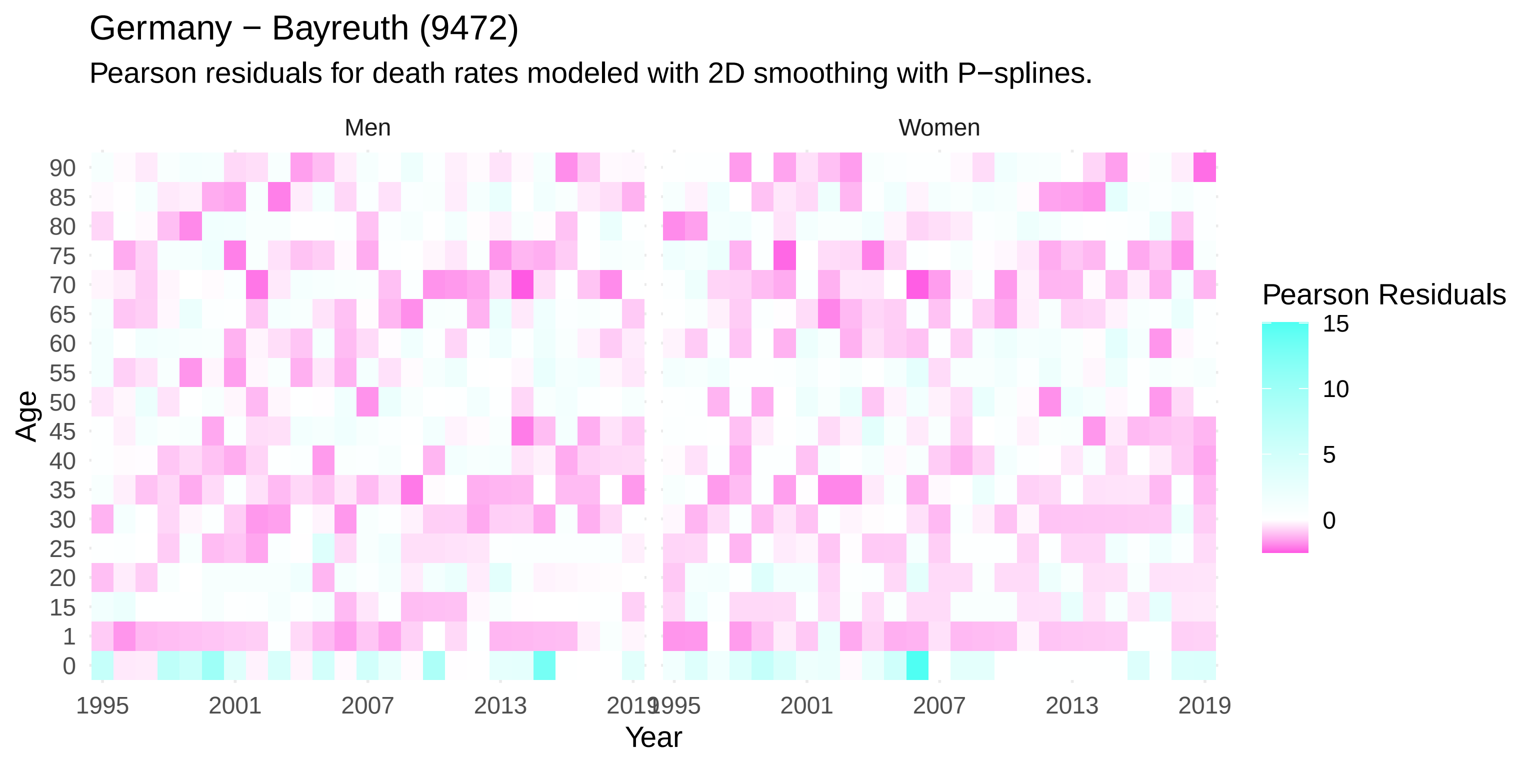

Germany − Bayreuth (9472)
Pearson residuals for death rates modeled with 2D smoothing with P−splines.
Men
Women
Age
90
85
80
75
70
65
60
55
50
45
40
35
30
25
20
15
1
0
1995
2001
2007
2013
2019
Year
Pearson Residuals
15
10
5
0

Germany − Hof (9475)

Pearson residuals for death rates modeled with 2D smoothing with P−splines.

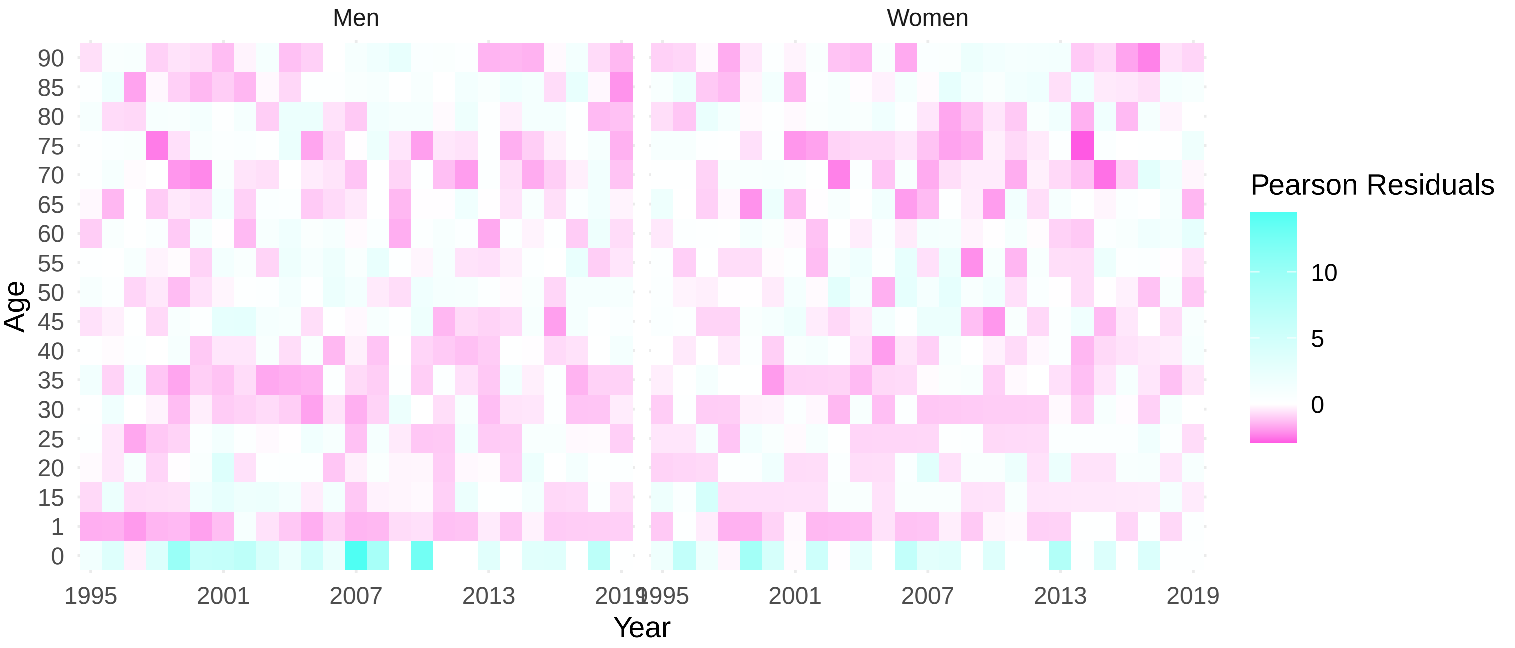

Germany − Wunsiedel i.Fichtelgebirge (9479)

Pearson residuals for death rates modeled with 2D smoothing with P−splines.

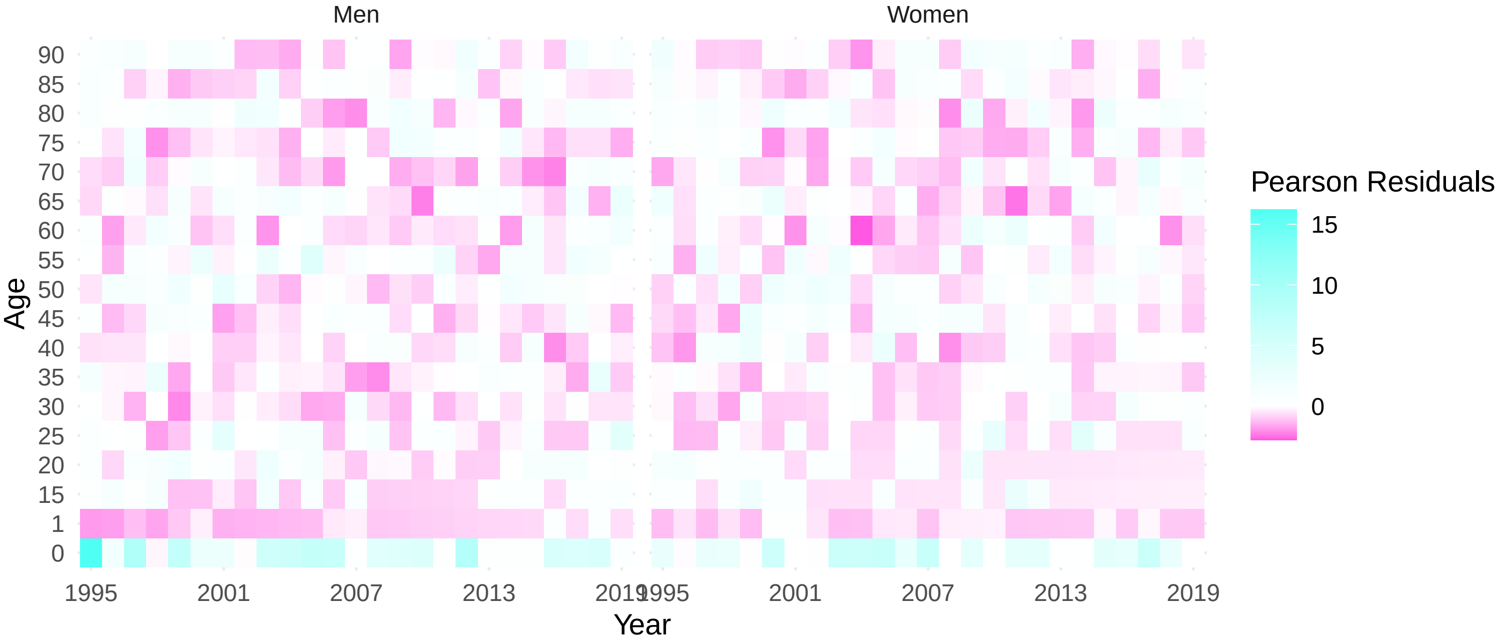

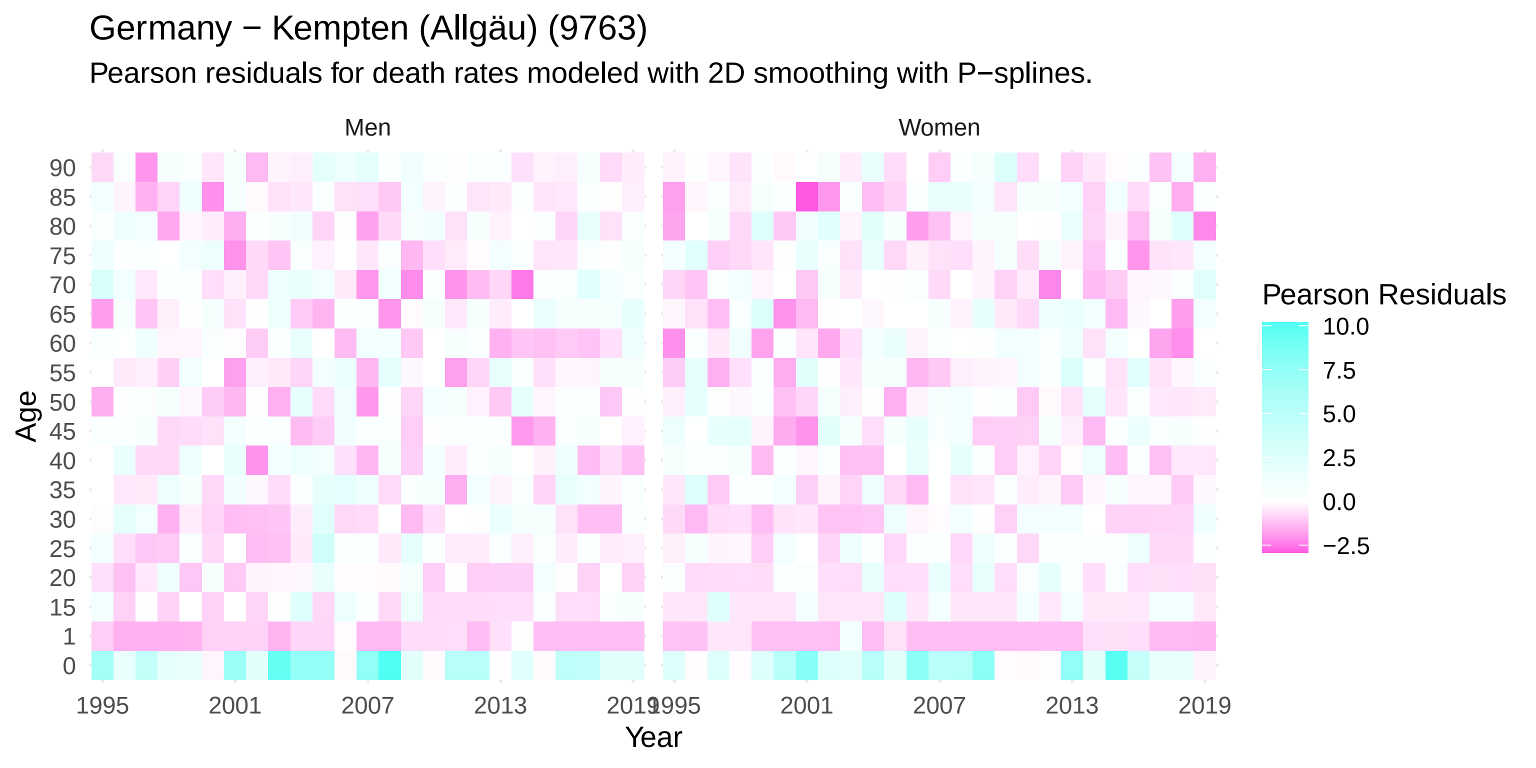

Germany − Kempten (Allgäu) (9763)
Pearson residuals for death rates modeled with 2D smoothing with P−splines.
Men
Women
Age
90
85
80
75
70
65
60
55
50
45
40
35
30
25
20
15
1
0
1995
2001
2007
2013
2019
Year
Pearson Residuals
10.0
7.5
5.0
2.5
0.0
−2.5

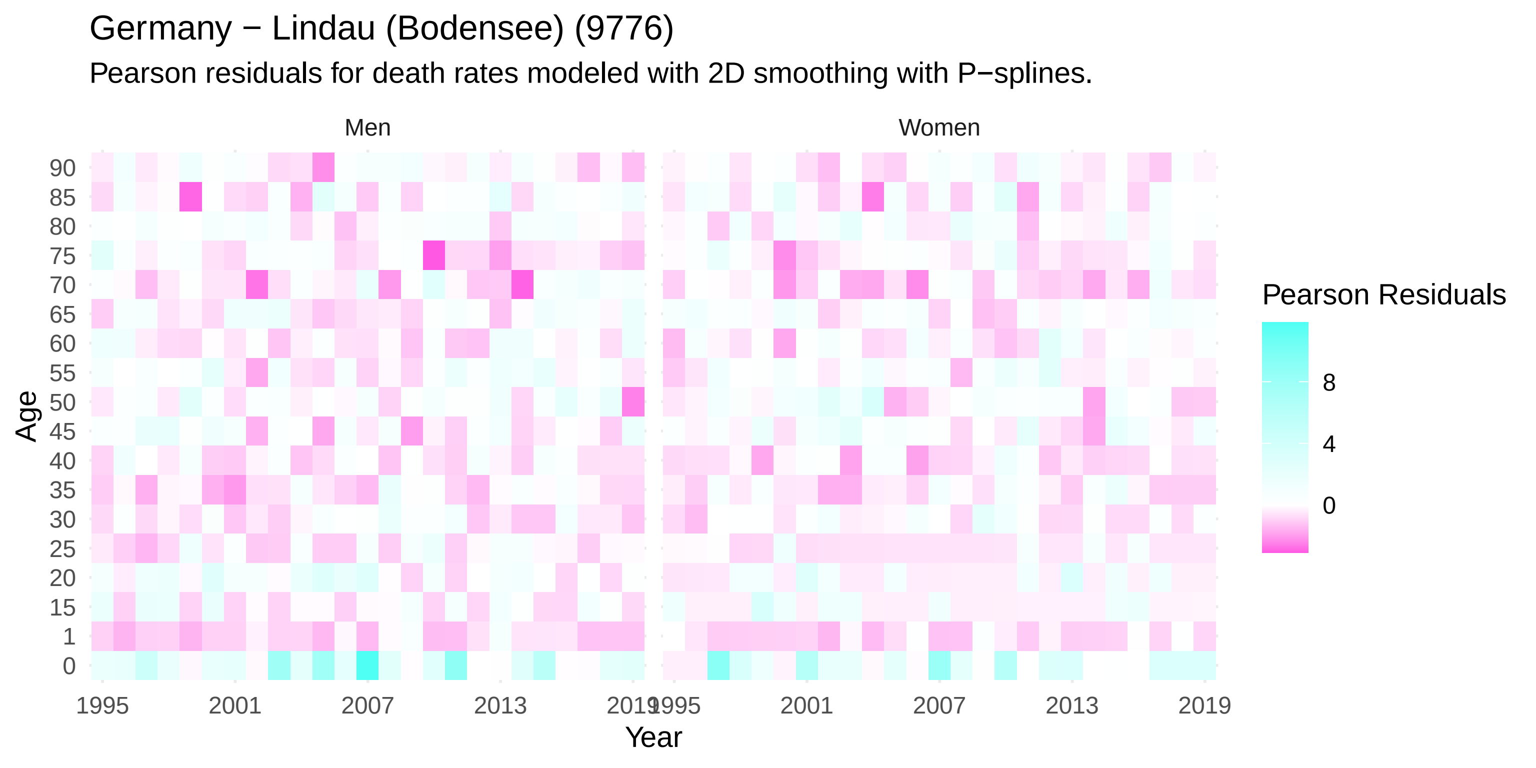

Germany − Lindau (Bodensee) (9776)
Pearson residuals for death rates modeled with 2D smoothing with P−splines.
Men
Women
Age
90
85
80
75
70
65
60
55
50
45
40
35
30
25
20
15
1
0
1995
2001
2007
2013
2019
Year
Pearson Residuals
8
4
0

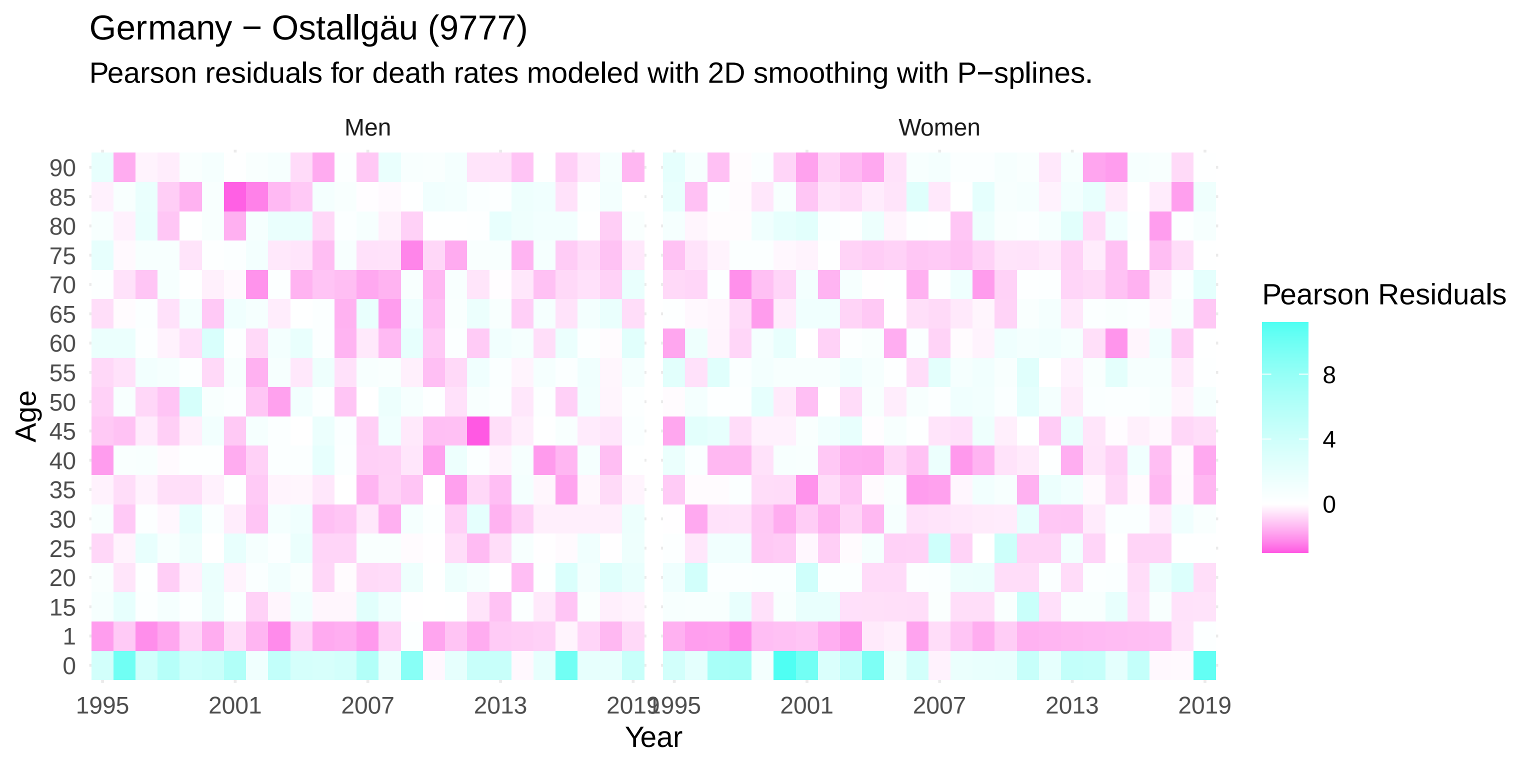
Germany − Ostallgäu (9777)
Pearson residuals for death rates modeled with 2D smoothing with P−splines.
Men
Women
Age
90
85
80
75
70
65
60
55
50
45
40
35
30
25
20
15
1
0
1995
2001
2007
2013
2019
Year
Pearson Residuals
8
4
0

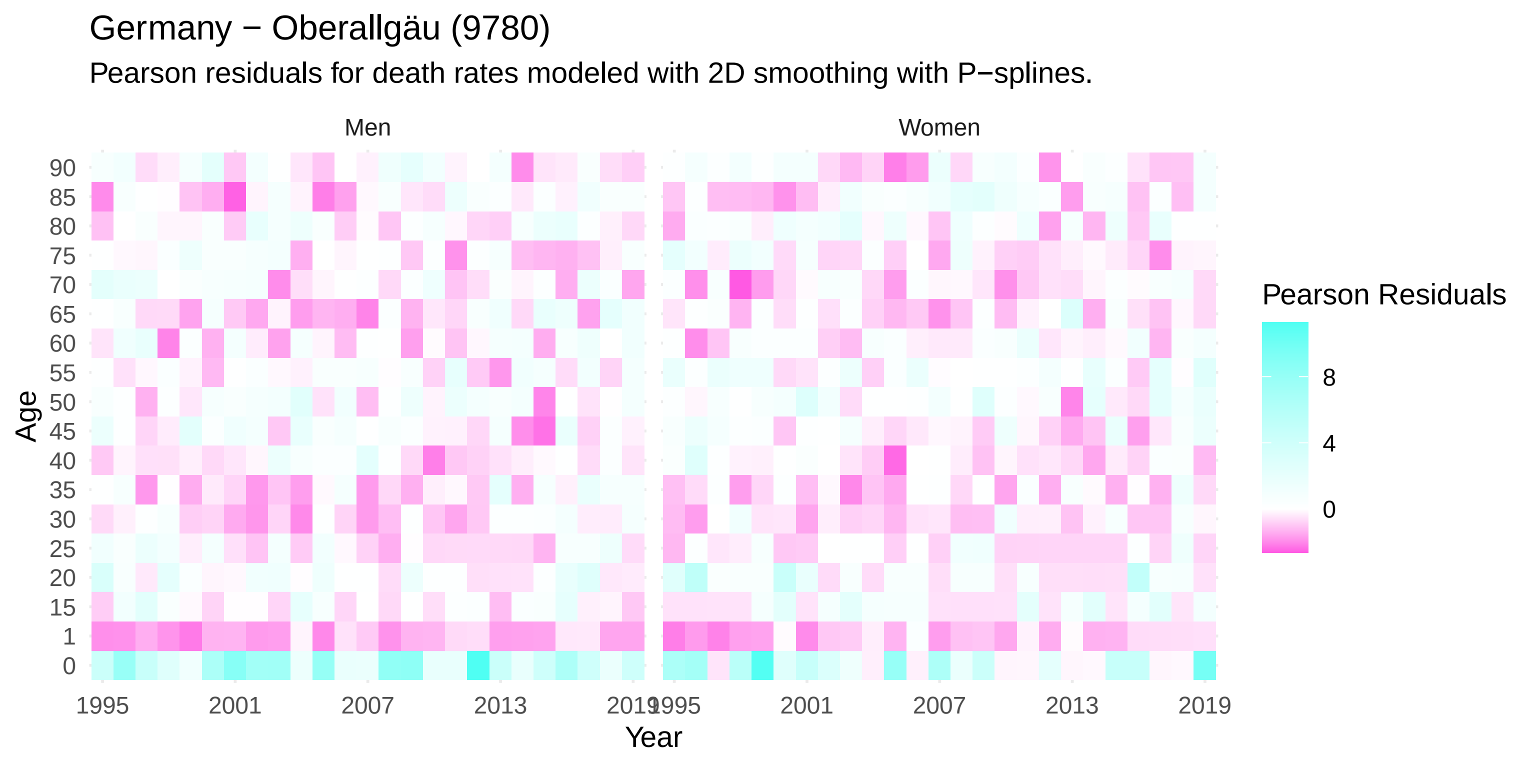

Germany − Oberallgäu (9780)
Pearson residuals for death rates modeled with 2D smoothing with P−splines.
Men
Women
Age
90
85
80
75
70
65
60
55
50
45
40
35
30
25
20
15
1
0
1995
2001
2007
2013
2019
Year
Pearson Residuals
8
4
0

Sweden − Skåne län (SE224)

Pearson residuals for death rates modeled with 2D smoothing with P−splines.

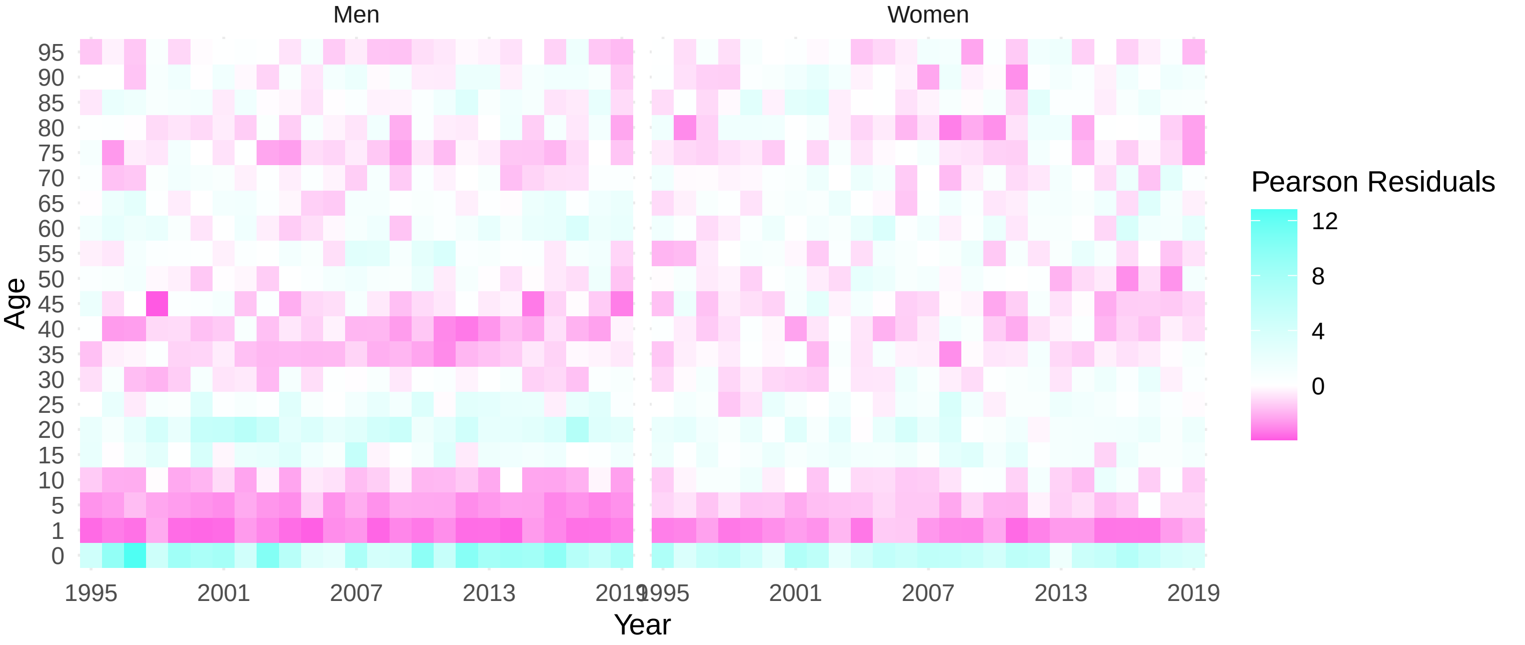

Sweden − Västra Götalands län (SE232)

Pearson residuals for death rates modeled with 2D smoothing with P−splines.

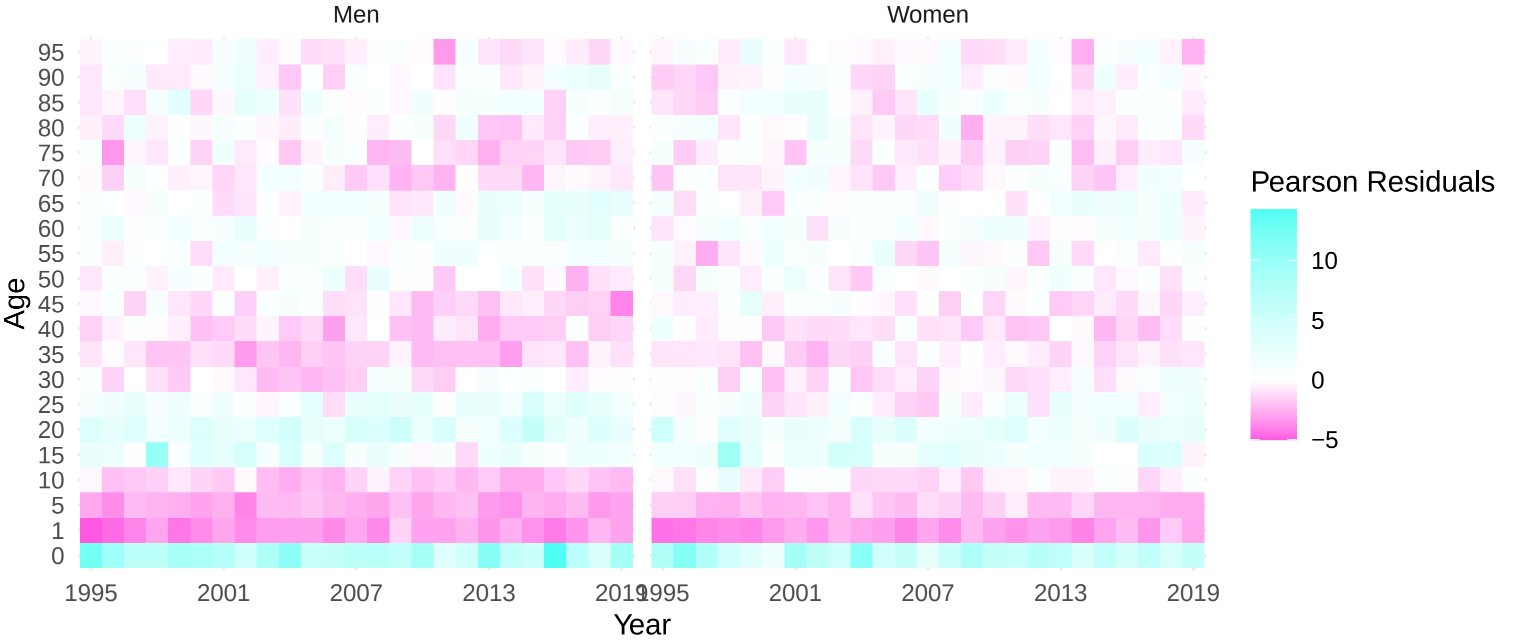

Sweden − Värmlands län (SE311)

Pearson residuals for death rates modeled with 2D smoothing with P−splines.

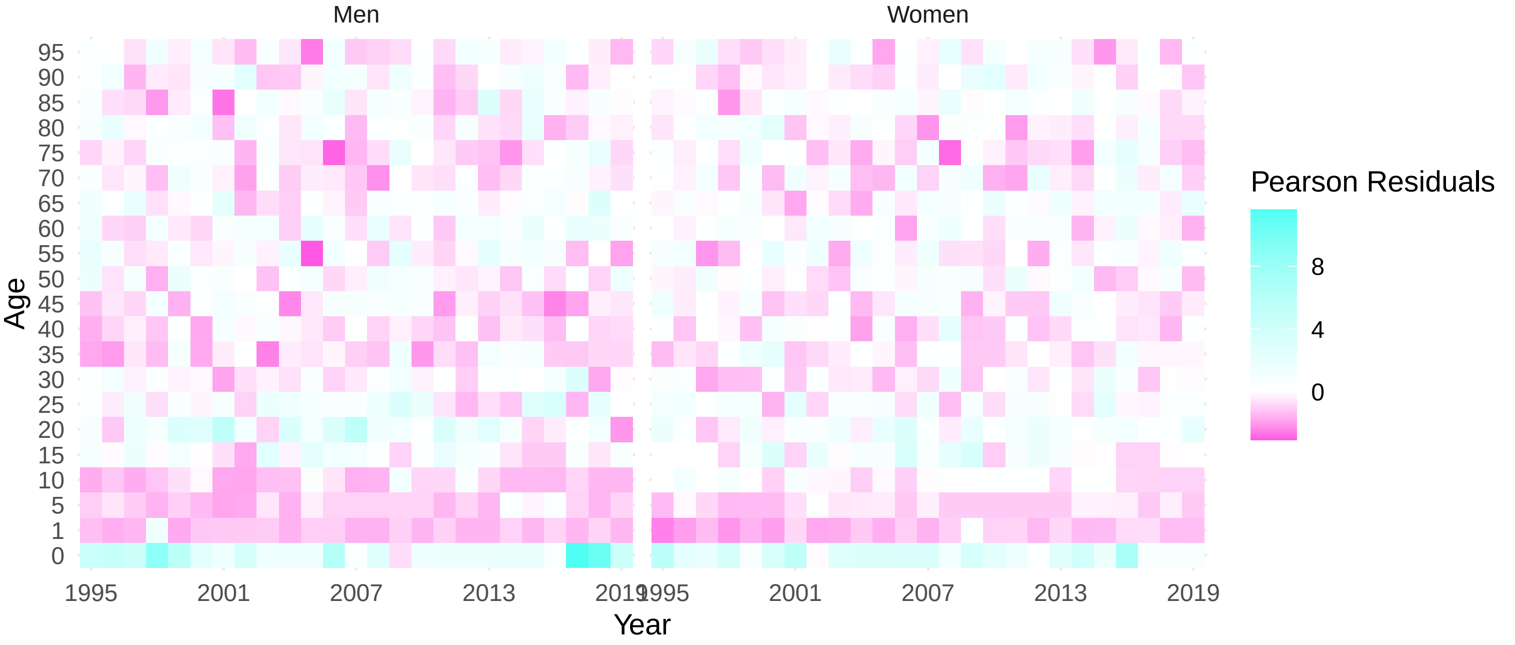

Sweden − Dalarnas län (SE312)

Pearson residuals for death rates modeled with 2D smoothing with P−splines.

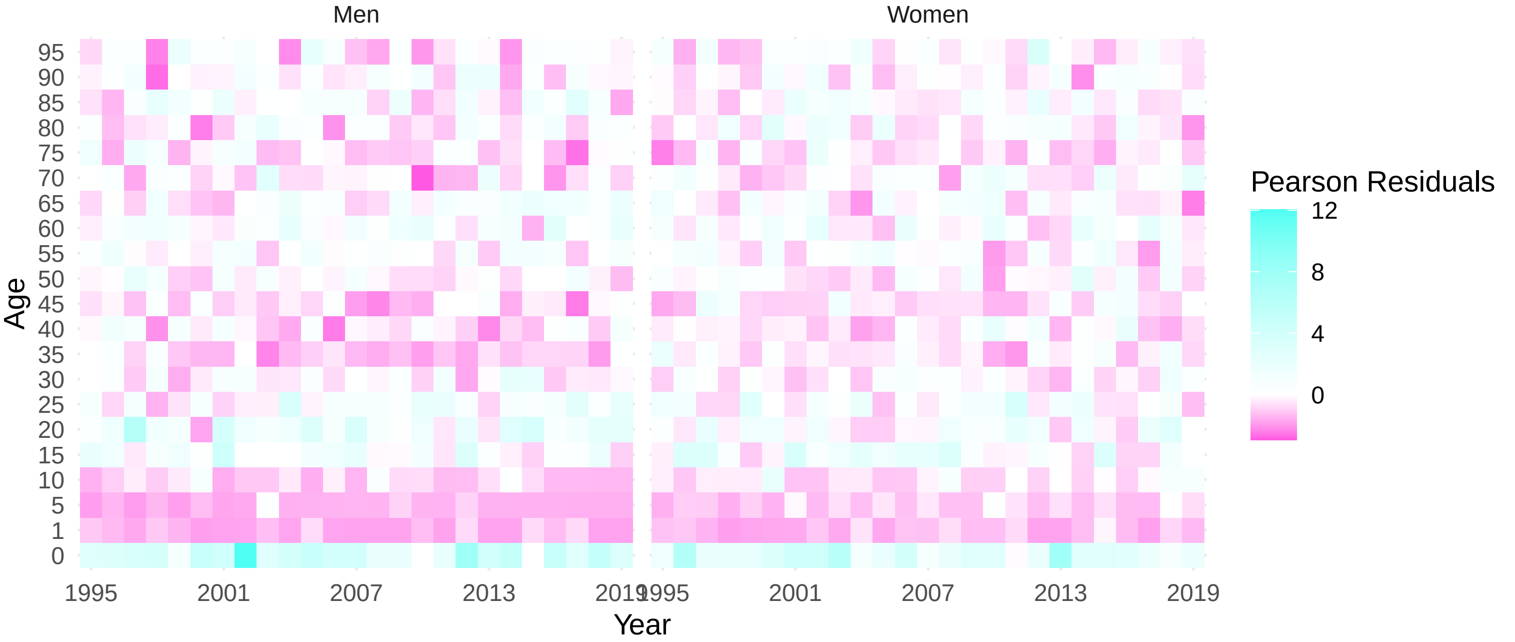

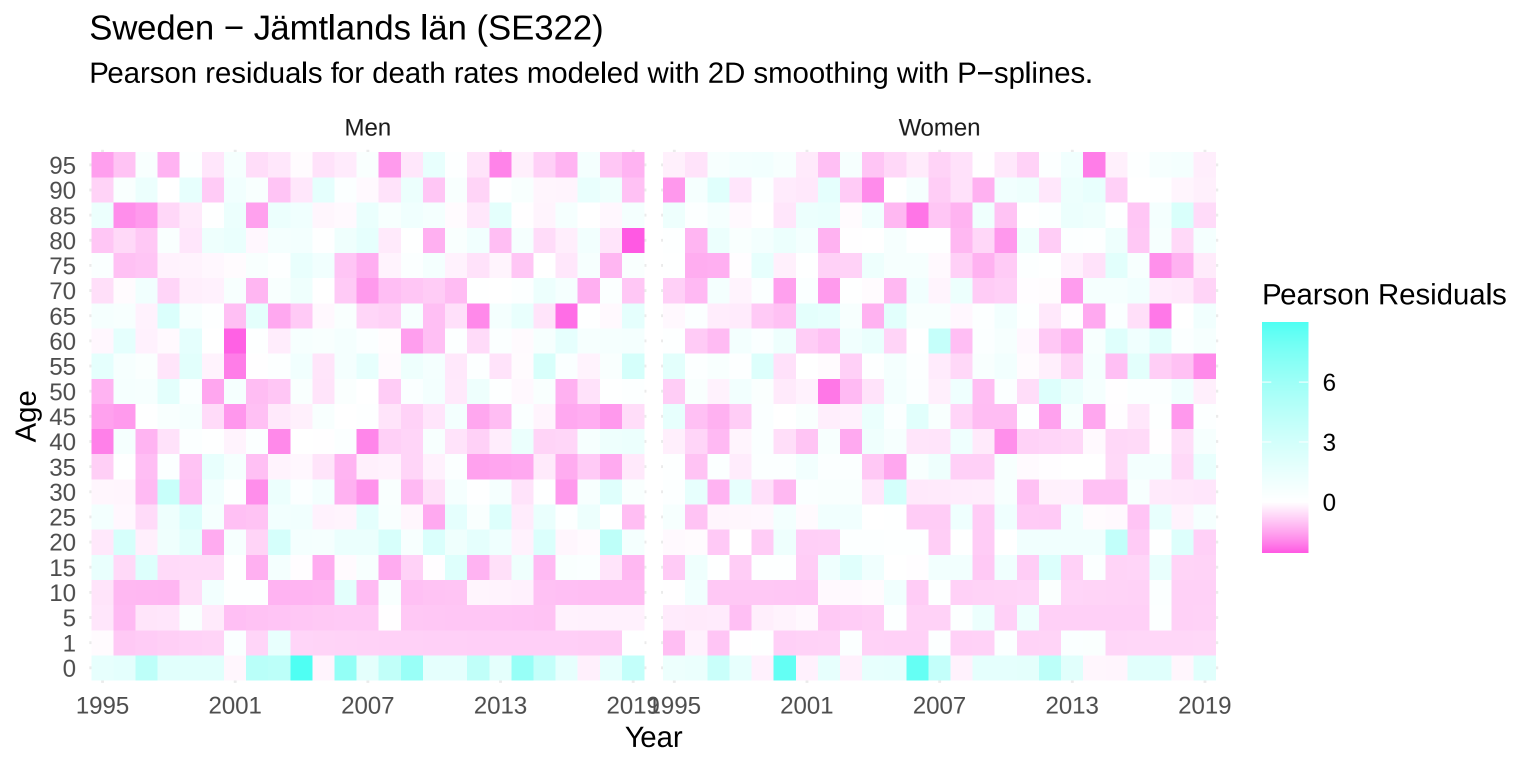
Sweden − Jämtlands län (SE322)
Pearson residuals for death rates modeled with 2D smoothing with P−splines.
Men
Women
Age
95
90
85
80
75
70
65
60
55
50
45
40
35
30
25
20
15
10
5
1
0
1995
2001
2007
2013
2019
Year
Pearson Residuals
6
3
0

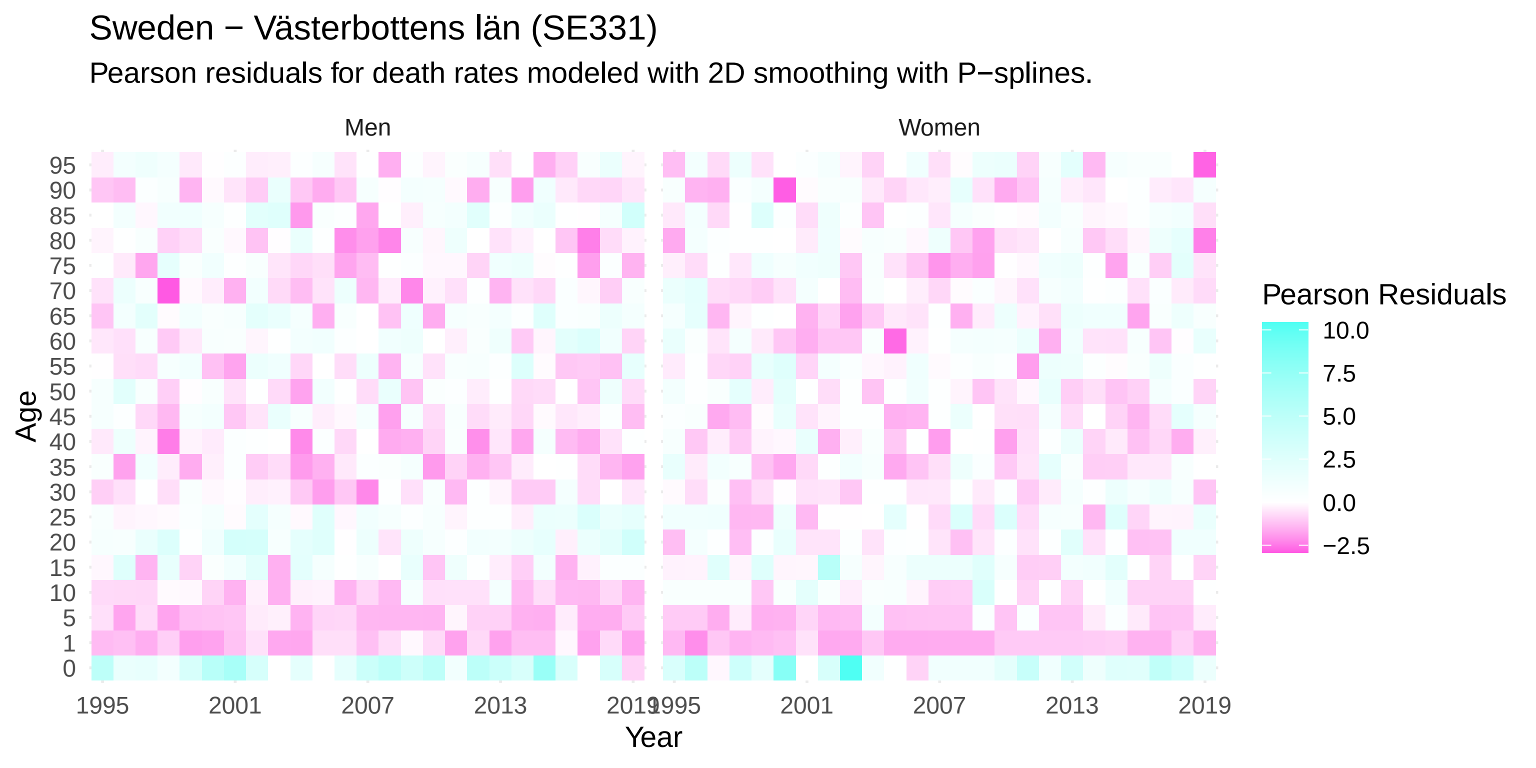

Sweden − Västerbottens län (SE331)
Pearson residuals for death rates modeled with 2D smoothing with P−splines.
Men
Women
Age
95
90
85
80
75
70
65
60
55
50
45
40
35
30
25
20
15
10
5
1
0
1995
2001
2007
2013
2019
Year
Pearson Residuals
10.0
7.5
5.0
2.5
0.0
−2.5

Sweden − Norrbottens län (SE332)

Pearson residuals for death rates modeled with 2D smoothing with P−splines.

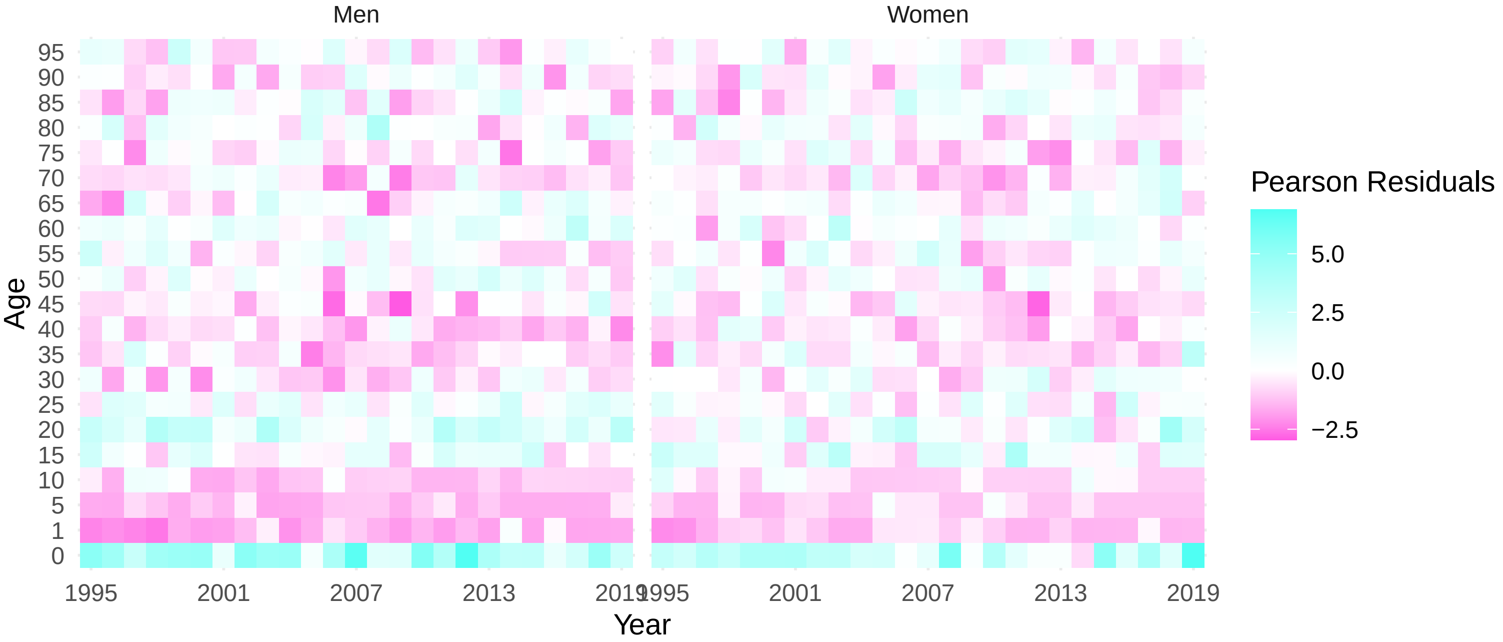

Spain − Ourense (ES113)

Pearson residuals for death rates modeled with 2D smoothing with P−splines.

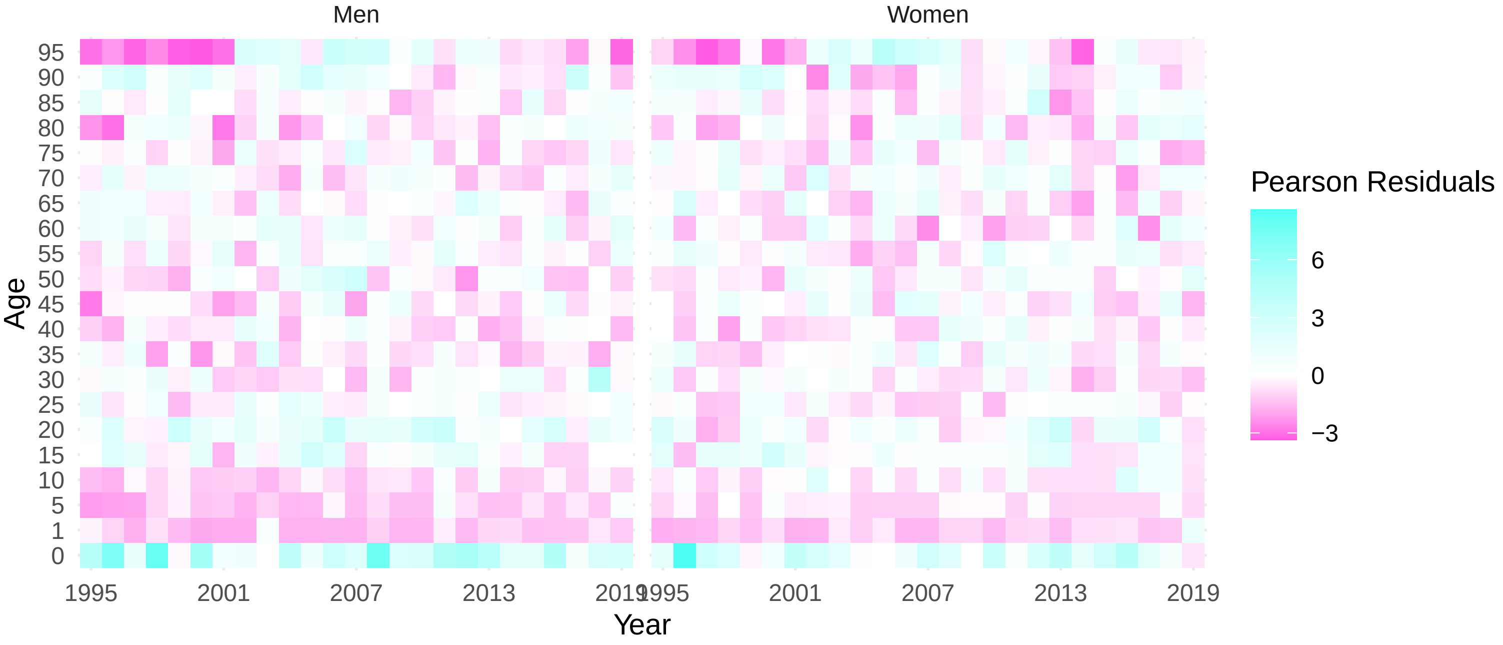

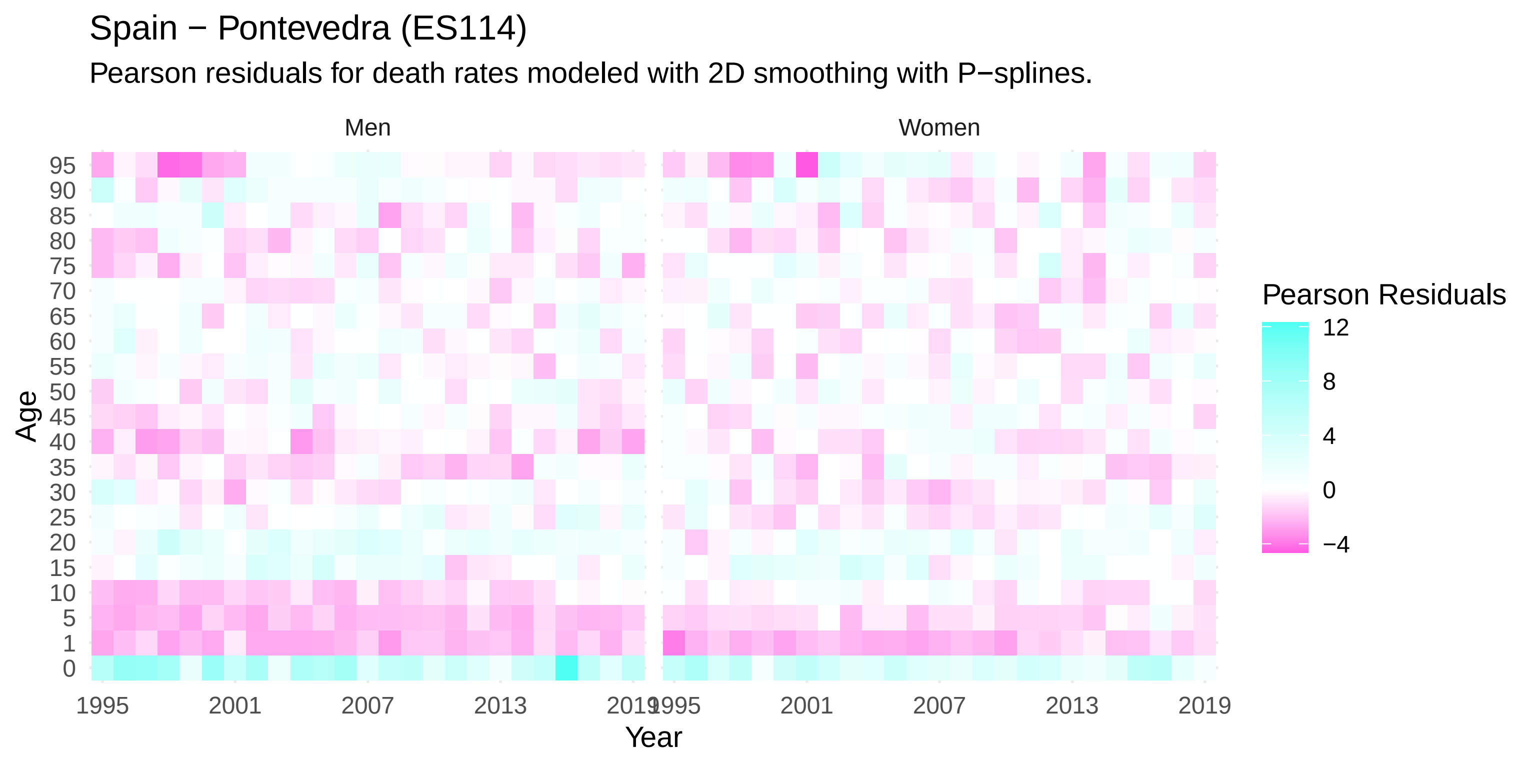

Spain − Pontevedra (ES114)
Pearson residuals for death rates modeled with 2D smoothing with P−splines.
Men
Women
Age
Year
Pearson Residuals
12
8
4
0
−4
95
90
85
80
75
70
65
60
55
50
45
40
35
30
25
20
15
10
5
1
0
1995
2001
2007
2013
2019

Spain − Gipuzkoa (ES212)

Pearson residuals for death rates modeled with 2D smoothing with P−splines.

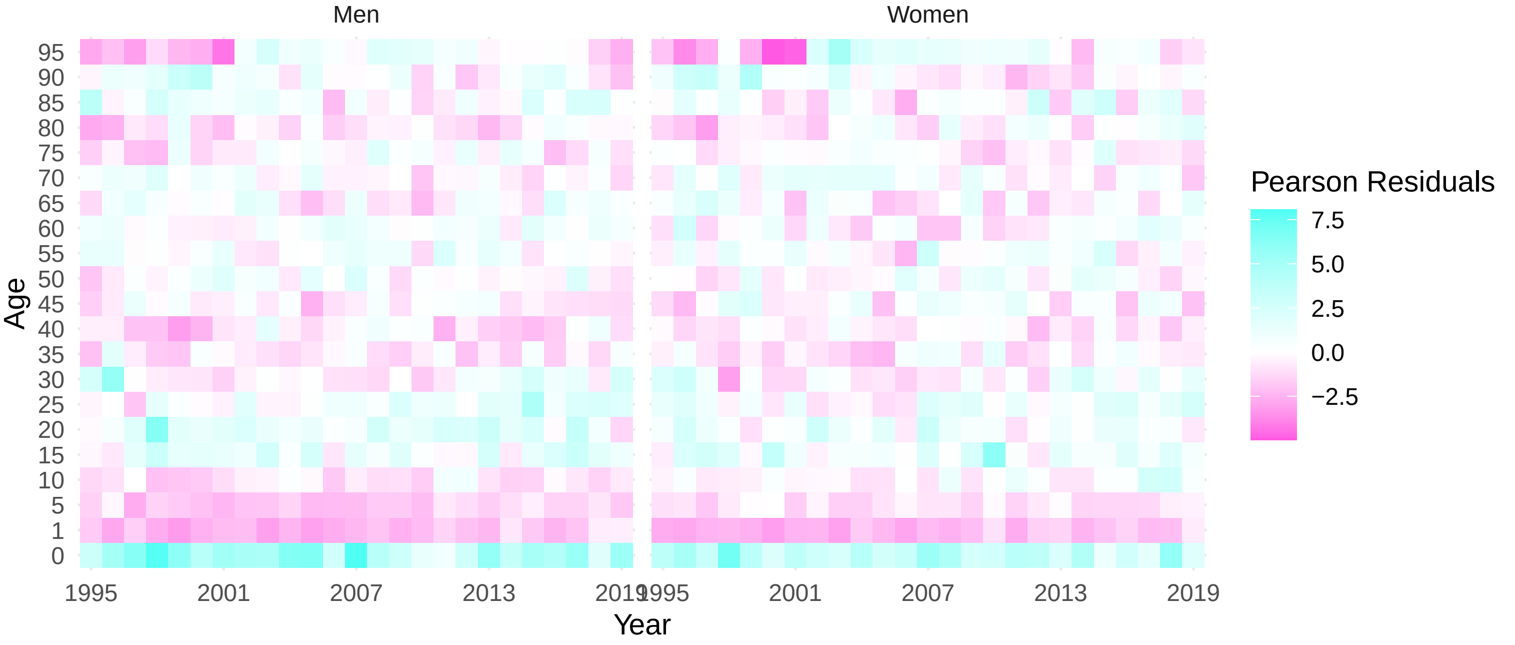

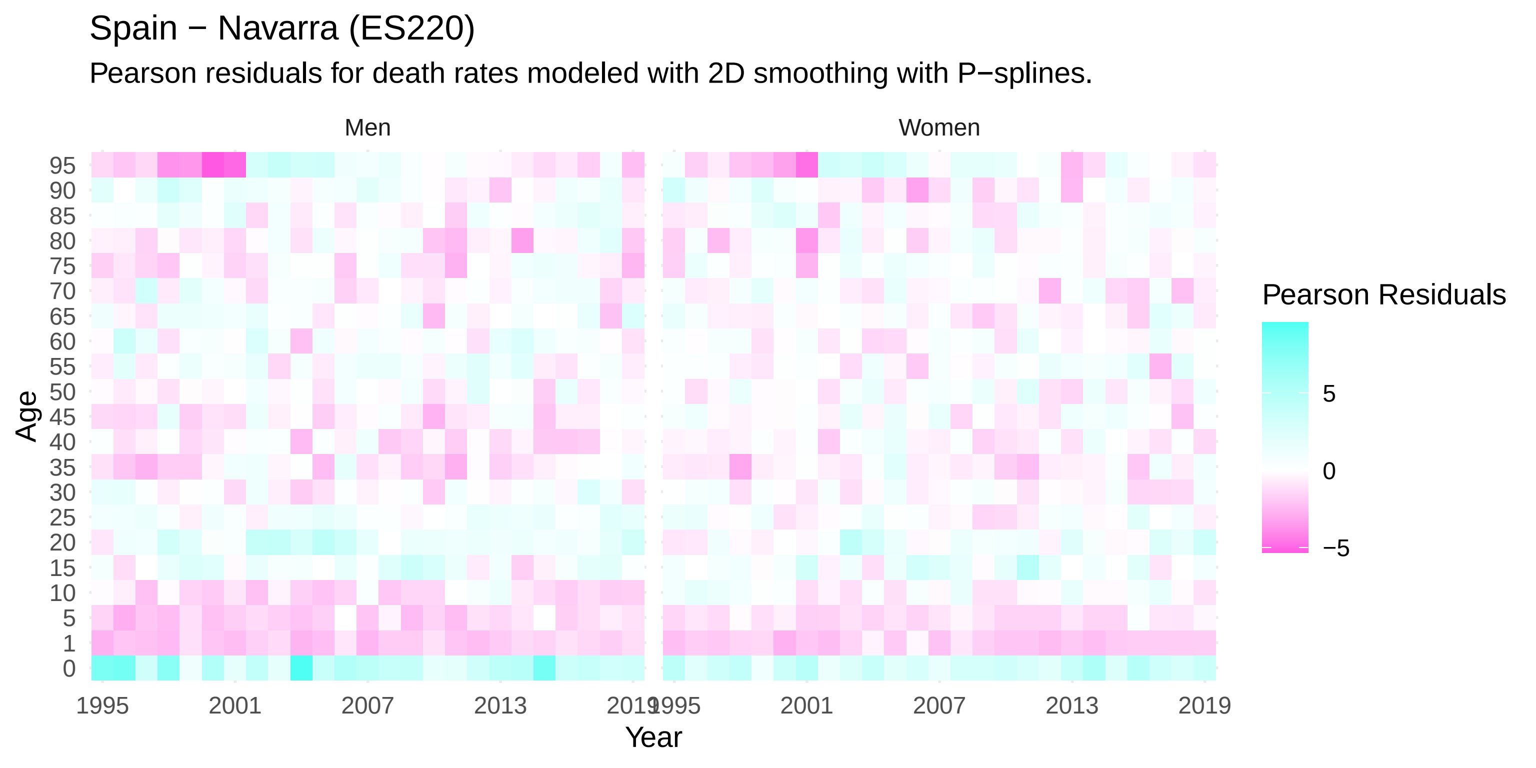

Spain − Navarra (ES220)
Pearson residuals for death rates modeled with 2D smoothing with P−splines.
Men
Women
Age
95
90
85
80
75
70
65
60
55
50
45
40
35
30
25
20
15
10
5
1
0
1995
2001
2007
2013
2019
Year
Pearson Residuals
5
0
−5

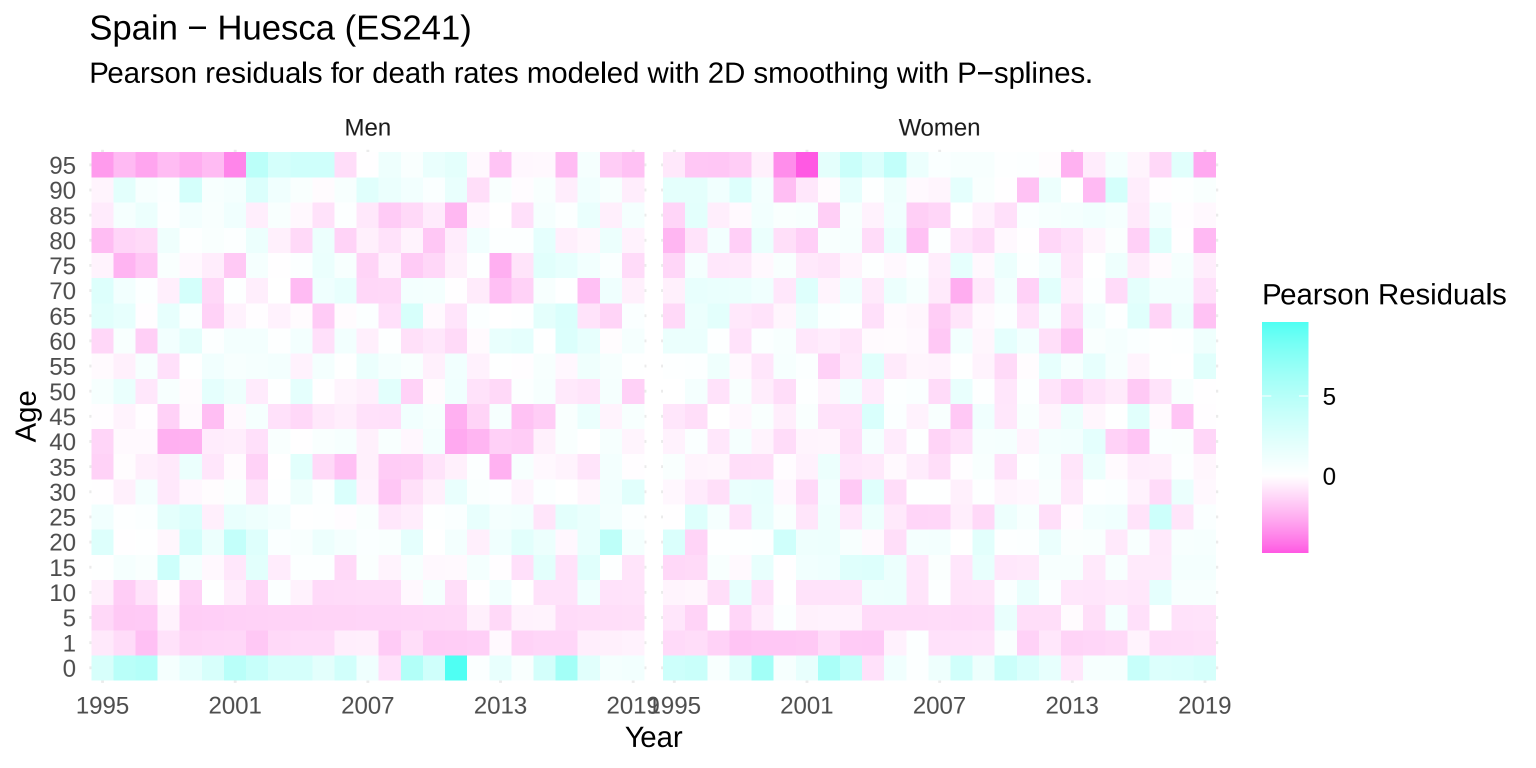

Spain − Huesca (ES241)
Pearson residuals for death rates modeled with 2D smoothing with P−splines.
Men
Women
Age
95
90
85
80
75
70
65
60
55
50
45
40
35
30
25
20
15
10
5
1
0
1995
2001
2007
2013
2019
Year
Pearson Residuals
5
0

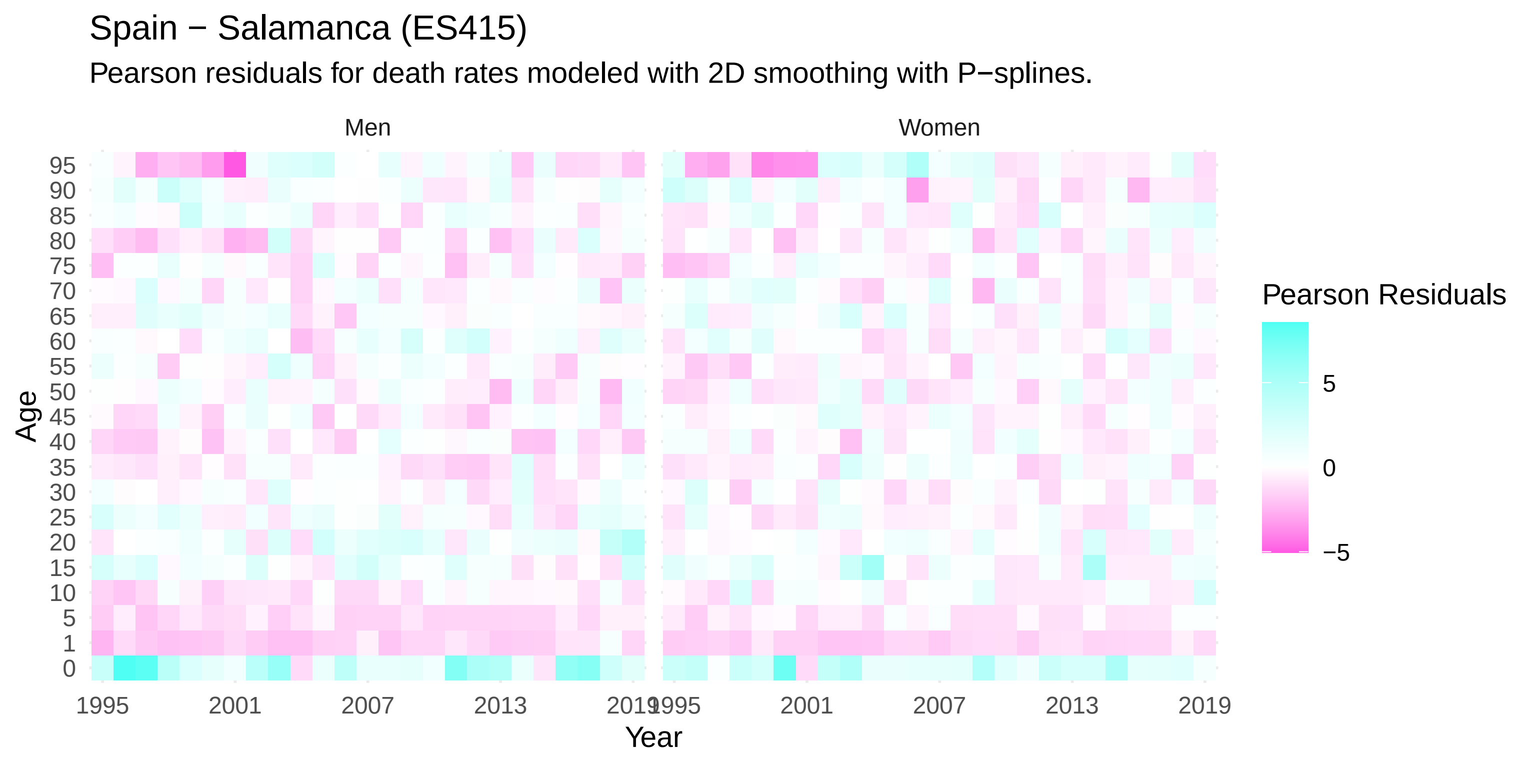

Spain − Salamanca (ES415)
Pearson residuals for death rates modeled with 2D smoothing with P−splines.
Men
Women
Age
95
90
85
80
75
70
65
60
55
50
45
40
35
30
25
20
15
10
5
1
0
1995
2001
2007
2013
2019
Year
Pearson Residuals
5
0
−5

Spain − Zamora (ES419)

Pearson residuals for death rates modeled with 2D smoothing with P−splines.

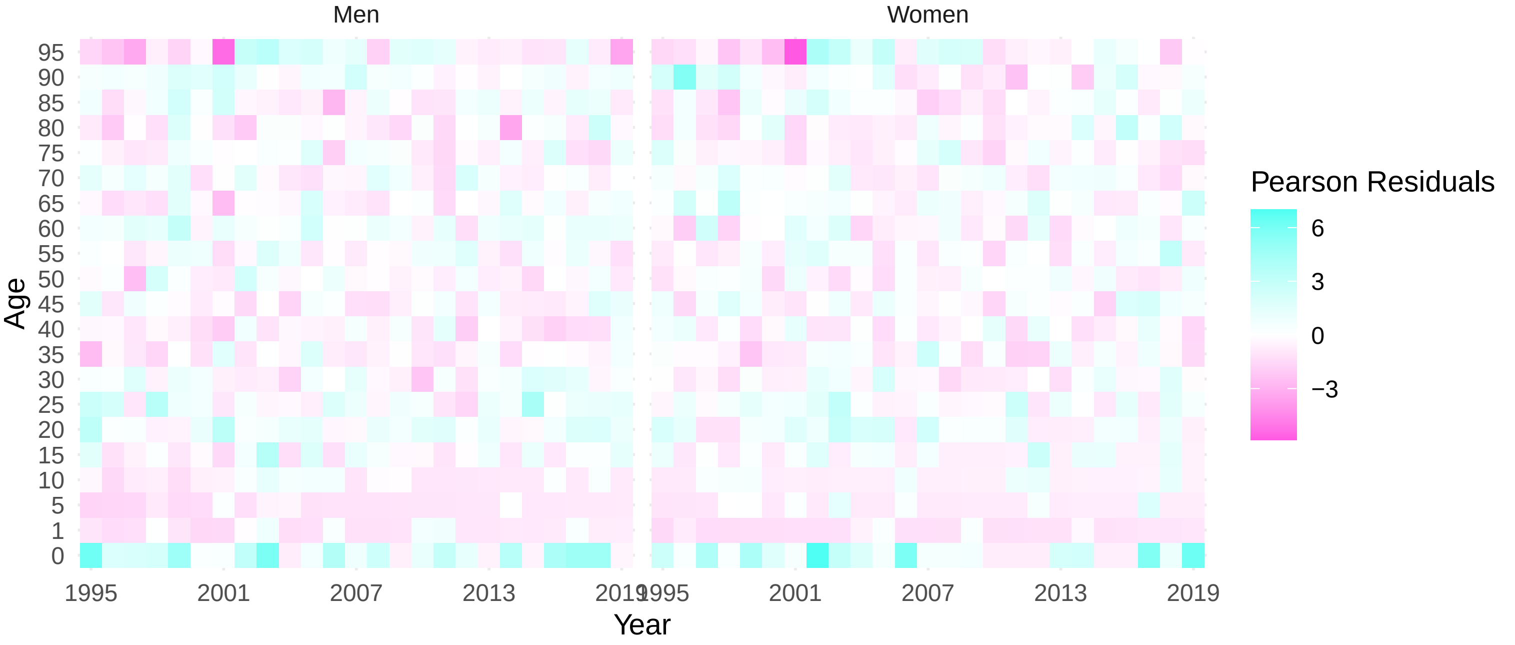

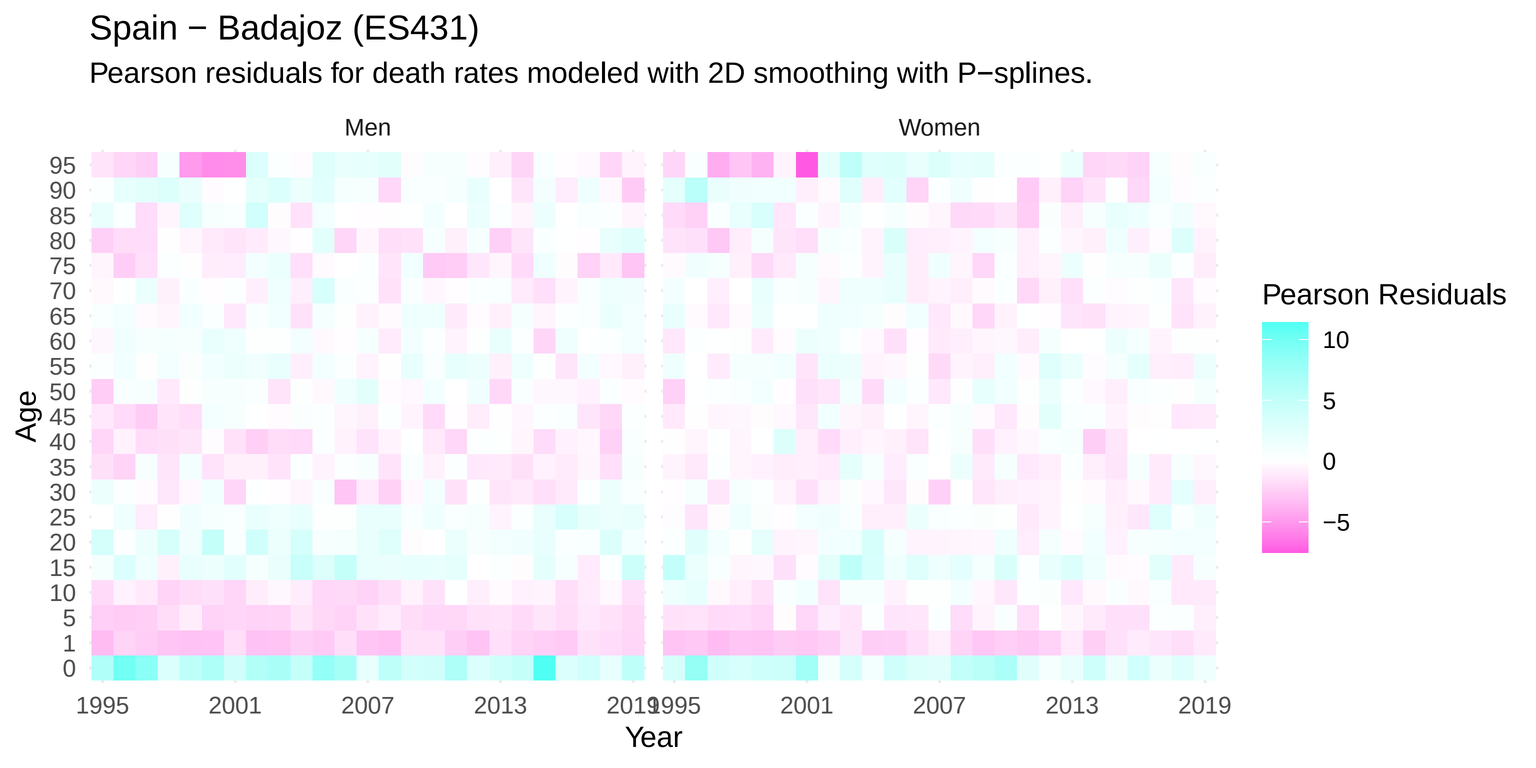
Spain − Badajoz (ES431)
Pearson residuals for death rates modeled with 2D smoothing with P−splines.
Men
Women
Age
95
90
85
80
75
70
65
60
55
50
45
40
35
30
25
20
15
10
5
1
0
1995
2001
2007
2013
2019
Year
Pearson Residuals
10
5
0
−5

Spain − Cáceres (ES432)

Pearson residuals for death rates modeled with 2D smoothing with P−splines.

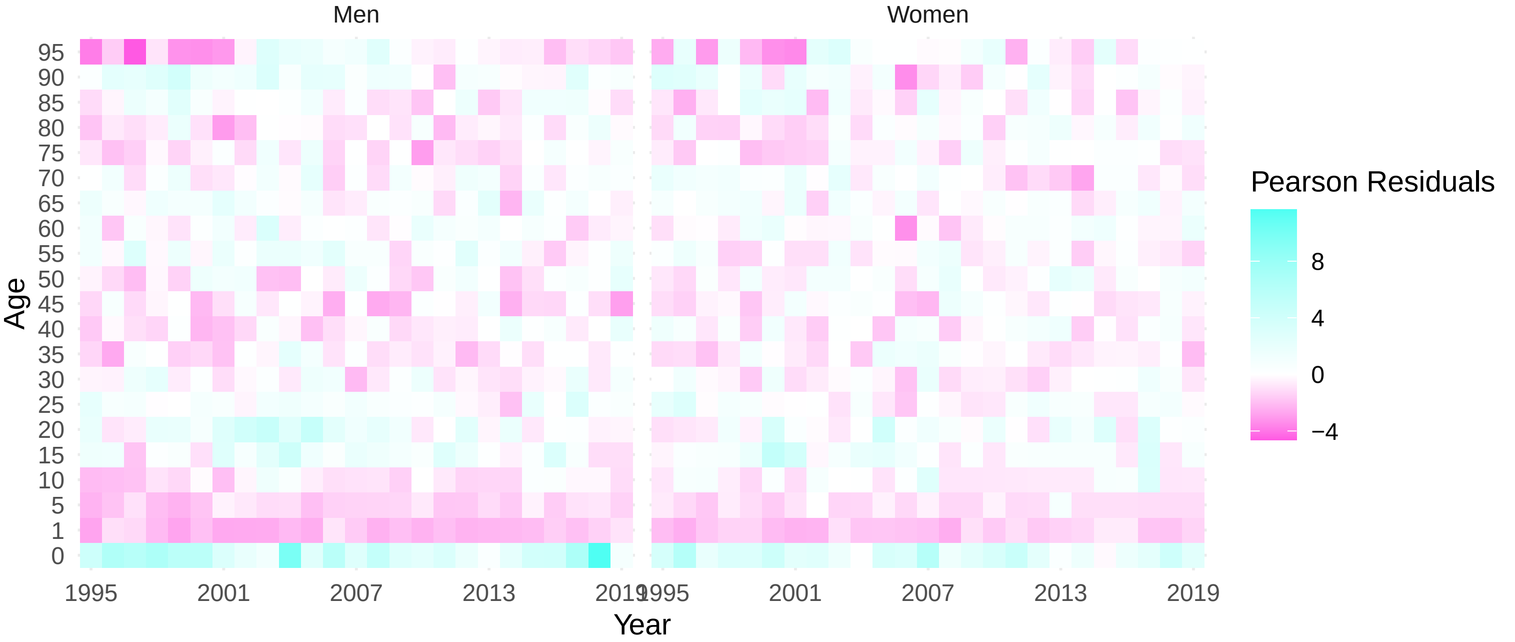

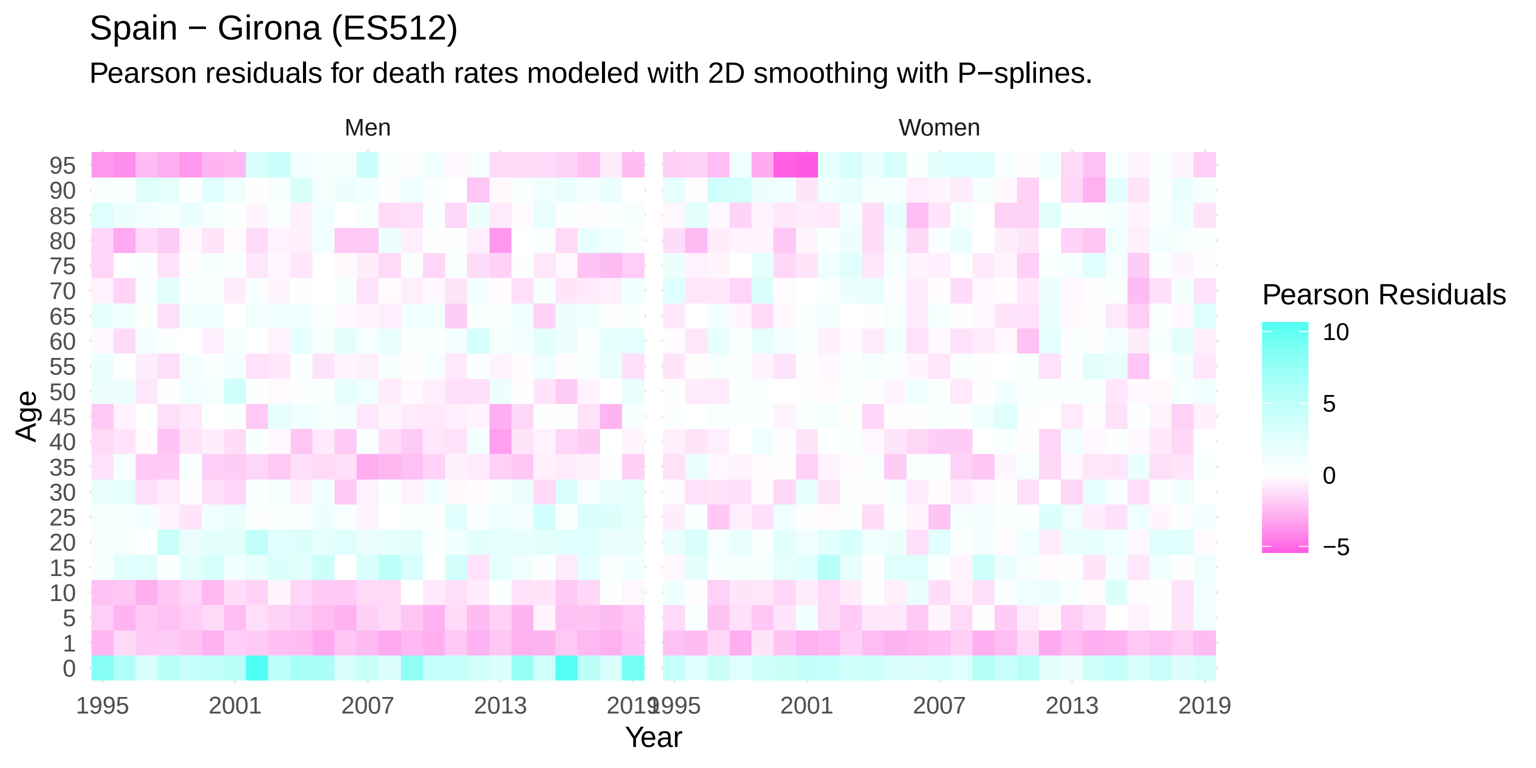
Spain − Girona (ES512)
Pearson residuals for death rates modeled with 2D smoothing with P−splines.
Men
Women
Age
95
90
85
80
75
70
65
60
55
50
45
40
35
30
25
20
15
10
5
1
0
1995
2001
2007
2013
2019
Year
Pearson Residuals
10
5
0
−5

Spain − Lleida (ES513)

Pearson residuals for death rates modeled with 2D smoothing with P−splines.

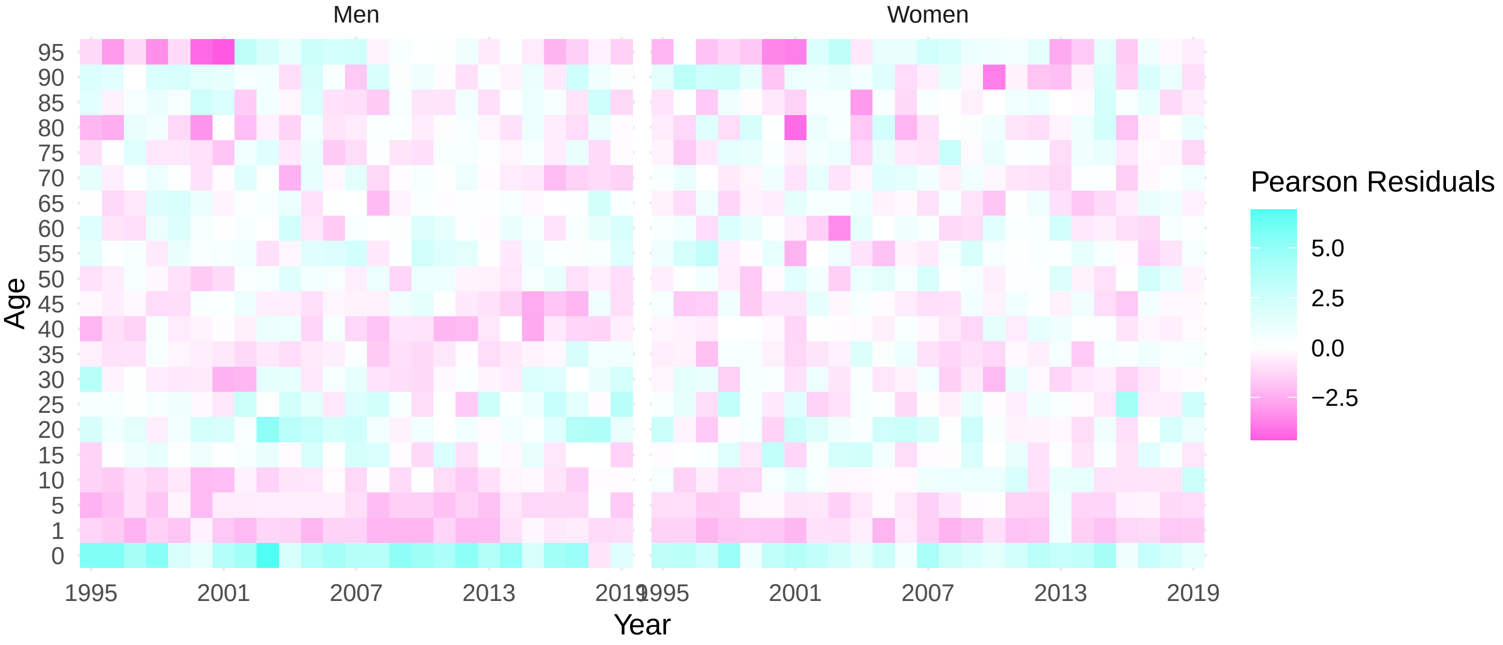

Spain − Huelva (ES615)

Pearson residuals for death rates modeled with 2D smoothing with P−splines.

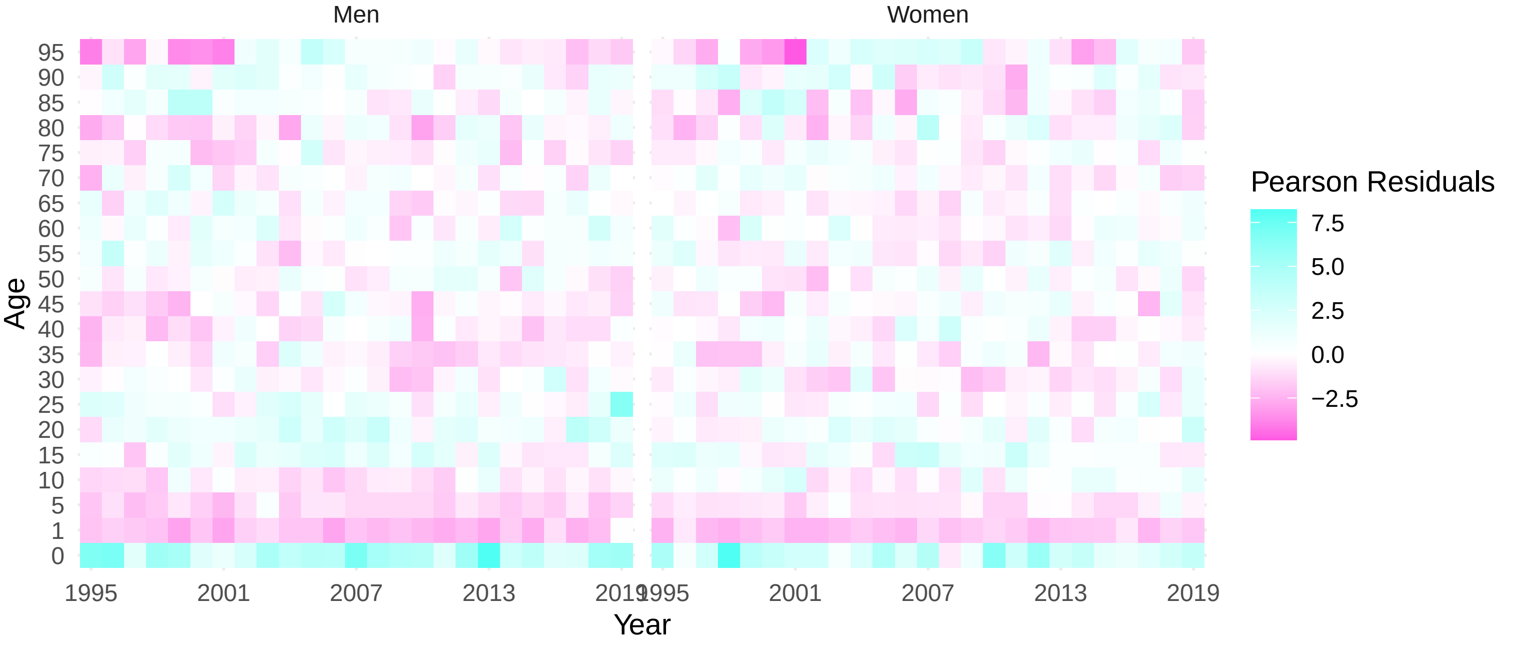

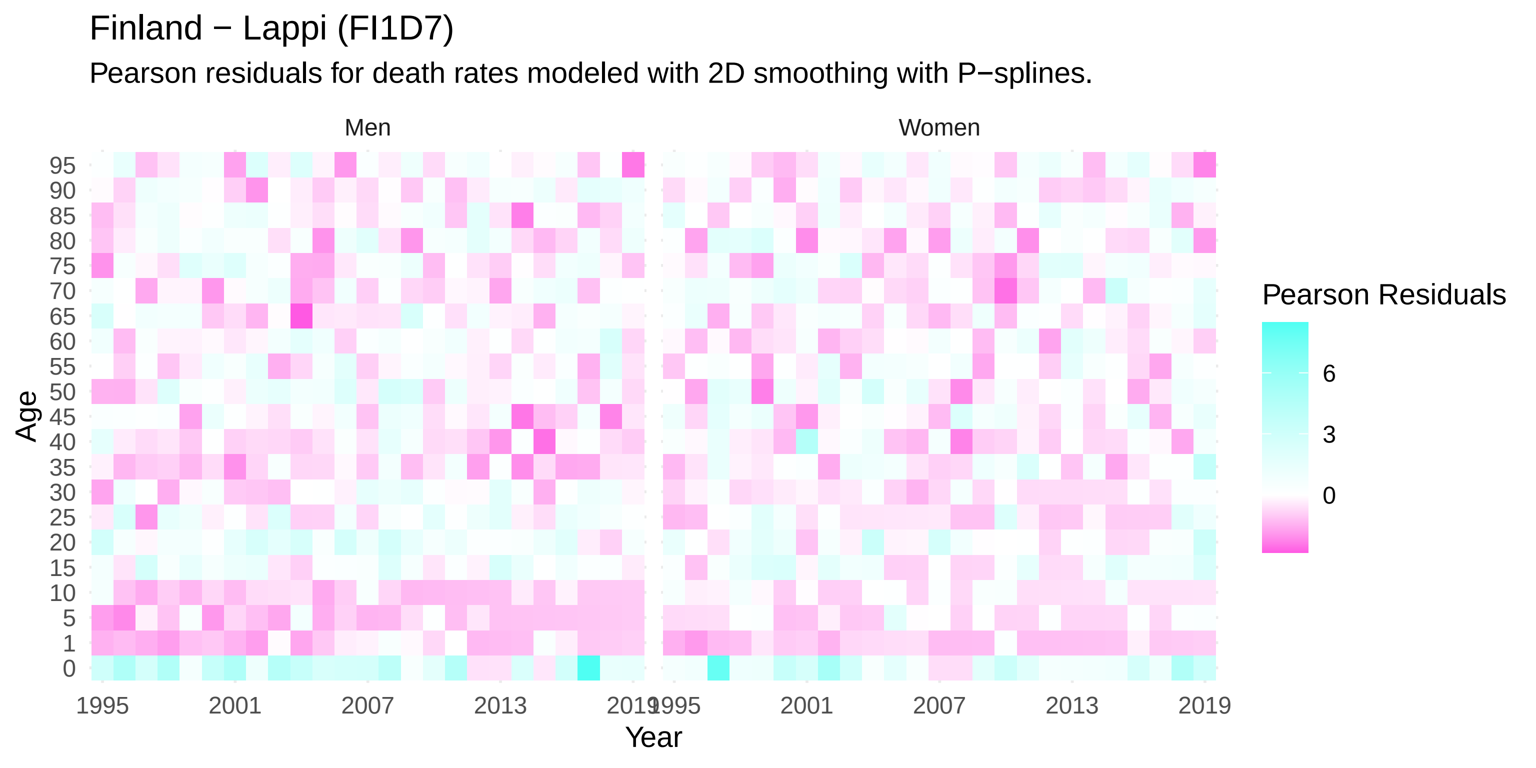
Finland − Lappi (FI1D7)
Pearson residuals for death rates modeled with 2D smoothing with P−splines.
Men
Women
Age
95
90
85
80
75
70
65
60
55
50
45
40
35
30
25
20
15
10
5
1
0
1995
2001
2007
2013
2019
Year
Pearson Residuals
6
3
0

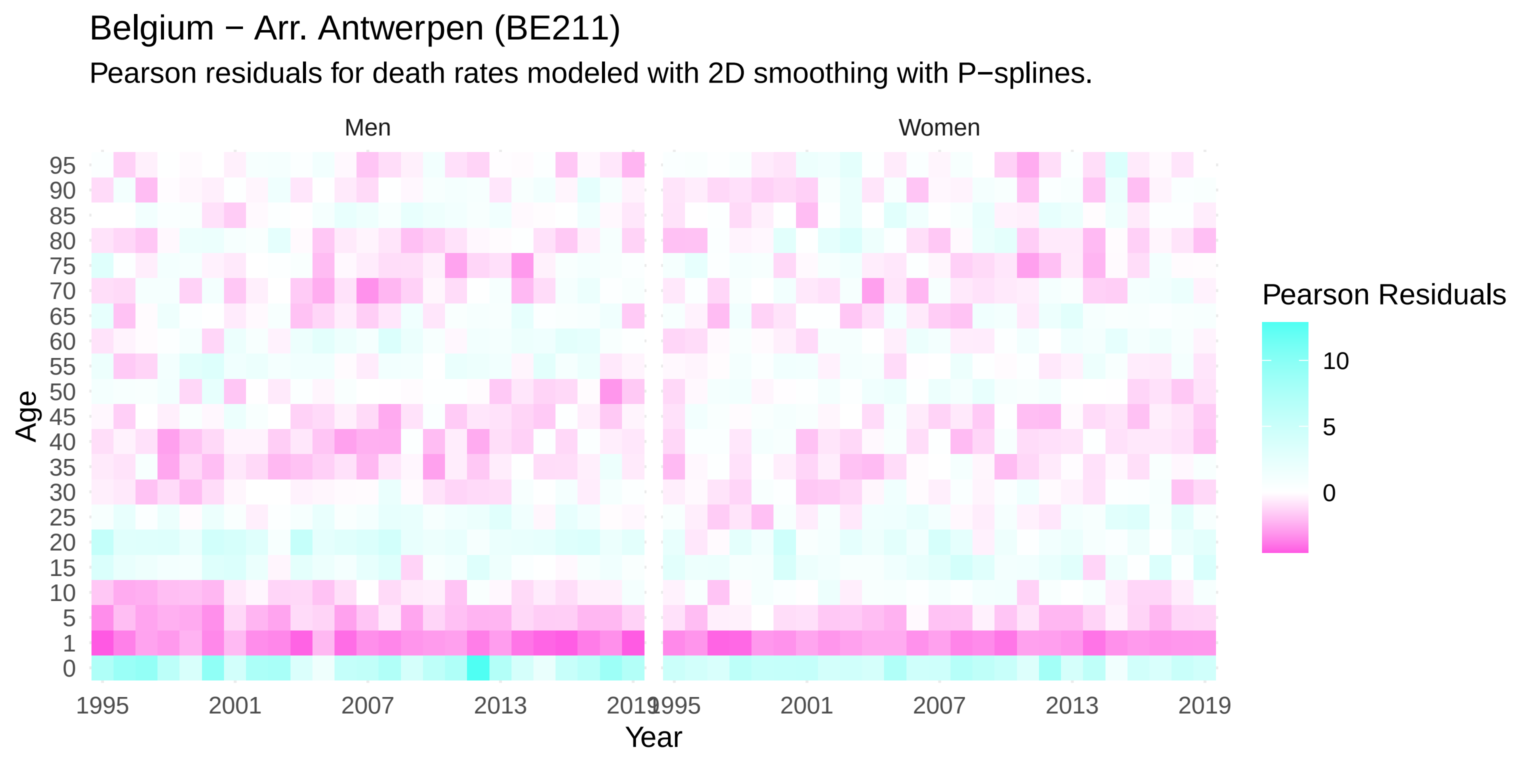
Belgium − Arr. Antwerpen (BE211)
Pearson residuals for death rates modeled with 2D smoothing with P−splines.
Men
Women
Age
95
90
85
80
75
70
65
60
55
50
45
40
35
30
25
20
15
10
5
1
0
1995
2001
2007
2013
2019
Year
Pearson Residuals
10
5
0

Belgium − Arr. Turnhout (BE213)

Pearson residuals for death rates modeled with 2D smoothing with P−splines.

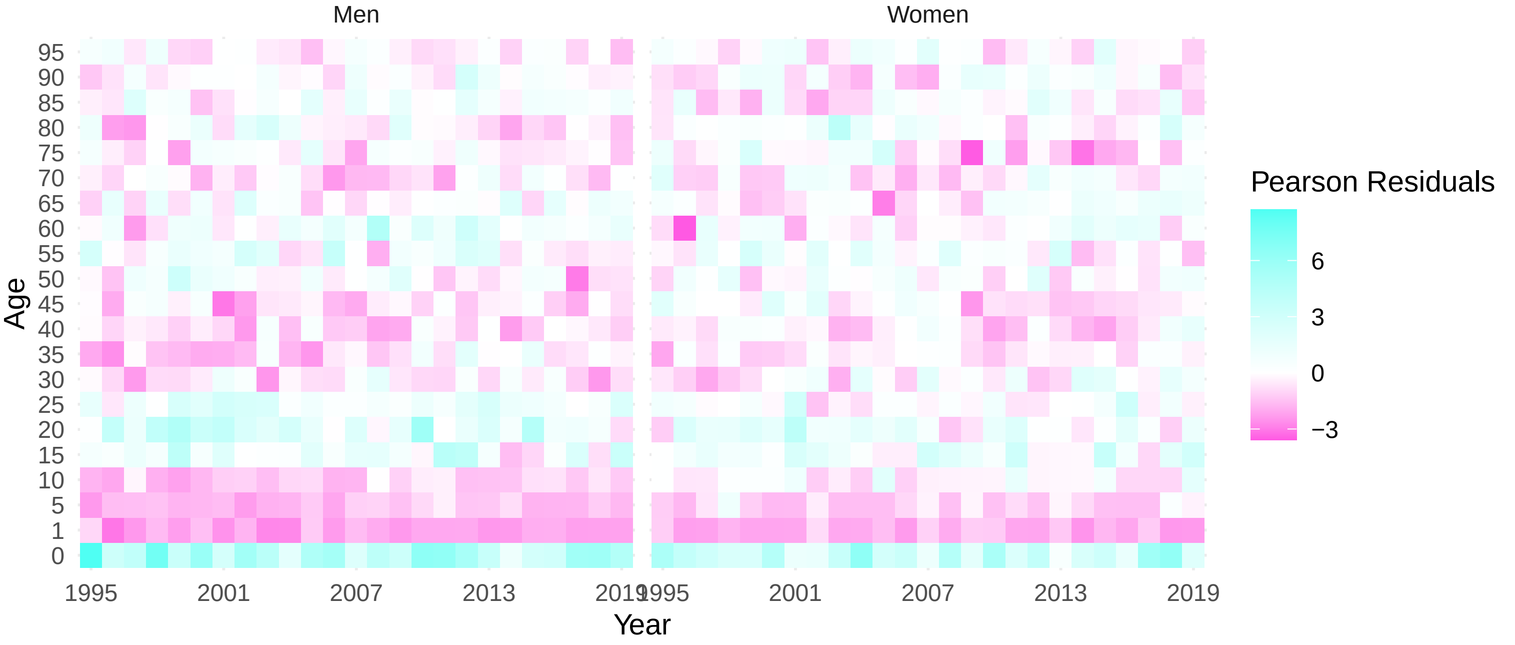

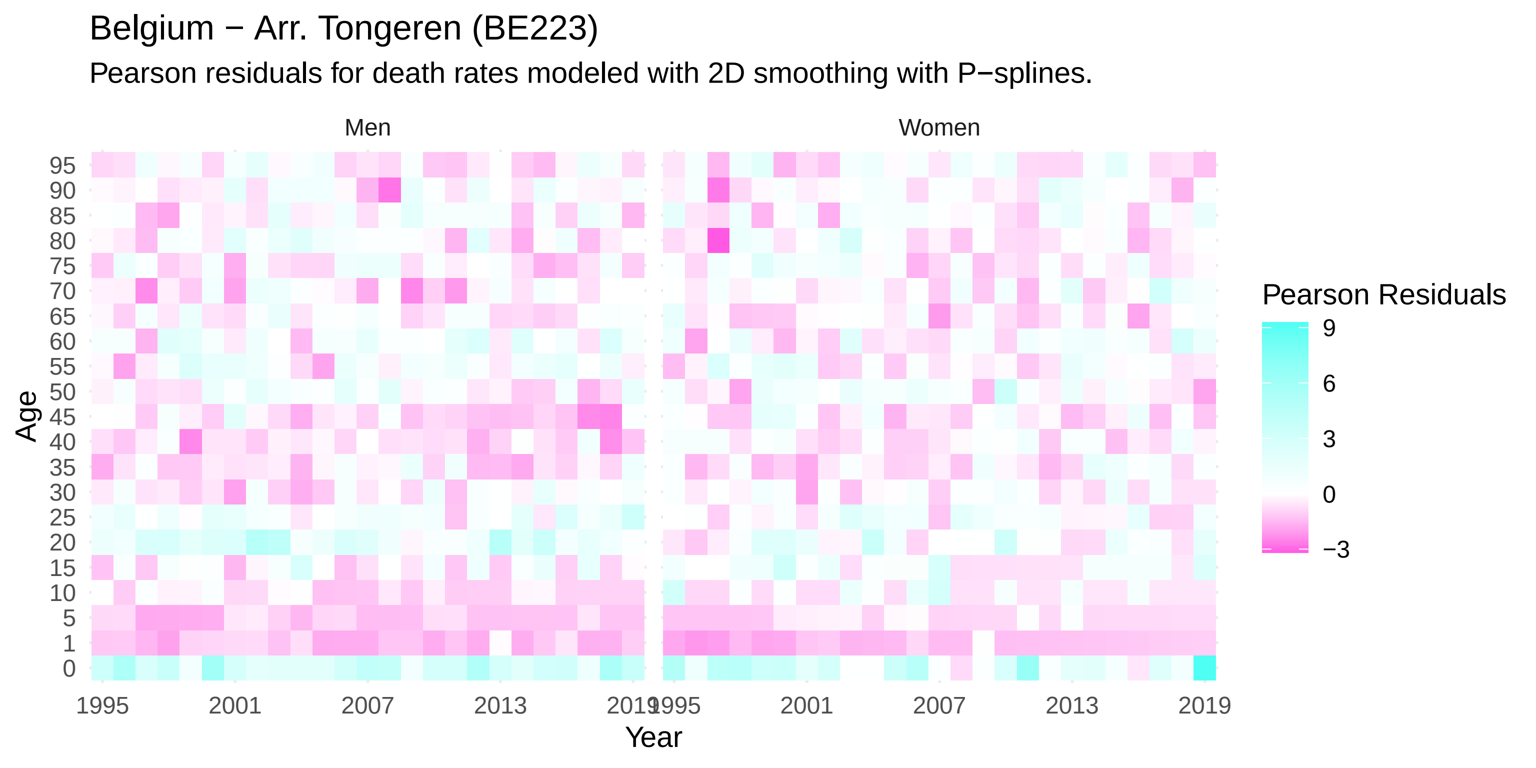
Belgium − Arr. Tongeren (BE223)
Pearson residuals for death rates modeled with 2D smoothing with P−splines.
Men
Women
Age
95
90
85
80
75
70
65
60
55
50
45
40
35
30
25
20
15
10
5
1
0
1995
2001
2007
2013
2019
Year
Pearson Residuals
9
6
3
0
−3

Belgium − Arr. Maaseik (BE225)

Pearson residuals for death rates modeled with 2D smoothing with P−splines.

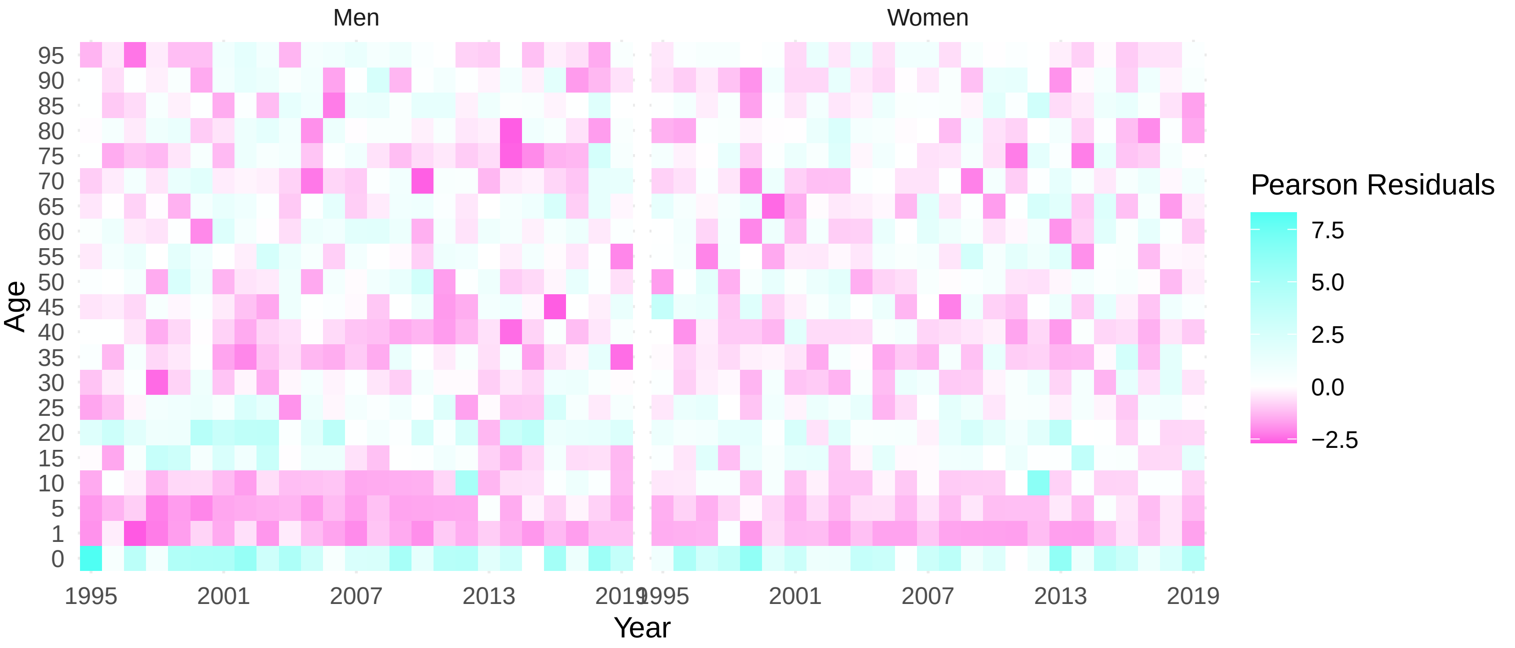

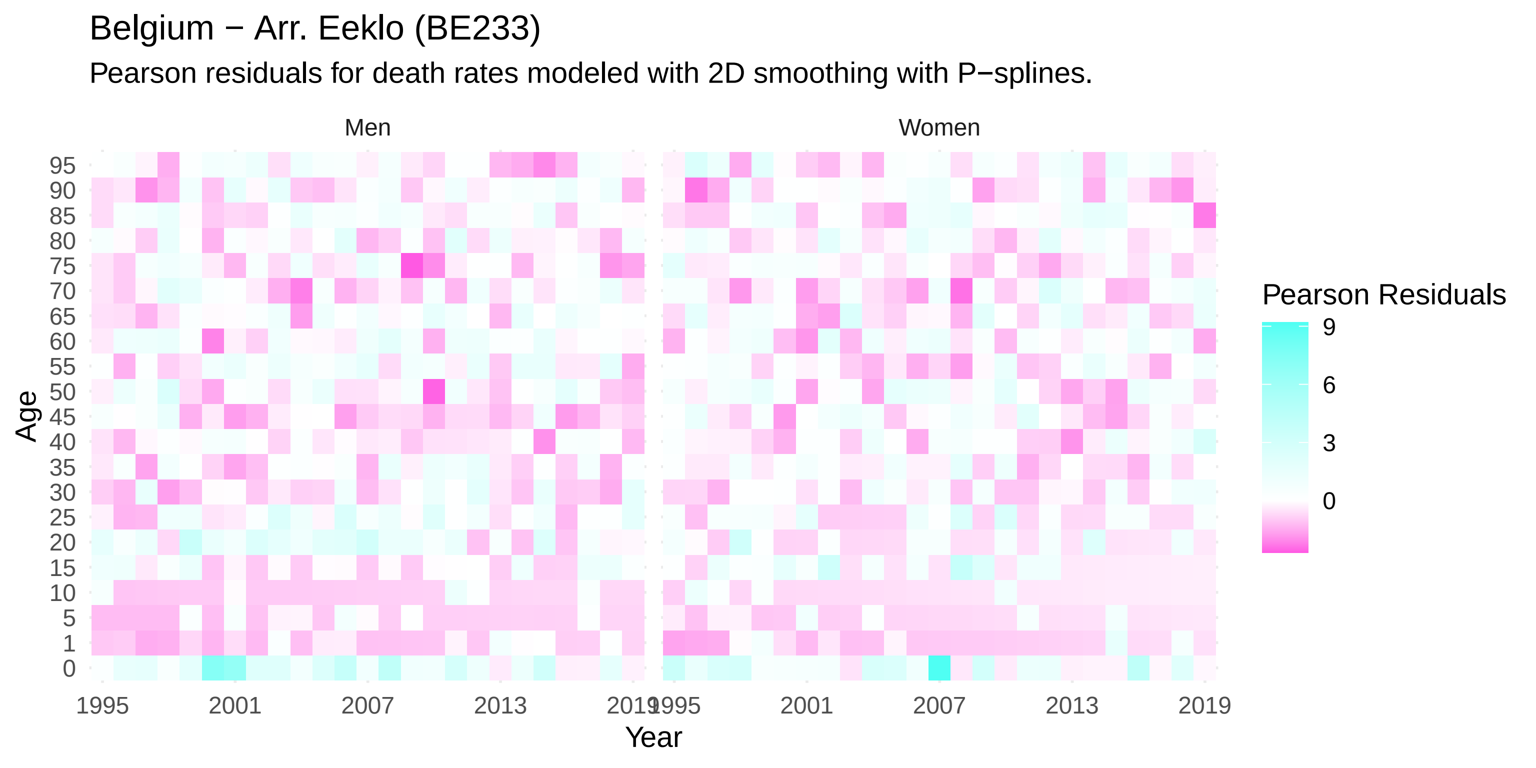

Belgium − Arr. Eeklo (BE233)
Pearson residuals for death rates modeled with 2D smoothing with P−splines.
Men
Women
Age
95
90
85
80
75
70
65
60
55
50
45
40
35
30
25
20
15
10
5
1
0
1995
2001
2007
2013
2019
Year
Pearson Residuals
9
6
3
0

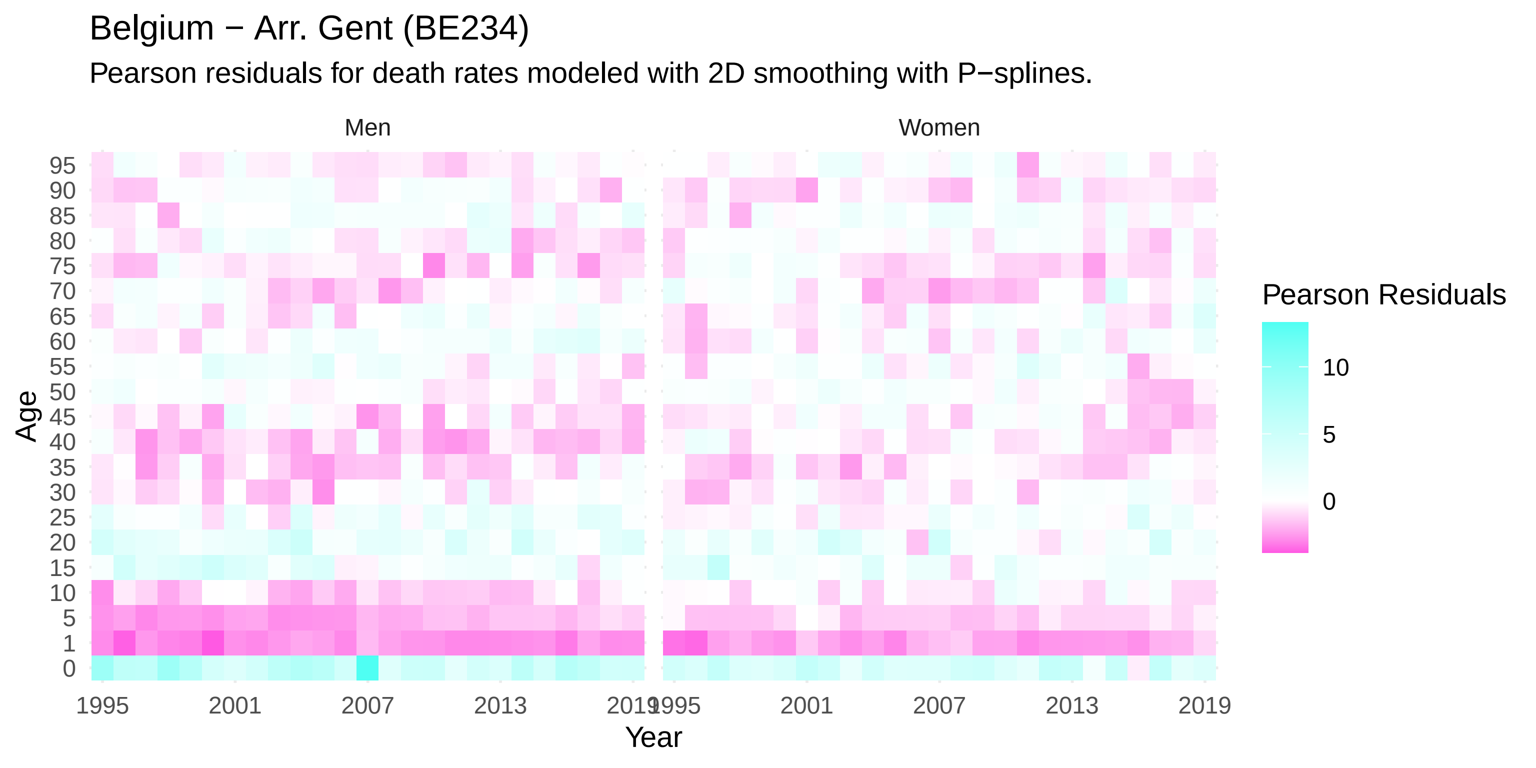
Belgium − Arr. Gent (BE234)
Pearson residuals for death rates modeled with 2D smoothing with P−splines.
Men
Women
Age
95
90
85
80
75
70
65
60
55
50
45
40
35
30
25
20
15
10
5
1
0
1995
2001
2007
2013
2019
Year
Pearson Residuals
10
5
0

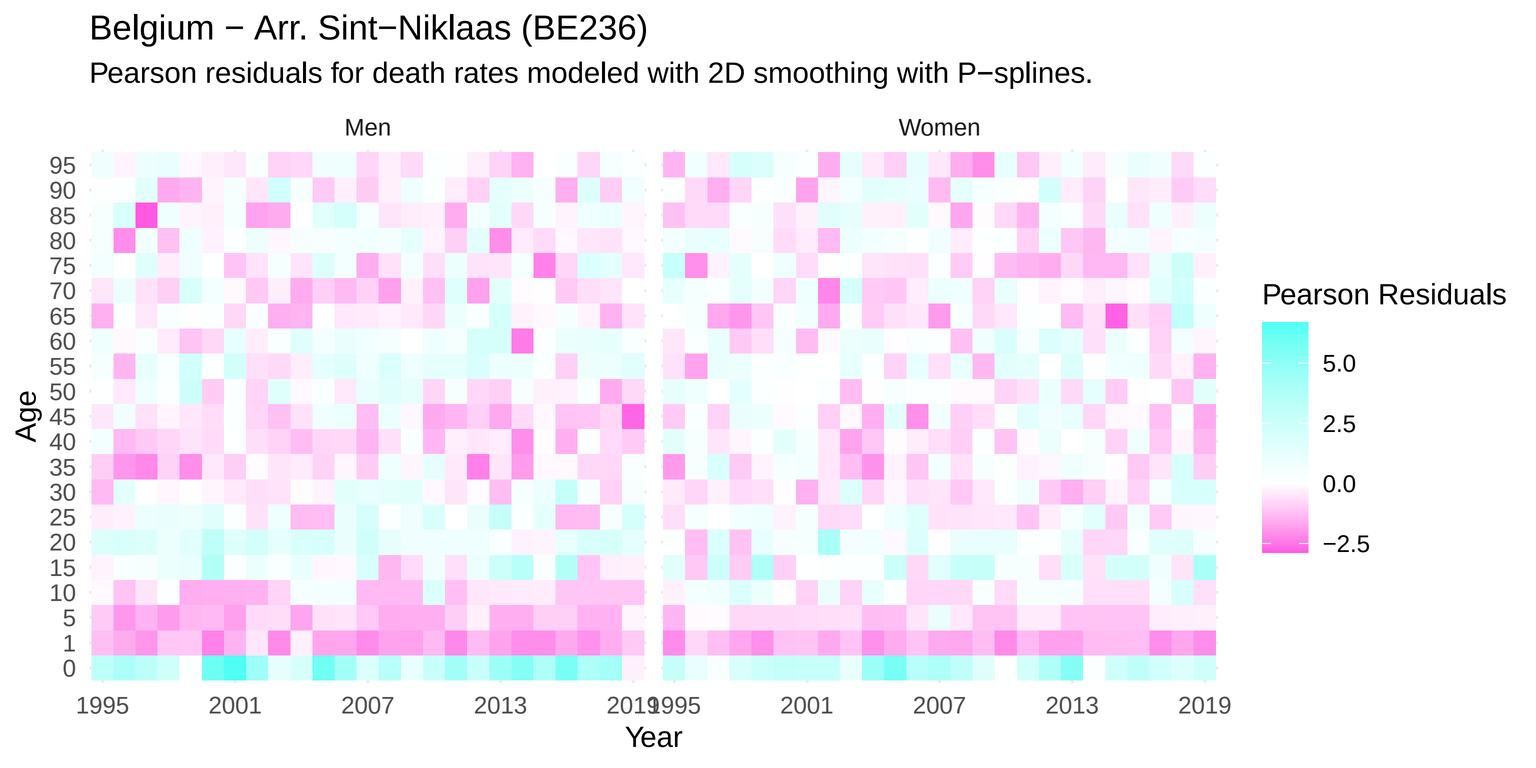
Belgium − Arr. Sint−Niklaas (BE236)
Pearson residuals for death rates modeled with 2D smoothing with P−splines.
Men
Women
Age
95
90
85
80
75
70
65
60
55
50
45
40
35
30
25
20
15
10
5
1
0
1995
2001
2007
2013
2019
Year
Pearson Residuals
5.0
2.5
0.0
−2.5

Belgium − Arr. Brugge (BE251)

Pearson residuals for death rates modeled with 2D smoothing with P−splines.

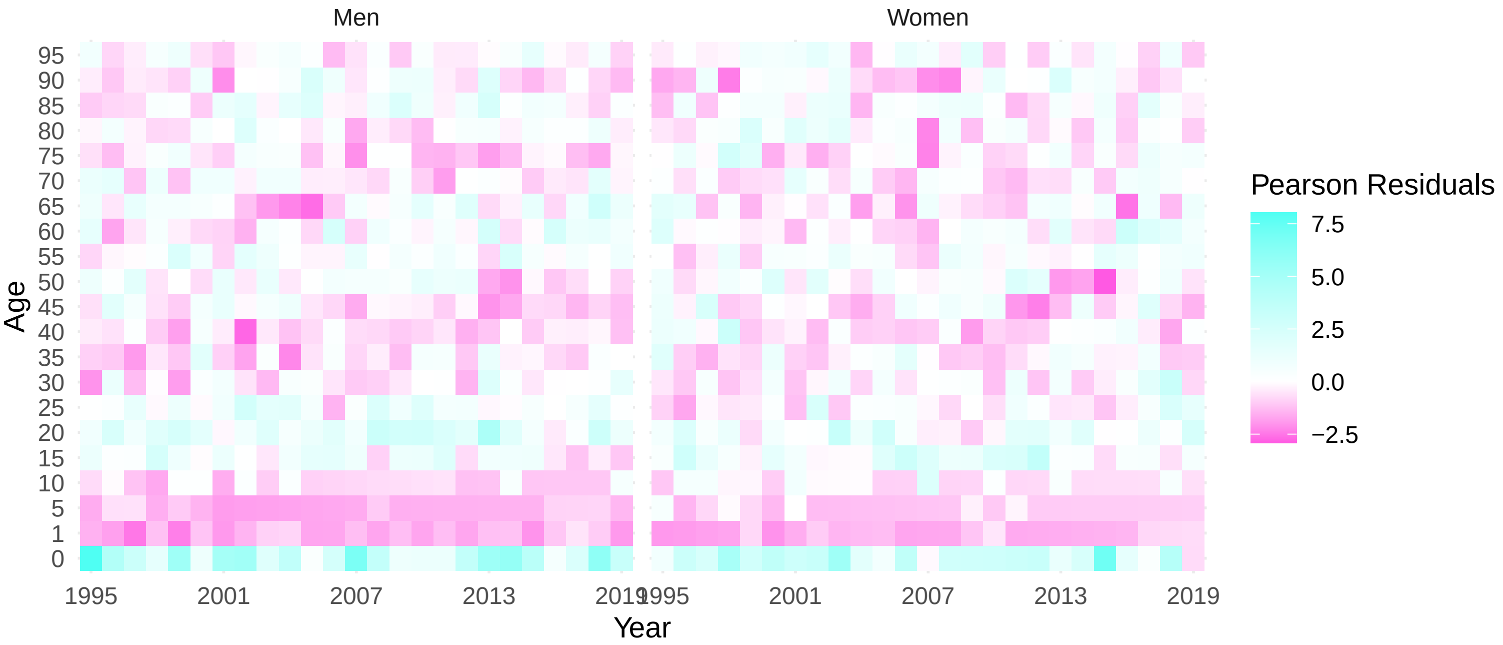

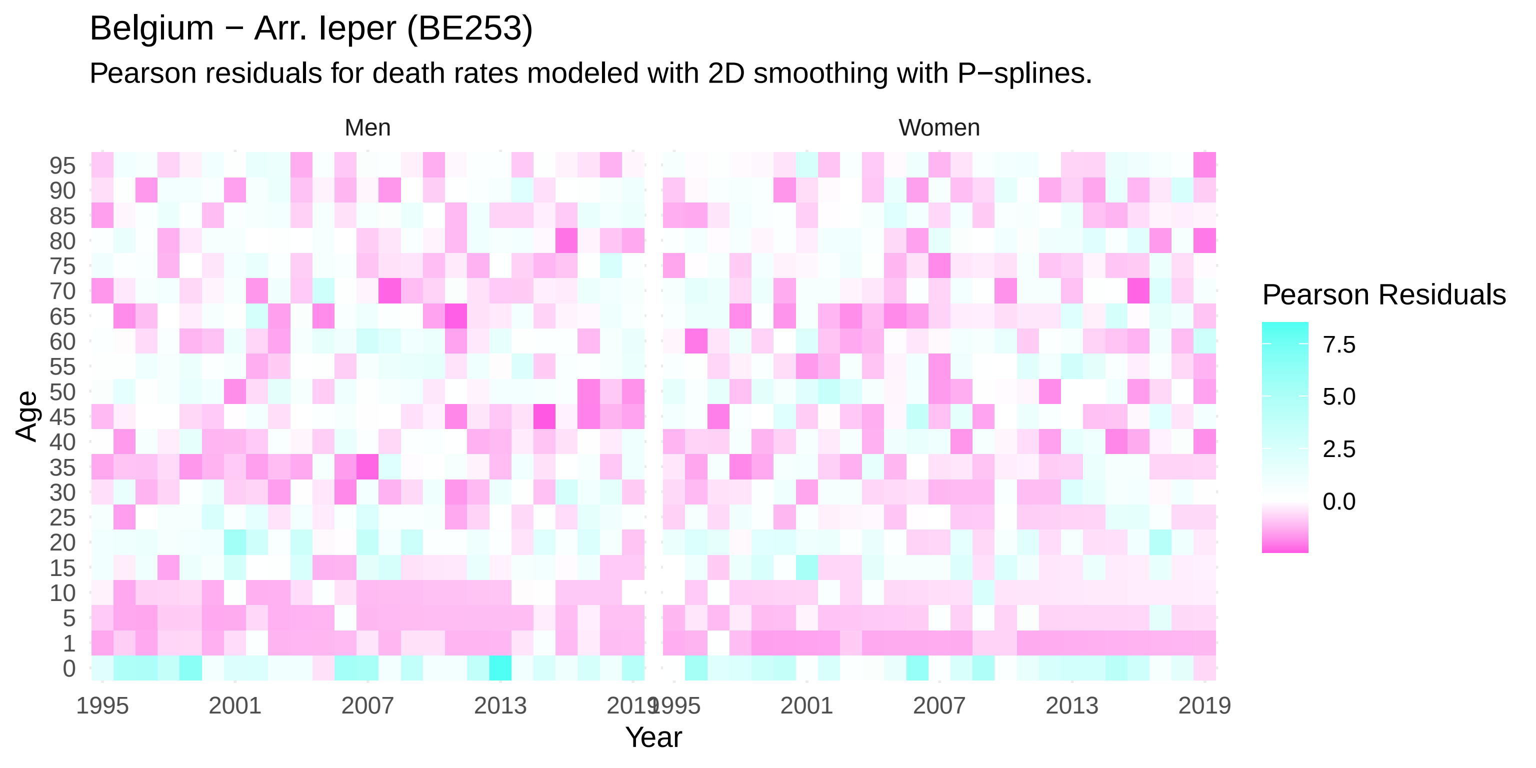

Belgium − Arr. Ieper (BE253)
Pearson residuals for death rates modeled with 2D smoothing with P−splines.
Men
Women
Age
95
90
85
80
75
70
65
60
55
50
45
40
35
30
25
20
15
10
5
1
0
1995
2001
2007
2013
2019
Year
Pearson Residuals
7.5
5.0
2.5
0.0

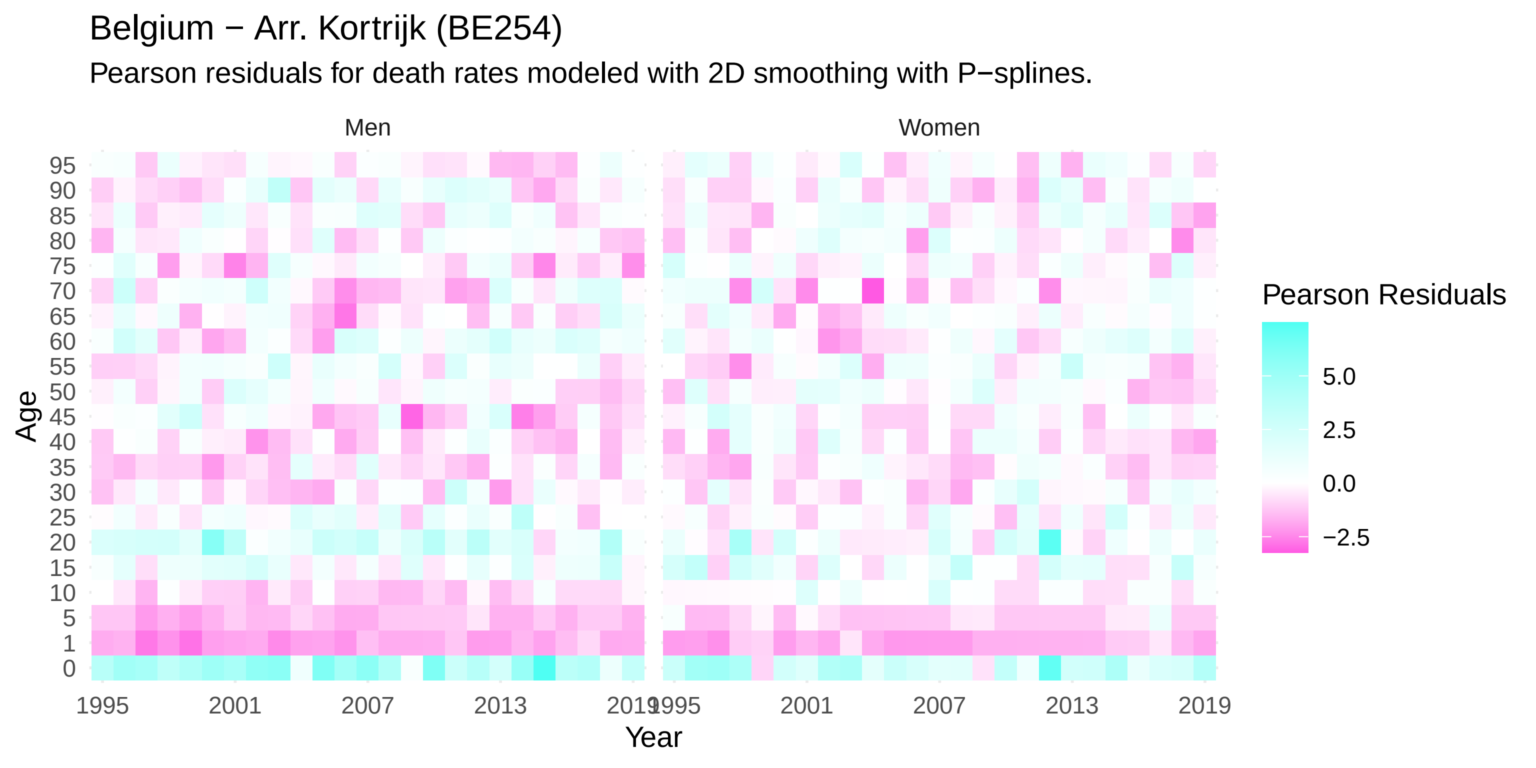
Belgium − Arr. Kortrijk (BE254)
Pearson residuals for death rates modeled with 2D smoothing with P−splines.
Men
Women
Age
95
90
85
80
75
70
65
60
55
50
45
40
35
30
25
20
15
10
5
1
0
1995
2001
2007
2013
2019
Year
Pearson Residuals
5.0
2.5
0.0
−2.5

Belgium − Arr. Veurne (BE258)

Pearson residuals for death rates modeled with 2D smoothing with P−splines.

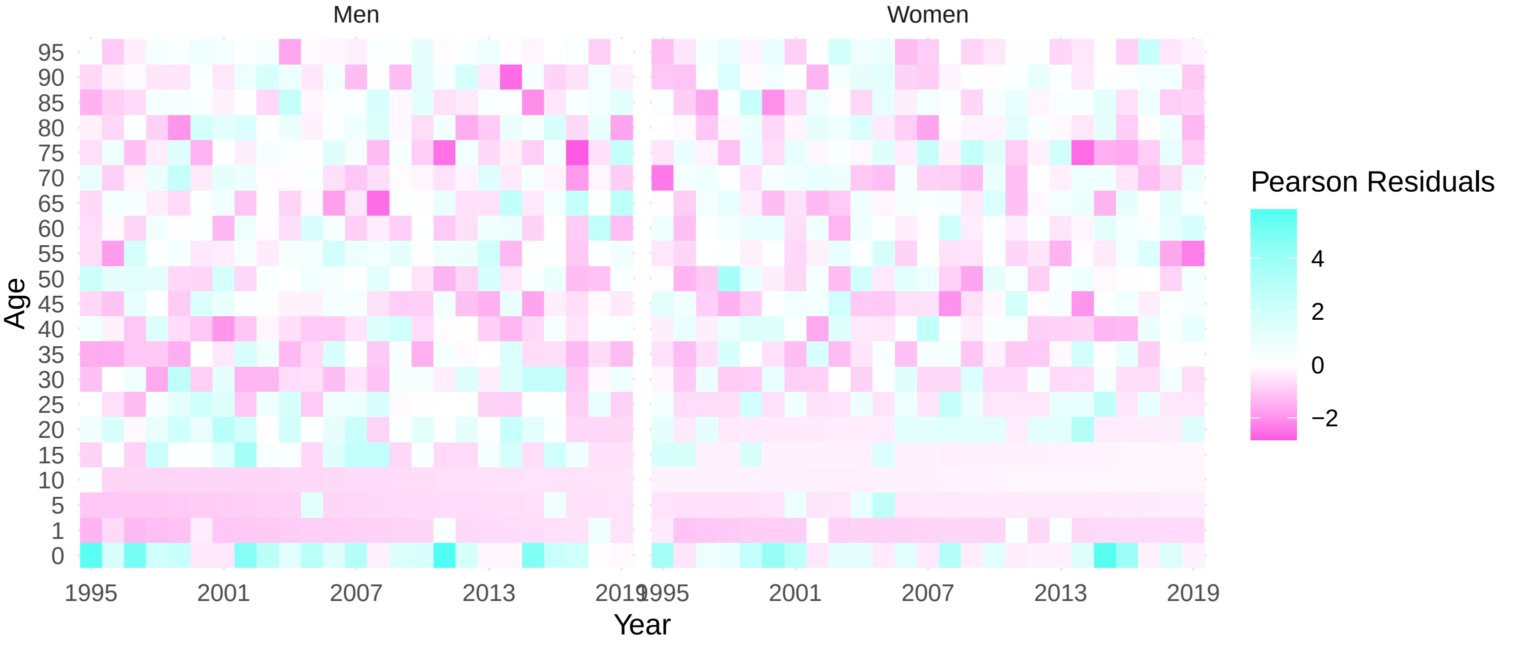

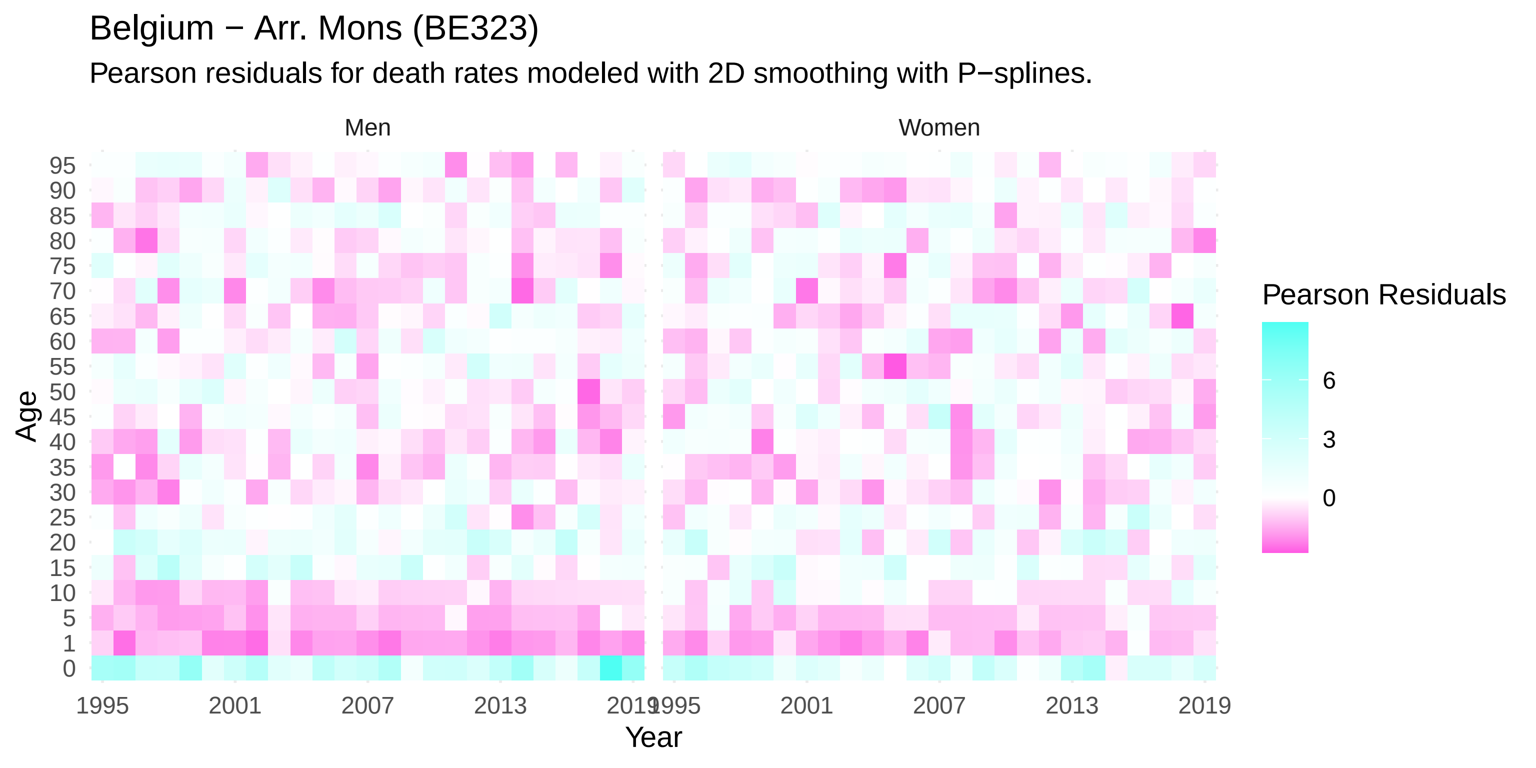
Belgium − Arr. Mons (BE323)
Pearson residuals for death rates modeled with 2D smoothing with P−splines.
Men
Women
Age
95
90
85
80
75
70
65
60
55
50
45
40
35
30
25
20
15
10
5
1
0
1995
2001
2007
2013
2019
Year
Pearson Residuals
6
3
0

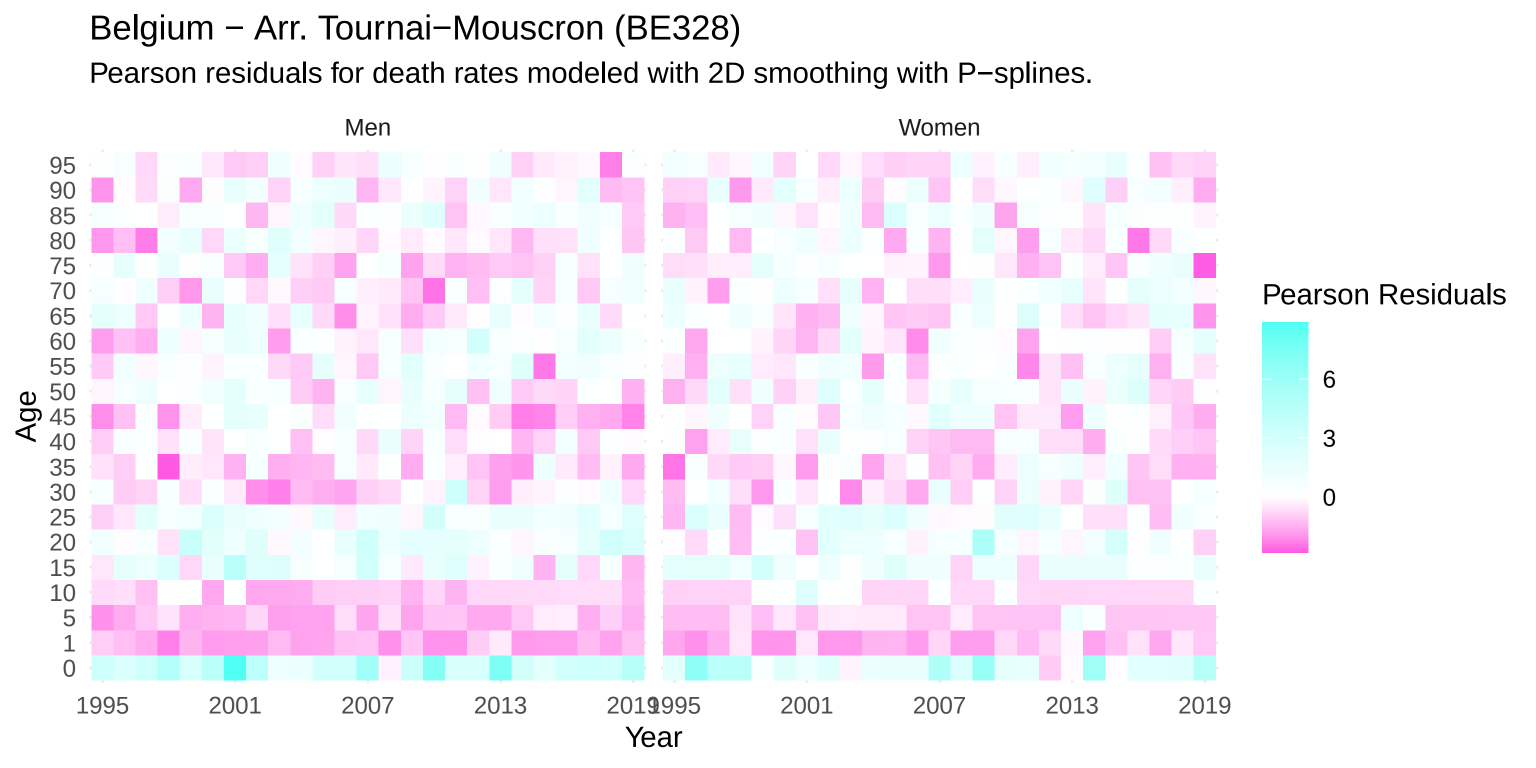

Belgium − Arr. Tournai−Mouscron (BE328)
Pearson residuals for death rates modeled with 2D smoothing with P−splines.
Men
Women
Age
95
90
85
80
75
70
65
60
55
50
45
40
35
30
25
20
15
10
5
1
0
1995
2001
2007
2013
2019
Year
Pearson Residuals
6
3
0

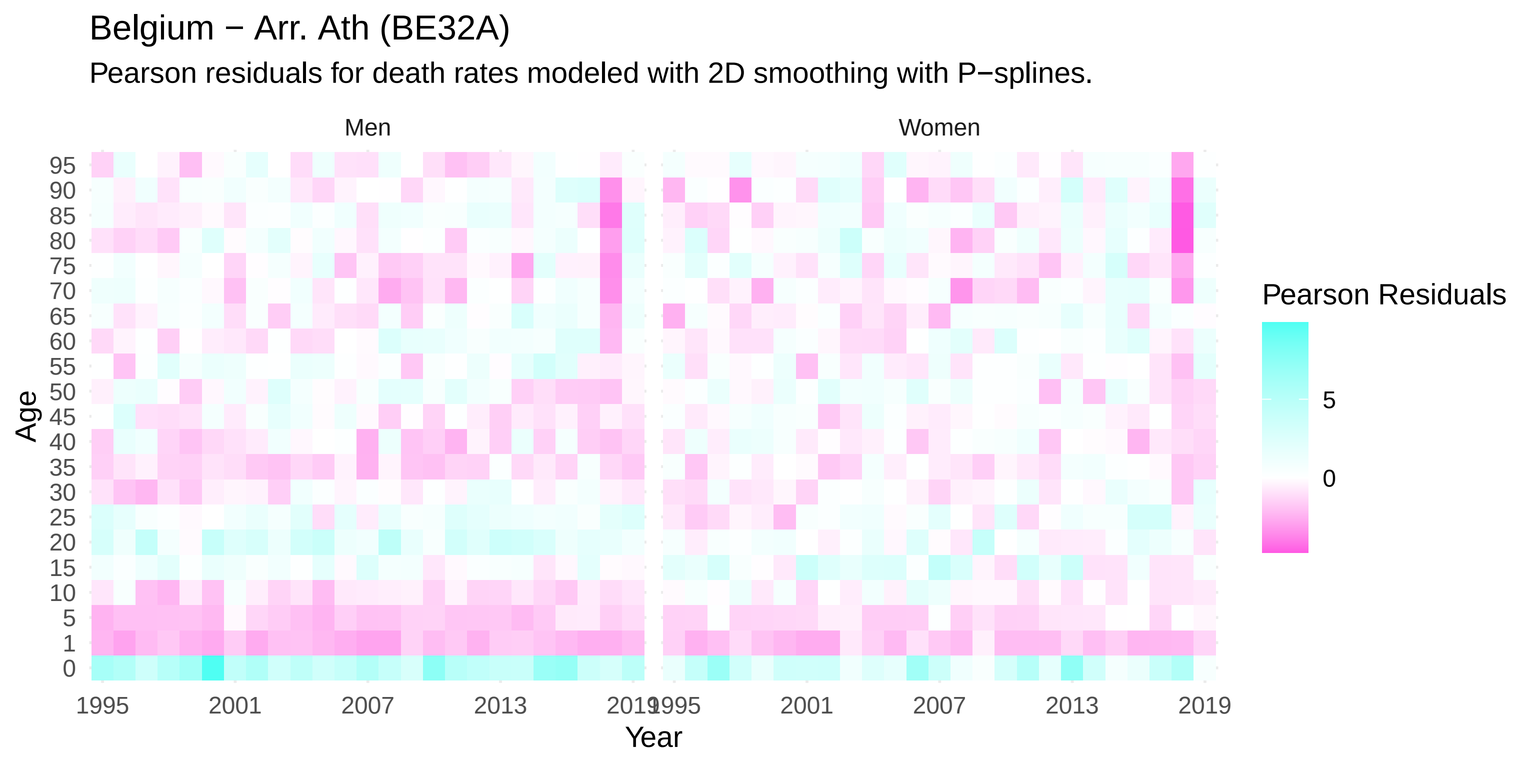

Belgium − Arr. Ath (BE32A)
Pearson residuals for death rates modeled with 2D smoothing with P−splines.
Men
Women
Age
95
90
85
80
75
70
65
60
55
50
45
40
35
30
25
20
15
10
5
1
0
1995
2001
2007
2013
2019
Year
Pearson Residuals
5
0

Belgium − Arr. Liège (BE332)

Pearson residuals for death rates modeled with 2D smoothing with P−splines.

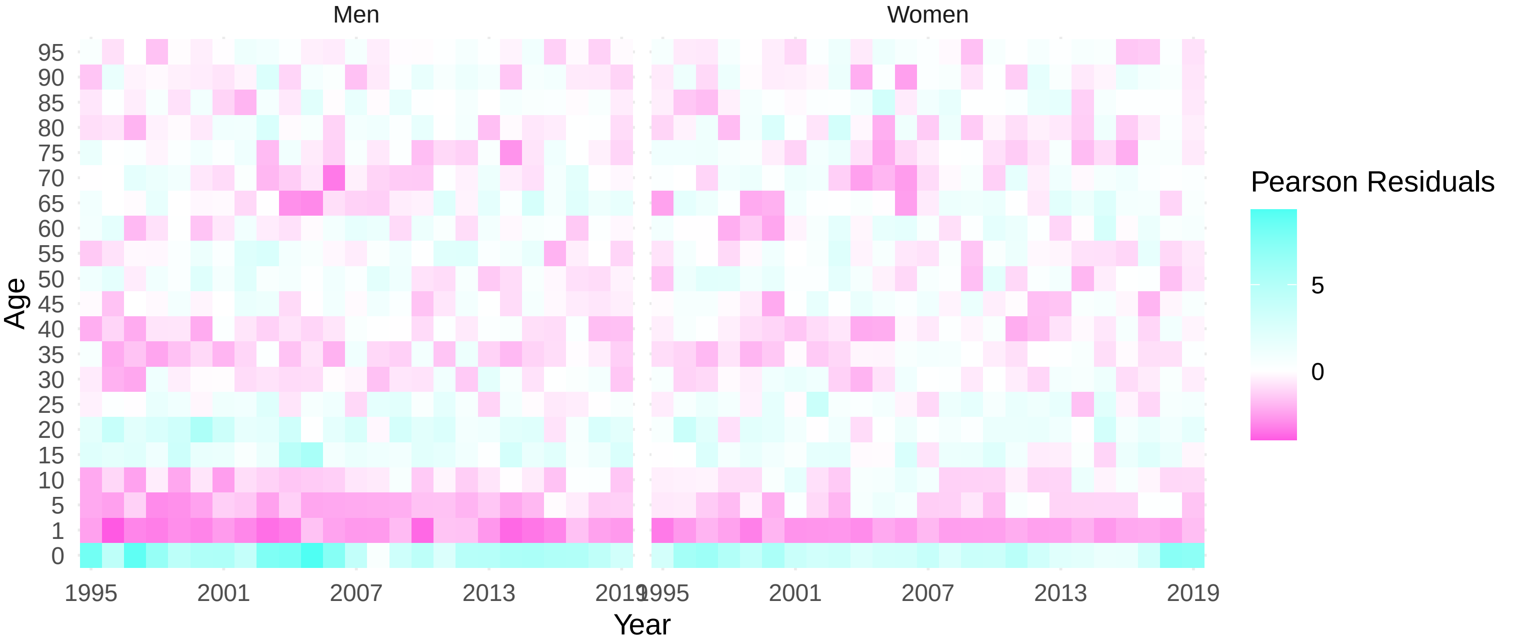

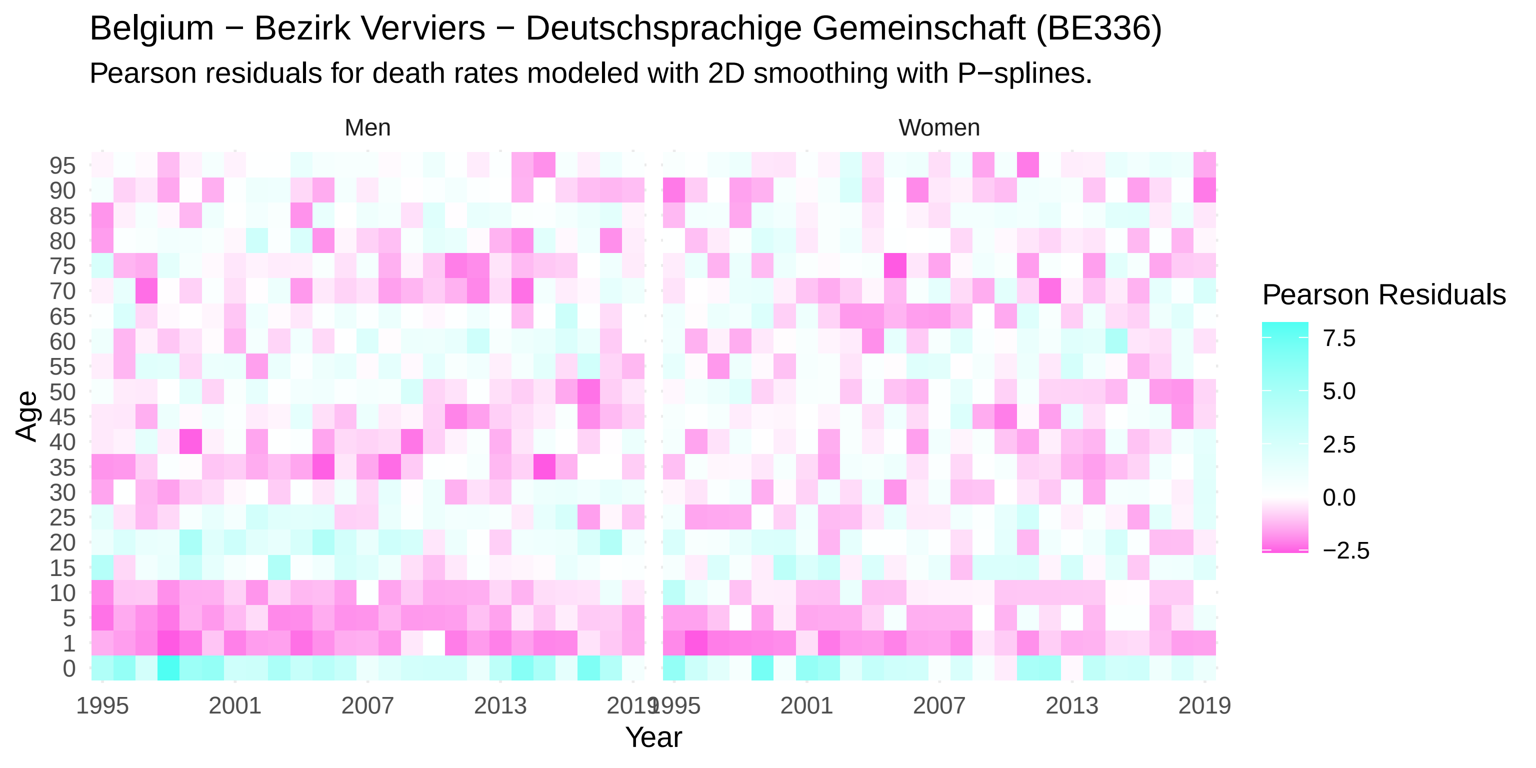

Belgium − Bezirk Verviers − Deutschsprachige Gemeinschaft (BE336)
Pearson residuals for death rates modeled with 2D smoothing with P−splines.
Men
Women
Age
95
90
85
80
75
70
65
60
55
50
45
40
35
30
25
20
15
10
5
1
0
1995
2001
2007
2013
2019
Year
Pearson Residuals
7.5
5.0
2.5
0.0
−2.5

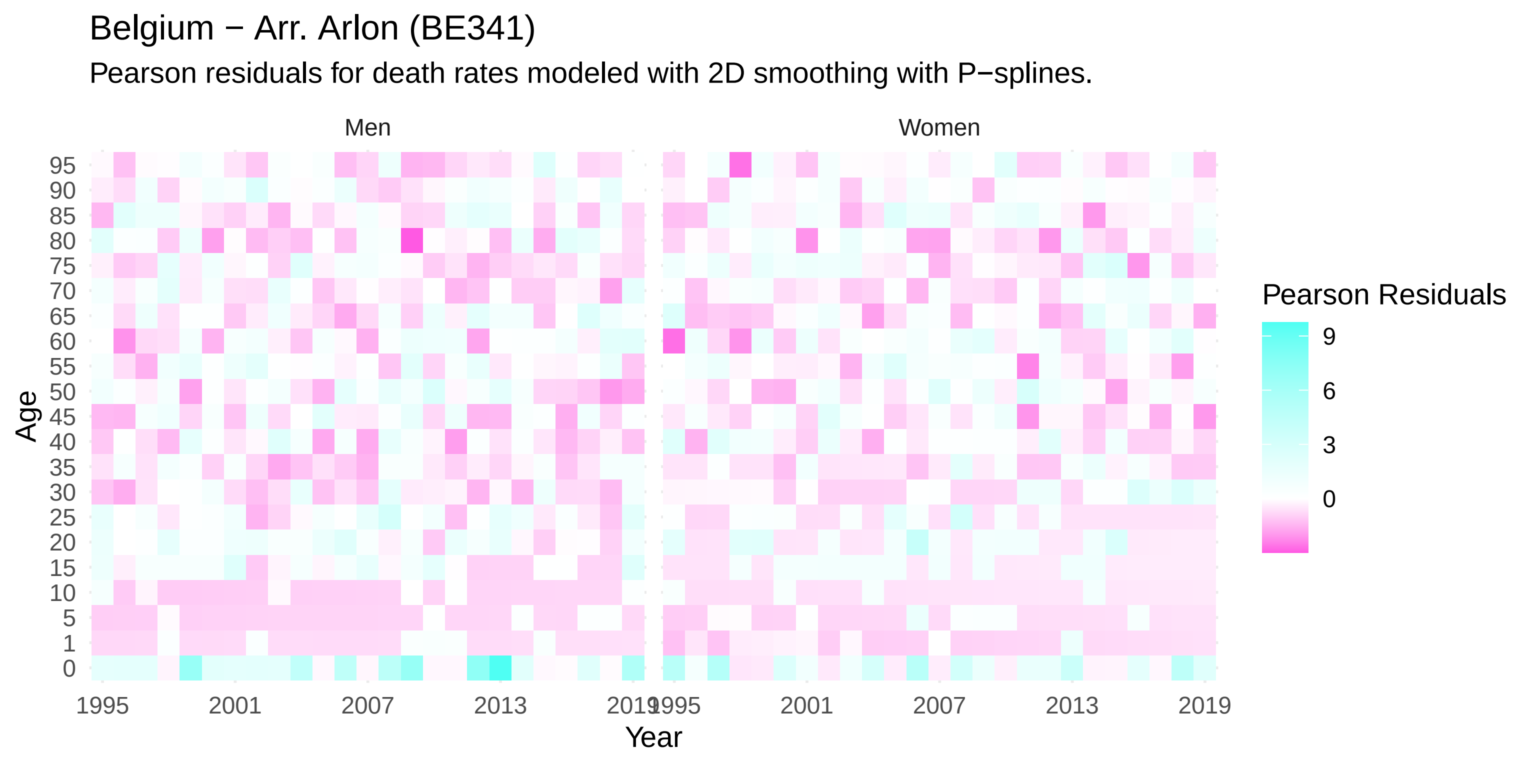

Belgium − Arr. Arlon (BE341)
Pearson residuals for death rates modeled with 2D smoothing with P−splines.
Men
Women
Age
95
90
85
80
75
70
65
60
55
50
45
40
35
30
25
20
15
10
5
1
0
1995
2001
2007
2013
2019
Year
Pearson Residuals
9
6
3
0

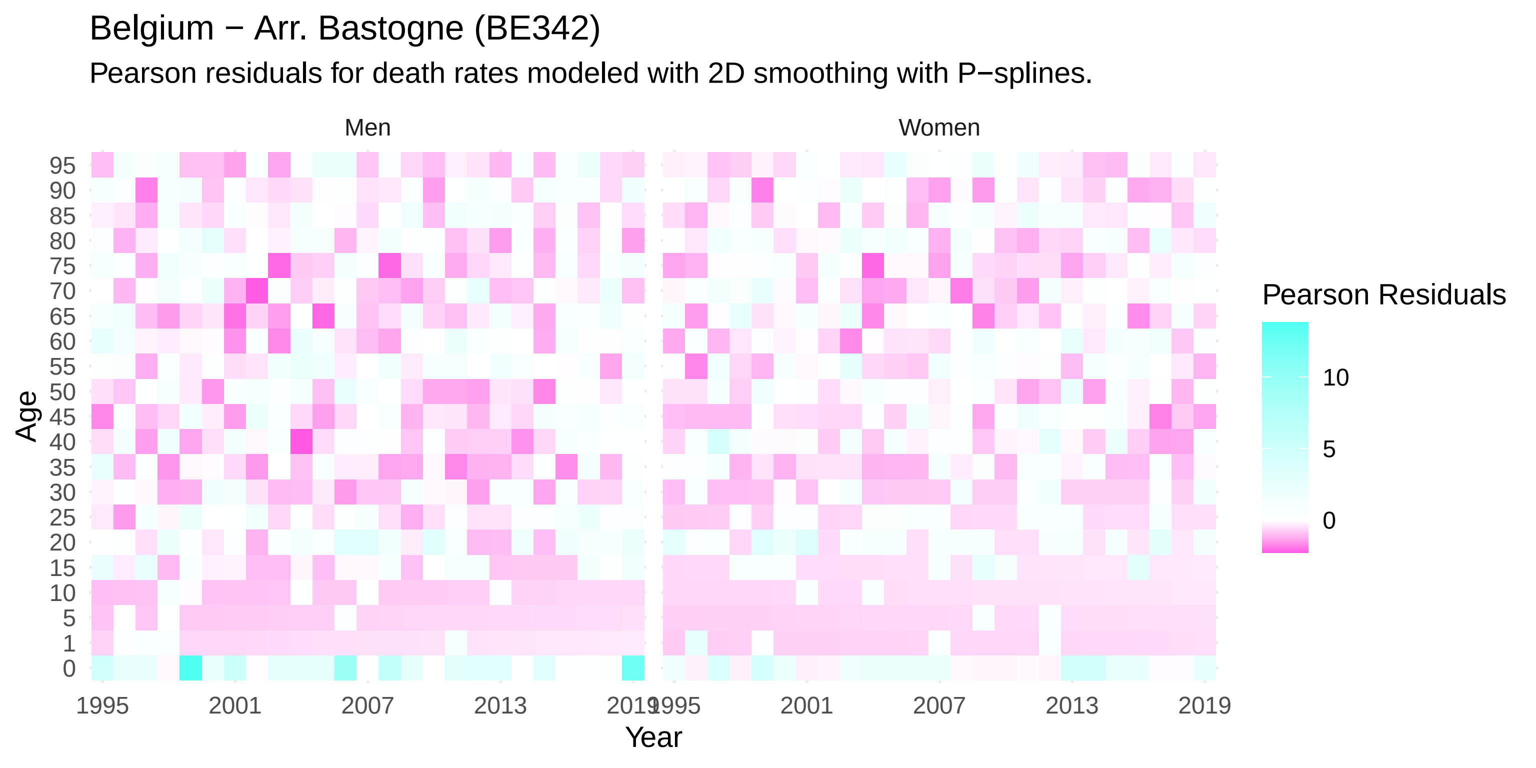
Belgium − Arr. Bastogne (BE342)
Pearson residuals for death rates modeled with 2D smoothing with P−splines.
Men
Women
Age
95
90
85
80
75
70
65
60
55
50
45
40
35
30
25
20
15
10
5
1
0
1995
2001
2007
2013
2019
Year
Pearson Residuals
10
5
0

Belgium − Arr. Neufchâteau (BE344)

Pearson residuals for death rates modeled with 2D smoothing with P−splines.

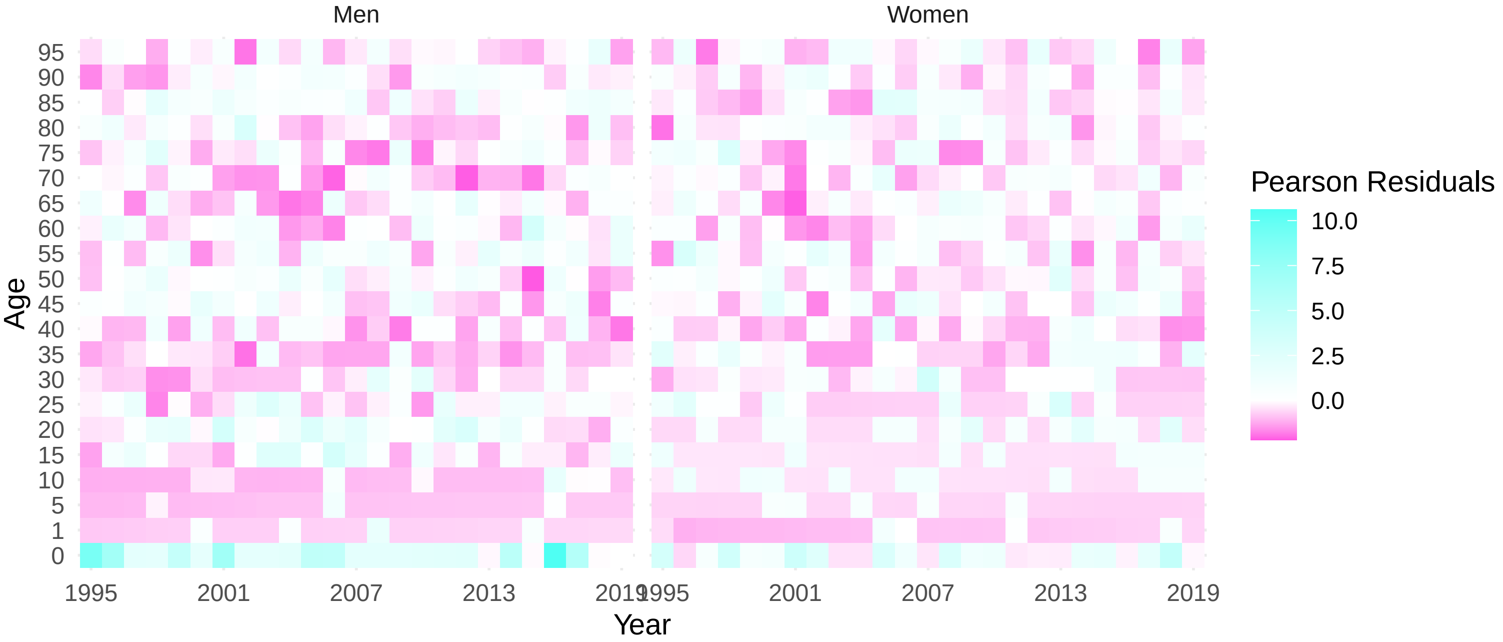

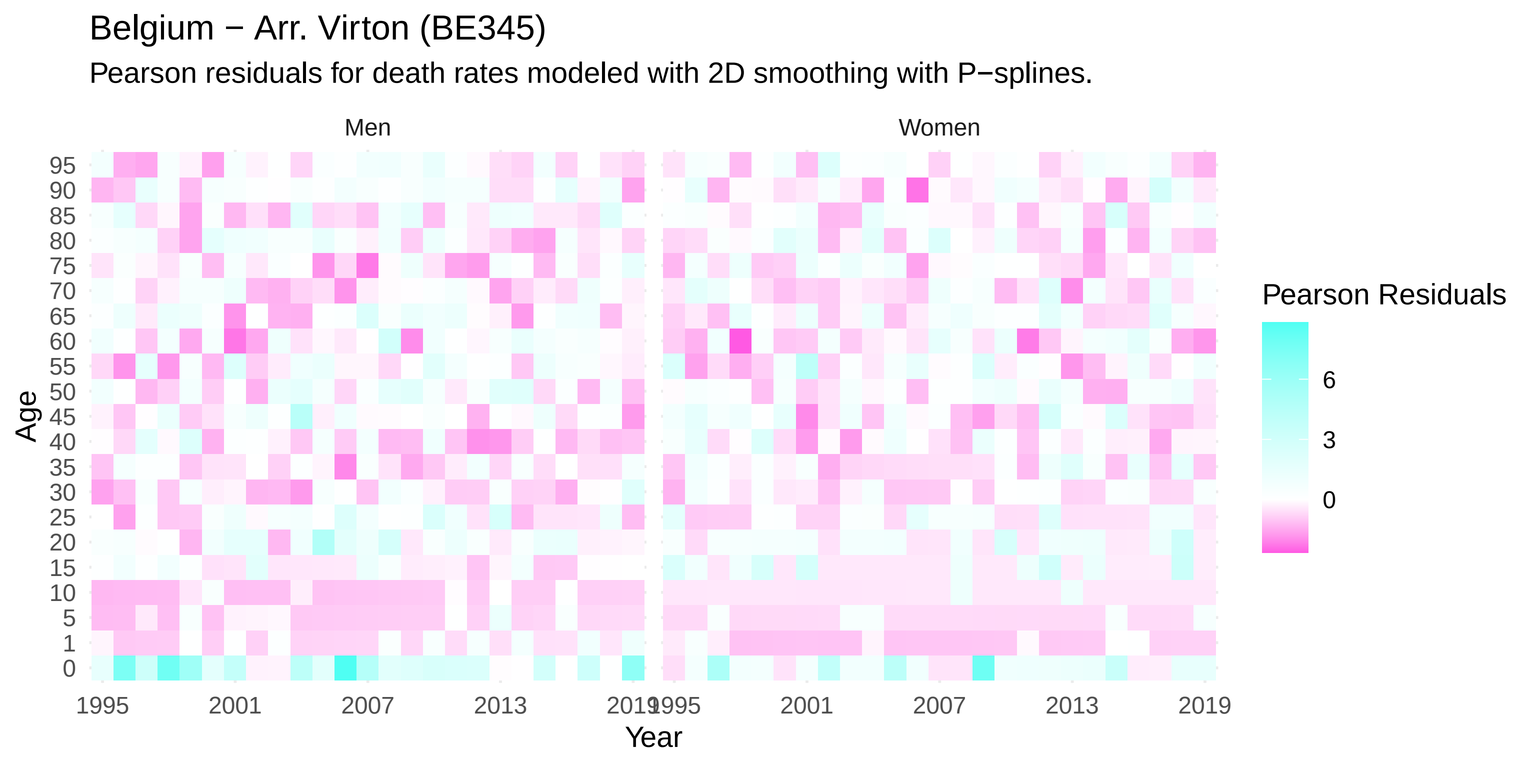
Belgium − Arr. Virton (BE345)
Pearson residuals for death rates modeled with 2D smoothing with P−splines.
Men
Women
Age
95
90
85
80
75
70
65
60
55
50
45
40
35
30
25
20
15
10
5
1
0
1995
2001
2007
2013
2019
Year
Pearson Residuals
6
3
0

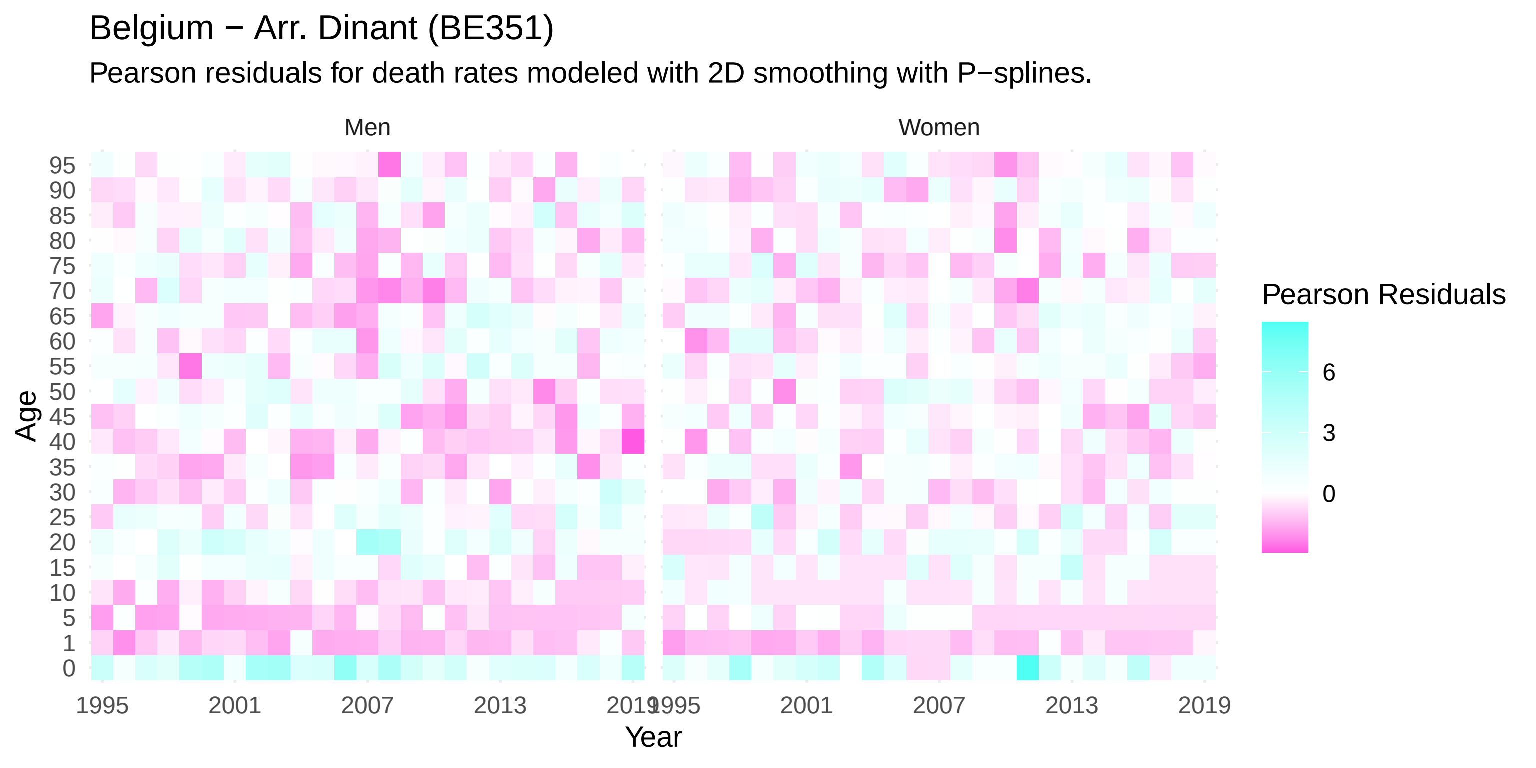

Belgium − Arr. Dinant (BE351)
Pearson residuals for death rates modeled with 2D smoothing with P−splines.
Men
Women
Age
95
90
85
80
75
70
65
60
55
50
45
40
35
30
25
20
15
10
5
1
0
1995
2001
2007
2013
2019
1995
2001
2007
2013
2019
Year
Pearson Residuals
6
3
0

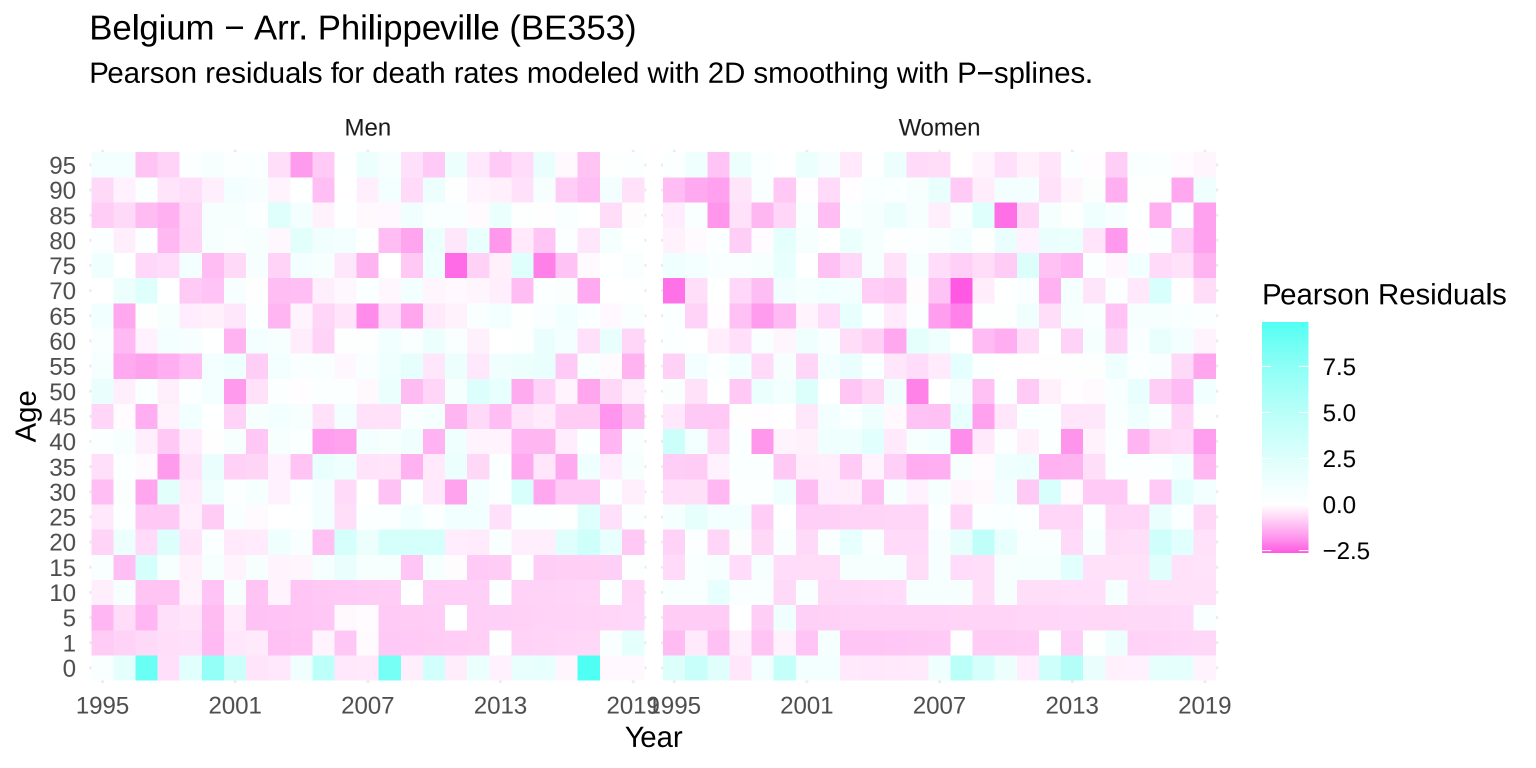
Belgium − Arr. Philippeville (BE353)
Pearson residuals for death rates modeled with 2D smoothing with P−splines.
Men
Women
Age
95
90
85
80
75
70
65
60
55
50
45
40
35
30
25
20
15
10
5
1
0
1995
2001
2007
2013
2019
Year
Pearson Residuals
7.5
5.0
2.5
0.0
−2.5
