# Supplementary material for: Getting closer to each other? Convergence and divergence patterns of life expectancy in 277 border regions of Western Europe 1995–2019
Source: Eur J Epidemiol. 2025 Jul 19;40(9):1031–43. doi: 10.1007/s10654-025-01279-w (PMC12537618; doi:10.1007/s10654-025-01279-w)
Supplement: Supplementary file 6 — Supplementary Material 6 [file 10654_2025_1279_MOESM6_ESM.pdf]

Boxplots

Reference group 1: neighbouring border regions

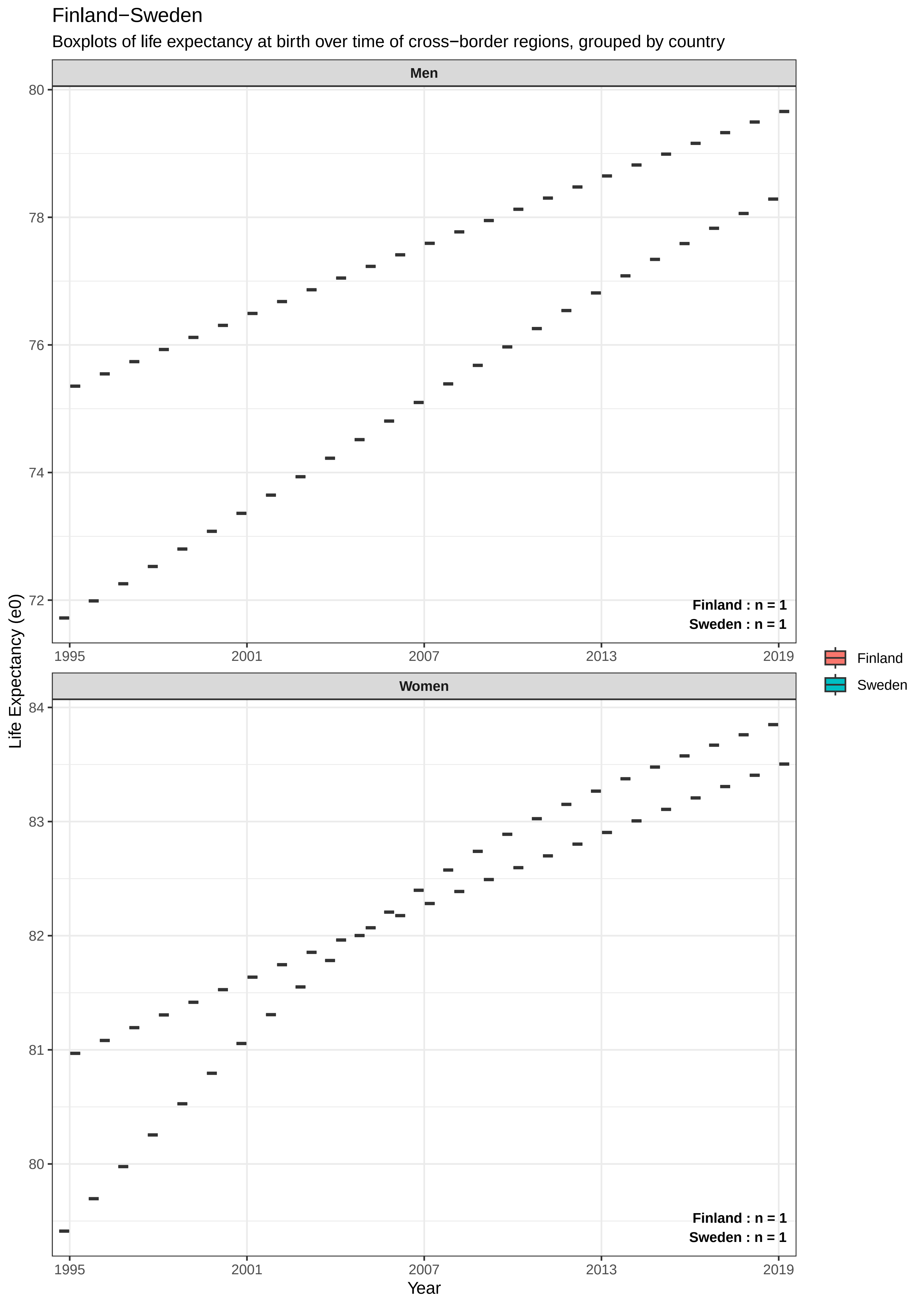

Finland−Sweden
Boxplots of life expectancy at birth over time of cross−border regions, grouped by country
Men
Women
Finland : n = 1
Sweden : n = 1
Finland : n = 1
Sweden : n = 1
Life Expectancy (e0)
Year
1995
2001
2007
2013
2019
72
74
76
78
80
81
82
83
84
Finland
Sweden

Denmark−Sweden

Boxplots of life expectancy at birth over time of cross−border regions, grouped by country

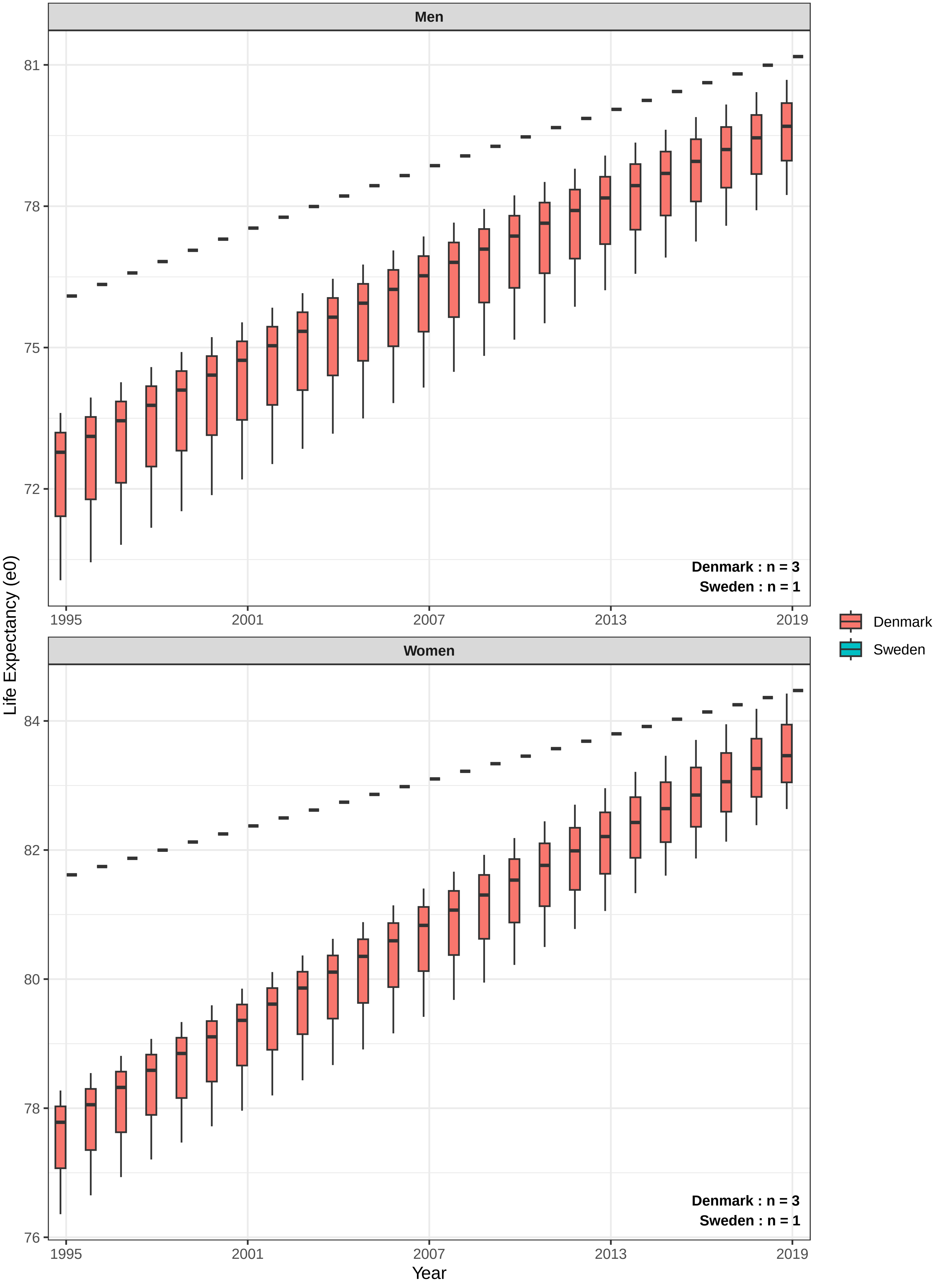

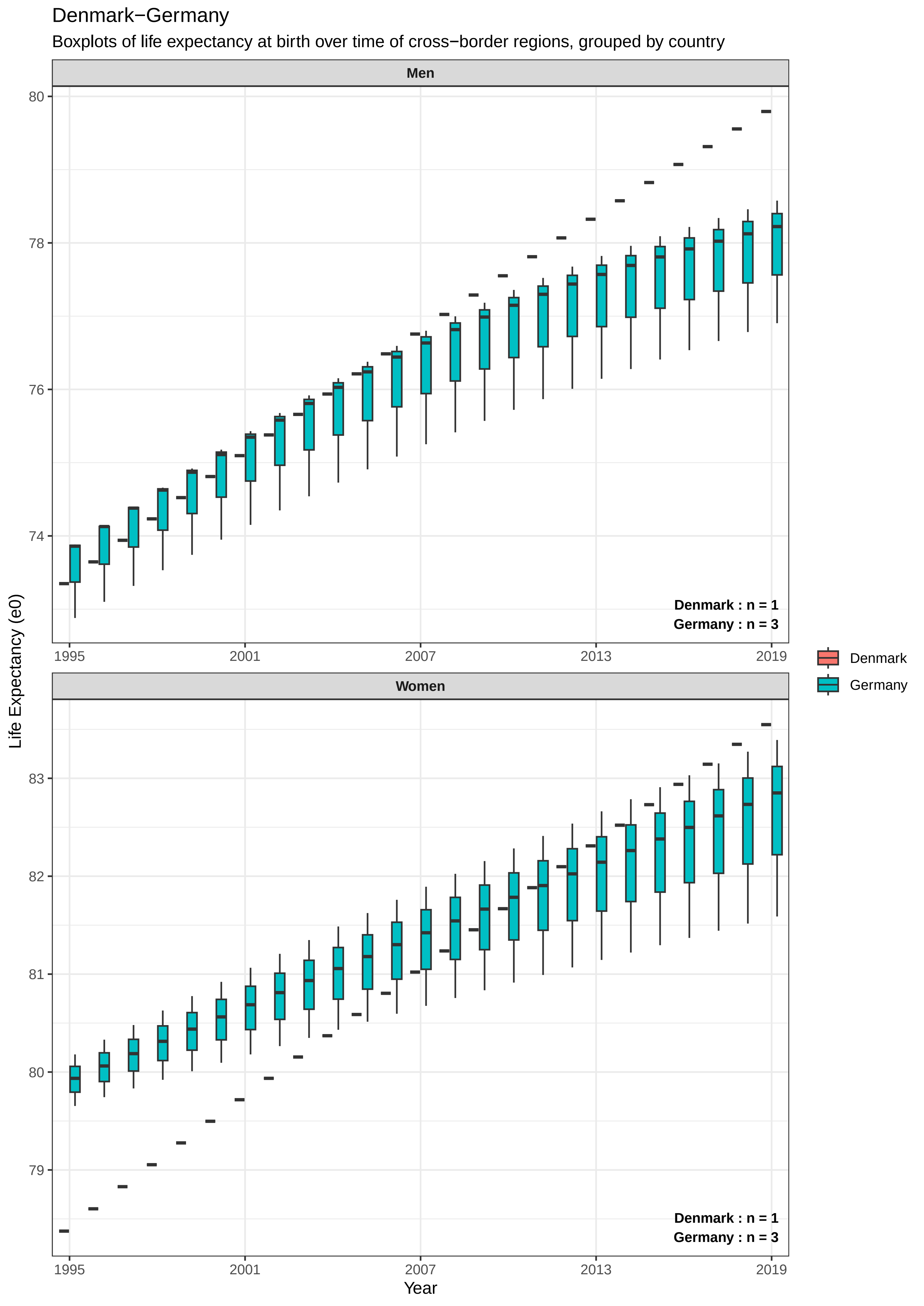

Denmark−Germany
Boxplots of life expectancy at birth over time of cross−border regions, grouped by country
Men
Women
Denmark : n = 1
Germany : n = 3
Life Expectancy (e0)
Year
80
78
76
74
83
82
81
80
79
1995
2001
2007
2013
2019
Denmark
Germany

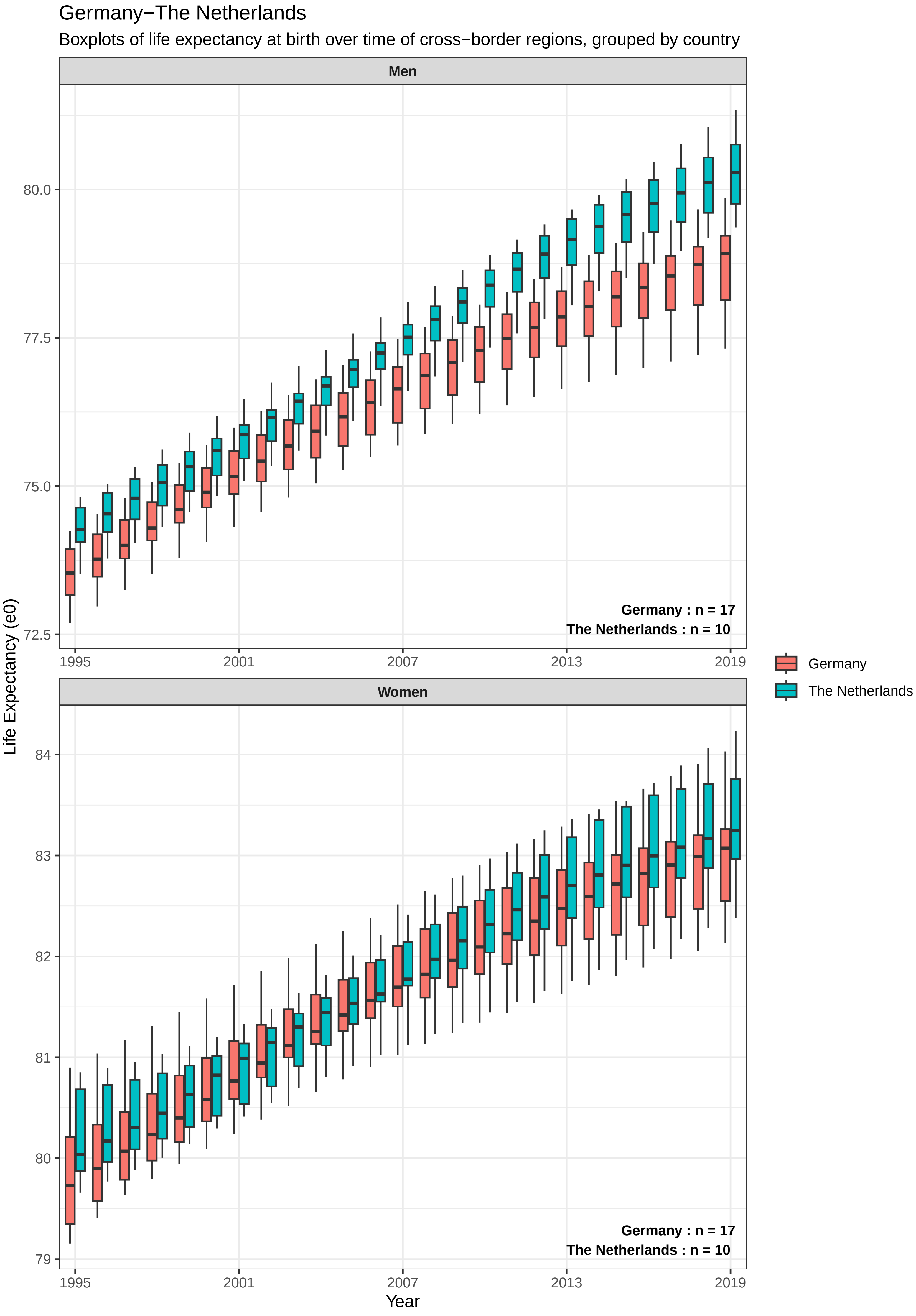

Germany−The Netherlands
Boxplots of life expectancy at birth over time of cross−border regions, grouped by country
Men
Women
Germany : n = 17
The Netherlands : n = 10
Life Expectancy (e0)
Year
1995
2001
2007
2013
2019
72.5
75.0
77.5
80.0
79
80
81
82
83
84
Germany
The Netherlands

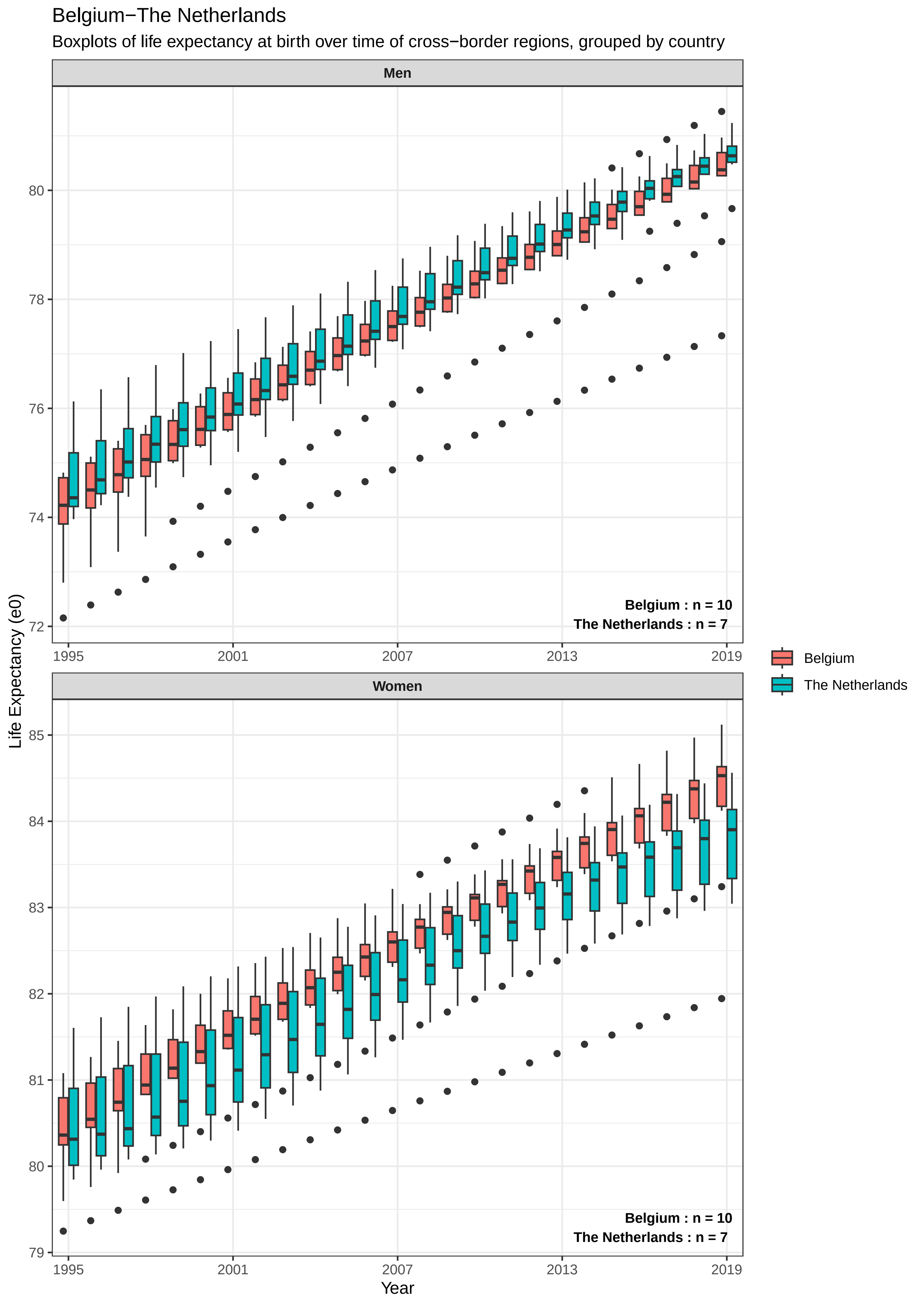

Belgium−The Netherlands
Boxplots of life expectancy at birth over time of cross−border regions, grouped by country
Men
Women
Life Expectancy (e0)
Year
1995
2001
2007
2013
2019
72
74
76
78
80
79
80
81
82
83
84
85
Belgium : n = 10
The Netherlands : n = 7
Belgium
The Netherlands

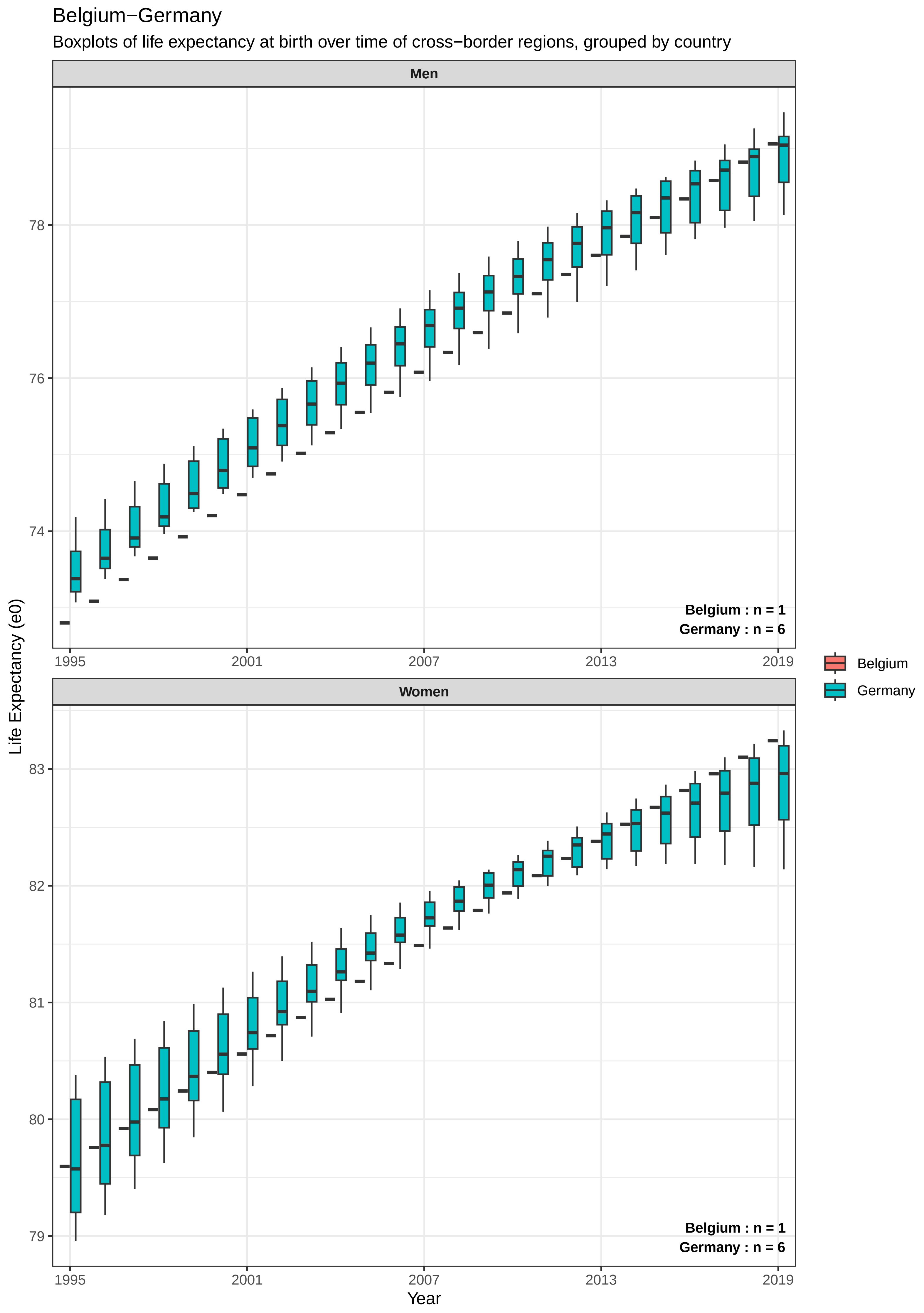

Belgium−Germany
Boxplots of life expectancy at birth over time of cross−border regions, grouped by country
Men
Women
Belgium : n = 1
Germany : n = 6
Life Expectancy (e0)
Year
1995
2001
2007
2013
2019
74
76
78
79
80
81
82
83
Belgium
Germany

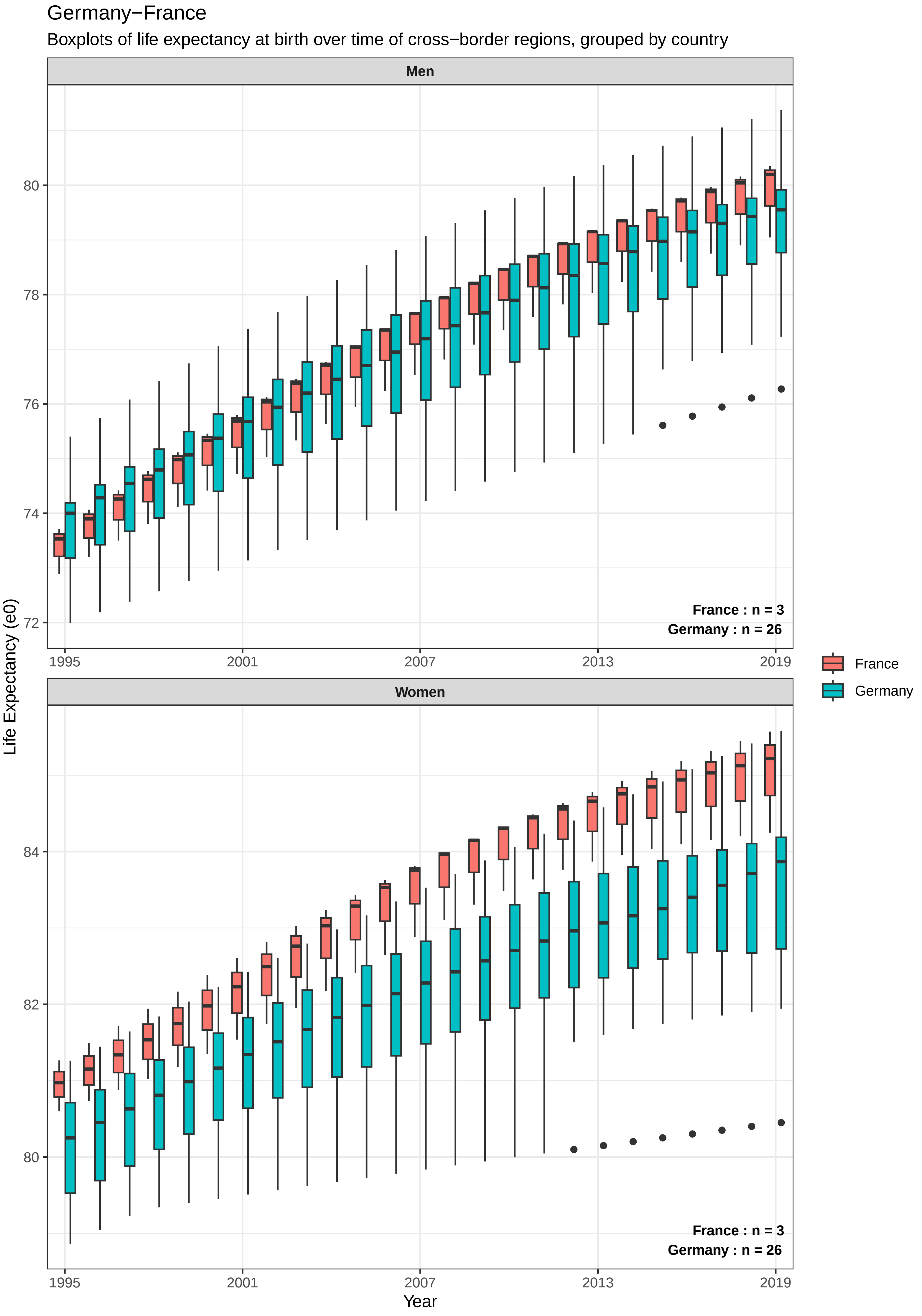
Germany−France
Boxplots of life expectancy at birth over time of cross−border regions, grouped by country
Men
Women
Life Expectancy (e0)
Year
80
78
76
74
72
84
82
80
1995
2001
2007
2013
2019
France : n = 3
Germany : n = 26
France
Germany

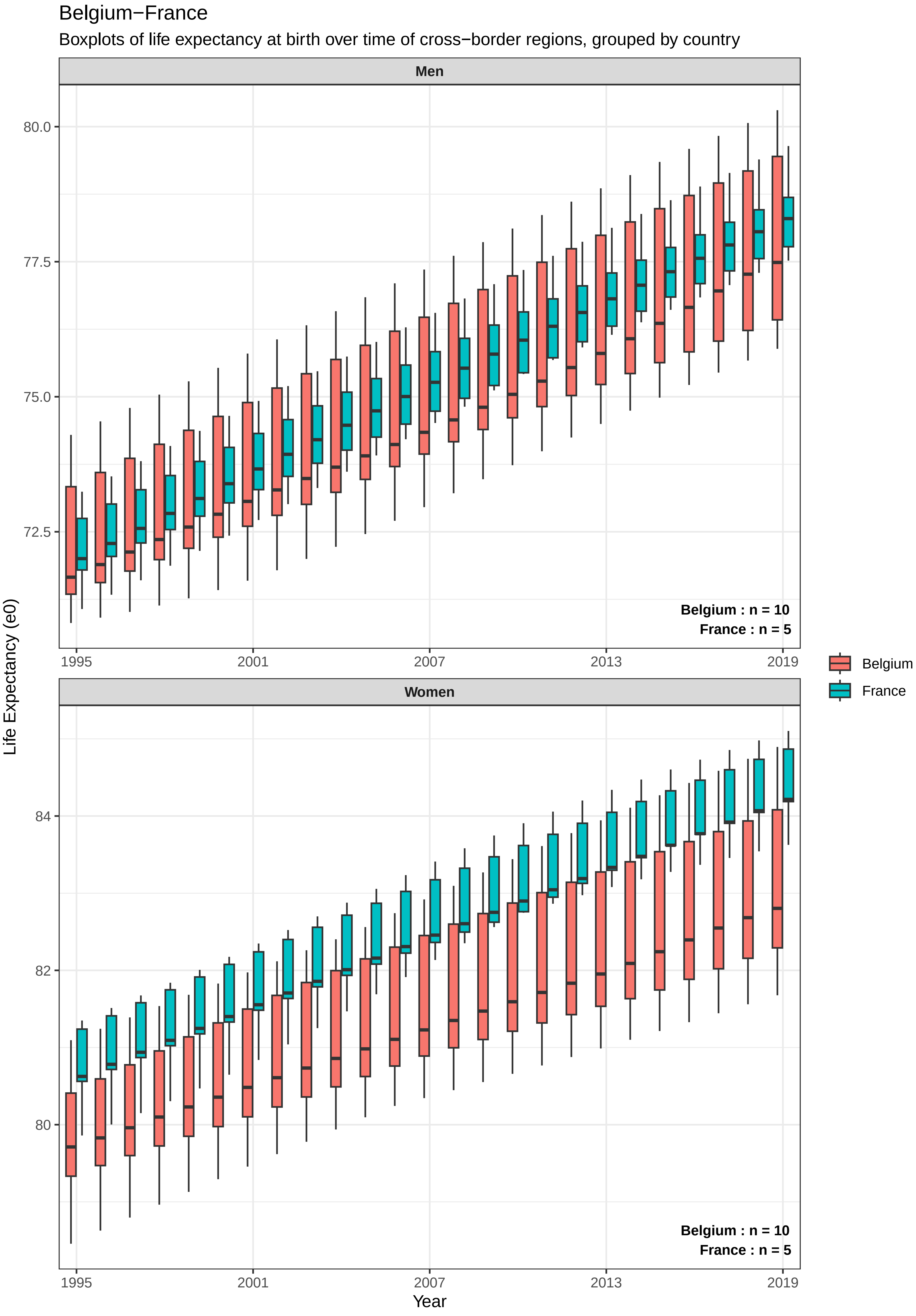

Belgium−France
Boxplots of life expectancy at birth over time of cross−border regions, grouped by country
Men
80.0
77.5
75.0
72.5
Belgium : n = 10
France : n = 5
1995
2001
2007
2013
2019
Women
84
82
80
Belgium : n = 10
France : n = 5
1995
2001
2007
2013
2019
Life Expectancy (e0)
Year
Belgium
France

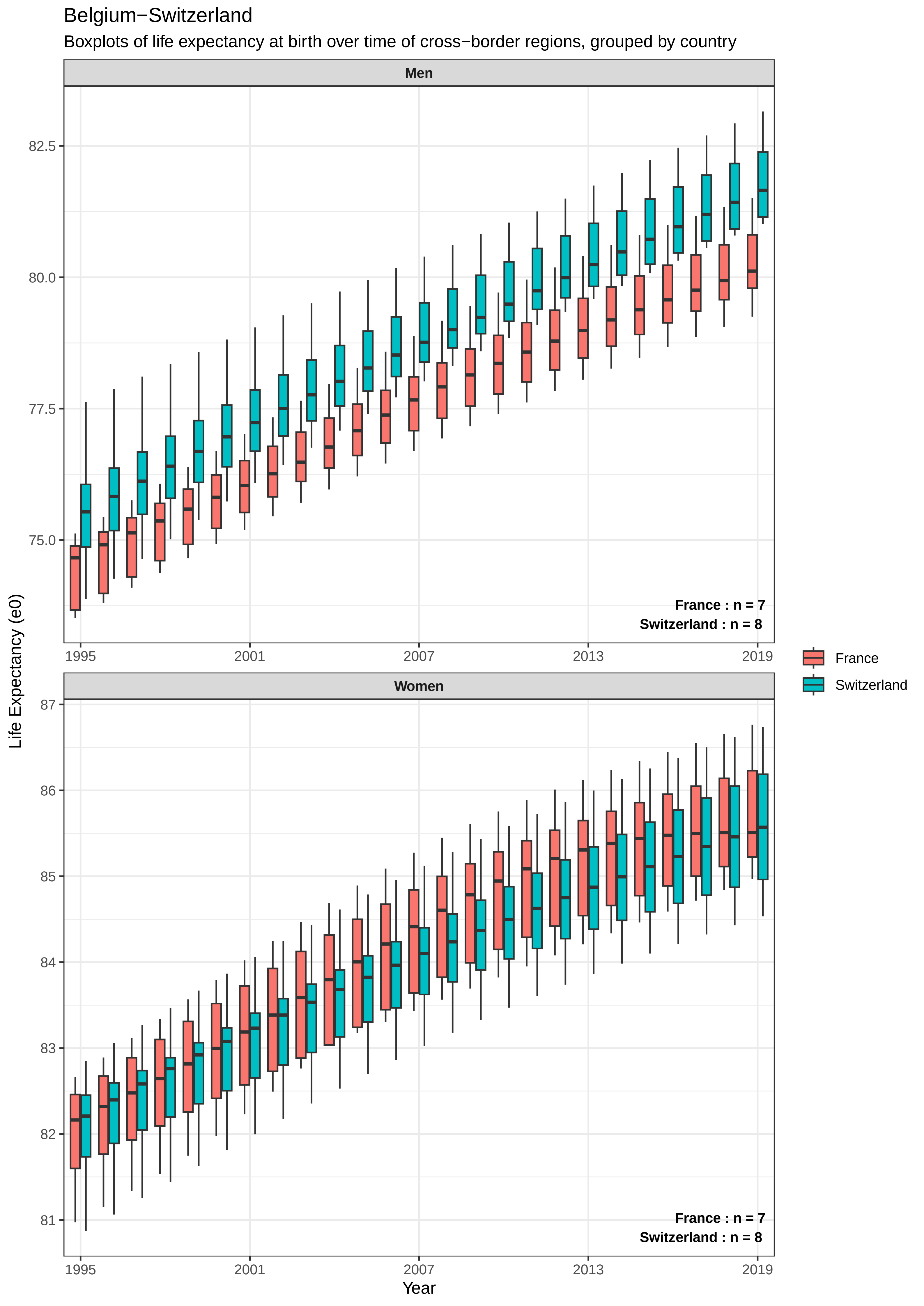
Belgium−Switzerland
Boxplots of life expectancy at birth over time of cross−border regions, grouped by country
Men
Women
Life Expectancy (e0)
Year
France : n = 7
Switzerland : n = 8
France
Switzerland
1995
2001
2007
2013
2019
75.0
77.5
80.0
82.5
81
82
83
84
85
86
87

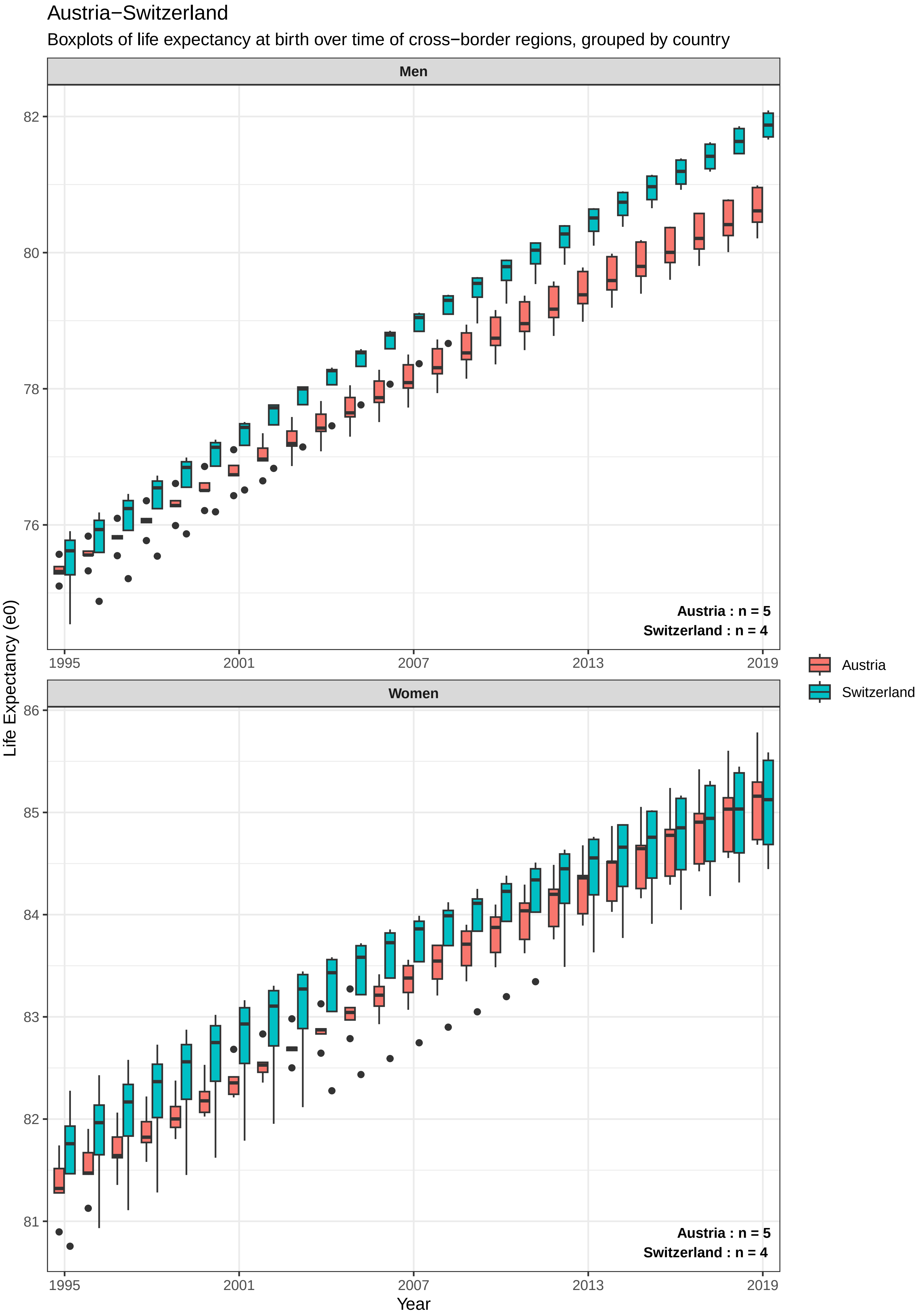

Austria−Switzerland
Boxplots of life expectancy at birth over time of cross−border regions, grouped by country
Men
Women
Austria : n = 5
Switzerland : n = 4
Austria : n = 5
Switzerland : n = 4
82
80
78
76
86
85
84
83
82
81
1995
2001
2007
2013
2019
Life Expectancy (e0)
Year
Austria
Switzerland

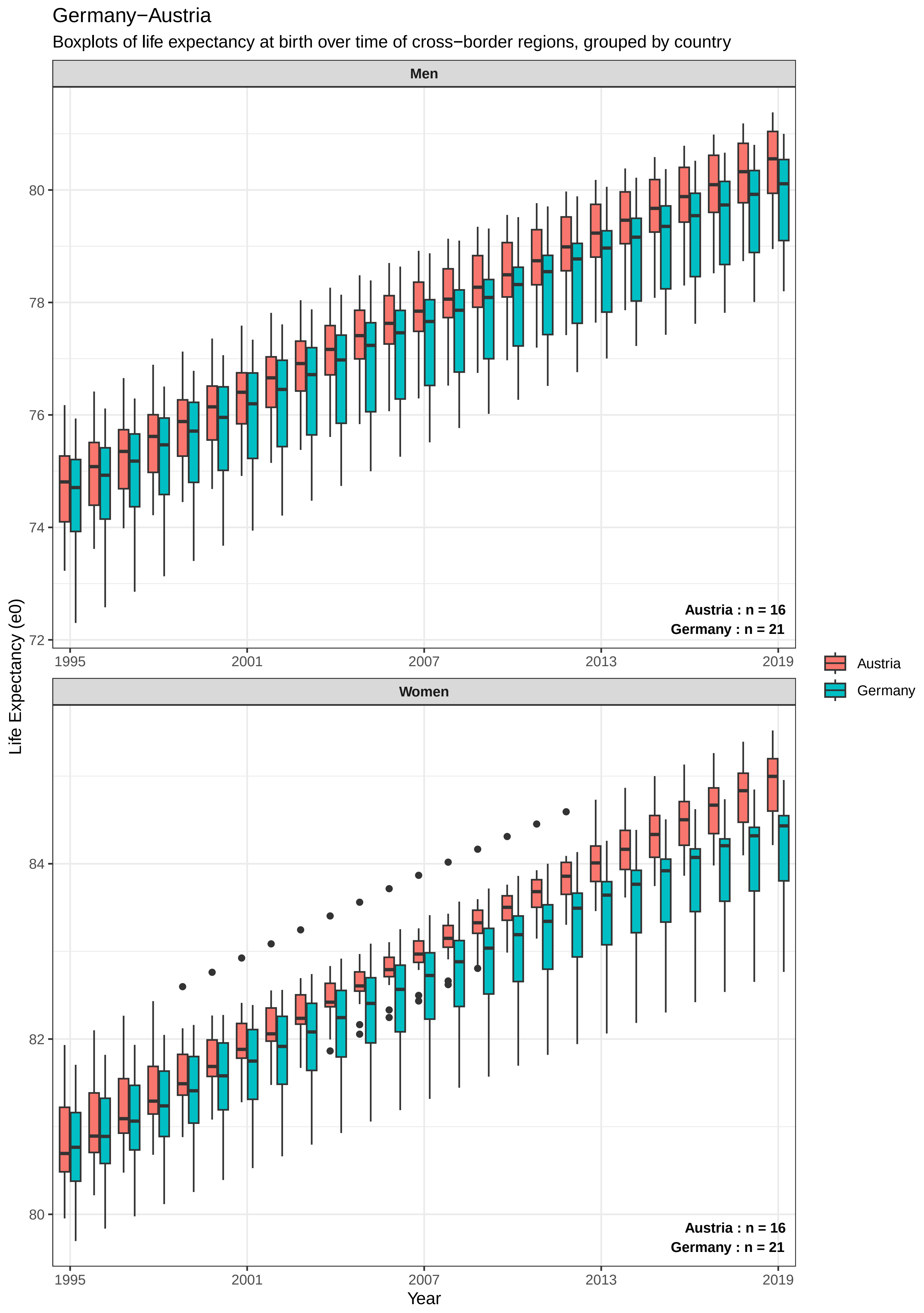
Germany−Austria
Boxplots of life expectancy at birth over time of cross−border regions, grouped by country
Men
Women
Life Expectancy (e0)
Year
Austria : n = 16
Germany : n = 21
Austria
Germany
72
74
76
78
80
82
84
1995
2001
2007
2013
2019

Germany−Switzerland

Boxplots of life expectancy at birth over time of cross−border regions, grouped by country

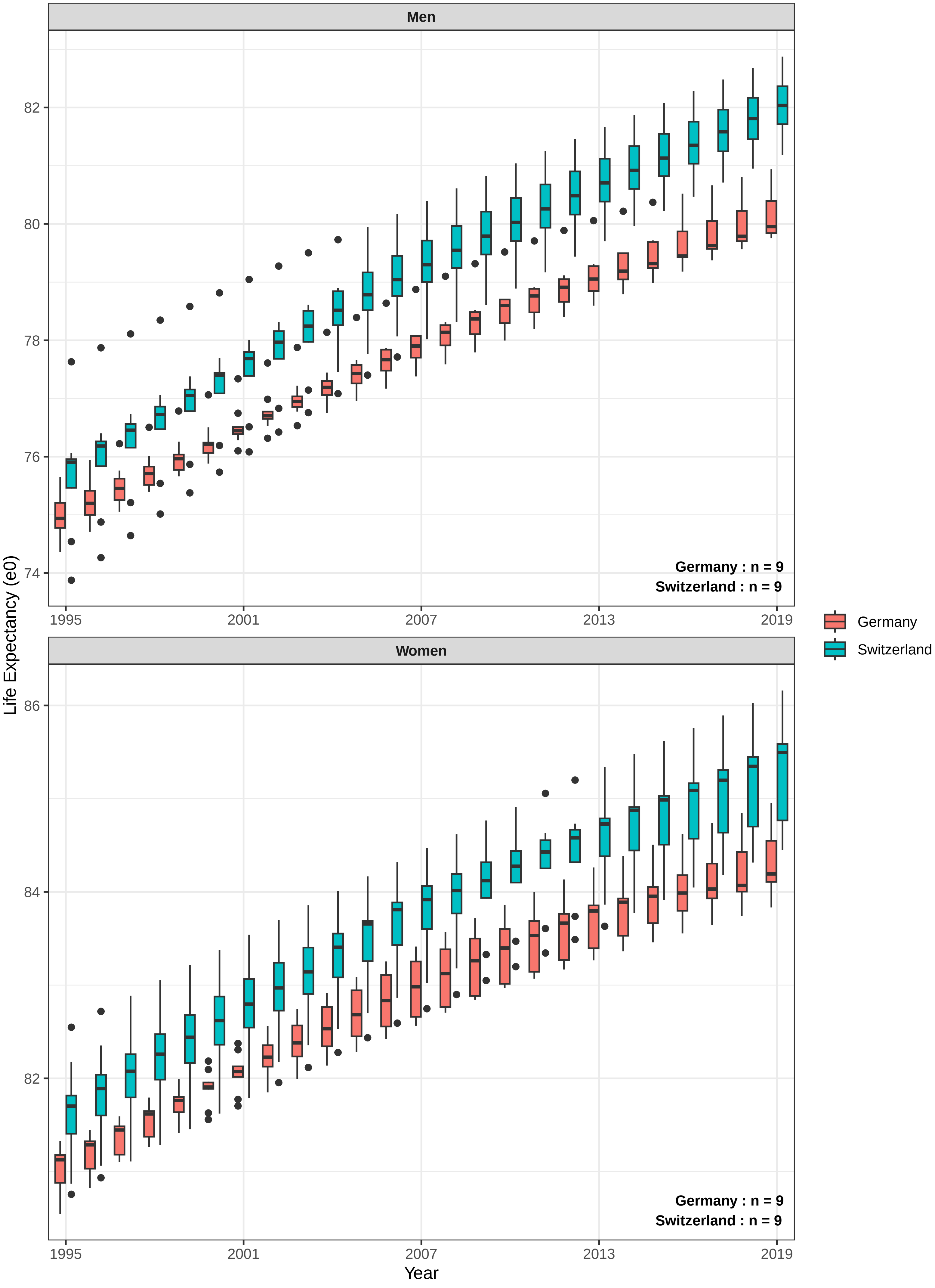

Austria−Italy

Boxplots of life expectancy at birth over time of cross−border regions, grouped by country

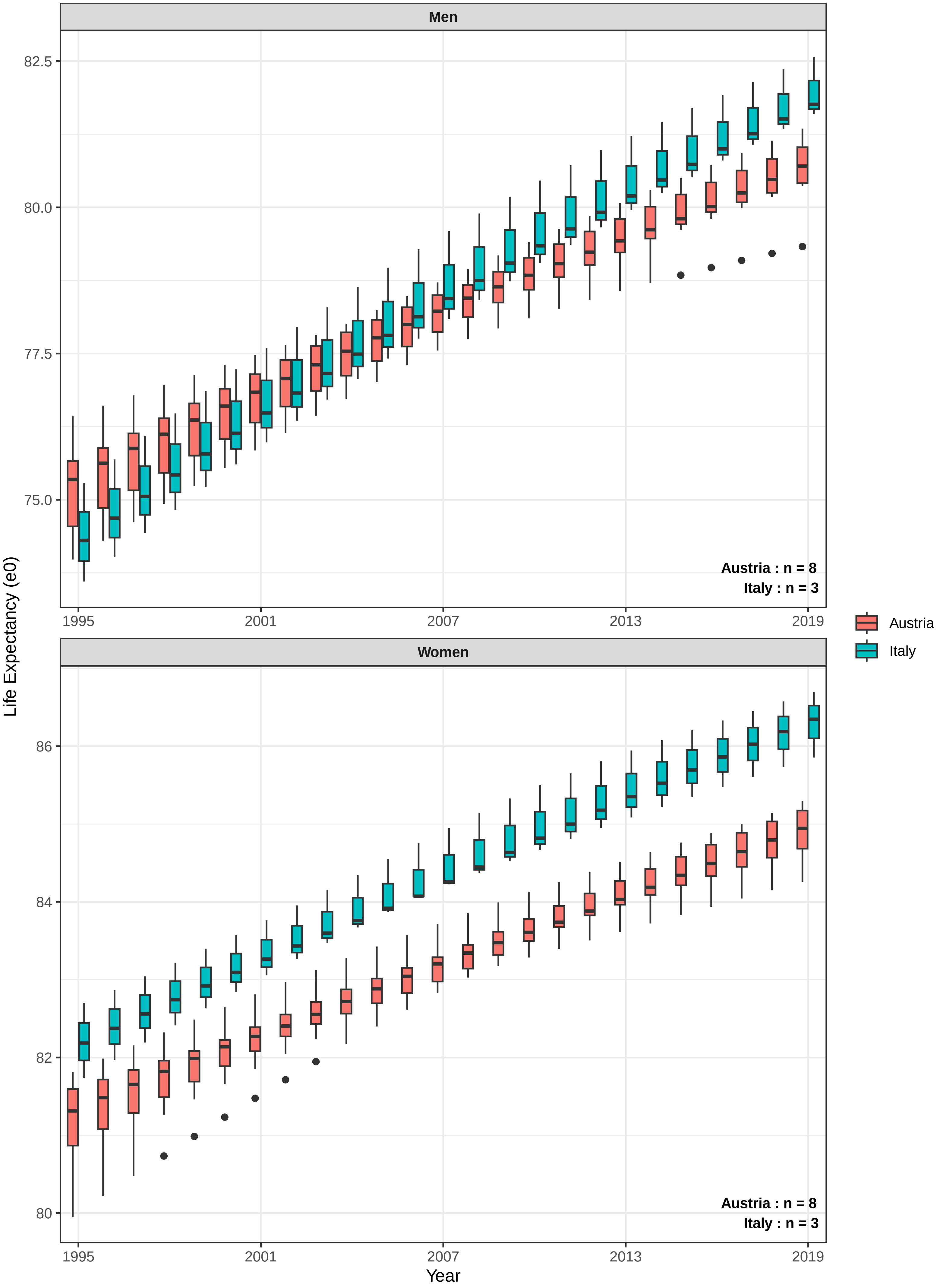

'Italy−Switzerland

Boxplots of life expectancy at birth over time of cross−border regions, grouped by country

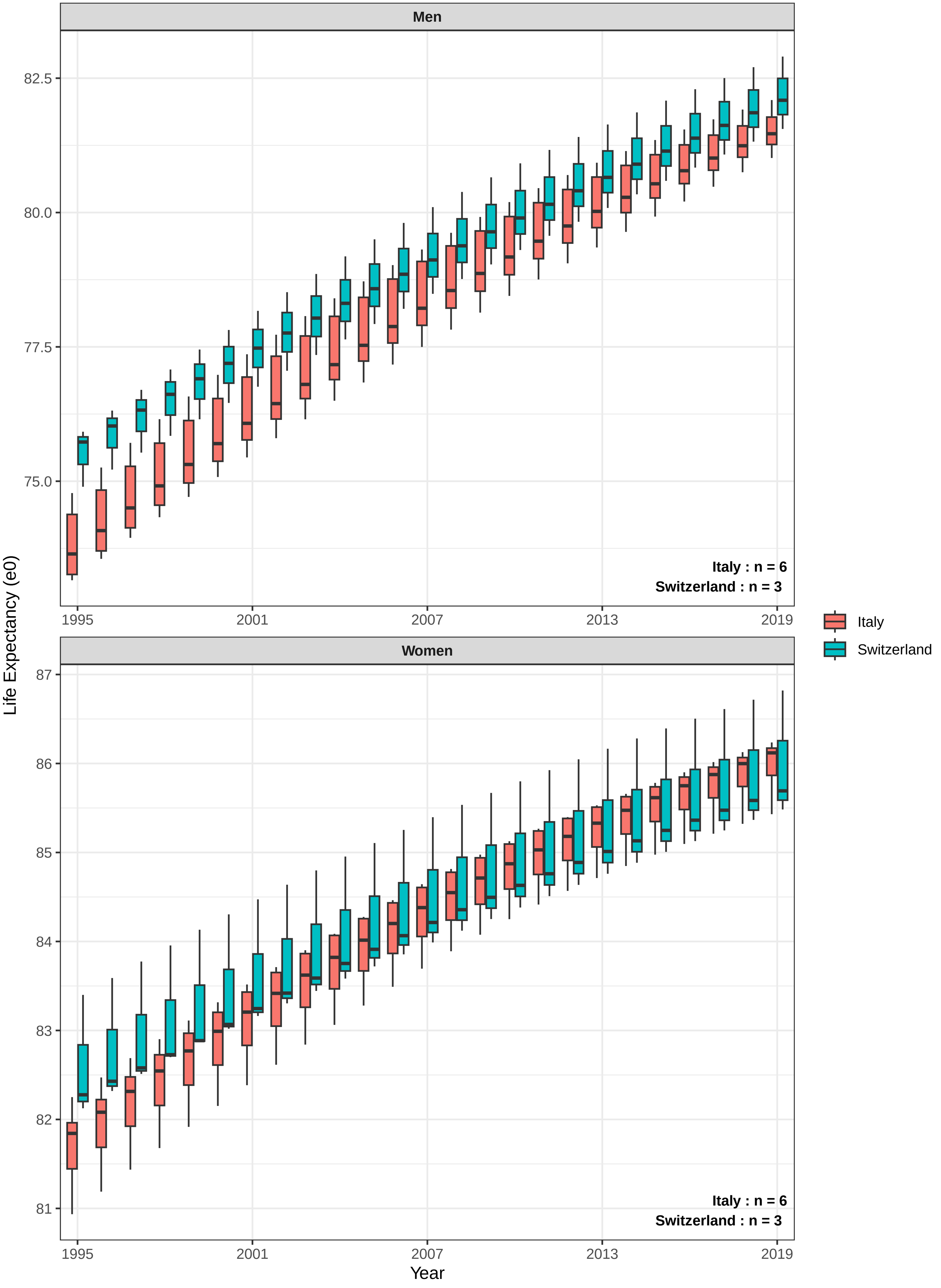

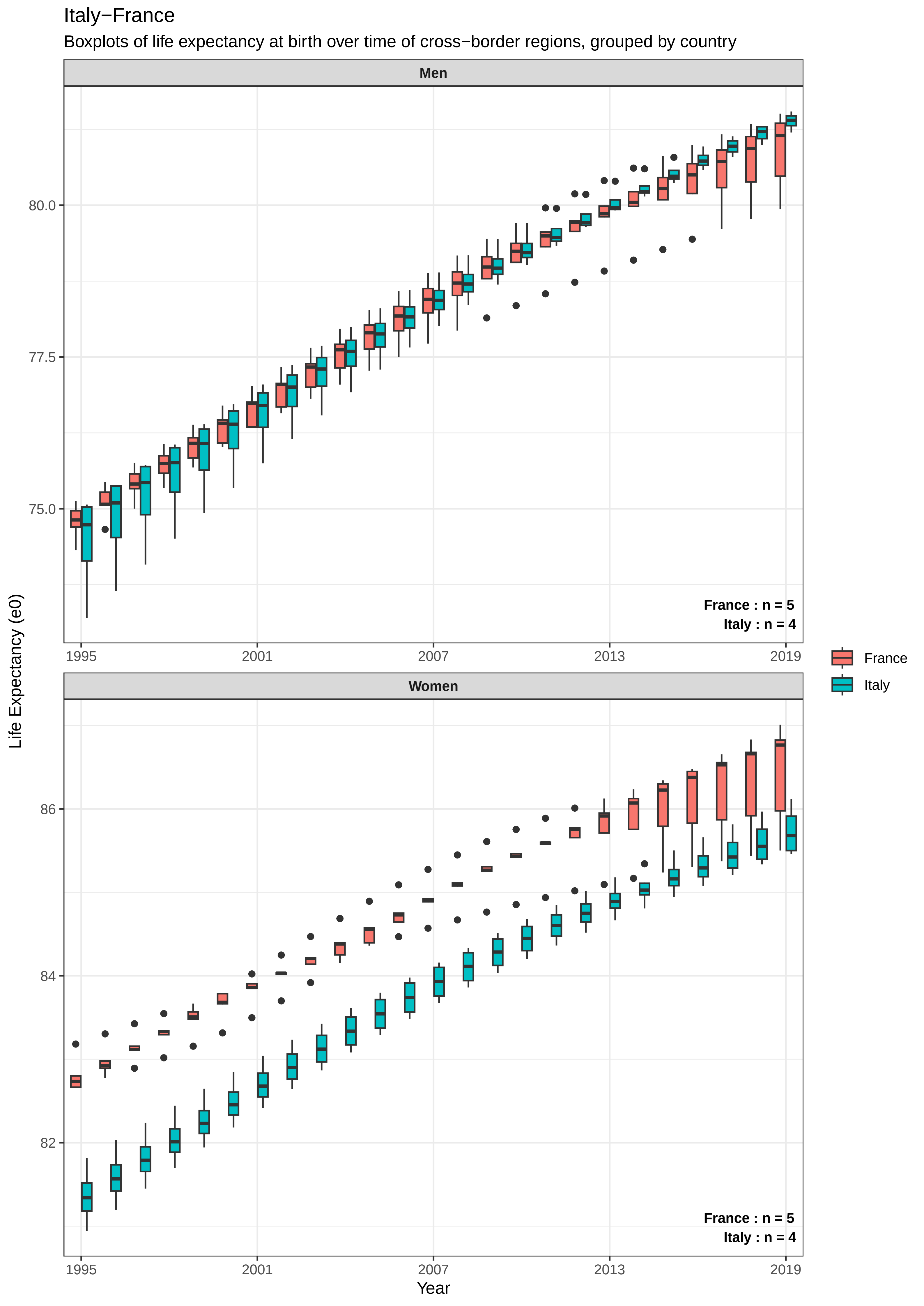

Italy−France
Boxplots of life expectancy at birth over time of cross−border regions, grouped by country
Men
Women
Life Expectancy (e0)
Year
80.0
77.5
75.0
86
84
82
1995
2001
2007
2013
2019
France : n = 5
Italy : n = 4
France
Italy

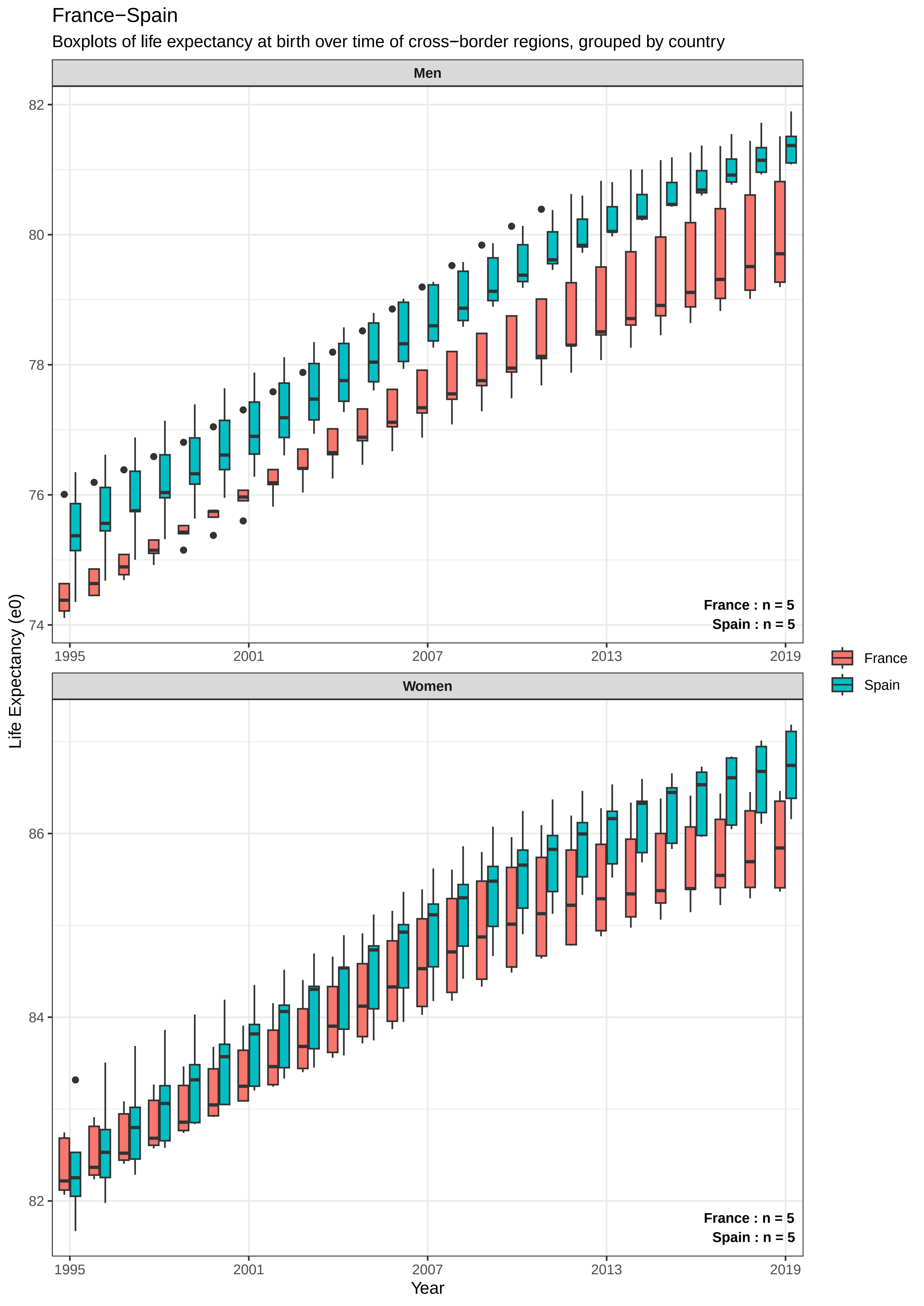
France−Spain
Boxplots of life expectancy at birth over time of cross−border regions, grouped by country
Men
Women
Life Expectancy (e0)
Year
1995
2001
2007
2013
2019
74
76
78
80
82
84
86
France : n = 5
Spain : n = 5
France
Spain

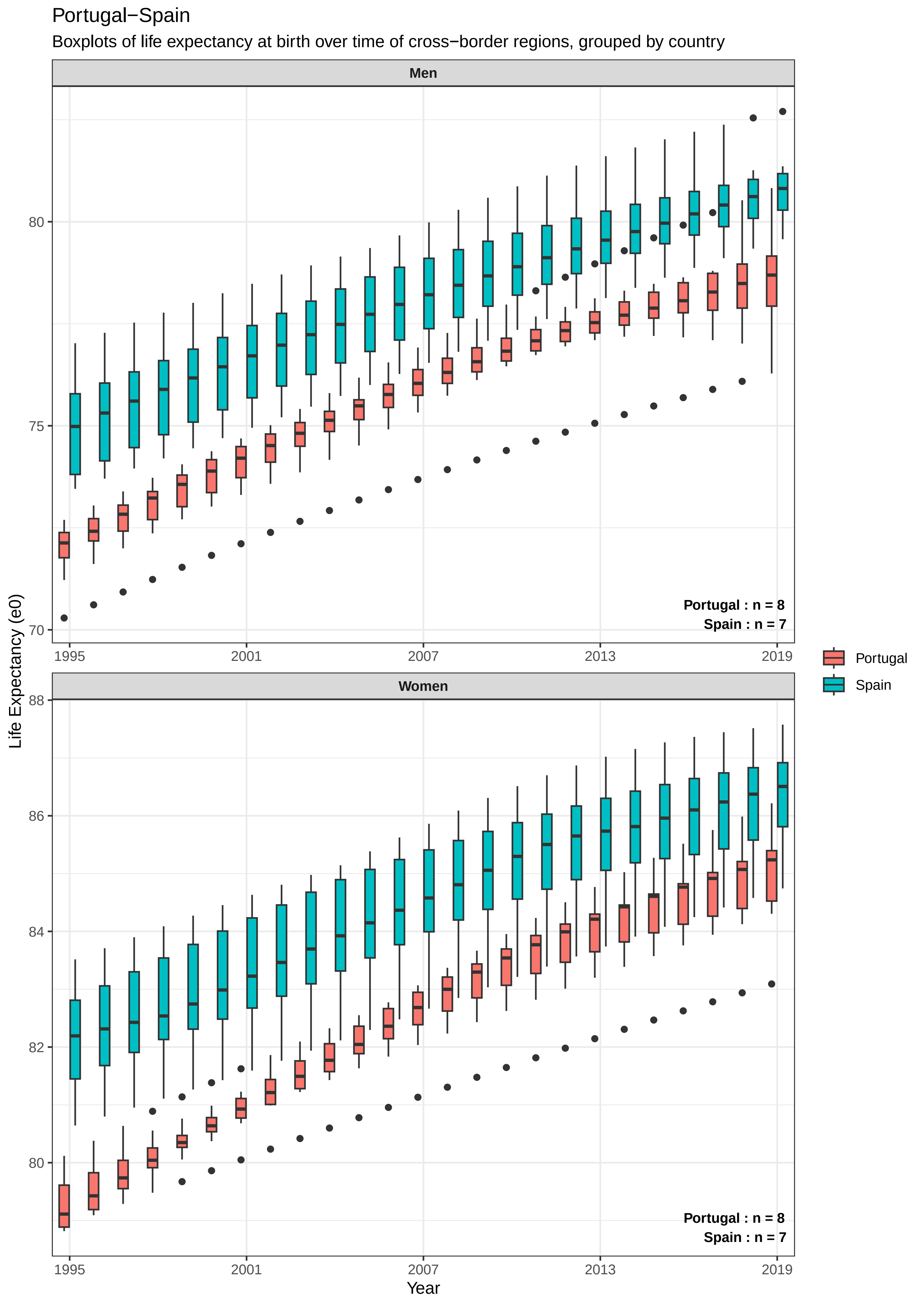

Portugal−Spain
Boxplots of life expectancy at birth over time of cross−border regions, grouped by country
Men
Women
Life Expectancy (e0)
Year
1995
2001
2007
2013
2019
70
75
80
82
84
86
88
Portugal : n = 8
Spain : n = 7
Portugal
Spain

Boxplots

Reference group 2: Non-border regions of the same country

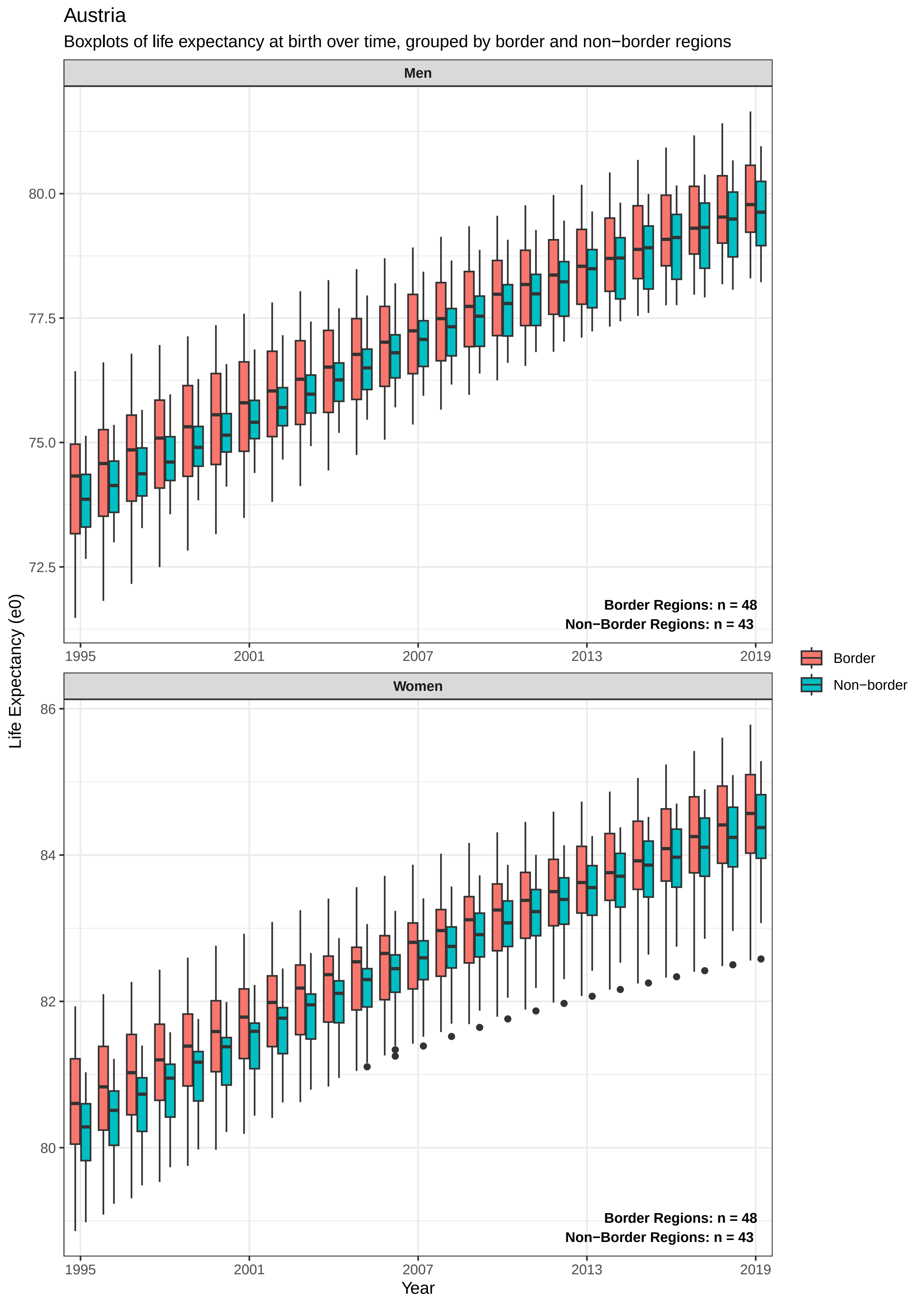
Austria
Boxplots of life expectancy at birth over time, grouped by border and non−border regions
Men
Women
Life Expectancy (e0)
Year
80.0
77.5
75.0
72.5
86
84
82
80
1995
2001
2007
2013
2019
Border Regions: n = 48
Non−Border Regions: n = 43
Border
Non−border

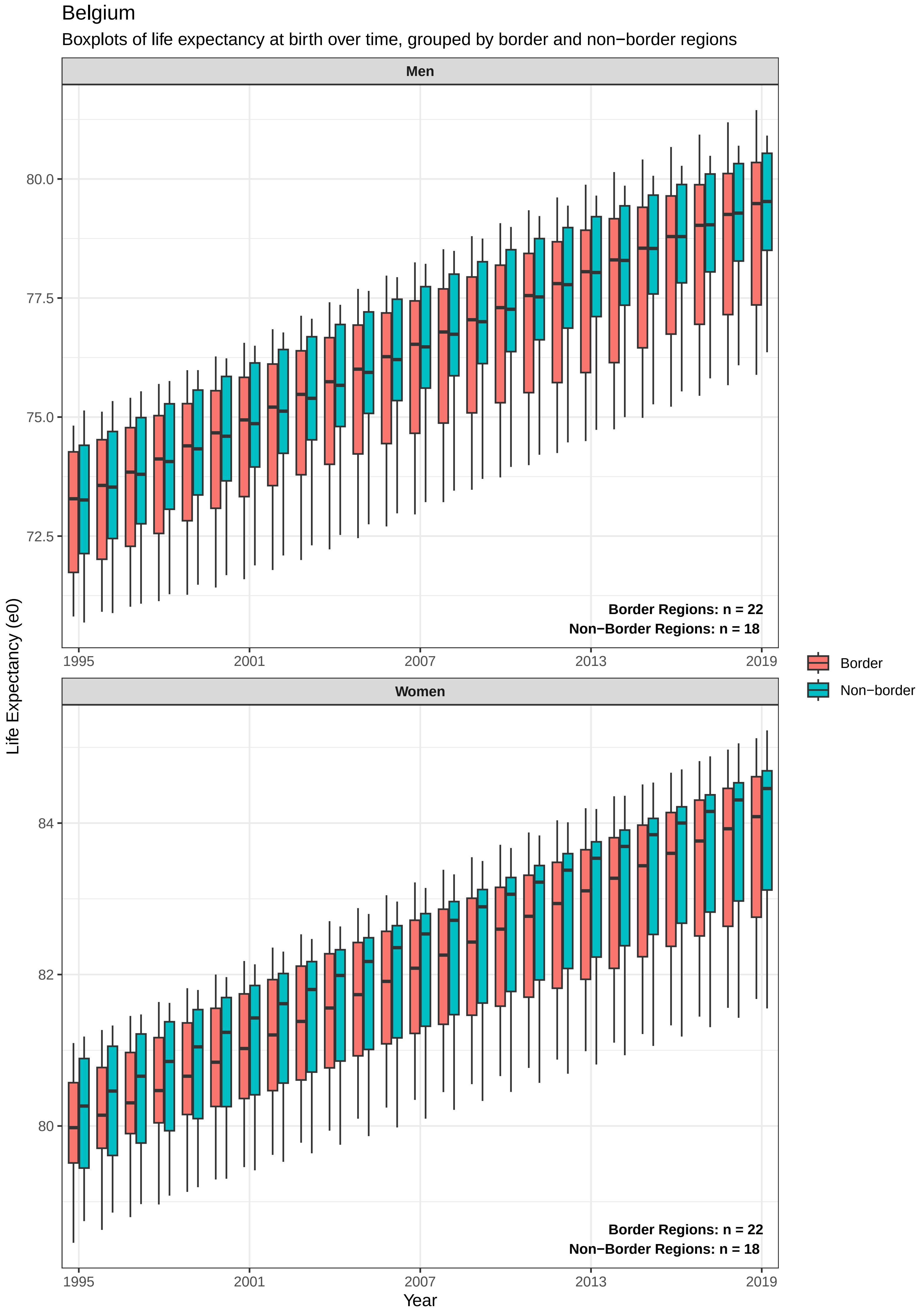

Belgium
Boxplots of life expectancy at birth over time, grouped by border and non−border regions
Men
Women
Life Expectancy (e0)
Year
80.0
77.5
75.0
72.5
84
82
80
1995
2001
2007
2013
2019
Border Regions: n = 22
Non−Border Regions: n = 18
Border
Non−border

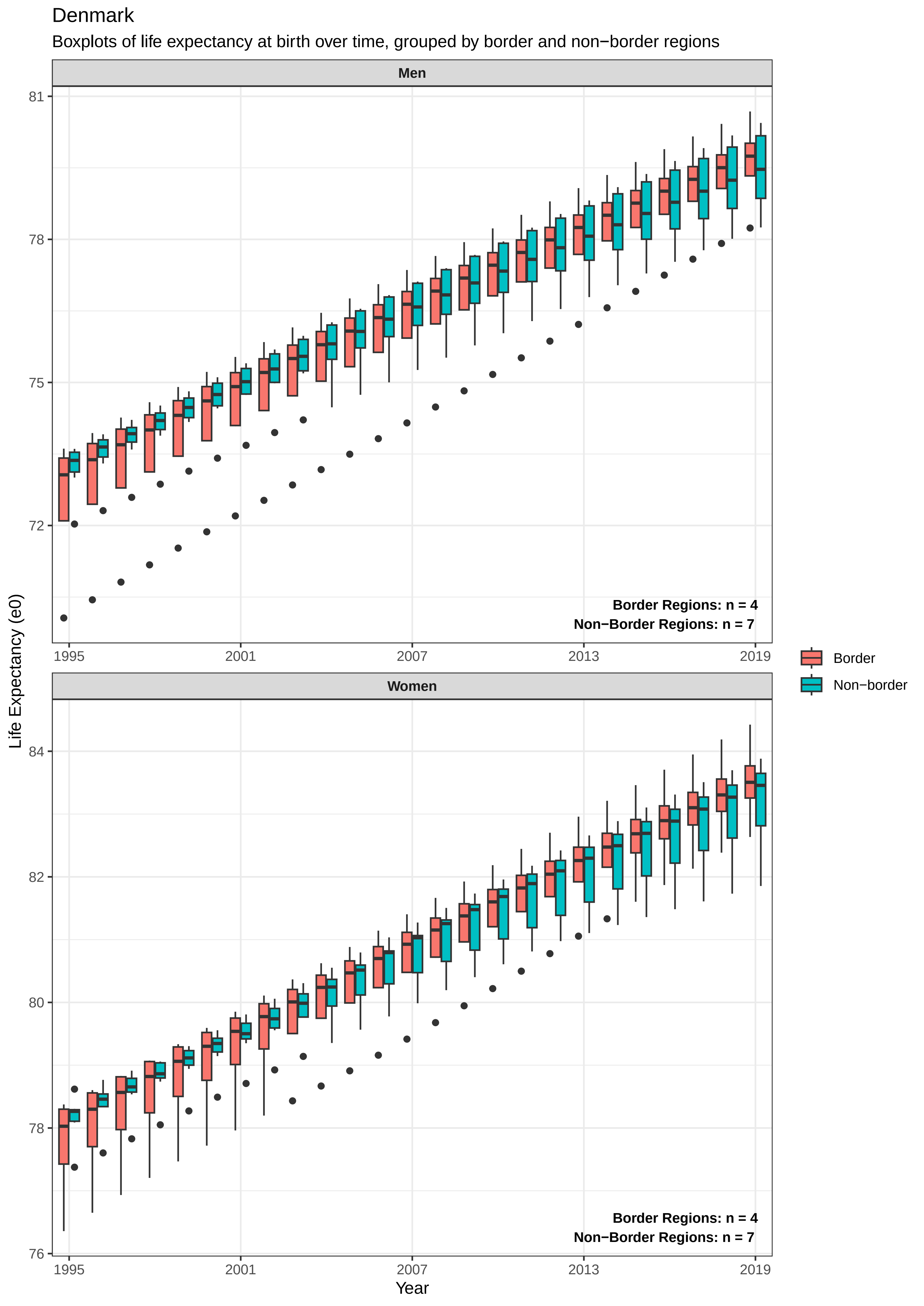

Denmark
Boxplots of life expectancy at birth over time, grouped by border and non−border regions
Men
Women
Life Expectancy (e0)
Year
81
78
75
72
84
82
80
78
76
1995
2001
2007
2013
2019
Border Regions: n = 4
Non−Border Regions: n = 7
Border
Non−border

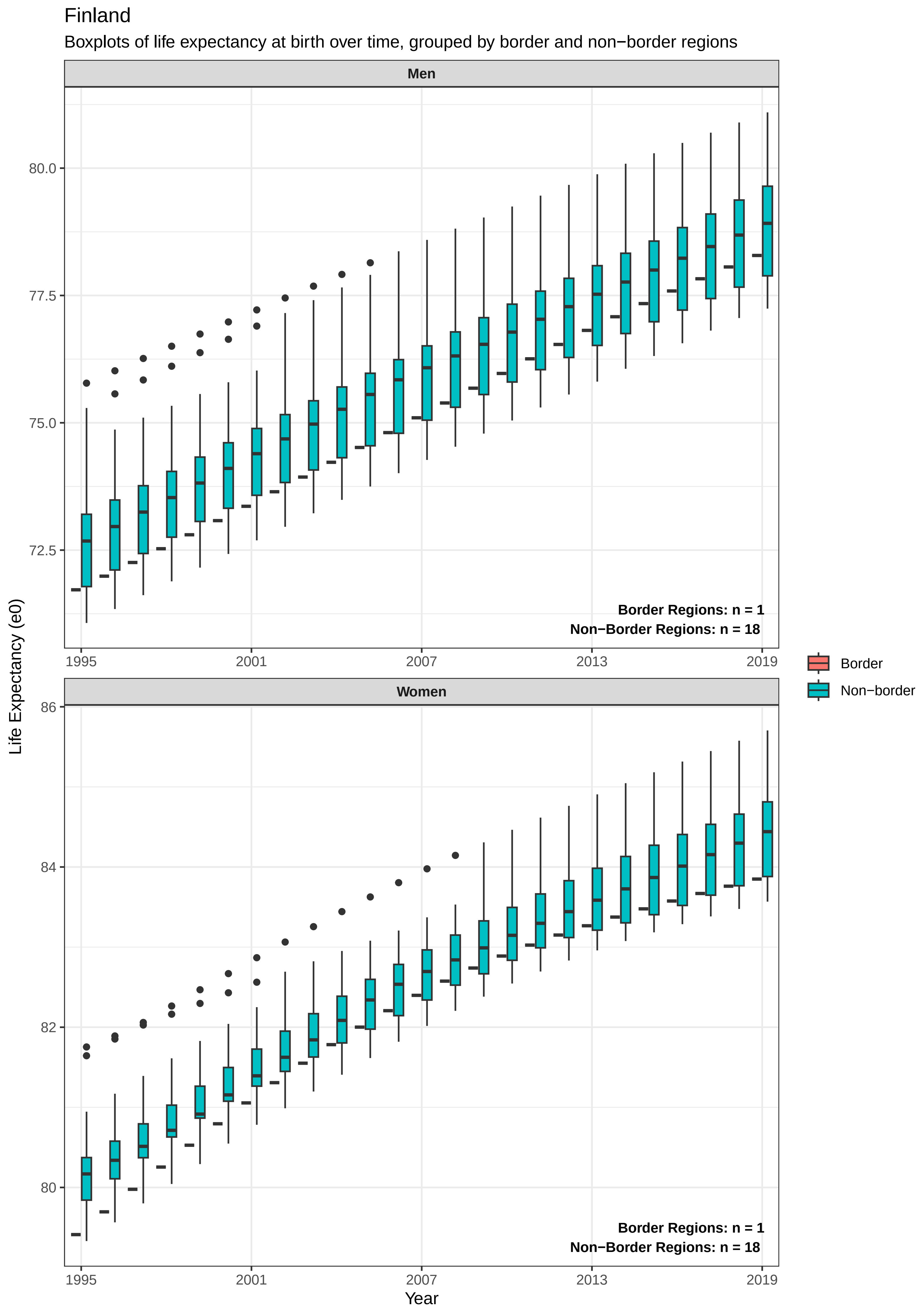
Finland
Boxplots of life expectancy at birth over time, grouped by border and non−border regions
Men
Women
Life Expectancy (e0)
Year
Border Regions: n = 1
Non−Border Regions: n = 18
Border
Non−border
80.0
77.5
75.0
72.5
86
84
82
80
1995
2001
2007
2013
2019

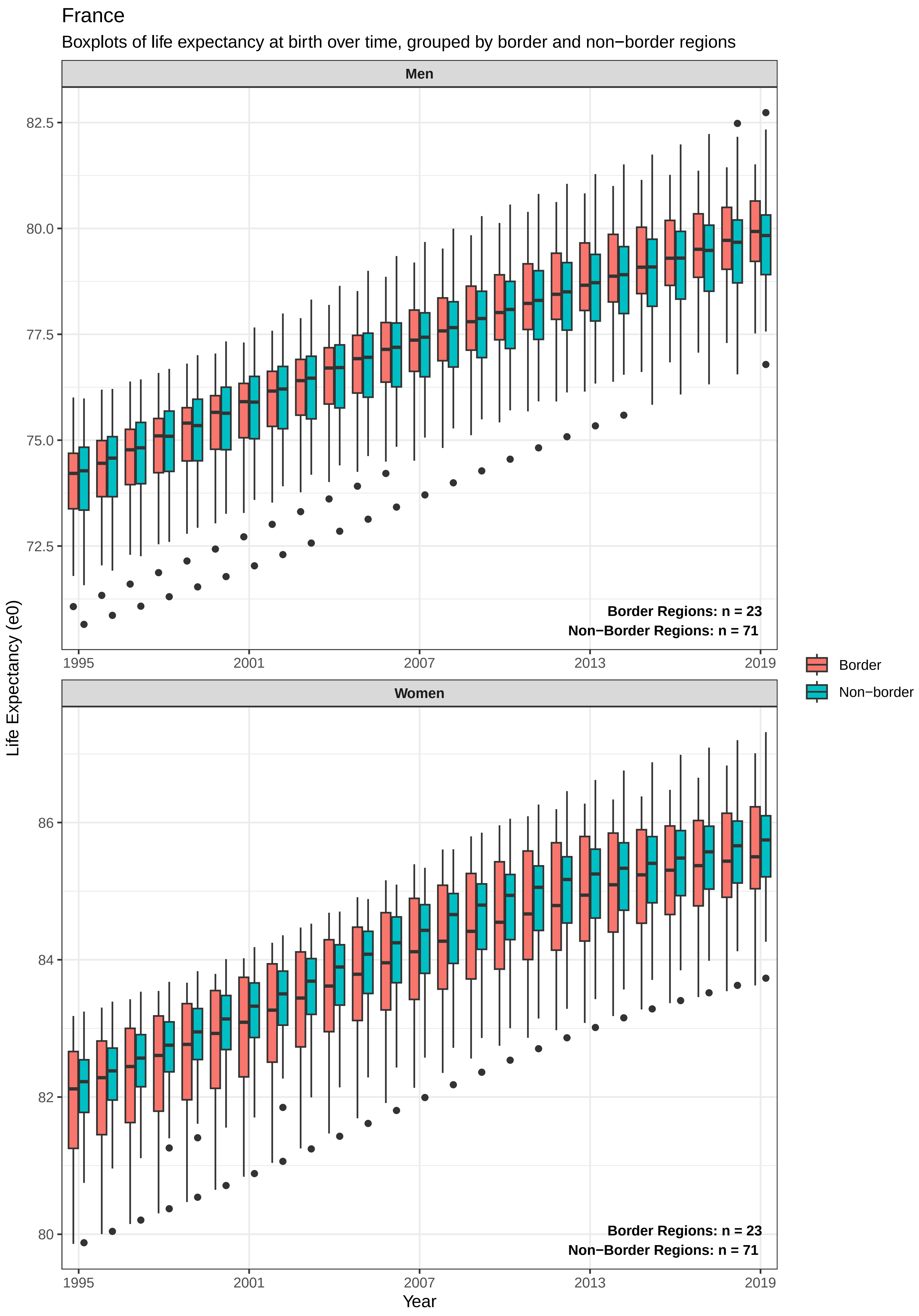
France
Boxplots of life expectancy at birth over time, grouped by border and non−border regions
Men
Women
Border Regions: n = 23
Non−Border Regions: n = 71
Life Expectancy (e0)
Year
1995
2001
2007
2013
2019
82.5
80.0
77.5
75.0
72.5
86
84
82
80
Border
Non−border

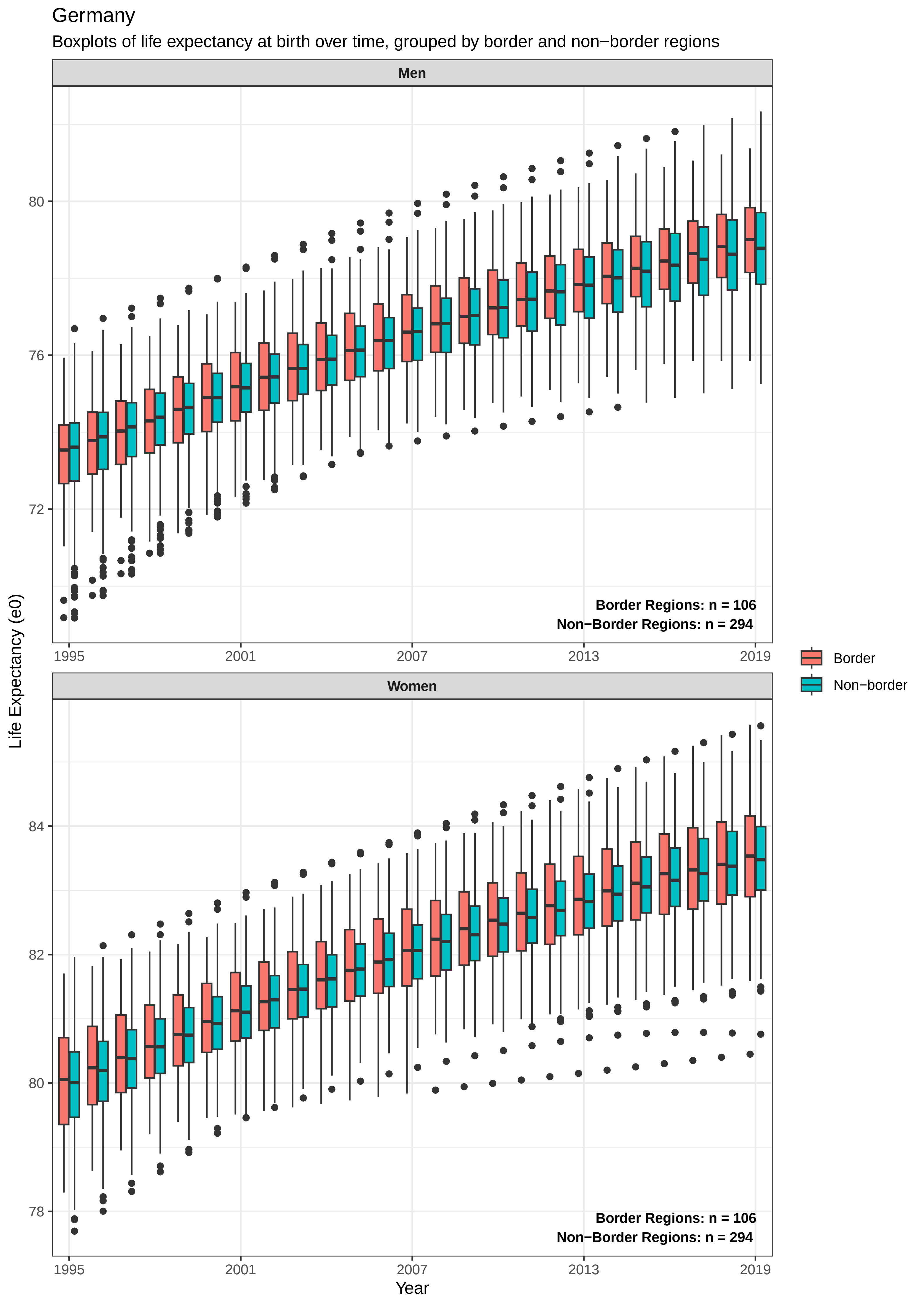

Germany
Boxplots of life expectancy at birth over time, grouped by border and non−border regions
Men
Women
Border Regions: n = 106
Non−Border Regions: n = 294
Border Regions: n = 106
Non−Border Regions: n = 294
Life Expectancy (e0)
Year
1995
2001
2007
2013
2019
72
76
80
78
80
82
84
Border
Non−border

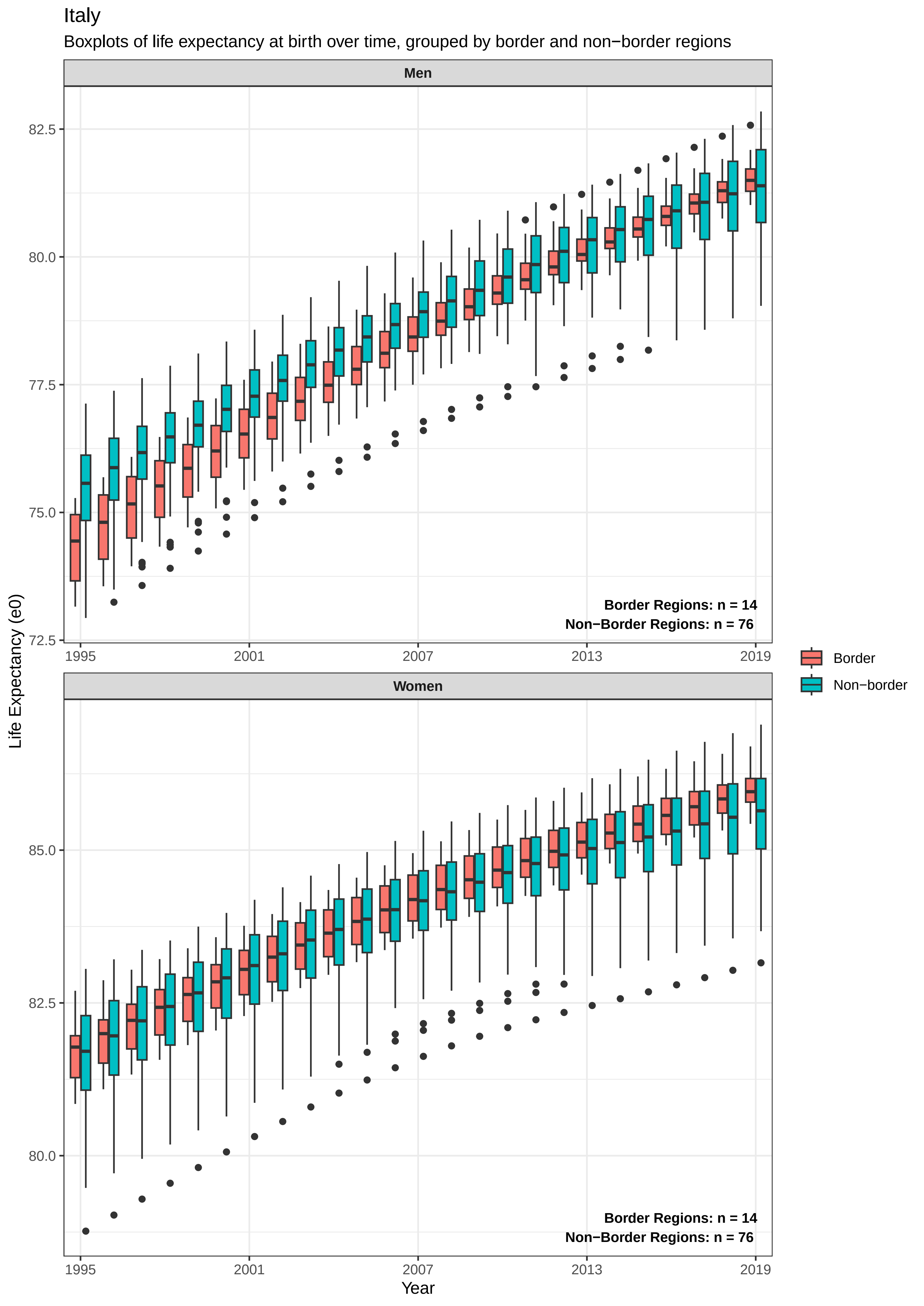
Italy
Boxplots of life expectancy at birth over time, grouped by border and non−border regions
Men
Women
Border Regions: n = 14
Non−Border Regions: n = 76
Life Expectancy (e0)
Year
1995
2001
2007
2013
2019
72.5
75.0
77.5
80.0
82.5
85.0
Border
Non−border

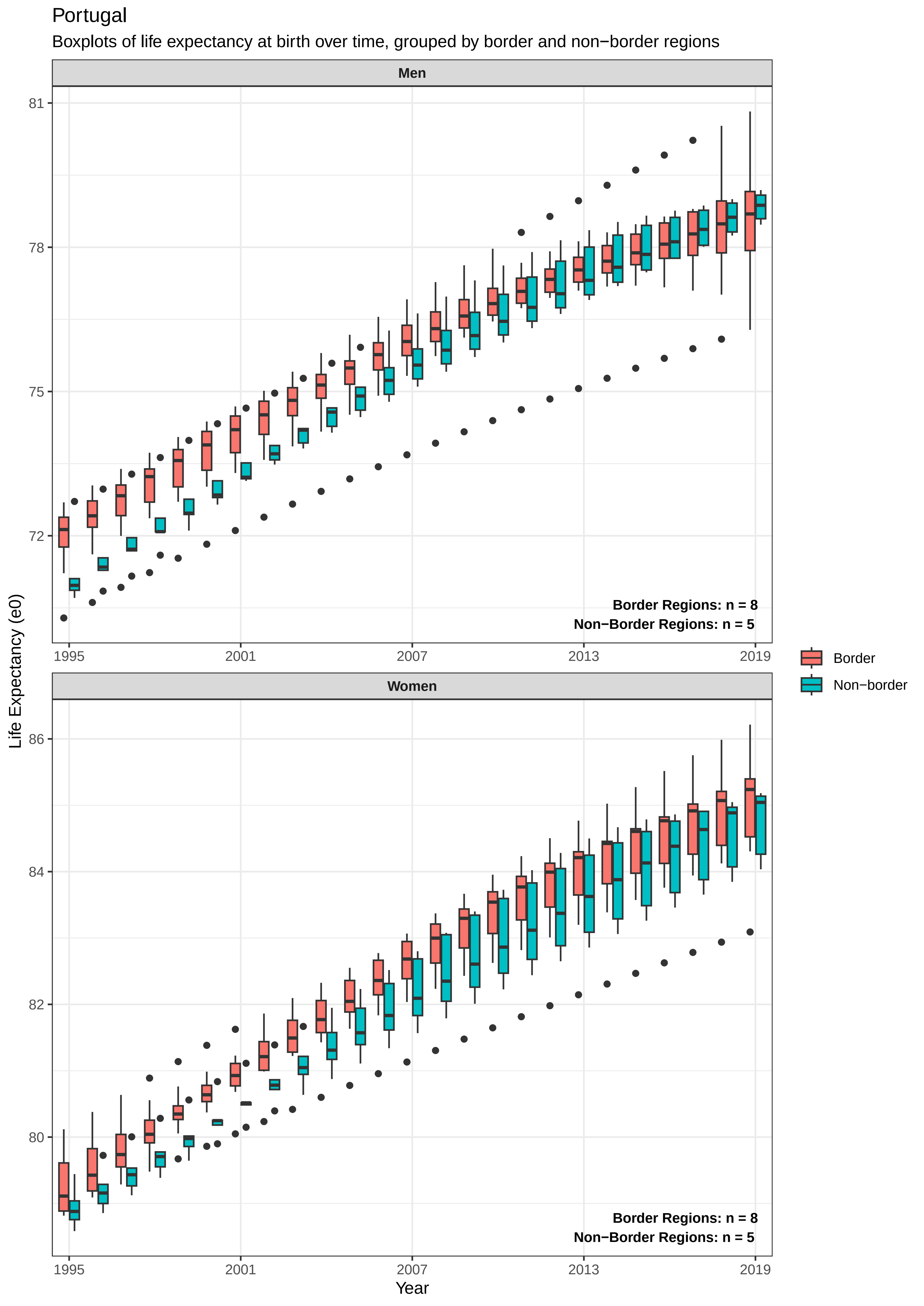

Portugal
Boxplots of life expectancy at birth over time, grouped by border and non−border regions
Men
Women
Border Regions: n = 8
Non−Border Regions: n = 5
Life Expectancy (e0)
Year
Border
Non−border

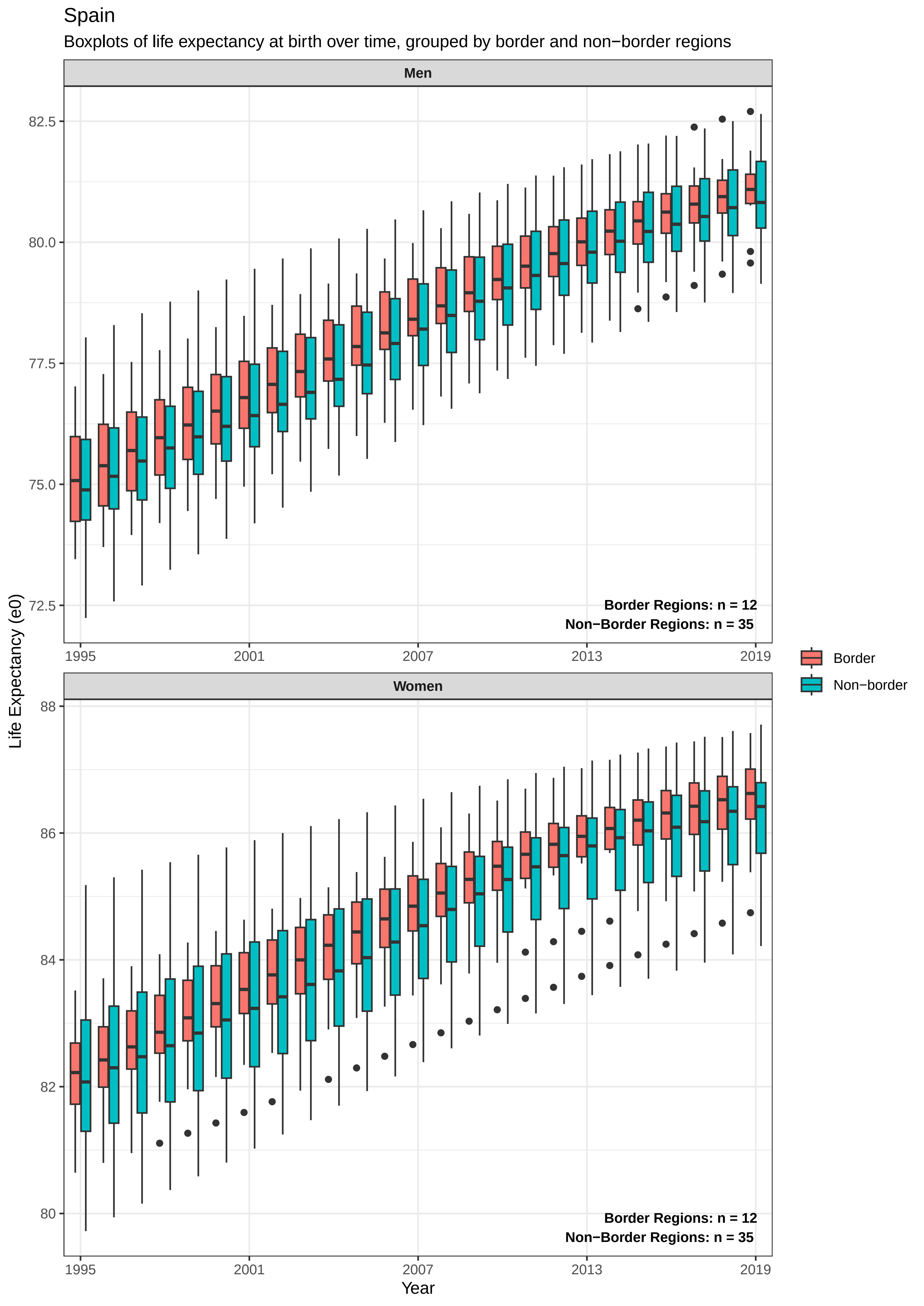
Spain
Boxplots of life expectancy at birth over time, grouped by border and non−border regions
Men
Women
Border Regions: n = 12
Non−Border Regions: n = 35
Life Expectancy (e0)
Year
82.5
80.0
77.5
75.0
72.5
88
86
84
82
80
1995
2001
2007
2013
2019
Border
Non−border

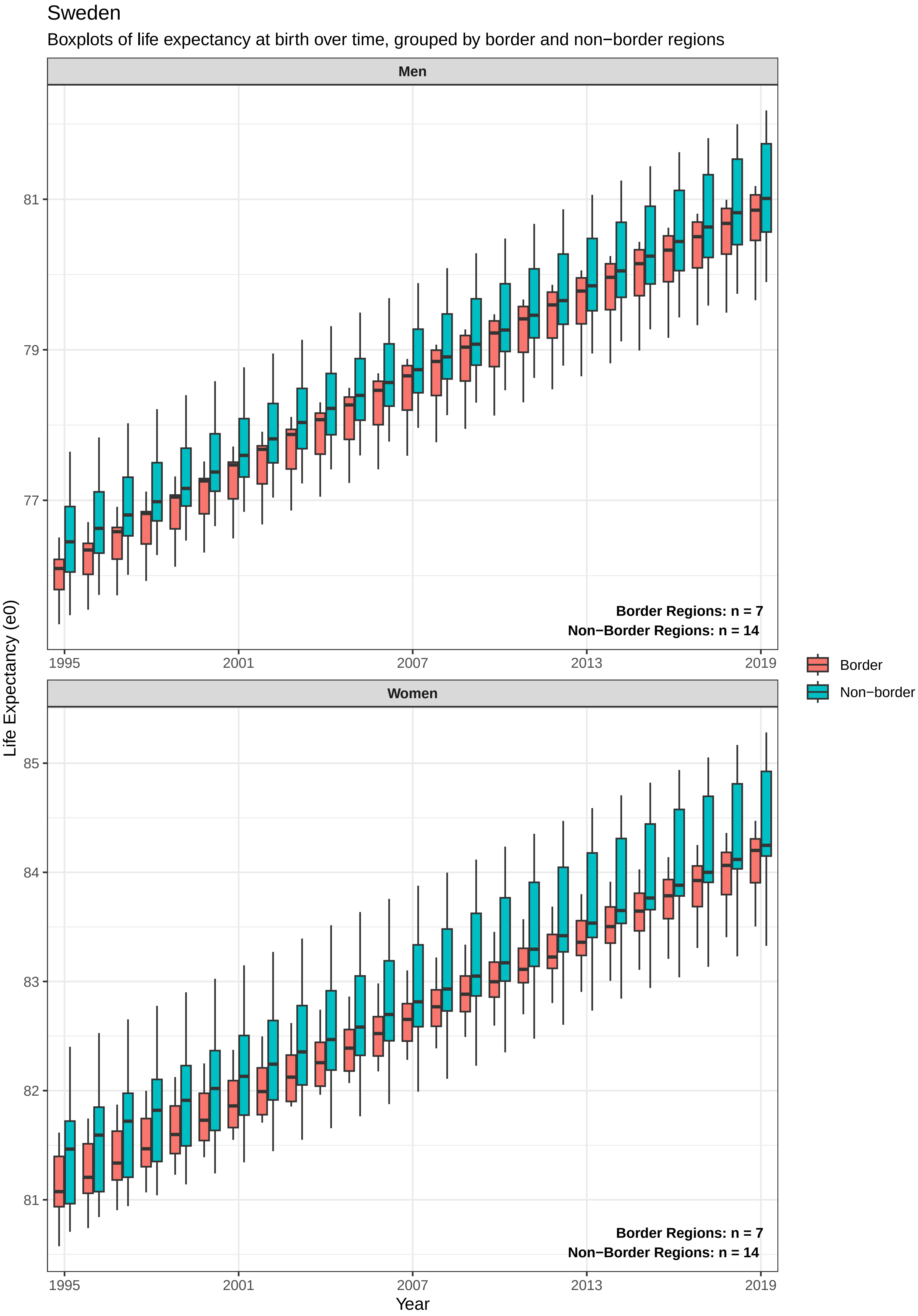
Sweden
Boxplots of life expectancy at birth over time, grouped by border and non−border regions
Men
Women
Border Regions: n = 7
Non−Border Regions: n = 14
Life Expectancy (e0)
Year
1995
2001
2007
2013
2019
77
79
81
82
83
84
85
Border
Non−border

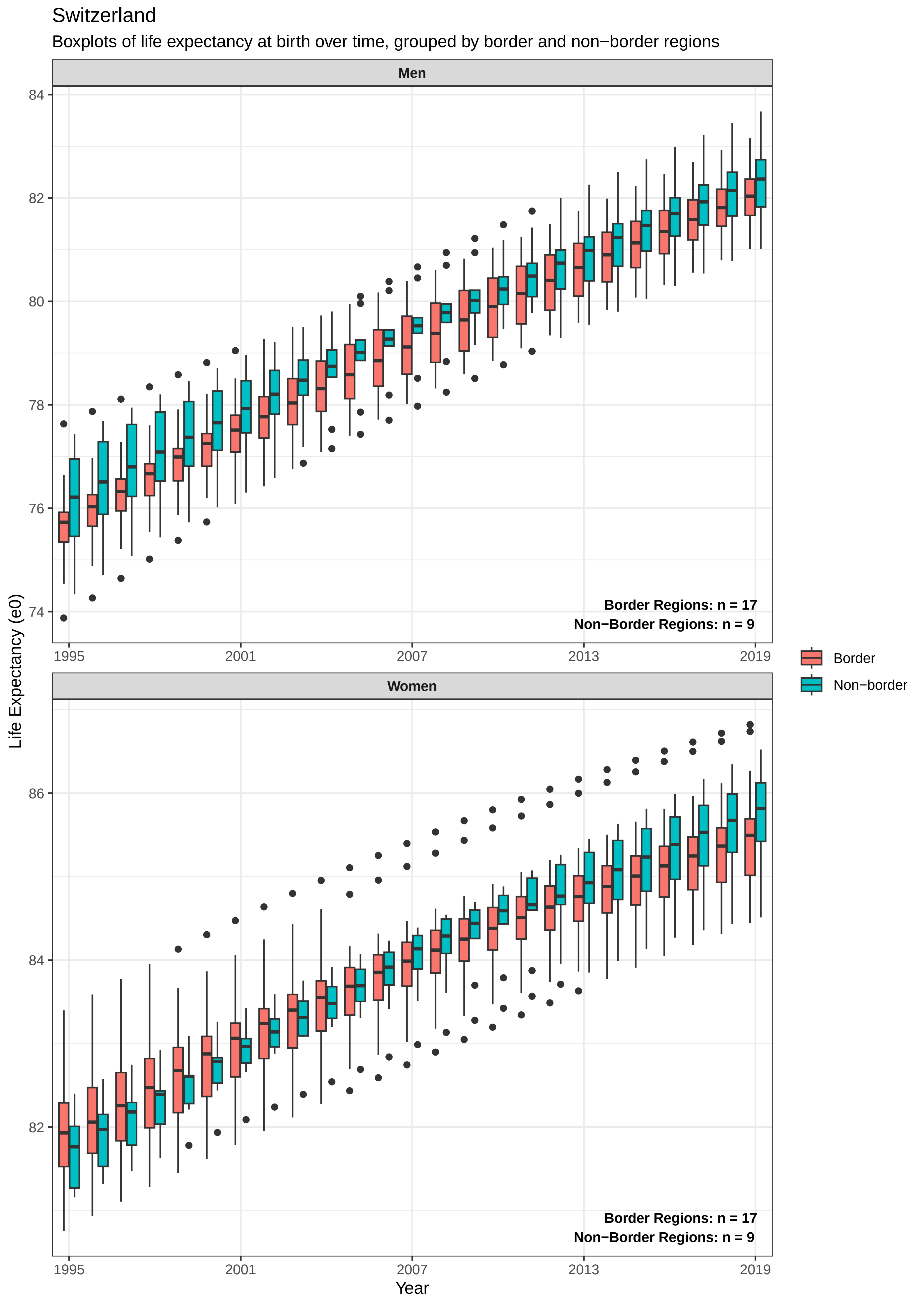

Switzerland
Boxplots of life expectancy at birth over time, grouped by border and non−border regions
Men
Women
Border Regions: n = 17
Non−Border Regions: n = 9
84
82
80
78
76
74
86
1995
2001
2007
2013
2019
Life Expectancy (e0)
Year
Border
Non−border

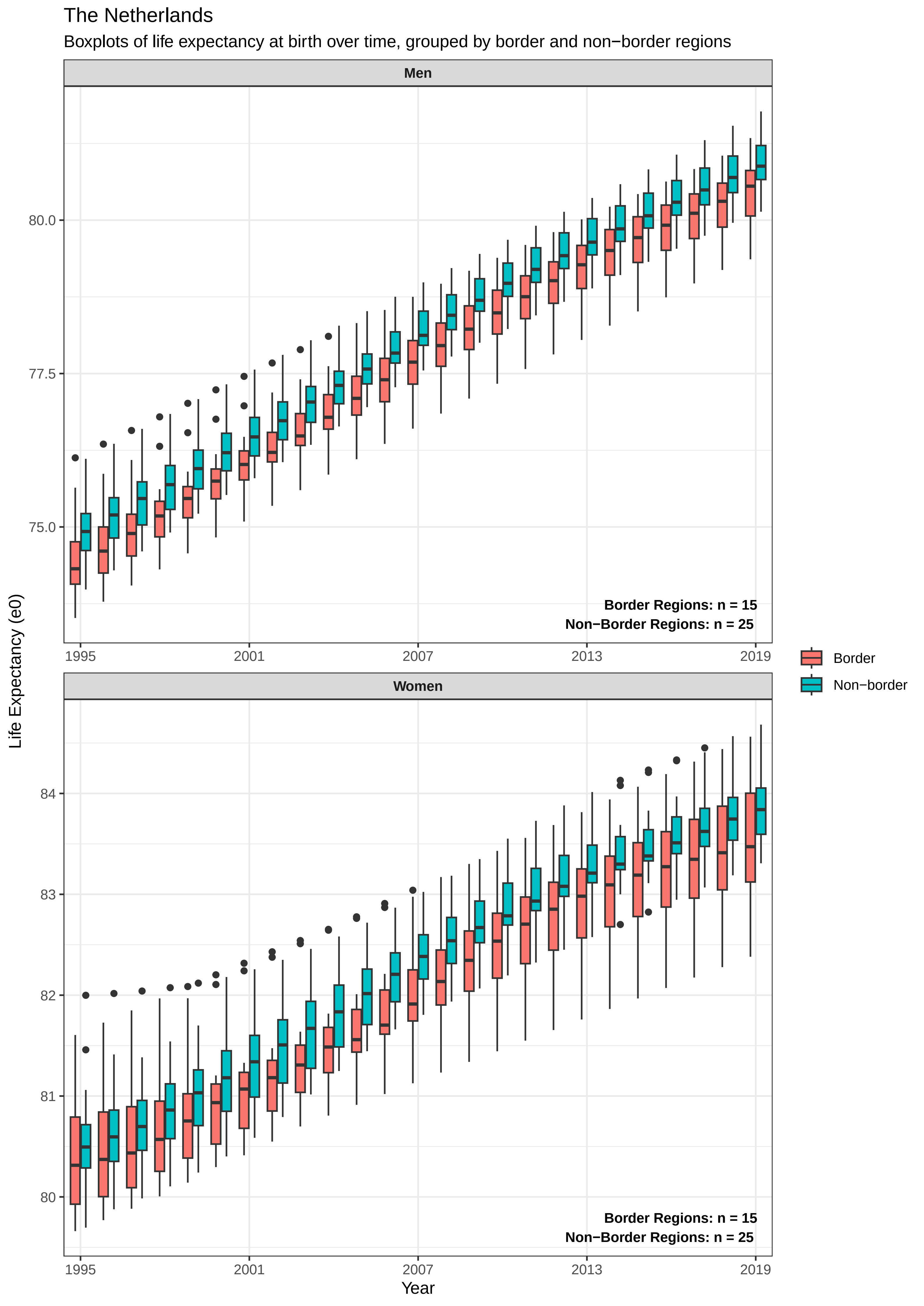

The Netherlands
Boxplots of life expectancy at birth over time, grouped by border and non−border regions
Men
80.0
77.5
75.0
Border Regions: n = 15
Non−Border Regions: n = 25
1995
2001
2007
2013
2019
Women
84
83
82
81
80
Border Regions: n = 15
Non−Border Regions: n = 25
1995
2001
2007
2013
2019
Year
Life Expectancy (e0)
Border
Non−border
